# Bacterial Colonization in the Airways and Intestines of Twin and Singleton Preterm Neonates: A Single-Center Study

**DOI:** 10.1155/2023/2973605

**Published:** 2023-08-01

**Authors:** Jiawei Yao, Tao Ai, Lei Zhang, Wei Tang, Zijin Chen, Yuedong Huang, Yinghong Fan

**Affiliations:** Division of Pediatric Pulmonology, Chengdu Women's and Children's Central Hospital, School of Medicine, University of Electronic Science and Technology of China, Chengdu, Sichuan, China

## Abstract

Limited studies have investigated the microbial colonization of the airways and intestines in preterm neonates. We studied the composition of intestinal and airway bacterial colonies in several preterm twin pairs and singletons to explore the dominant bacteria, assess their variability, and predict their phenotypic and metabolic functions. In this descriptive study, we collected sputum and fetal stool specimens from 10 twin pairs (20 cases) and 20 singleton preterm neonates. These specimens were analyzed using 16S rRNA deep sequencing to study the alpha and beta diversities and community structures of airway and intestinal bacteria and predict their metabolic functions. Specimens from twins and singleton neonates had distinct aggregations of intestinal and airway bacteria but showed similarities and high microbial diversities during initial colonization. The top five phyla were Proteobacteria, Firmicutes, Actinobacteriota, Bacteroidota, and Cyanobacteria. The top ten genera were *Streptococcus*, *Acinetobacter*, *Ralstonia*, *Staphylococcus*, *Comamonas*, *Enterococcus*, *Stenotrophomonas*, *Dechlorosoma*, *Sphingopyxis*, and *Rothia*. Potentially pathogenic and highly stress-tolerant Gram-negative bacteria were predominant in the intestinal flora. A considerable proportion of colonies recovered from the airway and intestines of preterm neonates were functional bacteria. The richness of the intestinal and airway flora was not significantly different between twins and singletons, and the flora clustered together. Both intestinal and airway bacteria of twins and singletons were similar. The species involved in initial colonization were similar but different in proportions; therefore, changes in microbial structure and richness may not be attributed to these species.

## 1. Introduction

Human microecology refers to the microorganisms colonizing parts of the human body, including the skin, digestive tract, respiratory tract, and genitourinary tract. Intestinal microecology is the most complex ecosystem in the human body, accounting for approximately 78% of the total microbial load in the body [1]. The lungs were previously assumed to be sterile; however, the refinement of group analysis techniques for 16S ribosomal RNA gene sequencing has revealed that the lungs harbor bacteria [2, 3]. Although the total number of bacteria in the lungs is low, the airway microbiome is likely critical for initiating host immunity, even at low population densities [4, 5]. The association between intestinal and airway bacterial colonization may be crucial in certain pulmonary and intestinal diseases. Studies have identified various roles of intestinal and airway bacteria in immune homeostasis and related conditions in the respiratory and digestive tracts, leading to the development of the “gut-lung axis” concept [6, 7]. Numerous studies have focused on the microbial colonization of individual body sites, whereas few have explored the association between the airway and intestinal bacteria. Understanding interactions between microbial communities in multiple organs remains a daunting challenge [8]. Research on the “gut-lung axis” focuses mainly on the impact of bacterial colonization on diseases and rarely on colonization in preterm twin and singleton neonates. Elucidating the colony structures of these microbiomes may provide new insights into the pathogenesis and progression of intestinal and airway diseases.

Our contact with microorganisms begins *in utero*, and the limited postnatal bacterial community expands into a dense, fixed-value, and diverse bacterial ecosystem during the first weeks of life [9]. Cases of twin pregnancies have increased significantly because of increased incidences of advanced maternal age and use of assisted reproductive technologies. Twins born at the same gestational age as singletons often have a lower body mass, comprise a higher proportion of preterm births, and present a higher risk during pregnancy [10]. Many studies have analyzed the gut and airway microbiota in full-term neonates, focusing on neonatal necrotizing small bowel colitis and bronchopulmonary dysplasia [11, 12]. However, few have examined the composition of airway and intestinal bacterial colonies in preterm twin and singleton neonates. Herein, we aimed to describe the differences in colonization between gut and airway bacteria in preterm singleton and twin neonates to investigate potential differences between the bacterial communities and predict their phenotypic and metabolic functions.

## 2. Materials and Methods

We collected fetal stool (groups A and B—twins and singletons, respectively) and sputum (groups C and D—twins and singletons, respectively) samples from 10 twin pairs and 20 singletons born preterm (gestational age, 32–35 weeks) in the obstetrics and gynecology department of our hospital from April to July 2021. At enrollment, gestational age, total leukocyte counts, hemoglobin levels, parents' ages, and reason for cesarean section were recorded. This study was approved by the Medical Ethics Committees of Chengdu Women's and Children's Central Hospital (approval number: 2021/123). All guardians signed the informed consent form.

Upon crying at birth, newborns were suctioned endotracheally. The sputum was added to 0.5 mL sterile saline in a sterile tube. A stool specimen was taken from the first fetal stool and added to 0.5 mL sterile saline in a sterile tube. These specimens were sent for analysis.

DNA was extracted using the E.Z.N.A. Bacterial DNA and E.Z.N.A. Stool DNA kits (Omega Bio-Tek, Norcross, GA, USA) following the manufacturer's instructions. PCR primers were designed to target the variable region of the 16S/ITS2 rDNA gene. The 16S V3–V4 region was amplified using PCR with 341F (5′-CCTACGGGNGGCWGCAG-3′) and 805R (5′-GACTACHVGGGTATCTAATCC-3′). After 35 cycles, sequencing adapters and barcodes were added for amplification. Amplification products were detected using 1.5% agarose gel electrophoresis. Amplified fragments were recovered using AxyPrep PCR Cleanup Kit (Beckman Coulter Genomics, Danvers, MA, USA) and purified using the Quant-iT PicoGreen dsDNA assay kit (Invitrogen, Carlsbad, CA, USA). Amplicon libraries were prepared for sequencing; their size and number were evaluated using an Agilent 2100 bioanalyzer (Agilent Technologies, Santa Clara, CA, USA) and an Illumina library quantification kit (Kapa Biosciences, Woburn, MA, USA), respectively. These libraries were sequenced using NovaSeq PE250.

Paired-end reads were assigned to the samples using their unique barcodes and truncated by cutting off the barcode and primer sequences. They were merged using FLASH (v1.2.8) for 16S rRNA and PEAR (v0.9.6) for ITS2 rDNA. Raw reads were quality filtered to obtain high-quality clean tags using fqtrim (v0.94). Chimeric sequences were filtered using VSEARCH software (v2.3.4). After dereplication using DADA2, amplicon sequence variants (ASVs) were employed to build operational taxonomic units (Supplementary Material 1). Using these OTUs, we obtained feature tables and sequences. Alpha and beta diversities were calculated using QIIME2. The exact numbers of sequences were extracted randomly, reducing their numbers to the minimum of the samples. The relative abundance was calculated (bacterial count/total count). Pictures were drawn in R (v3.5.2) [13].

### 2.1. Statistical Analysis

Data were analyzed using SPSS (v20.0; IBM, Armonk, NY, USA). Continuous variables were assessed using an independent samples *t*-test, and categorical variables were compared using the Fisher exact test. *P* < 0.05 was considered significant.

Sparse curves were plotted to assess whether sequencing depth could reveal the microbial community diversity. Alpha-diversity analyses (Chao1 and Shannon) were performed to determine the richness and evenness of communities, and violin plots were constructed to analyze the differences among subgroups [14]. Beta-diversity analyses were performed using analysis of similarity (ANOSIM) and nonmetric multidimensional scaling (NMDS) based on the Jaccard algorithm to assess species diversity among subgroups. The unweighted pair group method with arithmetic mean (UPGMA) was employed to cluster the samples evaluating differences in species diversity [15]. Species richness at phylum, order, family, genus, and species levels was obtained based on ASV annotation results and abundance table for each sample (Supplementary Material 2). The data were displayed using a heat map and stacked bar graphs. Species richness was assessed using the Kruskal–Wallis test based on the abundance of different groups. Sankey diagrams demonstrated the relative abundances of bacterial groups at phylum and genus levels for different grouped samples. Linear discriminant analysis (LDA) effect size was applied to visualize differential species at all levels [16]. The evolutionary tree of ASV signature sequences was constructed using multiple sequence comparison results. Scatter plots showed the evolution of each sample in the predicted groups, classified as aerobic, anaerobic, facultatively anaerobic, containing mobile elements, biofilm-forming, and Gram-negative [17, 18]. Functional genes of the metabolic pathways in gut and airway microbiota were predicted using PICRUSt2 [19].

## 3. Results

Forty newborns participated in this study (10 twin pairs and 20 singletons). Their stool and sputum samples were divided into four groups based on the source. The differences in gestational age, Apgar score (1 min), mode of birth, parents' ages, white blood cell and neutrophil counts, and hemoglobin levels between twins and singletons were not significant (Table 1). Cesarean delivery in twin pregnancies was caused by preterm labor (three cases), intrauterine distress (two cases), severe intrahepatic cholestasis during pregnancy (two cases), premature rupture (one case), and social factors of fetal membranes (one case). Cesarean delivery in singleton pregnancies was caused by severe eclampsia (four cases: B10, B11, B17, and B20), intrauterine distress (four cases: B5, B6, B14, and B16), scarred uterus (two cases: B13 and B19), premature rupture of fetal membranes (three cases: B1, B2, and B12), severe intrahepatic cholestasis (two cases: B4 and B9), and breech (one case: B8).

**Figure 1:**
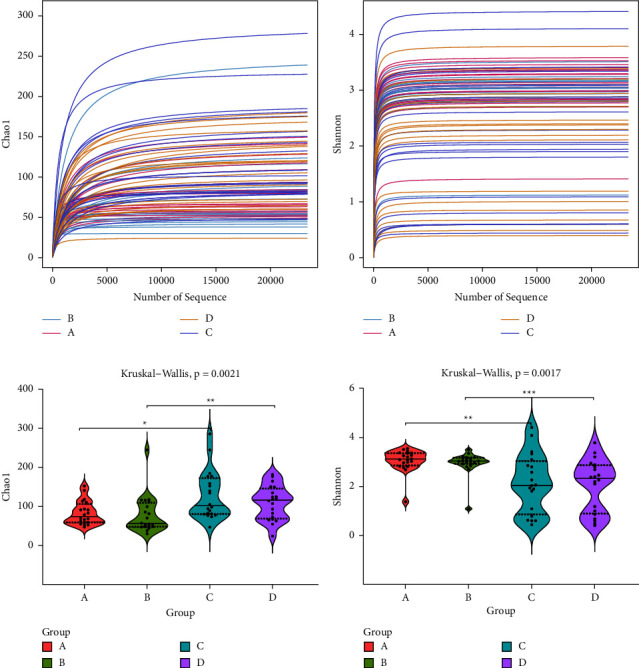
The dilution curve reflects the rationality of the amount of sequencing data and the abundance of species in the sample. The Chao1 (a) and Shannon (b) indices in the alpha-diversity analyses reflect the richness and homogeneity of bacterial colonies. The violin plot comparing the differences among the groups through the Kruskal–Wallis test (c, d). The *P* value in the upper left corner was obtained via grouping using the rank-sum test; ^*∗*^*P* < 0.05; ^*∗∗*^*P* < 0.01; ^*∗∗∗*^*P* < 0.001.

The rarefaction curves of groups A–D show that the samples had sufficient sequencing depth, suggesting a uniform distribution of samples and high species richness (Figures 1(a) and 1(b)). Chao1 (Figure 1(c)) and Shannon (Figure 1(d)) violin plots show that alpha diversities were not significantly different between groups A and B and between groups C and D. This result suggests that preterm twins and singletons of the same gestational age at birth do not exhibit decreased colonization of intestinal and airway microbiota.

**Figure 2:**
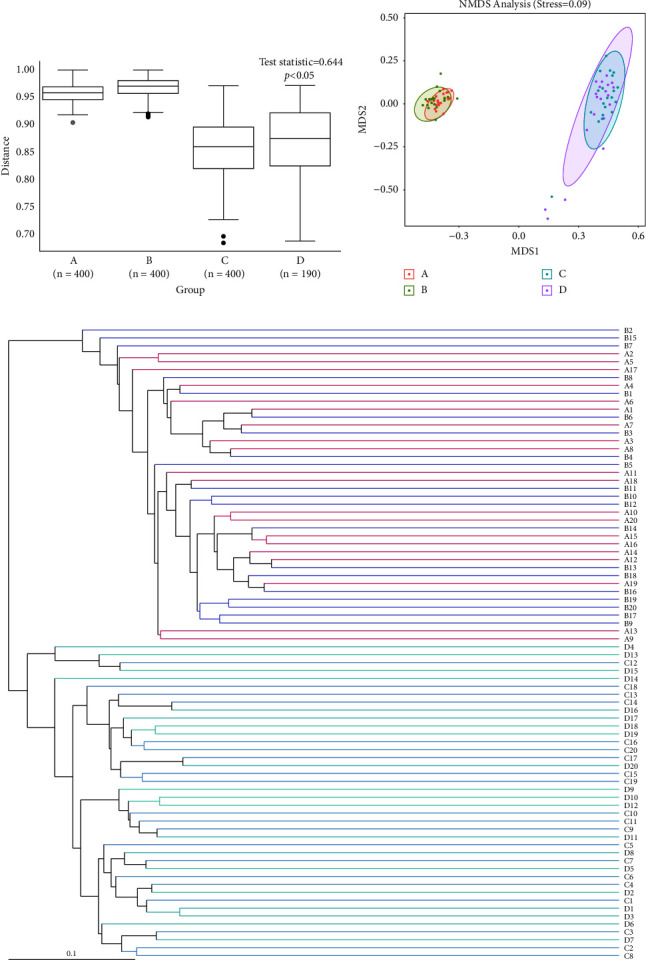
The statistic values between −1 and 1 were evaluated using ANOSIM. Values close to 1 exhibited a greater sample difference among the groups and among samples within the group (a). In the NMDS plot, the points represent samples, different colors represent different groups, and the distance between points represents the degree of difference between samples. Stress was used to measure the merit of the NMDS results ((b): stress <0.1 indicated a good ranking). In the UPGMA clustering diagram, the branches of different colors represent different groups ((c): the shorter the branch, the more the similarities). *P* < 0.05 is statistically significant.

Based on the Jaccard algorithm, ANOSIM, NMDS, and UPGMA beta-diversity analyses were performed. ANOSIM revealed that between-group differences were higher than within-group differences (Figure 2(a)). NMDS used the ranking method of the sample distance matrix, and dimensionality reduction was calculated (Figure 2(b)). Based on the results of ASV analysis of each sample, the coefficient of dissimilarity between samples was measured using the Jaccard index in the UPGMA method of clustering (Figure 2(c)). Similar clustering patterns were seen within the airway and intestinal tract samples. In general, there were similarities between the intestinal flora as well as the airway flora of twins and singletons.

**Figure 3:**
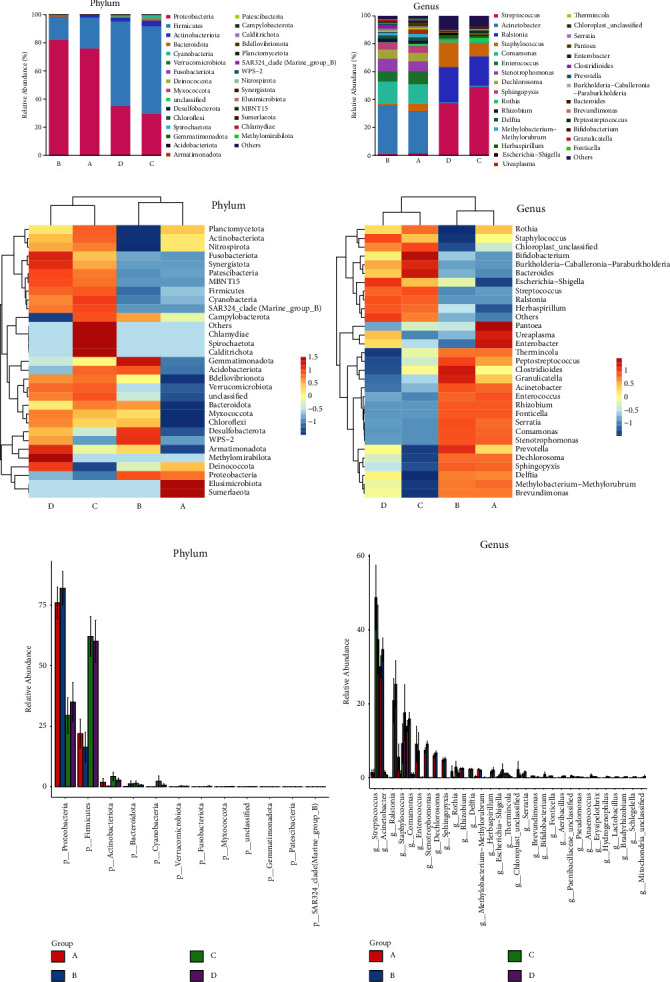
In each level of the stacked bar diagram, the dominant phylum and genus levels of bacteria are displayed ((a, b): the horizontal axis shows sample grouping, and the vertical axis shows relative abundance. See supplementary materials for order, family, and species data). Bacterial colony composition is clustered according to the relative abundance of species at phylum and genus levels in the heat maps ((c, d): rows represent species, columns represent sample groups, and gradient colors from blue to red represent variations in abundance from low to high. The heat map was converted by *z* values, so boxes can only be compared horizontally). The phylum and genus levels were analyzed for differences; the abscissa in the figure represents the different species (ranging from left to right according to the abundance), and the ordinate represents the relative abundance (e, f).

We created stacked bar plots of groups A–D for abundance at the phylum, order, family, genus, and species levels. We constructed heat maps displaying the top 30 compositions for relative abundance.  Table 2 presents the top genera and phyla. *Acinetobacter*, *Comamonas*, and *Enterococcus* were relatively more abundant in the gut, whereas *Streptococcus*, *Ralstonia*, and *Staphylococcus* were more abundant in the airways of both singletons and twins. The principal genera were observed in the airways and intestinal tracts in different proportions. Phylum (Figures 3(a), 3(c) and 3(e)) and genus (Figures 3(b), 3(d) and 3(f)) level differences determined using the Kruskal–Wallis test are shown in Figure 3 (for class, order, family, and species level differences, refer to Supplementary Material 3).

**Figure 4:**
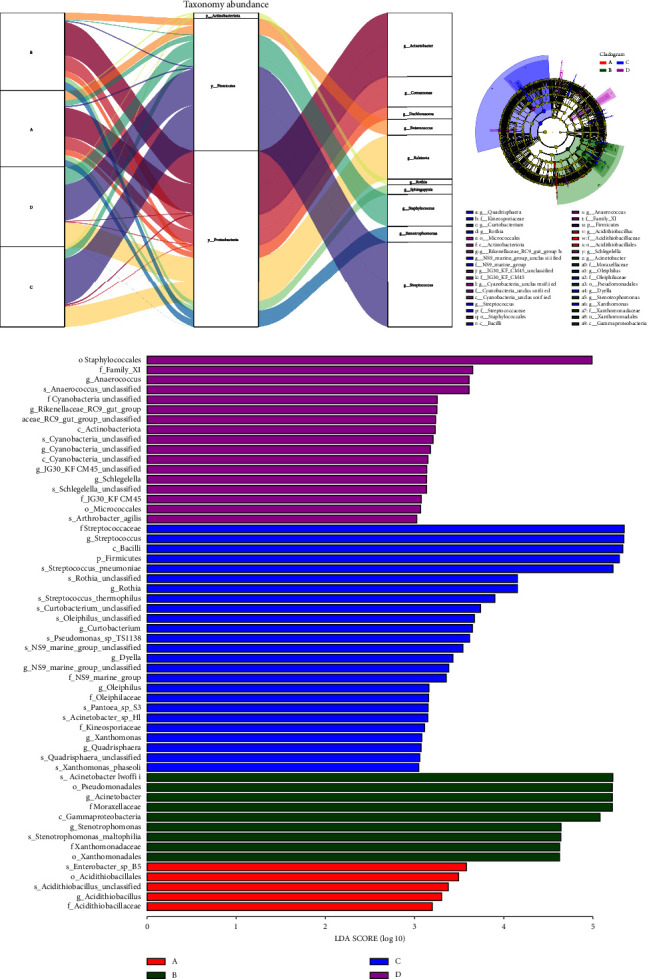
The Sankey diagram shows the relative abundance of bacteria at the phylum (middle) and genus levels (right) corresponding to the different groups (left) (a). The species annotation information and proportion of the bacteria at the phylum and genus levels (b). The linear discriminant analysis score between the four groups (c).

Figure 4(a) shows the relative abundance of bacterial groups at the phylum and genus levels. LDA effect size was used to detect variability among the subgroups using the rank-sum test and obtain differential species (biomarkers) (Figure 4(b)) and their effect sizes. *Acidithiobacillus* in group A, *Acinetobacter* and *Stenotrophomonas* in group B, *Streptococcus*, *Rothia*, *Curtobacterium*, and *Dyella* in group C, and *Anaerococcus* in group D were the ultimate potential species of difference (Figure 4(c)).

**Figure 5:**
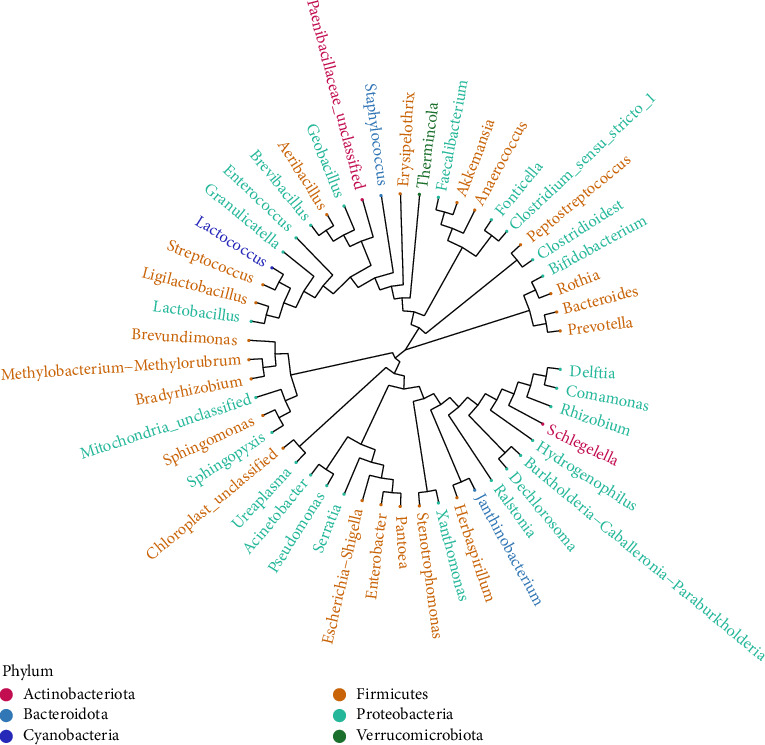
The different branches in the evolutionary tree diagram represent different genera classified horizontally. Different genera of the same color belong to the same phylum, and the closer two species are to each other, the closer the evolutionary relationship between them (a).

Species evolutionary trees were constructed for *Streptococcus* and *Lactococcus*, *Acinetobacter* and *Pseudomonas*, and *Ralstonia* and *Dechlorosoma* using signature sequences. *Comamonas* and *Delftia* exhibited a similar evolutionary relationship (Figure 5).

**Figure 6:**
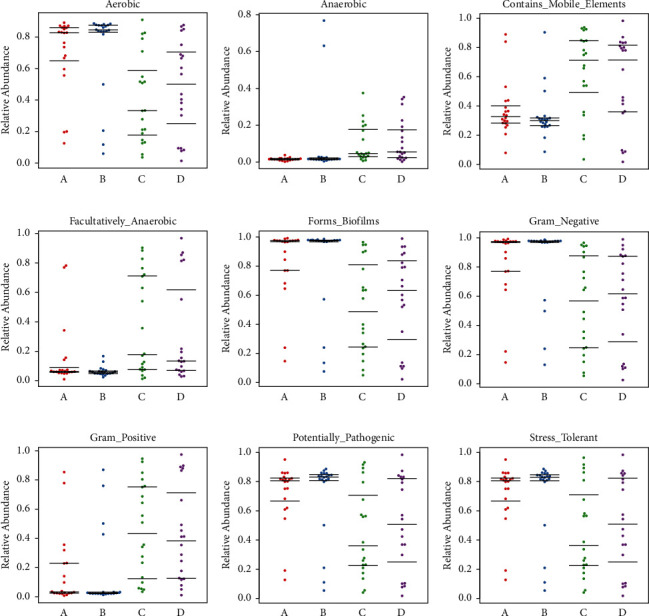
The scatter plot shows the relative abundance of the bacterial phenotypes of each sample according to the different groups (a–i). Each dot represents one sample, the abscissa represents the group, and the ordinate shows the relative abundance of species of different phenotypes in each sample.

**Figure 7:**
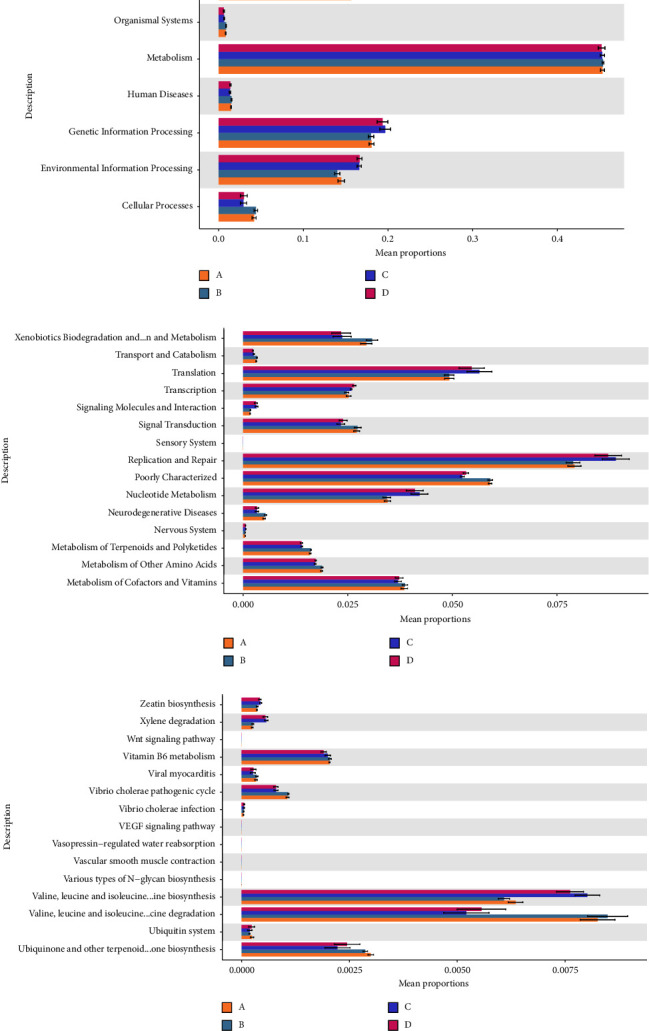
In the bacterial function prediction map, different colors represent different groups, which in turn provide the result of the metabolic pathway of bacteria at levels 1 (a), 2 (b), and 3 (c). Multigroup comparison only shows richness.

Preterm twins and singletons have lower abundances of anaerobic bacteria in their airways and guts. Premature newborns have a high proportion of Gram-negative bacteria in the digestive tract (Figures 6(a)–6(i)). Sample gene sequences were annotated using the KEGG database on metabolic pathways based on their metabolic function prediction using PICRUSt2. Metabolism, genetic and environmental information processing, cellular processes, and human diseases were the main processes in level 1. In level 3, the main pathways were valine, leucine, and isoleucine degradation and biosynthesis, terpenoid-quinone biosynthesis, and vitamin B6 metabolism (Figures 7(a)–7(c), Supplementary Material 4).

## 4. Discussion

This study determined the diversity and community structure in the bacterial microbiome of the airways and intestines of preterm twin and singleton neonates. The patient characteristics between twin and singleton neonates were not significantly different. Alpha-diversity analyses showed that bacterial colonies were more abundant and homogeneous in the intestine than in the airway, likely because gut microorganism homeostasis is more stable as fetal stool samples are expelled less frequently. The neonates did not exhibit significant differences in intestinal or airway bacterial colonies, suggesting that the diversity and richness of the initially colonized bacteria were similar. Therefore, the differences in the flora of adult twins are most likely due to acquired factors [20]. Beta-diversity analyses demonstrated that between-group differences were higher than within-group differences.

A consistent finding of many previous studies is the reduced abundance of bifidobacteria, anaphylactic bacteria, and lactobacilli in infants born by cesarean section compared to those born vaginally, with their intestinal flora composition similar to microorganisms in the maternal skin and hospital environment [21–23]. Different birth modes may result in altered immune functions, with reduced proportions of regulatory T cells and downregulation of the regulatory markers Foxp3, Il10, and Ctla4 in cesarean-born mice, which may suggest that preterm infants born by cesarean section are more susceptible to allergic diseases [24, 25]. Our study revealed that the dominant intestinal phylum and genera were Proteobacteria and *Acinetobacter* and *Comamonas*, respectively. This finding differs from that reported by Turunen that the dominant intestinal phylum and genera in full-term neonates were Firmicutes and *Lactobacillus* and *Streptococcus*, respectively [26]. Herein, the most abundant phylum and genera in the airways were Firmicutes and *Streptococcus* and *Ralstonia*, respectively. *Streptococcus* has previously been reported to be the single dominant genus [27]. Interestingly, however, it has also been shown that the microbiomes of fetal feces and airways are influenced by placental microorganisms regardless of the mode of delivery [3, 28]. Bacterial species are similar in the airways and intestines. In this study, the dominant genus in fecal samples was *Acinetobacter*, which Doyle et al. reported as the dominant genus in the placenta [29]. Although we chose to collect airway specimens aseptically, contamination is possible because the airway microbiome includes inhaled microbiota. The impact of various causes of premature birth on colonization was not evaluated but will be the focus of our next study.

The preterm infants in the study were breastfed. Breastmilk is one of the primary sources of gut bacteria in infants. Infants consuming approximately 800 ml of breastmilk ingest approximately 1 × 10^5^ to 1 × 10^7^ bacteria. Newborns receive colostrum from breastmilk, which results in a more complex intestinal bacterial colony structure and contributes to a more complete and stable intestinal immune system [30]. However, in this study, we did not sequence the microflora of breastmilk; we plan to investigate this in the future.

The similarity of the microbiomes of preterm single and twin births at the genus level may suggest that the initial neonatal flora colonization occurs more often during and shortly before delivery. It has been emphasized that evidence for the presence of the placental microbiome is insufficient in the case of either physiological delivery or spontaneous preterm birth because of the high potential for contamination during sample collection [31]. Furthermore, during the course of life, the microbiome constantly adapts and dynamically responds to external stressor events to ensure homeostasis *in vivo*. A follow-up study on 903 children showed that gut microbial development is divided into developmental, transitional, and stable phases [32]. A study of premature twins reported that the reduced abundance of *Enterococcus* and *Fusobacterium* may downregulate methionine and cysteine levels, leading to excessive oxidative stress and low levels of 1-C metabolism [33]. Dysbiosis of microbial flora may be associated with bronchopulmonary dysplasia, inadequate colonization of the intestinal flora in preterm infants with delayed immune development, and inflammatory diseases (necrotizing small bowel colitis) [27, 34]. This highlights the need for neonatal microecological correction in preterm infants, both in twins and singletons. There are studies on transplantation of maternal fecal microorganisms to rapidly restore normal gut microorganisms in infants born by cesarean section [35]; however, there have not yet been any reports on the transplantation of airway microorganisms, and related studies may serve as stepping stones for future early intervention strategies to place infants on a healthy trajectory.

We observed that most intestinal bacteria were Gram-negative with potential pathogenicity and a high stress-tolerance capacity. Predicting intestinal and airway bacterial phenotypes can help clinicians manage infections in preterm newborns. The onset of intestinal function after birth may also impact the intestinal flora.

The catabolism and synthesis of valine, leucine, and isoleucine, biosynthesis of terpene quinones, overexpression of vitamin B6, and metabolism of *Vibrio cholerae* pathogenic cycles were the primary pathways in level 3. These findings suggest the presence of functional microorganisms in preterm neonates. Intestinal bacteria catabolized the three amino acids more but synthesized them less than airway bacteria. These amino acids are critical for regulating protein synthesis and metabolism but cannot be synthesized endogenously in mammals. They are obtained only through diet, and their deficiency is associated with sarcopenia, obesity, and insulin and glucose metabolism [36]. The biosynthetic pathways in gut microbes are dominated by the degradation of the three amino acids in children compared to their synthesis in adults, suggesting that metabolic pathways change with age [37]. Vitamin B6 is a water-soluble vitamin involved in several metabolic reactions, particularly those involving amino acids. Vitamin B6 and probiotic treatment are known to alleviate the symptoms of lactose intolerance and functional gastrointestinal disorders in patients, improving their intestinal microbiota and metabolism [38]. These findings suggest that colonized bacteria undergo robust replication and repair, engage in nutrient exchange with the host, and play a vital role in maintaining the immune stability of the microenvironment. Because we did not test the amniotic fluid or placenta for bacteria or perform detailed proteomic assays on the samples, changes in metabolic pathways were projected using sequencing data, and their accuracy should be verified.

## 5. Conclusions

The richness of the intestinal and airway flora was not significantly different between twins and singletons, and the flora clustered together. The species involved in initial colonization of the intestine and airway were similar in twins and singletons but differed in proportions. This may indicate that colonization of the intestinal and airway bacteria may occur at the time of delivery or shortly before delivery. Therefore, changes in microbial structure and richness may not be attributed to these species. Dysbiosis of microflora may be associated with bronchopulmonary dysplasia, necrotizing enterocolitis, and allergic diseases, and early correction of the initial colonizing microorganisms in preterm infants may prevent the occurrence of adverse outcomes in preterm infants. Correction regarding airway microecology remains a challenge at present.

## Figures and Tables

**Table 1:** Sample characteristics of 10 pairs of twins and 20 singleton newborns.

	A (*n* = 20)	B (*n* = 20)	Statistics	*P*
Sex (male/female)	6/14	7/13	—	0.736^a^
Birth method (vaginal delivery/cesarean section)	2/18	4/16	—	0.661^a^
Gestational week	34.30 ± 0.80	33.95 ± 1.00	1.222	0.229^b^
Apgar score (1 min)	9.05 ± 0.69	9.00 ± 0.80	0.213	0.833^b^
Weight (kg)	2.17 ± 0.19	2.20 ± 0.43	−0.304	0.763^b^
Length (cm)	43.85 ± 1.63	44.30 ± 2.13	−0.750	0.458^b^
Head circumference (cm)	31.43 ± 1.20	31.38 ± 1.87	0.091	0.928^b^
Number of breaths (times/min)	46.90 ± 5.75	48.70 ± 6.66	−0.915	0.366^b^
Blood oxygen saturation (%)	96.50 ± 1.00	95.10 ± 2.31	2.483	0.200^b^
White blood cells (10^9^/L)	10.15 ± 2.55	11.97 ± 4.67	−1.525	0.136^b^
Absolute neutrophil count (10^9^/L)	5.17 ± 2.23	6.93 ± 5.05	−1.424	0.163^b^
Age of the father (years)	32.80 ± 2.63	29.25 ± 4.73	2.932	0.060^b^
Age of the mother (years)	30.40 ± 3.32	28.35 ± 4.39	1.666	0.104^b^
Number of pregnancies (times)	2.00 ± 1.03	1.90 ± 0.97	0.317	0.753^b^
Number of births (times)	1.80 ± 0.70	1.50 ± 0.51	1.552	0.129^b^

Significant differences in continuous and categorical variables were examined using student' *t*-test and *chi*-squared test, respectively (^*∗*^*P* < 0.05). ^a^Values shown for categorical variables are the number. ^b^Values shown for continuous variables are the mean ± SD.

**Table 2:** Characterization of major bacterial distributions at phylum and genus levels based on relative taxonomic abundance.

Phyla	A (%)	B (%)	C (%)	D (%)
Proteobacteria	76.01	81.98	29.48	34.99
Firmicutes	21.95	16.37	62.07	60.05
Actinobacteriota	1.87	0.25	4.26	2.74
Bacteroidota	0.11	1.25	1.38	0.72
Cyanobacteria	0.01	0.00	2.30	0.71

Genera	A (%)	B (%)	C (%)	D (%)

*Streptococcus*	1.45	1.21	48.76	37.41
*Acinetobacter*	30.04	34.70	1.44	0.67
*Ralstonia*	0.02	0.02	20.86	25.29
*Staphylococcus*	5.54	1.00	9.28	17.57
*Comamonas*	13.96	15.94	0.89	0.92
*Enterococcus*	9.12	7.28	0.09	0.14
*Stenotrophomonas*	7.38	9.12	0.04	0.05
*Dechlorosoma*	5.99	6.71	0.00	0.00
*Sphingopyxis*	4.79	5.06	0.00	0.00
*Rothia*	1.73	0.11	2.88	1.32

## Data Availability

All data generated or analyzed in this study are included in this published article and its supplementary files.

## Supplementary Materials

Supplementary Materials Supplementary Material 1: sequence information identified in singleton and twin preterm neonates. Supplementary Material 2: abundance of bacterial taxa and corresponding 16SrRNA gene sequences in singleton and twin preterm neonates. Supplementary Material 3: class-, order-, family-, and species-level differences were determined using the Kruskal–Wallis test. Supplementary Material 4: three levels of annotation in the KEGG database on metabolic pathways.
