# Supplementary material for: Bacterial Colonization in the Airways and Intestines of Twin and Singleton Preterm Neonates: A Single-Center Study
Source: Can J Infect Dis Med Microbiol. 2023 Aug 1;2023:2973605. doi: 10.1155/2023/2973605 (PMC10409585; doi:10.1155/2023/2973605)
Supplement: Supplementary Materials — Supplementary Material 1: sequence information identified in singleton and twin preterm neonates. Supplementary Material 2: abundance of bacterial taxa and corresponding 16SrRNA gene sequences in singleton and twin preterm neonates. Supplementary Material 3: class-, order-, family-, and species-level differences were determined using the Kruskal–Wallis test. Supplementary Material 4: three levels of annotation in the KEGG database on metabolic pathways. [file 2973605.f1.zip › Supplementary Material 1 Sequence information.docx]

**Sequence information identified in singleton and twin preterm neonates.**

Paired-end reads were assigned to the samples using their unique barcodes and truncated by cutting off the barcode and primer sequences. They were merged using FLASH for 16S rRNA and PEAR for ITS2 rDNA. Raw reads were quality filtered to obtain high-quality clean tags using fqtrim. Chimeric sequences were filtered using VSEARCH software. After dereplication using DADA2, amplicon sequence variants (ASVs) were employed to build operational taxonomic units.

| **Sample** | **Raw_Tags** | **Raw_Bases** | **Valid_Tags** | **Valid_Bases** | **Valid%** | **Q20%** | **Q30%** | **GC%** |
| --- | --- | --- | --- | --- | --- | --- | --- | --- |
| **B1** | **80425** | **34.28M** | **80425** | **34.28M** | **100.00** | **98.08** | **93.86** | **52.41** |
| **A1** | **78221** | **33.21M** | **78221** | **33.21M** | **100.00** | **98.69** | **95.69** | **52.86** |
| **A2** | **80190** | **34.19M** | **80190** | **34.19M** | **100.00** | **98.58** | **95.39** | **55.83** |
| **B2** | **70241** | **29.78M** | **70241** | **29.78M** | **100.00** | **98.47** | **95.12** | **53.64** |
| **A3** | **80561** | **34.16M** | **80561** | **34.16M** | **100.00** | **98.60** | **95.48** | **53.07** |
| **A4** | **78372** | **33.39M** | **78372** | **33.39M** | **100.00** | **98.41** | **94.81** | **47.04** |
| **A5** | **74089** | **31.17M** | **74089** | **31.17M** | **100.00** | **98.47** | **95.20** | **52.62** |
| **A6** | **79733** | **33.91M** | **79733** | **33.91M** | **100.00** | **98.49** | **95.16** | **52.76** |
| **B3** | **82972** | **35.19M** | **82972** | **35.19M** | **100.00** | **98.10** | **93.79** | **53.17** |
| **B4** | **79088** | **33.53M** | **79088** | **33.53M** | **100.00** | **98.76** | **95.83** | **53.06** |
| **B5** | **62244** | **26.45M** | **62244** | **26.45M** | **100.00** | **91.54** | **81.15** | **53.47** |
| **B6** | **78136** | **33.13M** | **78136** | **33.13M** | **100.00** | **98.35** | **94.74** | **53.00** |
| **B7** | **81475** | **34.63M** | **81475** | **34.63M** | **100.00** | **98.22** | **94.38** | **52.98** |
| **B8** | **83465** | **35.38M** | **83465** | **35.38M** | **100.00** | **98.64** | **95.57** | **53.05** |
| **A7** | **80692** | **34.19M** | **80692** | **34.19M** | **100.00** | **97.57** | **92.52** | **53.19** |
| **A8** | **80494** | **34.12M** | **80494** | **34.12M** | **100.00** | **98.59** | **95.40** | **53.02** |
| **A9** | **78439** | **33.16M** | **78439** | **33.16M** | **100.00** | **96.20** | **89.77** | **53.30** |
| **A10** | **71750** | **30.39M** | **71750** | **30.39M** | **100.00** | **95.56** | **88.40** | **53.29** |
| **B9** | **59449** | **25.30M** | **59449** | **25.30M** | **100.00** | **94.75** | **86.66** | **52.98** |
| **A11** | **75229** | **32.09M** | **75229** | **32.09M** | **100.00** | **95.55** | **88.28** | **52.34** |
| **A12** | **68710** | **29.07M** | **68710** | **29.07M** | **100.00** | **95.54** | **88.17** | **53.41** |
| **B10** | **69404** | **29.41M** | **69404** | **29.41M** | **100.00** | **95.34** | **87.93** | **53.31** |
| **B11** | **71294** | **30.27M** | **71294** | **30.27M** | **100.00** | **95.54** | **88.40** | **53.01** |
| **B12** | **78437** | **33.23M** | **78437** | **33.23M** | **100.00** | **95.94** | **89.19** | **53.00** |
| **A13** | **77955** | **33.01M** | **77955** | **33.01M** | **100.00** | **95.99** | **89.25** | **53.19** |
| **A14** | **63681** | **26.95M** | **63681** | **26.95M** | **100.00** | **95.01** | **87.18** | **53.51** |
| **B13** | **63283** | **26.84M** | **63283** | **26.84M** | **100.00** | **95.16** | **87.48** | **53.27** |
| **B14** | **68916** | **29.20M** | **68916** | **29.20M** | **100.00** | **95.46** | **88.03** | **53.23** |
| **B15** | **69848** | **29.28M** | **69848** | **29.28M** | **100.00** | **95.75** | **88.69** | **51.96** |
| **A15** | **59690** | **25.32M** | **59690** | **25.32M** | **100.00** | **94.86** | **86.93** | **52.97** |
| **A16** | **64775** | **27.53M** | **64775** | **27.53M** | **100.00** | **94.98** | **87.18** | **52.96** |
| **A17** | **79931** | **33.83M** | **79931** | **33.83M** | **100.00** | **96.28** | **89.93** | **53.19** |
| **A18** | **64862** | **27.53M** | **64862** | **27.53M** | **100.00** | **94.37** | **85.85** | **53.17** |
| **B16** | **64473** | **27.33M** | **64473** | **27.33M** | **100.00** | **94.41** | **85.87** | **53.30** |
| **B17** | **57338** | **23.70M** | **57338** | **23.70M** | **100.00** | **94.37** | **85.59** | **52.93** |
| **B18** | **66137** | **28.05M** | **66137** | **28.05M** | **100.00** | **94.05** | **85.13** | **53.28** |
| **B19** | **64829** | **27.50M** | **64829** | **27.50M** | **100.00** | **93.99** | **85.00** | **53.31** |
| **A19** | **66808** | **28.34M** | **66808** | **28.34M** | **100.00** | **94.42** | **85.99** | **53.13** |
| **A20** | **63248** | **26.93M** | **63248** | **26.93M** | **100.00** | **94.23** | **85.72** | **51.92** |
| **B20** | **67144** | **28.50M** | **67144** | **28.50M** | **100.00** | **93.17** | **83.57** | **53.42** |
| **D20** | **78572** | **33.49M** | **78572** | **33.49M** | **100.00** | **94.52** | **86.46** | **51.47** |
| **C20** | **76030** | **32.19M** | **76030** | **32.19M** | **100.00** | **95.16** | **87.67** | **52.77** |
| **C19** | **72651** | **30.75M** | **72651** | **30.75M** | **100.00** | **96.03** | **90.14** | **52.57** |
| **D19** | **79191** | **33.77M** | **79191** | **33.77M** | **100.00** | **95.63** | **89.05** | **51.23** |
| **D18** | **79902** | **34.08M** | **79902** | **34.08M** | **100.00** | **95.82** | **89.69** | **51.22** |
| **D17** | **73445** | **31.33M** | **73445** | **31.33M** | **100.00** | **96.21** | **90.44** | **50.54** |
| **D16** | **72162** | **30.77M** | **72162** | **30.77M** | **100.00** | **95.90** | **89.82** | **50.99** |
| **C18** | **76732** | **31.83M** | **76732** | **31.83M** | **100.00** | **96.30** | **90.53** | **54.24** |
| **C17** | **77939** | **33.23M** | **77939** | **33.23M** | **100.00** | **94.49** | **86.30** | **51.40** |
| **C16** | **76404** | **32.59M** | **76404** | **32.59M** | **100.00** | **95.40** | **88.35** | **51.29** |
| **C15** | **78207** | **33.34M** | **78207** | **33.34M** | **100.00** | **95.98** | **90.13** | **51.27** |
| **D15** | **61439** | **26.02M** | **61439** | **26.02M** | **100.00** | **95.93** | **89.72** | **53.66** |
| **D14** | **72945** | **30.87M** | **72945** | **30.87M** | **100.00** | **95.88** | **89.64** | **53.33** |
| **D13** | **74806** | **31.57M** | **74806** | **31.57M** | **100.00** | **95.76** | **89.20** | **53.24** |
| **C14** | **75002** | **31.98M** | **75002** | **31.98M** | **100.00** | **95.73** | **89.49** | **51.25** |
| **C13** | **73905** | **31.51M** | **73905** | **31.51M** | **100.00** | **96.28** | **90.59** | **50.76** |
| **D12** | **73495** | **31.33M** | **73495** | **31.33M** | **100.00** | **95.36** | **88.24** | **50.95** |
| **D11** | **78453** | **33.45M** | **78453** | **33.45M** | **100.00** | **95.44** | **88.32** | **51.30** |
| **D10** | **76683** | **32.67M** | **76683** | **32.67M** | **100.00** | **96.19** | **90.46** | **51.33** |
| **C12** | **74131** | **31.56M** | **74131** | **31.56M** | **100.00** | **94.44** | **86.28** | **53.62** |
| **C11** | **78822** | **33.60M** | **78822** | **33.60M** | **100.00** | **96.16** | **90.37** | **51.28** |
| **D9** | **72797** | **30.82M** | **72797** | **30.82M** | **100.00** | **96.06** | **89.97** | **53.12** |
| **C10** | **75585** | **32.16M** | **75585** | **32.16M** | **100.00** | **95.62** | **89.10** | **53.42** |
| **C9** | **75629** | **32.14M** | **75629** | **32.14M** | **100.00** | **95.59** | **88.96** | **53.49** |
| **C8** | **68316** | **28.89M** | **68316** | **28.89M** | **100.00** | **95.31** | **88.23** | **53.54** |
| **C7** | **78798** | **33.59M** | **78798** | **33.59M** | **100.00** | **95.53** | **88.63** | **51.11** |
| **D8** | **72947** | **31.07M** | **72947** | **31.07M** | **100.00** | **95.30** | **88.32** | **51.46** |
| **D7** | **73213** | **31.00M** | **73213** | **31.00M** | **100.00** | **96.16** | **90.21** | **53.92** |
| **D6** | **75657** | **31.80M** | **75657** | **31.80M** | **100.00** | **95.86** | **89.55** | **52.91** |
| **D5** | **74377** | **31.51M** | **74377** | **31.51M** | **100.00** | **96.07** | **89.84** | **53.41** |
| **D4** | **68388** | **29.15M** | **68388** | **29.15M** | **100.00** | **90.50** | **78.75** | **54.23** |
| **D3** | **80171** | **34.15M** | **80171** | **34.15M** | **100.00** | **96.08** | **90.15** | **50.91** |
| **C6** | **73842** | **31.06M** | **73842** | **31.06M** | **100.00** | **95.93** | **89.65** | **53.65** |
| **C5** | **77858** | **33.17M** | **77858** | **33.17M** | **100.00** | **95.34** | **88.10** | **51.41** |
| **C4** | **77093** | **32.79M** | **77093** | **32.79M** | **100.00** | **96.30** | **90.69** | **52.82** |
| **C3** | **64023** | **26.81M** | **64023** | **26.81M** | **100.00** | **96.07** | **89.96** | **52.44** |
| **D2** | **73275** | **30.84M** | **73275** | **30.84M** | **100.00** | **96.41** | **90.71** | **53.63** |
| **C2** | **70993** | **30.24M** | **70993** | **30.24M** | **100.00** | **95.85** | **89.65** | **52.82** |
| **C1** | **73398** | **31.26M** | **73398** | **31.26M** | **100.00** | **95.74** | **89.46** | **52.31** |
| **D1** | **72467** | **30.69M** | **72467** | **30.69M** | **100.00** | **96.02** | **90.04** | **52.43** |
