# Supplementary material for: Bacterial Colonization in the Airways and Intestines of Twin and Singleton Preterm Neonates: A Single-Center Study
Source: Can J Infect Dis Med Microbiol. 2023 Aug 1;2023:2973605. doi: 10.1155/2023/2973605 (PMC10409585; doi:10.1155/2023/2973605)
Supplement: Supplementary Materials — Supplementary Material 1: sequence information identified in singleton and twin preterm neonates. Supplementary Material 2: abundance of bacterial taxa and corresponding 16SrRNA gene sequences in singleton and twin preterm neonates. Supplementary Material 3: class-, order-, family-, and species-level differences were determined using the Kruskal–Wallis test. Supplementary Material 4: three levels of annotation in the KEGG database on metabolic pathways. [file Supplementary Material 2 ASV_table_with_taxonomy.pdf › Supplementary Material 2 ASV_table_with_taxonomy.pdf]

Species richness at phylum, order, family, genus, and species levels was obtained based on ASV annotation results and abundance table for each sample.

Exhibit 2. Abundance of bacterial taxa and corresponding 16SrRNA gene sequences for preterm neonates with single and twin births.

ASV ID	B1	A1	A2	B2	A3	A4
d0ab2c15400fe710288526c9a33083fb	122.00	0.00	0.00	0.00	116.00	10.00
b53457a0d3257bd9c3f27474dd074280	2804.00	21943.00	4888.00	17273.00	32177.00	11724.00
9f22da56e873ade2c09013cae621e715	7.00	6.00	0.00	50.00	28.00	21.00
b87db8a77db577f4217e4ea1ad190467	1200.00	9617.00	1736.00	21520.00	12898.00	3116.00
22cdeb3583258b9c420eb6b150b2a95c	613.00	4943.00	1000.00	4702.00	7889.00	3154.00
9158ab694988e84ec137cfaee88fcd2a	66683.00	8393.00	12.00	849.00	0.00	23.00
117ee8cc9921b631c9e1da435988e482	0.00	0.00	9.00	2.00	220.00	9.00
7abd9c7fdb376cea66a886bd93331c84	669.00	4884.00	645.00	3949.00	5128.00	2787.00
f0bde2f54893276b9ba2239c532b706a	323.00	7668.00	22.00	31.00	23.00	18.00
6f6c21347601c3e7f8cc2f7d046a2945	480.00	5003.00	856.00	5153.00	5489.00	1312.00
17a1d377f12b89a41e9312072db9a830	0.00	0.00	0.00	0.00	119.00	0.00
be9e7e2786b3ae134712e7b6bedc2259	10.00	715.00	0.00	0.00	0.00	0.00
adcdf6b293cb136520947d9e8372efe8	1620.00	390.00	3.00	6.00	9.00	11.00
c3819d8a7a0b47c1589ab0a9ffd74ca8	212.00	1500.00	236.00	1897.00	1821.00	619.00
c7615cfa79a022b2c15a870729718ca6	193.00	1713.00	244.00	1882.00	2047.00	738.00
68d23eeb14390d27e1039c8a8a2120c4	239.00	1732.00	353.00	2258.00	2151.00	572.00
b22c20766a485c6c33611162cd2c712d	0.00	0.00	0.00	0.00	0.00	0.00
37e9605fff775ec9bd4f333e32d52bd3	105.00	865.00	435.00	919.00	2567.00	786.00
591ed1d6afd1e79ae33d6b6cd0c6b185	50.00	81.00	38.00	43.00	113.00	62.00
ac5da2c3752edbcf9ea1781564d1eaac	0.00	67.00	0.00	0.00	0.00	0.00
fa767a4f0a3504815eb7c9fffa6a4433	0.00	0.00	28076.00	0.00	0.00	20.00
f959e9b73713a6728bde710156d58a08	0.00	0.00	5.00	14.00	0.00	25780.00
4fad55daa352356af40293871c2a8150	0.00	0.00	0.00	0.00	0.00	0.00
2e35dc6f89afe48f93be66d924b2db2b	158.00	1111.00	148.00	744.00	994.00	486.00
7e025abcd45c6c965bc125dc918d8d4f	7.00	9.00	0.00	0.00	12.00	23093.00
07ffc6ebabe04c7c960c5d345b3cb413	0.00	0.00	0.00	0.00	0.00	0.00
0891f77ff7374eb908d4d39dd6269daf	0.00	14.00	19480.00	0.00	18.00	0.00
baedcb79a8c5d41784ccc49d578d1545	138.00	198.00	102.00	405.00	754.00	477.00
1ec0db01c02665365280803a7037c14c	405.00	1604.00	0.00	171.00	0.00	0.00
944d454586bd0b71c8e83a0c582d40b4	0.00	0.00	0.00	0.00	0.00	0.00
49e57a0f8791fcdfdb36e4b80ce95dd1	0.00	0.00	0.00	0.00	0.00	0.00
a373d7bae015c12ce145a54872026fcb	458.00	0.00	0.00	0.00	0.00	0.00
e557f5545e323ea31149e29c01487407	0.00	323.00	0.00	0.00	0.00	0.00
ec7e92260f7251a68c82e786209fdcc2	0.00	0.00	0.00	0.00	0.00	0.00
302dc2e085ca4c6a51f27120315a28ca	0.00	0.00	0.00	0.00	0.00	0.00

0f6d46c70548d4ce709802b6e3162f6f	0.00	0.00	0.00	0.00	0.00	0.00
520f73051aeff21fefce0e79bb755fea	0.00	0.00	0.00	0.00	0.00	0.00
70933c76e5b6599fb610ff22c51afe56	0.00	0.00	0.00	0.00	0.00	0.00
7d00b639fb766cbf72f35eb8d9ba62df	0.00	0.00	9375.00	0.00	0.00	0.00
9fa545899131066bfe60e11645b38db4	0.00	0.00	0.00	0.00	0.00	0.00
becd8488d5c722efbb395eea7aa2b7a2	0.00	0.00	0.00	0.00	0.00	0.00
d3a223a1c12ed0c8f8e5ce5fe7e7e135	14.00	0.00	8475.00	0.00	0.00	0.00
37a8655b025334ad3da09e7fd4d58575	0.00	0.00	0.00	5.00	6.00	0.00
ac4c75479e1f2bce939ba8a839344c08	0.00	0.00	0.00	0.00	0.00	0.00
bfd4d446a5b960b55647a573d459ff58	0.00	0.00	0.00	0.00	0.00	0.00
b17271634405e797b345cb3b74780f3d	0.00	0.00	0.00	0.00	0.00	0.00
bcecf8ec1e9a85a4435046cd2d6f2d31	8.00	102.00	40.00	140.00	164.00	25.00
b15abeb3a1529af6232a1768a0c9ba9c	0.00	0.00	0.00	0.00	0.00	0.00
8ed5ff033d373cd7d2936c3f0ee1f3b4	0.00	13.00	11.00	0.00	0.00	0.00
4bcb09d6bd179a07c4fa327dab97d982	0.00	0.00	0.00	0.00	0.00	0.00
6c5cdeb6d6d67b1ba6d7a1c00852f627	0.00	0.00	0.00	0.00	0.00	0.00
c5894f9ace461ff1cf8031e5a2dc5902	23.00	204.00	60.00	117.00	311.00	44.00
6101d0c2746d60ba2c9671a4b2115c75	0.00	0.00	0.00	0.00	0.00	0.00
efc93483c808aade1c291fa653561f82	39.00	153.00	30.00	126.00	206.00	68.00
8b4b6cc793176122066b60900b469f64	24.00	200.00	66.00	165.00	208.00	41.00
3754bfa11bbdf1a54fb9d27defa5f45d	0.00	69.00	0.00	0.00	0.00	0.00
4b38473099dc5f18b73be535e282921a	0.00	0.00	0.00	0.00	0.00	0.00
cf47e69993c825fff20f7679e4b47c75	0.00	0.00	0.00	0.00	0.00	0.00
4cdf36c21adce2ca6150ad40f3a0fb7d	0.00	0.00	0.00	0.00	0.00	0.00
d2b3f7a7842df325eb9c80bcea240910	0.00	0.00	0.00	0.00	0.00	3.00
c204d4af5ac40400a9cd069734cc1389	0.00	0.00	0.00	0.00	0.00	0.00
6341b3358de97a3d5124cb15b0fb2dca	0.00	0.00	0.00	0.00	0.00	0.00
f8b81db4ed586a72102c46c9b47d6835	0.00	0.00	0.00	0.00	0.00	0.00
49fd865ca92f0bf1d388bba966628c5d	34.00	40.00	29.00	70.00	191.00	141.00
b2b048d66c408d1b92548956dd3d4502	14.00	156.00	22.00	55.00	189.00	45.00
f7e4d6e9e0a0370a86c8c5fd06c3abbb	0.00	0.00	0.00	0.00	0.00	0.00
fa2dbb002847db57f81ec9a54892d455	0.00	0.00	0.00	0.00	0.00	0.00
e5400356daabbc5a41f935af70513043	0.00	0.00	0.00	0.00	0.00	0.00
12a1485f502111a8f0c31727445ec2a6	22.00	0.00	0.00	75.00	124.00	137.00
ce29d072275aed555a8ab172b731f6f2	0.00	0.00	0.00	0.00	0.00	0.00
dc2f672520a7fb34d9f1730632be74b9	6.00	7.00	0.00	0.00	0.00	0.00
9aebed393fea7e5a1159aa22f95de5f8	0.00	0.00	0.00	0.00	0.00	0.00

ce39859a23740294b1e9804fbceb1cd1	0.00	0.00	0.00	0.00	0.00	0.00
39c450641f6e2a7eb42d143375a9718d	0.00	0.00	0.00	0.00	0.00	0.00
eced9a31beb1f70096852d7cfab737d9	0.00	0.00	0.00	0.00	0.00	0.00
39afceb9b3a69428d9b836f6cdf37b41	0.00	0.00	0.00	0.00	0.00	0.00
5601d2aa3c8cd239945113d8e02a5174	0.00	0.00	0.00	0.00	0.00	0.00
0cabd8675597ff0e4b2edac13235f5dc	0.00	0.00	0.00	0.00	0.00	0.00
468a5a3b25c331a1d3633316049af8d3	0.00	0.00	0.00	0.00	0.00	0.00
f896999f535650e7a0ea062d4a5ac029	0.00	0.00	0.00	0.00	0.00	0.00
583a986f5ebf9d33038ff60eee737e06	0.00	0.00	0.00	0.00	0.00	0.00
5cd0177ab07ae113507086dc531b2914	0.00	0.00	0.00	0.00	0.00	0.00
47e9a9a80a4e671ae523b7130866a68a	0.00	0.00	0.00	0.00	0.00	0.00
b99c048523de4f9acf729edd9baaf77e	0.00	88.00	15.00	90.00	103.00	44.00
d0b590fc10524746b75ff5793d1a3f13	0.00	0.00	0.00	0.00	0.00	0.00
b05f5459e068b529b2c41e659efdef34	0.00	29.00	0.00	9.00	0.00	0.00
5cdacb5de0893c2416c349e96070203b	0.00	0.00	0.00	0.00	0.00	0.00
2952a9bfc9933977c18631ea7a12e812	0.00	0.00	0.00	0.00	0.00	0.00
d7a7003ba37048ae2818ea0433d4c79a	13.00	68.00	17.00	93.00	113.00	29.00
32dc0fbdade07b9ae9411744946391e3	14.00	55.00	0.00	26.00	82.00	31.00
01045e285c70e73c0e1f9b0a608c1b5c	0.00	0.00	0.00	0.00	0.00	0.00
799024649e927daed7711a12e9a260ca	0.00	0.00	0.00	0.00	0.00	0.00
a3e5ed4f236ccc0603c2f0bb49cf6883	10.00	9.00	8.00	31.00	14.00	9.00
dfbc5a897102ae09df8a3362d56db38e	2.00	0.00	4.00	8.00	0.00	0.00
27e35c6bca99aca17f12e36857634b6b	0.00	0.00	0.00	0.00	0.00	0.00
ead034c20ec389f7f9d1c917cdc9d39c	0.00	0.00	0.00	0.00	0.00	0.00
a6efb58be72e419dfee0e94501e7be14	0.00	0.00	0.00	0.00	0.00	0.00
a3739fb5c91439a71fd9fff1e6ee75c8	0.00	0.00	0.00	0.00	0.00	0.00
8865d1755cb1b0c53294ef6c02d2d02a	0.00	0.00	0.00	0.00	0.00	0.00
49b83eda8c285fca56ddf645e5eab48d	3.00	0.00	0.00	9.00	11.00	0.00
ae7d8efb06e9c0ad71820823c3089849	4.00	9.00	7.00	11.00	4.00	5.00
042b61955468266269bd4653f1b79d59	0.00	0.00	0.00	0.00	0.00	0.00
975907c12f6cf8219b853ed0d7f88f66	16.00	89.00	0.00	45.00	54.00	33.00
40ef042cb2d7e9e51a8dad360a9c4cd6	174.00	0.00	0.00	0.00	0.00	0.00
ab3296a689216028c3a88b82cea6dde1	0.00	0.00	0.00	0.00	0.00	0.00
ed6b424d712d7cab317a817001c0d406	0.00	0.00	0.00	0.00	0.00	0.00
843b3f4e73293bbdd62e04c2870d56c4	0.00	80.00	17.00	50.00	64.00	0.00
d623c0cc47f33bb43e2ab75cbeb3b7b2	0.00	0.00	0.00	0.00	0.00	0.00
9590d72b2e0cf8d09c71c01a74c65da2	0.00	0.00	0.00	0.00	0.00	0.00

551fca5b60bf232e3525ef8ed68619da	0.00	0.00	0.00	0.00	0.00	0.00
1407b4dc119d00684717fdc1518a70dc	0.00	0.00	0.00	0.00	0.00	0.00
81c1cfb906a30d6c08cd40e291dfccc3	0.00	0.00	0.00	0.00	0.00	0.00
947d17450a7a050d5ef513d17fe71365	0.00	0.00	0.00	0.00	0.00	0.00
b999d7a1c47c27263b1ab952c8025484	0.00	0.00	0.00	0.00	0.00	0.00
7d5f1b2fd2112ae1e6351e4e65fae20a	0.00	0.00	0.00	0.00	0.00	0.00
bfa1149dfc1d223e749724d07e4afb05	0.00	0.00	0.00	0.00	0.00	0.00
ceadf3037efdf825c74f1249d2f68608	0.00	0.00	0.00	0.00	0.00	0.00
c0117ff6228132f15792b1426e73101a	0.00	0.00	0.00	0.00	0.00	0.00
87446dadac6367a1914fb5cc4867495c	0.00	0.00	0.00	0.00	0.00	0.00
07d4403021db296fe939625fac6e1880	0.00	0.00	0.00	0.00	0.00	0.00
f7c3650f627a482ac5252096b16b52ce	0.00	0.00	0.00	0.00	0.00	0.00
9368c9c453086f303f8b1a1af15e6050	0.00	0.00	0.00	0.00	0.00	0.00
7d138fc78be823b4e0b02068c37b1cf1	0.00	0.00	0.00	0.00	0.00	0.00
e3b283f4d4700af85f542a64222d614d	2.00	44.00	6.00	16.00	56.00	28.00
f72ea3cd46c720b071b79916607c8ef2	0.00	0.00	0.00	0.00	0.00	0.00
95cc28484fcae63ba3dae5da18ce0687	0.00	0.00	0.00	0.00	0.00	0.00
c1c2fdc36db3228a9032b44c71d329b5	52.00	0.00	0.00	0.00	0.00	0.00
8d708bd5282db7a20f13c76b8af9c2d6	0.00	1315.00	0.00	0.00	0.00	0.00
4769bc41e83fb69b6fbd24a50dd111f8	0.00	0.00	0.00	0.00	0.00	0.00
7cebdc973540236b3c43e3e989880297	0.00	0.00	0.00	0.00	0.00	0.00
165a6aac36dbb69c0ed7d040540a882e	0.00	0.00	0.00	0.00	0.00	0.00
7eae2f2d5ce02646d5d55316145e878d	0.00	0.00	0.00	0.00	0.00	0.00
4a91fbea81d4fc6aad46838da248268b	0.00	0.00	0.00	0.00	0.00	0.00
2ef6fd6e80302d3e5207a46316e4692e	0.00	0.00	0.00	0.00	0.00	0.00
67ce4f32ec0bb3e626e6a8e13a6b3ec0	0.00	0.00	0.00	0.00	0.00	0.00
eb4e90f5893fb317e7a130cde726a68a	0.00	0.00	0.00	0.00	0.00	0.00
cdd07976a8984507bcca243c80c255ea	0.00	0.00	0.00	0.00	24.00	0.00
1a050f83ef25ba3a1b8471c981c936bb	0.00	0.00	0.00	0.00	0.00	0.00
d8a1a771bd0445d2205e507f8151af29	0.00	0.00	0.00	0.00	0.00	0.00
f5946bdc25df7b8726f5ddb1ff1edce6	0.00	0.00	0.00	0.00	31.00	0.00
9372d7957f77bf8f6350897afa142537	0.00	0.00	0.00	0.00	0.00	0.00
af7dac7608a3d3a3d80e26ccd2d77f1d	0.00	0.00	0.00	0.00	0.00	0.00
e69b9ab5bef3304128cb59def0ae0645	0.00	0.00	0.00	0.00	41.00	39.00
1b1cc07c10f908d0b97142b8445a9469	0.00	0.00	0.00	0.00	0.00	0.00
e0c45f0af526cedd336769d5ed6e5a58	0.00	0.00	0.00	0.00	0.00	0.00
a4ee17c632bbd8bf8c211d0fb78cf545	0.00	0.00	0.00	0.00	0.00	0.00

8ab281f2ebda51eabb3561259ca04021	0.00	0.00	0.00	0.00	0.00	0.00
e6b63da5592cde7755db53f7a3227cda	0.00	0.00	0.00	0.00	0.00	0.00
51e6792cf390c56b9e780cc5cc217447	0.00	0.00	0.00	0.00	0.00	0.00
b05658c1613c15e35349371c4131bd4f	0.00	0.00	0.00	0.00	0.00	0.00
c925a2548da2197d594993344c28001e	0.00	0.00	0.00	0.00	0.00	0.00
769bdba49cfee5c70a1f74cd93102ce5	0.00	0.00	0.00	0.00	0.00	0.00
000fe74555b8d995714372caccc35381	0.00	0.00	0.00	0.00	0.00	0.00
de0e2a711fd078e04f0f2abb052f61ab	5.00	45.00	6.00	56.00	45.00	5.00
a4e54b458f0132819ca229fafd170d07	0.00	0.00	0.00	0.00	0.00	0.00
c13359a9f895b1a2b9fcf49ceaea0e8a	0.00	0.00	0.00	0.00	2.00	0.00
e4a2c469a635923613161a8c9aba0c98	0.00	0.00	0.00	0.00	0.00	0.00
da7b3c938a99ded6db6aebf9771a985d	0.00	0.00	0.00	0.00	0.00	0.00
a93e399de7b5da78166dcf9e125a32da	0.00	0.00	0.00	0.00	0.00	0.00
98a1342941554ef65853b41145e7f6c0	0.00	0.00	0.00	0.00	0.00	0.00
add96da95e233879db152d4b04f12dbb	12.00	19.00	0.00	9.00	59.00	32.00
819d0e760b6a1dee160ccb769de1c8f9	0.00	0.00	0.00	0.00	0.00	0.00
e8f8b6fac73aa88d22160b416cd36281	0.00	0.00	0.00	0.00	14.00	0.00
8f86ec4d21b60c38c9f26306289a07a4	0.00	0.00	0.00	0.00	0.00	0.00
5d5601873f9ab19e9197afb539d1fe9c	0.00	0.00	0.00	0.00	0.00	0.00
a5b1143e6d178acea76fdfab4fbc1119	0.00	0.00	0.00	0.00	0.00	0.00
7d1d1f64ec2c63ea7fc5cd23abcb11de	0.00	0.00	0.00	0.00	0.00	0.00
8af9214fc54cb6a20a80c46c9bd7f44c	0.00	0.00	0.00	0.00	0.00	0.00
7862462431c2d710680a356852bbcafa	0.00	0.00	0.00	0.00	0.00	0.00
34b3ea5fa7c80544e9f07517fae76298	12.00	15.00	11.00	0.00	30.00	58.00
e9345fdd620b56fcf84d35dbe5e0af58	0.00	0.00	0.00	0.00	0.00	0.00
e6be0e16e1ee3399e51941b49063c870	0.00	0.00	0.00	0.00	0.00	0.00
fe29415fbb672a1ac69bdf19f3a6e370	0.00	0.00	0.00	0.00	0.00	0.00
a579d3deee2e33574e0534cc31e5c0d6	0.00	0.00	0.00	0.00	23.00	0.00
fa8dc4d3f18766648b3dede4364172a5	0.00	0.00	0.00	0.00	0.00	0.00
068a6ab40fda8b907b8321ccdae35499	0.00	0.00	0.00	0.00	0.00	0.00
9017a32c55163971f70ddc07d8e278b8	0.00	0.00	0.00	0.00	0.00	0.00
0ea1f6c154ce927dd0d15eaeac0f1449	0.00	0.00	0.00	0.00	0.00	0.00
90a9a1b784cc6dcbc27138dedcbd8103	0.00	0.00	0.00	0.00	0.00	0.00
17c89448007c78371090d062d0c103a6	0.00	0.00	0.00	0.00	0.00	0.00
705762eb6b8ae5cdccc17e09653eeb69	0.00	0.00	0.00	0.00	0.00	0.00
dc22e0f49c71e85bc6fb960fe883e0c7	0.00	0.00	0.00	0.00	0.00	0.00
0bf1462ee1a7b9b992a94970bc774592	0.00	0.00	0.00	0.00	0.00	0.00

9055ab8e054f7f780fee244d8f55f8be	0.00	0.00	0.00	5.00	38.00	0.00
f6dab20f74d2337c570a1c3cfff16b1d	0.00	10.00	10.00	0.00	14.00	0.00
ca7abdea3abb42359176fbb48baded03	0.00	17.00	10.00	38.00	77.00	0.00
3832a349b6ca6431e9dbc941a45ea95f	0.00	0.00	0.00	0.00	0.00	0.00
15cc4ca53c9d72c9b6ae36f833548a7b	0.00	0.00	0.00	0.00	0.00	0.00
610804d735136582c4230c32341d8641	0.00	0.00	0.00	0.00	0.00	0.00
b45ace7dcfd22c27638af1b8b7efcbea	0.00	0.00	0.00	0.00	0.00	0.00
bcd39d97c1d805e8136309dbe17ebd91	0.00	0.00	0.00	0.00	0.00	0.00
3784cfb4d74a14b3f1dfd821c71dd7da	0.00	0.00	0.00	0.00	0.00	0.00
4a8edca4c0441ad2ce3be93a93ccf829	0.00	0.00	0.00	0.00	0.00	0.00
3743fc7e701a937476714f3c9dde13b5	0.00	32.00	0.00	26.00	74.00	0.00
2d3d21d699df476713a1b0be0286e6cc	0.00	0.00	0.00	0.00	0.00	0.00
da185943062e8dee1ad506d607285ac6	0.00	6.00	0.00	0.00	5.00	0.00
3373509227675cfa353818dcca31de66	0.00	0.00	0.00	0.00	0.00	0.00
8a2bda7032b26355a1676be6d037dcbb	0.00	0.00	0.00	0.00	0.00	0.00
ff01ee46c50f0a9a0b562c09780419e9	0.00	0.00	0.00	0.00	0.00	0.00
692ef3111a5143c64d5ec9d26603cc5a	0.00	0.00	0.00	49.00	37.00	35.00
724b7e2fc59833027d324a0f22d4aaa2	0.00	0.00	0.00	0.00	0.00	0.00
24aeef78ec910bc6b748bca73e7e4081	0.00	0.00	0.00	0.00	0.00	0.00
8eaaec402d6f454dbf4eab4d2eebe469	0.00	0.00	0.00	0.00	0.00	0.00
10fa487a6a85c78389151d08544c4eff	0.00	0.00	0.00	0.00	0.00	0.00
0f5ff7596e0c1a343b607d6ed3b3eca2	0.00	0.00	0.00	0.00	0.00	0.00
e9150b0c1c66f6cae88e5a4a99c8fcc8	0.00	22.00	5.00	18.00	56.00	10.00
ff9af5c8679a974647adb118ea741714	0.00	0.00	0.00	0.00	0.00	0.00
f89f86c7fab5bca3e2a76b1e3dd82cfa	0.00	0.00	0.00	0.00	0.00	0.00
558fc33a72ba0f4b6908830b4a0439ca	0.00	2.00	0.00	53.00	67.00	9.00
26f7f0a49c3d1622766c36c9fb3ea2a9	0.00	0.00	0.00	0.00	0.00	0.00
caf9425dcfebd91c4f26c9f0fbc35ba4	0.00	0.00	0.00	0.00	0.00	0.00
55146ce0f5f87cb9446276ee39d628d2	0.00	0.00	0.00	0.00	27.00	42.00
9f2a7f076bf692cd110a334accc8703c	0.00	0.00	0.00	0.00	0.00	0.00
a58a318b05fd4453394cd7b2fe137a6e	0.00	0.00	0.00	0.00	0.00	0.00
821b07dae793d819f476c78716aeed08	0.00	0.00	0.00	0.00	0.00	0.00
67906018c19981ab1e23e3e693bfc42b	0.00	0.00	0.00	0.00	0.00	0.00
ca0eb1b092edeff01cc3c9892bc63d42	42.00	26.00	33.00	0.00	38.00	0.00
80fe86a54c3cfceac505b2bac034ff4b	0.00	0.00	0.00	0.00	0.00	0.00
416cc65b495acd3865f7cf5ae23aa5b0	0.00	0.00	0.00	0.00	0.00	0.00
590b7f1b7864854c8b0d0616b397fb8f	0.00	0.00	0.00	0.00	0.00	0.00

1f4ea6cf7f8215690f3bcac79f0aaaf0	0.00	0.00	0.00	0.00	0.00	0.00
01801a5eb55097a2e4a820f9535d9284	0.00	0.00	0.00	0.00	0.00	0.00
f2fb6c97281ddcce018f167480272d14	0.00	0.00	0.00	0.00	0.00	0.00
bce71cf56c53dd06383c00d57fb4bea1	22.00	7.00	25.00	13.00	4.00	24.00
12bce7c795070e53c580eec862f05b33	0.00	0.00	0.00	0.00	0.00	0.00
e7e777b9463bff2fe35316778ac0a47e	0.00	0.00	0.00	0.00	0.00	0.00
d4567ee9c11281613198131ce7fbfc75	0.00	55.00	0.00	0.00	112.00	0.00
79bd309b6cf169860bccebf66742c4d6	0.00	0.00	0.00	0.00	0.00	0.00
5694768266f05a4015228cbe90c7c883	0.00	0.00	0.00	0.00	0.00	0.00
b78e29c0d44392b7a5b768c5c1bf7302	0.00	0.00	0.00	0.00	0.00	0.00
658f8e759ac7c72aa33a047b2ea59e41	9.00	0.00	0.00	0.00	0.00	0.00
41ca6346fd4b6ced4308fad7f9289ad9	0.00	0.00	0.00	0.00	0.00	0.00
5197d787ab70ba39138acfa196880ebc	0.00	0.00	0.00	0.00	0.00	0.00
a893c86550d90106fb1e80c2af9f99b8	0.00	0.00	0.00	0.00	0.00	0.00
9a5b6f7b7f3801dc2c79eb5a621d92f7	0.00	0.00	0.00	0.00	0.00	0.00
aa2883b46705f8234fc6877a7bb3ced3	0.00	0.00	0.00	0.00	0.00	0.00
558c31aa7c7a161a926a23a0c33cf7bd	0.00	0.00	0.00	0.00	0.00	0.00
b4e0bc9c675806b438a3ec9f3b6f907a	0.00	0.00	0.00	0.00	0.00	0.00
b94e07f817d7b4c462ac6051facf5029	0.00	0.00	0.00	0.00	0.00	0.00
50edb36a42aa5eebe2da11ce0e4706bf	0.00	0.00	0.00	0.00	0.00	0.00
a6df1e83064e11cec49fcf93ae828eab	0.00	0.00	0.00	0.00	0.00	0.00
0b598cdc2034cd9a7429a9be820676eb	16.00	0.00	12.00	62.00	27.00	0.00
980c589c26210db40cd56abf9adaa2f7	0.00	0.00	0.00	0.00	0.00	0.00
7c16a4e2d1c4b721261fb418447d85e0	0.00	0.00	478.00	0.00	0.00	0.00
03d8e5c49bfaeb86b45af54523476a88	0.00	0.00	0.00	0.00	0.00	0.00
42d874828983d27fe4446f61278b45ff	0.00	0.00	0.00	0.00	0.00	0.00
447caa1367d54cb0d35e5661a0f6b811	0.00	0.00	0.00	0.00	44.00	11.00
b298f5251478adc7e6851db88740abd0	0.00	0.00	0.00	0.00	0.00	0.00
b422534d9a46d19bebf780ca1ad8ea91	0.00	0.00	0.00	0.00	0.00	0.00
8f61e623de7d7f2e3072dcb712056fa7	4.00	9.00	15.00	103.00	8.00	0.00
0836bed1ed7e32b03ee935637af7c7bd	0.00	0.00	0.00	0.00	0.00	0.00
743786a1c693277e4745f56e72a9adcf	0.00	0.00	0.00	0.00	0.00	0.00
c201ebdff7295f4ba947586e99e0392f	0.00	0.00	0.00	0.00	0.00	0.00
5318160aef9981c5f597566c98a542e9	0.00	0.00	0.00	0.00	0.00	0.00
7ff70e72e7fd1f18a2114635000f310a	0.00	24.00	0.00	259.00	29.00	0.00
6c8e6b362e0a62be1976f84587fb3712	0.00	0.00	0.00	0.00	0.00	0.00
eaf85d45a6b65ecb32027748fab5073a	0.00	0.00	0.00	0.00	0.00	0.00

1453d36cb61ec46818d150c35e65a54f	3.00	25.00	0.00	4.00	21.00	0.00
1788f7c1d1017ec47bc2b2b88dc118fe	0.00	0.00	0.00	0.00	43.00	0.00
acf0e2c6bd1f325fcd01d46f12b9c146	0.00	0.00	0.00	0.00	0.00	0.00
575037da2eda0f9dda9e70d41ebd71c8	0.00	0.00	9.00	15.00	0.00	0.00
6f5e92308b62deecdbe8e19975e6b016	0.00	0.00	0.00	0.00	0.00	0.00
772d8a2652af08e2523e46a6779f8125	0.00	0.00	0.00	0.00	0.00	0.00
7575c5d1e36ca75c5a8171a29077f1d8	0.00	0.00	0.00	0.00	3.00	4.00
9f777d99c764a7996e9804cdcc2d1add	0.00	0.00	0.00	0.00	0.00	0.00
9617aaf5f44323c1cbf20afccbd0fdef	0.00	0.00	0.00	0.00	0.00	0.00
60af038f9e3f07e32f8fe6512b6ccbda	0.00	7.00	0.00	0.00	8.00	0.00
e909c7929ad8c2bf10053032d11a5f31	0.00	0.00	0.00	0.00	0.00	0.00
5bdb2ac353127cd55b0edc0da340f948	0.00	0.00	0.00	0.00	0.00	0.00
003159217cd1e569d13ed667ad2aae7a	0.00	0.00	0.00	0.00	0.00	0.00
a68f03eb3e4a978fd239d928d429e05a	0.00	0.00	0.00	0.00	14.00	0.00
ecb98658bfa2b240171796500e3f7708	0.00	0.00	0.00	0.00	0.00	0.00
95aadb4ab0a54308134f2bf3f5c320b3	0.00	10.00	0.00	24.00	15.00	0.00
c28dac20d59b30aa554e98ddb7bb5631	0.00	0.00	0.00	0.00	0.00	0.00
f3f9656c8688a001e5b5da0829f07c1a	0.00	0.00	0.00	0.00	0.00	0.00
95fe5ce4bc9f7d89a9f71f4cdf8eb813	0.00	0.00	0.00	0.00	14.00	0.00
a61a86699bd993c26f3ecdd1e65acb1b	0.00	0.00	0.00	0.00	0.00	0.00
f99e521f9b4728349ecdff4a48954d8e	0.00	0.00	0.00	0.00	0.00	0.00
40db79b650d8f39776b18a03076cc8c3	0.00	0.00	0.00	0.00	0.00	0.00
50d5f90c63f74ee345bd8a81da958756	0.00	0.00	0.00	0.00	0.00	0.00
b2677f4ff5d956ca31eb730b38ed5010	0.00	0.00	0.00	48.00	0.00	0.00
ec1650bb9386e39849cd446cb7b025a0	0.00	0.00	0.00	0.00	0.00	0.00
8391eaa6f061052fa6162b54a407e43b	0.00	0.00	0.00	165.00	75.00	0.00
82dbf7a5dba414abce2994e8eb6d58ea	0.00	0.00	0.00	0.00	0.00	0.00
bf5e208ed2c13e72d6080b0aa8941757	0.00	0.00	0.00	0.00	0.00	0.00
30627fa5fccc5bb2bf2bb293f9485d57	0.00	0.00	0.00	0.00	0.00	0.00
bd4306a2e99e4c1e3589f2c893bd0cbe	0.00	0.00	0.00	0.00	0.00	0.00
407cb1a5b90da56b51d4f1ee8cb29c2a	0.00	0.00	0.00	0.00	0.00	0.00
c88a9fea150a8f14ddfda1d76771c5f1	0.00	0.00	0.00	0.00	0.00	0.00
30c8f8445bfa8ba9cca9c903994bcba2	0.00	0.00	0.00	0.00	0.00	0.00
3876207928742711d18645217094065b	0.00	0.00	0.00	0.00	0.00	0.00
065cf4fe98f42bdfe4ad29c1fed2e177	0.00	0.00	0.00	0.00	0.00	0.00
a6633e572930e169f8c07813c3b3cf31	0.00	0.00	0.00	0.00	0.00	0.00
b033d3caf448ac8083a9b7c1a73aefc7	4.00	8.00	0.00	0.00	0.00	0.00

7a2c68f249ca3d35097857ef03f145f3	0.00	0.00	0.00	0.00	0.00	0.00
17dd07d606dd5a17db8d5aafdac1c0af	0.00	0.00	0.00	0.00	0.00	0.00
f8349b280c1ecfedac7fb6fe70533170	0.00	0.00	0.00	0.00	0.00	0.00
c958b0d4311afb81b4a7b94926a18d11	0.00	0.00	0.00	0.00	0.00	0.00
90355c17cb5a70ac15126d56758a3767	0.00	0.00	0.00	0.00	0.00	0.00
ca10092d75c9d4b1160575bf72932454	0.00	0.00	0.00	0.00	0.00	0.00
2a54f6016e419dec4e8684c3822d33de	0.00	0.00	0.00	0.00	0.00	0.00
ebddae577c570606f011382c5267ded2	0.00	0.00	0.00	0.00	10.00	0.00
a9a6d4e4a0821969a47b3c09ecbe2540	0.00	0.00	0.00	0.00	0.00	0.00
3bc875054ff1ca03c4b2419490818f87	0.00	0.00	0.00	0.00	0.00	0.00
21a549f181f4e518c902f5c8a5329535	0.00	0.00	0.00	0.00	0.00	0.00
b10c2d245a6762ae81110757753648b0	0.00	0.00	0.00	0.00	0.00	0.00
61a622a44ba5eb604e639c6915a15ebc	0.00	0.00	0.00	0.00	0.00	0.00
d7463dce1ab4db9cf8f134047723bc85	7.00	0.00	0.00	0.00	0.00	0.00
863a18ff53a85cde9dc45e32c8f1d463	0.00	0.00	0.00	0.00	0.00	0.00
4eaedc7619e6e8ed7c0371a5c7df608e	0.00	0.00	0.00	0.00	0.00	0.00
b0a30192203737a2da2ca93428cf5b47	0.00	0.00	0.00	0.00	0.00	0.00
590016f66e421ee890865f5d15e1706c	0.00	0.00	0.00	0.00	0.00	0.00
708d8dca150ab86d7713deda80aac992	0.00	0.00	0.00	0.00	0.00	0.00
ba62177a863b31f249dc794f7c47a288	0.00	0.00	0.00	0.00	0.00	0.00
846cb60f56eb48721f71d8b8341d8a48	0.00	0.00	0.00	0.00	10.00	0.00
8326c03784edf78eee20f25cc3599dff	0.00	0.00	0.00	0.00	0.00	0.00
6b11871f385bd9a1825529f8d412bdbd	0.00	0.00	0.00	0.00	0.00	0.00
aa113b76dac30af9523a18ff6b4febfd	0.00	110.00	0.00	0.00	0.00	0.00
73d0eb400b6d83e7c8bc554a008c5398	0.00	0.00	314.00	0.00	0.00	0.00
e5222bacc65a5b4ecd5377636254b2ba	0.00	0.00	0.00	0.00	0.00	0.00
928ce1926c7d12f813920b7985b58500	0.00	0.00	0.00	0.00	0.00	0.00
91bc5937f70cdd14000a8beabcf6108d	0.00	0.00	0.00	0.00	0.00	0.00
312555586fe415c688e5a9ae0314c988	0.00	0.00	0.00	12.00	0.00	0.00
9e20a235c547acc52759d1b7c6e32662	0.00	0.00	0.00	107.00	0.00	0.00
325814be6b183d393abb47ea1c8be612	0.00	0.00	0.00	0.00	0.00	0.00
8a138dd11b3f9201a81b4d38251547b0	0.00	0.00	0.00	0.00	0.00	0.00
d219e299400960c684d2d08cdaeac162	0.00	0.00	0.00	0.00	0.00	0.00
9a0c02dee7c86a685909f66a148ce0e4	0.00	0.00	0.00	0.00	0.00	0.00
3a43f94c1c0846d7a618fa9d56c894ac	0.00	28.00	0.00	37.00	27.00	0.00
cfd478c81bc07cfd51696563e021154b	0.00	0.00	0.00	0.00	0.00	0.00
09cf90a5956a0cad729a7bdb5255b224	0.00	0.00	0.00	0.00	0.00	0.00

77099473d0cda3e8462d80d459f5e836	0.00	0.00	0.00	0.00	0.00	0.00
32d011103dbf53a9c2219a33d8e79c27	0.00	0.00	0.00	0.00	0.00	0.00
194a2c0aebe478f53f7e8ac50c03b1b0	0.00	0.00	3.00	3.00	0.00	3.00
ee8400b8406d10d05017558f68a12adf	0.00	0.00	0.00	0.00	0.00	0.00
e169f07632cf27b56d1b156ecc4d07d6	0.00	0.00	0.00	0.00	0.00	0.00
644a3b47da63ac9566d0551f20728007	0.00	0.00	0.00	0.00	0.00	0.00
b90c196f8dd176af6e1c9e71fa3bdd88	0.00	0.00	0.00	0.00	0.00	0.00
1ee9e768a29f0bd8cc04c338d8711eef	0.00	0.00	0.00	0.00	0.00	0.00
ec2e0316d85d0d4ad78c8279ca11bf7d	0.00	0.00	0.00	0.00	0.00	0.00
9849a7d79f6c37027a4fa2749dfbfe6c	0.00	0.00	3.00	0.00	0.00	0.00
e49c1ee1db1d35535f09ff727bbe9f33	0.00	0.00	0.00	0.00	0.00	0.00
212643dcbfe7a1273dacc7c8ab2f6e49	0.00	0.00	0.00	0.00	0.00	0.00
55d4b1bed0189919756056c0fe1d4914	0.00	0.00	0.00	0.00	0.00	0.00
2569a036ac4ea38d39dcdff78dbda91f	0.00	0.00	0.00	0.00	0.00	0.00
0d9e8b7c5f6a42e0d11ac2aef5d8fcfe	0.00	0.00	0.00	0.00	0.00	0.00
6902a7a7eb0d29469958e7e2b8b9f9b4	0.00	0.00	0.00	0.00	0.00	0.00
8eba01dcca568337c0553907fe2acce0	0.00	0.00	0.00	0.00	0.00	0.00
68feca6590ab9964c5cf24490a57a1cd	0.00	0.00	0.00	131.00	26.00	0.00
f53d789d6c4e47823eceeea463229df6	0.00	0.00	0.00	0.00	0.00	0.00
73f5b6a7c6e926a1708eebb186c098c0	0.00	0.00	0.00	0.00	0.00	0.00
728012ab960f0ae54488b146fc60377f	0.00	0.00	0.00	0.00	0.00	0.00
9deff80117bc837dfe3b2bec8400cf46	10.00	0.00	0.00	0.00	0.00	52.00
5a095cdc28b73fd95af9b2ebaaa0b6b8	0.00	0.00	0.00	0.00	0.00	0.00
d41f18c7920d46ca39192ae8c158fb54	0.00	0.00	0.00	0.00	0.00	0.00
a9727a3339d1e8d583b49f82d4ebbdd7	0.00	0.00	0.00	0.00	0.00	0.00
9b3f6413586b0ef817039ac2cd63c700	0.00	0.00	0.00	0.00	0.00	0.00
a1e6380f7cf9f48f3ea0026643e0ff45	0.00	0.00	0.00	113.00	56.00	0.00
3679e32b27fbd5e27a41f630a7b0a783	0.00	0.00	0.00	0.00	0.00	0.00
dab1fcba6c45c268fdd093ff0cfdb1c6	0.00	0.00	0.00	0.00	0.00	0.00
aa2e9046ca48c55794530e5b65e8a13c	0.00	0.00	0.00	0.00	5.00	0.00
999f3b5776f0f2c75ff7892ed16ad4d8	0.00	0.00	0.00	0.00	0.00	0.00
9131f00cc3d6cfe53178a5aea8dd5be5	0.00	0.00	0.00	0.00	0.00	0.00
cfd59a6caac95fc8821748a94e3d3ad7	0.00	0.00	0.00	0.00	0.00	0.00
80e70165b755bf006c18a20951713f0d	0.00	0.00	0.00	0.00	0.00	0.00
82546d23702655864e4239409244ce02	0.00	0.00	0.00	0.00	0.00	0.00
1e57621f714df207cfa7aff2b6284a2c	0.00	0.00	0.00	7.00	16.00	18.00
fd5bde25e229fd15505d1cbf5c633a32	0.00	0.00	0.00	0.00	0.00	0.00

c2b11b0483acd1dadb8d25e3c4305491	0.00	0.00	0.00	141.00	0.00	4.00
42e353fe313638b31015c740bc3bdb25	0.00	0.00	0.00	0.00	0.00	0.00
09cde818a6609e2e6e9135bbb73f93f5	0.00	9.00	0.00	13.00	26.00	0.00
168ab6dd0dc826f9c5f7d027cf24a0df	0.00	0.00	0.00	0.00	0.00	0.00
699fcace188892d9c2c8f5b5e9272b1f	0.00	0.00	0.00	0.00	0.00	0.00
e3e0dc07289e3d769d4d059ab7c6fb29	0.00	0.00	0.00	0.00	0.00	0.00
2adda35da7b8fd25a52b909c300460bc	0.00	0.00	0.00	0.00	0.00	0.00
c8de07611d639d09a4d9ad6b9b2946e8	0.00	0.00	0.00	0.00	0.00	0.00
9011a918762796b630bb08f9dd5f6909	0.00	0.00	0.00	225.00	0.00	0.00
c3b2f7b2f81f8e1c3f26168bd52865ef	0.00	0.00	0.00	0.00	0.00	0.00
3fbf93f3736e44faedcb4ce044e009ce	0.00	0.00	0.00	0.00	0.00	0.00
97e320f20b736e11d284ef467602a26a	0.00	0.00	0.00	0.00	0.00	0.00
778e8d13740e4b5c89bb310db0b54960	0.00	0.00	0.00	0.00	0.00	0.00
ec3e432350a7e1d5fc6206857f07ddfd	0.00	0.00	0.00	0.00	0.00	0.00
e26edbd73d2102dd27e8802201162882	0.00	0.00	0.00	0.00	0.00	0.00
1ee3d41d5a6e8386151afb765afe672a	0.00	0.00	0.00	0.00	0.00	0.00
8a5d014d8312929c60bed94fea3f5223	0.00	0.00	0.00	0.00	0.00	0.00
4d95f7aae3d647763ab422902038c2db	0.00	0.00	0.00	3.00	0.00	0.00
acf9cb90c4d00e099c99b7fb38b3576e	0.00	0.00	0.00	0.00	0.00	0.00
0b646ae91bbf933af091c70af1119631	0.00	0.00	217.00	0.00	0.00	0.00
b35fc613772f3c0216d2246b168414c7	0.00	0.00	0.00	0.00	0.00	0.00
855056bf2ecf3aa42fc43c29098a134c	0.00	0.00	0.00	0.00	0.00	0.00
570a457ff271a2ffc44e82dc81cd5fe2	0.00	0.00	0.00	0.00	0.00	0.00
1feccfb39125c3c6d8614a674cf44b7e	0.00	6.00	12.00	43.00	18.00	0.00
b24dd27fac72fc74c728c27a6d408d98	0.00	0.00	0.00	0.00	0.00	0.00
da7965066503cde386552bb258634821	0.00	0.00	0.00	0.00	0.00	0.00
1298a28bc259d20f9d776b928bbc7848	0.00	0.00	0.00	0.00	0.00	0.00
8ba07acc3915649a76674f6e644bbe69	0.00	0.00	0.00	0.00	0.00	0.00
5cdfd1a5b5457b83a95344c3fc3d7018	0.00	0.00	0.00	0.00	0.00	0.00
da0c9891dc9249fc0950414fc8587fdb	0.00	0.00	0.00	0.00	0.00	0.00
fc09aae0332019d4c733aeeaffb3ab07	4.00	34.00	3.00	19.00	8.00	4.00
670395466276e84b91be2466281a0bd8	0.00	0.00	0.00	0.00	0.00	0.00
f08a6c036c948593ab40b73ba1c22346	0.00	0.00	0.00	0.00	0.00	0.00
2dbc7f48fe4bbc858b7427b9fdfb1463	0.00	0.00	0.00	0.00	0.00	0.00
041a45252498e4b1d56b4e23c2670ab0	0.00	0.00	0.00	0.00	0.00	0.00
66c9a6d120ce39d0bbd0cb4c4537a6ef	0.00	0.00	0.00	0.00	0.00	0.00
71c06f205cc41ac1ecc4c5dda59b01de	0.00	0.00	0.00	0.00	0.00	0.00

b6ee0b24d0f6b361ce8263fed28cd5e4	0.00	0.00	0.00	149.00	0.00	0.00
74450745cf6e2a7ba31e9562fe1fba6a	0.00	0.00	0.00	0.00	0.00	0.00
94ff2267819480da155892ad950968b3	0.00	0.00	0.00	138.00	0.00	0.00
f1c9f5b3122f321335cc85deb7e77de0	3.00	2.00	0.00	9.00	22.00	6.00
924b48cf897bb4394fda7e027a079c54	0.00	0.00	3.00	0.00	0.00	0.00
c215402b742c6e200bb8e13083116f4b	0.00	0.00	0.00	0.00	0.00	0.00
592034b38ba14dec5f60783052c2a5f5	0.00	0.00	0.00	0.00	0.00	0.00
a0cbb386c5d2ada73ad916ff99d2f53b	0.00	0.00	0.00	0.00	0.00	0.00
ff779a2ddcada8fdaa99132f6173b58b	0.00	0.00	0.00	0.00	0.00	37.00
c98b1c4e056a6dc70bc345c8ae44d17e	0.00	0.00	0.00	0.00	0.00	0.00
31f6176cffc9b42dc35e6d0e5a15587b	0.00	0.00	0.00	0.00	0.00	0.00
7bbacdcc3df6226450dd60e31e75b264	0.00	0.00	0.00	0.00	0.00	0.00
94156adf43b4b22df3ad9c13f979ecd0	0.00	0.00	0.00	0.00	0.00	0.00
771e19d9a36a522cfe3029c4306f0d84	0.00	0.00	0.00	0.00	0.00	0.00
1ab2f0f3a8310912b6022982f86ba646	0.00	0.00	0.00	0.00	0.00	0.00
a94a0a37a25eef21528e95e93fd748e0	0.00	0.00	0.00	0.00	0.00	0.00
25df228efbc79c1b3236ad9d1571011d	0.00	0.00	0.00	0.00	0.00	0.00
dde7915c2b470e6ab3e4787a2ac6b02a	0.00	0.00	0.00	0.00	0.00	0.00
4e4b59d9cc67daed236048e48002365a	0.00	0.00	0.00	0.00	0.00	0.00
243838aef4b3e0692e08e7ab857ffe0c	0.00	0.00	0.00	0.00	0.00	0.00
0a1d722507a187537b594b401f0edf9c	0.00	0.00	0.00	0.00	0.00	0.00
2c3af3641e69b45c79c0824fd8a20fc9	0.00	0.00	0.00	0.00	0.00	0.00
7115f1ef5686d12d1e1251b7ecf728c5	0.00	0.00	0.00	0.00	0.00	0.00
74c764cd57dcb0e8dc8945a1836bdc3a	22.00	24.00	0.00	0.00	0.00	17.00
43365dc1ec94622eecca4ff1d088f285	0.00	0.00	0.00	0.00	0.00	0.00
50dabf2c9db3c7e616fca047c30151be	0.00	0.00	0.00	0.00	0.00	0.00
dae4b1d1c7fa9bbdb8d6bafe3d0eae66	0.00	0.00	0.00	0.00	0.00	0.00
74c32f29f0e293876cd1cd9e92a4ed14	0.00	0.00	0.00	93.00	0.00	0.00
7b3f3036d1a2dd0a9034fcd6387814a2	0.00	0.00	0.00	0.00	0.00	0.00
477ede1b5b6e9203f1c2bf74b95a3eb8	0.00	0.00	0.00	0.00	0.00	0.00
b0297040c3d53c18843d04dcc681e856	6.00	0.00	0.00	0.00	17.00	18.00
5472d4c27c3cac3b9b059bc492e8d80c	0.00	0.00	0.00	0.00	0.00	0.00
8eb72d623a3f53f0360ff27ca0b63b55	0.00	0.00	0.00	0.00	0.00	0.00
45d79378119df07bf803a70ed76b3926	0.00	0.00	0.00	0.00	0.00	0.00
60644df542a45fe2b82fd7f984d55f05	0.00	0.00	0.00	0.00	0.00	0.00
dc4de0174445d7be78577356661dc187	0.00	0.00	0.00	0.00	0.00	0.00
46607f7aede9c897a3237799bc8939a5	9.00	0.00	11.00	6.00	3.00	0.00

f176383260160ae767730ca36c997f3e	0.00	0.00	0.00	175.00	0.00	0.00
65d0827c1b63dfbf959460561264502a	0.00	0.00	0.00	0.00	0.00	0.00
016061ebfbe1edda14c049c86118ae48	0.00	0.00	0.00	0.00	0.00	0.00
6561f839693cc0a38d3240d69fc547e6	0.00	0.00	0.00	0.00	0.00	0.00
58225734163209910fc7df83bd14263e	0.00	0.00	0.00	23.00	0.00	18.00
9c2f4f8fa91b61c9c6698eabf657f259	0.00	0.00	0.00	0.00	0.00	0.00
08a6d6fd99991a19f79d56cdbb9a0269	0.00	0.00	0.00	0.00	0.00	0.00
41f45c0ed64d083d2cc3bd5750efe9a2	0.00	0.00	0.00	23.00	0.00	0.00
32621e05d01cb45a5a3c8dce70f09e2e	0.00	0.00	0.00	0.00	0.00	0.00
da1416f8683c00fb282d44f68c65100d	0.00	0.00	0.00	0.00	0.00	0.00
7653269243c7039319cf8dfd94f00d8f	0.00	0.00	0.00	0.00	0.00	0.00
81258d139d435307c240321d7eb311aa	0.00	0.00	170.00	0.00	0.00	0.00
78de37b1427e7dacff4ef11f7d0c6fb9	0.00	0.00	0.00	0.00	0.00	0.00
8a6583719b7172475cae0a0c371be80c	0.00	0.00	0.00	0.00	0.00	0.00
1f22f6cf973b041af38c13f941fb9816	0.00	0.00	0.00	0.00	0.00	0.00
7eb54bcfc442979b3949f8463fcaccb1	0.00	0.00	0.00	0.00	0.00	0.00
0f2905c5c3b681a088a4b6214226702b	0.00	0.00	0.00	0.00	0.00	0.00
0790b32f5533f94587bb713cc252dca6	7.00	3.00	7.00	14.00	4.00	0.00
7fdf00df0b3cb489b715fe72115da244	0.00	0.00	0.00	0.00	0.00	0.00
e37d0259a2948afb6587241e964e6f78	0.00	0.00	0.00	0.00	0.00	0.00
a13d09db91b720773b7cb6f13d3c81ab	0.00	0.00	0.00	0.00	0.00	0.00
ab08624a9939f671d068b43da196e046	0.00	0.00	0.00	0.00	0.00	0.00
5c8c4178cf84266d9428416f20d37763	0.00	0.00	0.00	0.00	0.00	0.00
8ed16d04dbe9b520bee4a7facf690343	0.00	0.00	0.00	0.00	0.00	0.00
eb7ad8a98bb2f6131540591aea0ac722	0.00	0.00	0.00	0.00	0.00	0.00
4d55f652659a9fec85e07dc3e620cd51	0.00	0.00	0.00	0.00	0.00	0.00
454024102a30b975cb3001d24fc8201f	0.00	0.00	0.00	0.00	0.00	0.00
4708439d9ef8902f9345fb972f9b0c51	0.00	0.00	0.00	0.00	0.00	0.00
1ea4823ab9d723fe255d7d710ceb758e	0.00	0.00	0.00	0.00	0.00	0.00
2b6b8cfa6bb9548f9acba1fab6270fb2	0.00	0.00	0.00	0.00	0.00	0.00
b45a20f0a4ff8356af7b24b5f0f1db18	0.00	0.00	0.00	0.00	0.00	0.00
354c6d1c08579009e8952de15c5d75fd	0.00	0.00	0.00	0.00	0.00	0.00
e9b701f61b7096cb0d7bb24cb2ba68e2	0.00	6.00	0.00	6.00	2.00	9.00
3eb26f20682a56ae4157dc28dd790e52	0.00	0.00	0.00	0.00	0.00	0.00
18076380211d84723991f27dcd3d61b4	0.00	0.00	0.00	0.00	0.00	0.00
16462258b4af76b1a46cbb584b08f492	0.00	0.00	0.00	0.00	0.00	0.00
4455cb83217684d6116d9bde77a8d7ad	0.00	0.00	0.00	0.00	0.00	0.00

ee512c7f7621444df504d2c9ba7656c4	0.00	0.00	0.00	0.00	0.00	0.00
8e0833c69131866446f8c879f0dbbbd1	0.00	0.00	0.00	0.00	0.00	0.00
f967ec732b3de13f99f4972c741a06a8	0.00	13.00	0.00	13.00	14.00	0.00
e5889d9bc20824296dbb3c4421db56d2	0.00	0.00	0.00	0.00	0.00	0.00
1d4566cb474cd1a5b6a551fa4015ecea	3.00	3.00	8.00	5.00	5.00	0.00
cd0870a2a698f04120828a13d4ab6ced	0.00	0.00	0.00	14.00	9.00	0.00
b7f23e0c6988cb8e1f7f545f107a5924	0.00	0.00	0.00	0.00	0.00	0.00
57a8c605046826430a9c9e8d5b957c00	0.00	0.00	0.00	0.00	0.00	0.00
fd9276855a6e35641fbbd068c02a817b	0.00	0.00	0.00	0.00	0.00	0.00
7b43052ed962805f0ce8e581845d4d31	0.00	0.00	0.00	0.00	0.00	0.00
45463ccec584c519ef4cc18bf96bf570	0.00	0.00	0.00	152.00	0.00	0.00
474df462b484734264e33d1ce7894f22	0.00	0.00	0.00	0.00	0.00	0.00
ceaed2c449a8bd295e45d430d8531c69	0.00	0.00	0.00	0.00	0.00	0.00
325b2b38e1012dbaa306cd840b0754c4	0.00	0.00	0.00	0.00	0.00	0.00
5667335c6741ffbeed26c5db0b7636f5	0.00	0.00	0.00	20.00	25.00	0.00
1beb251bd26ef27682e55493ada004e7	0.00	0.00	0.00	0.00	0.00	0.00
2e3913ad2da704f1b5650e7281c21f67	0.00	0.00	0.00	0.00	0.00	0.00
636e0b3c08b142579bec1b536a0a3f43	0.00	0.00	0.00	0.00	0.00	0.00
a227a91ba8002d171ee8323222ab2acd	0.00	0.00	0.00	0.00	0.00	0.00
a5bd85f6c3b255b5c6b24e6edea3f328	17.00	0.00	16.00	24.00	16.00	21.00
8ad8f75ffd5cd7f2992a90e4a95affbd	0.00	0.00	0.00	0.00	0.00	0.00
0ab4cc20bf86c02f73cb10e93fa31f04	0.00	0.00	0.00	0.00	0.00	0.00
7f908cec399587a155c8657f0ff68b05	0.00	0.00	0.00	0.00	0.00	0.00
1b14b01c8c3eadc37e94b6c9a17e6b3a	0.00	0.00	0.00	0.00	0.00	0.00
9d0b96b80d5f6458750a58224055c32b	0.00	0.00	0.00	0.00	0.00	0.00
7ceb7ddbed64b162fde97bc4bfd5414b	0.00	0.00	0.00	0.00	0.00	0.00
f6e727c898c0e2be2412d076cbaf13dc	0.00	0.00	0.00	0.00	0.00	0.00
aace41611fed3aa5a8fcd1032eb0c446	0.00	0.00	0.00	0.00	0.00	0.00
f3471602fc338f3573bcae00188332b9	0.00	0.00	0.00	0.00	0.00	0.00
a97cbdf0743224528506c63df2d44fbc	0.00	0.00	0.00	16.00	0.00	0.00
8a5f0f6a2b67ce1efaf8940684074156	0.00	0.00	0.00	0.00	0.00	0.00
11d46dde8be56673a1bace5221b8cc1e	0.00	0.00	0.00	0.00	0.00	0.00
8ca69dfd6a88cffad1d15d675d460149	0.00	0.00	0.00	0.00	0.00	0.00
097c3bc1dc57ba121b733098a6f4c0a4	0.00	0.00	0.00	0.00	0.00	0.00
4a75ff0bae5bda54eea0477cd9facd33	4.00	0.00	0.00	0.00	0.00	0.00
3d4b6ee44225e224e55c655edcfa797e	0.00	0.00	0.00	0.00	0.00	0.00
ddab1e883f3cc6f350d8f462dd1f5eba	0.00	0.00	0.00	0.00	0.00	0.00

cf7149eae5b54db7e01e6b56dbd93741	0.00	0.00	0.00	0.00	0.00	0.00
3c4264e19388db984affdda5e89a1863	0.00	0.00	0.00	0.00	0.00	0.00
e3e618b6c0b173feb5ba627d9a96d485	0.00	0.00	0.00	0.00	0.00	0.00
dc999453e94b1433642ad6986dff8a31	0.00	0.00	0.00	0.00	0.00	0.00
af1c9e100b97b354d7fc6d4684895a21	0.00	0.00	0.00	0.00	0.00	0.00
e33431033731537b4cabace92841ed12	0.00	0.00	0.00	0.00	0.00	0.00
be50648759a0f91ea94fec2f4bc2d87f	0.00	0.00	0.00	0.00	0.00	0.00
36ed65c50be83df720061e921a829086	0.00	0.00	0.00	0.00	0.00	0.00
b3bde86dce17e6fded609f05ca502116	0.00	23.00	0.00	17.00	0.00	0.00
bd19ca83793669ede5bb51ca3761bb96	0.00	0.00	0.00	19.00	14.00	0.00
2dbae3f3a14f11df3e6be35e609b8db5	0.00	0.00	0.00	0.00	0.00	0.00
f210f24acd41d35d6bedaef2fd581db4	0.00	0.00	0.00	0.00	0.00	0.00
9bcfe0fe66e62e4c4b49a893008eabfa	0.00	0.00	0.00	0.00	0.00	0.00
06698b940debbc7349d3b5fe5c91fa88	0.00	0.00	0.00	0.00	0.00	0.00
f5b692d109f3fee93de8a715af4aed51	0.00	0.00	0.00	0.00	0.00	0.00
5963d94c508144e4257298ef834e99a0	0.00	0.00	0.00	0.00	0.00	0.00
f0da7f0a0fe831d6cd9b25d210f8d710	0.00	0.00	0.00	0.00	0.00	0.00
41bd7f7c68ba0c12fd68357581092f47	0.00	0.00	0.00	0.00	0.00	0.00
0e9679c124f5029642cc42cd92bbe25f	10.00	3.00	4.00	28.00	5.00	10.00
e6e1d7b0c20d54e35ce56fde476decaa	0.00	0.00	0.00	0.00	0.00	0.00
c05d39c97c187c0c24b50473aa3cd21c	0.00	0.00	0.00	0.00	0.00	0.00
617e4cd931d86b25916fbf002e3906f7	0.00	0.00	0.00	0.00	0.00	0.00
3778edb07bd137313a044db84b7a607e	0.00	0.00	0.00	0.00	0.00	0.00
e5b198502cdd6b3ab14c859c11a8f2e3	0.00	0.00	0.00	0.00	0.00	0.00
8b0daac26087f00f1789aecf1a75dbfe	0.00	0.00	0.00	0.00	0.00	0.00
634b6fc524ffc39fd205b0a21467dfd4	0.00	0.00	0.00	0.00	0.00	0.00
a41b72842290a9e865efc83d63ad2818	0.00	0.00	0.00	0.00	0.00	0.00
252a044d15ef6b9f96d15ea1667ebe01	0.00	0.00	0.00	0.00	0.00	0.00
f7a9a4c9e4803b4772d327b3e94e80e0	0.00	0.00	0.00	0.00	0.00	0.00
a676dbcf5d5602d78ca7e9580772332e	0.00	0.00	0.00	0.00	0.00	0.00
d2c23e41a40586349cba342a37810c5c	0.00	0.00	0.00	0.00	0.00	0.00
91e172b09707d64ff14d455972425a2a	0.00	0.00	0.00	0.00	0.00	0.00
7be6536a9f4488db7aa3ed6a562a4899	0.00	0.00	0.00	0.00	0.00	0.00
7730744253017a090a1b3ed9f67967ef	0.00	0.00	0.00	0.00	0.00	0.00
2fa94cc6048c8911f3ba491241149330	0.00	0.00	0.00	0.00	0.00	0.00
9ee951537ba132f3d631bc8dde229d06	0.00	0.00	0.00	0.00	0.00	0.00
efb09d5977cb5cb310ffda7959b60eac	0.00	0.00	0.00	0.00	0.00	0.00

71d76418089b46faa22caf079938288b	0.00	0.00	0.00	0.00	0.00	0.00
91b2333db37817e8683c358a37dbb7c9	0.00	0.00	0.00	0.00	0.00	0.00
acdfa9fe0f8349b573096117d62a6581	0.00	0.00	0.00	0.00	0.00	0.00
aabf8ae4421e51d772aad77908e7fc15	0.00	0.00	0.00	0.00	0.00	0.00
cc83579f70bd1fc1b8b7f9a8779250b7	0.00	0.00	0.00	0.00	0.00	0.00
21fa7ca7c0acc6f386720010fa9a4ce9	0.00	0.00	0.00	0.00	0.00	0.00
90092392ebf972f13bbc15ff40e5089e	0.00	0.00	0.00	0.00	0.00	0.00
56056539e75456101379f62ce76c8215	0.00	0.00	0.00	0.00	0.00	0.00
e0e5044794bfd5043a2e47f411ba2dfd	0.00	0.00	0.00	0.00	0.00	0.00
d99bcd8ada1e30aedb3d0ca391e6b08c	0.00	0.00	0.00	0.00	0.00	0.00
62108afeba2c378ce6b6f95777a92654	0.00	0.00	0.00	0.00	0.00	0.00
ee442da7c362a67312baa9babfe7eb71	0.00	0.00	0.00	0.00	0.00	0.00
f27aa987d85bb2adc1a28169d06c4f67	0.00	0.00	0.00	12.00	0.00	0.00
473c3c7cc14e71847f0456cb9a1a9e30	0.00	0.00	0.00	0.00	0.00	0.00
7e90247990ccb931a3440c2e64c466cc	0.00	0.00	0.00	0.00	0.00	0.00
6a29ec14eff5d1dc7e7b509ca87abe90	0.00	0.00	0.00	90.00	17.00	0.00
4d7f68b04359334b95f54b8149d9b1e8	0.00	0.00	0.00	0.00	0.00	0.00
7b532ba9d8f31afae1eb7bbd7b36b2ee	0.00	0.00	0.00	0.00	0.00	0.00
46b5cbf0c4dbe5bec23ec2ddf4767606	0.00	0.00	0.00	0.00	0.00	0.00
a06f14991f9fc24fafe3ae3d09a3ce8e	0.00	0.00	0.00	0.00	0.00	0.00
5d263ffad0329832f496f1f52e3b7ede	0.00	3.00	0.00	0.00	0.00	0.00
d7ae9ed2fda78a98e463907aceed7f57	0.00	0.00	0.00	0.00	0.00	0.00
7f5aaa1d92aca49e42295744849858d7	0.00	0.00	0.00	0.00	0.00	0.00
fae0101bd79bcda82003353eb77e075e	0.00	0.00	0.00	29.00	0.00	0.00
f8f662f8d0238a2bf30dac644113c65b	0.00	0.00	0.00	104.00	0.00	0.00
eff88d99ab5d3f4613fa231cefbc3568	0.00	0.00	0.00	0.00	0.00	0.00
a132187e06bc748eb6fea0d89f1618d7	0.00	0.00	0.00	0.00	0.00	0.00
7fbcb41ee6aea47a9d441a3f72b806d1	0.00	0.00	0.00	0.00	0.00	0.00
d5686ae433b1236e12d30c4acdd7bfc6	0.00	0.00	0.00	0.00	0.00	0.00
4f9ca1501c797d8ce1889b7a3d478545	0.00	0.00	0.00	47.00	8.00	0.00
71602523e1912c8c7022003ef70c6756	0.00	0.00	0.00	0.00	0.00	0.00
32f94fbb1500a344d6fc4b9b3cafd66b	0.00	0.00	0.00	0.00	0.00	0.00
8321b9aed145ceb3bcdb860cdcb7c126	0.00	0.00	0.00	0.00	0.00	0.00
bddbc6fc40260b60a9150998193dd9de	0.00	0.00	0.00	0.00	0.00	0.00
5bc05ab361dc823b7cfd4ce396f89776	0.00	0.00	0.00	0.00	0.00	0.00
b8dd0ddc5d1b70a90297408c6c0cbb34	0.00	0.00	0.00	0.00	0.00	0.00
677fce3589da83eebda88859ce0c8305	0.00	0.00	0.00	0.00	0.00	0.00

898edfeed0604c59d484185f31b1056c	0.00	0.00	0.00	0.00	0.00	0.00
e80cfc5e799867021ec8ed379f3bd2dc	0.00	0.00	0.00	0.00	0.00	0.00
a5773a6efdaf0578499de016709abda3	0.00	0.00	0.00	0.00	0.00	0.00
754957eb22ac1e4e790874724e3d6f10	0.00	0.00	0.00	0.00	0.00	0.00
017f5f530b93520d57830bb52cee3f67	0.00	0.00	0.00	0.00	0.00	0.00
3c32da919e984f9a6a428b8e8d2e8377	0.00	0.00	0.00	0.00	30.00	0.00
c8fcda5b5ebfa5f8477531cb74fdd7d2	0.00	0.00	0.00	0.00	0.00	0.00
ec402a73c4581a089f980c6276ca5347	0.00	0.00	0.00	0.00	0.00	0.00
057a6615331d5a65adc54d6500dc4b77	0.00	0.00	0.00	0.00	0.00	0.00
05441919ec70f83a9cc1c747d36d3edf	0.00	0.00	0.00	0.00	0.00	0.00
bb75d6c7377bd6a67d37f4febcc11467	0.00	0.00	0.00	0.00	0.00	0.00
480fbba63e6268108188512ad75cfddb	0.00	0.00	0.00	0.00	0.00	0.00
6c32038e18c7101989076bd70e96442c	0.00	0.00	0.00	0.00	0.00	0.00
6fbc831d62aed982ac123c534f9efc3e	0.00	0.00	0.00	0.00	0.00	0.00
3ebef88a73315985b5978d2138762e61	0.00	0.00	0.00	0.00	0.00	0.00
0d7eef12e0869edaa1a46f9bd13386ff	0.00	0.00	0.00	0.00	0.00	0.00
89889395b586dd54d3deb8557255bada	0.00	0.00	0.00	0.00	0.00	0.00
6cf2c98d7c8932db32daabdb8f707b73	0.00	0.00	97.00	0.00	0.00	0.00
6e20638ce91fa2e147cf9930b8a0b49f	0.00	0.00	0.00	0.00	0.00	0.00
567299f555cda1b21bb628d1bb803712	0.00	0.00	0.00	0.00	0.00	0.00
8fb2a044999306f23d170f538519c842	0.00	0.00	0.00	0.00	0.00	0.00
4895e12a7bd83ba04cb6a6b6788142ef	0.00	0.00	0.00	0.00	0.00	0.00
04148628ef89c487375cb99c4eb5439a	0.00	0.00	0.00	0.00	0.00	0.00
76b49756ca1b4cebb92bf168dc0f03e5	0.00	0.00	0.00	5.00	0.00	0.00
eb529134cfb8434625ecc736d2c87aa4	0.00	0.00	0.00	0.00	0.00	0.00
3d565bc14b95748bbef589da0427b497	0.00	0.00	0.00	0.00	0.00	0.00
555f03dc1d846d2ab5a95f9e1f2b10c8	0.00	0.00	0.00	0.00	0.00	0.00
67fac1a0978d7ae0d302238d447a1d26	0.00	0.00	0.00	0.00	0.00	0.00
fa479e1e8d76ccfafce1e9c8b17399a1	0.00	0.00	0.00	0.00	0.00	0.00
8baf527d88114988b5fdaf5df440bde8	0.00	0.00	0.00	0.00	0.00	0.00
9325ff7b1306cc63d72909803d6cfaeb	0.00	0.00	0.00	0.00	24.00	0.00
de7fcf84f28b955de6904c30531d0429	0.00	0.00	0.00	0.00	0.00	0.00
525724ad61abb3d51c4a404295a0d4a3	0.00	0.00	0.00	0.00	0.00	0.00
df6ab375df75e2977b1381fdbf418a85	0.00	0.00	0.00	0.00	0.00	0.00
e5084546676d262b0af7425e5b7a3a7e	0.00	0.00	0.00	0.00	0.00	0.00
4efc2510e69865e357d8829a5d2375b4	0.00	0.00	0.00	0.00	0.00	0.00
e692fcf963c630541c141757c5eb197a	10.00	0.00	5.00	8.00	9.00	3.00

15819cbe05fbe6e31f5c7a509eab8f0b	0.00	0.00	0.00	0.00	0.00	0.00
e03fa61d31a9ed1cbcea711650ad4062	0.00	0.00	0.00	0.00	0.00	0.00
da385a4e28af4624815de6a7877d19c6	0.00	9.00	0.00	0.00	17.00	0.00
4d16b1314e64e8c7007fb7068c316f71	0.00	0.00	0.00	0.00	0.00	0.00
611bcf6461cfadd4e7584a45afc21249	0.00	0.00	0.00	0.00	0.00	0.00
cea66f920d0b6145eedc4fa812ddf68d	0.00	0.00	0.00	0.00	0.00	0.00
ae68210610cfba93dddce87f5046e32e	0.00	0.00	0.00	0.00	0.00	0.00
c5c39f9e5d5a02ccf975bb9162d33546	0.00	0.00	0.00	0.00	0.00	0.00
0020bf4e57f7e5b0333f98476720784e	0.00	0.00	0.00	0.00	0.00	0.00
99b3b800355763da459369f7d76a989a	0.00	0.00	0.00	0.00	0.00	0.00
85c8566e0c0a8564f506a6e737f3647d	0.00	0.00	0.00	0.00	0.00	0.00
247dd0a79d8e26c29bc03c26e71d2651	0.00	0.00	0.00	0.00	0.00	0.00
e74e6606d7e12d6580be31096c0701fd	0.00	0.00	0.00	37.00	0.00	0.00
717b7cbd0bf1509b2c39b82194445d09	0.00	0.00	0.00	0.00	0.00	0.00
a3d4448130f09ab52a7eb8906e442151	0.00	0.00	0.00	0.00	0.00	0.00
57893ff3ca5ad08c9618f482eb2d1032	0.00	0.00	0.00	0.00	0.00	0.00
651a39130899c705e5299ed6b918d367	0.00	0.00	0.00	0.00	0.00	0.00
7b9e999c0223c9edca54f707246ca75c	0.00	0.00	0.00	0.00	0.00	0.00
fc5b0daf2f05349778e75690855f30b6	0.00	0.00	0.00	0.00	0.00	0.00
3ed7861da51934a4d1d1744ae76a2184	0.00	0.00	0.00	0.00	0.00	0.00
7a7d5be0e96dd9f13acdd74f9536aa64	0.00	9.00	0.00	52.00	0.00	0.00
fb6a3178f688262ffd941b2db3d1da5b	0.00	0.00	0.00	0.00	0.00	0.00
a77f6519c7d9dfbab697bf2f3194af77	0.00	0.00	0.00	0.00	0.00	0.00
4304399387adf1a24b90ba3d3f62e24a	0.00	0.00	0.00	0.00	0.00	0.00
a895bb4612651babbc930a9a2006321c	0.00	0.00	0.00	0.00	0.00	0.00
62b2a34e2c963227aff1a118764f22bc	0.00	0.00	0.00	0.00	0.00	0.00
bf48f8a84b099cd94e39cf8053586ea4	0.00	0.00	0.00	0.00	0.00	0.00
69764c21e9dd7dba7852d3254306a724	0.00	0.00	0.00	0.00	0.00	0.00
b36f2de05d6cdab25626ceb8d9ca3867	0.00	0.00	0.00	0.00	0.00	0.00
ea7a1c4855101a336c0155acccd3f848	0.00	0.00	0.00	0.00	0.00	0.00
b3e148309b6696cd6d3d638f71e559c4	0.00	0.00	0.00	0.00	0.00	0.00
caf59bb18d824276464b64335db83af0	0.00	0.00	0.00	0.00	0.00	0.00
a2860598f7a7433e10226aaa211f2707	0.00	0.00	0.00	80.00	0.00	0.00
16399452af664116fe40843f7a70c85b	0.00	0.00	0.00	0.00	0.00	0.00
a7623a135f06e7c9bc0f8bcfd89e9e47	0.00	0.00	0.00	0.00	0.00	0.00
fe56601cd3ddc2c7bfa03556370e78fe	0.00	0.00	0.00	0.00	0.00	0.00
06ac7a0c8114c7c55cbb277d0122f248	0.00	0.00	0.00	0.00	0.00	0.00

0aff98a2d34186789ba705cd915faa45	0.00	0.00	0.00	0.00	0.00	0.00
2191102f2e336aea7b5887f47ac75626	0.00	0.00	0.00	0.00	0.00	0.00
33db7b189dcfe16b551c393c56f3b5d5	0.00	0.00	0.00	0.00	0.00	0.00
21f9c6b9e703c470266461226bbc93e4	0.00	0.00	0.00	0.00	0.00	3.00
4a70fc21e83c0c92f7464f289d2dd100	0.00	0.00	0.00	0.00	0.00	0.00
14c1d1ba955ad78a8d1364fc0650ec1b	0.00	0.00	0.00	0.00	0.00	0.00
eb3f292e9020c70f38d43742d5bd88c6	0.00	0.00	0.00	0.00	0.00	0.00
71d3e349e229fa6ccc1ec681f676bdea	0.00	9.00	4.00	9.00	12.00	7.00
162d380468428be715f6403345eaa137	0.00	0.00	0.00	0.00	0.00	0.00
081eece90f50a286ffc33537e748ae93	0.00	0.00	0.00	0.00	0.00	0.00
15a0942044fd9391f322c7275eeb1f8c	0.00	0.00	0.00	0.00	0.00	0.00
9c1e05e1e5fe55a17e2815c77dfaf07b	0.00	0.00	0.00	0.00	0.00	0.00
531f18265e2000b1f000f86b0f545913	0.00	0.00	0.00	0.00	0.00	0.00
ac12728429838f06387c50ddc5d7fe8d	0.00	0.00	0.00	0.00	0.00	0.00
725ae61d0cfdb7b1505ce9bc890cb8c3	0.00	0.00	0.00	0.00	0.00	0.00
bb5a94b8426f40b2a26f05cb4e85a5cd	0.00	0.00	0.00	0.00	0.00	0.00
f7940732594bf6c301427fb0e569b034	0.00	0.00	0.00	0.00	0.00	0.00
c4be62e6ec66c57dd02515f44881f00d	0.00	0.00	0.00	0.00	0.00	0.00
b4f7747d256bb2b8cd5b13e7463b8b53	0.00	0.00	0.00	0.00	0.00	0.00
04d34c2af12c10d8dd3cc067e23f8863	0.00	8.00	0.00	9.00	13.00	13.00
1cd5af74e85fb48ba416f6b563ea787f	0.00	0.00	0.00	0.00	4.00	5.00
4e6689e2ba8260a8573c6f0d96313961	0.00	0.00	0.00	0.00	0.00	0.00
f977f8aa988f4aa5bd8af1afee1c34e1	0.00	0.00	0.00	2.00	0.00	0.00
eb73bffdfd9b1e97fc8cd861b92835eb	0.00	0.00	0.00	0.00	0.00	0.00
e7e1188ad1f706ddc5573c33bfb33cd9	0.00	0.00	0.00	0.00	0.00	0.00
590fd7217a614eec4aa30fa4d1beaea1	0.00	0.00	0.00	0.00	0.00	0.00
e5cb017030dc6135dc7d2091c2106fc5	0.00	15.00	0.00	0.00	19.00	0.00
3fe3f8f8090b1c07a5c512345f836eb4	0.00	0.00	73.00	0.00	0.00	0.00
1ad20c0e4ced6c44f511ebce94b1c540	3.00	0.00	0.00	6.00	0.00	0.00
eb179281a68fccc2fa671894c66c04ad	0.00	0.00	0.00	0.00	0.00	0.00
b914343686cd2549a4f919c767950ce4	0.00	0.00	0.00	0.00	0.00	0.00
ef5882b82bb3f0e89bcd864aaa43a6ea	0.00	0.00	0.00	0.00	0.00	0.00
8864c48123c9a986ebb507c7d7b59c05	0.00	0.00	0.00	0.00	0.00	0.00
605a21afb4c7683a3d46658f6455379e	0.00	0.00	0.00	0.00	0.00	0.00
9cc3f7f95ddfcf67153bf6969602a86b	0.00	0.00	0.00	0.00	0.00	0.00
feccd42cbe803f722300bce5c253ba6a	0.00	0.00	0.00	0.00	0.00	0.00
e0052e0ddb394b1c18890d9bd4da39d3	0.00	0.00	0.00	0.00	0.00	0.00

8e65a7929171d7fc6512fc22ccdc1289	0.00	0.00	0.00	0.00	0.00	0.00
dc88e7eea9e1d32eef9331e3748a30cd	0.00	0.00	0.00	0.00	0.00	0.00
6d816b8fa226eb26b395f1fb33b3de51	0.00	0.00	0.00	0.00	0.00	0.00
bdbff17b6234f118169cfcf8c7b795b3	0.00	0.00	0.00	0.00	0.00	0.00
11165fe4e60c489624f3263a1bca9939	0.00	0.00	0.00	0.00	0.00	0.00
5e374bfa1b45f75acf234eb1a6e1edd3	0.00	0.00	0.00	0.00	0.00	0.00
250daf6c791429c5a83658212043ef67	0.00	0.00	0.00	0.00	0.00	0.00
e13b2f04724e1a9d558d9f45b768bff7	0.00	0.00	0.00	0.00	0.00	0.00
81dc5e7d2545165a45485fcabe25bc16	0.00	0.00	0.00	0.00	0.00	0.00
35b6cd68dd5e17772e6fa1591359a972	0.00	0.00	0.00	0.00	0.00	0.00
da1dbaa408f3ce3c0ca87606553efbbd	0.00	0.00	0.00	0.00	0.00	0.00
7cbe80ba41832ebf606b9a964d8e9629	0.00	0.00	0.00	0.00	0.00	0.00
c3e5f7598af251f705eadd9c96b44db7	0.00	0.00	0.00	0.00	0.00	0.00
b5b4aadaa7c260918cb6cc927f1c41b9	0.00	0.00	0.00	0.00	0.00	0.00
5844f13ec8276669db8251c1c706d9e1	0.00	0.00	0.00	0.00	0.00	0.00
d73c54bf849a2483f84558503c9decfa	0.00	0.00	0.00	0.00	0.00	0.00
58cf041dd61515fed71af03b19739339	0.00	0.00	0.00	0.00	0.00	0.00
039acc671ac09232df38402bfaf09ac2	3.00	0.00	0.00	10.00	11.00	0.00
9749690728b9b51beb044763e138e7d3	0.00	0.00	0.00	0.00	0.00	0.00
c71084f41acb16eb9816d552e412415a	0.00	0.00	0.00	0.00	0.00	0.00
f7339355ca575f466f41fbd988afcbff	0.00	0.00	0.00	0.00	0.00	0.00
b68a58ab28fbd2ff308c3df5eaa71051	0.00	0.00	0.00	0.00	0.00	0.00
63988a057e29eed15a5f05408091b884	0.00	0.00	0.00	0.00	0.00	0.00
5c6eac63fb614dfe9f8648e336186465	0.00	0.00	0.00	0.00	0.00	0.00
507fc93570d7d2874c27b1b35cea70f5	0.00	0.00	0.00	0.00	0.00	0.00
f1ec652460a7e11a3d66cc663ece1311	0.00	0.00	0.00	0.00	0.00	0.00
da73fa82cffc0b7ba46bcd9de6d84668	0.00	0.00	0.00	0.00	0.00	0.00
088b74f384bb0a6946b25b22c5ae4776	0.00	0.00	0.00	0.00	0.00	0.00
272081470f2129434d90a7fb610fa4c7	0.00	0.00	0.00	0.00	0.00	0.00
221f427232911280469e6452b0db730c	0.00	0.00	0.00	0.00	0.00	0.00
9b26acb66c7c20ee09ce3213ebe93592	0.00	0.00	0.00	0.00	0.00	0.00
8d66b868db5e16b0f057f33f0a72e7c5	0.00	0.00	0.00	0.00	0.00	0.00
b257826ec50ae5a96c55eb16f177b29f	0.00	0.00	0.00	0.00	0.00	0.00
d6edd4a7f528e5711a600b46e73022bd	0.00	0.00	0.00	0.00	0.00	0.00
15f0817307880568b95a0915668c30c6	0.00	0.00	0.00	0.00	0.00	14.00
ed71f3f894be6a168adf50eef10e35d8	0.00	0.00	0.00	0.00	0.00	0.00
9cd304aa65f636bcae585142504216e4	0.00	0.00	0.00	0.00	0.00	0.00

fc82fb26aebeaf99fd0d57679acfa8d2	0.00	0.00	0.00	0.00	0.00	0.00
4ee7918ef95bc121acc52890b3010b68	0.00	0.00	0.00	0.00	0.00	0.00
02a070e0f5028b3042c95553ef39163f	0.00	0.00	0.00	0.00	0.00	0.00
4797220c5bbe12b8452ea2cf56d031c9	0.00	0.00	0.00	0.00	0.00	0.00
c7a7edd1dfbe300760e415faa238782f	0.00	0.00	0.00	0.00	0.00	0.00
48e91ad5940561cd1f8a3cea8c793cd3	0.00	0.00	0.00	0.00	0.00	0.00
21a7e0bedd04b6ae75bcfdfc58622131	0.00	0.00	0.00	0.00	0.00	0.00
4e835dab5ef3578f095bb8f7ca80a5e1	0.00	0.00	0.00	0.00	0.00	0.00
072eb32aec6130966ea4d89e33531f47	0.00	0.00	0.00	0.00	0.00	0.00
e5424d58ff3be60b6e277203b8e76ee6	0.00	0.00	0.00	0.00	0.00	0.00
4a8e1ee01f5abfe7dc972122bf12991f	0.00	0.00	0.00	0.00	0.00	0.00
d146c978d01a20c7f40bf3efa189233a	0.00	0.00	0.00	0.00	0.00	0.00
f4b95f1d118625611201c7f515f1b74d	0.00	0.00	0.00	0.00	0.00	0.00
6c77f879211ab0fbf5388dd3b50b736f	0.00	0.00	0.00	0.00	0.00	0.00
b4e3207d0c753480069badae058f0a67	0.00	0.00	0.00	0.00	0.00	0.00
7ed94e5cd4c92fb2e02eee664afe5ec3	0.00	0.00	0.00	0.00	0.00	0.00
f9fa47e61e672d6c5c4b350835bade82	0.00	0.00	0.00	39.00	0.00	0.00
2298026978b305feaa68c0c007cbdf4d	0.00	0.00	0.00	0.00	0.00	0.00
d8010ead0663eec090c6e3be02cec6e3	0.00	0.00	0.00	0.00	0.00	0.00
4f2772ee25486808847242a3b7ac5a93	0.00	0.00	0.00	0.00	0.00	0.00
235cbbc9407dd08ca65d8e5a457909aa	0.00	0.00	0.00	0.00	0.00	0.00
10fcb8a0e5668b6eb162645a0747209c	0.00	4.00	0.00	0.00	3.00	0.00
c0e85a3c2f3427b1d299d8d0d9dcbf01	0.00	0.00	0.00	0.00	0.00	0.00
0ecb3ab461f2c559a855175d266026af	0.00	0.00	0.00	0.00	0.00	0.00
6218861281695df9b65a4d21814559a7	0.00	0.00	0.00	0.00	0.00	0.00
68fa42a9b54c495bfef248b461d4eb1a	0.00	0.00	0.00	0.00	0.00	0.00
b002a0e077413d14186bec03dd1fdb41	0.00	0.00	0.00	0.00	0.00	0.00
5a84fc5c189246021d10b19b9def55ca	0.00	0.00	0.00	0.00	0.00	0.00
ad216decd50b7d842594be8d41469d82	0.00	0.00	0.00	0.00	0.00	0.00
46a51ba86413b85aaad4b3b5640b6925	0.00	0.00	0.00	0.00	0.00	0.00
374a7dd271acb04ded346ba2f9042049	0.00	0.00	0.00	0.00	0.00	0.00
eb9b7a364ed207affcf6a0aa00e424e5	0.00	0.00	0.00	0.00	0.00	0.00
7dd08325f5b5e48c78aa23e6481b84b0	0.00	0.00	0.00	0.00	0.00	0.00
069f4250613e3f132598aef3359e2093	0.00	0.00	0.00	0.00	0.00	0.00
932d70b76d839b7553bb90df617aea66	0.00	0.00	0.00	20.00	0.00	0.00
1bb0a6571079947d4c079fb99b64f69d	0.00	0.00	0.00	0.00	0.00	0.00
799a40b2ec00542781197166b2e77f88	0.00	0.00	0.00	0.00	0.00	0.00

2863a555ff8d8ab29d5c39f539f2a858	0.00	0.00	0.00	0.00	0.00	0.00
45f5dcecf074c1a274da86a67b486a8c	0.00	0.00	0.00	0.00	0.00	0.00
2c2d242f5a32f4738a5d49e9e9487902	0.00	0.00	0.00	0.00	0.00	0.00
f778d68adf4b72dd9dcaac63b0eb6bfb	0.00	0.00	0.00	0.00	0.00	0.00
71c1d7796f5fb0352aed47c4de467c53	0.00	0.00	0.00	0.00	0.00	0.00
4b92124ca0782d9b9e690ae561af1d9e	0.00	0.00	0.00	0.00	0.00	0.00
08f28df221bd9bb47a459ba3e0173f76	0.00	0.00	0.00	0.00	0.00	0.00
939a838a2f6c220b5c87c0683126331b	0.00	0.00	0.00	0.00	0.00	0.00
fbe037eecb5daeb52c1e68bc7aa11c0e	0.00	0.00	0.00	0.00	0.00	0.00
03f435a455ed5bd105d051367ce87e20	0.00	0.00	0.00	0.00	0.00	0.00
9869865991002d17d80892cb033e9304	0.00	0.00	0.00	0.00	0.00	0.00
46fc3726bcb7c0ac0b5ea7671b441acf	0.00	0.00	0.00	0.00	0.00	0.00
200dc506f3b9e7909b56661d84a70384	0.00	0.00	0.00	0.00	0.00	0.00
cdf13497788be6853bfd74b4e5da209b	0.00	0.00	0.00	0.00	0.00	0.00
1bdc5f832a6a930f52760e463194b783	0.00	0.00	0.00	0.00	0.00	0.00
22080e653c0c1f8919b1c55562ba3690	0.00	0.00	0.00	0.00	0.00	0.00
53c148f12b893ca60ace94d70d744257	0.00	0.00	0.00	0.00	0.00	0.00
04221bcff6fe31a654b3184a02b9e65b	0.00	0.00	0.00	0.00	0.00	0.00
00954da950b5304b3c26bfb9552bdae6	0.00	0.00	0.00	0.00	0.00	0.00
b9cd548eccc6408a9799398e600db95a	0.00	0.00	0.00	0.00	0.00	0.00
58e5d003da5a9d184ecec823ed320bf0	0.00	0.00	0.00	0.00	0.00	0.00
9878da55559f6cdd07000a64687ea926	0.00	0.00	0.00	0.00	0.00	0.00
d02e06a7b1b102addff2849419582110	0.00	0.00	0.00	0.00	0.00	0.00
01cbcae2e50b1582818643fc1bfce099	0.00	0.00	0.00	0.00	0.00	0.00
56eea9d6a8b8fd06dd9837dfbc039708	0.00	0.00	0.00	0.00	0.00	0.00
93f867c5a0807640faf70a308e967e3c	0.00	0.00	0.00	0.00	0.00	0.00
319b5d15a4147d5de99f66dbd9a89e85	0.00	0.00	0.00	0.00	0.00	0.00
fd4b7ef02d54409772ea358c35006970	0.00	0.00	0.00	0.00	0.00	0.00
c25cb9a3da827299e720136b2280c707	0.00	0.00	0.00	0.00	0.00	0.00
7b8b30ef5a8b72d51071ebcbf8cb33c8	0.00	0.00	0.00	0.00	0.00	0.00
e552ba4552199dde768fbb463c987b7d	0.00	0.00	0.00	0.00	0.00	0.00
ddbc680fdfc3fa0df242a3ccedcb932a	0.00	0.00	0.00	0.00	0.00	0.00
aaaecf31a0d863c8b534ad1ba41d95ca	0.00	0.00	0.00	0.00	0.00	0.00
44fe26df06a2ade131fdacb4c1c1c012	0.00	0.00	0.00	0.00	0.00	0.00
1a2e12038377659c9f7552762e11f4e5	0.00	0.00	0.00	55.00	0.00	0.00
af03462b5ca00bd7671ffd91e6bf4003	0.00	0.00	0.00	0.00	0.00	0.00
6e18e97e41a7e31200da323b61362613	0.00	0.00	0.00	0.00	0.00	0.00

8edcd07b053a66b5438ca007c3ed7b45	0.00	0.00	0.00	0.00	0.00	0.00
29a408a0d90dcd9bae3bf7058d05cc05	0.00	0.00	0.00	0.00	0.00	0.00
e01ab3246c50141a8720883ecc40f2cf	0.00	0.00	0.00	0.00	0.00	0.00
3874d8e3903ce59e8eeb6a5d16f06845	0.00	0.00	0.00	0.00	0.00	0.00
2d33d0f64a8e0b9f62f62266e60ec6ce	0.00	0.00	0.00	0.00	0.00	0.00
c63bbfa754ad2394486552d0906bd600	0.00	0.00	0.00	0.00	0.00	0.00
88c5ec241c80b89b2b7c2813806e57d1	0.00	0.00	0.00	0.00	0.00	0.00
62dc390932947ee70fa496d75396ec48	0.00	0.00	0.00	0.00	0.00	0.00
babf71bc3ba4b2701d480a3a698cd6b4	0.00	0.00	0.00	0.00	0.00	0.00
c5637a7d8123f8bee59cc4e55fe2b577	0.00	0.00	0.00	53.00	0.00	0.00
87fd332739f5fcc06c80117ba192eeee	0.00	0.00	0.00	0.00	0.00	0.00
29620c4424509cb7ff76afd606ec07ed	0.00	0.00	0.00	28.00	8.00	0.00
82fdd601a10f8e2ff1dd1099629eb3ed	0.00	0.00	0.00	0.00	0.00	0.00
3448d1bf6cccbbf7b14c71b09817cba4	0.00	0.00	0.00	0.00	0.00	0.00
f8cbeb5d541e3b903b0c0adf44774d4e	0.00	0.00	0.00	0.00	0.00	0.00
ecb7a0437a08d3e2a85eaf208b357803	0.00	0.00	0.00	0.00	0.00	0.00
446c7a6f20a576912bccbb4a190d5652	0.00	0.00	0.00	0.00	0.00	0.00
c12824d4f207121d5f0b43568dce3db9	0.00	0.00	0.00	32.00	0.00	0.00
337d338caa2ee065625b62dca1e82685	0.00	0.00	0.00	0.00	0.00	0.00
ffa1e305ab3ba026dd044948a407cfb3	0.00	0.00	0.00	51.00	0.00	0.00
ba985466ef7d0da4631044bfcb452ee1	0.00	0.00	0.00	0.00	0.00	0.00
fb2b1ca1e581b1f7cdba9a7645cdbf91	0.00	0.00	0.00	0.00	0.00	0.00
a8559ffb3daa33638793d28010ef2c03	0.00	0.00	0.00	0.00	0.00	0.00
2457188c02b21b4e228c436873a15b5a	0.00	0.00	2.00	6.00	4.00	0.00
11e66d32453afb66499f7d0638263ba6	0.00	0.00	0.00	50.00	0.00	0.00
1d8ef5908476853dc08e019348fe3bdc	0.00	0.00	0.00	0.00	0.00	0.00
bf02cddb401b48c39b5d95c3e09a2658	0.00	0.00	0.00	0.00	0.00	0.00
8bc5ba56c32ccf8ce9074f76c8573219	0.00	0.00	0.00	0.00	0.00	0.00
f8271cbc6322b8f322ace2e9fcc46657	0.00	0.00	0.00	0.00	0.00	0.00
4b7399a6276c3627937b32dd2639fac8	0.00	0.00	0.00	0.00	0.00	0.00
d019178ad4c9ec80af38d45a890db676	0.00	0.00	0.00	0.00	0.00	0.00
5a2dcb0f453cd553cb6bc2cdcb0bba87	0.00	0.00	0.00	0.00	0.00	0.00
d1f6bdbb6ef0c62f79b6a51b770dfda7	0.00	0.00	0.00	49.00	0.00	0.00
8c4e537fb07992cce08f4117209d65a3	0.00	0.00	0.00	0.00	0.00	0.00
06aa1652176be0d4bf38768aaa7e2f1d	0.00	0.00	0.00	0.00	0.00	0.00
24084f7475ddb1cd3562cd5fe6d8a829	0.00	0.00	0.00	0.00	0.00	0.00
e2296499bfd90e99f3e07314d9d5eab0	0.00	0.00	0.00	0.00	0.00	0.00

dded3c70201c56cb8e046e0ac3363ac2	0.00	0.00	0.00	0.00	0.00	0.00
937f4d287e85c9cc5dae434e70922576	0.00	0.00	0.00	0.00	0.00	0.00
91e2a9afc85e4014fff31dac97d45114	0.00	0.00	0.00	0.00	0.00	0.00
debc33e7811554cfb6fb6cf98c8c1ecd	0.00	0.00	0.00	0.00	0.00	0.00
a769fd25cb4cb3e65fb0b5f752ddf4cb	0.00	0.00	0.00	0.00	0.00	0.00
2372013317a61df1c1196972f4c6a23a	0.00	0.00	0.00	0.00	0.00	0.00
a5eef8793ddf5fb4b141fbd69429e7c5	0.00	0.00	0.00	0.00	0.00	0.00
d2f7f8ff7569d323a00570a0222a7177	0.00	0.00	0.00	0.00	0.00	0.00
c2988c69112b357cb20c4ab16233f292	0.00	0.00	0.00	0.00	0.00	0.00
ed26236aae38f65779b2528aff5af347	0.00	0.00	0.00	0.00	0.00	0.00
8b91faecf2083fe02d1e94ddd36fe0f7	0.00	0.00	0.00	0.00	0.00	0.00
9716da9b0dc2a3fa69ad143df88a18e4	0.00	0.00	0.00	0.00	0.00	0.00
d90e35ae59b1fa0c42fd70b8d8b8bb9f	0.00	0.00	0.00	0.00	0.00	0.00
52ec5adedde8fed8b2cad1767cb56bce	0.00	0.00	0.00	0.00	0.00	0.00
8b159b82d11fee7797ea38a880fcb15f	0.00	0.00	0.00	0.00	0.00	0.00
ad51e1a01557d5b08150da55e751a774	0.00	0.00	0.00	0.00	0.00	0.00
5fb58f42d19c0cd33fc37af4b9e07129	0.00	0.00	0.00	0.00	0.00	0.00
33c81a2f594c56c4b355cf410ce59eb6	0.00	0.00	0.00	0.00	0.00	0.00
738334f2aa10c6eec1fde1f1c9e89c18	0.00	0.00	0.00	0.00	0.00	0.00
c6e43a8cfa12c6e0930f413bff89cf0f	0.00	0.00	0.00	0.00	0.00	0.00
5efee9c3095843438b4dc482a7525812	0.00	0.00	0.00	0.00	0.00	0.00
76b33b978db4f5cb0a882d975c3c09f2	0.00	0.00	0.00	0.00	10.00	3.00
842da85e22037b51e8814d157582e44c	0.00	0.00	0.00	0.00	0.00	0.00
833242afb8b1856ae128b35880cbb982	0.00	0.00	0.00	0.00	0.00	0.00
c107d02c1fe8b1f207e5d9f35058d50e	0.00	0.00	0.00	0.00	0.00	0.00
cb0a6d2ffcec2b8bcc4b9f2ab0eed340	0.00	0.00	0.00	0.00	0.00	0.00
dee5d9a6fc6c7e8d8008e49fcd5e45f6	0.00	0.00	0.00	0.00	0.00	0.00
0ca49e4f46b9dfe5899cea1b0a5126a3	0.00	0.00	0.00	0.00	0.00	0.00
89a8f5b20a396a6cb096672dc1c4836c	0.00	0.00	0.00	0.00	0.00	0.00
d517c13b929bee05b0971b06ae334f24	0.00	0.00	0.00	0.00	0.00	0.00
0f368c525be9e3516219305eeda32f30	0.00	0.00	0.00	0.00	0.00	0.00
2257d58ebb22a4168da71744993d1ce9	0.00	5.00	0.00	0.00	0.00	0.00
0f6033c3c096c6132bb3318dae7913ef	0.00	4.00	0.00	36.00	5.00	0.00
14a09cd4dffd7653490e6bf21cb0aa07	0.00	0.00	0.00	0.00	0.00	0.00
57ffcda523f20870604cf1e70a5bb1a7	0.00	0.00	0.00	0.00	0.00	9.00
fa4000a79e20fd89506bcc9f583e09ac	0.00	0.00	0.00	0.00	0.00	0.00
ee12618d89bf204727b86de8b534bb22	0.00	0.00	0.00	0.00	0.00	0.00

31ff0e5a05fc9f8ddf9d52d0a971da49	0.00	0.00	0.00	0.00	0.00	0.00
0ec57ed096c6afa987cb6a618ca2c0d6	0.00	0.00	0.00	0.00	0.00	0.00
ad41cc2e1ee27e81d4b68cc70043d63b	0.00	0.00	0.00	0.00	0.00	0.00
4c1dec0752d66c271d5e4b51b2a2ac04	0.00	0.00	0.00	0.00	0.00	44.00
09a1bccfc09609ad6a116c8647fc54cb	0.00	0.00	0.00	0.00	0.00	0.00
f25c482791bdaeb2ab77c8bc15c447d9	0.00	0.00	0.00	0.00	0.00	0.00
c3f253fa6e85382b4456741133c54523	0.00	0.00	0.00	0.00	0.00	0.00
f82def37e8543f757fad5257a104301b	0.00	0.00	0.00	0.00	0.00	0.00
f1a4cd5f626e7502bc8edd237d7aa2c5	0.00	0.00	0.00	0.00	0.00	0.00
26599c6e17eea913818f27d5db5dfd39	0.00	0.00	0.00	0.00	0.00	0.00
f885881ff64031b61c32ea8321abfed3	0.00	0.00	0.00	0.00	0.00	0.00
034f8e14b2739a8e81b63c8f69ed18e6	0.00	0.00	0.00	0.00	0.00	0.00
03ff5e3ba8dd3cf5f374c96252b6baa0	0.00	4.00	0.00	0.00	0.00	0.00
c5adeb5d2d97d4fa61e4685b84ca9244	0.00	0.00	0.00	0.00	0.00	0.00
f563ae3c82572c093de6a9312eacf32b	0.00	0.00	0.00	0.00	0.00	0.00
88a986b5d09acc74da1bf4b9c8354c29	0.00	0.00	0.00	0.00	0.00	0.00
367b05d0c161b0a1f75fc5cd656d3f9e	0.00	0.00	0.00	0.00	0.00	0.00
abb5e2dbdde5d32f886e9e86d7def86e	0.00	0.00	0.00	0.00	0.00	0.00
fc7e53e01d083df591ac17430a7c965f	0.00	0.00	0.00	0.00	0.00	0.00
89368bcec46f82c4304c8bd5e41c015e	0.00	0.00	0.00	0.00	0.00	0.00
7f88de5f67a9165611b96cf1886df146	3.00	0.00	0.00	0.00	0.00	0.00
058d926ac34c1c0b6e0361feee596dc0	0.00	0.00	0.00	0.00	0.00	2.00
68dfb1d7eef9eb3d2507e0178443e55c	0.00	0.00	0.00	0.00	0.00	0.00
60a5d021700947c089f09c34ea202a6b	0.00	0.00	0.00	0.00	0.00	0.00
4c13685f0aab13675b07eda3e79defc6	0.00	0.00	0.00	0.00	0.00	0.00
49c4ae4588c2a5122e4995b1b2a3d84c	0.00	0.00	0.00	0.00	0.00	0.00
2511da09a754cbbb896cc5c84d3ab8df	0.00	0.00	0.00	0.00	0.00	0.00
4a3d18e4d05a0fbb548f6d399dfb6f81	0.00	0.00	0.00	0.00	0.00	0.00
2bb2834ca255d48c0ccb9b5c293d690e	0.00	0.00	0.00	0.00	0.00	0.00
763b6677c9381b4d162f5541912369d6	0.00	0.00	0.00	0.00	0.00	0.00
16bbf3e164a08992a5df0fc32f7a22b2	0.00	0.00	41.00	0.00	0.00	0.00
f45816e0e97879e21a00791c0567e183	0.00	0.00	0.00	0.00	18.00	0.00
6b642708032d840b43c290b8af88becf	0.00	0.00	0.00	0.00	0.00	0.00
86a5282ea5df80c6b91f73bab78ea34a	0.00	0.00	0.00	0.00	0.00	0.00
4f4572ae0f074d89ee126a1d2197c9cd	0.00	0.00	0.00	0.00	0.00	0.00
d231b54702ca1a31206fc88f9af0d533	0.00	0.00	0.00	0.00	0.00	0.00
ba49e04170826f2502791987340e540e	0.00	0.00	0.00	0.00	0.00	0.00

09c5b4c082eebcc07129fc03cd7c99b8	0.00	0.00	0.00	0.00	0.00	0.00
c798b9afbf4041fafc6b6b3656dbcdbb	0.00	2.00	7.00	0.00	0.00	0.00
77049d75f92fc520bc58d4f5e9f1335c	0.00	0.00	2.00	3.00	0.00	0.00
e604514b21d22f08811c9fa13eafb8c5	0.00	0.00	0.00	22.00	18.00	0.00
dae9e3bcbe8d6f6e3919b91df6fc9700	0.00	0.00	0.00	0.00	7.00	0.00
f25fcc2bd0dcdf764e2ccefed377db09	0.00	0.00	0.00	0.00	0.00	0.00
e4ac74f054f84338f7a8a7fe943661a9	0.00	0.00	0.00	0.00	0.00	0.00
531b79bdb8899fe863b655334ead5d1a	0.00	0.00	0.00	0.00	0.00	0.00
8a4ab4634d1782bff27595f7c82824fa	0.00	0.00	0.00	0.00	0.00	0.00
8fdf67a64309b199a8c6e65622b00197	0.00	0.00	0.00	0.00	0.00	0.00
63503a799f9dcce3a07db9f081c86145	0.00	0.00	24.00	0.00	0.00	0.00
6859fb0037d9dff7bbd9652fe91554a8	0.00	0.00	0.00	0.00	0.00	0.00
c4aa9ec8aa8f6f499049780c5f0013ae	0.00	0.00	0.00	0.00	0.00	0.00
38d2519553049327b886ed87319a6f68	0.00	0.00	0.00	0.00	0.00	0.00
af7390f88610b6c0312814560553298b	0.00	0.00	0.00	0.00	0.00	0.00
9ecd8615c5fbaf174fe204ac0b55336a	0.00	0.00	0.00	0.00	0.00	0.00
bf9bcb13fd876a50bb54bb5419f7319d	0.00	0.00	0.00	0.00	0.00	0.00
9e4f6732752e5ea35d579f1b55c4e689	0.00	0.00	0.00	0.00	0.00	0.00
12cc654fc2fa1e5d20844415e038f516	0.00	0.00	0.00	0.00	0.00	0.00
8fd8abd8c930726f76324fcba08751e8	0.00	0.00	0.00	0.00	0.00	0.00
c80b3d1adcf8ab54b1b218f44967ca20	0.00	14.00	0.00	0.00	0.00	0.00
1dfdcd7db65f345b41fbdf1d04386565	2.00	0.00	0.00	0.00	0.00	2.00
4dbe3bdab41aeec79e52c78c1281615d	0.00	0.00	0.00	0.00	0.00	0.00
2a25d9a914d0991e7ae76213883a9d2b	0.00	0.00	0.00	0.00	0.00	0.00
fbeb9053f9bf01a9a9bb37f223eb4611	0.00	0.00	0.00	0.00	0.00	0.00
6f762df47c9506eddc041be0fef57077	0.00	0.00	0.00	0.00	0.00	0.00
ae84f2bb01a0d6c53b4ef5a2b8ba3cc1	0.00	0.00	0.00	0.00	0.00	0.00
664e8cfe10825f9ab2f3d2ac3889d14a	0.00	0.00	0.00	0.00	0.00	0.00
179f372ec625aa16e30ff5649e3950d8	0.00	0.00	0.00	0.00	0.00	0.00
b4b94432618bbdc67593a0d88058fd5c	0.00	0.00	0.00	0.00	0.00	0.00
7a5910d122f12c870f0745dbf51259f6	0.00	0.00	0.00	0.00	0.00	0.00
240c80d2b769302115cd17e19b3dd4da	0.00	0.00	0.00	0.00	0.00	0.00
2cf1bab1634b7e2332311d2d98d1cef9	0.00	0.00	0.00	0.00	0.00	0.00
6359ccb5e56e9bf432a30cba2540c34c	0.00	0.00	0.00	0.00	0.00	0.00
60bcddd7f79de23c58dc1c323f7733cd	0.00	0.00	0.00	0.00	0.00	0.00
1b598fab51ad67234b99d1632a136f69	0.00	0.00	0.00	0.00	0.00	0.00
15f410376c42a2a3617e9e4e39d58d7d	0.00	0.00	0.00	0.00	0.00	0.00

d3ad926a31b1606ab40e019127bf3b96	0.00	0.00	0.00	0.00	0.00	0.00
b5404f71dc723483cdc497f978d3f132	0.00	0.00	0.00	37.00	0.00	0.00
0072b0040e39e5d64afc5038682721dc	0.00	0.00	0.00	0.00	0.00	0.00
dac5ff72546bbc26137b128defe558c4	0.00	0.00	0.00	0.00	0.00	0.00
b31be54e9b701d1d978d9e22fc999d99	0.00	0.00	0.00	0.00	0.00	0.00
838cf9dfd6fbf7970ca96b9c50698d5c	0.00	0.00	0.00	0.00	0.00	0.00
938d4e309164930fc947787da64ac634	0.00	0.00	0.00	0.00	0.00	0.00
ae92751ce0103cf1fe77dd7f219da00c	2.00	2.00	5.00	0.00	5.00	0.00
1d79b9510e8f0bcc3a7aa6a9aea58bd9	0.00	0.00	0.00	0.00	0.00	0.00
a5fa49d3b80baf925ed7f607bb7d1154	0.00	0.00	0.00	0.00	0.00	0.00
96f64b6abe313a652236c89fb0b8eb5d	0.00	0.00	0.00	0.00	0.00	0.00
7ea66768a2f9a959c8d525ec785c3cd0	0.00	0.00	0.00	0.00	0.00	0.00
85d8915983e425adb141438d74ef9e47	0.00	0.00	0.00	0.00	0.00	0.00
a027b017575a1c3ef925d7fb1192b9d8	0.00	0.00	0.00	0.00	0.00	0.00
20b4df82de0c3c8cc410c2f3942dfcae	0.00	0.00	0.00	0.00	0.00	0.00
03212076333c0b956a43868d6aba0235	0.00	0.00	0.00	0.00	0.00	0.00
e0940b1a127eae44bd5f5ebd833ca639	0.00	0.00	0.00	0.00	0.00	0.00
56aa91b83f97f0e967295d3f17554ed5	0.00	0.00	0.00	0.00	0.00	17.00
aa27af2b3ae7640bff67b8c596f81407	12.00	0.00	0.00	0.00	0.00	0.00
e03a2cdd0bb51190a95d1133f3a8338c	0.00	0.00	0.00	6.00	0.00	0.00
63aef7cb872cb63727d16ad8a1109902	0.00	0.00	0.00	0.00	0.00	0.00
7521c39ea76080cfa9eac884f1890bb2	0.00	0.00	0.00	0.00	0.00	0.00
e68379fb0f5079fe493f3d8b95b9fe3c	0.00	0.00	0.00	0.00	0.00	0.00
95aded8ec7b1cfe05b7737bee17ab350	0.00	0.00	0.00	0.00	0.00	0.00
d6e45c76d5ddcae0057978a6e07c39aa	0.00	0.00	0.00	0.00	0.00	0.00
6632332cf3c086cd31edfc0c8fa430d6	0.00	0.00	0.00	0.00	0.00	0.00
dbe016c1518678a948f9d28258b35f9a	0.00	0.00	0.00	34.00	0.00	0.00
0f252d8a353b3576b9cff9255c3f6d48	0.00	0.00	0.00	0.00	0.00	0.00
a12a42ea7e4bb807c91ceb3636312308	0.00	0.00	0.00	0.00	0.00	0.00
204fb2f1e67ca5a28ce357e0f52ab0dc	0.00	0.00	0.00	0.00	0.00	0.00
e589a639d458b581ea775a1bef84906b	0.00	0.00	0.00	0.00	0.00	0.00
dd59ed4328f6a51da2019387b881acbd	0.00	0.00	0.00	0.00	0.00	0.00
f56b725c8fddb2e1fe9b06ddd9e636ad	0.00	0.00	0.00	0.00	0.00	0.00
e9653330ac1b375e62c96b741bf93d3e	0.00	0.00	0.00	0.00	0.00	0.00
2e06a484b8f97b03db6d2f2074fc4728	0.00	0.00	0.00	0.00	0.00	0.00
5740cdc008dc21e3e2b8e2cb5a2fd8b4	0.00	0.00	0.00	0.00	0.00	0.00
37fbaf0f480dcd6a587d8dd107c6ffda	0.00	0.00	0.00	0.00	0.00	0.00

e09f472ad49df5249fbd6dca53b78ba5	0.00	0.00	0.00	0.00	0.00	0.00
c3a5f727430f22f6d3fb64ff6235eefa	0.00	0.00	0.00	0.00	0.00	0.00
66320203e0ce75bf3b710408a1f21b75	0.00	0.00	0.00	0.00	0.00	0.00
d6782eba325f4f8f30dcc7a29b479be1	0.00	0.00	0.00	0.00	0.00	0.00
525ab6e17f2a5039c135ed54a9662b61	0.00	0.00	0.00	0.00	0.00	0.00
4b8a2135a97ee2d3bbaf5e33da60580c	0.00	0.00	0.00	0.00	0.00	0.00
710fa6af24c5f48bc290c13d882dbac0	0.00	0.00	0.00	25.00	0.00	0.00
a5484685b1136a5f4cffe68fbc6340de	0.00	0.00	0.00	0.00	0.00	0.00
1ec815431d4a89c8babc3b0a251eef88	0.00	0.00	0.00	0.00	0.00	0.00
24eb6965618bdb55df1eb32aa8b929ee	0.00	0.00	0.00	0.00	0.00	0.00
250e368bb6650f7a923f9016c2af1272	0.00	0.00	0.00	0.00	0.00	0.00
915eff1940374bf823d7d8833a4e939a	0.00	0.00	0.00	32.00	0.00	0.00
48ac7eb08f9fdff4c13559f18bbd6d24	0.00	0.00	0.00	0.00	0.00	0.00
6b6795ae348b96ece97d2f3aa4a75e5a	0.00	0.00	0.00	0.00	0.00	0.00
7d64125e4fdd67e9a9a54d802efd665c	0.00	0.00	0.00	0.00	0.00	0.00
9cfaa684bd1c73b4f03505a3a1b40529	0.00	0.00	0.00	0.00	0.00	0.00
f6160c1ccee6d7f4c4ed9d0a7b931701	0.00	0.00	0.00	0.00	0.00	0.00
c0d1e675ef43b3d0ef7ae0105700209a	0.00	0.00	0.00	0.00	0.00	0.00
6c540009c0c02df5baad73a2576a6cba	0.00	0.00	0.00	6.00	0.00	0.00
76ea4c5bca1c0f7e1f1591fb2544e356	0.00	0.00	0.00	0.00	0.00	0.00
9d498b422921d1d2c4cc441a86d93f5c	0.00	0.00	0.00	31.00	0.00	0.00
3b7e6649a8182df0a88f85bc8d283e62	0.00	0.00	0.00	31.00	0.00	0.00
d59fd89455eb2c949333a3b03f9dca16	0.00	0.00	0.00	31.00	0.00	0.00
25bb8fdf9c9190b48ac25a105400b37d	0.00	0.00	0.00	31.00	0.00	0.00
30480b99b384fe9bd79e86ab176a51c4	0.00	0.00	0.00	0.00	0.00	0.00
f7f26b454de0915e27dc96cb3f94ed6d	0.00	0.00	0.00	0.00	0.00	0.00
c945805cd5515446c8e5ef37d0589884	0.00	0.00	0.00	0.00	0.00	0.00
b055d667e1ad72dbfec4ceecc6a42a6b	0.00	0.00	0.00	0.00	0.00	0.00
fe808260d3bf3ef97e67cbc8df6c18ba	0.00	0.00	0.00	0.00	0.00	0.00
eebf0984ed8ed0f04f0b7ba319c872ff	0.00	0.00	0.00	0.00	0.00	0.00
53e68c2e767ab23c5c93aa68e5b8c439	0.00	0.00	0.00	0.00	0.00	0.00
ab90bae8cd813e156512aeef32aae88a	0.00	0.00	0.00	0.00	0.00	0.00
aad078fbbe80f6cb67f8f66c1c065b36	0.00	0.00	0.00	0.00	0.00	0.00
29ad8576ae7b732ecb965f0b371b6967	0.00	0.00	0.00	0.00	10.00	0.00
3ea51db1e451d380412fd2188040f1f8	0.00	0.00	0.00	0.00	0.00	0.00
638009b7e1fbd98d19892769282ae35d	0.00	0.00	0.00	0.00	0.00	0.00
238ca540b32601307b53c8b735a32574	0.00	0.00	0.00	0.00	0.00	0.00

91ef2da4e2c80a9e0d07779d43a22c96	0.00	0.00	0.00	0.00	0.00	0.00
5c0ac68883e3295c9dff09693313f723	0.00	0.00	0.00	0.00	0.00	0.00
e749fa7b9d6c12fc8034e3cbc0f15ade	0.00	0.00	0.00	0.00	0.00	0.00
3f7e05fa6d0c5937f4d9ad031e55bb3c	0.00	0.00	0.00	0.00	0.00	0.00
93b54ce9241235396ca7368033e08319	0.00	0.00	0.00	0.00	0.00	0.00
3015914df5942416853f681a487c44e9	0.00	0.00	0.00	0.00	0.00	0.00
35aa089107d0b1c813d8034a915a04e8	0.00	0.00	0.00	0.00	0.00	0.00
1a2a1c42a4036689b888a8d5b20faca5	0.00	0.00	0.00	0.00	0.00	0.00
23998569c5ce9a374f4b813f79799ea4	0.00	0.00	0.00	0.00	0.00	0.00
327818bfc4950e25f683b00fcee3af19	0.00	0.00	0.00	0.00	0.00	0.00
3e686f166f364e8b5b0b568d43b8875b	0.00	0.00	0.00	0.00	0.00	0.00
e39f1a4d93cb61b45426ad0c5b3466c8	0.00	0.00	0.00	0.00	0.00	0.00
9756286ae1783ce81aa1b75fe067d73d	0.00	0.00	0.00	0.00	0.00	0.00
58f1edac64af5378fd82e3ee097a64a9	0.00	2.00	0.00	0.00	0.00	0.00
c33174077687af320cc02117efae9355	0.00	8.00	0.00	21.00	0.00	0.00
f33c5e33659ce53afa45b0d52c021249	0.00	0.00	0.00	0.00	0.00	0.00
af77baff8a86a9ebf52ba1cbc534ab54	0.00	0.00	0.00	0.00	0.00	0.00
d2d444fd54bca80fbe5fa5a4c9be57fc	3.00	0.00	0.00	0.00	0.00	3.00
0019db7066a3a6e30c7df7da72843966	0.00	0.00	0.00	0.00	0.00	0.00
93059a3e70e92e9d903a2225eda6f7f7	0.00	0.00	0.00	0.00	0.00	0.00
6910c4c03eaec99975faf72b6a2a01ab	0.00	0.00	0.00	0.00	0.00	0.00
ac2c7eed66100de57980539055342cc0	0.00	0.00	0.00	0.00	0.00	0.00
ffa28c0c4f9f3720b646af2871b729a4	0.00	0.00	0.00	0.00	0.00	0.00
7e09c8b438376367a8ccfb08cfbd111b	0.00	0.00	0.00	0.00	0.00	0.00
08f4073691ab5eda6a6e49fd414de91d	0.00	0.00	0.00	0.00	0.00	0.00
4d432a50e008d9a209e0ecf1eea2bb14	2.00	4.00	6.00	0.00	4.00	0.00
d68c559f223d8ba995425040c804352c	0.00	0.00	0.00	0.00	0.00	0.00
aa92f97ed7191399f223582ca5751d7e	0.00	0.00	0.00	0.00	0.00	0.00
6e1866aaa5199c898a75ca81d8054aed	0.00	0.00	0.00	0.00	0.00	0.00
6a96a870237af92c5c33f6229358f227	0.00	0.00	0.00	0.00	0.00	0.00
7bb117d1521f0d4d94b0d5ba5224c316	0.00	0.00	0.00	0.00	0.00	0.00
bc3a9abe1aee4a9b1432a213380f8e63	0.00	0.00	0.00	0.00	0.00	0.00
98f47889e275976d2894732f28ec2e65	0.00	0.00	0.00	0.00	0.00	0.00
d6b780550b7231b0dcb3381d414c38fd	0.00	0.00	0.00	0.00	0.00	0.00
172fdaba7d7a1d94b5346e9bc0995ec9	0.00	0.00	0.00	0.00	0.00	0.00
e5147e0e6fd7aafd49103abd85a6af62	0.00	0.00	27.00	0.00	0.00	0.00
96c01ef6f47fe14d6742c1a8e02fdad8	0.00	0.00	0.00	0.00	3.00	0.00

31d5ab366a86a8a34fd361957c8bb4e9	0.00	0.00	0.00	0.00	0.00	0.00
1b3433088ac8b0afe98257fc61a05ddf	0.00	0.00	0.00	0.00	0.00	0.00
0387a73fddaa01b5ed4261fda49f4622	0.00	0.00	0.00	0.00	0.00	0.00
bffbe6a9327f107aee0f2bb01618cc6f	0.00	0.00	0.00	27.00	0.00	0.00
f4edf21b2076c9a5ce41c99fdf214e6e	0.00	0.00	0.00	27.00	0.00	0.00
0fe82f6749ff15959a7df6e27948c1ad	0.00	0.00	0.00	0.00	0.00	0.00
3ec0b087b065832f08fba70d98bf6935	0.00	0.00	0.00	0.00	0.00	0.00
bec92fb96d0a98cd9cacf88d6a0365ef	0.00	0.00	0.00	0.00	0.00	0.00
d45b52fa2031d4796c3678d23a23da33	0.00	0.00	0.00	0.00	0.00	0.00
bfb9c054915bb876f542b784e4a51ddf	0.00	0.00	0.00	0.00	0.00	0.00
d531a1bcfa2094268b49c787178ba5ec	0.00	0.00	0.00	0.00	0.00	0.00
3d022a5dc897fbfd2d5ae58791e258c1	0.00	0.00	0.00	0.00	0.00	0.00
58c1bc510a08ce4b02fbd1137281ee38	0.00	0.00	0.00	0.00	0.00	0.00
0ebb9a3c8fe5d528562c9fda512cc5fc	0.00	0.00	0.00	0.00	0.00	0.00
301a7f080e8f1eacf27ae55b1431acd4	0.00	0.00	0.00	0.00	0.00	0.00
8963e936dac25547932bda2fd90c9ee3	0.00	0.00	0.00	0.00	0.00	0.00
2258e0f79706060afcb1b187bdf78587	0.00	0.00	0.00	0.00	0.00	0.00
490bdaf9596f84e0de6eb178d461a952	0.00	0.00	0.00	0.00	0.00	0.00
a605b6003e7f0f92705852bff6cd97c7	0.00	0.00	0.00	0.00	0.00	0.00
eeebc571ecf39da0fcb78a8b7dde47df	0.00	0.00	0.00	0.00	0.00	0.00
6e6054fc695481706ef659261661107f	0.00	0.00	0.00	0.00	0.00	0.00
e9cef5d0f6fc0ca20857694c64389da5	0.00	26.00	0.00	0.00	0.00	0.00
a7174350002e8baf61e2d992912688a0	0.00	0.00	0.00	0.00	0.00	0.00
5fcab3f2b702695ef44ab03a5104c952	0.00	0.00	26.00	0.00	0.00	0.00
dafce97b585259d5fa9b92527e43f910	0.00	0.00	0.00	26.00	0.00	0.00
8b2214feadd825c3e070e20a89c54e5f	0.00	0.00	0.00	0.00	0.00	0.00
42005de024bc684bfa6de9f56de2862a	0.00	0.00	0.00	0.00	0.00	0.00
7afc387d070a43d924bbf2f8540c1014	0.00	0.00	0.00	0.00	0.00	0.00
e469411be93d59d005f998068149364f	0.00	0.00	0.00	0.00	0.00	0.00
50a9aba898c2ee24cd82c9a426ace190	0.00	0.00	0.00	0.00	0.00	0.00
f705dd8a427df1ac8c2190d5b86ae31d	0.00	0.00	0.00	0.00	0.00	0.00
5f279925d0d6f3bdeca226e5bae070ff	11.00	14.00	0.00	0.00	0.00	0.00
6a0d6919c45cca7fe11b4982f4a90a48	0.00	0.00	0.00	0.00	0.00	0.00
348c097189a2d3b7c082301f4b15198e	0.00	0.00	6.00	0.00	0.00	15.00
36357a788ae72978f49bec48e4463acd	0.00	0.00	0.00	0.00	0.00	0.00
08c782e0744f5076f7fc81795b4ea7fc	0.00	0.00	0.00	25.00	0.00	0.00
afc9eb9c36a33c5449989eb7f09ac63b	0.00	0.00	0.00	25.00	0.00	0.00

68c3a34f2ef0796320db20baf34043ab	0.00	0.00	0.00	25.00	0.00	0.00
5d6e8dafe9fc924f5875e7bc2d63e908	0.00	0.00	0.00	0.00	0.00	0.00
1ae07c6d06e89d4d22f765001c38656a	0.00	0.00	0.00	0.00	0.00	0.00
bb336f3634c1aeb63586fcc4fbdaaf5e	0.00	0.00	0.00	0.00	0.00	0.00
3d0409f8d3cc4518ffc12eeb1b1feed8	0.00	0.00	0.00	0.00	0.00	0.00
4d39e11cf782037a50566473cf48f574	0.00	0.00	0.00	0.00	0.00	0.00
fb11d1c820f765bb02d5121725aeaaf7	0.00	0.00	0.00	0.00	0.00	0.00
3b21b22101fdcf1143a46ded7e073a04	0.00	0.00	0.00	0.00	0.00	0.00
eb597b9769e8ffc1f6ce62927dba66b4	0.00	0.00	0.00	0.00	0.00	0.00
845a3b87cce635241e34a73a56b8c84b	0.00	0.00	0.00	0.00	0.00	0.00
e0fb6d17b5a44bb15475b9a50eb21696	0.00	0.00	0.00	0.00	0.00	0.00
6156f4f779000470670564fdf982ddc2	0.00	0.00	0.00	0.00	0.00	0.00
5f0fe35704a82df5fca6a53b7c2bd3a7	0.00	0.00	0.00	0.00	20.00	0.00
2c40a6c6f8ece60013a5bd0c4f0080a5	0.00	0.00	0.00	0.00	0.00	0.00
aeb45d4555a30e901eb50596beac8c88	0.00	0.00	0.00	0.00	0.00	0.00
83af309f031f918e296f080b663e8dbe	0.00	0.00	0.00	0.00	0.00	0.00
9eb4d1ffb5f94c5266950014efa05daf	0.00	0.00	0.00	0.00	0.00	0.00
75a2fb16178a80aa5a040986f426feac	0.00	0.00	0.00	0.00	0.00	0.00
a6f145d681807ba00fab8f418a9b4baf	0.00	0.00	0.00	0.00	0.00	0.00
96f2c5b670dc93ba2dd766b508fb12ca	0.00	0.00	0.00	0.00	0.00	0.00
9786622e67bc4782f3dec217a40a6e5a	0.00	0.00	0.00	0.00	0.00	0.00
f22d7276c7cff29f70dba472ec48076b	0.00	0.00	0.00	0.00	0.00	0.00
fc12fe2a61b8072cc81d3624ce43ddac	0.00	0.00	0.00	0.00	0.00	0.00
bb8ed159e591180f2dbf846ba359e676	0.00	0.00	0.00	0.00	0.00	0.00
ee5e49e8cfec5fc498f9f8e135c98571	0.00	0.00	0.00	0.00	0.00	0.00
04398b52b455073d9aba6a3a930f76f8	0.00	0.00	0.00	0.00	0.00	0.00
923051981e348e9b2412a081f7b77da4	0.00	0.00	0.00	0.00	0.00	0.00
0c4560511f165652968b5becbbb27713	0.00	0.00	0.00	0.00	0.00	0.00
0897f5701faf86dea626fb525535add0	0.00	0.00	0.00	0.00	0.00	0.00
db1ef97b9c2c2ef5763a0852ad67fcc1	0.00	0.00	0.00	0.00	0.00	0.00
6156fd18a91f1949a6d27585260551a1	0.00	0.00	0.00	0.00	0.00	0.00
41b0539141e529558b4ba2a209cf6df5	0.00	0.00	8.00	0.00	0.00	0.00
1d164f94c4b13e11f35817ff016e8f01	0.00	0.00	0.00	0.00	0.00	0.00
d1898417503a1db3852f696dd8c1f3b0	0.00	0.00	0.00	0.00	0.00	0.00
9e9b74b1ccd8d82f4fef7650dd2fdc1f	0.00	0.00	0.00	0.00	0.00	0.00
f02e330683bd88f572dc3a63a3d92a9d	0.00	0.00	0.00	0.00	0.00	0.00
ba0f62debca6882964046f925d7da206	0.00	0.00	0.00	0.00	0.00	0.00

6e18bd5f07d097ca3912e306d4375b45	0.00	0.00	0.00	0.00	0.00	0.00
19ea9936b45858b90508132fd233e645	0.00	0.00	0.00	0.00	0.00	0.00
8724150e28b5b08ce2bd4f1d05cfda2f	0.00	0.00	0.00	0.00	0.00	0.00
8deefbf775c3b682e4854b26441890d1	0.00	0.00	0.00	0.00	0.00	0.00
dafc80d594d7a9b05ecc0368d3d4d7f1	0.00	0.00	0.00	0.00	0.00	0.00
a2b48cc2faf915571fda275fbb7a404e	0.00	0.00	0.00	0.00	0.00	0.00
10c807b9ae7c51434f618885e679e1cf	0.00	0.00	0.00	0.00	0.00	0.00
05a1ac81d0c6ce4df6d09b21b7faa7f1	3.00	0.00	6.00	0.00	0.00	0.00
0f91539f458a1b14e65aa384f250c0f9	0.00	0.00	0.00	0.00	11.00	0.00
0a84a91a9c24a635acc70a4f0b7a8fa5	0.00	0.00	0.00	22.00	0.00	0.00
55005bf35fb08688734ddd97acd43371	0.00	0.00	0.00	22.00	0.00	0.00
cb01cd315edd57878298308ebd2d1053	0.00	0.00	0.00	0.00	0.00	0.00
33a095ddbeb6a060e98aaf0fbc885801	0.00	0.00	0.00	0.00	0.00	0.00
11ea9b495c26c2935ad79e55f6cb5f9d	0.00	0.00	0.00	0.00	0.00	0.00
9c6454f05a4a314db5b0a4d49cef3f83	0.00	0.00	0.00	0.00	0.00	0.00
9f009d244cac331e17e06e4a9aec4119	0.00	0.00	0.00	0.00	0.00	0.00
8752d647c05e8109a6a7d176915b1c93	0.00	0.00	0.00	0.00	0.00	0.00
a418d313733bc77dc9bd11e0c9c5021d	0.00	0.00	0.00	0.00	0.00	0.00
2891141c9782d16d46981ad36421ef9d	0.00	0.00	0.00	0.00	0.00	0.00
8bd1b0b33b6eb8a9af0088b805118d8d	0.00	0.00	0.00	0.00	0.00	0.00
df2051abd85f04bbfbfe7698fe24419f	0.00	0.00	0.00	0.00	0.00	0.00
75dfdb31a28fa1463e225e2369fa6c07	0.00	0.00	0.00	0.00	0.00	0.00
e43655f59fc072a14b340db22ce1bc7c	0.00	0.00	0.00	0.00	0.00	0.00
0a38fc94fafd3077e9ebcaa254e75a87	0.00	0.00	0.00	0.00	0.00	0.00
a526d7201660b93001d014ad84b2965b	0.00	0.00	0.00	0.00	0.00	0.00
a733eb1e1e256a8b52dbd367a37d9460	0.00	0.00	0.00	0.00	0.00	0.00
19f59de8f29a6556376200e32f7c4684	0.00	5.00	0.00	0.00	0.00	0.00
e3c5f9bb6b22d5930bde7a44e456cfef	0.00	0.00	11.00	0.00	3.00	7.00
0e9835f14673546563cce6ec729464f4	0.00	0.00	0.00	0.00	0.00	0.00
9744cec9f1b0337270dfb13e79bf7095	0.00	0.00	0.00	0.00	0.00	0.00
b428e627977a7fd74c57d202e315cbda	0.00	0.00	0.00	0.00	0.00	0.00
e230a638fb3f748c90fbf43a6cc7b6f7	0.00	0.00	0.00	21.00	0.00	0.00
878fd5ad92e2f7bea2881e6df1f9bdda	0.00	0.00	0.00	21.00	0.00	0.00
d1936a86defac30e61a0595fab1b5db3	0.00	0.00	0.00	15.00	0.00	0.00
aa8d20f08616b380e3d4bb820afc8d30	0.00	0.00	0.00	0.00	0.00	0.00
dc2297af5997aa75a8f0ced329d6e115	0.00	0.00	0.00	0.00	0.00	0.00
e9c4784dd3616810c67e8904fa3055b8	0.00	0.00	0.00	0.00	0.00	0.00

4503794b642e07c9b13d759c06f15d21	0.00	0.00	0.00	0.00	0.00	0.00
11f07e3dac6a7271fe139563c85a483f	0.00	0.00	0.00	0.00	0.00	0.00
2575a46997c095ced97187a198338a33	0.00	0.00	0.00	0.00	0.00	0.00
9b3c1d68558f1548d42e902ee248d262	0.00	0.00	0.00	0.00	0.00	0.00
5fb67209ba9659c7990f108cddcd845c	0.00	0.00	0.00	0.00	0.00	0.00
470f59b7f6af687729940307d4a50f89	0.00	0.00	0.00	0.00	0.00	0.00
c745446b5af209667bced6b11dfb1265	0.00	0.00	0.00	0.00	0.00	0.00
0c7adf4e33084dad18b34066215fa066	0.00	0.00	0.00	0.00	0.00	0.00
2b1b78f7b92e69d1b7ad7ebe1db1ad77	0.00	0.00	0.00	0.00	0.00	0.00
dab8501a9aad34031c910e0094b5e785	0.00	0.00	0.00	0.00	0.00	0.00
765f1eb74ddbeef72492ea58cb573acf	0.00	0.00	0.00	0.00	0.00	0.00
4574c6347c9c911314c3e276468c3386	0.00	0.00	0.00	0.00	0.00	0.00
20ba619f4fa7dff20c0c0e4f7fe69be8	0.00	0.00	0.00	0.00	0.00	0.00
3a9ae0fe7da1cd0ddc70785ad815c717	0.00	0.00	0.00	20.00	0.00	0.00
faba84cc5c32378df017ef08e3e74a52	0.00	0.00	0.00	20.00	0.00	0.00
f9b668201c6aed4516c4560c4b7793af	0.00	0.00	0.00	0.00	0.00	0.00
14ebb4d6ca7c1392d59f21f52d37841e	0.00	0.00	0.00	0.00	0.00	0.00
a427625a303e8eddae88e4c65689723b	0.00	0.00	0.00	0.00	0.00	0.00
298a20d99e94396bc76bdfe2f7ba9689	0.00	0.00	0.00	0.00	0.00	0.00
b26a4fbd6bd5b3540c92d797b52ff9a1	0.00	0.00	0.00	0.00	0.00	0.00
83bfa98d28fff4f4001015f751bc8338	0.00	0.00	0.00	0.00	0.00	0.00
dd13548694afdb5844156b586df576c8	0.00	0.00	0.00	0.00	0.00	0.00
2d0c6bd7601f0c7848f1597213df6148	0.00	0.00	0.00	0.00	0.00	0.00
4fb5b65850bdaf3d8180ee0e04437c21	0.00	0.00	0.00	0.00	0.00	0.00
0ce249e8b2d381db88ef43702e213e22	0.00	0.00	0.00	0.00	0.00	0.00
5a2ae7395885031b9d4c9e8cbc488d06	0.00	0.00	0.00	0.00	0.00	0.00
425ddb2a6ac3527353805f2e4b864562	0.00	0.00	0.00	0.00	0.00	0.00
4b32215da7f3bc25b59f4a73d5c61e85	0.00	0.00	0.00	0.00	0.00	0.00
5b9c4cf767fbb4312976b3094ca7a328	0.00	0.00	0.00	0.00	0.00	0.00
4fd5fc3225b06958b57b5cd2814a2af4	0.00	0.00	0.00	0.00	0.00	0.00
54c75881c98032cd2dc3460302a97faa	0.00	0.00	0.00	0.00	0.00	0.00
214d0d21b674e4e001c2bfe054124bb3	0.00	0.00	0.00	0.00	0.00	0.00
862f30035cd0259dde06ae2b50c38bd3	0.00	0.00	0.00	0.00	0.00	0.00
2da69fe62ccf106c32d22afe8cb8913f	0.00	0.00	0.00	0.00	0.00	0.00
ddd38c21245b5d6ba3976039e7e7953f	0.00	0.00	0.00	0.00	0.00	0.00
57dd979944b9493df6fb61b654f8a80a	0.00	19.00	0.00	0.00	0.00	0.00
2518395d84a96802904b2299447d2bba	0.00	0.00	0.00	0.00	0.00	0.00

750faa6e6a3e61b3193f2bc017ddfb90	0.00	0.00	0.00	0.00	0.00	0.00
86f8b1d580942ff577a32c37964e4cf8	0.00	0.00	0.00	0.00	0.00	19.00
20c57e2c0fff998a2d9771727266795d	0.00	0.00	0.00	19.00	0.00	0.00
e9616da2db8438a4449b605d41bbe6d2	0.00	0.00	0.00	19.00	0.00	0.00
7a479581fbd914407ce742e28ca0546f	0.00	0.00	0.00	0.00	0.00	0.00
c3425b43ff7cf6ab8713b36b73b8c29d	0.00	0.00	0.00	0.00	0.00	0.00
832055a5fed3e421d136058cc74725ab	0.00	0.00	0.00	0.00	0.00	0.00
44db67973a362886a170291b0f1c5bb7	0.00	0.00	0.00	0.00	0.00	0.00
6c12db596f5638739e999be951cf1d94	0.00	0.00	0.00	0.00	0.00	0.00
083113e22077e462a57c5603e9a0118e	0.00	0.00	0.00	0.00	0.00	0.00
0c6a5c76f5a7b7cf68fe520e00acd214	0.00	0.00	0.00	0.00	0.00	0.00
f2148f6cdd525affb66a49b7c5bdf609	0.00	0.00	0.00	0.00	0.00	0.00
a2f088cae72697076c50c607e62a3a5a	0.00	0.00	0.00	0.00	0.00	0.00
dcd557897dc9de6b9590d15edf1d7c74	0.00	0.00	0.00	0.00	0.00	0.00
f96955eed82cc52106d4a22c3b7147d2	0.00	0.00	0.00	0.00	0.00	0.00
e0f86dc04a408ec753a6862751829728	0.00	0.00	0.00	0.00	0.00	0.00
aff5574488c4921323d2777556f625da	0.00	0.00	0.00	0.00	0.00	0.00
5bdfae65ce95099865ffe021cc92ec9e	0.00	0.00	0.00	0.00	0.00	0.00
0112629670509c003c21dd5cd6dc9d9c	0.00	0.00	0.00	0.00	0.00	0.00
b37213ef0ec77451dc5615102f1fadd7	0.00	0.00	0.00	0.00	0.00	0.00
b882c8f92806a5cceae079731fbdf3df	0.00	0.00	12.00	0.00	0.00	0.00
22632c954ef155ebb0c3d54d46fac742	0.00	0.00	2.00	0.00	0.00	0.00
8be449490a4877d30785e30a483fb586	0.00	0.00	2.00	0.00	4.00	0.00
90fb51e1a56241ae033e5c1806939f24	0.00	0.00	0.00	0.00	0.00	0.00
8681f3381b313d8c7d0326da9e73204d	0.00	0.00	0.00	0.00	0.00	0.00
40733c349bb72e5a657ae7f501f86eaf	0.00	0.00	0.00	0.00	0.00	0.00
9c5c81e7bf00adad6a67767e60681225	0.00	0.00	0.00	0.00	0.00	0.00
a7aa4a9c9a987e46d3f0330457f15693	0.00	0.00	0.00	0.00	0.00	0.00
70d1622fbb37b1a7de863284fb83e241	7.00	0.00	0.00	0.00	0.00	0.00
1b83d2e5e671a7a0b46764746b60572a	0.00	0.00	0.00	0.00	0.00	0.00
1b78092535b5e4acefb9d7fb8859aad4	0.00	0.00	0.00	0.00	0.00	0.00
23357e9b7760997ac73b1a043046744f	0.00	0.00	0.00	18.00	0.00	0.00
63e0f7a3d9ecd273956314a549270088	0.00	0.00	0.00	18.00	0.00	0.00
fcb780fe9c2804cc5cd71fffe07a9058	0.00	0.00	0.00	0.00	0.00	0.00
daf82d9d149245810577a23a2f6edb35	0.00	0.00	0.00	0.00	0.00	0.00
09462f7c035f29c4e8d80065f1046b2e	0.00	0.00	0.00	0.00	0.00	0.00
396d4e52986686878f090c34d57d0277	0.00	0.00	0.00	0.00	0.00	0.00

2e5714313fedcefe8d5711c4d3abaef5	0.00	0.00	0.00	0.00	0.00	0.00
3a931039af24a74567c403a4a8c6e197	0.00	0.00	0.00	0.00	0.00	0.00
b2e00478b39a414d1b07acdd6728c169	0.00	0.00	0.00	0.00	0.00	0.00
d4408009d15b84729f49893e40d6c47a	0.00	0.00	0.00	0.00	0.00	0.00
9ac56e3f3085545c72c446b8873af0c9	0.00	0.00	0.00	0.00	0.00	0.00
139a072b913e2c8dd4248c4115a77324	0.00	0.00	0.00	0.00	0.00	0.00
f074470521c2390a892ca6c9bb123577	0.00	0.00	0.00	0.00	0.00	0.00
2ee56ac19d6bc18954e6baebd2202684	0.00	0.00	0.00	0.00	0.00	0.00
6d5b61381210a18b77252c6e27c4be89	0.00	0.00	0.00	0.00	0.00	0.00
f999b1fa28dfa32a7c082eb2a5282d9a	0.00	0.00	0.00	0.00	0.00	0.00
8407b072e31bb49278c1e2b65bac2966	0.00	0.00	0.00	0.00	0.00	0.00
18cc68155c372fe28f99ec54cfee2357	0.00	0.00	0.00	0.00	0.00	0.00
da252502b9584b5209de33ab5503c66c	0.00	0.00	0.00	0.00	0.00	0.00
926456ff94bd8dd3623860386de92526	5.00	4.00	0.00	0.00	0.00	0.00
57a84111972fa0b9f3bc9773a4bb4380	0.00	0.00	0.00	0.00	0.00	0.00
fb6a894cb0b4427f95f8a888aa7830be	0.00	0.00	0.00	0.00	0.00	17.00
e81c34578094aa237bd9127bcc7217bd	0.00	0.00	0.00	0.00	0.00	0.00
2e8352da64f6314af9e17f1b500bad2f	0.00	0.00	0.00	0.00	0.00	0.00
05bdfc28e4a775dfaf60933c5336c5b1	0.00	0.00	0.00	0.00	0.00	0.00
ba30b285fb6e735d63bd8d7c28c45077	0.00	0.00	0.00	0.00	0.00	0.00
3c683b5dd07f8117f3f9324341d3c84e	0.00	0.00	0.00	0.00	0.00	0.00
1f81f9d2061057ecc701f09687b1afa2	0.00	0.00	0.00	0.00	0.00	0.00
3af5804c9c037a23603247b560d9e2d5	0.00	0.00	0.00	0.00	0.00	0.00
b2f79af98b0735432bd3c876b81ab8cb	0.00	0.00	0.00	0.00	0.00	0.00
8e8989386f0974d2c72b3b088b8fada5	0.00	0.00	0.00	0.00	0.00	0.00
54d5089091ce096ed599535d91cb50f7	0.00	0.00	0.00	0.00	0.00	0.00
59579ffa6e78e0f9db5e9e5dcff39d3f	0.00	0.00	0.00	0.00	0.00	0.00
98cedaeb6530038a7c03909a8fffac4d	0.00	0.00	0.00	0.00	0.00	0.00
b51cc2da6b9e829137a512e897e5ded3	0.00	0.00	0.00	0.00	0.00	0.00
ebac7f753d17f2638779534cd67758ca	0.00	0.00	0.00	0.00	0.00	0.00
2d98769676d82e2ba980c9ced8998d6b	0.00	0.00	0.00	0.00	0.00	0.00
7f8cab0ee83c3fe5e45679b389fcaf8d	0.00	0.00	0.00	0.00	0.00	0.00
59a07f752b0cd0569494e81f3c8099d2	0.00	0.00	0.00	0.00	0.00	0.00
4f015704faa4de201968672a2eaf54b4	0.00	0.00	0.00	0.00	0.00	0.00
45dcf6624605d10d5d77dfa9828aed94	0.00	0.00	0.00	0.00	0.00	0.00
a0dec7e4e119ebb36542bae064ed2b32	0.00	0.00	0.00	0.00	0.00	0.00
87201741e31efaaeefad9b4a6271c887	0.00	0.00	0.00	0.00	0.00	0.00

988771442aff6bfc123e698073c5c83b	0.00	0.00	0.00	0.00	0.00	0.00
0fe4ab78e70636f0337495f16383c146	0.00	0.00	0.00	0.00	0.00	0.00
bac75964180d6ecfc5ff330224e7386f	0.00	0.00	0.00	0.00	0.00	0.00
27424562dc7fec9471bc96c302a0f247	0.00	0.00	0.00	0.00	0.00	0.00
9f6b743196dec0a975b51fc0939be135	0.00	0.00	0.00	0.00	0.00	0.00
cfbd09d591c768806b5b8348cd24a2cb	0.00	0.00	0.00	0.00	0.00	0.00
e4a70c2b6a9724a88d302dca239582f7	0.00	0.00	0.00	0.00	0.00	0.00
c7d55d7c1d1891b2adb74a71040a3e46	0.00	0.00	0.00	0.00	0.00	0.00
5aefc0b0b021b0d975daab59babb2da9	0.00	0.00	0.00	0.00	0.00	0.00
d8294848e221b290b71ab4f35f00d53b	0.00	0.00	0.00	0.00	0.00	0.00
7fdc39f7ca25e2c4c5ba0651b99a2930	0.00	16.00	0.00	0.00	0.00	0.00
e47a5934b8268df12db21c1ad937bf70	0.00	0.00	0.00	0.00	0.00	0.00
e9a16f07e8d04500d165ffbf294505d7	0.00	0.00	16.00	0.00	0.00	0.00
7612acb9f0cc83ee6e3aa6306620493b	0.00	0.00	0.00	4.00	12.00	0.00
79a17e9a0112982ba1524fd854da1a11	0.00	0.00	0.00	0.00	0.00	16.00
41ace1e5b93047d86819bdd5cfeeedbe	0.00	0.00	0.00	0.00	0.00	0.00
5c3631fa3ea6630b187851b44920dba1	0.00	0.00	0.00	0.00	0.00	0.00
887c33afe1a2fdce1a72bc797481953c	0.00	0.00	0.00	0.00	0.00	0.00
676bca96663cf7634df642608a68255e	0.00	0.00	0.00	0.00	0.00	0.00
b7b8924f3ea452a34148120303263038	0.00	0.00	0.00	16.00	0.00	0.00
fa82430f38d69cfbf539bd89c2d08488	0.00	0.00	0.00	0.00	0.00	0.00
8fd8d9edadcfebeb6d365a512a6b7780	0.00	0.00	0.00	0.00	0.00	0.00
f31b51ad838c71c129d32714fb8962f1	0.00	0.00	0.00	0.00	0.00	0.00
813309858f982055f2b4eb6a5e818f57	0.00	0.00	0.00	0.00	0.00	0.00
1767e07f5ee77a7f9c8cfddf910ceff7	0.00	0.00	0.00	0.00	0.00	0.00
95e8678cd25a9c1b6d2f1ade5a66f4a8	0.00	0.00	0.00	0.00	0.00	0.00
ee595d4475e4eb0c50864be4dc94ce07	0.00	0.00	0.00	0.00	0.00	0.00
9a1a6c127188a5fa7f448ffa4c5209ab	0.00	0.00	0.00	0.00	0.00	0.00
5934afd21148d32eff29664fe8464c7c	0.00	0.00	0.00	0.00	0.00	0.00
4bbe4ec33dd4913537d678e1b716aa72	0.00	0.00	0.00	0.00	0.00	0.00
03ac181bf688f0927a75a75d6edeeb0c	0.00	0.00	0.00	0.00	0.00	0.00
d55dfd1d247d45da3a6d8fc46db06039	0.00	0.00	0.00	0.00	0.00	0.00
238c7f7d48b2e2334d67239ff78cd1fb	0.00	0.00	0.00	0.00	0.00	0.00
958e0287786c118a890182f719466126	0.00	0.00	0.00	0.00	0.00	0.00
8b4cf01bac14f2d6604e67f4f715a38b	0.00	0.00	0.00	0.00	0.00	0.00
04d1884fb0a14d1650ef0f46dc68ad88	0.00	0.00	0.00	0.00	0.00	0.00
13ead4efc43ef6b592a82c6f86928f94	0.00	0.00	0.00	0.00	0.00	0.00

eae20a84c11bc45bb978366a2b53a12b	0.00	0.00	0.00	0.00	0.00	0.00
a1b4defc70eaa6536f50e3d09eb72c5f	0.00	0.00	0.00	0.00	0.00	0.00
69f919928849b157a0495d621f78205f	0.00	0.00	0.00	0.00	0.00	0.00
4cc0976e47901a8bf18098cf113e1f2d	0.00	0.00	0.00	0.00	0.00	0.00
94598e8068462a14c9b1de8437feec22	0.00	0.00	0.00	0.00	0.00	0.00
ac88e30045322cef933e94a130c4bb54	0.00	0.00	0.00	0.00	0.00	0.00
4b01aaf57b7206baf136b2ae78dc9a64	0.00	0.00	6.00	6.00	0.00	0.00
ef735cbd4fa4b8ec9261638e625abcc8	0.00	0.00	0.00	0.00	6.00	0.00
144c97040fec812b334b123d6298991f	0.00	0.00	0.00	0.00	15.00	0.00
b1bc63d1b4bb8bcfb0660fa4d3026021	0.00	0.00	0.00	0.00	0.00	5.00
305570165ffec3279825d82aac88d0b7	0.00	0.00	0.00	0.00	0.00	0.00
2bc0a0d8259ae62cd4d6a3e6328644b4	0.00	0.00	0.00	0.00	0.00	0.00
362613301e67a9e3681194d22ef4bccb	0.00	0.00	0.00	3.00	0.00	0.00
2f7b935f347144ce7ac3bf2f2172621d	0.00	0.00	0.00	15.00	0.00	0.00
7ad524f4ccacf47d2c9ab920b5015a97	0.00	0.00	0.00	15.00	0.00	0.00
43aed8f2738c3f7a1c358182e2ac101e	0.00	0.00	0.00	15.00	0.00	0.00
57bede056dee96d0d6b08bdcb7e6497c	0.00	0.00	0.00	15.00	0.00	0.00
9398a4be30775f5638936cb4ad92e472	0.00	0.00	0.00	15.00	0.00	0.00
b88168539a30a16f2a01b2ca24b912a4	0.00	0.00	0.00	15.00	0.00	0.00
350de39a770735b180cf080e7ffe7281	0.00	0.00	0.00	0.00	0.00	0.00
3c8cd344136ca2bd7d27016008bcb969	0.00	0.00	0.00	0.00	0.00	0.00
53ffa9398ea985fe962f6390b4e58228	0.00	0.00	0.00	0.00	0.00	0.00
6b5f69cef4000adaf5623aea3cdbe72b	0.00	0.00	0.00	0.00	0.00	0.00
15c7ae4d50f917e27cfd537941423cd3	0.00	0.00	0.00	0.00	0.00	0.00
0a6c32c9aac6338d9d6dfc5c58d43183	0.00	0.00	0.00	0.00	0.00	0.00
fd88fc091688973c50015642a25f36f5	0.00	0.00	0.00	0.00	0.00	0.00
432f8fcf5ba79a2fe41aa8beb2976dab	0.00	0.00	0.00	0.00	0.00	0.00
fa86c035922a87f3d30d614be9c84e09	0.00	0.00	0.00	0.00	0.00	0.00
3dd0c5af8219fe26e7e482c091c53bb7	0.00	0.00	0.00	0.00	0.00	0.00
e56da2523f817b5c9c223d427f97b04c	0.00	0.00	0.00	0.00	0.00	0.00
33454eb29b6d2fd484b9d232fba58f87	0.00	6.00	0.00	0.00	0.00	0.00
a5283d000834a3a312a7d4743fd0b4ef	0.00	0.00	0.00	0.00	0.00	0.00
6a7ceb6570b459186bf3b178cee2ed3f	0.00	0.00	0.00	0.00	0.00	0.00
860ee0d286b3370fadc849d26693709b	0.00	0.00	0.00	0.00	0.00	0.00
054c8e081fdcf0efa8f7c783272a2d0d	0.00	0.00	0.00	0.00	0.00	14.00
57428e92349d0ab586e88aa6e6f44cb2	0.00	0.00	0.00	0.00	0.00	8.00
99b411a53ccea764f8f4c1686fc2dc83	0.00	0.00	0.00	0.00	0.00	0.00

fdf92b772d69bd1dee6a1c5b531d8b59	0.00	0.00	0.00	0.00	0.00	0.00
624818589efe4aa06ae20f08226cc028	0.00	0.00	0.00	0.00	0.00	0.00
ef7947bab18427ac8c6eda06bf3f0eb5	0.00	0.00	0.00	14.00	0.00	0.00
ed342d3996366a54651539af9c81187c	0.00	0.00	0.00	14.00	0.00	0.00
8135eff65019e995709c7af01942deab	0.00	0.00	0.00	0.00	0.00	0.00
1364dfce3796b749e877e0aa06ac8206	0.00	0.00	0.00	0.00	0.00	0.00
17562bd59c022647b3faeef9184d0a20	0.00	0.00	0.00	0.00	0.00	0.00
52fe6a793774c80794a950df0cfc46ca	0.00	0.00	0.00	0.00	0.00	0.00
e1ad0dee6820a4c1e8dbc600efb3819f	0.00	0.00	0.00	0.00	0.00	0.00
cc5729acb43fb7ba64729eb69e5f9387	0.00	0.00	0.00	0.00	0.00	0.00
58633a269a7a05a85b71b2baa5369961	0.00	0.00	0.00	0.00	0.00	0.00
c30c6469c54c1511650bb867bb822989	0.00	0.00	0.00	0.00	0.00	0.00
1e7a8abcf6fbd0b6cd2fb73ff5af0989	0.00	0.00	0.00	0.00	0.00	0.00
48245be49a0f2481abb4b679b8a95d2e	0.00	0.00	0.00	0.00	0.00	0.00
bbd6d052e2dcf1a296d1e7bf182e0f7c	0.00	0.00	0.00	0.00	0.00	0.00
3d5482d0ee6cc5c812b9cc111688f467	0.00	0.00	0.00	0.00	0.00	0.00
3105a7cca2e029c29f652342b678313d	0.00	0.00	0.00	0.00	0.00	0.00
dbdede58fef92d2cd0a7711aa9622e92	0.00	0.00	0.00	0.00	0.00	0.00
b086e99b3d23e8a0c31575d6c16ecbbd	0.00	0.00	0.00	0.00	0.00	0.00
0afa3d1196f253e04f5ac7056c793dbd	0.00	0.00	0.00	0.00	0.00	0.00
c77914fb8fa590cb182fa190f5f76973	0.00	0.00	0.00	0.00	0.00	0.00
e6b6a607d2c270db8493e069ce28ff2c	0.00	0.00	13.00	0.00	0.00	0.00
b2ad15b91add50f689142d06df109642	0.00	0.00	0.00	0.00	0.00	6.00
b3b8fece93cb2029d4669013ada23518	0.00	0.00	0.00	0.00	0.00	0.00
f9a0a021ac414bde40c3d310e280bf5e	0.00	0.00	0.00	0.00	0.00	0.00
4fad99dbdef7ad09d81e9c4956bc2eb4	0.00	0.00	0.00	0.00	0.00	0.00
4160756d2542e964f65ad9fd17d23502	0.00	0.00	0.00	13.00	0.00	0.00
34bd6ea451346af9a50fda2f6f1123a3	0.00	0.00	0.00	11.00	0.00	0.00
3b697020d61c070ef86008df30bae33c	0.00	0.00	0.00	13.00	0.00	0.00
1f01f1de0f275ed1ebe4ce615cf6d41a	0.00	0.00	0.00	13.00	0.00	0.00
09b644d915c6cb5c8e8f7c42fa553bdb	0.00	0.00	0.00	13.00	0.00	0.00
ff32767d7e7fdd82d579b01e5ad08e2f	0.00	0.00	0.00	13.00	0.00	0.00
91e1d22b5531edd0fd5d1a2badc8ff80	0.00	0.00	0.00	0.00	0.00	0.00
0e3256cf30a218160e5eb9c1026b729c	0.00	0.00	0.00	0.00	0.00	0.00
6d2ac891b964a84bb554a2560e99be5c	0.00	0.00	0.00	0.00	0.00	0.00
6e2714cd551d9003501f665de7a55aaa	0.00	0.00	0.00	0.00	0.00	0.00
f0db98390310c32327a687cd488c78a3	0.00	0.00	0.00	0.00	0.00	0.00

b390b101ac44e17ddd226074941c29d3	0.00	0.00	0.00	0.00	0.00	0.00
daad7c7e2954c5f781d39c470020f3fd	0.00	0.00	0.00	0.00	0.00	0.00
58777c2ae77edc9f85d00eaefc150c64	0.00	0.00	0.00	0.00	0.00	0.00
cee25e6a8da2c1dc599e73698085f395	0.00	0.00	0.00	0.00	0.00	0.00
c8e01ba78f901a575c0c59a7796839d2	0.00	0.00	0.00	0.00	0.00	0.00
8682f68f3ecbef11caea9fa17adc9125	0.00	0.00	0.00	0.00	0.00	0.00
afe2d989b3f694c6b289da3032dcf190	0.00	0.00	0.00	0.00	0.00	0.00
bc166be5a62b7bb3c4701ed3e46fff01	0.00	0.00	0.00	0.00	0.00	0.00
751ceb3e57e77614f886d2c02228777f	0.00	0.00	0.00	0.00	0.00	0.00
ef213128514c6acd147e2d182031965b	0.00	0.00	0.00	0.00	0.00	0.00
43e708375c9a8a204842f87f27b6d4cd	0.00	0.00	0.00	0.00	0.00	0.00
4326630c9d4d1370700c3162c5e604b8	0.00	0.00	0.00	0.00	0.00	0.00
3fd022715ec136c3c582763d38d69bfc	0.00	0.00	0.00	0.00	0.00	0.00
27d2eaa2d4ce10c2fd73648113efabb1	0.00	0.00	0.00	0.00	0.00	0.00
0be4ceda2ea9112bd3b034cc5c3679c8	0.00	0.00	0.00	0.00	0.00	0.00
9a9ed2eb287ccb711f67f2dcef4ccde1	0.00	6.00	0.00	0.00	0.00	0.00
d6de9f30414651d86b3766f9b24e3e22	0.00	12.00	0.00	0.00	0.00	0.00
eca360c64e0087dfb2869c5fdc62ee3a	0.00	0.00	0.00	0.00	0.00	0.00
446a382c90413c34293d169fa9bd91c7	0.00	0.00	0.00	0.00	0.00	0.00
42e021e6f452f3dd3ca9b4adcb8f05b2	0.00	0.00	0.00	0.00	0.00	0.00
504cc28a2586725789e4d0e2a56323e0	0.00	0.00	0.00	0.00	0.00	0.00
049a4fe41fb80c8ca93502400c85dc55	0.00	0.00	0.00	0.00	0.00	0.00
ef9b184ec490c8d88d6932d6f6729973	0.00	0.00	0.00	0.00	0.00	0.00
8b1b44ce298d1096a92f1cd560e00d88	0.00	0.00	5.00	0.00	2.00	0.00
748ffecaa7fcfb2afc24f8fa3e7aeab3	5.00	0.00	3.00	0.00	0.00	0.00
e71424cfe651c1c108dd6ee019f7d279	0.00	0.00	0.00	0.00	12.00	0.00
d4beacd43d7fa18c27b24b4af477b135	0.00	0.00	0.00	0.00	0.00	0.00
71da0681535ca07058a4466ce59fed89	0.00	0.00	0.00	0.00	0.00	0.00
f3833b39e1782fb860635d775741f23a	12.00	0.00	0.00	0.00	0.00	0.00
394116fefb6d2825c7699b6c688a6483	12.00	0.00	0.00	0.00	0.00	0.00
a286c2114d5e7015bc1b4e2800d7948d	0.00	0.00	0.00	0.00	0.00	0.00
5265217613ed9c237a7736e4b80dfdb1	0.00	0.00	0.00	0.00	0.00	0.00
d7b30508190defbe893f5d4a55402849	0.00	0.00	0.00	0.00	0.00	0.00
656e4715b191a48551b1c4b9cdc9acd7	0.00	0.00	0.00	0.00	0.00	0.00
07f73b12ca4c16b1638a32a1ca2a8313	0.00	0.00	0.00	12.00	0.00	0.00
3b2980683edf693d4fe84f36442fbcc3	0.00	0.00	0.00	12.00	0.00	0.00
5337fb4f96b129e5d63d4129fdebb13c	0.00	0.00	0.00	12.00	0.00	0.00

b225d78f3606aaea3222a90099d7b744	0.00	0.00	0.00	0.00	0.00	0.00
975f295430b3dd8c9ea1226b75a00989	0.00	0.00	0.00	0.00	0.00	0.00
90e60ebd23082f141045ccb3ad73079f	0.00	0.00	0.00	0.00	0.00	0.00
3293a131c99853ac0f5b7cc5e8cb3327	0.00	0.00	0.00	0.00	0.00	0.00
12642e6aaa8986e8500a302e87b3aa0f	0.00	0.00	0.00	0.00	0.00	0.00
84844541ba3be9fc8a2adf536d656ded	0.00	0.00	0.00	0.00	0.00	0.00
5a1031a24764e0f2d87aa044b7bc9dce	0.00	0.00	0.00	0.00	0.00	0.00
276fc7b6883033c3cc76638aa8f5ed5c	0.00	0.00	0.00	0.00	0.00	0.00
80580e5ea02d298713912ab6a7586252	0.00	0.00	0.00	0.00	0.00	0.00
8dc254619ecdb83185ff6fde2484974f	0.00	0.00	0.00	0.00	0.00	0.00
dad817972c4293da30c6baeccb80334b	0.00	0.00	0.00	0.00	0.00	0.00
2536bdc1b1de5c9e916b1f913bd4231a	0.00	0.00	0.00	0.00	0.00	0.00
66c4212afbea18ed6c91448beba1e994	0.00	0.00	0.00	0.00	0.00	0.00
bbae1dd5fc01aa026653c0bdc21d154f	0.00	0.00	0.00	0.00	0.00	0.00
86a140234a0e8afb8eb40abfa9684f6f	0.00	0.00	0.00	0.00	0.00	0.00
c5cbcfe2012b851904ebaf177e715b54	0.00	0.00	0.00	0.00	0.00	0.00
851816d31f54c2ffab64993d217d364c	0.00	0.00	0.00	0.00	0.00	0.00
8fccc47ac1a25aabc2ec4a081fe64503	0.00	0.00	0.00	0.00	0.00	0.00
e7a68d8a956630234b6bf566be8c6c8d	0.00	0.00	0.00	0.00	0.00	0.00
8605e5dbb5037c16d59580d722cb87fc	0.00	0.00	0.00	0.00	0.00	0.00
e10f15eb4f6d116b6273d41a797d7588	0.00	0.00	0.00	0.00	0.00	0.00
689030063c8edfb8240b5d1788bafcda	0.00	0.00	0.00	0.00	0.00	0.00
f68d9b7a447905f340b84e1f3328bbd4	0.00	0.00	0.00	0.00	0.00	0.00
fbe538d45e691a518fce958a189cbba4	0.00	0.00	0.00	0.00	0.00	0.00
715f0c80a2ce6bd6108fe6a79aeb0bfa	0.00	0.00	0.00	0.00	0.00	0.00
65aef04571d954f2c53101ba6a84a363	0.00	0.00	0.00	0.00	0.00	0.00
3cf0bf7d0b5214cfb23a02c67d115953	0.00	0.00	0.00	0.00	0.00	0.00
fa84b43d750a4b1849c25fe4b31c8aa2	0.00	0.00	0.00	0.00	0.00	0.00
62815c07f0b5b73ec8b59ce0b1bdfbec	0.00	0.00	0.00	0.00	0.00	0.00
c106e3aafd6435e93463fd68caade41b	0.00	0.00	0.00	0.00	3.00	0.00
e1fa413b1adbebeeb53519e0746db973	6.00	0.00	0.00	0.00	5.00	0.00
04a804ecf8392f0d725d04f6ac1d9d04	0.00	0.00	0.00	0.00	11.00	0.00
a439b79c3b773ab378d27d240d2db5c5	0.00	0.00	0.00	0.00	0.00	11.00
fd4262fa0b2d2b1d52af1638feb30745	0.00	0.00	0.00	0.00	0.00	0.00
fabb8e0032e3cfec402f98f809fa1d0c	0.00	0.00	0.00	11.00	0.00	0.00
5040b2a2e66ba01e8b06698518b025d8	0.00	0.00	0.00	11.00	0.00	0.00
7c51a04f01faecd74aae7ae82b574598	0.00	0.00	0.00	11.00	0.00	0.00

34772aca2c6abcc1715c385c81902ce1	0.00	0.00	0.00	0.00	0.00	0.00
8d2f9a3d2a2ffa0cfa322358145f651a	0.00	0.00	0.00	0.00	0.00	0.00
603dd6b8f7878f7d4d41d16f6618124f	0.00	0.00	0.00	0.00	0.00	0.00
ecad007f1796bf71e44f14c1f6c3ad41	0.00	0.00	0.00	0.00	0.00	0.00
c8857b153c134d796be7443bf64392c2	0.00	0.00	0.00	0.00	0.00	0.00
896973a81350fc9d9e0e4541a60c65f0	0.00	0.00	0.00	0.00	0.00	0.00
217846f9898c36b2bf6c2effe1c0c837	0.00	0.00	0.00	0.00	0.00	0.00
d2652255a1de796a7dc2261a9af574cd	0.00	0.00	0.00	0.00	0.00	0.00
952382061798f4d507ae18387d4804f5	0.00	0.00	0.00	0.00	0.00	0.00
45b573fa85067eb8be30fe24543740a5	0.00	0.00	0.00	0.00	0.00	0.00
8d6e4a28290ef46a667b2513bcab3437	0.00	0.00	0.00	0.00	0.00	0.00
ca25eaafd35515e84db7e41d08aebc03	0.00	0.00	0.00	0.00	0.00	0.00
8784c31264b3c490c5f8c8bf0840584a	0.00	0.00	0.00	0.00	0.00	0.00
b11605197f704e5bdc6e3ef165d8d4fa	0.00	0.00	0.00	0.00	0.00	0.00
9a67238fd5164cb1f5c8ca9712a27cfd	0.00	0.00	0.00	0.00	0.00	0.00
00ca0b4d4a9ba4946e8ae227c7450dd8	0.00	0.00	0.00	0.00	0.00	0.00
98e0282e1379510a794ef813ada880bc	0.00	0.00	0.00	0.00	0.00	0.00
14c8018027f6ac92347184db3eb7db92	0.00	0.00	0.00	0.00	0.00	0.00
b075b0179f282c7fb16566ed7136ef1f	0.00	0.00	0.00	0.00	0.00	0.00
6b1647f809b80be62d738d10e3df83e3	0.00	0.00	0.00	0.00	0.00	0.00
28b038f2d54001d6f0f88cbfb88150eb	0.00	0.00	0.00	0.00	0.00	0.00
e767e3bc52300a5ef99367c5abbc79b4	0.00	0.00	0.00	0.00	0.00	0.00
e93766918e4221dad48c69fc21ff48f6	0.00	0.00	0.00	0.00	0.00	0.00
3475fcb58ea78a75282d0080b900b15f	0.00	0.00	0.00	0.00	0.00	0.00
755b9e35cd85b0460e934e68a79a7bc0	0.00	0.00	0.00	0.00	0.00	0.00
cd99c28649250b27ac159846f513aaa9	0.00	0.00	0.00	0.00	0.00	0.00
295c33d03ee409d168e64b2e2c1e6d09	0.00	0.00	0.00	0.00	0.00	0.00
a0c22e8f01c9ace0dd5ff1a88f090d0a	0.00	10.00	0.00	0.00	0.00	0.00
e35d6e0559e83c6cac8033e798ef9af5	0.00	10.00	0.00	0.00	0.00	0.00
30c128578ca993ef333a7caca3b491ef	0.00	0.00	0.00	0.00	0.00	0.00
829118df97673445051b63e470f2d18a	0.00	0.00	0.00	0.00	0.00	0.00
b9cd13838a665f0edb5d3ec1d353988e	0.00	0.00	0.00	0.00	0.00	0.00
9df0d0ba69898da3764c709d92d4d2c9	0.00	0.00	0.00	0.00	0.00	0.00
ad752f04842cf5f4368fbc4ce35eaaa4	0.00	0.00	0.00	0.00	0.00	0.00
19e968c7972fe0da5fee6dbc65c3fb0a	0.00	0.00	0.00	0.00	10.00	0.00
97485773e8c6866fc5fb37e1b51b6bf3	0.00	0.00	0.00	0.00	10.00	0.00
4dc38524660c60eccc1c4128e52fd66c	0.00	0.00	0.00	0.00	0.00	5.00

e7ac2d0dd90b000dc7b8090f700eb641	0.00	0.00	0.00	0.00	0.00	0.00
691f76ae13a4b86daf69ade05006bbbc	0.00	0.00	0.00	0.00	0.00	0.00
dfe15dd6d2cd680cf2c20cfcb53337d2	0.00	0.00	0.00	0.00	0.00	0.00
88303b340630e0c0929c9c4a149b88a4	0.00	0.00	0.00	0.00	0.00	0.00
9794614b8d1a145a1883973c80981bb8	0.00	0.00	0.00	10.00	0.00	0.00
4c4f42ed64b10c9e098b94629d48ffdb	0.00	0.00	0.00	10.00	0.00	0.00
75705cad2a82832413efb70bf09f0a9e	0.00	0.00	0.00	10.00	0.00	0.00
612527979ad219bcbe60c0e5680144e4	0.00	0.00	0.00	10.00	0.00	0.00
d3080708834bca193669dbd1c6b7ac97	0.00	0.00	0.00	10.00	0.00	0.00
142513f65b67e89b8ef089f367b40f07	0.00	0.00	0.00	10.00	0.00	0.00
5fe5c2df9563addebedd2e142a4bac91	0.00	0.00	0.00	0.00	0.00	0.00
6ee1717739bd04c8b428630cf8945503	0.00	0.00	0.00	0.00	0.00	0.00
56e602326ae31a7831e2740bf32e262c	0.00	0.00	0.00	0.00	0.00	0.00
52a95b09f3dbf479efb31d2d7829c3cf	0.00	0.00	0.00	0.00	0.00	0.00
3c5213a221fa0a679e39bd8785477fd8	0.00	0.00	0.00	0.00	0.00	0.00
17d962561e0d63a89b503df75a55a75f	0.00	0.00	0.00	0.00	0.00	0.00
56632573f65310ac3b9af9f0a77d910e	0.00	0.00	0.00	0.00	0.00	0.00
ff639bdcfe072a3f1ef5bf6cd35f55f6	0.00	0.00	0.00	0.00	0.00	0.00
6bcfd9b2ccebb437770076623d3251d8	0.00	0.00	0.00	0.00	0.00	0.00
ad835ede648e6bfaee0008826b261083	0.00	0.00	0.00	0.00	0.00	0.00
98b288bb5d09704d71c66935371de63f	0.00	0.00	0.00	0.00	0.00	0.00
7e291e81db35e489da78b5c64cae198e	0.00	0.00	0.00	0.00	0.00	0.00
363304bd5171afdb0d0fccf96a075781	0.00	0.00	0.00	0.00	0.00	0.00
87253b9f499e853eac66f40f6cec4a01	0.00	0.00	0.00	0.00	0.00	0.00
4a8471ec1fe41c6f6cc3ece24349d02e	0.00	0.00	0.00	0.00	0.00	0.00
6ee991579e410a2352a6ffe2dd33a55f	0.00	0.00	0.00	0.00	0.00	0.00
64841daa963917fe2c4cdd900679a5ff	0.00	0.00	0.00	0.00	0.00	0.00
89c91e4eb013da95dd55ac30e1d5e6a2	0.00	0.00	0.00	0.00	0.00	0.00
07e3071d772df3c477d1ecbf73c89cb6	0.00	0.00	0.00	0.00	0.00	0.00
480909b7c0396a15206a5e72286e235d	0.00	0.00	0.00	0.00	0.00	0.00
ef1d86fb3c14f5c9a04ad76e78e70f3f	0.00	0.00	0.00	0.00	0.00	0.00
b0039d581865bd3a72de4db54dd046db	0.00	0.00	0.00	0.00	0.00	0.00
11ab96dadb908c3fd1cf2b6a9b948887	0.00	0.00	0.00	0.00	0.00	0.00
f2e11f6a0c249114402a3eb8850adc91	0.00	0.00	0.00	0.00	0.00	0.00
73e095a0aa2e0d3999e931a9686723bc	0.00	0.00	0.00	0.00	0.00	0.00
5c832b95d05e992da26c8b6bcb34a845	0.00	0.00	0.00	0.00	0.00	0.00
8f31d855482d5c406fd255c5a5b5d02c	0.00	0.00	0.00	0.00	0.00	0.00

acccb4d20d270875f82212a83d223acb	0.00	0.00	0.00	0.00	0.00	0.00
4ab21eff7848e1062fdd3afc7a4d67ff	0.00	0.00	0.00	0.00	0.00	0.00
24ed632251478148772634e7001ad0bc	0.00	0.00	0.00	0.00	0.00	0.00
88318cb52dc53178963fc854363f7cc2	0.00	0.00	0.00	0.00	0.00	0.00
0b0b3de3b7ed8d6dd1ba8442647ae154	0.00	0.00	0.00	0.00	0.00	0.00
cad7c950af09c03fa7c557decc6ec198	0.00	0.00	0.00	0.00	0.00	0.00
33de2741f3990f51a24255ab6b9d4a70	0.00	0.00	0.00	0.00	0.00	0.00
a2e0436fe481d127af67e35e67e9d1e6	0.00	0.00	0.00	0.00	0.00	0.00
beefec8525bfefee8be315bbb04c38e1	0.00	0.00	0.00	0.00	0.00	0.00
2cb75fc29330e3c2814e576cf8b2db50	0.00	0.00	0.00	2.00	0.00	0.00
0ba2f562e6e334861f89a452dccb7899	0.00	0.00	0.00	0.00	0.00	0.00
86bbb08187b7301e4d48d4dd90b8384b	0.00	0.00	9.00	0.00	0.00	0.00
bcebd1941769f9ec6cd3da5f4e393f47	0.00	0.00	0.00	0.00	9.00	0.00
b9bf0aefc28a55ce28697a9d38efa58c	0.00	0.00	0.00	0.00	9.00	0.00
74b833939178fd67539f477451853600	0.00	0.00	0.00	0.00	0.00	3.00
ebee50399ca39594f971feed4ce3cd79	0.00	0.00	0.00	0.00	0.00	9.00
2f4aa40c4c4ac0d7e0786190b4400f1a	0.00	0.00	0.00	0.00	0.00	0.00
575c928a468bfbb4a26c158a06b28f20	0.00	0.00	0.00	0.00	0.00	0.00
b6141922f96ba9511d855e738984d917	0.00	0.00	0.00	0.00	0.00	0.00
121417cecb89ef4f07d57ac3b02b29c9	0.00	0.00	0.00	0.00	0.00	0.00
a57951bd51ce7e1068a8069350681aa2	0.00	0.00	0.00	0.00	0.00	0.00
1358e300fe6385f7317cc48520a05dde	9.00	0.00	0.00	0.00	0.00	0.00
012d63c3be78cd7d0acff29372ba632c	9.00	0.00	0.00	0.00	0.00	0.00
d54b41d8e36f6ff6400c1c1f5f7dd5f1	0.00	0.00	0.00	0.00	0.00	0.00
16768d3604fc2533104a5a832ff22d38	0.00	0.00	0.00	0.00	0.00	0.00
cbcb2c3da35cd04771bd198c552497b0	0.00	0.00	0.00	9.00	0.00	0.00
4a65d8a13eca87112c1c214a24639030	0.00	0.00	0.00	9.00	0.00	0.00
ca614d20bd1635ffbb94ec8745358732	0.00	0.00	0.00	9.00	0.00	0.00
bf37db00d7643c18059b549cdd74b00e	0.00	0.00	0.00	9.00	0.00	0.00
f1f4a64d56d788fd73313101ea81cd9b	0.00	0.00	0.00	4.00	0.00	0.00
534ae050bbeaa9fd8e645050ba1bc2a4	0.00	0.00	0.00	0.00	0.00	0.00
cb1d2333bf4c16002db2d1a486532959	0.00	0.00	0.00	0.00	0.00	0.00
8674aa3d8b8cc0c59cf3ce14d81f061c	0.00	0.00	0.00	0.00	0.00	0.00
28be6b5f269720b4be9c20ae687d1192	0.00	0.00	0.00	0.00	0.00	0.00
5ed3c7539051c635846b6b46e0536635	0.00	0.00	0.00	0.00	0.00	0.00
c656d7b5dab38660ca5792bae9835cd9	0.00	0.00	0.00	0.00	0.00	0.00
446cad7eaeb8bbf49e91127579e13f0a	0.00	0.00	0.00	0.00	0.00	0.00

b467b11b4f4db9fee2639c58037af918	0.00	0.00	0.00	0.00	0.00	0.00
5e2f801c6bdd4ee934e6ee7234729007	0.00	0.00	0.00	0.00	0.00	0.00
71a198d98d71287211247554fcbd9f25	0.00	0.00	0.00	0.00	0.00	0.00
dcdaef119c8959a851af9101b61a9bf4	0.00	0.00	0.00	0.00	0.00	0.00
f14171d2ed7fc65119b6ab358fb009be	0.00	0.00	0.00	0.00	0.00	0.00
d9f106b54ad46e4f14a0bbd86a853bea	0.00	0.00	0.00	0.00	0.00	0.00
92331a5735587b72d603e49795490cb2	0.00	0.00	0.00	0.00	0.00	0.00
b6fa047d3d396db4abcf1d0672e1c2a2	0.00	0.00	0.00	0.00	0.00	0.00
1168f47307ec2ffde4c58621a781f86f	0.00	0.00	0.00	0.00	0.00	0.00
afc34a5d63b5907162aafaadf8222738	0.00	0.00	0.00	0.00	0.00	0.00
a83f48ca31e1ba99f71263a99aea792e	0.00	0.00	0.00	0.00	0.00	0.00
cbd13a8722959b7b2a27fde6dcc1fa67	0.00	0.00	0.00	0.00	0.00	0.00
4c883295f98f67aff3d216307554e781	0.00	0.00	0.00	0.00	0.00	0.00
4fda08d792054b6c5c0eedf34350368a	0.00	0.00	0.00	0.00	0.00	0.00
c2050f57c4f01949ad80d653d73275f5	0.00	0.00	0.00	0.00	0.00	0.00
1ffc3884b4df1422f95f8a4c53e2f9c1	0.00	0.00	0.00	0.00	0.00	0.00
f17e07d4088b2c0ed991f6b1da9879a4	0.00	0.00	0.00	0.00	0.00	0.00
c4627979828b77d68572279b5fedb901	0.00	0.00	0.00	0.00	0.00	0.00
11aa8a2a8d4a703e5abebc9ddc56a6be	0.00	0.00	0.00	0.00	0.00	0.00
31863fb3ae4ab1d4d2dfba654d6cb9eb	0.00	0.00	0.00	0.00	0.00	0.00
27e1b3bb529108621d625641e1651aac	0.00	0.00	0.00	0.00	0.00	0.00
8654e9c9984919c217aadf662d2f62c4	0.00	0.00	0.00	0.00	0.00	0.00
15cdfebe36e7426d3ae40b41833049be	0.00	0.00	0.00	0.00	0.00	0.00
fc1c58ce0f140b08763af0661f743042	0.00	0.00	0.00	0.00	0.00	0.00
63c72391469c9c2dc7f8752b240f5a96	0.00	0.00	0.00	0.00	0.00	0.00
4eced0d3565bd92985339d0a14862280	0.00	0.00	0.00	0.00	0.00	0.00
52ced752925528292c90abbe29a38d1c	0.00	0.00	0.00	0.00	0.00	0.00
55141c9f2f454d04a8dfbc97dade920e	0.00	0.00	0.00	0.00	0.00	0.00
618f2fc84b9d11c47d5b04e2944bcea7	0.00	0.00	0.00	0.00	0.00	0.00
3da1c78fda19070624f8065d97947505	0.00	2.00	0.00	0.00	0.00	0.00
7e86b61daceaa47c89b841b7384e6176	0.00	0.00	0.00	0.00	0.00	0.00
1e86f1f65a4f002b27e0d77c95d43942	0.00	0.00	0.00	0.00	0.00	0.00
538db69b652e7b9c2779115e38b135a8	0.00	0.00	0.00	0.00	0.00	0.00
31ba9aca418d9e2a55f280fc1cd74805	0.00	0.00	0.00	0.00	0.00	0.00
4f09dca9e3d14756a4b353afb238aa51	0.00	0.00	0.00	0.00	0.00	0.00
5d7fdcb9b3889e60bd6e71979cac023f	0.00	0.00	0.00	0.00	0.00	0.00
e9879d6f03d971e286a95cd1f79951e2	0.00	0.00	0.00	0.00	0.00	0.00

aa7de122beceaad7e77f8dbb273b1c5d	0.00	0.00	0.00	0.00	0.00	0.00
86ad43e094808d8f3fc2a639b9fc78f6	0.00	0.00	8.00	0.00	0.00	0.00
92f26f39aed46aa467d2d2f1c83f1934	0.00	0.00	0.00	0.00	3.00	0.00
ab3d4a9f2815c76e41f26ff6f57648bb	0.00	0.00	0.00	2.00	0.00	2.00
8534d3c7a3095a542aa2731fd8c62f62	0.00	0.00	0.00	0.00	0.00	8.00
9d23f9400412f7909eed44a3f6a230b1	0.00	0.00	0.00	0.00	0.00	0.00
d36286210314df547be0ff7945d84c84	0.00	0.00	0.00	0.00	0.00	0.00
8c4076912de234d1e021b226224e16f7	0.00	0.00	0.00	0.00	0.00	0.00
d6318717cc071ea15aa832ba13a082e4	0.00	0.00	0.00	8.00	0.00	0.00
681218e8ca74e0fb557a2f9bb4120834	0.00	0.00	0.00	8.00	0.00	0.00
5556388c13ce3c501de4589d7779702c	0.00	0.00	0.00	8.00	0.00	0.00
db55f88068612dc64d6efdb6f648cf80	0.00	0.00	0.00	8.00	0.00	0.00
292160339c638eeade4df896b7d3845a	0.00	0.00	0.00	8.00	0.00	0.00
ca32e7fc944cddc84cb1de2869218635	0.00	0.00	0.00	0.00	0.00	0.00
9e385c7474f0f3ac7f2ecfc28d057e38	0.00	0.00	0.00	0.00	0.00	0.00
fe4e2deae37b20114c6d833dbff2f853	0.00	0.00	0.00	0.00	0.00	0.00
dcdc752935f32dbcd40eb31581603006	0.00	0.00	0.00	0.00	0.00	0.00
270a6d6c898cb82931034c6871829262	0.00	0.00	0.00	0.00	0.00	0.00
9b1e51ff176556985a98b99e511d9a73	0.00	0.00	0.00	0.00	0.00	0.00
0930689c6d16583a90e28a5a05320ac0	0.00	0.00	0.00	0.00	0.00	0.00
b2809f1e496903d573cfa1f2a0f1c487	0.00	0.00	0.00	0.00	0.00	0.00
552d2bd102822152de09103a7a8addd7	0.00	0.00	0.00	0.00	0.00	0.00
f9011de4c63cdfa8a8feeb243dff708b	0.00	0.00	0.00	0.00	0.00	0.00
16a7e0c7497a3e7ca8c137313618f5dc	0.00	0.00	0.00	0.00	0.00	0.00
26272d708f014f96d0512781f9413b35	0.00	0.00	0.00	0.00	0.00	0.00
67d29fade00c49b504a4a2e7e39aa6c5	0.00	0.00	0.00	0.00	0.00	0.00
1b84483d0ee155c67514fbebe62fa696	0.00	0.00	0.00	0.00	0.00	0.00
fd958e1f9d4b2310ea2a9080a712333e	0.00	0.00	0.00	0.00	0.00	0.00
a066b31b960631208add28e30313bb97	0.00	0.00	0.00	0.00	0.00	0.00
0bfc23948833b6da22f6eed5716c57d1	0.00	0.00	0.00	0.00	0.00	0.00
15fa43ec458347f01c31b49bd15d47b0	0.00	0.00	0.00	0.00	0.00	0.00
f1dee099b887c9cca23fcf848dc7dde2	0.00	0.00	0.00	0.00	0.00	0.00
b64633032675b9a0178a44ac80c1bdc2	0.00	0.00	0.00	0.00	0.00	0.00
5229880d69fbf3c5243806c9b65ed780	0.00	0.00	0.00	0.00	0.00	0.00
cc3f30c61caf29d782c1751abc5f1c36	0.00	0.00	0.00	0.00	0.00	0.00
5a1c93b65325f3f78313cd643ff238fa	0.00	0.00	0.00	0.00	0.00	0.00
3d2948495d73fa9368f26cc50869ea20	0.00	0.00	0.00	0.00	0.00	0.00

1fd33b261feadf912f35bfc8927d36de	3.00	4.00	0.00	0.00	0.00	0.00
407150b9decca8bf9c3a0cbbb7bcbe89	0.00	0.00	0.00	0.00	0.00	0.00
37025fea1ec58b4870d86093393200a2	0.00	0.00	0.00	0.00	0.00	0.00
02302b9befec9a0ea3da41f9eff0d638	0.00	0.00	0.00	0.00	0.00	0.00
8b003a9fb0258ebffdec8dbcfd40f151	0.00	0.00	0.00	0.00	0.00	0.00
a0b5ebb22b806dfd71e77f35a03d3a27	0.00	0.00	0.00	0.00	0.00	0.00
c7751b785d964f3a8aeb4b5cefb7fbae	0.00	0.00	0.00	0.00	0.00	0.00
4c99a4069250b78531d2cde7e42c710d	0.00	0.00	7.00	0.00	0.00	0.00
78a1bb51a35fc2bb66b2627d12eee07c	0.00	0.00	7.00	0.00	0.00	0.00
2873454966d999f56925702d7f9c03af	0.00	0.00	0.00	0.00	7.00	0.00
6650bf31a4f385ea316f8ced85b7b072	0.00	0.00	0.00	0.00	7.00	0.00
e7b2c24c5f0d48a0677e3bc80d3e000d	0.00	0.00	0.00	0.00	7.00	0.00
ada02c1a5f9574b335d60afc50ef7026	0.00	0.00	0.00	0.00	4.00	0.00
e326d92bab43870ad4407f8399d87701	0.00	0.00	0.00	0.00	0.00	7.00
abc678856f80ffba6d817c97f3f7733c	0.00	0.00	0.00	0.00	0.00	0.00
ae4748a714220ba7393f0aed19001c14	4.00	0.00	0.00	0.00	0.00	0.00
2e0b3a62081b3d375c5c08ee4f08aa34	0.00	0.00	0.00	0.00	0.00	0.00
aabb5626faa5983b59a294cc0ae967da	0.00	0.00	0.00	0.00	0.00	0.00
27c39dfc97a33345cadd564f2497b68b	0.00	0.00	0.00	0.00	0.00	0.00
965a0bb6d4316557c6647dc457c578f1	0.00	0.00	0.00	7.00	0.00	0.00
89a872dfc309c5fc2ae4a2eebacde930	0.00	0.00	0.00	7.00	0.00	0.00
262d9b1f51fd825b09659c4e1ce0b451	0.00	0.00	0.00	7.00	0.00	0.00
dcd1d7bcd77be5b4897943dad3fc3402	0.00	0.00	0.00	7.00	0.00	0.00
73fbe9b778b095aa07b5c8f37ed24094	0.00	0.00	0.00	7.00	0.00	0.00
9cecf857badffe4599022924da6edb6b	0.00	0.00	0.00	7.00	0.00	0.00
3910607a776521bcbc3845ea6d46c596	0.00	0.00	0.00	0.00	0.00	0.00
167635d0c78052c79e6a71f2757077bc	0.00	0.00	0.00	0.00	0.00	0.00
7d4826e24c0c80f291eb5f7ec57abc1b	0.00	0.00	0.00	0.00	0.00	0.00
41863ef719becd6fa3b6d8d65c06ee5a	0.00	0.00	0.00	0.00	0.00	0.00
b0160164177bd3022f4c23b0bd8f1e82	0.00	0.00	0.00	0.00	0.00	0.00
a7c292cc5d8b5602674aa078b5fb9c3e	0.00	0.00	0.00	0.00	0.00	0.00
4d2a030dc7f0e60fcb3f2b6520e4a2a3	0.00	0.00	0.00	0.00	0.00	0.00
757252a96fb9bb1d5982e0b2bcad096a	0.00	0.00	0.00	0.00	0.00	0.00
8e9387f8d3d2af8b7805f31bbe5c18d1	0.00	0.00	0.00	0.00	0.00	0.00
f69045904699eefc4f5f8b0c3f809d7e	0.00	0.00	0.00	0.00	0.00	0.00
03050b8effcc2563a06be84c9bfddf74	0.00	0.00	0.00	0.00	0.00	0.00
5b5ad29c19134c5e31d765001b1969cc	0.00	0.00	0.00	0.00	0.00	0.00

f4682667584bdc006c599f7972b347b2	0.00	0.00	0.00	0.00	0.00	0.00
916caf16a253aeef1e80e5d6aa95e1f0	0.00	0.00	0.00	0.00	0.00	0.00
61edb41d2d41ec00744defaa6e4837d3	0.00	0.00	0.00	0.00	0.00	0.00
b1a1e255c0b2866f3d9fa34cb382c4d3	0.00	0.00	0.00	0.00	0.00	0.00
cc8323d12abb9681d32a25b77a3b3cad	0.00	0.00	0.00	0.00	0.00	0.00
b465b1e16216096c7518a072298ba9f5	0.00	0.00	0.00	0.00	0.00	0.00
a490db6f4bd90a8aa0902f8114360681	0.00	0.00	0.00	0.00	0.00	0.00
1c39cd37919596733b4938d797283d13	0.00	0.00	0.00	0.00	0.00	0.00
d4a8b72fae2fe84c9645229314c1107e	0.00	0.00	0.00	0.00	0.00	0.00
87a0e33b65f5baa4cbc90aefe315ed0c	0.00	0.00	0.00	0.00	0.00	0.00
ddfa018f2cab35b490aa97a702d84836	0.00	0.00	0.00	0.00	0.00	0.00
2b10bd8c8c0bf139c87fca8186f9c6c2	0.00	0.00	0.00	0.00	0.00	0.00
717d683e852510da081e4ae04f0bc6c7	0.00	0.00	0.00	0.00	0.00	0.00
6fb45a5e921a17404955e72a88f5e98e	0.00	0.00	0.00	0.00	0.00	0.00
308b2a4510c9d7ddaeda7bad6ebb91b1	0.00	0.00	0.00	0.00	0.00	0.00
d7cca22b6b7b3045ed03fcf5e2e58545	0.00	0.00	0.00	0.00	0.00	0.00
6f68558b78e4b8aa23bdd9ff462a7c88	0.00	0.00	0.00	0.00	0.00	0.00
e6a9db651b6fffa30998b534557901cd	0.00	0.00	0.00	0.00	0.00	0.00
8b988ef8bd3dbe1e570ea44799835e56	0.00	0.00	0.00	0.00	0.00	0.00
fc2f2bb27b0624d8bfd8d5478e36977e	0.00	0.00	0.00	0.00	0.00	0.00
85f56ecc056f5453b7fe901e0dcf5478	0.00	0.00	0.00	0.00	0.00	0.00
8d99a40a173bb3ac78f7318f569b6982	0.00	0.00	0.00	0.00	0.00	0.00
a9811f20e934a51566524acfae4838c0	0.00	6.00	0.00	0.00	0.00	0.00
011d89b56c2d415bf29e23f0ef557c7c	0.00	6.00	0.00	0.00	0.00	0.00
43bd30c1bde8368ddb61149a53873a61	0.00	0.00	0.00	0.00	0.00	0.00
9e46bed84a674bc6f493770c0cf3ca39	0.00	0.00	0.00	0.00	0.00	0.00
ae6fb19a4301f9458f9c32a622c5c195	0.00	0.00	0.00	0.00	0.00	0.00
d1271a8b3731a0336735fa278f5e6987	0.00	0.00	0.00	0.00	0.00	0.00
4e433ec8335b29e206dc0d0d1022fd61	0.00	0.00	6.00	0.00	0.00	0.00
0cb9b1cf1d1cea7caf274009a55059c4	0.00	0.00	0.00	0.00	0.00	0.00
d9543658dfb43008980ca827a0c18f6f	0.00	0.00	0.00	0.00	0.00	0.00
c2b05c4a91dd3ef8bc05190377df0bc6	0.00	0.00	0.00	0.00	0.00	0.00
d9315b3ce45c5c5ad41f466546d9c0d2	0.00	0.00	0.00	0.00	6.00	0.00
f607c58da8d27f170ba690a82a0e43a9	0.00	0.00	0.00	0.00	6.00	0.00
02be585acfa4d922ceca797daf2bdcb7	0.00	0.00	0.00	0.00	0.00	0.00
b0379482ea1133c23d713e74cc61ebb9	0.00	0.00	0.00	0.00	0.00	0.00
41ec7d99df12f824da718abd38792bab	0.00	0.00	0.00	0.00	0.00	0.00

f3be9c848b0d220c6c1081c60b59b63d	0.00	0.00	0.00	0.00	0.00	0.00
ae87d16535a1a1f48a3f600b037f0cc3	0.00	0.00	0.00	0.00	0.00	0.00
52f146393407252251fe7c6a26323bff	0.00	0.00	0.00	0.00	0.00	0.00
a9e56af0d6f672c26726c7daef7fd90d	0.00	0.00	0.00	0.00	0.00	0.00
1ec3b9dc277866628a9169cd963d6423	0.00	0.00	0.00	0.00	0.00	0.00
f50dccf112d19417fff2eaf62ed372ef	0.00	0.00	0.00	0.00	0.00	0.00
69bfeea4f30b074791b47b62d0886f4c	0.00	0.00	0.00	0.00	0.00	0.00
a82a06b44b3c69a6ce96c61a4d9a77c9	3.00	0.00	0.00	0.00	0.00	0.00
e15f568598bbc307237b15259fee6e4c	0.00	0.00	0.00	0.00	0.00	0.00
4578c5b4703b24dd7fc017cbc0810f5a	6.00	0.00	0.00	0.00	0.00	0.00
76e72d858816572f535b6f50a10f4073	3.00	0.00	0.00	3.00	0.00	0.00
a3589638c122f413a5dded7f617b0030	0.00	0.00	0.00	0.00	0.00	0.00
7ec5f606341e9b1c91c0d5cbf15786ca	0.00	0.00	0.00	0.00	0.00	0.00
51a4c760f6e00ebc8211e284afd84910	0.00	0.00	0.00	0.00	0.00	0.00
102d065c18645bca22cae406f21e393d	0.00	0.00	0.00	0.00	0.00	0.00
537e6ee0091268fd2fb447c51c4c285f	0.00	0.00	0.00	6.00	0.00	0.00
0d9028e0c6737af27ef6e31c96a28119	0.00	0.00	0.00	6.00	0.00	0.00
389fbcd230e513db0374cd11bb83221a	0.00	0.00	0.00	6.00	0.00	0.00
c723f4e097c7dce7dbbaac5034359b56	0.00	0.00	0.00	6.00	0.00	0.00
c33194f103be74111367b92d78f90895	0.00	0.00	0.00	6.00	0.00	0.00
f3693f841ddf6e408f6761f448c9dd2b	0.00	0.00	0.00	6.00	0.00	0.00
efc16dd4dc3fb7f2d27bc46456af4652	0.00	0.00	0.00	0.00	0.00	0.00
67251f9ad829cbbbd12dbefb0ccc0071	0.00	0.00	0.00	0.00	0.00	0.00
99a61e95b8d7490605e2fb7eeea7ad36	0.00	0.00	0.00	0.00	0.00	0.00
134c8efd78b550f083815401f5c21d31	0.00	0.00	0.00	0.00	0.00	0.00
f2ce57f110b36bf95ad211fa2d8046df	0.00	0.00	0.00	0.00	0.00	0.00
6b2b7d1e8b85e62fafb1a09cb1818809	0.00	0.00	0.00	0.00	0.00	0.00
ddab08e453048791109c941df255343f	0.00	0.00	0.00	0.00	0.00	0.00
b8448310ee33930fa954dcad8069a49b	0.00	0.00	0.00	0.00	0.00	0.00
1d357d07caccc633c906b2b9a6767e26	0.00	0.00	0.00	0.00	0.00	0.00
1f0f5c47eadedb8b2cbab17a71e39c55	0.00	0.00	0.00	0.00	0.00	0.00
9301d1c369edd4573d619b95bbe6cc36	0.00	0.00	0.00	0.00	0.00	0.00
6ba070b344a11207b875673610be06a0	0.00	0.00	0.00	0.00	0.00	0.00
ec1a7218fa66ecd44a2e8911f0c7b9dd	0.00	0.00	0.00	0.00	0.00	0.00
0f8e53236b56d2b7f17a63153f55a5b4	0.00	0.00	0.00	0.00	0.00	0.00
6d188f73d9cc3076cfa4d561a456775f	0.00	0.00	0.00	0.00	0.00	0.00
e6e1b741918f075421700ae080f5ef38	0.00	0.00	0.00	0.00	0.00	0.00

30b81ff11474019d342df79845c4b48e	0.00	0.00	0.00	0.00	0.00	0.00
06dcdec3bfc08d08324ec7279342c402	0.00	0.00	0.00	0.00	0.00	0.00
1b07a7a2f41961cc76521aa26f2bb466	0.00	0.00	0.00	0.00	0.00	0.00
a57946692fc53bb821ab34d492a704ee	0.00	0.00	0.00	0.00	0.00	0.00
48d916964f31cd630b2442f6d7bedf2b	0.00	0.00	0.00	0.00	0.00	0.00
5e2c8573aaa4c712ff9081cc1a599059	0.00	0.00	0.00	0.00	0.00	0.00
beefb95286af3a3840a12c3b20bb6927	0.00	0.00	0.00	0.00	0.00	0.00
d26c1c4af13832d872da4ab8ce5c614c	0.00	0.00	0.00	0.00	0.00	0.00
0256dab41dfe9859f30b2011bc5d9cff	0.00	0.00	0.00	0.00	0.00	0.00
e878b402ca9264efb86d9b67b93a88cd	0.00	0.00	0.00	0.00	0.00	0.00
f36d6ed727a22bf8c1ccf4d6fea8b82e	0.00	0.00	0.00	0.00	0.00	0.00
a06f63c40a77acf26d798eaab0f8a767	0.00	0.00	0.00	0.00	0.00	0.00
a176a679f0900952e3c6afa03d54f78b	0.00	0.00	0.00	0.00	0.00	0.00
b23c56d52b8c7464c375e802606d8edf	0.00	0.00	0.00	0.00	0.00	0.00
ff4ff830e7f9c8e10a734b77ac249078	0.00	0.00	0.00	0.00	0.00	0.00
6af61876151167c516081678b9d806d4	0.00	0.00	0.00	0.00	0.00	0.00
9def08780869fb85b87cb6ef1ddd4f0d	0.00	0.00	0.00	0.00	0.00	0.00
60dfd10c78be620d9e829923a0e44e91	0.00	0.00	0.00	0.00	0.00	0.00
0fd2e0c02af99f0463f4af425ec55c74	0.00	0.00	0.00	0.00	0.00	0.00
00e0008348f66e14f36da59d3360a5a2	0.00	0.00	0.00	0.00	0.00	0.00
04183bef00e0f9a89359c3775794eadb	0.00	0.00	0.00	0.00	0.00	0.00
fc2c513d2281caaa58fb4a1e9d42e1c1	0.00	0.00	0.00	0.00	0.00	0.00
b7246c2fad406f6204cfb5c695553e35	0.00	0.00	0.00	0.00	0.00	0.00
2dc969b371dd8c6e5b9358ee9c236620	0.00	0.00	0.00	0.00	0.00	0.00
35444716732e5d6feeb3b67561a7d3d4	0.00	0.00	0.00	0.00	0.00	0.00
98993736e8671c1e537dbd7987b38547	0.00	0.00	0.00	0.00	0.00	0.00
36fd2a6b33a2dd84251c525644f658b8	0.00	0.00	0.00	0.00	0.00	0.00
8483176bab17d3bdfc86ad9b34cbf7e2	0.00	0.00	0.00	0.00	0.00	0.00
9686e4c1c4fbb3c4a8b45a409637de27	0.00	0.00	0.00	0.00	0.00	0.00
316c3d86567b0ccc0aacbbcdd5b0a350	0.00	0.00	0.00	0.00	0.00	0.00
d3c7684c33b9c9f4c3f2761dfd46661e	0.00	5.00	0.00	0.00	0.00	0.00
44bdc7584f89e3d3d7603cb8d3b9be4f	0.00	5.00	0.00	0.00	0.00	0.00
d27d4a67b13fea4c37e693fbb989a9e2	0.00	0.00	0.00	0.00	0.00	0.00
081d874054d940cba4fc5b2c564bcdfc	0.00	0.00	0.00	0.00	0.00	0.00
3e15d795293c915d735e5d9aad8d4115	0.00	0.00	0.00	0.00	0.00	0.00
0966f70fc6eb8aa99930cdf06e555b27	0.00	0.00	0.00	0.00	0.00	0.00
072fc3555f60436b85d6b70b7c7edd6c	0.00	0.00	0.00	0.00	0.00	0.00

f1d0954eead011d7eac58760f4beba7c	0.00	0.00	0.00	0.00	0.00	0.00
cd2391c06d8cce8a5d1e08342eef3b59	0.00	0.00	0.00	0.00	0.00	0.00
7a744271a80d6e5083d2c5c4529627b2	0.00	0.00	0.00	0.00	0.00	0.00
c38b148043ac4be2d8fe46d47fb32a74	0.00	0.00	0.00	0.00	0.00	0.00
76ee3919ac6ca3c80b78f58e361e55cf	0.00	0.00	0.00	0.00	0.00	0.00
1128b37de09b56e172d35167567a29d2	0.00	0.00	0.00	0.00	5.00	0.00
0871d1fd5fe7058e87cfa2cd1101b6f9	0.00	0.00	0.00	0.00	5.00	0.00
7b4e3e3ede55fc5ea0478585c7f6e68d	0.00	0.00	0.00	0.00	3.00	0.00
955cb950daef7021cde4cf5edd6ce962	0.00	0.00	0.00	0.00	5.00	0.00
28f6725fd27d82c88d036b99af9e7351	0.00	0.00	0.00	0.00	0.00	5.00
ad4af49e14551e9f14a730bf72022e7d	0.00	0.00	0.00	0.00	0.00	3.00
bd645cb8582dfc1ab9430fb92b52ad4e	0.00	0.00	0.00	0.00	0.00	0.00
068d559fdae9463199f1e4275febac1e	0.00	0.00	0.00	0.00	0.00	0.00
c6ba59eba57037f06d98e68f06c1dc13	0.00	0.00	0.00	0.00	0.00	0.00
2f432d7d7d0f6cd24b1ec1f97e1297b4	0.00	0.00	0.00	0.00	0.00	0.00
e0f4cdcbfdccefe449bb4ba41e4852b4	0.00	0.00	0.00	0.00	0.00	0.00
0997059ee27b0d76b6c76b4bff99a0dd	0.00	0.00	0.00	0.00	0.00	0.00
9787a9e27728b4e1d931d4ce92cbd809	0.00	0.00	0.00	0.00	0.00	0.00
10e22845c57f016c077d14ca18070818	0.00	0.00	0.00	0.00	0.00	0.00
001d1baa27473120ab630f0e6ba31cf1	0.00	0.00	0.00	0.00	0.00	0.00
ce251b6ad291d993be4eedb770553551	0.00	0.00	0.00	0.00	0.00	0.00
d238fd63fb194be8b72229e7610e76c5	0.00	0.00	0.00	5.00	0.00	0.00
bf1ac9d5605ead69898296bef2994d54	0.00	0.00	0.00	5.00	0.00	0.00
2f7b19b490094c190d07819b8c304924	0.00	0.00	0.00	5.00	0.00	0.00
1dd2bafef23f0b7757e6cfbf1a4bce83	0.00	0.00	0.00	5.00	0.00	0.00
9bb0042879d443d34240fc3edb207ffe	0.00	0.00	0.00	0.00	0.00	0.00
4254f0a459cd4ac583adee47f3082908	0.00	0.00	0.00	0.00	0.00	0.00
1228fef8617a1a82880da753c1052bc3	0.00	0.00	0.00	0.00	0.00	0.00
af26dfcf9973ee237a97bbefdbf3ba95	0.00	0.00	0.00	0.00	0.00	0.00
0fa5c604ee424c1a930742e1e52f8991	0.00	0.00	0.00	0.00	0.00	0.00
4ccf82180ebaf1f3935efa5a44a9e8af	0.00	0.00	0.00	0.00	0.00	0.00
3e4524a0e65a778911f6e88848d99aad	0.00	0.00	0.00	0.00	0.00	0.00
02934c4550551579ab8dc769b9a34b9a	0.00	0.00	0.00	0.00	0.00	0.00
96b56406633e6e69b5fd65e8803d1e2b	0.00	0.00	0.00	0.00	0.00	0.00
4e7199a4f686c77b1d4f6ef90c9ce2ca	0.00	0.00	0.00	0.00	0.00	0.00
0eb024eb1700b14ffb9a42caef59fa6d	0.00	0.00	0.00	0.00	0.00	0.00
9a2ab70acb36287177390e29c7a381b9	0.00	0.00	0.00	0.00	0.00	0.00

d8535a30f65d4a3fa30bc04b407ace8a	0.00	0.00	0.00	0.00	0.00	0.00
0c7dba8057b7478e64e3fcc2496b1266	0.00	0.00	0.00	0.00	0.00	0.00
9887332f99823c802f6c9ace7c9f05ba	0.00	0.00	0.00	0.00	0.00	0.00
d287f2c588173fcfdeec843961e32bfb	0.00	0.00	0.00	0.00	0.00	0.00
591554f613fc595e6c11c86d22c5bfcf	0.00	0.00	0.00	0.00	0.00	0.00
dfcea040b44ca236872127becd77462d	0.00	0.00	0.00	0.00	0.00	0.00
a07aa2478b99c737bb07e47632fddc2e	0.00	0.00	0.00	0.00	0.00	0.00
a154f80c5f63fef91e612dbfccfa6ffe	0.00	0.00	0.00	0.00	0.00	0.00
6e32bcc8307816adc938cdc4ff0844b4	0.00	0.00	0.00	0.00	0.00	0.00
43a2d89011505ccdfa3a3451d07b6a7f	0.00	0.00	0.00	0.00	0.00	0.00
469943eb8929db8150ce91acf9b073ea	0.00	0.00	0.00	0.00	0.00	0.00
9dd78e0f25ef3bac4182039928ec448f	0.00	0.00	0.00	0.00	0.00	0.00
ed8a22a11164a0f83a7cdf671e217035	0.00	0.00	0.00	0.00	0.00	0.00
7763d969dc92fbe85ad7c5fdbcf4a3ae	0.00	0.00	0.00	0.00	0.00	0.00
6d69862961940e24d41867a3da1dea0a	0.00	0.00	0.00	0.00	0.00	0.00
43431df1c8b720d6f043294c6c9e2877	0.00	0.00	0.00	0.00	0.00	0.00
f51af5c58e2c6d0d91065ae69f7f7463	0.00	0.00	0.00	0.00	0.00	0.00
3b0d3a8ef4217a267e6a2c99bfd5097f	0.00	0.00	0.00	0.00	0.00	0.00
817d7b35feeb0af5b45c5f330f20791b	0.00	0.00	0.00	0.00	0.00	0.00
ead25b7a8a7c763ad69b8b31195a4697	0.00	0.00	0.00	0.00	0.00	0.00
e20bf5dee63cb48c7d6c960f598bfd11	0.00	0.00	0.00	0.00	0.00	0.00
0f2e1947ec75df7d244066e2da2a6578	0.00	0.00	0.00	0.00	0.00	0.00
f6831c086ca9572b816c71d95f12505c	0.00	0.00	0.00	0.00	0.00	0.00
0887c9673086c3343e5e92fbc8bb8e27	0.00	0.00	0.00	0.00	0.00	0.00
d1209865a150bc425aa4bddb9215c21e	0.00	0.00	0.00	0.00	0.00	0.00
ec905f17a37ec5d5589f1e9cfbce12e7	0.00	0.00	0.00	0.00	0.00	0.00
77e0617d2d20935b350b85e9c32354fd	0.00	0.00	0.00	0.00	0.00	0.00
78ce80a526c4708b4922435909001482	0.00	0.00	0.00	0.00	0.00	0.00
5c2ceaa91e5d4a43f0cba6c3b8959c53	0.00	0.00	0.00	0.00	0.00	0.00
13bb5f3bcfd79b5a0ce2163ef4362197	0.00	0.00	0.00	0.00	0.00	0.00
ebeae07a2602df11855e41eaaf2b7458	0.00	0.00	0.00	0.00	0.00	0.00
4281bc4b9724e577b557bcf2bcc12218	0.00	0.00	0.00	0.00	0.00	0.00
e467e9be5b5320ae87052fc26e26101e	0.00	0.00	0.00	0.00	0.00	0.00
5e0e8c2e0266ba2c9b5ca63ca6be44e8	0.00	0.00	0.00	0.00	0.00	0.00
e66e9defb1baf94de5e8be873c20f7a3	0.00	0.00	0.00	0.00	0.00	0.00
2b78e3f0ed591b1d2516a880a9379d12	0.00	0.00	0.00	0.00	0.00	0.00
47ca90b4c55a2b7a06be9a386f4f92f5	0.00	0.00	0.00	0.00	0.00	0.00

9078a587aac0670e0696eeaaff2acc78	0.00	0.00	0.00	0.00	0.00	0.00
e9890ee2b07062b01003070afabe6774	0.00	0.00	0.00	0.00	0.00	0.00
7f9b6837aaba9f973706e978ab47c491	0.00	0.00	0.00	0.00	0.00	0.00
788cfabccabf5ff8dd1124a54e97fa9d	0.00	0.00	0.00	0.00	0.00	0.00
d8d11103a1c85ed7d08cd51e134ccc62	0.00	0.00	0.00	0.00	0.00	0.00
5ed5432f757f49935745a63c3c536f57	0.00	0.00	0.00	0.00	0.00	0.00
db4af5433d32d77b380f8f713c5cb568	0.00	0.00	0.00	0.00	0.00	0.00
cb043348c14ead0bc9e40f3f0b5b418e	0.00	0.00	0.00	0.00	0.00	0.00
491a8ffe42ce46ba80189d47e6dd0e43	0.00	0.00	0.00	0.00	0.00	0.00
d47f6f29147d8b66a6b5adfa31e4d705	0.00	0.00	0.00	0.00	0.00	0.00
cb5c21c84a9df06e35099c39e1e7729f	0.00	0.00	0.00	0.00	0.00	0.00
ddb48ce03e659deaf8eae0e96b06cf5c	0.00	0.00	0.00	0.00	0.00	0.00
f93863c2cca1e0b864ee785c8d2e008d	0.00	0.00	0.00	0.00	0.00	0.00
7d130265a4510922f5731f36057fba50	0.00	0.00	0.00	0.00	0.00	0.00
c9b47f4772d1a041557ae62da6e3aee0	0.00	0.00	0.00	2.00	0.00	0.00
0df94e423697e55c9f11b631a661f242	0.00	0.00	0.00	0.00	0.00	0.00
eb571bb7099d1f4274dd203167e4ecfc	0.00	0.00	0.00	0.00	0.00	0.00
35eea786273ecda88bce81022a61c1a9	0.00	0.00	0.00	0.00	0.00	0.00
16dddb5de4b0885da4e06ee56012ee9c	0.00	0.00	0.00	0.00	0.00	0.00
96bf7315f9dfffe2da98fb0a45c8935e	0.00	0.00	2.00	0.00	0.00	0.00
86500a101004e65c736c7c72d9f36fa2	0.00	0.00	4.00	0.00	0.00	0.00
dc6500c0074a1fbb0a8bc44315bb9380	0.00	0.00	4.00	0.00	0.00	0.00
f0717a52c2c8b3f261e5e9fbc234459e	0.00	0.00	4.00	0.00	0.00	0.00
5454442c2bd72a90ceeda26f52c66be3	0.00	0.00	0.00	0.00	0.00	0.00
6235a038e987a6eb8f2dcdf2f4bee979	0.00	0.00	0.00	0.00	4.00	0.00
ad0140e97b44feba274a4dc4ac374781	0.00	0.00	0.00	0.00	4.00	0.00
1717330e67e429fbe7e155d419f5b4f8	0.00	0.00	0.00	0.00	4.00	0.00
249ecb192bb77652fcf1e9dbfc77f15d	0.00	0.00	0.00	0.00	0.00	0.00
4d55b111538958f10e53634167b4bb54	0.00	0.00	0.00	0.00	0.00	0.00
c3bf628368dec5393a59b42c0d62ee6a	0.00	0.00	0.00	0.00	0.00	0.00
d11ea8b65257ee809b69193888eededf	4.00	0.00	0.00	0.00	0.00	0.00
8913ea5a132117996816e6025a207839	4.00	0.00	0.00	0.00	0.00	0.00
3e8dc060018a792646808355cf546037	0.00	0.00	0.00	0.00	0.00	0.00
7b67c3122a28d314afe52ef3cbbfae3b	0.00	0.00	0.00	0.00	0.00	0.00
e63aabd089ff93f66752a5436ccaacc0	0.00	0.00	0.00	0.00	0.00	0.00
b2576f68753fd1d9a7f2096450294369	0.00	0.00	0.00	0.00	0.00	0.00
1d57af814fbb3d4ad9d0e08e774c49f4	0.00	0.00	0.00	0.00	0.00	0.00

176464878e78480515fc9004867fda80	0.00	0.00	0.00	0.00	0.00	0.00
cf25632e39a9ff2acf65a13d6b791833	0.00	0.00	0.00	0.00	0.00	0.00
887d35bb1a24683dd34b15007829675e	0.00	0.00	0.00	0.00	0.00	0.00
91f24f32182241f3c185120ec8b0647e	0.00	0.00	0.00	0.00	0.00	0.00
916344262b02db933329bd4f734c7f8a	0.00	0.00	0.00	4.00	0.00	0.00
9d74ace509cb12522dfef0379ba9928e	0.00	0.00	0.00	4.00	0.00	0.00
d1db60a3c90f2bca8c2291650cd7bf59	0.00	0.00	0.00	4.00	0.00	0.00
2edf4f20df823c2137c1f26959824823	0.00	0.00	0.00	4.00	0.00	0.00
c35601485c997d472411274a47515348	0.00	0.00	0.00	4.00	0.00	0.00
9eba3bc6f1fd5787a5f5dfd7bb7f9b93	0.00	0.00	0.00	4.00	0.00	0.00
58947b01262e3f98893db6c2076976b0	0.00	0.00	0.00	4.00	0.00	0.00
4509fd6b027a2383221ddc35213fa0e0	0.00	0.00	0.00	4.00	0.00	0.00
4fffac36e24619c3f699b18824e4d1f7	0.00	0.00	0.00	4.00	0.00	0.00
4ca19f46b0a9a2bf0052c45fc2fa60f8	0.00	0.00	0.00	4.00	0.00	0.00
760f25d2247fb6c00087b2136447c029	0.00	0.00	0.00	0.00	0.00	0.00
2adb34d1085a0540681756c7765b6fde	0.00	0.00	0.00	0.00	0.00	0.00
deabd65cff022196373d4488be489a63	0.00	0.00	0.00	0.00	0.00	0.00
7bc011b2cd398c42e9669a7aa48bb6b2	0.00	0.00	0.00	0.00	0.00	0.00
3630abc54dd0c5132f64f8bbf5e75cf1	0.00	0.00	0.00	0.00	0.00	0.00
c834a9c1416f85dc61d5501a5ab096ed	0.00	0.00	0.00	0.00	0.00	0.00
baa95d2cfeceaac972e9e7ff68e67b03	0.00	0.00	0.00	0.00	0.00	0.00
122996e5ab94e779e87b3d84e0ab3371	0.00	0.00	0.00	0.00	0.00	0.00
deed9b0082492eb0e3b648a141e99872	0.00	0.00	0.00	0.00	0.00	0.00
663c05fce31677a4684c526848903a36	0.00	0.00	0.00	0.00	0.00	0.00
7246c24a4e0ac367f008643c6a9648e1	0.00	0.00	0.00	0.00	0.00	0.00
80d05cc4af8d90cb638d7f16568fba55	0.00	0.00	0.00	0.00	0.00	0.00
5e8e7cfb0feae3b018eba98aa0bfa514	0.00	0.00	0.00	0.00	0.00	0.00
c923b3f1468384285874f67194886b51	0.00	0.00	0.00	0.00	0.00	0.00
0297a9f48ab96a3f90fd01ebf55e00dd	0.00	0.00	0.00	0.00	0.00	0.00
cc34f964109779dab9888d26986d1ae9	0.00	0.00	0.00	0.00	0.00	0.00
66548af211d5cf6b7da508fc3a7208fe	0.00	0.00	0.00	0.00	0.00	0.00
18a1733f40105448e7bb6a76b59990d7	0.00	0.00	0.00	0.00	0.00	0.00
6556df8eee98783e0811e91367afe212	0.00	0.00	0.00	0.00	0.00	0.00
915c5de1ef6c487092713295ef0b265c	0.00	0.00	0.00	0.00	0.00	0.00
bf0c11f867e053d7872a1d1c55bfc35f	0.00	0.00	0.00	0.00	0.00	0.00
19e0dea20674b9b58a22063e69dc7399	0.00	0.00	0.00	0.00	0.00	0.00
a12bda150ad5f91fa1817d059f106144	0.00	0.00	0.00	0.00	0.00	0.00

2c2de32bcfa3bab7ae26b71536813553	0.00	0.00	0.00	0.00	0.00	0.00
3a0b3fecddd5c3ea7f80669b61a23157	0.00	0.00	0.00	0.00	0.00	0.00
d1ea8cd4aca1dba2502d9a14351e1bce	0.00	0.00	0.00	0.00	0.00	0.00
9b9ccfd5ca6ef3c46c0ae8a656eb2500	0.00	0.00	0.00	0.00	0.00	0.00
2096e02bb95c9d2cd0004b3c62c05713	0.00	0.00	0.00	0.00	0.00	0.00
3559f0b4ea3930a9f61ab2351764d831	0.00	0.00	0.00	0.00	0.00	0.00
8421acc1ea2a6ada7ab12d721231d9ca	0.00	0.00	0.00	0.00	0.00	0.00
5fee3e48a1c5b89c9e3337a3e1c4a659	0.00	0.00	0.00	0.00	0.00	0.00
c33b7c57538ef4ecf42961a8691c3363	0.00	0.00	0.00	0.00	0.00	0.00
cad9651da9ec765266299fe0527bcbd5	0.00	0.00	0.00	0.00	0.00	0.00
32c9f0f354e8d05bce207df6cdbe186e	0.00	0.00	0.00	0.00	0.00	0.00
b585a25febeb543facb64a3701cbaff0	0.00	0.00	0.00	0.00	0.00	0.00
89e29cf93d512a3fd97503d11e777f5b	0.00	0.00	0.00	0.00	0.00	0.00
28b8656c9d9c6134806eeea9b34480aa	0.00	0.00	0.00	0.00	0.00	0.00
5deef4d55d4f7d391f2f0f3a35bcbaae	0.00	0.00	0.00	0.00	0.00	0.00
7ac92beb5a6c653255714f355f424ea8	0.00	0.00	0.00	0.00	0.00	0.00
ce7016030c934d577cf090112a0bcd94	0.00	0.00	0.00	0.00	0.00	0.00
ce76af6ee12a2d6abc21cd1864b2d47e	0.00	0.00	0.00	0.00	0.00	0.00
c35198cfea6d04b8bd3c8296d45194e6	0.00	0.00	0.00	0.00	0.00	0.00
e63f60ebbe43715a5feee262c4111c9d	0.00	0.00	0.00	0.00	0.00	0.00
c647b7749a76e9f72db436cf06753464	0.00	0.00	0.00	0.00	0.00	0.00
973a65267dd816b0598265fd28184990	0.00	0.00	0.00	0.00	0.00	0.00
9004354993f364a3e03fae244aa05fdf	0.00	0.00	0.00	0.00	0.00	0.00
44f66804b96555b4bb8a05553ecbe3f3	0.00	0.00	0.00	0.00	0.00	0.00
ea263d3dc44036d5a621597b1c2af818	0.00	0.00	0.00	0.00	0.00	0.00
82ce4b09a267f5e0a9575a6711a9cc34	0.00	0.00	0.00	0.00	0.00	0.00
1dcddd697abfaa6b222e34c8e604f68e	0.00	0.00	0.00	0.00	0.00	0.00
abfbfd281e9408414d92b93ef1c9aba3	0.00	0.00	0.00	0.00	0.00	0.00
2ce318bd134427836cda4d372814bc75	0.00	0.00	0.00	0.00	0.00	0.00
ac7662568b7632d0dcc654615623c864	0.00	0.00	0.00	0.00	0.00	0.00
37b308510d6747d030128bb544a46310	0.00	0.00	0.00	0.00	0.00	0.00
4a2a1bdcdf24c8925e5977403e7df1cb	0.00	0.00	0.00	0.00	0.00	0.00
15f491f4cb4be8f96fa58b6f3ac7ad9b	0.00	0.00	0.00	0.00	0.00	0.00
c6e478cbe79341ad227848c706b21c9f	0.00	0.00	0.00	0.00	0.00	0.00
e498ab1aa28968975e96457c4be1a4d0	0.00	0.00	0.00	0.00	0.00	0.00
09b56a05c01fb3950cc557c6c29d3b34	0.00	0.00	0.00	0.00	0.00	0.00
4318456c503083209591d1a2b6551f93	0.00	0.00	0.00	0.00	0.00	0.00

1af3b5e868c1050321eaca123abbd943	0.00	0.00	0.00	0.00	0.00	0.00
0c52ef58fb80bf071b4412d4cbd7d303	0.00	0.00	0.00	0.00	0.00	0.00
e11462ea65fb320980c412477771f2bc	0.00	0.00	0.00	0.00	0.00	0.00
3631055dceb899b3d83c56deccbf0f47	0.00	0.00	0.00	0.00	0.00	0.00
2ab700dab24ef87cc14def3b0a98d866	0.00	0.00	0.00	0.00	0.00	0.00
db35e5601bbcdfbd3e2e98652063f651	0.00	0.00	0.00	0.00	0.00	0.00
b5331d95978b203c938da28e618e5a10	0.00	0.00	0.00	0.00	0.00	0.00
44ba656ed791aa131a82c4cde1bc1856	0.00	0.00	0.00	0.00	0.00	0.00
9e45e46d21de89773cefd34489e9c144	0.00	0.00	0.00	0.00	0.00	0.00
16029531ef83ce6b77d9a4850914fa46	0.00	0.00	0.00	0.00	0.00	0.00
c8c552415871df071765f9411943cea0	0.00	0.00	0.00	0.00	0.00	0.00
57e2dca5a0244a0ced04a85fa1ee3439	0.00	0.00	0.00	0.00	0.00	0.00
8639386973165b4c37c07340c4bb08d1	0.00	0.00	0.00	0.00	0.00	0.00
470a2be5bb9e534dd9ef4146bc5f7eb3	0.00	0.00	0.00	0.00	0.00	0.00
8fcb29337da4f5bfe94732ba080791f2	0.00	0.00	0.00	0.00	0.00	0.00
e28140cd24eee6271df67f491e234ab5	0.00	0.00	0.00	0.00	0.00	0.00
be070806a371cc79e6cc2c25c2fc0b46	0.00	0.00	0.00	0.00	0.00	0.00
79c7d2bbf403a7dd1c5c5b0002e3e341	0.00	0.00	0.00	0.00	0.00	0.00
c219dc74c3c17ef9bd6dc1d7a46a35c1	0.00	0.00	0.00	0.00	0.00	0.00
612711092be5ac85baaade0af0b70bb5	0.00	0.00	0.00	0.00	0.00	0.00
30aa2f9bd4347cb4b3f4011b76d0b79c	0.00	0.00	0.00	0.00	0.00	0.00
968392476815ea8de4c2edd904335c3d	0.00	0.00	0.00	0.00	0.00	0.00
cc8b3964d049aacb1991e5158cf7c3e2	0.00	3.00	0.00	0.00	0.00	0.00
bbca02d48114ac171d0fd84545ed3f1f	0.00	3.00	0.00	0.00	0.00	0.00
05c3030d73c355501c2c786a00c56488	0.00	0.00	0.00	0.00	0.00	0.00
3c2934680d564f24be3099a620adb243	0.00	0.00	0.00	0.00	0.00	0.00
554d539b9d9bd5be0359bba5dfb70d0b	0.00	0.00	0.00	0.00	0.00	0.00
3a6b3e75192182b675d46f0a8fb590c4	0.00	0.00	0.00	0.00	0.00	0.00
ef29e92e6bfb3e663662f81e730cbdd1	0.00	0.00	0.00	0.00	0.00	0.00
cfec875a97c330ce86668ce2719fac4b	0.00	0.00	0.00	0.00	0.00	0.00
b0b3e4ec533f7d6b08210182fbdb30b7	0.00	0.00	0.00	0.00	0.00	0.00
90254dab523c41930b2d1812de164ec8	0.00	0.00	0.00	0.00	0.00	0.00
8d633a40c2d7ff37a1858180b75e6c59	0.00	0.00	0.00	0.00	0.00	0.00
45eaac1ac4e7abd150b4a46dcd0a3cbc	0.00	0.00	3.00	0.00	0.00	0.00
144fcd166e354941d0397a097b68cc75	0.00	0.00	3.00	0.00	0.00	0.00
8e17975e8478af8fc0dcd19ce7534b50	0.00	0.00	0.00	0.00	0.00	0.00
fb13a4d425c47bd2caf33ab816494cc9	0.00	0.00	0.00	0.00	3.00	0.00

61cd1abfe6c82591691b3930fecbf246	0.00	0.00	0.00	0.00	3.00	0.00
dff38e05470586b6a22b18b5e5c6feae	0.00	0.00	0.00	0.00	0.00	3.00
d338d888c505699e880b3e7e11ad011b	0.00	0.00	0.00	0.00	0.00	3.00
666290285660ad7a46bafc9dc57edb89	0.00	0.00	0.00	0.00	0.00	3.00
747aa4db7095ff25575528b22ad92ca9	0.00	0.00	0.00	0.00	0.00	0.00
da8b4e3322c5f448dd1920256d0f7494	0.00	0.00	0.00	0.00	0.00	0.00
f6c1894b9f56628b8b3dcd2ac78519ab	0.00	0.00	0.00	0.00	0.00	0.00
9ccc8e92b56e342a357323a021cb0873	0.00	0.00	0.00	0.00	0.00	0.00
11bfefdf62f3b4247a5c3fee81041609	0.00	0.00	0.00	0.00	0.00	0.00
2f934e81f360bf94b0f1321a65164c18	0.00	0.00	0.00	0.00	0.00	0.00
000ff65081cc3f2cd921d447d6df4ded	0.00	0.00	0.00	0.00	0.00	0.00
6bc78df0c09b8ae38d15ee2d196a938d	0.00	0.00	0.00	0.00	0.00	0.00
7655c0b98a23f51b85c9e370a83f16d8	0.00	0.00	0.00	0.00	0.00	0.00
0d8c17171186585d8514af8e51dc41a5	0.00	0.00	0.00	0.00	0.00	0.00
8d0d9c270faaf1d1f12f1b7edce3144b	0.00	0.00	0.00	0.00	0.00	0.00
d146e1b47e40fecaa202f1efccb6a344	0.00	0.00	0.00	0.00	0.00	0.00
89bb6c090bb114b46432d089347c50b9	0.00	0.00	0.00	0.00	0.00	0.00
98c6cb4bad469d5beea8606d8eb7f64a	3.00	0.00	0.00	0.00	0.00	0.00
0a0d7512457464c7835a387cbd027d35	3.00	0.00	0.00	0.00	0.00	0.00
9b53838c9cac3d6ed7856a675180a731	0.00	0.00	0.00	0.00	0.00	0.00
f03385ab9fbb9fca155e0956d3339d65	0.00	0.00	0.00	0.00	0.00	0.00
6265ada705d9aff1c4ff1c7abf53aa90	0.00	0.00	0.00	0.00	0.00	0.00
d48a99b0aa559b4bc678e79f86134acc	0.00	0.00	0.00	0.00	0.00	0.00
166885f98b020649d63a117d60845090	0.00	0.00	0.00	0.00	0.00	0.00
cf1daa9f36199afe082bb023947944c4	0.00	0.00	0.00	3.00	0.00	0.00
bb9603d4efb92bcf80d8463ebcad51fa	0.00	0.00	0.00	3.00	0.00	0.00
5db928a6082fa132d4b483dc29f324fc	0.00	0.00	0.00	3.00	0.00	0.00
e0d25f7af941fbf698ea58e30a88b215	0.00	0.00	0.00	3.00	0.00	0.00
3ca53903eabce6ddb11f1a8b8e99c40b	0.00	0.00	0.00	3.00	0.00	0.00
0f41528a8cf9643522d97d094d2061f3	0.00	0.00	0.00	3.00	0.00	0.00
9e443b2f145b328cd7a22abda9be2aaa	0.00	0.00	0.00	3.00	0.00	0.00
8f05ee64ba1aeb74c4adfce45401374f	0.00	0.00	0.00	3.00	0.00	0.00
24e597c64eeb24210cdd115b338619c8	0.00	0.00	0.00	3.00	0.00	0.00
97dfb71585624e3236e7072b099b19a1	0.00	0.00	0.00	3.00	0.00	0.00
073e68a4bc405ecd756258bae92afd44	0.00	0.00	0.00	3.00	0.00	0.00
cdebd472acb76c8534a99dc0f0f7e58b	0.00	0.00	0.00	3.00	0.00	0.00
8e98068ab20f73bcbf8255f32da36179	0.00	0.00	0.00	3.00	0.00	0.00

fcd55f8e6e6e40bc30e881ac334450f3	0.00	0.00	0.00	0.00	0.00	0.00
1e6e91a9cb938f17d62f2bfaf6025013	0.00	0.00	0.00	0.00	0.00	0.00
d2ccc8599ab8031e012f0daa7d47667f	0.00	0.00	0.00	0.00	0.00	0.00
a4153adbe09ff6d4db9133bcfcbf4858	0.00	0.00	0.00	0.00	0.00	0.00
9d6054ffb34d94d456270ea5f8be3250	0.00	0.00	0.00	0.00	0.00	0.00
e56114436f0345248b8a26e8d7424611	0.00	0.00	0.00	0.00	0.00	0.00
191759331df04a2d8e2ba9085e6eaf2a	0.00	0.00	0.00	0.00	0.00	0.00
fc2310d2b3becbb907698a022b2f7c5a	0.00	0.00	0.00	0.00	0.00	0.00
816c3d478ebffd167938153c8dc4faa0	0.00	0.00	0.00	0.00	0.00	0.00
4d2b917e3b6dd2b503d13186551575f6	0.00	0.00	0.00	0.00	0.00	0.00
2429f41b4260cbefb67422e4e4288623	0.00	0.00	0.00	0.00	0.00	0.00
dd645ee559309050da790849bea5146b	0.00	0.00	0.00	0.00	0.00	0.00
aa07e18a6e7cd391a5f2bd21067447b7	0.00	0.00	0.00	0.00	0.00	0.00
59a5e1c111aa410dfed789ab94ce9875	0.00	0.00	0.00	0.00	0.00	0.00
b53ae695ee1fad86bebb21aedc1993ef	0.00	0.00	0.00	0.00	0.00	0.00
51cdf9fb849201b7099cda4cdef53ced	0.00	0.00	0.00	0.00	0.00	0.00
92673d04cfd1859bdae7bf19390ddf09	0.00	0.00	0.00	0.00	0.00	0.00
e59dd888ac2fc185604afa39d17081ce	0.00	0.00	0.00	0.00	0.00	0.00
ed8b01644209de80626ed918e9028d9e	0.00	0.00	0.00	0.00	0.00	0.00
ac14a87d93a3305b5867be59df5325ea	0.00	0.00	0.00	0.00	0.00	0.00
dd48db332ca238985db31eba1824f968	0.00	0.00	0.00	0.00	0.00	0.00
c7e2612b7a15f0e65cdaef55ccf38de0	0.00	0.00	0.00	0.00	0.00	0.00
9504abee7c788fc31d262cac68691db0	0.00	0.00	0.00	0.00	0.00	0.00
3f8774ba5f5d93d3d43a96a8de11ba04	0.00	0.00	0.00	0.00	0.00	0.00
d316906e063911eb46e1fd04c11bf889	0.00	0.00	0.00	0.00	0.00	0.00
db7f1ad61a4e5164891c3a0d0b2ede70	0.00	0.00	0.00	0.00	0.00	0.00
ef97d79b77b357b4efe61b0a31153be2	0.00	0.00	0.00	0.00	0.00	0.00
736a4665e1e248e6644ceb2cd7c56660	0.00	0.00	0.00	0.00	0.00	0.00
ebdd1fa51b115f9fcfca78b07fd1e85c	0.00	0.00	0.00	0.00	0.00	0.00
9b20483bf842b1279071636337814030	0.00	0.00	0.00	0.00	0.00	0.00
ece3c02646e8ca07815ba2b64aa89494	0.00	0.00	0.00	0.00	0.00	0.00
b8c032cabc03cf7f74d156e6cfcd0702	0.00	0.00	0.00	0.00	0.00	0.00
8960b86499d433f439528a84ead75bc8	0.00	0.00	0.00	0.00	0.00	0.00
4434103370917f9aa40b9a25268e231b	0.00	0.00	0.00	0.00	0.00	0.00
a16aa99a4d765944949125a260699442	0.00	0.00	0.00	0.00	0.00	0.00
85b9e27a090987e383d9d8c02493aa0e	0.00	0.00	0.00	0.00	0.00	0.00
5186ec053798f41cc76fe02fed9f63ad	0.00	0.00	0.00	0.00	0.00	0.00

3d40a0f48d974fec907303f5fef949aa	0.00	0.00	0.00	0.00	0.00	0.00
da6d33c117e54ae4ad9ca296aa5ecdd8	0.00	0.00	0.00	0.00	0.00	0.00
3365f0f8f3f11dc5bb02abb74d7e19ce	0.00	0.00	0.00	0.00	0.00	0.00
58635fb03dc4eb1769a99347bf2506ee	0.00	0.00	0.00	0.00	0.00	0.00
20d09ed0a0b406f3d6bcbd72668870e9	0.00	0.00	0.00	0.00	0.00	0.00
6c7d873dae81440781440336e6870157	0.00	0.00	0.00	0.00	0.00	0.00
bda2e5e6d69c2ea2fd1ed07ec39820f4	0.00	0.00	0.00	0.00	0.00	0.00
0cd7d6bce8a117857ed4e9539eabde80	0.00	0.00	0.00	0.00	0.00	0.00
ad087a56f3710fceaf345af053702fcb	0.00	0.00	0.00	0.00	0.00	0.00
322707434a7a3f22ea72fc4730ce0be6	0.00	0.00	0.00	0.00	0.00	0.00
2b4499629a9afabb1862e5aabf0158e2	0.00	0.00	0.00	0.00	0.00	0.00
9239feb91eb8e56bd45b349bed315133	0.00	0.00	0.00	0.00	0.00	0.00
d6ce8022ce80ab19e3f168387e377d45	0.00	0.00	0.00	0.00	0.00	0.00
3eec0cfb2fa38d91a54e24cbd92b3022	0.00	0.00	0.00	0.00	0.00	0.00
4e7a968dff82060ab09dd3498de6fe56	0.00	0.00	0.00	0.00	0.00	0.00
43428eba6a7d1cbcf596d946d1249fd7	0.00	0.00	0.00	0.00	0.00	0.00
17ac2cfdb5b054c6cf980c8df13bd71d	0.00	0.00	0.00	0.00	0.00	0.00
929c4151baf4e8c2260f4f121576abc5	0.00	0.00	0.00	0.00	0.00	0.00
8a43d3ba9c44252f3d4094d816340a57	0.00	0.00	0.00	0.00	0.00	0.00
4ae6badf8a9f87bc4b1beb4470c88145	0.00	0.00	0.00	0.00	0.00	0.00
2f3f13d5be4fc008477f9c40ece9051c	0.00	0.00	0.00	0.00	0.00	0.00
cd0a40d92f7e3a48434d517e3a5ec1ae	0.00	0.00	0.00	0.00	0.00	0.00
8b72f7c1c62308e7ffb1d3c727e2a585	0.00	0.00	0.00	0.00	0.00	0.00
3415820fc8034fe9d6659500ca049c27	0.00	0.00	0.00	0.00	0.00	0.00
dc1d47823e38b4571139af9df7309910	0.00	0.00	0.00	0.00	0.00	0.00
0500d5aa3d7b9f5ec4ce2d0ddf3dc1c0	0.00	0.00	0.00	0.00	0.00	0.00
28f109aea95f74781134dec86bd31a04	0.00	0.00	0.00	0.00	0.00	0.00
8621c1186edbbb214e8e83c694a3b605	0.00	0.00	0.00	0.00	0.00	0.00
c0e857481a9043ca313aa1327ed7a71c	0.00	0.00	0.00	0.00	0.00	0.00
6aac50907fa9863f16db0db1edb15450	0.00	0.00	0.00	0.00	0.00	0.00
0d73e3384915e845acf6d32d949061c1	0.00	0.00	0.00	0.00	0.00	0.00
6865d5263c6a6326491e0fbf3e007d82	0.00	0.00	0.00	0.00	0.00	0.00
8704f87d1df53de9fe2c3c42ea20b7fa	0.00	0.00	0.00	0.00	0.00	0.00
81472d3bcd62e7a6caded534f59c5f87	0.00	0.00	0.00	0.00	0.00	0.00
b0ae020cf6702eead2634f3d7dec60ad	0.00	0.00	0.00	0.00	0.00	0.00
2275956a9190087cd4afcdc98fbe9c04	0.00	0.00	0.00	0.00	0.00	0.00
249c57900408ec1a7a2fca1ee556d6f7	0.00	0.00	0.00	0.00	0.00	0.00

057c959b4f532886c8ee47e08b786c4f	0.00	0.00	0.00	0.00	0.00	0.00
1eac4e014d58f7da80ff3cd0099e6986	0.00	0.00	0.00	0.00	0.00	0.00
e67be099b13ae8978967a1e49e610f47	0.00	0.00	0.00	0.00	0.00	0.00
3712132030a7a689da3c568bfbbd3ed7	0.00	0.00	0.00	0.00	0.00	0.00
4050199ca96b13b733fc19ba9c65385b	0.00	0.00	0.00	0.00	0.00	0.00
3176bdbb073fa98ebe5d318e362757a6	0.00	0.00	0.00	0.00	0.00	0.00
92d7fb9db8ad15bd23a79e96dca56cef	0.00	0.00	0.00	0.00	0.00	0.00
2737fda2e6c5bbcca28b4d12141319f3	0.00	0.00	0.00	0.00	0.00	0.00
6671181cd66298277c8d124f333c2cfe	0.00	0.00	0.00	0.00	0.00	0.00
d9714005cc4ee6e354942da8bff69bc6	0.00	0.00	0.00	0.00	0.00	0.00
cd711bf7426f2bd97a56739b144726c6	0.00	0.00	0.00	0.00	0.00	0.00
1ebe1a2712a6f558c5855ae03ebfd00a	0.00	0.00	0.00	0.00	0.00	0.00
e51f49c6344f3ec129ab4e5ecbc3b69c	0.00	0.00	0.00	0.00	0.00	0.00
39f49e62e8c527df377b65ffa5a5ba53	0.00	0.00	0.00	0.00	0.00	0.00
890ff72d8a1eaede8a9718c3c7a4134b	0.00	0.00	0.00	0.00	0.00	0.00
bbdc0a606849fe38f85be6427c050ae8	0.00	2.00	0.00	0.00	0.00	0.00
2064f619e9d74573d10e55167ef86266	0.00	0.00	0.00	0.00	0.00	0.00
9a4fd7e6b4c85a3c9b5a2b2ae80d6eaa	0.00	0.00	0.00	0.00	0.00	0.00
a1c6504e8981cc8645a7f588e5f8408c	0.00	0.00	0.00	0.00	0.00	0.00
3a4c7a200b9dc854bf615cbd960ded2f	0.00	0.00	0.00	0.00	0.00	0.00
a60a9724c73cb25999af033bd73c9fec	0.00	0.00	0.00	0.00	0.00	0.00
3b3926aa0fcebb1ef6b8c3f7f2e162e7	0.00	0.00	0.00	0.00	0.00	0.00
5562116461e3bd52602a03e1e1ab1244	0.00	0.00	0.00	0.00	0.00	0.00
5d90f65585bb61dbe11216369c8953bc	0.00	0.00	0.00	0.00	0.00	0.00
b7940359acbe0fe72e75f4877ca54177	0.00	0.00	0.00	0.00	0.00	0.00
c28b5fc53b935bcc1632e5ea4989c80e	0.00	0.00	0.00	0.00	2.00	0.00
706c11167a80d35dc6431dbe33fdd0eb	0.00	0.00	0.00	0.00	2.00	0.00
fc6353d71ef977c2414c22d8aefac48a	0.00	0.00	0.00	0.00	2.00	0.00
946a1ed41e8a4775ceb0e5a7517176e8	0.00	0.00	0.00	0.00	0.00	0.00
287c50c67985e954b2810af998b5b030	0.00	0.00	0.00	0.00	0.00	0.00
15c6952576f5f6d441f00792aacb86bf	0.00	0.00	0.00	0.00	0.00	0.00
af21743f972967e615c4762695dc3a2b	0.00	0.00	0.00	0.00	0.00	0.00
03d705f22e6af6022d4d7bacde095ed6	0.00	0.00	0.00	0.00	0.00	0.00
f659dd4ff0fac85ec37a1bedd2946721	0.00	0.00	0.00	0.00	0.00	0.00
afddbd3efe01c9fb39bbd9066490776f	0.00	0.00	0.00	0.00	0.00	0.00
e72d6bfe388740891a9c686af409ee17	0.00	0.00	0.00	0.00	0.00	0.00
03a854123d29b5cb275476ca0ab9c7eb	0.00	0.00	0.00	0.00	0.00	0.00

10cddaeaa96f61befb0b216aadb1a042	0.00	0.00	0.00	0.00	0.00	0.00
1bda662ae4cc025a1245bbc8c3e6e2f0	0.00	0.00	0.00	0.00	0.00	0.00
6724c390cb160aa61a7609d8c890c1de	0.00	0.00	0.00	0.00	0.00	0.00
5b7054410ac1af175be6f81c9243f73d	0.00	0.00	0.00	0.00	0.00	0.00
d158d1db981808c90fccfd8cb2f26f6f	0.00	0.00	0.00	0.00	0.00	0.00
e687c7aa4d6dd85a59c6a39d637730cf	0.00	0.00	0.00	0.00	0.00	0.00
18486623607ab353204323fd78320920	0.00	0.00	0.00	0.00	0.00	0.00
f591209e26d27536bac8dd1c1528725d	0.00	0.00	0.00	0.00	0.00	0.00
a33a27f81ae58fbe01833ac4c171a828	0.00	0.00	0.00	0.00	0.00	0.00
8f31bf6388745d073246b5e095dd9e95	2.00	0.00	0.00	0.00	0.00	0.00
dec3727a8cd95c1b97c0aa3ac7e9cb0f	2.00	0.00	0.00	0.00	0.00	0.00
b8e1fa7c35fa1391d609df2a260fd114	2.00	0.00	0.00	0.00	0.00	0.00
122ea5778927e13bf8c9c5f07d337e9e	2.00	0.00	0.00	0.00	0.00	0.00
ac55cc6fe98d75c9dfdb0f69cf2611ac	0.00	0.00	0.00	0.00	0.00	0.00
daa9d6ae544563617b640cf914bc5e6c	0.00	0.00	0.00	0.00	0.00	0.00
e4225dd6d93cf0f444c243ee0be94165	0.00	0.00	0.00	0.00	0.00	0.00
51f03fe67b64d0f860d49ca681572872	0.00	0.00	0.00	0.00	0.00	0.00
3520c815646e0253ca92da98f85899e5	0.00	0.00	0.00	0.00	0.00	0.00
ecd072de4b0d5e4526c6ced0d2c96366	0.00	0.00	0.00	0.00	0.00	0.00
bd538de5b94dcd7e8ee09923c6aa5e20	0.00	0.00	0.00	0.00	0.00	0.00
66fb7a34e78b3600a6918893c72b8050	0.00	0.00	0.00	0.00	0.00	0.00
aa279c1008b2c378d3bc0ed92f90da65	0.00	0.00	0.00	2.00	0.00	0.00
60385ac98a59ed74e133d999446c7fd2	0.00	0.00	0.00	2.00	0.00	0.00
dcb23ce444d2963a23e18c4f23ee48fb	0.00	0.00	0.00	2.00	0.00	0.00
569ae6e38a27aec08a0934f22784dbc3	0.00	0.00	0.00	2.00	0.00	0.00
4c08f3ffec550bb1d5560def1001c3bc	0.00	0.00	0.00	2.00	0.00	0.00
2ceb81989af89414c2b6b4b08a84033b	0.00	0.00	0.00	2.00	0.00	0.00
ef16fcc0a0a0c9630f868985ca0fd330	0.00	0.00	0.00	2.00	0.00	0.00
251558413977082b0ec2d8b13fd4df56	0.00	0.00	0.00	2.00	0.00	0.00
e96d72808e1cd1e836f885fb35c6d099	0.00	0.00	0.00	2.00	0.00	0.00
153f6a2d9dcdd3b6c18452842f9630ce	0.00	0.00	0.00	2.00	0.00	0.00
8ec24ea91e203e84712f887bc596197a	0.00	0.00	0.00	2.00	0.00	0.00
028f0d0e0705061f56837469d26eed3e	0.00	0.00	0.00	2.00	0.00	0.00
430c9ce13594aa6e6d1eb57ad5e776c5	0.00	0.00	0.00	2.00	0.00	0.00
fa4c905a74f94db42df2632c3c47a397	0.00	0.00	0.00	2.00	0.00	0.00
ec42f5a125f042d847ecbc17d8274ef1	0.00	0.00	0.00	0.00	0.00	0.00
6f124f086861a9cabbbb612bf0e09629	0.00	0.00	0.00	0.00	0.00	0.00

74a2ac255501fa22d62326401147a19e	0.00	0.00	0.00	0.00	0.00	0.00
6f5ced9aa195b6583e98c616d7fe6829	0.00	0.00	0.00	0.00	0.00	0.00
673943b14d9c1a216a242f5030309c4e	0.00	0.00	0.00	0.00	0.00	0.00
df2f9399e80bea8413f73823459b1812	0.00	0.00	0.00	0.00	0.00	0.00
b3a0feea9a5ea74add6fca3d1148c4bb	0.00	0.00	0.00	0.00	0.00	0.00
ee46da23eba19076257769bd630ccdf7	0.00	0.00	0.00	0.00	0.00	0.00
78d079874fc37930e4ae6e8c14eb82cb	0.00	0.00	0.00	0.00	0.00	0.00
900680d69244c96a33ea420a5da3df58	0.00	0.00	0.00	0.00	0.00	0.00
b86ee240170d9ee9cab7639f86771b9b	0.00	0.00	0.00	0.00	0.00	0.00
79fc6c20df266587daf8ffadb8036e83	0.00	0.00	0.00	0.00	0.00	0.00
0e41d846a3ef9022e34c1b08ad594ed0	0.00	0.00	0.00	0.00	0.00	0.00
158bed7f9ee5afcf3ec649723eb9922f	0.00	0.00	0.00	0.00	0.00	0.00
45f6c705260b22c8365a5ad922470f5a	0.00	0.00	0.00	0.00	0.00	0.00
ee7cdf71bd8538a2899ec23b46985b7b	0.00	0.00	0.00	0.00	0.00	0.00
24137c5e8f411160d6a224d09b428fff	0.00	0.00	0.00	0.00	0.00	0.00
407aaa3f43e11d634553857602566220	0.00	0.00	0.00	0.00	0.00	0.00
e4b330234fbc296df8a08ad0b36253bd	0.00	0.00	0.00	0.00	0.00	0.00
af16fa94757f7e247b029020b508cd51	0.00	0.00	0.00	0.00	0.00	0.00
ee50a67a94fa49177dd6bffc490ae37e	0.00	0.00	0.00	0.00	0.00	0.00
47cf2ff22963772f4b226bf005cb8869	0.00	0.00	0.00	0.00	0.00	0.00
a9c1755e7d9e7730b7cf595316fea370	0.00	0.00	0.00	0.00	0.00	0.00
82b0f65f6cdc8f4532bf8e5a869ca336	0.00	0.00	0.00	0.00	0.00	0.00
f2776290ecab40993ad226f87a9c245c	0.00	0.00	0.00	0.00	0.00	0.00
f045ac798c84fc5eb948ab3a8f872984	0.00	0.00	0.00	0.00	0.00	0.00
848eb8b5e9f7dcec058286d7df4c358c	0.00	0.00	0.00	0.00	0.00	0.00
64835c62ad26dcf0e37799239aeed70b	0.00	0.00	0.00	0.00	0.00	0.00
55a2fc2bedafa31dd4f41e0569b9fe47	0.00	0.00	0.00	0.00	0.00	0.00
87f1bf3f697e4dd44da7bcb318368bf3	0.00	0.00	0.00	0.00	0.00	0.00
10777fa6627bc9fcc56333d230864b4b	0.00	0.00	0.00	0.00	0.00	0.00
82d7050a26d929cf0cb998c8019f61b2	0.00	0.00	0.00	0.00	0.00	0.00
b1c261abfdbc9a4b7538178dd43976be	0.00	0.00	0.00	0.00	0.00	0.00
ecd7740a2138efb2527fa9314327728a	0.00	0.00	0.00	0.00	0.00	0.00
769ff951930bfc6ff9039f1763776134	0.00	0.00	0.00	0.00	0.00	0.00
8739c8e14c0853535e7b7912a44c01bc	0.00	0.00	0.00	0.00	0.00	0.00
fe17b0febef6942071c6fe30b8b31e67	0.00	0.00	0.00	0.00	0.00	0.00
e6f8491d00512790afa2d465942cad28	0.00	0.00	0.00	0.00	0.00	0.00
9105559705f629fa50b77b8855e7f287	0.00	0.00	0.00	0.00	0.00	0.00

6f5f3c9c1ea8bdceaf7b25f1cb7b3b2a	0.00	0.00	0.00	0.00	0.00	0.00
cd245284e364dd60c4bffa5e44073290	0.00	0.00	0.00	0.00	0.00	0.00
3ff6bc9622277e50c4e1d8daec2b7503	0.00	0.00	0.00	0.00	0.00	0.00
c391af85c5fe19362c7beabbee0c0abf	0.00	0.00	0.00	0.00	0.00	0.00
4b60ec700908470685473534 7c458fa2	0.00	0.00	0.00	0.00	0.00	0.00
b68b798fdc7e79c665003885bed0332c	0.00	0.00	0.00	0.00	0.00	0.00
b56f5dff55cdcf6586e051abcb70044a	0.00	0.00	0.00	0.00	0.00	0.00
c51d5af66e9152e8b01b8e56e8f7e4fa	0.00	0.00	0.00	0.00	0.00	0.00
a6563b2ba361c80dce694251075003a1	0.00	0.00	0.00	0.00	0.00	0.00
3f2471a10fcda5594bc1d079df1789bc	0.00	0.00	0.00	0.00	0.00	0.00
474434d6578a7af2b3aeaa4d45ff6a6a	0.00	0.00	0.00	0.00	0.00	0.00
ebd2a67a4d309724306eaa32cd4b8de0	0.00	0.00	0.00	0.00	0.00	0.00
4cfd754400a01b1e68bbd30631954b38	0.00	0.00	0.00	0.00	0.00	0.00
3517888230e15abfcf04af272cb08183	0.00	0.00	0.00	0.00	0.00	0.00
dae854254211c91ae28a2e04d605bd30	0.00	0.00	0.00	0.00	0.00	0.00
a14606191a27028f11df05584c6570f0	0.00	0.00	0.00	0.00	0.00	0.00
5b21ad8904948f9b043d08c003344389	0.00	0.00	0.00	0.00	0.00	0.00
0514aeb94e18da958ce0f89e9e0140c8	0.00	0.00	0.00	0.00	0.00	0.00
8c9ba448ca5d5d68e74eda7b0b9d9a68	0.00	0.00	0.00	0.00	0.00	0.00
e12625411220e1f842cdcb7d927b3d17	0.00	0.00	0.00	0.00	0.00	0.00
9b632413b46eeee1b61a6bbaca8d481a	0.00	0.00	0.00	0.00	0.00	0.00
7ab48a94c18c68b8295fdd11fc8b983c	0.00	0.00	0.00	0.00	0.00	0.00
6d8959c0f86974fc9873bf2fd195c95e	0.00	0.00	0.00	0.00	0.00	0.00
c4a43c9c814e46c4c3a9b0c4ad00aee4	0.00	0.00	0.00	0.00	0.00	0.00
9f1d40f60696b656791b42e185a4f388	0.00	0.00	0.00	0.00	0.00	0.00
1014097f8c49430231b868734701560e	0.00	0.00	0.00	0.00	0.00	0.00
06b83e97027706db3461adfb96ace38e	0.00	0.00	0.00	0.00	0.00	0.00
777218be56b94693b8241ccf4880a96b	0.00	0.00	0.00	0.00	0.00	0.00
d01748fce0086b3329f5a9e7201c878f	0.00	0.00	0.00	0.00	0.00	0.00
1a10a5776078e2c6c717e6d02d7e063d	0.00	0.00	0.00	0.00	0.00	0.00
855fff22020aaf476e14a378e9881d2a	0.00	0.00	0.00	0.00	0.00	0.00
30c5d94ab1b97a02e5df7235a57e7e52	0.00	0.00	0.00	0.00	0.00	0.00
3e336840cde79db6fa6652f734dbecb8	0.00	0.00	0.00	0.00	0.00	0.00
1eb00defbe0aaf2383dd263d04cab706	0.00	0.00	0.00	0.00	0.00	0.00
979ada1d28f7e48a59bcf8019f8d1c51	0.00	0.00	0.00	0.00	0.00	0.00
bf5180b958951fed6276faa42ab2707c	0.00	0.00	0.00	0.00	0.00	0.00
d4537a6a6e278220b1a18c1d1a903a89	0.00	0.00	0.00	0.00	0.00	0.00

05c6a601e771eb672b996f778521f901	0.00	0.00	0.00	0.00	0.00	0.00
bf57f4c94085074c92907c534f3c02dc	0.00	0.00	0.00	0.00	0.00	0.00
20e8e90c4a6fd879a07a389850d4a719	0.00	0.00	0.00	0.00	0.00	0.00
53969a51983461d8851f91fab68a886f	0.00	0.00	0.00	0.00	0.00	0.00
9db73460b06c7e6d43a54397337bd973	0.00	0.00	0.00	0.00	0.00	0.00
f9aec5ee543c4d552e6512da84c210f1	0.00	0.00	0.00	0.00	0.00	0.00
ce0a3c9e68edbd577c19c89b8645fcfe	0.00	0.00	0.00	0.00	0.00	0.00
ea4049ac90516f7b2f0b687cb57f7bac	0.00	0.00	0.00	0.00	0.00	0.00
3d040dce82ef66a2583dd8a466b2d3c9	0.00	0.00	0.00	0.00	0.00	0.00
5b4e5baf03b6d8c915a42d6154015b13	0.00	0.00	0.00	0.00	0.00	0.00
4726fc66e73c5cbf91f4949023af2f8e	0.00	0.00	0.00	0.00	0.00	0.00
6c81d83e6348820af68af4715df6a41b	0.00	0.00	0.00	0.00	0.00	0.00
2bb32011ba790f9d9d7fce827a763027	0.00	0.00	0.00	0.00	0.00	0.00
a3a66caac59d3cbab1de061b670e4632	0.00	0.00	0.00	0.00	0.00	0.00
46aca1fa932c332d4727594b44172a57	0.00	0.00	0.00	0.00	0.00	0.00
e9d391ebedf40f23c7a2f65d9054de06	0.00	0.00	0.00	0.00	0.00	0.00
4fff1daf767436f118b740f4931e401f	0.00	0.00	0.00	0.00	0.00	0.00
055a41b4f718a494d8fc3ceebc10adcd	0.00	0.00	0.00	0.00	0.00	0.00
ed05801ee9fdedda3eed5743ceb299d4	0.00	0.00	0.00	0.00	0.00	0.00
b5f6504effadcd277d8d41f82c026436	0.00	0.00	0.00	0.00	0.00	0.00
d955f579ec8454657d7969cdd0af660e	0.00	0.00	0.00	0.00	0.00	0.00
af9b53cb53d341984dcca2e50b67b870	0.00	0.00	0.00	0.00	0.00	0.00
4b215e9d79afe1d781b58b329f37f2f2	0.00	0.00	0.00	0.00	0.00	0.00
a2098c2ea4aeb1551407655177b7373c	0.00	0.00	0.00	0.00	0.00	0.00
59dde47c3364bd103724395d7a601904	0.00	0.00	0.00	0.00	0.00	0.00
eac6aaec6caed72199c8f5e8e694676a	0.00	0.00	0.00	0.00	0.00	0.00
a930d77e367cf0f62b7855d589a11dd4	0.00	0.00	0.00	0.00	0.00	0.00
d6351506b7d0d1eb43bdb2d7d5a26328	0.00	0.00	0.00	0.00	0.00	0.00
7d06ea641176bf4bbc5781aab52c64a1	0.00	0.00	0.00	0.00	0.00	0.00
fb071855da323603d825b39a39c916e2	0.00	0.00	0.00	0.00	0.00	0.00
319b6bb357296a37b9742abe1550b254	0.00	0.00	0.00	0.00	0.00	0.00
44a9e497235ce15e265c746152e33e07	0.00	0.00	0.00	0.00	0.00	0.00
9c18d06ade31d342c677cdb5d0514393	0.00	0.00	0.00	0.00	0.00	0.00

A5	A6	B3	B4	B5	B6	B7	B8	A7
788.00	82.00	0.00	0.00	0.00	0.00	0.00	0.00	0.00
3096.00	16650.00	30732.00	33150.00	12440.00	33187.00	34135.00	32835.00	30704.00
5.00	8.00	6.00	79.00	0.00	4.00	0.00	0.00	33.00
1390.00	6590.00	14642.00	12085.00	4395.00	11865.00	9513.00	10315.00	14071.00
619.00	4501.00	8306.00	6937.00	4322.00	7416.00	9342.00	8841.00	7387.00
18.00	26464.00	29.00	0.00	0.00	0.00	0.00	0.00	0.00
17901.00	0.00	0.00	0.00	0.00	0.00	0.00	0.00	7.00
602.00	3965.00	6110.00	4580.00	2417.00	5719.00	7097.00	7546.00	4449.00
0.00	0.00	11.00	18.00	0.00	15.00	13.00	21.00	17.00
428.00	2811.00	6282.00	5407.00	955.00	5791.00	3449.00	6029.00	5351.00
11476.00	0.00	0.00	0.00	0.00	0.00	0.00	0.00	0.00
3251.00	0.00	0.00	3.00	0.00	0.00	0.00	44.00	0.00
21365.00	4.00	0.00	0.00	0.00	0.00	0.00	0.00	0.00
152.00	1117.00	3015.00	1711.00	486.00	2043.00	1630.00	1803.00	2543.00
312.00	1425.00	2313.00	1881.00	1298.00	1997.00	1768.00	2679.00	2359.00
212.00	1264.00	2705.00	2073.00	683.00	2332.00	2548.00	2715.00	2215.00
5700.00	0.00	0.00	0.00	0.00	0.00	0.00	0.00	0.00
168.00	1213.00	1602.00	3100.00	268.00	2101.00	1728.00	1444.00	3001.00
39.00	91.00	31.00	65.00	0.00	24.00	119.00	54.00	44.00
56.00	31.00	0.00	0.00	0.00	0.00	0.00	0.00	0.00
13.00	0.00	0.00	0.00	0.00	0.00	0.00	0.00	0.00
7.00	0.00	0.00	0.00	0.00	0.00	0.00	0.00	0.00
0.00	0.00	0.00	0.00	0.00	0.00	0.00	0.00	0.00
88.00	711.00	1003.00	747.00	355.00	835.00	548.00	1256.00	541.00
0.00	3.00	0.00	0.00	0.00	0.00	0.00	6.00	0.00
0.00	0.00	0.00	0.00	0.00	0.00	0.00	0.00	0.00
0.00	13.00	0.00	0.00	0.00	0.00	0.00	0.00	0.00
133.00	624.00	379.00	1295.00	731.00	75.00	605.00	647.00	719.00
0.00	5544.00	0.00	0.00	0.00	0.00	0.00	0.00	0.00
0.00	0.00	0.00	0.00	0.00	0.00	0.00	0.00	0.00
0.00	0.00	0.00	0.00	0.00	0.00	0.00	0.00	0.00
0.00	0.00	0.00	0.00	0.00	0.00	0.00	0.00	0.00
615.00	0.00	0.00	0.00	0.00	0.00	0.00	0.00	0.00
0.00	0.00	0.00	0.00	0.00	0.00	0.00	0.00	0.00
0.00	0.00	0.00	0.00	0.00	0.00	0.00	0.00	0.00

0.00	0.00	0.00	0.00	0.00	0.00	0.00	0.00	0.00
0.00	0.00	0.00	0.00	0.00	0.00	0.00	0.00	0.00
0.00	0.00	0.00	0.00	0.00	0.00	0.00	0.00	0.00
0.00	0.00	0.00	0.00	0.00	0.00	0.00	0.00	0.00
0.00	0.00	0.00	0.00	0.00	0.00	0.00	0.00	0.00
0.00	0.00	0.00	0.00	0.00	0.00	0.00	0.00	0.00
0.00	0.00	0.00	0.00	0.00	0.00	0.00	0.00	0.00
0.00	0.00	8.00	0.00	0.00	0.00	0.00	0.00	25.00
0.00	0.00	0.00	0.00	0.00	0.00	0.00	0.00	0.00
0.00	0.00	0.00	0.00	0.00	0.00	0.00	0.00	0.00
0.00	0.00	0.00	0.00	0.00	0.00	0.00	0.00	0.00
17.00	56.00	280.00	189.00	0.00	161.00	204.00	220.00	542.00
0.00	0.00	0.00	0.00	215.00	0.00	0.00	0.00	0.00
0.00	0.00	0.00	4.00	0.00	12.00	0.00	0.00	0.00
0.00	0.00	0.00	0.00	0.00	0.00	0.00	0.00	0.00
0.00	0.00	0.00	0.00	0.00	0.00	0.00	0.00	0.00
19.00	103.00	231.00	194.00	131.00	312.00	73.00	445.00	320.00
0.00	0.00	0.00	0.00	0.00	0.00	0.00	0.00	0.00
23.00	109.00	228.00	229.00	97.00	146.00	224.00	204.00	77.00
13.00	116.00	252.00	193.00	0.00	248.00	229.00	353.00	342.00
0.00	0.00	0.00	0.00	0.00	0.00	0.00	0.00	0.00
0.00	0.00	0.00	0.00	0.00	0.00	0.00	0.00	0.00
0.00	0.00	0.00	0.00	0.00	0.00	0.00	0.00	0.00
0.00	0.00	0.00	0.00	0.00	0.00	0.00	0.00	0.00
0.00	0.00	0.00	0.00	0.00	0.00	0.00	10.00	0.00
0.00	0.00	0.00	0.00	0.00	0.00	0.00	0.00	0.00
0.00	0.00	0.00	0.00	0.00	0.00	0.00	0.00	0.00
0.00	0.00	0.00	0.00	0.00	0.00	0.00	0.00	0.00
23.00	189.00	70.00	424.00	0.00	15.00	293.00	204.00	69.00
18.00	99.00	110.00	224.00	65.00	170.00	92.00	97.00	67.00
0.00	0.00	0.00	0.00	0.00	0.00	0.00	0.00	0.00
0.00	0.00	0.00	0.00	0.00	0.00	0.00	0.00	0.00
0.00	0.00	0.00	0.00	0.00	0.00	0.00	0.00	0.00
0.00	89.00	70.00	314.00	0.00	0.00	279.00	233.00	221.00
0.00	0.00	0.00	0.00	0.00	0.00	0.00	0.00	0.00
0.00	0.00	0.00	0.00	0.00	0.00	0.00	0.00	0.00
0.00	0.00	0.00	0.00	0.00	0.00	0.00	0.00	0.00

0.00	0.00	0.00	0.00	0.00	0.00	0.00	0.00	0.00
0.00	0.00	0.00	0.00	0.00	0.00	0.00	0.00	0.00
0.00	0.00	0.00	0.00	0.00	0.00	0.00	0.00	0.00
0.00	0.00	0.00	0.00	0.00	0.00	0.00	0.00	0.00
0.00	0.00	0.00	0.00	0.00	0.00	0.00	0.00	0.00
0.00	0.00	0.00	0.00	0.00	0.00	0.00	0.00	0.00
0.00	0.00	0.00	0.00	0.00	0.00	0.00	0.00	0.00
0.00	0.00	0.00	0.00	0.00	0.00	0.00	0.00	0.00
0.00	0.00	0.00	0.00	0.00	0.00	0.00	0.00	0.00
0.00	0.00	0.00	0.00	0.00	0.00	0.00	0.00	0.00
0.00	0.00	0.00	0.00	0.00	0.00	0.00	0.00	0.00
22.00	52.00	76.00	98.00	53.00	124.00	215.00	71.00	196.00
0.00	0.00	2.00	0.00	0.00	0.00	0.00	8.00	0.00
0.00	10.00	5.00	47.00	0.00	0.00	0.00	0.00	0.00
0.00	0.00	0.00	0.00	0.00	0.00	0.00	0.00	0.00
0.00	0.00	0.00	0.00	0.00	0.00	0.00	0.00	0.00
5.00	47.00	297.00	74.00	0.00	52.00	7.00	87.00	6.00
13.00	111.00	33.00	52.00	208.00	32.00	29.00	163.00	79.00
0.00	0.00	0.00	0.00	0.00	0.00	0.00	0.00	0.00
0.00	0.00	0.00	0.00	0.00	0.00	0.00	0.00	0.00
23.00	22.00	8.00	16.00	0.00	17.00	0.00	11.00	26.00
0.00	0.00	0.00	2.00	0.00	6.00	0.00	0.00	0.00
0.00	0.00	0.00	0.00	0.00	0.00	0.00	0.00	0.00
0.00	0.00	0.00	0.00	0.00	0.00	0.00	0.00	0.00
0.00	0.00	0.00	0.00	0.00	0.00	0.00	0.00	0.00
0.00	14.00	0.00	0.00	0.00	0.00	0.00	0.00	0.00
0.00	0.00	0.00	0.00	0.00	0.00	0.00	0.00	0.00
0.00	13.00	3.00	26.00	95.00	3.00	15.00	31.00	0.00
4.00	6.00	0.00	10.00	0.00	5.00	0.00	376.00	0.00
0.00	0.00	0.00	0.00	0.00	0.00	0.00	0.00	0.00
7.00	64.00	110.00	48.00	0.00	198.00	203.00	46.00	19.00
0.00	0.00	2.00	0.00	0.00	0.00	0.00	0.00	0.00
0.00	22.00	0.00	0.00	0.00	0.00	0.00	0.00	0.00
0.00	0.00	0.00	0.00	0.00	0.00	0.00	0.00	0.00
0.00	72.00	135.00	103.00	0.00	75.00	20.00	0.00	91.00
0.00	0.00	0.00	0.00	0.00	0.00	0.00	0.00	0.00
0.00	0.00	0.00	0.00	0.00	0.00	0.00	0.00	0.00

0.00	0.00	0.00	0.00	0.00	0.00	0.00	0.00	0.00
0.00	0.00	0.00	0.00	0.00	0.00	0.00	0.00	0.00
0.00	0.00	0.00	0.00	0.00	0.00	0.00	0.00	0.00
0.00	0.00	0.00	0.00	0.00	0.00	0.00	0.00	0.00
0.00	0.00	0.00	0.00	0.00	0.00	0.00	0.00	0.00
0.00	0.00	0.00	0.00	0.00	0.00	0.00	0.00	0.00
0.00	0.00	0.00	0.00	0.00	0.00	0.00	0.00	0.00
0.00	0.00	0.00	0.00	0.00	0.00	0.00	0.00	0.00
0.00	0.00	0.00	0.00	0.00	0.00	0.00	0.00	0.00
0.00	0.00	0.00	0.00	0.00	0.00	0.00	0.00	0.00
0.00	0.00	0.00	0.00	0.00	0.00	0.00	0.00	0.00
0.00	0.00	0.00	0.00	0.00	0.00	0.00	0.00	0.00
0.00	0.00	0.00	0.00	0.00	0.00	0.00	0.00	0.00
0.00	0.00	0.00	0.00	0.00	0.00	0.00	0.00	0.00
7.00	96.00	14.00	78.00	223.00	8.00	69.00	21.00	97.00
0.00	0.00	0.00	0.00	0.00	0.00	0.00	0.00	0.00
0.00	0.00	0.00	0.00	0.00	0.00	0.00	0.00	0.00
0.00	0.00	0.00	0.00	0.00	0.00	0.00	0.00	0.00
0.00	0.00	0.00	0.00	0.00	0.00	0.00	0.00	0.00
0.00	0.00	0.00	0.00	0.00	0.00	0.00	0.00	0.00
0.00	0.00	0.00	0.00	0.00	0.00	0.00	0.00	0.00
0.00	0.00	0.00	0.00	0.00	0.00	0.00	0.00	0.00
0.00	0.00	0.00	0.00	0.00	0.00	0.00	0.00	0.00
0.00	0.00	0.00	0.00	0.00	0.00	0.00	0.00	0.00
0.00	0.00	0.00	0.00	0.00	0.00	0.00	0.00	0.00
0.00	0.00	0.00	0.00	0.00	0.00	0.00	0.00	0.00
0.00	0.00	0.00	0.00	0.00	0.00	0.00	0.00	0.00
0.00	0.00	0.00	11.00	0.00	0.00	0.00	14.00	0.00
0.00	0.00	0.00	0.00	0.00	0.00	0.00	0.00	0.00
0.00	0.00	0.00	0.00	0.00	0.00	0.00	0.00	0.00
0.00	0.00	26.00	32.00	0.00	0.00	0.00	0.00	0.00
0.00	0.00	0.00	0.00	0.00	0.00	0.00	0.00	0.00
0.00	0.00	0.00	0.00	0.00	0.00	0.00	0.00	0.00
0.00	24.00	38.00	211.00	0.00	0.00	127.00	0.00	29.00
0.00	0.00	0.00	0.00	0.00	0.00	0.00	0.00	0.00
0.00	0.00	0.00	0.00	0.00	0.00	0.00	0.00	0.00
0.00	0.00	0.00	0.00	0.00	0.00	0.00	0.00	0.00

0.00	0.00	0.00	0.00	0.00	0.00	0.00	0.00	0.00
0.00	0.00	0.00	0.00	0.00	0.00	0.00	0.00	0.00
0.00	0.00	0.00	0.00	0.00	0.00	0.00	0.00	0.00
0.00	0.00	0.00	0.00	0.00	0.00	0.00	0.00	0.00
0.00	0.00	0.00	0.00	0.00	0.00	0.00	0.00	0.00
0.00	0.00	0.00	0.00	0.00	0.00	0.00	0.00	0.00
0.00	0.00	0.00	0.00	0.00	0.00	0.00	0.00	0.00
9.00	10.00	94.00	6.00	0.00	23.00	0.00	33.00	10.00
0.00	0.00	0.00	0.00	0.00	0.00	0.00	0.00	0.00
0.00	0.00	3.00	7.00	0.00	0.00	3.00	0.00	6.00
0.00	0.00	0.00	0.00	0.00	0.00	0.00	0.00	0.00
0.00	0.00	0.00	0.00	0.00	0.00	0.00	0.00	0.00
0.00	0.00	0.00	0.00	0.00	0.00	0.00	0.00	0.00
0.00	0.00	0.00	0.00	0.00	0.00	0.00	0.00	0.00
2.00	5.00	44.00	76.00	0.00	5.00	25.00	40.00	38.00
0.00	0.00	0.00	0.00	0.00	0.00	0.00	0.00	0.00
0.00	0.00	0.00	0.00	0.00	0.00	30.00	0.00	0.00
0.00	0.00	0.00	0.00	0.00	0.00	0.00	0.00	0.00
0.00	0.00	0.00	0.00	0.00	0.00	0.00	0.00	0.00
0.00	0.00	0.00	0.00	0.00	0.00	373.00	0.00	17.00
0.00	0.00	0.00	0.00	0.00	0.00	0.00	0.00	0.00
0.00	0.00	0.00	0.00	0.00	0.00	0.00	0.00	0.00
0.00	0.00	0.00	0.00	0.00	0.00	0.00	0.00	0.00
0.00	18.00	49.00	7.00	0.00	0.00	148.00	131.00	32.00
0.00	0.00	0.00	0.00	22.00	0.00	0.00	0.00	0.00
439.00	0.00	0.00	0.00	0.00	0.00	0.00	0.00	0.00
0.00	0.00	0.00	0.00	0.00	0.00	0.00	0.00	0.00
0.00	0.00	0.00	21.00	0.00	0.00	0.00	49.00	0.00
0.00	0.00	0.00	0.00	0.00	0.00	0.00	0.00	0.00
0.00	0.00	0.00	0.00	0.00	0.00	0.00	0.00	0.00
0.00	0.00	0.00	0.00	0.00	0.00	0.00	0.00	0.00
0.00	0.00	0.00	0.00	0.00	0.00	0.00	0.00	0.00
0.00	0.00	0.00	0.00	0.00	0.00	0.00	0.00	0.00
0.00	0.00	0.00	0.00	0.00	0.00	0.00	0.00	0.00
0.00	0.00	0.00	0.00	0.00	0.00	0.00	0.00	0.00
0.00	0.00	0.00	0.00	0.00	0.00	0.00	0.00	0.00
0.00	0.00	0.00	0.00	0.00	0.00	0.00	0.00	0.00

0.00	0.00	0.00	0.00	0.00	0.00	263.00	10.00	0.00
0.00	0.00	9.00	19.00	130.00	22.00	0.00	39.00	26.00
0.00	24.00	13.00	0.00	0.00	56.00	55.00	12.00	0.00
0.00	0.00	0.00	0.00	0.00	0.00	0.00	0.00	0.00
0.00	0.00	0.00	0.00	0.00	0.00	0.00	0.00	0.00
0.00	0.00	0.00	0.00	0.00	0.00	0.00	0.00	0.00
0.00	692.00	0.00	0.00	0.00	0.00	0.00	0.00	0.00
0.00	0.00	0.00	0.00	0.00	0.00	0.00	0.00	0.00
0.00	0.00	0.00	0.00	0.00	0.00	0.00	0.00	0.00
0.00	0.00	0.00	0.00	0.00	0.00	0.00	0.00	0.00
0.00	56.00	52.00	76.00	0.00	39.00	0.00	102.00	21.00
0.00	0.00	0.00	0.00	0.00	0.00	0.00	0.00	0.00
6.00	5.00	0.00	0.00	0.00	0.00	227.00	2.00	0.00
0.00	0.00	0.00	0.00	0.00	0.00	0.00	0.00	0.00
0.00	0.00	0.00	0.00	0.00	0.00	0.00	0.00	0.00
0.00	0.00	0.00	0.00	0.00	0.00	0.00	0.00	0.00
12.00	39.00	0.00	64.00	96.00	19.00	0.00	0.00	27.00
0.00	0.00	0.00	0.00	0.00	0.00	0.00	0.00	0.00
0.00	0.00	0.00	0.00	0.00	0.00	0.00	0.00	0.00
0.00	0.00	0.00	0.00	0.00	0.00	0.00	0.00	0.00
0.00	0.00	0.00	0.00	0.00	0.00	0.00	0.00	0.00
0.00	0.00	0.00	0.00	0.00	0.00	0.00	0.00	0.00
4.00	3.00	38.00	57.00	0.00	15.00	10.00	138.00	11.00
0.00	0.00	0.00	0.00	0.00	0.00	0.00	0.00	0.00
0.00	0.00	0.00	0.00	0.00	0.00	0.00	0.00	0.00
3.00	3.00	29.00	11.00	0.00	30.00	126.00	0.00	28.00
0.00	0.00	0.00	0.00	0.00	0.00	0.00	0.00	0.00
0.00	0.00	0.00	0.00	0.00	0.00	0.00	0.00	0.00
0.00	0.00	0.00	197.00	0.00	0.00	34.00	0.00	0.00
0.00	0.00	0.00	0.00	0.00	0.00	0.00	0.00	0.00
0.00	0.00	0.00	0.00	0.00	0.00	0.00	0.00	0.00
0.00	0.00	0.00	0.00	0.00	0.00	0.00	0.00	0.00
0.00	0.00	0.00	0.00	0.00	0.00	0.00	0.00	0.00
20.00	33.00	22.00	24.00	0.00	18.00	25.00	0.00	31.00
0.00	0.00	0.00	0.00	0.00	0.00	0.00	0.00	0.00
0.00	0.00	0.00	0.00	0.00	0.00	0.00	0.00	0.00
0.00	0.00	0.00	0.00	0.00	0.00	0.00	0.00	0.00

0.00	0.00	0.00	0.00	0.00	0.00	0.00	0.00	0.00
0.00	0.00	0.00	0.00	0.00	0.00	0.00	0.00	0.00
0.00	0.00	0.00	0.00	0.00	0.00	0.00	0.00	0.00
16.00	22.00	8.00	4.00	0.00	16.00	7.00	27.00	7.00
0.00	0.00	0.00	0.00	0.00	0.00	0.00	0.00	0.00
0.00	0.00	0.00	0.00	0.00	0.00	0.00	0.00	0.00
0.00	0.00	27.00	0.00	0.00	33.00	0.00	0.00	64.00
0.00	0.00	0.00	0.00	0.00	0.00	0.00	0.00	0.00
0.00	0.00	0.00	0.00	0.00	0.00	0.00	0.00	0.00
0.00	0.00	0.00	0.00	0.00	0.00	0.00	0.00	0.00
0.00	0.00	0.00	0.00	0.00	6.00	9.00	10.00	7.00
0.00	0.00	0.00	0.00	0.00	0.00	0.00	0.00	0.00
0.00	0.00	0.00	0.00	0.00	0.00	0.00	0.00	0.00
0.00	0.00	0.00	0.00	0.00	0.00	0.00	0.00	0.00
0.00	0.00	0.00	0.00	0.00	0.00	0.00	0.00	0.00
0.00	0.00	0.00	0.00	0.00	0.00	0.00	0.00	0.00
0.00	0.00	0.00	0.00	0.00	0.00	0.00	0.00	0.00
0.00	0.00	0.00	0.00	0.00	0.00	0.00	0.00	0.00
0.00	0.00	0.00	0.00	0.00	0.00	0.00	0.00	0.00
0.00	0.00	0.00	0.00	0.00	0.00	0.00	0.00	0.00
0.00	0.00	0.00	0.00	0.00	0.00	0.00	0.00	0.00
0.00	29.00	0.00	0.00	0.00	0.00	35.00	49.00	63.00
0.00	0.00	0.00	0.00	0.00	0.00	0.00	0.00	0.00
0.00	0.00	0.00	0.00	0.00	0.00	0.00	0.00	0.00
0.00	0.00	0.00	0.00	0.00	0.00	0.00	0.00	0.00
0.00	0.00	0.00	0.00	0.00	0.00	0.00	0.00	0.00
0.00	0.00	0.00	0.00	0.00	0.00	269.00	23.00	0.00
301.00	0.00	0.00	0.00	0.00	0.00	0.00	0.00	0.00
0.00	0.00	0.00	0.00	0.00	0.00	0.00	0.00	0.00
12.00	4.00	0.00	9.00	0.00	5.00	8.00	0.00	0.00
0.00	0.00	0.00	0.00	0.00	0.00	0.00	0.00	0.00
0.00	0.00	0.00	0.00	0.00	0.00	0.00	0.00	0.00
0.00	0.00	0.00	0.00	0.00	0.00	0.00	0.00	0.00
0.00	0.00	0.00	0.00	0.00	0.00	0.00	0.00	0.00
0.00	0.00	0.00	0.00	0.00	0.00	0.00	0.00	0.00
0.00	0.00	0.00	0.00	0.00	0.00	0.00	0.00	0.00
0.00	0.00	0.00	0.00	0.00	0.00	0.00	0.00	0.00

4.00	4.00	69.00	0.00	0.00	5.00	7.00	0.00	31.00
0.00	0.00	0.00	0.00	0.00	0.00	242.00	110.00	0.00
0.00	0.00	0.00	0.00	0.00	0.00	0.00	0.00	0.00
0.00	5.00	0.00	0.00	0.00	0.00	381.00	0.00	0.00
0.00	0.00	0.00	0.00	0.00	0.00	0.00	0.00	0.00
0.00	0.00	0.00	0.00	0.00	0.00	0.00	0.00	0.00
0.00	10.00	0.00	0.00	0.00	0.00	0.00	7.00	0.00
0.00	0.00	0.00	0.00	0.00	0.00	0.00	0.00	0.00
0.00	0.00	0.00	0.00	0.00	0.00	0.00	0.00	0.00
0.00	0.00	33.00	0.00	0.00	50.00	16.00	0.00	37.00
0.00	0.00	0.00	0.00	0.00	0.00	0.00	0.00	0.00
0.00	0.00	0.00	0.00	0.00	0.00	0.00	0.00	0.00
0.00	0.00	0.00	0.00	0.00	0.00	0.00	0.00	0.00
0.00	24.00	0.00	52.00	0.00	0.00	4.00	36.00	0.00
0.00	0.00	0.00	0.00	0.00	0.00	0.00	0.00	0.00
4.00	5.00	8.00	0.00	0.00	11.00	0.00	0.00	0.00
0.00	0.00	0.00	0.00	0.00	0.00	0.00	0.00	0.00
0.00	0.00	0.00	0.00	0.00	0.00	0.00	0.00	0.00
0.00	0.00	0.00	0.00	0.00	0.00	0.00	36.00	8.00
0.00	0.00	0.00	0.00	0.00	0.00	0.00	0.00	0.00
0.00	0.00	0.00	0.00	0.00	0.00	0.00	0.00	0.00
0.00	0.00	0.00	0.00	0.00	0.00	0.00	0.00	0.00
0.00	0.00	0.00	0.00	0.00	0.00	0.00	0.00	0.00
0.00	0.00	22.00	0.00	97.00	29.00	0.00	0.00	0.00
0.00	0.00	0.00	0.00	0.00	0.00	0.00	0.00	0.00
0.00	0.00	35.00	0.00	0.00	0.00	0.00	0.00	0.00
0.00	0.00	0.00	0.00	0.00	0.00	0.00	0.00	0.00
0.00	0.00	0.00	0.00	0.00	0.00	0.00	0.00	0.00
0.00	0.00	0.00	0.00	0.00	0.00	0.00	0.00	0.00
0.00	2.00	0.00	0.00	0.00	0.00	3.00	2.00	0.00
0.00	0.00	0.00	0.00	0.00	0.00	0.00	0.00	0.00
0.00	0.00	0.00	0.00	0.00	0.00	0.00	0.00	0.00
0.00	0.00	0.00	0.00	0.00	0.00	0.00	0.00	0.00
0.00	16.00	0.00	0.00	0.00	0.00	0.00	0.00	0.00
0.00	0.00	0.00	0.00	0.00	0.00	0.00	0.00	0.00
0.00	0.00	0.00	0.00	0.00	0.00	0.00	19.00	0.00
0.00	0.00	14.00	6.00	0.00	0.00	0.00	23.00	3.00

0.00	0.00	0.00	0.00	0.00	0.00	0.00	0.00	0.00
0.00	0.00	0.00	0.00	0.00	0.00	0.00	0.00	0.00
0.00	0.00	0.00	0.00	0.00	0.00	0.00	0.00	0.00
0.00	0.00	0.00	0.00	0.00	0.00	0.00	0.00	0.00
0.00	0.00	0.00	0.00	0.00	0.00	0.00	0.00	0.00
0.00	0.00	0.00	0.00	0.00	0.00	0.00	0.00	0.00
0.00	0.00	0.00	0.00	0.00	0.00	0.00	0.00	0.00
0.00	14.00	3.00	115.00	0.00	0.00	0.00	0.00	0.00
0.00	0.00	0.00	0.00	0.00	0.00	0.00	0.00	0.00
0.00	0.00	0.00	0.00	0.00	0.00	0.00	0.00	0.00
0.00	0.00	0.00	0.00	0.00	0.00	0.00	0.00	0.00
0.00	0.00	0.00	0.00	0.00	0.00	0.00	0.00	0.00
0.00	0.00	0.00	0.00	0.00	0.00	0.00	0.00	0.00
6.00	9.00	8.00	9.00	0.00	4.00	10.00	5.00	13.00
0.00	0.00	0.00	0.00	0.00	0.00	0.00	0.00	0.00
0.00	0.00	0.00	0.00	0.00	0.00	0.00	0.00	0.00
0.00	0.00	0.00	0.00	0.00	0.00	0.00	0.00	0.00
0.00	0.00	0.00	0.00	0.00	0.00	0.00	0.00	0.00
0.00	0.00	0.00	0.00	0.00	0.00	0.00	0.00	0.00
0.00	0.00	0.00	0.00	0.00	0.00	0.00	0.00	0.00
0.00	0.00	0.00	0.00	0.00	0.00	0.00	0.00	0.00
0.00	0.00	0.00	0.00	0.00	0.00	0.00	0.00	0.00
0.00	315.00	0.00	0.00	0.00	0.00	0.00	0.00	0.00
204.00	0.00	0.00	0.00	0.00	0.00	0.00	0.00	0.00
0.00	0.00	0.00	0.00	0.00	0.00	0.00	0.00	0.00
0.00	0.00	0.00	0.00	0.00	0.00	0.00	0.00	0.00
0.00	0.00	0.00	0.00	0.00	0.00	0.00	0.00	0.00
0.00	0.00	0.00	0.00	0.00	0.00	0.00	0.00	0.00
0.00	284.00	0.00	0.00	0.00	0.00	0.00	0.00	0.00
0.00	0.00	0.00	0.00	0.00	0.00	0.00	0.00	0.00
0.00	0.00	0.00	0.00	0.00	0.00	0.00	0.00	0.00
0.00	0.00	0.00	0.00	0.00	0.00	0.00	0.00	0.00
0.00	300.00	0.00	0.00	0.00	0.00	0.00	0.00	0.00
0.00	0.00	0.00	0.00	0.00	0.00	0.00	0.00	0.00
0.00	8.00	23.00	10.00	0.00	18.00	0.00	0.00	0.00
0.00	0.00	0.00	0.00	0.00	0.00	0.00	0.00	0.00
0.00	0.00	0.00	0.00	0.00	0.00	0.00	0.00	0.00

0.00	291.00	0.00	0.00	0.00	0.00	0.00	0.00	0.00
0.00	0.00	0.00	0.00	0.00	0.00	0.00	0.00	0.00
0.00	0.00	0.00	0.00	0.00	0.00	0.00	0.00	0.00
0.00	0.00	0.00	0.00	0.00	0.00	0.00	0.00	0.00
0.00	0.00	0.00	0.00	0.00	0.00	0.00	0.00	0.00
0.00	0.00	0.00	0.00	0.00	0.00	0.00	0.00	0.00
0.00	0.00	0.00	0.00	0.00	0.00	0.00	0.00	0.00
0.00	0.00	0.00	0.00	0.00	0.00	0.00	0.00	0.00
0.00	0.00	0.00	0.00	0.00	0.00	0.00	0.00	0.00
0.00	0.00	0.00	0.00	0.00	0.00	0.00	0.00	0.00
0.00	0.00	0.00	0.00	0.00	0.00	0.00	0.00	0.00
0.00	0.00	0.00	0.00	0.00	0.00	0.00	0.00	0.00
0.00	0.00	0.00	0.00	0.00	0.00	0.00	0.00	0.00
0.00	0.00	0.00	0.00	0.00	0.00	0.00	0.00	0.00
0.00	0.00	0.00	0.00	0.00	0.00	0.00	0.00	0.00
0.00	0.00	0.00	0.00	0.00	0.00	0.00	0.00	0.00
0.00	0.00	0.00	0.00	0.00	0.00	0.00	0.00	0.00
0.00	0.00	0.00	0.00	0.00	8.00	5.00	0.00	0.00
0.00	0.00	0.00	0.00	0.00	0.00	0.00	0.00	0.00
0.00	0.00	0.00	0.00	0.00	0.00	0.00	0.00	0.00
0.00	0.00	0.00	0.00	130.00	0.00	0.00	0.00	0.00
6.00	0.00	0.00	0.00	0.00	0.00	0.00	0.00	59.00
0.00	0.00	0.00	0.00	0.00	0.00	0.00	0.00	0.00
19.00	0.00	0.00	0.00	0.00	0.00	19.00	0.00	0.00
0.00	0.00	0.00	0.00	0.00	0.00	0.00	0.00	0.00
0.00	0.00	0.00	0.00	0.00	0.00	0.00	0.00	0.00
0.00	0.00	0.00	0.00	0.00	0.00	0.00	0.00	0.00
0.00	0.00	0.00	0.00	0.00	37.00	0.00	20.00	0.00
0.00	0.00	0.00	0.00	0.00	0.00	0.00	0.00	0.00
0.00	0.00	0.00	14.00	0.00	55.00	0.00	0.00	0.00
0.00	0.00	0.00	0.00	0.00	0.00	0.00	0.00	0.00
0.00	0.00	0.00	0.00	0.00	0.00	0.00	0.00	0.00
0.00	0.00	0.00	0.00	0.00	0.00	0.00	0.00	0.00
0.00	0.00	0.00	0.00	0.00	0.00	0.00	0.00	0.00
0.00	0.00	0.00	0.00	0.00	0.00	0.00	0.00	0.00
0.00	14.00	0.00	56.00	0.00	0.00	0.00	0.00	0.00
0.00	0.00	0.00	0.00	0.00	0.00	0.00	0.00	0.00

11.00	0.00	0.00	0.00	0.00	0.00	0.00	0.00	0.00
2.00	15.00	0.00	23.00	0.00	11.00	0.00	20.00	0.00
0.00	0.00	13.00	10.00	0.00	12.00	0.00	0.00	0.00
0.00	0.00	0.00	3.00	0.00	0.00	0.00	10.00	0.00
0.00	0.00	0.00	0.00	0.00	0.00	0.00	0.00	0.00
0.00	0.00	0.00	0.00	0.00	0.00	0.00	0.00	0.00
0.00	0.00	0.00	0.00	0.00	0.00	0.00	0.00	0.00
0.00	0.00	0.00	0.00	0.00	0.00	0.00	0.00	0.00
0.00	0.00	0.00	0.00	0.00	0.00	0.00	0.00	0.00
0.00	0.00	0.00	0.00	0.00	0.00	0.00	0.00	0.00
0.00	0.00	0.00	0.00	0.00	0.00	0.00	0.00	0.00
0.00	0.00	0.00	0.00	0.00	0.00	0.00	0.00	0.00
0.00	0.00	0.00	0.00	0.00	0.00	0.00	0.00	0.00
0.00	0.00	0.00	0.00	0.00	0.00	0.00	0.00	0.00
0.00	0.00	0.00	0.00	0.00	0.00	0.00	0.00	0.00
0.00	0.00	0.00	0.00	0.00	0.00	0.00	0.00	0.00
0.00	0.00	0.00	0.00	0.00	0.00	0.00	0.00	0.00
2.00	3.00	4.00	0.00	0.00	0.00	0.00	84.00	34.00
0.00	0.00	0.00	0.00	0.00	0.00	0.00	0.00	0.00
0.00	0.00	0.00	0.00	0.00	0.00	0.00	0.00	0.00
0.00	0.00	0.00	0.00	0.00	0.00	0.00	0.00	0.00
0.00	0.00	0.00	0.00	0.00	0.00	0.00	0.00	0.00
0.00	0.00	0.00	0.00	0.00	0.00	0.00	0.00	0.00
0.00	0.00	0.00	28.00	0.00	0.00	0.00	0.00	0.00
0.00	0.00	0.00	0.00	0.00	0.00	0.00	0.00	0.00
0.00	0.00	0.00	0.00	0.00	0.00	0.00	0.00	0.00
0.00	0.00	0.00	0.00	0.00	0.00	0.00	0.00	0.00
0.00	0.00	0.00	0.00	0.00	0.00	0.00	0.00	0.00
0.00	0.00	0.00	0.00	0.00	0.00	0.00	0.00	0.00
0.00	0.00	0.00	0.00	0.00	0.00	0.00	0.00	0.00
0.00	0.00	0.00	6.00	0.00	10.00	0.00	28.00	10.00
0.00	0.00	0.00	0.00	0.00	0.00	0.00	0.00	0.00
0.00	0.00	0.00	0.00	0.00	0.00	0.00	0.00	0.00
0.00	0.00	0.00	0.00	0.00	0.00	0.00	0.00	0.00
0.00	0.00	0.00	0.00	0.00	0.00	0.00	0.00	0.00
0.00	0.00	0.00	0.00	0.00	0.00	0.00	0.00	0.00
0.00	0.00	0.00	0.00	0.00	0.00	0.00	0.00	0.00

0.00	0.00	0.00	0.00	0.00	0.00	0.00	0.00	0.00
0.00	0.00	0.00	0.00	0.00	0.00	0.00	0.00	0.00
0.00	0.00	0.00	0.00	0.00	0.00	0.00	0.00	0.00
0.00	16.00	3.00	5.00	0.00	0.00	0.00	10.00	0.00
7.00	7.00	0.00	0.00	0.00	0.00	0.00	0.00	5.00
0.00	0.00	0.00	0.00	0.00	0.00	0.00	0.00	0.00
0.00	0.00	0.00	0.00	0.00	0.00	0.00	0.00	0.00
0.00	0.00	0.00	0.00	0.00	0.00	0.00	0.00	0.00
0.00	20.00	0.00	0.00	0.00	0.00	0.00	0.00	0.00
0.00	0.00	0.00	0.00	0.00	0.00	0.00	0.00	0.00
0.00	0.00	0.00	0.00	0.00	0.00	0.00	0.00	0.00
0.00	0.00	0.00	0.00	0.00	0.00	0.00	0.00	0.00
0.00	0.00	0.00	0.00	0.00	0.00	0.00	0.00	0.00
0.00	0.00	0.00	0.00	0.00	0.00	0.00	0.00	0.00
0.00	0.00	0.00	0.00	0.00	0.00	0.00	0.00	0.00
0.00	0.00	0.00	0.00	0.00	0.00	0.00	0.00	0.00
0.00	0.00	0.00	0.00	0.00	0.00	0.00	0.00	0.00
0.00	0.00	0.00	0.00	0.00	0.00	0.00	0.00	0.00
0.00	0.00	0.00	0.00	0.00	0.00	0.00	0.00	0.00
0.00	0.00	0.00	0.00	0.00	0.00	0.00	0.00	0.00
0.00	0.00	0.00	0.00	0.00	0.00	0.00	0.00	0.00
0.00	0.00	0.00	0.00	0.00	0.00	0.00	0.00	0.00
0.00	0.00	0.00	0.00	0.00	0.00	0.00	0.00	0.00
0.00	13.00	0.00	0.00	0.00	0.00	21.00	9.00	31.00
0.00	0.00	0.00	0.00	0.00	0.00	0.00	0.00	0.00
0.00	0.00	0.00	0.00	0.00	0.00	0.00	0.00	0.00
0.00	0.00	0.00	0.00	0.00	0.00	0.00	0.00	0.00
0.00	0.00	0.00	0.00	0.00	0.00	0.00	0.00	0.00
0.00	0.00	0.00	0.00	0.00	0.00	0.00	0.00	0.00
0.00	0.00	0.00	0.00	0.00	0.00	0.00	0.00	0.00
0.00	3.00	0.00	0.00	0.00	0.00	0.00	0.00	0.00
0.00	0.00	0.00	0.00	0.00	0.00	0.00	0.00	0.00
0.00	0.00	0.00	0.00	0.00	0.00	0.00	0.00	0.00
0.00	0.00	0.00	0.00	0.00	0.00	0.00	0.00	0.00
0.00	0.00	0.00	0.00	0.00	0.00	0.00	0.00	0.00
0.00	0.00	0.00	0.00	0.00	0.00	0.00	0.00	0.00
4.00	9.00	0.00	4.00	0.00	0.00	6.00	0.00	0.00

0.00	0.00	0.00	0.00	0.00	0.00	0.00	0.00	0.00
0.00	0.00	0.00	0.00	0.00	0.00	0.00	0.00	0.00
0.00	0.00	0.00	0.00	0.00	0.00	0.00	0.00	0.00
0.00	0.00	0.00	0.00	0.00	0.00	0.00	0.00	0.00
0.00	0.00	0.00	10.00	0.00	0.00	0.00	21.00	0.00
0.00	0.00	0.00	0.00	173.00	0.00	0.00	0.00	0.00
0.00	0.00	0.00	0.00	0.00	0.00	173.00	0.00	0.00
0.00	0.00	0.00	0.00	0.00	0.00	0.00	0.00	0.00
0.00	0.00	0.00	0.00	0.00	0.00	0.00	0.00	0.00
0.00	0.00	0.00	0.00	0.00	0.00	0.00	0.00	0.00
0.00	0.00	0.00	0.00	0.00	0.00	0.00	0.00	0.00
0.00	0.00	0.00	0.00	0.00	0.00	0.00	0.00	0.00
0.00	0.00	0.00	0.00	0.00	0.00	0.00	0.00	0.00
0.00	0.00	0.00	0.00	0.00	0.00	0.00	0.00	0.00
0.00	0.00	0.00	0.00	0.00	0.00	0.00	0.00	0.00
0.00	0.00	0.00	0.00	0.00	0.00	0.00	0.00	0.00
0.00	0.00	0.00	0.00	0.00	0.00	0.00	0.00	0.00
6.00	9.00	0.00	0.00	0.00	0.00	3.00	0.00	0.00
0.00	0.00	0.00	0.00	0.00	0.00	0.00	0.00	0.00
0.00	0.00	0.00	0.00	0.00	0.00	0.00	0.00	0.00
0.00	0.00	0.00	0.00	0.00	0.00	0.00	0.00	0.00
0.00	0.00	0.00	0.00	0.00	0.00	0.00	0.00	0.00
0.00	0.00	0.00	0.00	0.00	0.00	0.00	0.00	0.00
0.00	0.00	0.00	0.00	0.00	0.00	0.00	0.00	0.00
0.00	0.00	0.00	0.00	0.00	0.00	0.00	0.00	0.00
0.00	0.00	0.00	0.00	0.00	0.00	0.00	0.00	0.00
0.00	0.00	0.00	0.00	0.00	0.00	0.00	0.00	0.00
0.00	0.00	0.00	0.00	0.00	0.00	0.00	0.00	0.00
160.00	0.00	0.00	0.00	0.00	0.00	0.00	0.00	0.00
0.00	0.00	0.00	0.00	0.00	0.00	0.00	0.00	0.00
0.00	0.00	0.00	0.00	0.00	0.00	0.00	0.00	0.00
0.00	0.00	0.00	0.00	0.00	0.00	0.00	0.00	0.00
0.00	17.00	0.00	0.00	0.00	0.00	0.00	0.00	0.00
0.00	0.00	0.00	0.00	0.00	0.00	0.00	0.00	0.00
0.00	0.00	0.00	0.00	0.00	0.00	0.00	0.00	0.00
0.00	0.00	0.00	0.00	0.00	0.00	0.00	0.00	0.00
0.00	0.00	0.00	0.00	0.00	0.00	0.00	0.00	0.00

0.00	0.00	0.00	0.00	0.00	0.00	0.00	0.00	0.00
0.00	0.00	0.00	0.00	0.00	0.00	0.00	0.00	0.00
0.00	6.00	12.00	13.00	0.00	0.00	0.00	0.00	10.00
0.00	0.00	0.00	0.00	0.00	0.00	0.00	0.00	0.00
3.00	33.00	0.00	0.00	0.00	21.00	0.00	0.00	10.00
0.00	0.00	0.00	0.00	0.00	0.00	0.00	13.00	0.00
0.00	0.00	0.00	154.00	0.00	0.00	0.00	0.00	0.00
0.00	0.00	0.00	0.00	0.00	0.00	0.00	0.00	0.00
0.00	0.00	0.00	0.00	0.00	0.00	0.00	0.00	0.00
0.00	0.00	0.00	0.00	0.00	0.00	0.00	0.00	0.00
0.00	0.00	0.00	0.00	0.00	0.00	0.00	0.00	0.00
0.00	0.00	0.00	0.00	0.00	0.00	0.00	0.00	0.00
0.00	0.00	0.00	0.00	0.00	0.00	0.00	0.00	0.00
0.00	0.00	0.00	0.00	0.00	0.00	0.00	0.00	0.00
0.00	0.00	0.00	13.00	0.00	0.00	0.00	0.00	0.00
0.00	0.00	0.00	0.00	0.00	0.00	0.00	0.00	0.00
0.00	0.00	0.00	0.00	0.00	0.00	0.00	0.00	0.00
0.00	0.00	0.00	0.00	0.00	0.00	0.00	0.00	0.00
0.00	0.00	0.00	0.00	0.00	0.00	0.00	0.00	0.00
19.00	31.00	0.00	0.00	0.00	0.00	0.00	0.00	0.00
0.00	0.00	0.00	9.00	90.00	0.00	37.00	0.00	0.00
0.00	0.00	0.00	0.00	0.00	0.00	0.00	0.00	0.00
0.00	0.00	0.00	0.00	0.00	0.00	0.00	0.00	0.00
0.00	0.00	0.00	0.00	0.00	0.00	0.00	0.00	0.00
0.00	0.00	0.00	0.00	0.00	0.00	0.00	0.00	0.00
0.00	0.00	0.00	0.00	0.00	0.00	0.00	0.00	0.00
0.00	0.00	0.00	0.00	0.00	0.00	0.00	0.00	0.00
0.00	0.00	0.00	0.00	0.00	0.00	0.00	0.00	0.00
0.00	0.00	0.00	0.00	0.00	0.00	0.00	0.00	0.00
0.00	0.00	0.00	0.00	0.00	10.00	0.00	0.00	0.00
0.00	0.00	0.00	0.00	0.00	0.00	0.00	0.00	0.00
0.00	0.00	0.00	0.00	0.00	0.00	0.00	0.00	0.00
0.00	0.00	0.00	0.00	0.00	0.00	0.00	0.00	0.00
0.00	0.00	0.00	0.00	0.00	0.00	0.00	0.00	0.00
0.00	0.00	0.00	0.00	0.00	0.00	0.00	0.00	0.00
0.00	0.00	0.00	0.00	0.00	0.00	0.00	0.00	0.00
0.00	0.00	0.00	0.00	0.00	0.00	136.00	0.00	0.00

0.00	0.00	0.00	0.00	0.00	0.00	0.00	0.00	0.00
0.00	0.00	0.00	0.00	0.00	0.00	0.00	0.00	0.00
0.00	0.00	0.00	0.00	0.00	0.00	0.00	0.00	0.00
8.00	10.00	0.00	0.00	0.00	0.00	0.00	0.00	0.00
0.00	0.00	0.00	0.00	0.00	0.00	0.00	0.00	0.00
0.00	0.00	0.00	0.00	0.00	0.00	0.00	0.00	0.00
0.00	0.00	0.00	0.00	0.00	0.00	0.00	0.00	0.00
0.00	0.00	0.00	0.00	0.00	0.00	0.00	0.00	0.00
0.00	0.00	0.00	24.00	18.00	19.00	0.00	0.00	0.00
0.00	0.00	0.00	0.00	0.00	0.00	0.00	10.00	0.00
0.00	0.00	0.00	0.00	0.00	0.00	0.00	0.00	0.00
0.00	0.00	0.00	0.00	0.00	0.00	0.00	0.00	0.00
0.00	0.00	0.00	0.00	0.00	0.00	0.00	0.00	0.00
0.00	0.00	0.00	0.00	0.00	0.00	0.00	0.00	0.00
0.00	0.00	0.00	0.00	0.00	0.00	0.00	0.00	0.00
0.00	0.00	0.00	0.00	0.00	0.00	0.00	0.00	0.00
0.00	0.00	0.00	0.00	0.00	0.00	0.00	0.00	0.00
0.00	0.00	0.00	0.00	0.00	0.00	0.00	0.00	0.00
8.00	20.00	0.00	0.00	0.00	12.00	0.00	15.00	3.00
0.00	0.00	0.00	0.00	0.00	0.00	0.00	0.00	0.00
0.00	0.00	0.00	0.00	0.00	0.00	0.00	0.00	0.00
0.00	0.00	0.00	0.00	0.00	0.00	0.00	0.00	0.00
0.00	0.00	0.00	0.00	0.00	0.00	0.00	121.00	0.00
0.00	0.00	0.00	0.00	0.00	0.00	0.00	0.00	0.00
0.00	0.00	0.00	0.00	0.00	0.00	0.00	0.00	0.00
0.00	0.00	0.00	0.00	0.00	0.00	0.00	0.00	0.00
0.00	0.00	0.00	0.00	0.00	0.00	0.00	0.00	0.00
0.00	0.00	0.00	0.00	0.00	0.00	0.00	0.00	0.00
0.00	0.00	0.00	0.00	0.00	0.00	118.00	0.00	0.00
0.00	0.00	0.00	0.00	0.00	0.00	0.00	0.00	0.00
0.00	0.00	0.00	0.00	0.00	0.00	0.00	0.00	0.00
0.00	0.00	0.00	0.00	0.00	0.00	117.00	0.00	0.00
0.00	0.00	0.00	0.00	0.00	0.00	0.00	0.00	0.00
0.00	0.00	0.00	0.00	0.00	0.00	0.00	0.00	0.00
0.00	0.00	0.00	0.00	0.00	0.00	0.00	0.00	0.00
0.00	0.00	0.00	0.00	0.00	0.00	0.00	0.00	0.00
0.00	0.00	0.00	0.00	0.00	0.00	0.00	0.00	0.00

0.00	0.00	0.00	0.00	0.00	0.00	0.00	0.00	0.00
0.00	0.00	0.00	0.00	0.00	0.00	0.00	0.00	0.00
0.00	0.00	0.00	0.00	0.00	0.00	0.00	0.00	0.00
0.00	0.00	0.00	0.00	0.00	0.00	0.00	0.00	0.00
0.00	0.00	0.00	0.00	0.00	0.00	0.00	0.00	0.00
0.00	0.00	0.00	0.00	0.00	0.00	0.00	0.00	0.00
0.00	0.00	0.00	0.00	0.00	0.00	0.00	0.00	0.00
0.00	0.00	0.00	0.00	0.00	0.00	0.00	0.00	0.00
0.00	0.00	0.00	0.00	0.00	0.00	0.00	0.00	0.00
0.00	0.00	0.00	0.00	0.00	0.00	0.00	0.00	0.00
0.00	0.00	0.00	0.00	0.00	0.00	0.00	0.00	0.00
0.00	0.00	0.00	0.00	0.00	0.00	0.00	0.00	0.00
7.00	17.00	0.00	5.00	0.00	0.00	0.00	0.00	0.00
0.00	0.00	0.00	0.00	0.00	0.00	0.00	0.00	0.00
0.00	0.00	0.00	0.00	0.00	0.00	0.00	0.00	0.00
0.00	0.00	0.00	0.00	0.00	0.00	0.00	0.00	0.00
0.00	0.00	0.00	0.00	0.00	0.00	0.00	0.00	0.00
0.00	0.00	0.00	0.00	0.00	0.00	107.00	0.00	0.00
0.00	0.00	0.00	0.00	0.00	0.00	0.00	0.00	0.00
0.00	0.00	0.00	0.00	0.00	0.00	0.00	0.00	0.00
0.00	0.00	0.00	80.00	0.00	0.00	6.00	0.00	6.00
0.00	0.00	0.00	0.00	0.00	0.00	0.00	0.00	0.00
0.00	0.00	0.00	0.00	0.00	0.00	0.00	0.00	0.00
0.00	0.00	9.00	11.00	0.00	0.00	0.00	9.00	0.00
0.00	0.00	0.00	0.00	0.00	0.00	0.00	0.00	0.00
0.00	0.00	0.00	0.00	0.00	0.00	0.00	0.00	0.00
0.00	0.00	0.00	0.00	0.00	0.00	0.00	0.00	0.00
0.00	0.00	0.00	0.00	0.00	0.00	0.00	0.00	0.00
0.00	0.00	0.00	0.00	0.00	0.00	0.00	0.00	0.00
0.00	0.00	0.00	0.00	0.00	5.00	0.00	0.00	0.00
18.00	0.00	0.00	0.00	0.00	0.00	0.00	0.00	13.00
0.00	0.00	0.00	0.00	0.00	0.00	0.00	0.00	0.00
0.00	0.00	0.00	0.00	0.00	0.00	0.00	0.00	0.00
0.00	0.00	0.00	0.00	0.00	0.00	0.00	0.00	0.00
0.00	0.00	0.00	0.00	0.00	0.00	0.00	0.00	0.00
0.00	0.00	0.00	0.00	0.00	0.00	0.00	0.00	0.00
0.00	0.00	0.00	0.00	0.00	0.00	0.00	0.00	0.00

0.00	0.00	0.00	0.00	0.00	0.00	0.00	0.00	0.00
0.00	0.00	0.00	0.00	0.00	0.00	0.00	0.00	0.00
0.00	0.00	0.00	0.00	0.00	0.00	0.00	0.00	0.00
0.00	0.00	0.00	0.00	0.00	0.00	0.00	0.00	0.00
0.00	0.00	0.00	0.00	0.00	0.00	0.00	0.00	0.00
0.00	0.00	0.00	0.00	0.00	0.00	24.00	0.00	0.00
100.00	0.00	0.00	0.00	0.00	0.00	0.00	0.00	0.00
0.00	0.00	0.00	0.00	0.00	0.00	0.00	0.00	0.00
0.00	0.00	0.00	0.00	0.00	0.00	0.00	0.00	0.00
0.00	0.00	0.00	0.00	0.00	0.00	0.00	0.00	0.00
0.00	0.00	0.00	0.00	0.00	0.00	0.00	0.00	0.00
0.00	0.00	0.00	0.00	0.00	0.00	0.00	0.00	0.00
0.00	0.00	0.00	0.00	0.00	0.00	0.00	0.00	0.00
0.00	0.00	0.00	0.00	0.00	0.00	0.00	0.00	0.00
0.00	0.00	0.00	0.00	0.00	0.00	0.00	0.00	0.00
0.00	0.00	0.00	0.00	0.00	0.00	0.00	0.00	0.00
3.00	0.00	17.00	0.00	0.00	0.00	0.00	0.00	0.00
0.00	0.00	0.00	0.00	0.00	0.00	0.00	0.00	0.00
0.00	0.00	0.00	0.00	0.00	0.00	0.00	0.00	0.00
0.00	0.00	0.00	0.00	0.00	0.00	0.00	0.00	0.00
0.00	0.00	0.00	0.00	0.00	0.00	0.00	0.00	0.00
0.00	0.00	0.00	0.00	0.00	0.00	95.00	0.00	0.00
0.00	0.00	0.00	0.00	0.00	0.00	0.00	0.00	0.00
0.00	0.00	0.00	24.00	0.00	0.00	0.00	52.00	0.00
0.00	0.00	0.00	0.00	0.00	0.00	90.00	0.00	0.00
0.00	0.00	0.00	0.00	0.00	0.00	0.00	0.00	0.00
0.00	0.00	0.00	0.00	0.00	0.00	0.00	0.00	0.00
0.00	0.00	0.00	0.00	0.00	0.00	0.00	0.00	0.00
0.00	0.00	0.00	0.00	0.00	0.00	0.00	93.00	0.00
0.00	0.00	0.00	0.00	0.00	0.00	0.00	0.00	0.00
0.00	0.00	0.00	0.00	0.00	0.00	0.00	0.00	0.00
0.00	0.00	0.00	0.00	0.00	0.00	92.00	0.00	0.00
0.00	0.00	0.00	0.00	0.00	0.00	0.00	92.00	0.00
0.00	0.00	0.00	0.00	0.00	0.00	0.00	0.00	0.00
0.00	0.00	0.00	0.00	0.00	0.00	0.00	0.00	0.00
0.00	0.00	0.00	0.00	0.00	0.00	0.00	0.00	0.00
6.00	17.00	4.00	0.00	0.00	0.00	0.00	0.00	0.00

0.00	0.00	0.00	0.00	0.00	0.00	0.00	0.00	0.00
0.00	0.00	0.00	0.00	0.00	0.00	0.00	0.00	0.00
0.00	0.00	0.00	0.00	0.00	7.00	0.00	7.00	0.00
0.00	0.00	0.00	0.00	0.00	0.00	56.00	0.00	0.00
0.00	0.00	0.00	0.00	0.00	0.00	0.00	0.00	0.00
0.00	0.00	0.00	0.00	0.00	0.00	0.00	0.00	0.00
0.00	0.00	0.00	0.00	0.00	0.00	0.00	0.00	0.00
0.00	0.00	0.00	0.00	0.00	0.00	0.00	0.00	0.00
0.00	0.00	0.00	0.00	0.00	0.00	0.00	0.00	0.00
0.00	0.00	0.00	0.00	0.00	0.00	0.00	0.00	0.00
0.00	0.00	0.00	0.00	0.00	0.00	0.00	0.00	0.00
0.00	0.00	0.00	0.00	0.00	0.00	0.00	0.00	0.00
0.00	0.00	0.00	0.00	0.00	0.00	0.00	0.00	0.00
0.00	0.00	0.00	0.00	0.00	0.00	0.00	0.00	0.00
0.00	0.00	0.00	0.00	0.00	0.00	0.00	0.00	0.00
0.00	0.00	0.00	0.00	0.00	0.00	0.00	0.00	0.00
0.00	0.00	0.00	0.00	0.00	0.00	0.00	86.00	0.00
0.00	0.00	0.00	0.00	0.00	0.00	0.00	0.00	0.00
0.00	0.00	0.00	0.00	0.00	0.00	0.00	0.00	0.00
0.00	0.00	0.00	0.00	0.00	0.00	0.00	0.00	0.00
7.00	0.00	0.00	0.00	0.00	0.00	0.00	4.00	0.00
0.00	0.00	0.00	0.00	0.00	0.00	0.00	0.00	0.00
0.00	0.00	0.00	0.00	0.00	0.00	0.00	0.00	0.00
0.00	0.00	0.00	0.00	0.00	0.00	0.00	0.00	0.00
0.00	0.00	0.00	0.00	0.00	0.00	0.00	0.00	0.00
0.00	0.00	0.00	0.00	0.00	0.00	0.00	0.00	0.00
81.00	0.00	0.00	0.00	0.00	0.00	0.00	0.00	0.00
0.00	0.00	0.00	0.00	0.00	0.00	0.00	0.00	0.00
0.00	0.00	0.00	0.00	0.00	0.00	0.00	0.00	0.00
0.00	0.00	0.00	0.00	0.00	0.00	0.00	0.00	0.00
0.00	0.00	0.00	0.00	0.00	0.00	0.00	0.00	0.00
0.00	80.00	0.00	0.00	0.00	0.00	0.00	0.00	0.00
0.00	0.00	0.00	0.00	0.00	0.00	0.00	0.00	0.00
0.00	0.00	0.00	0.00	0.00	0.00	0.00	0.00	0.00
0.00	0.00	0.00	0.00	0.00	0.00	0.00	0.00	0.00
0.00	0.00	0.00	0.00	0.00	0.00	0.00	0.00	0.00
0.00	0.00	0.00	0.00	0.00	0.00	0.00	79.00	0.00

0.00	0.00	0.00	0.00	0.00	0.00	0.00	0.00	0.00
0.00	0.00	0.00	0.00	0.00	0.00	0.00	0.00	0.00
0.00	0.00	0.00	0.00	0.00	0.00	0.00	0.00	0.00
7.00	0.00	0.00	0.00	0.00	0.00	0.00	0.00	0.00
0.00	0.00	0.00	0.00	0.00	0.00	0.00	0.00	0.00
0.00	0.00	0.00	0.00	0.00	0.00	0.00	0.00	0.00
0.00	0.00	0.00	0.00	0.00	0.00	0.00	0.00	0.00
7.00	15.00	0.00	0.00	0.00	0.00	4.00	0.00	0.00
0.00	0.00	0.00	0.00	0.00	0.00	0.00	0.00	0.00
0.00	0.00	0.00	0.00	0.00	0.00	0.00	0.00	0.00
0.00	0.00	0.00	0.00	0.00	0.00	0.00	0.00	0.00
0.00	0.00	0.00	0.00	0.00	0.00	0.00	0.00	0.00
0.00	0.00	0.00	0.00	0.00	0.00	0.00	0.00	0.00
0.00	0.00	0.00	0.00	0.00	0.00	0.00	0.00	0.00
0.00	0.00	0.00	0.00	0.00	0.00	0.00	0.00	0.00
0.00	0.00	0.00	0.00	0.00	0.00	0.00	75.00	0.00
0.00	0.00	0.00	0.00	0.00	0.00	0.00	0.00	0.00
0.00	0.00	0.00	0.00	0.00	0.00	0.00	0.00	0.00
0.00	0.00	0.00	0.00	0.00	0.00	0.00	0.00	0.00
0.00	0.00	7.00	0.00	0.00	0.00	0.00	15.00	0.00
0.00	0.00	4.00	7.00	0.00	0.00	0.00	0.00	0.00
0.00	0.00	0.00	0.00	0.00	0.00	0.00	0.00	0.00
0.00	4.00	8.00	4.00	0.00	4.00	7.00	6.00	12.00
0.00	0.00	0.00	0.00	0.00	0.00	0.00	0.00	0.00
0.00	0.00	0.00	0.00	0.00	0.00	0.00	0.00	0.00
0.00	0.00	0.00	0.00	0.00	0.00	0.00	0.00	0.00
0.00	0.00	0.00	0.00	0.00	0.00	0.00	39.00	0.00
0.00	0.00	0.00	0.00	0.00	0.00	0.00	0.00	0.00
0.00	0.00	0.00	0.00	0.00	0.00	0.00	0.00	0.00
0.00	0.00	0.00	0.00	0.00	0.00	0.00	73.00	0.00
0.00	0.00	0.00	0.00	0.00	0.00	0.00	0.00	0.00
0.00	0.00	0.00	0.00	0.00	0.00	72.00	0.00	0.00
0.00	0.00	0.00	0.00	0.00	0.00	0.00	0.00	0.00
70.00	0.00	0.00	0.00	0.00	0.00	0.00	0.00	0.00
0.00	0.00	0.00	0.00	0.00	0.00	0.00	0.00	0.00
0.00	0.00	0.00	0.00	0.00	0.00	0.00	0.00	0.00
0.00	0.00	0.00	0.00	0.00	0.00	0.00	0.00	0.00

0.00	0.00	0.00	0.00	0.00	0.00	0.00	0.00	0.00
0.00	0.00	0.00	0.00	0.00	0.00	0.00	0.00	0.00
0.00	7.00	8.00	0.00	0.00	0.00	0.00	0.00	0.00
0.00	0.00	0.00	0.00	0.00	0.00	0.00	0.00	0.00
0.00	0.00	0.00	0.00	0.00	0.00	0.00	0.00	0.00
0.00	0.00	0.00	0.00	0.00	0.00	0.00	0.00	0.00
0.00	0.00	0.00	0.00	0.00	0.00	0.00	0.00	0.00
0.00	0.00	0.00	0.00	0.00	0.00	0.00	0.00	0.00
0.00	0.00	0.00	0.00	0.00	0.00	0.00	0.00	0.00
0.00	0.00	0.00	0.00	0.00	0.00	0.00	25.00	0.00
0.00	0.00	0.00	0.00	0.00	0.00	0.00	0.00	0.00
0.00	0.00	0.00	0.00	0.00	0.00	0.00	0.00	0.00
0.00	0.00	0.00	0.00	0.00	0.00	0.00	0.00	0.00
0.00	0.00	0.00	0.00	0.00	0.00	0.00	0.00	0.00
0.00	0.00	0.00	0.00	0.00	0.00	0.00	0.00	0.00
0.00	0.00	0.00	0.00	0.00	0.00	0.00	0.00	0.00
0.00	0.00	0.00	0.00	0.00	0.00	0.00	0.00	0.00
0.00	22.00	0.00	11.00	0.00	0.00	0.00	10.00	0.00
0.00	0.00	0.00	0.00	0.00	0.00	0.00	0.00	0.00
0.00	0.00	0.00	0.00	0.00	0.00	0.00	0.00	0.00
0.00	0.00	0.00	0.00	0.00	0.00	0.00	0.00	0.00
0.00	0.00	0.00	0.00	0.00	0.00	0.00	0.00	0.00
0.00	0.00	0.00	0.00	0.00	0.00	0.00	0.00	0.00
66.00	0.00	0.00	0.00	0.00	0.00	0.00	0.00	0.00
66.00	0.00	0.00	0.00	0.00	0.00	0.00	0.00	0.00
0.00	0.00	0.00	0.00	0.00	0.00	0.00	0.00	0.00
0.00	0.00	0.00	0.00	0.00	0.00	0.00	0.00	0.00
0.00	0.00	0.00	0.00	0.00	0.00	0.00	0.00	0.00
0.00	0.00	0.00	0.00	0.00	0.00	0.00	0.00	0.00
0.00	0.00	0.00	0.00	0.00	0.00	0.00	0.00	0.00
0.00	0.00	0.00	0.00	0.00	0.00	0.00	0.00	0.00
0.00	0.00	0.00	0.00	0.00	0.00	0.00	0.00	0.00
0.00	0.00	0.00	0.00	0.00	0.00	0.00	0.00	0.00
0.00	0.00	0.00	0.00	0.00	0.00	0.00	0.00	0.00
0.00	0.00	29.00	0.00	0.00	0.00	0.00	0.00	0.00
0.00	24.00	0.00	0.00	0.00	0.00	0.00	0.00	0.00
0.00	0.00	0.00	0.00	0.00	0.00	64.00	0.00	0.00

0.00	0.00	0.00	0.00	0.00	0.00	0.00	0.00	0.00
0.00	0.00	0.00	0.00	0.00	0.00	0.00	0.00	0.00
0.00	0.00	0.00	0.00	0.00	0.00	0.00	0.00	0.00
0.00	0.00	0.00	0.00	0.00	0.00	0.00	0.00	0.00
0.00	0.00	0.00	0.00	0.00	0.00	0.00	0.00	0.00
0.00	0.00	0.00	0.00	0.00	0.00	0.00	0.00	0.00
0.00	0.00	0.00	0.00	0.00	0.00	0.00	0.00	0.00
0.00	0.00	0.00	0.00	0.00	0.00	0.00	0.00	0.00
63.00	0.00	0.00	0.00	0.00	0.00	0.00	0.00	0.00
0.00	0.00	0.00	0.00	0.00	0.00	0.00	0.00	0.00
0.00	0.00	0.00	0.00	0.00	0.00	0.00	0.00	0.00
0.00	0.00	0.00	0.00	0.00	0.00	0.00	0.00	0.00
0.00	4.00	4.00	0.00	0.00	2.00	5.00	3.00	0.00
0.00	62.00	0.00	0.00	0.00	0.00	0.00	0.00	0.00
0.00	0.00	0.00	0.00	0.00	0.00	0.00	30.00	32.00
0.00	0.00	0.00	0.00	0.00	0.00	0.00	0.00	0.00
0.00	0.00	0.00	0.00	0.00	0.00	0.00	0.00	0.00
0.00	0.00	0.00	0.00	0.00	0.00	0.00	62.00	0.00
0.00	0.00	0.00	0.00	0.00	0.00	0.00	0.00	0.00
0.00	0.00	0.00	0.00	0.00	0.00	0.00	0.00	0.00
0.00	0.00	0.00	0.00	0.00	0.00	0.00	0.00	0.00
0.00	0.00	0.00	37.00	0.00	0.00	0.00	0.00	0.00
0.00	0.00	0.00	0.00	0.00	0.00	0.00	0.00	0.00
0.00	0.00	0.00	0.00	0.00	0.00	0.00	0.00	0.00
0.00	0.00	0.00	0.00	0.00	0.00	0.00	0.00	0.00
0.00	0.00	0.00	0.00	0.00	0.00	0.00	0.00	0.00
0.00	0.00	0.00	0.00	0.00	0.00	0.00	0.00	0.00
0.00	0.00	0.00	0.00	0.00	0.00	0.00	0.00	0.00
0.00	0.00	0.00	0.00	0.00	0.00	0.00	0.00	0.00
0.00	0.00	0.00	0.00	0.00	0.00	0.00	0.00	0.00
0.00	0.00	0.00	0.00	0.00	0.00	0.00	0.00	0.00
0.00	0.00	0.00	0.00	0.00	0.00	0.00	0.00	0.00
0.00	0.00	0.00	0.00	0.00	0.00	0.00	0.00	0.00
0.00	0.00	0.00	0.00	0.00	0.00	0.00	0.00	0.00
0.00	0.00	0.00	27.00	0.00	0.00	0.00	0.00	0.00
59.00	0.00	0.00	0.00	0.00	0.00	0.00	0.00	0.00
0.00	37.00	0.00	0.00	0.00	0.00	0.00	0.00	0.00

0.00	0.00	0.00	14.00	0.00	0.00	0.00	0.00	0.00
0.00	0.00	0.00	0.00	0.00	0.00	59.00	0.00	0.00
0.00	0.00	0.00	0.00	0.00	0.00	0.00	0.00	0.00
0.00	0.00	0.00	0.00	0.00	0.00	0.00	0.00	0.00
58.00	0.00	0.00	0.00	0.00	0.00	0.00	0.00	0.00
0.00	0.00	0.00	0.00	0.00	0.00	0.00	0.00	0.00
0.00	0.00	0.00	0.00	0.00	0.00	0.00	0.00	0.00
0.00	0.00	0.00	0.00	0.00	0.00	0.00	0.00	0.00
0.00	0.00	0.00	0.00	0.00	0.00	0.00	0.00	0.00
0.00	0.00	0.00	0.00	0.00	0.00	0.00	0.00	0.00
0.00	0.00	0.00	0.00	0.00	0.00	0.00	0.00	0.00
0.00	0.00	0.00	0.00	0.00	0.00	0.00	0.00	0.00
0.00	0.00	0.00	0.00	0.00	0.00	0.00	0.00	0.00
0.00	0.00	0.00	0.00	0.00	0.00	0.00	0.00	0.00
0.00	0.00	0.00	0.00	57.00	0.00	0.00	0.00	0.00
0.00	0.00	0.00	0.00	0.00	0.00	0.00	0.00	0.00
0.00	0.00	0.00	0.00	0.00	0.00	0.00	0.00	0.00
0.00	0.00	0.00	0.00	0.00	0.00	0.00	0.00	0.00
0.00	0.00	0.00	0.00	0.00	0.00	0.00	0.00	0.00
0.00	0.00	0.00	0.00	0.00	0.00	0.00	0.00	0.00
0.00	0.00	0.00	0.00	0.00	0.00	0.00	0.00	0.00
0.00	0.00	0.00	0.00	0.00	0.00	0.00	0.00	0.00
0.00	0.00	0.00	0.00	0.00	0.00	0.00	0.00	0.00
0.00	0.00	0.00	0.00	0.00	0.00	0.00	0.00	56.00
0.00	0.00	0.00	0.00	0.00	0.00	0.00	0.00	56.00
0.00	0.00	0.00	0.00	0.00	0.00	56.00	0.00	0.00
0.00	0.00	0.00	0.00	0.00	0.00	0.00	0.00	0.00
0.00	0.00	0.00	0.00	0.00	0.00	0.00	0.00	0.00
0.00	0.00	0.00	0.00	0.00	0.00	0.00	0.00	0.00
0.00	0.00	0.00	0.00	0.00	0.00	0.00	0.00	0.00
0.00	0.00	0.00	0.00	0.00	0.00	0.00	0.00	0.00
0.00	0.00	0.00	0.00	0.00	0.00	0.00	0.00	0.00
0.00	0.00	0.00	0.00	0.00	0.00	0.00	0.00	0.00
0.00	0.00	0.00	0.00	0.00	0.00	0.00	0.00	0.00
0.00	0.00	0.00	0.00	0.00	0.00	0.00	0.00	0.00
0.00	0.00	0.00	0.00	0.00	0.00	0.00	0.00	0.00
0.00	0.00	0.00	0.00	0.00	0.00	0.00	0.00	0.00

0.00	0.00	0.00	0.00	0.00	0.00	0.00	0.00	0.00
0.00	0.00	0.00	0.00	0.00	0.00	0.00	0.00	0.00
0.00	0.00	0.00	0.00	0.00	0.00	0.00	0.00	0.00
0.00	0.00	0.00	0.00	0.00	0.00	0.00	0.00	0.00
0.00	0.00	0.00	0.00	0.00	0.00	0.00	0.00	0.00
0.00	0.00	0.00	0.00	0.00	0.00	0.00	0.00	0.00
0.00	0.00	0.00	0.00	0.00	0.00	0.00	0.00	0.00
0.00	0.00	0.00	0.00	0.00	0.00	0.00	0.00	0.00
0.00	0.00	0.00	0.00	0.00	0.00	0.00	0.00	0.00
0.00	0.00	0.00	0.00	0.00	0.00	0.00	0.00	0.00
0.00	0.00	0.00	0.00	0.00	0.00	0.00	0.00	0.00
0.00	0.00	0.00	0.00	0.00	0.00	0.00	0.00	0.00
0.00	0.00	0.00	0.00	0.00	0.00	27.00	0.00	0.00
0.00	0.00	0.00	0.00	0.00	0.00	0.00	28.00	0.00
0.00	0.00	0.00	0.00	0.00	0.00	0.00	0.00	0.00
0.00	0.00	0.00	0.00	0.00	0.00	0.00	0.00	0.00
0.00	0.00	0.00	0.00	0.00	0.00	0.00	0.00	0.00
0.00	19.00	0.00	0.00	0.00	0.00	0.00	0.00	0.00
0.00	16.00	0.00	0.00	0.00	0.00	0.00	0.00	0.00
0.00	0.00	0.00	0.00	0.00	0.00	0.00	0.00	0.00
0.00	0.00	0.00	0.00	0.00	0.00	0.00	0.00	0.00
0.00	0.00	0.00	0.00	0.00	0.00	0.00	0.00	0.00
0.00	0.00	0.00	0.00	0.00	0.00	0.00	0.00	0.00
3.00	4.00	0.00	0.00	0.00	0.00	0.00	2.00	0.00
0.00	0.00	0.00	0.00	0.00	0.00	0.00	0.00	0.00
0.00	0.00	0.00	50.00	0.00	0.00	0.00	0.00	0.00
0.00	0.00	0.00	0.00	0.00	0.00	0.00	50.00	0.00
0.00	0.00	0.00	0.00	0.00	0.00	0.00	0.00	0.00
0.00	0.00	0.00	0.00	0.00	0.00	0.00	0.00	0.00
0.00	0.00	0.00	0.00	0.00	0.00	0.00	0.00	0.00
0.00	0.00	0.00	0.00	0.00	0.00	0.00	0.00	0.00
0.00	0.00	0.00	0.00	0.00	0.00	27.00	0.00	0.00
0.00	0.00	0.00	0.00	0.00	0.00	0.00	0.00	0.00
0.00	0.00	0.00	49.00	0.00	0.00	0.00	0.00	0.00
0.00	0.00	0.00	0.00	0.00	0.00	0.00	0.00	0.00
0.00	0.00	0.00	0.00	0.00	0.00	0.00	0.00	0.00
0.00	0.00	0.00	0.00	0.00	0.00	0.00	0.00	0.00

0.00	0.00	0.00	0.00	0.00	0.00	0.00	0.00	0.00
0.00	0.00	0.00	0.00	0.00	0.00	0.00	0.00	0.00
0.00	0.00	0.00	0.00	0.00	0.00	48.00	0.00	0.00
0.00	0.00	0.00	0.00	0.00	0.00	0.00	0.00	0.00
0.00	0.00	0.00	0.00	0.00	0.00	0.00	0.00	0.00
0.00	0.00	0.00	0.00	0.00	0.00	0.00	0.00	0.00
0.00	0.00	0.00	0.00	0.00	0.00	0.00	0.00	0.00
0.00	0.00	0.00	0.00	0.00	0.00	0.00	0.00	0.00
0.00	0.00	0.00	0.00	0.00	0.00	0.00	0.00	0.00
0.00	0.00	0.00	0.00	0.00	0.00	0.00	0.00	0.00
0.00	7.00	0.00	0.00	0.00	0.00	0.00	34.00	0.00
0.00	0.00	0.00	0.00	0.00	0.00	0.00	0.00	0.00
0.00	0.00	0.00	0.00	0.00	0.00	0.00	47.00	0.00
0.00	0.00	0.00	0.00	0.00	0.00	0.00	47.00	0.00
0.00	0.00	0.00	0.00	0.00	0.00	0.00	0.00	0.00
0.00	0.00	0.00	0.00	0.00	0.00	0.00	0.00	0.00
0.00	0.00	0.00	0.00	0.00	0.00	0.00	0.00	0.00
0.00	0.00	0.00	0.00	0.00	0.00	0.00	0.00	0.00
0.00	0.00	0.00	0.00	0.00	0.00	0.00	0.00	0.00
0.00	0.00	0.00	0.00	0.00	0.00	0.00	0.00	0.00
0.00	0.00	0.00	0.00	0.00	0.00	0.00	0.00	0.00
0.00	14.00	0.00	0.00	0.00	0.00	7.00	0.00	0.00
0.00	0.00	0.00	22.00	0.00	0.00	0.00	0.00	0.00
0.00	0.00	0.00	0.00	0.00	0.00	46.00	0.00	0.00
0.00	0.00	0.00	0.00	0.00	0.00	0.00	46.00	0.00
0.00	0.00	0.00	0.00	0.00	0.00	0.00	0.00	0.00
0.00	0.00	0.00	0.00	0.00	0.00	0.00	0.00	0.00
0.00	0.00	0.00	0.00	0.00	0.00	0.00	0.00	0.00
0.00	0.00	0.00	0.00	0.00	0.00	0.00	0.00	0.00
0.00	0.00	0.00	0.00	0.00	0.00	0.00	0.00	0.00
0.00	0.00	0.00	0.00	0.00	0.00	0.00	0.00	0.00
0.00	0.00	0.00	0.00	0.00	0.00	11.00	0.00	0.00
0.00	0.00	0.00	0.00	0.00	0.00	0.00	0.00	0.00
0.00	0.00	0.00	0.00	0.00	0.00	0.00	22.00	0.00
0.00	11.00	0.00	0.00	0.00	0.00	0.00	12.00	0.00
0.00	0.00	0.00	0.00	0.00	45.00	0.00	0.00	0.00
0.00	0.00	0.00	0.00	0.00	0.00	0.00	0.00	0.00

0.00	0.00	0.00	0.00	0.00	0.00	0.00	0.00	0.00
0.00	0.00	0.00	0.00	0.00	0.00	0.00	0.00	0.00
0.00	0.00	0.00	0.00	0.00	0.00	0.00	0.00	0.00
0.00	0.00	0.00	0.00	0.00	0.00	0.00	0.00	0.00
0.00	0.00	0.00	0.00	0.00	0.00	0.00	0.00	44.00
0.00	0.00	0.00	0.00	0.00	0.00	0.00	0.00	0.00
0.00	0.00	44.00	0.00	0.00	0.00	0.00	0.00	0.00
0.00	0.00	0.00	0.00	0.00	0.00	3.00	0.00	0.00
0.00	0.00	0.00	0.00	0.00	0.00	0.00	0.00	0.00
0.00	0.00	0.00	0.00	0.00	0.00	0.00	0.00	0.00
0.00	0.00	0.00	0.00	0.00	0.00	0.00	0.00	0.00
0.00	0.00	0.00	0.00	0.00	0.00	0.00	0.00	0.00
0.00	0.00	0.00	0.00	0.00	0.00	0.00	0.00	0.00
0.00	0.00	0.00	0.00	0.00	0.00	0.00	0.00	0.00
0.00	0.00	0.00	0.00	0.00	0.00	43.00	0.00	0.00
0.00	0.00	0.00	0.00	0.00	0.00	0.00	0.00	0.00
0.00	0.00	0.00	0.00	0.00	0.00	0.00	0.00	0.00
0.00	0.00	0.00	0.00	0.00	0.00	0.00	0.00	0.00
0.00	0.00	0.00	0.00	0.00	0.00	0.00	0.00	0.00
0.00	0.00	0.00	0.00	0.00	0.00	0.00	0.00	0.00
0.00	0.00	0.00	7.00	0.00	0.00	0.00	0.00	0.00
0.00	0.00	0.00	0.00	0.00	0.00	40.00	0.00	0.00
0.00	0.00	0.00	0.00	0.00	0.00	0.00	42.00	0.00
0.00	0.00	0.00	0.00	0.00	0.00	0.00	0.00	0.00
0.00	0.00	0.00	0.00	0.00	0.00	0.00	0.00	0.00
0.00	0.00	0.00	0.00	0.00	0.00	0.00	0.00	0.00
0.00	0.00	0.00	0.00	0.00	0.00	0.00	0.00	0.00
0.00	0.00	0.00	0.00	0.00	0.00	0.00	0.00	0.00
0.00	0.00	0.00	0.00	0.00	0.00	0.00	0.00	0.00
0.00	0.00	0.00	0.00	0.00	0.00	0.00	0.00	0.00
0.00	0.00	0.00	0.00	0.00	0.00	0.00	0.00	0.00
0.00	0.00	0.00	0.00	0.00	9.00	0.00	0.00	0.00
0.00	0.00	0.00	0.00	0.00	0.00	0.00	0.00	0.00
0.00	0.00	0.00	0.00	0.00	0.00	0.00	0.00	0.00
0.00	0.00	0.00	0.00	0.00	0.00	0.00	0.00	0.00
0.00	0.00	0.00	0.00	0.00	0.00	0.00	0.00	0.00
0.00	0.00	0.00	0.00	0.00	0.00	0.00	0.00	0.00

0.00	0.00	0.00	0.00	0.00	0.00	0.00	0.00	0.00
7.00	8.00	0.00	0.00	0.00	0.00	0.00	5.00	0.00
0.00	0.00	0.00	0.00	0.00	4.00	3.00	0.00	2.00
0.00	0.00	0.00	0.00	0.00	0.00	0.00	0.00	0.00
0.00	0.00	0.00	0.00	0.00	0.00	0.00	0.00	0.00
0.00	0.00	0.00	0.00	0.00	0.00	0.00	0.00	0.00
0.00	0.00	0.00	0.00	0.00	0.00	0.00	0.00	0.00
0.00	0.00	0.00	0.00	0.00	0.00	0.00	0.00	0.00
0.00	0.00	0.00	0.00	0.00	0.00	0.00	0.00	0.00
0.00	0.00	0.00	0.00	0.00	0.00	0.00	0.00	0.00
15.00	0.00	0.00	0.00	0.00	0.00	0.00	0.00	0.00
0.00	0.00	39.00	0.00	0.00	0.00	0.00	0.00	0.00
0.00	0.00	0.00	0.00	0.00	0.00	0.00	0.00	0.00
0.00	0.00	0.00	0.00	0.00	0.00	0.00	0.00	0.00
0.00	0.00	0.00	0.00	0.00	0.00	0.00	0.00	0.00
0.00	0.00	0.00	0.00	0.00	0.00	0.00	0.00	0.00
0.00	0.00	0.00	0.00	0.00	0.00	0.00	0.00	0.00
0.00	0.00	0.00	0.00	0.00	0.00	0.00	0.00	0.00
0.00	0.00	0.00	0.00	0.00	0.00	0.00	0.00	0.00
0.00	0.00	0.00	0.00	0.00	0.00	0.00	0.00	0.00
0.00	0.00	0.00	18.00	0.00	0.00	0.00	0.00	0.00
0.00	0.00	0.00	3.00	0.00	0.00	0.00	0.00	0.00
7.00	0.00	14.00	5.00	0.00	0.00	6.00	0.00	0.00
0.00	0.00	0.00	0.00	0.00	0.00	0.00	0.00	0.00
0.00	0.00	0.00	0.00	0.00	0.00	0.00	0.00	0.00
0.00	0.00	0.00	0.00	0.00	0.00	0.00	0.00	0.00
0.00	0.00	0.00	32.00	0.00	0.00	0.00	0.00	0.00
0.00	0.00	0.00	0.00	0.00	0.00	9.00	9.00	0.00
0.00	0.00	0.00	0.00	0.00	0.00	0.00	0.00	0.00
0.00	0.00	0.00	0.00	0.00	0.00	0.00	0.00	0.00
0.00	0.00	0.00	0.00	0.00	0.00	0.00	0.00	0.00
0.00	0.00	0.00	0.00	0.00	0.00	0.00	0.00	0.00
0.00	0.00	0.00	0.00	0.00	0.00	0.00	0.00	0.00
0.00	0.00	0.00	0.00	0.00	0.00	0.00	0.00	0.00
0.00	0.00	0.00	0.00	0.00	0.00	0.00	0.00	0.00
37.00	0.00	0.00	0.00	0.00	0.00	0.00	0.00	0.00
0.00	0.00	0.00	0.00	0.00	0.00	0.00	0.00	37.00

0.00	0.00	0.00	0.00	0.00	0.00	0.00	0.00	0.00
0.00	0.00	0.00	0.00	0.00	0.00	0.00	0.00	0.00
0.00	0.00	0.00	0.00	0.00	0.00	0.00	2.00	0.00
0.00	0.00	0.00	0.00	0.00	0.00	0.00	0.00	0.00
0.00	0.00	0.00	0.00	0.00	0.00	0.00	0.00	0.00
0.00	0.00	0.00	0.00	0.00	0.00	0.00	0.00	0.00
0.00	0.00	0.00	0.00	0.00	0.00	0.00	0.00	0.00
6.00	5.00	0.00	2.00	0.00	0.00	0.00	0.00	0.00
0.00	0.00	0.00	0.00	0.00	0.00	0.00	0.00	0.00
0.00	0.00	0.00	0.00	0.00	0.00	0.00	0.00	0.00
0.00	0.00	0.00	0.00	0.00	0.00	0.00	0.00	0.00
0.00	0.00	0.00	0.00	0.00	0.00	0.00	0.00	0.00
0.00	0.00	0.00	0.00	0.00	0.00	0.00	0.00	0.00
0.00	0.00	0.00	0.00	0.00	0.00	0.00	0.00	0.00
0.00	0.00	0.00	0.00	0.00	0.00	0.00	0.00	0.00
0.00	0.00	0.00	0.00	0.00	0.00	0.00	0.00	0.00
0.00	0.00	0.00	0.00	0.00	0.00	0.00	0.00	0.00
18.00	0.00	0.00	0.00	0.00	0.00	0.00	0.00	0.00
0.00	0.00	0.00	0.00	0.00	0.00	0.00	0.00	0.00
0.00	0.00	0.00	0.00	0.00	0.00	0.00	0.00	0.00
0.00	0.00	0.00	0.00	0.00	0.00	0.00	0.00	0.00
0.00	0.00	0.00	0.00	0.00	0.00	0.00	0.00	0.00
0.00	0.00	0.00	0.00	0.00	0.00	0.00	0.00	0.00
0.00	0.00	0.00	0.00	0.00	0.00	0.00	0.00	0.00
0.00	0.00	0.00	0.00	0.00	0.00	0.00	0.00	0.00
0.00	0.00	0.00	0.00	0.00	0.00	0.00	0.00	0.00
0.00	0.00	0.00	0.00	0.00	0.00	0.00	0.00	0.00
0.00	0.00	34.00	0.00	0.00	0.00	0.00	0.00	0.00
0.00	0.00	0.00	0.00	0.00	0.00	0.00	0.00	0.00
0.00	0.00	0.00	0.00	0.00	0.00	0.00	0.00	0.00
0.00	0.00	0.00	0.00	0.00	0.00	0.00	0.00	0.00
0.00	0.00	0.00	5.00	0.00	0.00	0.00	8.00	9.00
0.00	0.00	0.00	0.00	0.00	0.00	0.00	0.00	0.00
0.00	0.00	0.00	0.00	0.00	0.00	0.00	0.00	0.00
0.00	0.00	0.00	0.00	0.00	0.00	0.00	0.00	0.00
0.00	0.00	0.00	0.00	0.00	0.00	0.00	0.00	0.00
0.00	0.00	0.00	0.00	0.00	0.00	0.00	0.00	0.00

0.00	0.00	0.00	0.00	0.00	0.00	0.00	0.00	0.00
0.00	0.00	0.00	0.00	0.00	0.00	0.00	0.00	0.00
0.00	0.00	0.00	0.00	0.00	0.00	0.00	0.00	0.00
0.00	0.00	0.00	0.00	0.00	0.00	0.00	0.00	0.00
0.00	0.00	0.00	0.00	0.00	0.00	0.00	0.00	0.00
0.00	0.00	0.00	0.00	0.00	0.00	0.00	0.00	0.00
0.00	0.00	0.00	0.00	0.00	0.00	0.00	0.00	0.00
0.00	0.00	0.00	0.00	0.00	0.00	0.00	0.00	0.00
3.00	14.00	0.00	0.00	0.00	0.00	0.00	0.00	0.00
0.00	32.00	0.00	0.00	0.00	0.00	0.00	0.00	0.00
0.00	0.00	0.00	0.00	0.00	0.00	0.00	0.00	32.00
0.00	0.00	0.00	0.00	0.00	0.00	0.00	0.00	0.00
0.00	0.00	0.00	0.00	0.00	0.00	0.00	0.00	0.00
0.00	0.00	0.00	0.00	0.00	0.00	0.00	0.00	0.00
0.00	0.00	0.00	0.00	0.00	0.00	0.00	0.00	0.00
0.00	0.00	0.00	0.00	0.00	0.00	0.00	0.00	0.00
0.00	0.00	0.00	0.00	0.00	0.00	0.00	0.00	0.00
0.00	0.00	0.00	0.00	0.00	0.00	0.00	0.00	0.00
0.00	0.00	0.00	0.00	0.00	0.00	0.00	10.00	0.00
0.00	0.00	0.00	0.00	0.00	0.00	0.00	0.00	31.00
0.00	0.00	0.00	0.00	0.00	0.00	0.00	0.00	0.00
0.00	0.00	0.00	0.00	0.00	0.00	0.00	0.00	0.00
0.00	0.00	0.00	0.00	0.00	0.00	0.00	0.00	0.00
0.00	0.00	0.00	0.00	0.00	0.00	0.00	0.00	0.00
0.00	0.00	0.00	0.00	0.00	0.00	31.00	0.00	0.00
0.00	0.00	0.00	0.00	0.00	0.00	0.00	31.00	0.00
0.00	0.00	0.00	0.00	0.00	0.00	0.00	0.00	0.00
0.00	0.00	0.00	0.00	0.00	0.00	0.00	0.00	0.00
0.00	0.00	0.00	0.00	0.00	0.00	0.00	0.00	0.00
0.00	0.00	0.00	0.00	0.00	0.00	0.00	0.00	0.00
0.00	0.00	0.00	0.00	0.00	0.00	0.00	0.00	0.00
0.00	0.00	0.00	0.00	0.00	0.00	0.00	0.00	0.00
0.00	0.00	0.00	0.00	0.00	0.00	0.00	0.00	0.00
0.00	0.00	0.00	0.00	0.00	0.00	0.00	0.00	0.00
0.00	0.00	0.00	0.00	0.00	0.00	0.00	0.00	0.00
0.00	0.00	0.00	0.00	0.00	0.00	0.00	0.00	0.00
0.00	0.00	0.00	0.00	0.00	0.00	0.00	0.00	0.00

0.00	0.00	30.00	0.00	0.00	0.00	0.00	0.00	0.00
0.00	0.00	0.00	0.00	0.00	0.00	30.00	0.00	0.00
0.00	0.00	0.00	0.00	0.00	0.00	0.00	0.00	0.00
0.00	0.00	0.00	0.00	0.00	0.00	0.00	0.00	0.00
0.00	0.00	0.00	0.00	0.00	0.00	0.00	0.00	0.00
0.00	0.00	0.00	0.00	0.00	0.00	0.00	0.00	0.00
0.00	0.00	0.00	0.00	0.00	0.00	0.00	0.00	0.00
0.00	0.00	0.00	0.00	0.00	0.00	0.00	0.00	0.00
0.00	0.00	0.00	0.00	0.00	0.00	0.00	0.00	0.00
0.00	0.00	0.00	0.00	0.00	0.00	0.00	0.00	0.00
0.00	0.00	0.00	0.00	0.00	0.00	0.00	0.00	0.00
0.00	0.00	0.00	0.00	0.00	0.00	0.00	0.00	0.00
0.00	0.00	0.00	0.00	0.00	0.00	0.00	0.00	0.00
0.00	0.00	0.00	0.00	0.00	0.00	0.00	0.00	0.00
0.00	0.00	0.00	0.00	0.00	0.00	0.00	0.00	0.00
0.00	0.00	0.00	0.00	0.00	0.00	0.00	0.00	0.00
0.00	0.00	0.00	0.00	0.00	0.00	0.00	0.00	0.00
5.00	0.00	0.00	0.00	0.00	0.00	0.00	0.00	0.00
0.00	0.00	0.00	0.00	0.00	0.00	0.00	0.00	0.00
0.00	0.00	0.00	0.00	0.00	0.00	0.00	0.00	0.00
0.00	0.00	0.00	0.00	0.00	0.00	0.00	0.00	0.00
0.00	0.00	0.00	0.00	0.00	0.00	0.00	0.00	0.00
0.00	0.00	0.00	0.00	0.00	0.00	0.00	0.00	0.00
0.00	0.00	0.00	0.00	0.00	0.00	0.00	0.00	0.00
0.00	0.00	0.00	0.00	0.00	0.00	0.00	0.00	0.00
0.00	5.00	0.00	0.00	0.00	0.00	7.00	0.00	0.00
0.00	14.00	0.00	0.00	0.00	0.00	0.00	0.00	0.00
0.00	0.00	0.00	0.00	0.00	0.00	0.00	0.00	28.00
0.00	0.00	0.00	28.00	0.00	0.00	0.00	0.00	0.00
0.00	0.00	0.00	0.00	0.00	0.00	0.00	0.00	0.00
0.00	0.00	0.00	0.00	0.00	0.00	0.00	0.00	0.00
0.00	0.00	0.00	0.00	0.00	0.00	0.00	0.00	0.00
0.00	0.00	0.00	0.00	0.00	0.00	0.00	0.00	0.00
0.00	0.00	0.00	0.00	0.00	0.00	0.00	0.00	0.00
0.00	0.00	0.00	0.00	0.00	0.00	0.00	0.00	0.00
0.00	0.00	0.00	0.00	0.00	0.00	0.00	0.00	0.00
0.00	2.00	0.00	2.00	0.00	0.00	0.00	0.00	0.00

0.00	25.00	0.00	0.00	0.00	0.00	0.00	0.00	0.00
0.00	0.00	0.00	0.00	0.00	0.00	0.00	0.00	0.00
0.00	0.00	0.00	0.00	0.00	0.00	0.00	0.00	0.00
0.00	0.00	0.00	0.00	0.00	0.00	0.00	0.00	0.00
0.00	0.00	0.00	0.00	0.00	0.00	0.00	0.00	0.00
0.00	0.00	0.00	0.00	0.00	0.00	0.00	0.00	0.00
0.00	0.00	0.00	27.00	0.00	0.00	0.00	0.00	0.00
0.00	0.00	0.00	0.00	0.00	0.00	0.00	0.00	0.00
0.00	0.00	0.00	0.00	0.00	0.00	0.00	0.00	0.00
0.00	0.00	0.00	0.00	0.00	0.00	0.00	0.00	0.00
0.00	0.00	0.00	0.00	0.00	0.00	0.00	0.00	0.00
0.00	0.00	0.00	0.00	0.00	0.00	0.00	0.00	0.00
0.00	0.00	0.00	0.00	0.00	0.00	0.00	0.00	0.00
0.00	0.00	0.00	0.00	0.00	0.00	0.00	0.00	0.00
0.00	0.00	0.00	0.00	0.00	0.00	0.00	0.00	0.00
0.00	0.00	0.00	0.00	0.00	0.00	0.00	0.00	0.00
0.00	0.00	0.00	0.00	0.00	0.00	0.00	0.00	0.00
0.00	0.00	0.00	0.00	0.00	0.00	0.00	0.00	0.00
0.00	0.00	0.00	0.00	0.00	0.00	0.00	0.00	0.00
0.00	0.00	0.00	0.00	0.00	0.00	0.00	0.00	0.00
0.00	0.00	0.00	0.00	0.00	0.00	0.00	0.00	0.00
0.00	0.00	0.00	0.00	0.00	0.00	0.00	0.00	0.00
0.00	0.00	0.00	0.00	0.00	0.00	0.00	0.00	0.00
0.00	0.00	0.00	0.00	0.00	0.00	0.00	0.00	0.00
0.00	0.00	0.00	0.00	0.00	0.00	0.00	0.00	0.00
0.00	0.00	0.00	0.00	0.00	0.00	0.00	0.00	0.00
0.00	0.00	0.00	0.00	0.00	0.00	0.00	0.00	0.00
0.00	0.00	0.00	0.00	0.00	0.00	0.00	0.00	0.00
0.00	0.00	0.00	0.00	0.00	0.00	0.00	0.00	0.00
0.00	0.00	0.00	0.00	0.00	0.00	0.00	0.00	0.00
0.00	0.00	0.00	0.00	0.00	0.00	0.00	0.00	0.00
0.00	0.00	0.00	0.00	0.00	0.00	0.00	0.00	0.00
0.00	0.00	0.00	0.00	0.00	0.00	0.00	0.00	0.00
0.00	0.00	0.00	0.00	0.00	0.00	0.00	0.00	0.00
0.00	0.00	0.00	0.00	0.00	0.00	0.00	0.00	0.00
0.00	0.00	0.00	0.00	0.00	0.00	0.00	0.00	0.00
0.00	0.00	0.00	0.00	0.00	0.00	0.00	0.00	0.00

0.00	0.00	0.00	0.00	0.00	0.00	0.00	0.00	0.00
0.00	0.00	0.00	0.00	0.00	0.00	0.00	25.00	0.00
0.00	0.00	0.00	0.00	0.00	0.00	0.00	0.00	0.00
0.00	0.00	0.00	0.00	0.00	0.00	0.00	0.00	0.00
0.00	0.00	0.00	0.00	0.00	0.00	0.00	0.00	0.00
0.00	0.00	0.00	0.00	0.00	0.00	0.00	0.00	0.00
0.00	0.00	0.00	0.00	0.00	0.00	0.00	0.00	0.00
0.00	0.00	0.00	0.00	0.00	0.00	0.00	0.00	0.00
0.00	0.00	0.00	0.00	0.00	0.00	0.00	0.00	0.00
0.00	0.00	0.00	0.00	0.00	0.00	0.00	0.00	0.00
0.00	0.00	0.00	0.00	0.00	0.00	0.00	0.00	0.00
0.00	0.00	0.00	0.00	0.00	0.00	0.00	0.00	0.00
0.00	0.00	0.00	0.00	0.00	0.00	0.00	0.00	0.00
24.00	0.00	0.00	0.00	0.00	0.00	0.00	0.00	0.00
0.00	3.00	0.00	0.00	0.00	0.00	0.00	0.00	0.00
0.00	0.00	0.00	4.00	0.00	2.00	8.00	0.00	0.00
0.00	0.00	0.00	0.00	0.00	0.00	0.00	0.00	0.00
0.00	0.00	0.00	0.00	0.00	0.00	15.00	0.00	0.00
0.00	0.00	0.00	0.00	0.00	24.00	0.00	0.00	0.00
0.00	0.00	0.00	0.00	0.00	0.00	24.00	0.00	0.00
0.00	0.00	0.00	0.00	0.00	0.00	0.00	24.00	0.00
0.00	0.00	0.00	0.00	0.00	0.00	0.00	0.00	0.00
0.00	0.00	0.00	0.00	0.00	0.00	0.00	0.00	0.00
0.00	0.00	0.00	0.00	0.00	0.00	0.00	0.00	0.00
0.00	0.00	0.00	0.00	0.00	0.00	0.00	0.00	0.00
0.00	0.00	0.00	0.00	0.00	0.00	0.00	0.00	0.00
0.00	0.00	0.00	0.00	0.00	0.00	0.00	0.00	0.00
0.00	0.00	0.00	0.00	0.00	0.00	0.00	0.00	0.00
0.00	0.00	0.00	0.00	0.00	0.00	0.00	0.00	0.00
0.00	0.00	0.00	0.00	0.00	0.00	0.00	0.00	0.00
0.00	0.00	0.00	0.00	0.00	0.00	0.00	0.00	0.00
0.00	0.00	0.00	0.00	0.00	0.00	0.00	0.00	0.00
0.00	0.00	0.00	0.00	0.00	0.00	0.00	0.00	0.00
0.00	0.00	0.00	0.00	0.00	0.00	0.00	0.00	0.00
0.00	0.00	0.00	0.00	0.00	0.00	0.00	0.00	0.00
0.00	0.00	0.00	0.00	0.00	0.00	0.00	0.00	0.00
0.00	0.00	0.00	0.00	0.00	0.00	0.00	0.00	0.00

0.00	0.00	0.00	0.00	0.00	0.00	0.00	0.00	0.00
0.00	0.00	0.00	0.00	0.00	0.00	0.00	0.00	0.00
0.00	0.00	0.00	0.00	0.00	0.00	0.00	0.00	0.00
0.00	0.00	0.00	0.00	0.00	0.00	0.00	0.00	0.00
0.00	0.00	0.00	0.00	0.00	0.00	0.00	0.00	0.00
0.00	0.00	0.00	0.00	0.00	0.00	0.00	0.00	0.00
0.00	0.00	0.00	0.00	0.00	0.00	0.00	0.00	0.00
5.00	0.00	0.00	5.00	0.00	0.00	0.00	0.00	0.00
0.00	0.00	0.00	0.00	0.00	0.00	0.00	0.00	0.00
0.00	0.00	0.00	0.00	0.00	0.00	0.00	0.00	0.00
0.00	0.00	0.00	0.00	0.00	0.00	0.00	0.00	0.00
0.00	0.00	22.00	0.00	0.00	0.00	0.00	0.00	0.00
0.00	0.00	0.00	0.00	0.00	0.00	0.00	0.00	0.00
0.00	0.00	0.00	0.00	0.00	0.00	0.00	0.00	0.00
0.00	0.00	0.00	0.00	0.00	0.00	0.00	0.00	0.00
0.00	0.00	0.00	0.00	0.00	0.00	0.00	0.00	0.00
0.00	0.00	0.00	0.00	0.00	0.00	0.00	0.00	0.00
0.00	0.00	0.00	0.00	0.00	0.00	0.00	0.00	0.00
0.00	0.00	0.00	0.00	0.00	0.00	0.00	0.00	0.00
0.00	0.00	0.00	0.00	0.00	0.00	0.00	0.00	0.00
0.00	0.00	0.00	0.00	0.00	0.00	0.00	0.00	0.00
0.00	0.00	0.00	0.00	0.00	0.00	0.00	0.00	0.00
0.00	0.00	0.00	0.00	0.00	0.00	0.00	0.00	0.00
0.00	0.00	0.00	0.00	0.00	0.00	0.00	0.00	0.00
0.00	0.00	0.00	0.00	0.00	0.00	0.00	0.00	0.00
0.00	0.00	0.00	0.00	0.00	0.00	0.00	0.00	0.00
0.00	5.00	0.00	0.00	0.00	0.00	0.00	0.00	0.00
0.00	0.00	0.00	0.00	0.00	0.00	0.00	0.00	0.00
0.00	21.00	0.00	0.00	0.00	0.00	0.00	0.00	0.00
0.00	0.00	0.00	0.00	0.00	0.00	0.00	0.00	0.00
0.00	0.00	0.00	0.00	0.00	0.00	0.00	0.00	0.00
0.00	0.00	0.00	0.00	0.00	0.00	0.00	0.00	0.00
0.00	0.00	0.00	0.00	0.00	0.00	0.00	0.00	0.00
0.00	0.00	0.00	0.00	0.00	0.00	0.00	0.00	0.00
0.00	0.00	13.00	8.00	0.00	0.00	0.00	0.00	0.00
0.00	0.00	0.00	0.00	0.00	0.00	0.00	0.00	0.00
0.00	0.00	0.00	0.00	0.00	0.00	0.00	0.00	0.00

0.00	0.00	0.00	0.00	0.00	0.00	0.00	0.00	0.00
0.00	0.00	0.00	0.00	0.00	0.00	0.00	0.00	0.00
0.00	0.00	0.00	0.00	0.00	0.00	0.00	0.00	0.00
0.00	0.00	0.00	0.00	0.00	0.00	0.00	0.00	0.00
0.00	0.00	0.00	0.00	0.00	0.00	0.00	0.00	0.00
0.00	0.00	0.00	0.00	0.00	0.00	0.00	0.00	0.00
0.00	0.00	0.00	0.00	0.00	0.00	0.00	0.00	0.00
0.00	0.00	0.00	0.00	0.00	0.00	0.00	0.00	0.00
0.00	0.00	0.00	0.00	0.00	0.00	0.00	0.00	0.00
0.00	0.00	0.00	0.00	0.00	0.00	0.00	0.00	0.00
0.00	0.00	0.00	0.00	0.00	0.00	0.00	0.00	0.00
0.00	0.00	0.00	0.00	0.00	0.00	0.00	0.00	0.00
0.00	2.00	0.00	0.00	0.00	0.00	0.00	0.00	0.00
0.00	0.00	0.00	0.00	0.00	0.00	0.00	0.00	0.00
0.00	0.00	0.00	0.00	0.00	0.00	0.00	0.00	0.00
0.00	0.00	0.00	0.00	0.00	20.00	0.00	0.00	0.00
0.00	0.00	0.00	0.00	0.00	0.00	20.00	0.00	0.00
0.00	0.00	0.00	0.00	0.00	0.00	0.00	0.00	0.00
0.00	0.00	0.00	0.00	0.00	0.00	0.00	0.00	0.00
0.00	0.00	0.00	0.00	0.00	0.00	0.00	0.00	0.00
0.00	0.00	0.00	0.00	0.00	0.00	0.00	0.00	0.00
0.00	0.00	0.00	0.00	0.00	0.00	0.00	0.00	0.00
0.00	0.00	0.00	0.00	0.00	0.00	0.00	0.00	0.00
0.00	0.00	0.00	0.00	0.00	0.00	0.00	0.00	0.00
0.00	0.00	0.00	0.00	0.00	0.00	0.00	0.00	0.00
0.00	0.00	0.00	0.00	0.00	0.00	0.00	0.00	0.00
0.00	0.00	0.00	0.00	0.00	0.00	0.00	0.00	0.00
0.00	0.00	0.00	0.00	0.00	0.00	0.00	0.00	0.00
0.00	0.00	0.00	0.00	0.00	0.00	0.00	0.00	0.00
0.00	0.00	0.00	0.00	0.00	0.00	0.00	0.00	0.00
0.00	0.00	0.00	0.00	0.00	0.00	0.00	0.00	0.00
0.00	0.00	0.00	0.00	0.00	0.00	0.00	0.00	0.00
0.00	0.00	0.00	0.00	0.00	0.00	0.00	0.00	0.00
0.00	0.00	0.00	0.00	0.00	0.00	0.00	0.00	0.00
0.00	0.00	0.00	0.00	0.00	0.00	0.00	0.00	0.00
0.00	0.00	0.00	0.00	0.00	0.00	0.00	0.00	0.00
0.00	0.00	0.00	0.00	0.00	0.00	0.00	0.00	0.00

0.00	0.00	0.00	0.00	0.00	0.00	0.00	0.00	0.00
0.00	0.00	0.00	0.00	0.00	0.00	0.00	0.00	0.00
0.00	0.00	0.00	0.00	0.00	0.00	0.00	0.00	0.00
0.00	0.00	0.00	0.00	0.00	0.00	0.00	0.00	0.00
0.00	0.00	0.00	0.00	0.00	0.00	8.00	0.00	0.00
0.00	0.00	0.00	0.00	0.00	0.00	0.00	19.00	0.00
0.00	0.00	0.00	0.00	0.00	0.00	0.00	0.00	0.00
0.00	0.00	0.00	0.00	0.00	0.00	0.00	0.00	0.00
0.00	0.00	0.00	0.00	0.00	0.00	0.00	0.00	0.00
0.00	0.00	0.00	0.00	0.00	0.00	0.00	0.00	0.00
0.00	0.00	0.00	0.00	0.00	0.00	0.00	0.00	0.00
0.00	0.00	0.00	0.00	0.00	0.00	0.00	0.00	0.00
0.00	0.00	0.00	0.00	0.00	0.00	0.00	0.00	0.00
0.00	0.00	0.00	0.00	0.00	0.00	0.00	0.00	0.00
0.00	0.00	0.00	0.00	0.00	0.00	0.00	0.00	0.00
0.00	0.00	0.00	0.00	0.00	0.00	0.00	0.00	0.00
0.00	0.00	0.00	0.00	0.00	0.00	0.00	0.00	0.00
0.00	0.00	0.00	0.00	0.00	0.00	0.00	0.00	0.00
0.00	0.00	0.00	0.00	0.00	0.00	0.00	0.00	0.00
0.00	0.00	0.00	0.00	0.00	0.00	0.00	0.00	0.00
6.00	0.00	0.00	0.00	0.00	0.00	0.00	0.00	0.00
3.00	0.00	0.00	0.00	0.00	0.00	7.00	0.00	0.00
0.00	0.00	0.00	0.00	0.00	2.00	0.00	0.00	0.00
8.00	0.00	0.00	0.00	0.00	0.00	0.00	0.00	10.00
18.00	0.00	0.00	0.00	0.00	0.00	0.00	0.00	0.00
0.00	0.00	0.00	0.00	0.00	4.00	8.00	2.00	4.00
0.00	0.00	0.00	0.00	0.00	0.00	0.00	0.00	0.00
0.00	0.00	0.00	0.00	0.00	0.00	0.00	0.00	0.00
0.00	0.00	5.00	0.00	0.00	6.00	0.00	0.00	0.00
0.00	0.00	0.00	0.00	0.00	0.00	0.00	0.00	0.00
0.00	0.00	0.00	0.00	0.00	0.00	0.00	0.00	0.00
0.00	0.00	0.00	0.00	0.00	0.00	0.00	0.00	0.00
0.00	0.00	0.00	0.00	0.00	0.00	0.00	0.00	0.00
0.00	0.00	0.00	0.00	0.00	18.00	0.00	0.00	0.00
0.00	0.00	0.00	0.00	0.00	0.00	0.00	0.00	0.00
0.00	0.00	0.00	0.00	0.00	0.00	0.00	0.00	0.00
0.00	0.00	0.00	0.00	0.00	0.00	0.00	0.00	0.00

0.00	0.00	0.00	0.00	0.00	0.00	0.00	0.00	0.00
0.00	0.00	0.00	0.00	0.00	0.00	0.00	0.00	0.00
0.00	0.00	0.00	0.00	0.00	0.00	0.00	0.00	0.00
0.00	0.00	0.00	0.00	0.00	0.00	0.00	0.00	0.00
0.00	0.00	0.00	0.00	0.00	0.00	0.00	0.00	0.00
0.00	0.00	0.00	0.00	0.00	0.00	0.00	0.00	0.00
0.00	0.00	0.00	0.00	0.00	0.00	0.00	0.00	0.00
0.00	0.00	0.00	0.00	0.00	0.00	0.00	0.00	0.00
0.00	0.00	0.00	0.00	0.00	0.00	0.00	0.00	0.00
0.00	0.00	0.00	0.00	0.00	0.00	0.00	0.00	0.00
0.00	0.00	0.00	0.00	0.00	0.00	0.00	0.00	0.00
0.00	0.00	0.00	0.00	0.00	0.00	0.00	0.00	0.00
0.00	0.00	0.00	0.00	0.00	0.00	0.00	0.00	0.00
0.00	6.00	0.00	0.00	0.00	0.00	0.00	0.00	0.00
0.00	0.00	0.00	0.00	0.00	0.00	0.00	0.00	0.00
0.00	0.00	0.00	0.00	0.00	0.00	0.00	0.00	0.00
17.00	0.00	0.00	0.00	0.00	0.00	0.00	0.00	0.00
0.00	17.00	0.00	0.00	0.00	0.00	0.00	0.00	0.00
0.00	17.00	0.00	0.00	0.00	0.00	0.00	0.00	0.00
0.00	0.00	0.00	0.00	0.00	0.00	0.00	0.00	5.00
0.00	0.00	0.00	0.00	0.00	0.00	0.00	0.00	0.00
0.00	0.00	0.00	0.00	0.00	0.00	0.00	0.00	0.00
0.00	0.00	0.00	0.00	0.00	17.00	0.00	0.00	0.00
0.00	0.00	0.00	0.00	0.00	0.00	0.00	0.00	0.00
0.00	0.00	0.00	0.00	0.00	0.00	0.00	0.00	0.00
0.00	0.00	0.00	0.00	0.00	0.00	0.00	0.00	0.00
0.00	0.00	0.00	0.00	0.00	0.00	0.00	0.00	0.00
0.00	0.00	0.00	0.00	0.00	0.00	0.00	0.00	0.00
0.00	0.00	0.00	0.00	0.00	0.00	0.00	0.00	0.00
0.00	0.00	0.00	0.00	0.00	0.00	0.00	0.00	0.00
0.00	0.00	0.00	0.00	0.00	0.00	0.00	0.00	0.00
0.00	0.00	0.00	0.00	0.00	0.00	0.00	0.00	0.00
0.00	0.00	0.00	0.00	0.00	0.00	0.00	0.00	0.00
0.00	0.00	0.00	0.00	0.00	0.00	0.00	0.00	0.00
0.00	0.00	0.00	0.00	0.00	0.00	0.00	0.00	0.00
0.00	0.00	0.00	0.00	0.00	0.00	0.00	0.00	0.00
0.00	0.00	0.00	0.00	0.00	0.00	0.00	0.00	0.00

0.00	0.00	0.00	0.00	0.00	0.00	0.00	0.00	0.00
0.00	0.00	0.00	0.00	0.00	0.00	0.00	0.00	0.00
0.00	0.00	0.00	0.00	0.00	0.00	0.00	0.00	0.00
0.00	0.00	0.00	0.00	0.00	0.00	0.00	0.00	0.00
0.00	0.00	0.00	0.00	0.00	0.00	0.00	0.00	0.00
0.00	0.00	0.00	0.00	0.00	0.00	0.00	0.00	0.00
0.00	0.00	0.00	0.00	0.00	0.00	0.00	0.00	0.00
0.00	0.00	0.00	0.00	0.00	0.00	0.00	0.00	0.00
0.00	0.00	0.00	0.00	0.00	0.00	0.00	0.00	0.00
0.00	0.00	0.00	0.00	0.00	0.00	0.00	0.00	0.00
0.00	0.00	0.00	0.00	0.00	0.00	0.00	0.00	0.00
0.00	0.00	0.00	0.00	0.00	0.00	0.00	0.00	0.00
0.00	0.00	0.00	0.00	0.00	0.00	0.00	0.00	0.00
0.00	0.00	0.00	0.00	0.00	0.00	0.00	0.00	0.00
0.00	0.00	0.00	0.00	0.00	0.00	0.00	0.00	0.00
16.00	0.00	0.00	0.00	0.00	0.00	0.00	0.00	0.00
0.00	16.00	0.00	0.00	0.00	0.00	0.00	0.00	0.00
0.00	0.00	0.00	0.00	0.00	0.00	0.00	0.00	0.00
0.00	0.00	0.00	0.00	0.00	0.00	0.00	0.00	0.00
0.00	0.00	0.00	0.00	0.00	0.00	0.00	0.00	0.00
0.00	0.00	0.00	0.00	0.00	0.00	0.00	0.00	0.00
0.00	0.00	0.00	0.00	0.00	0.00	16.00	0.00	0.00
0.00	0.00	0.00	0.00	0.00	0.00	0.00	0.00	0.00
0.00	0.00	0.00	0.00	0.00	0.00	0.00	0.00	0.00
0.00	0.00	0.00	0.00	0.00	0.00	0.00	0.00	0.00
0.00	0.00	0.00	0.00	0.00	0.00	0.00	0.00	0.00
0.00	0.00	0.00	0.00	0.00	0.00	0.00	0.00	0.00
0.00	0.00	0.00	0.00	0.00	0.00	0.00	0.00	0.00
0.00	0.00	0.00	0.00	0.00	0.00	0.00	0.00	0.00
0.00	0.00	0.00	0.00	0.00	0.00	0.00	0.00	0.00
0.00	0.00	0.00	0.00	0.00	0.00	0.00	0.00	0.00
0.00	0.00	0.00	0.00	0.00	0.00	0.00	0.00	0.00
0.00	0.00	0.00	0.00	0.00	0.00	0.00	0.00	0.00
0.00	0.00	0.00	0.00	0.00	0.00	0.00	0.00	0.00
0.00	0.00	0.00	0.00	0.00	0.00	0.00	0.00	0.00
0.00	0.00	0.00	0.00	0.00	0.00	0.00	0.00	0.00
0.00	0.00	0.00	0.00	0.00	0.00	0.00	0.00	0.00

0.00	0.00	0.00	0.00	0.00	0.00	0.00	0.00	0.00
0.00	0.00	0.00	0.00	0.00	0.00	0.00	0.00	0.00
0.00	0.00	0.00	0.00	0.00	0.00	0.00	0.00	0.00
0.00	0.00	0.00	0.00	0.00	0.00	0.00	0.00	0.00
0.00	0.00	0.00	0.00	0.00	0.00	0.00	0.00	0.00
0.00	0.00	0.00	0.00	0.00	0.00	0.00	0.00	0.00
0.00	0.00	0.00	0.00	0.00	0.00	3.00	0.00	0.00
0.00	0.00	0.00	0.00	0.00	0.00	9.00	0.00	0.00
0.00	0.00	0.00	0.00	0.00	0.00	0.00	0.00	0.00
3.00	5.00	0.00	2.00	0.00	0.00	0.00	0.00	0.00
0.00	5.00	0.00	0.00	0.00	0.00	0.00	0.00	0.00
0.00	15.00	0.00	0.00	0.00	0.00	0.00	0.00	0.00
0.00	0.00	0.00	0.00	0.00	0.00	0.00	5.00	0.00
0.00	0.00	0.00	0.00	0.00	0.00	0.00	0.00	0.00
0.00	0.00	0.00	0.00	0.00	0.00	0.00	0.00	0.00
0.00	0.00	0.00	0.00	0.00	0.00	0.00	0.00	0.00
0.00	0.00	0.00	0.00	0.00	0.00	0.00	0.00	0.00
0.00	0.00	0.00	0.00	0.00	0.00	0.00	0.00	0.00
0.00	0.00	0.00	0.00	0.00	0.00	0.00	0.00	0.00
0.00	0.00	0.00	0.00	0.00	0.00	15.00	0.00	0.00
0.00	0.00	0.00	0.00	0.00	0.00	0.00	0.00	0.00
0.00	0.00	0.00	0.00	0.00	0.00	0.00	0.00	0.00
0.00	0.00	0.00	0.00	0.00	0.00	0.00	0.00	0.00
0.00	0.00	0.00	0.00	0.00	0.00	0.00	0.00	0.00
0.00	0.00	0.00	0.00	0.00	0.00	0.00	0.00	0.00
0.00	0.00	0.00	0.00	0.00	0.00	0.00	0.00	0.00
0.00	0.00	0.00	0.00	0.00	0.00	0.00	0.00	0.00
0.00	0.00	0.00	0.00	0.00	0.00	0.00	0.00	0.00
0.00	0.00	0.00	0.00	0.00	0.00	0.00	0.00	0.00
0.00	0.00	0.00	0.00	0.00	0.00	0.00	0.00	0.00
0.00	0.00	0.00	0.00	0.00	0.00	0.00	0.00	0.00
0.00	0.00	0.00	0.00	0.00	0.00	0.00	0.00	0.00
0.00	0.00	0.00	0.00	0.00	0.00	0.00	0.00	0.00
0.00	0.00	0.00	0.00	0.00	0.00	0.00	0.00	0.00
0.00	0.00	0.00	0.00	0.00	0.00	0.00	0.00	0.00
0.00	0.00	6.00	0.00	0.00	0.00	0.00	0.00	0.00
0.00	0.00	0.00	0.00	0.00	0.00	0.00	0.00	0.00

0.00	0.00	0.00	0.00	0.00	0.00	0.00	0.00	0.00
0.00	0.00	0.00	0.00	0.00	0.00	0.00	0.00	0.00
0.00	0.00	0.00	0.00	0.00	0.00	0.00	0.00	0.00
0.00	0.00	0.00	0.00	0.00	0.00	0.00	0.00	0.00
0.00	0.00	0.00	0.00	0.00	0.00	0.00	0.00	0.00
0.00	0.00	0.00	0.00	0.00	0.00	0.00	0.00	0.00
0.00	0.00	0.00	0.00	0.00	0.00	0.00	0.00	0.00
0.00	0.00	0.00	0.00	0.00	0.00	0.00	0.00	0.00
0.00	0.00	0.00	0.00	0.00	0.00	0.00	0.00	0.00
0.00	0.00	0.00	0.00	0.00	0.00	0.00	0.00	0.00
0.00	0.00	0.00	0.00	0.00	0.00	0.00	0.00	0.00
0.00	0.00	0.00	0.00	0.00	0.00	0.00	0.00	0.00
0.00	0.00	0.00	0.00	0.00	0.00	0.00	0.00	0.00
0.00	0.00	0.00	0.00	0.00	0.00	0.00	0.00	0.00
0.00	0.00	0.00	0.00	0.00	0.00	0.00	0.00	0.00
0.00	0.00	0.00	0.00	0.00	0.00	0.00	0.00	0.00
0.00	0.00	0.00	0.00	0.00	0.00	0.00	0.00	0.00
0.00	0.00	0.00	0.00	0.00	0.00	0.00	0.00	0.00
0.00	0.00	0.00	0.00	0.00	0.00	0.00	0.00	0.00
0.00	0.00	0.00	0.00	0.00	0.00	0.00	0.00	0.00
0.00	0.00	0.00	0.00	0.00	0.00	0.00	0.00	0.00
0.00	0.00	0.00	0.00	0.00	0.00	0.00	0.00	0.00
7.00	0.00	0.00	0.00	0.00	0.00	0.00	0.00	0.00
6.00	0.00	0.00	3.00	0.00	4.00	0.00	0.00	0.00
0.00	8.00	0.00	0.00	0.00	0.00	0.00	0.00	0.00
0.00	0.00	0.00	0.00	0.00	0.00	0.00	0.00	0.00
0.00	0.00	0.00	0.00	0.00	0.00	0.00	0.00	0.00
0.00	0.00	0.00	0.00	0.00	0.00	0.00	0.00	0.00
0.00	0.00	0.00	0.00	0.00	0.00	0.00	0.00	0.00
0.00	0.00	0.00	0.00	0.00	0.00	0.00	0.00	0.00
0.00	0.00	0.00	0.00	0.00	0.00	0.00	0.00	0.00
0.00	0.00	0.00	0.00	0.00	0.00	0.00	0.00	0.00
0.00	0.00	0.00	0.00	0.00	13.00	0.00	0.00	0.00
0.00	0.00	0.00	0.00	0.00	0.00	13.00	0.00	0.00
0.00	0.00	0.00	0.00	0.00	0.00	13.00	0.00	0.00
0.00	0.00	0.00	0.00	0.00	0.00	0.00	0.00	0.00
0.00	0.00	0.00	0.00	0.00	0.00	0.00	0.00	0.00

0.00	0.00	0.00	0.00	0.00	0.00	0.00	0.00	0.00
0.00	0.00	0.00	0.00	0.00	0.00	0.00	0.00	0.00
0.00	0.00	0.00	0.00	0.00	0.00	0.00	0.00	0.00
0.00	0.00	0.00	0.00	0.00	0.00	0.00	0.00	0.00
0.00	0.00	0.00	0.00	0.00	0.00	0.00	0.00	0.00
0.00	0.00	0.00	0.00	0.00	0.00	0.00	0.00	0.00
0.00	0.00	0.00	0.00	0.00	0.00	0.00	0.00	0.00
0.00	0.00	0.00	0.00	0.00	0.00	0.00	0.00	0.00
0.00	0.00	0.00	0.00	0.00	0.00	0.00	0.00	0.00
0.00	0.00	0.00	0.00	0.00	0.00	0.00	0.00	0.00
0.00	0.00	0.00	0.00	0.00	0.00	0.00	0.00	0.00
0.00	0.00	0.00	0.00	0.00	0.00	0.00	0.00	0.00
0.00	0.00	0.00	0.00	0.00	0.00	0.00	0.00	0.00
0.00	0.00	0.00	0.00	0.00	0.00	0.00	0.00	0.00
0.00	0.00	0.00	0.00	0.00	0.00	0.00	0.00	0.00
0.00	0.00	0.00	6.00	0.00	0.00	0.00	0.00	0.00
0.00	0.00	0.00	0.00	0.00	0.00	0.00	0.00	0.00
0.00	0.00	0.00	0.00	0.00	0.00	0.00	0.00	0.00
0.00	0.00	0.00	0.00	0.00	0.00	0.00	0.00	0.00
0.00	0.00	0.00	0.00	0.00	0.00	0.00	0.00	0.00
0.00	0.00	0.00	0.00	0.00	0.00	0.00	0.00	0.00
0.00	0.00	0.00	0.00	0.00	0.00	0.00	0.00	0.00
0.00	0.00	0.00	0.00	0.00	0.00	0.00	0.00	0.00
0.00	0.00	0.00	0.00	0.00	2.00	0.00	0.00	0.00
4.00	0.00	0.00	0.00	0.00	0.00	0.00	0.00	0.00
0.00	0.00	0.00	0.00	0.00	0.00	0.00	0.00	0.00
12.00	0.00	0.00	0.00	0.00	0.00	0.00	0.00	0.00
0.00	0.00	0.00	0.00	0.00	0.00	0.00	0.00	12.00
0.00	0.00	0.00	0.00	0.00	0.00	0.00	0.00	0.00
0.00	0.00	0.00	0.00	0.00	0.00	0.00	0.00	0.00
0.00	0.00	0.00	0.00	0.00	0.00	0.00	0.00	0.00
0.00	0.00	0.00	0.00	0.00	0.00	0.00	0.00	0.00
0.00	0.00	0.00	0.00	0.00	0.00	0.00	0.00	0.00
0.00	0.00	0.00	0.00	0.00	0.00	0.00	0.00	0.00
0.00	0.00	0.00	0.00	0.00	0.00	0.00	0.00	0.00
0.00	0.00	0.00	0.00	0.00	0.00	0.00	0.00	0.00
0.00	0.00	0.00	0.00	0.00	0.00	0.00	0.00	0.00

0.00	0.00	0.00	0.00	0.00	0.00	12.00	0.00	0.00
0.00	0.00	0.00	0.00	0.00	0.00	0.00	0.00	0.00
0.00	0.00	0.00	0.00	0.00	0.00	0.00	0.00	0.00
0.00	0.00	0.00	0.00	0.00	0.00	0.00	0.00	0.00
0.00	0.00	0.00	0.00	0.00	0.00	0.00	0.00	0.00
0.00	0.00	0.00	0.00	0.00	0.00	0.00	0.00	0.00
0.00	0.00	0.00	0.00	0.00	0.00	0.00	0.00	0.00
0.00	0.00	0.00	0.00	0.00	0.00	0.00	0.00	0.00
0.00	0.00	0.00	0.00	0.00	0.00	0.00	0.00	0.00
0.00	0.00	0.00	0.00	0.00	0.00	0.00	0.00	0.00
0.00	0.00	0.00	0.00	0.00	0.00	0.00	0.00	0.00
0.00	0.00	0.00	0.00	0.00	0.00	0.00	0.00	0.00
0.00	0.00	0.00	0.00	0.00	0.00	0.00	0.00	0.00
0.00	0.00	0.00	0.00	0.00	0.00	0.00	0.00	0.00
0.00	0.00	0.00	0.00	0.00	0.00	0.00	0.00	0.00
0.00	0.00	0.00	0.00	0.00	0.00	0.00	0.00	0.00
0.00	0.00	0.00	0.00	0.00	0.00	0.00	0.00	0.00
0.00	0.00	0.00	0.00	0.00	0.00	0.00	0.00	0.00
0.00	0.00	0.00	0.00	0.00	0.00	0.00	0.00	0.00
0.00	0.00	0.00	0.00	0.00	0.00	0.00	0.00	0.00
0.00	0.00	0.00	0.00	0.00	0.00	0.00	0.00	0.00
0.00	0.00	0.00	0.00	0.00	0.00	0.00	0.00	0.00
0.00	0.00	0.00	0.00	0.00	0.00	0.00	0.00	0.00
0.00	0.00	0.00	0.00	0.00	0.00	0.00	0.00	0.00
0.00	0.00	0.00	0.00	0.00	0.00	0.00	0.00	0.00
0.00	0.00	0.00	0.00	0.00	0.00	0.00	0.00	0.00
0.00	0.00	0.00	0.00	0.00	0.00	0.00	0.00	0.00
0.00	0.00	0.00	0.00	0.00	0.00	0.00	0.00	0.00
0.00	0.00	0.00	0.00	0.00	0.00	0.00	0.00	0.00
0.00	0.00	0.00	0.00	0.00	0.00	0.00	0.00	0.00
0.00	0.00	0.00	0.00	0.00	0.00	0.00	0.00	0.00
0.00	0.00	0.00	0.00	0.00	0.00	0.00	0.00	0.00
0.00	0.00	0.00	0.00	0.00	0.00	0.00	0.00	0.00
0.00	11.00	0.00	0.00	0.00	0.00	0.00	0.00	0.00
0.00	0.00	0.00	0.00	0.00	0.00	0.00	0.00	0.00
0.00	0.00	0.00	0.00	0.00	0.00	0.00	0.00	0.00
0.00	0.00	0.00	0.00	0.00	0.00	0.00	0.00	0.00

0.00	0.00	0.00	0.00	11.00	0.00	0.00	0.00	0.00
0.00	0.00	0.00	0.00	0.00	0.00	0.00	0.00	0.00
0.00	0.00	0.00	0.00	0.00	0.00	0.00	0.00	0.00
0.00	0.00	0.00	0.00	0.00	0.00	0.00	0.00	0.00
0.00	0.00	0.00	0.00	0.00	0.00	0.00	0.00	0.00
0.00	0.00	0.00	0.00	0.00	0.00	0.00	0.00	0.00
0.00	0.00	0.00	0.00	0.00	0.00	0.00	0.00	0.00
0.00	0.00	0.00	0.00	0.00	0.00	0.00	0.00	0.00
0.00	0.00	0.00	0.00	0.00	0.00	0.00	0.00	0.00
0.00	0.00	0.00	0.00	0.00	0.00	0.00	0.00	0.00
0.00	0.00	0.00	0.00	0.00	0.00	0.00	0.00	0.00
0.00	0.00	0.00	0.00	0.00	0.00	0.00	0.00	0.00
0.00	0.00	0.00	0.00	0.00	0.00	0.00	0.00	0.00
0.00	0.00	0.00	0.00	0.00	0.00	0.00	0.00	0.00
0.00	0.00	0.00	0.00	0.00	0.00	0.00	0.00	0.00
0.00	0.00	0.00	0.00	0.00	0.00	0.00	0.00	0.00
0.00	0.00	0.00	0.00	0.00	0.00	0.00	0.00	0.00
0.00	0.00	0.00	0.00	0.00	0.00	0.00	0.00	0.00
0.00	0.00	0.00	0.00	0.00	0.00	0.00	0.00	0.00
0.00	0.00	0.00	0.00	0.00	0.00	0.00	0.00	0.00
0.00	0.00	0.00	0.00	0.00	0.00	0.00	0.00	0.00
0.00	0.00	0.00	0.00	0.00	0.00	0.00	0.00	0.00
0.00	0.00	0.00	0.00	0.00	0.00	0.00	0.00	0.00
0.00	0.00	0.00	0.00	0.00	0.00	0.00	0.00	0.00
0.00	0.00	0.00	0.00	0.00	0.00	0.00	0.00	0.00
0.00	0.00	0.00	0.00	0.00	0.00	0.00	0.00	0.00
0.00	0.00	0.00	0.00	0.00	0.00	0.00	0.00	0.00
0.00	0.00	0.00	0.00	0.00	0.00	0.00	0.00	0.00
0.00	0.00	0.00	0.00	0.00	0.00	0.00	0.00	0.00
0.00	0.00	0.00	0.00	0.00	0.00	0.00	0.00	0.00
0.00	0.00	0.00	0.00	0.00	0.00	0.00	0.00	0.00
0.00	0.00	0.00	0.00	0.00	0.00	0.00	0.00	0.00
0.00	0.00	0.00	0.00	0.00	0.00	0.00	0.00	0.00
0.00	0.00	0.00	0.00	0.00	0.00	0.00	0.00	0.00
0.00	0.00	0.00	0.00	0.00	0.00	0.00	0.00	0.00
0.00	0.00	0.00	0.00	0.00	0.00	0.00	0.00	0.00
0.00	0.00	0.00	0.00	0.00	0.00	0.00	0.00	0.00

0.00	10.00	0.00	0.00	0.00	0.00	0.00	0.00	0.00
0.00	3.00	0.00	0.00	0.00	0.00	0.00	0.00	0.00
0.00	10.00	0.00	0.00	0.00	0.00	0.00	0.00	0.00
0.00	0.00	0.00	0.00	0.00	0.00	0.00	0.00	0.00
0.00	0.00	0.00	0.00	0.00	0.00	0.00	0.00	0.00
0.00	0.00	0.00	0.00	0.00	0.00	0.00	0.00	0.00
0.00	0.00	0.00	0.00	0.00	0.00	0.00	0.00	0.00
0.00	0.00	0.00	0.00	0.00	0.00	0.00	0.00	0.00
0.00	0.00	0.00	0.00	0.00	0.00	0.00	0.00	0.00
0.00	0.00	0.00	0.00	0.00	0.00	0.00	0.00	0.00
0.00	0.00	10.00	0.00	0.00	0.00	0.00	0.00	0.00
0.00	0.00	0.00	10.00	0.00	0.00	0.00	0.00	0.00
0.00	0.00	0.00	0.00	0.00	0.00	0.00	0.00	0.00
0.00	0.00	0.00	0.00	0.00	0.00	0.00	0.00	0.00
0.00	0.00	0.00	0.00	0.00	0.00	0.00	0.00	0.00
0.00	0.00	0.00	0.00	0.00	0.00	0.00	0.00	0.00
0.00	0.00	0.00	0.00	0.00	0.00	0.00	0.00	0.00
0.00	0.00	0.00	0.00	0.00	0.00	0.00	0.00	0.00
0.00	0.00	0.00	0.00	0.00	0.00	0.00	0.00	0.00
0.00	0.00	0.00	0.00	0.00	0.00	0.00	0.00	0.00
0.00	0.00	0.00	0.00	0.00	0.00	0.00	0.00	0.00
0.00	0.00	0.00	0.00	0.00	0.00	0.00	0.00	0.00
0.00	0.00	0.00	0.00	0.00	0.00	0.00	0.00	0.00
0.00	0.00	0.00	0.00	0.00	0.00	0.00	0.00	0.00
0.00	0.00	0.00	0.00	0.00	0.00	0.00	0.00	0.00
0.00	0.00	0.00	0.00	0.00	0.00	0.00	0.00	0.00
0.00	0.00	0.00	0.00	0.00	0.00	0.00	0.00	0.00
0.00	0.00	0.00	0.00	0.00	0.00	0.00	0.00	0.00
0.00	0.00	0.00	0.00	0.00	0.00	0.00	0.00	0.00
0.00	0.00	0.00	0.00	0.00	0.00	0.00	0.00	0.00
0.00	0.00	0.00	0.00	0.00	0.00	0.00	0.00	0.00
0.00	0.00	0.00	0.00	0.00	0.00	0.00	0.00	0.00
0.00	0.00	0.00	0.00	0.00	0.00	0.00	0.00	0.00
0.00	0.00	0.00	0.00	0.00	0.00	0.00	0.00	0.00
0.00	0.00	0.00	0.00	0.00	0.00	0.00	0.00	0.00
0.00	0.00	0.00	0.00	0.00	0.00	0.00	0.00	0.00
0.00	0.00	0.00	0.00	0.00	0.00	0.00	0.00	0.00

0.00	0.00	0.00	0.00	0.00	0.00	0.00	0.00	0.00
0.00	0.00	0.00	0.00	0.00	0.00	0.00	0.00	0.00
0.00	0.00	0.00	0.00	0.00	0.00	0.00	0.00	0.00
0.00	0.00	0.00	0.00	0.00	0.00	0.00	0.00	0.00
0.00	0.00	0.00	0.00	0.00	0.00	0.00	0.00	0.00
0.00	0.00	0.00	0.00	0.00	0.00	0.00	0.00	0.00
0.00	0.00	0.00	0.00	0.00	0.00	0.00	0.00	0.00
0.00	0.00	0.00	0.00	0.00	0.00	0.00	0.00	0.00
0.00	0.00	0.00	0.00	0.00	0.00	0.00	0.00	0.00
0.00	0.00	0.00	0.00	0.00	0.00	0.00	0.00	0.00
0.00	0.00	0.00	0.00	0.00	0.00	0.00	0.00	0.00
0.00	0.00	0.00	0.00	0.00	0.00	0.00	0.00	0.00
0.00	0.00	0.00	0.00	0.00	0.00	0.00	0.00	0.00
0.00	0.00	0.00	0.00	0.00	0.00	0.00	0.00	0.00
0.00	6.00	0.00	0.00	0.00	0.00	0.00	0.00	0.00
0.00	0.00	0.00	0.00	0.00	0.00	0.00	0.00	0.00
9.00	0.00	0.00	0.00	0.00	0.00	0.00	0.00	0.00
0.00	9.00	0.00	0.00	0.00	0.00	0.00	0.00	0.00
0.00	9.00	0.00	0.00	0.00	0.00	0.00	0.00	0.00
0.00	0.00	0.00	0.00	0.00	0.00	0.00	0.00	9.00
0.00	0.00	0.00	0.00	0.00	0.00	0.00	0.00	0.00
0.00	0.00	0.00	0.00	0.00	0.00	0.00	0.00	0.00
0.00	0.00	0.00	0.00	0.00	0.00	0.00	0.00	0.00
0.00	0.00	0.00	0.00	0.00	0.00	0.00	0.00	0.00
0.00	0.00	0.00	0.00	0.00	0.00	0.00	0.00	0.00
0.00	0.00	0.00	0.00	0.00	0.00	0.00	0.00	0.00
0.00	0.00	0.00	0.00	0.00	0.00	0.00	0.00	0.00
0.00	0.00	0.00	0.00	0.00	0.00	0.00	0.00	0.00
0.00	0.00	0.00	0.00	0.00	0.00	0.00	0.00	0.00
0.00	0.00	0.00	0.00	0.00	0.00	0.00	0.00	0.00
0.00	0.00	3.00	0.00	0.00	0.00	6.00	0.00	0.00
0.00	0.00	0.00	0.00	0.00	0.00	5.00	4.00	0.00
0.00	0.00	0.00	0.00	0.00	0.00	0.00	9.00	0.00
0.00	0.00	0.00	0.00	0.00	0.00	0.00	0.00	0.00
0.00	0.00	0.00	0.00	0.00	0.00	0.00	0.00	0.00
0.00	0.00	0.00	0.00	0.00	0.00	0.00	0.00	0.00
0.00	0.00	0.00	0.00	0.00	0.00	0.00	0.00	0.00

0.00	0.00	0.00	0.00	0.00	0.00	0.00	0.00	0.00
0.00	0.00	0.00	0.00	0.00	0.00	0.00	0.00	0.00
0.00	0.00	0.00	0.00	0.00	0.00	0.00	0.00	0.00
0.00	0.00	0.00	0.00	0.00	0.00	0.00	0.00	0.00
0.00	0.00	0.00	0.00	0.00	0.00	0.00	0.00	0.00
0.00	0.00	0.00	0.00	0.00	0.00	0.00	0.00	0.00
0.00	0.00	0.00	0.00	0.00	0.00	0.00	0.00	0.00
0.00	0.00	0.00	0.00	0.00	0.00	0.00	0.00	0.00
0.00	0.00	0.00	0.00	0.00	0.00	0.00	0.00	0.00
0.00	0.00	0.00	0.00	0.00	0.00	0.00	0.00	0.00
0.00	0.00	0.00	0.00	0.00	0.00	0.00	0.00	0.00
0.00	0.00	0.00	0.00	0.00	0.00	0.00	0.00	0.00
0.00	0.00	0.00	0.00	0.00	0.00	0.00	0.00	0.00
0.00	0.00	0.00	0.00	0.00	0.00	0.00	0.00	0.00
0.00	0.00	0.00	0.00	0.00	0.00	0.00	0.00	0.00
0.00	0.00	0.00	0.00	0.00	0.00	0.00	0.00	0.00
0.00	0.00	0.00	0.00	0.00	0.00	0.00	0.00	0.00
0.00	0.00	0.00	0.00	0.00	0.00	0.00	0.00	0.00
0.00	0.00	0.00	0.00	0.00	0.00	0.00	0.00	0.00
0.00	0.00	0.00	0.00	0.00	0.00	0.00	0.00	0.00
0.00	0.00	0.00	0.00	0.00	0.00	0.00	0.00	0.00
0.00	0.00	0.00	0.00	0.00	0.00	0.00	0.00	0.00
0.00	0.00	0.00	0.00	0.00	0.00	0.00	0.00	0.00
0.00	0.00	0.00	0.00	0.00	0.00	0.00	0.00	0.00
0.00	0.00	0.00	0.00	0.00	0.00	0.00	0.00	0.00
0.00	0.00	0.00	0.00	0.00	0.00	0.00	0.00	0.00
0.00	0.00	0.00	0.00	0.00	0.00	0.00	0.00	0.00
0.00	0.00	0.00	0.00	0.00	0.00	0.00	0.00	0.00
0.00	0.00	0.00	0.00	0.00	0.00	0.00	0.00	0.00
0.00	0.00	0.00	0.00	0.00	0.00	6.00	0.00	0.00
0.00	0.00	0.00	0.00	0.00	0.00	0.00	0.00	0.00
0.00	0.00	0.00	0.00	0.00	0.00	0.00	0.00	0.00
0.00	0.00	0.00	0.00	0.00	0.00	0.00	0.00	0.00
0.00	0.00	0.00	0.00	0.00	0.00	0.00	0.00	0.00
0.00	0.00	0.00	0.00	0.00	0.00	0.00	0.00	0.00
0.00	0.00	0.00	0.00	0.00	0.00	0.00	0.00	0.00
0.00	0.00	0.00	0.00	0.00	0.00	0.00	0.00	0.00

0.00	0.00	0.00	0.00	0.00	0.00	0.00	0.00	0.00
0.00	0.00	0.00	0.00	0.00	0.00	0.00	0.00	0.00
5.00	0.00	0.00	0.00	0.00	0.00	0.00	0.00	0.00
4.00	0.00	0.00	0.00	0.00	0.00	0.00	0.00	0.00
0.00	0.00	0.00	0.00	0.00	0.00	0.00	0.00	0.00
4.00	0.00	0.00	0.00	0.00	0.00	2.00	0.00	0.00
0.00	0.00	0.00	0.00	0.00	0.00	0.00	0.00	0.00
0.00	0.00	0.00	0.00	0.00	0.00	0.00	0.00	0.00
0.00	0.00	0.00	0.00	0.00	0.00	0.00	0.00	0.00
0.00	0.00	0.00	0.00	0.00	0.00	0.00	0.00	0.00
0.00	0.00	0.00	0.00	0.00	0.00	0.00	0.00	0.00
0.00	0.00	0.00	0.00	0.00	0.00	0.00	0.00	0.00
0.00	0.00	0.00	0.00	0.00	0.00	0.00	0.00	0.00
0.00	0.00	0.00	0.00	0.00	8.00	0.00	0.00	0.00
0.00	0.00	0.00	0.00	0.00	0.00	0.00	0.00	0.00
0.00	0.00	0.00	0.00	0.00	0.00	0.00	0.00	0.00
0.00	0.00	0.00	0.00	0.00	0.00	0.00	0.00	0.00
0.00	0.00	0.00	0.00	0.00	0.00	0.00	0.00	0.00
0.00	0.00	0.00	0.00	0.00	0.00	0.00	0.00	0.00
0.00	0.00	0.00	0.00	0.00	0.00	0.00	0.00	0.00
0.00	0.00	0.00	0.00	0.00	0.00	0.00	0.00	0.00
0.00	0.00	0.00	0.00	0.00	0.00	0.00	0.00	0.00
0.00	0.00	0.00	0.00	0.00	0.00	0.00	0.00	0.00
0.00	0.00	0.00	0.00	0.00	0.00	0.00	0.00	0.00
0.00	0.00	0.00	0.00	0.00	0.00	0.00	0.00	0.00
0.00	0.00	0.00	0.00	0.00	0.00	0.00	0.00	0.00
0.00	0.00	0.00	0.00	0.00	0.00	0.00	0.00	0.00
0.00	0.00	0.00	0.00	0.00	0.00	0.00	0.00	0.00
0.00	0.00	0.00	0.00	0.00	0.00	0.00	0.00	0.00
0.00	0.00	0.00	0.00	0.00	0.00	0.00	0.00	0.00
0.00	0.00	0.00	0.00	0.00	0.00	0.00	0.00	0.00
0.00	0.00	0.00	0.00	0.00	0.00	0.00	0.00	0.00
0.00	0.00	0.00	0.00	0.00	0.00	0.00	0.00	0.00
0.00	0.00	0.00	0.00	0.00	0.00	0.00	0.00	0.00
0.00	0.00	0.00	0.00	0.00	0.00	0.00	0.00	0.00
0.00	0.00	0.00	0.00	0.00	0.00	0.00	0.00	0.00
0.00	0.00	0.00	0.00	0.00	0.00	0.00	0.00	0.00

0.00	0.00	0.00	0.00	0.00	0.00	0.00	0.00	0.00
0.00	0.00	0.00	0.00	0.00	0.00	0.00	0.00	0.00
0.00	0.00	0.00	0.00	0.00	0.00	0.00	0.00	0.00
0.00	0.00	0.00	0.00	0.00	0.00	0.00	0.00	0.00
0.00	0.00	0.00	0.00	0.00	0.00	0.00	0.00	0.00
0.00	0.00	0.00	0.00	0.00	0.00	0.00	0.00	0.00
0.00	0.00	0.00	0.00	0.00	0.00	0.00	0.00	0.00
0.00	0.00	0.00	0.00	0.00	0.00	0.00	0.00	0.00
0.00	0.00	0.00	0.00	0.00	0.00	0.00	0.00	0.00
0.00	0.00	0.00	0.00	0.00	0.00	0.00	0.00	0.00
0.00	0.00	0.00	0.00	0.00	0.00	0.00	0.00	0.00
0.00	0.00	0.00	0.00	0.00	0.00	0.00	0.00	0.00
3.00	0.00	0.00	0.00	0.00	0.00	0.00	0.00	0.00
0.00	0.00	0.00	0.00	0.00	0.00	0.00	0.00	0.00
7.00	0.00	0.00	0.00	0.00	0.00	0.00	0.00	0.00
0.00	0.00	0.00	3.00	0.00	0.00	0.00	0.00	0.00
0.00	0.00	0.00	0.00	0.00	0.00	0.00	0.00	0.00
0.00	0.00	0.00	0.00	0.00	0.00	0.00	0.00	0.00
0.00	0.00	0.00	0.00	0.00	0.00	0.00	0.00	0.00
0.00	0.00	0.00	0.00	0.00	0.00	0.00	0.00	0.00
0.00	0.00	0.00	0.00	0.00	0.00	0.00	0.00	0.00
0.00	0.00	0.00	0.00	0.00	0.00	0.00	0.00	0.00
0.00	0.00	0.00	0.00	0.00	0.00	0.00	0.00	0.00
0.00	0.00	0.00	0.00	0.00	0.00	0.00	0.00	0.00
0.00	0.00	0.00	0.00	0.00	0.00	0.00	0.00	0.00
0.00	0.00	7.00	0.00	0.00	0.00	0.00	0.00	0.00
0.00	0.00	7.00	0.00	0.00	0.00	0.00	0.00	0.00
0.00	0.00	7.00	0.00	0.00	0.00	0.00	0.00	0.00
0.00	0.00	0.00	7.00	0.00	0.00	0.00	0.00	0.00
0.00	0.00	0.00	7.00	0.00	0.00	0.00	0.00	0.00
0.00	0.00	0.00	0.00	0.00	7.00	0.00	0.00	0.00
0.00	0.00	0.00	0.00	0.00	0.00	7.00	0.00	0.00
0.00	0.00	0.00	0.00	0.00	0.00	0.00	0.00	0.00
0.00	0.00	0.00	0.00	0.00	0.00	0.00	0.00	0.00
0.00	0.00	0.00	0.00	0.00	0.00	0.00	0.00	0.00
0.00	0.00	0.00	0.00	0.00	0.00	0.00	0.00	0.00
0.00	0.00	0.00	0.00	0.00	0.00	0.00	0.00	0.00

0.00	0.00	0.00	0.00	0.00	0.00	0.00	0.00	0.00
0.00	0.00	0.00	0.00	0.00	0.00	0.00	0.00	0.00
0.00	0.00	0.00	0.00	0.00	0.00	0.00	0.00	0.00
0.00	0.00	0.00	0.00	0.00	0.00	0.00	0.00	0.00
0.00	0.00	0.00	0.00	0.00	0.00	0.00	0.00	0.00
0.00	0.00	0.00	0.00	0.00	0.00	0.00	0.00	0.00
0.00	0.00	0.00	0.00	0.00	0.00	0.00	0.00	0.00
0.00	0.00	0.00	0.00	0.00	0.00	0.00	0.00	0.00
0.00	0.00	0.00	0.00	0.00	0.00	0.00	0.00	0.00
0.00	0.00	0.00	0.00	0.00	0.00	0.00	0.00	0.00
0.00	0.00	0.00	0.00	0.00	0.00	0.00	0.00	0.00
0.00	0.00	0.00	0.00	0.00	0.00	0.00	0.00	0.00
0.00	0.00	0.00	0.00	0.00	0.00	0.00	0.00	0.00
0.00	0.00	0.00	0.00	0.00	0.00	0.00	0.00	0.00
0.00	0.00	0.00	0.00	0.00	0.00	0.00	0.00	0.00
0.00	0.00	0.00	0.00	0.00	0.00	0.00	0.00	0.00
0.00	0.00	0.00	0.00	0.00	0.00	0.00	0.00	0.00
0.00	0.00	0.00	0.00	0.00	0.00	0.00	0.00	0.00
0.00	0.00	0.00	0.00	0.00	0.00	0.00	0.00	0.00
0.00	0.00	0.00	0.00	0.00	0.00	0.00	0.00	0.00
0.00	0.00	0.00	0.00	0.00	0.00	0.00	0.00	0.00
0.00	0.00	0.00	0.00	0.00	0.00	0.00	0.00	0.00
0.00	0.00	0.00	0.00	0.00	0.00	0.00	0.00	0.00
0.00	0.00	0.00	0.00	0.00	0.00	0.00	0.00	0.00
0.00	0.00	0.00	0.00	0.00	0.00	0.00	0.00	0.00
0.00	0.00	0.00	0.00	0.00	0.00	0.00	0.00	0.00
0.00	0.00	0.00	0.00	0.00	0.00	0.00	0.00	0.00
0.00	0.00	0.00	0.00	0.00	0.00	0.00	0.00	0.00
0.00	0.00	0.00	0.00	0.00	0.00	0.00	0.00	0.00
0.00	0.00	0.00	0.00	0.00	0.00	0.00	0.00	0.00
0.00	0.00	0.00	0.00	0.00	0.00	0.00	0.00	0.00
0.00	0.00	0.00	0.00	0.00	0.00	0.00	0.00	0.00
0.00	0.00	0.00	0.00	0.00	0.00	0.00	0.00	0.00
0.00	0.00	0.00	0.00	0.00	0.00	0.00	0.00	0.00
6.00	0.00	0.00	0.00	0.00	0.00	0.00	0.00	0.00
2.00	0.00	0.00	0.00	0.00	0.00	0.00	4.00	0.00
6.00	0.00	0.00	0.00	0.00	0.00	0.00	0.00	0.00

6.00	0.00	0.00	0.00	0.00	0.00	0.00	0.00	0.00
6.00	0.00	0.00	0.00	0.00	0.00	0.00	0.00	0.00
0.00	6.00	0.00	0.00	0.00	0.00	0.00	0.00	0.00
0.00	6.00	0.00	0.00	0.00	0.00	0.00	0.00	0.00
0.00	6.00	0.00	0.00	0.00	0.00	0.00	0.00	0.00
0.00	6.00	0.00	0.00	0.00	0.00	0.00	0.00	0.00
0.00	0.00	0.00	0.00	0.00	0.00	0.00	0.00	6.00
0.00	0.00	0.00	0.00	0.00	0.00	0.00	0.00	0.00
0.00	0.00	0.00	0.00	0.00	0.00	3.00	0.00	0.00
0.00	0.00	0.00	0.00	0.00	0.00	0.00	0.00	0.00
0.00	0.00	0.00	0.00	0.00	0.00	0.00	0.00	0.00
0.00	0.00	0.00	0.00	0.00	0.00	0.00	0.00	0.00
0.00	0.00	0.00	0.00	0.00	0.00	0.00	0.00	0.00
0.00	0.00	0.00	0.00	0.00	0.00	0.00	0.00	0.00
0.00	0.00	0.00	0.00	0.00	0.00	0.00	0.00	0.00
0.00	0.00	0.00	0.00	0.00	0.00	0.00	0.00	0.00
0.00	0.00	0.00	0.00	0.00	0.00	0.00	0.00	0.00
0.00	0.00	0.00	0.00	0.00	0.00	0.00	0.00	0.00
0.00	0.00	0.00	0.00	0.00	0.00	0.00	0.00	0.00
0.00	0.00	0.00	0.00	0.00	0.00	0.00	0.00	0.00
0.00	0.00	0.00	0.00	0.00	0.00	0.00	0.00	0.00
0.00	0.00	0.00	0.00	6.00	0.00	0.00	0.00	0.00
0.00	0.00	0.00	0.00	0.00	6.00	0.00	0.00	0.00
0.00	0.00	0.00	0.00	0.00	0.00	3.00	3.00	0.00
0.00	0.00	0.00	0.00	0.00	0.00	0.00	0.00	0.00
0.00	0.00	0.00	0.00	0.00	0.00	0.00	0.00	0.00
0.00	0.00	0.00	0.00	0.00	0.00	0.00	0.00	0.00
0.00	0.00	0.00	0.00	0.00	0.00	0.00	0.00	0.00
0.00	0.00	0.00	0.00	0.00	0.00	0.00	0.00	0.00
0.00	0.00	0.00	0.00	0.00	0.00	0.00	0.00	0.00
0.00	0.00	0.00	0.00	0.00	0.00	0.00	0.00	0.00
0.00	0.00	0.00	0.00	0.00	0.00	0.00	0.00	0.00
0.00	0.00	0.00	0.00	0.00	0.00	0.00	0.00	0.00
0.00	0.00	0.00	0.00	0.00	0.00	0.00	0.00	0.00
0.00	0.00	0.00	0.00	0.00	0.00	0.00	0.00	0.00
0.00	0.00	0.00	0.00	0.00	0.00	0.00	0.00	0.00
0.00	0.00	0.00	0.00	0.00	0.00	0.00	0.00	0.00

0.00	0.00	0.00	0.00	0.00	0.00	0.00	0.00	0.00
0.00	0.00	0.00	0.00	0.00	0.00	0.00	0.00	0.00
0.00	0.00	0.00	0.00	0.00	0.00	0.00	0.00	0.00
0.00	0.00	0.00	0.00	0.00	0.00	0.00	0.00	0.00
0.00	0.00	0.00	0.00	0.00	0.00	0.00	0.00	0.00
0.00	0.00	0.00	0.00	0.00	0.00	0.00	0.00	0.00
0.00	0.00	0.00	0.00	0.00	0.00	0.00	0.00	0.00
0.00	0.00	0.00	0.00	0.00	0.00	0.00	0.00	0.00
0.00	0.00	0.00	0.00	0.00	0.00	0.00	0.00	0.00
0.00	0.00	0.00	0.00	0.00	0.00	0.00	0.00	0.00
0.00	0.00	0.00	0.00	0.00	0.00	0.00	0.00	0.00
0.00	0.00	0.00	0.00	0.00	0.00	0.00	0.00	0.00
0.00	0.00	0.00	0.00	0.00	0.00	0.00	0.00	0.00
0.00	0.00	0.00	0.00	0.00	0.00	0.00	0.00	0.00
0.00	0.00	0.00	0.00	0.00	0.00	0.00	0.00	0.00
0.00	0.00	0.00	0.00	0.00	0.00	0.00	0.00	0.00
0.00	0.00	0.00	0.00	0.00	0.00	0.00	0.00	0.00
0.00	0.00	0.00	0.00	0.00	0.00	0.00	0.00	0.00
0.00	0.00	0.00	0.00	0.00	0.00	0.00	0.00	0.00
0.00	0.00	0.00	0.00	0.00	0.00	0.00	0.00	0.00
0.00	0.00	0.00	0.00	0.00	0.00	0.00	0.00	0.00
0.00	0.00	0.00	0.00	0.00	0.00	0.00	0.00	0.00
0.00	0.00	0.00	0.00	0.00	0.00	0.00	0.00	0.00
0.00	0.00	0.00	0.00	0.00	0.00	0.00	0.00	0.00
0.00	0.00	0.00	0.00	0.00	0.00	0.00	0.00	0.00
0.00	0.00	0.00	0.00	0.00	0.00	0.00	0.00	0.00
0.00	0.00	0.00	0.00	0.00	0.00	0.00	0.00	0.00
0.00	0.00	0.00	0.00	0.00	0.00	0.00	0.00	0.00
0.00	0.00	0.00	0.00	0.00	0.00	0.00	0.00	0.00
0.00	0.00	0.00	0.00	0.00	0.00	0.00	0.00	0.00
0.00	0.00	0.00	0.00	0.00	0.00	0.00	0.00	0.00
0.00	0.00	0.00	0.00	0.00	0.00	0.00	0.00	0.00
0.00	0.00	0.00	0.00	0.00	0.00	0.00	0.00	0.00
0.00	0.00	0.00	0.00	0.00	0.00	0.00	0.00	0.00
0.00	0.00	0.00	0.00	0.00	0.00	0.00	0.00	0.00
0.00	0.00	0.00	0.00	0.00	0.00	0.00	0.00	0.00
0.00	0.00	0.00	0.00	0.00	0.00	0.00	0.00	0.00

0.00	0.00	0.00	0.00	0.00	0.00	0.00	0.00	0.00
0.00	0.00	0.00	0.00	0.00	0.00	0.00	0.00	0.00
0.00	0.00	0.00	0.00	0.00	0.00	0.00	0.00	0.00
0.00	0.00	0.00	0.00	0.00	0.00	0.00	0.00	0.00
0.00	0.00	0.00	0.00	0.00	0.00	0.00	0.00	0.00
0.00	0.00	0.00	0.00	0.00	0.00	0.00	0.00	0.00
0.00	0.00	0.00	0.00	0.00	0.00	0.00	0.00	0.00
0.00	2.00	0.00	0.00	0.00	0.00	0.00	0.00	0.00
0.00	0.00	0.00	0.00	0.00	0.00	0.00	0.00	0.00
0.00	0.00	0.00	0.00	0.00	0.00	0.00	0.00	0.00
0.00	0.00	0.00	0.00	0.00	0.00	0.00	0.00	2.00
5.00	0.00	0.00	0.00	0.00	0.00	0.00	0.00	0.00
0.00	5.00	0.00	0.00	0.00	0.00	0.00	0.00	0.00
0.00	5.00	0.00	0.00	0.00	0.00	0.00	0.00	0.00
0.00	0.00	0.00	0.00	0.00	0.00	0.00	0.00	2.00
0.00	0.00	0.00	0.00	0.00	0.00	0.00	0.00	5.00
0.00	0.00	0.00	0.00	0.00	0.00	0.00	0.00	0.00
0.00	0.00	0.00	0.00	0.00	0.00	0.00	0.00	0.00
0.00	0.00	0.00	0.00	0.00	0.00	0.00	0.00	0.00
0.00	0.00	0.00	0.00	0.00	0.00	0.00	0.00	0.00
0.00	0.00	0.00	0.00	0.00	0.00	0.00	0.00	0.00
0.00	0.00	0.00	0.00	0.00	0.00	0.00	0.00	0.00
0.00	0.00	0.00	0.00	0.00	0.00	0.00	0.00	0.00
0.00	0.00	0.00	0.00	0.00	0.00	0.00	0.00	0.00
0.00	0.00	0.00	0.00	0.00	0.00	0.00	0.00	0.00
0.00	0.00	0.00	5.00	0.00	0.00	0.00	0.00	0.00
0.00	0.00	0.00	0.00	0.00	0.00	5.00	0.00	0.00
0.00	0.00	0.00	0.00	0.00	0.00	5.00	0.00	0.00
0.00	0.00	0.00	0.00	0.00	0.00	5.00	0.00	0.00
0.00	0.00	0.00	0.00	0.00	0.00	5.00	0.00	0.00
0.00	0.00	0.00	0.00	0.00	0.00	5.00	0.00	0.00
0.00	0.00	0.00	0.00	0.00	0.00	0.00	5.00	0.00
0.00	0.00	0.00	0.00	0.00	0.00	0.00	5.00	0.00
0.00	0.00	0.00	0.00	0.00	0.00	0.00	0.00	0.00
0.00	0.00	0.00	0.00	0.00	0.00	0.00	0.00	0.00
0.00	0.00	0.00	0.00	0.00	0.00	0.00	0.00	0.00
0.00	0.00	0.00	0.00	0.00	0.00	0.00	0.00	0.00

0.00	0.00	0.00	0.00	0.00	0.00	0.00	0.00	0.00
0.00	0.00	0.00	0.00	0.00	0.00	0.00	0.00	0.00
0.00	0.00	0.00	0.00	0.00	0.00	0.00	0.00	0.00
0.00	0.00	0.00	0.00	0.00	0.00	0.00	0.00	0.00
0.00	0.00	0.00	0.00	0.00	0.00	0.00	0.00	0.00
0.00	0.00	0.00	0.00	0.00	0.00	0.00	0.00	0.00
0.00	0.00	0.00	0.00	0.00	0.00	0.00	0.00	0.00
0.00	0.00	0.00	0.00	0.00	0.00	0.00	0.00	0.00
0.00	0.00	0.00	0.00	0.00	0.00	0.00	0.00	0.00
0.00	0.00	0.00	0.00	0.00	0.00	0.00	0.00	0.00
0.00	0.00	0.00	0.00	0.00	0.00	0.00	0.00	0.00
0.00	0.00	0.00	0.00	0.00	0.00	0.00	0.00	0.00
0.00	0.00	0.00	0.00	0.00	0.00	0.00	0.00	0.00
0.00	0.00	0.00	0.00	0.00	0.00	0.00	0.00	0.00
0.00	0.00	0.00	0.00	0.00	0.00	0.00	0.00	0.00
0.00	0.00	0.00	0.00	0.00	0.00	0.00	0.00	0.00
0.00	0.00	0.00	0.00	0.00	0.00	0.00	0.00	0.00
0.00	0.00	0.00	0.00	0.00	0.00	0.00	0.00	0.00
0.00	0.00	0.00	0.00	0.00	0.00	0.00	0.00	0.00
0.00	0.00	0.00	0.00	0.00	0.00	0.00	0.00	0.00
0.00	0.00	0.00	0.00	0.00	0.00	0.00	0.00	0.00
0.00	0.00	0.00	0.00	0.00	0.00	0.00	0.00	0.00
0.00	0.00	0.00	0.00	0.00	0.00	0.00	0.00	0.00
0.00	0.00	0.00	0.00	0.00	0.00	0.00	0.00	0.00
0.00	0.00	0.00	0.00	0.00	0.00	0.00	0.00	0.00
0.00	0.00	0.00	0.00	0.00	0.00	0.00	0.00	0.00
0.00	0.00	0.00	0.00	0.00	0.00	0.00	0.00	0.00
0.00	0.00	0.00	0.00	0.00	0.00	0.00	0.00	0.00
0.00	0.00	0.00	0.00	0.00	0.00	0.00	0.00	0.00
0.00	0.00	0.00	0.00	0.00	0.00	0.00	0.00	0.00
0.00	0.00	0.00	0.00	0.00	0.00	0.00	0.00	0.00
0.00	0.00	0.00	0.00	0.00	0.00	0.00	0.00	0.00
0.00	0.00	0.00	0.00	0.00	0.00	0.00	0.00	0.00
0.00	0.00	0.00	0.00	0.00	0.00	0.00	0.00	0.00
0.00	0.00	0.00	0.00	0.00	0.00	0.00	0.00	0.00
0.00	0.00	0.00	0.00	0.00	0.00	0.00	0.00	0.00

0.00	0.00	0.00	0.00	0.00	0.00	0.00	0.00	0.00
0.00	0.00	0.00	0.00	0.00	0.00	0.00	0.00	0.00
0.00	0.00	0.00	0.00	0.00	0.00	0.00	0.00	0.00
0.00	0.00	0.00	0.00	0.00	0.00	0.00	0.00	0.00
0.00	0.00	0.00	0.00	0.00	0.00	0.00	0.00	0.00
0.00	0.00	0.00	0.00	0.00	0.00	0.00	0.00	0.00
0.00	0.00	0.00	0.00	0.00	0.00	0.00	0.00	0.00
0.00	0.00	0.00	0.00	0.00	0.00	0.00	0.00	0.00
0.00	0.00	0.00	0.00	0.00	0.00	0.00	0.00	0.00
0.00	0.00	0.00	0.00	0.00	0.00	0.00	0.00	0.00
0.00	0.00	0.00	0.00	0.00	0.00	0.00	0.00	0.00
0.00	0.00	0.00	0.00	0.00	0.00	0.00	0.00	0.00
0.00	0.00	0.00	0.00	0.00	0.00	0.00	0.00	0.00
0.00	0.00	0.00	0.00	0.00	0.00	0.00	0.00	0.00
0.00	0.00	0.00	0.00	0.00	0.00	0.00	0.00	0.00
0.00	0.00	0.00	0.00	0.00	0.00	0.00	0.00	0.00
0.00	0.00	0.00	0.00	0.00	0.00	0.00	0.00	0.00
0.00	0.00	0.00	0.00	0.00	0.00	0.00	0.00	0.00
0.00	0.00	0.00	0.00	0.00	0.00	0.00	0.00	0.00
0.00	0.00	0.00	0.00	0.00	0.00	0.00	0.00	0.00
0.00	0.00	0.00	0.00	0.00	0.00	0.00	0.00	0.00
0.00	0.00	0.00	0.00	0.00	0.00	0.00	0.00	0.00
0.00	0.00	0.00	0.00	0.00	0.00	0.00	0.00	0.00
0.00	0.00	0.00	0.00	0.00	0.00	0.00	0.00	0.00
0.00	0.00	0.00	0.00	0.00	0.00	0.00	0.00	0.00
0.00	0.00	0.00	0.00	0.00	0.00	0.00	0.00	0.00
0.00	0.00	0.00	0.00	0.00	0.00	0.00	0.00	0.00
0.00	4.00	0.00	0.00	0.00	0.00	0.00	0.00	0.00
0.00	4.00	0.00	0.00	0.00	0.00	0.00	0.00	0.00
0.00	0.00	0.00	0.00	0.00	0.00	0.00	0.00	0.00
0.00	0.00	0.00	0.00	0.00	0.00	0.00	0.00	0.00
0.00	0.00	0.00	0.00	0.00	0.00	0.00	0.00	0.00
0.00	0.00	0.00	0.00	0.00	0.00	0.00	0.00	0.00
0.00	0.00	0.00	0.00	0.00	0.00	0.00	0.00	0.00
0.00	0.00	0.00	0.00	0.00	0.00	0.00	0.00	0.00
0.00	0.00	0.00	0.00	0.00	0.00	0.00	0.00	0.00
0.00	0.00	0.00	0.00	0.00	0.00	0.00	0.00	0.00

0.00	0.00	0.00	0.00	0.00	2.00	0.00	0.00	0.00
0.00	0.00	0.00	0.00	0.00	0.00	0.00	0.00	0.00
0.00	0.00	0.00	0.00	0.00	0.00	0.00	0.00	0.00
0.00	0.00	0.00	0.00	0.00	0.00	0.00	0.00	0.00
0.00	0.00	0.00	0.00	0.00	0.00	0.00	0.00	0.00
0.00	0.00	0.00	0.00	0.00	0.00	0.00	0.00	0.00
0.00	0.00	0.00	0.00	0.00	0.00	0.00	0.00	0.00
0.00	0.00	0.00	0.00	0.00	0.00	0.00	0.00	0.00
0.00	0.00	0.00	0.00	0.00	0.00	0.00	0.00	0.00
0.00	0.00	0.00	0.00	0.00	0.00	0.00	0.00	0.00
0.00	0.00	0.00	0.00	0.00	0.00	0.00	0.00	0.00
0.00	0.00	0.00	0.00	0.00	0.00	0.00	0.00	0.00
0.00	0.00	0.00	0.00	0.00	0.00	0.00	0.00	0.00
0.00	0.00	0.00	0.00	0.00	0.00	0.00	0.00	0.00
0.00	0.00	4.00	0.00	0.00	0.00	0.00	0.00	0.00
0.00	0.00	0.00	0.00	0.00	4.00	0.00	0.00	0.00
0.00	0.00	0.00	0.00	0.00	4.00	0.00	0.00	0.00
0.00	0.00	0.00	0.00	0.00	0.00	4.00	0.00	0.00
0.00	0.00	0.00	0.00	0.00	0.00	2.00	2.00	0.00
0.00	0.00	0.00	0.00	0.00	0.00	0.00	0.00	0.00
0.00	0.00	0.00	0.00	0.00	0.00	0.00	0.00	0.00
0.00	0.00	0.00	0.00	0.00	0.00	0.00	0.00	0.00
0.00	0.00	0.00	0.00	0.00	0.00	0.00	0.00	0.00
0.00	0.00	0.00	0.00	0.00	0.00	0.00	0.00	0.00
0.00	0.00	0.00	0.00	0.00	0.00	0.00	0.00	0.00
0.00	0.00	0.00	0.00	0.00	0.00	0.00	0.00	0.00
0.00	0.00	0.00	0.00	0.00	0.00	0.00	0.00	0.00
0.00	0.00	0.00	0.00	0.00	0.00	0.00	0.00	0.00
0.00	0.00	0.00	0.00	0.00	0.00	0.00	0.00	0.00
0.00	0.00	0.00	0.00	0.00	0.00	0.00	0.00	0.00
0.00	0.00	0.00	0.00	0.00	0.00	0.00	0.00	0.00
0.00	0.00	0.00	0.00	0.00	0.00	0.00	0.00	0.00
0.00	0.00	0.00	0.00	0.00	0.00	0.00	0.00	0.00
0.00	0.00	0.00	0.00	0.00	0.00	0.00	0.00	0.00
0.00	0.00	0.00	0.00	0.00	0.00	0.00	0.00	0.00
0.00	0.00	0.00	0.00	0.00	0.00	0.00	0.00	0.00
0.00	0.00	0.00	0.00	0.00	0.00	0.00	0.00	0.00

0.00	0.00	0.00	0.00	0.00	0.00	0.00	0.00	0.00
0.00	0.00	0.00	0.00	0.00	0.00	0.00	0.00	0.00
0.00	0.00	0.00	0.00	0.00	0.00	0.00	0.00	0.00
0.00	0.00	0.00	0.00	0.00	0.00	0.00	0.00	0.00
0.00	0.00	0.00	0.00	0.00	0.00	0.00	0.00	0.00
0.00	0.00	0.00	0.00	0.00	0.00	0.00	0.00	0.00
0.00	0.00	0.00	0.00	0.00	0.00	0.00	0.00	0.00
0.00	0.00	0.00	0.00	0.00	0.00	0.00	0.00	0.00
0.00	0.00	0.00	0.00	0.00	0.00	0.00	0.00	0.00
0.00	0.00	0.00	0.00	0.00	0.00	0.00	0.00	0.00
0.00	0.00	0.00	0.00	0.00	0.00	0.00	0.00	0.00
0.00	0.00	0.00	0.00	0.00	0.00	0.00	0.00	0.00
0.00	0.00	0.00	0.00	0.00	0.00	0.00	0.00	0.00
0.00	0.00	0.00	0.00	0.00	0.00	0.00	0.00	0.00
0.00	0.00	0.00	0.00	0.00	0.00	0.00	0.00	0.00
0.00	0.00	0.00	0.00	0.00	0.00	0.00	0.00	0.00
0.00	0.00	0.00	0.00	0.00	0.00	0.00	0.00	0.00
0.00	0.00	0.00	0.00	0.00	0.00	0.00	0.00	0.00
0.00	0.00	0.00	0.00	0.00	0.00	0.00	0.00	0.00
0.00	0.00	0.00	0.00	0.00	0.00	0.00	0.00	0.00
0.00	0.00	0.00	0.00	0.00	0.00	0.00	0.00	0.00
0.00	0.00	0.00	0.00	0.00	0.00	0.00	0.00	0.00
0.00	0.00	0.00	0.00	0.00	0.00	0.00	0.00	0.00
0.00	0.00	0.00	0.00	0.00	0.00	0.00	0.00	0.00
0.00	0.00	0.00	0.00	0.00	0.00	0.00	0.00	0.00
0.00	0.00	0.00	0.00	0.00	0.00	0.00	0.00	0.00
0.00	0.00	0.00	0.00	0.00	0.00	0.00	0.00	0.00
0.00	0.00	0.00	0.00	0.00	0.00	0.00	0.00	0.00
0.00	0.00	0.00	0.00	0.00	0.00	0.00	0.00	0.00
0.00	0.00	0.00	0.00	0.00	0.00	0.00	0.00	0.00
0.00	0.00	0.00	0.00	0.00	0.00	0.00	0.00	0.00
0.00	0.00	0.00	0.00	0.00	0.00	0.00	0.00	0.00
0.00	0.00	0.00	0.00	0.00	0.00	0.00	0.00	0.00
0.00	0.00	0.00	0.00	0.00	0.00	0.00	0.00	0.00
0.00	0.00	0.00	0.00	0.00	0.00	0.00	0.00	0.00
0.00	0.00	0.00	0.00	0.00	0.00	0.00	0.00	0.00
0.00	0.00	0.00	0.00	0.00	0.00	0.00	0.00	0.00

0.00	0.00	0.00	0.00	0.00	0.00	0.00	0.00	0.00
0.00	0.00	0.00	0.00	0.00	0.00	0.00	0.00	0.00
0.00	0.00	0.00	0.00	0.00	0.00	0.00	0.00	0.00
0.00	0.00	0.00	0.00	0.00	0.00	0.00	0.00	0.00
0.00	0.00	0.00	0.00	0.00	0.00	0.00	0.00	0.00
0.00	0.00	0.00	0.00	0.00	0.00	0.00	0.00	0.00
0.00	0.00	0.00	0.00	0.00	0.00	0.00	0.00	0.00
0.00	0.00	0.00	0.00	0.00	0.00	0.00	0.00	0.00
0.00	0.00	0.00	0.00	0.00	0.00	0.00	0.00	0.00
0.00	0.00	0.00	0.00	0.00	0.00	0.00	0.00	0.00
0.00	0.00	0.00	0.00	0.00	0.00	0.00	0.00	0.00
0.00	0.00	0.00	0.00	0.00	0.00	0.00	0.00	0.00
0.00	0.00	0.00	0.00	0.00	0.00	0.00	0.00	0.00
0.00	0.00	0.00	0.00	0.00	0.00	0.00	0.00	0.00
0.00	0.00	0.00	0.00	0.00	0.00	0.00	0.00	0.00
0.00	0.00	0.00	0.00	0.00	0.00	0.00	0.00	0.00
0.00	0.00	0.00	0.00	0.00	0.00	0.00	0.00	0.00
0.00	0.00	0.00	0.00	0.00	0.00	0.00	0.00	0.00
0.00	0.00	0.00	0.00	0.00	0.00	0.00	0.00	0.00
0.00	0.00	0.00	0.00	0.00	0.00	0.00	0.00	0.00
0.00	0.00	0.00	0.00	0.00	0.00	0.00	0.00	0.00
0.00	0.00	0.00	0.00	0.00	0.00	0.00	0.00	0.00
0.00	0.00	0.00	0.00	0.00	0.00	0.00	0.00	0.00
0.00	0.00	0.00	0.00	0.00	0.00	0.00	0.00	0.00
0.00	0.00	0.00	0.00	0.00	0.00	0.00	0.00	0.00
0.00	0.00	0.00	0.00	0.00	0.00	0.00	0.00	0.00
0.00	0.00	0.00	0.00	0.00	0.00	0.00	0.00	0.00
0.00	0.00	0.00	0.00	0.00	0.00	0.00	0.00	0.00
0.00	0.00	0.00	0.00	0.00	0.00	0.00	0.00	0.00
0.00	0.00	0.00	0.00	0.00	0.00	0.00	0.00	0.00
0.00	0.00	0.00	0.00	0.00	0.00	0.00	0.00	0.00
0.00	0.00	0.00	0.00	0.00	0.00	0.00	0.00	0.00
0.00	0.00	0.00	0.00	0.00	0.00	0.00	0.00	0.00
0.00	0.00	0.00	0.00	0.00	0.00	0.00	0.00	0.00
0.00	0.00	0.00	0.00	0.00	0.00	0.00	0.00	0.00
0.00	0.00	0.00	0.00	0.00	0.00	0.00	0.00	0.00
0.00	0.00	0.00	0.00	0.00	0.00	0.00	0.00	0.00

0.00	0.00	0.00	0.00	0.00	0.00	0.00	0.00	0.00
0.00	0.00	0.00	0.00	0.00	0.00	0.00	0.00	0.00
0.00	0.00	0.00	0.00	0.00	0.00	0.00	0.00	0.00
0.00	0.00	0.00	0.00	0.00	0.00	0.00	0.00	0.00
3.00	0.00	0.00	0.00	0.00	0.00	0.00	0.00	0.00
3.00	0.00	0.00	0.00	0.00	0.00	0.00	0.00	0.00
3.00	0.00	0.00	0.00	0.00	0.00	0.00	0.00	0.00
3.00	0.00	0.00	0.00	0.00	0.00	0.00	0.00	0.00
3.00	0.00	0.00	0.00	0.00	0.00	0.00	0.00	0.00
0.00	3.00	0.00	0.00	0.00	0.00	0.00	0.00	0.00
0.00	3.00	0.00	0.00	0.00	0.00	0.00	0.00	0.00
0.00	0.00	0.00	0.00	0.00	0.00	0.00	0.00	3.00
0.00	0.00	0.00	0.00	0.00	0.00	0.00	0.00	0.00
0.00	0.00	0.00	0.00	0.00	0.00	0.00	0.00	0.00
0.00	0.00	0.00	0.00	0.00	0.00	0.00	0.00	0.00
0.00	0.00	0.00	0.00	0.00	0.00	0.00	0.00	0.00
0.00	0.00	0.00	0.00	0.00	0.00	0.00	0.00	0.00
0.00	0.00	0.00	0.00	0.00	0.00	0.00	0.00	0.00
0.00	0.00	0.00	0.00	0.00	0.00	0.00	0.00	0.00
0.00	0.00	0.00	0.00	0.00	0.00	0.00	0.00	0.00
0.00	0.00	0.00	0.00	0.00	0.00	0.00	0.00	0.00
0.00	0.00	0.00	0.00	0.00	0.00	0.00	0.00	0.00
0.00	0.00	0.00	0.00	0.00	0.00	0.00	0.00	0.00
0.00	0.00	0.00	0.00	0.00	0.00	0.00	0.00	0.00
0.00	0.00	0.00	0.00	0.00	0.00	0.00	0.00	0.00
0.00	0.00	0.00	0.00	0.00	0.00	0.00	0.00	0.00
0.00	0.00	0.00	0.00	0.00	0.00	0.00	0.00	0.00
0.00	0.00	0.00	0.00	0.00	0.00	0.00	0.00	0.00
0.00	0.00	0.00	0.00	0.00	0.00	0.00	0.00	0.00
0.00	0.00	0.00	0.00	0.00	0.00	0.00	0.00	0.00
0.00	0.00	0.00	0.00	0.00	0.00	0.00	0.00	0.00
0.00	0.00	0.00	0.00	0.00	0.00	0.00	0.00	0.00
0.00	0.00	0.00	0.00	0.00	0.00	0.00	0.00	0.00
0.00	0.00	0.00	0.00	0.00	0.00	0.00	0.00	0.00
0.00	0.00	0.00	0.00	0.00	0.00	0.00	0.00	0.00
0.00	0.00	0.00	0.00	0.00	0.00	0.00	0.00	0.00
0.00	0.00	0.00	0.00	0.00	0.00	0.00	0.00	0.00
0.00	0.00	0.00	0.00	0.00	0.00	0.00	0.00	0.00

0.00	0.00	3.00	0.00	0.00	0.00	0.00	0.00	0.00
0.00	0.00	0.00	3.00	0.00	0.00	0.00	0.00	0.00
0.00	0.00	0.00	3.00	0.00	0.00	0.00	0.00	0.00
0.00	0.00	0.00	0.00	0.00	0.00	3.00	0.00	0.00
0.00	0.00	0.00	0.00	0.00	0.00	0.00	3.00	0.00
0.00	0.00	0.00	0.00	0.00	0.00	0.00	3.00	0.00
0.00	0.00	0.00	0.00	0.00	0.00	0.00	3.00	0.00
0.00	0.00	0.00	0.00	0.00	0.00	0.00	0.00	0.00
0.00	0.00	0.00	0.00	0.00	0.00	0.00	0.00	0.00
0.00	0.00	0.00	0.00	0.00	0.00	0.00	0.00	0.00
0.00	0.00	0.00	0.00	0.00	0.00	0.00	0.00	0.00
0.00	0.00	0.00	0.00	0.00	0.00	0.00	0.00	0.00
0.00	0.00	0.00	0.00	0.00	0.00	0.00	0.00	0.00
0.00	0.00	0.00	0.00	0.00	0.00	0.00	0.00	0.00
0.00	0.00	0.00	0.00	0.00	0.00	0.00	0.00	0.00
0.00	0.00	0.00	0.00	0.00	0.00	0.00	0.00	0.00
0.00	0.00	0.00	0.00	0.00	0.00	0.00	0.00	0.00
0.00	0.00	0.00	0.00	0.00	0.00	0.00	0.00	0.00
0.00	0.00	0.00	0.00	0.00	0.00	0.00	0.00	0.00
0.00	0.00	0.00	0.00	0.00	0.00	0.00	0.00	0.00
0.00	0.00	0.00	0.00	0.00	0.00	0.00	0.00	0.00
0.00	0.00	0.00	0.00	0.00	0.00	0.00	0.00	0.00
0.00	0.00	0.00	0.00	0.00	0.00	0.00	0.00	0.00
0.00	0.00	0.00	0.00	0.00	0.00	0.00	0.00	0.00
0.00	0.00	0.00	0.00	0.00	0.00	0.00	0.00	0.00
0.00	0.00	0.00	0.00	0.00	0.00	0.00	0.00	0.00
0.00	0.00	0.00	0.00	0.00	0.00	0.00	0.00	0.00
0.00	0.00	0.00	0.00	0.00	0.00	0.00	0.00	0.00
0.00	0.00	0.00	0.00	0.00	0.00	0.00	0.00	0.00
0.00	0.00	0.00	0.00	0.00	0.00	0.00	0.00	0.00
0.00	0.00	0.00	0.00	0.00	0.00	0.00	0.00	0.00
0.00	0.00	0.00	0.00	0.00	0.00	0.00	0.00	0.00
0.00	0.00	0.00	0.00	0.00	0.00	0.00	0.00	0.00
0.00	0.00	0.00	0.00	0.00	0.00	0.00	0.00	0.00
0.00	0.00	0.00	0.00	0.00	0.00	0.00	0.00	0.00
0.00	0.00	0.00	0.00	0.00	0.00	0.00	0.00	0.00
0.00	0.00	0.00	0.00	0.00	0.00	0.00	0.00	0.00

0.00	0.00	0.00	0.00	0.00	0.00	0.00	0.00	0.00
0.00	0.00	0.00	0.00	0.00	0.00	0.00	0.00	0.00
0.00	0.00	0.00	0.00	0.00	0.00	0.00	0.00	0.00
0.00	0.00	0.00	0.00	0.00	0.00	0.00	0.00	0.00
0.00	0.00	0.00	0.00	0.00	0.00	0.00	0.00	0.00
0.00	0.00	0.00	0.00	0.00	0.00	0.00	0.00	0.00
0.00	0.00	0.00	0.00	0.00	0.00	0.00	0.00	0.00
0.00	0.00	0.00	0.00	0.00	0.00	0.00	0.00	0.00
0.00	0.00	0.00	0.00	0.00	0.00	0.00	0.00	0.00
0.00	0.00	0.00	0.00	0.00	0.00	0.00	0.00	0.00
0.00	0.00	0.00	0.00	0.00	0.00	0.00	0.00	0.00
0.00	0.00	0.00	0.00	0.00	0.00	0.00	0.00	0.00
0.00	0.00	0.00	0.00	0.00	0.00	0.00	0.00	0.00
0.00	0.00	0.00	0.00	0.00	0.00	0.00	0.00	0.00
0.00	0.00	0.00	0.00	0.00	0.00	0.00	0.00	0.00
0.00	0.00	0.00	0.00	0.00	0.00	0.00	0.00	0.00
0.00	0.00	0.00	0.00	0.00	0.00	0.00	0.00	0.00
0.00	0.00	0.00	0.00	0.00	0.00	0.00	0.00	0.00
0.00	0.00	0.00	0.00	0.00	0.00	0.00	0.00	0.00
0.00	0.00	0.00	0.00	0.00	0.00	0.00	0.00	0.00
0.00	0.00	0.00	0.00	0.00	0.00	0.00	0.00	0.00
0.00	0.00	0.00	0.00	0.00	0.00	0.00	0.00	0.00
0.00	0.00	0.00	0.00	0.00	0.00	0.00	0.00	0.00
0.00	0.00	0.00	0.00	0.00	0.00	0.00	0.00	0.00
0.00	0.00	0.00	0.00	0.00	0.00	0.00	0.00	0.00
0.00	0.00	0.00	0.00	0.00	0.00	0.00	0.00	0.00
0.00	0.00	0.00	0.00	0.00	0.00	0.00	0.00	0.00
0.00	0.00	0.00	0.00	0.00	0.00	0.00	0.00	0.00
0.00	0.00	0.00	0.00	0.00	0.00	0.00	0.00	0.00
0.00	0.00	0.00	0.00	0.00	0.00	0.00	0.00	0.00
0.00	0.00	0.00	0.00	0.00	0.00	0.00	0.00	0.00
0.00	0.00	0.00	0.00	0.00	0.00	0.00	0.00	0.00
0.00	0.00	0.00	0.00	0.00	0.00	0.00	0.00	0.00
0.00	0.00	0.00	0.00	0.00	0.00	0.00	0.00	0.00
0.00	0.00	0.00	0.00	0.00	0.00	0.00	0.00	0.00
0.00	0.00	0.00	0.00	0.00	0.00	0.00	0.00	0.00
0.00	0.00	0.00	0.00	0.00	0.00	0.00	0.00	0.00

0.00	0.00	0.00	0.00	0.00	0.00	0.00	0.00	0.00
0.00	0.00	0.00	0.00	0.00	0.00	0.00	0.00	0.00
0.00	0.00	0.00	0.00	0.00	0.00	0.00	0.00	0.00
0.00	0.00	0.00	0.00	0.00	0.00	0.00	0.00	0.00
0.00	0.00	0.00	0.00	0.00	0.00	0.00	0.00	0.00
0.00	0.00	0.00	0.00	0.00	0.00	0.00	0.00	0.00
0.00	0.00	0.00	0.00	0.00	0.00	0.00	0.00	0.00
0.00	0.00	0.00	0.00	0.00	0.00	0.00	0.00	0.00
0.00	0.00	0.00	0.00	0.00	0.00	0.00	0.00	0.00
0.00	0.00	0.00	0.00	0.00	0.00	0.00	0.00	0.00
0.00	0.00	0.00	0.00	0.00	0.00	0.00	0.00	0.00
0.00	0.00	0.00	0.00	0.00	0.00	0.00	0.00	0.00
0.00	0.00	0.00	0.00	0.00	0.00	0.00	0.00	0.00
0.00	0.00	0.00	0.00	0.00	0.00	0.00	0.00	0.00
0.00	0.00	0.00	0.00	0.00	0.00	0.00	0.00	0.00
0.00	0.00	0.00	0.00	0.00	0.00	0.00	0.00	0.00
0.00	0.00	0.00	0.00	0.00	0.00	0.00	0.00	0.00
0.00	0.00	0.00	0.00	0.00	0.00	0.00	0.00	0.00
0.00	0.00	0.00	0.00	0.00	0.00	0.00	0.00	0.00
0.00	0.00	0.00	0.00	0.00	0.00	0.00	0.00	0.00
0.00	0.00	0.00	0.00	0.00	0.00	0.00	0.00	0.00
0.00	0.00	0.00	0.00	0.00	0.00	0.00	0.00	0.00
0.00	0.00	0.00	0.00	0.00	0.00	0.00	0.00	0.00
0.00	0.00	0.00	0.00	0.00	0.00	0.00	0.00	0.00
0.00	0.00	0.00	0.00	0.00	0.00	0.00	0.00	0.00
0.00	0.00	0.00	0.00	0.00	0.00	0.00	0.00	0.00
0.00	0.00	0.00	0.00	0.00	0.00	0.00	0.00	0.00
0.00	0.00	0.00	0.00	0.00	0.00	0.00	0.00	0.00
2.00	0.00	0.00	0.00	0.00	0.00	0.00	0.00	0.00
2.00	0.00	0.00	0.00	0.00	0.00	0.00	0.00	0.00
2.00	0.00	0.00	0.00	0.00	0.00	0.00	0.00	0.00
2.00	0.00	0.00	0.00	0.00	0.00	0.00	0.00	0.00
2.00	0.00	0.00	0.00	0.00	0.00	0.00	0.00	0.00
2.00	0.00	0.00	0.00	0.00	0.00	0.00	0.00	0.00
2.00	0.00	0.00	0.00	0.00	0.00	0.00	0.00	0.00
2.00	0.00	0.00	0.00	0.00	0.00	0.00	0.00	0.00
2.00	0.00	0.00	0.00	0.00	0.00	0.00	0.00	0.00

0.00	2.00	0.00	0.00	0.00	0.00	0.00	0.00	0.00
0.00	2.00	0.00	0.00	0.00	0.00	0.00	0.00	0.00
0.00	2.00	0.00	0.00	0.00	0.00	0.00	0.00	0.00
0.00	0.00	0.00	0.00	0.00	0.00	0.00	0.00	2.00
0.00	0.00	0.00	0.00	0.00	0.00	0.00	0.00	0.00
0.00	0.00	0.00	0.00	0.00	0.00	0.00	0.00	0.00
0.00	0.00	0.00	0.00	0.00	0.00	0.00	0.00	0.00
0.00	0.00	0.00	0.00	0.00	0.00	0.00	0.00	0.00
0.00	0.00	0.00	0.00	0.00	0.00	0.00	0.00	0.00
0.00	0.00	0.00	0.00	0.00	0.00	0.00	0.00	0.00
0.00	0.00	0.00	0.00	0.00	0.00	0.00	0.00	0.00
0.00	0.00	0.00	0.00	0.00	0.00	0.00	0.00	0.00
0.00	0.00	0.00	0.00	0.00	0.00	0.00	0.00	0.00
0.00	0.00	0.00	0.00	0.00	0.00	0.00	0.00	0.00
0.00	0.00	0.00	0.00	0.00	0.00	0.00	0.00	0.00
0.00	0.00	0.00	0.00	0.00	0.00	0.00	0.00	0.00
0.00	0.00	0.00	0.00	0.00	0.00	0.00	0.00	0.00
0.00	0.00	0.00	0.00	0.00	0.00	0.00	0.00	0.00
0.00	0.00	0.00	0.00	0.00	0.00	0.00	0.00	0.00
0.00	0.00	0.00	0.00	0.00	0.00	0.00	0.00	0.00
0.00	0.00	0.00	0.00	0.00	0.00	0.00	0.00	0.00
0.00	0.00	0.00	0.00	0.00	0.00	0.00	0.00	0.00
0.00	0.00	0.00	0.00	0.00	0.00	0.00	0.00	0.00
0.00	0.00	0.00	0.00	0.00	0.00	0.00	0.00	0.00
0.00	0.00	0.00	0.00	0.00	0.00	0.00	0.00	0.00
0.00	0.00	0.00	0.00	0.00	0.00	0.00	0.00	0.00
0.00	0.00	0.00	0.00	0.00	0.00	0.00	0.00	0.00
0.00	0.00	0.00	0.00	0.00	0.00	0.00	0.00	0.00
0.00	0.00	0.00	0.00	0.00	0.00	0.00	0.00	0.00
0.00	0.00	0.00	0.00	0.00	0.00	0.00	0.00	0.00
0.00	0.00	0.00	0.00	0.00	0.00	0.00	0.00	0.00
0.00	0.00	0.00	0.00	0.00	0.00	0.00	0.00	0.00
0.00	0.00	0.00	0.00	0.00	0.00	0.00	0.00	0.00
0.00	0.00	0.00	0.00	0.00	0.00	0.00	0.00	0.00
0.00	0.00	0.00	0.00	0.00	0.00	0.00	0.00	0.00
0.00	0.00	2.00	0.00	0.00	0.00	0.00	0.00	0.00
0.00	0.00	2.00	0.00	0.00	0.00	0.00	0.00	0.00

0.00	0.00	2.00	0.00	0.00	0.00	0.00	0.00	0.00
0.00	0.00	0.00	2.00	0.00	0.00	0.00	0.00	0.00
0.00	0.00	0.00	0.00	0.00	0.00	2.00	0.00	0.00
0.00	0.00	0.00	0.00	0.00	0.00	2.00	0.00	0.00
0.00	0.00	0.00	0.00	0.00	0.00	0.00	2.00	0.00
0.00	0.00	0.00	0.00	0.00	0.00	0.00	2.00	0.00
0.00	0.00	0.00	0.00	0.00	0.00	0.00	0.00	0.00
0.00	0.00	0.00	0.00	0.00	0.00	0.00	0.00	0.00
0.00	0.00	0.00	0.00	0.00	0.00	0.00	0.00	0.00
0.00	0.00	0.00	0.00	0.00	0.00	0.00	0.00	0.00
0.00	0.00	0.00	0.00	0.00	0.00	0.00	0.00	0.00
0.00	0.00	0.00	0.00	0.00	0.00	0.00	0.00	0.00
0.00	0.00	0.00	0.00	0.00	0.00	0.00	0.00	0.00
0.00	0.00	0.00	0.00	0.00	0.00	0.00	0.00	0.00
0.00	0.00	0.00	0.00	0.00	0.00	0.00	0.00	0.00
0.00	0.00	0.00	0.00	0.00	0.00	0.00	0.00	0.00
0.00	0.00	0.00	0.00	0.00	0.00	0.00	0.00	0.00
0.00	0.00	0.00	0.00	0.00	0.00	0.00	0.00	0.00
0.00	0.00	0.00	0.00	0.00	0.00	0.00	0.00	0.00
0.00	0.00	0.00	0.00	0.00	0.00	0.00	0.00	0.00
0.00	0.00	0.00	0.00	0.00	0.00	0.00	0.00	0.00
0.00	0.00	0.00	0.00	0.00	0.00	0.00	0.00	0.00
0.00	0.00	0.00	0.00	0.00	0.00	0.00	0.00	0.00
0.00	0.00	0.00	0.00	0.00	0.00	0.00	0.00	0.00
0.00	0.00	0.00	0.00	0.00	0.00	0.00	0.00	0.00
0.00	0.00	0.00	0.00	0.00	0.00	0.00	0.00	0.00
0.00	0.00	0.00	0.00	0.00	0.00	0.00	0.00	0.00
0.00	0.00	0.00	0.00	0.00	0.00	0.00	0.00	0.00
0.00	0.00	0.00	0.00	0.00	0.00	0.00	0.00	0.00
0.00	0.00	0.00	0.00	0.00	0.00	0.00	0.00	0.00
0.00	0.00	0.00	0.00	0.00	0.00	0.00	0.00	0.00
0.00	0.00	0.00	0.00	0.00	0.00	0.00	0.00	0.00
0.00	0.00	0.00	0.00	0.00	0.00	0.00	0.00	0.00
0.00	0.00	0.00	0.00	0.00	0.00	0.00	0.00	0.00
0.00	0.00	0.00	0.00	0.00	0.00	0.00	0.00	0.00
0.00	0.00	0.00	0.00	0.00	0.00	0.00	0.00	0.00
0.00	0.00	0.00	0.00	0.00	0.00	0.00	0.00	0.00

0.00	0.00	0.00	0.00	0.00	0.00	0.00	0.00	0.00
0.00	0.00	0.00	0.00	0.00	0.00	0.00	0.00	0.00
0.00	0.00	0.00	0.00	0.00	0.00	0.00	0.00	0.00
0.00	0.00	0.00	0.00	0.00	0.00	0.00	0.00	0.00
0.00	0.00	0.00	0.00	0.00	0.00	0.00	0.00	0.00
0.00	0.00	0.00	0.00	0.00	0.00	0.00	0.00	0.00
0.00	0.00	0.00	0.00	0.00	0.00	0.00	0.00	0.00
0.00	0.00	0.00	0.00	0.00	0.00	0.00	0.00	0.00
0.00	0.00	0.00	0.00	0.00	0.00	0.00	0.00	0.00
0.00	0.00	0.00	0.00	0.00	0.00	0.00	0.00	0.00
0.00	0.00	0.00	0.00	0.00	0.00	0.00	0.00	0.00
0.00	0.00	0.00	0.00	0.00	0.00	0.00	0.00	0.00
0.00	0.00	0.00	0.00	0.00	0.00	0.00	0.00	0.00
0.00	0.00	0.00	0.00	0.00	0.00	0.00	0.00	0.00
0.00	0.00	0.00	0.00	0.00	0.00	0.00	0.00	0.00
0.00	0.00	0.00	0.00	0.00	0.00	0.00	0.00	0.00
0.00	0.00	0.00	0.00	0.00	0.00	0.00	0.00	0.00
0.00	0.00	0.00	0.00	0.00	0.00	0.00	0.00	0.00
0.00	0.00	0.00	0.00	0.00	0.00	0.00	0.00	0.00
0.00	0.00	0.00	0.00	0.00	0.00	0.00	0.00	0.00
0.00	0.00	0.00	0.00	0.00	0.00	0.00	0.00	0.00
0.00	0.00	0.00	0.00	0.00	0.00	0.00	0.00	0.00
0.00	0.00	0.00	0.00	0.00	0.00	0.00	0.00	0.00
0.00	0.00	0.00	0.00	0.00	0.00	0.00	0.00	0.00
0.00	0.00	0.00	0.00	0.00	0.00	0.00	0.00	0.00
0.00	0.00	0.00	0.00	0.00	0.00	0.00	0.00	0.00
0.00	0.00	0.00	0.00	0.00	0.00	0.00	0.00	0.00
0.00	0.00	0.00	0.00	0.00	0.00	0.00	0.00	0.00
0.00	0.00	0.00	0.00	0.00	0.00	0.00	0.00	0.00
0.00	0.00	0.00	0.00	0.00	0.00	0.00	0.00	0.00
0.00	0.00	0.00	0.00	0.00	0.00	0.00	0.00	0.00
0.00	0.00	0.00	0.00	0.00	0.00	0.00	0.00	0.00
0.00	0.00	0.00	0.00	0.00	0.00	0.00	0.00	0.00
0.00	0.00	0.00	0.00	0.00	0.00	0.00	0.00	0.00
0.00	0.00	0.00	0.00	0.00	0.00	0.00	0.00	0.00
0.00	0.00	0.00	0.00	0.00	0.00	0.00	0.00	0.00
0.00	0.00	0.00	0.00	0.00	0.00	0.00	0.00	0.00

0.00	0.00	0.00	0.00	0.00	0.00	0.00	0.00	0.00
0.00	0.00	0.00	0.00	0.00	0.00	0.00	0.00	0.00
0.00	0.00	0.00	0.00	0.00	0.00	0.00	0.00	0.00
0.00	0.00	0.00	0.00	0.00	0.00	0.00	0.00	0.00
0.00	0.00	0.00	0.00	0.00	0.00	0.00	0.00	0.00
0.00	0.00	0.00	0.00	0.00	0.00	0.00	0.00	0.00
0.00	0.00	0.00	0.00	0.00	0.00	0.00	0.00	0.00
0.00	0.00	0.00	0.00	0.00	0.00	0.00	0.00	0.00
0.00	0.00	0.00	0.00	0.00	0.00	0.00	0.00	0.00
0.00	0.00	0.00	0.00	0.00	0.00	0.00	0.00	0.00
0.00	0.00	0.00	0.00	0.00	0.00	0.00	0.00	0.00
0.00	0.00	0.00	0.00	0.00	0.00	0.00	0.00	0.00
0.00	0.00	0.00	0.00	0.00	0.00	0.00	0.00	0.00
0.00	0.00	0.00	0.00	0.00	0.00	0.00	0.00	0.00
0.00	0.00	0.00	0.00	0.00	0.00	0.00	0.00	0.00
0.00	0.00	0.00	0.00	0.00	0.00	0.00	0.00	0.00
0.00	0.00	0.00	0.00	0.00	0.00	0.00	0.00	0.00
0.00	0.00	0.00	0.00	0.00	0.00	0.00	0.00	0.00
0.00	0.00	0.00	0.00	0.00	0.00	0.00	0.00	0.00
0.00	0.00	0.00	0.00	0.00	0.00	0.00	0.00	0.00
0.00	0.00	0.00	0.00	0.00	0.00	0.00	0.00	0.00
0.00	0.00	0.00	0.00	0.00	0.00	0.00	0.00	0.00
0.00	0.00	0.00	0.00	0.00	0.00	0.00	0.00	0.00
0.00	0.00	0.00	0.00	0.00	0.00	0.00	0.00	0.00
0.00	0.00	0.00	0.00	0.00	0.00	0.00	0.00	0.00
0.00	0.00	0.00	0.00	0.00	0.00	0.00	0.00	0.00
0.00	0.00	0.00	0.00	0.00	0.00	0.00	0.00	0.00
0.00	0.00	0.00	0.00	0.00	0.00	0.00	0.00	0.00
0.00	0.00	0.00	0.00	0.00	0.00	0.00	0.00	0.00
0.00	0.00	0.00	0.00	0.00	0.00	0.00	0.00	0.00
0.00	0.00	0.00	0.00	0.00	0.00	0.00	0.00	0.00
0.00	0.00	0.00	0.00	0.00	0.00	0.00	0.00	0.00
0.00	0.00	0.00	0.00	0.00	0.00	0.00	0.00	0.00

A8	A9	A10	B9	A11	A12	B10	B11	B12	
0.00	16.00	8.00	0.00	0.00	0.00	0.00	0.00	0.00	
32231.00	20575.00	19030.00	6849.00	2564.00	15945.00	17588.00	23906.00	26944.00	
14.00	79.00	3.00	7.00	0.00	18.00	26.00	0.00	29.00	
12013.00	10075.00	8154.00	2395.00	770.00	9990.00	5766.00	6133.00	6813.00	
8092.00	4955.00	5126.00	1565.00	596.00	4119.00	4772.00	5684.00	5727.00	
0.00	0.00	26.00	4747.00	39784.00	0.00	0.00	0.00	0.00	
0.00	0.00	19.00	0.00	0.00	0.00	0.00	0.00	0.00	
4913.00	5132.00	3585.00	1244.00	562.00	3392.00	4565.00	3243.00	4232.00	
18.00	0.00	0.00	0.00	0.00	0.00	0.00	0.00	0.00	
5773.00	4361.00	3214.00	822.00	313.00	4001.00	2645.00	2677.00	3069.00	
0.00	0.00	0.00	0.00	0.00	0.00	0.00	0.00	0.00	
0.00	0.00	0.00	13.00	0.00	0.00	0.00	0.00	0.00	
0.00	0.00	0.00	0.00	0.00	0.00	0.00	0.00	0.00	
3044.00	2249.00	1805.00	294.00	122.00	1514.00	1357.00	1865.00	1606.00	
3070.00	2279.00	1961.00	450.00	189.00	2081.00	1503.00	1645.00	1615.00	
2086.00	1471.00	1599.00	412.00	158.00	1807.00	1272.00	1155.00	1358.00	
0.00	0.00	0.00	0.00	0.00	0.00	0.00	0.00	0.00	
2181.00	2385.00	1429.00	496.00	99.00	1075.00	967.00	920.00	2400.00	
65.00	0.00	0.00	0.00	15.00	0.00	0.00	0.00	0.00	
0.00	0.00	0.00	0.00	20.00	15.00	0.00	0.00	0.00	
0.00	0.00	0.00	0.00	0.00	0.00	0.00	0.00	0.00	
0.00	0.00	0.00	0.00	0.00	0.00	0.00	0.00	0.00	
0.00	0.00	0.00	0.00	0.00	0.00	0.00	0.00	0.00	
696.00	968.00	994.00	234.00	109.00	763.00	948.00	476.00	1343.00	
0.00	0.00	0.00	0.00	0.00	0.00	0.00	0.00	0.00	
0.00	0.00	0.00	0.00	0.00	0.00	0.00	0.00	0.00	
0.00	0.00	0.00	0.00	0.00	0.00	0.00	0.00	0.00	
269.00	222.00	190.00	279.00	86.00	412.00	948.00	51.00	1029.00	
0.00	0.00	0.00	908.00	7464.00	0.00	0.00	0.00	0.00	
0.00	0.00	0.00	0.00	0.00	0.00	0.00	11.00	0.00	
0.00	0.00	0.00	0.00	0.00	0.00	0.00	0.00	0.00	
0.00	0.00	0.00	0.00	0.00	0.00	0.00	0.00	0.00	
0.00	0.00	0.00	0.00	0.00	0.00	0.00	0.00	0.00	
0.00	0.00	0.00	0.00	0.00	0.00	0.00	0.00	0.00	
0.00	0.00	0.00	0.00	0.00	0.00	0.00	0.00	0.00	

0.00	0.00	0.00	10151.00	0.00	0.00	0.00	0.00	13.00
0.00	0.00	0.00	0.00	0.00	0.00	0.00	0.00	0.00
0.00	0.00	0.00	0.00	0.00	0.00	0.00	0.00	0.00
0.00	0.00	0.00	0.00	0.00	0.00	0.00	0.00	0.00
0.00	0.00	0.00	0.00	0.00	0.00	0.00	0.00	0.00
0.00	0.00	0.00	0.00	0.00	0.00	0.00	0.00	0.00
0.00	0.00	0.00	0.00	0.00	0.00	0.00	0.00	0.00
0.00	20.00	0.00	0.00	0.00	0.00	9.00	0.00	2.00
0.00	0.00	0.00	0.00	0.00	0.00	0.00	0.00	0.00
0.00	0.00	0.00	0.00	0.00	0.00	0.00	0.00	0.00
0.00	0.00	0.00	0.00	0.00	0.00	0.00	0.00	0.00
386.00	219.00	121.00	8.00	24.00	141.00	203.00	337.00	144.00
0.00	0.00	0.00	0.00	0.00	0.00	0.00	0.00	0.00
0.00	0.00	0.00	0.00	15.00	22.00	93.00	0.00	17.00
0.00	0.00	0.00	0.00	0.00	0.00	0.00	0.00	0.00
0.00	0.00	0.00	0.00	0.00	0.00	0.00	0.00	0.00
393.00	79.00	253.00	32.00	11.00	127.00	196.00	37.00	193.00
0.00	0.00	0.00	0.00	0.00	0.00	0.00	0.00	0.00
166.00	336.00	108.00	47.00	18.00	222.00	143.00	20.00	158.00
350.00	97.00	92.00	39.00	12.00	212.00	119.00	97.00	152.00
0.00	0.00	0.00	0.00	12.00	0.00	26.00	12.00	12.00
0.00	19.00	0.00	0.00	0.00	0.00	11.00	16.00	16.00
0.00	0.00	0.00	0.00	0.00	0.00	0.00	0.00	0.00
0.00	0.00	0.00	0.00	0.00	0.00	0.00	0.00	0.00
0.00	0.00	0.00	0.00	0.00	0.00	0.00	0.00	0.00
0.00	0.00	0.00	0.00	0.00	0.00	0.00	0.00	0.00
0.00	0.00	0.00	0.00	0.00	0.00	0.00	0.00	0.00
0.00	0.00	0.00	0.00	0.00	0.00	0.00	0.00	0.00
108.00	13.00	20.00	73.00	22.00	141.00	182.00	0.00	163.00
247.00	388.00	123.00	40.00	13.00	60.00	118.00	113.00	149.00
0.00	0.00	0.00	0.00	0.00	0.00	0.00	0.00	0.00
0.00	0.00	0.00	0.00	0.00	0.00	0.00	0.00	0.00
0.00	0.00	0.00	0.00	10.00	0.00	0.00	0.00	0.00
103.00	0.00	0.00	65.00	0.00	77.00	230.00	0.00	323.00
0.00	0.00	0.00	0.00	0.00	0.00	0.00	0.00	0.00
0.00	0.00	0.00	0.00	0.00	0.00	0.00	0.00	0.00
0.00	0.00	0.00	0.00	0.00	0.00	0.00	0.00	0.00

0.00	0.00	0.00	0.00	0.00	0.00	0.00	0.00	0.00
0.00	0.00	0.00	0.00	0.00	0.00	0.00	0.00	0.00
0.00	0.00	0.00	0.00	0.00	0.00	0.00	0.00	0.00
0.00	0.00	0.00	0.00	0.00	0.00	0.00	0.00	0.00
0.00	0.00	0.00	0.00	0.00	0.00	0.00	0.00	0.00
0.00	0.00	0.00	0.00	0.00	0.00	0.00	0.00	0.00
0.00	0.00	0.00	2868.00	0.00	0.00	0.00	0.00	0.00
0.00	0.00	0.00	0.00	0.00	0.00	0.00	0.00	0.00
0.00	0.00	0.00	0.00	0.00	0.00	0.00	0.00	0.00
0.00	0.00	0.00	0.00	0.00	0.00	0.00	0.00	0.00
0.00	0.00	0.00	0.00	0.00	0.00	0.00	0.00	0.00
134.00	89.00	0.00	26.00	15.00	101.00	80.00	37.00	92.00
0.00	162.00	85.00	0.00	0.00	0.00	0.00	33.00	0.00
0.00	5.00	0.00	0.00	2.00	0.00	0.00	4.00	0.00
0.00	0.00	0.00	0.00	0.00	0.00	0.00	0.00	0.00
0.00	0.00	0.00	0.00	0.00	0.00	0.00	0.00	0.00
160.00	143.00	79.00	9.00	12.00	63.00	23.00	0.00	168.00
96.00	227.00	85.00	17.00	24.00	17.00	153.00	41.00	76.00
0.00	0.00	0.00	0.00	0.00	0.00	0.00	0.00	0.00
0.00	0.00	0.00	0.00	0.00	0.00	0.00	0.00	0.00
10.00	0.00	0.00	0.00	0.00	0.00	0.00	0.00	0.00
0.00	0.00	0.00	0.00	0.00	0.00	0.00	0.00	0.00
0.00	0.00	0.00	0.00	0.00	0.00	0.00	0.00	0.00
0.00	0.00	0.00	0.00	0.00	0.00	0.00	0.00	0.00
0.00	0.00	0.00	0.00	0.00	0.00	0.00	0.00	0.00
0.00	0.00	0.00	0.00	0.00	0.00	0.00	0.00	0.00
0.00	0.00	0.00	0.00	0.00	0.00	0.00	0.00	0.00
15.00	0.00	0.00	4.00	0.00	18.00	0.00	0.00	0.00
5.00	0.00	0.00	0.00	0.00	0.00	0.00	0.00	0.00
0.00	0.00	0.00	0.00	0.00	0.00	0.00	0.00	0.00
51.00	63.00	52.00	17.00	0.00	23.00	87.00	0.00	62.00
0.00	0.00	3.00	0.00	0.00	0.00	0.00	0.00	0.00
0.00	0.00	0.00	0.00	0.00	16.00	18.00	0.00	16.00
0.00	0.00	0.00	0.00	0.00	0.00	0.00	0.00	0.00
105.00	39.00	79.00	0.00	0.00	0.00	51.00	224.00	27.00
0.00	0.00	0.00	0.00	0.00	0.00	0.00	0.00	0.00
0.00	0.00	0.00	0.00	0.00	0.00	0.00	0.00	0.00

0.00	0.00	0.00	0.00	0.00	0.00	0.00	0.00	0.00
0.00	0.00	0.00	0.00	0.00	0.00	0.00	0.00	0.00
0.00	0.00	0.00	0.00	0.00	0.00	0.00	0.00	0.00
0.00	0.00	0.00	0.00	0.00	0.00	0.00	0.00	0.00
0.00	0.00	0.00	0.00	0.00	5.00	37.00	0.00	0.00
0.00	0.00	0.00	0.00	0.00	0.00	0.00	0.00	0.00
0.00	0.00	0.00	0.00	0.00	0.00	0.00	0.00	0.00
0.00	0.00	0.00	0.00	0.00	0.00	0.00	0.00	14.00
0.00	0.00	0.00	0.00	0.00	0.00	0.00	0.00	0.00
0.00	0.00	0.00	0.00	0.00	0.00	0.00	0.00	0.00
0.00	0.00	0.00	0.00	0.00	0.00	0.00	0.00	0.00
0.00	0.00	0.00	0.00	0.00	0.00	0.00	0.00	0.00
0.00	0.00	0.00	0.00	0.00	0.00	0.00	0.00	0.00
0.00	0.00	0.00	0.00	0.00	0.00	0.00	0.00	0.00
29.00	0.00	11.00	13.00	0.00	49.00	4.00	0.00	104.00
0.00	0.00	0.00	0.00	0.00	0.00	0.00	0.00	0.00
0.00	0.00	0.00	0.00	0.00	0.00	0.00	0.00	0.00
0.00	0.00	0.00	0.00	0.00	0.00	0.00	0.00	0.00
0.00	0.00	0.00	0.00	0.00	0.00	0.00	0.00	0.00
0.00	0.00	0.00	0.00	0.00	0.00	0.00	0.00	0.00
0.00	0.00	0.00	0.00	0.00	0.00	0.00	0.00	0.00
0.00	0.00	0.00	0.00	0.00	0.00	0.00	0.00	0.00
0.00	0.00	0.00	0.00	0.00	0.00	0.00	0.00	0.00
0.00	0.00	0.00	0.00	0.00	0.00	0.00	0.00	0.00
0.00	0.00	0.00	0.00	0.00	0.00	0.00	0.00	0.00
0.00	0.00	0.00	0.00	0.00	0.00	0.00	0.00	0.00
0.00	0.00	0.00	0.00	0.00	0.00	0.00	0.00	0.00
0.00	0.00	0.00	0.00	0.00	0.00	0.00	0.00	0.00
0.00	0.00	0.00	0.00	0.00	0.00	0.00	0.00	0.00
0.00	0.00	0.00	0.00	0.00	0.00	0.00	0.00	0.00
32.00	0.00	0.00	0.00	0.00	0.00	0.00	0.00	0.00
0.00	0.00	0.00	0.00	0.00	0.00	0.00	0.00	0.00
0.00	0.00	0.00	0.00	0.00	0.00	0.00	0.00	0.00
72.00	0.00	0.00	0.00	0.00	0.00	0.00	0.00	99.00
0.00	0.00	0.00	0.00	0.00	0.00	0.00	0.00	0.00
0.00	0.00	0.00	0.00	0.00	0.00	0.00	0.00	0.00
0.00	0.00	0.00	0.00	0.00	0.00	0.00	0.00	0.00

0.00	0.00	0.00	0.00	0.00	0.00	0.00	0.00	0.00
0.00	0.00	0.00	0.00	0.00	0.00	0.00	0.00	0.00
0.00	0.00	0.00	0.00	0.00	0.00	0.00	0.00	0.00
0.00	0.00	0.00	0.00	0.00	0.00	0.00	0.00	0.00
0.00	59.00	0.00	0.00	0.00	0.00	0.00	0.00	0.00
0.00	0.00	0.00	0.00	0.00	0.00	0.00	0.00	0.00
0.00	0.00	0.00	0.00	0.00	0.00	0.00	0.00	0.00
56.00	6.00	0.00	0.00	0.00	45.00	79.00	46.00	46.00
0.00	0.00	0.00	0.00	0.00	0.00	0.00	0.00	0.00
0.00	11.00	0.00	4.00	5.00	5.00	5.00	0.00	0.00
0.00	0.00	0.00	0.00	0.00	0.00	0.00	0.00	0.00
0.00	0.00	0.00	0.00	0.00	0.00	0.00	0.00	7.00
0.00	0.00	0.00	0.00	0.00	0.00	0.00	0.00	0.00
0.00	0.00	0.00	0.00	0.00	0.00	0.00	0.00	0.00
36.00	0.00	0.00	7.00	4.00	27.00	43.00	0.00	102.00
0.00	0.00	0.00	0.00	0.00	0.00	0.00	0.00	0.00
0.00	0.00	0.00	0.00	0.00	0.00	0.00	0.00	0.00
0.00	0.00	0.00	0.00	0.00	0.00	0.00	0.00	0.00
0.00	0.00	0.00	0.00	0.00	0.00	0.00	0.00	0.00
0.00	320.00	0.00	0.00	0.00	0.00	18.00	0.00	0.00
0.00	0.00	0.00	0.00	0.00	0.00	0.00	0.00	0.00
0.00	0.00	0.00	0.00	0.00	0.00	0.00	0.00	0.00
0.00	0.00	0.00	0.00	0.00	0.00	0.00	0.00	0.00
136.00	0.00	41.00	0.00	0.00	0.00	0.00	0.00	25.00
0.00	0.00	0.00	0.00	0.00	0.00	0.00	0.00	0.00
0.00	0.00	0.00	0.00	0.00	0.00	0.00	0.00	0.00
0.00	0.00	0.00	0.00	0.00	0.00	0.00	0.00	0.00
0.00	48.00	0.00	0.00	0.00	13.00	63.00	32.00	0.00
0.00	0.00	0.00	0.00	0.00	0.00	0.00	0.00	0.00
0.00	0.00	0.00	0.00	0.00	0.00	0.00	0.00	0.00
0.00	9.00	0.00	0.00	0.00	0.00	0.00	0.00	0.00
0.00	0.00	0.00	0.00	0.00	0.00	0.00	0.00	0.00
0.00	0.00	0.00	0.00	0.00	0.00	0.00	0.00	0.00
0.00	0.00	0.00	0.00	0.00	0.00	0.00	0.00	0.00
0.00	0.00	0.00	0.00	0.00	0.00	0.00	0.00	0.00
0.00	0.00	0.00	0.00	0.00	0.00	0.00	0.00	0.00
0.00	0.00	88.00	0.00	0.00	0.00	0.00	0.00	0.00

34.00	0.00	0.00	0.00	0.00	0.00	0.00	0.00	0.00
46.00	0.00	14.00	0.00	0.00	0.00	0.00	64.00	36.00
13.00	0.00	0.00	0.00	0.00	23.00	0.00	0.00	0.00
0.00	0.00	0.00	0.00	0.00	0.00	0.00	0.00	0.00
0.00	0.00	0.00	0.00	0.00	0.00	0.00	0.00	0.00
0.00	0.00	0.00	0.00	0.00	0.00	0.00	0.00	0.00
0.00	0.00	0.00	0.00	0.00	0.00	0.00	0.00	0.00
0.00	0.00	0.00	0.00	0.00	0.00	0.00	0.00	0.00
0.00	0.00	0.00	0.00	0.00	0.00	0.00	0.00	0.00
0.00	0.00	0.00	0.00	0.00	0.00	0.00	0.00	0.00
71.00	0.00	0.00	0.00	0.00	0.00	0.00	0.00	0.00
0.00	0.00	0.00	0.00	0.00	0.00	0.00	0.00	0.00
0.00	0.00	0.00	0.00	0.00	0.00	0.00	0.00	0.00
0.00	0.00	0.00	0.00	0.00	0.00	0.00	0.00	0.00
0.00	0.00	0.00	0.00	0.00	0.00	0.00	0.00	0.00
0.00	0.00	0.00	0.00	0.00	0.00	0.00	0.00	0.00
55.00	47.00	0.00	0.00	0.00	15.00	0.00	0.00	0.00
0.00	0.00	0.00	0.00	0.00	0.00	0.00	0.00	0.00
0.00	0.00	0.00	0.00	0.00	0.00	0.00	0.00	0.00
0.00	0.00	0.00	0.00	0.00	0.00	0.00	0.00	0.00
0.00	0.00	0.00	0.00	0.00	0.00	0.00	0.00	0.00
0.00	0.00	0.00	0.00	0.00	0.00	0.00	0.00	0.00
0.00	0.00	0.00	11.00	0.00	20.00	0.00	0.00	27.00
0.00	0.00	0.00	0.00	0.00	0.00	0.00	0.00	0.00
0.00	0.00	0.00	0.00	0.00	0.00	0.00	0.00	0.00
16.00	0.00	25.00	0.00	0.00	10.00	0.00	16.00	49.00
0.00	0.00	0.00	0.00	0.00	0.00	0.00	0.00	0.00
0.00	0.00	0.00	0.00	0.00	0.00	0.00	0.00	0.00
22.00	0.00	0.00	0.00	0.00	0.00	0.00	0.00	66.00
0.00	0.00	0.00	0.00	0.00	0.00	0.00	0.00	0.00
0.00	0.00	0.00	0.00	0.00	0.00	0.00	0.00	0.00
0.00	0.00	0.00	0.00	0.00	0.00	0.00	0.00	0.00
0.00	0.00	0.00	0.00	0.00	0.00	0.00	0.00	0.00
30.00	0.00	0.00	0.00	0.00	0.00	0.00	0.00	0.00
0.00	232.00	0.00	0.00	0.00	0.00	0.00	0.00	0.00
0.00	0.00	0.00	0.00	0.00	0.00	0.00	0.00	0.00
0.00	0.00	0.00	0.00	0.00	0.00	0.00	0.00	0.00

0.00	0.00	0.00	0.00	0.00	0.00	0.00	0.00	0.00
0.00	0.00	0.00	0.00	0.00	0.00	0.00	0.00	0.00
0.00	0.00	0.00	0.00	0.00	0.00	0.00	0.00	0.00
14.00	0.00	0.00	0.00	0.00	0.00	0.00	0.00	0.00
0.00	0.00	0.00	0.00	0.00	0.00	0.00	0.00	0.00
0.00	0.00	0.00	0.00	0.00	0.00	0.00	0.00	0.00
0.00	63.00	0.00	0.00	0.00	0.00	0.00	0.00	74.00
0.00	0.00	0.00	0.00	0.00	0.00	0.00	0.00	0.00
0.00	0.00	0.00	0.00	0.00	0.00	0.00	0.00	0.00
0.00	0.00	0.00	0.00	0.00	0.00	0.00	0.00	0.00
7.00	0.00	0.00	0.00	0.00	0.00	0.00	0.00	0.00
0.00	0.00	0.00	0.00	0.00	0.00	0.00	0.00	0.00
0.00	0.00	0.00	0.00	0.00	0.00	0.00	0.00	0.00
0.00	0.00	0.00	0.00	0.00	0.00	0.00	0.00	0.00
0.00	0.00	0.00	0.00	0.00	0.00	0.00	0.00	0.00
0.00	0.00	0.00	0.00	0.00	0.00	0.00	0.00	0.00
0.00	0.00	0.00	0.00	0.00	0.00	0.00	0.00	0.00
0.00	0.00	0.00	0.00	0.00	0.00	0.00	0.00	0.00
0.00	0.00	0.00	0.00	0.00	0.00	0.00	0.00	0.00
0.00	0.00	0.00	0.00	0.00	0.00	0.00	0.00	0.00
0.00	0.00	0.00	0.00	0.00	0.00	0.00	0.00	0.00
0.00	0.00	0.00	0.00	0.00	0.00	0.00	0.00	0.00
0.00	0.00	0.00	0.00	0.00	0.00	0.00	0.00	0.00
0.00	0.00	0.00	0.00	0.00	0.00	0.00	0.00	0.00
0.00	0.00	0.00	0.00	0.00	0.00	0.00	0.00	0.00
0.00	0.00	0.00	0.00	0.00	0.00	0.00	0.00	0.00
0.00	0.00	0.00	0.00	0.00	0.00	0.00	0.00	0.00
0.00	0.00	0.00	0.00	0.00	0.00	0.00	0.00	0.00
0.00	0.00	0.00	0.00	0.00	0.00	0.00	0.00	0.00
5.00	0.00	5.00	0.00	0.00	0.00	0.00	0.00	5.00
0.00	0.00	0.00	0.00	0.00	0.00	0.00	0.00	0.00
0.00	0.00	0.00	0.00	0.00	0.00	0.00	0.00	0.00
0.00	0.00	0.00	0.00	0.00	0.00	0.00	0.00	0.00
0.00	0.00	0.00	0.00	0.00	0.00	0.00	0.00	0.00
0.00	0.00	0.00	0.00	0.00	47.00	0.00	0.00	27.00
0.00	0.00	0.00	0.00	0.00	0.00	0.00	0.00	0.00
0.00	0.00	0.00	0.00	0.00	0.00	0.00	0.00	0.00

0.00	0.00	0.00	0.00	0.00	61.00	0.00	0.00	7.00
52.00	0.00	0.00	0.00	0.00	0.00	0.00	0.00	0.00
0.00	0.00	0.00	0.00	0.00	0.00	0.00	0.00	0.00
0.00	0.00	0.00	0.00	0.00	0.00	0.00	0.00	0.00
0.00	0.00	0.00	0.00	0.00	0.00	0.00	0.00	0.00
0.00	0.00	0.00	0.00	0.00	0.00	0.00	0.00	0.00
6.00	0.00	0.00	0.00	0.00	0.00	0.00	0.00	0.00
0.00	0.00	0.00	0.00	0.00	0.00	0.00	0.00	0.00
0.00	0.00	0.00	0.00	0.00	0.00	0.00	0.00	0.00
11.00	153.00	0.00	0.00	0.00	0.00	0.00	0.00	28.00
0.00	0.00	0.00	0.00	0.00	0.00	0.00	0.00	0.00
0.00	0.00	0.00	0.00	0.00	0.00	0.00	0.00	0.00
0.00	0.00	0.00	0.00	0.00	0.00	0.00	0.00	0.00
38.00	0.00	0.00	0.00	0.00	0.00	66.00	0.00	42.00
0.00	0.00	0.00	0.00	0.00	0.00	0.00	0.00	0.00
11.00	0.00	7.00	14.00	0.00	18.00	0.00	0.00	33.00
0.00	0.00	0.00	0.00	0.00	0.00	0.00	0.00	0.00
0.00	0.00	0.00	0.00	0.00	0.00	0.00	0.00	0.00
0.00	0.00	0.00	0.00	0.00	0.00	0.00	0.00	0.00
0.00	0.00	0.00	0.00	0.00	0.00	0.00	0.00	0.00
0.00	0.00	0.00	0.00	0.00	0.00	0.00	0.00	0.00
0.00	0.00	0.00	0.00	0.00	0.00	0.00	0.00	0.00
0.00	0.00	0.00	0.00	0.00	0.00	0.00	0.00	0.00
0.00	0.00	24.00	0.00	0.00	0.00	0.00	0.00	0.00
0.00	0.00	0.00	0.00	0.00	0.00	0.00	0.00	0.00
0.00	0.00	0.00	0.00	0.00	0.00	0.00	0.00	0.00
0.00	0.00	0.00	0.00	0.00	0.00	0.00	0.00	0.00
0.00	0.00	0.00	0.00	0.00	0.00	0.00	0.00	0.00
0.00	0.00	0.00	0.00	0.00	0.00	0.00	0.00	0.00
2.00	0.00	0.00	0.00	0.00	0.00	0.00	0.00	0.00
0.00	0.00	0.00	8.00	0.00	156.00	0.00	0.00	0.00
0.00	0.00	0.00	0.00	0.00	0.00	0.00	0.00	0.00
0.00	0.00	0.00	0.00	0.00	0.00	0.00	0.00	0.00
0.00	19.00	0.00	0.00	0.00	0.00	0.00	0.00	0.00
0.00	0.00	0.00	0.00	0.00	0.00	0.00	0.00	0.00
0.00	0.00	0.00	0.00	0.00	0.00	44.00	0.00	17.00
31.00	52.00	10.00	5.00	7.00	3.00	0.00	66.00	19.00

0.00	0.00	0.00	0.00	0.00	0.00	0.00	0.00	0.00
0.00	0.00	0.00	0.00	0.00	0.00	0.00	0.00	0.00
0.00	0.00	0.00	0.00	0.00	0.00	0.00	0.00	0.00
0.00	0.00	0.00	0.00	0.00	0.00	0.00	0.00	0.00
0.00	0.00	0.00	0.00	0.00	0.00	0.00	0.00	0.00
0.00	0.00	0.00	0.00	0.00	0.00	0.00	0.00	0.00
0.00	0.00	0.00	0.00	0.00	0.00	0.00	0.00	0.00
0.00	36.00	0.00	0.00	0.00	0.00	0.00	0.00	5.00
0.00	0.00	0.00	0.00	0.00	0.00	0.00	0.00	0.00
0.00	0.00	0.00	0.00	0.00	0.00	0.00	0.00	0.00
0.00	0.00	0.00	0.00	0.00	0.00	0.00	0.00	0.00
0.00	0.00	0.00	0.00	0.00	0.00	0.00	0.00	0.00
0.00	0.00	0.00	0.00	0.00	0.00	0.00	0.00	0.00
14.00	0.00	0.00	0.00	0.00	0.00	0.00	0.00	0.00
0.00	0.00	0.00	0.00	0.00	0.00	0.00	0.00	0.00
0.00	0.00	0.00	0.00	0.00	0.00	0.00	0.00	0.00
0.00	0.00	0.00	0.00	0.00	0.00	0.00	0.00	0.00
0.00	0.00	0.00	0.00	0.00	0.00	0.00	0.00	0.00
0.00	0.00	0.00	0.00	0.00	0.00	0.00	0.00	0.00
0.00	0.00	0.00	0.00	0.00	0.00	0.00	0.00	0.00
0.00	0.00	0.00	0.00	0.00	0.00	0.00	0.00	0.00
0.00	0.00	0.00	0.00	0.00	0.00	0.00	0.00	0.00
0.00	0.00	0.00	0.00	0.00	0.00	0.00	0.00	0.00
0.00	0.00	0.00	0.00	0.00	0.00	0.00	0.00	0.00
0.00	0.00	0.00	0.00	0.00	0.00	0.00	0.00	0.00
0.00	0.00	0.00	0.00	0.00	0.00	0.00	0.00	0.00
0.00	0.00	0.00	0.00	0.00	0.00	0.00	0.00	0.00
0.00	0.00	0.00	0.00	0.00	0.00	0.00	0.00	0.00
0.00	0.00	0.00	0.00	0.00	0.00	0.00	0.00	0.00
0.00	0.00	0.00	0.00	0.00	0.00	0.00	0.00	0.00
0.00	0.00	0.00	0.00	0.00	0.00	0.00	0.00	0.00
0.00	0.00	0.00	0.00	0.00	0.00	0.00	0.00	0.00
0.00	0.00	0.00	0.00	0.00	0.00	0.00	0.00	0.00
0.00	0.00	0.00	0.00	0.00	0.00	0.00	0.00	0.00
19.00	0.00	0.00	0.00	0.00	33.00	24.00	0.00	13.00
0.00	0.00	0.00	0.00	0.00	0.00	0.00	0.00	0.00
0.00	0.00	0.00	0.00	0.00	0.00	0.00	0.00	0.00

0.00	0.00	0.00	0.00	0.00	0.00	0.00	0.00	0.00
0.00	0.00	0.00	0.00	0.00	0.00	0.00	0.00	0.00
0.00	0.00	0.00	0.00	0.00	0.00	0.00	0.00	0.00
0.00	0.00	0.00	0.00	0.00	0.00	0.00	0.00	0.00
0.00	0.00	0.00	0.00	0.00	0.00	0.00	0.00	0.00
0.00	0.00	0.00	0.00	0.00	0.00	0.00	0.00	0.00
0.00	0.00	0.00	0.00	0.00	0.00	0.00	0.00	0.00
0.00	0.00	0.00	0.00	0.00	0.00	0.00	0.00	0.00
0.00	0.00	0.00	0.00	0.00	0.00	0.00	0.00	0.00
0.00	0.00	0.00	0.00	0.00	0.00	0.00	0.00	0.00
0.00	0.00	0.00	0.00	0.00	0.00	0.00	0.00	0.00
0.00	0.00	0.00	0.00	0.00	0.00	0.00	0.00	0.00
0.00	0.00	0.00	0.00	0.00	0.00	0.00	0.00	0.00
0.00	0.00	0.00	0.00	0.00	0.00	0.00	0.00	0.00
0.00	0.00	0.00	0.00	0.00	0.00	0.00	0.00	0.00
0.00	0.00	0.00	0.00	0.00	0.00	0.00	0.00	0.00
0.00	0.00	0.00	0.00	0.00	0.00	0.00	0.00	0.00
0.00	0.00	0.00	0.00	0.00	0.00	0.00	0.00	0.00
0.00	0.00	0.00	0.00	0.00	0.00	0.00	0.00	0.00
0.00	0.00	0.00	0.00	0.00	0.00	0.00	0.00	0.00
0.00	94.00	8.00	0.00	0.00	0.00	0.00	0.00	0.00
0.00	0.00	0.00	0.00	0.00	0.00	0.00	0.00	18.00
0.00	0.00	0.00	0.00	0.00	0.00	0.00	0.00	0.00
0.00	0.00	0.00	0.00	0.00	0.00	0.00	0.00	0.00
0.00	0.00	0.00	0.00	0.00	0.00	0.00	0.00	0.00
0.00	0.00	0.00	0.00	0.00	0.00	0.00	0.00	0.00
0.00	0.00	0.00	0.00	0.00	0.00	0.00	0.00	0.00
0.00	182.00	0.00	0.00	0.00	0.00	0.00	0.00	0.00
0.00	0.00	0.00	0.00	0.00	0.00	0.00	0.00	0.00
8.00	106.00	0.00	0.00	0.00	5.00	0.00	0.00	0.00
0.00	0.00	0.00	0.00	0.00	0.00	0.00	0.00	0.00
0.00	0.00	0.00	0.00	0.00	0.00	0.00	0.00	0.00
0.00	0.00	0.00	0.00	0.00	0.00	0.00	0.00	0.00
0.00	0.00	0.00	0.00	0.00	0.00	0.00	0.00	0.00
0.00	0.00	0.00	0.00	0.00	0.00	0.00	0.00	0.00
35.00	0.00	0.00	0.00	10.00	12.00	0.00	0.00	13.00
0.00	0.00	0.00	0.00	0.00	0.00	0.00	0.00	0.00

3.00	0.00	0.00	0.00	0.00	28.00	0.00	0.00	0.00
0.00	0.00	0.00	3.00	0.00	0.00	148.00	0.00	0.00
13.00	0.00	0.00	0.00	0.00	0.00	0.00	0.00	17.00
0.00	0.00	0.00	0.00	0.00	0.00	0.00	0.00	0.00
0.00	0.00	0.00	0.00	0.00	0.00	0.00	0.00	0.00
0.00	0.00	0.00	0.00	0.00	0.00	0.00	0.00	0.00
0.00	0.00	0.00	0.00	0.00	0.00	0.00	0.00	0.00
0.00	0.00	0.00	0.00	0.00	0.00	0.00	0.00	0.00
0.00	0.00	0.00	0.00	0.00	0.00	0.00	0.00	0.00
0.00	0.00	0.00	0.00	0.00	0.00	0.00	0.00	0.00
0.00	0.00	0.00	0.00	0.00	0.00	0.00	0.00	0.00
0.00	0.00	0.00	0.00	0.00	0.00	0.00	0.00	0.00
0.00	0.00	0.00	0.00	0.00	0.00	0.00	0.00	0.00
0.00	0.00	0.00	0.00	0.00	0.00	0.00	0.00	0.00
0.00	0.00	0.00	0.00	0.00	0.00	0.00	0.00	0.00
0.00	0.00	0.00	0.00	0.00	0.00	0.00	0.00	0.00
0.00	0.00	0.00	0.00	0.00	0.00	0.00	0.00	0.00
65.00	0.00	0.00	3.00	0.00	4.00	0.00	0.00	0.00
0.00	0.00	0.00	0.00	0.00	0.00	0.00	0.00	0.00
0.00	0.00	0.00	0.00	0.00	0.00	0.00	0.00	0.00
0.00	0.00	0.00	0.00	0.00	0.00	0.00	0.00	0.00
0.00	0.00	0.00	0.00	0.00	0.00	0.00	0.00	0.00
0.00	0.00	0.00	0.00	0.00	0.00	0.00	0.00	0.00
0.00	0.00	0.00	0.00	0.00	14.00	0.00	0.00	0.00
0.00	0.00	0.00	0.00	0.00	0.00	0.00	0.00	0.00
0.00	207.00	0.00	0.00	0.00	0.00	0.00	0.00	0.00
0.00	0.00	0.00	0.00	0.00	0.00	0.00	0.00	0.00
0.00	0.00	0.00	0.00	0.00	0.00	0.00	0.00	0.00
0.00	0.00	0.00	0.00	0.00	0.00	0.00	0.00	0.00
0.00	0.00	0.00	0.00	0.00	0.00	0.00	0.00	0.00
31.00	0.00	30.00	0.00	0.00	11.00	0.00	0.00	0.00
0.00	210.00	0.00	0.00	0.00	0.00	0.00	0.00	0.00
0.00	0.00	0.00	0.00	0.00	0.00	0.00	0.00	0.00
0.00	0.00	0.00	0.00	0.00	0.00	0.00	0.00	0.00
0.00	0.00	0.00	0.00	0.00	0.00	0.00	0.00	0.00
0.00	0.00	0.00	0.00	0.00	0.00	0.00	0.00	0.00
0.00	0.00	0.00	0.00	0.00	0.00	0.00	0.00	0.00

0.00	0.00	0.00	17.00	0.00	0.00	0.00	0.00	0.00
0.00	0.00	0.00	0.00	0.00	0.00	0.00	0.00	0.00
0.00	0.00	0.00	0.00	0.00	0.00	0.00	60.00	0.00
13.00	0.00	0.00	0.00	0.00	14.00	0.00	0.00	32.00
2.00	0.00	0.00	0.00	0.00	0.00	0.00	0.00	0.00
0.00	0.00	0.00	0.00	0.00	0.00	0.00	0.00	0.00
0.00	0.00	0.00	0.00	0.00	0.00	0.00	0.00	0.00
0.00	0.00	0.00	0.00	0.00	0.00	0.00	0.00	0.00
0.00	0.00	0.00	0.00	0.00	0.00	0.00	0.00	0.00
0.00	0.00	0.00	0.00	0.00	0.00	0.00	0.00	0.00
0.00	0.00	0.00	0.00	0.00	0.00	0.00	0.00	0.00
0.00	0.00	0.00	0.00	0.00	0.00	0.00	0.00	0.00
0.00	0.00	0.00	0.00	0.00	0.00	0.00	0.00	0.00
0.00	0.00	0.00	0.00	0.00	0.00	0.00	0.00	0.00
0.00	0.00	0.00	0.00	0.00	0.00	0.00	0.00	0.00
0.00	0.00	0.00	0.00	0.00	0.00	0.00	0.00	0.00
0.00	0.00	0.00	0.00	0.00	0.00	0.00	0.00	0.00
0.00	0.00	0.00	0.00	0.00	0.00	0.00	0.00	0.00
0.00	0.00	0.00	0.00	0.00	0.00	0.00	0.00	0.00
0.00	0.00	0.00	0.00	0.00	0.00	0.00	0.00	0.00
0.00	0.00	0.00	0.00	0.00	0.00	0.00	0.00	0.00
0.00	0.00	0.00	0.00	0.00	0.00	0.00	0.00	0.00
0.00	0.00	0.00	0.00	0.00	0.00	0.00	0.00	0.00
29.00	0.00	0.00	0.00	0.00	0.00	0.00	0.00	0.00
0.00	0.00	0.00	0.00	0.00	0.00	0.00	0.00	0.00
0.00	0.00	0.00	0.00	0.00	0.00	0.00	0.00	0.00
0.00	0.00	0.00	0.00	0.00	0.00	0.00	0.00	0.00
0.00	0.00	0.00	0.00	0.00	45.00	0.00	0.00	0.00
0.00	0.00	0.00	0.00	0.00	0.00	0.00	0.00	0.00
0.00	0.00	0.00	0.00	0.00	0.00	0.00	0.00	0.00
0.00	0.00	0.00	8.00	0.00	0.00	0.00	0.00	6.00
0.00	0.00	0.00	0.00	0.00	0.00	0.00	0.00	0.00
0.00	0.00	0.00	0.00	0.00	0.00	0.00	0.00	0.00
0.00	0.00	0.00	0.00	0.00	0.00	0.00	0.00	0.00
0.00	0.00	0.00	0.00	0.00	0.00	0.00	0.00	0.00
0.00	0.00	0.00	0.00	0.00	0.00	0.00	0.00	0.00
0.00	0.00	0.00	0.00	0.00	0.00	0.00	0.00	0.00

0.00	0.00	0.00	0.00	0.00	0.00	0.00	0.00	0.00
0.00	0.00	0.00	0.00	0.00	0.00	0.00	0.00	0.00
0.00	0.00	0.00	0.00	0.00	0.00	0.00	0.00	0.00
0.00	0.00	0.00	0.00	0.00	0.00	0.00	0.00	0.00
0.00	0.00	0.00	0.00	0.00	0.00	0.00	0.00	0.00
0.00	0.00	0.00	0.00	0.00	0.00	0.00	0.00	0.00
0.00	0.00	0.00	0.00	0.00	0.00	0.00	0.00	0.00
0.00	0.00	0.00	0.00	0.00	0.00	0.00	0.00	85.00
0.00	0.00	0.00	0.00	0.00	0.00	0.00	0.00	0.00
0.00	0.00	0.00	0.00	0.00	0.00	0.00	0.00	0.00
0.00	0.00	0.00	0.00	0.00	0.00	0.00	0.00	0.00
0.00	0.00	0.00	0.00	0.00	0.00	0.00	0.00	0.00
0.00	0.00	0.00	0.00	0.00	0.00	0.00	0.00	0.00
0.00	0.00	0.00	0.00	0.00	0.00	0.00	0.00	0.00
0.00	0.00	0.00	0.00	0.00	0.00	0.00	0.00	0.00
0.00	0.00	0.00	0.00	0.00	0.00	0.00	0.00	0.00
0.00	0.00	0.00	0.00	0.00	0.00	0.00	0.00	0.00
0.00	0.00	0.00	0.00	0.00	0.00	0.00	0.00	0.00
0.00	0.00	0.00	0.00	0.00	0.00	0.00	0.00	0.00
0.00	0.00	0.00	0.00	0.00	0.00	0.00	0.00	0.00
0.00	0.00	0.00	0.00	0.00	0.00	0.00	0.00	0.00
0.00	0.00	0.00	0.00	0.00	0.00	0.00	0.00	0.00
0.00	0.00	0.00	0.00	0.00	0.00	164.00	0.00	0.00
0.00	0.00	0.00	0.00	0.00	0.00	0.00	0.00	0.00
0.00	0.00	0.00	0.00	0.00	0.00	0.00	0.00	0.00
0.00	0.00	0.00	0.00	0.00	0.00	0.00	0.00	0.00
0.00	0.00	0.00	0.00	0.00	0.00	0.00	0.00	0.00
0.00	0.00	0.00	0.00	0.00	0.00	0.00	0.00	0.00
0.00	0.00	0.00	0.00	0.00	0.00	0.00	0.00	0.00
0.00	0.00	0.00	0.00	0.00	0.00	0.00	0.00	0.00
0.00	0.00	0.00	0.00	0.00	0.00	0.00	0.00	0.00
0.00	0.00	0.00	0.00	0.00	0.00	0.00	0.00	0.00
32.00	0.00	0.00	0.00	4.00	0.00	0.00	0.00	0.00
0.00	0.00	0.00	0.00	0.00	0.00	0.00	0.00	0.00
0.00	0.00	0.00	0.00	0.00	0.00	0.00	0.00	0.00
0.00	0.00	0.00	0.00	0.00	0.00	0.00	0.00	0.00
0.00	0.00	0.00	0.00	0.00	0.00	0.00	0.00	0.00

0.00	0.00	0.00	0.00	0.00	0.00	0.00	0.00	0.00
0.00	0.00	0.00	0.00	0.00	0.00	0.00	0.00	0.00
7.00	0.00	0.00	0.00	0.00	14.00	0.00	0.00	10.00
0.00	84.00	0.00	0.00	0.00	0.00	0.00	0.00	0.00
0.00	0.00	0.00	0.00	4.00	0.00	0.00	0.00	0.00
15.00	0.00	0.00	0.00	0.00	0.00	0.00	0.00	0.00
0.00	0.00	0.00	0.00	0.00	0.00	0.00	0.00	0.00
0.00	0.00	0.00	0.00	0.00	0.00	0.00	0.00	0.00
0.00	0.00	0.00	0.00	0.00	0.00	0.00	0.00	0.00
0.00	0.00	0.00	0.00	0.00	0.00	0.00	0.00	0.00
0.00	0.00	0.00	0.00	0.00	0.00	0.00	0.00	0.00
0.00	0.00	0.00	0.00	0.00	0.00	0.00	0.00	0.00
0.00	0.00	0.00	0.00	0.00	0.00	0.00	0.00	0.00
0.00	0.00	0.00	0.00	0.00	0.00	0.00	0.00	0.00
20.00	0.00	0.00	0.00	0.00	0.00	0.00	0.00	36.00
0.00	0.00	0.00	0.00	0.00	0.00	0.00	0.00	0.00
0.00	0.00	0.00	0.00	0.00	0.00	0.00	0.00	0.00
0.00	0.00	0.00	0.00	0.00	0.00	0.00	0.00	0.00
0.00	0.00	0.00	0.00	0.00	0.00	0.00	0.00	0.00
3.00	0.00	0.00	0.00	0.00	0.00	0.00	0.00	0.00
0.00	0.00	0.00	0.00	0.00	0.00	0.00	0.00	0.00
0.00	0.00	0.00	0.00	0.00	0.00	0.00	0.00	0.00
0.00	0.00	0.00	0.00	0.00	0.00	0.00	0.00	0.00
0.00	0.00	0.00	0.00	0.00	0.00	0.00	0.00	0.00
0.00	0.00	0.00	0.00	0.00	0.00	0.00	0.00	0.00
0.00	0.00	0.00	0.00	0.00	0.00	0.00	0.00	0.00
0.00	0.00	0.00	0.00	0.00	0.00	0.00	0.00	0.00
0.00	0.00	0.00	0.00	0.00	0.00	0.00	0.00	0.00
0.00	0.00	0.00	0.00	0.00	0.00	0.00	0.00	0.00
19.00	0.00	12.00	0.00	0.00	0.00	0.00	0.00	46.00
0.00	0.00	0.00	0.00	0.00	0.00	0.00	0.00	0.00
0.00	0.00	0.00	0.00	0.00	0.00	0.00	0.00	0.00
0.00	0.00	0.00	0.00	0.00	0.00	0.00	0.00	0.00
0.00	0.00	0.00	0.00	0.00	0.00	0.00	0.00	0.00
0.00	0.00	0.00	0.00	0.00	0.00	0.00	0.00	0.00
0.00	0.00	0.00	0.00	0.00	0.00	0.00	0.00	0.00
0.00	0.00	0.00	0.00	0.00	0.00	0.00	0.00	0.00

0.00	0.00	0.00	0.00	0.00	0.00	0.00	0.00	0.00
0.00	0.00	0.00	0.00	0.00	0.00	0.00	0.00	0.00
0.00	0.00	0.00	0.00	0.00	0.00	0.00	0.00	0.00
10.00	0.00	0.00	0.00	0.00	0.00	0.00	0.00	0.00
0.00	0.00	0.00	0.00	0.00	0.00	0.00	0.00	0.00
0.00	0.00	0.00	0.00	0.00	0.00	0.00	0.00	0.00
0.00	0.00	0.00	0.00	0.00	0.00	0.00	0.00	0.00
0.00	0.00	0.00	0.00	0.00	0.00	0.00	0.00	0.00
0.00	0.00	0.00	0.00	0.00	28.00	0.00	0.00	0.00
0.00	0.00	0.00	0.00	0.00	22.00	0.00	0.00	14.00
0.00	0.00	0.00	0.00	0.00	0.00	0.00	129.00	0.00
0.00	0.00	0.00	0.00	0.00	0.00	0.00	0.00	0.00
0.00	0.00	0.00	0.00	0.00	0.00	0.00	0.00	0.00
0.00	0.00	0.00	0.00	0.00	0.00	28.00	0.00	0.00
0.00	0.00	0.00	0.00	0.00	0.00	0.00	0.00	0.00
0.00	0.00	0.00	0.00	0.00	0.00	0.00	0.00	0.00
0.00	123.00	0.00	0.00	0.00	0.00	0.00	0.00	0.00
0.00	0.00	0.00	0.00	0.00	0.00	0.00	0.00	0.00
0.00	0.00	0.00	0.00	0.00	0.00	0.00	0.00	0.00
0.00	122.00	0.00	0.00	0.00	0.00	0.00	0.00	0.00
0.00	0.00	0.00	0.00	0.00	0.00	0.00	0.00	0.00
0.00	0.00	0.00	0.00	0.00	0.00	0.00	0.00	0.00
0.00	0.00	0.00	0.00	0.00	0.00	0.00	0.00	0.00
0.00	0.00	0.00	0.00	0.00	0.00	0.00	0.00	0.00
0.00	0.00	0.00	0.00	0.00	0.00	0.00	0.00	0.00
0.00	0.00	0.00	0.00	0.00	0.00	0.00	0.00	0.00
0.00	0.00	0.00	0.00	0.00	0.00	0.00	0.00	0.00
0.00	0.00	0.00	0.00	0.00	0.00	0.00	0.00	0.00
0.00	0.00	0.00	0.00	0.00	0.00	0.00	0.00	0.00
0.00	0.00	0.00	0.00	0.00	0.00	0.00	0.00	0.00
0.00	0.00	0.00	0.00	0.00	0.00	0.00	0.00	0.00
0.00	0.00	0.00	0.00	0.00	0.00	0.00	0.00	0.00
0.00	0.00	0.00	0.00	0.00	0.00	0.00	0.00	0.00
0.00	0.00	0.00	0.00	0.00	0.00	116.00	0.00	0.00
0.00	0.00	0.00	0.00	0.00	0.00	0.00	0.00	0.00
0.00	0.00	0.00	0.00	0.00	0.00	0.00	0.00	0.00
0.00	0.00	0.00	0.00	0.00	0.00	0.00	0.00	0.00

0.00	0.00	0.00	0.00	0.00	0.00	115.00	0.00	0.00
0.00	0.00	0.00	0.00	0.00	0.00	0.00	0.00	0.00
0.00	0.00	0.00	0.00	0.00	0.00	0.00	0.00	113.00
0.00	0.00	0.00	0.00	0.00	0.00	0.00	0.00	0.00
0.00	0.00	0.00	0.00	0.00	0.00	0.00	0.00	0.00
0.00	0.00	0.00	0.00	0.00	0.00	0.00	0.00	0.00
0.00	0.00	0.00	0.00	0.00	0.00	0.00	0.00	0.00
0.00	0.00	0.00	0.00	0.00	0.00	0.00	0.00	0.00
0.00	0.00	0.00	0.00	0.00	0.00	0.00	0.00	0.00
0.00	0.00	0.00	0.00	0.00	0.00	0.00	0.00	0.00
0.00	0.00	0.00	0.00	0.00	0.00	0.00	0.00	0.00
0.00	0.00	0.00	0.00	0.00	0.00	0.00	0.00	0.00
0.00	0.00	0.00	0.00	0.00	0.00	12.00	0.00	11.00
0.00	0.00	0.00	0.00	0.00	0.00	0.00	0.00	0.00
0.00	0.00	0.00	0.00	0.00	0.00	0.00	0.00	0.00
0.00	0.00	0.00	0.00	0.00	0.00	0.00	0.00	0.00
0.00	107.00	0.00	0.00	0.00	0.00	0.00	0.00	0.00
0.00	0.00	0.00	0.00	0.00	0.00	0.00	0.00	0.00
0.00	0.00	0.00	0.00	0.00	0.00	0.00	0.00	0.00
0.00	0.00	0.00	0.00	0.00	0.00	0.00	0.00	0.00
0.00	0.00	0.00	0.00	0.00	0.00	0.00	0.00	0.00
0.00	0.00	0.00	0.00	0.00	0.00	0.00	0.00	0.00
0.00	0.00	0.00	0.00	0.00	0.00	0.00	0.00	0.00
9.00	0.00	0.00	0.00	0.00	0.00	0.00	0.00	0.00
0.00	0.00	0.00	0.00	0.00	0.00	0.00	0.00	0.00
0.00	0.00	0.00	0.00	0.00	0.00	0.00	0.00	0.00
0.00	0.00	0.00	0.00	0.00	0.00	0.00	0.00	0.00
0.00	0.00	0.00	0.00	0.00	0.00	0.00	0.00	0.00
0.00	0.00	0.00	0.00	0.00	0.00	0.00	0.00	0.00
0.00	0.00	0.00	0.00	0.00	10.00	0.00	0.00	0.00
0.00	0.00	0.00	0.00	0.00	0.00	0.00	0.00	0.00
0.00	0.00	0.00	0.00	0.00	0.00	0.00	0.00	0.00
0.00	0.00	0.00	0.00	0.00	0.00	0.00	0.00	0.00
0.00	0.00	0.00	0.00	0.00	0.00	0.00	0.00	0.00
0.00	0.00	0.00	0.00	0.00	0.00	0.00	0.00	0.00
0.00	101.00	0.00	0.00	0.00	0.00	0.00	0.00	0.00
0.00	101.00	0.00	0.00	0.00	0.00	0.00	0.00	0.00

0.00	0.00	0.00	0.00	0.00	0.00	0.00	0.00	0.00
0.00	0.00	0.00	0.00	0.00	0.00	0.00	0.00	0.00
0.00	0.00	0.00	0.00	0.00	0.00	0.00	0.00	0.00
0.00	0.00	0.00	0.00	0.00	0.00	0.00	0.00	0.00
0.00	0.00	0.00	0.00	0.00	0.00	0.00	0.00	0.00
0.00	0.00	0.00	0.00	0.00	0.00	0.00	0.00	0.00
0.00	0.00	0.00	0.00	0.00	0.00	0.00	0.00	0.00
0.00	0.00	0.00	0.00	0.00	0.00	0.00	0.00	0.00
0.00	0.00	0.00	0.00	0.00	0.00	87.00	0.00	0.00
0.00	0.00	0.00	0.00	0.00	0.00	0.00	0.00	0.00
0.00	0.00	0.00	0.00	0.00	0.00	0.00	0.00	0.00
0.00	0.00	0.00	0.00	0.00	0.00	0.00	0.00	0.00
0.00	0.00	0.00	0.00	0.00	0.00	0.00	0.00	0.00
0.00	0.00	0.00	0.00	0.00	0.00	0.00	0.00	0.00
0.00	0.00	0.00	0.00	0.00	0.00	0.00	0.00	0.00
0.00	0.00	0.00	0.00	0.00	0.00	0.00	0.00	0.00
3.00	0.00	0.00	0.00	0.00	0.00	5.00	61.00	0.00
0.00	0.00	0.00	0.00	0.00	0.00	0.00	0.00	0.00
0.00	97.00	0.00	0.00	0.00	0.00	0.00	0.00	0.00
0.00	0.00	0.00	0.00	0.00	0.00	0.00	0.00	0.00
0.00	0.00	0.00	0.00	0.00	0.00	0.00	0.00	0.00
0.00	0.00	0.00	0.00	0.00	0.00	0.00	0.00	0.00
0.00	0.00	0.00	0.00	0.00	0.00	0.00	0.00	0.00
0.00	0.00	0.00	0.00	0.00	0.00	0.00	0.00	0.00
0.00	0.00	0.00	0.00	0.00	0.00	0.00	0.00	0.00
0.00	0.00	0.00	0.00	0.00	0.00	0.00	0.00	0.00
0.00	0.00	0.00	0.00	0.00	0.00	0.00	0.00	0.00
0.00	0.00	0.00	0.00	0.00	0.00	93.00	0.00	0.00
0.00	0.00	0.00	0.00	0.00	0.00	0.00	0.00	0.00
0.00	0.00	0.00	0.00	0.00	0.00	0.00	0.00	0.00
0.00	0.00	0.00	0.00	0.00	23.00	0.00	0.00	0.00
0.00	0.00	0.00	0.00	0.00	0.00	0.00	0.00	0.00
0.00	0.00	0.00	0.00	0.00	0.00	0.00	0.00	0.00
0.00	0.00	0.00	0.00	0.00	0.00	0.00	0.00	0.00
0.00	0.00	0.00	0.00	0.00	0.00	0.00	0.00	0.00
0.00	0.00	0.00	0.00	0.00	0.00	0.00	0.00	0.00
8.00	0.00	0.00	0.00	0.00	0.00	0.00	0.00	0.00

0.00	0.00	0.00	0.00	0.00	0.00	0.00	0.00	0.00
0.00	0.00	0.00	0.00	0.00	0.00	0.00	0.00	0.00
7.00	0.00	0.00	0.00	0.00	0.00	0.00	0.00	14.00
0.00	0.00	0.00	0.00	0.00	0.00	0.00	0.00	0.00
0.00	0.00	0.00	0.00	0.00	0.00	0.00	0.00	0.00
0.00	0.00	0.00	0.00	0.00	0.00	0.00	0.00	0.00
0.00	0.00	0.00	0.00	0.00	0.00	0.00	0.00	0.00
0.00	0.00	0.00	0.00	0.00	0.00	0.00	0.00	0.00
0.00	0.00	0.00	0.00	0.00	0.00	0.00	0.00	0.00
0.00	0.00	0.00	0.00	0.00	0.00	0.00	0.00	0.00
0.00	0.00	0.00	0.00	0.00	0.00	0.00	0.00	0.00
0.00	0.00	0.00	0.00	0.00	0.00	0.00	0.00	0.00
0.00	0.00	0.00	0.00	0.00	0.00	0.00	0.00	0.00
0.00	0.00	0.00	0.00	0.00	0.00	0.00	0.00	0.00
0.00	0.00	0.00	0.00	0.00	0.00	0.00	0.00	0.00
0.00	0.00	0.00	0.00	0.00	0.00	0.00	0.00	0.00
0.00	0.00	0.00	0.00	0.00	0.00	0.00	0.00	0.00
0.00	0.00	0.00	0.00	0.00	0.00	0.00	0.00	0.00
0.00	0.00	0.00	0.00	0.00	0.00	0.00	85.00	0.00
0.00	0.00	0.00	0.00	0.00	0.00	0.00	0.00	0.00
0.00	0.00	0.00	0.00	0.00	0.00	0.00	0.00	0.00
0.00	0.00	0.00	0.00	0.00	0.00	0.00	0.00	0.00
0.00	0.00	0.00	0.00	0.00	0.00	0.00	0.00	0.00
0.00	0.00	0.00	0.00	0.00	0.00	0.00	0.00	0.00
0.00	0.00	0.00	0.00	0.00	0.00	0.00	0.00	0.00
0.00	0.00	0.00	0.00	0.00	0.00	0.00	0.00	0.00
0.00	0.00	0.00	0.00	0.00	0.00	0.00	0.00	0.00
0.00	0.00	0.00	0.00	0.00	0.00	0.00	0.00	0.00
0.00	0.00	0.00	0.00	0.00	0.00	0.00	0.00	0.00
0.00	0.00	0.00	0.00	0.00	0.00	0.00	0.00	0.00
0.00	0.00	0.00	0.00	0.00	0.00	0.00	0.00	0.00
0.00	0.00	0.00	0.00	0.00	0.00	0.00	0.00	0.00
0.00	0.00	0.00	0.00	0.00	0.00	0.00	0.00	0.00
0.00	0.00	0.00	0.00	0.00	0.00	0.00	0.00	0.00
0.00	0.00	0.00	0.00	0.00	0.00	0.00	0.00	0.00
0.00	0.00	0.00	0.00	0.00	0.00	0.00	0.00	0.00
0.00	0.00	0.00	0.00	0.00	0.00	0.00	0.00	0.00

0.00	0.00	0.00	0.00	0.00	0.00	0.00	0.00	0.00
0.00	0.00	0.00	0.00	0.00	0.00	0.00	0.00	0.00
0.00	0.00	0.00	0.00	0.00	0.00	0.00	0.00	0.00
0.00	0.00	0.00	0.00	0.00	0.00	0.00	0.00	0.00
0.00	0.00	0.00	0.00	0.00	0.00	0.00	0.00	0.00
0.00	0.00	0.00	0.00	0.00	0.00	0.00	0.00	0.00
0.00	0.00	0.00	0.00	0.00	0.00	0.00	0.00	0.00
9.00	0.00	0.00	0.00	0.00	0.00	0.00	0.00	0.00
0.00	0.00	0.00	0.00	0.00	0.00	0.00	0.00	0.00
0.00	0.00	0.00	0.00	0.00	0.00	0.00	0.00	0.00
0.00	0.00	0.00	0.00	0.00	0.00	0.00	0.00	0.00
0.00	0.00	0.00	0.00	0.00	0.00	0.00	0.00	0.00
0.00	0.00	0.00	0.00	0.00	0.00	0.00	0.00	0.00
0.00	0.00	0.00	0.00	0.00	0.00	0.00	0.00	0.00
0.00	75.00	0.00	0.00	0.00	0.00	0.00	0.00	0.00
0.00	0.00	0.00	0.00	0.00	0.00	0.00	0.00	0.00
0.00	0.00	0.00	0.00	0.00	0.00	0.00	0.00	0.00
0.00	0.00	0.00	0.00	0.00	0.00	0.00	0.00	0.00
0.00	0.00	0.00	0.00	0.00	0.00	0.00	0.00	0.00
9.00	0.00	0.00	0.00	0.00	0.00	0.00	0.00	0.00
0.00	0.00	0.00	0.00	0.00	0.00	0.00	0.00	0.00
0.00	0.00	0.00	0.00	0.00	0.00	0.00	0.00	0.00
10.00	0.00	0.00	0.00	0.00	0.00	0.00	0.00	0.00
0.00	0.00	0.00	0.00	0.00	0.00	0.00	0.00	0.00
0.00	0.00	0.00	0.00	0.00	0.00	0.00	0.00	0.00
0.00	0.00	0.00	0.00	0.00	0.00	0.00	0.00	0.00
0.00	0.00	0.00	0.00	0.00	0.00	0.00	0.00	0.00
0.00	0.00	0.00	0.00	0.00	0.00	0.00	0.00	0.00
0.00	0.00	0.00	0.00	0.00	0.00	0.00	0.00	0.00
0.00	0.00	0.00	0.00	0.00	0.00	0.00	0.00	0.00
0.00	0.00	0.00	0.00	0.00	0.00	0.00	0.00	0.00
0.00	0.00	0.00	0.00	0.00	0.00	0.00	0.00	0.00
0.00	0.00	0.00	0.00	0.00	0.00	0.00	0.00	0.00
0.00	0.00	0.00	0.00	0.00	0.00	0.00	0.00	0.00
0.00	0.00	0.00	0.00	0.00	0.00	0.00	0.00	0.00
0.00	0.00	0.00	0.00	0.00	0.00	0.00	0.00	0.00
0.00	0.00	0.00	0.00	0.00	0.00	0.00	0.00	0.00

0.00	0.00	0.00	0.00	0.00	0.00	0.00	0.00	0.00
0.00	0.00	69.00	0.00	0.00	0.00	0.00	0.00	0.00
0.00	0.00	0.00	0.00	0.00	0.00	0.00	0.00	0.00
0.00	0.00	0.00	0.00	0.00	0.00	0.00	0.00	0.00
0.00	0.00	0.00	0.00	0.00	0.00	0.00	0.00	0.00
0.00	0.00	0.00	0.00	0.00	0.00	0.00	0.00	0.00
0.00	0.00	0.00	0.00	0.00	0.00	0.00	0.00	0.00
0.00	0.00	0.00	0.00	0.00	0.00	0.00	0.00	0.00
0.00	0.00	0.00	0.00	0.00	0.00	0.00	0.00	0.00
0.00	0.00	0.00	0.00	0.00	0.00	0.00	0.00	0.00
0.00	0.00	0.00	0.00	0.00	0.00	0.00	0.00	0.00
0.00	0.00	0.00	0.00	0.00	0.00	0.00	0.00	0.00
0.00	0.00	0.00	0.00	0.00	0.00	0.00	0.00	0.00
0.00	0.00	0.00	0.00	0.00	0.00	0.00	0.00	0.00
0.00	0.00	0.00	0.00	0.00	0.00	0.00	0.00	0.00
0.00	0.00	0.00	0.00	0.00	0.00	0.00	0.00	0.00
0.00	0.00	0.00	0.00	0.00	0.00	0.00	0.00	0.00
0.00	0.00	0.00	0.00	0.00	0.00	0.00	0.00	0.00
0.00	0.00	0.00	0.00	0.00	0.00	0.00	67.00	0.00
0.00	0.00	0.00	0.00	0.00	0.00	0.00	0.00	0.00
0.00	0.00	0.00	0.00	0.00	0.00	0.00	0.00	0.00
0.00	0.00	0.00	0.00	0.00	0.00	0.00	0.00	0.00
0.00	0.00	0.00	0.00	0.00	0.00	0.00	0.00	0.00
0.00	0.00	0.00	0.00	0.00	0.00	0.00	0.00	0.00
0.00	0.00	0.00	0.00	0.00	0.00	0.00	0.00	0.00
0.00	0.00	0.00	0.00	0.00	0.00	0.00	0.00	0.00
0.00	0.00	0.00	0.00	0.00	0.00	0.00	0.00	0.00
0.00	0.00	0.00	0.00	0.00	0.00	0.00	0.00	0.00
0.00	0.00	0.00	0.00	0.00	0.00	0.00	0.00	0.00
0.00	0.00	0.00	0.00	0.00	0.00	0.00	0.00	0.00
0.00	0.00	0.00	0.00	0.00	0.00	0.00	0.00	0.00
0.00	0.00	0.00	0.00	0.00	0.00	0.00	0.00	0.00
0.00	0.00	0.00	0.00	0.00	0.00	0.00	0.00	0.00
0.00	0.00	0.00	0.00	0.00	13.00	0.00	0.00	0.00
0.00	0.00	0.00	3.00	0.00	0.00	0.00	0.00	0.00
0.00	0.00	0.00	0.00	0.00	0.00	0.00	0.00	0.00

0.00	0.00	0.00	0.00	0.00	0.00	0.00	0.00	0.00
0.00	0.00	0.00	0.00	0.00	0.00	0.00	0.00	0.00
0.00	0.00	0.00	0.00	0.00	0.00	0.00	0.00	0.00
0.00	0.00	0.00	0.00	0.00	0.00	0.00	0.00	0.00
0.00	0.00	0.00	0.00	0.00	0.00	0.00	0.00	0.00
0.00	0.00	0.00	0.00	0.00	0.00	0.00	0.00	0.00
0.00	0.00	0.00	0.00	0.00	0.00	0.00	0.00	0.00
0.00	0.00	0.00	0.00	0.00	0.00	0.00	0.00	0.00
0.00	0.00	0.00	0.00	0.00	0.00	0.00	0.00	0.00
0.00	0.00	0.00	0.00	0.00	0.00	0.00	0.00	0.00
0.00	0.00	0.00	0.00	0.00	0.00	0.00	0.00	0.00
0.00	0.00	0.00	0.00	0.00	0.00	0.00	0.00	0.00
0.00	0.00	0.00	0.00	0.00	0.00	0.00	0.00	0.00
0.00	0.00	0.00	0.00	0.00	0.00	0.00	0.00	0.00
0.00	0.00	0.00	0.00	0.00	0.00	0.00	0.00	0.00
4.00	0.00	0.00	0.00	0.00	0.00	0.00	0.00	0.00
0.00	0.00	0.00	0.00	0.00	0.00	0.00	0.00	23.00
0.00	0.00	0.00	0.00	0.00	0.00	0.00	0.00	0.00
0.00	0.00	0.00	0.00	0.00	0.00	0.00	0.00	0.00
0.00	0.00	0.00	0.00	0.00	0.00	0.00	0.00	0.00
0.00	0.00	0.00	0.00	0.00	0.00	0.00	0.00	0.00
0.00	0.00	0.00	0.00	0.00	0.00	0.00	0.00	0.00
0.00	0.00	0.00	0.00	0.00	0.00	0.00	0.00	0.00
0.00	0.00	0.00	0.00	0.00	0.00	0.00	0.00	0.00
0.00	0.00	0.00	0.00	0.00	0.00	0.00	0.00	0.00
0.00	0.00	0.00	0.00	0.00	0.00	0.00	0.00	0.00
0.00	0.00	0.00	0.00	0.00	0.00	60.00	0.00	0.00
0.00	0.00	0.00	0.00	0.00	0.00	0.00	0.00	0.00
0.00	0.00	0.00	0.00	0.00	0.00	0.00	0.00	0.00
0.00	0.00	0.00	0.00	0.00	0.00	0.00	0.00	0.00
0.00	0.00	0.00	0.00	0.00	0.00	0.00	0.00	0.00
0.00	0.00	0.00	0.00	0.00	0.00	0.00	0.00	0.00
0.00	0.00	0.00	0.00	0.00	0.00	0.00	0.00	0.00
0.00	0.00	0.00	0.00	0.00	0.00	0.00	0.00	0.00
0.00	0.00	0.00	0.00	0.00	0.00	0.00	0.00	0.00
0.00	0.00	0.00	0.00	0.00	0.00	0.00	0.00	0.00
22.00	0.00	0.00	0.00	0.00	0.00	0.00	0.00	0.00

0.00	0.00	0.00	0.00	0.00	0.00	0.00	0.00	39.00
0.00	0.00	0.00	0.00	0.00	0.00	0.00	0.00	0.00
0.00	0.00	0.00	0.00	0.00	0.00	0.00	0.00	0.00
0.00	0.00	0.00	0.00	0.00	0.00	0.00	0.00	0.00
0.00	0.00	0.00	0.00	0.00	0.00	0.00	0.00	0.00
0.00	0.00	0.00	0.00	0.00	0.00	0.00	58.00	0.00
0.00	0.00	0.00	58.00	0.00	0.00	0.00	0.00	0.00
0.00	0.00	0.00	0.00	0.00	0.00	0.00	0.00	0.00
0.00	0.00	0.00	0.00	0.00	0.00	0.00	0.00	0.00
0.00	0.00	0.00	0.00	0.00	0.00	0.00	0.00	0.00
0.00	0.00	0.00	0.00	0.00	0.00	0.00	0.00	0.00
0.00	0.00	0.00	0.00	0.00	0.00	0.00	0.00	0.00
0.00	0.00	0.00	0.00	0.00	0.00	0.00	0.00	0.00
57.00	0.00	0.00	0.00	0.00	0.00	0.00	0.00	0.00
0.00	0.00	0.00	0.00	0.00	0.00	0.00	0.00	0.00
0.00	0.00	0.00	0.00	0.00	0.00	0.00	0.00	0.00
0.00	0.00	0.00	0.00	0.00	0.00	0.00	0.00	0.00
0.00	0.00	0.00	0.00	0.00	0.00	0.00	0.00	0.00
0.00	0.00	0.00	0.00	0.00	0.00	0.00	0.00	0.00
0.00	0.00	0.00	0.00	0.00	10.00	0.00	0.00	0.00
0.00	0.00	0.00	0.00	0.00	0.00	0.00	0.00	0.00
0.00	0.00	0.00	0.00	0.00	0.00	0.00	0.00	0.00
0.00	0.00	0.00	0.00	0.00	0.00	0.00	0.00	0.00
0.00	0.00	0.00	0.00	0.00	0.00	0.00	0.00	0.00
0.00	0.00	0.00	0.00	0.00	0.00	0.00	0.00	0.00
0.00	0.00	0.00	0.00	0.00	0.00	0.00	0.00	0.00
0.00	0.00	0.00	0.00	0.00	0.00	0.00	0.00	0.00
0.00	0.00	0.00	0.00	0.00	0.00	0.00	0.00	0.00
0.00	0.00	0.00	0.00	0.00	0.00	0.00	0.00	0.00
0.00	0.00	0.00	0.00	0.00	0.00	0.00	0.00	0.00
0.00	0.00	0.00	0.00	0.00	0.00	0.00	0.00	0.00
0.00	0.00	0.00	0.00	0.00	0.00	0.00	0.00	0.00
0.00	0.00	55.00	0.00	0.00	0.00	0.00	0.00	0.00
0.00	55.00	0.00	0.00	0.00	0.00	0.00	0.00	0.00
0.00	0.00	0.00	0.00	0.00	0.00	0.00	0.00	0.00
0.00	0.00	0.00	0.00	0.00	0.00	0.00	0.00	0.00
0.00	0.00	0.00	0.00	0.00	0.00	0.00	0.00	0.00

0.00	0.00	0.00	0.00	0.00	0.00	0.00	0.00	0.00
0.00	0.00	0.00	0.00	0.00	0.00	0.00	0.00	0.00
0.00	0.00	0.00	0.00	0.00	0.00	0.00	0.00	0.00
0.00	0.00	0.00	0.00	0.00	0.00	0.00	0.00	0.00
0.00	0.00	0.00	0.00	0.00	0.00	0.00	0.00	0.00
0.00	0.00	0.00	0.00	0.00	0.00	0.00	0.00	0.00
0.00	0.00	0.00	0.00	0.00	0.00	0.00	0.00	0.00
0.00	0.00	0.00	0.00	0.00	0.00	0.00	0.00	0.00
0.00	0.00	0.00	0.00	0.00	0.00	0.00	53.00	0.00
0.00	0.00	0.00	0.00	0.00	0.00	0.00	0.00	0.00
0.00	0.00	0.00	0.00	0.00	0.00	0.00	0.00	0.00
0.00	0.00	0.00	0.00	0.00	0.00	0.00	0.00	0.00
0.00	0.00	0.00	0.00	0.00	0.00	0.00	0.00	0.00
0.00	0.00	0.00	0.00	0.00	0.00	0.00	0.00	0.00
0.00	0.00	0.00	0.00	0.00	0.00	0.00	0.00	0.00
0.00	0.00	0.00	0.00	0.00	0.00	0.00	0.00	0.00
0.00	0.00	0.00	0.00	0.00	0.00	0.00	0.00	0.00
0.00	0.00	0.00	0.00	0.00	0.00	0.00	0.00	0.00
28.00	0.00	0.00	0.00	0.00	0.00	0.00	0.00	0.00
0.00	0.00	0.00	0.00	0.00	0.00	0.00	0.00	0.00
0.00	0.00	0.00	0.00	0.00	0.00	0.00	0.00	0.00
0.00	0.00	0.00	0.00	0.00	0.00	0.00	0.00	0.00
0.00	0.00	0.00	0.00	0.00	0.00	0.00	0.00	0.00
0.00	0.00	0.00	0.00	0.00	0.00	0.00	0.00	0.00
0.00	0.00	0.00	0.00	0.00	0.00	0.00	0.00	0.00
0.00	0.00	0.00	0.00	0.00	0.00	0.00	0.00	0.00
0.00	0.00	0.00	0.00	0.00	0.00	0.00	0.00	0.00
0.00	0.00	0.00	0.00	0.00	0.00	0.00	0.00	0.00
0.00	0.00	0.00	0.00	0.00	0.00	0.00	0.00	0.00
0.00	0.00	0.00	0.00	0.00	0.00	0.00	0.00	0.00
0.00	0.00	0.00	0.00	0.00	0.00	0.00	0.00	0.00
22.00	0.00	0.00	0.00	0.00	0.00	0.00	0.00	0.00
0.00	0.00	0.00	0.00	0.00	0.00	0.00	0.00	0.00
0.00	0.00	0.00	0.00	0.00	0.00	0.00	0.00	0.00
0.00	0.00	0.00	0.00	0.00	0.00	0.00	0.00	0.00
0.00	0.00	0.00	0.00	0.00	0.00	0.00	0.00	0.00
0.00	0.00	0.00	0.00	0.00	0.00	0.00	0.00	0.00

0.00	0.00	0.00	0.00	0.00	0.00	0.00	0.00	0.00
0.00	0.00	0.00	0.00	0.00	0.00	0.00	0.00	0.00
0.00	0.00	0.00	0.00	0.00	0.00	0.00	0.00	0.00
0.00	0.00	0.00	0.00	0.00	0.00	0.00	0.00	0.00
0.00	0.00	0.00	0.00	0.00	0.00	0.00	0.00	0.00
0.00	0.00	0.00	0.00	0.00	0.00	0.00	0.00	0.00
0.00	0.00	0.00	0.00	0.00	0.00	0.00	0.00	0.00
0.00	0.00	0.00	0.00	0.00	0.00	0.00	0.00	0.00
0.00	0.00	0.00	0.00	0.00	0.00	0.00	0.00	0.00
0.00	0.00	0.00	0.00	0.00	0.00	0.00	0.00	0.00
0.00	0.00	0.00	0.00	0.00	0.00	0.00	0.00	6.00
0.00	0.00	0.00	0.00	0.00	0.00	0.00	0.00	0.00
0.00	0.00	0.00	0.00	0.00	0.00	0.00	0.00	0.00
0.00	0.00	0.00	0.00	0.00	0.00	0.00	0.00	0.00
0.00	0.00	0.00	0.00	0.00	0.00	0.00	0.00	0.00
0.00	0.00	0.00	0.00	0.00	0.00	0.00	0.00	0.00
0.00	0.00	0.00	0.00	0.00	0.00	0.00	0.00	0.00
0.00	0.00	0.00	0.00	0.00	0.00	0.00	0.00	0.00
0.00	0.00	0.00	0.00	0.00	0.00	0.00	0.00	0.00
0.00	0.00	0.00	0.00	0.00	0.00	0.00	0.00	0.00
0.00	0.00	0.00	0.00	0.00	0.00	0.00	0.00	0.00
12.00	0.00	0.00	0.00	0.00	0.00	0.00	0.00	0.00
0.00	0.00	0.00	0.00	0.00	0.00	24.00	0.00	0.00
0.00	0.00	0.00	0.00	0.00	0.00	0.00	0.00	0.00
0.00	0.00	0.00	0.00	0.00	0.00	0.00	0.00	0.00
0.00	0.00	0.00	0.00	0.00	0.00	0.00	0.00	0.00
0.00	0.00	0.00	0.00	0.00	0.00	0.00	0.00	0.00
0.00	0.00	0.00	0.00	0.00	0.00	0.00	0.00	0.00
0.00	0.00	0.00	0.00	0.00	0.00	0.00	0.00	0.00
0.00	0.00	0.00	0.00	0.00	0.00	0.00	0.00	0.00
0.00	0.00	0.00	0.00	0.00	0.00	0.00	0.00	0.00
0.00	0.00	0.00	0.00	0.00	0.00	0.00	0.00	0.00
0.00	0.00	0.00	0.00	0.00	0.00	0.00	0.00	0.00
0.00	0.00	0.00	0.00	0.00	20.00	0.00	0.00	0.00
7.00	0.00	0.00	0.00	0.00	0.00	0.00	0.00	0.00
0.00	0.00	0.00	0.00	0.00	0.00	0.00	0.00	0.00
0.00	0.00	0.00	0.00	0.00	0.00	0.00	0.00	0.00

0.00	0.00	0.00	0.00	0.00	0.00	0.00	0.00	0.00
0.00	0.00	0.00	0.00	0.00	0.00	0.00	0.00	0.00
0.00	0.00	0.00	0.00	0.00	0.00	0.00	0.00	0.00
0.00	0.00	0.00	0.00	0.00	0.00	0.00	0.00	0.00
0.00	0.00	0.00	0.00	0.00	0.00	0.00	0.00	0.00
0.00	0.00	0.00	0.00	0.00	0.00	0.00	44.00	0.00
0.00	0.00	0.00	0.00	0.00	0.00	0.00	0.00	0.00
0.00	0.00	0.00	0.00	0.00	0.00	0.00	0.00	0.00
0.00	0.00	0.00	0.00	0.00	0.00	0.00	0.00	0.00
0.00	0.00	0.00	0.00	0.00	0.00	0.00	0.00	0.00
0.00	0.00	0.00	0.00	0.00	0.00	0.00	0.00	0.00
0.00	0.00	0.00	0.00	0.00	0.00	0.00	0.00	0.00
0.00	36.00	0.00	0.00	0.00	0.00	0.00	0.00	0.00
0.00	0.00	0.00	0.00	0.00	0.00	0.00	0.00	0.00
0.00	0.00	0.00	0.00	0.00	0.00	0.00	0.00	0.00
0.00	0.00	0.00	0.00	0.00	0.00	0.00	0.00	0.00
0.00	0.00	0.00	0.00	0.00	0.00	0.00	0.00	0.00
0.00	0.00	0.00	0.00	0.00	0.00	0.00	0.00	0.00
0.00	0.00	0.00	0.00	0.00	0.00	0.00	0.00	0.00
0.00	0.00	0.00	0.00	0.00	0.00	0.00	0.00	0.00
6.00	0.00	0.00	0.00	0.00	7.00	0.00	0.00	4.00
0.00	0.00	0.00	0.00	0.00	0.00	0.00	0.00	0.00
0.00	0.00	0.00	0.00	0.00	0.00	0.00	0.00	0.00
0.00	0.00	0.00	0.00	0.00	0.00	0.00	0.00	0.00
0.00	0.00	0.00	0.00	0.00	0.00	0.00	0.00	0.00
0.00	0.00	0.00	0.00	0.00	0.00	0.00	0.00	0.00
0.00	0.00	0.00	0.00	0.00	0.00	0.00	0.00	0.00
0.00	0.00	0.00	0.00	0.00	0.00	0.00	0.00	0.00
0.00	0.00	0.00	0.00	0.00	0.00	0.00	0.00	0.00
0.00	0.00	0.00	0.00	0.00	0.00	0.00	0.00	0.00
0.00	0.00	0.00	0.00	0.00	0.00	0.00	0.00	0.00
14.00	0.00	0.00	0.00	0.00	0.00	0.00	0.00	0.00
41.00	0.00	0.00	0.00	0.00	0.00	0.00	0.00	0.00
0.00	0.00	0.00	0.00	0.00	0.00	0.00	0.00	0.00
0.00	0.00	0.00	0.00	0.00	0.00	0.00	0.00	0.00
0.00	0.00	0.00	0.00	0.00	0.00	0.00	0.00	0.00
0.00	0.00	0.00	0.00	0.00	0.00	0.00	0.00	0.00

0.00	0.00	0.00	0.00	0.00	0.00	0.00	0.00	0.00
11.00	0.00	0.00	0.00	0.00	0.00	0.00	0.00	0.00
0.00	0.00	0.00	0.00	0.00	0.00	0.00	0.00	0.00
0.00	0.00	0.00	0.00	0.00	0.00	0.00	0.00	0.00
0.00	0.00	0.00	0.00	0.00	0.00	0.00	0.00	0.00
0.00	0.00	0.00	0.00	0.00	0.00	0.00	0.00	0.00
0.00	0.00	0.00	0.00	0.00	0.00	0.00	0.00	0.00
0.00	0.00	0.00	0.00	0.00	0.00	0.00	0.00	0.00
0.00	0.00	0.00	0.00	0.00	0.00	0.00	0.00	0.00
0.00	0.00	0.00	0.00	0.00	0.00	0.00	0.00	0.00
0.00	0.00	0.00	0.00	0.00	0.00	0.00	0.00	0.00
0.00	0.00	0.00	0.00	0.00	0.00	0.00	0.00	0.00
0.00	0.00	0.00	0.00	0.00	0.00	0.00	0.00	0.00
0.00	0.00	0.00	0.00	0.00	0.00	0.00	0.00	0.00
0.00	0.00	0.00	0.00	0.00	0.00	0.00	0.00	0.00
0.00	0.00	0.00	0.00	0.00	0.00	0.00	0.00	0.00
0.00	0.00	0.00	0.00	0.00	0.00	0.00	0.00	0.00
0.00	0.00	0.00	0.00	0.00	0.00	0.00	0.00	0.00
0.00	0.00	0.00	0.00	0.00	0.00	0.00	0.00	0.00
0.00	0.00	0.00	0.00	0.00	0.00	0.00	0.00	0.00
0.00	0.00	0.00	0.00	0.00	0.00	0.00	0.00	0.00
0.00	0.00	0.00	0.00	0.00	0.00	0.00	0.00	0.00
6.00	0.00	0.00	0.00	0.00	0.00	0.00	0.00	0.00
2.00	0.00	0.00	0.00	0.00	0.00	0.00	0.00	0.00
0.00	38.00	0.00	0.00	0.00	0.00	0.00	0.00	0.00
0.00	0.00	0.00	0.00	0.00	0.00	38.00	0.00	0.00
0.00	0.00	0.00	0.00	0.00	0.00	0.00	0.00	6.00
0.00	0.00	0.00	0.00	0.00	0.00	0.00	0.00	0.00
0.00	0.00	0.00	0.00	0.00	0.00	0.00	0.00	0.00
0.00	0.00	0.00	0.00	0.00	0.00	0.00	0.00	0.00
0.00	0.00	0.00	0.00	0.00	0.00	0.00	0.00	0.00
0.00	0.00	0.00	0.00	0.00	0.00	0.00	0.00	0.00
0.00	0.00	0.00	0.00	0.00	0.00	0.00	0.00	0.00
0.00	0.00	0.00	0.00	0.00	0.00	0.00	0.00	0.00
0.00	0.00	0.00	0.00	0.00	0.00	0.00	0.00	0.00
0.00	0.00	0.00	0.00	0.00	0.00	0.00	0.00	0.00
0.00	0.00	0.00	0.00	0.00	0.00	0.00	0.00	0.00

37.00	0.00	0.00	0.00	0.00	0.00	0.00	0.00	0.00
0.00	0.00	0.00	0.00	0.00	0.00	0.00	0.00	0.00
0.00	0.00	0.00	0.00	0.00	0.00	0.00	0.00	0.00
0.00	0.00	0.00	0.00	0.00	0.00	0.00	0.00	0.00
0.00	0.00	0.00	0.00	0.00	0.00	0.00	0.00	0.00
0.00	0.00	0.00	0.00	0.00	0.00	0.00	0.00	0.00
0.00	0.00	0.00	0.00	0.00	0.00	0.00	0.00	0.00
0.00	0.00	0.00	0.00	0.00	0.00	0.00	0.00	0.00
0.00	0.00	0.00	0.00	0.00	0.00	0.00	0.00	0.00
0.00	0.00	0.00	0.00	0.00	0.00	0.00	0.00	0.00
0.00	0.00	0.00	0.00	0.00	0.00	0.00	0.00	0.00
0.00	0.00	0.00	0.00	0.00	0.00	0.00	0.00	0.00
0.00	0.00	0.00	0.00	0.00	0.00	0.00	0.00	0.00
0.00	0.00	0.00	0.00	0.00	0.00	0.00	0.00	0.00
0.00	0.00	0.00	0.00	0.00	0.00	0.00	0.00	0.00
0.00	0.00	0.00	0.00	0.00	0.00	0.00	0.00	0.00
0.00	0.00	0.00	0.00	0.00	35.00	0.00	0.00	0.00
0.00	0.00	0.00	0.00	0.00	0.00	0.00	0.00	0.00
0.00	0.00	0.00	0.00	0.00	0.00	0.00	0.00	0.00
0.00	0.00	0.00	0.00	0.00	0.00	0.00	0.00	0.00
0.00	0.00	0.00	0.00	0.00	0.00	0.00	0.00	0.00
0.00	0.00	0.00	0.00	0.00	0.00	0.00	0.00	0.00
0.00	0.00	0.00	0.00	0.00	0.00	0.00	0.00	0.00
0.00	0.00	0.00	0.00	0.00	0.00	0.00	0.00	0.00
11.00	0.00	0.00	0.00	0.00	0.00	0.00	0.00	0.00
0.00	0.00	0.00	0.00	0.00	0.00	0.00	0.00	0.00
0.00	0.00	0.00	0.00	0.00	0.00	0.00	0.00	0.00
0.00	0.00	0.00	0.00	0.00	0.00	0.00	0.00	0.00
0.00	0.00	0.00	0.00	0.00	0.00	0.00	0.00	0.00
0.00	0.00	0.00	0.00	0.00	0.00	0.00	0.00	0.00
0.00	0.00	0.00	0.00	0.00	0.00	0.00	0.00	0.00
11.00	0.00	0.00	0.00	0.00	0.00	0.00	0.00	0.00
0.00	0.00	0.00	0.00	0.00	0.00	0.00	0.00	0.00
0.00	0.00	0.00	0.00	0.00	0.00	0.00	0.00	0.00
0.00	0.00	0.00	0.00	0.00	0.00	0.00	0.00	0.00
0.00	0.00	0.00	0.00	0.00	0.00	0.00	0.00	0.00
0.00	0.00	0.00	0.00	0.00	0.00	0.00	0.00	0.00

0.00	0.00	0.00	0.00	0.00	0.00	0.00	0.00	0.00
0.00	0.00	0.00	0.00	0.00	0.00	0.00	0.00	0.00
0.00	0.00	0.00	0.00	0.00	0.00	0.00	0.00	0.00
0.00	0.00	0.00	0.00	0.00	0.00	0.00	0.00	0.00
0.00	0.00	0.00	0.00	0.00	0.00	0.00	0.00	0.00
0.00	0.00	0.00	0.00	0.00	0.00	0.00	0.00	0.00
0.00	0.00	0.00	0.00	0.00	0.00	0.00	0.00	0.00
0.00	0.00	0.00	0.00	0.00	0.00	0.00	0.00	0.00
15.00	0.00	0.00	0.00	0.00	0.00	0.00	0.00	0.00
0.00	0.00	0.00	0.00	0.00	0.00	0.00	0.00	0.00
0.00	0.00	0.00	0.00	0.00	0.00	0.00	0.00	0.00
0.00	0.00	0.00	0.00	0.00	0.00	0.00	0.00	0.00
0.00	0.00	0.00	0.00	0.00	0.00	0.00	0.00	0.00
0.00	0.00	0.00	0.00	0.00	0.00	0.00	0.00	0.00
0.00	0.00	0.00	0.00	0.00	0.00	0.00	0.00	0.00
0.00	0.00	0.00	0.00	0.00	0.00	0.00	0.00	0.00
0.00	0.00	0.00	0.00	0.00	0.00	0.00	0.00	0.00
0.00	0.00	0.00	0.00	0.00	0.00	0.00	0.00	0.00
0.00	0.00	0.00	0.00	0.00	0.00	0.00	0.00	0.00
0.00	0.00	0.00	0.00	0.00	0.00	0.00	0.00	0.00
0.00	0.00	0.00	0.00	0.00	0.00	0.00	0.00	0.00
0.00	0.00	0.00	0.00	0.00	0.00	0.00	0.00	0.00
0.00	0.00	0.00	0.00	0.00	0.00	0.00	0.00	0.00
0.00	0.00	0.00	0.00	0.00	0.00	0.00	0.00	0.00
0.00	0.00	0.00	0.00	0.00	0.00	0.00	0.00	0.00
0.00	0.00	0.00	0.00	0.00	0.00	0.00	0.00	0.00
0.00	0.00	0.00	0.00	0.00	0.00	0.00	0.00	0.00
0.00	0.00	0.00	0.00	0.00	0.00	0.00	0.00	0.00
0.00	0.00	0.00	0.00	0.00	0.00	0.00	0.00	0.00
0.00	0.00	0.00	0.00	0.00	0.00	0.00	0.00	0.00
0.00	0.00	0.00	0.00	0.00	0.00	0.00	0.00	0.00
0.00	0.00	0.00	0.00	0.00	0.00	0.00	0.00	0.00
0.00	0.00	30.00	0.00	0.00	0.00	0.00	0.00	0.00
0.00	0.00	4.00	0.00	0.00	0.00	0.00	0.00	0.00
0.00	0.00	0.00	0.00	0.00	0.00	0.00	0.00	0.00
0.00	0.00	0.00	0.00	0.00	0.00	0.00	0.00	0.00
30.00	0.00	0.00	0.00	0.00	0.00	0.00	0.00	0.00

0.00	0.00	0.00	0.00	0.00	0.00	0.00	0.00	0.00
0.00	0.00	0.00	0.00	0.00	0.00	0.00	0.00	0.00
0.00	0.00	0.00	0.00	0.00	0.00	0.00	0.00	0.00
0.00	0.00	0.00	0.00	0.00	0.00	0.00	0.00	0.00
0.00	0.00	0.00	0.00	0.00	0.00	0.00	0.00	0.00
0.00	0.00	0.00	0.00	0.00	0.00	0.00	0.00	0.00
0.00	0.00	0.00	0.00	0.00	0.00	0.00	0.00	0.00
0.00	0.00	0.00	0.00	0.00	0.00	0.00	0.00	0.00
0.00	0.00	0.00	0.00	0.00	0.00	0.00	0.00	0.00
0.00	0.00	0.00	0.00	0.00	0.00	0.00	0.00	0.00
0.00	0.00	0.00	0.00	0.00	0.00	0.00	0.00	0.00
0.00	0.00	0.00	0.00	0.00	0.00	0.00	0.00	0.00
0.00	0.00	0.00	0.00	0.00	0.00	0.00	0.00	0.00
0.00	0.00	0.00	0.00	0.00	0.00	0.00	0.00	0.00
0.00	0.00	0.00	0.00	0.00	0.00	0.00	0.00	0.00
0.00	0.00	0.00	0.00	0.00	0.00	0.00	0.00	0.00
0.00	0.00	0.00	0.00	0.00	0.00	0.00	0.00	0.00
0.00	0.00	0.00	0.00	0.00	0.00	0.00	0.00	0.00
0.00	0.00	0.00	0.00	0.00	0.00	0.00	0.00	0.00
0.00	0.00	0.00	0.00	0.00	0.00	0.00	0.00	0.00
0.00	0.00	0.00	0.00	0.00	0.00	0.00	0.00	0.00
0.00	0.00	0.00	0.00	0.00	0.00	0.00	0.00	0.00
0.00	0.00	0.00	0.00	0.00	0.00	0.00	0.00	0.00
0.00	0.00	0.00	0.00	0.00	0.00	0.00	0.00	0.00
0.00	0.00	0.00	0.00	0.00	0.00	0.00	0.00	0.00
0.00	0.00	0.00	0.00	0.00	0.00	0.00	0.00	0.00
0.00	0.00	0.00	0.00	0.00	0.00	0.00	0.00	0.00
0.00	0.00	0.00	0.00	0.00	0.00	0.00	0.00	0.00
0.00	0.00	0.00	0.00	0.00	0.00	0.00	0.00	0.00
0.00	0.00	0.00	0.00	0.00	0.00	0.00	0.00	0.00
0.00	0.00	0.00	0.00	0.00	0.00	0.00	0.00	0.00
0.00	0.00	0.00	0.00	0.00	0.00	0.00	0.00	0.00
0.00	0.00	0.00	0.00	0.00	0.00	0.00	0.00	0.00
0.00	0.00	0.00	0.00	0.00	0.00	0.00	0.00	0.00
0.00	0.00	0.00	0.00	0.00	0.00	0.00	0.00	0.00
0.00	0.00	0.00	0.00	0.00	0.00	0.00	0.00	0.00
0.00	0.00	0.00	0.00	0.00	0.00	0.00	0.00	0.00

0.00	0.00	0.00	0.00	0.00	0.00	0.00	0.00	0.00
0.00	0.00	0.00	0.00	0.00	0.00	0.00	0.00	0.00
0.00	0.00	0.00	0.00	0.00	0.00	0.00	0.00	0.00
0.00	0.00	0.00	0.00	0.00	0.00	0.00	0.00	0.00
0.00	0.00	0.00	0.00	0.00	0.00	0.00	0.00	0.00
0.00	0.00	0.00	0.00	0.00	0.00	0.00	0.00	0.00
0.00	0.00	0.00	0.00	0.00	0.00	0.00	0.00	0.00
0.00	0.00	0.00	0.00	0.00	0.00	0.00	0.00	0.00
0.00	0.00	0.00	0.00	0.00	0.00	0.00	0.00	0.00
0.00	0.00	0.00	0.00	0.00	0.00	0.00	0.00	0.00
0.00	0.00	0.00	0.00	0.00	0.00	0.00	0.00	0.00
0.00	0.00	0.00	0.00	0.00	0.00	0.00	0.00	0.00
0.00	0.00	0.00	0.00	0.00	0.00	0.00	0.00	0.00
0.00	0.00	0.00	0.00	0.00	0.00	0.00	0.00	0.00
0.00	0.00	0.00	0.00	0.00	0.00	0.00	0.00	0.00
0.00	0.00	0.00	0.00	0.00	0.00	0.00	0.00	0.00
0.00	0.00	0.00	0.00	0.00	0.00	0.00	0.00	0.00
0.00	0.00	0.00	0.00	0.00	0.00	0.00	0.00	0.00
0.00	0.00	0.00	0.00	0.00	0.00	0.00	0.00	0.00
0.00	0.00	0.00	0.00	0.00	0.00	0.00	0.00	0.00
0.00	0.00	0.00	0.00	0.00	0.00	0.00	0.00	0.00
0.00	0.00	0.00	0.00	0.00	0.00	0.00	0.00	0.00
0.00	0.00	0.00	0.00	0.00	0.00	0.00	0.00	0.00
0.00	0.00	0.00	0.00	0.00	0.00	0.00	0.00	0.00
0.00	0.00	0.00	0.00	0.00	0.00	0.00	0.00	0.00
0.00	0.00	0.00	0.00	0.00	0.00	0.00	0.00	0.00
0.00	0.00	0.00	0.00	0.00	0.00	0.00	0.00	0.00
0.00	0.00	0.00	0.00	0.00	0.00	0.00	0.00	0.00
0.00	0.00	0.00	0.00	0.00	0.00	0.00	0.00	0.00
0.00	0.00	0.00	0.00	0.00	0.00	0.00	0.00	0.00
0.00	0.00	0.00	0.00	0.00	0.00	0.00	0.00	0.00
0.00	0.00	0.00	0.00	0.00	0.00	0.00	0.00	0.00
0.00	0.00	0.00	0.00	0.00	0.00	0.00	0.00	0.00
0.00	0.00	0.00	0.00	0.00	0.00	0.00	0.00	0.00
0.00	0.00	0.00	0.00	0.00	0.00	0.00	0.00	0.00
0.00	0.00	0.00	0.00	0.00	0.00	0.00	0.00	0.00
0.00	0.00	0.00	0.00	0.00	0.00	0.00	0.00	0.00

0.00	0.00	0.00	0.00	0.00	0.00	0.00	0.00	0.00
0.00	0.00	0.00	0.00	0.00	0.00	0.00	0.00	0.00
0.00	0.00	0.00	0.00	0.00	0.00	0.00	0.00	0.00
0.00	0.00	0.00	0.00	0.00	0.00	0.00	0.00	0.00
0.00	0.00	0.00	0.00	0.00	0.00	0.00	0.00	0.00
0.00	0.00	0.00	0.00	0.00	0.00	0.00	0.00	0.00
0.00	0.00	0.00	0.00	0.00	0.00	0.00	0.00	0.00
0.00	0.00	0.00	0.00	0.00	0.00	0.00	0.00	0.00
0.00	0.00	0.00	0.00	0.00	0.00	0.00	0.00	0.00
0.00	0.00	0.00	0.00	0.00	0.00	0.00	0.00	0.00
0.00	0.00	0.00	0.00	0.00	0.00	0.00	0.00	0.00
0.00	0.00	0.00	0.00	0.00	0.00	0.00	0.00	0.00
0.00	0.00	0.00	0.00	0.00	0.00	0.00	0.00	0.00
0.00	0.00	0.00	0.00	0.00	0.00	0.00	0.00	0.00
0.00	0.00	0.00	0.00	0.00	0.00	0.00	0.00	0.00
4.00	0.00	0.00	0.00	0.00	0.00	0.00	0.00	0.00
0.00	0.00	0.00	0.00	0.00	0.00	0.00	24.00	0.00
0.00	0.00	0.00	0.00	0.00	0.00	0.00	0.00	0.00
0.00	0.00	0.00	0.00	0.00	0.00	0.00	0.00	0.00
0.00	0.00	0.00	0.00	0.00	0.00	0.00	0.00	0.00
0.00	0.00	0.00	0.00	0.00	0.00	0.00	0.00	0.00
0.00	0.00	0.00	0.00	0.00	0.00	0.00	0.00	0.00
0.00	0.00	0.00	0.00	0.00	0.00	0.00	0.00	0.00
0.00	0.00	0.00	0.00	0.00	0.00	0.00	0.00	0.00
0.00	0.00	0.00	0.00	0.00	0.00	0.00	0.00	0.00
0.00	0.00	0.00	0.00	0.00	0.00	0.00	0.00	0.00
0.00	0.00	0.00	0.00	0.00	0.00	0.00	0.00	0.00
0.00	0.00	0.00	0.00	0.00	0.00	0.00	0.00	0.00
0.00	0.00	0.00	0.00	0.00	0.00	0.00	0.00	0.00
0.00	0.00	0.00	0.00	0.00	0.00	0.00	0.00	0.00
0.00	0.00	0.00	0.00	0.00	0.00	0.00	0.00	0.00
0.00	0.00	0.00	0.00	0.00	0.00	0.00	0.00	0.00
0.00	0.00	0.00	0.00	0.00	0.00	0.00	0.00	0.00
0.00	0.00	0.00	0.00	0.00	0.00	0.00	0.00	0.00
0.00	0.00	0.00	0.00	0.00	0.00	0.00	0.00	0.00
0.00	0.00	0.00	0.00	0.00	0.00	0.00	0.00	0.00
0.00	0.00	0.00	0.00	0.00	0.00	0.00	0.00	0.00

0.00	0.00	0.00	0.00	0.00	0.00	0.00	0.00	0.00
0.00	0.00	0.00	0.00	0.00	0.00	0.00	0.00	0.00
0.00	0.00	0.00	0.00	0.00	0.00	0.00	0.00	0.00
0.00	0.00	0.00	0.00	0.00	0.00	0.00	0.00	0.00
0.00	0.00	0.00	0.00	0.00	0.00	0.00	0.00	0.00
0.00	0.00	0.00	0.00	0.00	0.00	0.00	0.00	0.00
0.00	0.00	0.00	0.00	0.00	0.00	0.00	0.00	6.00
0.00	0.00	0.00	0.00	0.00	0.00	0.00	0.00	0.00
0.00	0.00	0.00	0.00	0.00	0.00	0.00	0.00	11.00
0.00	0.00	0.00	0.00	0.00	0.00	0.00	0.00	0.00
0.00	0.00	0.00	0.00	0.00	0.00	0.00	0.00	0.00
0.00	0.00	0.00	0.00	0.00	0.00	0.00	0.00	0.00
0.00	0.00	0.00	0.00	0.00	0.00	0.00	0.00	0.00
0.00	0.00	0.00	0.00	0.00	0.00	0.00	0.00	0.00
0.00	0.00	0.00	0.00	0.00	0.00	0.00	0.00	0.00
0.00	0.00	0.00	0.00	0.00	0.00	0.00	0.00	0.00
0.00	0.00	0.00	0.00	0.00	0.00	0.00	0.00	0.00
0.00	0.00	0.00	0.00	0.00	0.00	0.00	0.00	0.00
0.00	0.00	0.00	0.00	0.00	0.00	0.00	0.00	0.00
0.00	0.00	0.00	0.00	0.00	0.00	0.00	0.00	0.00
0.00	0.00	0.00	0.00	0.00	0.00	0.00	0.00	0.00
0.00	0.00	0.00	0.00	0.00	0.00	0.00	0.00	0.00
0.00	0.00	0.00	0.00	0.00	0.00	0.00	0.00	0.00
0.00	0.00	0.00	0.00	0.00	0.00	0.00	0.00	0.00
0.00	0.00	0.00	0.00	0.00	0.00	0.00	0.00	0.00
0.00	0.00	0.00	0.00	0.00	0.00	0.00	0.00	0.00
0.00	0.00	0.00	7.00	4.00	0.00	0.00	0.00	0.00
0.00	0.00	0.00	0.00	0.00	0.00	0.00	0.00	0.00
0.00	0.00	0.00	0.00	0.00	0.00	0.00	0.00	0.00
0.00	21.00	0.00	0.00	0.00	0.00	0.00	0.00	0.00
0.00	0.00	0.00	0.00	0.00	0.00	21.00	0.00	0.00
0.00	0.00	0.00	0.00	0.00	0.00	0.00	0.00	0.00
0.00	0.00	0.00	0.00	0.00	0.00	0.00	0.00	0.00
0.00	0.00	0.00	0.00	0.00	0.00	0.00	0.00	0.00
0.00	0.00	0.00	0.00	0.00	0.00	0.00	0.00	0.00
0.00	0.00	0.00	0.00	0.00	0.00	0.00	0.00	0.00
0.00	0.00	0.00	0.00	0.00	0.00	0.00	0.00	0.00

0.00	0.00	0.00	0.00	0.00	0.00	0.00	0.00	0.00
0.00	0.00	0.00	0.00	0.00	0.00	0.00	0.00	0.00
0.00	0.00	0.00	0.00	0.00	0.00	0.00	0.00	0.00
0.00	0.00	0.00	0.00	0.00	0.00	0.00	0.00	0.00
0.00	0.00	0.00	0.00	0.00	0.00	0.00	0.00	0.00
0.00	0.00	0.00	0.00	0.00	0.00	0.00	0.00	0.00
0.00	0.00	0.00	0.00	0.00	0.00	0.00	0.00	0.00
0.00	0.00	0.00	0.00	0.00	0.00	0.00	0.00	0.00
0.00	0.00	0.00	0.00	0.00	0.00	0.00	0.00	0.00
0.00	0.00	0.00	0.00	0.00	0.00	0.00	0.00	0.00
0.00	0.00	0.00	0.00	0.00	0.00	0.00	0.00	0.00
0.00	0.00	0.00	0.00	0.00	0.00	0.00	0.00	0.00
0.00	0.00	0.00	0.00	0.00	0.00	0.00	0.00	0.00
0.00	0.00	0.00	0.00	0.00	0.00	0.00	0.00	0.00
0.00	0.00	0.00	0.00	0.00	0.00	0.00	0.00	0.00
0.00	0.00	0.00	0.00	0.00	0.00	0.00	0.00	0.00
0.00	0.00	0.00	0.00	0.00	0.00	0.00	0.00	0.00
0.00	0.00	0.00	0.00	0.00	0.00	0.00	0.00	0.00
0.00	0.00	0.00	0.00	0.00	0.00	0.00	0.00	0.00
0.00	0.00	0.00	0.00	0.00	0.00	0.00	0.00	0.00
0.00	0.00	0.00	0.00	0.00	0.00	0.00	0.00	0.00
0.00	0.00	0.00	0.00	0.00	0.00	0.00	0.00	0.00
0.00	0.00	0.00	0.00	0.00	0.00	0.00	0.00	0.00
0.00	0.00	0.00	0.00	0.00	0.00	0.00	0.00	0.00
0.00	0.00	0.00	0.00	0.00	0.00	0.00	0.00	0.00
0.00	0.00	0.00	0.00	0.00	0.00	0.00	0.00	0.00
0.00	0.00	0.00	0.00	0.00	0.00	0.00	0.00	0.00
0.00	0.00	0.00	0.00	0.00	0.00	0.00	0.00	0.00
0.00	0.00	0.00	0.00	0.00	0.00	0.00	0.00	0.00
0.00	0.00	0.00	0.00	0.00	0.00	0.00	0.00	0.00
0.00	0.00	0.00	0.00	0.00	0.00	0.00	0.00	0.00
0.00	0.00	0.00	0.00	0.00	0.00	0.00	0.00	0.00
0.00	0.00	0.00	0.00	0.00	0.00	0.00	0.00	0.00
0.00	0.00	0.00	0.00	0.00	0.00	0.00	0.00	0.00
0.00	0.00	0.00	0.00	0.00	0.00	0.00	0.00	0.00
0.00	0.00	0.00	0.00	0.00	0.00	0.00	0.00	0.00
0.00	0.00	0.00	0.00	0.00	0.00	0.00	0.00	0.00

0.00	0.00	0.00	0.00	0.00	0.00	0.00	0.00	0.00
0.00	0.00	0.00	0.00	0.00	0.00	0.00	0.00	0.00
0.00	0.00	0.00	0.00	0.00	0.00	0.00	0.00	0.00
0.00	0.00	0.00	0.00	0.00	0.00	0.00	0.00	0.00
0.00	0.00	0.00	0.00	0.00	0.00	0.00	0.00	0.00
0.00	0.00	0.00	0.00	0.00	0.00	0.00	0.00	0.00
0.00	0.00	0.00	0.00	0.00	0.00	0.00	0.00	0.00
0.00	0.00	0.00	0.00	0.00	0.00	0.00	0.00	0.00
0.00	0.00	0.00	0.00	0.00	0.00	0.00	0.00	0.00
0.00	0.00	0.00	0.00	0.00	0.00	0.00	0.00	0.00
0.00	0.00	0.00	0.00	0.00	0.00	0.00	0.00	0.00
0.00	0.00	0.00	0.00	0.00	0.00	0.00	0.00	0.00
0.00	0.00	0.00	0.00	0.00	0.00	0.00	0.00	0.00
0.00	0.00	0.00	0.00	0.00	0.00	0.00	0.00	0.00
0.00	0.00	0.00	0.00	0.00	0.00	0.00	0.00	0.00
0.00	0.00	0.00	0.00	0.00	0.00	0.00	0.00	0.00
0.00	0.00	0.00	0.00	0.00	0.00	0.00	0.00	0.00
0.00	0.00	0.00	0.00	0.00	0.00	0.00	0.00	0.00
0.00	0.00	0.00	0.00	0.00	0.00	0.00	0.00	0.00
0.00	0.00	0.00	0.00	18.00	0.00	0.00	0.00	0.00
0.00	0.00	0.00	0.00	0.00	0.00	0.00	0.00	0.00
0.00	0.00	0.00	0.00	0.00	0.00	0.00	0.00	0.00
0.00	0.00	0.00	0.00	0.00	0.00	0.00	0.00	0.00
0.00	0.00	0.00	0.00	0.00	0.00	0.00	0.00	0.00
0.00	0.00	0.00	0.00	0.00	0.00	0.00	0.00	0.00
0.00	0.00	0.00	0.00	0.00	0.00	0.00	0.00	0.00
18.00	0.00	0.00	0.00	0.00	0.00	0.00	0.00	0.00
0.00	18.00	0.00	0.00	0.00	0.00	0.00	0.00	0.00
0.00	0.00	0.00	0.00	0.00	0.00	0.00	0.00	0.00
0.00	0.00	0.00	0.00	0.00	0.00	18.00	0.00	0.00
0.00	0.00	0.00	0.00	0.00	0.00	0.00	0.00	10.00
0.00	0.00	0.00	0.00	0.00	0.00	0.00	0.00	0.00
0.00	0.00	0.00	0.00	0.00	0.00	0.00	0.00	0.00
0.00	0.00	0.00	0.00	0.00	0.00	0.00	0.00	0.00
0.00	0.00	0.00	18.00	0.00	0.00	0.00	0.00	0.00
0.00	0.00	0.00	18.00	0.00	0.00	0.00	0.00	0.00
0.00	0.00	0.00	0.00	0.00	0.00	0.00	0.00	0.00

0.00	0.00	0.00	0.00	0.00	0.00	0.00	0.00	0.00
0.00	0.00	0.00	0.00	0.00	0.00	0.00	0.00	0.00
0.00	0.00	0.00	0.00	0.00	0.00	0.00	0.00	0.00
0.00	0.00	0.00	0.00	0.00	0.00	0.00	0.00	0.00
0.00	0.00	0.00	0.00	0.00	0.00	0.00	0.00	0.00
0.00	0.00	0.00	0.00	0.00	0.00	0.00	0.00	0.00
0.00	0.00	0.00	0.00	0.00	0.00	0.00	0.00	0.00
0.00	0.00	0.00	0.00	0.00	0.00	0.00	0.00	0.00
0.00	0.00	0.00	0.00	0.00	0.00	0.00	0.00	0.00
0.00	0.00	0.00	0.00	0.00	0.00	0.00	0.00	0.00
0.00	0.00	0.00	0.00	0.00	0.00	0.00	0.00	0.00
0.00	0.00	0.00	0.00	0.00	0.00	0.00	0.00	0.00
0.00	0.00	0.00	0.00	0.00	0.00	0.00	0.00	0.00
2.00	0.00	0.00	0.00	0.00	0.00	0.00	0.00	0.00
0.00	0.00	0.00	0.00	17.00	0.00	0.00	0.00	0.00
0.00	0.00	0.00	0.00	0.00	0.00	0.00	0.00	0.00
0.00	0.00	0.00	0.00	0.00	0.00	0.00	0.00	0.00
0.00	0.00	0.00	0.00	0.00	0.00	0.00	0.00	0.00
0.00	0.00	0.00	0.00	0.00	0.00	0.00	0.00	0.00
0.00	0.00	0.00	0.00	0.00	0.00	0.00	0.00	4.00
8.00	0.00	0.00	0.00	0.00	0.00	0.00	0.00	9.00
0.00	0.00	0.00	0.00	0.00	0.00	0.00	0.00	0.00
0.00	0.00	0.00	0.00	0.00	0.00	0.00	0.00	0.00
0.00	0.00	0.00	0.00	0.00	0.00	0.00	0.00	0.00
0.00	0.00	0.00	0.00	0.00	0.00	0.00	0.00	0.00
0.00	0.00	0.00	0.00	0.00	0.00	0.00	0.00	0.00
0.00	0.00	0.00	0.00	0.00	0.00	0.00	0.00	0.00
0.00	0.00	0.00	0.00	0.00	0.00	0.00	0.00	0.00
0.00	0.00	0.00	0.00	0.00	0.00	0.00	0.00	0.00
0.00	0.00	0.00	0.00	0.00	0.00	0.00	0.00	0.00
0.00	0.00	0.00	0.00	0.00	0.00	0.00	0.00	0.00
0.00	0.00	0.00	0.00	0.00	0.00	0.00	0.00	0.00
0.00	0.00	0.00	0.00	0.00	0.00	0.00	0.00	0.00
0.00	0.00	0.00	0.00	0.00	0.00	0.00	0.00	0.00
0.00	0.00	0.00	0.00	0.00	0.00	0.00	0.00	0.00
0.00	0.00	0.00	0.00	0.00	0.00	0.00	0.00	0.00
0.00	0.00	0.00	0.00	0.00	0.00	0.00	0.00	0.00

0.00	0.00	0.00	0.00	0.00	0.00	0.00	0.00	0.00
0.00	0.00	0.00	0.00	0.00	0.00	0.00	0.00	0.00
0.00	0.00	0.00	0.00	0.00	0.00	0.00	0.00	0.00
0.00	0.00	0.00	0.00	0.00	0.00	0.00	0.00	0.00
0.00	0.00	0.00	0.00	0.00	0.00	0.00	0.00	0.00
0.00	0.00	0.00	0.00	0.00	0.00	0.00	0.00	0.00
0.00	0.00	0.00	0.00	0.00	0.00	0.00	0.00	0.00
0.00	0.00	0.00	0.00	0.00	0.00	0.00	0.00	0.00
0.00	0.00	0.00	0.00	0.00	0.00	0.00	0.00	0.00
0.00	0.00	0.00	0.00	0.00	0.00	0.00	0.00	0.00
0.00	0.00	0.00	0.00	0.00	0.00	0.00	0.00	0.00
0.00	0.00	0.00	0.00	0.00	0.00	0.00	0.00	0.00
0.00	0.00	0.00	0.00	0.00	0.00	0.00	0.00	0.00
0.00	0.00	0.00	0.00	0.00	0.00	0.00	0.00	0.00
0.00	0.00	0.00	0.00	0.00	0.00	0.00	0.00	0.00
0.00	0.00	0.00	0.00	0.00	0.00	0.00	0.00	0.00
0.00	0.00	0.00	0.00	0.00	0.00	0.00	0.00	0.00
0.00	0.00	0.00	0.00	0.00	0.00	0.00	0.00	0.00
0.00	0.00	0.00	0.00	0.00	0.00	0.00	0.00	0.00
0.00	0.00	0.00	0.00	0.00	0.00	0.00	0.00	0.00
0.00	0.00	0.00	0.00	0.00	0.00	0.00	0.00	0.00
0.00	0.00	0.00	0.00	0.00	0.00	0.00	0.00	0.00
0.00	0.00	0.00	0.00	0.00	0.00	0.00	0.00	0.00
0.00	0.00	0.00	0.00	0.00	0.00	0.00	0.00	0.00
0.00	0.00	0.00	0.00	0.00	0.00	0.00	0.00	0.00
0.00	0.00	0.00	0.00	0.00	0.00	0.00	0.00	0.00
0.00	0.00	0.00	0.00	0.00	0.00	0.00	0.00	0.00
0.00	0.00	0.00	0.00	0.00	0.00	0.00	0.00	0.00
0.00	0.00	0.00	0.00	0.00	0.00	0.00	0.00	0.00
0.00	0.00	0.00	0.00	0.00	0.00	0.00	0.00	0.00
0.00	0.00	0.00	0.00	0.00	0.00	0.00	0.00	0.00
0.00	0.00	0.00	0.00	0.00	0.00	0.00	0.00	0.00
0.00	0.00	0.00	0.00	0.00	0.00	0.00	0.00	0.00
0.00	0.00	0.00	0.00	0.00	0.00	0.00	0.00	0.00
0.00	0.00	0.00	0.00	0.00	0.00	0.00	0.00	0.00
0.00	0.00	0.00	0.00	0.00	0.00	0.00	0.00	0.00
0.00	0.00	0.00	0.00	0.00	0.00	0.00	0.00	0.00

0.00	0.00	0.00	0.00	0.00	0.00	0.00	0.00	0.00
0.00	0.00	0.00	0.00	0.00	0.00	0.00	0.00	0.00
0.00	0.00	0.00	0.00	0.00	0.00	0.00	0.00	0.00
0.00	0.00	0.00	0.00	0.00	0.00	0.00	0.00	0.00
0.00	0.00	0.00	0.00	0.00	0.00	0.00	0.00	0.00
0.00	4.00	0.00	0.00	0.00	0.00	0.00	0.00	0.00
0.00	0.00	0.00	0.00	0.00	0.00	0.00	0.00	0.00
0.00	0.00	0.00	0.00	0.00	0.00	0.00	0.00	0.00
0.00	0.00	0.00	0.00	0.00	0.00	0.00	0.00	0.00
0.00	0.00	0.00	0.00	0.00	0.00	0.00	0.00	0.00
0.00	0.00	0.00	0.00	0.00	0.00	0.00	0.00	0.00
0.00	0.00	0.00	0.00	0.00	0.00	0.00	0.00	0.00
3.00	0.00	0.00	0.00	0.00	0.00	0.00	0.00	0.00
0.00	0.00	0.00	0.00	0.00	0.00	0.00	0.00	0.00
0.00	0.00	0.00	0.00	0.00	0.00	0.00	0.00	0.00
0.00	0.00	0.00	0.00	0.00	0.00	0.00	0.00	0.00
0.00	0.00	0.00	0.00	0.00	0.00	0.00	0.00	0.00
0.00	0.00	0.00	0.00	0.00	0.00	0.00	0.00	0.00
0.00	0.00	0.00	0.00	0.00	0.00	0.00	0.00	0.00
0.00	0.00	0.00	0.00	0.00	0.00	0.00	0.00	0.00
0.00	0.00	0.00	0.00	0.00	0.00	0.00	0.00	0.00
0.00	0.00	0.00	0.00	0.00	0.00	0.00	0.00	0.00
0.00	0.00	0.00	0.00	0.00	0.00	0.00	0.00	0.00
0.00	0.00	0.00	0.00	0.00	0.00	0.00	0.00	0.00
0.00	0.00	0.00	0.00	0.00	0.00	0.00	0.00	0.00
0.00	0.00	0.00	0.00	0.00	0.00	0.00	0.00	0.00
0.00	0.00	0.00	0.00	0.00	0.00	0.00	0.00	0.00
0.00	0.00	0.00	0.00	0.00	0.00	0.00	0.00	0.00
0.00	0.00	0.00	0.00	0.00	0.00	0.00	0.00	0.00
0.00	0.00	0.00	0.00	0.00	0.00	0.00	0.00	0.00
0.00	0.00	0.00	8.00	0.00	0.00	0.00	0.00	0.00
0.00	0.00	14.00	0.00	0.00	0.00	0.00	0.00	0.00
0.00	0.00	0.00	0.00	0.00	0.00	0.00	0.00	0.00
0.00	0.00	0.00	0.00	0.00	0.00	0.00	0.00	0.00
0.00	0.00	0.00	0.00	0.00	0.00	0.00	0.00	0.00
0.00	0.00	0.00	0.00	0.00	0.00	0.00	0.00	0.00
0.00	6.00	0.00	0.00	0.00	0.00	0.00	0.00	0.00

0.00	0.00	0.00	0.00	0.00	0.00	0.00	0.00	0.00
0.00	0.00	0.00	0.00	0.00	0.00	0.00	0.00	0.00
0.00	0.00	0.00	0.00	0.00	0.00	0.00	0.00	0.00
0.00	0.00	0.00	0.00	0.00	0.00	0.00	0.00	0.00
0.00	0.00	0.00	0.00	0.00	0.00	0.00	0.00	0.00
0.00	0.00	0.00	0.00	0.00	0.00	0.00	0.00	0.00
0.00	0.00	0.00	0.00	0.00	0.00	0.00	0.00	0.00
0.00	0.00	0.00	0.00	0.00	0.00	0.00	0.00	0.00
0.00	0.00	0.00	0.00	0.00	0.00	0.00	0.00	0.00
0.00	0.00	0.00	0.00	0.00	0.00	0.00	0.00	0.00
0.00	0.00	0.00	0.00	0.00	0.00	0.00	0.00	0.00
0.00	0.00	0.00	0.00	0.00	0.00	0.00	0.00	0.00
0.00	0.00	0.00	0.00	0.00	0.00	0.00	0.00	0.00
0.00	0.00	0.00	0.00	0.00	0.00	0.00	0.00	0.00
0.00	0.00	0.00	0.00	0.00	0.00	0.00	0.00	0.00
0.00	0.00	0.00	0.00	0.00	0.00	0.00	0.00	0.00
0.00	0.00	0.00	0.00	0.00	0.00	0.00	0.00	0.00
0.00	0.00	0.00	0.00	0.00	0.00	0.00	0.00	0.00
0.00	0.00	0.00	0.00	13.00	0.00	0.00	0.00	0.00
0.00	0.00	0.00	0.00	0.00	0.00	0.00	0.00	0.00
0.00	0.00	0.00	0.00	0.00	0.00	0.00	0.00	0.00
0.00	0.00	0.00	0.00	0.00	0.00	0.00	0.00	0.00
0.00	0.00	0.00	0.00	0.00	0.00	0.00	0.00	0.00
0.00	0.00	0.00	0.00	0.00	0.00	0.00	0.00	0.00
5.00	0.00	0.00	0.00	0.00	0.00	0.00	0.00	0.00
0.00	0.00	0.00	0.00	0.00	0.00	0.00	0.00	5.00
0.00	0.00	0.00	0.00	0.00	0.00	0.00	0.00	0.00
0.00	0.00	0.00	0.00	0.00	0.00	0.00	0.00	0.00
0.00	0.00	0.00	0.00	0.00	0.00	0.00	0.00	0.00
0.00	0.00	0.00	0.00	0.00	0.00	0.00	0.00	0.00
0.00	0.00	0.00	0.00	0.00	0.00	0.00	0.00	0.00
0.00	0.00	0.00	0.00	0.00	0.00	0.00	0.00	0.00
0.00	0.00	0.00	0.00	0.00	0.00	0.00	0.00	0.00
0.00	0.00	0.00	0.00	0.00	0.00	0.00	0.00	0.00
0.00	0.00	0.00	0.00	0.00	0.00	0.00	0.00	0.00
0.00	0.00	0.00	0.00	0.00	0.00	0.00	0.00	0.00
0.00	0.00	0.00	0.00	0.00	0.00	0.00	0.00	0.00

0.00	0.00	0.00	0.00	0.00	0.00	0.00	0.00	0.00
0.00	0.00	0.00	0.00	0.00	0.00	0.00	0.00	0.00
0.00	0.00	0.00	0.00	0.00	0.00	0.00	0.00	0.00
0.00	0.00	0.00	0.00	0.00	0.00	0.00	0.00	0.00
0.00	0.00	0.00	0.00	0.00	0.00	0.00	0.00	0.00
0.00	0.00	0.00	0.00	0.00	0.00	0.00	0.00	0.00
0.00	0.00	0.00	0.00	0.00	0.00	0.00	0.00	0.00
0.00	0.00	0.00	0.00	0.00	0.00	0.00	0.00	0.00
0.00	0.00	0.00	0.00	0.00	0.00	0.00	0.00	0.00
0.00	0.00	0.00	0.00	0.00	0.00	0.00	0.00	0.00
0.00	0.00	0.00	0.00	0.00	0.00	0.00	0.00	0.00
0.00	0.00	0.00	0.00	0.00	0.00	0.00	0.00	0.00
0.00	0.00	0.00	0.00	0.00	0.00	0.00	0.00	0.00
0.00	0.00	0.00	0.00	0.00	0.00	0.00	0.00	0.00
0.00	0.00	0.00	0.00	0.00	0.00	0.00	0.00	0.00
0.00	0.00	0.00	0.00	0.00	0.00	0.00	0.00	0.00
0.00	0.00	0.00	0.00	0.00	0.00	0.00	0.00	0.00
0.00	0.00	12.00	0.00	0.00	0.00	0.00	0.00	0.00
0.00	0.00	0.00	0.00	0.00	0.00	0.00	0.00	0.00
0.00	0.00	0.00	0.00	0.00	0.00	0.00	0.00	0.00
0.00	0.00	0.00	0.00	0.00	0.00	0.00	0.00	0.00
0.00	0.00	0.00	0.00	0.00	0.00	0.00	0.00	0.00
0.00	0.00	0.00	0.00	0.00	0.00	0.00	0.00	0.00
3.00	0.00	0.00	0.00	0.00	0.00	0.00	0.00	0.00
0.00	0.00	0.00	0.00	0.00	0.00	0.00	0.00	0.00
0.00	0.00	0.00	0.00	0.00	0.00	0.00	0.00	0.00
0.00	0.00	0.00	0.00	0.00	0.00	0.00	0.00	0.00
0.00	0.00	0.00	0.00	0.00	0.00	0.00	0.00	0.00
0.00	0.00	0.00	0.00	0.00	0.00	0.00	0.00	0.00
0.00	0.00	0.00	0.00	0.00	0.00	0.00	0.00	0.00
0.00	0.00	0.00	0.00	0.00	0.00	12.00	0.00	0.00
0.00	0.00	0.00	0.00	0.00	0.00	0.00	0.00	0.00
0.00	0.00	0.00	0.00	0.00	0.00	0.00	0.00	0.00
0.00	0.00	0.00	0.00	0.00	0.00	0.00	0.00	0.00
0.00	0.00	0.00	0.00	0.00	0.00	0.00	0.00	0.00
0.00	0.00	0.00	0.00	0.00	0.00	0.00	0.00	0.00
0.00	0.00	0.00	0.00	0.00	0.00	0.00	0.00	0.00

0.00	0.00	0.00	0.00	0.00	0.00	0.00	0.00	0.00
0.00	0.00	0.00	0.00	0.00	0.00	0.00	0.00	0.00
0.00	0.00	0.00	0.00	0.00	0.00	0.00	0.00	0.00
0.00	0.00	0.00	0.00	0.00	0.00	0.00	0.00	0.00
0.00	0.00	0.00	0.00	0.00	0.00	0.00	0.00	0.00
0.00	0.00	0.00	0.00	0.00	0.00	0.00	0.00	0.00
0.00	0.00	0.00	0.00	0.00	0.00	0.00	0.00	0.00
0.00	0.00	0.00	0.00	0.00	0.00	0.00	0.00	0.00
0.00	0.00	0.00	0.00	0.00	0.00	0.00	0.00	0.00
0.00	0.00	0.00	0.00	0.00	0.00	0.00	0.00	0.00
0.00	0.00	0.00	0.00	0.00	0.00	0.00	0.00	0.00
0.00	0.00	0.00	0.00	0.00	0.00	0.00	0.00	0.00
0.00	0.00	0.00	0.00	0.00	0.00	0.00	0.00	0.00
0.00	0.00	0.00	0.00	0.00	0.00	0.00	0.00	0.00
0.00	0.00	0.00	0.00	0.00	0.00	0.00	0.00	0.00
0.00	0.00	0.00	0.00	0.00	0.00	0.00	0.00	0.00
0.00	0.00	0.00	0.00	0.00	0.00	0.00	0.00	0.00
0.00	0.00	0.00	0.00	0.00	0.00	0.00	0.00	0.00
0.00	0.00	0.00	0.00	0.00	0.00	0.00	0.00	0.00
0.00	0.00	0.00	0.00	0.00	0.00	0.00	0.00	0.00
0.00	0.00	0.00	0.00	0.00	0.00	0.00	0.00	0.00
0.00	0.00	0.00	0.00	0.00	0.00	0.00	0.00	0.00
0.00	0.00	0.00	0.00	0.00	0.00	0.00	0.00	0.00
0.00	0.00	0.00	0.00	0.00	0.00	0.00	0.00	0.00
0.00	0.00	0.00	0.00	0.00	0.00	0.00	0.00	0.00
0.00	0.00	0.00	0.00	0.00	0.00	0.00	0.00	0.00
0.00	0.00	0.00	0.00	0.00	0.00	0.00	0.00	0.00
0.00	0.00	0.00	0.00	0.00	0.00	0.00	0.00	0.00
0.00	0.00	0.00	0.00	0.00	0.00	0.00	0.00	0.00
0.00	0.00	0.00	0.00	0.00	0.00	0.00	0.00	0.00
0.00	0.00	0.00	0.00	0.00	0.00	0.00	0.00	0.00
0.00	0.00	0.00	0.00	0.00	0.00	0.00	0.00	0.00
0.00	0.00	0.00	0.00	0.00	0.00	0.00	0.00	0.00
0.00	0.00	0.00	0.00	0.00	0.00	0.00	0.00	0.00
0.00	0.00	0.00	0.00	0.00	0.00	0.00	0.00	0.00
0.00	0.00	0.00	0.00	0.00	0.00	0.00	0.00	0.00
0.00	0.00	0.00	0.00	0.00	0.00	0.00	0.00	0.00

0.00	0.00	0.00	0.00	0.00	0.00	0.00	0.00	0.00
0.00	0.00	0.00	0.00	0.00	0.00	0.00	0.00	0.00
0.00	0.00	0.00	0.00	0.00	0.00	0.00	0.00	0.00
0.00	0.00	0.00	0.00	0.00	0.00	0.00	0.00	0.00
0.00	0.00	0.00	0.00	0.00	0.00	0.00	0.00	0.00
0.00	0.00	0.00	0.00	0.00	0.00	0.00	0.00	0.00
0.00	0.00	0.00	0.00	0.00	0.00	0.00	0.00	0.00
0.00	0.00	0.00	0.00	0.00	0.00	0.00	0.00	0.00
0.00	0.00	0.00	0.00	0.00	0.00	0.00	0.00	0.00
0.00	0.00	0.00	0.00	0.00	0.00	0.00	0.00	0.00
0.00	0.00	0.00	0.00	0.00	0.00	0.00	0.00	0.00
0.00	0.00	0.00	0.00	0.00	0.00	0.00	0.00	0.00
0.00	0.00	0.00	0.00	0.00	0.00	0.00	0.00	0.00
0.00	0.00	0.00	0.00	0.00	0.00	0.00	0.00	0.00
0.00	0.00	0.00	0.00	0.00	0.00	0.00	0.00	0.00
0.00	0.00	0.00	0.00	0.00	0.00	0.00	0.00	0.00
0.00	0.00	0.00	0.00	0.00	0.00	0.00	0.00	0.00
0.00	0.00	0.00	0.00	0.00	0.00	0.00	0.00	0.00
0.00	0.00	0.00	0.00	0.00	0.00	0.00	0.00	0.00
0.00	0.00	0.00	0.00	0.00	0.00	0.00	0.00	0.00
0.00	0.00	0.00	0.00	0.00	0.00	0.00	0.00	0.00
0.00	0.00	0.00	0.00	0.00	0.00	0.00	0.00	0.00
0.00	0.00	0.00	0.00	0.00	0.00	0.00	0.00	0.00
0.00	0.00	0.00	0.00	0.00	0.00	0.00	0.00	0.00
0.00	0.00	0.00	0.00	0.00	0.00	0.00	0.00	0.00
0.00	0.00	0.00	0.00	0.00	0.00	0.00	0.00	0.00
0.00	0.00	0.00	0.00	0.00	0.00	0.00	0.00	0.00
0.00	0.00	0.00	0.00	0.00	0.00	0.00	0.00	0.00
0.00	0.00	0.00	0.00	0.00	0.00	0.00	0.00	0.00
0.00	0.00	0.00	0.00	10.00	0.00	0.00	0.00	0.00
0.00	0.00	0.00	0.00	0.00	10.00	0.00	0.00	0.00
0.00	0.00	0.00	0.00	0.00	0.00	0.00	0.00	0.00
0.00	0.00	0.00	0.00	0.00	0.00	0.00	0.00	0.00
0.00	0.00	0.00	0.00	0.00	0.00	0.00	0.00	0.00
0.00	0.00	0.00	0.00	0.00	0.00	0.00	0.00	0.00
0.00	0.00	0.00	0.00	0.00	0.00	0.00	0.00	0.00
5.00	0.00	0.00	0.00	0.00	0.00	0.00	0.00	0.00

0.00	0.00	0.00	0.00	0.00	0.00	0.00	0.00	0.00
0.00	0.00	0.00	0.00	0.00	0.00	0.00	0.00	0.00
0.00	0.00	0.00	0.00	0.00	0.00	0.00	0.00	0.00
0.00	0.00	0.00	0.00	0.00	0.00	0.00	0.00	0.00
0.00	0.00	0.00	0.00	0.00	0.00	0.00	0.00	0.00
0.00	0.00	0.00	0.00	0.00	0.00	0.00	0.00	0.00
0.00	0.00	0.00	0.00	0.00	0.00	0.00	0.00	0.00
0.00	0.00	0.00	0.00	0.00	0.00	0.00	0.00	0.00
0.00	0.00	0.00	0.00	0.00	0.00	0.00	0.00	0.00
0.00	0.00	0.00	0.00	0.00	0.00	0.00	0.00	0.00
0.00	0.00	0.00	0.00	0.00	0.00	0.00	0.00	0.00
0.00	0.00	0.00	0.00	0.00	0.00	0.00	0.00	0.00
0.00	0.00	0.00	0.00	0.00	0.00	0.00	0.00	0.00
0.00	0.00	0.00	0.00	0.00	0.00	0.00	0.00	0.00
0.00	0.00	0.00	0.00	0.00	0.00	0.00	0.00	0.00
0.00	0.00	0.00	0.00	0.00	0.00	0.00	0.00	0.00
0.00	0.00	0.00	0.00	0.00	0.00	0.00	0.00	0.00
0.00	0.00	0.00	0.00	0.00	0.00	0.00	0.00	0.00
0.00	0.00	0.00	0.00	0.00	0.00	0.00	0.00	0.00
0.00	0.00	0.00	0.00	0.00	0.00	0.00	0.00	0.00
0.00	0.00	0.00	0.00	0.00	0.00	0.00	0.00	0.00
0.00	0.00	0.00	0.00	0.00	0.00	0.00	0.00	0.00
0.00	0.00	0.00	0.00	0.00	0.00	0.00	0.00	0.00
0.00	0.00	0.00	0.00	0.00	0.00	0.00	0.00	0.00
0.00	0.00	0.00	0.00	0.00	0.00	0.00	0.00	0.00
0.00	0.00	0.00	0.00	0.00	0.00	0.00	0.00	0.00
0.00	0.00	0.00	0.00	0.00	0.00	0.00	0.00	0.00
0.00	0.00	0.00	0.00	0.00	0.00	0.00	0.00	0.00
0.00	0.00	0.00	0.00	0.00	0.00	0.00	0.00	0.00
0.00	0.00	0.00	0.00	0.00	0.00	0.00	0.00	0.00
0.00	0.00	0.00	0.00	0.00	0.00	0.00	0.00	0.00
0.00	0.00	0.00	0.00	0.00	0.00	0.00	0.00	0.00
0.00	0.00	0.00	0.00	0.00	0.00	0.00	0.00	0.00
0.00	0.00	0.00	0.00	0.00	0.00	0.00	0.00	0.00
0.00	0.00	0.00	0.00	0.00	0.00	0.00	0.00	0.00
0.00	0.00	0.00	0.00	0.00	0.00	0.00	0.00	0.00
0.00	0.00	0.00	0.00	0.00	0.00	0.00	0.00	0.00

0.00	0.00	0.00	0.00	0.00	0.00	0.00	0.00	0.00
0.00	0.00	0.00	0.00	0.00	0.00	0.00	0.00	0.00
0.00	0.00	0.00	0.00	0.00	0.00	0.00	0.00	0.00
0.00	0.00	0.00	0.00	0.00	0.00	0.00	0.00	0.00
0.00	0.00	0.00	0.00	0.00	0.00	0.00	0.00	0.00
0.00	0.00	0.00	0.00	0.00	0.00	0.00	0.00	0.00
0.00	0.00	0.00	0.00	0.00	0.00	0.00	0.00	0.00
0.00	0.00	0.00	0.00	0.00	0.00	0.00	0.00	0.00
0.00	0.00	0.00	0.00	0.00	9.00	0.00	0.00	0.00
0.00	0.00	0.00	0.00	0.00	0.00	0.00	0.00	0.00
0.00	0.00	0.00	0.00	0.00	0.00	0.00	0.00	0.00
0.00	0.00	0.00	0.00	0.00	0.00	0.00	0.00	0.00
0.00	0.00	0.00	0.00	0.00	0.00	0.00	0.00	0.00
0.00	0.00	0.00	0.00	0.00	0.00	0.00	0.00	0.00
0.00	0.00	0.00	0.00	0.00	0.00	0.00	0.00	0.00
0.00	0.00	0.00	0.00	0.00	0.00	0.00	0.00	0.00
0.00	0.00	0.00	0.00	0.00	0.00	0.00	0.00	0.00
0.00	0.00	0.00	0.00	0.00	0.00	0.00	0.00	0.00
0.00	0.00	0.00	0.00	0.00	0.00	0.00	0.00	0.00
0.00	0.00	0.00	0.00	0.00	0.00	0.00	0.00	0.00
9.00	0.00	0.00	0.00	0.00	0.00	0.00	0.00	0.00
0.00	0.00	0.00	0.00	0.00	0.00	0.00	0.00	0.00
0.00	0.00	0.00	0.00	0.00	0.00	0.00	0.00	0.00
0.00	0.00	0.00	0.00	0.00	0.00	0.00	0.00	0.00
0.00	0.00	0.00	0.00	0.00	0.00	0.00	0.00	0.00
0.00	0.00	0.00	0.00	0.00	0.00	0.00	0.00	0.00
0.00	0.00	0.00	0.00	0.00	0.00	0.00	0.00	0.00
0.00	0.00	0.00	0.00	0.00	0.00	0.00	0.00	0.00
0.00	0.00	0.00	0.00	0.00	0.00	0.00	0.00	0.00
0.00	0.00	0.00	0.00	0.00	0.00	0.00	0.00	0.00
0.00	0.00	0.00	0.00	0.00	0.00	0.00	0.00	0.00
0.00	0.00	0.00	0.00	0.00	0.00	0.00	0.00	0.00
0.00	0.00	0.00	0.00	0.00	0.00	0.00	0.00	0.00
0.00	0.00	0.00	0.00	0.00	0.00	0.00	0.00	0.00
0.00	0.00	0.00	0.00	0.00	0.00	0.00	0.00	0.00
0.00	0.00	0.00	0.00	0.00	0.00	0.00	0.00	0.00
0.00	0.00	0.00	0.00	0.00	0.00	0.00	0.00	0.00

0.00	0.00	0.00	0.00	0.00	0.00	0.00	0.00	0.00
0.00	0.00	0.00	0.00	0.00	0.00	0.00	0.00	0.00
0.00	0.00	0.00	0.00	0.00	0.00	0.00	0.00	0.00
0.00	0.00	0.00	0.00	0.00	0.00	0.00	0.00	0.00
0.00	0.00	0.00	0.00	0.00	0.00	0.00	0.00	0.00
0.00	0.00	0.00	0.00	0.00	0.00	0.00	0.00	0.00
0.00	0.00	0.00	0.00	0.00	0.00	0.00	0.00	0.00
0.00	0.00	0.00	0.00	0.00	0.00	0.00	0.00	0.00
0.00	0.00	0.00	0.00	0.00	0.00	0.00	0.00	0.00
0.00	0.00	0.00	0.00	0.00	0.00	0.00	0.00	0.00
0.00	0.00	0.00	0.00	0.00	0.00	0.00	0.00	0.00
0.00	0.00	0.00	0.00	0.00	0.00	0.00	0.00	0.00
0.00	0.00	0.00	0.00	0.00	0.00	0.00	0.00	0.00
0.00	0.00	0.00	0.00	0.00	0.00	0.00	0.00	0.00
0.00	0.00	0.00	0.00	0.00	0.00	0.00	0.00	0.00
0.00	0.00	0.00	0.00	0.00	0.00	0.00	0.00	0.00
0.00	0.00	0.00	0.00	0.00	0.00	0.00	0.00	0.00
0.00	0.00	0.00	0.00	0.00	0.00	0.00	0.00	0.00
0.00	0.00	0.00	0.00	0.00	0.00	0.00	0.00	0.00
0.00	0.00	0.00	0.00	0.00	0.00	0.00	0.00	0.00
0.00	0.00	0.00	0.00	0.00	0.00	0.00	0.00	0.00
0.00	0.00	0.00	0.00	0.00	0.00	0.00	0.00	0.00
0.00	0.00	0.00	0.00	0.00	0.00	0.00	0.00	0.00
0.00	0.00	0.00	0.00	0.00	0.00	0.00	0.00	0.00
0.00	0.00	0.00	0.00	0.00	0.00	0.00	0.00	0.00
0.00	0.00	0.00	0.00	0.00	0.00	0.00	0.00	0.00
0.00	0.00	0.00	0.00	0.00	0.00	0.00	0.00	0.00
0.00	0.00	0.00	0.00	0.00	0.00	0.00	0.00	0.00
0.00	0.00	0.00	0.00	0.00	0.00	0.00	0.00	0.00
0.00	0.00	0.00	0.00	0.00	0.00	0.00	0.00	0.00
0.00	0.00	0.00	0.00	8.00	0.00	0.00	0.00	0.00
0.00	0.00	0.00	0.00	8.00	0.00	0.00	0.00	0.00
0.00	0.00	0.00	0.00	8.00	0.00	0.00	0.00	0.00
0.00	0.00	0.00	0.00	8.00	0.00	0.00	0.00	0.00
0.00	0.00	0.00	0.00	0.00	8.00	0.00	0.00	0.00
0.00	0.00	0.00	0.00	0.00	0.00	0.00	0.00	0.00
0.00	0.00	0.00	0.00	0.00	0.00	0.00	3.00	0.00

0.00	0.00	0.00	0.00	0.00	0.00	0.00	0.00	0.00
0.00	0.00	0.00	0.00	0.00	0.00	0.00	0.00	0.00
0.00	0.00	0.00	0.00	0.00	0.00	0.00	0.00	0.00
0.00	0.00	0.00	0.00	0.00	0.00	0.00	0.00	0.00
0.00	0.00	0.00	0.00	0.00	0.00	0.00	0.00	0.00
2.00	0.00	0.00	0.00	0.00	0.00	0.00	0.00	0.00
8.00	0.00	0.00	0.00	0.00	0.00	0.00	0.00	0.00
0.00	0.00	0.00	0.00	0.00	0.00	0.00	0.00	0.00
0.00	0.00	0.00	0.00	0.00	0.00	0.00	0.00	0.00
0.00	0.00	0.00	0.00	0.00	0.00	0.00	0.00	0.00
0.00	0.00	0.00	0.00	0.00	0.00	0.00	0.00	0.00
0.00	0.00	0.00	0.00	0.00	0.00	0.00	0.00	0.00
0.00	0.00	0.00	0.00	0.00	0.00	0.00	0.00	0.00
0.00	0.00	0.00	0.00	0.00	0.00	0.00	0.00	0.00
0.00	0.00	0.00	8.00	0.00	0.00	0.00	0.00	0.00
0.00	0.00	0.00	0.00	0.00	0.00	0.00	0.00	0.00
0.00	0.00	0.00	0.00	0.00	0.00	0.00	0.00	0.00
0.00	0.00	0.00	0.00	0.00	0.00	0.00	0.00	0.00
0.00	0.00	0.00	0.00	0.00	0.00	0.00	0.00	0.00
0.00	0.00	0.00	0.00	0.00	0.00	0.00	0.00	0.00
0.00	0.00	0.00	0.00	0.00	0.00	0.00	0.00	0.00
0.00	0.00	0.00	0.00	0.00	0.00	0.00	0.00	0.00
0.00	0.00	0.00	0.00	0.00	0.00	0.00	0.00	0.00
0.00	0.00	0.00	0.00	0.00	0.00	0.00	0.00	0.00
0.00	0.00	0.00	0.00	0.00	0.00	0.00	0.00	0.00
0.00	0.00	0.00	0.00	0.00	0.00	0.00	0.00	0.00
0.00	0.00	0.00	0.00	0.00	0.00	0.00	0.00	0.00
0.00	0.00	0.00	0.00	0.00	0.00	0.00	0.00	0.00
0.00	0.00	0.00	0.00	0.00	0.00	0.00	0.00	0.00
0.00	0.00	0.00	0.00	0.00	0.00	0.00	0.00	0.00
0.00	0.00	0.00	0.00	0.00	0.00	0.00	0.00	0.00
0.00	0.00	0.00	0.00	0.00	0.00	0.00	0.00	0.00
0.00	0.00	0.00	0.00	0.00	0.00	0.00	0.00	0.00
0.00	0.00	0.00	0.00	0.00	0.00	0.00	0.00	0.00
0.00	0.00	0.00	0.00	0.00	0.00	0.00	0.00	0.00
0.00	0.00	0.00	0.00	0.00	0.00	0.00	0.00	0.00
0.00	0.00	0.00	0.00	0.00	0.00	0.00	0.00	0.00

0.00	0.00	0.00	0.00	0.00	0.00	0.00	0.00	0.00
0.00	0.00	7.00	0.00	0.00	0.00	0.00	0.00	0.00
0.00	0.00	0.00	0.00	7.00	0.00	0.00	0.00	0.00
0.00	0.00	0.00	0.00	0.00	0.00	0.00	0.00	0.00
0.00	0.00	0.00	0.00	0.00	0.00	0.00	0.00	0.00
0.00	0.00	0.00	0.00	0.00	0.00	0.00	0.00	0.00
0.00	0.00	0.00	0.00	0.00	0.00	0.00	0.00	0.00
0.00	0.00	0.00	0.00	0.00	0.00	0.00	0.00	0.00
0.00	0.00	0.00	0.00	0.00	0.00	0.00	0.00	0.00
0.00	0.00	0.00	0.00	0.00	0.00	0.00	0.00	0.00
0.00	0.00	0.00	0.00	0.00	0.00	0.00	0.00	0.00
0.00	0.00	0.00	0.00	0.00	0.00	0.00	0.00	0.00
0.00	0.00	0.00	0.00	0.00	0.00	0.00	0.00	0.00
0.00	0.00	0.00	0.00	0.00	0.00	0.00	0.00	0.00
0.00	0.00	0.00	0.00	0.00	0.00	0.00	0.00	0.00
0.00	0.00	0.00	0.00	0.00	0.00	0.00	0.00	0.00
0.00	0.00	0.00	0.00	0.00	0.00	0.00	0.00	0.00
0.00	0.00	0.00	0.00	0.00	0.00	0.00	0.00	0.00
0.00	0.00	0.00	0.00	0.00	0.00	0.00	0.00	0.00
0.00	0.00	0.00	0.00	0.00	0.00	0.00	0.00	0.00
0.00	0.00	0.00	0.00	0.00	0.00	0.00	0.00	0.00
0.00	0.00	0.00	0.00	0.00	0.00	0.00	0.00	0.00
0.00	0.00	0.00	0.00	0.00	0.00	0.00	0.00	0.00
0.00	0.00	0.00	0.00	0.00	0.00	0.00	0.00	0.00
0.00	0.00	0.00	0.00	0.00	0.00	0.00	0.00	0.00
0.00	0.00	0.00	0.00	0.00	0.00	0.00	0.00	0.00
0.00	0.00	0.00	0.00	0.00	0.00	0.00	0.00	0.00
0.00	0.00	0.00	0.00	0.00	0.00	0.00	0.00	0.00
0.00	0.00	0.00	0.00	0.00	0.00	0.00	0.00	0.00
0.00	0.00	0.00	0.00	0.00	0.00	0.00	0.00	0.00
0.00	0.00	0.00	0.00	0.00	0.00	0.00	0.00	0.00
0.00	0.00	0.00	0.00	0.00	0.00	0.00	0.00	0.00
0.00	0.00	0.00	0.00	0.00	0.00	0.00	0.00	0.00
0.00	0.00	0.00	0.00	0.00	0.00	0.00	0.00	0.00
0.00	0.00	0.00	0.00	0.00	0.00	0.00	0.00	0.00
0.00	0.00	0.00	0.00	0.00	0.00	0.00	0.00	0.00
0.00	0.00	0.00	0.00	0.00	0.00	0.00	0.00	0.00

0.00	0.00	0.00	0.00	0.00	0.00	0.00	0.00	0.00
0.00	0.00	0.00	0.00	0.00	0.00	0.00	0.00	0.00
0.00	0.00	0.00	0.00	0.00	0.00	0.00	0.00	0.00
0.00	0.00	0.00	0.00	0.00	0.00	0.00	0.00	0.00
0.00	0.00	0.00	0.00	0.00	0.00	0.00	0.00	0.00
0.00	0.00	0.00	0.00	0.00	0.00	0.00	0.00	0.00
0.00	0.00	0.00	0.00	0.00	0.00	0.00	0.00	0.00
0.00	0.00	0.00	0.00	0.00	0.00	0.00	0.00	0.00
0.00	0.00	0.00	0.00	0.00	0.00	0.00	0.00	0.00
0.00	0.00	0.00	0.00	0.00	0.00	0.00	0.00	0.00
0.00	0.00	0.00	0.00	0.00	0.00	0.00	0.00	0.00
0.00	0.00	0.00	0.00	0.00	0.00	0.00	0.00	0.00
0.00	0.00	0.00	0.00	0.00	0.00	0.00	0.00	0.00
0.00	0.00	0.00	0.00	0.00	0.00	0.00	0.00	0.00
0.00	0.00	0.00	0.00	0.00	0.00	0.00	0.00	0.00
0.00	0.00	0.00	0.00	0.00	0.00	0.00	0.00	0.00
0.00	0.00	0.00	0.00	0.00	0.00	0.00	0.00	0.00
0.00	0.00	0.00	0.00	0.00	0.00	0.00	0.00	0.00
0.00	0.00	0.00	0.00	0.00	0.00	0.00	0.00	0.00
0.00	0.00	0.00	0.00	0.00	0.00	0.00	0.00	0.00
0.00	0.00	0.00	0.00	0.00	0.00	0.00	0.00	0.00
0.00	0.00	0.00	0.00	0.00	0.00	0.00	0.00	0.00
0.00	0.00	0.00	0.00	0.00	0.00	0.00	0.00	0.00
0.00	0.00	0.00	0.00	0.00	0.00	0.00	0.00	0.00
0.00	0.00	0.00	0.00	6.00	0.00	0.00	0.00	0.00
0.00	0.00	0.00	0.00	0.00	0.00	0.00	0.00	0.00
0.00	0.00	0.00	0.00	0.00	0.00	0.00	0.00	0.00
0.00	0.00	0.00	0.00	0.00	0.00	0.00	0.00	0.00
0.00	0.00	0.00	0.00	0.00	0.00	0.00	0.00	0.00
0.00	0.00	0.00	0.00	0.00	0.00	0.00	0.00	0.00
0.00	0.00	0.00	0.00	0.00	0.00	0.00	0.00	0.00
0.00	0.00	0.00	0.00	0.00	0.00	0.00	0.00	0.00
0.00	0.00	0.00	0.00	0.00	0.00	0.00	0.00	0.00
0.00	0.00	0.00	0.00	0.00	0.00	0.00	0.00	0.00
0.00	0.00	0.00	0.00	0.00	0.00	0.00	0.00	0.00
0.00	0.00	0.00	0.00	0.00	0.00	0.00	0.00	0.00
0.00	0.00	0.00	0.00	0.00	0.00	0.00	0.00	0.00

0.00	0.00	0.00	0.00	0.00	0.00	0.00	0.00	0.00
0.00	0.00	0.00	0.00	0.00	0.00	0.00	0.00	0.00
0.00	0.00	0.00	0.00	0.00	0.00	0.00	0.00	0.00
0.00	0.00	0.00	0.00	0.00	0.00	0.00	0.00	0.00
0.00	0.00	0.00	0.00	0.00	0.00	0.00	0.00	0.00
0.00	0.00	0.00	0.00	0.00	0.00	0.00	0.00	0.00
0.00	0.00	0.00	0.00	0.00	0.00	0.00	0.00	0.00
3.00	0.00	0.00	0.00	0.00	0.00	0.00	0.00	0.00
3.00	0.00	0.00	0.00	0.00	0.00	0.00	0.00	0.00
0.00	0.00	0.00	0.00	0.00	0.00	0.00	0.00	0.00
0.00	0.00	0.00	0.00	0.00	0.00	0.00	0.00	0.00
0.00	0.00	0.00	0.00	0.00	0.00	0.00	0.00	6.00
0.00	0.00	0.00	0.00	0.00	0.00	0.00	0.00	6.00
0.00	0.00	0.00	0.00	0.00	0.00	0.00	0.00	0.00
0.00	0.00	0.00	0.00	0.00	0.00	0.00	0.00	0.00
0.00	0.00	0.00	0.00	0.00	0.00	0.00	0.00	0.00
0.00	0.00	0.00	0.00	0.00	0.00	0.00	0.00	0.00
0.00	0.00	0.00	0.00	0.00	0.00	0.00	0.00	0.00
0.00	0.00	0.00	0.00	0.00	0.00	0.00	0.00	0.00
0.00	0.00	0.00	0.00	0.00	0.00	0.00	0.00	0.00
0.00	0.00	0.00	0.00	0.00	0.00	0.00	0.00	0.00
0.00	0.00	0.00	0.00	0.00	0.00	0.00	0.00	0.00
0.00	0.00	0.00	0.00	0.00	0.00	0.00	0.00	0.00
0.00	0.00	0.00	0.00	0.00	0.00	0.00	0.00	0.00
0.00	0.00	0.00	6.00	0.00	0.00	0.00	0.00	0.00
0.00	0.00	0.00	0.00	0.00	0.00	0.00	0.00	0.00
0.00	0.00	0.00	0.00	0.00	0.00	0.00	0.00	0.00
0.00	0.00	0.00	0.00	0.00	0.00	0.00	0.00	0.00
0.00	0.00	0.00	0.00	0.00	0.00	0.00	0.00	0.00
0.00	0.00	0.00	0.00	0.00	0.00	0.00	0.00	0.00
0.00	0.00	0.00	0.00	0.00	0.00	0.00	0.00	0.00
0.00	0.00	0.00	0.00	0.00	0.00	0.00	0.00	0.00
0.00	0.00	0.00	0.00	0.00	0.00	0.00	0.00	0.00
0.00	0.00	0.00	0.00	0.00	0.00	0.00	0.00	0.00
0.00	0.00	0.00	0.00	0.00	0.00	0.00	0.00	0.00
0.00	0.00	0.00	0.00	0.00	0.00	0.00	0.00	0.00
0.00	0.00	0.00	0.00	0.00	0.00	0.00	0.00	0.00

0.00	0.00	0.00	0.00	0.00	0.00	0.00	0.00	0.00
0.00	0.00	0.00	0.00	0.00	0.00	0.00	0.00	0.00
0.00	0.00	0.00	0.00	0.00	0.00	0.00	0.00	0.00
0.00	0.00	0.00	0.00	0.00	0.00	0.00	0.00	0.00
0.00	0.00	0.00	0.00	0.00	0.00	0.00	0.00	0.00
0.00	0.00	0.00	0.00	0.00	0.00	0.00	0.00	0.00
0.00	0.00	0.00	0.00	0.00	0.00	0.00	0.00	0.00
0.00	0.00	0.00	0.00	0.00	0.00	0.00	0.00	0.00
0.00	0.00	0.00	0.00	0.00	0.00	0.00	0.00	0.00
0.00	0.00	0.00	0.00	0.00	0.00	0.00	0.00	0.00
0.00	0.00	0.00	0.00	0.00	0.00	0.00	0.00	0.00
0.00	0.00	0.00	0.00	0.00	0.00	0.00	0.00	0.00
0.00	0.00	0.00	0.00	0.00	0.00	0.00	0.00	0.00
0.00	0.00	0.00	0.00	0.00	0.00	0.00	0.00	0.00
0.00	0.00	0.00	0.00	0.00	0.00	0.00	0.00	0.00
0.00	0.00	0.00	0.00	0.00	0.00	0.00	0.00	0.00
0.00	0.00	0.00	0.00	0.00	0.00	0.00	0.00	0.00
0.00	0.00	0.00	0.00	0.00	0.00	0.00	0.00	0.00
0.00	0.00	0.00	0.00	0.00	0.00	0.00	0.00	0.00
0.00	0.00	0.00	0.00	0.00	0.00	0.00	0.00	0.00
0.00	0.00	0.00	0.00	0.00	0.00	0.00	0.00	0.00
0.00	0.00	0.00	0.00	0.00	0.00	0.00	0.00	0.00
0.00	0.00	0.00	0.00	0.00	0.00	0.00	0.00	0.00
0.00	0.00	0.00	0.00	0.00	0.00	0.00	0.00	0.00
0.00	0.00	0.00	0.00	0.00	0.00	0.00	0.00	0.00
0.00	0.00	0.00	0.00	0.00	0.00	0.00	0.00	0.00
0.00	0.00	0.00	0.00	0.00	0.00	0.00	0.00	0.00
0.00	0.00	0.00	0.00	0.00	0.00	0.00	0.00	0.00
0.00	0.00	0.00	0.00	0.00	0.00	0.00	0.00	0.00
0.00	0.00	0.00	0.00	0.00	0.00	0.00	0.00	0.00
0.00	0.00	0.00	0.00	0.00	0.00	0.00	0.00	0.00
0.00	0.00	0.00	0.00	0.00	0.00	0.00	0.00	0.00
0.00	0.00	5.00	0.00	0.00	0.00	0.00	0.00	0.00
0.00	0.00	5.00	0.00	0.00	0.00	0.00	0.00	0.00
0.00	0.00	0.00	0.00	5.00	0.00	0.00	0.00	0.00
0.00	0.00	0.00	0.00	5.00	0.00	0.00	0.00	0.00
0.00	0.00	0.00	0.00	5.00	0.00	0.00	0.00	0.00

0.00	0.00	0.00	0.00	0.00	0.00	0.00	0.00	0.00
0.00	0.00	0.00	0.00	0.00	0.00	0.00	0.00	0.00
0.00	0.00	0.00	0.00	0.00	0.00	0.00	0.00	0.00
0.00	0.00	0.00	0.00	0.00	0.00	0.00	0.00	0.00
0.00	0.00	0.00	0.00	0.00	0.00	0.00	0.00	0.00
0.00	0.00	0.00	0.00	0.00	0.00	0.00	0.00	0.00
0.00	0.00	0.00	0.00	0.00	0.00	0.00	0.00	0.00
0.00	0.00	0.00	0.00	0.00	0.00	0.00	0.00	0.00
0.00	0.00	0.00	0.00	0.00	0.00	0.00	0.00	0.00
0.00	0.00	0.00	0.00	0.00	0.00	0.00	0.00	0.00
0.00	0.00	0.00	0.00	0.00	0.00	0.00	0.00	0.00
0.00	0.00	0.00	0.00	0.00	0.00	0.00	0.00	0.00
0.00	0.00	0.00	0.00	0.00	0.00	0.00	0.00	0.00
0.00	0.00	0.00	0.00	0.00	0.00	0.00	0.00	0.00
0.00	0.00	0.00	0.00	0.00	0.00	0.00	0.00	0.00
0.00	0.00	0.00	0.00	0.00	0.00	0.00	0.00	0.00
5.00	0.00	0.00	0.00	0.00	0.00	0.00	0.00	0.00
5.00	0.00	0.00	0.00	0.00	0.00	0.00	0.00	0.00
0.00	0.00	0.00	0.00	0.00	0.00	5.00	0.00	0.00
0.00	0.00	0.00	0.00	0.00	0.00	0.00	0.00	0.00
0.00	0.00	0.00	0.00	0.00	0.00	0.00	0.00	0.00
0.00	0.00	0.00	0.00	0.00	0.00	0.00	0.00	0.00
0.00	0.00	0.00	0.00	0.00	0.00	0.00	0.00	0.00
0.00	0.00	0.00	0.00	0.00	0.00	0.00	0.00	0.00
0.00	0.00	0.00	0.00	0.00	0.00	0.00	0.00	0.00
0.00	0.00	0.00	0.00	0.00	0.00	0.00	0.00	0.00
0.00	0.00	0.00	0.00	0.00	0.00	0.00	0.00	0.00
0.00	0.00	0.00	0.00	0.00	0.00	0.00	0.00	0.00
0.00	0.00	0.00	0.00	0.00	0.00	0.00	0.00	0.00
0.00	0.00	0.00	0.00	0.00	0.00	0.00	0.00	0.00
0.00	0.00	0.00	0.00	0.00	0.00	0.00	0.00	0.00
0.00	0.00	0.00	0.00	0.00	0.00	0.00	0.00	0.00
0.00	0.00	0.00	0.00	0.00	0.00	0.00	0.00	0.00
0.00	0.00	0.00	0.00	0.00	0.00	0.00	0.00	0.00
0.00	0.00	0.00	0.00	0.00	0.00	0.00	0.00	0.00
0.00	0.00	0.00	0.00	0.00	0.00	0.00	0.00	0.00
0.00	0.00	0.00	0.00	0.00	0.00	0.00	0.00	0.00

0.00	0.00	0.00	0.00	0.00	0.00	0.00	0.00	0.00
0.00	0.00	0.00	0.00	0.00	0.00	0.00	0.00	0.00
0.00	0.00	0.00	0.00	0.00	0.00	0.00	0.00	0.00
0.00	0.00	0.00	0.00	0.00	0.00	0.00	0.00	0.00
0.00	0.00	0.00	0.00	0.00	0.00	0.00	0.00	0.00
0.00	0.00	0.00	0.00	0.00	0.00	0.00	0.00	0.00
0.00	0.00	0.00	0.00	0.00	0.00	0.00	0.00	0.00
0.00	0.00	0.00	0.00	0.00	0.00	0.00	0.00	0.00
0.00	0.00	0.00	0.00	0.00	0.00	0.00	0.00	0.00
0.00	0.00	0.00	0.00	0.00	0.00	0.00	0.00	0.00
0.00	0.00	0.00	0.00	0.00	0.00	0.00	0.00	0.00
0.00	0.00	0.00	0.00	0.00	0.00	0.00	0.00	0.00
0.00	0.00	0.00	0.00	0.00	0.00	0.00	0.00	0.00
0.00	0.00	0.00	0.00	0.00	0.00	0.00	0.00	0.00
0.00	0.00	0.00	0.00	0.00	0.00	0.00	0.00	0.00
0.00	0.00	0.00	0.00	0.00	0.00	0.00	0.00	0.00
0.00	0.00	0.00	0.00	0.00	0.00	0.00	0.00	0.00
0.00	0.00	0.00	0.00	0.00	0.00	0.00	0.00	0.00
0.00	0.00	0.00	0.00	0.00	0.00	0.00	0.00	0.00
0.00	0.00	0.00	0.00	0.00	0.00	0.00	0.00	0.00
0.00	0.00	0.00	0.00	0.00	0.00	0.00	0.00	0.00
0.00	0.00	0.00	0.00	0.00	0.00	0.00	0.00	0.00
0.00	0.00	0.00	0.00	0.00	0.00	0.00	0.00	0.00
0.00	0.00	0.00	0.00	0.00	0.00	0.00	0.00	0.00
0.00	0.00	0.00	0.00	0.00	0.00	0.00	0.00	0.00
0.00	0.00	0.00	0.00	0.00	0.00	0.00	0.00	0.00
0.00	0.00	0.00	0.00	0.00	0.00	0.00	0.00	0.00
0.00	0.00	0.00	0.00	0.00	0.00	0.00	0.00	0.00
0.00	0.00	0.00	0.00	0.00	0.00	0.00	0.00	0.00
0.00	0.00	0.00	0.00	0.00	0.00	0.00	0.00	0.00
0.00	0.00	0.00	0.00	0.00	0.00	0.00	0.00	0.00
0.00	0.00	0.00	0.00	0.00	0.00	0.00	0.00	0.00
0.00	0.00	0.00	0.00	0.00	0.00	0.00	0.00	0.00
0.00	0.00	0.00	0.00	0.00	0.00	0.00	0.00	0.00
0.00	0.00	0.00	0.00	0.00	0.00	0.00	0.00	0.00
0.00	0.00	0.00	0.00	0.00	0.00	0.00	0.00	0.00
0.00	0.00	0.00	0.00	0.00	0.00	0.00	0.00	0.00

0.00	0.00	0.00	0.00	0.00	0.00	0.00	0.00	0.00
0.00	0.00	0.00	0.00	0.00	0.00	0.00	0.00	0.00
0.00	0.00	0.00	0.00	0.00	0.00	0.00	0.00	0.00
0.00	0.00	0.00	0.00	0.00	0.00	0.00	0.00	0.00
0.00	0.00	0.00	0.00	0.00	0.00	0.00	0.00	0.00
0.00	0.00	0.00	0.00	0.00	0.00	0.00	0.00	0.00
0.00	0.00	0.00	0.00	0.00	0.00	0.00	0.00	0.00
0.00	0.00	0.00	0.00	0.00	0.00	0.00	0.00	0.00
0.00	0.00	0.00	0.00	0.00	0.00	0.00	0.00	0.00
0.00	0.00	0.00	0.00	0.00	0.00	0.00	0.00	0.00
0.00	0.00	0.00	0.00	0.00	0.00	0.00	0.00	0.00
0.00	0.00	0.00	0.00	4.00	0.00	0.00	0.00	0.00
0.00	0.00	0.00	0.00	4.00	0.00	0.00	0.00	0.00
0.00	0.00	0.00	0.00	0.00	0.00	0.00	0.00	0.00
0.00	0.00	0.00	0.00	0.00	0.00	0.00	0.00	0.00
0.00	0.00	0.00	0.00	0.00	0.00	0.00	0.00	0.00
0.00	0.00	0.00	0.00	0.00	0.00	0.00	0.00	0.00
0.00	0.00	0.00	0.00	0.00	0.00	0.00	0.00	0.00
0.00	0.00	0.00	0.00	0.00	0.00	0.00	0.00	0.00
2.00	0.00	0.00	0.00	0.00	0.00	0.00	0.00	0.00
0.00	0.00	0.00	0.00	0.00	0.00	0.00	0.00	0.00
0.00	0.00	0.00	0.00	0.00	0.00	0.00	0.00	0.00
0.00	0.00	0.00	0.00	0.00	0.00	0.00	0.00	0.00
0.00	0.00	0.00	0.00	0.00	0.00	0.00	0.00	0.00
0.00	0.00	0.00	0.00	0.00	0.00	0.00	0.00	0.00
0.00	0.00	0.00	0.00	0.00	0.00	0.00	0.00	0.00
0.00	0.00	0.00	0.00	0.00	0.00	0.00	0.00	0.00
0.00	0.00	0.00	0.00	0.00	0.00	0.00	0.00	0.00
0.00	0.00	0.00	0.00	0.00	0.00	0.00	0.00	0.00
0.00	4.00	0.00	0.00	0.00	0.00	0.00	0.00	0.00
0.00	0.00	0.00	0.00	0.00	0.00	0.00	0.00	0.00
0.00	0.00	0.00	0.00	0.00	0.00	0.00	0.00	0.00
0.00	0.00	0.00	0.00	0.00	0.00	4.00	0.00	0.00
0.00	0.00	0.00	0.00	0.00	0.00	4.00	0.00	0.00
0.00	0.00	0.00	0.00	0.00	0.00	0.00	4.00	0.00
0.00	0.00	0.00	0.00	0.00	0.00	0.00	0.00	4.00
0.00	0.00	0.00	0.00	0.00	0.00	0.00	0.00	4.00

0.00	0.00	0.00	0.00	0.00	0.00	0.00	0.00	0.00
0.00	0.00	0.00	0.00	0.00	0.00	0.00	0.00	0.00
0.00	0.00	0.00	0.00	0.00	0.00	0.00	0.00	0.00
0.00	0.00	0.00	0.00	0.00	0.00	0.00	0.00	0.00
0.00	0.00	0.00	0.00	0.00	0.00	0.00	0.00	0.00
0.00	0.00	0.00	0.00	0.00	0.00	0.00	0.00	0.00
0.00	0.00	0.00	0.00	0.00	0.00	0.00	0.00	0.00
0.00	0.00	0.00	0.00	0.00	0.00	0.00	0.00	0.00
0.00	0.00	0.00	0.00	0.00	0.00	0.00	0.00	0.00
0.00	0.00	0.00	0.00	0.00	0.00	0.00	0.00	0.00
0.00	0.00	0.00	0.00	0.00	0.00	0.00	0.00	0.00
0.00	0.00	0.00	0.00	0.00	0.00	0.00	0.00	0.00
0.00	0.00	0.00	0.00	0.00	0.00	0.00	0.00	0.00
0.00	0.00	0.00	0.00	0.00	0.00	0.00	0.00	0.00
0.00	0.00	0.00	0.00	0.00	0.00	0.00	0.00	0.00
0.00	0.00	0.00	0.00	0.00	0.00	0.00	0.00	0.00
0.00	0.00	0.00	0.00	0.00	0.00	0.00	0.00	0.00
0.00	0.00	0.00	0.00	0.00	0.00	0.00	0.00	0.00
0.00	0.00	0.00	0.00	0.00	0.00	0.00	0.00	0.00
0.00	0.00	0.00	0.00	0.00	0.00	0.00	0.00	0.00
0.00	0.00	0.00	0.00	0.00	0.00	0.00	0.00	0.00
0.00	0.00	0.00	0.00	0.00	0.00	0.00	0.00	0.00
0.00	0.00	0.00	0.00	0.00	0.00	0.00	0.00	0.00
0.00	0.00	0.00	0.00	0.00	0.00	0.00	0.00	0.00
0.00	0.00	0.00	0.00	0.00	0.00	0.00	0.00	0.00
0.00	0.00	0.00	0.00	0.00	0.00	0.00	0.00	0.00
0.00	0.00	0.00	0.00	0.00	0.00	0.00	0.00	0.00
0.00	0.00	0.00	0.00	0.00	0.00	0.00	0.00	0.00
0.00	0.00	0.00	0.00	0.00	0.00	0.00	0.00	0.00
0.00	0.00	0.00	0.00	0.00	0.00	0.00	0.00	0.00
0.00	0.00	0.00	0.00	0.00	0.00	0.00	0.00	0.00
0.00	0.00	0.00	0.00	0.00	0.00	0.00	0.00	0.00
0.00	0.00	0.00	0.00	0.00	0.00	0.00	0.00	0.00
0.00	0.00	0.00	0.00	0.00	0.00	0.00	0.00	0.00
0.00	0.00	0.00	0.00	0.00	0.00	0.00	0.00	0.00
0.00	0.00	0.00	0.00	0.00	0.00	0.00	0.00	0.00
0.00	0.00	0.00	0.00	0.00	0.00	0.00	0.00	0.00

0.00	0.00	0.00	0.00	0.00	0.00	0.00	0.00	0.00
0.00	0.00	0.00	0.00	0.00	0.00	0.00	0.00	0.00
0.00	0.00	0.00	0.00	0.00	0.00	0.00	0.00	0.00
0.00	0.00	0.00	0.00	0.00	0.00	0.00	0.00	0.00
0.00	0.00	0.00	0.00	0.00	0.00	0.00	0.00	0.00
0.00	0.00	0.00	0.00	0.00	0.00	0.00	0.00	0.00
0.00	0.00	0.00	0.00	0.00	0.00	0.00	0.00	0.00
0.00	0.00	0.00	0.00	0.00	0.00	0.00	0.00	0.00
0.00	0.00	0.00	0.00	0.00	0.00	0.00	0.00	0.00
0.00	0.00	0.00	0.00	0.00	0.00	0.00	0.00	0.00
0.00	0.00	0.00	0.00	0.00	0.00	0.00	0.00	0.00
0.00	0.00	0.00	0.00	0.00	0.00	0.00	0.00	0.00
0.00	0.00	0.00	0.00	0.00	0.00	0.00	0.00	0.00
0.00	0.00	0.00	0.00	0.00	0.00	0.00	0.00	0.00
0.00	0.00	0.00	0.00	0.00	0.00	0.00	0.00	0.00
0.00	0.00	0.00	0.00	0.00	0.00	0.00	0.00	0.00
0.00	0.00	0.00	0.00	0.00	0.00	0.00	0.00	0.00
0.00	0.00	0.00	0.00	0.00	0.00	0.00	0.00	0.00
0.00	0.00	0.00	0.00	0.00	0.00	0.00	0.00	0.00
0.00	0.00	0.00	0.00	0.00	0.00	0.00	0.00	0.00
0.00	0.00	0.00	0.00	0.00	0.00	0.00	0.00	0.00
0.00	0.00	0.00	0.00	0.00	0.00	0.00	0.00	0.00
0.00	0.00	0.00	0.00	0.00	0.00	0.00	0.00	0.00
0.00	0.00	0.00	0.00	0.00	0.00	0.00	0.00	0.00
0.00	0.00	0.00	0.00	0.00	0.00	0.00	0.00	0.00
0.00	0.00	0.00	0.00	0.00	0.00	0.00	0.00	0.00
0.00	0.00	0.00	0.00	0.00	0.00	0.00	0.00	0.00
0.00	0.00	0.00	0.00	0.00	0.00	0.00	0.00	0.00
0.00	0.00	0.00	0.00	0.00	0.00	0.00	0.00	0.00
0.00	0.00	0.00	0.00	0.00	0.00	0.00	0.00	0.00
0.00	0.00	0.00	0.00	0.00	0.00	0.00	0.00	0.00
0.00	0.00	0.00	0.00	0.00	0.00	0.00	0.00	0.00
0.00	0.00	0.00	0.00	0.00	0.00	0.00	0.00	0.00
0.00	0.00	0.00	0.00	0.00	0.00	0.00	0.00	0.00
0.00	0.00	0.00	0.00	0.00	0.00	0.00	0.00	0.00
0.00	0.00	0.00	0.00	0.00	0.00	0.00	0.00	0.00
0.00	0.00	0.00	0.00	0.00	0.00	0.00	0.00	0.00

0.00	0.00	0.00	0.00	0.00	0.00	0.00	0.00	0.00
0.00	0.00	0.00	0.00	0.00	0.00	0.00	0.00	0.00
0.00	0.00	0.00	0.00	0.00	0.00	0.00	0.00	0.00
0.00	0.00	0.00	0.00	0.00	0.00	0.00	0.00	0.00
0.00	0.00	0.00	0.00	0.00	0.00	0.00	0.00	0.00
0.00	0.00	0.00	0.00	0.00	0.00	0.00	0.00	0.00
0.00	0.00	0.00	0.00	0.00	0.00	0.00	0.00	0.00
0.00	0.00	0.00	0.00	0.00	0.00	0.00	0.00	0.00
0.00	0.00	0.00	0.00	0.00	0.00	0.00	0.00	0.00
0.00	0.00	0.00	0.00	0.00	0.00	0.00	0.00	0.00
0.00	0.00	0.00	0.00	0.00	0.00	0.00	0.00	0.00
0.00	0.00	0.00	0.00	0.00	0.00	0.00	0.00	0.00
0.00	0.00	0.00	0.00	0.00	0.00	0.00	0.00	0.00
0.00	0.00	0.00	0.00	0.00	0.00	0.00	0.00	0.00
0.00	0.00	0.00	0.00	0.00	0.00	0.00	0.00	0.00
0.00	0.00	0.00	0.00	0.00	0.00	0.00	0.00	0.00
0.00	0.00	0.00	0.00	0.00	0.00	0.00	0.00	0.00
0.00	0.00	0.00	0.00	0.00	0.00	0.00	0.00	0.00
0.00	0.00	0.00	0.00	0.00	0.00	0.00	0.00	0.00
0.00	0.00	0.00	0.00	0.00	0.00	0.00	0.00	0.00
0.00	0.00	0.00	0.00	0.00	0.00	0.00	0.00	0.00
0.00	0.00	0.00	0.00	0.00	0.00	0.00	0.00	0.00
0.00	0.00	0.00	0.00	0.00	0.00	0.00	0.00	0.00
0.00	0.00	0.00	0.00	0.00	0.00	0.00	0.00	0.00
0.00	0.00	0.00	0.00	0.00	0.00	0.00	0.00	0.00
0.00	0.00	0.00	0.00	0.00	0.00	0.00	0.00	0.00
0.00	0.00	0.00	0.00	0.00	0.00	0.00	0.00	0.00
0.00	0.00	0.00	0.00	0.00	0.00	0.00	0.00	0.00
0.00	0.00	0.00	0.00	0.00	0.00	0.00	0.00	0.00
0.00	0.00	0.00	0.00	0.00	0.00	0.00	0.00	0.00
0.00	0.00	0.00	0.00	0.00	0.00	0.00	0.00	0.00
0.00	0.00	0.00	0.00	0.00	0.00	0.00	0.00	0.00
0.00	0.00	0.00	0.00	0.00	0.00	0.00	0.00	0.00
0.00	0.00	0.00	0.00	0.00	0.00	0.00	0.00	0.00
0.00	0.00	0.00	0.00	0.00	0.00	0.00	0.00	0.00
0.00	0.00	0.00	0.00	0.00	0.00	0.00	0.00	0.00
0.00	0.00	0.00	0.00	0.00	0.00	0.00	0.00	0.00

0.00	0.00	0.00	0.00	0.00	0.00	0.00	0.00	0.00
0.00	0.00	0.00	0.00	0.00	0.00	0.00	0.00	0.00
0.00	0.00	0.00	0.00	0.00	0.00	0.00	0.00	0.00
0.00	0.00	0.00	0.00	0.00	0.00	0.00	0.00	0.00
0.00	0.00	0.00	0.00	0.00	0.00	0.00	0.00	0.00
0.00	0.00	0.00	0.00	0.00	0.00	0.00	0.00	0.00
0.00	0.00	0.00	0.00	0.00	0.00	0.00	0.00	0.00
0.00	0.00	0.00	0.00	0.00	0.00	0.00	0.00	0.00
0.00	0.00	0.00	0.00	0.00	0.00	0.00	0.00	0.00
0.00	0.00	0.00	0.00	0.00	0.00	0.00	0.00	0.00
0.00	0.00	0.00	0.00	0.00	0.00	0.00	0.00	0.00
0.00	0.00	0.00	0.00	0.00	0.00	0.00	0.00	0.00
3.00	0.00	0.00	0.00	0.00	0.00	0.00	0.00	0.00
3.00	0.00	0.00	0.00	0.00	0.00	0.00	0.00	0.00
0.00	3.00	0.00	0.00	0.00	0.00	0.00	0.00	0.00
0.00	3.00	0.00	0.00	0.00	0.00	0.00	0.00	0.00
0.00	3.00	0.00	0.00	0.00	0.00	0.00	0.00	0.00
0.00	0.00	0.00	0.00	0.00	0.00	0.00	0.00	0.00
0.00	0.00	0.00	0.00	0.00	0.00	0.00	0.00	0.00
0.00	0.00	0.00	0.00	0.00	0.00	0.00	0.00	3.00
0.00	0.00	0.00	0.00	0.00	0.00	0.00	0.00	0.00
0.00	0.00	0.00	0.00	0.00	0.00	0.00	0.00	0.00
0.00	0.00	0.00	0.00	0.00	0.00	0.00	0.00	0.00
0.00	0.00	0.00	0.00	0.00	0.00	0.00	0.00	0.00
0.00	0.00	0.00	0.00	0.00	0.00	0.00	0.00	0.00
0.00	0.00	0.00	0.00	0.00	0.00	0.00	0.00	0.00
0.00	0.00	0.00	0.00	0.00	0.00	0.00	0.00	0.00
0.00	0.00	0.00	0.00	0.00	0.00	0.00	0.00	0.00
0.00	0.00	0.00	0.00	0.00	0.00	0.00	0.00	0.00
0.00	0.00	0.00	0.00	0.00	0.00	0.00	0.00	0.00
0.00	0.00	0.00	0.00	0.00	0.00	0.00	0.00	0.00
0.00	0.00	0.00	0.00	0.00	0.00	0.00	0.00	0.00
0.00	0.00	0.00	0.00	0.00	0.00	0.00	0.00	0.00
0.00	0.00	0.00	0.00	0.00	0.00	0.00	0.00	0.00
0.00	0.00	0.00	0.00	0.00	0.00	0.00	0.00	0.00
0.00	0.00	0.00	0.00	0.00	0.00	0.00	0.00	0.00
0.00	0.00	0.00	0.00	0.00	0.00	0.00	0.00	0.00

0.00	0.00	0.00	0.00	0.00	0.00	0.00	0.00	0.00
0.00	0.00	0.00	0.00	0.00	0.00	0.00	0.00	0.00
0.00	0.00	0.00	0.00	0.00	0.00	0.00	0.00	0.00
0.00	0.00	0.00	0.00	0.00	0.00	0.00	0.00	0.00
0.00	0.00	0.00	0.00	0.00	0.00	0.00	0.00	0.00
0.00	0.00	0.00	0.00	0.00	0.00	0.00	0.00	0.00
0.00	0.00	0.00	0.00	0.00	0.00	0.00	0.00	0.00
0.00	0.00	0.00	3.00	0.00	0.00	0.00	0.00	0.00
0.00	0.00	0.00	3.00	0.00	0.00	0.00	0.00	0.00
0.00	0.00	0.00	0.00	0.00	0.00	0.00	0.00	0.00
0.00	0.00	0.00	0.00	0.00	0.00	0.00	0.00	0.00
0.00	0.00	0.00	0.00	0.00	0.00	0.00	0.00	0.00
0.00	0.00	0.00	0.00	0.00	0.00	0.00	0.00	0.00
0.00	0.00	0.00	0.00	0.00	0.00	0.00	0.00	0.00
0.00	0.00	0.00	0.00	0.00	0.00	0.00	0.00	0.00
0.00	0.00	0.00	0.00	0.00	0.00	0.00	0.00	0.00
0.00	0.00	0.00	0.00	0.00	0.00	0.00	0.00	0.00
0.00	0.00	0.00	0.00	0.00	0.00	0.00	0.00	0.00
0.00	0.00	0.00	0.00	0.00	0.00	0.00	0.00	0.00
0.00	0.00	0.00	0.00	0.00	0.00	0.00	0.00	0.00
0.00	0.00	0.00	0.00	0.00	0.00	0.00	0.00	0.00
0.00	0.00	0.00	0.00	0.00	0.00	0.00	0.00	0.00
0.00	0.00	0.00	0.00	0.00	0.00	0.00	0.00	0.00
0.00	0.00	0.00	0.00	0.00	0.00	0.00	0.00	0.00
0.00	0.00	0.00	0.00	0.00	0.00	0.00	0.00	0.00
0.00	0.00	0.00	0.00	0.00	0.00	0.00	0.00	0.00
0.00	0.00	0.00	0.00	0.00	0.00	0.00	0.00	0.00
0.00	0.00	0.00	0.00	0.00	0.00	0.00	0.00	0.00
0.00	0.00	0.00	0.00	0.00	0.00	0.00	0.00	0.00
0.00	0.00	0.00	0.00	0.00	0.00	0.00	0.00	0.00
0.00	0.00	0.00	0.00	0.00	0.00	0.00	0.00	0.00
0.00	0.00	0.00	0.00	0.00	0.00	0.00	0.00	0.00
0.00	0.00	0.00	0.00	0.00	0.00	0.00	0.00	0.00
0.00	0.00	0.00	0.00	0.00	0.00	0.00	0.00	0.00
0.00	0.00	0.00	0.00	0.00	0.00	0.00	0.00	0.00
0.00	0.00	0.00	0.00	0.00	0.00	0.00	0.00	0.00
0.00	0.00	0.00	0.00	0.00	0.00	0.00	0.00	0.00

0.00	0.00	0.00	0.00	0.00	0.00	0.00	0.00	0.00
0.00	0.00	0.00	0.00	0.00	0.00	0.00	0.00	0.00
0.00	0.00	0.00	0.00	0.00	0.00	0.00	0.00	0.00
0.00	0.00	0.00	0.00	0.00	0.00	0.00	0.00	0.00
0.00	0.00	0.00	0.00	0.00	0.00	0.00	0.00	0.00
0.00	0.00	0.00	0.00	0.00	0.00	0.00	0.00	0.00
0.00	0.00	0.00	0.00	0.00	0.00	0.00	0.00	0.00
0.00	0.00	0.00	0.00	0.00	0.00	0.00	0.00	0.00
0.00	0.00	0.00	0.00	0.00	0.00	0.00	0.00	0.00
0.00	0.00	0.00	0.00	0.00	0.00	0.00	0.00	0.00
0.00	0.00	0.00	0.00	0.00	0.00	0.00	0.00	0.00
0.00	0.00	0.00	0.00	0.00	0.00	0.00	0.00	0.00
0.00	0.00	0.00	0.00	0.00	0.00	0.00	0.00	0.00
0.00	0.00	0.00	0.00	0.00	0.00	0.00	0.00	0.00
0.00	0.00	0.00	0.00	0.00	0.00	0.00	0.00	0.00
0.00	0.00	0.00	0.00	0.00	0.00	0.00	0.00	0.00
0.00	0.00	0.00	0.00	0.00	0.00	0.00	0.00	0.00
0.00	0.00	0.00	0.00	0.00	0.00	0.00	0.00	0.00
0.00	0.00	0.00	0.00	0.00	0.00	0.00	0.00	0.00
0.00	0.00	0.00	0.00	0.00	0.00	0.00	0.00	0.00
0.00	0.00	0.00	0.00	0.00	0.00	0.00	0.00	0.00
0.00	0.00	0.00	0.00	0.00	0.00	0.00	0.00	0.00
0.00	0.00	0.00	0.00	0.00	0.00	0.00	0.00	0.00
0.00	0.00	0.00	0.00	0.00	0.00	0.00	0.00	0.00
0.00	0.00	0.00	0.00	0.00	0.00	0.00	0.00	0.00
0.00	0.00	0.00	0.00	0.00	0.00	0.00	0.00	0.00
0.00	0.00	0.00	0.00	0.00	0.00	0.00	0.00	0.00
0.00	0.00	0.00	0.00	0.00	0.00	0.00	0.00	0.00
0.00	0.00	0.00	0.00	0.00	0.00	0.00	0.00	0.00
0.00	0.00	0.00	0.00	0.00	0.00	0.00	0.00	0.00
0.00	0.00	0.00	0.00	0.00	0.00	0.00	0.00	0.00
0.00	0.00	0.00	0.00	0.00	0.00	0.00	0.00	0.00
0.00	0.00	0.00	0.00	0.00	0.00	0.00	0.00	0.00
0.00	0.00	0.00	0.00	0.00	0.00	0.00	0.00	0.00
0.00	0.00	0.00	0.00	0.00	0.00	0.00	0.00	0.00
0.00	0.00	0.00	0.00	0.00	0.00	0.00	0.00	0.00
0.00	0.00	0.00	0.00	0.00	0.00	0.00	0.00	0.00

0.00	0.00	0.00	0.00	0.00	0.00	0.00	0.00	0.00
0.00	0.00	0.00	0.00	0.00	0.00	0.00	0.00	0.00
0.00	0.00	0.00	0.00	0.00	0.00	0.00	0.00	0.00
0.00	0.00	0.00	0.00	0.00	0.00	0.00	0.00	0.00
0.00	0.00	0.00	0.00	0.00	0.00	0.00	0.00	0.00
0.00	0.00	0.00	0.00	0.00	0.00	0.00	0.00	0.00
0.00	0.00	0.00	0.00	0.00	0.00	0.00	0.00	0.00
0.00	0.00	0.00	0.00	0.00	0.00	0.00	0.00	0.00
0.00	0.00	0.00	0.00	0.00	0.00	0.00	0.00	0.00
0.00	0.00	0.00	0.00	0.00	0.00	0.00	0.00	0.00
0.00	0.00	0.00	0.00	0.00	0.00	0.00	0.00	0.00
0.00	0.00	0.00	0.00	0.00	0.00	0.00	0.00	0.00
0.00	0.00	0.00	0.00	0.00	0.00	0.00	0.00	0.00
0.00	0.00	0.00	0.00	0.00	0.00	0.00	0.00	0.00
0.00	0.00	0.00	0.00	0.00	0.00	0.00	0.00	0.00
0.00	0.00	0.00	0.00	0.00	0.00	0.00	0.00	0.00
0.00	0.00	0.00	0.00	2.00	0.00	0.00	0.00	0.00
0.00	0.00	0.00	0.00	0.00	2.00	0.00	0.00	0.00
0.00	0.00	0.00	0.00	0.00	2.00	0.00	0.00	0.00
0.00	0.00	0.00	0.00	0.00	0.00	0.00	0.00	0.00
0.00	0.00	0.00	0.00	0.00	0.00	0.00	0.00	0.00
0.00	0.00	0.00	0.00	0.00	0.00	0.00	0.00	0.00
0.00	0.00	0.00	0.00	0.00	0.00	0.00	0.00	0.00
0.00	0.00	0.00	0.00	0.00	0.00	0.00	0.00	0.00
0.00	0.00	0.00	0.00	0.00	0.00	0.00	0.00	0.00
0.00	0.00	0.00	0.00	0.00	0.00	0.00	0.00	0.00
0.00	0.00	0.00	0.00	0.00	0.00	0.00	0.00	0.00
0.00	0.00	0.00	0.00	0.00	0.00	0.00	0.00	0.00
0.00	0.00	0.00	0.00	0.00	0.00	0.00	0.00	0.00
0.00	0.00	0.00	0.00	0.00	0.00	0.00	0.00	0.00
0.00	0.00	0.00	0.00	0.00	0.00	0.00	0.00	0.00
0.00	0.00	0.00	0.00	0.00	0.00	0.00	0.00	0.00
0.00	0.00	0.00	0.00	0.00	0.00	0.00	0.00	0.00
0.00	0.00	0.00	0.00	0.00	0.00	0.00	0.00	0.00
0.00	0.00	0.00	0.00	0.00	0.00	0.00	0.00	0.00
0.00	0.00	0.00	0.00	0.00	0.00	0.00	0.00	0.00
0.00	0.00	0.00	0.00	0.00	0.00	0.00	0.00	0.00

0.00	0.00	0.00	0.00	0.00	0.00	0.00	0.00	0.00
0.00	0.00	0.00	0.00	0.00	0.00	0.00	0.00	0.00
0.00	0.00	0.00	0.00	0.00	0.00	0.00	0.00	0.00
0.00	0.00	0.00	0.00	0.00	0.00	0.00	0.00	0.00
2.00	0.00	0.00	0.00	0.00	0.00	0.00	0.00	0.00
2.00	0.00	0.00	0.00	0.00	0.00	0.00	0.00	0.00
2.00	0.00	0.00	0.00	0.00	0.00	0.00	0.00	0.00
0.00	2.00	0.00	0.00	0.00	0.00	0.00	0.00	0.00
0.00	2.00	0.00	0.00	0.00	0.00	0.00	0.00	0.00
0.00	0.00	0.00	0.00	0.00	0.00	0.00	0.00	0.00
0.00	0.00	0.00	0.00	0.00	0.00	0.00	0.00	0.00
0.00	0.00	0.00	0.00	0.00	0.00	0.00	0.00	0.00
0.00	0.00	0.00	0.00	0.00	0.00	0.00	0.00	0.00
0.00	0.00	0.00	0.00	0.00	0.00	0.00	2.00	0.00
0.00	0.00	0.00	0.00	0.00	0.00	0.00	2.00	0.00
0.00	0.00	0.00	0.00	0.00	0.00	0.00	0.00	2.00
0.00	0.00	0.00	0.00	0.00	0.00	0.00	0.00	2.00
0.00	0.00	0.00	0.00	0.00	0.00	0.00	0.00	0.00
0.00	0.00	0.00	0.00	0.00	0.00	0.00	0.00	0.00
0.00	0.00	0.00	0.00	0.00	0.00	0.00	0.00	0.00
0.00	0.00	0.00	0.00	0.00	0.00	0.00	0.00	0.00
0.00	0.00	0.00	0.00	0.00	0.00	0.00	0.00	0.00
0.00	0.00	0.00	0.00	0.00	0.00	0.00	0.00	0.00
0.00	0.00	0.00	0.00	0.00	0.00	0.00	0.00	0.00
0.00	0.00	0.00	0.00	0.00	0.00	0.00	0.00	0.00
0.00	0.00	0.00	0.00	0.00	0.00	0.00	0.00	0.00
0.00	0.00	0.00	0.00	0.00	0.00	0.00	0.00	0.00
0.00	0.00	0.00	0.00	0.00	0.00	0.00	0.00	0.00
0.00	0.00	0.00	0.00	0.00	0.00	0.00	0.00	0.00
0.00	0.00	0.00	0.00	0.00	0.00	0.00	0.00	0.00
0.00	0.00	0.00	0.00	0.00	0.00	0.00	0.00	0.00
0.00	0.00	0.00	0.00	0.00	0.00	0.00	0.00	0.00
0.00	0.00	0.00	0.00	0.00	0.00	0.00	0.00	0.00
0.00	0.00	0.00	0.00	0.00	0.00	0.00	0.00	0.00
0.00	0.00	0.00	0.00	0.00	0.00	0.00	0.00	0.00
0.00	0.00	0.00	0.00	0.00	0.00	0.00	0.00	0.00
0.00	0.00	0.00	0.00	0.00	0.00	0.00	0.00	0.00
0.00	0.00	0.00	0.00	0.00	0.00	0.00	0.00	0.00

0.00	0.00	0.00	0.00	0.00	0.00	0.00	0.00	0.00
0.00	0.00	0.00	0.00	0.00	0.00	0.00	0.00	0.00
0.00	0.00	0.00	0.00	0.00	0.00	0.00	0.00	0.00
0.00	0.00	0.00	0.00	0.00	0.00	0.00	0.00	0.00
0.00	0.00	0.00	0.00	0.00	0.00	0.00	0.00	0.00
0.00	0.00	0.00	0.00	0.00	0.00	0.00	0.00	0.00
0.00	0.00	0.00	2.00	0.00	0.00	0.00	0.00	0.00
0.00	0.00	0.00	0.00	0.00	0.00	0.00	0.00	0.00
0.00	0.00	0.00	0.00	0.00	0.00	0.00	0.00	0.00
0.00	0.00	0.00	0.00	0.00	0.00	0.00	0.00	0.00
0.00	0.00	0.00	0.00	0.00	0.00	0.00	0.00	0.00
0.00	0.00	0.00	0.00	0.00	0.00	0.00	0.00	0.00
0.00	0.00	0.00	0.00	0.00	0.00	0.00	0.00	0.00
0.00	0.00	0.00	0.00	0.00	0.00	0.00	0.00	0.00
0.00	0.00	0.00	0.00	0.00	0.00	0.00	0.00	0.00
0.00	0.00	0.00	0.00	0.00	0.00	0.00	0.00	0.00
0.00	0.00	0.00	0.00	0.00	0.00	0.00	0.00	0.00
0.00	0.00	0.00	0.00	0.00	0.00	0.00	0.00	0.00
0.00	0.00	0.00	0.00	0.00	0.00	0.00	0.00	0.00
0.00	0.00	0.00	0.00	0.00	0.00	0.00	0.00	0.00
0.00	0.00	0.00	0.00	0.00	0.00	0.00	0.00	0.00
0.00	0.00	0.00	0.00	0.00	0.00	0.00	0.00	0.00
0.00	0.00	0.00	0.00	0.00	0.00	0.00	0.00	0.00
0.00	0.00	0.00	0.00	0.00	0.00	0.00	0.00	0.00
0.00	0.00	0.00	0.00	0.00	0.00	0.00	0.00	0.00
0.00	0.00	0.00	0.00	0.00	0.00	0.00	0.00	0.00
0.00	0.00	0.00	0.00	0.00	0.00	0.00	0.00	0.00
0.00	0.00	0.00	0.00	0.00	0.00	0.00	0.00	0.00
0.00	0.00	0.00	0.00	0.00	0.00	0.00	0.00	0.00
0.00	0.00	0.00	0.00	0.00	0.00	0.00	0.00	0.00
0.00	0.00	0.00	0.00	0.00	0.00	0.00	0.00	0.00
0.00	0.00	0.00	0.00	0.00	0.00	0.00	0.00	0.00
0.00	0.00	0.00	0.00	0.00	0.00	0.00	0.00	0.00
0.00	0.00	0.00	0.00	0.00	0.00	0.00	0.00	0.00
0.00	0.00	0.00	0.00	0.00	0.00	0.00	0.00	0.00
0.00	0.00	0.00	0.00	0.00	0.00	0.00	0.00	0.00
0.00	0.00	0.00	0.00	0.00	0.00	0.00	0.00	0.00

0.00	0.00	0.00	0.00	0.00	0.00	0.00	0.00	0.00
0.00	0.00	0.00	0.00	0.00	0.00	0.00	0.00	0.00
0.00	0.00	0.00	0.00	0.00	0.00	0.00	0.00	0.00
0.00	0.00	0.00	0.00	0.00	0.00	0.00	0.00	0.00
0.00	0.00	0.00	0.00	0.00	0.00	0.00	0.00	0.00
0.00	0.00	0.00	0.00	0.00	0.00	0.00	0.00	0.00
0.00	0.00	0.00	0.00	0.00	0.00	0.00	0.00	0.00
0.00	0.00	0.00	0.00	0.00	0.00	0.00	0.00	0.00
0.00	0.00	0.00	0.00	0.00	0.00	0.00	0.00	0.00
0.00	0.00	0.00	0.00	0.00	0.00	0.00	0.00	0.00
0.00	0.00	0.00	0.00	0.00	0.00	0.00	0.00	0.00
0.00	0.00	0.00	0.00	0.00	0.00	0.00	0.00	0.00
0.00	0.00	0.00	0.00	0.00	0.00	0.00	0.00	0.00
0.00	0.00	0.00	0.00	0.00	0.00	0.00	0.00	0.00
0.00	0.00	0.00	0.00	0.00	0.00	0.00	0.00	0.00
0.00	0.00	0.00	0.00	0.00	0.00	0.00	0.00	0.00
0.00	0.00	0.00	0.00	0.00	0.00	0.00	0.00	0.00
0.00	0.00	0.00	0.00	0.00	0.00	0.00	0.00	0.00
0.00	0.00	0.00	0.00	0.00	0.00	0.00	0.00	0.00
0.00	0.00	0.00	0.00	0.00	0.00	0.00	0.00	0.00
0.00	0.00	0.00	0.00	0.00	0.00	0.00	0.00	0.00
0.00	0.00	0.00	0.00	0.00	0.00	0.00	0.00	0.00
0.00	0.00	0.00	0.00	0.00	0.00	0.00	0.00	0.00
0.00	0.00	0.00	0.00	0.00	0.00	0.00	0.00	0.00
0.00	0.00	0.00	0.00	0.00	0.00	0.00	0.00	0.00
0.00	0.00	0.00	0.00	0.00	0.00	0.00	0.00	0.00
0.00	0.00	0.00	0.00	0.00	0.00	0.00	0.00	0.00
0.00	0.00	0.00	0.00	0.00	0.00	0.00	0.00	0.00
0.00	0.00	0.00	0.00	0.00	0.00	0.00	0.00	0.00
0.00	0.00	0.00	0.00	0.00	0.00	0.00	0.00	0.00
0.00	0.00	0.00	0.00	0.00	0.00	0.00	0.00	0.00
0.00	0.00	0.00	0.00	0.00	0.00	0.00	0.00	0.00
0.00	0.00	0.00	0.00	0.00	0.00	0.00	0.00	0.00
0.00	0.00	0.00	0.00	0.00	0.00	0.00	0.00	0.00
0.00	0.00	0.00	0.00	0.00	0.00	0.00	0.00	0.00
0.00	0.00	0.00	0.00	0.00	0.00	0.00	0.00	0.00
0.00	0.00	0.00	0.00	0.00	0.00	0.00	0.00	0.00

0.00	0.00	0.00	0.00	0.00	0.00	0.00	0.00	0.00
0.00	0.00	0.00	0.00	0.00	0.00	0.00	0.00	0.00
0.00	0.00	0.00	0.00	0.00	0.00	0.00	0.00	0.00
0.00	0.00	0.00	0.00	0.00	0.00	0.00	0.00	0.00
0.00	0.00	0.00	0.00	0.00	0.00	0.00	0.00	0.00
0.00	0.00	0.00	0.00	0.00	0.00	0.00	0.00	0.00
0.00	0.00	0.00	0.00	0.00	0.00	0.00	0.00	0.00
0.00	0.00	0.00	0.00	0.00	0.00	0.00	0.00	0.00
0.00	0.00	0.00	0.00	0.00	0.00	0.00	0.00	0.00
0.00	0.00	0.00	0.00	0.00	0.00	0.00	0.00	0.00
0.00	0.00	0.00	0.00	0.00	0.00	0.00	0.00	0.00
0.00	0.00	0.00	0.00	0.00	0.00	0.00	0.00	0.00
0.00	0.00	0.00	0.00	0.00	0.00	0.00	0.00	0.00
0.00	0.00	0.00	0.00	0.00	0.00	0.00	0.00	0.00
0.00	0.00	0.00	0.00	0.00	0.00	0.00	0.00	0.00
0.00	0.00	0.00	0.00	0.00	0.00	0.00	0.00	0.00
0.00	0.00	0.00	0.00	0.00	0.00	0.00	0.00	0.00
0.00	0.00	0.00	0.00	0.00	0.00	0.00	0.00	0.00
0.00	0.00	0.00	0.00	0.00	0.00	0.00	0.00	0.00
0.00	0.00	0.00	0.00	0.00	0.00	0.00	0.00	0.00
0.00	0.00	0.00	0.00	0.00	0.00	0.00	0.00	0.00
0.00	0.00	0.00	0.00	0.00	0.00	0.00	0.00	0.00
0.00	0.00	0.00	0.00	0.00	0.00	0.00	0.00	0.00
0.00	0.00	0.00	0.00	0.00	0.00	0.00	0.00	0.00
0.00	0.00	0.00	0.00	0.00	0.00	0.00	0.00	0.00
0.00	0.00	0.00	0.00	0.00	0.00	0.00	0.00	0.00
0.00	0.00	0.00	0.00	0.00	0.00	0.00	0.00	0.00
0.00	0.00	0.00	0.00	0.00	0.00	0.00	0.00	0.00
0.00	0.00	0.00	0.00	0.00	0.00	0.00	0.00	0.00
0.00	0.00	0.00	0.00	0.00	0.00	0.00	0.00	0.00
0.00	0.00	0.00	0.00	0.00	0.00	0.00	0.00	0.00
0.00	0.00	0.00	0.00	0.00	0.00	0.00	0.00	0.00
0.00	0.00	0.00	0.00	0.00	0.00	0.00	0.00	0.00

A13	A14	B13	B14	B15	A15	A16	A17	A18
506.00	516.00	0.00	0.00	4058.00	806.00	84.00	11.00	0.00
22014.00	11552.00	15707.00	18223.00	552.00	12771.00	12350.00	24634.00	16452.00
5.00	6.00	19.00	0.00	0.00	0.00	6.00	34.00	0.00
8081.00	6403.00	6810.00	7363.00	307.00	3538.00	3903.00	8466.00	5356.00
5340.00	2926.00	4301.00	4916.00	176.00	2714.00	2847.00	6222.00	3955.00
0.00	0.00	0.00	35.00	0.00	4609.00	9991.00	0.00	0.00
0.00	0.00	0.00	0.00	0.00	0.00	361.00	0.00	0.00
5406.00	3572.00	2845.00	3458.00	134.00	2100.00	2158.00	4785.00	3510.00
139.00	2913.00	0.00	6.00	0.00	0.00	0.00	0.00	0.00
3738.00	2519.00	2369.00	3056.00	176.00	1143.00	1309.00	3686.00	1829.00
0.00	0.00	0.00	0.00	0.00	0.00	224.00	0.00	0.00
0.00	11.00	0.00	0.00	104.00	19.00	0.00	192.00	0.00
0.00	1191.00	0.00	0.00	0.00	0.00	0.00	0.00	0.00
1671.00	654.00	1149.00	1452.00	62.00	759.00	828.00	1789.00	1141.00
1690.00	1149.00	1195.00	1325.00	58.00	677.00	990.00	2058.00	1133.00
1586.00	983.00	989.00	1282.00	55.00	767.00	891.00	1033.00	1051.00
0.00	0.00	0.00	0.00	0.00	0.00	174.00	0.00	0.00
1812.00	619.00	527.00	1187.00	15.00	1136.00	971.00	1144.00	754.00
8.00	0.00	0.00	3.00	2796.00	212.00	36.00	16.00	0.00
0.00	0.00	0.00	0.00	0.00	0.00	0.00	0.00	0.00
0.00	0.00	0.00	0.00	0.00	0.00	0.00	0.00	0.00
0.00	0.00	0.00	0.00	0.00	0.00	0.00	0.00	0.00
0.00	0.00	0.00	0.00	0.00	0.00	0.00	0.00	0.00
1121.00	526.00	578.00	744.00	28.00	402.00	329.00	1230.00	607.00
0.00	0.00	0.00	0.00	0.00	0.00	0.00	0.00	0.00
0.00	0.00	0.00	0.00	0.00	0.00	0.00	0.00	0.00
0.00	0.00	0.00	0.00	0.00	0.00	0.00	0.00	0.00
859.00	557.00	624.00	916.00	53.00	653.00	511.00	432.00	107.00
0.00	0.00	0.00	0.00	0.00	0.00	0.00	0.00	0.00
0.00	0.00	0.00	0.00	0.00	0.00	0.00	0.00	0.00
0.00	0.00	0.00	0.00	0.00	0.00	0.00	0.00	0.00
0.00	0.00	0.00	0.00	0.00	0.00	0.00	0.00	0.00
0.00	0.00	0.00	0.00	0.00	0.00	0.00	0.00	0.00
0.00	0.00	0.00	0.00	0.00	0.00	0.00	0.00	0.00
5.00	0.00	0.00	3.00	10267.00	426.00	144.00	106.00	0.00

0.00	0.00	0.00	0.00	0.00	0.00	0.00	138.00	0.00
0.00	0.00	0.00	0.00	0.00	10.00	0.00	0.00	0.00
0.00	0.00	0.00	0.00	0.00	0.00	0.00	0.00	0.00
0.00	0.00	0.00	0.00	0.00	0.00	0.00	0.00	0.00
0.00	0.00	8.00	0.00	0.00	0.00	0.00	0.00	3.00
0.00	0.00	0.00	0.00	8596.00	263.00	40.00	0.00	0.00
0.00	0.00	0.00	0.00	0.00	0.00	0.00	0.00	0.00
0.00	0.00	0.00	0.00	0.00	0.00	0.00	0.00	0.00
0.00	0.00	0.00	0.00	0.00	0.00	0.00	0.00	0.00
0.00	0.00	0.00	0.00	6156.00	397.00	105.00	0.00	0.00
0.00	0.00	0.00	0.00	0.00	0.00	0.00	0.00	0.00
189.00	96.00	81.00	106.00	0.00	40.00	56.00	197.00	86.00
0.00	0.00	0.00	0.00	0.00	0.00	0.00	0.00	0.00
0.00	0.00	0.00	0.00	0.00	0.00	0.00	0.00	0.00
0.00	0.00	0.00	0.00	0.00	0.00	0.00	0.00	0.00
0.00	0.00	0.00	0.00	0.00	0.00	0.00	0.00	0.00
240.00	74.00	178.00	92.00	10.00	111.00	66.00	275.00	96.00
0.00	0.00	0.00	0.00	0.00	0.00	0.00	0.00	0.00
84.00	109.00	53.00	111.00	0.00	92.00	106.00	253.00	195.00
162.00	69.00	97.00	163.00	14.00	98.00	66.00	7.00	107.00
0.00	0.00	0.00	0.00	0.00	0.00	0.00	7.00	11.00
0.00	0.00	0.00	18.00	0.00	0.00	0.00	21.00	9.00
0.00	0.00	0.00	0.00	0.00	0.00	0.00	0.00	0.00
0.00	0.00	0.00	0.00	0.00	0.00	0.00	0.00	0.00
0.00	0.00	0.00	0.00	0.00	0.00	0.00	68.00	25.00
0.00	0.00	0.00	0.00	3956.00	161.00	0.00	0.00	0.00
0.00	0.00	0.00	0.00	0.00	0.00	0.00	0.00	0.00
0.00	0.00	0.00	0.00	3916.00	123.00	74.00	0.00	0.00
158.00	118.00	170.00	226.00	10.00	78.00	146.00	0.00	0.00
133.00	41.00	75.00	101.00	0.00	67.00	74.00	96.00	121.00
0.00	0.00	0.00	0.00	0.00	0.00	0.00	0.00	0.00
0.00	0.00	0.00	0.00	0.00	0.00	0.00	0.00	0.00
0.00	0.00	0.00	0.00	0.00	0.00	0.00	0.00	0.00
218.00	147.00	46.00	206.00	0.00	91.00	98.00	77.00	0.00
0.00	0.00	0.00	0.00	0.00	0.00	0.00	0.00	0.00
0.00	0.00	0.00	0.00	0.00	0.00	0.00	0.00	0.00
0.00	0.00	0.00	0.00	0.00	0.00	0.00	0.00	0.00

0.00	0.00	0.00	0.00	0.00	0.00	0.00	0.00	0.00
0.00	0.00	0.00	0.00	0.00	24.00	0.00	563.00	0.00
0.00	0.00	0.00	0.00	2891.00	136.00	0.00	0.00	0.00
0.00	0.00	0.00	0.00	0.00	0.00	0.00	0.00	0.00
0.00	0.00	0.00	0.00	5.00	0.00	0.00	0.00	0.00
0.00	0.00	0.00	0.00	0.00	0.00	0.00	0.00	0.00
0.00	0.00	0.00	0.00	0.00	0.00	0.00	0.00	0.00
0.00	0.00	0.00	0.00	0.00	0.00	0.00	0.00	0.00
0.00	0.00	0.00	0.00	0.00	0.00	0.00	0.00	0.00
0.00	0.00	0.00	0.00	0.00	0.00	0.00	0.00	0.00
0.00	0.00	0.00	0.00	0.00	0.00	0.00	0.00	0.00
111.00	77.00	99.00	48.00	6.00	0.00	0.00	59.00	0.00
0.00	15.00	0.00	5.00	0.00	0.00	0.00	0.00	0.00
0.00	0.00	0.00	4.00	0.00	11.00	7.00	0.00	0.00
0.00	0.00	0.00	0.00	0.00	0.00	0.00	0.00	0.00
0.00	0.00	0.00	0.00	0.00	0.00	0.00	0.00	0.00
115.00	82.00	53.00	80.00	4.00	27.00	43.00	51.00	58.00
115.00	55.00	57.00	53.00	0.00	0.00	34.00	48.00	67.00
0.00	0.00	0.00	0.00	0.00	0.00	0.00	0.00	0.00
0.00	0.00	0.00	0.00	0.00	0.00	0.00	0.00	0.00
0.00	0.00	0.00	0.00	0.00	0.00	0.00	0.00	0.00
0.00	0.00	0.00	0.00	0.00	0.00	0.00	0.00	0.00
0.00	0.00	0.00	0.00	0.00	0.00	0.00	0.00	0.00
0.00	0.00	0.00	0.00	0.00	0.00	0.00	0.00	0.00
0.00	0.00	0.00	0.00	0.00	0.00	0.00	0.00	0.00
0.00	0.00	0.00	0.00	0.00	0.00	9.00	0.00	0.00
0.00	0.00	0.00	0.00	0.00	0.00	0.00	0.00	0.00
0.00	0.00	19.00	26.00	0.00	0.00	0.00	104.00	8.00
0.00	0.00	0.00	0.00	0.00	0.00	0.00	0.00	0.00
0.00	0.00	0.00	0.00	0.00	0.00	0.00	145.00	0.00
33.00	18.00	31.00	30.00	0.00	45.00	23.00	95.00	89.00
0.00	0.00	0.00	0.00	0.00	0.00	0.00	25.00	5.00
0.00	0.00	0.00	0.00	0.00	0.00	0.00	0.00	0.00
0.00	0.00	0.00	0.00	0.00	0.00	0.00	0.00	0.00
62.00	0.00	0.00	70.00	0.00	70.00	0.00	207.00	0.00
0.00	0.00	0.00	0.00	0.00	0.00	0.00	0.00	0.00
0.00	0.00	0.00	0.00	0.00	0.00	0.00	0.00	0.00

0.00	0.00	0.00	0.00	0.00	0.00	0.00	0.00	0.00
0.00	0.00	0.00	0.00	0.00	0.00	0.00	0.00	0.00
0.00	0.00	0.00	0.00	0.00	0.00	0.00	0.00	0.00
0.00	0.00	0.00	0.00	0.00	0.00	0.00	0.00	0.00
0.00	0.00	0.00	0.00	0.00	0.00	0.00	0.00	0.00
0.00	0.00	0.00	0.00	0.00	0.00	0.00	0.00	0.00
0.00	0.00	0.00	0.00	0.00	0.00	0.00	0.00	0.00
0.00	0.00	0.00	0.00	0.00	0.00	0.00	0.00	0.00
0.00	0.00	0.00	0.00	0.00	0.00	0.00	0.00	0.00
0.00	0.00	0.00	0.00	0.00	0.00	0.00	0.00	0.00
0.00	0.00	0.00	0.00	0.00	0.00	0.00	0.00	0.00
0.00	0.00	0.00	0.00	0.00	0.00	0.00	29.00	0.00
0.00	0.00	0.00	0.00	0.00	0.00	0.00	0.00	0.00
0.00	0.00	0.00	0.00	0.00	0.00	0.00	0.00	0.00
4.00	13.00	25.00	54.00	0.00	29.00	26.00	86.00	0.00
0.00	0.00	0.00	0.00	0.00	0.00	0.00	1356.00	0.00
0.00	0.00	0.00	0.00	0.00	0.00	0.00	0.00	0.00
0.00	0.00	0.00	0.00	0.00	0.00	0.00	0.00	0.00
0.00	0.00	0.00	0.00	0.00	0.00	0.00	0.00	0.00
0.00	0.00	0.00	0.00	0.00	0.00	0.00	0.00	0.00
0.00	0.00	0.00	0.00	0.00	0.00	0.00	0.00	0.00
0.00	0.00	0.00	0.00	0.00	0.00	0.00	0.00	0.00
0.00	0.00	0.00	0.00	0.00	0.00	0.00	0.00	0.00
0.00	0.00	0.00	0.00	0.00	0.00	0.00	0.00	0.00
0.00	0.00	0.00	0.00	0.00	0.00	0.00	0.00	0.00
0.00	0.00	0.00	0.00	0.00	0.00	0.00	0.00	0.00
0.00	0.00	0.00	0.00	0.00	0.00	0.00	0.00	0.00
0.00	0.00	0.00	0.00	0.00	0.00	0.00	0.00	0.00
0.00	0.00	0.00	0.00	0.00	0.00	0.00	0.00	0.00
0.00	0.00	0.00	0.00	0.00	0.00	0.00	0.00	0.00
13.00	0.00	0.00	0.00	0.00	20.00	31.00	0.00	0.00
0.00	0.00	0.00	0.00	0.00	0.00	0.00	0.00	0.00
0.00	0.00	0.00	0.00	0.00	0.00	0.00	0.00	0.00
133.00	0.00	0.00	83.00	0.00	52.00	0.00	0.00	0.00
0.00	0.00	0.00	0.00	169.00	0.00	0.00	0.00	0.00
0.00	0.00	0.00	0.00	0.00	0.00	0.00	0.00	0.00
0.00	0.00	0.00	0.00	0.00	0.00	0.00	0.00	0.00

0.00	0.00	0.00	0.00	0.00	0.00	0.00	0.00	0.00
0.00	0.00	0.00	0.00	0.00	0.00	0.00	0.00	0.00
0.00	0.00	0.00	0.00	0.00	0.00	0.00	0.00	0.00
0.00	0.00	0.00	0.00	0.00	0.00	0.00	0.00	0.00
0.00	0.00	0.00	0.00	0.00	0.00	0.00	0.00	0.00
0.00	0.00	0.00	0.00	0.00	0.00	0.00	0.00	0.00
0.00	0.00	0.00	0.00	0.00	0.00	0.00	0.00	0.00
30.00	21.00	14.00	41.00	0.00	17.00	23.00	66.00	23.00
0.00	0.00	0.00	0.00	0.00	0.00	0.00	0.00	0.00
0.00	0.00	0.00	0.00	0.00	0.00	0.00	0.00	0.00
0.00	0.00	0.00	0.00	0.00	0.00	0.00	0.00	0.00
0.00	0.00	0.00	0.00	0.00	0.00	0.00	6.00	0.00
0.00	0.00	0.00	0.00	0.00	0.00	0.00	0.00	0.00
0.00	0.00	0.00	0.00	0.00	0.00	0.00	0.00	0.00
53.00	22.00	14.00	32.00	0.00	42.00	13.00	0.00	9.00
0.00	0.00	0.00	0.00	0.00	0.00	0.00	0.00	0.00
0.00	0.00	0.00	0.00	0.00	0.00	0.00	0.00	0.00
0.00	0.00	0.00	0.00	0.00	0.00	0.00	0.00	0.00
0.00	0.00	0.00	0.00	0.00	0.00	0.00	0.00	0.00
79.00	0.00	3.00	0.00	0.00	2.00	0.00	0.00	0.00
0.00	0.00	0.00	0.00	0.00	0.00	0.00	0.00	0.00
0.00	0.00	0.00	0.00	0.00	0.00	0.00	0.00	0.00
0.00	0.00	0.00	0.00	293.00	0.00	0.00	0.00	0.00
0.00	0.00	0.00	0.00	0.00	0.00	0.00	0.00	0.00
0.00	0.00	0.00	0.00	0.00	0.00	0.00	0.00	0.00
0.00	0.00	0.00	0.00	0.00	0.00	0.00	0.00	0.00
0.00	0.00	0.00	0.00	0.00	0.00	0.00	0.00	0.00
12.00	0.00	21.00	0.00	0.00	0.00	0.00	64.00	0.00
0.00	0.00	0.00	0.00	0.00	0.00	0.00	0.00	0.00
0.00	0.00	0.00	0.00	0.00	0.00	0.00	0.00	0.00
6.00	0.00	0.00	0.00	0.00	0.00	0.00	0.00	0.00
0.00	0.00	0.00	0.00	0.00	0.00	0.00	0.00	0.00
0.00	0.00	0.00	0.00	0.00	0.00	0.00	0.00	0.00
0.00	0.00	0.00	0.00	0.00	0.00	0.00	0.00	0.00
0.00	0.00	0.00	0.00	0.00	0.00	0.00	0.00	0.00
0.00	0.00	0.00	0.00	0.00	0.00	0.00	0.00	0.00
0.00	0.00	0.00	0.00	0.00	0.00	0.00	0.00	0.00

0.00	3.00	0.00	0.00	0.00	0.00	0.00	0.00	0.00
96.00	0.00	90.00	8.00	0.00	10.00	14.00	0.00	35.00
18.00	0.00	0.00	0.00	0.00	34.00	0.00	27.00	0.00
0.00	0.00	0.00	0.00	0.00	0.00	0.00	0.00	0.00
0.00	0.00	0.00	0.00	0.00	0.00	0.00	0.00	0.00
0.00	0.00	0.00	0.00	0.00	0.00	0.00	40.00	0.00
0.00	0.00	0.00	0.00	0.00	0.00	0.00	0.00	0.00
0.00	0.00	0.00	0.00	0.00	0.00	0.00	0.00	0.00
0.00	0.00	0.00	0.00	0.00	0.00	0.00	0.00	0.00
0.00	0.00	0.00	0.00	0.00	0.00	0.00	0.00	0.00
114.00	0.00	0.00	0.00	0.00	0.00	0.00	0.00	0.00
0.00	0.00	0.00	0.00	0.00	0.00	0.00	0.00	0.00
0.00	0.00	0.00	0.00	0.00	0.00	0.00	0.00	0.00
0.00	0.00	0.00	0.00	0.00	0.00	0.00	0.00	0.00
0.00	0.00	0.00	0.00	0.00	0.00	0.00	0.00	0.00
0.00	0.00	0.00	0.00	0.00	0.00	0.00	0.00	0.00
26.00	0.00	14.00	33.00	0.00	39.00	0.00	0.00	0.00
0.00	0.00	0.00	0.00	0.00	0.00	0.00	0.00	0.00
0.00	0.00	0.00	0.00	0.00	0.00	0.00	0.00	0.00
0.00	0.00	0.00	0.00	0.00	0.00	0.00	0.00	0.00
0.00	0.00	0.00	0.00	0.00	0.00	0.00	0.00	0.00
0.00	0.00	0.00	0.00	0.00	0.00	0.00	0.00	0.00
35.00	0.00	21.00	19.00	0.00	30.00	15.00	0.00	0.00
0.00	0.00	0.00	0.00	0.00	0.00	0.00	0.00	0.00
0.00	0.00	0.00	0.00	0.00	0.00	0.00	0.00	0.00
0.00	22.00	0.00	0.00	0.00	0.00	3.00	0.00	14.00
0.00	0.00	0.00	0.00	0.00	0.00	0.00	0.00	0.00
0.00	0.00	0.00	0.00	0.00	0.00	0.00	0.00	0.00
69.00	0.00	0.00	65.00	0.00	0.00	52.00	0.00	0.00
4.00	0.00	0.00	0.00	0.00	0.00	0.00	0.00	0.00
0.00	0.00	0.00	0.00	0.00	0.00	0.00	0.00	0.00
0.00	0.00	0.00	0.00	0.00	0.00	0.00	0.00	0.00
0.00	0.00	0.00	0.00	0.00	0.00	0.00	0.00	0.00
0.00	0.00	0.00	0.00	0.00	0.00	0.00	0.00	0.00
0.00	0.00	0.00	0.00	0.00	0.00	0.00	0.00	0.00
0.00	0.00	0.00	0.00	0.00	0.00	0.00	0.00	0.00
0.00	0.00	0.00	0.00	0.00	0.00	0.00	0.00	0.00

0.00	0.00	0.00	0.00	0.00	0.00	0.00	0.00	0.00
0.00	0.00	0.00	0.00	0.00	0.00	0.00	0.00	0.00
0.00	0.00	0.00	0.00	0.00	0.00	0.00	0.00	0.00
0.00	0.00	0.00	0.00	0.00	0.00	0.00	0.00	0.00
0.00	0.00	0.00	0.00	0.00	0.00	0.00	0.00	0.00
0.00	0.00	0.00	0.00	0.00	0.00	0.00	0.00	0.00
40.00	0.00	0.00	0.00	0.00	0.00	0.00	39.00	0.00
0.00	0.00	0.00	0.00	0.00	0.00	0.00	0.00	0.00
0.00	0.00	0.00	0.00	0.00	0.00	0.00	0.00	0.00
0.00	0.00	0.00	0.00	0.00	0.00	0.00	0.00	0.00
0.00	0.00	0.00	0.00	0.00	0.00	0.00	280.00	0.00
0.00	0.00	0.00	0.00	0.00	0.00	0.00	0.00	0.00
0.00	0.00	0.00	0.00	0.00	0.00	0.00	0.00	0.00
0.00	0.00	0.00	0.00	0.00	0.00	0.00	0.00	0.00
0.00	0.00	0.00	0.00	0.00	0.00	0.00	0.00	0.00
0.00	0.00	0.00	0.00	0.00	0.00	0.00	0.00	0.00
0.00	0.00	0.00	0.00	0.00	0.00	0.00	491.00	0.00
0.00	0.00	0.00	0.00	0.00	0.00	0.00	0.00	0.00
0.00	0.00	0.00	0.00	0.00	0.00	0.00	0.00	0.00
0.00	0.00	0.00	0.00	0.00	0.00	0.00	0.00	0.00
0.00	0.00	0.00	0.00	0.00	0.00	0.00	0.00	0.00
0.00	0.00	0.00	0.00	0.00	0.00	0.00	0.00	0.00
0.00	0.00	0.00	0.00	0.00	0.00	0.00	0.00	0.00
0.00	0.00	0.00	0.00	0.00	0.00	0.00	0.00	0.00
0.00	0.00	0.00	0.00	0.00	0.00	0.00	0.00	0.00
0.00	0.00	0.00	0.00	0.00	0.00	0.00	0.00	0.00
0.00	0.00	0.00	0.00	0.00	0.00	0.00	0.00	0.00
0.00	0.00	0.00	0.00	0.00	0.00	0.00	0.00	0.00
0.00	0.00	0.00	0.00	0.00	0.00	0.00	0.00	0.00
0.00	0.00	0.00	0.00	0.00	0.00	0.00	0.00	0.00
0.00	0.00	0.00	0.00	0.00	0.00	0.00	0.00	0.00
0.00	0.00	0.00	0.00	0.00	0.00	0.00	0.00	0.00
0.00	0.00	0.00	0.00	0.00	0.00	0.00	0.00	0.00
0.00	0.00	0.00	0.00	0.00	0.00	0.00	0.00	0.00
0.00	41.00	0.00	0.00	0.00	0.00	0.00	0.00	0.00
0.00	0.00	0.00	0.00	0.00	0.00	0.00	0.00	0.00
0.00	0.00	0.00	0.00	0.00	0.00	0.00	0.00	0.00

0.00	15.00	58.00	25.00	0.00	11.00	12.00	0.00	15.00
0.00	0.00	0.00	0.00	0.00	0.00	0.00	0.00	0.00
0.00	0.00	0.00	0.00	0.00	0.00	0.00	0.00	0.00
0.00	0.00	0.00	0.00	0.00	0.00	0.00	0.00	0.00
0.00	0.00	0.00	0.00	0.00	0.00	0.00	0.00	0.00
0.00	0.00	0.00	0.00	0.00	0.00	0.00	0.00	0.00
0.00	0.00	0.00	0.00	0.00	0.00	0.00	0.00	0.00
0.00	0.00	0.00	0.00	0.00	0.00	0.00	0.00	0.00
0.00	0.00	0.00	0.00	0.00	0.00	0.00	0.00	0.00
0.00	0.00	0.00	0.00	0.00	14.00	0.00	0.00	0.00
0.00	0.00	0.00	0.00	0.00	0.00	0.00	0.00	0.00
0.00	0.00	0.00	0.00	0.00	0.00	0.00	0.00	0.00
0.00	0.00	0.00	0.00	0.00	0.00	0.00	0.00	0.00
0.00	0.00	0.00	23.00	0.00	75.00	0.00	0.00	0.00
0.00	0.00	0.00	0.00	0.00	0.00	0.00	0.00	0.00
0.00	23.00	25.00	37.00	0.00	18.00	20.00	0.00	0.00
0.00	0.00	0.00	0.00	0.00	0.00	0.00	0.00	0.00
0.00	0.00	0.00	0.00	0.00	0.00	0.00	0.00	0.00
0.00	0.00	0.00	0.00	0.00	0.00	0.00	0.00	0.00
0.00	0.00	0.00	0.00	0.00	0.00	0.00	0.00	0.00
0.00	0.00	0.00	0.00	0.00	0.00	0.00	0.00	0.00
0.00	0.00	0.00	0.00	0.00	0.00	0.00	0.00	0.00
0.00	0.00	0.00	0.00	0.00	0.00	0.00	0.00	0.00
40.00	0.00	0.00	0.00	0.00	0.00	50.00	0.00	49.00
0.00	0.00	0.00	0.00	0.00	0.00	0.00	0.00	0.00
0.00	0.00	0.00	0.00	0.00	0.00	0.00	0.00	0.00
0.00	0.00	0.00	0.00	0.00	0.00	0.00	0.00	0.00
0.00	0.00	0.00	0.00	0.00	0.00	0.00	0.00	0.00
0.00	0.00	0.00	0.00	0.00	0.00	0.00	0.00	0.00
0.00	0.00	0.00	0.00	0.00	0.00	0.00	0.00	0.00
0.00	0.00	0.00	0.00	0.00	0.00	0.00	0.00	0.00
0.00	0.00	0.00	0.00	0.00	0.00	0.00	0.00	0.00
0.00	0.00	0.00	0.00	0.00	0.00	0.00	0.00	0.00
0.00	0.00	21.00	0.00	0.00	0.00	0.00	0.00	0.00
0.00	0.00	0.00	0.00	0.00	0.00	0.00	0.00	0.00
0.00	0.00	0.00	14.00	0.00	0.00	0.00	0.00	0.00
10.00	4.00	13.00	24.00	0.00	3.00	4.00	0.00	19.00

0.00	0.00	0.00	0.00	0.00	0.00	0.00	0.00	0.00
0.00	0.00	0.00	0.00	0.00	0.00	0.00	0.00	0.00
0.00	0.00	0.00	0.00	0.00	0.00	0.00	0.00	0.00
0.00	0.00	0.00	0.00	0.00	0.00	0.00	50.00	0.00
0.00	0.00	0.00	0.00	0.00	0.00	0.00	0.00	0.00
0.00	0.00	0.00	0.00	0.00	0.00	0.00	0.00	0.00
0.00	0.00	0.00	0.00	0.00	0.00	0.00	0.00	0.00
0.00	0.00	0.00	0.00	0.00	0.00	0.00	134.00	0.00
0.00	0.00	0.00	0.00	0.00	0.00	0.00	0.00	0.00
0.00	0.00	0.00	0.00	0.00	0.00	0.00	0.00	0.00
0.00	0.00	0.00	0.00	0.00	0.00	0.00	0.00	0.00
0.00	0.00	0.00	0.00	0.00	0.00	0.00	0.00	0.00
0.00	0.00	0.00	0.00	0.00	0.00	0.00	0.00	0.00
0.00	0.00	0.00	0.00	0.00	0.00	0.00	0.00	0.00
0.00	0.00	0.00	0.00	0.00	0.00	0.00	0.00	0.00
0.00	0.00	0.00	0.00	0.00	0.00	0.00	0.00	0.00
0.00	0.00	0.00	0.00	0.00	0.00	0.00	0.00	0.00
0.00	0.00	0.00	0.00	0.00	0.00	0.00	0.00	0.00
0.00	0.00	0.00	0.00	0.00	0.00	0.00	0.00	0.00
0.00	0.00	0.00	0.00	0.00	0.00	0.00	0.00	0.00
0.00	0.00	0.00	0.00	0.00	0.00	0.00	0.00	0.00
0.00	0.00	0.00	0.00	0.00	0.00	0.00	0.00	0.00
0.00	0.00	0.00	0.00	0.00	0.00	0.00	0.00	0.00
0.00	0.00	0.00	0.00	0.00	0.00	0.00	0.00	0.00
0.00	0.00	0.00	0.00	0.00	0.00	0.00	0.00	0.00
0.00	0.00	0.00	0.00	0.00	0.00	0.00	0.00	0.00
0.00	0.00	0.00	0.00	0.00	0.00	0.00	0.00	0.00
0.00	0.00	0.00	0.00	0.00	0.00	0.00	0.00	0.00
0.00	0.00	0.00	0.00	0.00	0.00	0.00	0.00	3.00
0.00	0.00	0.00	0.00	0.00	0.00	0.00	0.00	0.00
0.00	0.00	0.00	0.00	0.00	0.00	0.00	0.00	0.00
0.00	0.00	0.00	0.00	0.00	0.00	0.00	0.00	0.00
0.00	0.00	0.00	0.00	0.00	0.00	0.00	0.00	0.00
0.00	0.00	0.00	0.00	0.00	0.00	0.00	0.00	0.00
0.00	27.00	0.00	0.00	0.00	0.00	0.00	0.00	0.00
0.00	0.00	0.00	0.00	0.00	0.00	0.00	0.00	0.00
0.00	0.00	0.00	0.00	0.00	0.00	0.00	0.00	0.00

0.00	0.00	0.00	0.00	0.00	0.00	0.00	0.00	0.00
0.00	0.00	0.00	0.00	290.00	0.00	0.00	0.00	0.00
0.00	0.00	0.00	0.00	0.00	0.00	0.00	0.00	0.00
0.00	0.00	0.00	0.00	0.00	0.00	0.00	0.00	0.00
0.00	0.00	0.00	0.00	0.00	0.00	0.00	0.00	0.00
0.00	0.00	0.00	0.00	0.00	0.00	0.00	0.00	0.00
0.00	0.00	0.00	0.00	0.00	0.00	0.00	0.00	0.00
0.00	0.00	0.00	0.00	0.00	0.00	0.00	0.00	0.00
0.00	0.00	0.00	0.00	0.00	0.00	0.00	0.00	0.00
0.00	0.00	0.00	0.00	0.00	0.00	0.00	0.00	0.00
0.00	0.00	0.00	0.00	0.00	0.00	0.00	0.00	0.00
0.00	0.00	0.00	0.00	0.00	0.00	0.00	0.00	0.00
0.00	0.00	0.00	0.00	0.00	0.00	0.00	0.00	0.00
0.00	0.00	0.00	0.00	0.00	0.00	0.00	0.00	0.00
0.00	0.00	0.00	0.00	0.00	0.00	0.00	0.00	0.00
0.00	0.00	0.00	0.00	0.00	0.00	0.00	0.00	0.00
0.00	0.00	0.00	0.00	0.00	0.00	0.00	0.00	0.00
0.00	19.00	24.00	0.00	0.00	0.00	46.00	0.00	0.00
0.00	0.00	0.00	0.00	0.00	0.00	0.00	0.00	0.00
0.00	0.00	0.00	0.00	0.00	0.00	0.00	0.00	0.00
0.00	0.00	0.00	0.00	0.00	0.00	0.00	0.00	0.00
0.00	41.00	0.00	0.00	0.00	0.00	0.00	0.00	0.00
0.00	0.00	0.00	0.00	0.00	0.00	0.00	0.00	0.00
0.00	0.00	0.00	0.00	0.00	0.00	0.00	0.00	0.00
0.00	0.00	0.00	0.00	0.00	0.00	0.00	0.00	0.00
0.00	0.00	0.00	0.00	0.00	0.00	0.00	0.00	0.00
0.00	0.00	0.00	77.00	0.00	0.00	0.00	0.00	0.00
0.00	0.00	0.00	0.00	0.00	0.00	0.00	0.00	0.00
0.00	0.00	0.00	0.00	0.00	0.00	0.00	0.00	0.00
38.00	0.00	0.00	0.00	0.00	12.00	0.00	0.00	0.00
0.00	0.00	0.00	0.00	0.00	0.00	0.00	0.00	0.00
0.00	0.00	0.00	0.00	0.00	0.00	0.00	0.00	0.00
0.00	0.00	0.00	0.00	0.00	0.00	0.00	241.00	0.00
0.00	0.00	0.00	0.00	0.00	0.00	0.00	0.00	0.00
0.00	0.00	0.00	0.00	0.00	0.00	0.00	0.00	0.00
23.00	0.00	0.00	16.00	0.00	0.00	0.00	0.00	0.00
0.00	0.00	0.00	0.00	0.00	0.00	0.00	0.00	0.00

0.00	0.00	0.00	0.00	0.00	0.00	0.00	0.00	13.00
0.00	0.00	0.00	6.00	0.00	0.00	5.00	0.00	0.00
14.00	13.00	0.00	23.00	0.00	10.00	0.00	0.00	0.00
0.00	0.00	0.00	0.00	0.00	0.00	0.00	0.00	0.00
0.00	0.00	0.00	0.00	0.00	0.00	0.00	0.00	0.00
0.00	0.00	0.00	0.00	0.00	0.00	0.00	0.00	0.00
0.00	0.00	0.00	0.00	0.00	0.00	0.00	0.00	0.00
0.00	0.00	0.00	0.00	0.00	0.00	0.00	0.00	0.00
0.00	0.00	0.00	0.00	0.00	0.00	0.00	0.00	0.00
0.00	0.00	0.00	0.00	0.00	0.00	0.00	0.00	0.00
0.00	0.00	0.00	0.00	0.00	0.00	0.00	0.00	0.00
0.00	0.00	0.00	0.00	0.00	0.00	0.00	0.00	0.00
0.00	0.00	0.00	0.00	0.00	0.00	0.00	0.00	0.00
0.00	0.00	0.00	0.00	0.00	0.00	0.00	0.00	0.00
0.00	0.00	0.00	0.00	0.00	0.00	0.00	0.00	0.00
0.00	0.00	0.00	0.00	0.00	0.00	0.00	0.00	0.00
0.00	0.00	0.00	0.00	0.00	0.00	0.00	0.00	0.00
0.00	0.00	0.00	0.00	0.00	0.00	0.00	0.00	0.00
0.00	0.00	0.00	0.00	0.00	0.00	0.00	0.00	0.00
0.00	0.00	0.00	0.00	0.00	0.00	0.00	0.00	0.00
0.00	0.00	0.00	0.00	0.00	0.00	0.00	0.00	0.00
0.00	0.00	0.00	0.00	0.00	0.00	0.00	0.00	0.00
0.00	0.00	0.00	0.00	0.00	0.00	0.00	0.00	0.00
0.00	0.00	16.00	50.00	0.00	0.00	0.00	0.00	0.00
0.00	0.00	0.00	0.00	0.00	0.00	0.00	0.00	0.00
0.00	0.00	0.00	0.00	0.00	0.00	0.00	0.00	6.00
0.00	0.00	0.00	0.00	0.00	0.00	0.00	0.00	0.00
0.00	0.00	0.00	0.00	0.00	0.00	0.00	0.00	0.00
0.00	0.00	0.00	0.00	0.00	0.00	0.00	0.00	0.00
0.00	0.00	0.00	0.00	0.00	0.00	0.00	0.00	0.00
0.00	0.00	0.00	9.00	0.00	0.00	2.00	0.00	0.00
0.00	0.00	0.00	0.00	0.00	0.00	0.00	0.00	0.00
0.00	0.00	0.00	0.00	0.00	0.00	0.00	0.00	0.00
0.00	0.00	0.00	0.00	0.00	0.00	0.00	0.00	0.00
0.00	0.00	0.00	0.00	0.00	0.00	0.00	0.00	0.00
0.00	0.00	0.00	0.00	0.00	0.00	0.00	0.00	0.00
0.00	0.00	0.00	0.00	0.00	0.00	0.00	0.00	0.00

0.00	38.00	0.00	0.00	0.00	0.00	0.00	0.00	0.00
0.00	0.00	0.00	0.00	0.00	0.00	0.00	0.00	0.00
0.00	0.00	0.00	0.00	0.00	0.00	0.00	0.00	0.00
0.00	2.00	0.00	13.00	0.00	0.00	26.00	0.00	7.00
0.00	0.00	0.00	0.00	0.00	0.00	0.00	0.00	0.00
0.00	0.00	0.00	0.00	0.00	0.00	0.00	0.00	0.00
0.00	0.00	0.00	0.00	0.00	0.00	0.00	0.00	0.00
0.00	0.00	0.00	0.00	0.00	0.00	0.00	0.00	0.00
0.00	0.00	0.00	0.00	0.00	0.00	0.00	0.00	0.00
0.00	0.00	0.00	0.00	0.00	0.00	0.00	0.00	0.00
0.00	0.00	0.00	0.00	0.00	0.00	0.00	0.00	0.00
0.00	0.00	0.00	0.00	0.00	0.00	0.00	0.00	0.00
0.00	0.00	0.00	0.00	0.00	0.00	0.00	0.00	0.00
0.00	0.00	0.00	0.00	0.00	0.00	0.00	0.00	0.00
0.00	0.00	0.00	0.00	0.00	0.00	0.00	191.00	0.00
0.00	0.00	0.00	0.00	0.00	0.00	0.00	0.00	0.00
0.00	0.00	0.00	0.00	6.00	0.00	0.00	0.00	0.00
0.00	0.00	0.00	0.00	0.00	0.00	0.00	0.00	0.00
0.00	0.00	0.00	0.00	0.00	0.00	0.00	0.00	0.00
0.00	0.00	0.00	0.00	0.00	0.00	0.00	0.00	0.00
0.00	0.00	0.00	0.00	0.00	0.00	0.00	0.00	0.00
0.00	0.00	0.00	0.00	0.00	0.00	0.00	0.00	0.00
0.00	0.00	0.00	0.00	0.00	0.00	0.00	0.00	0.00
0.00	0.00	0.00	0.00	0.00	0.00	0.00	0.00	0.00
0.00	0.00	0.00	0.00	0.00	0.00	0.00	0.00	0.00
0.00	0.00	0.00	0.00	179.00	0.00	0.00	0.00	0.00
0.00	0.00	0.00	0.00	0.00	0.00	0.00	0.00	0.00
0.00	40.00	0.00	0.00	0.00	0.00	0.00	0.00	0.00
0.00	0.00	0.00	0.00	0.00	0.00	0.00	0.00	0.00
14.00	0.00	0.00	0.00	0.00	0.00	0.00	0.00	0.00
0.00	0.00	0.00	0.00	0.00	0.00	0.00	0.00	0.00
0.00	0.00	0.00	0.00	0.00	0.00	0.00	0.00	0.00
0.00	0.00	0.00	0.00	0.00	0.00	0.00	0.00	0.00
0.00	0.00	0.00	0.00	0.00	0.00	0.00	0.00	0.00
0.00	0.00	0.00	0.00	0.00	0.00	0.00	0.00	0.00
0.00	0.00	0.00	0.00	0.00	0.00	0.00	0.00	0.00
0.00	0.00	0.00	0.00	0.00	0.00	0.00	0.00	0.00

0.00	0.00	0.00	0.00	0.00	0.00	0.00	0.00	0.00
0.00	0.00	0.00	0.00	0.00	0.00	0.00	0.00	0.00
0.00	0.00	0.00	0.00	0.00	0.00	0.00	0.00	0.00
0.00	0.00	0.00	0.00	0.00	0.00	0.00	0.00	0.00
0.00	0.00	0.00	0.00	0.00	0.00	0.00	0.00	0.00
0.00	0.00	0.00	0.00	0.00	0.00	0.00	0.00	0.00
0.00	0.00	0.00	0.00	0.00	0.00	0.00	0.00	0.00
0.00	0.00	0.00	0.00	0.00	0.00	0.00	0.00	0.00
0.00	0.00	0.00	0.00	0.00	0.00	0.00	0.00	0.00
0.00	0.00	0.00	0.00	0.00	0.00	0.00	0.00	0.00
0.00	0.00	0.00	0.00	0.00	0.00	0.00	0.00	0.00
0.00	0.00	0.00	0.00	0.00	0.00	0.00	0.00	0.00
0.00	0.00	0.00	0.00	0.00	0.00	0.00	0.00	0.00
0.00	0.00	0.00	0.00	0.00	0.00	0.00	0.00	0.00
0.00	0.00	0.00	0.00	0.00	0.00	0.00	0.00	0.00
0.00	0.00	0.00	0.00	0.00	0.00	0.00	0.00	0.00
0.00	0.00	0.00	0.00	0.00	0.00	0.00	0.00	0.00
0.00	0.00	0.00	0.00	0.00	0.00	0.00	0.00	0.00
0.00	0.00	0.00	0.00	0.00	0.00	0.00	0.00	0.00
0.00	0.00	0.00	0.00	0.00	0.00	0.00	0.00	0.00
0.00	0.00	0.00	0.00	0.00	0.00	0.00	0.00	0.00
164.00	0.00	0.00	0.00	0.00	0.00	0.00	0.00	0.00
0.00	0.00	0.00	0.00	0.00	0.00	0.00	0.00	0.00
0.00	0.00	0.00	0.00	0.00	0.00	0.00	0.00	0.00
0.00	0.00	0.00	0.00	0.00	0.00	0.00	0.00	0.00
0.00	0.00	0.00	0.00	0.00	0.00	0.00	0.00	0.00
0.00	0.00	0.00	0.00	0.00	0.00	0.00	0.00	0.00
0.00	0.00	0.00	0.00	0.00	0.00	0.00	0.00	0.00
0.00	0.00	0.00	0.00	0.00	0.00	0.00	0.00	0.00
0.00	0.00	0.00	0.00	0.00	0.00	0.00	0.00	0.00
0.00	0.00	0.00	0.00	0.00	0.00	0.00	0.00	0.00
14.00	0.00	0.00	0.00	0.00	0.00	0.00	0.00	0.00
0.00	0.00	0.00	15.00	0.00	7.00	14.00	0.00	12.00
0.00	0.00	0.00	0.00	0.00	0.00	0.00	0.00	0.00
0.00	0.00	0.00	0.00	0.00	0.00	0.00	156.00	0.00
0.00	0.00	0.00	0.00	0.00	0.00	0.00	0.00	0.00
0.00	0.00	0.00	0.00	0.00	0.00	0.00	0.00	0.00

0.00	0.00	0.00	0.00	0.00	0.00	0.00	0.00	0.00
0.00	0.00	0.00	0.00	0.00	0.00	0.00	0.00	0.00
0.00	0.00	12.00	22.00	0.00	0.00	0.00	0.00	9.00
0.00	0.00	0.00	0.00	0.00	0.00	0.00	0.00	0.00
0.00	0.00	5.00	9.00	3.00	4.00	10.00	0.00	12.00
0.00	23.00	17.00	38.00	0.00	0.00	0.00	0.00	0.00
0.00	0.00	0.00	0.00	0.00	0.00	0.00	0.00	0.00
0.00	0.00	0.00	0.00	0.00	0.00	0.00	0.00	0.00
0.00	0.00	0.00	0.00	0.00	0.00	0.00	0.00	0.00
0.00	0.00	0.00	0.00	0.00	0.00	0.00	0.00	0.00
0.00	0.00	0.00	0.00	0.00	0.00	0.00	0.00	0.00
0.00	0.00	0.00	0.00	0.00	0.00	0.00	0.00	0.00
0.00	0.00	0.00	0.00	14.00	0.00	0.00	0.00	0.00
0.00	0.00	0.00	0.00	0.00	0.00	0.00	0.00	0.00
0.00	0.00	0.00	0.00	0.00	36.00	0.00	0.00	0.00
0.00	0.00	0.00	0.00	0.00	0.00	0.00	0.00	0.00
0.00	0.00	0.00	0.00	0.00	0.00	0.00	0.00	0.00
0.00	0.00	0.00	0.00	0.00	0.00	0.00	0.00	0.00
0.00	0.00	0.00	0.00	0.00	0.00	0.00	0.00	0.00
0.00	0.00	0.00	0.00	0.00	0.00	0.00	0.00	0.00
0.00	0.00	0.00	0.00	0.00	0.00	0.00	0.00	0.00
0.00	0.00	0.00	0.00	0.00	0.00	0.00	0.00	0.00
0.00	0.00	0.00	0.00	0.00	0.00	0.00	0.00	0.00
0.00	0.00	0.00	0.00	0.00	0.00	0.00	0.00	0.00
0.00	0.00	0.00	0.00	0.00	0.00	0.00	0.00	0.00
0.00	0.00	0.00	0.00	0.00	0.00	0.00	0.00	0.00
0.00	0.00	0.00	0.00	0.00	0.00	0.00	0.00	0.00
0.00	0.00	0.00	0.00	0.00	0.00	0.00	0.00	0.00
0.00	0.00	0.00	0.00	0.00	0.00	0.00	0.00	0.00
0.00	26.00	0.00	0.00	0.00	0.00	0.00	0.00	0.00
0.00	0.00	0.00	0.00	0.00	0.00	0.00	0.00	0.00
0.00	0.00	0.00	0.00	0.00	0.00	0.00	0.00	0.00
0.00	0.00	0.00	0.00	0.00	0.00	0.00	0.00	0.00
0.00	0.00	0.00	0.00	0.00	0.00	0.00	0.00	0.00
0.00	0.00	0.00	0.00	131.00	0.00	0.00	0.00	0.00
0.00	0.00	0.00	0.00	0.00	0.00	0.00	0.00	0.00
0.00	0.00	0.00	0.00	0.00	0.00	0.00	0.00	0.00

0.00	0.00	0.00	0.00	0.00	0.00	0.00	0.00	0.00
0.00	0.00	0.00	0.00	0.00	0.00	0.00	0.00	0.00
0.00	0.00	0.00	0.00	0.00	0.00	0.00	0.00	0.00
0.00	0.00	0.00	0.00	0.00	0.00	0.00	0.00	0.00
0.00	0.00	0.00	0.00	133.00	0.00	0.00	0.00	0.00
0.00	0.00	0.00	0.00	0.00	0.00	0.00	0.00	0.00
0.00	0.00	0.00	0.00	0.00	0.00	0.00	0.00	0.00
0.00	0.00	0.00	0.00	0.00	0.00	0.00	0.00	0.00
0.00	0.00	0.00	0.00	0.00	0.00	0.00	0.00	0.00
0.00	22.00	20.00	0.00	0.00	0.00	0.00	0.00	0.00
0.00	0.00	0.00	0.00	0.00	0.00	0.00	0.00	0.00
0.00	0.00	0.00	0.00	0.00	0.00	0.00	0.00	0.00
0.00	0.00	0.00	0.00	0.00	0.00	0.00	77.00	0.00
0.00	0.00	0.00	0.00	0.00	0.00	0.00	0.00	0.00
0.00	0.00	0.00	0.00	126.00	0.00	0.00	0.00	0.00
0.00	0.00	0.00	0.00	0.00	0.00	0.00	0.00	0.00
0.00	0.00	0.00	0.00	0.00	0.00	0.00	0.00	0.00
0.00	0.00	0.00	0.00	0.00	0.00	0.00	0.00	0.00
0.00	0.00	0.00	0.00	0.00	0.00	0.00	0.00	0.00
0.00	0.00	0.00	0.00	0.00	0.00	0.00	0.00	0.00
0.00	0.00	0.00	0.00	0.00	0.00	0.00	0.00	0.00
0.00	0.00	0.00	0.00	0.00	0.00	0.00	0.00	0.00
0.00	0.00	0.00	0.00	0.00	0.00	0.00	0.00	0.00
0.00	0.00	0.00	0.00	0.00	0.00	0.00	0.00	0.00
0.00	0.00	0.00	0.00	0.00	0.00	0.00	0.00	0.00
0.00	0.00	0.00	0.00	0.00	0.00	0.00	0.00	0.00
0.00	0.00	0.00	0.00	0.00	0.00	0.00	0.00	0.00
0.00	0.00	0.00	0.00	0.00	0.00	0.00	0.00	0.00
0.00	0.00	0.00	0.00	0.00	0.00	0.00	0.00	0.00
0.00	0.00	0.00	0.00	0.00	0.00	0.00	0.00	0.00
0.00	0.00	0.00	0.00	0.00	0.00	0.00	0.00	0.00
0.00	0.00	0.00	0.00	0.00	0.00	0.00	0.00	0.00
0.00	0.00	0.00	0.00	0.00	0.00	0.00	0.00	0.00
0.00	0.00	0.00	0.00	0.00	0.00	0.00	0.00	0.00
0.00	0.00	0.00	0.00	0.00	0.00	0.00	0.00	0.00
0.00	0.00	0.00	0.00	0.00	0.00	0.00	0.00	0.00
0.00	0.00	0.00	0.00	0.00	0.00	0.00	0.00	0.00

0.00	0.00	0.00	0.00	0.00	0.00	0.00	0.00	0.00
0.00	0.00	0.00	0.00	0.00	0.00	0.00	0.00	0.00
0.00	0.00	0.00	0.00	0.00	0.00	0.00	0.00	0.00
0.00	0.00	0.00	0.00	0.00	0.00	0.00	0.00	0.00
0.00	0.00	0.00	0.00	0.00	0.00	0.00	0.00	0.00
0.00	0.00	0.00	0.00	0.00	0.00	0.00	0.00	0.00
0.00	0.00	0.00	0.00	0.00	0.00	0.00	0.00	0.00
0.00	0.00	0.00	0.00	0.00	0.00	0.00	0.00	0.00
0.00	0.00	0.00	0.00	0.00	0.00	0.00	0.00	0.00
0.00	0.00	0.00	0.00	0.00	0.00	0.00	0.00	0.00
0.00	0.00	0.00	0.00	110.00	0.00	0.00	0.00	0.00
0.00	0.00	0.00	0.00	0.00	0.00	0.00	0.00	0.00
15.00	17.00	0.00	0.00	0.00	0.00	0.00	0.00	0.00
0.00	0.00	0.00	0.00	0.00	0.00	0.00	0.00	0.00
0.00	0.00	0.00	0.00	0.00	0.00	0.00	0.00	0.00
0.00	0.00	0.00	0.00	0.00	0.00	0.00	0.00	0.00
0.00	0.00	0.00	0.00	0.00	0.00	0.00	0.00	0.00
0.00	0.00	0.00	0.00	0.00	0.00	0.00	0.00	0.00
0.00	0.00	0.00	0.00	0.00	0.00	0.00	0.00	0.00
0.00	0.00	0.00	0.00	0.00	0.00	0.00	0.00	0.00
0.00	0.00	11.00	0.00	0.00	0.00	0.00	0.00	0.00
0.00	0.00	0.00	0.00	0.00	0.00	0.00	0.00	0.00
0.00	0.00	0.00	0.00	0.00	0.00	0.00	0.00	0.00
13.00	0.00	0.00	24.00	0.00	0.00	0.00	0.00	0.00
0.00	0.00	0.00	0.00	0.00	0.00	0.00	0.00	0.00
0.00	0.00	0.00	0.00	0.00	0.00	0.00	0.00	0.00
0.00	0.00	0.00	0.00	0.00	0.00	0.00	0.00	0.00
0.00	0.00	0.00	0.00	0.00	0.00	0.00	0.00	0.00
0.00	0.00	0.00	0.00	0.00	0.00	0.00	0.00	0.00
0.00	0.00	12.00	20.00	0.00	0.00	0.00	0.00	0.00
0.00	0.00	0.00	0.00	0.00	0.00	0.00	0.00	0.00
0.00	0.00	0.00	0.00	0.00	0.00	0.00	0.00	0.00
0.00	0.00	0.00	0.00	0.00	0.00	0.00	0.00	0.00
0.00	0.00	0.00	0.00	0.00	0.00	0.00	48.00	0.00
0.00	0.00	0.00	0.00	0.00	0.00	0.00	101.00	0.00
0.00	0.00	0.00	0.00	0.00	0.00	0.00	0.00	0.00
0.00	0.00	0.00	0.00	0.00	0.00	0.00	0.00	0.00

0.00	0.00	0.00	0.00	0.00	0.00	0.00	0.00	0.00
0.00	0.00	0.00	0.00	0.00	0.00	0.00	0.00	0.00
0.00	0.00	0.00	0.00	0.00	0.00	0.00	0.00	0.00
0.00	0.00	0.00	0.00	0.00	0.00	0.00	0.00	0.00
0.00	0.00	0.00	0.00	0.00	0.00	0.00	0.00	0.00
0.00	0.00	0.00	0.00	0.00	0.00	0.00	0.00	0.00
0.00	0.00	0.00	0.00	0.00	0.00	0.00	0.00	0.00
0.00	0.00	0.00	0.00	0.00	0.00	0.00	0.00	0.00
3.00	0.00	0.00	9.00	0.00	0.00	0.00	0.00	0.00
0.00	0.00	0.00	0.00	8.00	0.00	0.00	0.00	0.00
0.00	0.00	0.00	0.00	0.00	0.00	0.00	0.00	0.00
0.00	0.00	0.00	0.00	0.00	0.00	0.00	0.00	0.00
0.00	0.00	0.00	0.00	0.00	0.00	0.00	0.00	0.00
0.00	0.00	0.00	0.00	0.00	0.00	0.00	0.00	0.00
0.00	0.00	0.00	0.00	0.00	0.00	0.00	0.00	0.00
0.00	0.00	0.00	0.00	0.00	0.00	0.00	0.00	0.00
0.00	0.00	0.00	0.00	0.00	0.00	0.00	0.00	8.00
0.00	0.00	0.00	0.00	0.00	0.00	0.00	0.00	0.00
0.00	0.00	0.00	0.00	0.00	0.00	0.00	0.00	0.00
0.00	0.00	0.00	0.00	0.00	0.00	0.00	0.00	0.00
0.00	0.00	0.00	0.00	0.00	0.00	0.00	0.00	0.00
0.00	0.00	0.00	0.00	0.00	0.00	0.00	0.00	0.00
0.00	0.00	0.00	0.00	0.00	0.00	0.00	0.00	0.00
0.00	0.00	7.00	6.00	0.00	0.00	0.00	0.00	0.00
0.00	0.00	0.00	0.00	0.00	0.00	0.00	0.00	0.00
0.00	0.00	0.00	0.00	0.00	0.00	0.00	0.00	0.00
0.00	0.00	0.00	0.00	0.00	0.00	0.00	0.00	0.00
0.00	0.00	0.00	0.00	0.00	0.00	0.00	0.00	0.00
0.00	0.00	0.00	0.00	0.00	0.00	0.00	0.00	0.00
0.00	0.00	0.00	0.00	0.00	0.00	0.00	0.00	0.00
0.00	0.00	0.00	0.00	0.00	0.00	0.00	0.00	28.00
0.00	0.00	0.00	0.00	0.00	0.00	0.00	0.00	0.00
0.00	0.00	0.00	0.00	0.00	0.00	0.00	0.00	0.00
0.00	0.00	0.00	0.00	0.00	0.00	0.00	0.00	0.00
0.00	0.00	0.00	0.00	0.00	0.00	0.00	0.00	0.00
0.00	0.00	0.00	0.00	0.00	0.00	0.00	0.00	0.00
0.00	0.00	0.00	0.00	0.00	0.00	0.00	0.00	0.00

0.00	0.00	0.00	0.00	0.00	0.00	0.00	0.00	0.00
0.00	0.00	0.00	0.00	0.00	0.00	0.00	0.00	0.00
0.00	0.00	0.00	14.00	0.00	0.00	0.00	0.00	0.00
33.00	0.00	0.00	0.00	0.00	0.00	0.00	0.00	0.00
0.00	0.00	0.00	0.00	0.00	0.00	0.00	0.00	0.00
0.00	0.00	0.00	0.00	0.00	0.00	0.00	0.00	0.00
0.00	0.00	0.00	0.00	0.00	0.00	0.00	0.00	0.00
88.00	0.00	0.00	0.00	0.00	0.00	0.00	0.00	0.00
0.00	0.00	0.00	0.00	88.00	0.00	0.00	0.00	0.00
0.00	0.00	0.00	0.00	0.00	0.00	0.00	0.00	0.00
0.00	0.00	0.00	0.00	0.00	0.00	0.00	0.00	0.00
0.00	0.00	0.00	0.00	0.00	0.00	0.00	0.00	0.00
0.00	0.00	0.00	0.00	0.00	0.00	0.00	0.00	0.00
0.00	0.00	0.00	0.00	0.00	0.00	0.00	0.00	0.00
0.00	0.00	0.00	0.00	0.00	0.00	0.00	0.00	0.00
0.00	0.00	0.00	0.00	0.00	0.00	0.00	0.00	0.00
0.00	0.00	0.00	0.00	0.00	0.00	0.00	0.00	0.00
0.00	0.00	0.00	0.00	0.00	0.00	0.00	0.00	0.00
0.00	0.00	0.00	0.00	0.00	0.00	0.00	0.00	0.00
0.00	0.00	0.00	0.00	0.00	0.00	0.00	0.00	0.00
0.00	0.00	0.00	0.00	0.00	0.00	0.00	0.00	0.00
0.00	0.00	0.00	0.00	0.00	0.00	0.00	0.00	0.00
0.00	0.00	0.00	0.00	0.00	0.00	0.00	0.00	0.00
0.00	0.00	0.00	0.00	0.00	0.00	0.00	0.00	0.00
0.00	0.00	0.00	0.00	0.00	0.00	0.00	0.00	0.00
0.00	0.00	0.00	0.00	0.00	0.00	0.00	0.00	0.00
0.00	0.00	0.00	0.00	0.00	0.00	0.00	0.00	0.00
0.00	0.00	0.00	0.00	0.00	0.00	0.00	0.00	0.00
0.00	0.00	0.00	0.00	0.00	0.00	0.00	0.00	0.00
0.00	0.00	0.00	0.00	0.00	0.00	0.00	0.00	0.00
0.00	0.00	0.00	0.00	0.00	0.00	0.00	80.00	0.00
0.00	0.00	0.00	0.00	0.00	0.00	0.00	0.00	0.00
0.00	0.00	0.00	0.00	0.00	0.00	0.00	0.00	0.00
0.00	0.00	0.00	0.00	0.00	0.00	0.00	0.00	0.00
0.00	0.00	0.00	0.00	0.00	0.00	0.00	0.00	0.00
0.00	0.00	0.00	0.00	0.00	0.00	0.00	0.00	0.00
0.00	0.00	0.00	0.00	0.00	0.00	0.00	0.00	0.00

0.00	0.00	0.00	0.00	0.00	0.00	0.00	0.00	0.00
0.00	0.00	0.00	0.00	0.00	0.00	0.00	0.00	0.00
0.00	0.00	0.00	0.00	0.00	0.00	0.00	0.00	0.00
0.00	0.00	0.00	0.00	0.00	0.00	0.00	0.00	0.00
0.00	0.00	0.00	0.00	0.00	0.00	0.00	0.00	0.00
0.00	0.00	0.00	0.00	0.00	0.00	0.00	0.00	0.00
0.00	0.00	0.00	0.00	0.00	0.00	0.00	0.00	0.00
0.00	0.00	0.00	0.00	0.00	0.00	0.00	0.00	0.00
0.00	0.00	0.00	0.00	0.00	0.00	0.00	0.00	11.00
0.00	0.00	0.00	0.00	0.00	0.00	0.00	0.00	0.00
0.00	0.00	0.00	0.00	0.00	0.00	0.00	0.00	0.00
0.00	0.00	0.00	0.00	0.00	0.00	0.00	0.00	0.00
0.00	0.00	0.00	0.00	0.00	0.00	0.00	0.00	0.00
0.00	0.00	0.00	0.00	0.00	0.00	0.00	0.00	0.00
0.00	0.00	0.00	0.00	0.00	0.00	0.00	0.00	0.00
0.00	0.00	0.00	0.00	0.00	0.00	0.00	0.00	0.00
0.00	0.00	0.00	0.00	0.00	0.00	0.00	0.00	0.00
0.00	0.00	0.00	0.00	0.00	0.00	0.00	0.00	0.00
0.00	0.00	0.00	0.00	0.00	0.00	0.00	0.00	0.00
0.00	0.00	0.00	0.00	0.00	0.00	0.00	0.00	0.00
54.00	0.00	0.00	0.00	0.00	0.00	0.00	0.00	0.00
0.00	0.00	0.00	0.00	0.00	0.00	0.00	74.00	0.00
0.00	0.00	0.00	0.00	0.00	0.00	0.00	0.00	0.00
0.00	0.00	0.00	0.00	0.00	0.00	0.00	0.00	0.00
0.00	0.00	0.00	0.00	0.00	0.00	0.00	0.00	0.00
0.00	0.00	0.00	0.00	0.00	0.00	0.00	0.00	0.00
0.00	0.00	0.00	0.00	0.00	0.00	0.00	0.00	0.00
0.00	0.00	0.00	0.00	0.00	0.00	0.00	0.00	0.00
0.00	0.00	0.00	0.00	0.00	0.00	0.00	0.00	0.00
0.00	0.00	0.00	0.00	0.00	0.00	0.00	0.00	0.00
0.00	0.00	0.00	0.00	0.00	0.00	0.00	0.00	0.00
0.00	0.00	0.00	0.00	0.00	0.00	0.00	0.00	0.00
0.00	0.00	0.00	0.00	0.00	0.00	0.00	0.00	0.00
0.00	0.00	0.00	0.00	0.00	0.00	0.00	0.00	0.00
0.00	0.00	0.00	0.00	0.00	0.00	0.00	0.00	0.00
0.00	0.00	0.00	0.00	0.00	0.00	0.00	0.00	0.00
0.00	0.00	0.00	0.00	0.00	0.00	0.00	0.00	0.00

0.00	0.00	0.00	0.00	0.00	0.00	0.00	0.00	0.00
0.00	0.00	0.00	0.00	0.00	0.00	0.00	0.00	0.00
0.00	0.00	0.00	0.00	0.00	0.00	0.00	0.00	0.00
0.00	0.00	0.00	0.00	0.00	0.00	0.00	0.00	0.00
0.00	0.00	0.00	0.00	0.00	0.00	0.00	0.00	0.00
0.00	0.00	0.00	0.00	0.00	0.00	0.00	0.00	0.00
0.00	0.00	0.00	0.00	0.00	0.00	0.00	0.00	0.00
0.00	0.00	0.00	0.00	0.00	0.00	0.00	0.00	0.00
0.00	0.00	0.00	0.00	0.00	0.00	0.00	68.00	0.00
0.00	0.00	0.00	0.00	0.00	0.00	0.00	0.00	0.00
0.00	0.00	0.00	0.00	0.00	0.00	0.00	0.00	0.00
0.00	0.00	0.00	0.00	0.00	0.00	0.00	0.00	0.00
0.00	0.00	0.00	0.00	0.00	0.00	0.00	0.00	0.00
0.00	0.00	0.00	0.00	0.00	0.00	0.00	0.00	0.00
0.00	0.00	0.00	0.00	0.00	0.00	0.00	0.00	0.00
0.00	0.00	0.00	0.00	0.00	0.00	0.00	0.00	0.00
0.00	0.00	0.00	0.00	0.00	0.00	0.00	0.00	0.00
0.00	0.00	0.00	0.00	0.00	0.00	0.00	0.00	0.00
0.00	0.00	0.00	0.00	0.00	0.00	0.00	0.00	0.00
0.00	0.00	0.00	0.00	0.00	0.00	0.00	0.00	0.00
0.00	0.00	0.00	0.00	0.00	0.00	0.00	0.00	0.00
0.00	0.00	0.00	0.00	0.00	0.00	0.00	0.00	0.00
0.00	0.00	0.00	0.00	0.00	0.00	0.00	0.00	0.00
0.00	0.00	0.00	0.00	0.00	0.00	0.00	0.00	0.00
0.00	0.00	0.00	0.00	0.00	0.00	0.00	0.00	0.00
0.00	0.00	0.00	0.00	0.00	0.00	0.00	0.00	0.00
0.00	0.00	0.00	0.00	0.00	0.00	0.00	0.00	0.00
0.00	0.00	0.00	0.00	0.00	0.00	0.00	0.00	0.00
0.00	0.00	0.00	0.00	0.00	0.00	0.00	0.00	0.00
0.00	0.00	0.00	0.00	0.00	0.00	0.00	0.00	0.00
0.00	0.00	0.00	0.00	0.00	0.00	0.00	0.00	0.00
0.00	0.00	0.00	0.00	0.00	0.00	0.00	0.00	0.00
0.00	0.00	0.00	0.00	0.00	0.00	0.00	0.00	0.00
0.00	0.00	0.00	0.00	0.00	0.00	0.00	0.00	0.00
0.00	0.00	0.00	0.00	0.00	0.00	0.00	0.00	0.00
0.00	0.00	0.00	0.00	0.00	0.00	0.00	0.00	0.00
0.00	0.00	0.00	0.00	0.00	0.00	0.00	0.00	0.00

0.00	0.00	0.00	0.00	0.00	0.00	0.00	0.00	0.00
0.00	0.00	0.00	0.00	0.00	0.00	0.00	0.00	0.00
0.00	0.00	0.00	0.00	0.00	0.00	0.00	0.00	0.00
0.00	0.00	0.00	0.00	0.00	0.00	0.00	0.00	0.00
63.00	0.00	0.00	0.00	0.00	0.00	0.00	0.00	0.00
63.00	0.00	0.00	0.00	0.00	0.00	0.00	0.00	0.00
63.00	0.00	0.00	0.00	0.00	0.00	0.00	0.00	0.00
0.00	6.00	0.00	0.00	0.00	0.00	0.00	0.00	0.00
0.00	0.00	0.00	0.00	0.00	0.00	0.00	0.00	0.00
0.00	0.00	0.00	0.00	0.00	0.00	0.00	0.00	0.00
0.00	0.00	0.00	0.00	0.00	0.00	0.00	0.00	0.00
0.00	0.00	0.00	0.00	0.00	0.00	0.00	0.00	0.00
0.00	0.00	0.00	0.00	0.00	0.00	0.00	0.00	0.00
0.00	0.00	0.00	0.00	0.00	0.00	0.00	0.00	0.00
0.00	0.00	0.00	0.00	0.00	0.00	0.00	0.00	0.00
0.00	0.00	0.00	0.00	0.00	0.00	0.00	0.00	0.00
0.00	0.00	0.00	0.00	0.00	0.00	0.00	0.00	0.00
0.00	0.00	0.00	0.00	0.00	0.00	0.00	0.00	0.00
0.00	0.00	0.00	0.00	0.00	0.00	0.00	0.00	0.00
0.00	0.00	0.00	0.00	0.00	0.00	0.00	0.00	0.00
0.00	0.00	0.00	0.00	0.00	0.00	0.00	0.00	0.00
0.00	0.00	0.00	0.00	0.00	8.00	0.00	0.00	0.00
0.00	0.00	0.00	0.00	0.00	0.00	0.00	0.00	0.00
0.00	0.00	0.00	0.00	0.00	0.00	0.00	0.00	0.00
0.00	0.00	0.00	0.00	0.00	0.00	0.00	0.00	0.00
60.00	0.00	0.00	0.00	0.00	0.00	0.00	0.00	0.00
0.00	0.00	0.00	0.00	0.00	0.00	0.00	0.00	0.00
0.00	0.00	0.00	0.00	0.00	0.00	0.00	0.00	0.00
0.00	0.00	0.00	0.00	0.00	0.00	0.00	0.00	0.00
0.00	0.00	0.00	0.00	0.00	0.00	0.00	0.00	0.00
0.00	0.00	0.00	0.00	0.00	0.00	0.00	0.00	0.00
0.00	0.00	0.00	0.00	0.00	0.00	0.00	0.00	0.00
0.00	0.00	0.00	0.00	0.00	0.00	0.00	0.00	0.00
0.00	0.00	0.00	0.00	0.00	0.00	0.00	0.00	0.00
12.00	0.00	0.00	0.00	0.00	0.00	0.00	0.00	0.00
0.00	0.00	0.00	0.00	0.00	0.00	0.00	0.00	0.00
0.00	0.00	0.00	0.00	0.00	0.00	0.00	0.00	0.00

0.00	0.00	0.00	0.00	0.00	0.00	0.00	0.00	0.00
0.00	0.00	0.00	0.00	0.00	0.00	0.00	0.00	0.00
0.00	0.00	0.00	0.00	0.00	0.00	0.00	0.00	0.00
0.00	0.00	0.00	0.00	0.00	0.00	0.00	0.00	0.00
0.00	0.00	0.00	0.00	0.00	0.00	0.00	0.00	0.00
0.00	0.00	0.00	0.00	0.00	0.00	0.00	0.00	0.00
0.00	0.00	0.00	0.00	0.00	0.00	0.00	0.00	0.00
0.00	0.00	0.00	0.00	0.00	0.00	0.00	0.00	0.00
0.00	0.00	0.00	0.00	0.00	0.00	0.00	0.00	0.00
0.00	0.00	0.00	0.00	0.00	0.00	0.00	0.00	0.00
0.00	0.00	0.00	0.00	0.00	0.00	0.00	0.00	0.00
0.00	0.00	0.00	0.00	0.00	0.00	0.00	0.00	0.00
0.00	0.00	0.00	0.00	0.00	0.00	0.00	0.00	0.00
0.00	0.00	0.00	0.00	0.00	0.00	0.00	0.00	0.00
0.00	0.00	0.00	0.00	0.00	0.00	0.00	0.00	0.00
0.00	0.00	0.00	0.00	0.00	0.00	0.00	0.00	0.00
0.00	0.00	0.00	0.00	0.00	0.00	0.00	0.00	0.00
0.00	0.00	0.00	0.00	0.00	0.00	0.00	0.00	0.00
0.00	0.00	0.00	0.00	0.00	0.00	0.00	0.00	0.00
0.00	0.00	0.00	0.00	0.00	0.00	0.00	0.00	44.00
56.00	0.00	0.00	0.00	0.00	0.00	0.00	0.00	0.00
21.00	11.00	0.00	0.00	0.00	0.00	0.00	0.00	19.00
0.00	0.00	0.00	0.00	0.00	0.00	0.00	56.00	0.00
0.00	0.00	0.00	0.00	0.00	0.00	0.00	0.00	0.00
0.00	0.00	0.00	0.00	0.00	0.00	0.00	0.00	0.00
0.00	0.00	0.00	0.00	0.00	0.00	0.00	0.00	0.00
0.00	0.00	0.00	0.00	0.00	0.00	0.00	0.00	0.00
0.00	0.00	0.00	0.00	0.00	0.00	0.00	0.00	0.00
0.00	0.00	0.00	0.00	0.00	0.00	0.00	0.00	0.00
0.00	0.00	0.00	0.00	0.00	0.00	0.00	0.00	0.00
0.00	0.00	0.00	0.00	0.00	0.00	0.00	0.00	0.00
0.00	0.00	0.00	0.00	0.00	0.00	0.00	0.00	0.00
0.00	0.00	0.00	0.00	0.00	0.00	0.00	0.00	0.00
0.00	0.00	0.00	0.00	0.00	0.00	0.00	0.00	0.00
0.00	0.00	0.00	0.00	0.00	0.00	0.00	0.00	0.00
0.00	0.00	0.00	0.00	0.00	0.00	0.00	0.00	0.00
0.00	0.00	0.00	0.00	0.00	0.00	0.00	0.00	0.00

54.00	0.00	0.00	0.00	0.00	0.00	0.00	0.00	0.00
0.00	0.00	0.00	0.00	0.00	0.00	0.00	0.00	0.00
0.00	0.00	0.00	0.00	0.00	0.00	0.00	0.00	0.00
0.00	0.00	0.00	0.00	0.00	0.00	0.00	0.00	0.00
0.00	0.00	0.00	0.00	0.00	0.00	0.00	0.00	0.00
0.00	0.00	0.00	0.00	0.00	0.00	0.00	0.00	0.00
0.00	0.00	0.00	0.00	0.00	0.00	0.00	0.00	0.00
0.00	0.00	0.00	0.00	0.00	0.00	0.00	0.00	0.00
0.00	0.00	0.00	0.00	0.00	0.00	0.00	0.00	0.00
0.00	0.00	0.00	0.00	0.00	0.00	0.00	0.00	0.00
0.00	0.00	0.00	0.00	0.00	0.00	0.00	0.00	0.00
0.00	0.00	0.00	16.00	0.00	0.00	0.00	0.00	0.00
0.00	0.00	0.00	0.00	0.00	0.00	0.00	0.00	0.00
0.00	0.00	0.00	0.00	0.00	0.00	0.00	0.00	0.00
0.00	0.00	0.00	0.00	0.00	0.00	0.00	0.00	0.00
0.00	0.00	0.00	0.00	0.00	0.00	0.00	0.00	0.00
0.00	0.00	0.00	0.00	0.00	0.00	0.00	0.00	0.00
0.00	0.00	0.00	0.00	0.00	0.00	0.00	0.00	0.00
0.00	0.00	0.00	0.00	0.00	0.00	0.00	0.00	0.00
0.00	0.00	0.00	0.00	0.00	0.00	0.00	0.00	0.00
0.00	0.00	0.00	0.00	0.00	0.00	0.00	0.00	0.00
0.00	0.00	0.00	0.00	0.00	0.00	0.00	0.00	0.00
0.00	0.00	0.00	0.00	0.00	0.00	0.00	0.00	0.00
0.00	0.00	0.00	0.00	0.00	0.00	0.00	0.00	0.00
0.00	0.00	0.00	0.00	0.00	0.00	0.00	0.00	0.00
0.00	0.00	0.00	0.00	0.00	0.00	0.00	0.00	0.00
0.00	0.00	0.00	0.00	0.00	0.00	0.00	0.00	0.00
0.00	0.00	0.00	0.00	0.00	0.00	0.00	0.00	0.00
0.00	0.00	0.00	0.00	0.00	0.00	0.00	0.00	0.00
0.00	0.00	0.00	0.00	0.00	0.00	0.00	0.00	0.00
0.00	0.00	0.00	0.00	0.00	0.00	0.00	0.00	0.00
0.00	0.00	0.00	0.00	0.00	0.00	0.00	0.00	0.00
0.00	0.00	0.00	0.00	0.00	0.00	0.00	0.00	0.00
0.00	0.00	0.00	0.00	0.00	0.00	0.00	0.00	0.00
0.00	0.00	0.00	0.00	0.00	0.00	0.00	0.00	0.00
0.00	0.00	0.00	0.00	0.00	0.00	0.00	0.00	0.00
0.00	0.00	0.00	0.00	0.00	0.00	0.00	0.00	0.00

0.00	0.00	0.00	0.00	0.00	0.00	0.00	0.00	0.00
0.00	0.00	0.00	0.00	0.00	0.00	0.00	0.00	0.00
0.00	0.00	0.00	0.00	0.00	0.00	0.00	0.00	0.00
0.00	0.00	0.00	0.00	0.00	0.00	0.00	0.00	0.00
0.00	0.00	0.00	0.00	0.00	0.00	0.00	0.00	0.00
0.00	0.00	0.00	0.00	0.00	0.00	0.00	0.00	0.00
0.00	0.00	0.00	0.00	0.00	0.00	0.00	0.00	0.00
0.00	0.00	0.00	0.00	0.00	0.00	0.00	0.00	0.00
0.00	0.00	0.00	0.00	0.00	0.00	0.00	0.00	0.00
0.00	0.00	0.00	0.00	0.00	0.00	0.00	0.00	0.00
0.00	0.00	0.00	0.00	0.00	0.00	0.00	0.00	0.00
0.00	0.00	0.00	0.00	47.00	0.00	0.00	0.00	0.00
0.00	0.00	0.00	0.00	0.00	0.00	0.00	0.00	0.00
0.00	0.00	0.00	0.00	0.00	0.00	0.00	0.00	0.00
0.00	0.00	0.00	0.00	0.00	0.00	0.00	0.00	0.00
0.00	0.00	0.00	0.00	0.00	0.00	0.00	0.00	0.00
0.00	0.00	0.00	0.00	0.00	0.00	0.00	0.00	0.00
0.00	0.00	0.00	0.00	0.00	0.00	0.00	0.00	0.00
0.00	0.00	0.00	0.00	0.00	0.00	0.00	0.00	0.00
0.00	0.00	0.00	0.00	0.00	0.00	0.00	0.00	0.00
0.00	0.00	0.00	0.00	0.00	0.00	0.00	0.00	0.00
0.00	0.00	0.00	0.00	0.00	0.00	0.00	0.00	0.00
0.00	0.00	0.00	0.00	0.00	0.00	0.00	0.00	0.00
0.00	0.00	0.00	0.00	0.00	0.00	0.00	0.00	0.00
0.00	0.00	0.00	0.00	0.00	0.00	0.00	0.00	0.00
0.00	0.00	0.00	0.00	0.00	0.00	0.00	0.00	0.00
0.00	0.00	0.00	0.00	0.00	0.00	0.00	0.00	0.00
0.00	0.00	0.00	0.00	0.00	0.00	0.00	0.00	0.00
0.00	0.00	0.00	0.00	0.00	0.00	0.00	0.00	0.00
0.00	0.00	0.00	0.00	0.00	0.00	0.00	0.00	0.00
0.00	0.00	0.00	0.00	0.00	0.00	0.00	0.00	0.00
0.00	0.00	0.00	0.00	0.00	0.00	0.00	0.00	0.00
0.00	0.00	0.00	0.00	0.00	0.00	0.00	0.00	0.00
0.00	0.00	0.00	0.00	0.00	0.00	0.00	0.00	0.00
0.00	0.00	0.00	0.00	0.00	0.00	0.00	0.00	0.00
0.00	0.00	0.00	0.00	0.00	0.00	0.00	0.00	0.00
0.00	0.00	0.00	0.00	0.00	0.00	0.00	0.00	0.00

0.00	0.00	0.00	0.00	0.00	0.00	0.00	0.00	0.00
4.00	0.00	0.00	0.00	0.00	0.00	0.00	40.00	0.00
0.00	0.00	0.00	0.00	0.00	0.00	4.00	0.00	0.00
0.00	0.00	0.00	0.00	0.00	0.00	0.00	0.00	0.00
0.00	0.00	0.00	0.00	0.00	0.00	0.00	0.00	0.00
0.00	0.00	0.00	0.00	0.00	0.00	0.00	0.00	0.00
0.00	0.00	0.00	0.00	0.00	0.00	0.00	0.00	0.00
0.00	0.00	0.00	0.00	0.00	0.00	0.00	0.00	0.00
0.00	0.00	0.00	0.00	0.00	0.00	0.00	0.00	0.00
0.00	0.00	0.00	0.00	0.00	0.00	0.00	0.00	0.00
0.00	0.00	0.00	0.00	0.00	0.00	0.00	0.00	0.00
0.00	0.00	0.00	0.00	0.00	0.00	0.00	0.00	0.00
0.00	0.00	0.00	0.00	0.00	0.00	0.00	0.00	0.00
0.00	0.00	0.00	0.00	0.00	0.00	0.00	43.00	0.00
0.00	0.00	0.00	0.00	0.00	0.00	0.00	0.00	0.00
0.00	0.00	0.00	0.00	0.00	0.00	0.00	0.00	0.00
0.00	0.00	0.00	0.00	0.00	0.00	0.00	0.00	0.00
0.00	0.00	0.00	0.00	0.00	0.00	0.00	0.00	0.00
0.00	0.00	0.00	0.00	0.00	0.00	0.00	0.00	0.00
0.00	0.00	0.00	0.00	0.00	0.00	0.00	0.00	0.00
0.00	5.00	0.00	10.00	0.00	0.00	0.00	0.00	0.00
0.00	0.00	0.00	0.00	0.00	0.00	0.00	0.00	0.00
0.00	0.00	0.00	0.00	0.00	0.00	0.00	0.00	0.00
0.00	0.00	0.00	0.00	0.00	0.00	0.00	0.00	0.00
0.00	0.00	0.00	0.00	0.00	0.00	0.00	0.00	0.00
0.00	0.00	0.00	0.00	0.00	0.00	0.00	0.00	0.00
0.00	0.00	0.00	0.00	0.00	0.00	0.00	0.00	0.00
0.00	0.00	0.00	0.00	0.00	0.00	0.00	0.00	0.00
0.00	0.00	0.00	0.00	0.00	0.00	0.00	0.00	0.00
0.00	41.00	0.00	0.00	0.00	0.00	0.00	0.00	0.00
0.00	0.00	0.00	0.00	0.00	0.00	0.00	0.00	0.00
0.00	0.00	0.00	0.00	0.00	0.00	0.00	0.00	0.00
0.00	0.00	0.00	0.00	0.00	0.00	0.00	0.00	0.00
0.00	0.00	0.00	0.00	0.00	0.00	0.00	0.00	0.00
0.00	0.00	0.00	0.00	0.00	0.00	0.00	0.00	0.00
0.00	0.00	0.00	0.00	0.00	0.00	0.00	0.00	0.00
0.00	0.00	0.00	0.00	0.00	0.00	0.00	0.00	0.00

0.00	0.00	0.00	0.00	0.00	0.00	0.00	0.00	0.00
0.00	0.00	0.00	0.00	0.00	0.00	0.00	0.00	0.00
0.00	0.00	0.00	0.00	0.00	0.00	0.00	0.00	0.00
0.00	0.00	0.00	0.00	0.00	0.00	0.00	0.00	0.00
0.00	0.00	0.00	15.00	0.00	0.00	0.00	0.00	0.00
0.00	0.00	0.00	0.00	0.00	0.00	0.00	0.00	0.00
0.00	0.00	0.00	0.00	0.00	0.00	0.00	0.00	0.00
0.00	0.00	0.00	0.00	0.00	0.00	0.00	0.00	0.00
0.00	0.00	0.00	0.00	0.00	0.00	0.00	0.00	0.00
0.00	0.00	0.00	0.00	0.00	0.00	0.00	0.00	0.00
0.00	0.00	0.00	0.00	0.00	0.00	0.00	0.00	0.00
0.00	0.00	0.00	0.00	0.00	0.00	0.00	0.00	0.00
0.00	0.00	0.00	0.00	0.00	0.00	0.00	0.00	0.00
0.00	0.00	0.00	0.00	0.00	0.00	0.00	0.00	0.00
0.00	0.00	0.00	0.00	0.00	0.00	0.00	0.00	0.00
0.00	0.00	0.00	0.00	0.00	0.00	0.00	0.00	0.00
0.00	0.00	0.00	0.00	0.00	0.00	0.00	0.00	0.00
0.00	0.00	0.00	0.00	0.00	0.00	0.00	0.00	0.00
0.00	0.00	0.00	0.00	0.00	0.00	0.00	0.00	0.00
0.00	0.00	0.00	0.00	0.00	0.00	0.00	0.00	0.00
0.00	0.00	0.00	0.00	0.00	0.00	0.00	0.00	0.00
0.00	0.00	0.00	0.00	0.00	0.00	0.00	0.00	0.00
0.00	0.00	0.00	0.00	0.00	0.00	0.00	0.00	0.00
0.00	0.00	0.00	0.00	0.00	0.00	0.00	0.00	0.00
0.00	0.00	0.00	0.00	0.00	0.00	0.00	0.00	0.00
0.00	0.00	0.00	0.00	0.00	0.00	0.00	0.00	0.00
0.00	0.00	0.00	0.00	0.00	0.00	0.00	0.00	0.00
0.00	0.00	17.00	0.00	0.00	0.00	0.00	0.00	0.00
0.00	0.00	0.00	0.00	38.00	0.00	0.00	0.00	0.00
0.00	0.00	0.00	0.00	0.00	0.00	0.00	0.00	0.00
0.00	0.00	0.00	0.00	0.00	0.00	0.00	0.00	0.00
0.00	0.00	0.00	0.00	0.00	0.00	0.00	0.00	0.00
0.00	0.00	0.00	0.00	0.00	0.00	0.00	0.00	0.00
0.00	0.00	0.00	0.00	0.00	0.00	0.00	0.00	0.00
37.00	0.00	0.00	0.00	0.00	0.00	0.00	0.00	0.00
0.00	0.00	0.00	0.00	0.00	0.00	0.00	0.00	0.00
0.00	0.00	0.00	0.00	0.00	0.00	0.00	0.00	0.00

0.00	0.00	0.00	0.00	0.00	0.00	0.00	0.00	0.00
0.00	0.00	0.00	0.00	0.00	0.00	0.00	0.00	0.00
0.00	0.00	0.00	0.00	0.00	0.00	0.00	0.00	0.00
0.00	0.00	0.00	0.00	0.00	0.00	0.00	0.00	0.00
0.00	0.00	0.00	0.00	0.00	0.00	0.00	0.00	0.00
0.00	0.00	0.00	0.00	0.00	0.00	0.00	0.00	0.00
0.00	0.00	0.00	0.00	0.00	0.00	0.00	0.00	0.00
0.00	0.00	0.00	0.00	0.00	0.00	0.00	0.00	0.00
0.00	0.00	0.00	0.00	0.00	0.00	0.00	36.00	0.00
0.00	0.00	0.00	0.00	0.00	0.00	0.00	0.00	0.00
0.00	0.00	0.00	0.00	0.00	0.00	0.00	0.00	0.00
0.00	0.00	0.00	0.00	0.00	0.00	0.00	0.00	0.00
0.00	0.00	0.00	0.00	0.00	0.00	0.00	0.00	0.00
0.00	0.00	0.00	0.00	0.00	0.00	0.00	0.00	0.00
0.00	0.00	0.00	0.00	0.00	0.00	0.00	0.00	0.00
0.00	0.00	0.00	0.00	0.00	0.00	0.00	0.00	0.00
0.00	0.00	0.00	0.00	0.00	0.00	0.00	0.00	0.00
0.00	0.00	0.00	0.00	0.00	0.00	0.00	0.00	0.00
0.00	0.00	0.00	0.00	0.00	0.00	0.00	0.00	0.00
0.00	0.00	0.00	0.00	0.00	0.00	0.00	0.00	0.00
0.00	0.00	0.00	0.00	0.00	0.00	0.00	0.00	0.00
0.00	0.00	0.00	0.00	0.00	0.00	0.00	0.00	0.00
0.00	0.00	0.00	0.00	0.00	0.00	0.00	0.00	0.00
0.00	0.00	0.00	0.00	0.00	0.00	0.00	0.00	0.00
0.00	0.00	0.00	0.00	0.00	0.00	0.00	0.00	0.00
0.00	0.00	0.00	0.00	0.00	0.00	0.00	0.00	0.00
0.00	0.00	0.00	0.00	0.00	0.00	0.00	0.00	0.00
0.00	0.00	0.00	0.00	0.00	0.00	0.00	0.00	0.00
0.00	0.00	0.00	0.00	0.00	0.00	0.00	0.00	0.00
0.00	0.00	0.00	0.00	0.00	0.00	0.00	0.00	0.00
0.00	0.00	0.00	0.00	0.00	0.00	0.00	0.00	14.00
0.00	0.00	0.00	0.00	0.00	0.00	0.00	0.00	0.00
0.00	0.00	0.00	0.00	0.00	0.00	0.00	0.00	0.00
0.00	0.00	0.00	0.00	0.00	0.00	0.00	0.00	0.00
0.00	0.00	0.00	0.00	0.00	0.00	0.00	0.00	0.00
0.00	0.00	0.00	0.00	0.00	0.00	0.00	0.00	0.00
0.00	0.00	0.00	0.00	0.00	0.00	0.00	0.00	0.00

0.00	0.00	0.00	0.00	0.00	0.00	0.00	0.00	0.00
0.00	0.00	0.00	0.00	0.00	0.00	0.00	0.00	0.00
0.00	0.00	0.00	0.00	0.00	0.00	0.00	0.00	0.00
0.00	0.00	0.00	0.00	0.00	0.00	0.00	0.00	0.00
0.00	0.00	0.00	0.00	0.00	0.00	0.00	0.00	0.00
0.00	0.00	0.00	0.00	0.00	0.00	0.00	0.00	0.00
0.00	0.00	0.00	0.00	0.00	0.00	7.00	0.00	0.00
0.00	0.00	0.00	0.00	0.00	0.00	0.00	32.00	0.00
0.00	0.00	0.00	0.00	0.00	0.00	0.00	0.00	0.00
0.00	0.00	0.00	0.00	0.00	0.00	0.00	0.00	0.00
0.00	0.00	0.00	0.00	0.00	0.00	0.00	0.00	0.00
0.00	0.00	0.00	0.00	0.00	0.00	0.00	0.00	0.00
0.00	0.00	0.00	0.00	0.00	0.00	0.00	0.00	0.00
0.00	0.00	0.00	0.00	0.00	0.00	0.00	0.00	0.00
0.00	0.00	0.00	0.00	0.00	0.00	0.00	0.00	0.00
0.00	0.00	0.00	0.00	0.00	0.00	0.00	0.00	0.00
0.00	0.00	0.00	0.00	0.00	0.00	0.00	0.00	0.00
0.00	0.00	0.00	0.00	0.00	0.00	0.00	0.00	0.00
15.00	0.00	0.00	0.00	0.00	0.00	0.00	0.00	0.00
0.00	0.00	0.00	0.00	0.00	0.00	0.00	0.00	0.00
0.00	0.00	0.00	0.00	0.00	0.00	0.00	0.00	0.00
0.00	0.00	0.00	0.00	0.00	0.00	0.00	0.00	0.00
0.00	0.00	0.00	0.00	0.00	0.00	0.00	0.00	0.00
0.00	0.00	0.00	0.00	0.00	0.00	0.00	0.00	0.00
0.00	0.00	0.00	0.00	0.00	0.00	0.00	0.00	0.00
0.00	0.00	0.00	0.00	0.00	0.00	0.00	0.00	0.00
0.00	0.00	0.00	0.00	0.00	0.00	0.00	0.00	0.00
0.00	0.00	0.00	0.00	0.00	0.00	0.00	0.00	0.00
0.00	0.00	0.00	0.00	0.00	0.00	0.00	0.00	0.00
0.00	0.00	0.00	0.00	0.00	0.00	0.00	0.00	0.00
0.00	0.00	0.00	0.00	0.00	0.00	0.00	0.00	0.00
0.00	0.00	0.00	0.00	0.00	0.00	0.00	0.00	0.00
0.00	0.00	0.00	0.00	0.00	0.00	0.00	0.00	0.00
0.00	0.00	0.00	0.00	0.00	0.00	0.00	5.00	0.00
0.00	0.00	0.00	0.00	0.00	0.00	0.00	30.00	0.00
0.00	0.00	0.00	0.00	0.00	0.00	0.00	30.00	0.00
0.00	0.00	0.00	0.00	0.00	0.00	0.00	0.00	0.00

0.00	0.00	0.00	0.00	0.00	0.00	0.00	0.00	0.00
0.00	0.00	0.00	0.00	0.00	0.00	0.00	0.00	0.00
0.00	0.00	0.00	0.00	0.00	0.00	0.00	0.00	0.00
0.00	0.00	0.00	0.00	0.00	0.00	0.00	0.00	0.00
0.00	0.00	0.00	0.00	0.00	0.00	0.00	0.00	0.00
0.00	0.00	0.00	0.00	0.00	0.00	0.00	0.00	0.00
0.00	0.00	0.00	0.00	0.00	0.00	0.00	0.00	0.00
0.00	0.00	0.00	0.00	0.00	0.00	0.00	0.00	0.00
0.00	0.00	0.00	0.00	0.00	0.00	0.00	0.00	0.00
0.00	0.00	0.00	0.00	0.00	0.00	0.00	0.00	0.00
0.00	0.00	0.00	0.00	0.00	0.00	0.00	0.00	0.00
0.00	0.00	0.00	0.00	0.00	0.00	0.00	0.00	0.00
0.00	0.00	0.00	0.00	0.00	0.00	0.00	0.00	0.00
0.00	0.00	0.00	0.00	0.00	0.00	0.00	0.00	0.00
0.00	0.00	0.00	0.00	0.00	0.00	0.00	0.00	0.00
0.00	0.00	0.00	0.00	0.00	0.00	0.00	29.00	0.00
0.00	0.00	0.00	0.00	0.00	0.00	0.00	29.00	0.00
0.00	0.00	0.00	0.00	0.00	0.00	0.00	0.00	0.00
0.00	0.00	0.00	0.00	29.00	0.00	0.00	0.00	0.00
0.00	0.00	0.00	0.00	0.00	0.00	0.00	0.00	0.00
0.00	0.00	0.00	0.00	0.00	0.00	0.00	0.00	0.00
0.00	0.00	0.00	0.00	0.00	0.00	0.00	0.00	0.00
0.00	0.00	0.00	0.00	0.00	0.00	0.00	0.00	0.00
0.00	0.00	0.00	0.00	0.00	0.00	0.00	0.00	0.00
0.00	0.00	0.00	0.00	0.00	0.00	0.00	0.00	0.00
0.00	0.00	0.00	0.00	0.00	0.00	0.00	0.00	0.00
0.00	0.00	0.00	0.00	0.00	0.00	0.00	0.00	0.00
0.00	0.00	0.00	0.00	0.00	0.00	0.00	0.00	0.00
0.00	0.00	0.00	0.00	0.00	0.00	0.00	0.00	0.00
0.00	0.00	0.00	0.00	0.00	0.00	0.00	0.00	0.00
0.00	0.00	0.00	0.00	0.00	0.00	0.00	0.00	0.00
0.00	0.00	0.00	0.00	0.00	0.00	0.00	0.00	0.00
0.00	0.00	0.00	0.00	0.00	0.00	0.00	0.00	0.00
0.00	0.00	0.00	0.00	0.00	0.00	0.00	0.00	0.00
0.00	0.00	0.00	9.00	0.00	0.00	18.00	0.00	0.00
0.00	0.00	0.00	0.00	0.00	0.00	0.00	0.00	0.00
0.00	0.00	0.00	0.00	0.00	0.00	0.00	0.00	0.00

0.00	0.00	0.00	0.00	0.00	0.00	0.00	0.00	0.00
0.00	0.00	0.00	0.00	0.00	0.00	0.00	0.00	0.00
0.00	0.00	0.00	0.00	0.00	0.00	0.00	0.00	0.00
0.00	0.00	0.00	0.00	0.00	0.00	0.00	0.00	0.00
0.00	0.00	0.00	0.00	0.00	0.00	0.00	0.00	0.00
0.00	0.00	0.00	0.00	0.00	0.00	0.00	0.00	0.00
0.00	0.00	0.00	0.00	0.00	0.00	0.00	0.00	0.00
0.00	0.00	0.00	0.00	0.00	0.00	0.00	0.00	0.00
0.00	0.00	0.00	0.00	0.00	0.00	0.00	0.00	0.00
0.00	0.00	0.00	0.00	0.00	0.00	0.00	0.00	0.00
0.00	0.00	0.00	0.00	0.00	0.00	0.00	0.00	0.00
0.00	0.00	0.00	0.00	0.00	0.00	0.00	0.00	0.00
0.00	0.00	0.00	0.00	0.00	0.00	0.00	0.00	0.00
0.00	0.00	0.00	0.00	0.00	0.00	0.00	0.00	0.00
0.00	0.00	0.00	0.00	0.00	0.00	0.00	0.00	0.00
0.00	0.00	0.00	0.00	0.00	0.00	0.00	0.00	0.00
0.00	0.00	0.00	0.00	0.00	0.00	0.00	0.00	0.00
0.00	0.00	0.00	0.00	0.00	0.00	0.00	0.00	0.00
0.00	0.00	0.00	0.00	0.00	0.00	0.00	0.00	0.00
0.00	0.00	0.00	0.00	0.00	0.00	0.00	0.00	0.00
0.00	0.00	0.00	0.00	0.00	0.00	0.00	0.00	0.00
0.00	0.00	0.00	0.00	0.00	0.00	0.00	0.00	0.00
0.00	0.00	0.00	0.00	0.00	0.00	0.00	0.00	17.00
0.00	0.00	0.00	0.00	0.00	0.00	0.00	0.00	0.00
0.00	0.00	0.00	0.00	0.00	0.00	0.00	0.00	0.00
0.00	0.00	0.00	0.00	0.00	0.00	0.00	0.00	0.00
0.00	0.00	0.00	0.00	0.00	0.00	0.00	0.00	0.00
0.00	0.00	0.00	0.00	0.00	0.00	0.00	0.00	0.00
0.00	0.00	0.00	0.00	0.00	0.00	0.00	0.00	0.00
0.00	0.00	0.00	0.00	0.00	0.00	0.00	0.00	0.00
0.00	0.00	0.00	0.00	0.00	0.00	0.00	0.00	0.00
0.00	0.00	0.00	0.00	0.00	0.00	0.00	0.00	0.00
25.00	0.00	0.00	0.00	0.00	0.00	0.00	0.00	0.00
0.00	0.00	0.00	0.00	0.00	0.00	0.00	0.00	0.00
0.00	0.00	0.00	0.00	25.00	0.00	0.00	0.00	0.00
0.00	0.00	0.00	0.00	0.00	0.00	0.00	0.00	0.00
0.00	0.00	0.00	0.00	0.00	0.00	0.00	0.00	0.00

0.00	0.00	0.00	0.00	0.00	0.00	0.00	0.00	0.00
0.00	0.00	0.00	0.00	0.00	0.00	0.00	0.00	0.00
0.00	0.00	0.00	0.00	0.00	0.00	0.00	0.00	0.00
0.00	0.00	0.00	0.00	0.00	0.00	0.00	0.00	0.00
0.00	0.00	0.00	0.00	0.00	0.00	0.00	0.00	0.00
0.00	0.00	0.00	0.00	0.00	0.00	0.00	0.00	0.00
0.00	0.00	0.00	0.00	0.00	0.00	0.00	0.00	0.00
0.00	0.00	0.00	0.00	0.00	0.00	0.00	0.00	0.00
0.00	0.00	0.00	0.00	0.00	0.00	0.00	0.00	0.00
0.00	0.00	0.00	0.00	0.00	0.00	0.00	0.00	0.00
0.00	0.00	0.00	0.00	0.00	0.00	0.00	0.00	0.00
0.00	0.00	0.00	0.00	0.00	0.00	0.00	0.00	0.00
0.00	0.00	0.00	4.00	0.00	0.00	0.00	0.00	0.00
0.00	0.00	0.00	0.00	0.00	0.00	0.00	0.00	0.00
0.00	0.00	0.00	0.00	0.00	0.00	0.00	0.00	0.00
0.00	0.00	0.00	0.00	0.00	0.00	0.00	0.00	0.00
0.00	0.00	0.00	0.00	0.00	0.00	0.00	0.00	0.00
0.00	0.00	9.00	0.00	0.00	0.00	0.00	0.00	0.00
0.00	0.00	0.00	0.00	0.00	0.00	0.00	0.00	0.00
0.00	0.00	0.00	0.00	0.00	0.00	0.00	0.00	0.00
0.00	0.00	0.00	0.00	0.00	0.00	0.00	0.00	0.00
0.00	0.00	0.00	0.00	0.00	0.00	0.00	0.00	0.00
0.00	0.00	0.00	0.00	0.00	0.00	0.00	0.00	0.00
0.00	0.00	0.00	0.00	0.00	0.00	0.00	0.00	0.00
0.00	0.00	0.00	0.00	0.00	0.00	0.00	0.00	0.00
0.00	0.00	0.00	0.00	0.00	0.00	0.00	0.00	0.00
0.00	0.00	0.00	0.00	0.00	0.00	0.00	0.00	0.00
0.00	0.00	0.00	0.00	0.00	0.00	0.00	0.00	0.00
0.00	0.00	0.00	0.00	0.00	0.00	0.00	0.00	0.00
0.00	0.00	0.00	0.00	0.00	0.00	0.00	0.00	0.00
0.00	0.00	0.00	0.00	0.00	0.00	0.00	0.00	0.00
0.00	0.00	0.00	0.00	0.00	0.00	0.00	0.00	0.00
0.00	0.00	0.00	0.00	0.00	0.00	0.00	0.00	0.00
0.00	0.00	0.00	0.00	0.00	0.00	0.00	0.00	0.00
0.00	0.00	0.00	0.00	0.00	0.00	0.00	0.00	0.00
0.00	0.00	0.00	0.00	0.00	0.00	0.00	0.00	0.00
0.00	0.00	0.00	0.00	0.00	0.00	0.00	0.00	0.00

0.00	0.00	0.00	0.00	0.00	0.00	0.00	0.00	0.00
0.00	0.00	0.00	0.00	0.00	0.00	0.00	0.00	0.00
0.00	0.00	0.00	0.00	0.00	0.00	0.00	0.00	0.00
0.00	0.00	0.00	0.00	0.00	0.00	0.00	0.00	0.00
0.00	0.00	0.00	0.00	0.00	0.00	0.00	0.00	0.00
0.00	0.00	0.00	0.00	0.00	0.00	0.00	0.00	0.00
0.00	0.00	0.00	0.00	0.00	0.00	0.00	7.00	0.00
0.00	0.00	0.00	0.00	0.00	0.00	0.00	0.00	0.00
0.00	0.00	0.00	0.00	0.00	0.00	0.00	0.00	0.00
0.00	0.00	0.00	0.00	0.00	0.00	0.00	0.00	0.00
0.00	0.00	0.00	0.00	0.00	0.00	0.00	0.00	0.00
0.00	0.00	0.00	0.00	0.00	0.00	0.00	0.00	0.00
0.00	0.00	0.00	0.00	0.00	0.00	0.00	0.00	0.00
0.00	0.00	0.00	0.00	0.00	0.00	0.00	0.00	0.00
0.00	0.00	0.00	0.00	0.00	0.00	0.00	0.00	0.00
0.00	0.00	0.00	0.00	0.00	0.00	0.00	0.00	0.00
0.00	0.00	0.00	0.00	0.00	0.00	0.00	0.00	0.00
0.00	0.00	0.00	0.00	0.00	0.00	0.00	0.00	0.00
0.00	0.00	0.00	0.00	0.00	0.00	0.00	0.00	0.00
0.00	0.00	0.00	0.00	0.00	0.00	0.00	0.00	0.00
0.00	0.00	0.00	0.00	0.00	0.00	0.00	0.00	0.00
0.00	0.00	0.00	0.00	0.00	0.00	0.00	0.00	0.00
0.00	0.00	0.00	0.00	0.00	0.00	0.00	0.00	0.00
0.00	0.00	0.00	0.00	0.00	0.00	0.00	0.00	0.00
0.00	0.00	0.00	0.00	0.00	0.00	0.00	0.00	0.00
0.00	0.00	0.00	0.00	0.00	0.00	0.00	0.00	0.00
0.00	0.00	0.00	0.00	0.00	0.00	0.00	0.00	0.00
0.00	0.00	0.00	0.00	0.00	0.00	0.00	0.00	0.00
0.00	0.00	0.00	0.00	0.00	0.00	0.00	0.00	0.00
0.00	0.00	0.00	0.00	0.00	0.00	0.00	0.00	0.00
0.00	0.00	0.00	0.00	0.00	0.00	0.00	0.00	0.00
0.00	0.00	0.00	0.00	0.00	0.00	0.00	0.00	0.00
0.00	0.00	0.00	0.00	0.00	0.00	0.00	0.00	0.00
0.00	0.00	0.00	0.00	0.00	0.00	0.00	0.00	0.00
0.00	0.00	0.00	0.00	0.00	0.00	0.00	0.00	0.00
0.00	0.00	0.00	0.00	0.00	0.00	0.00	0.00	0.00
0.00	0.00	0.00	0.00	0.00	0.00	0.00	0.00	0.00

0.00	0.00	0.00	0.00	0.00	0.00	0.00	0.00	0.00
0.00	0.00	0.00	0.00	0.00	0.00	0.00	0.00	0.00
0.00	0.00	0.00	0.00	0.00	0.00	0.00	0.00	0.00
0.00	0.00	0.00	0.00	0.00	0.00	0.00	0.00	0.00
0.00	0.00	0.00	0.00	0.00	0.00	0.00	0.00	0.00
0.00	0.00	0.00	0.00	0.00	0.00	0.00	0.00	0.00
0.00	0.00	0.00	0.00	0.00	0.00	0.00	0.00	0.00
0.00	0.00	0.00	0.00	0.00	0.00	0.00	0.00	0.00
0.00	0.00	0.00	0.00	0.00	0.00	0.00	0.00	0.00
0.00	0.00	0.00	0.00	0.00	0.00	0.00	0.00	0.00
0.00	0.00	0.00	0.00	0.00	0.00	0.00	0.00	0.00
20.00	0.00	0.00	0.00	0.00	0.00	0.00	0.00	0.00
0.00	0.00	0.00	0.00	0.00	0.00	0.00	0.00	0.00
0.00	0.00	0.00	0.00	0.00	0.00	0.00	0.00	0.00
0.00	0.00	0.00	0.00	0.00	0.00	0.00	0.00	0.00
0.00	0.00	0.00	0.00	0.00	0.00	0.00	0.00	0.00
0.00	0.00	0.00	0.00	0.00	0.00	0.00	0.00	0.00
0.00	0.00	0.00	0.00	0.00	0.00	0.00	0.00	0.00
0.00	0.00	0.00	0.00	0.00	0.00	0.00	0.00	0.00
0.00	0.00	0.00	0.00	0.00	0.00	0.00	0.00	0.00
0.00	0.00	0.00	0.00	0.00	0.00	0.00	0.00	0.00
0.00	0.00	0.00	0.00	0.00	0.00	0.00	0.00	0.00
0.00	0.00	0.00	0.00	0.00	0.00	0.00	0.00	0.00
0.00	0.00	0.00	0.00	0.00	0.00	0.00	0.00	0.00
0.00	0.00	0.00	0.00	0.00	0.00	0.00	0.00	0.00
0.00	0.00	0.00	0.00	0.00	0.00	0.00	0.00	0.00
0.00	0.00	0.00	0.00	0.00	0.00	0.00	0.00	0.00
0.00	0.00	0.00	0.00	0.00	0.00	0.00	0.00	0.00
0.00	0.00	0.00	0.00	0.00	0.00	0.00	0.00	0.00
0.00	0.00	0.00	0.00	0.00	0.00	0.00	0.00	0.00
0.00	0.00	0.00	0.00	0.00	0.00	0.00	0.00	0.00
0.00	0.00	0.00	0.00	0.00	0.00	0.00	0.00	0.00
0.00	0.00	0.00	0.00	0.00	0.00	0.00	0.00	0.00
0.00	0.00	0.00	0.00	0.00	0.00	0.00	0.00	0.00
0.00	0.00	0.00	0.00	0.00	0.00	0.00	0.00	0.00
0.00	0.00	0.00	0.00	0.00	0.00	0.00	0.00	0.00
19.00	0.00	0.00	0.00	0.00	0.00	0.00	0.00	0.00

0.00	0.00	0.00	0.00	0.00	0.00	0.00	0.00	0.00
0.00	0.00	0.00	0.00	0.00	0.00	0.00	0.00	0.00
0.00	0.00	0.00	0.00	0.00	0.00	0.00	0.00	0.00
0.00	0.00	0.00	0.00	0.00	0.00	0.00	0.00	0.00
0.00	0.00	0.00	0.00	0.00	0.00	0.00	0.00	0.00
0.00	0.00	0.00	0.00	0.00	0.00	0.00	0.00	0.00
0.00	0.00	0.00	0.00	0.00	0.00	0.00	0.00	0.00
0.00	0.00	0.00	0.00	0.00	0.00	0.00	0.00	0.00
0.00	0.00	0.00	0.00	0.00	0.00	0.00	0.00	0.00
0.00	0.00	0.00	0.00	0.00	0.00	0.00	0.00	0.00
0.00	0.00	0.00	0.00	0.00	0.00	0.00	0.00	0.00
0.00	0.00	0.00	0.00	0.00	0.00	0.00	0.00	0.00
0.00	0.00	0.00	0.00	0.00	0.00	0.00	0.00	0.00
0.00	0.00	0.00	0.00	0.00	0.00	0.00	0.00	0.00
0.00	0.00	0.00	0.00	0.00	0.00	0.00	0.00	0.00
0.00	0.00	0.00	0.00	0.00	0.00	0.00	0.00	0.00
0.00	0.00	0.00	0.00	0.00	0.00	0.00	0.00	0.00
0.00	0.00	0.00	0.00	0.00	0.00	0.00	0.00	0.00
0.00	0.00	0.00	0.00	0.00	0.00	0.00	0.00	0.00
0.00	0.00	0.00	0.00	0.00	0.00	0.00	0.00	0.00
0.00	0.00	0.00	0.00	0.00	0.00	0.00	0.00	0.00
0.00	0.00	0.00	0.00	0.00	0.00	0.00	0.00	0.00
0.00	0.00	0.00	0.00	0.00	0.00	0.00	0.00	0.00
0.00	0.00	0.00	0.00	0.00	0.00	0.00	0.00	0.00
0.00	0.00	0.00	0.00	0.00	0.00	0.00	0.00	0.00
0.00	0.00	0.00	0.00	0.00	0.00	0.00	0.00	0.00
0.00	0.00	0.00	0.00	0.00	0.00	0.00	0.00	0.00
0.00	0.00	0.00	0.00	0.00	0.00	0.00	0.00	0.00
0.00	0.00	0.00	0.00	0.00	0.00	0.00	0.00	0.00
0.00	0.00	0.00	0.00	0.00	0.00	0.00	0.00	0.00
0.00	0.00	0.00	0.00	0.00	0.00	0.00	0.00	0.00
0.00	0.00	0.00	0.00	0.00	0.00	0.00	0.00	0.00
0.00	0.00	0.00	0.00	0.00	0.00	0.00	0.00	0.00
0.00	0.00	0.00	0.00	0.00	0.00	0.00	0.00	0.00
0.00	0.00	0.00	0.00	0.00	0.00	0.00	0.00	0.00
0.00	0.00	0.00	0.00	0.00	0.00	0.00	0.00	0.00
0.00	0.00	0.00	0.00	0.00	0.00	0.00	0.00	0.00

0.00	0.00	0.00	0.00	0.00	0.00	0.00	0.00	0.00
0.00	0.00	0.00	0.00	0.00	0.00	0.00	0.00	0.00
0.00	0.00	0.00	0.00	0.00	0.00	0.00	0.00	0.00
0.00	0.00	0.00	0.00	0.00	0.00	0.00	0.00	0.00
0.00	0.00	0.00	0.00	0.00	0.00	0.00	0.00	0.00
0.00	0.00	0.00	0.00	0.00	0.00	0.00	0.00	0.00
0.00	0.00	0.00	0.00	0.00	0.00	0.00	0.00	0.00
0.00	0.00	0.00	0.00	0.00	0.00	0.00	0.00	0.00
0.00	0.00	0.00	0.00	0.00	0.00	0.00	0.00	0.00
0.00	0.00	0.00	0.00	0.00	0.00	0.00	0.00	0.00
0.00	0.00	0.00	0.00	0.00	0.00	0.00	0.00	0.00
0.00	0.00	0.00	0.00	0.00	0.00	0.00	0.00	0.00
0.00	0.00	0.00	0.00	0.00	0.00	0.00	0.00	0.00
0.00	0.00	0.00	0.00	0.00	0.00	0.00	0.00	0.00
0.00	0.00	0.00	0.00	0.00	0.00	0.00	0.00	0.00
0.00	0.00	0.00	0.00	0.00	0.00	0.00	0.00	0.00
0.00	0.00	0.00	0.00	0.00	0.00	0.00	0.00	0.00
0.00	0.00	0.00	0.00	0.00	0.00	0.00	0.00	0.00
0.00	0.00	0.00	0.00	0.00	0.00	0.00	0.00	0.00
0.00	0.00	0.00	0.00	0.00	0.00	0.00	0.00	0.00
0.00	0.00	0.00	0.00	0.00	0.00	0.00	0.00	0.00
0.00	0.00	17.00	0.00	0.00	0.00	0.00	0.00	0.00
0.00	0.00	0.00	0.00	0.00	0.00	0.00	0.00	0.00
0.00	0.00	0.00	0.00	0.00	0.00	0.00	0.00	0.00
0.00	0.00	0.00	0.00	0.00	0.00	0.00	0.00	0.00
0.00	0.00	0.00	0.00	0.00	0.00	0.00	0.00	0.00
0.00	0.00	0.00	0.00	0.00	0.00	0.00	0.00	0.00
0.00	0.00	0.00	0.00	0.00	0.00	0.00	0.00	0.00
0.00	0.00	0.00	0.00	0.00	0.00	0.00	0.00	0.00
0.00	0.00	0.00	0.00	0.00	0.00	0.00	0.00	0.00
0.00	0.00	0.00	0.00	0.00	0.00	0.00	0.00	0.00
0.00	0.00	0.00	0.00	0.00	0.00	0.00	0.00	0.00
0.00	0.00	0.00	0.00	0.00	0.00	0.00	0.00	0.00
0.00	0.00	0.00	0.00	0.00	0.00	0.00	0.00	0.00
0.00	0.00	0.00	0.00	0.00	0.00	0.00	0.00	0.00
0.00	0.00	0.00	0.00	0.00	0.00	0.00	0.00	0.00
0.00	0.00	0.00	0.00	0.00	0.00	0.00	0.00	0.00

0.00	0.00	0.00	0.00	0.00	0.00	0.00	0.00	0.00
0.00	0.00	0.00	0.00	0.00	0.00	0.00	0.00	0.00
0.00	0.00	0.00	0.00	0.00	0.00	0.00	0.00	0.00
0.00	0.00	0.00	0.00	0.00	0.00	0.00	0.00	0.00
0.00	0.00	0.00	0.00	0.00	0.00	0.00	0.00	0.00
0.00	0.00	0.00	0.00	0.00	0.00	0.00	0.00	0.00
0.00	0.00	0.00	0.00	0.00	0.00	0.00	0.00	0.00
0.00	0.00	0.00	0.00	0.00	0.00	0.00	0.00	0.00
0.00	0.00	0.00	0.00	0.00	0.00	0.00	0.00	0.00
0.00	0.00	0.00	0.00	0.00	0.00	0.00	0.00	0.00
0.00	0.00	0.00	0.00	0.00	0.00	0.00	0.00	0.00
0.00	16.00	0.00	0.00	0.00	0.00	0.00	0.00	0.00
0.00	0.00	0.00	0.00	0.00	0.00	0.00	0.00	0.00
0.00	0.00	0.00	0.00	0.00	0.00	0.00	0.00	0.00
0.00	0.00	0.00	0.00	0.00	0.00	0.00	0.00	0.00
0.00	0.00	0.00	0.00	0.00	0.00	0.00	0.00	0.00
0.00	0.00	0.00	0.00	0.00	0.00	0.00	0.00	0.00
0.00	0.00	0.00	0.00	0.00	0.00	0.00	0.00	0.00
0.00	0.00	0.00	0.00	0.00	0.00	0.00	0.00	0.00
0.00	0.00	0.00	0.00	0.00	0.00	0.00	0.00	0.00
0.00	0.00	0.00	0.00	0.00	0.00	0.00	0.00	0.00
0.00	0.00	0.00	0.00	0.00	0.00	0.00	0.00	0.00
0.00	0.00	0.00	0.00	0.00	0.00	0.00	0.00	0.00
0.00	0.00	0.00	0.00	0.00	0.00	0.00	0.00	0.00
0.00	0.00	0.00	0.00	0.00	0.00	0.00	0.00	0.00
0.00	0.00	0.00	0.00	0.00	0.00	0.00	0.00	0.00
0.00	0.00	0.00	0.00	0.00	0.00	0.00	0.00	0.00
0.00	0.00	0.00	0.00	0.00	0.00	0.00	0.00	0.00
0.00	0.00	0.00	0.00	0.00	0.00	0.00	0.00	0.00
0.00	0.00	0.00	0.00	0.00	0.00	0.00	0.00	0.00
0.00	0.00	0.00	0.00	0.00	0.00	0.00	0.00	0.00
0.00	0.00	0.00	0.00	0.00	0.00	0.00	0.00	0.00
0.00	0.00	0.00	0.00	0.00	0.00	0.00	0.00	0.00
0.00	0.00	0.00	0.00	0.00	0.00	0.00	0.00	0.00
0.00	0.00	0.00	0.00	0.00	0.00	0.00	0.00	0.00
0.00	0.00	0.00	0.00	0.00	0.00	0.00	0.00	0.00
0.00	0.00	0.00	0.00	0.00	0.00	0.00	0.00	0.00

0.00	0.00	0.00	0.00	0.00	0.00	0.00	0.00	0.00
0.00	0.00	0.00	0.00	0.00	0.00	0.00	0.00	0.00
0.00	0.00	0.00	0.00	0.00	0.00	0.00	0.00	0.00
0.00	0.00	0.00	0.00	0.00	0.00	0.00	0.00	0.00
15.00	0.00	0.00	0.00	0.00	0.00	0.00	0.00	0.00
2.00	0.00	0.00	0.00	0.00	0.00	0.00	0.00	0.00
0.00	0.00	0.00	0.00	0.00	0.00	0.00	0.00	0.00
0.00	0.00	0.00	0.00	0.00	0.00	0.00	0.00	0.00
0.00	0.00	0.00	0.00	0.00	0.00	0.00	0.00	0.00
0.00	0.00	0.00	0.00	0.00	0.00	0.00	0.00	0.00
0.00	0.00	0.00	0.00	0.00	0.00	0.00	0.00	0.00
0.00	0.00	0.00	0.00	0.00	0.00	0.00	0.00	0.00
0.00	0.00	0.00	0.00	0.00	0.00	0.00	0.00	0.00
0.00	0.00	0.00	0.00	0.00	0.00	0.00	0.00	0.00
0.00	0.00	0.00	0.00	0.00	0.00	0.00	0.00	0.00
0.00	0.00	0.00	0.00	0.00	0.00	0.00	0.00	0.00
0.00	0.00	0.00	0.00	0.00	0.00	0.00	0.00	0.00
0.00	0.00	0.00	0.00	0.00	0.00	0.00	0.00	0.00
0.00	0.00	0.00	0.00	0.00	0.00	0.00	0.00	0.00
0.00	0.00	0.00	0.00	0.00	0.00	0.00	0.00	0.00
0.00	0.00	0.00	0.00	0.00	0.00	0.00	0.00	0.00
0.00	0.00	0.00	0.00	0.00	0.00	0.00	0.00	0.00
0.00	0.00	0.00	0.00	0.00	0.00	0.00	0.00	0.00
0.00	0.00	0.00	0.00	0.00	0.00	0.00	0.00	0.00
0.00	0.00	0.00	0.00	0.00	0.00	0.00	0.00	0.00
0.00	0.00	0.00	0.00	0.00	0.00	0.00	0.00	0.00
0.00	0.00	0.00	0.00	0.00	0.00	0.00	0.00	0.00
0.00	0.00	0.00	0.00	0.00	0.00	0.00	0.00	0.00
0.00	0.00	0.00	0.00	0.00	0.00	0.00	0.00	0.00
0.00	0.00	0.00	0.00	0.00	0.00	0.00	0.00	0.00
0.00	0.00	0.00	0.00	0.00	0.00	0.00	0.00	0.00
0.00	0.00	0.00	0.00	0.00	0.00	0.00	0.00	0.00
0.00	0.00	0.00	0.00	0.00	0.00	0.00	14.00	0.00
0.00	0.00	0.00	0.00	0.00	0.00	0.00	14.00	0.00
0.00	0.00	0.00	0.00	0.00	0.00	0.00	0.00	0.00
0.00	0.00	0.00	0.00	0.00	0.00	0.00	0.00	0.00
0.00	0.00	0.00	0.00	0.00	0.00	0.00	0.00	0.00

0.00	0.00	14.00	0.00	0.00	0.00	0.00	0.00	0.00
0.00	0.00	0.00	0.00	14.00	0.00	0.00	0.00	0.00
0.00	0.00	0.00	0.00	0.00	0.00	0.00	0.00	0.00
0.00	0.00	0.00	0.00	0.00	0.00	0.00	0.00	0.00
0.00	0.00	0.00	0.00	0.00	0.00	0.00	0.00	0.00
0.00	0.00	0.00	0.00	0.00	0.00	0.00	0.00	0.00
0.00	0.00	0.00	0.00	0.00	0.00	0.00	0.00	0.00
0.00	0.00	0.00	0.00	0.00	0.00	0.00	0.00	0.00
0.00	0.00	0.00	0.00	0.00	0.00	0.00	0.00	0.00
0.00	0.00	0.00	0.00	0.00	0.00	0.00	0.00	0.00
0.00	0.00	0.00	0.00	0.00	0.00	0.00	0.00	0.00
0.00	0.00	0.00	0.00	0.00	0.00	0.00	0.00	0.00
0.00	0.00	0.00	0.00	0.00	0.00	0.00	0.00	0.00
0.00	0.00	0.00	0.00	0.00	0.00	0.00	0.00	0.00
0.00	0.00	0.00	0.00	0.00	0.00	0.00	0.00	0.00
0.00	0.00	0.00	0.00	0.00	0.00	0.00	0.00	0.00
0.00	0.00	0.00	0.00	0.00	0.00	0.00	0.00	0.00
0.00	0.00	0.00	0.00	0.00	0.00	0.00	0.00	0.00
0.00	0.00	0.00	0.00	0.00	0.00	0.00	0.00	0.00
0.00	0.00	0.00	0.00	0.00	0.00	0.00	13.00	0.00
0.00	0.00	0.00	0.00	0.00	0.00	0.00	0.00	13.00
0.00	0.00	0.00	0.00	0.00	0.00	0.00	0.00	0.00
0.00	0.00	0.00	0.00	0.00	0.00	0.00	0.00	0.00
0.00	0.00	0.00	0.00	0.00	0.00	0.00	0.00	0.00
0.00	0.00	0.00	0.00	0.00	0.00	0.00	0.00	0.00
0.00	0.00	0.00	8.00	0.00	0.00	0.00	0.00	0.00
0.00	0.00	0.00	0.00	0.00	0.00	0.00	0.00	0.00
0.00	0.00	0.00	0.00	0.00	0.00	0.00	0.00	0.00
0.00	0.00	0.00	0.00	0.00	0.00	0.00	0.00	0.00
0.00	0.00	0.00	0.00	0.00	0.00	0.00	0.00	0.00
0.00	0.00	0.00	0.00	0.00	0.00	0.00	0.00	0.00
0.00	0.00	0.00	0.00	0.00	0.00	0.00	0.00	0.00
0.00	0.00	0.00	0.00	0.00	0.00	0.00	0.00	0.00
0.00	0.00	0.00	0.00	0.00	0.00	0.00	0.00	0.00
0.00	0.00	0.00	0.00	0.00	0.00	0.00	0.00	0.00
0.00	0.00	0.00	0.00	0.00	0.00	0.00	0.00	0.00
0.00	0.00	0.00	0.00	0.00	0.00	0.00	0.00	0.00

0.00	0.00	0.00	0.00	0.00	0.00	0.00	0.00	0.00
0.00	0.00	0.00	0.00	0.00	0.00	0.00	0.00	0.00
0.00	0.00	0.00	0.00	0.00	0.00	0.00	0.00	0.00
0.00	0.00	0.00	0.00	0.00	0.00	0.00	0.00	0.00
0.00	0.00	0.00	0.00	0.00	0.00	0.00	0.00	0.00
0.00	0.00	0.00	0.00	0.00	0.00	0.00	0.00	0.00
0.00	0.00	0.00	0.00	0.00	0.00	0.00	0.00	0.00
0.00	0.00	0.00	0.00	0.00	0.00	0.00	0.00	0.00
0.00	0.00	0.00	0.00	0.00	0.00	0.00	0.00	0.00
0.00	0.00	0.00	0.00	0.00	0.00	0.00	0.00	0.00
0.00	0.00	0.00	0.00	0.00	0.00	0.00	0.00	0.00
0.00	0.00	0.00	0.00	0.00	0.00	0.00	0.00	0.00
0.00	0.00	0.00	0.00	0.00	0.00	0.00	0.00	0.00
0.00	0.00	0.00	0.00	0.00	0.00	0.00	0.00	0.00
0.00	0.00	0.00	0.00	0.00	0.00	0.00	0.00	0.00
0.00	0.00	0.00	0.00	0.00	0.00	0.00	0.00	0.00
0.00	0.00	0.00	0.00	0.00	0.00	0.00	0.00	0.00
0.00	0.00	0.00	0.00	0.00	0.00	0.00	0.00	0.00
12.00	0.00	0.00	0.00	0.00	0.00	0.00	0.00	0.00
12.00	0.00	0.00	0.00	0.00	0.00	0.00	0.00	0.00
0.00	0.00	0.00	0.00	0.00	0.00	0.00	12.00	0.00
0.00	0.00	0.00	0.00	0.00	0.00	0.00	0.00	12.00
0.00	0.00	0.00	0.00	0.00	0.00	0.00	0.00	12.00
0.00	0.00	0.00	0.00	0.00	0.00	0.00	0.00	0.00
0.00	0.00	0.00	0.00	0.00	0.00	0.00	0.00	0.00
0.00	0.00	0.00	0.00	0.00	0.00	0.00	0.00	0.00
0.00	0.00	0.00	0.00	0.00	0.00	0.00	0.00	0.00
0.00	0.00	0.00	0.00	0.00	0.00	0.00	0.00	0.00
0.00	0.00	0.00	0.00	0.00	0.00	0.00	0.00	0.00
0.00	0.00	0.00	0.00	0.00	0.00	0.00	0.00	0.00
0.00	0.00	0.00	0.00	0.00	0.00	0.00	0.00	0.00
0.00	0.00	0.00	0.00	12.00	0.00	0.00	0.00	0.00
0.00	0.00	0.00	0.00	12.00	0.00	0.00	0.00	0.00
0.00	0.00	0.00	0.00	0.00	0.00	0.00	0.00	0.00
0.00	0.00	0.00	0.00	0.00	0.00	0.00	0.00	0.00
0.00	0.00	0.00	0.00	0.00	0.00	0.00	0.00	0.00
0.00	0.00	0.00	0.00	0.00	0.00	0.00	0.00	0.00

0.00	0.00	0.00	0.00	0.00	0.00	0.00	0.00	0.00
0.00	0.00	0.00	0.00	0.00	0.00	0.00	0.00	0.00
0.00	0.00	0.00	0.00	0.00	0.00	0.00	0.00	0.00
0.00	0.00	0.00	0.00	0.00	0.00	0.00	0.00	0.00
0.00	0.00	0.00	0.00	0.00	0.00	0.00	0.00	0.00
0.00	0.00	0.00	0.00	0.00	0.00	0.00	0.00	0.00
0.00	0.00	0.00	0.00	0.00	0.00	0.00	0.00	0.00
0.00	0.00	0.00	0.00	0.00	0.00	0.00	0.00	0.00
0.00	0.00	0.00	0.00	0.00	0.00	0.00	0.00	0.00
0.00	0.00	0.00	0.00	0.00	0.00	0.00	0.00	0.00
0.00	0.00	0.00	0.00	0.00	0.00	0.00	0.00	0.00
0.00	0.00	0.00	0.00	0.00	0.00	0.00	0.00	0.00
0.00	0.00	0.00	0.00	0.00	0.00	0.00	0.00	0.00
0.00	0.00	0.00	0.00	0.00	0.00	0.00	0.00	0.00
0.00	0.00	0.00	0.00	0.00	0.00	0.00	0.00	0.00
0.00	0.00	0.00	0.00	0.00	0.00	0.00	0.00	0.00
0.00	0.00	0.00	0.00	0.00	0.00	0.00	0.00	0.00
0.00	0.00	0.00	0.00	0.00	0.00	0.00	0.00	0.00
0.00	0.00	0.00	0.00	0.00	0.00	0.00	0.00	0.00
0.00	0.00	0.00	0.00	0.00	0.00	0.00	0.00	0.00
0.00	0.00	0.00	0.00	0.00	0.00	0.00	0.00	0.00
0.00	0.00	0.00	0.00	0.00	0.00	0.00	0.00	0.00
0.00	0.00	0.00	0.00	0.00	0.00	0.00	0.00	0.00
0.00	0.00	0.00	0.00	0.00	0.00	0.00	0.00	0.00
0.00	0.00	0.00	0.00	0.00	0.00	0.00	0.00	0.00
0.00	0.00	0.00	0.00	0.00	0.00	0.00	0.00	0.00
0.00	0.00	0.00	0.00	0.00	0.00	0.00	0.00	0.00
0.00	0.00	0.00	0.00	0.00	0.00	0.00	0.00	0.00
2.00	0.00	0.00	0.00	0.00	0.00	0.00	0.00	6.00
0.00	0.00	0.00	0.00	0.00	0.00	0.00	0.00	8.00
0.00	0.00	0.00	0.00	0.00	0.00	0.00	0.00	0.00
0.00	0.00	0.00	0.00	0.00	0.00	0.00	0.00	0.00
0.00	0.00	0.00	0.00	0.00	0.00	0.00	0.00	0.00
0.00	0.00	0.00	0.00	0.00	0.00	0.00	0.00	0.00
0.00	0.00	0.00	0.00	0.00	0.00	0.00	0.00	0.00
0.00	0.00	0.00	0.00	0.00	0.00	0.00	0.00	0.00
0.00	0.00	0.00	0.00	0.00	0.00	0.00	0.00	0.00

0.00	0.00	0.00	0.00	0.00	0.00	0.00	0.00	0.00
0.00	0.00	0.00	0.00	0.00	0.00	0.00	0.00	0.00
0.00	0.00	0.00	0.00	0.00	0.00	0.00	0.00	0.00
0.00	0.00	0.00	0.00	0.00	0.00	0.00	0.00	0.00
0.00	0.00	0.00	0.00	0.00	0.00	0.00	0.00	0.00
0.00	0.00	0.00	0.00	0.00	0.00	0.00	0.00	0.00
0.00	0.00	0.00	0.00	0.00	0.00	0.00	0.00	0.00
0.00	0.00	0.00	0.00	0.00	0.00	0.00	0.00	0.00
0.00	0.00	0.00	0.00	0.00	0.00	0.00	0.00	0.00
0.00	0.00	0.00	0.00	0.00	0.00	0.00	0.00	0.00
0.00	0.00	0.00	0.00	0.00	0.00	0.00	0.00	0.00
0.00	0.00	0.00	0.00	0.00	0.00	0.00	0.00	0.00
0.00	0.00	0.00	0.00	0.00	0.00	0.00	0.00	0.00
0.00	0.00	0.00	0.00	0.00	0.00	0.00	0.00	0.00
0.00	0.00	0.00	0.00	0.00	0.00	0.00	0.00	0.00
0.00	0.00	0.00	0.00	0.00	0.00	0.00	0.00	0.00
0.00	0.00	0.00	0.00	0.00	0.00	0.00	0.00	0.00
0.00	0.00	0.00	0.00	0.00	0.00	0.00	0.00	0.00
0.00	0.00	0.00	0.00	0.00	0.00	0.00	0.00	0.00
0.00	0.00	0.00	0.00	0.00	0.00	0.00	0.00	0.00
0.00	0.00	0.00	0.00	0.00	0.00	0.00	0.00	0.00
0.00	0.00	0.00	0.00	0.00	0.00	0.00	0.00	0.00
0.00	0.00	0.00	0.00	0.00	0.00	0.00	0.00	0.00
0.00	0.00	0.00	0.00	0.00	0.00	0.00	0.00	0.00
0.00	0.00	0.00	0.00	0.00	0.00	0.00	0.00	0.00
0.00	0.00	0.00	0.00	0.00	0.00	0.00	0.00	0.00
0.00	0.00	0.00	0.00	0.00	0.00	0.00	0.00	0.00
0.00	0.00	0.00	0.00	0.00	0.00	0.00	0.00	0.00
0.00	0.00	0.00	0.00	0.00	0.00	0.00	0.00	0.00
0.00	0.00	0.00	0.00	0.00	0.00	0.00	0.00	0.00
0.00	0.00	0.00	0.00	0.00	0.00	0.00	0.00	0.00
10.00	0.00	0.00	0.00	0.00	0.00	0.00	0.00	0.00
0.00	0.00	0.00	0.00	0.00	3.00	0.00	4.00	0.00
0.00	0.00	0.00	0.00	0.00	0.00	0.00	0.00	0.00
0.00	0.00	0.00	0.00	0.00	0.00	0.00	0.00	0.00
0.00	0.00	0.00	0.00	0.00	0.00	0.00	0.00	0.00
0.00	0.00	0.00	0.00	0.00	0.00	0.00	0.00	0.00

0.00	0.00	0.00	0.00	0.00	0.00	0.00	0.00	0.00
0.00	0.00	0.00	0.00	0.00	0.00	0.00	0.00	0.00
0.00	0.00	0.00	0.00	0.00	0.00	0.00	0.00	0.00
0.00	0.00	0.00	0.00	0.00	0.00	0.00	0.00	0.00
0.00	0.00	0.00	0.00	0.00	0.00	0.00	0.00	0.00
0.00	0.00	0.00	0.00	0.00	0.00	0.00	0.00	0.00
0.00	0.00	0.00	0.00	0.00	0.00	0.00	0.00	0.00
0.00	0.00	0.00	0.00	0.00	0.00	0.00	0.00	0.00
0.00	0.00	0.00	0.00	0.00	0.00	0.00	0.00	0.00
0.00	0.00	0.00	0.00	0.00	0.00	0.00	0.00	0.00
0.00	0.00	0.00	0.00	0.00	0.00	0.00	0.00	0.00
0.00	0.00	0.00	0.00	0.00	0.00	0.00	0.00	0.00
0.00	0.00	0.00	0.00	0.00	0.00	0.00	0.00	0.00
0.00	0.00	0.00	0.00	0.00	0.00	0.00	0.00	0.00
0.00	0.00	0.00	0.00	0.00	0.00	0.00	0.00	0.00
0.00	0.00	0.00	0.00	0.00	0.00	0.00	0.00	0.00
0.00	0.00	0.00	0.00	0.00	0.00	0.00	0.00	0.00
0.00	0.00	0.00	0.00	0.00	0.00	0.00	0.00	0.00
0.00	0.00	0.00	0.00	0.00	0.00	0.00	0.00	0.00
0.00	0.00	0.00	0.00	0.00	0.00	0.00	0.00	0.00
0.00	0.00	0.00	0.00	0.00	0.00	0.00	0.00	0.00
0.00	0.00	0.00	0.00	0.00	0.00	0.00	0.00	0.00
0.00	0.00	0.00	0.00	0.00	0.00	0.00	0.00	0.00
0.00	0.00	0.00	0.00	0.00	0.00	0.00	0.00	0.00
0.00	0.00	0.00	0.00	0.00	0.00	0.00	0.00	0.00
0.00	0.00	0.00	0.00	0.00	0.00	0.00	0.00	0.00
0.00	0.00	0.00	0.00	0.00	0.00	0.00	0.00	0.00
0.00	0.00	0.00	0.00	0.00	0.00	0.00	0.00	0.00
0.00	0.00	0.00	0.00	0.00	0.00	0.00	0.00	0.00
0.00	0.00	0.00	0.00	0.00	0.00	0.00	0.00	0.00
0.00	0.00	0.00	0.00	0.00	0.00	0.00	0.00	0.00
0.00	0.00	0.00	0.00	0.00	0.00	0.00	0.00	0.00
0.00	0.00	0.00	0.00	0.00	0.00	0.00	0.00	0.00
0.00	0.00	0.00	0.00	0.00	0.00	0.00	0.00	0.00
0.00	0.00	0.00	0.00	0.00	0.00	0.00	0.00	0.00
0.00	0.00	0.00	0.00	0.00	0.00	0.00	0.00	0.00
0.00	0.00	0.00	0.00	0.00	0.00	0.00	0.00	0.00

0.00	0.00	0.00	0.00	0.00	0.00	0.00	0.00	0.00
0.00	0.00	0.00	0.00	0.00	0.00	0.00	0.00	0.00
0.00	0.00	0.00	0.00	0.00	0.00	0.00	0.00	0.00
0.00	0.00	0.00	0.00	0.00	0.00	0.00	0.00	0.00
0.00	0.00	0.00	0.00	0.00	0.00	0.00	0.00	0.00
0.00	0.00	0.00	0.00	0.00	0.00	0.00	0.00	0.00
0.00	0.00	0.00	0.00	0.00	0.00	0.00	0.00	0.00
0.00	0.00	0.00	0.00	0.00	0.00	0.00	0.00	0.00
0.00	0.00	0.00	0.00	0.00	0.00	0.00	0.00	0.00
0.00	3.00	0.00	4.00	0.00	0.00	0.00	0.00	0.00
0.00	0.00	0.00	0.00	0.00	0.00	0.00	9.00	0.00
0.00	0.00	0.00	0.00	0.00	0.00	0.00	0.00	0.00
0.00	0.00	0.00	0.00	0.00	0.00	0.00	0.00	0.00
0.00	0.00	0.00	0.00	0.00	0.00	0.00	0.00	0.00
0.00	0.00	0.00	0.00	0.00	0.00	0.00	0.00	0.00
0.00	0.00	0.00	0.00	0.00	0.00	0.00	0.00	0.00
0.00	0.00	0.00	0.00	0.00	0.00	0.00	0.00	0.00
0.00	0.00	0.00	0.00	0.00	0.00	0.00	0.00	0.00
0.00	0.00	0.00	0.00	0.00	0.00	0.00	0.00	0.00
0.00	0.00	0.00	0.00	0.00	0.00	0.00	0.00	0.00
0.00	0.00	0.00	0.00	0.00	0.00	0.00	0.00	0.00
0.00	0.00	0.00	0.00	0.00	0.00	0.00	0.00	0.00
0.00	0.00	0.00	0.00	0.00	0.00	0.00	0.00	0.00
0.00	0.00	9.00	0.00	0.00	0.00	0.00	0.00	0.00
0.00	0.00	0.00	0.00	0.00	0.00	0.00	0.00	0.00
0.00	0.00	0.00	0.00	0.00	0.00	0.00	0.00	0.00
0.00	0.00	0.00	0.00	0.00	0.00	0.00	0.00	0.00
0.00	0.00	0.00	0.00	0.00	0.00	0.00	0.00	0.00
0.00	0.00	0.00	0.00	0.00	0.00	0.00	0.00	0.00
0.00	0.00	0.00	0.00	0.00	0.00	0.00	0.00	0.00
0.00	0.00	0.00	0.00	0.00	0.00	0.00	0.00	0.00
0.00	0.00	0.00	0.00	0.00	0.00	0.00	0.00	0.00
0.00	0.00	0.00	0.00	0.00	0.00	0.00	0.00	0.00
0.00	0.00	0.00	0.00	0.00	0.00	0.00	0.00	0.00
0.00	0.00	0.00	0.00	0.00	0.00	0.00	0.00	0.00
0.00	0.00	0.00	0.00	0.00	0.00	0.00	0.00	0.00
0.00	0.00	0.00	0.00	0.00	0.00	0.00	0.00	0.00

0.00	0.00	0.00	0.00	0.00	0.00	0.00	0.00	0.00
0.00	0.00	0.00	0.00	0.00	0.00	0.00	0.00	0.00
0.00	0.00	0.00	0.00	0.00	0.00	0.00	0.00	0.00
0.00	0.00	0.00	0.00	0.00	0.00	0.00	0.00	0.00
0.00	0.00	0.00	0.00	0.00	0.00	0.00	0.00	0.00
0.00	0.00	0.00	0.00	0.00	0.00	0.00	0.00	0.00
0.00	0.00	0.00	0.00	0.00	0.00	0.00	0.00	0.00
0.00	0.00	0.00	0.00	0.00	0.00	0.00	0.00	0.00
0.00	0.00	0.00	0.00	0.00	0.00	0.00	0.00	0.00
0.00	0.00	0.00	0.00	0.00	0.00	0.00	0.00	0.00
0.00	0.00	0.00	0.00	0.00	0.00	0.00	0.00	0.00
0.00	0.00	0.00	0.00	0.00	0.00	0.00	0.00	0.00
0.00	0.00	0.00	0.00	0.00	0.00	0.00	0.00	0.00
0.00	0.00	0.00	0.00	0.00	0.00	0.00	0.00	0.00
0.00	0.00	0.00	0.00	0.00	0.00	0.00	0.00	0.00
0.00	0.00	0.00	0.00	0.00	0.00	0.00	0.00	0.00
0.00	0.00	0.00	0.00	0.00	0.00	0.00	0.00	0.00
0.00	0.00	0.00	0.00	0.00	0.00	0.00	0.00	0.00
0.00	0.00	0.00	0.00	0.00	0.00	0.00	0.00	0.00
0.00	0.00	0.00	0.00	0.00	0.00	0.00	0.00	0.00
0.00	0.00	0.00	0.00	0.00	0.00	0.00	0.00	0.00
0.00	0.00	0.00	0.00	0.00	0.00	0.00	0.00	0.00
0.00	0.00	0.00	0.00	0.00	0.00	0.00	0.00	0.00
0.00	0.00	0.00	0.00	0.00	0.00	0.00	0.00	0.00
0.00	0.00	0.00	0.00	0.00	0.00	0.00	0.00	0.00
0.00	0.00	0.00	0.00	0.00	0.00	0.00	0.00	0.00
0.00	0.00	0.00	0.00	0.00	0.00	0.00	0.00	0.00
0.00	0.00	0.00	0.00	0.00	0.00	0.00	0.00	0.00
0.00	0.00	0.00	0.00	0.00	0.00	0.00	0.00	0.00
0.00	0.00	0.00	0.00	0.00	0.00	0.00	0.00	0.00
0.00	0.00	0.00	0.00	0.00	0.00	0.00	0.00	0.00
0.00	0.00	0.00	0.00	0.00	0.00	0.00	0.00	0.00
0.00	0.00	0.00	0.00	0.00	0.00	0.00	0.00	0.00
0.00	0.00	0.00	0.00	0.00	0.00	0.00	0.00	0.00
0.00	0.00	0.00	0.00	0.00	0.00	0.00	0.00	0.00
5.00	0.00	0.00	0.00	0.00	3.00	0.00	0.00	0.00
0.00	0.00	0.00	0.00	0.00	5.00	0.00	0.00	0.00

0.00	0.00	0.00	0.00	0.00	0.00	0.00	0.00	8.00
0.00	0.00	0.00	0.00	0.00	0.00	0.00	0.00	0.00
0.00	0.00	0.00	0.00	0.00	0.00	0.00	0.00	0.00
0.00	0.00	0.00	0.00	0.00	0.00	0.00	0.00	0.00
0.00	0.00	0.00	0.00	0.00	0.00	0.00	0.00	0.00
0.00	0.00	0.00	0.00	0.00	0.00	0.00	0.00	0.00
0.00	0.00	0.00	0.00	0.00	0.00	0.00	0.00	0.00
0.00	0.00	0.00	0.00	0.00	0.00	0.00	0.00	0.00
0.00	0.00	0.00	0.00	0.00	0.00	0.00	0.00	0.00
0.00	0.00	0.00	0.00	0.00	0.00	0.00	0.00	0.00
0.00	0.00	0.00	0.00	0.00	0.00	0.00	0.00	0.00
0.00	0.00	0.00	0.00	0.00	0.00	0.00	0.00	0.00
0.00	0.00	0.00	0.00	0.00	0.00	0.00	0.00	0.00
0.00	0.00	0.00	0.00	0.00	0.00	0.00	0.00	0.00
0.00	0.00	0.00	0.00	0.00	0.00	0.00	0.00	0.00
0.00	0.00	0.00	0.00	0.00	0.00	0.00	0.00	0.00
0.00	0.00	0.00	0.00	0.00	0.00	0.00	0.00	0.00
0.00	0.00	0.00	0.00	0.00	0.00	0.00	0.00	0.00
0.00	0.00	0.00	0.00	0.00	0.00	0.00	0.00	0.00
0.00	0.00	0.00	0.00	0.00	0.00	0.00	0.00	0.00
0.00	0.00	0.00	0.00	0.00	0.00	0.00	0.00	0.00
0.00	0.00	0.00	0.00	0.00	0.00	0.00	0.00	0.00
0.00	0.00	0.00	0.00	0.00	0.00	0.00	0.00	0.00
0.00	0.00	0.00	0.00	0.00	0.00	0.00	0.00	0.00
0.00	0.00	0.00	0.00	0.00	0.00	0.00	0.00	0.00
0.00	0.00	0.00	0.00	0.00	0.00	0.00	0.00	0.00
0.00	0.00	0.00	0.00	0.00	0.00	0.00	0.00	0.00
0.00	0.00	0.00	0.00	0.00	0.00	0.00	0.00	0.00
0.00	0.00	0.00	0.00	0.00	0.00	0.00	0.00	0.00
0.00	0.00	0.00	0.00	0.00	0.00	0.00	0.00	0.00
0.00	0.00	0.00	0.00	0.00	0.00	0.00	0.00	0.00
0.00	0.00	0.00	0.00	0.00	0.00	0.00	0.00	0.00
0.00	0.00	0.00	0.00	0.00	0.00	0.00	0.00	0.00
0.00	0.00	0.00	0.00	0.00	0.00	0.00	0.00	0.00
0.00	0.00	0.00	0.00	0.00	0.00	0.00	0.00	0.00
0.00	0.00	0.00	0.00	0.00	0.00	0.00	0.00	0.00
0.00	0.00	0.00	0.00	0.00	0.00	0.00	0.00	0.00

0.00	0.00	0.00	0.00	0.00	0.00	0.00	0.00	0.00
0.00	0.00	0.00	0.00	0.00	0.00	0.00	0.00	0.00
0.00	0.00	0.00	0.00	0.00	0.00	0.00	0.00	0.00
0.00	7.00	0.00	0.00	0.00	0.00	0.00	0.00	0.00
0.00	0.00	0.00	0.00	0.00	7.00	0.00	0.00	0.00
0.00	0.00	0.00	0.00	0.00	0.00	7.00	0.00	0.00
0.00	0.00	0.00	0.00	0.00	0.00	7.00	0.00	0.00
0.00	0.00	0.00	0.00	0.00	0.00	0.00	0.00	0.00
0.00	0.00	0.00	0.00	0.00	0.00	0.00	0.00	0.00
0.00	0.00	0.00	0.00	0.00	0.00	0.00	0.00	0.00
0.00	0.00	0.00	0.00	0.00	0.00	0.00	0.00	0.00
0.00	0.00	0.00	0.00	0.00	0.00	0.00	0.00	0.00
0.00	0.00	0.00	0.00	0.00	0.00	0.00	0.00	0.00
0.00	0.00	0.00	0.00	0.00	0.00	0.00	0.00	0.00
0.00	0.00	0.00	0.00	0.00	0.00	0.00	0.00	0.00
0.00	0.00	0.00	0.00	0.00	0.00	0.00	0.00	0.00
0.00	0.00	0.00	7.00	0.00	0.00	0.00	0.00	0.00
0.00	0.00	0.00	0.00	0.00	0.00	0.00	0.00	0.00
0.00	0.00	0.00	0.00	0.00	0.00	0.00	0.00	0.00
0.00	0.00	0.00	0.00	0.00	0.00	0.00	0.00	0.00
0.00	0.00	0.00	0.00	0.00	0.00	0.00	0.00	0.00
0.00	0.00	0.00	0.00	0.00	0.00	0.00	0.00	0.00
0.00	0.00	0.00	0.00	0.00	0.00	0.00	0.00	0.00
0.00	0.00	0.00	0.00	0.00	0.00	0.00	0.00	0.00
0.00	0.00	0.00	0.00	0.00	0.00	0.00	0.00	0.00
0.00	0.00	0.00	0.00	0.00	0.00	0.00	0.00	0.00
0.00	0.00	0.00	0.00	0.00	0.00	0.00	0.00	0.00
0.00	0.00	0.00	0.00	0.00	0.00	0.00	0.00	0.00
0.00	0.00	0.00	0.00	0.00	0.00	0.00	0.00	0.00
0.00	0.00	0.00	0.00	0.00	0.00	0.00	0.00	0.00
0.00	0.00	0.00	0.00	0.00	0.00	0.00	0.00	0.00
0.00	0.00	0.00	0.00	0.00	0.00	0.00	0.00	0.00
0.00	0.00	0.00	0.00	0.00	0.00	0.00	0.00	0.00
0.00	0.00	0.00	0.00	0.00	0.00	0.00	0.00	0.00
0.00	0.00	0.00	0.00	0.00	0.00	0.00	0.00	0.00
0.00	0.00	0.00	0.00	0.00	0.00	0.00	0.00	0.00
0.00	0.00	0.00	0.00	0.00	0.00	0.00	0.00	0.00
0.00	0.00	0.00	0.00	0.00	0.00	0.00	0.00	0.00

0.00	0.00	0.00	0.00	0.00	0.00	0.00	0.00	0.00
0.00	0.00	0.00	0.00	0.00	0.00	0.00	0.00	0.00
0.00	0.00	0.00	0.00	0.00	0.00	0.00	0.00	0.00
0.00	0.00	0.00	0.00	0.00	0.00	0.00	0.00	0.00
0.00	0.00	0.00	0.00	0.00	0.00	0.00	0.00	0.00
0.00	0.00	0.00	0.00	0.00	0.00	0.00	0.00	0.00
0.00	0.00	0.00	0.00	0.00	0.00	0.00	0.00	0.00
0.00	0.00	0.00	0.00	0.00	0.00	0.00	0.00	0.00
0.00	0.00	0.00	0.00	0.00	0.00	0.00	0.00	0.00
0.00	0.00	0.00	0.00	0.00	0.00	0.00	0.00	0.00
0.00	0.00	0.00	0.00	0.00	0.00	0.00	0.00	0.00
0.00	0.00	0.00	0.00	0.00	0.00	0.00	0.00	0.00
0.00	0.00	0.00	0.00	0.00	0.00	0.00	0.00	0.00
0.00	0.00	0.00	0.00	0.00	0.00	0.00	0.00	0.00
0.00	0.00	0.00	0.00	0.00	0.00	0.00	0.00	0.00
0.00	0.00	0.00	0.00	0.00	0.00	0.00	0.00	0.00
0.00	0.00	0.00	0.00	0.00	0.00	0.00	0.00	0.00
0.00	0.00	0.00	0.00	0.00	0.00	0.00	0.00	0.00
0.00	0.00	0.00	0.00	0.00	0.00	0.00	0.00	0.00
0.00	0.00	0.00	0.00	0.00	0.00	0.00	0.00	0.00
0.00	0.00	0.00	0.00	0.00	0.00	0.00	0.00	0.00
0.00	0.00	0.00	0.00	0.00	0.00	0.00	0.00	0.00
0.00	0.00	0.00	0.00	0.00	0.00	0.00	0.00	0.00
0.00	0.00	0.00	0.00	0.00	0.00	0.00	0.00	0.00
0.00	0.00	0.00	0.00	0.00	0.00	0.00	0.00	0.00
0.00	0.00	0.00	0.00	0.00	0.00	6.00	0.00	0.00
0.00	0.00	0.00	0.00	0.00	0.00	6.00	0.00	0.00
0.00	0.00	0.00	0.00	0.00	0.00	0.00	6.00	0.00
0.00	0.00	0.00	0.00	0.00	0.00	0.00	0.00	0.00
0.00	0.00	0.00	0.00	0.00	0.00	0.00	0.00	0.00
0.00	0.00	0.00	0.00	0.00	0.00	0.00	0.00	0.00
0.00	0.00	0.00	0.00	0.00	0.00	0.00	0.00	0.00
0.00	0.00	0.00	0.00	0.00	0.00	0.00	0.00	0.00
0.00	0.00	0.00	0.00	0.00	0.00	0.00	0.00	0.00
0.00	0.00	0.00	0.00	0.00	0.00	0.00	0.00	0.00
0.00	0.00	0.00	0.00	0.00	0.00	0.00	0.00	0.00
0.00	0.00	0.00	0.00	0.00	0.00	0.00	0.00	0.00

0.00	0.00	0.00	0.00	0.00	0.00	0.00	0.00	0.00
0.00	0.00	0.00	0.00	0.00	0.00	0.00	0.00	0.00
0.00	0.00	0.00	0.00	0.00	0.00	0.00	0.00	0.00
0.00	0.00	0.00	0.00	0.00	0.00	0.00	0.00	0.00
0.00	0.00	0.00	0.00	0.00	0.00	0.00	0.00	0.00
0.00	0.00	0.00	0.00	0.00	0.00	0.00	0.00	0.00
0.00	0.00	0.00	0.00	0.00	0.00	0.00	0.00	0.00
0.00	0.00	0.00	0.00	0.00	0.00	0.00	0.00	0.00
0.00	0.00	0.00	0.00	0.00	0.00	0.00	0.00	0.00
0.00	0.00	0.00	0.00	0.00	0.00	0.00	0.00	0.00
0.00	0.00	0.00	0.00	0.00	0.00	0.00	0.00	0.00
0.00	0.00	0.00	0.00	0.00	0.00	0.00	0.00	0.00
0.00	0.00	0.00	0.00	0.00	0.00	0.00	0.00	0.00
0.00	0.00	6.00	0.00	0.00	0.00	0.00	0.00	0.00
0.00	0.00	0.00	0.00	0.00	0.00	0.00	0.00	0.00
0.00	0.00	0.00	0.00	0.00	0.00	0.00	0.00	0.00
0.00	0.00	0.00	0.00	0.00	0.00	0.00	0.00	0.00
0.00	0.00	0.00	0.00	0.00	0.00	0.00	0.00	0.00
0.00	0.00	0.00	0.00	0.00	0.00	0.00	0.00	0.00
0.00	0.00	0.00	0.00	0.00	0.00	0.00	0.00	0.00
0.00	0.00	0.00	0.00	0.00	0.00	0.00	0.00	0.00
0.00	0.00	0.00	0.00	0.00	0.00	0.00	0.00	0.00
0.00	0.00	0.00	0.00	0.00	0.00	0.00	0.00	0.00
0.00	0.00	0.00	0.00	0.00	0.00	0.00	0.00	0.00
0.00	0.00	0.00	0.00	0.00	0.00	0.00	0.00	0.00
0.00	0.00	0.00	0.00	0.00	0.00	0.00	0.00	0.00
0.00	0.00	0.00	0.00	0.00	0.00	0.00	0.00	0.00
0.00	0.00	0.00	0.00	0.00	0.00	0.00	0.00	0.00
0.00	0.00	0.00	0.00	0.00	0.00	0.00	0.00	0.00
0.00	0.00	0.00	0.00	0.00	0.00	0.00	0.00	0.00
0.00	0.00	0.00	0.00	0.00	0.00	0.00	0.00	0.00
0.00	0.00	0.00	0.00	0.00	0.00	0.00	0.00	0.00
0.00	0.00	0.00	0.00	0.00	0.00	0.00	0.00	0.00
0.00	0.00	0.00	0.00	0.00	0.00	0.00	0.00	0.00
0.00	0.00	0.00	0.00	0.00	0.00	0.00	0.00	0.00
0.00	0.00	0.00	0.00	0.00	0.00	0.00	0.00	0.00
0.00	0.00	0.00	0.00	0.00	0.00	0.00	0.00	0.00

0.00	0.00	0.00	0.00	0.00	0.00	0.00	0.00	0.00
0.00	0.00	0.00	0.00	0.00	0.00	0.00	0.00	0.00
0.00	0.00	0.00	0.00	0.00	0.00	0.00	0.00	0.00
0.00	0.00	0.00	0.00	0.00	0.00	0.00	0.00	0.00
0.00	0.00	0.00	0.00	0.00	0.00	0.00	0.00	0.00
0.00	0.00	0.00	0.00	0.00	0.00	0.00	0.00	0.00
0.00	0.00	0.00	0.00	0.00	0.00	0.00	0.00	0.00
0.00	0.00	0.00	0.00	0.00	0.00	0.00	0.00	0.00
0.00	0.00	0.00	0.00	0.00	0.00	0.00	0.00	0.00
0.00	0.00	0.00	0.00	0.00	0.00	0.00	0.00	0.00
0.00	0.00	0.00	0.00	0.00	0.00	0.00	0.00	0.00
0.00	0.00	0.00	0.00	0.00	0.00	0.00	0.00	0.00
0.00	0.00	0.00	0.00	0.00	0.00	0.00	0.00	0.00
0.00	0.00	0.00	0.00	0.00	0.00	0.00	0.00	0.00
0.00	0.00	0.00	0.00	0.00	0.00	0.00	0.00	0.00
0.00	0.00	0.00	0.00	0.00	0.00	0.00	0.00	0.00
0.00	0.00	0.00	0.00	0.00	0.00	0.00	0.00	0.00
0.00	0.00	0.00	0.00	0.00	0.00	0.00	0.00	0.00
0.00	0.00	0.00	0.00	0.00	0.00	0.00	0.00	0.00
0.00	0.00	0.00	0.00	0.00	0.00	0.00	0.00	0.00
0.00	0.00	0.00	0.00	0.00	0.00	0.00	0.00	0.00
0.00	0.00	0.00	0.00	0.00	0.00	0.00	0.00	0.00
0.00	0.00	0.00	0.00	0.00	0.00	0.00	0.00	0.00
0.00	0.00	0.00	0.00	0.00	0.00	0.00	0.00	0.00
0.00	0.00	0.00	0.00	0.00	0.00	0.00	0.00	0.00
0.00	0.00	0.00	0.00	0.00	0.00	0.00	0.00	0.00
0.00	0.00	0.00	0.00	0.00	0.00	0.00	0.00	0.00
0.00	0.00	0.00	0.00	0.00	0.00	0.00	0.00	0.00
0.00	0.00	0.00	0.00	0.00	0.00	0.00	0.00	0.00
0.00	0.00	0.00	0.00	0.00	0.00	0.00	0.00	0.00
0.00	0.00	0.00	0.00	0.00	0.00	0.00	0.00	0.00
0.00	0.00	0.00	0.00	0.00	0.00	0.00	0.00	0.00
0.00	0.00	0.00	0.00	0.00	0.00	0.00	0.00	0.00
0.00	0.00	0.00	0.00	0.00	0.00	0.00	0.00	0.00
0.00	0.00	0.00	0.00	0.00	0.00	0.00	0.00	0.00
0.00	0.00	0.00	0.00	0.00	0.00	0.00	0.00	0.00
0.00	0.00	0.00	0.00	0.00	0.00	0.00	0.00	0.00

0.00	0.00	0.00	0.00	0.00	2.00	0.00	0.00	0.00
0.00	0.00	0.00	0.00	0.00	5.00	0.00	0.00	0.00
0.00	0.00	0.00	2.00	0.00	0.00	3.00	0.00	0.00
0.00	0.00	0.00	0.00	0.00	0.00	0.00	5.00	0.00
0.00	0.00	0.00	0.00	0.00	0.00	0.00	0.00	5.00
0.00	0.00	0.00	0.00	0.00	0.00	0.00	0.00	0.00
0.00	0.00	0.00	0.00	0.00	0.00	0.00	0.00	0.00
0.00	0.00	0.00	0.00	0.00	0.00	0.00	0.00	0.00
0.00	0.00	0.00	0.00	0.00	0.00	0.00	0.00	0.00
0.00	0.00	0.00	0.00	0.00	0.00	0.00	0.00	0.00
0.00	0.00	0.00	0.00	0.00	0.00	0.00	0.00	0.00
0.00	0.00	0.00	0.00	0.00	0.00	0.00	0.00	0.00
0.00	0.00	0.00	0.00	0.00	0.00	0.00	0.00	0.00
0.00	0.00	0.00	0.00	0.00	0.00	0.00	0.00	0.00
0.00	0.00	0.00	0.00	0.00	0.00	0.00	0.00	0.00
0.00	0.00	0.00	0.00	0.00	0.00	0.00	0.00	0.00
0.00	0.00	0.00	0.00	0.00	0.00	0.00	0.00	0.00
0.00	0.00	0.00	0.00	0.00	0.00	0.00	0.00	0.00
0.00	0.00	0.00	0.00	0.00	0.00	0.00	0.00	0.00
0.00	0.00	0.00	5.00	0.00	0.00	0.00	0.00	0.00
0.00	0.00	0.00	0.00	0.00	0.00	0.00	0.00	0.00
0.00	0.00	0.00	0.00	0.00	0.00	0.00	0.00	0.00
0.00	0.00	0.00	0.00	0.00	0.00	0.00	0.00	0.00
0.00	0.00	0.00	0.00	0.00	0.00	0.00	0.00	0.00
0.00	0.00	0.00	0.00	0.00	0.00	0.00	0.00	0.00
0.00	0.00	0.00	0.00	0.00	0.00	0.00	0.00	0.00
0.00	0.00	0.00	0.00	0.00	0.00	0.00	0.00	0.00
0.00	0.00	0.00	0.00	0.00	0.00	0.00	0.00	0.00
0.00	0.00	0.00	0.00	0.00	0.00	0.00	0.00	0.00
0.00	0.00	0.00	0.00	0.00	0.00	0.00	0.00	0.00
0.00	0.00	0.00	0.00	0.00	0.00	0.00	0.00	0.00
0.00	0.00	0.00	0.00	0.00	0.00	0.00	0.00	0.00
0.00	0.00	0.00	0.00	0.00	0.00	0.00	0.00	0.00
0.00	0.00	0.00	0.00	0.00	0.00	0.00	0.00	0.00
0.00	0.00	0.00	0.00	0.00	0.00	0.00	0.00	0.00
0.00	0.00	0.00	0.00	0.00	0.00	0.00	0.00	0.00
0.00	0.00	0.00	0.00	0.00	0.00	0.00	0.00	0.00

0.00	0.00	0.00	0.00	0.00	0.00	0.00	0.00	0.00
0.00	0.00	0.00	0.00	0.00	0.00	0.00	0.00	0.00
0.00	0.00	0.00	0.00	0.00	0.00	0.00	0.00	0.00
0.00	0.00	0.00	0.00	0.00	0.00	0.00	0.00	0.00
0.00	0.00	0.00	0.00	0.00	0.00	0.00	0.00	0.00
0.00	0.00	0.00	0.00	0.00	0.00	0.00	0.00	0.00
0.00	0.00	0.00	0.00	0.00	0.00	0.00	0.00	0.00
0.00	0.00	0.00	0.00	0.00	0.00	0.00	0.00	0.00
0.00	0.00	0.00	0.00	0.00	0.00	0.00	0.00	0.00
0.00	0.00	0.00	0.00	0.00	0.00	0.00	0.00	0.00
0.00	0.00	0.00	0.00	0.00	0.00	0.00	0.00	0.00
0.00	0.00	0.00	0.00	0.00	0.00	0.00	0.00	0.00
0.00	0.00	0.00	0.00	0.00	0.00	0.00	0.00	0.00
0.00	0.00	0.00	0.00	0.00	0.00	0.00	0.00	0.00
0.00	0.00	0.00	0.00	0.00	0.00	0.00	0.00	0.00
0.00	0.00	0.00	0.00	0.00	0.00	0.00	0.00	0.00
0.00	0.00	0.00	0.00	0.00	0.00	0.00	0.00	0.00
0.00	0.00	0.00	0.00	0.00	0.00	0.00	0.00	0.00
0.00	0.00	0.00	0.00	0.00	0.00	0.00	0.00	0.00
0.00	0.00	0.00	0.00	0.00	0.00	0.00	0.00	0.00
0.00	0.00	0.00	0.00	0.00	0.00	0.00	0.00	0.00
0.00	0.00	0.00	0.00	0.00	0.00	0.00	0.00	0.00
0.00	0.00	0.00	0.00	0.00	0.00	0.00	0.00	0.00
0.00	0.00	0.00	0.00	0.00	0.00	0.00	0.00	0.00
0.00	0.00	0.00	0.00	0.00	0.00	0.00	0.00	0.00
0.00	0.00	0.00	0.00	0.00	0.00	0.00	0.00	0.00
0.00	0.00	0.00	0.00	0.00	0.00	0.00	0.00	0.00
0.00	0.00	0.00	0.00	0.00	0.00	0.00	0.00	0.00
0.00	0.00	0.00	0.00	0.00	0.00	0.00	0.00	0.00
0.00	0.00	0.00	0.00	0.00	0.00	0.00	0.00	0.00
0.00	0.00	0.00	0.00	0.00	0.00	0.00	0.00	0.00
0.00	0.00	0.00	0.00	0.00	0.00	0.00	0.00	0.00
0.00	0.00	0.00	0.00	0.00	0.00	0.00	0.00	0.00
0.00	0.00	0.00	0.00	0.00	0.00	0.00	0.00	0.00
0.00	0.00	0.00	0.00	0.00	0.00	0.00	0.00	0.00
0.00	0.00	0.00	0.00	0.00	0.00	0.00	0.00	0.00
0.00	0.00	0.00	0.00	0.00	0.00	0.00	0.00	0.00

0.00	0.00	0.00	0.00	0.00	0.00	0.00	0.00	0.00
0.00	0.00	0.00	0.00	0.00	0.00	0.00	0.00	0.00
0.00	0.00	0.00	0.00	0.00	0.00	0.00	0.00	0.00
0.00	0.00	0.00	0.00	0.00	0.00	0.00	0.00	0.00
0.00	0.00	0.00	0.00	0.00	0.00	0.00	0.00	0.00
0.00	0.00	0.00	0.00	0.00	0.00	0.00	0.00	0.00
0.00	0.00	0.00	0.00	0.00	0.00	0.00	0.00	0.00
0.00	0.00	0.00	0.00	0.00	0.00	0.00	0.00	0.00
0.00	0.00	0.00	0.00	0.00	0.00	0.00	0.00	0.00
0.00	0.00	0.00	0.00	0.00	0.00	0.00	0.00	0.00
0.00	0.00	0.00	0.00	0.00	0.00	0.00	0.00	0.00
0.00	0.00	0.00	0.00	0.00	0.00	0.00	0.00	0.00
0.00	0.00	0.00	0.00	0.00	0.00	0.00	0.00	0.00
4.00	0.00	0.00	0.00	0.00	0.00	0.00	0.00	0.00
2.00	0.00	0.00	0.00	0.00	0.00	0.00	0.00	0.00
4.00	0.00	0.00	0.00	0.00	0.00	0.00	0.00	0.00
0.00	4.00	0.00	0.00	0.00	0.00	0.00	0.00	0.00
0.00	0.00	0.00	0.00	0.00	4.00	0.00	0.00	0.00
0.00	0.00	0.00	0.00	0.00	0.00	0.00	0.00	4.00
0.00	0.00	0.00	0.00	0.00	0.00	0.00	0.00	0.00
0.00	0.00	0.00	0.00	0.00	0.00	0.00	0.00	0.00
0.00	0.00	0.00	0.00	0.00	0.00	0.00	0.00	0.00
0.00	0.00	0.00	0.00	0.00	0.00	0.00	0.00	0.00
0.00	0.00	0.00	0.00	0.00	0.00	0.00	0.00	0.00
0.00	0.00	0.00	0.00	0.00	0.00	0.00	0.00	0.00
0.00	0.00	0.00	0.00	0.00	0.00	0.00	0.00	0.00
0.00	0.00	0.00	0.00	0.00	0.00	0.00	0.00	0.00
0.00	0.00	0.00	0.00	0.00	0.00	0.00	0.00	0.00
0.00	0.00	0.00	0.00	0.00	0.00	0.00	0.00	0.00
0.00	0.00	0.00	0.00	0.00	0.00	0.00	0.00	0.00
0.00	0.00	0.00	0.00	0.00	0.00	0.00	0.00	0.00
0.00	0.00	0.00	0.00	0.00	0.00	0.00	0.00	0.00
0.00	0.00	0.00	0.00	0.00	0.00	0.00	0.00	0.00
0.00	0.00	0.00	0.00	0.00	0.00	0.00	0.00	0.00
0.00	0.00	0.00	0.00	0.00	0.00	0.00	0.00	0.00
0.00	0.00	0.00	0.00	0.00	0.00	0.00	0.00	0.00
0.00	0.00	0.00	0.00	0.00	0.00	0.00	0.00	0.00

0.00	0.00	0.00	2.00	0.00	0.00	0.00	0.00	0.00
0.00	0.00	0.00	0.00	0.00	0.00	0.00	0.00	0.00
0.00	0.00	0.00	0.00	0.00	0.00	0.00	0.00	0.00
0.00	0.00	0.00	0.00	0.00	0.00	0.00	0.00	0.00
0.00	0.00	0.00	0.00	0.00	0.00	0.00	0.00	0.00
0.00	0.00	0.00	0.00	0.00	0.00	0.00	0.00	0.00
0.00	0.00	0.00	0.00	0.00	0.00	0.00	0.00	0.00
0.00	0.00	0.00	0.00	0.00	0.00	0.00	0.00	0.00
0.00	0.00	0.00	0.00	0.00	0.00	0.00	0.00	0.00
0.00	0.00	0.00	0.00	0.00	0.00	0.00	0.00	0.00
0.00	0.00	0.00	0.00	0.00	0.00	0.00	0.00	0.00
0.00	0.00	0.00	0.00	0.00	0.00	0.00	0.00	0.00
0.00	0.00	0.00	0.00	0.00	0.00	0.00	0.00	0.00
0.00	0.00	0.00	0.00	0.00	0.00	0.00	0.00	0.00
0.00	0.00	0.00	0.00	0.00	0.00	0.00	0.00	0.00
0.00	0.00	0.00	0.00	0.00	0.00	0.00	0.00	0.00
0.00	0.00	0.00	0.00	0.00	0.00	0.00	0.00	0.00
0.00	0.00	0.00	0.00	0.00	0.00	0.00	0.00	0.00
0.00	0.00	0.00	0.00	0.00	0.00	0.00	0.00	0.00
0.00	0.00	0.00	0.00	0.00	0.00	0.00	0.00	0.00
0.00	0.00	0.00	0.00	0.00	0.00	0.00	0.00	0.00
0.00	0.00	0.00	0.00	0.00	0.00	0.00	0.00	0.00
0.00	0.00	0.00	0.00	0.00	0.00	0.00	0.00	0.00
0.00	0.00	0.00	0.00	0.00	0.00	0.00	0.00	0.00
0.00	0.00	0.00	0.00	0.00	0.00	0.00	0.00	0.00
0.00	0.00	0.00	0.00	0.00	0.00	0.00	0.00	0.00
0.00	0.00	0.00	0.00	0.00	0.00	0.00	0.00	0.00
0.00	0.00	0.00	0.00	0.00	0.00	0.00	0.00	0.00
0.00	0.00	0.00	0.00	0.00	0.00	0.00	0.00	0.00
0.00	0.00	0.00	0.00	0.00	0.00	0.00	0.00	0.00
0.00	0.00	0.00	0.00	0.00	0.00	0.00	0.00	0.00
0.00	0.00	0.00	0.00	0.00	0.00	0.00	0.00	0.00
0.00	0.00	0.00	0.00	0.00	0.00	0.00	0.00	0.00
0.00	0.00	0.00	0.00	0.00	0.00	0.00	0.00	0.00
0.00	0.00	0.00	0.00	0.00	0.00	0.00	0.00	0.00
0.00	0.00	0.00	0.00	0.00	0.00	0.00	0.00	0.00
0.00	0.00	0.00	0.00	0.00	0.00	0.00	0.00	0.00

0.00	0.00	0.00	0.00	0.00	0.00	0.00	0.00	0.00
0.00	0.00	0.00	0.00	0.00	0.00	0.00	0.00	0.00
0.00	0.00	0.00	0.00	0.00	0.00	0.00	0.00	0.00
0.00	0.00	0.00	0.00	0.00	0.00	0.00	0.00	0.00
0.00	0.00	0.00	0.00	0.00	0.00	0.00	0.00	0.00
0.00	0.00	0.00	0.00	0.00	0.00	0.00	0.00	0.00
0.00	0.00	0.00	0.00	0.00	0.00	0.00	0.00	0.00
0.00	0.00	0.00	0.00	0.00	0.00	0.00	0.00	0.00
0.00	0.00	0.00	0.00	0.00	0.00	0.00	0.00	0.00
0.00	0.00	0.00	0.00	0.00	0.00	0.00	0.00	0.00
0.00	0.00	0.00	0.00	0.00	0.00	0.00	0.00	0.00
0.00	0.00	0.00	0.00	0.00	0.00	0.00	0.00	0.00
0.00	0.00	0.00	0.00	0.00	0.00	0.00	0.00	0.00
0.00	0.00	0.00	0.00	0.00	0.00	0.00	0.00	0.00
0.00	0.00	0.00	0.00	0.00	0.00	0.00	0.00	0.00
0.00	0.00	0.00	0.00	0.00	0.00	0.00	0.00	0.00
0.00	0.00	0.00	0.00	0.00	0.00	0.00	0.00	0.00
0.00	0.00	0.00	0.00	0.00	0.00	0.00	0.00	0.00
0.00	0.00	0.00	0.00	0.00	0.00	0.00	0.00	0.00
0.00	0.00	0.00	0.00	0.00	0.00	0.00	0.00	0.00
0.00	0.00	0.00	0.00	0.00	0.00	0.00	0.00	0.00
0.00	0.00	0.00	0.00	0.00	0.00	0.00	0.00	0.00
0.00	0.00	0.00	0.00	0.00	0.00	0.00	0.00	0.00
0.00	0.00	0.00	0.00	0.00	0.00	0.00	0.00	0.00
0.00	0.00	0.00	0.00	0.00	0.00	0.00	0.00	0.00
0.00	0.00	0.00	0.00	0.00	0.00	0.00	0.00	0.00
0.00	0.00	0.00	0.00	0.00	0.00	0.00	0.00	0.00
0.00	0.00	0.00	0.00	0.00	0.00	0.00	0.00	0.00
0.00	0.00	0.00	0.00	0.00	0.00	0.00	0.00	0.00
0.00	0.00	0.00	0.00	0.00	0.00	0.00	0.00	0.00
0.00	0.00	0.00	0.00	0.00	0.00	0.00	0.00	0.00
0.00	0.00	0.00	0.00	0.00	0.00	0.00	0.00	0.00
0.00	0.00	0.00	0.00	0.00	0.00	0.00	0.00	0.00
0.00	0.00	0.00	0.00	0.00	0.00	0.00	0.00	0.00
0.00	0.00	0.00	0.00	0.00	0.00	0.00	0.00	0.00
0.00	0.00	0.00	0.00	0.00	0.00	0.00	0.00	0.00
0.00	0.00	0.00	0.00	0.00	0.00	0.00	0.00	0.00

0.00	0.00	0.00	0.00	0.00	0.00	0.00	0.00	0.00
0.00	0.00	0.00	0.00	0.00	0.00	0.00	0.00	0.00
0.00	0.00	0.00	0.00	0.00	0.00	0.00	0.00	0.00
0.00	0.00	0.00	0.00	0.00	0.00	0.00	0.00	0.00
0.00	0.00	0.00	0.00	0.00	0.00	0.00	0.00	0.00
0.00	0.00	0.00	0.00	0.00	0.00	0.00	0.00	0.00
0.00	0.00	0.00	0.00	0.00	0.00	0.00	0.00	0.00
0.00	0.00	0.00	0.00	0.00	0.00	0.00	0.00	0.00
0.00	0.00	0.00	0.00	0.00	0.00	0.00	0.00	0.00
0.00	0.00	0.00	0.00	0.00	0.00	0.00	0.00	0.00
0.00	0.00	0.00	0.00	0.00	0.00	0.00	0.00	0.00
0.00	0.00	0.00	0.00	0.00	0.00	0.00	0.00	0.00
0.00	0.00	0.00	0.00	0.00	0.00	0.00	0.00	0.00
0.00	0.00	0.00	0.00	0.00	0.00	0.00	0.00	0.00
0.00	0.00	0.00	0.00	0.00	0.00	0.00	0.00	0.00
0.00	0.00	0.00	0.00	0.00	0.00	0.00	0.00	0.00
0.00	0.00	0.00	0.00	0.00	0.00	0.00	0.00	0.00
0.00	0.00	0.00	0.00	0.00	0.00	0.00	0.00	0.00
0.00	0.00	0.00	0.00	0.00	0.00	0.00	0.00	0.00
0.00	0.00	0.00	0.00	0.00	0.00	0.00	0.00	0.00
0.00	0.00	0.00	0.00	0.00	0.00	0.00	0.00	0.00
0.00	0.00	0.00	0.00	0.00	0.00	0.00	0.00	0.00
0.00	0.00	0.00	0.00	0.00	0.00	0.00	0.00	0.00
0.00	0.00	0.00	0.00	0.00	0.00	0.00	0.00	0.00
3.00	0.00	0.00	0.00	0.00	0.00	0.00	0.00	0.00
3.00	0.00	0.00	0.00	0.00	0.00	0.00	0.00	0.00
3.00	0.00	0.00	0.00	0.00	0.00	0.00	0.00	0.00
0.00	0.00	0.00	0.00	0.00	3.00	0.00	0.00	0.00
0.00	0.00	0.00	0.00	0.00	3.00	0.00	0.00	0.00
0.00	0.00	0.00	0.00	0.00	0.00	3.00	0.00	0.00
0.00	0.00	0.00	0.00	0.00	0.00	3.00	0.00	0.00
0.00	0.00	0.00	0.00	0.00	0.00	0.00	3.00	0.00
0.00	0.00	0.00	0.00	0.00	0.00	0.00	3.00	0.00
0.00	0.00	0.00	0.00	0.00	0.00	0.00	0.00	0.00
0.00	0.00	0.00	0.00	0.00	0.00	0.00	0.00	0.00
0.00	0.00	0.00	0.00	0.00	0.00	0.00	0.00	0.00
0.00	0.00	0.00	0.00	0.00	0.00	0.00	0.00	0.00

0.00	0.00	0.00	0.00	0.00	0.00	0.00	0.00	0.00
0.00	0.00	0.00	0.00	0.00	0.00	0.00	0.00	0.00
0.00	0.00	0.00	0.00	0.00	0.00	0.00	0.00	0.00
0.00	0.00	0.00	0.00	0.00	0.00	0.00	0.00	0.00
0.00	0.00	0.00	0.00	0.00	0.00	0.00	0.00	0.00
0.00	0.00	0.00	0.00	0.00	0.00	0.00	0.00	0.00
0.00	0.00	0.00	0.00	0.00	0.00	0.00	0.00	0.00
0.00	0.00	0.00	0.00	0.00	0.00	0.00	0.00	0.00
0.00	0.00	0.00	0.00	0.00	0.00	0.00	0.00	0.00
0.00	0.00	0.00	0.00	0.00	0.00	0.00	0.00	0.00
0.00	0.00	0.00	0.00	0.00	0.00	0.00	0.00	0.00
0.00	0.00	0.00	0.00	0.00	0.00	0.00	0.00	0.00
0.00	0.00	0.00	0.00	0.00	0.00	0.00	0.00	0.00
0.00	0.00	0.00	0.00	0.00	0.00	0.00	0.00	0.00
0.00	0.00	0.00	0.00	0.00	0.00	0.00	0.00	0.00
0.00	0.00	0.00	0.00	0.00	0.00	0.00	0.00	0.00
0.00	0.00	0.00	0.00	0.00	0.00	0.00	0.00	0.00
0.00	0.00	0.00	0.00	0.00	0.00	0.00	0.00	0.00
0.00	0.00	0.00	0.00	0.00	0.00	0.00	0.00	0.00
0.00	0.00	0.00	0.00	0.00	0.00	0.00	0.00	0.00
0.00	0.00	3.00	0.00	0.00	0.00	0.00	0.00	0.00
0.00	0.00	0.00	3.00	0.00	0.00	0.00	0.00	0.00
0.00	0.00	0.00	0.00	0.00	0.00	0.00	0.00	0.00
0.00	0.00	0.00	0.00	0.00	0.00	0.00	0.00	0.00
0.00	0.00	0.00	0.00	0.00	0.00	0.00	0.00	0.00
0.00	0.00	0.00	0.00	0.00	0.00	0.00	0.00	0.00
0.00	0.00	0.00	0.00	0.00	0.00	0.00	0.00	0.00
0.00	0.00	0.00	0.00	0.00	0.00	0.00	0.00	0.00
0.00	0.00	0.00	0.00	0.00	0.00	0.00	0.00	0.00
0.00	0.00	0.00	0.00	0.00	0.00	0.00	0.00	0.00
0.00	0.00	0.00	0.00	0.00	0.00	0.00	0.00	0.00
0.00	0.00	0.00	0.00	0.00	0.00	0.00	0.00	0.00
0.00	0.00	0.00	0.00	0.00	0.00	0.00	0.00	0.00
0.00	0.00	0.00	0.00	0.00	0.00	0.00	0.00	0.00
0.00	0.00	0.00	0.00	0.00	0.00	0.00	0.00	0.00
0.00	0.00	0.00	0.00	0.00	0.00	0.00	0.00	0.00
0.00	0.00	0.00	0.00	0.00	0.00	0.00	0.00	0.00

0.00	0.00	0.00	0.00	0.00	0.00	0.00	0.00	0.00
0.00	0.00	0.00	0.00	0.00	0.00	0.00	0.00	0.00
0.00	0.00	0.00	0.00	0.00	0.00	0.00	0.00	0.00
0.00	0.00	0.00	0.00	0.00	0.00	0.00	0.00	0.00
0.00	0.00	0.00	0.00	0.00	0.00	0.00	0.00	0.00
0.00	0.00	0.00	0.00	0.00	0.00	0.00	0.00	0.00
0.00	0.00	0.00	0.00	0.00	0.00	0.00	0.00	0.00
0.00	0.00	0.00	0.00	0.00	0.00	0.00	0.00	0.00
0.00	0.00	0.00	0.00	0.00	0.00	0.00	0.00	0.00
0.00	0.00	0.00	0.00	0.00	0.00	0.00	0.00	0.00
0.00	0.00	0.00	0.00	0.00	0.00	0.00	0.00	0.00
0.00	0.00	0.00	0.00	0.00	0.00	0.00	0.00	0.00
0.00	0.00	0.00	0.00	0.00	0.00	0.00	0.00	0.00
0.00	0.00	0.00	0.00	0.00	0.00	0.00	0.00	0.00
0.00	0.00	0.00	0.00	0.00	0.00	0.00	0.00	0.00
0.00	0.00	0.00	0.00	0.00	0.00	0.00	0.00	0.00
0.00	0.00	0.00	0.00	0.00	0.00	0.00	0.00	0.00
0.00	0.00	0.00	0.00	0.00	0.00	0.00	0.00	0.00
0.00	0.00	0.00	0.00	0.00	0.00	0.00	0.00	0.00
0.00	0.00	0.00	0.00	0.00	0.00	0.00	0.00	0.00
0.00	0.00	0.00	0.00	0.00	0.00	0.00	0.00	0.00
0.00	0.00	0.00	0.00	0.00	0.00	0.00	0.00	0.00
0.00	0.00	0.00	0.00	0.00	0.00	0.00	0.00	0.00
0.00	0.00	0.00	0.00	0.00	0.00	0.00	0.00	0.00
0.00	0.00	0.00	0.00	0.00	0.00	0.00	0.00	0.00
0.00	0.00	0.00	0.00	0.00	0.00	0.00	0.00	0.00
0.00	0.00	0.00	0.00	0.00	0.00	0.00	0.00	0.00
0.00	0.00	0.00	0.00	0.00	0.00	0.00	0.00	0.00
0.00	0.00	0.00	0.00	0.00	0.00	0.00	0.00	0.00
0.00	0.00	0.00	0.00	0.00	0.00	0.00	0.00	0.00
0.00	0.00	0.00	0.00	0.00	0.00	0.00	0.00	0.00
0.00	0.00	0.00	0.00	0.00	0.00	0.00	0.00	0.00
0.00	0.00	0.00	0.00	0.00	0.00	0.00	0.00	0.00
0.00	0.00	0.00	0.00	0.00	0.00	0.00	0.00	0.00
0.00	0.00	0.00	0.00	0.00	0.00	0.00	0.00	0.00
0.00	0.00	0.00	0.00	0.00	0.00	0.00	0.00	0.00
0.00	0.00	0.00	0.00	0.00	0.00	0.00	0.00	0.00

0.00	0.00	0.00	0.00	0.00	0.00	0.00	0.00	0.00
0.00	0.00	0.00	0.00	0.00	0.00	0.00	0.00	0.00
0.00	0.00	0.00	0.00	0.00	0.00	0.00	0.00	0.00
0.00	0.00	0.00	0.00	0.00	0.00	0.00	0.00	0.00
0.00	0.00	0.00	0.00	0.00	0.00	0.00	0.00	0.00
0.00	0.00	0.00	0.00	0.00	0.00	0.00	0.00	0.00
0.00	0.00	0.00	0.00	0.00	0.00	0.00	0.00	0.00
0.00	0.00	0.00	0.00	0.00	0.00	0.00	0.00	0.00
0.00	0.00	0.00	0.00	0.00	0.00	0.00	0.00	0.00
0.00	0.00	0.00	0.00	0.00	0.00	0.00	0.00	0.00
0.00	0.00	0.00	0.00	0.00	0.00	0.00	0.00	0.00
0.00	0.00	0.00	0.00	0.00	0.00	0.00	0.00	0.00
0.00	0.00	0.00	0.00	0.00	0.00	0.00	0.00	0.00
0.00	0.00	0.00	0.00	0.00	0.00	0.00	0.00	0.00
0.00	0.00	0.00	0.00	0.00	0.00	0.00	0.00	0.00
0.00	0.00	0.00	0.00	0.00	0.00	0.00	0.00	0.00
0.00	0.00	0.00	0.00	0.00	0.00	0.00	0.00	0.00
0.00	0.00	0.00	0.00	0.00	0.00	0.00	0.00	0.00
0.00	0.00	0.00	0.00	0.00	0.00	0.00	0.00	0.00
0.00	0.00	0.00	0.00	0.00	0.00	0.00	0.00	0.00
0.00	0.00	0.00	0.00	0.00	0.00	0.00	0.00	0.00
0.00	0.00	0.00	0.00	0.00	0.00	0.00	0.00	0.00
0.00	0.00	0.00	0.00	0.00	0.00	0.00	0.00	0.00
0.00	0.00	0.00	0.00	0.00	0.00	0.00	0.00	0.00
0.00	0.00	0.00	0.00	0.00	0.00	0.00	0.00	0.00
0.00	0.00	0.00	0.00	0.00	0.00	0.00	0.00	0.00
0.00	0.00	0.00	0.00	0.00	0.00	0.00	0.00	0.00
0.00	0.00	0.00	0.00	0.00	0.00	0.00	0.00	0.00
0.00	0.00	0.00	0.00	0.00	0.00	0.00	0.00	0.00
0.00	0.00	0.00	0.00	0.00	0.00	0.00	0.00	0.00
0.00	0.00	0.00	0.00	0.00	0.00	0.00	0.00	0.00
0.00	0.00	0.00	0.00	0.00	0.00	0.00	0.00	0.00
0.00	0.00	0.00	0.00	0.00	0.00	0.00	0.00	0.00
0.00	0.00	0.00	0.00	0.00	0.00	0.00	0.00	0.00
0.00	0.00	0.00	0.00	0.00	0.00	0.00	0.00	0.00
0.00	0.00	0.00	0.00	0.00	0.00	0.00	0.00	0.00
0.00	0.00	0.00	0.00	0.00	0.00	0.00	0.00	0.00

0.00	0.00	0.00	0.00	0.00	0.00	0.00	0.00	0.00
0.00	0.00	0.00	0.00	0.00	0.00	0.00	0.00	0.00
0.00	0.00	0.00	0.00	0.00	0.00	0.00	0.00	0.00
0.00	0.00	0.00	0.00	0.00	0.00	0.00	0.00	0.00
0.00	0.00	0.00	0.00	0.00	0.00	0.00	0.00	0.00
0.00	0.00	0.00	0.00	0.00	0.00	0.00	0.00	0.00
0.00	0.00	0.00	0.00	0.00	0.00	0.00	0.00	0.00
0.00	0.00	0.00	0.00	0.00	0.00	0.00	0.00	0.00
0.00	0.00	0.00	0.00	0.00	0.00	0.00	0.00	0.00
0.00	0.00	0.00	0.00	0.00	0.00	0.00	0.00	0.00
0.00	0.00	0.00	0.00	0.00	0.00	0.00	0.00	0.00
0.00	0.00	0.00	0.00	0.00	0.00	0.00	0.00	0.00
0.00	0.00	0.00	0.00	0.00	0.00	0.00	0.00	0.00
0.00	0.00	0.00	0.00	0.00	0.00	0.00	0.00	0.00
0.00	0.00	0.00	0.00	0.00	0.00	0.00	0.00	0.00
0.00	0.00	0.00	0.00	0.00	0.00	0.00	0.00	0.00
0.00	0.00	0.00	0.00	0.00	0.00	0.00	0.00	0.00
0.00	0.00	0.00	0.00	0.00	0.00	0.00	0.00	0.00
0.00	0.00	0.00	0.00	0.00	0.00	0.00	0.00	0.00
0.00	2.00	0.00	0.00	0.00	0.00	0.00	0.00	0.00
0.00	0.00	0.00	0.00	0.00	0.00	2.00	0.00	0.00
0.00	0.00	0.00	0.00	0.00	0.00	0.00	2.00	0.00
0.00	0.00	0.00	0.00	0.00	0.00	0.00	0.00	2.00
0.00	0.00	0.00	0.00	0.00	0.00	0.00	0.00	2.00
0.00	0.00	0.00	0.00	0.00	0.00	0.00	0.00	0.00
0.00	0.00	0.00	0.00	0.00	0.00	0.00	0.00	0.00
0.00	0.00	0.00	0.00	0.00	0.00	0.00	0.00	0.00
0.00	0.00	0.00	0.00	0.00	0.00	0.00	0.00	0.00
0.00	0.00	0.00	0.00	0.00	0.00	0.00	0.00	0.00
0.00	0.00	0.00	0.00	0.00	0.00	0.00	0.00	0.00
0.00	0.00	0.00	0.00	0.00	0.00	0.00	0.00	0.00
0.00	0.00	0.00	0.00	0.00	0.00	0.00	0.00	0.00
0.00	0.00	0.00	0.00	0.00	0.00	0.00	0.00	0.00
0.00	0.00	0.00	0.00	0.00	0.00	0.00	0.00	0.00
0.00	0.00	0.00	0.00	0.00	0.00	0.00	0.00	0.00
0.00	0.00	0.00	0.00	0.00	0.00	0.00	0.00	0.00
0.00	0.00	0.00	0.00	0.00	0.00	0.00	0.00	0.00

0.00	0.00	0.00	0.00	0.00	0.00	0.00	0.00	0.00
0.00	0.00	0.00	0.00	0.00	0.00	0.00	0.00	0.00
0.00	0.00	0.00	0.00	0.00	0.00	0.00	0.00	0.00
0.00	0.00	0.00	0.00	0.00	0.00	0.00	0.00	0.00
0.00	0.00	0.00	0.00	0.00	0.00	0.00	0.00	0.00
0.00	0.00	0.00	0.00	0.00	0.00	0.00	0.00	0.00
0.00	0.00	0.00	0.00	0.00	0.00	0.00	0.00	0.00
0.00	0.00	0.00	0.00	0.00	0.00	0.00	0.00	0.00
0.00	0.00	0.00	0.00	0.00	0.00	0.00	0.00	0.00
0.00	0.00	0.00	0.00	0.00	0.00	0.00	0.00	0.00
0.00	0.00	0.00	0.00	0.00	0.00	0.00	0.00	0.00
0.00	0.00	0.00	0.00	0.00	0.00	0.00	0.00	0.00
0.00	0.00	0.00	0.00	0.00	0.00	0.00	0.00	0.00
0.00	0.00	0.00	0.00	0.00	0.00	0.00	0.00	0.00
0.00	0.00	0.00	0.00	0.00	0.00	0.00	0.00	0.00
0.00	0.00	0.00	0.00	0.00	0.00	0.00	0.00	0.00
0.00	0.00	0.00	0.00	0.00	0.00	0.00	0.00	0.00
0.00	0.00	2.00	0.00	0.00	0.00	0.00	0.00	0.00
0.00	0.00	0.00	0.00	2.00	0.00	0.00	0.00	0.00
0.00	0.00	0.00	0.00	0.00	0.00	0.00	0.00	0.00
0.00	0.00	0.00	0.00	0.00	0.00	0.00	0.00	0.00
0.00	0.00	0.00	0.00	0.00	0.00	0.00	0.00	0.00
0.00	0.00	0.00	0.00	0.00	0.00	0.00	0.00	0.00
0.00	0.00	0.00	0.00	0.00	0.00	0.00	0.00	0.00
0.00	0.00	0.00	0.00	0.00	0.00	0.00	0.00	0.00
0.00	0.00	0.00	0.00	0.00	0.00	0.00	0.00	0.00
0.00	0.00	0.00	0.00	0.00	0.00	0.00	0.00	0.00
0.00	0.00	0.00	0.00	0.00	0.00	0.00	0.00	0.00
0.00	0.00	0.00	0.00	0.00	0.00	0.00	0.00	0.00
0.00	0.00	0.00	0.00	0.00	0.00	0.00	0.00	0.00
0.00	0.00	0.00	0.00	0.00	0.00	0.00	0.00	0.00
0.00	0.00	0.00	0.00	0.00	0.00	0.00	0.00	0.00
0.00	0.00	0.00	0.00	0.00	0.00	0.00	0.00	0.00
0.00	0.00	0.00	0.00	0.00	0.00	0.00	0.00	0.00
0.00	0.00	0.00	0.00	0.00	0.00	0.00	0.00	0.00
0.00	0.00	0.00	0.00	0.00	0.00	0.00	0.00	0.00
0.00	0.00	0.00	0.00	0.00	0.00	0.00	0.00	0.00

0.00	0.00	0.00	0.00	0.00	0.00	0.00	0.00	0.00
0.00	0.00	0.00	0.00	0.00	0.00	0.00	0.00	0.00
0.00	0.00	0.00	0.00	0.00	0.00	0.00	0.00	0.00
0.00	0.00	0.00	0.00	0.00	0.00	0.00	0.00	0.00
0.00	0.00	0.00	0.00	0.00	0.00	0.00	0.00	0.00
0.00	0.00	0.00	0.00	0.00	0.00	0.00	0.00	0.00
0.00	0.00	0.00	0.00	0.00	0.00	0.00	0.00	0.00
0.00	0.00	0.00	0.00	0.00	0.00	0.00	0.00	0.00
0.00	0.00	0.00	0.00	0.00	0.00	0.00	0.00	0.00
0.00	0.00	0.00	0.00	0.00	0.00	0.00	0.00	0.00
0.00	0.00	0.00	0.00	0.00	0.00	0.00	0.00	0.00
0.00	0.00	0.00	0.00	0.00	0.00	0.00	0.00	0.00
0.00	0.00	0.00	0.00	0.00	0.00	0.00	0.00	0.00
0.00	0.00	0.00	0.00	0.00	0.00	0.00	0.00	0.00
0.00	0.00	0.00	0.00	0.00	0.00	0.00	0.00	0.00
0.00	0.00	0.00	0.00	0.00	0.00	0.00	0.00	0.00
0.00	0.00	0.00	0.00	0.00	0.00	0.00	0.00	0.00
0.00	0.00	0.00	0.00	0.00	0.00	0.00	0.00	0.00
0.00	0.00	0.00	0.00	0.00	0.00	0.00	0.00	0.00
0.00	0.00	0.00	0.00	0.00	0.00	0.00	0.00	0.00
0.00	0.00	0.00	0.00	0.00	0.00	0.00	0.00	0.00
0.00	0.00	0.00	0.00	0.00	0.00	0.00	0.00	0.00
0.00	0.00	0.00	0.00	0.00	0.00	0.00	0.00	0.00
0.00	0.00	0.00	0.00	0.00	0.00	0.00	0.00	0.00
0.00	0.00	0.00	0.00	0.00	0.00	0.00	0.00	0.00
0.00	0.00	0.00	0.00	0.00	0.00	0.00	0.00	0.00
0.00	0.00	0.00	0.00	0.00	0.00	0.00	0.00	0.00
0.00	0.00	0.00	0.00	0.00	0.00	0.00	0.00	0.00
0.00	0.00	0.00	0.00	0.00	0.00	0.00	0.00	0.00
0.00	0.00	0.00	0.00	0.00	0.00	0.00	0.00	0.00
0.00	0.00	0.00	0.00	0.00	0.00	0.00	0.00	0.00
0.00	0.00	0.00	0.00	0.00	0.00	0.00	0.00	0.00
0.00	0.00	0.00	0.00	0.00	0.00	0.00	0.00	0.00
0.00	0.00	0.00	0.00	0.00	0.00	0.00	0.00	0.00
0.00	0.00	0.00	0.00	0.00	0.00	0.00	0.00	0.00
0.00	0.00	0.00	0.00	0.00	0.00	0.00	0.00	0.00
0.00	0.00	0.00	0.00	0.00	0.00	0.00	0.00	0.00

0.00	0.00	0.00	0.00	0.00	0.00	0.00	0.00	0.00
0.00	0.00	0.00	0.00	0.00	0.00	0.00	0.00	0.00
0.00	0.00	0.00	0.00	0.00	0.00	0.00	0.00	0.00
0.00	0.00	0.00	0.00	0.00	0.00	0.00	0.00	0.00
0.00	0.00	0.00	0.00	0.00	0.00	0.00	0.00	0.00
0.00	0.00	0.00	0.00	0.00	0.00	0.00	0.00	0.00
0.00	0.00	0.00	0.00	0.00	0.00	0.00	0.00	0.00
0.00	0.00	0.00	0.00	0.00	0.00	0.00	0.00	0.00
0.00	0.00	0.00	0.00	0.00	0.00	0.00	0.00	0.00
0.00	0.00	0.00	0.00	0.00	0.00	0.00	0.00	0.00
0.00	0.00	0.00	0.00	0.00	0.00	0.00	0.00	0.00
0.00	0.00	0.00	0.00	0.00	0.00	0.00	0.00	0.00
0.00	0.00	0.00	0.00	0.00	0.00	0.00	0.00	0.00
0.00	0.00	0.00	0.00	0.00	0.00	0.00	0.00	0.00
0.00	0.00	0.00	0.00	0.00	0.00	0.00	0.00	0.00
0.00	0.00	0.00	0.00	0.00	0.00	0.00	0.00	0.00
0.00	0.00	0.00	0.00	0.00	0.00	0.00	0.00	0.00
0.00	0.00	0.00	0.00	0.00	0.00	0.00	0.00	0.00
0.00	0.00	0.00	0.00	0.00	0.00	0.00	0.00	0.00
0.00	0.00	0.00	0.00	0.00	0.00	0.00	0.00	0.00
0.00	0.00	0.00	0.00	0.00	0.00	0.00	0.00	0.00
0.00	0.00	0.00	0.00	0.00	0.00	0.00	0.00	0.00
0.00	0.00	0.00	0.00	0.00	0.00	0.00	0.00	0.00
0.00	0.00	0.00	0.00	0.00	0.00	0.00	0.00	0.00
0.00	0.00	0.00	0.00	0.00	0.00	0.00	0.00	0.00
0.00	0.00	0.00	0.00	0.00	0.00	0.00	0.00	0.00
0.00	0.00	0.00	0.00	0.00	0.00	0.00	0.00	0.00
0.00	0.00	0.00	0.00	0.00	0.00	0.00	0.00	0.00
0.00	0.00	0.00	0.00	0.00	0.00	0.00	0.00	0.00
0.00	0.00	0.00	0.00	0.00	0.00	0.00	0.00	0.00
0.00	0.00	0.00	0.00	0.00	0.00	0.00	0.00	0.00
0.00	0.00	0.00	0.00	0.00	0.00	0.00	0.00	0.00
0.00	0.00	0.00	0.00	0.00	0.00	0.00	0.00	0.00
0.00	0.00	0.00	0.00	0.00	0.00	0.00	0.00	0.00
0.00	0.00	0.00	0.00	0.00	0.00	0.00	0.00	0.00
0.00	0.00	0.00	0.00	0.00	0.00	0.00	0.00	0.00
0.00	0.00	0.00	0.00	0.00	0.00	0.00	0.00	0.00

0.00	0.00	0.00	0.00	0.00	0.00	0.00	0.00	0.00
0.00	0.00	0.00	0.00	0.00	0.00	0.00	0.00	0.00
0.00	0.00	0.00	0.00	0.00	0.00	0.00	0.00	0.00
0.00	0.00	0.00	0.00	0.00	0.00	0.00	0.00	0.00
0.00	0.00	0.00	0.00	0.00	0.00	0.00	0.00	0.00
0.00	0.00	0.00	0.00	0.00	0.00	0.00	0.00	0.00
0.00	0.00	0.00	0.00	0.00	0.00	0.00	0.00	0.00
0.00	0.00	0.00	0.00	0.00	0.00	0.00	0.00	0.00
0.00	0.00	0.00	0.00	0.00	0.00	0.00	0.00	0.00
0.00	0.00	0.00	0.00	0.00	0.00	0.00	0.00	0.00
0.00	0.00	0.00	0.00	0.00	0.00	0.00	0.00	0.00
0.00	0.00	0.00	0.00	0.00	0.00	0.00	0.00	0.00
0.00	0.00	0.00	0.00	0.00	0.00	0.00	0.00	0.00
0.00	0.00	0.00	0.00	0.00	0.00	0.00	0.00	0.00
0.00	0.00	0.00	0.00	0.00	0.00	0.00	0.00	0.00
0.00	0.00	0.00	0.00	0.00	0.00	0.00	0.00	0.00
0.00	0.00	0.00	0.00	0.00	0.00	0.00	0.00	0.00
0.00	0.00	0.00	0.00	0.00	0.00	0.00	0.00	0.00
0.00	0.00	0.00	0.00	0.00	0.00	0.00	0.00	0.00
0.00	0.00	0.00	0.00	0.00	0.00	0.00	0.00	0.00
0.00	0.00	0.00	0.00	0.00	0.00	0.00	0.00	0.00
0.00	0.00	0.00	0.00	0.00	0.00	0.00	0.00	0.00
0.00	0.00	0.00	0.00	0.00	0.00	0.00	0.00	0.00
0.00	0.00	0.00	0.00	0.00	0.00	0.00	0.00	0.00
0.00	0.00	0.00	0.00	0.00	0.00	0.00	0.00	0.00
0.00	0.00	0.00	0.00	0.00	0.00	0.00	0.00	0.00
0.00	0.00	0.00	0.00	0.00	0.00	0.00	0.00	0.00
0.00	0.00	0.00	0.00	0.00	0.00	0.00	0.00	0.00
0.00	0.00	0.00	0.00	0.00	0.00	0.00	0.00	0.00
0.00	0.00	0.00	0.00	0.00	0.00	0.00	0.00	0.00
0.00	0.00	0.00	0.00	0.00	0.00	0.00	0.00	0.00
0.00	0.00	0.00	0.00	0.00	0.00	0.00	0.00	0.00
0.00	0.00	0.00	0.00	0.00	0.00	0.00	0.00	0.00

B16	B17	B18	B19	A19	A20	B20	D20	C20
0.00	0.00	0.00	0.00	11.00	282.00	0.00	55314.00	26065.00
14627.00	3159.00	14937.00	14550.00	17264.00	7574.00	14091.00	21.00	37.00
0.00	0.00	0.00	14.00	15.00	2.00	26.00	3469.00	2362.00
5915.00	1167.00	5662.00	4457.00	4927.00	2379.00	5631.00	0.00	0.00
3928.00	740.00	3883.00	4000.00	4081.00	1697.00	3939.00	0.00	0.00
0.00	549.00	0.00	0.00	0.00	12.00	0.00	136.00	0.00
0.00	0.00	0.00	0.00	0.00	6504.00	0.00	116.00	40.00
2694.00	756.00	2607.00	2906.00	2759.00	1470.00	3630.00	0.00	0.00
0.00	0.00	0.00	0.00	0.00	0.00	0.00	87.00	16990.00
2266.00	411.00	1973.00	1447.00	1792.00	701.00	1888.00	0.00	0.00
0.00	0.00	0.00	0.00	0.00	0.00	0.00	0.00	0.00
0.00	2863.00	0.00	0.00	0.00	968.00	31.00	75.00	66.00
0.00	0.00	0.00	0.00	0.00	0.00	0.00	48.00	9759.00
1023.00	187.00	992.00	1030.00	1146.00	645.00	1110.00	0.00	0.00
1008.00	234.00	824.00	1136.00	1056.00	532.00	1208.00	0.00	0.00
982.00	226.00	1077.00	1010.00	1267.00	470.00	1207.00	0.00	0.00
0.00	0.00	0.00	0.00	30.00	2566.00	0.00	0.00	0.00
892.00	268.00	990.00	1252.00	1237.00	452.00	570.00	0.00	0.00
0.00	0.00	7.00	0.00	0.00	0.00	0.00	112.00	5.00
0.00	0.00	0.00	0.00	0.00	0.00	0.00	66.00	0.00
0.00	0.00	0.00	0.00	0.00	0.00	0.00	0.00	0.00
0.00	0.00	0.00	0.00	0.00	0.00	0.00	0.00	0.00
0.00	0.00	0.00	0.00	0.00	0.00	0.00	17.00	9.00
487.00	87.00	407.00	444.00	574.00	227.00	562.00	0.00	0.00
0.00	0.00	0.00	0.00	0.00	0.00	0.00	0.00	0.00
0.00	0.00	0.00	0.00	0.00	0.00	0.00	0.00	132.00
0.00	0.00	0.00	0.00	0.00	0.00	0.00	0.00	0.00
115.00	151.00	498.00	962.00	183.00	99.00	343.00	25.00	0.00
0.00	0.00	0.00	0.00	0.00	0.00	0.00	0.00	0.00
0.00	43.00	0.00	0.00	0.00	34.00	0.00	0.00	0.00
0.00	0.00	0.00	0.00	0.00	0.00	0.00	31.00	131.00
0.00	0.00	0.00	0.00	0.00	0.00	0.00	0.00	1182.00
0.00	688.00	0.00	0.00	0.00	0.00	0.00	0.00	0.00
0.00	0.00	0.00	0.00	0.00	0.00	0.00	28.00	0.00
0.00	0.00	0.00	0.00	0.00	0.00	0.00	0.00	0.00

0.00	0.00	0.00	0.00	0.00	0.00	0.00	0.00	0.00
0.00	0.00	0.00	0.00	0.00	0.00	0.00	5.00	35.00
0.00	0.00	0.00	0.00	21.00	0.00	0.00	29.00	0.00
0.00	0.00	0.00	0.00	0.00	0.00	0.00	0.00	0.00
4.00	9321.00	0.00	0.00	3.00	2.00	0.00	0.00	0.00
0.00	0.00	0.00	0.00	0.00	0.00	0.00	0.00	0.00
0.00	0.00	0.00	0.00	0.00	0.00	0.00	0.00	0.00
0.00	0.00	0.00	0.00	0.00	0.00	0.00	67.00	0.00
0.00	0.00	0.00	0.00	0.00	0.00	0.00	0.00	0.00
0.00	0.00	0.00	0.00	0.00	0.00	0.00	0.00	0.00
0.00	0.00	0.00	0.00	0.00	0.00	0.00	0.00	0.00
70.00	21.00	143.00	88.00	52.00	35.00	76.00	0.00	0.00
0.00	0.00	0.00	0.00	0.00	0.00	0.00	0.00	0.00
0.00	0.00	0.00	0.00	0.00	0.00	0.00	28.00	15.00
0.00	0.00	0.00	0.00	0.00	0.00	0.00	0.00	0.00
0.00	0.00	0.00	0.00	0.00	0.00	0.00	0.00	0.00
107.00	30.00	130.00	93.00	112.00	50.00	70.00	0.00	0.00
0.00	0.00	0.00	0.00	0.00	5305.00	0.00	0.00	0.00
185.00	24.00	196.00	116.00	168.00	51.00	146.00	0.00	0.00
101.00	7.00	121.00	64.00	180.00	54.00	76.00	0.00	0.00
0.00	0.00	21.00	0.00	50.00	0.00	0.00	0.00	0.00
0.00	0.00	10.00	0.00	3.00	8.00	7.00	0.00	0.00
0.00	716.00	0.00	0.00	0.00	0.00	0.00	0.00	0.00
0.00	0.00	0.00	0.00	0.00	0.00	0.00	0.00	0.00
0.00	0.00	0.00	0.00	0.00	0.00	18.00	85.00	0.00
0.00	0.00	0.00	0.00	0.00	0.00	0.00	0.00	0.00
0.00	0.00	0.00	0.00	0.00	0.00	0.00	0.00	0.00
0.00	0.00	0.00	0.00	0.00	0.00	0.00	0.00	0.00
25.00	21.00	92.00	176.00	29.00	38.00	148.00	0.00	0.00
102.00	18.00	85.00	89.00	149.00	48.00	54.00	0.00	0.00
0.00	0.00	0.00	0.00	0.00	0.00	0.00	0.00	50.00
0.00	0.00	0.00	0.00	0.00	0.00	0.00	0.00	0.00
0.00	0.00	0.00	0.00	0.00	0.00	0.00	25.00	33.00
0.00	0.00	82.00	164.00	40.00	0.00	127.00	0.00	0.00
0.00	0.00	0.00	0.00	0.00	0.00	0.00	0.00	2342.00
0.00	0.00	0.00	0.00	0.00	0.00	0.00	0.00	0.00
0.00	0.00	0.00	0.00	0.00	0.00	0.00	54.00	16.00

0.00	0.00	0.00	0.00	0.00	0.00	0.00	0.00	0.00
0.00	0.00	0.00	0.00	0.00	0.00	0.00	0.00	0.00
0.00	0.00	0.00	0.00	0.00	0.00	0.00	0.00	0.00
0.00	0.00	0.00	0.00	0.00	0.00	0.00	0.00	0.00
0.00	0.00	0.00	0.00	0.00	0.00	0.00	172.00	0.00
0.00	0.00	0.00	0.00	0.00	0.00	0.00	0.00	0.00
0.00	0.00	0.00	0.00	0.00	0.00	0.00	0.00	0.00
0.00	0.00	0.00	0.00	0.00	0.00	0.00	0.00	0.00
0.00	0.00	0.00	0.00	0.00	0.00	0.00	0.00	0.00
0.00	0.00	0.00	0.00	0.00	0.00	0.00	0.00	0.00
0.00	0.00	0.00	0.00	0.00	0.00	0.00	0.00	0.00
0.00	0.00	79.00	72.00	85.00	0.00	0.00	0.00	0.00
0.00	0.00	0.00	0.00	0.00	0.00	0.00	0.00	0.00
0.00	0.00	0.00	0.00	0.00	5.00	0.00	9.00	8.00
0.00	0.00	0.00	0.00	0.00	0.00	0.00	0.00	0.00
0.00	0.00	0.00	0.00	0.00	0.00	0.00	0.00	0.00
41.00	11.00	51.00	35.00	53.00	17.00	75.00	0.00	0.00
62.00	10.00	59.00	33.00	56.00	23.00	23.00	0.00	0.00
0.00	0.00	0.00	0.00	0.00	0.00	0.00	0.00	0.00
0.00	0.00	0.00	0.00	0.00	0.00	0.00	0.00	0.00
0.00	0.00	0.00	0.00	0.00	0.00	0.00	16.00	0.00
0.00	0.00	0.00	0.00	0.00	0.00	0.00	214.00	122.00
0.00	0.00	0.00	0.00	0.00	0.00	0.00	0.00	0.00
0.00	0.00	0.00	0.00	0.00	0.00	0.00	0.00	0.00
0.00	0.00	0.00	0.00	0.00	0.00	0.00	0.00	0.00
0.00	0.00	0.00	0.00	0.00	0.00	0.00	0.00	0.00
0.00	0.00	0.00	0.00	0.00	0.00	0.00	23.00	15.00
0.00	0.00	0.00	30.00	7.00	5.00	7.00	0.00	0.00
0.00	0.00	0.00	11.00	0.00	0.00	0.00	73.00	0.00
0.00	0.00	0.00	0.00	0.00	0.00	0.00	0.00	0.00
35.00	0.00	40.00	19.00	67.00	13.00	53.00	0.00	0.00
0.00	0.00	0.00	0.00	0.00	0.00	0.00	3.00	0.00
0.00	0.00	0.00	0.00	5.00	0.00	0.00	0.00	151.00
0.00	0.00	0.00	0.00	0.00	0.00	0.00	62.00	48.00
92.00	0.00	0.00	0.00	0.00	41.00	0.00	0.00	0.00
0.00	0.00	0.00	0.00	0.00	0.00	0.00	0.00	0.00
0.00	0.00	0.00	0.00	0.00	0.00	0.00	0.00	0.00

0.00	0.00	0.00	0.00	0.00	0.00	24.00	167.00	118.00
0.00	0.00	0.00	0.00	0.00	0.00	0.00	0.00	0.00
0.00	0.00	0.00	0.00	0.00	0.00	0.00	0.00	0.00
0.00	0.00	0.00	0.00	33.00	0.00	0.00	0.00	0.00
0.00	0.00	0.00	0.00	0.00	0.00	0.00	0.00	0.00
0.00	0.00	0.00	0.00	0.00	0.00	0.00	0.00	0.00
0.00	0.00	0.00	0.00	0.00	0.00	0.00	0.00	0.00
0.00	0.00	0.00	0.00	0.00	0.00	0.00	0.00	0.00
0.00	0.00	9.00	0.00	0.00	0.00	0.00	95.00	28.00
0.00	0.00	0.00	0.00	0.00	0.00	0.00	0.00	0.00
0.00	0.00	0.00	0.00	0.00	0.00	0.00	0.00	0.00
0.00	0.00	0.00	0.00	0.00	0.00	0.00	65.00	0.00
0.00	0.00	0.00	0.00	0.00	0.00	0.00	0.00	0.00
0.00	0.00	0.00	0.00	0.00	0.00	0.00	0.00	71.00
5.00	11.00	27.00	55.00	3.00	8.00	60.00	0.00	0.00
0.00	0.00	0.00	0.00	0.00	0.00	0.00	0.00	0.00
0.00	0.00	0.00	0.00	0.00	0.00	0.00	0.00	0.00
0.00	0.00	0.00	0.00	0.00	0.00	0.00	0.00	0.00
0.00	0.00	0.00	0.00	0.00	0.00	0.00	0.00	0.00
0.00	0.00	0.00	0.00	0.00	0.00	0.00	0.00	0.00
0.00	0.00	0.00	0.00	0.00	0.00	0.00	0.00	0.00
0.00	1257.00	0.00	0.00	0.00	0.00	0.00	0.00	0.00
0.00	0.00	0.00	0.00	0.00	0.00	0.00	0.00	0.00
0.00	0.00	0.00	0.00	0.00	0.00	0.00	0.00	42.00
0.00	0.00	0.00	0.00	0.00	288.00	0.00	0.00	0.00
0.00	0.00	0.00	0.00	0.00	0.00	0.00	0.00	0.00
0.00	0.00	0.00	0.00	0.00	0.00	0.00	0.00	0.00
0.00	0.00	0.00	0.00	0.00	0.00	0.00	0.00	0.00
0.00	0.00	0.00	0.00	0.00	0.00	0.00	0.00	0.00
0.00	0.00	0.00	0.00	0.00	0.00	0.00	0.00	0.00
0.00	0.00	0.00	22.00	0.00	0.00	0.00	0.00	0.00
0.00	0.00	0.00	0.00	0.00	0.00	0.00	0.00	0.00
0.00	0.00	0.00	0.00	0.00	0.00	0.00	0.00	0.00
0.00	0.00	0.00	92.00	0.00	0.00	74.00	0.00	0.00
0.00	0.00	0.00	0.00	0.00	2.00	0.00	0.00	0.00
0.00	0.00	0.00	0.00	0.00	0.00	0.00	0.00	0.00
0.00	0.00	0.00	0.00	0.00	0.00	0.00	0.00	0.00

0.00	0.00	0.00	0.00	0.00	0.00	0.00	0.00	0.00
0.00	0.00	0.00	0.00	0.00	0.00	0.00	0.00	0.00
0.00	0.00	0.00	0.00	0.00	0.00	0.00	58.00	35.00
0.00	0.00	0.00	0.00	0.00	0.00	0.00	0.00	0.00
0.00	0.00	0.00	0.00	0.00	0.00	0.00	0.00	5.00
0.00	0.00	0.00	0.00	0.00	0.00	0.00	0.00	0.00
0.00	0.00	0.00	0.00	0.00	0.00	0.00	0.00	0.00
5.00	0.00	17.00	21.00	21.00	10.00	11.00	0.00	0.00
0.00	0.00	0.00	0.00	0.00	0.00	0.00	0.00	0.00
0.00	0.00	0.00	0.00	0.00	0.00	0.00	0.00	0.00
0.00	0.00	0.00	0.00	0.00	0.00	0.00	0.00	0.00
0.00	0.00	0.00	0.00	0.00	0.00	0.00	0.00	0.00
0.00	0.00	0.00	0.00	0.00	0.00	0.00	0.00	0.00
0.00	0.00	0.00	0.00	0.00	0.00	0.00	39.00	0.00
0.00	7.00	29.00	40.00	0.00	10.00	0.00	0.00	0.00
0.00	0.00	0.00	0.00	0.00	0.00	0.00	4.00	0.00
0.00	0.00	0.00	0.00	0.00	0.00	0.00	0.00	0.00
0.00	0.00	0.00	0.00	0.00	0.00	0.00	0.00	0.00
0.00	0.00	0.00	0.00	0.00	0.00	0.00	0.00	0.00
8.00	0.00	0.00	0.00	0.00	0.00	0.00	0.00	0.00
0.00	0.00	0.00	0.00	0.00	0.00	0.00	21.00	13.00
0.00	0.00	0.00	0.00	0.00	0.00	0.00	0.00	0.00
0.00	0.00	0.00	0.00	0.00	0.00	0.00	0.00	0.00
0.00	0.00	0.00	0.00	0.00	0.00	109.00	0.00	0.00
0.00	0.00	0.00	0.00	0.00	0.00	0.00	0.00	0.00
0.00	0.00	0.00	0.00	0.00	0.00	0.00	0.00	0.00
0.00	0.00	0.00	0.00	0.00	0.00	0.00	0.00	0.00
0.00	0.00	0.00	21.00	0.00	0.00	0.00	0.00	0.00
0.00	0.00	0.00	0.00	0.00	0.00	0.00	0.00	0.00
0.00	0.00	0.00	0.00	0.00	0.00	0.00	0.00	0.00
0.00	0.00	0.00	0.00	0.00	0.00	0.00	0.00	0.00
0.00	0.00	0.00	0.00	0.00	0.00	0.00	40.00	17.00
0.00	0.00	0.00	0.00	0.00	0.00	0.00	0.00	0.00
0.00	0.00	0.00	0.00	0.00	0.00	0.00	0.00	0.00
0.00	0.00	0.00	0.00	0.00	0.00	0.00	0.00	0.00
0.00	0.00	0.00	0.00	0.00	0.00	0.00	0.00	0.00
0.00	0.00	6.00	0.00	0.00	0.00	0.00	0.00	0.00

0.00	0.00	0.00	0.00	0.00	0.00	0.00	0.00	0.00
12.00	0.00	21.00	0.00	0.00	0.00	0.00	0.00	0.00
0.00	0.00	0.00	0.00	0.00	0.00	0.00	0.00	0.00
0.00	0.00	0.00	0.00	0.00	0.00	0.00	0.00	0.00
0.00	0.00	0.00	0.00	0.00	0.00	0.00	0.00	0.00
0.00	0.00	0.00	0.00	0.00	0.00	0.00	0.00	0.00
0.00	0.00	0.00	0.00	0.00	0.00	0.00	0.00	0.00
0.00	0.00	0.00	0.00	0.00	0.00	0.00	0.00	0.00
0.00	0.00	0.00	0.00	0.00	0.00	0.00	0.00	0.00
0.00	0.00	0.00	0.00	0.00	0.00	0.00	0.00	0.00
0.00	0.00	0.00	0.00	0.00	0.00	0.00	0.00	0.00
0.00	0.00	0.00	0.00	0.00	0.00	0.00	0.00	0.00
0.00	0.00	0.00	0.00	0.00	0.00	0.00	49.00	0.00
0.00	0.00	0.00	0.00	0.00	0.00	0.00	0.00	0.00
0.00	0.00	0.00	0.00	0.00	0.00	0.00	0.00	0.00
0.00	0.00	0.00	0.00	0.00	0.00	0.00	0.00	0.00
0.00	11.00	0.00	0.00	0.00	17.00	0.00	0.00	0.00
0.00	0.00	0.00	0.00	0.00	0.00	0.00	6.00	0.00
0.00	0.00	0.00	0.00	0.00	0.00	0.00	0.00	0.00
0.00	0.00	0.00	0.00	0.00	0.00	0.00	0.00	0.00
0.00	0.00	0.00	0.00	0.00	0.00	0.00	0.00	0.00
0.00	0.00	0.00	0.00	0.00	0.00	0.00	4.00	0.00
4.00	0.00	12.00	0.00	20.00	0.00	11.00	0.00	0.00
0.00	0.00	0.00	0.00	0.00	0.00	0.00	0.00	0.00
0.00	0.00	0.00	0.00	0.00	0.00	0.00	0.00	0.00
10.00	0.00	7.00	15.00	7.00	18.00	12.00	0.00	0.00
0.00	0.00	0.00	0.00	0.00	0.00	0.00	0.00	0.00
0.00	0.00	0.00	0.00	0.00	0.00	0.00	0.00	0.00
0.00	0.00	0.00	0.00	0.00	0.00	0.00	0.00	0.00
0.00	0.00	0.00	0.00	0.00	0.00	0.00	0.00	0.00
0.00	0.00	0.00	0.00	0.00	0.00	0.00	0.00	0.00
0.00	0.00	0.00	0.00	0.00	0.00	0.00	0.00	0.00
0.00	0.00	0.00	0.00	0.00	0.00	0.00	0.00	0.00
0.00	0.00	0.00	0.00	0.00	0.00	55.00	0.00	0.00
0.00	0.00	0.00	0.00	0.00	0.00	0.00	0.00	0.00
0.00	0.00	0.00	0.00	0.00	0.00	0.00	273.00	0.00
0.00	0.00	0.00	0.00	0.00	0.00	0.00	0.00	0.00

0.00	0.00	0.00	0.00	0.00	0.00	0.00	0.00	0.00
0.00	0.00	0.00	0.00	0.00	0.00	0.00	0.00	0.00
0.00	0.00	0.00	0.00	0.00	0.00	0.00	0.00	2.00
0.00	0.00	0.00	0.00	0.00	0.00	0.00	10.00	7.00
0.00	0.00	0.00	0.00	0.00	0.00	0.00	0.00	0.00
0.00	0.00	0.00	0.00	0.00	0.00	0.00	0.00	0.00
0.00	0.00	0.00	0.00	0.00	0.00	0.00	0.00	0.00
0.00	0.00	0.00	0.00	0.00	0.00	0.00	0.00	0.00
0.00	0.00	0.00	0.00	0.00	0.00	0.00	0.00	0.00
0.00	0.00	0.00	0.00	0.00	0.00	0.00	0.00	0.00
0.00	0.00	0.00	0.00	0.00	0.00	0.00	13.00	0.00
0.00	0.00	0.00	0.00	0.00	0.00	0.00	0.00	0.00
0.00	0.00	0.00	0.00	0.00	0.00	0.00	0.00	0.00
0.00	0.00	0.00	0.00	0.00	0.00	0.00	0.00	0.00
0.00	0.00	0.00	0.00	0.00	0.00	0.00	2.00	0.00
0.00	0.00	0.00	0.00	0.00	0.00	0.00	0.00	0.00
0.00	0.00	0.00	0.00	0.00	0.00	0.00	0.00	0.00
0.00	0.00	0.00	0.00	0.00	0.00	0.00	5.00	0.00
0.00	0.00	0.00	0.00	0.00	0.00	0.00	0.00	0.00
0.00	0.00	0.00	0.00	0.00	0.00	0.00	0.00	0.00
0.00	0.00	0.00	0.00	0.00	0.00	0.00	0.00	0.00
0.00	0.00	0.00	0.00	0.00	0.00	34.00	0.00	0.00
0.00	0.00	0.00	0.00	0.00	0.00	0.00	0.00	0.00
0.00	0.00	0.00	0.00	0.00	0.00	0.00	0.00	0.00
0.00	0.00	0.00	0.00	0.00	0.00	0.00	0.00	0.00
0.00	0.00	0.00	0.00	0.00	0.00	0.00	0.00	0.00
0.00	0.00	0.00	0.00	0.00	0.00	0.00	0.00	0.00
0.00	0.00	0.00	0.00	0.00	0.00	0.00	0.00	0.00
0.00	0.00	0.00	0.00	0.00	0.00	0.00	0.00	0.00
0.00	0.00	0.00	0.00	2.00	0.00	0.00	0.00	0.00
0.00	0.00	0.00	0.00	0.00	0.00	0.00	0.00	0.00
0.00	0.00	0.00	0.00	0.00	0.00	0.00	0.00	0.00
0.00	0.00	0.00	0.00	0.00	0.00	0.00	0.00	0.00
0.00	0.00	0.00	0.00	0.00	0.00	0.00	0.00	0.00
0.00	0.00	0.00	0.00	25.00	0.00	0.00	0.00	0.00
0.00	0.00	0.00	0.00	0.00	0.00	0.00	0.00	0.00
0.00	0.00	0.00	0.00	0.00	0.00	0.00	0.00	0.00

27.00	0.00	30.00	0.00	0.00	15.00	0.00	0.00	0.00
0.00	0.00	0.00	0.00	0.00	0.00	0.00	0.00	0.00
0.00	0.00	0.00	0.00	0.00	0.00	0.00	0.00	0.00
0.00	0.00	0.00	0.00	0.00	0.00	0.00	0.00	0.00
0.00	0.00	0.00	0.00	0.00	0.00	0.00	0.00	0.00
0.00	0.00	0.00	0.00	0.00	0.00	0.00	0.00	0.00
0.00	0.00	0.00	0.00	0.00	0.00	0.00	0.00	0.00
0.00	0.00	0.00	0.00	0.00	0.00	0.00	0.00	0.00
0.00	0.00	0.00	0.00	0.00	0.00	0.00	0.00	0.00
0.00	0.00	0.00	0.00	0.00	0.00	0.00	0.00	0.00
0.00	0.00	0.00	0.00	0.00	0.00	0.00	0.00	0.00
0.00	0.00	0.00	0.00	0.00	0.00	0.00	0.00	0.00
0.00	0.00	0.00	0.00	0.00	0.00	0.00	0.00	0.00
0.00	0.00	0.00	0.00	0.00	0.00	9.00	0.00	0.00
0.00	0.00	0.00	0.00	0.00	0.00	0.00	39.00	0.00
23.00	0.00	42.00	0.00	18.00	10.00	0.00	0.00	0.00
0.00	0.00	0.00	0.00	0.00	0.00	0.00	0.00	3.00
0.00	0.00	0.00	0.00	0.00	0.00	0.00	22.00	7.00
0.00	0.00	0.00	0.00	0.00	0.00	0.00	0.00	0.00
0.00	0.00	0.00	0.00	0.00	0.00	0.00	0.00	0.00
0.00	0.00	0.00	0.00	0.00	0.00	0.00	0.00	0.00
0.00	0.00	0.00	0.00	0.00	0.00	0.00	0.00	0.00
0.00	0.00	0.00	0.00	0.00	0.00	0.00	0.00	0.00
0.00	0.00	0.00	0.00	0.00	0.00	0.00	0.00	0.00
0.00	0.00	0.00	0.00	0.00	0.00	0.00	0.00	0.00
82.00	0.00	0.00	0.00	0.00	0.00	0.00	0.00	0.00
0.00	0.00	0.00	0.00	0.00	4.00	0.00	0.00	0.00
0.00	0.00	0.00	0.00	0.00	0.00	0.00	0.00	0.00
0.00	0.00	0.00	0.00	0.00	0.00	0.00	0.00	0.00
0.00	0.00	0.00	0.00	0.00	0.00	0.00	45.00	0.00
0.00	16.00	0.00	0.00	0.00	0.00	63.00	0.00	0.00
0.00	0.00	0.00	0.00	0.00	0.00	0.00	0.00	0.00
0.00	0.00	0.00	0.00	0.00	0.00	0.00	0.00	0.00
0.00	0.00	0.00	0.00	0.00	0.00	0.00	0.00	0.00
0.00	0.00	0.00	0.00	0.00	0.00	0.00	0.00	0.00
0.00	0.00	0.00	0.00	0.00	0.00	0.00	0.00	0.00
0.00	0.00	0.00	0.00	0.00	8.00	0.00	0.00	0.00

0.00	0.00	0.00	0.00	0.00	0.00	0.00	41.00	36.00
0.00	0.00	0.00	0.00	0.00	0.00	0.00	0.00	0.00
0.00	0.00	0.00	0.00	0.00	0.00	0.00	0.00	0.00
0.00	0.00	0.00	0.00	0.00	0.00	0.00	17.00	12.00
0.00	0.00	0.00	0.00	0.00	0.00	0.00	0.00	0.00
0.00	0.00	0.00	0.00	0.00	0.00	0.00	0.00	0.00
0.00	0.00	0.00	0.00	0.00	0.00	0.00	0.00	0.00
0.00	0.00	0.00	0.00	0.00	9.00	0.00	0.00	0.00
0.00	0.00	0.00	0.00	0.00	326.00	0.00	0.00	0.00
0.00	0.00	0.00	0.00	0.00	0.00	0.00	9.00	9.00
0.00	0.00	0.00	0.00	0.00	0.00	0.00	0.00	0.00
0.00	0.00	0.00	0.00	0.00	0.00	0.00	0.00	0.00
0.00	0.00	0.00	0.00	0.00	0.00	0.00	0.00	0.00
0.00	0.00	0.00	0.00	0.00	0.00	0.00	0.00	0.00
0.00	0.00	0.00	0.00	0.00	0.00	0.00	0.00	0.00
0.00	0.00	0.00	0.00	0.00	0.00	0.00	0.00	0.00
0.00	0.00	0.00	0.00	0.00	0.00	0.00	0.00	0.00
0.00	0.00	0.00	0.00	0.00	0.00	0.00	3.00	0.00
0.00	0.00	0.00	0.00	0.00	0.00	0.00	0.00	0.00
0.00	0.00	0.00	0.00	0.00	0.00	0.00	0.00	0.00
0.00	0.00	0.00	0.00	0.00	0.00	0.00	0.00	0.00
0.00	0.00	0.00	0.00	0.00	0.00	0.00	0.00	0.00
0.00	0.00	0.00	0.00	0.00	0.00	0.00	0.00	0.00
0.00	0.00	0.00	0.00	0.00	0.00	0.00	0.00	0.00
0.00	0.00	0.00	0.00	0.00	0.00	0.00	0.00	0.00
0.00	0.00	0.00	0.00	0.00	0.00	0.00	0.00	0.00
0.00	0.00	0.00	0.00	0.00	0.00	0.00	0.00	0.00
0.00	0.00	0.00	0.00	0.00	0.00	0.00	45.00	0.00
0.00	0.00	0.00	0.00	0.00	0.00	0.00	0.00	0.00
0.00	0.00	0.00	0.00	0.00	0.00	0.00	0.00	0.00
0.00	0.00	0.00	0.00	0.00	0.00	0.00	0.00	0.00
0.00	0.00	0.00	0.00	0.00	0.00	0.00	0.00	0.00
0.00	0.00	0.00	0.00	0.00	0.00	0.00	0.00	0.00
0.00	0.00	0.00	0.00	0.00	0.00	0.00	0.00	0.00
0.00	0.00	0.00	0.00	29.00	0.00	0.00	0.00	0.00
0.00	0.00	0.00	0.00	0.00	0.00	0.00	0.00	0.00
0.00	0.00	0.00	0.00	0.00	0.00	0.00	0.00	0.00

0.00	0.00	0.00	0.00	0.00	0.00	0.00	0.00	0.00
0.00	0.00	0.00	0.00	0.00	0.00	0.00	0.00	0.00
0.00	0.00	0.00	0.00	0.00	0.00	0.00	0.00	0.00
0.00	0.00	0.00	0.00	0.00	0.00	0.00	0.00	0.00
0.00	0.00	0.00	0.00	0.00	0.00	0.00	0.00	0.00
0.00	0.00	0.00	0.00	0.00	0.00	0.00	9.00	11.00
0.00	0.00	0.00	0.00	0.00	0.00	0.00	0.00	0.00
0.00	0.00	0.00	0.00	0.00	0.00	0.00	0.00	0.00
0.00	0.00	0.00	0.00	0.00	0.00	0.00	0.00	0.00
0.00	0.00	0.00	0.00	0.00	0.00	0.00	34.00	0.00
0.00	0.00	0.00	0.00	0.00	0.00	0.00	0.00	0.00
0.00	0.00	0.00	0.00	0.00	0.00	0.00	0.00	0.00
0.00	0.00	0.00	0.00	0.00	0.00	0.00	0.00	0.00
0.00	0.00	0.00	0.00	0.00	0.00	0.00	0.00	0.00
0.00	0.00	0.00	0.00	0.00	0.00	0.00	0.00	0.00
0.00	0.00	0.00	0.00	0.00	0.00	0.00	0.00	0.00
0.00	0.00	0.00	0.00	0.00	0.00	0.00	0.00	0.00
0.00	0.00	0.00	0.00	0.00	0.00	0.00	0.00	0.00
0.00	0.00	0.00	0.00	0.00	0.00	0.00	0.00	0.00
0.00	0.00	0.00	0.00	0.00	0.00	0.00	0.00	0.00
0.00	0.00	0.00	0.00	0.00	0.00	0.00	0.00	0.00
0.00	0.00	0.00	69.00	0.00	0.00	0.00	0.00	0.00
0.00	0.00	0.00	37.00	0.00	0.00	0.00	0.00	0.00
0.00	0.00	0.00	19.00	16.00	0.00	0.00	0.00	0.00
0.00	0.00	0.00	0.00	0.00	0.00	0.00	0.00	51.00
0.00	0.00	0.00	0.00	0.00	0.00	0.00	0.00	0.00
0.00	0.00	0.00	0.00	0.00	0.00	0.00	0.00	0.00
0.00	0.00	0.00	0.00	0.00	0.00	0.00	0.00	0.00
0.00	0.00	0.00	0.00	0.00	0.00	0.00	0.00	0.00
0.00	0.00	0.00	0.00	0.00	0.00	0.00	0.00	0.00
0.00	0.00	0.00	0.00	0.00	242.00	0.00	0.00	0.00
0.00	0.00	0.00	0.00	0.00	0.00	0.00	0.00	0.00
0.00	0.00	0.00	0.00	0.00	0.00	0.00	0.00	0.00
0.00	0.00	0.00	0.00	0.00	0.00	0.00	0.00	0.00
0.00	0.00	0.00	0.00	0.00	0.00	0.00	0.00	0.00
0.00	0.00	17.00	0.00	0.00	0.00	0.00	0.00	0.00
0.00	0.00	0.00	0.00	0.00	0.00	0.00	0.00	0.00

20.00	0.00	0.00	0.00	13.00	0.00	0.00	0.00	0.00
0.00	0.00	0.00	0.00	0.00	0.00	0.00	0.00	0.00
18.00	0.00	0.00	24.00	17.00	0.00	0.00	0.00	0.00
0.00	0.00	0.00	0.00	0.00	0.00	0.00	0.00	0.00
0.00	0.00	0.00	0.00	0.00	0.00	0.00	0.00	0.00
0.00	0.00	0.00	0.00	0.00	0.00	0.00	0.00	0.00
0.00	0.00	0.00	0.00	0.00	0.00	0.00	0.00	0.00
0.00	0.00	0.00	0.00	0.00	0.00	0.00	0.00	0.00
0.00	0.00	0.00	0.00	0.00	0.00	0.00	0.00	0.00
0.00	0.00	0.00	0.00	0.00	0.00	0.00	0.00	0.00
0.00	0.00	0.00	0.00	0.00	0.00	0.00	0.00	0.00
0.00	0.00	0.00	0.00	0.00	0.00	0.00	0.00	0.00
0.00	0.00	0.00	0.00	0.00	0.00	0.00	0.00	0.00
0.00	0.00	0.00	0.00	0.00	0.00	0.00	0.00	6.00
0.00	0.00	0.00	0.00	0.00	0.00	0.00	0.00	0.00
0.00	0.00	0.00	0.00	0.00	0.00	0.00	0.00	0.00
0.00	0.00	0.00	0.00	0.00	0.00	0.00	0.00	0.00
14.00	0.00	2.00	0.00	0.00	0.00	0.00	0.00	0.00
0.00	0.00	0.00	0.00	0.00	0.00	0.00	0.00	0.00
0.00	0.00	0.00	0.00	0.00	0.00	0.00	0.00	0.00
0.00	0.00	0.00	0.00	0.00	0.00	0.00	34.00	0.00
0.00	0.00	0.00	0.00	0.00	0.00	0.00	0.00	30.00
0.00	0.00	0.00	0.00	0.00	0.00	0.00	0.00	0.00
0.00	0.00	27.00	0.00	0.00	0.00	0.00	0.00	0.00
0.00	0.00	0.00	0.00	0.00	0.00	0.00	0.00	0.00
0.00	0.00	0.00	0.00	0.00	0.00	0.00	0.00	0.00
0.00	0.00	0.00	0.00	0.00	213.00	0.00	0.00	0.00
0.00	0.00	0.00	0.00	0.00	0.00	0.00	0.00	0.00
0.00	0.00	0.00	0.00	0.00	0.00	0.00	0.00	0.00
0.00	0.00	0.00	0.00	0.00	0.00	0.00	0.00	29.00
0.00	2.00	0.00	0.00	0.00	0.00	0.00	0.00	0.00
0.00	0.00	0.00	0.00	0.00	0.00	0.00	0.00	0.00
0.00	0.00	0.00	0.00	0.00	0.00	0.00	0.00	0.00
0.00	0.00	0.00	0.00	0.00	0.00	0.00	0.00	0.00
0.00	0.00	0.00	0.00	0.00	0.00	0.00	0.00	0.00
0.00	0.00	0.00	0.00	0.00	0.00	0.00	0.00	0.00
0.00	0.00	0.00	0.00	0.00	0.00	0.00	0.00	0.00

0.00	0.00	0.00	0.00	0.00	0.00	0.00	0.00	0.00
0.00	0.00	0.00	0.00	0.00	0.00	0.00	0.00	0.00
0.00	0.00	0.00	0.00	0.00	0.00	0.00	0.00	0.00
8.00	0.00	6.00	0.00	0.00	0.00	0.00	0.00	0.00
0.00	0.00	0.00	0.00	0.00	0.00	0.00	30.00	0.00
0.00	0.00	0.00	0.00	0.00	0.00	0.00	0.00	0.00
0.00	0.00	0.00	0.00	0.00	0.00	0.00	0.00	0.00
0.00	0.00	0.00	0.00	0.00	0.00	0.00	0.00	0.00
0.00	0.00	0.00	0.00	0.00	0.00	0.00	0.00	0.00
0.00	0.00	0.00	0.00	0.00	0.00	0.00	0.00	0.00
0.00	0.00	0.00	0.00	0.00	0.00	0.00	0.00	0.00
0.00	0.00	0.00	0.00	0.00	0.00	0.00	0.00	0.00
0.00	0.00	0.00	0.00	0.00	0.00	0.00	0.00	0.00
0.00	0.00	0.00	0.00	0.00	0.00	0.00	0.00	0.00
0.00	0.00	0.00	0.00	0.00	0.00	0.00	0.00	0.00
0.00	0.00	0.00	0.00	0.00	0.00	0.00	0.00	0.00
0.00	0.00	0.00	0.00	0.00	0.00	0.00	35.00	0.00
0.00	0.00	0.00	0.00	0.00	0.00	0.00	19.00	8.00
0.00	0.00	0.00	0.00	0.00	0.00	0.00	0.00	0.00
0.00	0.00	0.00	0.00	0.00	0.00	0.00	20.00	0.00
0.00	0.00	0.00	0.00	0.00	0.00	0.00	5.00	0.00
0.00	0.00	0.00	0.00	0.00	0.00	0.00	0.00	0.00
0.00	0.00	0.00	0.00	0.00	0.00	0.00	0.00	0.00
0.00	0.00	0.00	0.00	0.00	0.00	0.00	0.00	0.00
0.00	0.00	0.00	0.00	0.00	0.00	0.00	0.00	0.00
0.00	0.00	0.00	0.00	0.00	0.00	0.00	0.00	0.00
0.00	0.00	0.00	0.00	0.00	0.00	0.00	0.00	0.00
0.00	0.00	0.00	0.00	0.00	0.00	0.00	0.00	0.00
0.00	0.00	0.00	0.00	0.00	0.00	0.00	0.00	13.00
0.00	0.00	0.00	0.00	0.00	0.00	0.00	0.00	0.00
0.00	0.00	0.00	0.00	0.00	6.00	0.00	0.00	0.00
0.00	0.00	0.00	0.00	0.00	0.00	0.00	15.00	19.00
0.00	0.00	0.00	0.00	0.00	0.00	0.00	0.00	0.00
0.00	0.00	0.00	0.00	0.00	0.00	0.00	0.00	0.00
0.00	0.00	0.00	0.00	0.00	0.00	0.00	0.00	0.00
0.00	0.00	0.00	0.00	0.00	0.00	0.00	0.00	0.00
0.00	0.00	0.00	0.00	0.00	0.00	0.00	0.00	0.00

0.00	0.00	0.00	0.00	0.00	0.00	0.00	0.00	0.00
0.00	0.00	0.00	0.00	0.00	0.00	0.00	0.00	0.00
0.00	0.00	0.00	0.00	0.00	0.00	0.00	132.00	0.00
0.00	0.00	0.00	0.00	0.00	0.00	0.00	3.00	0.00
0.00	0.00	0.00	0.00	0.00	0.00	0.00	0.00	0.00
0.00	0.00	0.00	0.00	0.00	0.00	0.00	0.00	0.00
0.00	0.00	0.00	0.00	0.00	0.00	0.00	0.00	0.00
0.00	0.00	0.00	0.00	0.00	0.00	0.00	0.00	0.00
0.00	0.00	0.00	0.00	0.00	0.00	0.00	0.00	0.00
0.00	0.00	0.00	0.00	0.00	0.00	0.00	0.00	0.00
0.00	0.00	0.00	0.00	0.00	0.00	0.00	0.00	0.00
0.00	0.00	0.00	0.00	0.00	0.00	0.00	0.00	0.00
0.00	170.00	0.00	0.00	0.00	0.00	0.00	0.00	0.00
0.00	0.00	0.00	0.00	0.00	0.00	0.00	0.00	0.00
0.00	0.00	0.00	0.00	0.00	0.00	0.00	0.00	0.00
0.00	0.00	0.00	0.00	0.00	0.00	0.00	0.00	0.00
0.00	0.00	0.00	0.00	0.00	0.00	0.00	0.00	0.00
0.00	0.00	0.00	0.00	0.00	0.00	0.00	0.00	0.00
0.00	0.00	0.00	0.00	0.00	0.00	0.00	0.00	15.00
0.00	0.00	0.00	0.00	0.00	0.00	0.00	13.00	0.00
0.00	0.00	0.00	0.00	0.00	0.00	0.00	14.00	0.00
0.00	0.00	0.00	0.00	0.00	0.00	0.00	0.00	0.00
0.00	0.00	0.00	0.00	0.00	0.00	0.00	0.00	0.00
0.00	0.00	0.00	0.00	0.00	0.00	0.00	6.00	0.00
0.00	0.00	0.00	0.00	0.00	0.00	0.00	0.00	0.00
0.00	0.00	0.00	0.00	0.00	0.00	0.00	0.00	0.00
0.00	0.00	0.00	0.00	0.00	0.00	0.00	0.00	0.00
0.00	0.00	0.00	0.00	0.00	0.00	0.00	0.00	0.00
0.00	0.00	0.00	0.00	0.00	0.00	0.00	0.00	0.00
0.00	0.00	0.00	0.00	0.00	0.00	0.00	0.00	0.00
0.00	0.00	0.00	0.00	0.00	0.00	0.00	0.00	0.00
0.00	0.00	0.00	0.00	0.00	0.00	0.00	0.00	0.00
13.00	0.00	6.00	0.00	9.00	5.00	0.00	0.00	0.00
0.00	0.00	0.00	0.00	0.00	0.00	0.00	0.00	0.00
0.00	0.00	0.00	0.00	0.00	0.00	0.00	0.00	0.00
0.00	0.00	0.00	0.00	0.00	0.00	0.00	0.00	0.00
0.00	0.00	0.00	0.00	0.00	0.00	0.00	0.00	0.00

0.00	0.00	0.00	0.00	0.00	0.00	0.00	0.00	0.00
0.00	0.00	0.00	0.00	0.00	0.00	0.00	0.00	0.00
0.00	0.00	0.00	0.00	0.00	0.00	0.00	0.00	0.00
0.00	0.00	0.00	0.00	0.00	0.00	0.00	0.00	0.00
11.00	0.00	0.00	0.00	0.00	5.00	0.00	0.00	0.00
0.00	0.00	25.00	0.00	0.00	0.00	0.00	0.00	0.00
0.00	0.00	0.00	0.00	0.00	0.00	0.00	0.00	0.00
0.00	0.00	0.00	0.00	0.00	0.00	0.00	0.00	9.00
0.00	0.00	0.00	0.00	0.00	0.00	0.00	0.00	0.00
0.00	0.00	0.00	0.00	0.00	0.00	0.00	0.00	0.00
0.00	0.00	0.00	0.00	0.00	0.00	0.00	0.00	0.00
0.00	0.00	0.00	0.00	0.00	0.00	0.00	0.00	0.00
0.00	0.00	0.00	0.00	0.00	0.00	0.00	0.00	0.00
0.00	0.00	0.00	0.00	0.00	0.00	0.00	0.00	0.00
0.00	0.00	0.00	0.00	0.00	0.00	0.00	0.00	0.00
0.00	0.00	0.00	0.00	0.00	0.00	0.00	9.00	8.00
0.00	0.00	0.00	0.00	0.00	0.00	0.00	0.00	0.00
0.00	0.00	0.00	0.00	0.00	0.00	0.00	0.00	0.00
0.00	0.00	0.00	0.00	0.00	0.00	0.00	0.00	0.00
0.00	0.00	0.00	0.00	0.00	0.00	0.00	0.00	0.00
0.00	0.00	0.00	0.00	0.00	0.00	0.00	0.00	0.00
0.00	0.00	0.00	0.00	0.00	0.00	0.00	6.00	0.00
0.00	0.00	0.00	0.00	0.00	0.00	0.00	0.00	0.00
0.00	0.00	0.00	0.00	0.00	0.00	0.00	28.00	0.00
0.00	0.00	0.00	0.00	0.00	0.00	0.00	0.00	0.00
0.00	0.00	0.00	0.00	0.00	0.00	0.00	0.00	0.00
0.00	0.00	0.00	0.00	0.00	0.00	0.00	13.00	8.00
0.00	0.00	0.00	0.00	0.00	0.00	0.00	0.00	0.00
0.00	0.00	0.00	0.00	0.00	0.00	0.00	0.00	0.00
0.00	0.00	0.00	12.00	0.00	0.00	0.00	0.00	0.00
0.00	0.00	0.00	0.00	0.00	0.00	0.00	0.00	0.00
0.00	0.00	0.00	0.00	0.00	0.00	0.00	16.00	3.00
0.00	0.00	0.00	0.00	0.00	0.00	0.00	0.00	0.00
0.00	0.00	0.00	0.00	0.00	0.00	0.00	0.00	0.00
0.00	0.00	0.00	0.00	0.00	0.00	0.00	0.00	0.00
0.00	0.00	0.00	0.00	0.00	0.00	0.00	0.00	0.00
0.00	0.00	0.00	0.00	0.00	0.00	0.00	0.00	0.00

0.00	0.00	0.00	0.00	0.00	0.00	0.00	0.00	0.00
0.00	0.00	0.00	0.00	0.00	0.00	0.00	0.00	0.00
0.00	0.00	0.00	0.00	0.00	0.00	0.00	0.00	0.00
0.00	0.00	0.00	2.00	0.00	0.00	0.00	0.00	0.00
0.00	0.00	0.00	0.00	0.00	0.00	0.00	0.00	0.00
0.00	0.00	0.00	0.00	0.00	0.00	0.00	0.00	0.00
0.00	0.00	0.00	0.00	0.00	0.00	0.00	0.00	0.00
0.00	130.00	0.00	0.00	0.00	0.00	0.00	0.00	0.00
0.00	0.00	0.00	0.00	0.00	0.00	0.00	0.00	0.00
0.00	0.00	0.00	0.00	0.00	8.00	0.00	0.00	0.00
0.00	0.00	0.00	0.00	0.00	0.00	0.00	0.00	0.00
0.00	0.00	0.00	0.00	0.00	0.00	0.00	0.00	0.00
0.00	0.00	0.00	0.00	0.00	0.00	0.00	0.00	0.00
0.00	0.00	0.00	0.00	0.00	0.00	0.00	0.00	0.00
0.00	0.00	0.00	0.00	0.00	0.00	0.00	0.00	0.00
0.00	0.00	0.00	0.00	0.00	0.00	0.00	0.00	0.00
0.00	0.00	0.00	0.00	0.00	0.00	0.00	0.00	0.00
0.00	0.00	0.00	0.00	0.00	0.00	0.00	0.00	0.00
0.00	0.00	0.00	0.00	0.00	0.00	0.00	0.00	0.00
0.00	0.00	0.00	0.00	0.00	0.00	0.00	0.00	0.00
0.00	0.00	0.00	0.00	0.00	0.00	0.00	0.00	0.00
0.00	0.00	0.00	0.00	0.00	0.00	0.00	0.00	0.00
0.00	0.00	0.00	0.00	0.00	0.00	0.00	0.00	0.00
0.00	0.00	0.00	0.00	0.00	0.00	0.00	0.00	0.00
0.00	0.00	0.00	0.00	0.00	0.00	0.00	0.00	0.00
0.00	0.00	0.00	0.00	0.00	0.00	0.00	0.00	0.00
0.00	0.00	0.00	0.00	0.00	0.00	0.00	0.00	0.00
0.00	0.00	0.00	0.00	0.00	0.00	0.00	0.00	0.00
0.00	0.00	0.00	0.00	0.00	0.00	0.00	0.00	0.00
0.00	0.00	0.00	0.00	0.00	0.00	0.00	0.00	0.00
0.00	0.00	0.00	0.00	0.00	0.00	0.00	0.00	0.00
0.00	0.00	0.00	0.00	0.00	0.00	0.00	0.00	0.00
0.00	0.00	0.00	0.00	0.00	0.00	0.00	0.00	0.00
0.00	0.00	0.00	0.00	0.00	0.00	0.00	0.00	0.00
0.00	0.00	0.00	0.00	0.00	0.00	0.00	0.00	0.00
0.00	0.00	0.00	0.00	0.00	0.00	0.00	116.00	0.00

0.00	0.00	0.00	0.00	0.00	0.00	0.00	0.00	0.00
0.00	0.00	0.00	0.00	0.00	0.00	0.00	0.00	0.00
0.00	0.00	0.00	0.00	0.00	0.00	0.00	0.00	0.00
0.00	0.00	0.00	0.00	0.00	0.00	0.00	0.00	0.00
0.00	0.00	0.00	0.00	0.00	0.00	0.00	0.00	0.00
0.00	0.00	0.00	0.00	0.00	0.00	0.00	0.00	0.00
0.00	0.00	0.00	0.00	0.00	0.00	0.00	0.00	0.00
0.00	0.00	0.00	0.00	0.00	0.00	0.00	0.00	0.00
0.00	0.00	0.00	0.00	0.00	0.00	0.00	0.00	0.00
0.00	0.00	0.00	0.00	0.00	0.00	0.00	0.00	0.00
0.00	0.00	0.00	0.00	0.00	0.00	0.00	0.00	0.00
0.00	0.00	0.00	0.00	0.00	0.00	0.00	0.00	0.00
0.00	0.00	13.00	0.00	0.00	0.00	0.00	0.00	0.00
0.00	0.00	0.00	0.00	0.00	0.00	0.00	0.00	0.00
0.00	0.00	0.00	0.00	0.00	0.00	0.00	0.00	0.00
0.00	0.00	0.00	0.00	0.00	0.00	0.00	0.00	0.00
0.00	0.00	0.00	0.00	0.00	0.00	0.00	0.00	0.00
0.00	0.00	0.00	0.00	0.00	0.00	0.00	0.00	0.00
0.00	0.00	0.00	0.00	0.00	0.00	0.00	0.00	0.00
0.00	0.00	0.00	0.00	0.00	0.00	0.00	107.00	0.00
0.00	0.00	0.00	0.00	0.00	0.00	0.00	0.00	0.00
0.00	0.00	0.00	0.00	0.00	0.00	0.00	0.00	0.00
0.00	0.00	0.00	0.00	0.00	0.00	0.00	0.00	0.00
0.00	0.00	0.00	0.00	0.00	0.00	0.00	0.00	0.00
0.00	0.00	0.00	0.00	0.00	0.00	0.00	0.00	0.00
0.00	0.00	0.00	0.00	0.00	0.00	0.00	0.00	0.00
0.00	0.00	0.00	0.00	0.00	0.00	0.00	0.00	0.00
0.00	0.00	0.00	0.00	0.00	0.00	0.00	0.00	103.00
0.00	0.00	0.00	0.00	0.00	0.00	0.00	0.00	0.00
0.00	0.00	0.00	0.00	0.00	0.00	0.00	0.00	0.00
0.00	0.00	0.00	12.00	0.00	0.00	0.00	0.00	0.00
0.00	0.00	0.00	0.00	0.00	0.00	0.00	0.00	0.00
0.00	0.00	0.00	0.00	0.00	0.00	0.00	0.00	0.00
0.00	0.00	0.00	0.00	0.00	0.00	0.00	0.00	0.00
0.00	0.00	0.00	0.00	0.00	0.00	0.00	0.00	0.00
0.00	0.00	0.00	0.00	0.00	0.00	0.00	0.00	0.00
0.00	0.00	0.00	0.00	0.00	0.00	0.00	0.00	0.00

0.00	0.00	0.00	0.00	0.00	0.00	0.00	0.00	0.00
0.00	0.00	0.00	0.00	0.00	0.00	0.00	0.00	0.00
0.00	0.00	0.00	0.00	0.00	0.00	0.00	0.00	0.00
0.00	0.00	0.00	0.00	0.00	0.00	0.00	0.00	0.00
0.00	0.00	0.00	0.00	0.00	0.00	0.00	0.00	0.00
0.00	0.00	0.00	0.00	0.00	0.00	0.00	0.00	0.00
0.00	0.00	0.00	0.00	0.00	0.00	0.00	0.00	0.00
0.00	0.00	0.00	0.00	0.00	0.00	0.00	0.00	0.00
0.00	0.00	0.00	0.00	0.00	0.00	0.00	0.00	0.00
0.00	0.00	0.00	0.00	0.00	0.00	0.00	0.00	0.00
0.00	0.00	0.00	0.00	0.00	0.00	0.00	0.00	0.00
0.00	0.00	0.00	0.00	0.00	0.00	0.00	0.00	0.00
0.00	0.00	0.00	0.00	0.00	0.00	0.00	0.00	0.00
0.00	0.00	0.00	0.00	0.00	0.00	0.00	0.00	0.00
0.00	0.00	0.00	0.00	0.00	0.00	0.00	0.00	0.00
0.00	0.00	0.00	0.00	0.00	0.00	0.00	0.00	0.00
0.00	0.00	0.00	0.00	0.00	0.00	0.00	0.00	0.00
0.00	0.00	0.00	0.00	0.00	0.00	0.00	0.00	0.00
0.00	0.00	0.00	0.00	0.00	0.00	0.00	0.00	0.00
0.00	0.00	0.00	0.00	0.00	0.00	0.00	0.00	0.00
0.00	0.00	0.00	0.00	0.00	0.00	0.00	0.00	0.00
0.00	0.00	0.00	0.00	0.00	0.00	0.00	0.00	0.00
0.00	0.00	0.00	0.00	0.00	0.00	0.00	0.00	0.00
0.00	0.00	0.00	0.00	0.00	0.00	0.00	0.00	0.00
0.00	0.00	0.00	0.00	0.00	0.00	0.00	0.00	0.00
0.00	0.00	0.00	0.00	0.00	0.00	0.00	0.00	21.00
0.00	0.00	0.00	0.00	0.00	0.00	0.00	0.00	0.00
0.00	0.00	0.00	0.00	0.00	0.00	0.00	0.00	0.00
0.00	0.00	0.00	0.00	0.00	0.00	0.00	0.00	0.00
0.00	0.00	0.00	0.00	0.00	0.00	0.00	0.00	0.00
0.00	0.00	17.00	0.00	0.00	0.00	0.00	0.00	0.00
0.00	0.00	0.00	0.00	0.00	0.00	0.00	0.00	0.00
0.00	0.00	0.00	0.00	0.00	0.00	0.00	0.00	0.00
0.00	0.00	0.00	0.00	0.00	0.00	0.00	0.00	0.00
0.00	0.00	0.00	0.00	0.00	0.00	0.00	0.00	0.00
0.00	0.00	0.00	0.00	0.00	0.00	0.00	0.00	0.00
0.00	0.00	0.00	0.00	8.00	0.00	0.00	0.00	0.00

0.00	0.00	0.00	0.00	0.00	0.00	0.00	4.00	0.00
0.00	0.00	0.00	0.00	0.00	0.00	0.00	0.00	0.00
0.00	0.00	0.00	0.00	14.00	0.00	0.00	0.00	0.00
0.00	0.00	0.00	0.00	0.00	0.00	0.00	0.00	0.00
0.00	0.00	0.00	0.00	0.00	0.00	0.00	11.00	0.00
0.00	0.00	0.00	0.00	0.00	0.00	0.00	29.00	0.00
0.00	0.00	0.00	0.00	0.00	0.00	0.00	0.00	0.00
0.00	0.00	0.00	0.00	0.00	0.00	0.00	0.00	0.00
0.00	0.00	0.00	0.00	0.00	0.00	0.00	0.00	0.00
0.00	0.00	0.00	0.00	0.00	0.00	0.00	0.00	0.00
0.00	0.00	0.00	0.00	0.00	0.00	0.00	0.00	0.00
0.00	0.00	0.00	0.00	0.00	0.00	0.00	0.00	0.00
50.00	0.00	0.00	0.00	0.00	0.00	0.00	0.00	0.00
0.00	0.00	0.00	0.00	0.00	0.00	0.00	0.00	0.00
0.00	0.00	0.00	0.00	0.00	0.00	0.00	0.00	0.00
0.00	0.00	0.00	0.00	0.00	0.00	0.00	0.00	0.00
0.00	0.00	0.00	0.00	0.00	0.00	0.00	0.00	0.00
0.00	0.00	0.00	0.00	0.00	0.00	0.00	0.00	0.00
0.00	0.00	0.00	0.00	0.00	0.00	0.00	0.00	0.00
0.00	0.00	0.00	0.00	0.00	0.00	0.00	0.00	0.00
12.00	0.00	0.00	0.00	0.00	0.00	0.00	0.00	0.00
0.00	0.00	0.00	0.00	0.00	0.00	0.00	0.00	0.00
0.00	0.00	0.00	0.00	0.00	0.00	0.00	0.00	0.00
0.00	0.00	0.00	0.00	0.00	0.00	0.00	0.00	0.00
0.00	0.00	0.00	0.00	0.00	0.00	0.00	0.00	0.00
0.00	0.00	0.00	0.00	0.00	0.00	0.00	0.00	0.00
0.00	0.00	0.00	0.00	0.00	0.00	0.00	0.00	0.00
0.00	0.00	0.00	0.00	0.00	0.00	0.00	3.00	4.00
0.00	0.00	0.00	0.00	0.00	0.00	0.00	0.00	81.00
0.00	0.00	0.00	0.00	0.00	0.00	0.00	0.00	0.00
0.00	0.00	0.00	0.00	0.00	0.00	0.00	0.00	0.00
0.00	0.00	0.00	0.00	0.00	0.00	0.00	0.00	0.00
0.00	0.00	0.00	0.00	0.00	0.00	0.00	0.00	0.00
0.00	0.00	0.00	0.00	0.00	0.00	0.00	0.00	0.00
0.00	0.00	0.00	0.00	0.00	0.00	0.00	0.00	0.00
0.00	0.00	0.00	0.00	0.00	0.00	79.00	0.00	0.00
0.00	0.00	0.00	0.00	0.00	0.00	0.00	0.00	0.00

0.00	0.00	0.00	0.00	0.00	0.00	0.00	0.00	0.00
0.00	0.00	0.00	0.00	0.00	0.00	0.00	0.00	0.00
0.00	0.00	0.00	0.00	0.00	0.00	0.00	0.00	0.00
0.00	0.00	8.00	0.00	0.00	0.00	59.00	0.00	0.00
0.00	0.00	0.00	0.00	0.00	0.00	0.00	0.00	0.00
0.00	0.00	0.00	0.00	0.00	0.00	0.00	0.00	0.00
0.00	0.00	0.00	0.00	0.00	0.00	0.00	0.00	0.00
0.00	0.00	0.00	0.00	0.00	0.00	0.00	0.00	0.00
0.00	0.00	0.00	0.00	0.00	0.00	0.00	0.00	0.00
0.00	0.00	0.00	0.00	0.00	0.00	0.00	0.00	0.00
0.00	0.00	0.00	0.00	0.00	0.00	0.00	0.00	0.00
0.00	0.00	0.00	0.00	0.00	0.00	0.00	0.00	0.00
0.00	0.00	0.00	0.00	0.00	0.00	0.00	0.00	0.00
0.00	0.00	0.00	0.00	0.00	0.00	0.00	0.00	0.00
0.00	0.00	0.00	0.00	0.00	0.00	0.00	0.00	0.00
0.00	0.00	0.00	0.00	0.00	0.00	0.00	0.00	0.00
0.00	0.00	0.00	0.00	0.00	0.00	0.00	0.00	0.00
0.00	0.00	0.00	0.00	0.00	0.00	0.00	0.00	0.00
0.00	0.00	0.00	0.00	0.00	0.00	0.00	0.00	0.00
0.00	0.00	0.00	0.00	0.00	0.00	0.00	0.00	0.00
0.00	0.00	0.00	0.00	0.00	0.00	0.00	0.00	0.00
0.00	0.00	0.00	0.00	0.00	0.00	0.00	0.00	0.00
0.00	0.00	0.00	0.00	0.00	0.00	0.00	0.00	0.00
0.00	0.00	0.00	0.00	0.00	0.00	0.00	0.00	0.00
0.00	0.00	0.00	0.00	0.00	0.00	0.00	0.00	0.00
0.00	0.00	0.00	0.00	0.00	0.00	0.00	0.00	0.00
0.00	0.00	0.00	0.00	0.00	0.00	0.00	0.00	0.00
0.00	0.00	0.00	0.00	0.00	0.00	0.00	0.00	0.00
0.00	0.00	0.00	0.00	0.00	0.00	0.00	0.00	0.00
0.00	0.00	0.00	0.00	0.00	0.00	0.00	0.00	0.00
0.00	0.00	0.00	0.00	0.00	0.00	0.00	0.00	0.00
0.00	0.00	0.00	0.00	0.00	0.00	0.00	0.00	0.00
0.00	0.00	0.00	0.00	0.00	0.00	0.00	0.00	0.00
0.00	0.00	0.00	0.00	0.00	0.00	0.00	0.00	0.00
0.00	0.00	0.00	0.00	0.00	0.00	0.00	0.00	0.00
0.00	0.00	0.00	0.00	0.00	0.00	0.00	0.00	0.00
0.00	0.00	0.00	0.00	0.00	0.00	0.00	0.00	0.00

0.00	0.00	0.00	0.00	0.00	0.00	0.00	0.00	0.00
0.00	0.00	0.00	0.00	0.00	0.00	0.00	0.00	0.00
0.00	0.00	0.00	0.00	0.00	0.00	0.00	0.00	0.00
0.00	0.00	0.00	0.00	0.00	0.00	0.00	0.00	0.00
0.00	0.00	0.00	0.00	0.00	0.00	0.00	21.00	0.00
0.00	0.00	0.00	0.00	0.00	0.00	0.00	0.00	0.00
0.00	0.00	0.00	0.00	0.00	0.00	0.00	0.00	0.00
0.00	0.00	0.00	0.00	0.00	0.00	0.00	0.00	23.00
0.00	0.00	0.00	0.00	0.00	0.00	0.00	0.00	0.00
0.00	0.00	0.00	0.00	0.00	0.00	0.00	0.00	0.00
0.00	0.00	0.00	0.00	0.00	0.00	0.00	0.00	0.00
0.00	0.00	0.00	0.00	0.00	0.00	0.00	0.00	0.00
0.00	0.00	0.00	0.00	0.00	0.00	0.00	0.00	0.00
0.00	0.00	0.00	0.00	0.00	0.00	0.00	0.00	0.00
0.00	0.00	0.00	0.00	0.00	0.00	0.00	0.00	0.00
0.00	0.00	0.00	0.00	0.00	0.00	0.00	0.00	0.00
0.00	0.00	0.00	0.00	0.00	0.00	0.00	68.00	0.00
0.00	0.00	0.00	0.00	0.00	0.00	0.00	0.00	0.00
0.00	0.00	0.00	0.00	0.00	0.00	0.00	0.00	0.00
0.00	0.00	0.00	0.00	0.00	0.00	0.00	0.00	4.00
0.00	0.00	0.00	0.00	0.00	0.00	0.00	0.00	0.00
0.00	0.00	0.00	0.00	0.00	0.00	0.00	0.00	0.00
0.00	0.00	0.00	0.00	0.00	0.00	0.00	0.00	0.00
0.00	0.00	0.00	0.00	0.00	0.00	0.00	0.00	0.00
0.00	0.00	0.00	0.00	0.00	0.00	0.00	0.00	0.00
0.00	0.00	0.00	0.00	0.00	0.00	0.00	0.00	5.00
0.00	0.00	0.00	0.00	0.00	0.00	0.00	0.00	0.00
0.00	0.00	0.00	0.00	0.00	0.00	0.00	0.00	0.00
0.00	0.00	0.00	0.00	0.00	0.00	0.00	0.00	0.00
0.00	0.00	0.00	0.00	0.00	0.00	0.00	0.00	0.00
0.00	0.00	0.00	0.00	0.00	0.00	0.00	5.00	5.00
0.00	0.00	0.00	0.00	0.00	0.00	0.00	0.00	0.00
0.00	0.00	0.00	0.00	0.00	0.00	0.00	0.00	0.00
0.00	0.00	0.00	0.00	0.00	0.00	0.00	0.00	0.00
0.00	0.00	0.00	0.00	0.00	8.00	0.00	0.00	0.00
0.00	0.00	0.00	0.00	0.00	0.00	0.00	0.00	0.00
0.00	0.00	0.00	0.00	0.00	0.00	0.00	0.00	0.00

0.00	0.00	0.00	0.00	0.00	0.00	0.00	0.00	0.00
0.00	0.00	0.00	0.00	0.00	0.00	0.00	0.00	0.00
0.00	0.00	0.00	0.00	0.00	0.00	0.00	0.00	0.00
0.00	0.00	0.00	0.00	0.00	0.00	0.00	0.00	0.00
0.00	0.00	0.00	0.00	0.00	0.00	0.00	0.00	0.00
0.00	0.00	0.00	0.00	0.00	0.00	0.00	0.00	0.00
0.00	0.00	0.00	0.00	0.00	0.00	0.00	0.00	0.00
0.00	0.00	0.00	0.00	0.00	0.00	0.00	0.00	34.00
0.00	0.00	0.00	0.00	0.00	0.00	0.00	0.00	0.00
0.00	0.00	0.00	0.00	0.00	0.00	0.00	0.00	0.00
0.00	0.00	0.00	0.00	0.00	0.00	0.00	0.00	16.00
0.00	0.00	0.00	0.00	0.00	0.00	0.00	0.00	0.00
0.00	0.00	0.00	0.00	0.00	0.00	0.00	12.00	0.00
0.00	0.00	0.00	0.00	0.00	0.00	0.00	0.00	0.00
0.00	0.00	0.00	0.00	0.00	0.00	0.00	0.00	0.00
0.00	0.00	0.00	0.00	0.00	0.00	0.00	0.00	0.00
0.00	0.00	0.00	0.00	0.00	0.00	0.00	0.00	0.00
0.00	0.00	0.00	0.00	0.00	0.00	0.00	0.00	0.00
0.00	0.00	0.00	0.00	0.00	0.00	0.00	0.00	0.00
0.00	0.00	0.00	0.00	0.00	0.00	0.00	0.00	0.00
0.00	0.00	0.00	0.00	0.00	0.00	0.00	0.00	0.00
0.00	0.00	0.00	9.00	0.00	0.00	0.00	0.00	0.00
0.00	0.00	0.00	0.00	0.00	0.00	32.00	0.00	0.00
0.00	0.00	0.00	0.00	0.00	0.00	0.00	0.00	0.00
0.00	0.00	0.00	0.00	0.00	0.00	0.00	0.00	0.00
0.00	0.00	0.00	0.00	0.00	0.00	0.00	0.00	0.00
0.00	0.00	0.00	0.00	0.00	0.00	0.00	0.00	0.00
0.00	0.00	0.00	0.00	0.00	0.00	0.00	0.00	0.00
0.00	0.00	0.00	0.00	0.00	0.00	0.00	0.00	0.00
0.00	0.00	0.00	0.00	0.00	0.00	0.00	0.00	0.00
0.00	0.00	0.00	0.00	0.00	0.00	0.00	0.00	0.00
0.00	0.00	0.00	0.00	0.00	0.00	0.00	0.00	0.00
0.00	0.00	0.00	0.00	0.00	0.00	0.00	0.00	0.00
0.00	0.00	0.00	0.00	0.00	0.00	0.00	0.00	0.00
0.00	0.00	0.00	0.00	0.00	0.00	0.00	0.00	0.00
0.00	0.00	0.00	0.00	0.00	0.00	0.00	0.00	0.00
0.00	0.00	0.00	0.00	0.00	0.00	0.00	0.00	0.00

0.00	0.00	0.00	0.00	0.00	0.00	0.00	0.00	0.00
0.00	0.00	0.00	0.00	0.00	0.00	0.00	0.00	0.00
0.00	0.00	0.00	0.00	0.00	0.00	0.00	0.00	0.00
0.00	0.00	0.00	0.00	0.00	0.00	0.00	59.00	0.00
0.00	0.00	0.00	0.00	0.00	0.00	0.00	0.00	0.00
0.00	0.00	0.00	0.00	0.00	0.00	0.00	0.00	0.00
0.00	0.00	0.00	0.00	0.00	0.00	0.00	0.00	0.00
0.00	0.00	0.00	0.00	0.00	0.00	0.00	0.00	0.00
0.00	0.00	0.00	0.00	0.00	0.00	0.00	9.00	0.00
0.00	0.00	0.00	0.00	0.00	0.00	0.00	0.00	0.00
0.00	0.00	0.00	0.00	0.00	0.00	0.00	0.00	0.00
0.00	0.00	0.00	0.00	0.00	0.00	0.00	0.00	0.00
0.00	0.00	0.00	0.00	0.00	0.00	0.00	0.00	0.00
0.00	0.00	0.00	0.00	0.00	0.00	0.00	0.00	0.00
0.00	0.00	0.00	0.00	0.00	0.00	0.00	0.00	0.00
0.00	0.00	0.00	0.00	0.00	0.00	0.00	0.00	0.00
0.00	0.00	0.00	0.00	0.00	0.00	0.00	0.00	0.00
0.00	0.00	0.00	0.00	0.00	0.00	0.00	0.00	0.00
0.00	0.00	0.00	0.00	0.00	0.00	0.00	0.00	0.00
0.00	0.00	0.00	0.00	0.00	0.00	0.00	0.00	0.00
0.00	0.00	0.00	0.00	0.00	0.00	0.00	0.00	0.00
0.00	0.00	0.00	0.00	0.00	0.00	0.00	0.00	0.00
0.00	0.00	0.00	0.00	0.00	0.00	0.00	0.00	0.00
0.00	0.00	0.00	0.00	0.00	0.00	0.00	0.00	0.00
0.00	0.00	0.00	0.00	0.00	0.00	0.00	0.00	0.00
0.00	0.00	0.00	0.00	0.00	0.00	0.00	0.00	0.00
0.00	0.00	0.00	0.00	0.00	0.00	0.00	0.00	0.00
0.00	0.00	0.00	0.00	0.00	0.00	0.00	0.00	0.00
0.00	0.00	0.00	0.00	0.00	0.00	0.00	0.00	0.00
0.00	0.00	0.00	0.00	0.00	0.00	0.00	0.00	0.00
0.00	0.00	0.00	0.00	0.00	0.00	0.00	0.00	0.00
0.00	0.00	0.00	0.00	0.00	0.00	0.00	0.00	0.00
0.00	0.00	0.00	0.00	0.00	0.00	0.00	0.00	0.00
0.00	0.00	0.00	0.00	0.00	0.00	0.00	0.00	0.00
0.00	0.00	0.00	0.00	0.00	0.00	0.00	0.00	0.00
0.00	0.00	0.00	0.00	0.00	0.00	0.00	0.00	0.00
0.00	0.00	0.00	0.00	0.00	0.00	0.00	0.00	0.00

0.00	0.00	0.00	0.00	0.00	0.00	0.00	0.00	0.00
0.00	0.00	0.00	0.00	0.00	0.00	54.00	0.00	0.00
0.00	0.00	0.00	0.00	0.00	0.00	0.00	0.00	0.00
0.00	0.00	0.00	0.00	0.00	0.00	0.00	5.00	0.00
0.00	0.00	0.00	0.00	0.00	0.00	0.00	0.00	0.00
0.00	0.00	0.00	0.00	0.00	0.00	0.00	0.00	0.00
0.00	0.00	0.00	0.00	0.00	0.00	0.00	0.00	0.00
0.00	0.00	0.00	0.00	0.00	0.00	0.00	0.00	0.00
0.00	0.00	0.00	0.00	0.00	0.00	0.00	0.00	0.00
0.00	0.00	0.00	0.00	0.00	0.00	0.00	0.00	0.00
0.00	0.00	0.00	0.00	0.00	0.00	0.00	0.00	0.00
0.00	0.00	0.00	0.00	0.00	0.00	0.00	0.00	0.00
0.00	0.00	0.00	0.00	0.00	0.00	0.00	0.00	0.00
0.00	0.00	0.00	0.00	0.00	0.00	0.00	0.00	0.00
0.00	0.00	0.00	0.00	0.00	0.00	0.00	6.00	0.00
0.00	0.00	0.00	0.00	0.00	0.00	0.00	0.00	0.00
0.00	0.00	0.00	0.00	0.00	0.00	0.00	0.00	0.00
0.00	0.00	0.00	0.00	0.00	0.00	0.00	0.00	0.00
0.00	0.00	0.00	0.00	0.00	0.00	0.00	0.00	0.00
0.00	0.00	0.00	0.00	0.00	0.00	0.00	0.00	0.00
0.00	0.00	0.00	0.00	0.00	0.00	0.00	0.00	0.00
0.00	0.00	0.00	0.00	0.00	0.00	0.00	0.00	0.00
0.00	0.00	0.00	0.00	0.00	0.00	0.00	0.00	0.00
0.00	0.00	0.00	11.00	0.00	0.00	0.00	3.00	0.00
0.00	0.00	0.00	0.00	0.00	0.00	0.00	0.00	0.00
0.00	0.00	0.00	0.00	0.00	0.00	0.00	0.00	0.00
0.00	0.00	0.00	0.00	0.00	0.00	0.00	0.00	0.00
0.00	0.00	0.00	0.00	0.00	0.00	0.00	0.00	0.00
0.00	0.00	0.00	0.00	0.00	0.00	0.00	0.00	0.00
0.00	0.00	0.00	0.00	0.00	0.00	0.00	0.00	0.00
0.00	0.00	0.00	0.00	0.00	0.00	0.00	0.00	0.00
0.00	0.00	0.00	0.00	0.00	0.00	0.00	0.00	0.00
0.00	0.00	0.00	0.00	0.00	0.00	0.00	0.00	0.00
0.00	0.00	0.00	0.00	0.00	0.00	0.00	0.00	0.00
0.00	0.00	0.00	0.00	0.00	0.00	0.00	0.00	0.00
0.00	0.00	0.00	0.00	0.00	0.00	0.00	0.00	0.00
0.00	0.00	0.00	0.00	0.00	0.00	0.00	0.00	0.00

0.00	0.00	0.00	0.00	0.00	0.00	0.00	0.00	49.00
0.00	0.00	0.00	0.00	0.00	0.00	0.00	0.00	0.00
0.00	0.00	0.00	0.00	0.00	0.00	0.00	0.00	0.00
0.00	0.00	0.00	0.00	0.00	0.00	0.00	0.00	0.00
0.00	0.00	0.00	0.00	0.00	0.00	0.00	22.00	0.00
0.00	0.00	0.00	0.00	0.00	0.00	0.00	0.00	0.00
0.00	0.00	0.00	0.00	0.00	0.00	0.00	0.00	0.00
0.00	0.00	0.00	0.00	0.00	0.00	0.00	0.00	0.00
0.00	0.00	0.00	0.00	0.00	0.00	0.00	0.00	0.00
0.00	0.00	0.00	0.00	0.00	0.00	0.00	0.00	0.00
0.00	0.00	0.00	0.00	0.00	0.00	0.00	0.00	0.00
0.00	0.00	0.00	0.00	0.00	0.00	0.00	0.00	0.00
0.00	0.00	0.00	0.00	0.00	0.00	0.00	0.00	0.00
0.00	0.00	0.00	0.00	0.00	0.00	0.00	0.00	0.00
0.00	0.00	0.00	0.00	0.00	0.00	0.00	0.00	0.00
0.00	0.00	0.00	0.00	0.00	0.00	0.00	0.00	5.00
0.00	0.00	0.00	0.00	0.00	0.00	0.00	0.00	0.00
0.00	0.00	0.00	0.00	0.00	0.00	0.00	0.00	0.00
0.00	0.00	0.00	0.00	0.00	0.00	0.00	0.00	0.00
0.00	0.00	0.00	0.00	0.00	0.00	0.00	0.00	0.00
0.00	0.00	0.00	0.00	0.00	0.00	0.00	0.00	0.00
0.00	0.00	0.00	0.00	0.00	0.00	0.00	0.00	0.00
0.00	0.00	0.00	0.00	0.00	0.00	0.00	0.00	0.00
0.00	0.00	0.00	0.00	0.00	0.00	0.00	0.00	0.00
0.00	0.00	0.00	0.00	0.00	0.00	0.00	0.00	0.00
0.00	0.00	0.00	0.00	0.00	0.00	0.00	0.00	3.00
0.00	0.00	0.00	0.00	0.00	0.00	0.00	0.00	0.00
0.00	0.00	0.00	0.00	0.00	0.00	0.00	0.00	0.00
0.00	0.00	0.00	0.00	0.00	0.00	0.00	0.00	0.00
0.00	0.00	0.00	0.00	0.00	0.00	0.00	0.00	0.00
0.00	0.00	0.00	0.00	0.00	0.00	0.00	0.00	0.00
0.00	0.00	0.00	0.00	0.00	0.00	0.00	0.00	0.00
0.00	0.00	0.00	0.00	0.00	0.00	0.00	0.00	0.00
0.00	0.00	0.00	0.00	0.00	0.00	0.00	0.00	0.00
0.00	0.00	0.00	0.00	0.00	0.00	0.00	0.00	0.00
0.00	0.00	0.00	0.00	0.00	0.00	0.00	0.00	0.00
0.00	0.00	0.00	0.00	0.00	0.00	0.00	0.00	0.00

0.00	0.00	0.00	0.00	0.00	0.00	0.00	0.00	0.00
0.00	0.00	0.00	0.00	0.00	0.00	0.00	0.00	0.00
0.00	0.00	0.00	0.00	0.00	0.00	0.00	0.00	0.00
0.00	0.00	0.00	0.00	0.00	0.00	0.00	0.00	0.00
0.00	0.00	0.00	0.00	0.00	0.00	0.00	0.00	0.00
0.00	0.00	0.00	0.00	0.00	0.00	0.00	0.00	0.00
0.00	0.00	0.00	0.00	0.00	0.00	0.00	0.00	0.00
0.00	0.00	0.00	0.00	0.00	0.00	0.00	8.00	0.00
0.00	0.00	0.00	0.00	0.00	0.00	0.00	0.00	0.00
0.00	0.00	0.00	0.00	0.00	0.00	0.00	0.00	0.00
0.00	0.00	0.00	0.00	0.00	0.00	0.00	0.00	0.00
0.00	0.00	0.00	0.00	0.00	0.00	0.00	0.00	0.00
0.00	0.00	3.00	0.00	0.00	0.00	0.00	0.00	0.00
0.00	0.00	0.00	0.00	0.00	0.00	0.00	0.00	0.00
0.00	0.00	0.00	0.00	0.00	0.00	0.00	0.00	0.00
0.00	0.00	0.00	0.00	0.00	0.00	0.00	4.00	0.00
0.00	0.00	0.00	0.00	0.00	0.00	0.00	0.00	0.00
0.00	0.00	0.00	0.00	0.00	0.00	0.00	0.00	0.00
0.00	0.00	0.00	0.00	0.00	0.00	0.00	0.00	0.00
0.00	0.00	0.00	0.00	0.00	0.00	0.00	0.00	0.00
0.00	0.00	0.00	0.00	0.00	0.00	0.00	0.00	0.00
0.00	0.00	0.00	0.00	0.00	0.00	0.00	0.00	0.00
0.00	0.00	0.00	0.00	0.00	0.00	0.00	0.00	0.00
0.00	0.00	0.00	0.00	0.00	0.00	0.00	0.00	0.00
0.00	0.00	0.00	0.00	0.00	0.00	0.00	0.00	0.00
0.00	0.00	0.00	0.00	0.00	0.00	0.00	0.00	0.00
0.00	0.00	0.00	0.00	0.00	0.00	0.00	0.00	0.00
0.00	0.00	0.00	0.00	0.00	0.00	0.00	0.00	0.00
0.00	0.00	0.00	0.00	0.00	0.00	0.00	0.00	0.00
0.00	0.00	0.00	0.00	0.00	0.00	0.00	0.00	0.00
0.00	0.00	0.00	0.00	0.00	0.00	0.00	0.00	0.00
0.00	0.00	0.00	0.00	0.00	0.00	0.00	0.00	0.00
0.00	0.00	0.00	0.00	0.00	0.00	0.00	0.00	0.00
0.00	0.00	0.00	0.00	0.00	0.00	0.00	0.00	0.00
0.00	0.00	0.00	0.00	0.00	0.00	0.00	0.00	0.00
0.00	0.00	0.00	0.00	0.00	0.00	0.00	0.00	0.00
0.00	0.00	0.00	0.00	0.00	0.00	0.00	0.00	19.00

0.00	0.00	0.00	0.00	0.00	0.00	0.00	0.00	0.00
0.00	0.00	0.00	0.00	0.00	0.00	0.00	0.00	0.00
0.00	0.00	0.00	0.00	0.00	0.00	0.00	0.00	0.00
0.00	0.00	0.00	0.00	0.00	0.00	0.00	0.00	0.00
18.00	0.00	0.00	0.00	0.00	0.00	0.00	0.00	0.00
0.00	0.00	40.00	0.00	0.00	0.00	0.00	0.00	0.00
0.00	0.00	0.00	0.00	0.00	0.00	0.00	0.00	0.00
0.00	0.00	0.00	0.00	0.00	0.00	0.00	0.00	0.00
0.00	0.00	0.00	0.00	0.00	0.00	0.00	40.00	0.00
0.00	0.00	0.00	0.00	0.00	0.00	0.00	0.00	0.00
0.00	0.00	0.00	0.00	0.00	0.00	0.00	0.00	0.00
0.00	0.00	0.00	0.00	0.00	0.00	0.00	0.00	0.00
0.00	0.00	0.00	0.00	0.00	0.00	0.00	0.00	0.00
0.00	0.00	0.00	0.00	0.00	0.00	0.00	0.00	0.00
0.00	0.00	0.00	0.00	0.00	0.00	0.00	21.00	0.00
0.00	0.00	0.00	0.00	0.00	0.00	0.00	3.00	0.00
0.00	0.00	0.00	0.00	0.00	0.00	0.00	0.00	0.00
0.00	0.00	0.00	0.00	0.00	0.00	0.00	0.00	0.00
0.00	0.00	0.00	0.00	0.00	0.00	0.00	0.00	0.00
0.00	0.00	0.00	0.00	0.00	0.00	0.00	0.00	0.00
0.00	0.00	0.00	0.00	0.00	0.00	0.00	0.00	0.00
0.00	0.00	0.00	0.00	0.00	0.00	0.00	0.00	0.00
0.00	0.00	0.00	0.00	0.00	0.00	0.00	0.00	0.00
0.00	0.00	0.00	0.00	0.00	0.00	0.00	0.00	0.00
0.00	0.00	0.00	0.00	0.00	0.00	0.00	0.00	0.00
0.00	0.00	0.00	0.00	0.00	0.00	0.00	0.00	0.00
0.00	0.00	0.00	0.00	0.00	0.00	0.00	0.00	0.00
0.00	0.00	0.00	0.00	0.00	0.00	0.00	0.00	0.00
0.00	0.00	0.00	0.00	0.00	0.00	0.00	0.00	0.00
0.00	0.00	0.00	0.00	0.00	0.00	0.00	0.00	0.00
0.00	0.00	0.00	0.00	0.00	0.00	0.00	0.00	0.00
0.00	0.00	0.00	0.00	0.00	0.00	0.00	0.00	0.00
0.00	0.00	0.00	0.00	0.00	0.00	0.00	0.00	0.00
0.00	0.00	0.00	0.00	0.00	0.00	0.00	0.00	0.00
0.00	0.00	0.00	0.00	0.00	0.00	0.00	0.00	0.00
0.00	0.00	0.00	0.00	0.00	0.00	0.00	0.00	0.00
0.00	0.00	0.00	0.00	0.00	0.00	0.00	0.00	0.00

0.00	0.00	0.00	0.00	0.00	0.00	0.00	0.00	0.00
0.00	0.00	0.00	0.00	0.00	0.00	0.00	0.00	0.00
0.00	0.00	0.00	0.00	0.00	0.00	0.00	0.00	0.00
0.00	0.00	0.00	0.00	0.00	0.00	0.00	0.00	0.00
0.00	0.00	0.00	0.00	0.00	0.00	0.00	0.00	37.00
0.00	0.00	0.00	0.00	0.00	0.00	0.00	0.00	0.00
0.00	0.00	0.00	0.00	0.00	0.00	0.00	0.00	0.00
0.00	0.00	0.00	0.00	0.00	0.00	0.00	0.00	0.00
0.00	0.00	0.00	0.00	0.00	0.00	0.00	0.00	0.00
0.00	0.00	0.00	0.00	0.00	0.00	0.00	0.00	0.00
0.00	0.00	0.00	0.00	0.00	0.00	0.00	0.00	0.00
0.00	0.00	0.00	0.00	0.00	0.00	0.00	0.00	0.00
0.00	0.00	0.00	0.00	0.00	0.00	0.00	0.00	0.00
0.00	0.00	0.00	0.00	0.00	0.00	0.00	0.00	0.00
0.00	0.00	0.00	0.00	0.00	0.00	0.00	0.00	0.00
0.00	0.00	0.00	0.00	0.00	0.00	0.00	0.00	0.00
0.00	0.00	0.00	0.00	0.00	0.00	0.00	0.00	0.00
0.00	0.00	0.00	0.00	0.00	0.00	0.00	0.00	0.00
0.00	0.00	0.00	0.00	0.00	0.00	0.00	0.00	0.00
0.00	0.00	0.00	0.00	0.00	0.00	0.00	0.00	0.00
0.00	0.00	0.00	0.00	0.00	0.00	0.00	0.00	0.00
0.00	0.00	0.00	0.00	0.00	0.00	0.00	0.00	0.00
0.00	0.00	0.00	0.00	0.00	0.00	0.00	0.00	0.00
0.00	0.00	0.00	0.00	0.00	0.00	0.00	35.00	0.00
0.00	0.00	0.00	0.00	3.00	0.00	0.00	0.00	0.00
0.00	0.00	0.00	34.00	0.00	0.00	0.00	0.00	0.00
0.00	0.00	0.00	0.00	0.00	0.00	0.00	0.00	0.00
0.00	0.00	0.00	0.00	0.00	0.00	0.00	0.00	0.00
0.00	0.00	0.00	0.00	0.00	0.00	0.00	0.00	0.00
0.00	0.00	0.00	0.00	0.00	0.00	0.00	0.00	0.00
0.00	0.00	0.00	0.00	0.00	0.00	0.00	0.00	0.00
0.00	0.00	0.00	0.00	0.00	0.00	0.00	0.00	0.00
0.00	33.00	0.00	0.00	0.00	0.00	0.00	0.00	0.00
0.00	0.00	0.00	0.00	0.00	0.00	0.00	4.00	0.00
0.00	0.00	0.00	0.00	0.00	0.00	0.00	0.00	0.00
0.00	0.00	0.00	0.00	0.00	0.00	0.00	0.00	0.00
0.00	0.00	0.00	0.00	0.00	0.00	0.00	0.00	0.00

0.00	0.00	0.00	0.00	0.00	0.00	0.00	0.00	0.00
0.00	0.00	0.00	0.00	0.00	0.00	0.00	0.00	0.00
0.00	0.00	0.00	0.00	0.00	0.00	0.00	0.00	33.00
0.00	0.00	0.00	0.00	0.00	0.00	0.00	0.00	0.00
0.00	0.00	0.00	0.00	0.00	0.00	0.00	0.00	0.00
0.00	0.00	0.00	0.00	0.00	0.00	0.00	0.00	0.00
0.00	0.00	0.00	0.00	0.00	0.00	0.00	0.00	0.00
0.00	0.00	0.00	0.00	0.00	0.00	0.00	0.00	0.00
0.00	0.00	0.00	0.00	0.00	0.00	0.00	0.00	0.00
0.00	0.00	0.00	0.00	0.00	0.00	0.00	0.00	0.00
0.00	0.00	0.00	0.00	0.00	0.00	0.00	0.00	0.00
0.00	0.00	0.00	0.00	0.00	0.00	0.00	0.00	0.00
0.00	0.00	0.00	0.00	0.00	0.00	0.00	0.00	0.00
0.00	0.00	0.00	0.00	0.00	0.00	0.00	0.00	0.00
0.00	0.00	0.00	0.00	0.00	0.00	0.00	0.00	0.00
0.00	0.00	0.00	0.00	0.00	0.00	0.00	0.00	0.00
0.00	0.00	0.00	0.00	0.00	0.00	0.00	0.00	0.00
0.00	0.00	0.00	0.00	0.00	0.00	0.00	0.00	0.00
0.00	0.00	0.00	0.00	0.00	0.00	0.00	0.00	0.00
0.00	0.00	0.00	0.00	0.00	0.00	0.00	0.00	0.00
0.00	0.00	0.00	0.00	0.00	0.00	0.00	0.00	0.00
0.00	0.00	0.00	0.00	0.00	0.00	0.00	0.00	0.00
0.00	0.00	0.00	0.00	0.00	0.00	0.00	0.00	0.00
0.00	0.00	0.00	0.00	0.00	0.00	0.00	0.00	0.00
0.00	0.00	0.00	0.00	0.00	0.00	0.00	0.00	0.00
0.00	0.00	0.00	0.00	0.00	0.00	0.00	0.00	0.00
0.00	0.00	0.00	0.00	0.00	0.00	0.00	0.00	0.00
0.00	0.00	0.00	0.00	0.00	0.00	0.00	0.00	0.00
0.00	0.00	0.00	0.00	0.00	0.00	0.00	0.00	0.00
0.00	0.00	0.00	0.00	0.00	0.00	0.00	0.00	0.00
0.00	0.00	0.00	0.00	0.00	0.00	0.00	0.00	0.00
0.00	0.00	0.00	0.00	0.00	0.00	0.00	0.00	0.00
0.00	0.00	0.00	0.00	0.00	0.00	0.00	0.00	0.00
0.00	0.00	0.00	11.00	0.00	0.00	0.00	0.00	0.00
0.00	0.00	0.00	0.00	0.00	0.00	0.00	0.00	0.00
0.00	0.00	0.00	0.00	0.00	0.00	0.00	0.00	0.00
0.00	0.00	0.00	0.00	0.00	0.00	0.00	0.00	0.00

0.00	0.00	0.00	0.00	0.00	0.00	0.00	0.00	0.00
0.00	0.00	0.00	0.00	0.00	0.00	0.00	0.00	0.00
0.00	0.00	0.00	0.00	0.00	0.00	0.00	0.00	0.00
0.00	0.00	0.00	0.00	0.00	0.00	0.00	0.00	0.00
0.00	0.00	0.00	0.00	0.00	0.00	0.00	0.00	0.00
0.00	0.00	0.00	0.00	0.00	0.00	0.00	0.00	0.00
0.00	0.00	0.00	0.00	0.00	0.00	0.00	0.00	0.00
0.00	0.00	0.00	0.00	0.00	0.00	0.00	0.00	0.00
0.00	0.00	0.00	0.00	0.00	0.00	0.00	0.00	0.00
0.00	0.00	0.00	0.00	0.00	0.00	0.00	0.00	0.00
0.00	0.00	0.00	0.00	0.00	0.00	0.00	0.00	0.00
0.00	0.00	0.00	0.00	0.00	0.00	0.00	0.00	0.00
0.00	0.00	0.00	0.00	0.00	0.00	0.00	0.00	0.00
0.00	0.00	0.00	0.00	0.00	0.00	27.00	0.00	0.00
0.00	0.00	0.00	0.00	0.00	0.00	0.00	0.00	0.00
0.00	0.00	0.00	0.00	0.00	0.00	0.00	0.00	0.00
0.00	0.00	0.00	0.00	0.00	0.00	0.00	0.00	0.00
0.00	0.00	0.00	0.00	0.00	0.00	0.00	0.00	0.00
0.00	0.00	0.00	0.00	0.00	0.00	0.00	0.00	0.00
0.00	0.00	0.00	0.00	0.00	0.00	0.00	3.00	0.00
0.00	0.00	0.00	0.00	0.00	0.00	0.00	0.00	0.00
0.00	0.00	0.00	0.00	0.00	0.00	0.00	0.00	0.00
0.00	0.00	0.00	0.00	0.00	0.00	0.00	0.00	0.00
0.00	0.00	0.00	0.00	0.00	0.00	0.00	0.00	0.00
0.00	0.00	0.00	0.00	0.00	0.00	0.00	0.00	0.00
0.00	0.00	0.00	0.00	0.00	0.00	0.00	0.00	0.00
0.00	0.00	0.00	0.00	0.00	0.00	0.00	0.00	0.00
0.00	0.00	0.00	0.00	0.00	0.00	0.00	0.00	0.00
0.00	0.00	0.00	0.00	0.00	0.00	0.00	0.00	0.00
0.00	0.00	0.00	0.00	0.00	0.00	0.00	0.00	0.00
0.00	0.00	0.00	0.00	0.00	0.00	0.00	6.00	0.00
0.00	0.00	0.00	0.00	0.00	0.00	0.00	0.00	0.00
0.00	0.00	0.00	0.00	0.00	0.00	0.00	0.00	0.00
0.00	0.00	0.00	0.00	0.00	0.00	0.00	0.00	0.00
0.00	0.00	0.00	0.00	0.00	0.00	0.00	0.00	0.00
0.00	0.00	0.00	0.00	0.00	0.00	0.00	0.00	0.00
0.00	0.00	0.00	0.00	0.00	0.00	20.00	0.00	0.00

0.00	0.00	0.00	0.00	0.00	0.00	0.00	0.00	0.00
0.00	27.00	0.00	0.00	0.00	0.00	0.00	0.00	0.00
0.00	0.00	0.00	12.00	0.00	0.00	15.00	0.00	0.00
0.00	0.00	0.00	0.00	0.00	0.00	0.00	0.00	0.00
0.00	0.00	0.00	0.00	0.00	0.00	0.00	0.00	0.00
0.00	0.00	0.00	0.00	0.00	0.00	27.00	0.00	0.00
0.00	0.00	0.00	0.00	0.00	0.00	0.00	0.00	0.00
0.00	0.00	0.00	0.00	0.00	0.00	0.00	0.00	0.00
0.00	0.00	0.00	0.00	0.00	0.00	0.00	0.00	0.00
0.00	0.00	0.00	0.00	0.00	0.00	0.00	0.00	0.00
0.00	0.00	0.00	0.00	0.00	0.00	0.00	0.00	0.00
0.00	0.00	0.00	0.00	0.00	0.00	0.00	0.00	0.00
0.00	0.00	0.00	0.00	0.00	0.00	0.00	0.00	0.00
0.00	0.00	0.00	0.00	0.00	0.00	0.00	0.00	0.00
0.00	0.00	0.00	0.00	0.00	0.00	0.00	0.00	0.00
0.00	0.00	0.00	0.00	0.00	0.00	0.00	0.00	0.00
0.00	0.00	0.00	0.00	0.00	0.00	0.00	0.00	0.00
0.00	0.00	0.00	0.00	0.00	0.00	0.00	0.00	0.00
0.00	0.00	0.00	0.00	0.00	0.00	0.00	0.00	0.00
0.00	0.00	0.00	0.00	0.00	0.00	0.00	0.00	0.00
0.00	0.00	0.00	0.00	0.00	0.00	0.00	0.00	0.00
0.00	0.00	0.00	0.00	0.00	0.00	0.00	0.00	0.00
0.00	0.00	0.00	9.00	0.00	0.00	0.00	0.00	0.00
0.00	0.00	0.00	0.00	0.00	0.00	0.00	0.00	0.00
0.00	0.00	0.00	0.00	0.00	0.00	0.00	0.00	0.00
0.00	0.00	0.00	0.00	0.00	0.00	26.00	0.00	0.00
0.00	0.00	0.00	0.00	0.00	0.00	0.00	0.00	0.00
0.00	0.00	0.00	0.00	0.00	0.00	0.00	0.00	0.00
0.00	0.00	0.00	0.00	0.00	0.00	0.00	0.00	0.00
0.00	0.00	0.00	0.00	0.00	0.00	0.00	0.00	0.00
0.00	0.00	0.00	0.00	0.00	0.00	0.00	0.00	0.00
0.00	0.00	0.00	0.00	0.00	0.00	0.00	0.00	0.00
0.00	0.00	0.00	0.00	0.00	0.00	0.00	0.00	0.00
0.00	0.00	0.00	4.00	0.00	0.00	0.00	0.00	0.00
0.00	0.00	0.00	0.00	0.00	0.00	0.00	0.00	0.00
0.00	0.00	0.00	0.00	0.00	0.00	0.00	0.00	0.00
0.00	0.00	0.00	0.00	0.00	0.00	0.00	0.00	0.00

0.00	0.00	0.00	0.00	0.00	0.00	0.00	0.00	0.00
0.00	0.00	0.00	0.00	0.00	0.00	0.00	0.00	0.00
0.00	0.00	0.00	0.00	0.00	0.00	0.00	0.00	0.00
0.00	0.00	0.00	0.00	0.00	0.00	0.00	0.00	0.00
0.00	0.00	0.00	0.00	0.00	0.00	0.00	0.00	0.00
0.00	0.00	0.00	0.00	0.00	0.00	0.00	0.00	0.00
0.00	0.00	0.00	0.00	0.00	0.00	0.00	0.00	0.00
0.00	0.00	0.00	0.00	0.00	0.00	0.00	0.00	0.00
0.00	0.00	0.00	0.00	0.00	0.00	0.00	0.00	0.00
0.00	0.00	0.00	0.00	0.00	0.00	0.00	0.00	0.00
0.00	0.00	0.00	0.00	0.00	0.00	0.00	0.00	0.00
0.00	0.00	0.00	0.00	24.00	0.00	0.00	0.00	0.00
0.00	0.00	0.00	0.00	0.00	0.00	0.00	0.00	0.00
0.00	0.00	0.00	0.00	0.00	0.00	0.00	0.00	0.00
0.00	0.00	0.00	0.00	0.00	0.00	0.00	0.00	0.00
0.00	0.00	0.00	6.00	0.00	0.00	0.00	0.00	0.00
0.00	0.00	0.00	0.00	0.00	0.00	0.00	0.00	0.00
0.00	0.00	0.00	0.00	0.00	0.00	0.00	0.00	0.00
0.00	0.00	0.00	0.00	0.00	0.00	0.00	0.00	0.00
0.00	0.00	0.00	0.00	0.00	0.00	0.00	0.00	0.00
0.00	0.00	0.00	0.00	0.00	0.00	0.00	0.00	0.00
0.00	0.00	0.00	0.00	0.00	0.00	0.00	0.00	0.00
0.00	0.00	0.00	0.00	0.00	0.00	0.00	0.00	0.00
0.00	0.00	0.00	0.00	0.00	0.00	0.00	0.00	0.00
0.00	0.00	0.00	0.00	0.00	0.00	0.00	0.00	0.00
0.00	0.00	0.00	0.00	0.00	0.00	0.00	5.00	0.00
0.00	0.00	0.00	0.00	0.00	0.00	0.00	0.00	0.00
0.00	0.00	0.00	0.00	0.00	0.00	0.00	0.00	0.00
0.00	0.00	0.00	0.00	0.00	0.00	0.00	0.00	0.00
0.00	0.00	0.00	0.00	0.00	0.00	0.00	24.00	0.00
0.00	0.00	0.00	0.00	0.00	0.00	0.00	24.00	0.00
0.00	0.00	0.00	0.00	0.00	0.00	0.00	0.00	0.00
23.00	0.00	0.00	0.00	0.00	0.00	0.00	0.00	0.00
0.00	0.00	0.00	0.00	0.00	0.00	0.00	0.00	0.00
0.00	0.00	0.00	0.00	0.00	0.00	0.00	0.00	0.00
0.00	0.00	0.00	0.00	0.00	0.00	0.00	0.00	0.00
0.00	0.00	0.00	0.00	0.00	0.00	0.00	0.00	0.00

0.00	0.00	0.00	0.00	0.00	0.00	0.00	0.00	0.00
0.00	0.00	0.00	0.00	0.00	0.00	0.00	0.00	6.00
0.00	0.00	0.00	0.00	0.00	0.00	0.00	0.00	0.00
0.00	0.00	0.00	0.00	0.00	0.00	0.00	0.00	0.00
0.00	0.00	0.00	0.00	0.00	0.00	0.00	0.00	0.00
0.00	0.00	0.00	0.00	0.00	0.00	0.00	0.00	0.00
0.00	0.00	0.00	0.00	0.00	0.00	9.00	0.00	0.00
0.00	0.00	0.00	0.00	0.00	0.00	0.00	0.00	0.00
0.00	0.00	0.00	0.00	0.00	0.00	0.00	0.00	0.00
0.00	0.00	0.00	0.00	0.00	0.00	0.00	0.00	0.00
0.00	0.00	0.00	0.00	0.00	0.00	0.00	0.00	0.00
0.00	0.00	0.00	0.00	0.00	0.00	0.00	0.00	0.00
0.00	0.00	0.00	0.00	0.00	0.00	0.00	0.00	0.00
0.00	0.00	0.00	0.00	0.00	0.00	0.00	0.00	0.00
0.00	0.00	0.00	0.00	0.00	0.00	0.00	0.00	0.00
0.00	0.00	0.00	0.00	0.00	0.00	0.00	0.00	0.00
0.00	0.00	0.00	0.00	0.00	0.00	0.00	0.00	0.00
0.00	0.00	0.00	0.00	0.00	0.00	0.00	0.00	0.00
0.00	0.00	0.00	0.00	0.00	0.00	0.00	0.00	0.00
0.00	0.00	0.00	0.00	0.00	0.00	0.00	0.00	0.00
0.00	0.00	0.00	0.00	0.00	0.00	0.00	0.00	0.00
0.00	0.00	0.00	0.00	0.00	0.00	0.00	0.00	0.00
0.00	0.00	0.00	0.00	0.00	0.00	0.00	0.00	0.00
0.00	0.00	0.00	0.00	0.00	0.00	0.00	0.00	0.00
0.00	0.00	0.00	0.00	0.00	0.00	0.00	0.00	0.00
0.00	0.00	0.00	0.00	0.00	0.00	0.00	0.00	0.00
0.00	0.00	0.00	0.00	0.00	0.00	0.00	0.00	0.00
0.00	0.00	0.00	0.00	0.00	0.00	0.00	0.00	0.00
0.00	0.00	0.00	0.00	0.00	0.00	0.00	0.00	0.00
0.00	0.00	0.00	0.00	0.00	0.00	0.00	0.00	0.00
0.00	0.00	0.00	0.00	0.00	0.00	0.00	0.00	0.00
0.00	0.00	0.00	0.00	0.00	0.00	0.00	0.00	0.00
0.00	0.00	0.00	0.00	0.00	0.00	0.00	0.00	0.00
0.00	0.00	0.00	0.00	0.00	0.00	0.00	0.00	0.00
0.00	0.00	0.00	0.00	0.00	0.00	0.00	0.00	0.00
0.00	0.00	0.00	0.00	0.00	0.00	0.00	0.00	0.00
0.00	0.00	0.00	0.00	0.00	0.00	0.00	0.00	0.00

0.00	0.00	0.00	0.00	0.00	0.00	0.00	0.00	0.00
0.00	0.00	0.00	0.00	0.00	0.00	0.00	0.00	0.00
0.00	0.00	0.00	0.00	0.00	0.00	0.00	0.00	0.00
0.00	0.00	0.00	0.00	0.00	0.00	0.00	0.00	0.00
0.00	0.00	0.00	0.00	0.00	0.00	0.00	0.00	0.00
0.00	0.00	0.00	0.00	0.00	0.00	0.00	0.00	0.00
0.00	0.00	0.00	0.00	0.00	0.00	0.00	0.00	0.00
0.00	0.00	0.00	0.00	0.00	0.00	0.00	0.00	0.00
0.00	0.00	0.00	0.00	0.00	0.00	0.00	0.00	0.00
0.00	0.00	0.00	0.00	0.00	0.00	0.00	0.00	0.00
0.00	0.00	0.00	0.00	0.00	0.00	0.00	0.00	0.00
0.00	0.00	0.00	0.00	0.00	0.00	0.00	0.00	0.00
0.00	0.00	0.00	0.00	0.00	0.00	0.00	0.00	0.00
0.00	0.00	0.00	0.00	0.00	0.00	0.00	0.00	0.00
0.00	0.00	0.00	0.00	0.00	0.00	0.00	0.00	0.00
0.00	0.00	0.00	0.00	0.00	0.00	0.00	0.00	0.00
0.00	0.00	0.00	0.00	0.00	0.00	0.00	0.00	0.00
0.00	0.00	0.00	0.00	0.00	0.00	0.00	0.00	0.00
0.00	0.00	0.00	0.00	0.00	0.00	0.00	4.00	2.00
0.00	0.00	0.00	0.00	0.00	0.00	0.00	0.00	0.00
0.00	0.00	0.00	0.00	0.00	0.00	0.00	0.00	0.00
0.00	0.00	0.00	0.00	0.00	0.00	0.00	0.00	0.00
0.00	0.00	0.00	0.00	0.00	0.00	0.00	0.00	20.00
0.00	0.00	0.00	0.00	0.00	0.00	0.00	0.00	0.00
0.00	0.00	0.00	0.00	0.00	0.00	0.00	0.00	0.00
0.00	0.00	0.00	0.00	0.00	0.00	0.00	0.00	0.00
0.00	0.00	0.00	0.00	0.00	0.00	0.00	0.00	0.00
0.00	0.00	0.00	0.00	0.00	0.00	0.00	0.00	0.00
0.00	0.00	0.00	0.00	0.00	0.00	0.00	0.00	0.00
0.00	0.00	0.00	0.00	0.00	0.00	0.00	0.00	0.00
0.00	0.00	0.00	0.00	0.00	0.00	0.00	0.00	0.00
0.00	0.00	0.00	0.00	0.00	0.00	0.00	0.00	0.00
0.00	0.00	0.00	0.00	0.00	0.00	0.00	0.00	0.00
0.00	0.00	0.00	0.00	0.00	0.00	0.00	0.00	0.00
0.00	0.00	0.00	0.00	0.00	0.00	0.00	0.00	0.00
0.00	0.00	0.00	0.00	0.00	0.00	0.00	0.00	0.00
0.00	0.00	0.00	0.00	0.00	0.00	0.00	0.00	0.00

0.00	0.00	0.00	0.00	19.00	0.00	0.00	0.00	0.00
0.00	0.00	0.00	0.00	0.00	0.00	0.00	0.00	0.00
0.00	0.00	0.00	0.00	0.00	0.00	0.00	0.00	0.00
0.00	0.00	0.00	0.00	0.00	0.00	0.00	0.00	0.00
0.00	0.00	0.00	0.00	0.00	0.00	0.00	0.00	0.00
0.00	0.00	0.00	0.00	0.00	0.00	0.00	0.00	0.00
0.00	0.00	0.00	0.00	0.00	0.00	0.00	0.00	0.00
0.00	0.00	0.00	0.00	0.00	0.00	0.00	0.00	0.00
0.00	0.00	0.00	0.00	0.00	0.00	0.00	2.00	0.00
0.00	0.00	0.00	0.00	0.00	0.00	0.00	0.00	0.00
0.00	0.00	0.00	0.00	0.00	0.00	0.00	0.00	0.00
0.00	0.00	0.00	0.00	0.00	0.00	0.00	0.00	0.00
0.00	0.00	0.00	0.00	0.00	0.00	0.00	0.00	0.00
0.00	0.00	0.00	0.00	0.00	0.00	0.00	0.00	0.00
0.00	0.00	0.00	0.00	0.00	0.00	0.00	0.00	0.00
0.00	0.00	0.00	0.00	0.00	0.00	0.00	0.00	0.00
0.00	0.00	0.00	0.00	0.00	0.00	0.00	0.00	0.00
0.00	0.00	0.00	0.00	0.00	0.00	0.00	0.00	0.00
0.00	0.00	0.00	0.00	0.00	0.00	0.00	0.00	0.00
0.00	0.00	0.00	0.00	0.00	0.00	0.00	0.00	0.00
0.00	0.00	0.00	0.00	0.00	0.00	0.00	0.00	0.00
0.00	0.00	0.00	0.00	0.00	0.00	0.00	0.00	0.00
0.00	0.00	0.00	6.00	0.00	0.00	0.00	0.00	0.00
0.00	0.00	0.00	0.00	0.00	0.00	0.00	0.00	0.00
0.00	0.00	0.00	0.00	0.00	0.00	0.00	0.00	0.00
0.00	0.00	0.00	0.00	0.00	0.00	0.00	0.00	0.00
0.00	0.00	0.00	0.00	0.00	0.00	0.00	0.00	0.00
0.00	0.00	0.00	0.00	0.00	0.00	0.00	0.00	0.00
0.00	0.00	0.00	0.00	0.00	0.00	0.00	0.00	0.00
0.00	0.00	0.00	0.00	0.00	0.00	0.00	0.00	0.00
8.00	0.00	0.00	0.00	0.00	0.00	0.00	0.00	0.00
0.00	0.00	0.00	0.00	0.00	0.00	0.00	0.00	0.00
0.00	0.00	0.00	0.00	0.00	0.00	0.00	0.00	0.00
0.00	0.00	0.00	0.00	0.00	0.00	0.00	0.00	0.00
0.00	0.00	0.00	0.00	0.00	0.00	0.00	0.00	0.00
0.00	0.00	0.00	0.00	0.00	0.00	0.00	0.00	0.00
0.00	0.00	0.00	0.00	0.00	0.00	0.00	0.00	0.00

0.00	0.00	0.00	0.00	0.00	0.00	0.00	0.00	0.00
0.00	0.00	0.00	0.00	0.00	0.00	0.00	0.00	0.00
0.00	0.00	0.00	0.00	0.00	0.00	0.00	5.00	0.00
0.00	0.00	0.00	0.00	0.00	0.00	0.00	0.00	0.00
0.00	0.00	0.00	0.00	0.00	0.00	0.00	0.00	0.00
0.00	0.00	0.00	0.00	0.00	0.00	0.00	0.00	0.00
0.00	0.00	0.00	0.00	0.00	0.00	0.00	0.00	0.00
0.00	0.00	0.00	0.00	0.00	0.00	0.00	0.00	0.00
0.00	0.00	0.00	0.00	0.00	0.00	0.00	0.00	0.00
0.00	0.00	0.00	0.00	0.00	0.00	0.00	0.00	0.00
0.00	0.00	0.00	0.00	0.00	0.00	0.00	0.00	0.00
0.00	0.00	0.00	0.00	0.00	0.00	0.00	0.00	0.00
0.00	0.00	0.00	0.00	0.00	0.00	0.00	0.00	0.00
0.00	0.00	0.00	0.00	0.00	0.00	0.00	0.00	0.00
0.00	0.00	0.00	0.00	0.00	0.00	0.00	0.00	0.00
0.00	0.00	0.00	0.00	0.00	0.00	0.00	0.00	0.00
0.00	0.00	0.00	0.00	0.00	0.00	0.00	0.00	0.00
0.00	0.00	0.00	0.00	0.00	0.00	0.00	0.00	0.00
0.00	0.00	0.00	0.00	0.00	0.00	0.00	0.00	0.00
0.00	0.00	0.00	0.00	0.00	0.00	0.00	0.00	2.00
0.00	0.00	0.00	0.00	0.00	0.00	0.00	0.00	0.00
0.00	0.00	0.00	0.00	0.00	0.00	0.00	0.00	0.00
0.00	0.00	0.00	0.00	0.00	0.00	0.00	0.00	0.00
0.00	0.00	0.00	0.00	0.00	0.00	0.00	0.00	0.00
0.00	0.00	0.00	0.00	0.00	0.00	0.00	0.00	0.00
0.00	0.00	0.00	0.00	0.00	0.00	0.00	0.00	0.00
0.00	0.00	0.00	0.00	0.00	0.00	0.00	0.00	0.00
0.00	0.00	0.00	0.00	0.00	0.00	0.00	0.00	5.00
0.00	0.00	0.00	0.00	0.00	0.00	0.00	0.00	0.00
0.00	0.00	0.00	0.00	0.00	0.00	0.00	7.00	0.00
0.00	0.00	0.00	0.00	0.00	0.00	0.00	0.00	0.00
0.00	0.00	0.00	0.00	0.00	0.00	0.00	0.00	0.00
0.00	0.00	0.00	0.00	0.00	0.00	0.00	0.00	0.00
0.00	0.00	0.00	0.00	0.00	0.00	0.00	0.00	0.00
0.00	0.00	0.00	0.00	0.00	0.00	0.00	0.00	17.00
0.00	0.00	0.00	0.00	0.00	0.00	0.00	0.00	0.00
0.00	0.00	0.00	0.00	0.00	0.00	0.00	0.00	0.00

0.00	0.00	0.00	0.00	0.00	0.00	0.00	0.00	0.00
0.00	0.00	0.00	0.00	0.00	0.00	0.00	0.00	0.00
0.00	0.00	0.00	0.00	0.00	0.00	0.00	0.00	0.00
0.00	0.00	0.00	0.00	0.00	0.00	0.00	0.00	0.00
0.00	0.00	0.00	0.00	0.00	0.00	0.00	0.00	0.00
0.00	0.00	0.00	0.00	0.00	0.00	0.00	0.00	0.00
0.00	0.00	0.00	0.00	0.00	0.00	0.00	0.00	0.00
0.00	0.00	0.00	0.00	0.00	0.00	0.00	0.00	0.00
0.00	0.00	0.00	0.00	0.00	0.00	0.00	0.00	0.00
0.00	0.00	0.00	0.00	0.00	0.00	0.00	0.00	0.00
0.00	0.00	0.00	0.00	0.00	0.00	0.00	0.00	0.00
0.00	0.00	0.00	0.00	0.00	0.00	0.00	0.00	0.00
0.00	0.00	0.00	0.00	0.00	0.00	0.00	0.00	0.00
0.00	0.00	0.00	0.00	0.00	0.00	0.00	0.00	0.00
0.00	0.00	0.00	0.00	0.00	0.00	0.00	0.00	0.00
0.00	0.00	0.00	0.00	0.00	0.00	0.00	0.00	0.00
0.00	0.00	0.00	0.00	0.00	0.00	0.00	0.00	0.00
0.00	0.00	16.00	0.00	0.00	0.00	0.00	0.00	0.00
0.00	0.00	0.00	16.00	0.00	0.00	0.00	0.00	0.00
0.00	0.00	0.00	0.00	0.00	0.00	0.00	0.00	0.00
0.00	0.00	0.00	0.00	0.00	0.00	16.00	0.00	0.00
0.00	0.00	0.00	0.00	0.00	0.00	0.00	0.00	0.00
0.00	0.00	0.00	0.00	0.00	0.00	0.00	0.00	0.00
0.00	0.00	0.00	0.00	0.00	0.00	0.00	0.00	0.00
0.00	0.00	0.00	0.00	0.00	0.00	0.00	0.00	0.00
0.00	0.00	0.00	0.00	0.00	0.00	0.00	0.00	0.00
0.00	0.00	0.00	0.00	0.00	0.00	0.00	0.00	0.00
0.00	0.00	0.00	0.00	0.00	0.00	0.00	0.00	0.00
0.00	0.00	0.00	0.00	0.00	0.00	0.00	0.00	0.00
0.00	0.00	0.00	0.00	0.00	0.00	0.00	0.00	0.00
0.00	0.00	0.00	0.00	0.00	0.00	0.00	0.00	0.00
0.00	0.00	0.00	0.00	0.00	0.00	0.00	0.00	0.00
0.00	0.00	0.00	0.00	0.00	0.00	0.00	0.00	0.00
0.00	0.00	0.00	0.00	0.00	0.00	0.00	0.00	0.00
0.00	0.00	0.00	0.00	0.00	0.00	0.00	0.00	0.00
0.00	0.00	0.00	0.00	0.00	0.00	0.00	0.00	0.00
0.00	0.00	0.00	0.00	0.00	0.00	0.00	0.00	0.00

0.00	0.00	0.00	0.00	0.00	0.00	0.00	0.00	0.00
0.00	0.00	0.00	0.00	0.00	0.00	0.00	0.00	0.00
0.00	0.00	0.00	0.00	0.00	0.00	0.00	0.00	0.00
0.00	0.00	0.00	0.00	0.00	0.00	0.00	0.00	0.00
0.00	0.00	0.00	0.00	0.00	0.00	0.00	0.00	0.00
0.00	0.00	0.00	0.00	9.00	0.00	0.00	0.00	0.00
0.00	0.00	0.00	0.00	0.00	0.00	0.00	0.00	0.00
0.00	0.00	0.00	0.00	0.00	0.00	0.00	0.00	0.00
0.00	0.00	0.00	0.00	0.00	0.00	0.00	0.00	0.00
0.00	0.00	0.00	0.00	0.00	0.00	0.00	0.00	0.00
0.00	0.00	0.00	0.00	0.00	0.00	0.00	0.00	0.00
0.00	0.00	0.00	0.00	0.00	0.00	0.00	0.00	0.00
0.00	0.00	0.00	0.00	0.00	0.00	0.00	0.00	0.00
0.00	0.00	0.00	0.00	0.00	0.00	0.00	0.00	0.00
0.00	0.00	0.00	0.00	0.00	0.00	0.00	0.00	0.00
0.00	0.00	0.00	0.00	0.00	0.00	0.00	0.00	0.00
0.00	0.00	0.00	0.00	0.00	0.00	0.00	0.00	0.00
0.00	0.00	0.00	0.00	0.00	0.00	0.00	0.00	0.00
0.00	0.00	0.00	0.00	0.00	0.00	0.00	0.00	0.00
0.00	0.00	0.00	0.00	0.00	0.00	0.00	0.00	0.00
0.00	0.00	0.00	0.00	0.00	0.00	0.00	0.00	0.00
0.00	0.00	0.00	0.00	0.00	0.00	0.00	0.00	0.00
0.00	0.00	0.00	0.00	0.00	0.00	0.00	0.00	0.00
0.00	0.00	0.00	0.00	0.00	0.00	0.00	0.00	0.00
0.00	0.00	0.00	0.00	0.00	0.00	0.00	0.00	0.00
0.00	0.00	0.00	0.00	0.00	0.00	0.00	0.00	0.00
0.00	0.00	0.00	0.00	0.00	0.00	0.00	0.00	0.00
0.00	0.00	0.00	0.00	0.00	0.00	0.00	0.00	0.00
0.00	0.00	0.00	0.00	0.00	0.00	0.00	0.00	0.00
0.00	0.00	0.00	0.00	0.00	0.00	0.00	0.00	0.00
0.00	0.00	0.00	0.00	0.00	0.00	0.00	0.00	0.00
0.00	0.00	0.00	0.00	0.00	0.00	0.00	0.00	0.00
0.00	0.00	0.00	0.00	0.00	0.00	0.00	0.00	0.00
0.00	0.00	0.00	0.00	0.00	0.00	0.00	0.00	0.00
0.00	0.00	0.00	0.00	0.00	0.00	0.00	0.00	0.00
0.00	0.00	0.00	0.00	0.00	0.00	0.00	0.00	0.00
0.00	0.00	0.00	0.00	0.00	0.00	0.00	0.00	0.00

0.00	0.00	0.00	0.00	0.00	0.00	0.00	0.00	0.00
0.00	0.00	0.00	0.00	0.00	0.00	0.00	0.00	0.00
0.00	0.00	0.00	0.00	0.00	0.00	0.00	0.00	0.00
0.00	0.00	0.00	0.00	0.00	0.00	0.00	0.00	0.00
0.00	0.00	0.00	0.00	0.00	0.00	0.00	0.00	0.00
0.00	0.00	0.00	0.00	0.00	0.00	0.00	0.00	0.00
0.00	0.00	0.00	0.00	0.00	0.00	0.00	0.00	0.00
0.00	0.00	0.00	0.00	0.00	0.00	0.00	0.00	0.00
0.00	0.00	0.00	0.00	0.00	0.00	0.00	0.00	0.00
0.00	0.00	0.00	0.00	0.00	0.00	0.00	0.00	0.00
0.00	0.00	0.00	0.00	0.00	0.00	0.00	12.00	0.00
0.00	0.00	0.00	0.00	0.00	0.00	0.00	0.00	0.00
0.00	0.00	0.00	0.00	0.00	0.00	0.00	0.00	0.00
0.00	0.00	0.00	0.00	0.00	0.00	0.00	0.00	0.00
0.00	0.00	0.00	0.00	0.00	0.00	0.00	0.00	0.00
0.00	0.00	0.00	0.00	0.00	0.00	0.00	0.00	0.00
0.00	0.00	0.00	0.00	0.00	0.00	0.00	0.00	0.00
0.00	0.00	0.00	0.00	0.00	0.00	0.00	0.00	0.00
0.00	0.00	0.00	0.00	0.00	0.00	0.00	0.00	0.00
0.00	0.00	0.00	0.00	0.00	0.00	0.00	0.00	0.00
0.00	0.00	0.00	0.00	0.00	0.00	0.00	0.00	0.00
0.00	0.00	0.00	0.00	0.00	0.00	0.00	0.00	0.00
0.00	0.00	0.00	0.00	0.00	0.00	0.00	0.00	0.00
0.00	0.00	0.00	0.00	0.00	0.00	0.00	0.00	0.00
0.00	0.00	0.00	0.00	0.00	0.00	0.00	0.00	0.00
0.00	0.00	0.00	0.00	0.00	0.00	0.00	0.00	0.00
0.00	0.00	0.00	0.00	0.00	0.00	0.00	0.00	0.00
0.00	0.00	0.00	0.00	0.00	0.00	0.00	0.00	0.00
0.00	0.00	0.00	0.00	0.00	0.00	0.00	0.00	0.00
0.00	0.00	0.00	0.00	0.00	0.00	0.00	0.00	0.00
0.00	0.00	0.00	0.00	0.00	0.00	0.00	0.00	0.00
0.00	0.00	0.00	0.00	0.00	0.00	0.00	0.00	0.00
0.00	0.00	0.00	0.00	0.00	0.00	0.00	0.00	0.00
0.00	0.00	0.00	0.00	0.00	0.00	0.00	0.00	0.00
0.00	0.00	0.00	0.00	0.00	0.00	0.00	0.00	0.00
0.00	0.00	0.00	0.00	0.00	0.00	0.00	0.00	0.00
0.00	0.00	0.00	0.00	0.00	0.00	0.00	0.00	0.00

0.00	0.00	0.00	0.00	0.00	0.00	0.00	0.00	0.00
0.00	0.00	0.00	0.00	0.00	0.00	0.00	0.00	0.00
0.00	0.00	0.00	0.00	0.00	0.00	0.00	0.00	0.00
0.00	0.00	0.00	0.00	0.00	0.00	0.00	0.00	0.00
0.00	0.00	0.00	0.00	0.00	0.00	0.00	0.00	0.00
0.00	0.00	0.00	0.00	0.00	0.00	0.00	0.00	0.00
0.00	0.00	0.00	0.00	0.00	0.00	0.00	0.00	0.00
0.00	0.00	0.00	0.00	0.00	0.00	0.00	0.00	6.00
0.00	0.00	0.00	0.00	0.00	0.00	0.00	0.00	0.00
0.00	0.00	0.00	0.00	0.00	0.00	0.00	0.00	0.00
0.00	0.00	0.00	0.00	0.00	0.00	0.00	0.00	0.00
0.00	0.00	0.00	0.00	0.00	0.00	0.00	0.00	0.00
0.00	0.00	0.00	0.00	0.00	0.00	0.00	0.00	0.00
0.00	0.00	0.00	0.00	0.00	0.00	0.00	0.00	0.00
0.00	0.00	0.00	0.00	0.00	0.00	0.00	7.00	0.00
0.00	0.00	0.00	0.00	0.00	0.00	0.00	0.00	0.00
0.00	0.00	0.00	0.00	0.00	0.00	0.00	0.00	0.00
0.00	0.00	0.00	0.00	0.00	0.00	0.00	0.00	0.00
0.00	0.00	0.00	0.00	0.00	0.00	0.00	0.00	0.00
0.00	0.00	0.00	0.00	0.00	0.00	0.00	0.00	0.00
0.00	0.00	0.00	0.00	0.00	0.00	0.00	0.00	0.00
0.00	0.00	0.00	0.00	0.00	0.00	0.00	0.00	0.00
0.00	0.00	0.00	0.00	0.00	0.00	0.00	0.00	0.00
0.00	0.00	0.00	0.00	0.00	0.00	0.00	0.00	0.00
0.00	0.00	0.00	0.00	0.00	0.00	0.00	0.00	0.00
0.00	0.00	0.00	0.00	0.00	0.00	0.00	0.00	0.00
0.00	0.00	0.00	0.00	0.00	0.00	0.00	0.00	0.00
0.00	0.00	0.00	0.00	0.00	0.00	0.00	0.00	0.00
0.00	0.00	0.00	0.00	0.00	0.00	0.00	0.00	0.00
0.00	0.00	0.00	0.00	0.00	0.00	0.00	0.00	0.00
0.00	0.00	0.00	0.00	0.00	0.00	0.00	0.00	0.00
0.00	0.00	0.00	0.00	0.00	0.00	0.00	0.00	0.00
0.00	0.00	0.00	0.00	0.00	0.00	0.00	0.00	0.00
0.00	12.00	0.00	0.00	0.00	0.00	0.00	0.00	0.00
0.00	0.00	0.00	0.00	0.00	0.00	0.00	0.00	0.00
0.00	0.00	0.00	0.00	0.00	0.00	0.00	0.00	0.00
0.00	0.00	0.00	0.00	0.00	0.00	0.00	0.00	0.00

0.00	0.00	0.00	0.00	0.00	0.00	0.00	0.00	0.00
0.00	0.00	0.00	0.00	0.00	0.00	0.00	0.00	0.00
0.00	0.00	0.00	0.00	0.00	0.00	0.00	0.00	0.00
0.00	0.00	0.00	0.00	0.00	0.00	0.00	0.00	0.00
0.00	0.00	0.00	0.00	0.00	0.00	0.00	0.00	0.00
0.00	0.00	0.00	0.00	0.00	0.00	0.00	0.00	0.00
0.00	0.00	0.00	0.00	0.00	0.00	0.00	0.00	0.00
0.00	0.00	0.00	0.00	0.00	0.00	0.00	0.00	0.00
0.00	0.00	0.00	0.00	0.00	0.00	0.00	0.00	0.00
0.00	0.00	0.00	0.00	0.00	0.00	0.00	0.00	0.00
0.00	0.00	0.00	0.00	0.00	0.00	0.00	0.00	0.00
0.00	0.00	0.00	0.00	0.00	0.00	0.00	0.00	0.00
0.00	0.00	0.00	0.00	0.00	0.00	0.00	0.00	0.00
0.00	0.00	0.00	0.00	0.00	0.00	0.00	0.00	0.00
0.00	0.00	0.00	0.00	0.00	0.00	0.00	0.00	0.00
0.00	0.00	0.00	0.00	0.00	0.00	0.00	0.00	0.00
0.00	0.00	0.00	0.00	0.00	0.00	0.00	0.00	5.00
0.00	0.00	0.00	0.00	0.00	0.00	0.00	0.00	0.00
0.00	0.00	0.00	0.00	0.00	0.00	0.00	0.00	0.00
0.00	0.00	0.00	0.00	0.00	0.00	0.00	0.00	0.00
0.00	0.00	0.00	0.00	0.00	0.00	0.00	0.00	0.00
0.00	0.00	0.00	0.00	0.00	0.00	0.00	0.00	0.00
0.00	0.00	0.00	0.00	0.00	0.00	0.00	0.00	0.00
0.00	0.00	0.00	0.00	0.00	0.00	0.00	0.00	0.00
0.00	0.00	0.00	0.00	0.00	0.00	0.00	12.00	0.00
0.00	0.00	0.00	0.00	0.00	0.00	0.00	0.00	0.00
0.00	0.00	0.00	0.00	0.00	0.00	0.00	0.00	0.00
0.00	0.00	0.00	0.00	0.00	0.00	0.00	0.00	0.00
0.00	0.00	0.00	0.00	3.00	0.00	0.00	0.00	0.00
0.00	0.00	0.00	0.00	0.00	0.00	0.00	0.00	0.00
0.00	0.00	0.00	0.00	0.00	0.00	0.00	0.00	0.00
0.00	0.00	0.00	0.00	0.00	0.00	0.00	0.00	0.00
0.00	0.00	0.00	0.00	0.00	0.00	0.00	0.00	0.00
0.00	0.00	0.00	0.00	0.00	0.00	0.00	0.00	0.00
0.00	0.00	0.00	0.00	0.00	0.00	0.00	0.00	0.00
0.00	0.00	0.00	0.00	0.00	0.00	0.00	0.00	0.00
0.00	0.00	0.00	0.00	0.00	0.00	0.00	0.00	0.00

0.00	0.00	0.00	0.00	0.00	0.00	0.00	0.00	0.00
0.00	0.00	0.00	0.00	0.00	0.00	0.00	0.00	0.00
0.00	0.00	0.00	0.00	0.00	0.00	0.00	0.00	0.00
0.00	0.00	0.00	0.00	0.00	0.00	0.00	0.00	0.00
0.00	0.00	0.00	0.00	0.00	0.00	0.00	0.00	0.00
0.00	0.00	0.00	0.00	0.00	0.00	0.00	0.00	0.00
0.00	0.00	0.00	0.00	0.00	0.00	0.00	0.00	0.00
0.00	0.00	0.00	0.00	0.00	0.00	0.00	0.00	0.00
0.00	0.00	0.00	0.00	0.00	0.00	0.00	0.00	0.00
0.00	0.00	0.00	0.00	0.00	0.00	0.00	6.00	0.00
0.00	0.00	0.00	0.00	0.00	0.00	0.00	0.00	0.00
0.00	0.00	0.00	0.00	0.00	0.00	0.00	0.00	0.00
0.00	0.00	0.00	0.00	0.00	0.00	0.00	0.00	7.00
0.00	0.00	0.00	0.00	0.00	0.00	0.00	0.00	0.00
0.00	0.00	0.00	0.00	0.00	0.00	0.00	0.00	0.00
0.00	0.00	0.00	0.00	0.00	0.00	0.00	0.00	0.00
0.00	0.00	0.00	0.00	0.00	0.00	0.00	0.00	0.00
0.00	0.00	0.00	0.00	0.00	0.00	0.00	0.00	0.00
0.00	0.00	0.00	0.00	0.00	0.00	0.00	0.00	0.00
0.00	0.00	0.00	0.00	0.00	0.00	0.00	0.00	0.00
0.00	0.00	0.00	0.00	0.00	0.00	0.00	0.00	0.00
0.00	0.00	0.00	0.00	0.00	0.00	0.00	0.00	0.00
0.00	0.00	0.00	0.00	0.00	0.00	0.00	0.00	0.00
0.00	0.00	0.00	0.00	0.00	0.00	0.00	0.00	0.00
0.00	0.00	0.00	0.00	0.00	0.00	0.00	0.00	0.00
0.00	0.00	0.00	0.00	0.00	0.00	0.00	0.00	0.00
0.00	0.00	0.00	0.00	0.00	0.00	0.00	0.00	0.00
0.00	0.00	0.00	0.00	0.00	0.00	0.00	0.00	0.00
0.00	0.00	0.00	0.00	0.00	0.00	0.00	0.00	0.00
0.00	0.00	0.00	0.00	0.00	0.00	0.00	0.00	0.00
0.00	0.00	0.00	0.00	0.00	0.00	0.00	0.00	0.00
0.00	0.00	0.00	0.00	0.00	0.00	0.00	0.00	0.00
0.00	0.00	3.00	0.00	0.00	0.00	0.00	0.00	0.00
0.00	0.00	0.00	0.00	0.00	10.00	0.00	0.00	0.00
0.00	0.00	0.00	0.00	0.00	0.00	0.00	0.00	0.00
0.00	0.00	0.00	0.00	0.00	0.00	0.00	0.00	0.00
0.00	0.00	0.00	0.00	0.00	0.00	0.00	0.00	0.00

0.00	0.00	0.00	0.00	0.00	0.00	0.00	0.00	0.00
0.00	0.00	0.00	0.00	0.00	0.00	0.00	0.00	0.00
0.00	0.00	0.00	0.00	0.00	0.00	0.00	0.00	0.00
0.00	0.00	0.00	10.00	0.00	0.00	0.00	0.00	0.00
0.00	0.00	0.00	0.00	0.00	0.00	0.00	0.00	0.00
0.00	0.00	0.00	0.00	0.00	0.00	0.00	0.00	0.00
0.00	0.00	0.00	0.00	0.00	0.00	0.00	0.00	0.00
0.00	0.00	0.00	0.00	0.00	0.00	0.00	0.00	0.00
0.00	0.00	0.00	0.00	0.00	0.00	0.00	0.00	0.00
0.00	0.00	0.00	0.00	0.00	0.00	0.00	0.00	0.00
0.00	0.00	0.00	0.00	0.00	0.00	0.00	0.00	0.00
0.00	0.00	0.00	0.00	0.00	0.00	0.00	0.00	0.00
0.00	0.00	0.00	0.00	0.00	0.00	0.00	0.00	0.00
0.00	0.00	0.00	0.00	0.00	0.00	0.00	0.00	0.00
0.00	0.00	0.00	0.00	0.00	0.00	0.00	0.00	0.00
0.00	0.00	0.00	0.00	0.00	0.00	0.00	0.00	0.00
0.00	0.00	0.00	0.00	0.00	0.00	0.00	0.00	0.00
0.00	0.00	0.00	0.00	0.00	0.00	0.00	0.00	0.00
0.00	0.00	0.00	0.00	0.00	0.00	0.00	0.00	0.00
0.00	0.00	0.00	0.00	0.00	0.00	0.00	0.00	0.00
0.00	0.00	0.00	0.00	0.00	0.00	0.00	0.00	0.00
0.00	0.00	0.00	0.00	0.00	0.00	0.00	0.00	0.00
0.00	0.00	0.00	0.00	0.00	0.00	0.00	0.00	0.00
0.00	0.00	0.00	0.00	0.00	0.00	0.00	0.00	0.00
0.00	0.00	0.00	0.00	0.00	0.00	0.00	0.00	0.00
0.00	0.00	0.00	0.00	0.00	0.00	0.00	0.00	0.00
0.00	0.00	0.00	0.00	0.00	0.00	0.00	0.00	0.00
0.00	0.00	0.00	0.00	0.00	0.00	0.00	0.00	0.00
0.00	0.00	0.00	0.00	0.00	0.00	0.00	0.00	0.00
0.00	0.00	0.00	0.00	0.00	0.00	0.00	0.00	0.00
0.00	0.00	0.00	0.00	0.00	0.00	0.00	0.00	0.00
0.00	0.00	0.00	0.00	0.00	0.00	0.00	5.00	0.00
0.00	0.00	0.00	0.00	0.00	0.00	0.00	0.00	0.00
0.00	0.00	0.00	0.00	0.00	0.00	0.00	0.00	0.00
0.00	0.00	0.00	0.00	0.00	0.00	0.00	0.00	0.00
0.00	0.00	0.00	0.00	0.00	0.00	0.00	0.00	0.00
0.00	0.00	0.00	0.00	0.00	0.00	0.00	0.00	0.00

0.00	0.00	0.00	0.00	0.00	0.00	0.00	0.00	0.00
0.00	0.00	0.00	0.00	0.00	0.00	0.00	0.00	0.00
0.00	0.00	0.00	0.00	0.00	0.00	0.00	0.00	0.00
0.00	0.00	0.00	0.00	0.00	0.00	0.00	0.00	0.00
0.00	0.00	0.00	0.00	0.00	0.00	0.00	0.00	0.00
0.00	0.00	0.00	0.00	0.00	0.00	0.00	0.00	0.00
0.00	0.00	0.00	0.00	0.00	0.00	0.00	0.00	0.00
0.00	0.00	0.00	0.00	0.00	0.00	0.00	0.00	0.00
0.00	0.00	0.00	0.00	0.00	0.00	0.00	0.00	0.00
0.00	0.00	0.00	0.00	0.00	0.00	0.00	0.00	0.00
0.00	0.00	0.00	0.00	0.00	0.00	0.00	0.00	0.00
0.00	0.00	0.00	0.00	0.00	0.00	0.00	0.00	0.00
0.00	0.00	0.00	0.00	0.00	0.00	0.00	0.00	0.00
0.00	0.00	0.00	0.00	0.00	0.00	0.00	0.00	0.00
0.00	0.00	0.00	0.00	0.00	0.00	0.00	0.00	0.00
0.00	0.00	0.00	0.00	0.00	0.00	0.00	0.00	0.00
0.00	0.00	0.00	0.00	0.00	0.00	0.00	0.00	0.00
0.00	0.00	0.00	0.00	0.00	0.00	0.00	0.00	0.00
0.00	0.00	0.00	0.00	0.00	0.00	0.00	0.00	0.00
0.00	0.00	0.00	0.00	0.00	0.00	0.00	0.00	0.00
0.00	0.00	0.00	0.00	0.00	0.00	0.00	0.00	0.00
0.00	0.00	0.00	0.00	0.00	0.00	0.00	0.00	0.00
0.00	0.00	0.00	0.00	0.00	0.00	0.00	0.00	0.00
0.00	0.00	0.00	0.00	0.00	0.00	0.00	0.00	0.00
0.00	0.00	9.00	0.00	0.00	0.00	0.00	0.00	0.00
0.00	0.00	0.00	0.00	0.00	0.00	0.00	0.00	0.00
0.00	0.00	0.00	0.00	0.00	0.00	0.00	0.00	0.00
0.00	0.00	0.00	0.00	0.00	0.00	0.00	0.00	0.00
0.00	0.00	0.00	0.00	0.00	0.00	0.00	0.00	0.00
0.00	0.00	0.00	0.00	0.00	0.00	0.00	0.00	0.00
0.00	0.00	0.00	0.00	0.00	0.00	0.00	0.00	0.00
0.00	0.00	0.00	0.00	0.00	0.00	0.00	0.00	0.00
0.00	0.00	0.00	0.00	0.00	0.00	0.00	0.00	0.00
0.00	0.00	0.00	0.00	0.00	0.00	0.00	0.00	0.00
0.00	0.00	0.00	0.00	0.00	0.00	0.00	0.00	0.00
0.00	0.00	0.00	0.00	0.00	0.00	0.00	0.00	0.00
0.00	0.00	0.00	0.00	0.00	0.00	0.00	0.00	0.00

0.00	0.00	0.00	0.00	0.00	0.00	0.00	0.00	0.00
0.00	0.00	0.00	0.00	0.00	0.00	0.00	0.00	0.00
0.00	0.00	0.00	0.00	0.00	0.00	0.00	0.00	0.00
0.00	0.00	0.00	0.00	0.00	0.00	0.00	0.00	0.00
0.00	0.00	0.00	0.00	0.00	0.00	0.00	0.00	0.00
0.00	0.00	0.00	0.00	0.00	0.00	0.00	0.00	0.00
0.00	0.00	0.00	0.00	0.00	0.00	0.00	0.00	0.00
0.00	0.00	0.00	0.00	0.00	0.00	0.00	0.00	0.00
0.00	0.00	0.00	0.00	0.00	0.00	0.00	0.00	0.00
0.00	0.00	0.00	0.00	0.00	0.00	0.00	0.00	0.00
0.00	0.00	0.00	0.00	0.00	0.00	0.00	0.00	0.00
0.00	0.00	0.00	0.00	0.00	0.00	0.00	0.00	0.00
0.00	0.00	0.00	0.00	0.00	0.00	0.00	0.00	0.00
0.00	0.00	0.00	0.00	0.00	0.00	0.00	0.00	0.00
0.00	0.00	0.00	0.00	0.00	0.00	0.00	0.00	0.00
0.00	0.00	0.00	0.00	0.00	0.00	0.00	0.00	0.00
0.00	0.00	0.00	0.00	0.00	0.00	0.00	0.00	0.00
0.00	0.00	0.00	0.00	0.00	0.00	0.00	0.00	0.00
0.00	0.00	0.00	0.00	0.00	0.00	0.00	0.00	0.00
0.00	0.00	0.00	0.00	0.00	0.00	0.00	0.00	0.00
0.00	0.00	0.00	0.00	0.00	0.00	0.00	0.00	0.00
0.00	0.00	0.00	0.00	0.00	0.00	0.00	0.00	0.00
0.00	0.00	0.00	0.00	0.00	0.00	0.00	0.00	0.00
0.00	0.00	0.00	0.00	0.00	0.00	0.00	0.00	0.00
0.00	0.00	0.00	0.00	0.00	0.00	0.00	0.00	0.00
0.00	0.00	0.00	0.00	0.00	0.00	0.00	9.00	0.00
0.00	0.00	0.00	0.00	0.00	0.00	0.00	0.00	0.00
0.00	0.00	0.00	0.00	0.00	0.00	0.00	0.00	0.00
0.00	0.00	0.00	0.00	0.00	0.00	0.00	0.00	0.00
0.00	0.00	0.00	0.00	0.00	0.00	0.00	0.00	0.00
0.00	0.00	0.00	0.00	0.00	0.00	0.00	0.00	0.00
0.00	0.00	0.00	0.00	0.00	0.00	0.00	0.00	0.00
0.00	0.00	0.00	0.00	0.00	0.00	0.00	0.00	0.00
0.00	0.00	0.00	0.00	0.00	0.00	0.00	0.00	0.00
0.00	0.00	0.00	0.00	0.00	0.00	0.00	0.00	0.00
0.00	0.00	0.00	0.00	0.00	0.00	0.00	0.00	0.00
0.00	0.00	0.00	0.00	0.00	0.00	0.00	0.00	0.00

0.00	0.00	0.00	0.00	0.00	0.00	0.00	0.00	0.00
0.00	0.00	0.00	0.00	0.00	0.00	0.00	0.00	0.00
0.00	0.00	0.00	0.00	0.00	0.00	0.00	0.00	0.00
0.00	0.00	0.00	0.00	0.00	0.00	0.00	0.00	0.00
0.00	0.00	0.00	0.00	0.00	0.00	0.00	0.00	0.00
0.00	0.00	0.00	0.00	0.00	0.00	0.00	0.00	0.00
0.00	0.00	0.00	0.00	0.00	0.00	0.00	0.00	0.00
0.00	0.00	0.00	8.00	0.00	0.00	0.00	0.00	0.00
0.00	0.00	0.00	0.00	0.00	0.00	0.00	0.00	0.00
0.00	0.00	0.00	0.00	0.00	0.00	0.00	0.00	0.00
0.00	0.00	0.00	0.00	0.00	0.00	0.00	0.00	0.00
0.00	0.00	0.00	0.00	0.00	0.00	0.00	0.00	0.00
0.00	0.00	0.00	0.00	0.00	0.00	0.00	0.00	0.00
0.00	0.00	0.00	0.00	0.00	0.00	0.00	0.00	0.00
0.00	0.00	0.00	0.00	0.00	0.00	0.00	0.00	0.00
0.00	0.00	0.00	0.00	0.00	0.00	0.00	0.00	0.00
0.00	0.00	0.00	0.00	0.00	0.00	0.00	0.00	0.00
0.00	0.00	0.00	0.00	0.00	0.00	0.00	0.00	0.00
0.00	0.00	0.00	0.00	0.00	0.00	0.00	0.00	0.00
0.00	0.00	0.00	0.00	0.00	0.00	0.00	0.00	0.00
0.00	0.00	0.00	0.00	0.00	0.00	0.00	0.00	0.00
0.00	0.00	0.00	0.00	0.00	0.00	0.00	0.00	0.00
0.00	0.00	0.00	0.00	0.00	0.00	0.00	0.00	0.00
0.00	0.00	0.00	0.00	0.00	0.00	0.00	0.00	0.00
0.00	0.00	0.00	0.00	0.00	0.00	0.00	0.00	0.00
0.00	0.00	0.00	0.00	0.00	0.00	0.00	0.00	0.00
0.00	0.00	0.00	0.00	0.00	0.00	0.00	0.00	3.00
0.00	0.00	0.00	0.00	0.00	0.00	0.00	0.00	0.00
0.00	0.00	0.00	0.00	0.00	0.00	0.00	0.00	0.00
0.00	0.00	0.00	0.00	0.00	0.00	0.00	0.00	8.00
0.00	0.00	0.00	0.00	0.00	0.00	0.00	0.00	0.00
0.00	0.00	0.00	0.00	0.00	0.00	0.00	0.00	0.00
0.00	0.00	0.00	0.00	0.00	0.00	0.00	0.00	0.00
0.00	0.00	0.00	0.00	0.00	0.00	0.00	0.00	0.00
0.00	0.00	0.00	0.00	0.00	0.00	0.00	0.00	0.00
0.00	0.00	0.00	0.00	0.00	0.00	0.00	0.00	0.00
0.00	0.00	0.00	0.00	0.00	0.00	0.00	0.00	0.00

0.00	0.00	0.00	0.00	0.00	0.00	0.00	0.00	0.00
0.00	0.00	0.00	0.00	0.00	0.00	0.00	0.00	0.00
0.00	0.00	0.00	0.00	0.00	0.00	0.00	0.00	0.00
0.00	0.00	0.00	0.00	0.00	0.00	0.00	0.00	0.00
0.00	0.00	0.00	0.00	0.00	0.00	0.00	0.00	0.00
0.00	0.00	0.00	0.00	0.00	0.00	0.00	0.00	0.00
0.00	0.00	0.00	0.00	0.00	0.00	0.00	0.00	0.00
0.00	0.00	0.00	0.00	0.00	0.00	0.00	0.00	0.00
0.00	0.00	0.00	0.00	0.00	0.00	0.00	0.00	0.00
0.00	0.00	0.00	0.00	0.00	0.00	0.00	0.00	0.00
0.00	0.00	0.00	0.00	0.00	0.00	0.00	0.00	0.00
0.00	0.00	0.00	0.00	0.00	0.00	0.00	0.00	0.00
0.00	0.00	0.00	0.00	0.00	0.00	0.00	0.00	0.00
0.00	0.00	0.00	0.00	0.00	0.00	0.00	0.00	0.00
0.00	0.00	0.00	0.00	0.00	0.00	0.00	0.00	0.00
0.00	0.00	0.00	0.00	0.00	0.00	0.00	0.00	0.00
0.00	0.00	0.00	0.00	0.00	0.00	0.00	0.00	0.00
7.00	0.00	0.00	0.00	0.00	0.00	0.00	0.00	0.00
0.00	0.00	7.00	0.00	0.00	0.00	0.00	0.00	0.00
0.00	0.00	0.00	0.00	0.00	0.00	0.00	0.00	0.00
0.00	0.00	0.00	0.00	0.00	0.00	0.00	0.00	0.00
0.00	0.00	0.00	0.00	0.00	0.00	0.00	0.00	0.00
0.00	0.00	0.00	0.00	0.00	0.00	0.00	0.00	0.00
0.00	0.00	0.00	0.00	0.00	0.00	0.00	0.00	0.00
0.00	0.00	0.00	0.00	0.00	0.00	0.00	0.00	0.00
0.00	0.00	0.00	0.00	0.00	0.00	0.00	0.00	0.00
0.00	0.00	0.00	0.00	0.00	0.00	0.00	0.00	0.00
0.00	0.00	0.00	0.00	0.00	0.00	0.00	0.00	0.00
0.00	0.00	0.00	0.00	0.00	0.00	0.00	0.00	0.00
0.00	0.00	0.00	0.00	0.00	0.00	0.00	0.00	0.00
0.00	0.00	0.00	0.00	0.00	0.00	0.00	0.00	0.00
0.00	0.00	0.00	0.00	0.00	0.00	0.00	0.00	0.00
0.00	0.00	0.00	0.00	0.00	0.00	0.00	0.00	0.00
0.00	0.00	0.00	0.00	0.00	0.00	0.00	0.00	0.00
0.00	0.00	0.00	0.00	0.00	0.00	0.00	0.00	0.00
0.00	0.00	0.00	0.00	0.00	0.00	0.00	0.00	0.00
0.00	0.00	0.00	0.00	0.00	0.00	0.00	0.00	0.00

0.00	0.00	0.00	0.00	0.00	0.00	0.00	0.00	0.00
0.00	0.00	0.00	0.00	0.00	0.00	0.00	0.00	0.00
0.00	0.00	0.00	0.00	0.00	0.00	0.00	0.00	0.00
0.00	0.00	0.00	0.00	0.00	0.00	0.00	0.00	0.00
0.00	0.00	0.00	0.00	0.00	0.00	0.00	0.00	0.00
0.00	0.00	0.00	0.00	0.00	0.00	0.00	0.00	0.00
0.00	0.00	0.00	0.00	0.00	0.00	0.00	0.00	0.00
0.00	0.00	0.00	0.00	0.00	0.00	0.00	0.00	0.00
0.00	0.00	0.00	0.00	0.00	0.00	0.00	0.00	0.00
0.00	0.00	0.00	0.00	0.00	0.00	0.00	0.00	0.00
0.00	0.00	0.00	0.00	0.00	0.00	0.00	0.00	0.00
0.00	0.00	0.00	0.00	0.00	0.00	0.00	0.00	0.00
0.00	0.00	0.00	0.00	0.00	0.00	0.00	0.00	0.00
0.00	0.00	0.00	0.00	0.00	0.00	0.00	0.00	0.00
0.00	0.00	0.00	0.00	0.00	0.00	0.00	0.00	0.00
0.00	0.00	0.00	0.00	0.00	0.00	0.00	0.00	0.00
0.00	0.00	0.00	0.00	0.00	0.00	0.00	0.00	0.00
0.00	0.00	0.00	0.00	0.00	0.00	0.00	0.00	0.00
0.00	0.00	0.00	0.00	0.00	0.00	0.00	0.00	0.00
0.00	0.00	0.00	0.00	0.00	0.00	0.00	0.00	0.00
0.00	0.00	0.00	0.00	0.00	0.00	0.00	0.00	0.00
0.00	0.00	0.00	0.00	0.00	0.00	0.00	0.00	0.00
0.00	0.00	0.00	0.00	0.00	0.00	0.00	0.00	0.00
0.00	0.00	0.00	0.00	0.00	0.00	0.00	0.00	0.00
0.00	0.00	0.00	0.00	0.00	0.00	0.00	0.00	0.00
0.00	0.00	0.00	0.00	0.00	0.00	0.00	0.00	0.00
0.00	0.00	0.00	0.00	0.00	0.00	0.00	0.00	0.00
0.00	0.00	0.00	0.00	0.00	0.00	0.00	0.00	0.00
0.00	0.00	0.00	0.00	0.00	0.00	0.00	0.00	0.00
0.00	0.00	0.00	0.00	0.00	6.00	0.00	0.00	0.00
0.00	0.00	0.00	0.00	0.00	6.00	0.00	0.00	0.00
0.00	0.00	0.00	0.00	0.00	6.00	0.00	0.00	0.00
0.00	0.00	0.00	0.00	0.00	0.00	0.00	0.00	0.00
0.00	0.00	0.00	0.00	0.00	0.00	0.00	0.00	0.00
0.00	0.00	0.00	0.00	0.00	0.00	0.00	0.00	0.00
0.00	0.00	0.00	0.00	0.00	0.00	0.00	0.00	0.00
0.00	0.00	0.00	0.00	0.00	0.00	0.00	0.00	0.00

0.00	0.00	0.00	0.00	0.00	0.00	0.00	0.00	0.00
0.00	0.00	0.00	0.00	0.00	0.00	0.00	0.00	0.00
0.00	0.00	0.00	0.00	0.00	0.00	0.00	0.00	0.00
0.00	0.00	0.00	0.00	0.00	0.00	0.00	0.00	0.00
0.00	0.00	0.00	0.00	0.00	0.00	0.00	0.00	0.00
0.00	0.00	0.00	0.00	0.00	0.00	0.00	0.00	0.00
0.00	0.00	0.00	0.00	0.00	0.00	0.00	0.00	0.00
0.00	0.00	0.00	0.00	0.00	0.00	0.00	0.00	0.00
0.00	0.00	0.00	0.00	0.00	0.00	0.00	0.00	0.00
0.00	0.00	0.00	0.00	0.00	0.00	0.00	0.00	0.00
0.00	0.00	0.00	0.00	0.00	0.00	0.00	0.00	0.00
0.00	0.00	0.00	0.00	0.00	0.00	0.00	0.00	0.00
0.00	0.00	0.00	0.00	0.00	0.00	0.00	0.00	0.00
0.00	0.00	0.00	0.00	0.00	0.00	0.00	0.00	0.00
6.00	0.00	0.00	0.00	0.00	0.00	0.00	0.00	0.00
0.00	0.00	0.00	0.00	0.00	0.00	0.00	0.00	0.00
0.00	0.00	0.00	0.00	0.00	0.00	0.00	0.00	0.00
0.00	0.00	0.00	0.00	0.00	0.00	0.00	0.00	0.00
0.00	0.00	0.00	0.00	0.00	0.00	0.00	0.00	0.00
0.00	0.00	0.00	0.00	0.00	0.00	0.00	0.00	0.00
0.00	0.00	0.00	0.00	0.00	0.00	0.00	0.00	0.00
0.00	0.00	0.00	0.00	0.00	0.00	0.00	0.00	0.00
0.00	0.00	0.00	0.00	0.00	0.00	0.00	0.00	0.00
0.00	0.00	0.00	0.00	0.00	0.00	0.00	0.00	0.00
0.00	0.00	0.00	0.00	0.00	0.00	0.00	0.00	0.00
0.00	0.00	0.00	0.00	0.00	0.00	0.00	0.00	0.00
0.00	0.00	0.00	0.00	0.00	0.00	0.00	0.00	0.00
0.00	0.00	0.00	0.00	0.00	0.00	0.00	0.00	0.00
0.00	0.00	0.00	0.00	0.00	0.00	0.00	0.00	0.00
0.00	0.00	0.00	0.00	0.00	0.00	0.00	0.00	0.00
0.00	0.00	0.00	0.00	0.00	0.00	0.00	0.00	0.00
0.00	0.00	0.00	0.00	0.00	0.00	0.00	0.00	0.00
0.00	0.00	0.00	0.00	0.00	0.00	0.00	0.00	0.00
0.00	0.00	0.00	0.00	0.00	0.00	0.00	0.00	0.00
0.00	0.00	0.00	0.00	0.00	0.00	0.00	0.00	0.00
0.00	0.00	0.00	0.00	0.00	0.00	0.00	0.00	0.00
0.00	0.00	0.00	0.00	0.00	0.00	0.00	0.00	0.00

0.00	0.00	0.00	0.00	0.00	0.00	0.00	0.00	0.00
0.00	0.00	0.00	0.00	0.00	0.00	0.00	0.00	0.00
0.00	0.00	0.00	0.00	0.00	0.00	0.00	0.00	0.00
0.00	0.00	0.00	0.00	0.00	0.00	0.00	0.00	0.00
0.00	0.00	0.00	0.00	0.00	0.00	0.00	0.00	0.00
0.00	0.00	0.00	0.00	0.00	0.00	0.00	0.00	0.00
0.00	0.00	0.00	0.00	0.00	0.00	0.00	0.00	0.00
0.00	0.00	0.00	0.00	0.00	0.00	0.00	0.00	0.00
0.00	0.00	0.00	0.00	0.00	0.00	0.00	0.00	0.00
0.00	0.00	0.00	0.00	0.00	0.00	0.00	0.00	0.00
0.00	0.00	0.00	0.00	0.00	0.00	0.00	0.00	0.00
0.00	0.00	0.00	0.00	0.00	0.00	0.00	0.00	0.00
0.00	0.00	0.00	0.00	0.00	0.00	0.00	0.00	0.00
0.00	0.00	0.00	0.00	0.00	0.00	0.00	0.00	0.00
0.00	0.00	0.00	0.00	0.00	0.00	0.00	0.00	0.00
0.00	0.00	0.00	0.00	0.00	0.00	0.00	0.00	0.00
0.00	0.00	0.00	0.00	0.00	0.00	0.00	0.00	0.00
0.00	0.00	0.00	0.00	0.00	0.00	0.00	0.00	0.00
0.00	0.00	0.00	0.00	0.00	0.00	0.00	0.00	0.00
0.00	0.00	0.00	0.00	0.00	0.00	0.00	0.00	0.00
0.00	0.00	0.00	0.00	0.00	0.00	0.00	0.00	0.00
0.00	0.00	0.00	0.00	0.00	0.00	0.00	0.00	0.00
0.00	0.00	0.00	0.00	0.00	0.00	0.00	0.00	0.00
0.00	0.00	0.00	0.00	0.00	0.00	0.00	0.00	0.00
0.00	0.00	0.00	0.00	0.00	0.00	0.00	0.00	0.00
0.00	0.00	0.00	0.00	0.00	0.00	0.00	0.00	0.00
0.00	0.00	0.00	0.00	0.00	0.00	0.00	0.00	0.00
0.00	0.00	0.00	0.00	0.00	0.00	0.00	0.00	0.00
0.00	0.00	0.00	0.00	0.00	0.00	0.00	0.00	0.00
0.00	0.00	0.00	0.00	0.00	0.00	0.00	0.00	0.00
0.00	0.00	0.00	0.00	0.00	0.00	0.00	0.00	0.00
0.00	0.00	0.00	0.00	0.00	0.00	0.00	0.00	0.00
0.00	0.00	0.00	0.00	0.00	0.00	0.00	0.00	0.00
0.00	0.00	0.00	0.00	0.00	0.00	0.00	0.00	0.00
0.00	0.00	0.00	0.00	0.00	0.00	0.00	0.00	0.00
0.00	0.00	0.00	0.00	0.00	0.00	0.00	0.00	0.00
0.00	0.00	0.00	0.00	0.00	0.00	0.00	0.00	0.00

0.00	0.00	3.00	0.00	0.00	0.00	0.00	0.00	0.00
0.00	0.00	0.00	0.00	0.00	0.00	0.00	0.00	0.00
0.00	0.00	0.00	0.00	0.00	0.00	0.00	0.00	0.00
0.00	0.00	0.00	0.00	0.00	0.00	0.00	0.00	0.00
0.00	0.00	0.00	0.00	0.00	0.00	0.00	0.00	0.00
0.00	0.00	0.00	0.00	0.00	0.00	0.00	0.00	0.00
0.00	0.00	0.00	0.00	0.00	0.00	0.00	0.00	0.00
0.00	0.00	0.00	0.00	0.00	0.00	0.00	0.00	0.00
0.00	0.00	0.00	0.00	0.00	0.00	0.00	0.00	0.00
0.00	0.00	0.00	0.00	0.00	0.00	0.00	0.00	0.00
0.00	0.00	0.00	0.00	0.00	0.00	0.00	0.00	0.00
0.00	0.00	0.00	0.00	0.00	0.00	0.00	0.00	0.00
0.00	0.00	0.00	0.00	0.00	0.00	0.00	0.00	0.00
0.00	0.00	0.00	0.00	0.00	0.00	0.00	0.00	0.00
0.00	0.00	0.00	0.00	0.00	0.00	0.00	0.00	0.00
0.00	0.00	0.00	0.00	0.00	0.00	0.00	0.00	0.00
0.00	0.00	0.00	0.00	0.00	0.00	0.00	0.00	0.00
0.00	0.00	0.00	0.00	0.00	0.00	0.00	0.00	0.00
0.00	0.00	0.00	0.00	0.00	0.00	0.00	0.00	0.00
0.00	0.00	0.00	0.00	0.00	0.00	0.00	0.00	0.00
0.00	0.00	5.00	0.00	0.00	0.00	0.00	0.00	0.00
0.00	0.00	0.00	0.00	0.00	0.00	0.00	0.00	0.00
0.00	0.00	0.00	0.00	0.00	0.00	0.00	0.00	0.00
0.00	0.00	0.00	0.00	0.00	0.00	0.00	0.00	0.00
0.00	0.00	0.00	0.00	0.00	0.00	0.00	0.00	0.00
0.00	0.00	0.00	0.00	0.00	0.00	0.00	0.00	0.00
0.00	0.00	0.00	0.00	0.00	0.00	0.00	0.00	0.00
0.00	0.00	0.00	0.00	0.00	0.00	0.00	0.00	0.00
0.00	0.00	0.00	0.00	0.00	0.00	0.00	0.00	0.00
0.00	0.00	0.00	0.00	0.00	0.00	0.00	0.00	0.00
0.00	0.00	0.00	0.00	0.00	0.00	0.00	0.00	0.00
0.00	0.00	0.00	0.00	0.00	0.00	0.00	0.00	0.00
0.00	0.00	3.00	0.00	0.00	0.00	0.00	0.00	0.00
0.00	0.00	0.00	0.00	0.00	0.00	0.00	0.00	0.00
0.00	0.00	0.00	0.00	0.00	0.00	0.00	0.00	0.00
0.00	0.00	0.00	0.00	0.00	0.00	0.00	0.00	0.00
0.00	0.00	0.00	0.00	0.00	0.00	0.00	0.00	0.00

0.00	0.00	0.00	0.00	0.00	0.00	0.00	0.00	0.00
0.00	0.00	0.00	0.00	0.00	0.00	0.00	0.00	0.00
0.00	0.00	0.00	0.00	0.00	0.00	0.00	0.00	0.00
0.00	0.00	0.00	0.00	0.00	0.00	0.00	0.00	0.00
0.00	0.00	0.00	0.00	0.00	0.00	0.00	0.00	0.00
0.00	0.00	0.00	0.00	0.00	0.00	0.00	0.00	0.00
0.00	0.00	0.00	0.00	0.00	0.00	0.00	0.00	0.00
0.00	0.00	0.00	0.00	0.00	0.00	0.00	0.00	0.00
0.00	0.00	0.00	0.00	0.00	0.00	0.00	0.00	0.00
0.00	0.00	0.00	0.00	0.00	0.00	0.00	0.00	0.00
0.00	0.00	0.00	0.00	0.00	0.00	0.00	0.00	0.00
0.00	0.00	0.00	0.00	0.00	0.00	0.00	0.00	0.00
0.00	0.00	0.00	0.00	0.00	0.00	0.00	0.00	0.00
0.00	0.00	0.00	0.00	0.00	0.00	0.00	0.00	0.00
0.00	0.00	0.00	0.00	0.00	0.00	0.00	0.00	0.00
0.00	0.00	0.00	0.00	0.00	0.00	0.00	0.00	0.00
0.00	0.00	0.00	0.00	0.00	0.00	0.00	0.00	0.00
0.00	0.00	0.00	0.00	0.00	0.00	0.00	0.00	0.00
0.00	0.00	0.00	0.00	0.00	0.00	0.00	0.00	0.00
0.00	0.00	0.00	0.00	0.00	0.00	0.00	0.00	0.00
0.00	0.00	0.00	0.00	0.00	0.00	0.00	0.00	0.00
0.00	0.00	0.00	0.00	0.00	0.00	0.00	0.00	0.00
0.00	0.00	0.00	0.00	0.00	0.00	0.00	0.00	0.00
0.00	0.00	0.00	0.00	0.00	0.00	0.00	0.00	0.00
0.00	0.00	0.00	0.00	0.00	0.00	0.00	0.00	0.00
0.00	0.00	0.00	0.00	0.00	0.00	0.00	0.00	0.00
0.00	0.00	0.00	0.00	0.00	0.00	0.00	0.00	0.00
0.00	0.00	0.00	0.00	0.00	0.00	0.00	0.00	0.00
0.00	0.00	0.00	0.00	0.00	0.00	0.00	0.00	0.00
0.00	0.00	0.00	0.00	0.00	0.00	0.00	0.00	0.00
0.00	0.00	0.00	0.00	0.00	0.00	0.00	0.00	0.00
0.00	0.00	0.00	0.00	0.00	0.00	0.00	0.00	0.00
0.00	0.00	0.00	0.00	0.00	0.00	0.00	0.00	0.00
0.00	0.00	0.00	0.00	0.00	0.00	0.00	0.00	0.00
0.00	0.00	0.00	0.00	0.00	0.00	0.00	0.00	0.00
0.00	0.00	0.00	0.00	0.00	0.00	0.00	0.00	0.00
0.00	0.00	0.00	0.00	0.00	0.00	0.00	0.00	0.00

0.00	0.00	0.00	0.00	0.00	0.00	0.00	0.00	0.00
0.00	0.00	0.00	0.00	0.00	0.00	0.00	0.00	0.00
0.00	0.00	0.00	0.00	0.00	0.00	0.00	5.00	0.00
0.00	0.00	0.00	0.00	0.00	0.00	0.00	5.00	0.00
0.00	0.00	0.00	0.00	0.00	0.00	0.00	5.00	0.00
0.00	0.00	0.00	0.00	0.00	0.00	0.00	0.00	0.00
0.00	0.00	0.00	0.00	0.00	0.00	0.00	0.00	0.00
0.00	0.00	0.00	0.00	0.00	0.00	0.00	0.00	0.00
0.00	0.00	0.00	0.00	0.00	0.00	0.00	0.00	0.00
0.00	0.00	0.00	0.00	0.00	0.00	0.00	0.00	0.00
0.00	0.00	0.00	0.00	0.00	0.00	0.00	0.00	0.00
0.00	0.00	0.00	0.00	0.00	0.00	0.00	0.00	0.00
0.00	0.00	0.00	0.00	0.00	0.00	0.00	0.00	0.00
0.00	0.00	0.00	0.00	0.00	0.00	0.00	0.00	0.00
0.00	0.00	0.00	0.00	0.00	0.00	0.00	0.00	0.00
0.00	0.00	0.00	0.00	0.00	0.00	0.00	0.00	0.00
0.00	0.00	0.00	0.00	0.00	0.00	0.00	0.00	0.00
0.00	0.00	0.00	0.00	0.00	0.00	0.00	0.00	0.00
0.00	0.00	0.00	0.00	0.00	0.00	0.00	0.00	0.00
0.00	0.00	0.00	0.00	0.00	0.00	0.00	0.00	0.00
0.00	0.00	0.00	0.00	0.00	0.00	0.00	0.00	0.00
0.00	0.00	0.00	0.00	0.00	0.00	0.00	0.00	0.00
0.00	0.00	0.00	0.00	0.00	0.00	0.00	0.00	0.00
0.00	0.00	0.00	0.00	0.00	4.00	0.00	0.00	0.00
0.00	0.00	0.00	0.00	0.00	0.00	0.00	0.00	0.00
0.00	0.00	0.00	0.00	0.00	0.00	0.00	0.00	0.00
0.00	0.00	0.00	0.00	0.00	0.00	0.00	0.00	0.00
0.00	0.00	0.00	0.00	0.00	0.00	0.00	0.00	0.00
0.00	0.00	0.00	0.00	0.00	0.00	0.00	0.00	0.00
0.00	0.00	0.00	0.00	0.00	0.00	0.00	0.00	0.00
0.00	0.00	0.00	0.00	0.00	0.00	0.00	0.00	0.00
0.00	0.00	0.00	0.00	0.00	0.00	0.00	0.00	0.00
0.00	0.00	0.00	0.00	0.00	0.00	0.00	0.00	0.00
0.00	0.00	0.00	0.00	0.00	0.00	0.00	0.00	0.00
0.00	0.00	0.00	0.00	0.00	0.00	0.00	0.00	0.00
0.00	0.00	0.00	0.00	0.00	0.00	0.00	0.00	0.00
0.00	0.00	0.00	0.00	0.00	0.00	0.00	0.00	0.00

0.00	0.00	0.00	0.00	0.00	0.00	0.00	0.00	0.00
0.00	4.00	0.00	0.00	0.00	0.00	0.00	0.00	0.00
0.00	0.00	0.00	4.00	0.00	0.00	0.00	0.00	0.00
0.00	0.00	0.00	4.00	0.00	0.00	0.00	0.00	0.00
0.00	0.00	0.00	0.00	0.00	0.00	0.00	0.00	0.00
0.00	0.00	0.00	0.00	0.00	0.00	0.00	0.00	0.00
0.00	0.00	0.00	0.00	0.00	0.00	0.00	0.00	0.00
0.00	0.00	0.00	0.00	0.00	0.00	0.00	0.00	0.00
0.00	0.00	0.00	0.00	0.00	0.00	0.00	0.00	0.00
0.00	0.00	0.00	0.00	0.00	0.00	0.00	0.00	0.00
0.00	0.00	0.00	0.00	0.00	0.00	0.00	0.00	0.00
0.00	0.00	0.00	0.00	0.00	0.00	0.00	0.00	0.00
0.00	0.00	0.00	0.00	0.00	0.00	0.00	0.00	0.00
0.00	0.00	0.00	0.00	0.00	0.00	0.00	0.00	0.00
0.00	0.00	0.00	0.00	0.00	0.00	0.00	0.00	0.00
0.00	0.00	0.00	0.00	0.00	0.00	0.00	0.00	0.00
0.00	0.00	0.00	0.00	0.00	0.00	0.00	0.00	0.00
0.00	0.00	0.00	0.00	0.00	0.00	0.00	0.00	0.00
0.00	0.00	0.00	0.00	0.00	0.00	0.00	0.00	0.00
0.00	0.00	0.00	0.00	0.00	0.00	0.00	0.00	0.00
0.00	0.00	0.00	0.00	0.00	0.00	0.00	0.00	0.00
0.00	0.00	0.00	0.00	0.00	0.00	0.00	0.00	0.00
0.00	0.00	0.00	0.00	0.00	0.00	0.00	0.00	0.00
0.00	0.00	0.00	0.00	0.00	0.00	0.00	0.00	0.00
0.00	0.00	0.00	0.00	0.00	0.00	0.00	0.00	0.00
0.00	0.00	0.00	0.00	0.00	0.00	0.00	0.00	0.00
0.00	0.00	0.00	0.00	0.00	0.00	0.00	0.00	0.00
0.00	0.00	0.00	0.00	0.00	0.00	0.00	0.00	0.00
0.00	0.00	0.00	0.00	0.00	0.00	0.00	0.00	0.00
0.00	0.00	0.00	0.00	0.00	0.00	0.00	0.00	0.00
0.00	0.00	0.00	0.00	0.00	0.00	0.00	0.00	0.00
0.00	0.00	0.00	0.00	0.00	0.00	0.00	0.00	0.00
0.00	0.00	0.00	0.00	0.00	0.00	0.00	0.00	0.00
0.00	0.00	0.00	0.00	0.00	0.00	0.00	0.00	0.00
0.00	0.00	0.00	0.00	0.00	0.00	0.00	0.00	0.00
0.00	0.00	0.00	0.00	0.00	0.00	0.00	0.00	0.00
0.00	0.00	0.00	0.00	0.00	0.00	0.00	0.00	0.00

0.00	0.00	0.00	0.00	0.00	0.00	0.00	0.00	0.00
0.00	0.00	0.00	0.00	0.00	0.00	0.00	0.00	0.00
0.00	0.00	0.00	0.00	0.00	0.00	0.00	0.00	0.00
0.00	0.00	0.00	0.00	0.00	0.00	0.00	0.00	0.00
0.00	0.00	0.00	0.00	0.00	0.00	0.00	0.00	0.00
0.00	0.00	0.00	0.00	0.00	0.00	0.00	0.00	0.00
0.00	0.00	0.00	0.00	0.00	0.00	0.00	0.00	4.00
0.00	0.00	0.00	0.00	0.00	0.00	0.00	0.00	0.00
0.00	0.00	0.00	0.00	0.00	0.00	0.00	0.00	0.00
0.00	0.00	0.00	0.00	0.00	0.00	0.00	0.00	0.00
0.00	0.00	0.00	0.00	0.00	0.00	0.00	0.00	0.00
0.00	0.00	0.00	0.00	0.00	0.00	0.00	0.00	0.00
0.00	0.00	0.00	0.00	0.00	0.00	0.00	0.00	0.00
0.00	0.00	0.00	0.00	0.00	0.00	0.00	0.00	0.00
0.00	0.00	0.00	0.00	0.00	0.00	0.00	0.00	0.00
0.00	0.00	0.00	0.00	0.00	0.00	0.00	0.00	0.00
0.00	0.00	0.00	0.00	0.00	0.00	0.00	0.00	0.00
0.00	0.00	0.00	0.00	0.00	0.00	0.00	0.00	0.00
0.00	0.00	0.00	0.00	0.00	0.00	0.00	0.00	0.00
0.00	0.00	0.00	0.00	0.00	0.00	0.00	0.00	0.00
0.00	0.00	0.00	0.00	0.00	0.00	0.00	0.00	0.00
0.00	0.00	0.00	0.00	0.00	0.00	0.00	0.00	0.00
0.00	0.00	0.00	0.00	0.00	0.00	0.00	0.00	0.00
0.00	0.00	0.00	0.00	0.00	0.00	0.00	0.00	0.00
0.00	0.00	0.00	0.00	0.00	0.00	0.00	0.00	0.00
0.00	0.00	0.00	0.00	0.00	0.00	0.00	0.00	0.00
0.00	0.00	0.00	0.00	0.00	0.00	0.00	0.00	0.00
0.00	0.00	0.00	0.00	0.00	0.00	0.00	0.00	0.00
0.00	0.00	0.00	0.00	0.00	0.00	0.00	0.00	0.00
0.00	0.00	0.00	0.00	0.00	0.00	0.00	0.00	0.00
0.00	0.00	0.00	0.00	0.00	0.00	0.00	0.00	0.00
0.00	0.00	0.00	0.00	0.00	0.00	0.00	0.00	0.00
0.00	0.00	0.00	0.00	0.00	0.00	0.00	0.00	0.00
0.00	0.00	0.00	0.00	0.00	0.00	0.00	0.00	0.00
0.00	0.00	0.00	0.00	0.00	0.00	0.00	0.00	0.00
0.00	0.00	0.00	0.00	0.00	0.00	0.00	0.00	0.00
0.00	0.00	0.00	0.00	0.00	0.00	0.00	0.00	0.00

0.00	0.00	0.00	0.00	0.00	0.00	0.00	0.00	0.00
0.00	0.00	0.00	0.00	0.00	0.00	0.00	0.00	0.00
0.00	0.00	0.00	0.00	0.00	0.00	0.00	0.00	0.00
0.00	0.00	0.00	0.00	0.00	0.00	0.00	0.00	0.00
0.00	0.00	0.00	0.00	0.00	0.00	0.00	0.00	0.00
0.00	0.00	0.00	0.00	0.00	0.00	0.00	0.00	0.00
0.00	0.00	0.00	0.00	0.00	0.00	0.00	0.00	0.00
0.00	0.00	0.00	0.00	0.00	0.00	0.00	0.00	0.00
0.00	0.00	0.00	0.00	0.00	0.00	0.00	0.00	0.00
0.00	0.00	0.00	0.00	0.00	0.00	0.00	0.00	0.00
0.00	0.00	0.00	0.00	0.00	0.00	0.00	4.00	0.00
0.00	0.00	0.00	0.00	0.00	0.00	0.00	0.00	0.00
0.00	0.00	0.00	0.00	0.00	0.00	0.00	0.00	0.00
0.00	0.00	0.00	0.00	0.00	0.00	0.00	0.00	0.00
0.00	0.00	0.00	0.00	0.00	0.00	0.00	0.00	0.00
0.00	0.00	0.00	0.00	0.00	0.00	0.00	0.00	0.00
0.00	0.00	0.00	0.00	0.00	0.00	0.00	0.00	0.00
0.00	0.00	0.00	0.00	0.00	0.00	0.00	0.00	0.00
0.00	0.00	0.00	0.00	0.00	0.00	0.00	0.00	0.00
0.00	0.00	0.00	0.00	0.00	0.00	0.00	0.00	0.00
0.00	0.00	0.00	0.00	0.00	0.00	0.00	0.00	0.00
0.00	0.00	0.00	0.00	0.00	0.00	0.00	0.00	0.00
0.00	0.00	0.00	0.00	0.00	0.00	0.00	0.00	0.00
0.00	0.00	0.00	0.00	0.00	0.00	0.00	0.00	0.00
0.00	0.00	0.00	0.00	0.00	0.00	0.00	0.00	0.00
0.00	0.00	0.00	0.00	0.00	0.00	0.00	0.00	0.00
0.00	0.00	0.00	0.00	0.00	0.00	0.00	0.00	0.00
0.00	0.00	0.00	0.00	0.00	0.00	0.00	0.00	0.00
0.00	0.00	0.00	0.00	0.00	0.00	0.00	0.00	0.00
0.00	0.00	0.00	0.00	0.00	0.00	0.00	0.00	0.00
0.00	0.00	0.00	0.00	0.00	0.00	0.00	0.00	0.00
0.00	0.00	0.00	0.00	0.00	0.00	0.00	0.00	0.00
0.00	0.00	0.00	0.00	0.00	0.00	0.00	0.00	0.00
0.00	0.00	0.00	0.00	0.00	0.00	0.00	0.00	0.00
0.00	0.00	0.00	0.00	0.00	3.00	0.00	0.00	0.00
0.00	0.00	0.00	0.00	0.00	0.00	0.00	0.00	0.00

0.00	0.00	0.00	0.00	0.00	0.00	0.00	0.00	0.00
0.00	0.00	0.00	0.00	0.00	0.00	0.00	0.00	0.00
0.00	0.00	0.00	0.00	0.00	0.00	0.00	0.00	0.00
0.00	0.00	0.00	0.00	0.00	0.00	0.00	0.00	0.00
0.00	0.00	0.00	0.00	0.00	0.00	0.00	0.00	0.00
0.00	0.00	0.00	0.00	0.00	0.00	0.00	0.00	0.00
0.00	0.00	0.00	0.00	0.00	0.00	0.00	0.00	0.00
0.00	0.00	0.00	0.00	0.00	0.00	0.00	0.00	0.00
0.00	0.00	0.00	0.00	0.00	0.00	0.00	0.00	0.00
0.00	0.00	0.00	0.00	0.00	0.00	0.00	0.00	0.00
0.00	0.00	0.00	0.00	0.00	0.00	0.00	0.00	0.00
0.00	0.00	0.00	0.00	0.00	0.00	0.00	0.00	0.00
0.00	0.00	0.00	0.00	0.00	0.00	0.00	0.00	0.00
0.00	0.00	0.00	0.00	0.00	0.00	0.00	0.00	0.00
0.00	0.00	0.00	0.00	0.00	0.00	0.00	0.00	0.00
0.00	0.00	0.00	0.00	0.00	0.00	0.00	0.00	0.00
0.00	0.00	0.00	0.00	0.00	0.00	0.00	0.00	0.00
0.00	0.00	0.00	0.00	0.00	0.00	0.00	0.00	0.00
0.00	0.00	0.00	0.00	0.00	0.00	0.00	0.00	0.00
0.00	0.00	0.00	0.00	0.00	0.00	0.00	0.00	0.00
0.00	0.00	0.00	0.00	0.00	0.00	0.00	0.00	0.00
0.00	0.00	0.00	0.00	0.00	0.00	0.00	0.00	0.00
0.00	3.00	0.00	0.00	0.00	0.00	0.00	0.00	0.00
0.00	0.00	3.00	0.00	0.00	0.00	0.00	0.00	0.00
0.00	0.00	0.00	0.00	0.00	0.00	0.00	0.00	0.00
0.00	0.00	0.00	0.00	0.00	0.00	0.00	0.00	0.00
0.00	0.00	0.00	0.00	0.00	0.00	0.00	0.00	0.00
0.00	0.00	0.00	0.00	0.00	0.00	0.00	0.00	0.00
0.00	0.00	0.00	0.00	0.00	0.00	0.00	0.00	0.00
0.00	0.00	0.00	0.00	0.00	0.00	0.00	0.00	0.00
0.00	0.00	0.00	0.00	0.00	0.00	0.00	0.00	0.00
0.00	0.00	0.00	0.00	0.00	0.00	0.00	0.00	0.00
0.00	0.00	0.00	0.00	0.00	0.00	0.00	0.00	0.00
0.00	0.00	0.00	0.00	0.00	0.00	0.00	0.00	0.00
0.00	0.00	0.00	0.00	0.00	0.00	0.00	0.00	0.00
0.00	0.00	0.00	0.00	0.00	0.00	0.00	0.00	0.00
0.00	0.00	0.00	0.00	0.00	0.00	0.00	0.00	0.00

0.00	0.00	0.00	0.00	0.00	0.00	0.00	0.00	0.00
0.00	0.00	0.00	0.00	0.00	0.00	0.00	0.00	0.00
0.00	0.00	0.00	0.00	0.00	0.00	0.00	0.00	0.00
0.00	0.00	0.00	0.00	0.00	0.00	0.00	0.00	0.00
0.00	0.00	0.00	0.00	0.00	0.00	0.00	0.00	0.00
0.00	0.00	0.00	0.00	0.00	0.00	0.00	0.00	0.00
0.00	0.00	0.00	0.00	0.00	0.00	0.00	0.00	0.00
0.00	0.00	0.00	0.00	0.00	0.00	0.00	0.00	0.00
0.00	0.00	0.00	0.00	0.00	0.00	0.00	0.00	0.00
0.00	0.00	0.00	0.00	0.00	0.00	0.00	0.00	0.00
0.00	0.00	0.00	0.00	0.00	0.00	0.00	0.00	0.00
0.00	0.00	0.00	0.00	0.00	0.00	0.00	0.00	0.00
0.00	0.00	0.00	0.00	0.00	0.00	0.00	0.00	0.00
0.00	0.00	0.00	0.00	0.00	0.00	0.00	0.00	0.00
0.00	0.00	0.00	0.00	0.00	0.00	0.00	0.00	0.00
0.00	0.00	0.00	0.00	0.00	0.00	0.00	0.00	0.00
0.00	0.00	0.00	0.00	0.00	0.00	0.00	0.00	0.00
0.00	0.00	0.00	0.00	0.00	0.00	0.00	0.00	0.00
0.00	0.00	0.00	0.00	0.00	0.00	0.00	0.00	0.00
0.00	0.00	0.00	0.00	0.00	0.00	0.00	0.00	0.00
0.00	0.00	0.00	0.00	0.00	0.00	0.00	0.00	0.00
0.00	0.00	0.00	0.00	0.00	0.00	0.00	0.00	0.00
0.00	0.00	0.00	0.00	0.00	0.00	0.00	0.00	0.00
0.00	0.00	0.00	0.00	0.00	0.00	0.00	0.00	0.00
0.00	0.00	0.00	0.00	0.00	0.00	0.00	0.00	0.00
0.00	0.00	0.00	0.00	0.00	0.00	0.00	0.00	0.00
0.00	0.00	0.00	0.00	0.00	0.00	0.00	0.00	0.00
0.00	0.00	0.00	0.00	0.00	0.00	0.00	0.00	0.00
0.00	0.00	0.00	0.00	0.00	0.00	0.00	0.00	0.00
0.00	0.00	0.00	0.00	0.00	0.00	0.00	0.00	0.00
0.00	0.00	0.00	0.00	0.00	0.00	0.00	0.00	0.00
0.00	0.00	0.00	0.00	0.00	0.00	0.00	0.00	0.00
0.00	0.00	0.00	0.00	0.00	0.00	0.00	0.00	0.00
0.00	0.00	0.00	0.00	0.00	0.00	0.00	0.00	0.00
0.00	0.00	0.00	0.00	0.00	0.00	0.00	0.00	0.00
0.00	0.00	0.00	0.00	0.00	0.00	0.00	0.00	0.00
0.00	0.00	0.00	0.00	0.00	0.00	0.00	0.00	3.00

0.00	0.00	0.00	0.00	0.00	0.00	0.00	0.00	0.00
0.00	0.00	0.00	0.00	0.00	0.00	0.00	0.00	0.00
0.00	0.00	0.00	0.00	0.00	0.00	0.00	0.00	0.00
0.00	0.00	0.00	0.00	0.00	0.00	0.00	0.00	0.00
0.00	0.00	0.00	0.00	0.00	0.00	0.00	0.00	0.00
0.00	0.00	0.00	0.00	0.00	0.00	0.00	0.00	0.00
0.00	0.00	0.00	0.00	0.00	0.00	0.00	0.00	0.00
0.00	0.00	0.00	0.00	0.00	0.00	0.00	0.00	0.00
0.00	0.00	0.00	0.00	0.00	0.00	0.00	0.00	0.00
0.00	0.00	0.00	0.00	0.00	0.00	0.00	0.00	0.00
0.00	0.00	0.00	0.00	0.00	0.00	0.00	0.00	0.00
0.00	0.00	0.00	0.00	0.00	0.00	0.00	0.00	0.00
0.00	0.00	0.00	0.00	0.00	0.00	0.00	0.00	0.00
0.00	0.00	0.00	0.00	0.00	0.00	0.00	0.00	0.00
0.00	0.00	0.00	0.00	0.00	0.00	0.00	0.00	0.00
0.00	0.00	0.00	0.00	0.00	0.00	0.00	0.00	0.00
0.00	0.00	0.00	0.00	0.00	0.00	0.00	0.00	0.00
0.00	0.00	0.00	0.00	0.00	0.00	0.00	0.00	0.00
0.00	0.00	0.00	0.00	0.00	0.00	0.00	0.00	0.00
0.00	0.00	0.00	0.00	0.00	0.00	0.00	0.00	0.00
0.00	0.00	0.00	0.00	0.00	0.00	0.00	0.00	0.00
0.00	0.00	0.00	0.00	0.00	0.00	0.00	0.00	0.00
0.00	0.00	0.00	0.00	0.00	0.00	0.00	0.00	0.00
0.00	0.00	0.00	0.00	0.00	0.00	0.00	0.00	0.00
0.00	0.00	0.00	0.00	0.00	0.00	0.00	0.00	0.00
0.00	0.00	0.00	0.00	0.00	0.00	0.00	0.00	0.00
0.00	0.00	0.00	0.00	0.00	0.00	0.00	0.00	0.00
0.00	0.00	0.00	0.00	0.00	0.00	0.00	0.00	0.00
0.00	0.00	0.00	0.00	0.00	0.00	0.00	0.00	0.00
0.00	0.00	0.00	0.00	0.00	0.00	0.00	0.00	0.00
0.00	0.00	0.00	0.00	0.00	0.00	0.00	0.00	0.00
0.00	0.00	0.00	0.00	0.00	0.00	0.00	0.00	0.00
0.00	0.00	0.00	0.00	0.00	0.00	0.00	0.00	0.00
0.00	0.00	0.00	0.00	0.00	0.00	0.00	0.00	0.00
0.00	0.00	0.00	0.00	0.00	0.00	0.00	0.00	0.00
0.00	0.00	0.00	0.00	0.00	0.00	0.00	0.00	0.00
0.00	0.00	0.00	0.00	0.00	0.00	0.00	0.00	0.00

0.00	0.00	0.00	0.00	0.00	0.00	0.00	0.00	0.00
0.00	0.00	0.00	0.00	0.00	0.00	0.00	0.00	0.00
0.00	0.00	0.00	0.00	0.00	0.00	0.00	0.00	0.00
0.00	0.00	0.00	0.00	0.00	0.00	0.00	0.00	0.00
0.00	0.00	0.00	0.00	0.00	0.00	0.00	0.00	0.00
0.00	0.00	0.00	0.00	0.00	0.00	0.00	3.00	0.00
0.00	0.00	0.00	0.00	0.00	0.00	0.00	0.00	0.00
0.00	0.00	0.00	0.00	0.00	0.00	0.00	0.00	0.00
0.00	0.00	0.00	0.00	0.00	0.00	0.00	0.00	0.00
0.00	0.00	0.00	0.00	0.00	0.00	0.00	0.00	0.00
0.00	0.00	0.00	0.00	0.00	0.00	0.00	0.00	0.00
0.00	0.00	0.00	0.00	0.00	0.00	0.00	0.00	0.00
0.00	0.00	0.00	0.00	0.00	0.00	0.00	0.00	0.00
0.00	0.00	0.00	0.00	0.00	0.00	0.00	0.00	0.00
0.00	0.00	0.00	0.00	0.00	0.00	0.00	0.00	0.00
0.00	0.00	0.00	0.00	0.00	0.00	0.00	0.00	0.00
0.00	0.00	0.00	0.00	0.00	0.00	0.00	0.00	0.00
0.00	0.00	0.00	0.00	0.00	0.00	0.00	0.00	0.00
0.00	0.00	0.00	0.00	0.00	0.00	0.00	0.00	0.00
0.00	0.00	0.00	0.00	0.00	0.00	0.00	0.00	0.00
0.00	0.00	0.00	0.00	0.00	0.00	0.00	0.00	0.00
0.00	0.00	0.00	0.00	0.00	0.00	0.00	0.00	0.00
0.00	0.00	0.00	0.00	0.00	0.00	0.00	0.00	0.00
0.00	0.00	0.00	0.00	0.00	0.00	0.00	0.00	0.00
0.00	0.00	0.00	0.00	2.00	0.00	0.00	0.00	0.00
0.00	0.00	0.00	0.00	0.00	0.00	0.00	0.00	0.00
0.00	0.00	0.00	0.00	0.00	0.00	0.00	0.00	0.00
0.00	0.00	0.00	0.00	0.00	0.00	0.00	0.00	0.00
0.00	0.00	0.00	0.00	0.00	0.00	0.00	0.00	0.00
0.00	0.00	0.00	0.00	0.00	0.00	0.00	0.00	0.00
0.00	0.00	0.00	0.00	0.00	0.00	0.00	0.00	0.00
0.00	0.00	0.00	0.00	0.00	0.00	0.00	0.00	0.00
0.00	0.00	0.00	0.00	0.00	0.00	0.00	0.00	0.00
0.00	0.00	0.00	0.00	0.00	0.00	0.00	0.00	0.00
0.00	0.00	0.00	0.00	0.00	0.00	0.00	0.00	0.00
0.00	0.00	0.00	0.00	0.00	0.00	0.00	0.00	0.00
0.00	0.00	0.00	0.00	0.00	0.00	0.00	0.00	0.00

0.00	0.00	0.00	0.00	0.00	0.00	0.00	0.00	0.00
0.00	0.00	0.00	0.00	0.00	0.00	0.00	0.00	0.00
0.00	0.00	0.00	0.00	0.00	0.00	0.00	0.00	0.00
0.00	0.00	0.00	0.00	0.00	0.00	0.00	0.00	0.00
0.00	0.00	0.00	0.00	0.00	0.00	0.00	0.00	0.00
0.00	0.00	0.00	0.00	0.00	0.00	0.00	0.00	0.00
0.00	0.00	0.00	0.00	0.00	0.00	0.00	0.00	0.00
0.00	0.00	0.00	0.00	0.00	0.00	0.00	0.00	0.00
0.00	0.00	0.00	0.00	0.00	0.00	0.00	0.00	0.00
0.00	0.00	0.00	0.00	0.00	0.00	0.00	0.00	0.00
0.00	0.00	0.00	0.00	0.00	0.00	0.00	0.00	0.00
0.00	0.00	0.00	0.00	0.00	0.00	0.00	0.00	0.00
0.00	0.00	0.00	0.00	0.00	0.00	0.00	0.00	0.00
0.00	0.00	0.00	0.00	0.00	0.00	0.00	0.00	0.00
0.00	0.00	0.00	0.00	0.00	0.00	0.00	0.00	0.00
0.00	0.00	0.00	0.00	0.00	0.00	0.00	0.00	0.00
0.00	0.00	0.00	0.00	0.00	0.00	0.00	0.00	0.00
0.00	0.00	0.00	0.00	0.00	0.00	0.00	0.00	0.00
0.00	0.00	0.00	0.00	0.00	0.00	0.00	0.00	0.00
0.00	0.00	2.00	0.00	0.00	0.00	0.00	0.00	0.00
0.00	0.00	2.00	0.00	0.00	0.00	0.00	0.00	0.00
0.00	0.00	0.00	0.00	0.00	0.00	0.00	0.00	0.00
0.00	0.00	0.00	0.00	0.00	0.00	0.00	0.00	0.00
0.00	0.00	0.00	0.00	0.00	0.00	0.00	0.00	0.00
0.00	0.00	0.00	0.00	0.00	0.00	0.00	0.00	0.00
0.00	0.00	0.00	0.00	0.00	0.00	0.00	0.00	0.00
0.00	0.00	0.00	0.00	0.00	0.00	0.00	0.00	0.00
0.00	0.00	0.00	0.00	0.00	0.00	0.00	0.00	0.00
0.00	0.00	0.00	0.00	0.00	0.00	0.00	0.00	0.00
0.00	0.00	0.00	0.00	0.00	0.00	0.00	0.00	0.00
0.00	0.00	0.00	0.00	0.00	0.00	0.00	0.00	0.00
0.00	0.00	0.00	0.00	0.00	0.00	0.00	0.00	0.00
0.00	0.00	0.00	0.00	0.00	0.00	0.00	0.00	0.00
0.00	0.00	0.00	0.00	0.00	0.00	0.00	0.00	0.00
0.00	0.00	0.00	0.00	0.00	0.00	0.00	0.00	0.00
0.00	0.00	0.00	0.00	0.00	0.00	0.00	0.00	0.00
0.00	0.00	0.00	0.00	0.00	0.00	0.00	0.00	0.00

0.00	0.00	0.00	0.00	0.00	0.00	0.00	0.00	0.00
0.00	0.00	0.00	0.00	0.00	0.00	0.00	0.00	0.00
0.00	0.00	0.00	0.00	0.00	0.00	0.00	0.00	0.00
0.00	0.00	0.00	0.00	0.00	0.00	0.00	0.00	0.00
0.00	0.00	0.00	0.00	0.00	0.00	0.00	0.00	0.00
0.00	0.00	0.00	0.00	0.00	0.00	0.00	0.00	0.00
0.00	0.00	0.00	0.00	0.00	0.00	0.00	0.00	0.00
0.00	0.00	0.00	0.00	0.00	0.00	0.00	0.00	0.00
0.00	0.00	0.00	0.00	0.00	0.00	0.00	0.00	0.00
0.00	0.00	0.00	0.00	0.00	0.00	0.00	0.00	0.00
0.00	0.00	0.00	0.00	0.00	0.00	0.00	0.00	0.00
0.00	0.00	0.00	0.00	0.00	0.00	0.00	0.00	0.00
0.00	0.00	0.00	0.00	0.00	0.00	0.00	0.00	0.00
0.00	0.00	0.00	0.00	0.00	0.00	0.00	0.00	0.00
0.00	0.00	0.00	0.00	0.00	0.00	0.00	0.00	0.00
0.00	0.00	0.00	0.00	0.00	0.00	0.00	0.00	0.00
0.00	0.00	0.00	0.00	0.00	0.00	0.00	0.00	0.00
0.00	0.00	0.00	0.00	0.00	0.00	0.00	0.00	0.00
0.00	0.00	0.00	0.00	0.00	0.00	0.00	0.00	0.00
0.00	0.00	0.00	0.00	0.00	0.00	0.00	0.00	0.00
0.00	0.00	0.00	0.00	0.00	0.00	0.00	0.00	0.00
0.00	0.00	0.00	0.00	0.00	0.00	0.00	0.00	0.00
0.00	0.00	0.00	0.00	0.00	0.00	0.00	0.00	0.00
0.00	0.00	0.00	0.00	0.00	0.00	0.00	0.00	0.00
0.00	0.00	0.00	0.00	0.00	0.00	0.00	0.00	0.00
0.00	0.00	0.00	0.00	0.00	0.00	0.00	0.00	0.00
0.00	0.00	0.00	0.00	0.00	0.00	0.00	0.00	0.00
0.00	0.00	0.00	0.00	0.00	0.00	0.00	0.00	0.00
0.00	0.00	0.00	0.00	0.00	0.00	0.00	0.00	0.00
0.00	0.00	0.00	0.00	0.00	0.00	0.00	0.00	0.00
0.00	0.00	0.00	0.00	0.00	0.00	0.00	0.00	0.00
0.00	0.00	0.00	0.00	0.00	0.00	0.00	0.00	0.00
0.00	0.00	0.00	0.00	0.00	0.00	0.00	0.00	0.00
0.00	0.00	0.00	0.00	0.00	0.00	0.00	0.00	0.00
0.00	0.00	0.00	0.00	0.00	0.00	0.00	0.00	0.00
0.00	0.00	0.00	0.00	0.00	0.00	0.00	0.00	0.00
0.00	0.00	0.00	0.00	0.00	0.00	0.00	0.00	0.00

0.00	0.00	0.00	0.00	0.00	0.00	0.00	0.00	0.00
0.00	0.00	0.00	0.00	0.00	0.00	0.00	0.00	0.00
0.00	0.00	0.00	0.00	0.00	0.00	0.00	0.00	0.00
0.00	0.00	0.00	0.00	0.00	0.00	0.00	0.00	0.00
0.00	0.00	0.00	0.00	0.00	0.00	0.00	0.00	0.00
0.00	0.00	0.00	0.00	0.00	0.00	0.00	0.00	0.00
0.00	0.00	0.00	0.00	0.00	0.00	0.00	0.00	0.00
0.00	0.00	0.00	0.00	0.00	0.00	0.00	0.00	0.00
0.00	0.00	0.00	0.00	0.00	0.00	0.00	0.00	2.00
0.00	0.00	0.00	0.00	0.00	0.00	0.00	0.00	0.00
0.00	0.00	0.00	0.00	0.00	0.00	0.00	0.00	0.00
0.00	0.00	0.00	0.00	0.00	0.00	0.00	0.00	0.00
0.00	0.00	0.00	0.00	0.00	0.00	0.00	0.00	0.00
0.00	0.00	0.00	0.00	0.00	0.00	0.00	0.00	0.00
0.00	0.00	0.00	0.00	0.00	0.00	0.00	0.00	0.00
0.00	0.00	0.00	0.00	0.00	0.00	0.00	0.00	0.00
0.00	0.00	0.00	0.00	0.00	0.00	0.00	0.00	0.00
0.00	0.00	0.00	0.00	0.00	0.00	0.00	0.00	0.00
0.00	0.00	0.00	0.00	0.00	0.00	0.00	0.00	0.00
0.00	0.00	0.00	0.00	0.00	0.00	0.00	0.00	0.00
0.00	0.00	0.00	0.00	0.00	0.00	0.00	0.00	0.00
0.00	0.00	0.00	0.00	0.00	0.00	0.00	0.00	0.00
0.00	0.00	0.00	0.00	0.00	0.00	0.00	0.00	0.00
0.00	0.00	0.00	0.00	0.00	0.00	0.00	0.00	0.00
0.00	0.00	0.00	0.00	0.00	0.00	0.00	0.00	0.00
0.00	0.00	0.00	0.00	0.00	0.00	0.00	0.00	0.00
0.00	0.00	0.00	0.00	0.00	0.00	0.00	0.00	0.00
0.00	0.00	0.00	0.00	0.00	0.00	0.00	0.00	0.00
0.00	0.00	0.00	0.00	0.00	0.00	0.00	0.00	0.00
0.00	0.00	0.00	0.00	0.00	0.00	0.00	0.00	0.00
0.00	0.00	0.00	0.00	0.00	0.00	0.00	0.00	0.00
0.00	0.00	0.00	0.00	0.00	0.00	0.00	0.00	0.00
0.00	0.00	0.00	0.00	0.00	0.00	0.00	0.00	0.00
0.00	0.00	0.00	0.00	0.00	0.00	0.00	0.00	0.00
0.00	0.00	0.00	0.00	0.00	0.00	0.00	0.00	0.00
0.00	0.00	0.00	0.00	0.00	0.00	0.00	0.00	0.00
0.00	0.00	0.00	0.00	0.00	0.00	0.00	0.00	0.00

0.00	0.00	0.00	0.00	0.00	0.00	0.00	0.00	0.00
0.00	0.00	0.00	0.00	0.00	0.00	0.00	0.00	0.00
0.00	0.00	0.00	0.00	0.00	0.00	0.00	0.00	0.00
0.00	0.00	0.00	0.00	0.00	0.00	0.00	0.00	0.00
0.00	0.00	0.00	0.00	0.00	0.00	0.00	0.00	0.00
0.00	0.00	0.00	0.00	0.00	0.00	0.00	0.00	0.00
0.00	0.00	0.00	0.00	0.00	0.00	0.00	0.00	0.00
0.00	0.00	0.00	0.00	0.00	0.00	0.00	0.00	0.00
0.00	0.00	0.00	0.00	0.00	0.00	0.00	0.00	0.00
0.00	0.00	0.00	0.00	0.00	0.00	0.00	0.00	0.00
0.00	0.00	0.00	0.00	0.00	0.00	0.00	0.00	0.00
0.00	0.00	0.00	0.00	0.00	0.00	0.00	0.00	0.00
0.00	0.00	0.00	0.00	0.00	0.00	0.00	0.00	0.00
0.00	0.00	0.00	0.00	0.00	0.00	0.00	0.00	0.00
0.00	0.00	0.00	0.00	0.00	0.00	0.00	0.00	0.00
0.00	0.00	0.00	0.00	0.00	0.00	0.00	0.00	0.00
0.00	0.00	0.00	0.00	0.00	0.00	0.00	0.00	0.00
0.00	0.00	0.00	0.00	0.00	0.00	0.00	0.00	0.00
0.00	0.00	0.00	0.00	0.00	0.00	0.00	0.00	0.00
0.00	0.00	0.00	0.00	0.00	0.00	0.00	2.00	0.00
0.00	0.00	0.00	0.00	0.00	0.00	0.00	2.00	0.00
0.00	0.00	0.00	0.00	0.00	0.00	0.00	0.00	0.00
0.00	0.00	0.00	0.00	0.00	0.00	0.00	0.00	0.00
0.00	0.00	0.00	0.00	0.00	0.00	0.00	0.00	0.00
0.00	0.00	0.00	0.00	0.00	0.00	0.00	0.00	0.00
0.00	0.00	0.00	0.00	0.00	0.00	0.00	0.00	0.00
0.00	0.00	0.00	0.00	0.00	0.00	0.00	0.00	0.00
0.00	0.00	0.00	0.00	0.00	0.00	0.00	0.00	0.00
0.00	0.00	0.00	0.00	0.00	0.00	0.00	0.00	0.00
0.00	0.00	0.00	0.00	0.00	0.00	0.00	0.00	0.00
0.00	0.00	0.00	0.00	0.00	0.00	0.00	0.00	0.00
0.00	0.00	0.00	0.00	0.00	0.00	0.00	0.00	0.00
0.00	0.00	0.00	0.00	0.00	0.00	0.00	0.00	0.00

C19	D19	D18	D17	D16	C18	C17	C16	C15
17913.00	65511.00	65283.00	461.00	24811.00	398.00	57818.00	61081.00	64231.00
0.00	0.00	0.00	0.00	0.00	0.00	0.00	0.00	8.00
1007.00	658.00	992.00	368.00	1060.00	10486.00	1569.00	1194.00	1035.00
0.00	38.00	0.00	0.00	0.00	11.00	0.00	0.00	0.00
0.00	0.00	0.00	0.00	0.00	0.00	0.00	0.00	0.00
0.00	0.00	0.00	0.00	0.00	0.00	76.00	0.00	0.00
33.00	24.00	235.00	6664.00	15099.00	140.00	111.00	0.00	48.00
0.00	0.00	0.00	0.00	0.00	0.00	0.00	0.00	0.00
14091.00	43.00	51.00	67.00	309.00	54.00	165.00	55.00	109.00
0.00	0.00	0.00	0.00	0.00	0.00	0.00	0.00	0.00
0.00	0.00	87.00	2743.00	11550.00	83.00	0.00	0.00	0.00
46.00	39.00	55.00	38505.00	103.00	44.00	0.00	0.00	0.00
9214.00	22.00	25.00	24.00	71.00	22.00	359.00	7.00	46.00
0.00	0.00	10.00	8.00	0.00	0.00	0.00	0.00	0.00
0.00	0.00	0.00	0.00	0.00	0.00	0.00	0.00	0.00
0.00	0.00	0.00	0.00	0.00	0.00	0.00	0.00	0.00
0.00	0.00	104.00	2025.00	5831.00	64.00	0.00	0.00	0.00
0.00	0.00	0.00	0.00	0.00	0.00	0.00	0.00	0.00
120.00	17.00	7.00	69.00	18.00	8225.00	103.00	19.00	164.00
0.00	0.00	0.00	0.00	54.00	0.00	0.00	0.00	0.00
0.00	0.00	0.00	0.00	0.00	0.00	0.00	0.00	0.00
0.00	0.00	9.00	0.00	0.00	0.00	3.00	0.00	5.00
12.00	8.00	17.00	10.00	27.00	25146.00	0.00	0.00	0.00
0.00	0.00	0.00	0.00	0.00	0.00	0.00	0.00	0.00
0.00	0.00	0.00	0.00	0.00	0.00	0.00	0.00	0.00
84.00	37.00	60.00	33.00	126.00	303.00	0.00	47.00	0.00
0.00	0.00	0.00	0.00	0.00	0.00	0.00	0.00	0.00
0.00	0.00	0.00	0.00	0.00	0.00	15.00	0.00	0.00
0.00	0.00	0.00	0.00	0.00	0.00	0.00	0.00	0.00
0.00	0.00	240.00	1025.00	0.00	0.00	0.00	78.00	0.00
56.00	24.00	30.00	0.00	0.00	540.00	70.00	45.00	62.00
6700.00	13.00	22.00	0.00	20.00	0.00	0.00	0.00	55.00
0.00	0.00	0.00	7205.00	0.00	0.00	0.00	0.00	0.00
6887.00	0.00	0.00	26.00	0.00	15.00	0.00	0.00	0.00
0.00	0.00	0.00	0.00	0.00	0.00	0.00	0.00	0.00

8.00	0.00	0.00	0.00	0.00	0.00	0.00	0.00	6.00
148.00	0.00	11.00	18.00	108.00	1418.00	4.00	0.00	12.00
7.00	9.00	27.00	8.00	0.00	88.00	31.00	10.00	11.00
0.00	0.00	0.00	0.00	0.00	0.00	0.00	0.00	0.00
0.00	0.00	0.00	0.00	0.00	0.00	0.00	0.00	0.00
7.00	0.00	0.00	0.00	2.00	0.00	0.00	0.00	0.00
0.00	0.00	0.00	0.00	0.00	0.00	0.00	0.00	0.00
0.00	33.00	6.00	0.00	13.00	0.00	0.00	28.00	17.00
0.00	0.00	40.00	0.00	0.00	374.00	0.00	54.00	18.00
0.00	0.00	0.00	0.00	0.00	0.00	0.00	0.00	0.00
0.00	0.00	0.00	0.00	0.00	0.00	0.00	0.00	0.00
0.00	3.00	5.00	0.00	0.00	0.00	0.00	0.00	0.00
0.00	0.00	0.00	0.00	0.00	113.00	0.00	0.00	0.00
16.00	6.00	30.00	10.00	0.00	199.00	10.00	0.00	16.00
0.00	0.00	0.00	0.00	0.00	0.00	0.00	0.00	0.00
52.00	0.00	0.00	5.00	31.00	143.00	0.00	0.00	0.00
0.00	0.00	0.00	0.00	0.00	0.00	0.00	0.00	0.00
0.00	0.00	136.00	0.00	0.00	0.00	0.00	0.00	0.00
0.00	0.00	0.00	0.00	0.00	0.00	0.00	0.00	0.00
0.00	0.00	0.00	0.00	0.00	0.00	0.00	0.00	0.00
32.00	20.00	0.00	7.00	46.00	332.00	44.00	0.00	0.00
0.00	0.00	0.00	0.00	0.00	0.00	0.00	0.00	0.00
0.00	0.00	0.00	0.00	0.00	0.00	0.00	0.00	0.00
0.00	0.00	12.00	0.00	0.00	0.00	0.00	0.00	0.00
14.00	0.00	6.00	0.00	0.00	0.00	33.00	0.00	8.00
0.00	0.00	0.00	0.00	0.00	0.00	0.00	0.00	0.00
0.00	0.00	0.00	2.00	0.00	170.00	0.00	0.00	0.00
0.00	0.00	0.00	0.00	0.00	0.00	0.00	0.00	0.00
0.00	0.00	0.00	0.00	0.00	0.00	0.00	0.00	0.00
0.00	0.00	0.00	0.00	0.00	0.00	0.00	0.00	0.00
0.00	37.00	0.00	0.00	17.00	0.00	36.00	0.00	0.00
0.00	0.00	0.00	0.00	0.00	0.00	0.00	0.00	0.00
21.00	0.00	17.00	0.00	0.00	0.00	31.00	0.00	12.00
0.00	0.00	0.00	0.00	0.00	0.00	0.00	0.00	0.00
1259.00	0.00	0.00	0.00	0.00	0.00	0.00	0.00	0.00
70.00	11.00	0.00	97.00	3.00	0.00	0.00	0.00	0.00
22.00	22.00	23.00	21.00	31.00	59.00	0.00	0.00	0.00

0.00	0.00	0.00	0.00	0.00	0.00	0.00	0.00	0.00
10.00	0.00	0.00	0.00	0.00	0.00	10.00	0.00	0.00
0.00	0.00	0.00	0.00	0.00	0.00	0.00	0.00	0.00
0.00	18.00	0.00	0.00	0.00	0.00	0.00	0.00	0.00
0.00	0.00	35.00	0.00	8.00	0.00	0.00	0.00	37.00
0.00	0.00	0.00	4.00	0.00	0.00	0.00	0.00	0.00
0.00	0.00	0.00	0.00	0.00	0.00	0.00	0.00	0.00
0.00	0.00	0.00	5.00	0.00	0.00	0.00	0.00	0.00
17.00	0.00	0.00	0.00	0.00	7.00	0.00	0.00	0.00
0.00	0.00	0.00	0.00	0.00	0.00	0.00	0.00	0.00
0.00	0.00	10.00	0.00	0.00	0.00	3.00	5.00	6.00
0.00	0.00	0.00	0.00	0.00	0.00	0.00	0.00	0.00
0.00	0.00	0.00	0.00	10.00	50.00	0.00	54.00	0.00
0.00	6.00	0.00	0.00	17.00	0.00	0.00	0.00	0.00
0.00	0.00	0.00	0.00	0.00	0.00	0.00	0.00	0.00
10.00	0.00	0.00	0.00	0.00	0.00	0.00	0.00	0.00
0.00	0.00	0.00	0.00	0.00	0.00	0.00	0.00	0.00
0.00	0.00	0.00	0.00	0.00	0.00	0.00	0.00	0.00
0.00	0.00	0.00	0.00	0.00	0.00	46.00	0.00	0.00
0.00	6.00	0.00	0.00	0.00	0.00	0.00	0.00	0.00
206.00	0.00	0.00	0.00	0.00	26.00	18.00	0.00	240.00
188.00	94.00	167.00	155.00	204.00	250.00	44.00	70.00	78.00
43.00	0.00	0.00	15.00	20.00	280.00	0.00	33.00	0.00
0.00	0.00	0.00	0.00	0.00	0.00	0.00	0.00	0.00
0.00	0.00	0.00	0.00	0.00	274.00	0.00	0.00	0.00
0.00	0.00	0.00	0.00	0.00	0.00	0.00	0.00	0.00
11.00	11.00	19.00	11.00	29.00	50.00	0.00	0.00	0.00
0.00	0.00	0.00	0.00	0.00	248.00	0.00	0.00	0.00
135.00	0.00	0.00	0.00	2.00	72.00	54.00	0.00	143.00
0.00	0.00	0.00	0.00	0.00	0.00	0.00	0.00	0.00
0.00	0.00	0.00	0.00	0.00	0.00	0.00	0.00	0.00
0.00	0.00	0.00	0.00	29.00	0.00	2.00	0.00	0.00
0.00	0.00	10.00	0.00	0.00	0.00	10.00	35.00	3.00
45.00	79.00	106.00	73.00	69.00	45.00	44.00	54.00	0.00
0.00	0.00	0.00	0.00	0.00	0.00	0.00	0.00	0.00
0.00	0.00	0.00	0.00	0.00	0.00	5.00	0.00	0.00
0.00	10.00	0.00	0.00	0.00	0.00	0.00	0.00	0.00

169.00	121.00	153.00	113.00	156.00	169.00	103.00	60.00	104.00
11.00	0.00	0.00	0.00	0.00	0.00	0.00	0.00	0.00
0.00	0.00	0.00	0.00	0.00	0.00	0.00	0.00	0.00
0.00	0.00	0.00	0.00	0.00	0.00	0.00	20.00	0.00
0.00	19.00	0.00	0.00	26.00	125.00	0.00	20.00	0.00
0.00	0.00	0.00	0.00	0.00	0.00	0.00	0.00	0.00
0.00	0.00	7.00	0.00	0.00	0.00	2.00	6.00	5.00
0.00	0.00	0.00	0.00	0.00	0.00	0.00	0.00	0.00
0.00	23.00	0.00	0.00	125.00	174.00	77.00	31.00	7.00
0.00	0.00	0.00	0.00	0.00	0.00	0.00	0.00	0.00
0.00	0.00	0.00	0.00	0.00	0.00	0.00	0.00	0.00
0.00	0.00	0.00	0.00	0.00	0.00	0.00	0.00	0.00
0.00	0.00	0.00	0.00	0.00	0.00	0.00	0.00	0.00
58.00	72.00	0.00	59.00	0.00	168.00	0.00	75.00	82.00
0.00	0.00	0.00	0.00	0.00	0.00	0.00	0.00	0.00
0.00	0.00	0.00	0.00	0.00	0.00	0.00	0.00	0.00
0.00	0.00	0.00	0.00	0.00	0.00	0.00	0.00	0.00
1292.00	0.00	0.00	0.00	0.00	0.00	0.00	0.00	0.00
0.00	0.00	0.00	0.00	0.00	0.00	0.00	0.00	0.00
0.00	4.00	0.00	0.00	0.00	0.00	0.00	77.00	0.00
0.00	0.00	0.00	0.00	0.00	0.00	0.00	0.00	0.00
0.00	0.00	0.00	0.00	0.00	0.00	0.00	0.00	0.00
0.00	0.00	0.00	0.00	0.00	0.00	0.00	0.00	0.00
0.00	0.00	0.00	0.00	0.00	0.00	0.00	0.00	6.00
0.00	0.00	0.00	0.00	0.00	0.00	0.00	0.00	0.00
0.00	0.00	0.00	0.00	0.00	0.00	0.00	0.00	0.00
10.00	0.00	16.00	0.00	16.00	30.00	0.00	0.00	0.00
0.00	0.00	0.00	0.00	0.00	0.00	0.00	0.00	0.00
0.00	0.00	0.00	0.00	0.00	0.00	0.00	0.00	0.00
0.00	0.00	0.00	0.00	0.00	0.00	0.00	0.00	0.00
0.00	0.00	0.00	0.00	0.00	0.00	0.00	0.00	0.00
0.00	0.00	0.00	0.00	0.00	0.00	0.00	0.00	0.00
0.00	0.00	0.00	0.00	0.00	322.00	0.00	0.00	0.00
0.00	0.00	0.00	0.00	0.00	0.00	0.00	0.00	0.00
0.00	0.00	0.00	0.00	0.00	0.00	0.00	0.00	0.00
0.00	5.00	0.00	0.00	0.00	0.00	0.00	0.00	0.00
0.00	0.00	0.00	0.00	0.00	0.00	0.00	0.00	0.00

0.00	0.00	0.00	0.00	0.00	0.00	0.00	0.00	0.00
0.00	0.00	0.00	0.00	0.00	0.00	0.00	0.00	0.00
55.00	37.00	52.00	36.00	54.00	90.00	41.00	27.00	59.00
0.00	0.00	0.00	0.00	0.00	0.00	0.00	0.00	0.00
0.00	0.00	0.00	0.00	0.00	0.00	0.00	0.00	0.00
0.00	0.00	0.00	0.00	0.00	0.00	0.00	0.00	0.00
0.00	0.00	0.00	0.00	0.00	0.00	0.00	0.00	0.00
0.00	0.00	0.00	0.00	0.00	0.00	0.00	0.00	0.00
0.00	0.00	6.00	0.00	0.00	0.00	0.00	0.00	0.00
0.00	0.00	0.00	0.00	0.00	0.00	0.00	4.00	0.00
0.00	0.00	0.00	0.00	0.00	0.00	0.00	0.00	0.00
0.00	0.00	0.00	0.00	11.00	0.00	0.00	0.00	0.00
0.00	0.00	0.00	0.00	0.00	0.00	0.00	0.00	0.00
12.00	0.00	0.00	5.00	0.00	0.00	0.00	0.00	46.00
3.00	0.00	0.00	0.00	0.00	0.00	0.00	0.00	0.00
0.00	0.00	0.00	0.00	0.00	0.00	0.00	0.00	0.00
0.00	0.00	0.00	0.00	0.00	0.00	0.00	0.00	0.00
0.00	0.00	0.00	17.00	20.00	26.00	0.00	0.00	0.00
0.00	0.00	0.00	0.00	0.00	0.00	0.00	0.00	0.00
0.00	0.00	0.00	0.00	0.00	0.00	0.00	0.00	13.00
20.00	10.00	20.00	23.00	31.00	39.00	0.00	0.00	0.00
0.00	0.00	0.00	0.00	0.00	0.00	0.00	0.00	0.00
0.00	0.00	0.00	0.00	0.00	0.00	0.00	0.00	0.00
0.00	0.00	0.00	0.00	0.00	0.00	0.00	0.00	0.00
0.00	0.00	0.00	0.00	0.00	0.00	0.00	0.00	4.00
0.00	0.00	0.00	0.00	0.00	0.00	0.00	0.00	0.00
13.00	11.00	7.00	0.00	6.00	11.00	0.00	8.00	8.00
22.00	0.00	0.00	7.00	0.00	24.00	0.00	0.00	0.00
0.00	0.00	0.00	0.00	0.00	0.00	0.00	0.00	0.00
0.00	0.00	0.00	0.00	0.00	0.00	0.00	0.00	0.00
0.00	0.00	0.00	0.00	0.00	0.00	0.00	0.00	0.00
38.00	9.00	37.00	23.00	27.00	61.00	37.00	23.00	64.00
0.00	0.00	0.00	0.00	0.00	0.00	0.00	0.00	0.00
0.00	0.00	0.00	0.00	0.00	0.00	0.00	0.00	0.00
0.00	0.00	0.00	0.00	0.00	0.00	0.00	0.00	0.00
0.00	0.00	21.00	0.00	22.00	18.00	0.00	0.00	0.00
0.00	0.00	0.00	9.00	0.00	184.00	0.00	0.00	0.00

0.00	0.00	0.00	0.00	0.00	0.00	0.00	0.00	0.00
0.00	0.00	0.00	0.00	0.00	0.00	0.00	0.00	0.00
0.00	0.00	0.00	0.00	0.00	0.00	0.00	0.00	0.00
0.00	0.00	0.00	0.00	0.00	0.00	0.00	0.00	0.00
0.00	0.00	0.00	0.00	0.00	0.00	0.00	0.00	0.00
0.00	0.00	0.00	0.00	0.00	150.00	0.00	0.00	0.00
0.00	0.00	0.00	0.00	0.00	0.00	0.00	0.00	0.00
0.00	0.00	0.00	0.00	0.00	0.00	0.00	0.00	0.00
0.00	0.00	0.00	0.00	0.00	0.00	0.00	0.00	0.00
0.00	0.00	0.00	0.00	0.00	0.00	0.00	7.00	0.00
0.00	0.00	0.00	0.00	0.00	0.00	0.00	0.00	0.00
0.00	0.00	0.00	0.00	0.00	2.00	0.00	0.00	0.00
34.00	0.00	0.00	0.00	0.00	0.00	26.00	0.00	32.00
0.00	0.00	0.00	0.00	0.00	0.00	0.00	0.00	0.00
0.00	3.00	0.00	0.00	0.00	0.00	0.00	0.00	0.00
0.00	0.00	0.00	0.00	0.00	0.00	0.00	0.00	0.00
0.00	0.00	0.00	0.00	0.00	0.00	0.00	0.00	0.00
0.00	0.00	0.00	0.00	0.00	0.00	0.00	0.00	0.00
0.00	0.00	0.00	0.00	0.00	0.00	0.00	0.00	0.00
0.00	0.00	0.00	0.00	0.00	0.00	0.00	0.00	0.00
0.00	0.00	0.00	0.00	0.00	0.00	0.00	0.00	0.00
0.00	0.00	0.00	0.00	0.00	0.00	0.00	0.00	0.00
0.00	0.00	0.00	0.00	0.00	0.00	0.00	0.00	0.00
0.00	0.00	0.00	0.00	0.00	588.00	0.00	0.00	0.00
0.00	0.00	0.00	0.00	0.00	0.00	0.00	0.00	0.00
0.00	0.00	0.00	0.00	0.00	0.00	0.00	0.00	0.00
0.00	3.00	0.00	0.00	0.00	0.00	0.00	0.00	0.00
0.00	0.00	0.00	0.00	0.00	0.00	0.00	0.00	0.00
0.00	0.00	0.00	0.00	0.00	0.00	0.00	0.00	0.00
0.00	0.00	0.00	0.00	0.00	0.00	0.00	0.00	0.00
0.00	0.00	0.00	0.00	0.00	0.00	0.00	0.00	0.00
0.00	0.00	0.00	0.00	0.00	145.00	0.00	0.00	0.00
0.00	0.00	0.00	0.00	0.00	567.00	0.00	0.00	0.00
21.00	0.00	0.00	0.00	0.00	19.00	0.00	0.00	0.00
0.00	0.00	0.00	0.00	0.00	0.00	0.00	0.00	0.00
61.00	0.00	0.00	0.00	0.00	0.00	35.00	31.00	38.00
0.00	0.00	0.00	0.00	0.00	0.00	0.00	0.00	0.00

0.00	0.00	0.00	0.00	0.00	0.00	0.00	0.00	0.00
0.00	0.00	0.00	0.00	0.00	538.00	0.00	0.00	0.00
0.00	0.00	0.00	0.00	0.00	0.00	0.00	0.00	0.00
11.00	15.00	9.00	16.00	13.00	42.00	6.00	7.00	17.00
0.00	0.00	0.00	0.00	0.00	0.00	0.00	0.00	0.00
0.00	0.00	0.00	2.00	0.00	0.00	0.00	0.00	0.00
0.00	0.00	0.00	0.00	0.00	0.00	0.00	0.00	0.00
0.00	0.00	0.00	0.00	0.00	0.00	0.00	0.00	0.00
0.00	0.00	5.00	0.00	0.00	0.00	0.00	0.00	0.00
0.00	0.00	0.00	0.00	0.00	0.00	0.00	0.00	0.00
0.00	0.00	0.00	0.00	0.00	0.00	11.00	0.00	0.00
0.00	0.00	0.00	0.00	0.00	0.00	0.00	0.00	0.00
0.00	0.00	0.00	0.00	0.00	0.00	0.00	0.00	0.00
0.00	0.00	0.00	0.00	0.00	0.00	0.00	0.00	0.00
6.00	0.00	0.00	0.00	0.00	0.00	0.00	0.00	0.00
0.00	0.00	0.00	0.00	0.00	0.00	0.00	0.00	0.00
0.00	0.00	0.00	0.00	0.00	0.00	0.00	0.00	0.00
0.00	0.00	0.00	0.00	0.00	0.00	0.00	0.00	0.00
0.00	0.00	0.00	0.00	0.00	0.00	0.00	0.00	0.00
0.00	0.00	0.00	0.00	0.00	0.00	0.00	0.00	0.00
13.00	0.00	53.00	0.00	0.00	30.00	0.00	42.00	0.00
0.00	0.00	0.00	0.00	0.00	0.00	0.00	0.00	0.00
0.00	0.00	0.00	0.00	0.00	0.00	0.00	0.00	0.00
0.00	0.00	0.00	0.00	0.00	0.00	0.00	0.00	0.00
0.00	0.00	0.00	0.00	0.00	0.00	0.00	0.00	0.00
40.00	0.00	0.00	0.00	0.00	34.00	0.00	0.00	50.00
0.00	0.00	0.00	0.00	0.00	128.00	0.00	0.00	0.00
0.00	0.00	0.00	174.00	0.00	0.00	0.00	0.00	0.00
0.00	0.00	0.00	0.00	0.00	0.00	0.00	0.00	0.00
3.00	0.00	0.00	8.00	0.00	169.00	0.00	0.00	0.00
0.00	0.00	4.00	0.00	0.00	0.00	2.00	0.00	0.00
0.00	0.00	0.00	0.00	0.00	463.00	0.00	0.00	0.00
0.00	0.00	0.00	0.00	0.00	0.00	0.00	0.00	0.00
0.00	0.00	0.00	0.00	0.00	0.00	0.00	0.00	0.00
0.00	0.00	0.00	0.00	0.00	0.00	0.00	0.00	0.00
0.00	0.00	0.00	0.00	0.00	0.00	0.00	0.00	0.00
0.00	0.00	0.00	0.00	0.00	0.00	0.00	0.00	0.00

0.00	0.00	0.00	0.00	0.00	0.00	0.00	0.00	0.00
0.00	0.00	0.00	0.00	0.00	0.00	0.00	0.00	0.00
0.00	0.00	0.00	0.00	0.00	0.00	0.00	0.00	0.00
0.00	0.00	0.00	0.00	0.00	0.00	0.00	0.00	0.00
0.00	0.00	0.00	0.00	4.00	0.00	7.00	0.00	21.00
0.00	0.00	0.00	0.00	0.00	424.00	0.00	0.00	0.00
0.00	0.00	0.00	0.00	0.00	0.00	0.00	0.00	0.00
0.00	0.00	0.00	0.00	0.00	0.00	0.00	0.00	0.00
0.00	0.00	0.00	0.00	0.00	0.00	0.00	0.00	0.00
0.00	0.00	0.00	0.00	0.00	0.00	0.00	0.00	0.00
0.00	0.00	0.00	0.00	0.00	0.00	0.00	0.00	0.00
0.00	0.00	0.00	0.00	0.00	0.00	0.00	0.00	0.00
0.00	0.00	3.00	0.00	0.00	0.00	0.00	0.00	0.00
0.00	0.00	0.00	0.00	0.00	0.00	0.00	0.00	0.00
40.00	52.00	55.00	0.00	36.00	75.00	0.00	0.00	37.00
0.00	0.00	0.00	0.00	0.00	0.00	0.00	0.00	0.00
4.00	7.00	3.00	3.00	10.00	16.00	0.00	0.00	0.00
13.00	10.00	11.00	12.00	17.00	19.00	0.00	0.00	0.00
0.00	0.00	0.00	0.00	0.00	0.00	0.00	0.00	0.00
0.00	0.00	0.00	0.00	0.00	224.00	0.00	0.00	0.00
0.00	0.00	0.00	0.00	0.00	0.00	0.00	0.00	0.00
10.00	0.00	0.00	0.00	0.00	318.00	0.00	0.00	0.00
0.00	0.00	0.00	0.00	0.00	0.00	0.00	0.00	0.00
0.00	0.00	0.00	0.00	0.00	0.00	0.00	0.00	0.00
0.00	31.00	33.00	31.00	0.00	0.00	0.00	51.00	0.00
0.00	0.00	0.00	0.00	0.00	0.00	0.00	0.00	0.00
0.00	0.00	0.00	0.00	0.00	0.00	0.00	0.00	0.00
0.00	0.00	0.00	0.00	0.00	354.00	0.00	0.00	0.00
0.00	0.00	0.00	0.00	0.00	354.00	0.00	0.00	0.00
7.00	0.00	0.00	0.00	27.00	2.00	31.00	0.00	13.00
0.00	0.00	0.00	0.00	0.00	0.00	0.00	0.00	0.00
0.00	0.00	0.00	0.00	0.00	0.00	0.00	0.00	0.00
0.00	0.00	0.00	0.00	0.00	0.00	0.00	0.00	0.00
0.00	0.00	0.00	0.00	0.00	0.00	0.00	0.00	0.00
0.00	0.00	0.00	0.00	0.00	0.00	0.00	0.00	0.00
0.00	0.00	0.00	0.00	0.00	0.00	0.00	0.00	0.00
0.00	0.00	0.00	0.00	0.00	0.00	0.00	0.00	0.00

32.00	0.00	0.00	28.00	35.00	48.00	23.00	21.00	0.00
19.00	13.00	22.00	14.00	18.00	24.00	17.00	10.00	18.00
0.00	0.00	0.00	0.00	0.00	0.00	0.00	0.00	0.00
0.00	0.00	0.00	0.00	0.00	0.00	0.00	0.00	0.00
46.00	33.00	0.00	0.00	0.00	0.00	0.00	0.00	61.00
0.00	0.00	0.00	0.00	0.00	0.00	0.00	0.00	0.00
0.00	0.00	0.00	0.00	0.00	0.00	0.00	0.00	0.00
0.00	0.00	0.00	0.00	0.00	0.00	0.00	0.00	0.00
0.00	0.00	0.00	0.00	0.00	0.00	0.00	0.00	0.00
15.00	5.00	12.00	0.00	12.00	19.00	0.00	0.00	0.00
0.00	0.00	0.00	0.00	6.00	0.00	0.00	0.00	0.00
0.00	0.00	0.00	0.00	0.00	0.00	0.00	0.00	0.00
0.00	0.00	3.00	0.00	0.00	11.00	0.00	0.00	0.00
0.00	0.00	0.00	0.00	0.00	0.00	0.00	0.00	5.00
0.00	0.00	0.00	0.00	0.00	0.00	0.00	0.00	0.00
0.00	0.00	0.00	0.00	0.00	0.00	0.00	0.00	0.00
0.00	0.00	0.00	318.00	0.00	0.00	0.00	0.00	0.00
0.00	0.00	0.00	0.00	0.00	5.00	0.00	0.00	0.00
0.00	0.00	0.00	0.00	0.00	0.00	0.00	0.00	0.00
0.00	0.00	0.00	0.00	0.00	0.00	0.00	0.00	0.00
0.00	0.00	0.00	0.00	0.00	0.00	0.00	0.00	11.00
0.00	0.00	0.00	0.00	0.00	0.00	0.00	0.00	0.00
0.00	0.00	0.00	0.00	0.00	0.00	0.00	0.00	0.00
0.00	0.00	0.00	0.00	0.00	0.00	0.00	0.00	0.00
0.00	0.00	0.00	0.00	0.00	0.00	0.00	0.00	0.00
0.00	0.00	0.00	0.00	0.00	0.00	0.00	0.00	0.00
0.00	0.00	0.00	0.00	0.00	0.00	0.00	0.00	0.00
0.00	0.00	0.00	0.00	40.00	0.00	41.00	0.00	0.00
0.00	0.00	0.00	0.00	0.00	0.00	0.00	0.00	0.00
0.00	0.00	0.00	0.00	0.00	9.00	0.00	0.00	0.00
0.00	0.00	0.00	0.00	0.00	0.00	0.00	0.00	0.00
0.00	0.00	0.00	0.00	0.00	0.00	0.00	0.00	0.00
0.00	0.00	0.00	0.00	0.00	0.00	0.00	0.00	0.00
0.00	0.00	0.00	0.00	0.00	0.00	0.00	0.00	0.00
0.00	0.00	0.00	0.00	0.00	0.00	0.00	0.00	0.00
0.00	0.00	0.00	0.00	0.00	296.00	0.00	0.00	0.00
0.00	0.00	0.00	294.00	0.00	0.00	0.00	0.00	0.00

0.00	0.00	0.00	0.00	0.00	0.00	0.00	0.00	0.00
0.00	0.00	0.00	0.00	0.00	0.00	0.00	0.00	0.00
0.00	0.00	0.00	0.00	0.00	0.00	0.00	0.00	0.00
0.00	0.00	0.00	0.00	0.00	0.00	0.00	0.00	0.00
0.00	0.00	0.00	0.00	0.00	0.00	0.00	0.00	0.00
0.00	0.00	7.00	10.00	10.00	16.00	11.00	15.00	0.00
0.00	0.00	0.00	0.00	0.00	0.00	0.00	0.00	0.00
0.00	0.00	0.00	0.00	0.00	0.00	0.00	0.00	0.00
44.00	0.00	0.00	15.00	0.00	0.00	0.00	0.00	0.00
7.00	0.00	0.00	0.00	35.00	0.00	21.00	0.00	0.00
0.00	0.00	0.00	0.00	0.00	0.00	0.00	0.00	0.00
0.00	0.00	0.00	0.00	0.00	0.00	0.00	0.00	0.00
0.00	0.00	0.00	0.00	0.00	8.00	0.00	0.00	0.00
0.00	0.00	0.00	0.00	0.00	0.00	0.00	0.00	0.00
0.00	0.00	54.00	0.00	0.00	0.00	0.00	0.00	0.00
0.00	0.00	0.00	0.00	0.00	0.00	0.00	0.00	0.00
0.00	0.00	0.00	0.00	0.00	260.00	0.00	0.00	0.00
0.00	0.00	0.00	0.00	0.00	0.00	0.00	0.00	0.00
0.00	0.00	0.00	0.00	0.00	0.00	0.00	0.00	0.00
0.00	0.00	0.00	0.00	0.00	256.00	0.00	0.00	0.00
0.00	0.00	0.00	0.00	0.00	0.00	0.00	0.00	0.00
0.00	0.00	0.00	0.00	0.00	0.00	0.00	0.00	0.00
0.00	6.00	0.00	4.00	0.00	4.00	0.00	0.00	0.00
12.00	0.00	0.00	0.00	0.00	0.00	0.00	0.00	9.00
0.00	0.00	0.00	0.00	0.00	0.00	0.00	74.00	0.00
0.00	0.00	0.00	0.00	0.00	247.00	0.00	0.00	0.00
0.00	0.00	0.00	0.00	0.00	0.00	0.00	0.00	0.00
6.00	0.00	0.00	0.00	0.00	0.00	0.00	0.00	0.00
0.00	0.00	0.00	0.00	0.00	0.00	0.00	0.00	0.00
0.00	0.00	0.00	0.00	0.00	0.00	0.00	0.00	0.00
0.00	0.00	0.00	0.00	0.00	0.00	0.00	0.00	0.00
0.00	0.00	0.00	0.00	0.00	0.00	0.00	0.00	0.00
0.00	0.00	0.00	0.00	0.00	0.00	0.00	0.00	0.00
0.00	0.00	0.00	0.00	0.00	0.00	0.00	0.00	0.00
0.00	8.00	0.00	0.00	0.00	0.00	0.00	19.00	0.00
0.00	0.00	0.00	0.00	0.00	0.00	0.00	0.00	0.00
0.00	0.00	0.00	0.00	0.00	0.00	0.00	0.00	0.00

0.00	0.00	0.00	0.00	0.00	0.00	0.00	0.00	0.00
0.00	0.00	0.00	0.00	0.00	0.00	0.00	0.00	0.00
0.00	0.00	0.00	0.00	0.00	0.00	0.00	0.00	0.00
0.00	0.00	0.00	0.00	0.00	218.00	0.00	0.00	0.00
0.00	0.00	0.00	0.00	0.00	0.00	0.00	0.00	0.00
0.00	0.00	0.00	0.00	0.00	0.00	0.00	0.00	0.00
0.00	0.00	0.00	3.00	0.00	3.00	0.00	0.00	0.00
0.00	0.00	0.00	0.00	0.00	0.00	0.00	0.00	0.00
0.00	0.00	0.00	0.00	0.00	0.00	0.00	0.00	0.00
48.00	0.00	0.00	0.00	0.00	0.00	0.00	0.00	0.00
0.00	0.00	0.00	0.00	0.00	200.00	0.00	0.00	0.00
0.00	0.00	0.00	0.00	0.00	222.00	0.00	0.00	0.00
0.00	0.00	0.00	0.00	0.00	0.00	0.00	0.00	0.00
3.00	0.00	5.00	14.00	23.00	34.00	5.00	10.00	5.00
0.00	0.00	0.00	0.00	0.00	0.00	0.00	0.00	0.00
0.00	0.00	0.00	0.00	0.00	0.00	0.00	0.00	0.00
0.00	0.00	0.00	0.00	0.00	0.00	0.00	0.00	0.00
0.00	0.00	0.00	0.00	0.00	0.00	0.00	0.00	0.00
0.00	0.00	0.00	0.00	0.00	0.00	0.00	0.00	0.00
0.00	0.00	0.00	0.00	0.00	0.00	0.00	0.00	0.00
0.00	0.00	0.00	0.00	8.00	0.00	27.00	0.00	0.00
0.00	0.00	0.00	0.00	18.00	23.00	22.00	26.00	34.00
0.00	0.00	0.00	0.00	0.00	0.00	0.00	0.00	0.00
0.00	0.00	0.00	0.00	0.00	0.00	0.00	0.00	0.00
0.00	0.00	10.00	0.00	15.00	18.00	6.00	0.00	0.00
0.00	0.00	0.00	0.00	0.00	0.00	0.00	0.00	0.00
0.00	0.00	0.00	0.00	0.00	0.00	0.00	0.00	0.00
0.00	0.00	0.00	0.00	0.00	0.00	0.00	0.00	0.00
8.00	0.00	2.00	4.00	6.00	6.00	3.00	6.00	4.00
20.00	0.00	0.00	0.00	0.00	0.00	0.00	0.00	53.00
0.00	0.00	0.00	0.00	0.00	0.00	0.00	0.00	0.00
0.00	0.00	0.00	0.00	0.00	0.00	0.00	0.00	0.00
0.00	0.00	0.00	0.00	0.00	0.00	21.00	19.00	25.00
0.00	0.00	0.00	208.00	0.00	0.00	0.00	0.00	0.00
0.00	0.00	0.00	5.00	0.00	0.00	0.00	0.00	0.00
0.00	0.00	0.00	0.00	0.00	0.00	0.00	0.00	0.00
0.00	0.00	0.00	0.00	0.00	0.00	0.00	0.00	0.00

0.00	0.00	0.00	0.00	0.00	0.00	0.00	0.00	0.00
10.00	0.00	0.00	0.00	0.00	0.00	0.00	0.00	0.00
0.00	0.00	0.00	0.00	0.00	0.00	0.00	0.00	0.00
0.00	0.00	0.00	0.00	0.00	0.00	0.00	0.00	0.00
0.00	0.00	0.00	0.00	0.00	0.00	25.00	0.00	0.00
0.00	0.00	0.00	0.00	0.00	183.00	0.00	0.00	0.00
2.00	0.00	0.00	0.00	2.00	0.00	0.00	11.00	2.00
0.00	0.00	0.00	0.00	0.00	196.00	0.00	0.00	0.00
0.00	0.00	0.00	0.00	0.00	0.00	0.00	0.00	0.00
0.00	0.00	0.00	0.00	0.00	0.00	0.00	0.00	0.00
0.00	0.00	0.00	0.00	0.00	194.00	0.00	0.00	0.00
0.00	0.00	0.00	0.00	0.00	0.00	0.00	0.00	0.00
0.00	0.00	0.00	0.00	0.00	0.00	0.00	0.00	0.00
0.00	0.00	0.00	0.00	0.00	0.00	0.00	0.00	0.00
0.00	0.00	0.00	0.00	0.00	0.00	0.00	0.00	0.00
0.00	0.00	0.00	5.00	8.00	6.00	0.00	0.00	0.00
20.00	33.00	29.00	17.00	17.00	28.00	0.00	0.00	0.00
18.00	12.00	0.00	0.00	0.00	24.00	9.00	7.00	11.00
0.00	0.00	0.00	0.00	0.00	0.00	0.00	0.00	0.00
0.00	19.00	11.00	0.00	0.00	18.00	10.00	0.00	0.00
4.00	6.00	2.00	14.00	6.00	7.00	9.00	0.00	0.00
0.00	0.00	0.00	0.00	0.00	181.00	0.00	0.00	0.00
0.00	0.00	0.00	0.00	0.00	0.00	0.00	0.00	0.00
0.00	0.00	0.00	0.00	0.00	0.00	0.00	0.00	0.00
0.00	0.00	0.00	0.00	0.00	0.00	0.00	0.00	0.00
0.00	0.00	0.00	0.00	0.00	0.00	0.00	0.00	0.00
0.00	0.00	0.00	0.00	0.00	0.00	0.00	0.00	0.00
0.00	0.00	0.00	0.00	0.00	0.00	0.00	0.00	0.00
0.00	10.00	6.00	0.00	9.00	0.00	0.00	11.00	21.00
0.00	0.00	0.00	0.00	0.00	0.00	0.00	0.00	0.00
0.00	0.00	8.00	0.00	0.00	90.00	0.00	0.00	0.00
20.00	10.00	13.00	8.00	27.00	50.00	0.00	0.00	0.00
0.00	0.00	0.00	0.00	0.00	0.00	0.00	0.00	0.00
0.00	0.00	0.00	0.00	0.00	0.00	0.00	0.00	0.00
0.00	0.00	47.00	0.00	0.00	0.00	0.00	0.00	0.00
0.00	0.00	0.00	0.00	0.00	0.00	0.00	0.00	0.00
0.00	0.00	0.00	0.00	20.00	16.00	0.00	0.00	10.00

0.00	0.00	0.00	0.00	0.00	0.00	0.00	0.00	0.00
0.00	0.00	0.00	0.00	0.00	0.00	0.00	0.00	0.00
42.00	0.00	0.00	0.00	0.00	0.00	0.00	0.00	0.00
0.00	0.00	0.00	0.00	0.00	0.00	0.00	0.00	0.00
0.00	0.00	0.00	0.00	0.00	0.00	0.00	0.00	0.00
0.00	0.00	0.00	0.00	0.00	0.00	0.00	0.00	0.00
0.00	0.00	0.00	0.00	0.00	0.00	0.00	0.00	0.00
0.00	0.00	0.00	0.00	0.00	64.00	0.00	0.00	0.00
0.00	0.00	0.00	0.00	0.00	0.00	0.00	0.00	0.00
0.00	0.00	0.00	0.00	0.00	171.00	0.00	0.00	0.00
0.00	0.00	0.00	0.00	0.00	0.00	0.00	0.00	0.00
0.00	0.00	0.00	0.00	0.00	0.00	0.00	0.00	0.00
0.00	0.00	0.00	0.00	0.00	0.00	0.00	0.00	0.00
0.00	0.00	0.00	0.00	0.00	170.00	0.00	0.00	0.00
0.00	0.00	0.00	0.00	0.00	0.00	0.00	0.00	0.00
0.00	0.00	0.00	0.00	0.00	0.00	0.00	0.00	0.00
94.00	0.00	0.00	0.00	0.00	0.00	0.00	0.00	0.00
0.00	0.00	0.00	0.00	0.00	114.00	0.00	0.00	0.00
17.00	12.00	0.00	13.00	0.00	31.00	0.00	8.00	26.00
9.00	0.00	13.00	7.00	10.00	16.00	17.00	16.00	15.00
13.00	9.00	0.00	16.00	0.00	25.00	23.00	7.00	20.00
0.00	0.00	0.00	0.00	0.00	0.00	0.00	0.00	0.00
0.00	0.00	0.00	0.00	0.00	0.00	0.00	0.00	0.00
9.00	27.00	2.00	0.00	0.00	0.00	0.00	0.00	3.00
0.00	0.00	0.00	0.00	0.00	163.00	0.00	0.00	0.00
0.00	0.00	0.00	0.00	0.00	0.00	0.00	0.00	0.00
0.00	0.00	0.00	0.00	0.00	0.00	0.00	0.00	0.00
0.00	0.00	0.00	0.00	0.00	0.00	0.00	0.00	0.00
0.00	0.00	0.00	0.00	0.00	0.00	0.00	0.00	0.00
0.00	0.00	0.00	0.00	0.00	160.00	0.00	0.00	0.00
0.00	0.00	0.00	0.00	0.00	0.00	0.00	0.00	0.00
0.00	0.00	0.00	0.00	0.00	0.00	0.00	0.00	0.00
0.00	0.00	0.00	0.00	0.00	0.00	0.00	0.00	0.00
0.00	0.00	0.00	0.00	0.00	2.00	0.00	0.00	0.00
0.00	0.00	0.00	0.00	0.00	0.00	0.00	0.00	0.00
0.00	0.00	0.00	0.00	0.00	0.00	0.00	0.00	0.00
0.00	0.00	0.00	0.00	0.00	0.00	0.00	0.00	0.00

24.00	9.00	23.00	0.00	0.00	0.00	0.00	0.00	25.00
0.00	0.00	0.00	0.00	0.00	0.00	0.00	0.00	0.00
0.00	0.00	0.00	0.00	0.00	0.00	0.00	0.00	0.00
0.00	0.00	0.00	0.00	0.00	0.00	0.00	0.00	0.00
0.00	0.00	0.00	0.00	0.00	0.00	0.00	0.00	0.00
0.00	0.00	0.00	0.00	0.00	0.00	0.00	0.00	0.00
0.00	0.00	0.00	0.00	0.00	0.00	0.00	0.00	0.00
10.00	18.00	4.00	10.00	0.00	0.00	0.00	0.00	0.00
0.00	0.00	0.00	0.00	0.00	153.00	0.00	0.00	0.00
0.00	0.00	0.00	0.00	0.00	0.00	0.00	0.00	0.00
0.00	0.00	0.00	0.00	0.00	0.00	0.00	0.00	0.00
11.00	0.00	4.00	0.00	0.00	137.00	0.00	0.00	0.00
0.00	0.00	0.00	0.00	0.00	0.00	0.00	0.00	0.00
0.00	0.00	0.00	0.00	0.00	0.00	0.00	0.00	0.00
0.00	0.00	0.00	0.00	0.00	0.00	0.00	0.00	0.00
0.00	10.00	12.00	5.00	10.00	9.00	0.00	13.00	19.00
0.00	0.00	0.00	0.00	0.00	0.00	0.00	0.00	0.00
0.00	0.00	0.00	0.00	0.00	0.00	0.00	0.00	0.00
4.00	0.00	2.00	2.00	0.00	2.00	0.00	0.00	0.00
0.00	0.00	0.00	0.00	0.00	0.00	0.00	0.00	0.00
0.00	0.00	0.00	0.00	0.00	0.00	0.00	0.00	0.00
10.00	0.00	0.00	0.00	0.00	10.00	0.00	0.00	0.00
0.00	0.00	0.00	0.00	0.00	0.00	0.00	0.00	0.00
0.00	0.00	0.00	0.00	0.00	8.00	37.00	0.00	0.00
0.00	0.00	0.00	0.00	0.00	0.00	0.00	0.00	0.00
0.00	0.00	0.00	0.00	0.00	0.00	0.00	0.00	0.00
11.00	12.00	8.00	9.00	0.00	14.00	10.00	8.00	15.00
0.00	0.00	0.00	0.00	0.00	0.00	0.00	0.00	0.00
0.00	0.00	0.00	0.00	0.00	0.00	0.00	0.00	0.00
0.00	0.00	0.00	0.00	0.00	0.00	0.00	0.00	0.00
0.00	0.00	0.00	0.00	0.00	139.00	0.00	0.00	0.00
7.00	0.00	10.00	7.00	9.00	18.00	8.00	0.00	18.00
0.00	0.00	20.00	0.00	22.00	0.00	0.00	0.00	0.00
0.00	0.00	0.00	0.00	0.00	0.00	0.00	0.00	0.00
0.00	0.00	0.00	0.00	0.00	0.00	0.00	0.00	0.00
0.00	0.00	0.00	0.00	0.00	0.00	0.00	0.00	0.00
0.00	0.00	0.00	0.00	0.00	0.00	0.00	0.00	0.00

0.00	0.00	0.00	0.00	27.00	0.00	11.00	0.00	0.00
0.00	0.00	0.00	0.00	0.00	0.00	0.00	0.00	0.00
0.00	0.00	0.00	0.00	0.00	0.00	0.00	22.00	35.00
0.00	0.00	0.00	0.00	0.00	0.00	0.00	0.00	0.00
0.00	0.00	0.00	0.00	0.00	0.00	0.00	0.00	0.00
5.00	6.00	0.00	3.00	4.00	10.00	4.00	8.00	12.00
0.00	0.00	0.00	0.00	0.00	0.00	0.00	0.00	0.00
0.00	0.00	0.00	0.00	0.00	0.00	0.00	0.00	0.00
0.00	0.00	0.00	0.00	0.00	0.00	0.00	0.00	0.00
0.00	0.00	0.00	0.00	0.00	0.00	0.00	0.00	0.00
0.00	0.00	0.00	0.00	0.00	0.00	0.00	0.00	0.00
0.00	0.00	0.00	0.00	0.00	0.00	0.00	0.00	0.00
0.00	0.00	0.00	0.00	0.00	0.00	0.00	0.00	0.00
0.00	0.00	0.00	0.00	0.00	0.00	0.00	0.00	0.00
0.00	0.00	0.00	0.00	0.00	0.00	0.00	0.00	0.00
0.00	0.00	0.00	0.00	20.00	40.00	0.00	0.00	0.00
0.00	0.00	0.00	0.00	0.00	0.00	0.00	0.00	0.00
0.00	0.00	0.00	0.00	0.00	0.00	0.00	0.00	0.00
0.00	0.00	0.00	0.00	0.00	0.00	0.00	0.00	0.00
0.00	0.00	0.00	0.00	0.00	0.00	0.00	0.00	0.00
0.00	0.00	0.00	0.00	0.00	3.00	0.00	0.00	0.00
0.00	0.00	0.00	0.00	0.00	0.00	0.00	0.00	0.00
0.00	0.00	0.00	0.00	0.00	0.00	0.00	0.00	0.00
0.00	0.00	28.00	0.00	0.00	0.00	0.00	0.00	0.00
0.00	0.00	0.00	0.00	0.00	121.00	0.00	0.00	0.00
0.00	0.00	0.00	0.00	0.00	0.00	0.00	0.00	0.00
0.00	0.00	0.00	0.00	30.00	0.00	0.00	0.00	0.00
0.00	0.00	0.00	0.00	0.00	119.00	0.00	0.00	0.00
0.00	0.00	0.00	0.00	0.00	0.00	0.00	0.00	0.00
8.00	0.00	0.00	0.00	0.00	0.00	0.00	0.00	0.00
0.00	0.00	43.00	0.00	0.00	0.00	0.00	0.00	0.00
0.00	0.00	0.00	0.00	0.00	0.00	0.00	0.00	0.00
10.00	17.00	12.00	0.00	0.00	0.00	0.00	0.00	10.00
0.00	0.00	0.00	0.00	0.00	0.00	0.00	0.00	0.00
0.00	0.00	0.00	0.00	0.00	116.00	0.00	0.00	0.00
0.00	0.00	0.00	0.00	0.00	0.00	0.00	0.00	0.00
0.00	0.00	0.00	0.00	0.00	0.00	0.00	0.00	0.00

0.00	0.00	0.00	0.00	0.00	0.00	0.00	0.00	0.00
0.00	0.00	0.00	0.00	0.00	0.00	0.00	0.00	0.00
0.00	0.00	0.00	0.00	0.00	0.00	0.00	0.00	0.00
0.00	0.00	0.00	0.00	0.00	0.00	0.00	0.00	0.00
0.00	0.00	0.00	0.00	0.00	0.00	0.00	0.00	0.00
0.00	0.00	0.00	0.00	0.00	113.00	0.00	0.00	0.00
0.00	0.00	0.00	0.00	0.00	113.00	0.00	0.00	0.00
0.00	0.00	0.00	0.00	0.00	112.00	0.00	0.00	0.00
0.00	0.00	0.00	0.00	0.00	0.00	0.00	0.00	0.00
0.00	0.00	0.00	0.00	0.00	0.00	0.00	0.00	0.00
0.00	0.00	0.00	0.00	0.00	0.00	0.00	0.00	0.00
0.00	0.00	0.00	0.00	0.00	0.00	4.00	7.00	11.00
0.00	0.00	0.00	0.00	0.00	0.00	0.00	0.00	0.00
0.00	0.00	0.00	0.00	0.00	0.00	0.00	0.00	0.00
0.00	0.00	0.00	0.00	0.00	0.00	0.00	0.00	0.00
0.00	0.00	0.00	0.00	0.00	0.00	0.00	0.00	0.00
0.00	0.00	0.00	0.00	0.00	0.00	0.00	0.00	0.00
0.00	0.00	0.00	0.00	0.00	0.00	0.00	0.00	0.00
0.00	0.00	0.00	0.00	0.00	0.00	0.00	0.00	0.00
0.00	0.00	0.00	0.00	0.00	0.00	0.00	0.00	0.00
0.00	0.00	0.00	0.00	0.00	0.00	0.00	0.00	0.00
0.00	0.00	0.00	0.00	0.00	0.00	0.00	0.00	0.00
0.00	0.00	0.00	0.00	0.00	105.00	0.00	0.00	0.00
0.00	0.00	0.00	0.00	0.00	0.00	0.00	0.00	0.00
0.00	0.00	0.00	0.00	0.00	0.00	0.00	0.00	0.00
0.00	0.00	0.00	0.00	0.00	104.00	0.00	0.00	0.00
0.00	0.00	0.00	0.00	0.00	103.00	0.00	0.00	0.00
0.00	0.00	0.00	0.00	0.00	0.00	0.00	0.00	0.00
0.00	0.00	0.00	0.00	0.00	0.00	0.00	0.00	0.00
0.00	0.00	0.00	0.00	0.00	0.00	0.00	0.00	0.00
0.00	0.00	0.00	0.00	0.00	0.00	0.00	0.00	0.00
0.00	0.00	0.00	0.00	0.00	0.00	0.00	0.00	0.00
0.00	0.00	0.00	0.00	0.00	0.00	0.00	0.00	0.00
0.00	0.00	0.00	0.00	0.00	0.00	0.00	0.00	0.00
0.00	0.00	0.00	0.00	0.00	0.00	0.00	0.00	0.00
0.00	0.00	0.00	0.00	0.00	0.00	0.00	0.00	0.00
0.00	0.00	0.00	0.00	0.00	0.00	0.00	0.00	0.00

0.00	0.00	0.00	0.00	33.00	0.00	0.00	0.00	0.00
0.00	0.00	0.00	0.00	0.00	0.00	0.00	0.00	0.00
0.00	0.00	0.00	0.00	0.00	0.00	0.00	0.00	0.00
0.00	0.00	0.00	0.00	0.00	0.00	0.00	0.00	0.00
0.00	0.00	0.00	0.00	0.00	0.00	0.00	0.00	0.00
0.00	0.00	0.00	46.00	0.00	0.00	0.00	0.00	0.00
0.00	0.00	0.00	0.00	0.00	0.00	0.00	0.00	0.00
0.00	0.00	0.00	0.00	0.00	100.00	0.00	0.00	0.00
0.00	0.00	0.00	0.00	0.00	0.00	0.00	0.00	0.00
0.00	0.00	28.00	0.00	0.00	0.00	0.00	0.00	0.00
0.00	0.00	0.00	0.00	0.00	99.00	0.00	0.00	0.00
0.00	0.00	0.00	0.00	0.00	0.00	0.00	0.00	0.00
0.00	0.00	0.00	0.00	0.00	2.00	4.00	0.00	0.00
0.00	0.00	0.00	0.00	0.00	98.00	0.00	0.00	0.00
0.00	0.00	0.00	0.00	0.00	98.00	0.00	0.00	0.00
0.00	0.00	0.00	0.00	0.00	0.00	0.00	0.00	0.00
0.00	0.00	0.00	0.00	0.00	0.00	0.00	0.00	0.00
0.00	0.00	0.00	0.00	0.00	0.00	0.00	0.00	0.00
0.00	0.00	0.00	0.00	0.00	0.00	0.00	0.00	0.00
0.00	0.00	0.00	0.00	0.00	97.00	0.00	0.00	0.00
0.00	0.00	0.00	0.00	0.00	0.00	0.00	0.00	0.00
0.00	0.00	0.00	0.00	0.00	0.00	0.00	0.00	0.00
0.00	0.00	0.00	0.00	0.00	0.00	0.00	0.00	0.00
0.00	0.00	0.00	0.00	0.00	0.00	0.00	0.00	0.00
4.00	0.00	0.00	0.00	0.00	0.00	0.00	0.00	0.00
0.00	0.00	0.00	0.00	0.00	0.00	0.00	19.00	0.00
0.00	0.00	0.00	0.00	0.00	0.00	0.00	0.00	0.00
0.00	0.00	0.00	0.00	0.00	0.00	0.00	0.00	0.00
0.00	0.00	0.00	0.00	0.00	0.00	0.00	0.00	0.00
0.00	0.00	0.00	0.00	0.00	0.00	0.00	0.00	0.00
0.00	0.00	0.00	0.00	0.00	0.00	0.00	0.00	0.00
0.00	0.00	0.00	0.00	0.00	0.00	0.00	0.00	0.00
0.00	0.00	0.00	0.00	0.00	0.00	0.00	0.00	0.00
0.00	0.00	0.00	0.00	0.00	0.00	0.00	0.00	0.00
0.00	0.00	0.00	0.00	0.00	84.00	0.00	0.00	0.00
0.00	0.00	0.00	0.00	0.00	91.00	0.00	0.00	0.00
0.00	0.00	0.00	0.00	0.00	0.00	0.00	0.00	0.00

0.00	0.00	5.00	4.00	0.00	12.00	0.00	0.00	0.00
0.00	0.00	0.00	0.00	0.00	0.00	0.00	0.00	0.00
0.00	0.00	0.00	0.00	0.00	0.00	0.00	0.00	0.00
0.00	0.00	0.00	0.00	0.00	0.00	0.00	0.00	0.00
5.00	9.00	0.00	0.00	0.00	0.00	8.00	0.00	0.00
25.00	0.00	14.00	21.00	0.00	0.00	0.00	0.00	0.00
0.00	0.00	0.00	0.00	0.00	0.00	0.00	0.00	0.00
0.00	0.00	0.00	0.00	0.00	0.00	0.00	0.00	0.00
0.00	0.00	0.00	0.00	0.00	0.00	0.00	0.00	0.00
0.00	0.00	0.00	0.00	0.00	0.00	0.00	0.00	0.00
0.00	0.00	0.00	0.00	0.00	88.00	0.00	0.00	0.00
0.00	0.00	0.00	0.00	0.00	88.00	0.00	0.00	0.00
0.00	0.00	0.00	0.00	0.00	0.00	0.00	0.00	0.00
5.00	6.00	2.00	0.00	0.00	5.00	0.00	4.00	2.00
0.00	0.00	0.00	0.00	0.00	87.00	0.00	0.00	0.00
0.00	0.00	0.00	0.00	0.00	87.00	0.00	0.00	0.00
0.00	0.00	0.00	0.00	0.00	0.00	0.00	0.00	0.00
0.00	0.00	0.00	0.00	0.00	0.00	0.00	0.00	0.00
0.00	0.00	0.00	0.00	0.00	0.00	0.00	0.00	0.00
5.00	0.00	0.00	3.00	8.00	12.00	5.00	10.00	8.00
0.00	0.00	0.00	0.00	0.00	0.00	0.00	0.00	0.00
84.00	0.00	0.00	0.00	0.00	0.00	0.00	0.00	0.00
0.00	0.00	0.00	0.00	0.00	0.00	0.00	0.00	0.00
0.00	0.00	0.00	0.00	0.00	0.00	0.00	0.00	0.00
0.00	27.00	0.00	0.00	0.00	0.00	0.00	0.00	0.00
0.00	0.00	0.00	0.00	0.00	0.00	29.00	0.00	17.00
0.00	0.00	0.00	0.00	0.00	0.00	0.00	0.00	0.00
9.00	0.00	0.00	0.00	4.00	11.00	0.00	0.00	0.00
0.00	0.00	0.00	0.00	0.00	0.00	0.00	0.00	0.00
0.00	0.00	0.00	0.00	0.00	0.00	0.00	0.00	0.00
0.00	0.00	0.00	0.00	0.00	0.00	0.00	0.00	0.00
0.00	0.00	0.00	0.00	0.00	0.00	0.00	0.00	0.00
0.00	0.00	0.00	0.00	0.00	0.00	0.00	0.00	0.00
0.00	9.00	7.00	0.00	6.00	10.00	0.00	0.00	9.00
0.00	0.00	0.00	0.00	0.00	80.00	0.00	0.00	0.00
0.00	0.00	0.00	0.00	0.00	0.00	0.00	0.00	0.00
0.00	0.00	0.00	0.00	0.00	0.00	0.00	0.00	0.00

0.00	0.00	0.00	0.00	0.00	0.00	0.00	0.00	15.00
0.00	0.00	0.00	0.00	0.00	78.00	0.00	0.00	0.00
0.00	0.00	0.00	0.00	0.00	0.00	0.00	0.00	0.00
0.00	0.00	0.00	0.00	0.00	0.00	0.00	0.00	0.00
12.00	0.00	15.00	0.00	0.00	0.00	0.00	0.00	0.00
0.00	0.00	0.00	0.00	27.00	0.00	0.00	0.00	0.00
0.00	0.00	0.00	0.00	0.00	0.00	0.00	0.00	0.00
0.00	0.00	0.00	0.00	0.00	0.00	0.00	0.00	0.00
0.00	0.00	0.00	0.00	0.00	0.00	0.00	0.00	0.00
0.00	0.00	0.00	0.00	0.00	0.00	0.00	0.00	0.00
0.00	0.00	0.00	0.00	0.00	76.00	0.00	0.00	0.00
0.00	0.00	0.00	0.00	0.00	76.00	0.00	0.00	0.00
0.00	0.00	0.00	0.00	0.00	76.00	0.00	0.00	0.00
0.00	0.00	0.00	0.00	0.00	0.00	0.00	0.00	0.00
0.00	0.00	0.00	0.00	0.00	0.00	0.00	0.00	0.00
0.00	0.00	0.00	0.00	0.00	0.00	0.00	0.00	0.00
0.00	0.00	0.00	0.00	0.00	0.00	0.00	0.00	0.00
0.00	0.00	0.00	0.00	0.00	75.00	0.00	0.00	0.00
0.00	0.00	0.00	0.00	0.00	0.00	0.00	0.00	0.00
0.00	0.00	0.00	0.00	0.00	0.00	0.00	0.00	0.00
0.00	0.00	0.00	0.00	0.00	0.00	0.00	0.00	0.00
0.00	0.00	0.00	0.00	0.00	0.00	0.00	0.00	0.00
0.00	0.00	0.00	0.00	0.00	0.00	0.00	0.00	0.00
2.00	0.00	4.00	0.00	0.00	0.00	16.00	4.00	11.00
0.00	0.00	0.00	0.00	0.00	0.00	0.00	0.00	0.00
0.00	0.00	0.00	0.00	0.00	0.00	0.00	0.00	0.00
0.00	0.00	0.00	0.00	0.00	0.00	0.00	0.00	0.00
0.00	0.00	0.00	0.00	0.00	0.00	0.00	0.00	0.00
0.00	0.00	0.00	0.00	0.00	0.00	0.00	0.00	0.00
0.00	0.00	0.00	0.00	0.00	0.00	0.00	0.00	0.00
0.00	6.00	0.00	0.00	0.00	61.00	0.00	0.00	0.00
0.00	0.00	0.00	0.00	0.00	0.00	0.00	0.00	0.00
0.00	0.00	0.00	0.00	0.00	0.00	0.00	0.00	0.00
0.00	0.00	0.00	0.00	0.00	0.00	0.00	0.00	0.00
0.00	0.00	0.00	0.00	0.00	0.00	0.00	0.00	0.00
0.00	0.00	0.00	0.00	0.00	0.00	0.00	0.00	0.00
0.00	0.00	0.00	0.00	0.00	0.00	0.00	0.00	0.00

0.00	0.00	0.00	0.00	0.00	0.00	0.00	0.00	0.00
0.00	0.00	0.00	0.00	0.00	0.00	0.00	0.00	0.00
0.00	0.00	0.00	0.00	0.00	8.00	0.00	0.00	0.00
20.00	0.00	0.00	0.00	0.00	0.00	0.00	0.00	15.00
0.00	0.00	0.00	0.00	0.00	0.00	22.00	0.00	4.00
0.00	0.00	0.00	0.00	0.00	69.00	0.00	0.00	0.00
0.00	0.00	0.00	0.00	0.00	69.00	0.00	0.00	0.00
0.00	0.00	0.00	0.00	0.00	0.00	0.00	0.00	0.00
0.00	0.00	0.00	0.00	0.00	0.00	0.00	0.00	0.00
0.00	0.00	0.00	0.00	0.00	0.00	0.00	0.00	0.00
0.00	0.00	0.00	0.00	0.00	0.00	0.00	0.00	0.00
0.00	0.00	0.00	0.00	0.00	9.00	0.00	0.00	0.00
0.00	0.00	0.00	0.00	0.00	45.00	0.00	0.00	0.00
0.00	0.00	0.00	0.00	0.00	68.00	0.00	0.00	0.00
0.00	0.00	0.00	0.00	31.00	37.00	0.00	0.00	0.00
0.00	0.00	0.00	0.00	0.00	0.00	0.00	0.00	0.00
0.00	0.00	0.00	0.00	0.00	0.00	0.00	0.00	0.00
0.00	0.00	0.00	0.00	0.00	0.00	0.00	0.00	0.00
0.00	0.00	0.00	0.00	0.00	0.00	0.00	0.00	0.00
0.00	7.00	2.00	0.00	0.00	0.00	0.00	5.00	3.00
0.00	0.00	0.00	0.00	0.00	67.00	0.00	0.00	0.00
0.00	0.00	0.00	0.00	0.00	0.00	0.00	0.00	0.00
0.00	0.00	0.00	0.00	0.00	0.00	0.00	0.00	0.00
0.00	0.00	0.00	0.00	0.00	0.00	0.00	0.00	0.00
0.00	0.00	0.00	0.00	0.00	0.00	0.00	0.00	0.00
0.00	0.00	8.00	0.00	0.00	0.00	0.00	0.00	7.00
0.00	0.00	0.00	0.00	0.00	0.00	0.00	0.00	0.00
0.00	0.00	2.00	0.00	0.00	0.00	0.00	0.00	0.00
0.00	0.00	0.00	0.00	0.00	0.00	0.00	0.00	0.00
0.00	0.00	0.00	0.00	0.00	65.00	0.00	0.00	0.00
4.00	0.00	0.00	5.00	4.00	8.00	0.00	0.00	0.00
3.00	0.00	0.00	0.00	0.00	0.00	0.00	0.00	0.00
0.00	0.00	0.00	0.00	0.00	0.00	0.00	0.00	0.00
0.00	51.00	0.00	0.00	0.00	0.00	0.00	0.00	0.00
0.00	0.00	0.00	0.00	0.00	0.00	0.00	0.00	0.00
4.00	0.00	0.00	0.00	0.00	0.00	0.00	0.00	0.00
0.00	0.00	0.00	0.00	0.00	0.00	0.00	0.00	0.00

0.00	0.00	0.00	0.00	20.00	0.00	0.00	0.00	0.00
0.00	0.00	0.00	0.00	0.00	0.00	0.00	0.00	0.00
0.00	3.00	0.00	0.00	0.00	0.00	0.00	0.00	0.00
0.00	0.00	0.00	0.00	0.00	0.00	0.00	0.00	0.00
0.00	0.00	0.00	0.00	0.00	0.00	0.00	0.00	0.00
0.00	0.00	0.00	0.00	0.00	0.00	0.00	0.00	0.00
0.00	0.00	0.00	0.00	0.00	0.00	0.00	0.00	0.00
0.00	0.00	0.00	0.00	0.00	0.00	0.00	0.00	0.00
0.00	0.00	0.00	0.00	0.00	0.00	0.00	0.00	0.00
0.00	0.00	0.00	0.00	0.00	0.00	0.00	0.00	0.00
0.00	0.00	0.00	0.00	0.00	0.00	0.00	0.00	0.00
0.00	0.00	0.00	0.00	0.00	0.00	0.00	0.00	0.00
0.00	0.00	0.00	0.00	0.00	0.00	0.00	0.00	0.00
0.00	0.00	0.00	0.00	0.00	0.00	0.00	0.00	0.00
0.00	0.00	0.00	0.00	0.00	0.00	0.00	0.00	0.00
0.00	0.00	0.00	0.00	5.00	0.00	0.00	0.00	0.00
0.00	0.00	0.00	0.00	0.00	0.00	0.00	0.00	0.00
0.00	0.00	0.00	0.00	0.00	0.00	0.00	0.00	0.00
0.00	0.00	0.00	0.00	22.00	0.00	0.00	0.00	0.00
0.00	0.00	0.00	0.00	0.00	0.00	0.00	0.00	0.00
0.00	0.00	0.00	0.00	0.00	0.00	0.00	0.00	0.00
0.00	0.00	0.00	0.00	0.00	0.00	0.00	0.00	0.00
0.00	0.00	0.00	0.00	0.00	0.00	0.00	0.00	0.00
0.00	0.00	0.00	0.00	0.00	61.00	0.00	0.00	0.00
0.00	0.00	0.00	0.00	0.00	4.00	0.00	0.00	0.00
0.00	0.00	0.00	0.00	0.00	0.00	0.00	0.00	0.00
0.00	0.00	0.00	0.00	0.00	0.00	0.00	0.00	0.00
10.00	0.00	0.00	0.00	9.00	0.00	0.00	0.00	12.00
0.00	0.00	0.00	0.00	2.00	0.00	4.00	0.00	9.00
0.00	0.00	0.00	0.00	0.00	60.00	0.00	0.00	0.00
10.00	0.00	0.00	0.00	0.00	50.00	0.00	0.00	0.00
0.00	0.00	0.00	0.00	0.00	0.00	0.00	0.00	0.00
0.00	0.00	0.00	0.00	0.00	0.00	0.00	0.00	0.00
0.00	0.00	0.00	0.00	0.00	0.00	0.00	0.00	0.00
0.00	0.00	0.00	0.00	0.00	0.00	0.00	0.00	0.00
0.00	0.00	0.00	0.00	0.00	0.00	0.00	0.00	0.00
0.00	0.00	0.00	0.00	0.00	0.00	0.00	0.00	0.00

0.00	6.00	0.00	0.00	0.00	0.00	0.00	0.00	0.00
0.00	0.00	0.00	0.00	0.00	0.00	0.00	0.00	0.00
0.00	15.00	0.00	0.00	0.00	0.00	0.00	0.00	0.00
0.00	0.00	0.00	0.00	0.00	0.00	0.00	0.00	0.00
0.00	0.00	0.00	0.00	0.00	0.00	0.00	0.00	0.00
0.00	0.00	0.00	0.00	0.00	0.00	0.00	0.00	0.00
0.00	0.00	0.00	0.00	0.00	0.00	0.00	0.00	0.00
7.00	0.00	0.00	0.00	0.00	0.00	0.00	0.00	9.00
0.00	10.00	10.00	6.00	11.00	12.00	0.00	0.00	0.00
0.00	0.00	0.00	0.00	0.00	58.00	0.00	0.00	0.00
0.00	0.00	0.00	0.00	0.00	0.00	0.00	0.00	0.00
0.00	0.00	0.00	0.00	0.00	0.00	0.00	0.00	0.00
0.00	0.00	0.00	0.00	0.00	0.00	0.00	0.00	0.00
0.00	0.00	0.00	0.00	0.00	0.00	0.00	0.00	0.00
0.00	0.00	0.00	0.00	0.00	0.00	0.00	0.00	0.00
0.00	0.00	0.00	0.00	0.00	0.00	0.00	0.00	0.00
57.00	0.00	0.00	0.00	0.00	0.00	0.00	0.00	0.00
0.00	0.00	0.00	0.00	0.00	0.00	0.00	0.00	0.00
0.00	0.00	0.00	0.00	0.00	0.00	0.00	0.00	0.00
0.00	0.00	0.00	0.00	0.00	0.00	0.00	0.00	0.00
0.00	0.00	0.00	0.00	0.00	0.00	0.00	0.00	0.00
0.00	0.00	0.00	0.00	0.00	0.00	0.00	0.00	0.00
0.00	0.00	0.00	0.00	0.00	0.00	0.00	0.00	0.00
0.00	0.00	0.00	0.00	0.00	0.00	0.00	0.00	0.00
0.00	0.00	0.00	0.00	0.00	0.00	0.00	0.00	0.00
0.00	0.00	0.00	0.00	0.00	0.00	0.00	0.00	0.00
0.00	0.00	0.00	0.00	0.00	0.00	0.00	0.00	0.00
3.00	9.00	8.00	0.00	6.00	4.00	0.00	5.00	4.00
15.00	0.00	0.00	0.00	0.00	0.00	0.00	0.00	11.00
56.00	0.00	0.00	0.00	0.00	0.00	0.00	0.00	0.00
0.00	0.00	0.00	0.00	0.00	0.00	0.00	0.00	0.00
0.00	0.00	0.00	0.00	0.00	0.00	0.00	0.00	0.00
0.00	0.00	0.00	0.00	0.00	0.00	0.00	0.00	0.00
0.00	0.00	0.00	0.00	0.00	0.00	0.00	0.00	0.00
0.00	0.00	0.00	0.00	0.00	0.00	0.00	0.00	0.00
0.00	0.00	0.00	0.00	0.00	55.00	0.00	0.00	0.00
55.00	0.00	0.00	0.00	0.00	0.00	0.00	0.00	0.00

0.00	0.00	0.00	0.00	0.00	0.00	0.00	0.00	0.00
0.00	0.00	0.00	0.00	0.00	0.00	0.00	0.00	0.00
0.00	0.00	0.00	0.00	0.00	0.00	0.00	0.00	0.00
0.00	0.00	4.00	3.00	5.00	8.00	0.00	0.00	9.00
0.00	0.00	0.00	0.00	0.00	0.00	0.00	15.00	15.00
0.00	0.00	0.00	0.00	0.00	54.00	0.00	0.00	0.00
0.00	0.00	0.00	0.00	0.00	0.00	0.00	0.00	0.00
0.00	0.00	0.00	0.00	0.00	0.00	0.00	0.00	0.00
0.00	0.00	0.00	0.00	0.00	0.00	0.00	0.00	0.00
0.00	0.00	0.00	0.00	0.00	0.00	0.00	0.00	0.00
0.00	0.00	0.00	0.00	0.00	0.00	0.00	0.00	0.00
0.00	0.00	0.00	0.00	0.00	0.00	0.00	0.00	0.00
15.00	0.00	10.00	0.00	0.00	0.00	0.00	0.00	0.00
0.00	0.00	0.00	0.00	0.00	0.00	0.00	0.00	0.00
6.00	0.00	5.00	0.00	6.00	8.00	0.00	0.00	0.00
13.00	14.00	0.00	0.00	0.00	0.00	0.00	0.00	7.00
0.00	0.00	0.00	0.00	0.00	0.00	0.00	0.00	0.00
0.00	0.00	0.00	0.00	0.00	0.00	0.00	0.00	0.00
0.00	0.00	0.00	0.00	0.00	0.00	0.00	0.00	0.00
0.00	0.00	0.00	0.00	0.00	0.00	0.00	0.00	0.00
0.00	0.00	0.00	21.00	0.00	0.00	0.00	0.00	0.00
0.00	0.00	0.00	0.00	0.00	0.00	0.00	0.00	0.00
0.00	0.00	0.00	0.00	0.00	0.00	0.00	0.00	0.00
0.00	0.00	0.00	0.00	0.00	0.00	0.00	0.00	0.00
0.00	0.00	0.00	0.00	0.00	0.00	0.00	0.00	0.00
0.00	0.00	0.00	0.00	0.00	0.00	0.00	0.00	0.00
0.00	0.00	0.00	0.00	0.00	0.00	0.00	0.00	0.00
0.00	0.00	0.00	0.00	0.00	50.00	0.00	0.00	0.00
0.00	0.00	0.00	0.00	0.00	0.00	0.00	0.00	0.00
0.00	0.00	0.00	0.00	0.00	0.00	0.00	0.00	0.00
0.00	0.00	0.00	0.00	0.00	0.00	0.00	0.00	0.00
0.00	0.00	0.00	0.00	0.00	0.00	0.00	0.00	0.00
0.00	0.00	0.00	0.00	0.00	0.00	0.00	0.00	0.00
0.00	0.00	0.00	0.00	0.00	0.00	0.00	0.00	0.00
0.00	0.00	0.00	0.00	0.00	0.00	0.00	0.00	0.00
0.00	0.00	0.00	0.00	0.00	22.00	0.00	0.00	0.00
0.00	0.00	0.00	0.00	0.00	49.00	0.00	0.00	0.00

0.00	0.00	0.00	0.00	0.00	0.00	0.00	0.00	0.00
0.00	0.00	0.00	0.00	0.00	0.00	0.00	0.00	0.00
0.00	0.00	0.00	0.00	0.00	0.00	0.00	0.00	0.00
0.00	0.00	0.00	0.00	0.00	0.00	0.00	0.00	0.00
3.00	0.00	0.00	0.00	0.00	0.00	6.00	0.00	0.00
0.00	0.00	0.00	0.00	0.00	48.00	0.00	0.00	0.00
0.00	0.00	0.00	0.00	0.00	48.00	0.00	0.00	0.00
0.00	0.00	3.00	0.00	6.00	7.00	0.00	0.00	0.00
0.00	0.00	0.00	0.00	0.00	48.00	0.00	0.00	0.00
0.00	0.00	0.00	0.00	0.00	0.00	0.00	0.00	0.00
0.00	0.00	0.00	0.00	0.00	0.00	0.00	0.00	0.00
0.00	0.00	0.00	0.00	0.00	0.00	0.00	0.00	0.00
0.00	0.00	0.00	0.00	0.00	0.00	0.00	0.00	0.00
0.00	0.00	0.00	0.00	0.00	0.00	0.00	0.00	0.00
0.00	6.00	7.00	0.00	0.00	0.00	0.00	0.00	0.00
0.00	0.00	0.00	7.00	0.00	0.00	0.00	6.00	0.00
0.00	0.00	23.00	0.00	0.00	0.00	0.00	0.00	12.00
0.00	0.00	0.00	0.00	0.00	47.00	0.00	0.00	0.00
0.00	0.00	0.00	0.00	0.00	0.00	0.00	0.00	0.00
0.00	0.00	0.00	0.00	0.00	0.00	0.00	0.00	0.00
0.00	0.00	0.00	0.00	0.00	0.00	0.00	0.00	0.00
0.00	0.00	0.00	0.00	0.00	0.00	0.00	0.00	0.00
0.00	0.00	0.00	0.00	0.00	0.00	0.00	0.00	0.00
0.00	0.00	0.00	0.00	0.00	0.00	0.00	0.00	0.00
0.00	0.00	0.00	0.00	0.00	0.00	0.00	0.00	0.00
7.00	0.00	0.00	0.00	0.00	0.00	0.00	0.00	6.00
0.00	0.00	0.00	0.00	0.00	46.00	0.00	0.00	0.00
0.00	0.00	0.00	0.00	0.00	46.00	0.00	0.00	0.00
46.00	0.00	0.00	0.00	0.00	0.00	0.00	0.00	0.00
0.00	0.00	0.00	0.00	0.00	0.00	0.00	0.00	0.00
0.00	46.00	0.00	0.00	0.00	0.00	0.00	0.00	0.00
0.00	0.00	0.00	0.00	0.00	13.00	0.00	0.00	0.00
0.00	0.00	0.00	0.00	0.00	0.00	0.00	0.00	0.00
0.00	0.00	0.00	0.00	0.00	0.00	0.00	0.00	0.00
0.00	0.00	0.00	0.00	0.00	0.00	0.00	0.00	0.00
0.00	0.00	0.00	0.00	0.00	0.00	0.00	0.00	0.00
0.00	0.00	0.00	0.00	0.00	45.00	0.00	0.00	0.00

0.00	0.00	0.00	0.00	0.00	0.00	0.00	0.00	0.00
0.00	0.00	0.00	0.00	0.00	0.00	0.00	0.00	0.00
0.00	0.00	0.00	0.00	0.00	0.00	0.00	0.00	0.00
0.00	0.00	0.00	0.00	0.00	0.00	0.00	0.00	0.00
0.00	0.00	0.00	0.00	0.00	0.00	0.00	0.00	0.00
0.00	0.00	0.00	0.00	0.00	0.00	0.00	0.00	0.00
0.00	0.00	0.00	0.00	0.00	0.00	0.00	0.00	0.00
0.00	15.00	0.00	0.00	0.00	0.00	0.00	0.00	0.00
2.00	6.00	7.00	0.00	0.00	0.00	0.00	0.00	3.00
0.00	0.00	4.00	0.00	3.00	0.00	4.00	0.00	6.00
0.00	0.00	0.00	0.00	0.00	0.00	0.00	0.00	0.00
0.00	0.00	0.00	0.00	0.00	0.00	0.00	0.00	0.00
0.00	0.00	0.00	0.00	0.00	0.00	0.00	0.00	0.00
0.00	0.00	0.00	0.00	0.00	0.00	0.00	0.00	0.00
0.00	0.00	0.00	0.00	0.00	0.00	0.00	0.00	0.00
3.00	4.00	4.00	0.00	4.00	6.00	0.00	0.00	0.00
43.00	0.00	0.00	0.00	0.00	0.00	0.00	0.00	0.00
0.00	0.00	0.00	12.00	0.00	0.00	0.00	0.00	0.00
0.00	26.00	0.00	0.00	0.00	0.00	0.00	0.00	0.00
0.00	0.00	0.00	0.00	0.00	0.00	0.00	0.00	0.00
0.00	0.00	0.00	0.00	0.00	0.00	0.00	0.00	0.00
0.00	0.00	0.00	0.00	0.00	0.00	0.00	0.00	0.00
0.00	0.00	0.00	0.00	0.00	0.00	0.00	0.00	0.00
0.00	0.00	0.00	0.00	0.00	42.00	0.00	0.00	0.00
0.00	0.00	0.00	0.00	0.00	42.00	0.00	0.00	0.00
0.00	0.00	0.00	0.00	0.00	0.00	0.00	0.00	0.00
0.00	0.00	0.00	0.00	0.00	0.00	0.00	0.00	0.00
0.00	0.00	0.00	0.00	0.00	0.00	0.00	0.00	0.00
0.00	42.00	0.00	0.00	0.00	0.00	0.00	0.00	0.00
0.00	0.00	0.00	0.00	0.00	0.00	0.00	0.00	0.00
0.00	0.00	0.00	0.00	0.00	0.00	0.00	0.00	0.00
0.00	0.00	0.00	0.00	0.00	0.00	0.00	0.00	0.00
0.00	0.00	0.00	0.00	0.00	0.00	0.00	0.00	0.00
0.00	0.00	0.00	0.00	0.00	41.00	0.00	0.00	0.00
0.00	0.00	0.00	0.00	0.00	41.00	0.00	0.00	0.00
0.00	0.00	0.00	0.00	0.00	41.00	0.00	0.00	0.00
22.00	0.00	0.00	0.00	0.00	0.00	0.00	0.00	0.00

0.00	0.00	0.00	0.00	0.00	0.00	0.00	0.00	0.00
0.00	0.00	0.00	0.00	0.00	0.00	0.00	0.00	0.00
0.00	0.00	0.00	0.00	0.00	0.00	0.00	0.00	0.00
0.00	0.00	0.00	0.00	0.00	0.00	0.00	0.00	0.00
0.00	0.00	0.00	0.00	0.00	0.00	0.00	0.00	0.00
0.00	0.00	0.00	0.00	0.00	0.00	0.00	0.00	0.00
0.00	0.00	0.00	0.00	0.00	40.00	0.00	0.00	0.00
0.00	0.00	0.00	0.00	0.00	0.00	0.00	0.00	0.00
0.00	0.00	0.00	0.00	0.00	0.00	0.00	0.00	0.00
0.00	0.00	0.00	0.00	0.00	0.00	0.00	0.00	0.00
0.00	0.00	0.00	0.00	0.00	0.00	0.00	0.00	0.00
0.00	0.00	0.00	0.00	0.00	0.00	0.00	0.00	0.00
0.00	0.00	0.00	0.00	11.00	0.00	0.00	0.00	0.00
0.00	0.00	0.00	0.00	0.00	0.00	0.00	0.00	0.00
0.00	0.00	0.00	0.00	0.00	0.00	9.00	0.00	0.00
5.00	3.00	5.00	4.00	9.00	10.00	0.00	0.00	0.00
0.00	0.00	0.00	0.00	0.00	0.00	0.00	0.00	0.00
0.00	0.00	0.00	0.00	0.00	0.00	0.00	0.00	0.00
0.00	0.00	0.00	0.00	0.00	0.00	0.00	0.00	0.00
0.00	0.00	0.00	0.00	0.00	0.00	0.00	0.00	0.00
0.00	0.00	0.00	0.00	0.00	0.00	0.00	0.00	0.00
0.00	0.00	3.00	0.00	0.00	0.00	0.00	0.00	3.00
0.00	0.00	0.00	0.00	0.00	0.00	0.00	0.00	0.00
0.00	0.00	0.00	0.00	0.00	0.00	0.00	0.00	0.00
0.00	0.00	0.00	0.00	0.00	0.00	0.00	0.00	0.00
0.00	0.00	0.00	0.00	0.00	0.00	0.00	0.00	0.00
0.00	0.00	0.00	0.00	0.00	0.00	0.00	0.00	0.00
0.00	0.00	0.00	0.00	0.00	0.00	0.00	0.00	0.00
0.00	0.00	0.00	0.00	0.00	0.00	0.00	0.00	0.00
0.00	0.00	0.00	0.00	14.00	0.00	0.00	0.00	0.00
0.00	2.00	0.00	3.00	0.00	7.00	0.00	0.00	3.00
0.00	0.00	0.00	0.00	0.00	38.00	0.00	0.00	0.00
0.00	0.00	0.00	0.00	0.00	0.00	0.00	0.00	0.00
0.00	0.00	0.00	0.00	0.00	0.00	0.00	0.00	0.00
0.00	0.00	0.00	0.00	0.00	0.00	0.00	0.00	0.00
0.00	0.00	0.00	0.00	0.00	0.00	0.00	0.00	0.00
0.00	0.00	0.00	0.00	0.00	0.00	0.00	0.00	0.00

0.00	0.00	0.00	0.00	0.00	0.00	0.00	0.00	0.00
0.00	0.00	0.00	0.00	0.00	0.00	0.00	0.00	0.00
0.00	0.00	0.00	0.00	0.00	0.00	0.00	0.00	0.00
0.00	0.00	0.00	0.00	0.00	0.00	0.00	0.00	14.00
0.00	0.00	0.00	0.00	0.00	0.00	0.00	0.00	0.00
0.00	0.00	0.00	0.00	0.00	0.00	0.00	0.00	0.00
0.00	0.00	0.00	0.00	0.00	0.00	0.00	0.00	0.00
0.00	0.00	0.00	0.00	0.00	0.00	0.00	0.00	0.00
0.00	0.00	0.00	0.00	0.00	0.00	0.00	0.00	0.00
0.00	0.00	0.00	0.00	6.00	15.00	0.00	0.00	0.00
14.00	0.00	0.00	0.00	0.00	0.00	0.00	0.00	0.00
0.00	0.00	0.00	0.00	0.00	0.00	0.00	0.00	0.00
0.00	0.00	0.00	0.00	0.00	0.00	0.00	0.00	0.00
0.00	0.00	0.00	0.00	0.00	0.00	0.00	0.00	0.00
0.00	0.00	0.00	0.00	0.00	0.00	0.00	0.00	0.00
0.00	0.00	0.00	0.00	0.00	0.00	0.00	0.00	0.00
0.00	0.00	0.00	0.00	0.00	0.00	0.00	0.00	0.00
0.00	0.00	0.00	0.00	0.00	0.00	0.00	0.00	0.00
23.00	0.00	0.00	0.00	0.00	0.00	0.00	0.00	0.00
0.00	0.00	0.00	0.00	0.00	29.00	0.00	0.00	0.00
0.00	0.00	0.00	0.00	0.00	0.00	0.00	0.00	0.00
0.00	0.00	0.00	0.00	0.00	35.00	0.00	0.00	0.00
0.00	0.00	0.00	0.00	0.00	0.00	0.00	0.00	0.00
0.00	0.00	0.00	0.00	0.00	0.00	0.00	0.00	0.00
0.00	0.00	0.00	0.00	0.00	0.00	0.00	0.00	0.00
0.00	0.00	0.00	0.00	0.00	0.00	0.00	0.00	0.00
0.00	0.00	0.00	0.00	0.00	0.00	0.00	0.00	0.00
0.00	0.00	0.00	0.00	0.00	0.00	0.00	0.00	0.00
0.00	0.00	0.00	0.00	0.00	0.00	0.00	0.00	0.00
0.00	0.00	0.00	0.00	0.00	0.00	0.00	0.00	0.00
0.00	0.00	0.00	0.00	0.00	0.00	0.00	0.00	0.00
0.00	0.00	0.00	0.00	0.00	0.00	0.00	0.00	0.00
0.00	0.00	0.00	0.00	0.00	0.00	0.00	0.00	0.00
0.00	0.00	0.00	0.00	0.00	5.00	0.00	4.00	0.00
0.00	0.00	0.00	0.00	0.00	0.00	0.00	0.00	0.00
0.00	0.00	0.00	0.00	0.00	0.00	0.00	0.00	8.00
0.00	0.00	0.00	0.00	0.00	33.00	0.00	0.00	0.00

0.00	0.00	0.00	0.00	0.00	33.00	0.00	0.00	0.00
33.00	0.00	0.00	0.00	0.00	0.00	0.00	0.00	0.00
0.00	0.00	0.00	0.00	0.00	0.00	0.00	0.00	0.00
0.00	0.00	0.00	0.00	0.00	0.00	0.00	0.00	0.00
0.00	0.00	0.00	0.00	33.00	0.00	0.00	0.00	0.00
0.00	0.00	0.00	0.00	0.00	0.00	0.00	0.00	0.00
0.00	0.00	0.00	0.00	0.00	0.00	0.00	0.00	0.00
0.00	0.00	0.00	0.00	0.00	0.00	0.00	0.00	0.00
0.00	0.00	0.00	0.00	0.00	0.00	0.00	0.00	0.00
0.00	0.00	0.00	0.00	0.00	0.00	0.00	0.00	0.00
0.00	0.00	0.00	0.00	0.00	0.00	0.00	0.00	0.00
0.00	0.00	0.00	0.00	0.00	0.00	0.00	0.00	0.00
0.00	0.00	0.00	0.00	0.00	0.00	0.00	0.00	0.00
0.00	0.00	0.00	0.00	0.00	10.00	0.00	0.00	0.00
0.00	0.00	0.00	0.00	0.00	32.00	0.00	0.00	0.00
0.00	0.00	0.00	0.00	0.00	0.00	0.00	0.00	0.00
0.00	0.00	0.00	0.00	0.00	0.00	0.00	0.00	0.00
0.00	0.00	0.00	0.00	0.00	0.00	0.00	0.00	0.00
0.00	0.00	0.00	0.00	0.00	0.00	0.00	0.00	0.00
0.00	0.00	0.00	0.00	0.00	0.00	0.00	0.00	0.00
0.00	0.00	0.00	0.00	0.00	0.00	0.00	0.00	0.00
0.00	0.00	0.00	0.00	0.00	0.00	0.00	0.00	0.00
0.00	0.00	0.00	0.00	0.00	0.00	0.00	0.00	0.00
0.00	0.00	0.00	0.00	0.00	0.00	0.00	0.00	0.00
0.00	0.00	0.00	0.00	0.00	0.00	0.00	0.00	0.00
0.00	0.00	0.00	0.00	0.00	0.00	0.00	0.00	0.00
0.00	0.00	0.00	0.00	8.00	0.00	0.00	0.00	0.00
0.00	0.00	0.00	0.00	4.00	0.00	0.00	0.00	0.00
0.00	0.00	0.00	0.00	0.00	0.00	0.00	0.00	0.00
0.00	0.00	0.00	6.00	0.00	5.00	0.00	0.00	0.00
31.00	0.00	0.00	0.00	0.00	0.00	0.00	0.00	0.00
0.00	0.00	0.00	0.00	0.00	0.00	0.00	0.00	0.00
0.00	0.00	0.00	0.00	0.00	0.00	0.00	0.00	0.00
0.00	0.00	0.00	0.00	0.00	0.00	0.00	0.00	0.00
0.00	0.00	0.00	0.00	0.00	0.00	0.00	0.00	0.00
0.00	0.00	0.00	0.00	0.00	0.00	0.00	0.00	0.00
0.00	0.00	0.00	0.00	0.00	0.00	0.00	0.00	0.00

0.00	0.00	0.00	0.00	0.00	0.00	0.00	0.00	0.00
0.00	0.00	0.00	0.00	0.00	0.00	0.00	0.00	0.00
0.00	0.00	0.00	0.00	0.00	0.00	0.00	0.00	0.00
0.00	0.00	0.00	0.00	5.00	0.00	0.00	0.00	7.00
0.00	0.00	0.00	0.00	0.00	0.00	0.00	13.00	0.00
8.00	0.00	0.00	0.00	0.00	0.00	0.00	0.00	9.00
13.00	0.00	0.00	0.00	0.00	0.00	0.00	0.00	5.00
0.00	0.00	0.00	0.00	0.00	30.00	0.00	0.00	0.00
0.00	6.00	0.00	0.00	0.00	11.00	0.00	0.00	0.00
0.00	0.00	0.00	0.00	0.00	0.00	0.00	0.00	0.00
0.00	0.00	0.00	0.00	0.00	0.00	0.00	0.00	0.00
0.00	0.00	0.00	0.00	0.00	0.00	0.00	0.00	0.00
0.00	0.00	0.00	0.00	0.00	0.00	0.00	0.00	0.00
0.00	0.00	0.00	0.00	0.00	0.00	0.00	0.00	0.00
0.00	0.00	0.00	0.00	0.00	0.00	0.00	0.00	0.00
0.00	0.00	0.00	0.00	0.00	0.00	0.00	0.00	0.00
0.00	0.00	0.00	0.00	0.00	0.00	0.00	0.00	0.00
0.00	0.00	0.00	0.00	0.00	0.00	0.00	0.00	0.00
0.00	0.00	0.00	0.00	0.00	0.00	0.00	0.00	0.00
0.00	0.00	0.00	0.00	0.00	0.00	0.00	0.00	0.00
6.00	0.00	6.00	0.00	0.00	0.00	0.00	0.00	5.00
0.00	0.00	0.00	0.00	0.00	0.00	0.00	0.00	8.00
0.00	0.00	0.00	0.00	0.00	0.00	9.00	0.00	0.00
0.00	0.00	0.00	0.00	0.00	0.00	0.00	0.00	0.00
0.00	0.00	0.00	0.00	0.00	0.00	0.00	0.00	0.00
0.00	0.00	0.00	0.00	0.00	0.00	0.00	0.00	0.00
0.00	0.00	0.00	0.00	0.00	0.00	0.00	0.00	0.00
0.00	0.00	0.00	0.00	0.00	0.00	0.00	0.00	0.00
0.00	0.00	0.00	0.00	0.00	0.00	0.00	0.00	0.00
0.00	0.00	0.00	0.00	0.00	0.00	0.00	0.00	0.00
6.00	5.00	0.00	0.00	6.00	5.00	0.00	0.00	0.00
0.00	0.00	0.00	0.00	0.00	0.00	0.00	0.00	0.00
0.00	0.00	0.00	0.00	0.00	0.00	0.00	0.00	0.00
0.00	0.00	0.00	28.00	0.00	0.00	0.00	0.00	0.00
0.00	0.00	0.00	0.00	0.00	0.00	0.00	0.00	0.00
0.00	0.00	0.00	0.00	0.00	0.00	0.00	0.00	0.00
0.00	0.00	0.00	0.00	0.00	0.00	0.00	0.00	0.00

0.00	0.00	0.00	0.00	0.00	0.00	0.00	0.00	0.00
0.00	0.00	0.00	0.00	0.00	0.00	0.00	0.00	0.00
0.00	0.00	0.00	0.00	0.00	0.00	0.00	0.00	0.00
0.00	0.00	0.00	0.00	0.00	0.00	0.00	0.00	0.00
0.00	0.00	0.00	0.00	0.00	0.00	0.00	0.00	0.00
0.00	0.00	0.00	0.00	0.00	0.00	0.00	0.00	0.00
0.00	0.00	0.00	0.00	0.00	0.00	0.00	0.00	0.00
0.00	0.00	0.00	0.00	5.00	0.00	0.00	0.00	0.00
0.00	0.00	0.00	0.00	0.00	0.00	0.00	0.00	0.00
0.00	0.00	0.00	0.00	0.00	6.00	0.00	0.00	0.00
0.00	0.00	0.00	0.00	0.00	0.00	0.00	0.00	0.00
0.00	0.00	2.00	0.00	4.00	0.00	0.00	0.00	7.00
27.00	0.00	0.00	0.00	0.00	0.00	0.00	0.00	0.00
19.00	0.00	0.00	0.00	0.00	0.00	0.00	0.00	0.00
0.00	0.00	0.00	0.00	0.00	0.00	0.00	0.00	0.00
0.00	0.00	0.00	0.00	0.00	0.00	0.00	0.00	0.00
0.00	0.00	0.00	0.00	0.00	0.00	0.00	0.00	0.00
0.00	0.00	0.00	0.00	0.00	0.00	0.00	0.00	0.00
0.00	0.00	0.00	0.00	0.00	0.00	0.00	0.00	0.00
0.00	0.00	0.00	0.00	0.00	0.00	0.00	0.00	0.00
0.00	0.00	0.00	0.00	0.00	0.00	0.00	0.00	0.00
0.00	0.00	0.00	0.00	0.00	0.00	0.00	0.00	0.00
0.00	0.00	0.00	0.00	0.00	0.00	0.00	0.00	0.00
0.00	0.00	0.00	0.00	0.00	0.00	0.00	0.00	0.00
0.00	0.00	0.00	0.00	0.00	0.00	0.00	0.00	0.00
0.00	0.00	0.00	0.00	0.00	0.00	0.00	0.00	0.00
6.00	0.00	0.00	3.00	0.00	0.00	0.00	7.00	0.00
0.00	0.00	0.00	0.00	0.00	10.00	0.00	0.00	0.00
0.00	0.00	0.00	0.00	0.00	0.00	0.00	0.00	0.00
0.00	0.00	0.00	0.00	0.00	0.00	0.00	0.00	0.00
0.00	3.00	5.00	0.00	0.00	0.00	0.00	0.00	0.00
0.00	0.00	0.00	0.00	0.00	0.00	0.00	0.00	0.00
0.00	0.00	0.00	0.00	0.00	0.00	0.00	0.00	0.00
0.00	0.00	0.00	0.00	0.00	0.00	0.00	0.00	0.00
0.00	0.00	0.00	0.00	0.00	0.00	0.00	0.00	0.00
0.00	0.00	0.00	0.00	0.00	0.00	0.00	0.00	0.00
0.00	0.00	0.00	0.00	0.00	0.00	0.00	0.00	0.00

0.00	0.00	0.00	0.00	0.00	0.00	0.00	0.00	0.00
0.00	0.00	0.00	0.00	0.00	0.00	0.00	0.00	0.00
0.00	0.00	0.00	0.00	0.00	0.00	0.00	0.00	0.00
0.00	0.00	0.00	0.00	0.00	0.00	0.00	0.00	0.00
0.00	0.00	0.00	0.00	0.00	0.00	0.00	0.00	0.00
0.00	0.00	0.00	0.00	0.00	0.00	0.00	0.00	0.00
25.00	0.00	0.00	0.00	0.00	0.00	0.00	0.00	0.00
25.00	0.00	0.00	0.00	0.00	0.00	0.00	0.00	0.00
0.00	0.00	0.00	0.00	0.00	0.00	0.00	0.00	0.00
0.00	0.00	0.00	0.00	0.00	0.00	0.00	0.00	0.00
0.00	0.00	0.00	0.00	0.00	0.00	0.00	0.00	0.00
0.00	0.00	0.00	0.00	0.00	0.00	0.00	0.00	0.00
0.00	0.00	0.00	0.00	0.00	0.00	0.00	0.00	0.00
0.00	0.00	0.00	0.00	0.00	0.00	0.00	0.00	0.00
21.00	0.00	0.00	0.00	0.00	0.00	0.00	0.00	0.00
0.00	0.00	0.00	0.00	0.00	0.00	0.00	0.00	0.00
0.00	0.00	0.00	0.00	0.00	0.00	0.00	0.00	0.00
0.00	0.00	0.00	0.00	0.00	0.00	0.00	0.00	0.00
0.00	0.00	0.00	0.00	0.00	0.00	0.00	0.00	0.00
0.00	0.00	0.00	0.00	0.00	0.00	0.00	0.00	0.00
0.00	0.00	0.00	0.00	0.00	0.00	0.00	0.00	0.00
0.00	0.00	0.00	0.00	0.00	9.00	0.00	0.00	0.00
0.00	0.00	0.00	0.00	0.00	0.00	0.00	0.00	24.00
9.00	0.00	11.00	0.00	0.00	0.00	0.00	0.00	4.00
0.00	0.00	6.00	5.00	0.00	0.00	0.00	2.00	0.00
0.00	0.00	0.00	0.00	0.00	0.00	7.00	0.00	0.00
0.00	0.00	0.00	0.00	0.00	0.00	0.00	0.00	0.00
0.00	0.00	0.00	0.00	24.00	0.00	0.00	0.00	0.00
0.00	0.00	10.00	0.00	14.00	0.00	0.00	0.00	0.00
0.00	0.00	0.00	0.00	0.00	0.00	0.00	0.00	0.00
0.00	0.00	0.00	0.00	0.00	0.00	0.00	0.00	0.00
0.00	0.00	0.00	0.00	0.00	0.00	0.00	0.00	0.00
0.00	0.00	0.00	0.00	0.00	0.00	0.00	0.00	0.00
0.00	0.00	0.00	0.00	0.00	0.00	0.00	0.00	0.00
0.00	0.00	0.00	0.00	0.00	0.00	0.00	0.00	0.00
0.00	0.00	4.00	0.00	0.00	0.00	0.00	0.00	0.00
6.00	8.00	0.00	0.00	0.00	0.00	0.00	0.00	4.00

0.00	0.00	0.00	0.00	0.00	23.00	0.00	0.00	0.00
0.00	0.00	0.00	0.00	0.00	0.00	0.00	0.00	0.00
0.00	0.00	0.00	0.00	0.00	0.00	0.00	0.00	0.00
0.00	0.00	0.00	0.00	0.00	0.00	0.00	0.00	0.00
0.00	0.00	0.00	0.00	0.00	0.00	0.00	0.00	0.00
0.00	0.00	0.00	0.00	0.00	0.00	0.00	0.00	0.00
0.00	0.00	0.00	0.00	0.00	0.00	0.00	0.00	0.00
0.00	0.00	0.00	0.00	0.00	0.00	0.00	0.00	0.00
0.00	0.00	0.00	0.00	0.00	0.00	0.00	0.00	0.00
0.00	0.00	0.00	0.00	0.00	0.00	0.00	0.00	0.00
0.00	0.00	0.00	0.00	0.00	0.00	0.00	0.00	0.00
0.00	0.00	0.00	0.00	0.00	0.00	0.00	0.00	0.00
0.00	0.00	0.00	0.00	0.00	3.00	0.00	0.00	0.00
0.00	0.00	3.00	0.00	0.00	0.00	0.00	0.00	0.00
0.00	0.00	0.00	0.00	0.00	0.00	0.00	0.00	0.00
0.00	0.00	0.00	0.00	11.00	0.00	0.00	0.00	0.00
0.00	0.00	0.00	0.00	0.00	0.00	0.00	0.00	0.00
6.00	0.00	0.00	0.00	0.00	0.00	0.00	0.00	4.00
0.00	0.00	5.00	0.00	0.00	0.00	0.00	6.00	0.00
6.00	0.00	0.00	0.00	0.00	0.00	16.00	0.00	0.00
0.00	0.00	0.00	0.00	0.00	8.00	0.00	0.00	0.00
0.00	0.00	0.00	0.00	0.00	22.00	0.00	0.00	0.00
0.00	0.00	0.00	0.00	0.00	0.00	0.00	0.00	0.00
0.00	0.00	0.00	0.00	0.00	0.00	0.00	0.00	0.00
0.00	0.00	0.00	0.00	0.00	0.00	0.00	0.00	0.00
0.00	0.00	0.00	0.00	0.00	0.00	0.00	0.00	0.00
0.00	0.00	0.00	0.00	0.00	0.00	0.00	0.00	0.00
0.00	0.00	0.00	0.00	0.00	0.00	0.00	0.00	0.00
0.00	0.00	0.00	0.00	0.00	0.00	0.00	0.00	0.00
0.00	0.00	0.00	0.00	0.00	0.00	0.00	0.00	0.00
0.00	0.00	0.00	0.00	0.00	0.00	0.00	0.00	0.00
0.00	0.00	0.00	0.00	0.00	0.00	0.00	0.00	0.00
0.00	0.00	0.00	0.00	0.00	0.00	0.00	0.00	0.00
0.00	0.00	0.00	0.00	0.00	0.00	0.00	0.00	0.00
0.00	0.00	0.00	0.00	0.00	0.00	0.00	0.00	0.00
0.00	0.00	0.00	0.00	0.00	0.00	0.00	0.00	0.00
0.00	0.00	0.00	0.00	0.00	0.00	0.00	0.00	0.00

0.00	0.00	0.00	0.00	0.00	0.00	0.00	0.00	0.00
0.00	0.00	0.00	0.00	0.00	0.00	0.00	0.00	4.00
0.00	0.00	0.00	0.00	0.00	21.00	0.00	0.00	0.00
0.00	2.00	0.00	0.00	0.00	11.00	0.00	0.00	0.00
0.00	0.00	0.00	0.00	0.00	21.00	0.00	0.00	0.00
0.00	0.00	0.00	0.00	0.00	21.00	0.00	0.00	0.00
0.00	0.00	0.00	0.00	0.00	0.00	0.00	0.00	0.00
0.00	0.00	0.00	0.00	0.00	0.00	0.00	0.00	0.00
0.00	0.00	0.00	0.00	0.00	0.00	0.00	0.00	0.00
0.00	0.00	0.00	0.00	0.00	0.00	0.00	0.00	0.00
0.00	0.00	0.00	0.00	0.00	0.00	0.00	0.00	0.00
0.00	0.00	0.00	0.00	0.00	0.00	0.00	0.00	0.00
0.00	0.00	0.00	0.00	0.00	0.00	0.00	0.00	0.00
0.00	0.00	0.00	0.00	0.00	0.00	0.00	0.00	0.00
0.00	0.00	0.00	0.00	0.00	0.00	0.00	0.00	0.00
0.00	0.00	0.00	0.00	0.00	0.00	0.00	0.00	0.00
0.00	0.00	0.00	0.00	0.00	0.00	0.00	0.00	0.00
7.00	0.00	0.00	0.00	0.00	0.00	0.00	0.00	9.00
0.00	0.00	0.00	0.00	0.00	0.00	0.00	5.00	0.00
20.00	0.00	0.00	0.00	0.00	0.00	0.00	0.00	0.00
20.00	0.00	0.00	0.00	0.00	0.00	0.00	0.00	0.00
0.00	0.00	0.00	9.00	0.00	0.00	0.00	0.00	0.00
0.00	0.00	0.00	0.00	0.00	0.00	0.00	0.00	0.00
0.00	0.00	0.00	0.00	0.00	0.00	0.00	0.00	0.00
0.00	0.00	0.00	0.00	0.00	0.00	0.00	0.00	0.00
0.00	0.00	0.00	0.00	0.00	0.00	0.00	0.00	0.00
0.00	0.00	0.00	0.00	0.00	0.00	0.00	0.00	0.00
0.00	0.00	0.00	0.00	0.00	0.00	0.00	0.00	0.00
0.00	0.00	0.00	0.00	0.00	0.00	0.00	0.00	0.00
0.00	0.00	0.00	0.00	0.00	0.00	0.00	0.00	0.00
0.00	0.00	0.00	0.00	0.00	0.00	0.00	0.00	0.00
0.00	0.00	0.00	0.00	20.00	0.00	0.00	0.00	0.00
0.00	0.00	0.00	20.00	0.00	0.00	0.00	0.00	0.00
0.00	0.00	0.00	0.00	0.00	0.00	0.00	0.00	0.00
0.00	0.00	0.00	0.00	0.00	0.00	0.00	0.00	0.00
0.00	0.00	0.00	0.00	0.00	0.00	0.00	0.00	0.00
0.00	0.00	0.00	0.00	0.00	0.00	0.00	0.00	0.00

0.00	0.00	0.00	0.00	0.00	0.00	0.00	0.00	0.00
0.00	0.00	0.00	0.00	0.00	0.00	0.00	0.00	0.00
0.00	0.00	0.00	0.00	0.00	0.00	0.00	0.00	0.00
0.00	0.00	0.00	0.00	0.00	0.00	0.00	0.00	0.00
0.00	0.00	0.00	0.00	0.00	0.00	0.00	0.00	0.00
0.00	0.00	0.00	0.00	0.00	0.00	0.00	0.00	0.00
0.00	0.00	0.00	0.00	0.00	0.00	3.00	0.00	6.00
0.00	0.00	0.00	0.00	0.00	0.00	0.00	0.00	0.00
0.00	0.00	0.00	0.00	0.00	0.00	0.00	0.00	0.00
0.00	0.00	0.00	0.00	0.00	0.00	0.00	0.00	15.00
0.00	0.00	0.00	0.00	0.00	0.00	0.00	12.00	0.00
19.00	0.00	0.00	0.00	0.00	0.00	0.00	0.00	0.00
0.00	0.00	0.00	0.00	0.00	0.00	0.00	0.00	0.00
0.00	0.00	0.00	0.00	0.00	0.00	0.00	0.00	0.00
0.00	0.00	0.00	0.00	0.00	0.00	0.00	0.00	0.00
0.00	0.00	0.00	0.00	0.00	0.00	0.00	0.00	0.00
0.00	0.00	0.00	0.00	0.00	0.00	0.00	0.00	0.00
0.00	0.00	0.00	0.00	0.00	0.00	0.00	0.00	0.00
0.00	0.00	0.00	0.00	0.00	0.00	0.00	0.00	0.00
0.00	0.00	0.00	0.00	0.00	0.00	0.00	0.00	0.00
0.00	0.00	0.00	0.00	0.00	0.00	0.00	0.00	0.00
0.00	0.00	0.00	0.00	0.00	0.00	0.00	0.00	0.00
0.00	0.00	0.00	0.00	0.00	0.00	0.00	0.00	0.00
0.00	0.00	0.00	0.00	0.00	0.00	0.00	0.00	0.00
0.00	0.00	0.00	0.00	0.00	0.00	0.00	0.00	0.00
0.00	0.00	0.00	0.00	0.00	0.00	0.00	0.00	0.00
0.00	0.00	0.00	0.00	0.00	0.00	0.00	0.00	0.00
0.00	0.00	0.00	0.00	0.00	0.00	0.00	0.00	0.00
0.00	0.00	0.00	0.00	0.00	0.00	0.00	0.00	0.00
0.00	0.00	0.00	0.00	0.00	0.00	0.00	0.00	0.00
0.00	0.00	0.00	0.00	0.00	0.00	0.00	0.00	0.00
0.00	0.00	0.00	0.00	0.00	0.00	0.00	0.00	0.00
0.00	0.00	0.00	0.00	0.00	0.00	0.00	0.00	0.00
0.00	0.00	0.00	0.00	0.00	0.00	0.00	0.00	0.00
0.00	0.00	0.00	0.00	0.00	0.00	0.00	0.00	0.00
0.00	0.00	0.00	0.00	0.00	0.00	0.00	0.00	0.00
0.00	0.00	5.00	0.00	0.00	0.00	0.00	0.00	0.00

0.00	0.00	0.00	0.00	0.00	6.00	0.00	0.00	0.00
0.00	0.00	5.00	0.00	0.00	0.00	0.00	0.00	0.00
3.00	0.00	0.00	0.00	0.00	0.00	0.00	0.00	0.00
0.00	0.00	0.00	0.00	0.00	0.00	8.00	0.00	0.00
0.00	0.00	0.00	0.00	0.00	0.00	0.00	0.00	11.00
0.00	0.00	0.00	0.00	0.00	6.00	2.00	0.00	2.00
7.00	0.00	4.00	0.00	0.00	0.00	0.00	0.00	3.00
0.00	0.00	0.00	0.00	0.00	0.00	18.00	0.00	0.00
0.00	0.00	0.00	0.00	0.00	18.00	0.00	0.00	0.00
0.00	0.00	0.00	0.00	0.00	0.00	0.00	0.00	0.00
0.00	0.00	0.00	0.00	0.00	0.00	0.00	0.00	0.00
0.00	0.00	0.00	0.00	0.00	0.00	0.00	0.00	0.00
0.00	0.00	0.00	0.00	0.00	0.00	0.00	0.00	0.00
0.00	0.00	0.00	0.00	0.00	0.00	0.00	0.00	0.00
0.00	0.00	0.00	0.00	0.00	0.00	0.00	0.00	0.00
0.00	0.00	0.00	0.00	0.00	0.00	0.00	0.00	0.00
0.00	0.00	0.00	0.00	0.00	0.00	0.00	0.00	0.00
0.00	0.00	0.00	0.00	0.00	0.00	0.00	0.00	0.00
0.00	0.00	0.00	0.00	0.00	0.00	0.00	0.00	0.00
0.00	0.00	0.00	0.00	0.00	0.00	0.00	0.00	0.00
0.00	0.00	0.00	0.00	0.00	0.00	0.00	0.00	0.00
0.00	0.00	0.00	0.00	0.00	0.00	0.00	0.00	0.00
0.00	0.00	0.00	0.00	0.00	0.00	0.00	0.00	0.00
0.00	0.00	0.00	0.00	0.00	0.00	0.00	0.00	0.00
0.00	0.00	0.00	6.00	0.00	0.00	0.00	0.00	0.00
0.00	0.00	0.00	0.00	0.00	0.00	0.00	0.00	0.00
0.00	0.00	0.00	0.00	0.00	0.00	0.00	0.00	0.00
0.00	0.00	0.00	0.00	0.00	0.00	0.00	8.00	0.00
0.00	0.00	0.00	0.00	0.00	0.00	17.00	0.00	0.00
0.00	0.00	0.00	0.00	0.00	6.00	4.00	0.00	0.00
9.00	0.00	0.00	0.00	5.00	3.00	0.00	0.00	0.00
0.00	0.00	0.00	0.00	0.00	5.00	0.00	0.00	0.00
17.00	0.00	0.00	0.00	0.00	0.00	0.00	0.00	0.00
17.00	0.00	0.00	0.00	0.00	0.00	0.00	0.00	0.00
0.00	0.00	0.00	0.00	0.00	0.00	0.00	0.00	0.00
0.00	0.00	0.00	0.00	0.00	0.00	0.00	0.00	0.00
0.00	0.00	0.00	0.00	0.00	0.00	0.00	0.00	0.00

0.00	0.00	0.00	0.00	0.00	0.00	0.00	0.00	0.00
0.00	0.00	0.00	0.00	0.00	0.00	0.00	0.00	0.00
0.00	0.00	0.00	0.00	0.00	0.00	0.00	0.00	0.00
0.00	0.00	0.00	0.00	0.00	0.00	0.00	0.00	0.00
0.00	0.00	0.00	0.00	0.00	0.00	0.00	0.00	0.00
0.00	0.00	0.00	0.00	0.00	0.00	0.00	0.00	0.00
0.00	0.00	0.00	0.00	0.00	0.00	0.00	0.00	0.00
0.00	0.00	0.00	0.00	0.00	0.00	0.00	0.00	0.00
0.00	0.00	0.00	0.00	0.00	0.00	0.00	0.00	0.00
0.00	0.00	0.00	0.00	0.00	0.00	0.00	0.00	0.00
0.00	0.00	0.00	0.00	0.00	0.00	0.00	0.00	0.00
0.00	0.00	0.00	0.00	0.00	0.00	0.00	0.00	0.00
0.00	0.00	0.00	0.00	0.00	0.00	0.00	0.00	0.00
0.00	0.00	0.00	0.00	0.00	0.00	0.00	0.00	0.00
0.00	0.00	0.00	0.00	0.00	0.00	0.00	0.00	0.00
0.00	0.00	0.00	0.00	0.00	0.00	0.00	0.00	0.00
0.00	0.00	0.00	0.00	0.00	0.00	0.00	0.00	0.00
0.00	0.00	0.00	0.00	0.00	0.00	0.00	0.00	0.00
0.00	0.00	0.00	0.00	0.00	0.00	0.00	0.00	0.00
0.00	0.00	0.00	0.00	0.00	0.00	0.00	0.00	0.00
0.00	0.00	0.00	0.00	0.00	0.00	0.00	0.00	0.00
0.00	0.00	0.00	0.00	0.00	0.00	0.00	0.00	0.00
0.00	0.00	0.00	0.00	0.00	0.00	0.00	0.00	0.00
0.00	0.00	0.00	0.00	0.00	0.00	0.00	7.00	0.00
0.00	0.00	0.00	0.00	0.00	0.00	0.00	0.00	0.00
0.00	0.00	0.00	0.00	0.00	0.00	7.00	0.00	0.00
0.00	0.00	0.00	0.00	0.00	0.00	0.00	0.00	0.00
0.00	0.00	0.00	0.00	0.00	0.00	0.00	0.00	10.00
0.00	0.00	0.00	0.00	6.00	10.00	0.00	0.00	0.00
0.00	0.00	0.00	0.00	0.00	0.00	0.00	0.00	0.00
0.00	0.00	0.00	0.00	0.00	0.00	0.00	0.00	0.00
0.00	0.00	0.00	0.00	0.00	0.00	0.00	0.00	0.00
0.00	0.00	0.00	0.00	0.00	0.00	0.00	0.00	0.00
0.00	0.00	0.00	0.00	0.00	0.00	0.00	0.00	0.00
0.00	0.00	0.00	0.00	0.00	0.00	0.00	0.00	0.00
0.00	0.00	0.00	0.00	0.00	0.00	0.00	0.00	0.00
0.00	0.00	0.00	0.00	0.00	0.00	0.00	0.00	0.00

0.00	0.00	0.00	0.00	0.00	0.00	0.00	0.00	0.00
0.00	0.00	0.00	0.00	0.00	0.00	0.00	0.00	0.00
0.00	0.00	0.00	0.00	16.00	0.00	0.00	0.00	0.00
0.00	0.00	0.00	0.00	0.00	0.00	0.00	0.00	0.00
0.00	0.00	0.00	0.00	0.00	0.00	0.00	0.00	0.00
0.00	0.00	0.00	0.00	0.00	0.00	0.00	0.00	0.00
0.00	0.00	0.00	0.00	0.00	0.00	0.00	0.00	0.00
0.00	0.00	0.00	0.00	0.00	0.00	0.00	0.00	0.00
0.00	0.00	0.00	0.00	0.00	0.00	0.00	0.00	0.00
0.00	0.00	0.00	0.00	0.00	0.00	0.00	0.00	0.00
0.00	0.00	0.00	0.00	0.00	0.00	0.00	0.00	0.00
0.00	0.00	0.00	0.00	0.00	0.00	0.00	0.00	0.00
0.00	0.00	0.00	0.00	0.00	0.00	0.00	0.00	0.00
0.00	0.00	0.00	0.00	0.00	0.00	0.00	0.00	0.00
0.00	0.00	0.00	0.00	0.00	0.00	0.00	0.00	0.00
0.00	0.00	0.00	0.00	0.00	0.00	0.00	0.00	0.00
0.00	0.00	0.00	0.00	0.00	0.00	0.00	0.00	0.00
0.00	0.00	0.00	0.00	0.00	0.00	0.00	0.00	0.00
0.00	0.00	0.00	0.00	0.00	0.00	0.00	0.00	0.00
0.00	0.00	0.00	0.00	0.00	0.00	0.00	0.00	0.00
0.00	0.00	0.00	0.00	6.00	7.00	0.00	0.00	0.00
0.00	0.00	0.00	0.00	0.00	0.00	8.00	0.00	0.00
0.00	0.00	0.00	0.00	4.00	6.00	3.00	0.00	0.00
0.00	0.00	0.00	0.00	0.00	0.00	0.00	9.00	0.00
0.00	0.00	0.00	3.00	9.00	3.00	0.00	0.00	0.00
4.00	0.00	0.00	0.00	0.00	0.00	0.00	0.00	0.00
0.00	0.00	0.00	0.00	3.00	0.00	0.00	0.00	0.00
0.00	0.00	0.00	0.00	15.00	0.00	0.00	0.00	0.00
0.00	0.00	0.00	15.00	0.00	0.00	0.00	0.00	0.00
0.00	0.00	0.00	0.00	0.00	0.00	0.00	0.00	0.00
0.00	0.00	0.00	0.00	0.00	0.00	0.00	0.00	0.00
0.00	0.00	0.00	0.00	0.00	0.00	0.00	0.00	0.00
0.00	0.00	0.00	0.00	0.00	0.00	0.00	0.00	0.00
0.00	0.00	0.00	0.00	0.00	0.00	0.00	0.00	0.00
0.00	0.00	0.00	0.00	0.00	0.00	0.00	0.00	0.00
0.00	0.00	0.00	0.00	0.00	0.00	0.00	0.00	0.00
0.00	0.00	0.00	0.00	8.00	0.00	0.00	0.00	0.00

0.00	0.00	0.00	0.00	0.00	0.00	0.00	0.00	0.00
0.00	0.00	0.00	0.00	0.00	0.00	0.00	0.00	0.00
0.00	0.00	0.00	0.00	0.00	0.00	0.00	0.00	0.00
0.00	0.00	0.00	0.00	0.00	0.00	0.00	0.00	0.00
0.00	0.00	0.00	0.00	6.00	0.00	0.00	0.00	0.00
0.00	3.00	0.00	0.00	0.00	3.00	0.00	0.00	4.00
3.00	0.00	0.00	0.00	4.00	5.00	2.00	0.00	0.00
0.00	0.00	0.00	0.00	0.00	5.00	0.00	0.00	0.00
0.00	0.00	0.00	0.00	0.00	0.00	0.00	0.00	0.00
0.00	0.00	0.00	0.00	0.00	0.00	0.00	0.00	0.00
0.00	0.00	0.00	0.00	0.00	0.00	0.00	0.00	0.00
0.00	0.00	2.00	0.00	0.00	0.00	0.00	0.00	0.00
0.00	0.00	0.00	0.00	0.00	0.00	0.00	0.00	0.00
0.00	0.00	0.00	0.00	0.00	0.00	0.00	0.00	0.00
0.00	0.00	0.00	0.00	0.00	0.00	0.00	0.00	0.00
0.00	0.00	0.00	0.00	0.00	0.00	0.00	0.00	0.00
0.00	0.00	0.00	14.00	0.00	0.00	0.00	0.00	0.00
0.00	0.00	0.00	0.00	0.00	0.00	0.00	0.00	0.00
0.00	0.00	0.00	0.00	0.00	0.00	0.00	0.00	0.00
0.00	0.00	0.00	0.00	0.00	0.00	0.00	0.00	0.00
0.00	0.00	0.00	0.00	0.00	0.00	0.00	0.00	0.00
0.00	0.00	0.00	0.00	0.00	0.00	0.00	0.00	0.00
0.00	0.00	0.00	0.00	0.00	0.00	0.00	0.00	0.00
0.00	0.00	0.00	0.00	0.00	0.00	0.00	0.00	0.00
0.00	0.00	0.00	0.00	0.00	0.00	0.00	0.00	0.00
0.00	0.00	0.00	0.00	0.00	0.00	0.00	0.00	0.00
0.00	0.00	0.00	0.00	0.00	0.00	0.00	0.00	0.00
0.00	0.00	0.00	0.00	0.00	0.00	0.00	0.00	0.00
0.00	0.00	0.00	0.00	0.00	0.00	0.00	0.00	0.00
0.00	0.00	0.00	0.00	0.00	0.00	0.00	0.00	0.00
0.00	0.00	0.00	0.00	0.00	0.00	0.00	0.00	0.00
0.00	0.00	0.00	0.00	0.00	0.00	0.00	0.00	0.00
0.00	0.00	0.00	0.00	0.00	0.00	0.00	0.00	0.00
0.00	0.00	0.00	0.00	0.00	0.00	0.00	0.00	0.00
0.00	0.00	0.00	0.00	0.00	0.00	0.00	0.00	0.00
0.00	0.00	0.00	0.00	0.00	0.00	0.00	0.00	0.00
0.00	0.00	0.00	0.00	0.00	0.00	0.00	0.00	0.00
0.00	0.00	0.00	0.00	0.00	0.00	0.00	0.00	0.00

0.00	0.00	0.00	0.00	0.00	0.00	0.00	0.00	0.00
0.00	0.00	0.00	0.00	0.00	0.00	0.00	0.00	0.00
0.00	0.00	0.00	0.00	0.00	0.00	0.00	6.00	0.00
0.00	0.00	0.00	0.00	0.00	0.00	0.00	0.00	0.00
0.00	0.00	0.00	0.00	0.00	0.00	0.00	0.00	7.00
0.00	0.00	0.00	0.00	4.00	6.00	0.00	0.00	3.00
6.00	0.00	0.00	0.00	0.00	0.00	0.00	0.00	2.00
0.00	0.00	0.00	0.00	0.00	0.00	0.00	0.00	7.00
0.00	0.00	0.00	0.00	0.00	13.00	0.00	0.00	0.00
0.00	0.00	0.00	0.00	0.00	0.00	0.00	0.00	0.00
0.00	0.00	0.00	0.00	0.00	0.00	0.00	0.00	0.00
0.00	0.00	0.00	0.00	0.00	0.00	0.00	0.00	0.00
0.00	0.00	0.00	0.00	0.00	0.00	0.00	0.00	0.00
0.00	0.00	13.00	0.00	0.00	0.00	0.00	0.00	0.00
0.00	0.00	0.00	0.00	0.00	0.00	0.00	0.00	0.00
0.00	0.00	0.00	0.00	0.00	0.00	0.00	0.00	0.00
0.00	0.00	0.00	0.00	0.00	0.00	0.00	0.00	0.00
0.00	0.00	0.00	0.00	0.00	0.00	0.00	0.00	0.00
0.00	0.00	0.00	0.00	0.00	0.00	0.00	0.00	0.00
0.00	0.00	0.00	0.00	0.00	0.00	0.00	0.00	0.00
0.00	0.00	0.00	0.00	0.00	0.00	0.00	0.00	0.00
0.00	0.00	0.00	0.00	0.00	0.00	0.00	0.00	0.00
0.00	0.00	0.00	0.00	0.00	0.00	0.00	0.00	0.00
0.00	0.00	0.00	0.00	0.00	0.00	0.00	0.00	0.00
0.00	0.00	0.00	0.00	0.00	0.00	0.00	0.00	0.00
0.00	0.00	0.00	0.00	0.00	0.00	0.00	0.00	0.00
0.00	0.00	0.00	0.00	0.00	0.00	0.00	0.00	0.00
0.00	0.00	0.00	0.00	0.00	0.00	0.00	0.00	0.00
0.00	0.00	0.00	0.00	0.00	0.00	0.00	0.00	0.00
0.00	0.00	0.00	0.00	0.00	0.00	0.00	0.00	0.00
0.00	0.00	0.00	0.00	0.00	0.00	0.00	0.00	0.00
0.00	0.00	0.00	0.00	0.00	0.00	0.00	0.00	0.00
0.00	0.00	0.00	0.00	0.00	0.00	0.00	0.00	0.00
0.00	0.00	0.00	0.00	0.00	0.00	0.00	0.00	0.00
0.00	0.00	0.00	0.00	0.00	0.00	0.00	0.00	0.00
0.00	0.00	0.00	0.00	0.00	0.00	0.00	0.00	0.00
0.00	0.00	0.00	0.00	0.00	0.00	0.00	0.00	0.00

0.00	0.00	0.00	0.00	0.00	0.00	0.00	0.00	0.00
0.00	0.00	0.00	0.00	0.00	0.00	0.00	0.00	0.00
0.00	0.00	0.00	0.00	0.00	0.00	0.00	0.00	0.00
0.00	0.00	0.00	0.00	0.00	0.00	0.00	0.00	0.00
0.00	0.00	0.00	0.00	0.00	0.00	0.00	0.00	0.00
0.00	0.00	0.00	0.00	0.00	0.00	0.00	0.00	0.00
0.00	0.00	0.00	0.00	0.00	0.00	0.00	0.00	0.00
0.00	0.00	0.00	0.00	0.00	0.00	0.00	0.00	0.00
0.00	0.00	0.00	0.00	0.00	0.00	0.00	0.00	0.00
0.00	0.00	0.00	0.00	0.00	0.00	0.00	0.00	0.00
0.00	0.00	0.00	0.00	0.00	0.00	0.00	0.00	0.00
0.00	0.00	0.00	0.00	0.00	0.00	0.00	0.00	12.00
4.00	0.00	0.00	0.00	0.00	0.00	4.00	0.00	4.00
0.00	0.00	0.00	0.00	0.00	12.00	0.00	0.00	0.00
0.00	0.00	0.00	0.00	0.00	12.00	0.00	0.00	0.00
12.00	0.00	0.00	0.00	0.00	0.00	0.00	0.00	0.00
0.00	0.00	0.00	4.00	0.00	0.00	0.00	0.00	0.00
0.00	0.00	0.00	0.00	0.00	0.00	0.00	0.00	0.00
0.00	0.00	0.00	0.00	0.00	0.00	0.00	0.00	0.00
0.00	0.00	0.00	0.00	0.00	0.00	0.00	0.00	0.00
0.00	0.00	0.00	0.00	0.00	0.00	0.00	0.00	0.00
0.00	0.00	0.00	12.00	0.00	0.00	0.00	0.00	0.00
0.00	0.00	12.00	0.00	0.00	0.00	0.00	0.00	0.00
0.00	0.00	0.00	0.00	0.00	0.00	0.00	0.00	0.00
0.00	0.00	0.00	0.00	0.00	0.00	0.00	0.00	0.00
0.00	0.00	0.00	0.00	0.00	0.00	0.00	0.00	0.00
0.00	0.00	0.00	0.00	0.00	0.00	0.00	0.00	0.00
0.00	0.00	0.00	0.00	0.00	0.00	0.00	0.00	0.00
0.00	0.00	0.00	0.00	0.00	0.00	0.00	0.00	0.00
0.00	0.00	0.00	0.00	0.00	0.00	0.00	0.00	0.00
0.00	0.00	0.00	0.00	0.00	0.00	0.00	0.00	0.00
0.00	0.00	0.00	0.00	0.00	0.00	0.00	0.00	0.00
0.00	0.00	0.00	0.00	0.00	0.00	0.00	0.00	0.00
0.00	0.00	0.00	0.00	0.00	0.00	0.00	0.00	0.00
0.00	0.00	0.00	0.00	0.00	0.00	0.00	0.00	0.00
0.00	0.00	0.00	0.00	0.00	0.00	0.00	0.00	0.00
0.00	0.00	0.00	0.00	0.00	0.00	0.00	0.00	0.00

0.00	0.00	0.00	0.00	0.00	0.00	0.00	0.00	0.00
0.00	0.00	0.00	0.00	0.00	0.00	0.00	0.00	0.00
0.00	0.00	0.00	0.00	0.00	0.00	0.00	0.00	0.00
0.00	0.00	0.00	0.00	0.00	0.00	0.00	0.00	0.00
0.00	0.00	0.00	0.00	0.00	0.00	0.00	0.00	0.00
0.00	0.00	0.00	0.00	0.00	0.00	0.00	0.00	0.00
6.00	0.00	0.00	0.00	0.00	0.00	0.00	0.00	0.00
0.00	0.00	0.00	0.00	0.00	0.00	0.00	0.00	11.00
0.00	0.00	0.00	0.00	0.00	0.00	0.00	0.00	11.00
0.00	0.00	0.00	0.00	0.00	0.00	5.00	0.00	0.00
0.00	0.00	0.00	0.00	0.00	11.00	0.00	0.00	0.00
7.00	0.00	0.00	0.00	0.00	0.00	0.00	0.00	0.00
0.00	0.00	0.00	0.00	0.00	0.00	0.00	0.00	0.00
0.00	0.00	0.00	0.00	0.00	0.00	0.00	0.00	0.00
0.00	0.00	0.00	0.00	0.00	0.00	0.00	0.00	0.00
0.00	0.00	0.00	0.00	0.00	0.00	0.00	0.00	0.00
0.00	0.00	0.00	0.00	0.00	0.00	0.00	0.00	0.00
0.00	0.00	0.00	0.00	0.00	0.00	0.00	0.00	0.00
0.00	0.00	0.00	0.00	0.00	0.00	0.00	0.00	0.00
0.00	0.00	0.00	0.00	0.00	0.00	0.00	0.00	0.00
0.00	0.00	0.00	0.00	0.00	0.00	0.00	0.00	0.00
0.00	0.00	0.00	11.00	0.00	0.00	0.00	0.00	0.00
0.00	0.00	0.00	11.00	0.00	0.00	0.00	0.00	0.00
0.00	0.00	5.00	0.00	0.00	0.00	0.00	0.00	0.00
0.00	0.00	11.00	0.00	0.00	0.00	0.00	0.00	0.00
0.00	0.00	11.00	0.00	0.00	0.00	0.00	0.00	0.00
0.00	0.00	0.00	0.00	0.00	0.00	0.00	0.00	0.00
0.00	0.00	0.00	0.00	0.00	0.00	0.00	0.00	0.00
0.00	0.00	0.00	0.00	0.00	0.00	0.00	0.00	0.00
0.00	0.00	0.00	0.00	0.00	0.00	0.00	0.00	0.00
0.00	0.00	0.00	0.00	0.00	0.00	0.00	0.00	0.00
0.00	0.00	0.00	0.00	0.00	0.00	0.00	0.00	0.00
0.00	0.00	0.00	0.00	0.00	0.00	0.00	0.00	0.00
0.00	0.00	0.00	0.00	0.00	0.00	0.00	0.00	0.00
0.00	0.00	0.00	0.00	0.00	0.00	0.00	0.00	0.00
0.00	0.00	0.00	0.00	0.00	0.00	0.00	0.00	0.00
0.00	0.00	0.00	0.00	0.00	0.00	0.00	0.00	0.00

0.00	0.00	0.00	0.00	0.00	0.00	0.00	0.00	0.00
0.00	0.00	0.00	0.00	0.00	0.00	0.00	0.00	0.00
0.00	0.00	0.00	0.00	0.00	0.00	0.00	0.00	0.00
0.00	0.00	0.00	0.00	0.00	0.00	0.00	0.00	0.00
0.00	0.00	0.00	0.00	0.00	0.00	0.00	0.00	0.00
0.00	0.00	0.00	0.00	0.00	0.00	0.00	0.00	0.00
0.00	0.00	0.00	0.00	0.00	0.00	0.00	0.00	0.00
0.00	0.00	0.00	0.00	0.00	0.00	0.00	0.00	0.00
0.00	0.00	0.00	0.00	0.00	0.00	0.00	0.00	0.00
0.00	0.00	0.00	0.00	0.00	0.00	0.00	0.00	0.00
0.00	0.00	0.00	0.00	0.00	0.00	0.00	0.00	0.00
0.00	0.00	0.00	0.00	0.00	0.00	0.00	0.00	0.00
0.00	0.00	0.00	0.00	0.00	0.00	0.00	0.00	0.00
0.00	0.00	0.00	0.00	4.00	0.00	0.00	0.00	0.00
0.00	0.00	0.00	0.00	0.00	2.00	0.00	0.00	0.00
0.00	0.00	0.00	0.00	0.00	0.00	0.00	0.00	0.00
0.00	0.00	0.00	0.00	0.00	0.00	0.00	0.00	0.00
0.00	0.00	0.00	0.00	0.00	0.00	0.00	0.00	0.00
0.00	0.00	0.00	0.00	0.00	0.00	0.00	0.00	0.00
0.00	0.00	0.00	0.00	0.00	0.00	0.00	0.00	0.00
0.00	0.00	0.00	0.00	0.00	0.00	0.00	0.00	5.00
0.00	0.00	0.00	0.00	0.00	0.00	0.00	0.00	10.00
0.00	0.00	0.00	0.00	0.00	0.00	0.00	0.00	10.00
0.00	0.00	0.00	0.00	2.00	3.00	0.00	0.00	5.00
3.00	0.00	0.00	0.00	0.00	0.00	0.00	0.00	3.00
0.00	0.00	0.00	0.00	0.00	0.00	0.00	0.00	6.00
0.00	0.00	0.00	0.00	0.00	0.00	0.00	0.00	10.00
0.00	0.00	0.00	0.00	0.00	0.00	0.00	4.00	0.00
0.00	0.00	0.00	0.00	0.00	10.00	0.00	0.00	0.00
0.00	0.00	0.00	0.00	4.00	6.00	0.00	0.00	0.00
3.00	0.00	0.00	0.00	0.00	7.00	0.00	0.00	0.00
0.00	0.00	0.00	0.00	0.00	5.00	0.00	0.00	0.00
0.00	0.00	0.00	0.00	0.00	10.00	0.00	0.00	0.00
10.00	0.00	0.00	0.00	0.00	0.00	0.00	0.00	0.00
10.00	0.00	0.00	0.00	0.00	0.00	0.00	0.00	0.00
10.00	0.00	0.00	0.00	0.00	0.00	0.00	0.00	0.00
0.00	0.00	0.00	0.00	0.00	0.00	0.00	0.00	0.00

0.00	0.00	0.00	0.00	0.00	0.00	0.00	0.00	0.00
0.00	0.00	0.00	0.00	0.00	0.00	0.00	0.00	0.00
0.00	0.00	0.00	0.00	0.00	0.00	0.00	0.00	0.00
0.00	0.00	0.00	0.00	0.00	0.00	0.00	0.00	0.00
0.00	0.00	0.00	0.00	0.00	0.00	0.00	0.00	0.00
0.00	0.00	0.00	0.00	0.00	0.00	0.00	0.00	0.00
0.00	0.00	0.00	0.00	0.00	0.00	0.00	0.00	0.00
0.00	0.00	0.00	0.00	0.00	0.00	0.00	0.00	0.00
0.00	0.00	0.00	0.00	0.00	0.00	0.00	0.00	0.00
0.00	0.00	0.00	0.00	0.00	0.00	0.00	0.00	0.00
0.00	0.00	0.00	0.00	0.00	0.00	0.00	0.00	0.00
0.00	0.00	0.00	0.00	0.00	0.00	0.00	0.00	0.00
0.00	0.00	0.00	0.00	0.00	0.00	0.00	0.00	0.00
0.00	0.00	0.00	0.00	0.00	0.00	0.00	0.00	0.00
0.00	0.00	0.00	0.00	0.00	0.00	0.00	0.00	0.00
0.00	0.00	0.00	0.00	0.00	0.00	0.00	0.00	0.00
0.00	0.00	0.00	0.00	0.00	0.00	0.00	0.00	0.00
0.00	0.00	0.00	0.00	0.00	0.00	0.00	0.00	0.00
0.00	0.00	0.00	0.00	0.00	0.00	0.00	0.00	0.00
0.00	0.00	0.00	0.00	0.00	0.00	0.00	0.00	0.00
0.00	0.00	0.00	0.00	0.00	0.00	0.00	0.00	0.00
0.00	0.00	0.00	0.00	0.00	0.00	0.00	0.00	0.00
0.00	0.00	0.00	0.00	0.00	0.00	0.00	0.00	0.00
0.00	0.00	0.00	0.00	0.00	0.00	0.00	0.00	0.00
0.00	0.00	0.00	0.00	0.00	0.00	0.00	0.00	0.00
0.00	0.00	0.00	0.00	0.00	0.00	0.00	0.00	0.00
0.00	0.00	0.00	0.00	0.00	0.00	0.00	0.00	0.00
0.00	0.00	0.00	0.00	0.00	0.00	0.00	0.00	0.00
0.00	0.00	0.00	0.00	0.00	0.00	0.00	0.00	0.00
0.00	0.00	0.00	0.00	0.00	0.00	0.00	0.00	0.00
0.00	0.00	0.00	0.00	0.00	0.00	0.00	0.00	0.00
0.00	0.00	0.00	0.00	0.00	0.00	0.00	0.00	0.00
0.00	0.00	0.00	0.00	0.00	0.00	0.00	0.00	0.00
0.00	0.00	0.00	0.00	0.00	0.00	0.00	0.00	0.00
0.00	0.00	0.00	0.00	0.00	0.00	0.00	0.00	0.00
0.00	0.00	0.00	0.00	0.00	0.00	0.00	0.00	0.00
0.00	0.00	0.00	0.00	0.00	0.00	0.00	0.00	0.00

0.00	0.00	0.00	0.00	0.00	0.00	0.00	0.00	0.00
0.00	0.00	0.00	0.00	0.00	0.00	0.00	0.00	0.00
0.00	0.00	0.00	0.00	0.00	0.00	0.00	0.00	0.00
0.00	0.00	0.00	0.00	0.00	0.00	0.00	0.00	0.00
0.00	0.00	0.00	0.00	0.00	0.00	0.00	0.00	0.00
0.00	0.00	0.00	0.00	0.00	0.00	4.00	0.00	0.00
0.00	0.00	0.00	0.00	3.00	4.00	0.00	0.00	0.00
0.00	0.00	0.00	0.00	0.00	0.00	0.00	0.00	4.00
0.00	0.00	0.00	6.00	0.00	0.00	0.00	0.00	3.00
0.00	0.00	0.00	0.00	0.00	0.00	0.00	0.00	9.00
0.00	0.00	0.00	0.00	0.00	0.00	9.00	0.00	0.00
0.00	0.00	0.00	0.00	0.00	9.00	0.00	0.00	0.00
9.00	0.00	0.00	0.00	0.00	0.00	0.00	0.00	0.00
4.00	0.00	0.00	0.00	0.00	0.00	0.00	0.00	0.00
0.00	0.00	0.00	5.00	0.00	0.00	0.00	0.00	0.00
0.00	0.00	0.00	0.00	0.00	0.00	0.00	0.00	0.00
0.00	0.00	0.00	0.00	0.00	0.00	0.00	0.00	0.00
0.00	0.00	0.00	0.00	0.00	0.00	0.00	0.00	0.00
0.00	0.00	0.00	0.00	0.00	0.00	0.00	0.00	0.00
0.00	0.00	0.00	0.00	0.00	0.00	0.00	0.00	0.00
0.00	0.00	0.00	0.00	0.00	0.00	0.00	0.00	0.00
0.00	0.00	0.00	0.00	0.00	0.00	0.00	0.00	0.00
0.00	0.00	0.00	0.00	0.00	0.00	0.00	0.00	0.00
0.00	0.00	3.00	0.00	0.00	0.00	0.00	0.00	0.00
0.00	0.00	0.00	0.00	0.00	0.00	0.00	0.00	0.00
0.00	0.00	0.00	0.00	0.00	0.00	0.00	0.00	0.00
0.00	0.00	0.00	0.00	0.00	0.00	0.00	0.00	0.00
0.00	0.00	0.00	0.00	0.00	0.00	0.00	0.00	0.00
0.00	0.00	0.00	0.00	0.00	0.00	0.00	0.00	0.00
0.00	0.00	0.00	0.00	0.00	0.00	0.00	0.00	0.00
0.00	0.00	0.00	0.00	0.00	0.00	0.00	0.00	0.00
0.00	0.00	0.00	0.00	0.00	0.00	0.00	0.00	0.00
0.00	0.00	0.00	0.00	0.00	0.00	0.00	0.00	0.00
0.00	0.00	0.00	0.00	0.00	0.00	0.00	0.00	0.00
0.00	0.00	0.00	0.00	0.00	0.00	0.00	0.00	0.00
0.00	0.00	0.00	0.00	0.00	0.00	0.00	0.00	0.00
0.00	0.00	0.00	0.00	0.00	0.00	0.00	0.00	0.00

0.00	0.00	0.00	0.00	0.00	0.00	0.00	0.00	0.00
0.00	0.00	0.00	0.00	0.00	0.00	0.00	0.00	0.00
0.00	0.00	0.00	0.00	0.00	0.00	0.00	0.00	0.00
0.00	0.00	0.00	0.00	0.00	0.00	0.00	0.00	0.00
0.00	0.00	0.00	0.00	0.00	0.00	0.00	0.00	0.00
0.00	0.00	0.00	0.00	0.00	0.00	0.00	0.00	0.00
0.00	0.00	0.00	0.00	0.00	0.00	0.00	0.00	0.00
0.00	0.00	0.00	0.00	0.00	0.00	0.00	0.00	0.00
0.00	0.00	0.00	0.00	0.00	0.00	0.00	0.00	0.00
0.00	0.00	0.00	0.00	0.00	0.00	0.00	0.00	0.00
0.00	0.00	0.00	0.00	0.00	0.00	0.00	0.00	0.00
0.00	0.00	0.00	0.00	0.00	0.00	0.00	0.00	0.00
0.00	0.00	0.00	0.00	0.00	0.00	0.00	0.00	0.00
0.00	0.00	0.00	0.00	0.00	0.00	0.00	0.00	0.00
0.00	0.00	0.00	0.00	0.00	0.00	0.00	0.00	0.00
0.00	0.00	0.00	0.00	0.00	6.00	0.00	0.00	0.00
0.00	0.00	0.00	0.00	0.00	0.00	0.00	0.00	0.00
0.00	0.00	0.00	0.00	0.00	0.00	0.00	0.00	0.00
0.00	0.00	0.00	0.00	0.00	0.00	0.00	0.00	0.00
0.00	0.00	0.00	2.00	0.00	0.00	0.00	0.00	0.00
0.00	0.00	0.00	0.00	0.00	0.00	0.00	0.00	0.00
0.00	0.00	0.00	0.00	0.00	0.00	0.00	0.00	4.00
4.00	0.00	0.00	0.00	0.00	0.00	0.00	0.00	4.00
0.00	0.00	0.00	0.00	0.00	0.00	0.00	0.00	2.00
0.00	0.00	0.00	0.00	0.00	0.00	0.00	0.00	8.00
0.00	0.00	0.00	0.00	0.00	3.00	2.00	0.00	0.00
2.00	0.00	0.00	0.00	0.00	0.00	0.00	0.00	0.00
8.00	0.00	0.00	0.00	0.00	0.00	0.00	0.00	0.00
0.00	0.00	0.00	3.00	0.00	0.00	0.00	0.00	0.00
0.00	0.00	0.00	0.00	0.00	0.00	0.00	0.00	0.00
0.00	0.00	0.00	0.00	0.00	0.00	0.00	0.00	0.00
0.00	0.00	0.00	0.00	0.00	0.00	0.00	0.00	0.00
0.00	0.00	0.00	0.00	0.00	0.00	0.00	0.00	0.00
0.00	0.00	0.00	8.00	0.00	0.00	0.00	0.00	0.00
0.00	0.00	0.00	0.00	0.00	0.00	0.00	0.00	0.00
0.00	0.00	0.00	0.00	0.00	0.00	0.00	0.00	0.00
0.00	0.00	0.00	0.00	0.00	0.00	0.00	0.00	0.00

0.00	0.00	0.00	0.00	0.00	0.00	0.00	0.00	0.00
0.00	0.00	0.00	0.00	0.00	0.00	0.00	0.00	0.00
0.00	0.00	0.00	0.00	0.00	0.00	0.00	0.00	0.00
0.00	0.00	0.00	0.00	0.00	0.00	0.00	0.00	0.00
0.00	0.00	0.00	0.00	0.00	0.00	0.00	0.00	0.00
0.00	0.00	0.00	0.00	0.00	0.00	0.00	0.00	0.00
0.00	0.00	0.00	0.00	0.00	0.00	0.00	0.00	0.00
0.00	0.00	0.00	0.00	0.00	0.00	0.00	0.00	0.00
0.00	0.00	0.00	0.00	0.00	0.00	0.00	0.00	0.00
0.00	0.00	0.00	0.00	0.00	0.00	0.00	0.00	0.00
0.00	0.00	0.00	0.00	0.00	0.00	0.00	0.00	0.00
0.00	0.00	0.00	0.00	0.00	0.00	0.00	0.00	0.00
0.00	0.00	0.00	0.00	0.00	0.00	0.00	0.00	0.00
0.00	0.00	0.00	0.00	0.00	0.00	0.00	0.00	0.00
0.00	0.00	0.00	0.00	0.00	0.00	0.00	0.00	0.00
0.00	0.00	0.00	0.00	0.00	0.00	0.00	0.00	0.00
0.00	0.00	0.00	0.00	0.00	0.00	0.00	0.00	0.00
0.00	0.00	0.00	0.00	0.00	0.00	0.00	0.00	0.00
0.00	0.00	0.00	0.00	0.00	0.00	0.00	0.00	0.00
0.00	0.00	0.00	0.00	0.00	0.00	0.00	0.00	0.00
0.00	0.00	0.00	0.00	0.00	0.00	0.00	0.00	0.00
0.00	0.00	0.00	0.00	0.00	0.00	0.00	0.00	0.00
0.00	0.00	0.00	0.00	0.00	0.00	0.00	0.00	0.00
0.00	0.00	0.00	0.00	0.00	0.00	0.00	0.00	0.00
0.00	0.00	0.00	0.00	0.00	0.00	0.00	0.00	0.00
0.00	0.00	0.00	0.00	0.00	0.00	0.00	0.00	0.00
0.00	0.00	0.00	0.00	0.00	0.00	0.00	0.00	0.00
0.00	0.00	0.00	0.00	0.00	0.00	0.00	0.00	0.00
0.00	0.00	0.00	0.00	0.00	0.00	0.00	0.00	0.00
0.00	0.00	0.00	0.00	0.00	0.00	0.00	0.00	0.00
0.00	0.00	0.00	0.00	0.00	0.00	0.00	0.00	0.00
0.00	0.00	0.00	0.00	0.00	0.00	0.00	0.00	0.00
0.00	0.00	0.00	4.00	0.00	0.00	0.00	0.00	0.00
0.00	0.00	0.00	0.00	0.00	0.00	0.00	0.00	0.00
0.00	0.00	0.00	0.00	0.00	0.00	0.00	0.00	0.00
0.00	0.00	0.00	0.00	0.00	0.00	0.00	0.00	0.00
0.00	0.00	0.00	2.00	0.00	0.00	0.00	0.00	0.00

0.00	0.00	0.00	0.00	0.00	0.00	0.00	0.00	0.00
0.00	0.00	0.00	0.00	0.00	0.00	0.00	0.00	0.00
0.00	0.00	0.00	0.00	0.00	0.00	0.00	0.00	0.00
0.00	0.00	0.00	0.00	0.00	0.00	0.00	0.00	3.00
0.00	0.00	0.00	0.00	0.00	0.00	0.00	0.00	7.00
0.00	0.00	0.00	0.00	0.00	0.00	0.00	0.00	7.00
0.00	0.00	3.00	2.00	0.00	0.00	0.00	0.00	2.00
7.00	0.00	0.00	0.00	0.00	0.00	0.00	0.00	0.00
0.00	0.00	0.00	0.00	0.00	0.00	0.00	0.00	0.00
0.00	0.00	0.00	0.00	0.00	0.00	0.00	0.00	0.00
0.00	0.00	0.00	0.00	0.00	0.00	0.00	0.00	0.00
0.00	0.00	0.00	0.00	0.00	0.00	0.00	0.00	0.00
0.00	0.00	0.00	0.00	0.00	0.00	0.00	0.00	0.00
0.00	0.00	0.00	0.00	0.00	0.00	0.00	0.00	0.00
0.00	0.00	0.00	0.00	0.00	0.00	0.00	0.00	0.00
0.00	0.00	0.00	0.00	0.00	0.00	0.00	0.00	0.00
0.00	0.00	0.00	7.00	0.00	0.00	0.00	0.00	0.00
0.00	0.00	7.00	0.00	0.00	0.00	0.00	0.00	0.00
0.00	0.00	0.00	0.00	0.00	0.00	0.00	0.00	0.00
0.00	0.00	0.00	0.00	0.00	0.00	0.00	0.00	0.00
0.00	0.00	0.00	0.00	0.00	0.00	0.00	0.00	0.00
0.00	0.00	0.00	0.00	0.00	0.00	0.00	0.00	0.00
0.00	0.00	0.00	0.00	0.00	0.00	0.00	0.00	0.00
0.00	0.00	0.00	0.00	0.00	0.00	0.00	0.00	0.00
0.00	0.00	0.00	0.00	0.00	0.00	0.00	0.00	0.00
0.00	0.00	0.00	0.00	0.00	0.00	0.00	0.00	0.00
0.00	0.00	0.00	0.00	0.00	0.00	0.00	0.00	0.00
0.00	0.00	0.00	0.00	0.00	0.00	0.00	0.00	0.00
0.00	0.00	0.00	0.00	0.00	0.00	0.00	0.00	0.00
0.00	0.00	0.00	0.00	0.00	0.00	0.00	0.00	0.00
0.00	0.00	0.00	0.00	0.00	0.00	0.00	0.00	0.00
0.00	0.00	0.00	0.00	0.00	0.00	0.00	0.00	0.00
0.00	0.00	0.00	0.00	0.00	0.00	0.00	0.00	0.00
0.00	0.00	0.00	0.00	0.00	0.00	0.00	0.00	0.00
0.00	0.00	0.00	0.00	0.00	0.00	0.00	0.00	0.00
0.00	0.00	0.00	0.00	0.00	0.00	0.00	0.00	0.00
0.00	0.00	0.00	0.00	0.00	0.00	0.00	0.00	0.00

0.00	0.00	0.00	0.00	0.00	0.00	0.00	0.00	0.00
0.00	0.00	0.00	0.00	0.00	0.00	0.00	0.00	0.00
0.00	0.00	0.00	0.00	0.00	0.00	0.00	0.00	0.00
0.00	0.00	0.00	0.00	0.00	0.00	0.00	0.00	0.00
0.00	0.00	0.00	0.00	0.00	0.00	0.00	0.00	0.00
0.00	0.00	0.00	0.00	0.00	0.00	0.00	0.00	0.00
0.00	0.00	0.00	0.00	0.00	0.00	0.00	0.00	0.00
0.00	0.00	0.00	0.00	0.00	0.00	0.00	0.00	0.00
0.00	0.00	0.00	0.00	0.00	0.00	0.00	0.00	0.00
0.00	0.00	0.00	0.00	0.00	0.00	0.00	0.00	0.00
0.00	0.00	0.00	0.00	0.00	0.00	0.00	0.00	0.00
0.00	0.00	0.00	0.00	0.00	0.00	0.00	0.00	0.00
0.00	0.00	0.00	0.00	0.00	0.00	0.00	0.00	0.00
0.00	0.00	0.00	0.00	0.00	0.00	0.00	0.00	0.00
0.00	0.00	0.00	0.00	0.00	0.00	0.00	0.00	0.00
0.00	0.00	0.00	0.00	0.00	0.00	0.00	0.00	0.00
0.00	0.00	0.00	0.00	0.00	0.00	0.00	0.00	0.00
0.00	0.00	0.00	0.00	0.00	0.00	0.00	0.00	0.00
0.00	0.00	0.00	0.00	0.00	0.00	0.00	0.00	0.00
0.00	0.00	0.00	0.00	0.00	0.00	0.00	0.00	0.00
0.00	0.00	0.00	0.00	0.00	0.00	0.00	0.00	0.00
0.00	0.00	0.00	0.00	0.00	0.00	0.00	0.00	0.00
0.00	0.00	0.00	0.00	0.00	0.00	0.00	0.00	0.00
0.00	0.00	0.00	0.00	0.00	0.00	0.00	0.00	0.00
0.00	0.00	0.00	0.00	0.00	0.00	0.00	0.00	0.00
0.00	0.00	0.00	0.00	0.00	0.00	0.00	0.00	0.00
0.00	0.00	0.00	0.00	0.00	0.00	0.00	0.00	0.00
0.00	0.00	0.00	0.00	0.00	0.00	0.00	0.00	0.00
0.00	0.00	0.00	0.00	0.00	0.00	0.00	0.00	0.00
0.00	0.00	0.00	0.00	0.00	0.00	0.00	0.00	0.00
0.00	0.00	0.00	0.00	0.00	0.00	0.00	0.00	0.00
0.00	0.00	0.00	0.00	0.00	0.00	2.00	0.00	0.00
0.00	0.00	0.00	0.00	0.00	0.00	0.00	0.00	0.00
0.00	0.00	0.00	0.00	4.00	0.00	0.00	0.00	0.00
0.00	0.00	0.00	0.00	0.00	0.00	0.00	6.00	0.00
0.00	0.00	0.00	0.00	0.00	0.00	6.00	0.00	0.00
0.00	0.00	0.00	0.00	0.00	6.00	0.00	0.00	0.00

0.00	0.00	0.00	0.00	0.00	4.00	0.00	0.00	0.00
0.00	0.00	0.00	0.00	0.00	6.00	0.00	0.00	0.00
4.00	0.00	0.00	0.00	0.00	0.00	0.00	0.00	0.00
6.00	0.00	0.00	0.00	0.00	0.00	0.00	0.00	0.00
0.00	0.00	0.00	0.00	0.00	0.00	0.00	0.00	0.00
0.00	0.00	0.00	0.00	0.00	0.00	0.00	0.00	0.00
0.00	2.00	0.00	0.00	0.00	0.00	0.00	0.00	0.00
0.00	0.00	0.00	0.00	0.00	0.00	0.00	0.00	0.00
0.00	0.00	0.00	0.00	0.00	0.00	0.00	0.00	0.00
0.00	0.00	0.00	0.00	0.00	0.00	0.00	0.00	0.00
0.00	0.00	0.00	0.00	0.00	0.00	0.00	0.00	0.00
0.00	0.00	0.00	0.00	0.00	0.00	0.00	0.00	0.00
0.00	0.00	0.00	0.00	0.00	0.00	0.00	0.00	0.00
0.00	0.00	0.00	0.00	0.00	0.00	0.00	0.00	0.00
0.00	0.00	0.00	0.00	0.00	0.00	0.00	0.00	0.00
0.00	0.00	0.00	0.00	0.00	0.00	0.00	0.00	0.00
0.00	0.00	0.00	0.00	0.00	0.00	0.00	0.00	0.00
0.00	0.00	2.00	0.00	4.00	0.00	0.00	0.00	0.00
0.00	0.00	0.00	6.00	0.00	0.00	0.00	0.00	0.00
0.00	6.00	0.00	0.00	0.00	0.00	0.00	0.00	0.00
0.00	0.00	0.00	0.00	0.00	0.00	0.00	0.00	0.00
0.00	0.00	0.00	0.00	0.00	0.00	0.00	0.00	0.00
0.00	0.00	0.00	0.00	0.00	0.00	0.00	0.00	0.00
0.00	0.00	0.00	0.00	0.00	0.00	0.00	0.00	0.00
0.00	0.00	0.00	0.00	0.00	0.00	0.00	0.00	0.00
0.00	0.00	0.00	0.00	0.00	0.00	0.00	0.00	0.00
0.00	0.00	0.00	0.00	0.00	0.00	0.00	0.00	0.00
0.00	0.00	0.00	0.00	0.00	0.00	0.00	0.00	0.00
0.00	0.00	0.00	0.00	0.00	0.00	0.00	0.00	0.00
0.00	0.00	0.00	0.00	0.00	0.00	0.00	0.00	0.00
0.00	0.00	0.00	0.00	0.00	0.00	0.00	0.00	0.00
0.00	0.00	0.00	0.00	0.00	0.00	0.00	0.00	0.00
0.00	0.00	0.00	0.00	0.00	0.00	0.00	0.00	0.00
0.00	0.00	0.00	0.00	0.00	0.00	0.00	0.00	0.00
0.00	0.00	0.00	0.00	0.00	0.00	0.00	0.00	0.00
0.00	0.00	0.00	0.00	0.00	0.00	0.00	0.00	0.00
0.00	0.00	0.00	0.00	0.00	0.00	0.00	0.00	0.00

0.00	0.00	0.00	0.00	0.00	0.00	0.00	0.00	0.00
0.00	0.00	0.00	0.00	0.00	0.00	0.00	0.00	0.00
0.00	0.00	0.00	0.00	0.00	0.00	0.00	0.00	0.00
0.00	0.00	0.00	0.00	0.00	0.00	0.00	0.00	0.00
0.00	0.00	0.00	0.00	0.00	0.00	0.00	0.00	0.00
0.00	0.00	0.00	0.00	0.00	0.00	0.00	0.00	0.00
0.00	0.00	0.00	0.00	0.00	0.00	0.00	0.00	0.00
0.00	0.00	0.00	0.00	0.00	0.00	0.00	0.00	0.00
0.00	0.00	0.00	0.00	0.00	0.00	0.00	0.00	0.00
0.00	0.00	0.00	0.00	0.00	0.00	0.00	0.00	0.00
0.00	0.00	0.00	0.00	0.00	0.00	0.00	0.00	0.00
0.00	0.00	0.00	0.00	0.00	0.00	0.00	0.00	0.00
0.00	0.00	0.00	0.00	0.00	0.00	0.00	0.00	0.00
0.00	0.00	0.00	0.00	0.00	0.00	0.00	0.00	0.00
0.00	0.00	0.00	0.00	0.00	0.00	0.00	0.00	0.00
0.00	0.00	0.00	0.00	0.00	0.00	0.00	0.00	0.00
0.00	0.00	0.00	0.00	0.00	0.00	0.00	0.00	0.00
0.00	0.00	0.00	0.00	0.00	0.00	0.00	0.00	0.00
0.00	0.00	0.00	0.00	0.00	0.00	0.00	0.00	0.00
0.00	0.00	0.00	0.00	0.00	0.00	0.00	0.00	0.00
0.00	0.00	0.00	0.00	0.00	0.00	0.00	0.00	0.00
0.00	0.00	0.00	0.00	0.00	0.00	0.00	0.00	0.00
0.00	0.00	0.00	0.00	0.00	0.00	0.00	0.00	0.00
0.00	0.00	0.00	0.00	0.00	0.00	0.00	0.00	0.00
0.00	0.00	0.00	0.00	0.00	0.00	0.00	0.00	0.00
0.00	0.00	0.00	0.00	0.00	0.00	0.00	0.00	0.00
0.00	0.00	0.00	0.00	0.00	0.00	0.00	0.00	0.00
0.00	0.00	0.00	0.00	0.00	0.00	0.00	0.00	0.00
0.00	0.00	0.00	0.00	0.00	0.00	0.00	0.00	0.00
0.00	0.00	0.00	0.00	0.00	0.00	0.00	0.00	0.00
0.00	0.00	0.00	0.00	0.00	0.00	0.00	0.00	0.00
0.00	0.00	0.00	0.00	0.00	0.00	0.00	0.00	0.00
0.00	0.00	0.00	0.00	0.00	0.00	0.00	0.00	0.00
0.00	0.00	0.00	0.00	0.00	0.00	0.00	0.00	0.00
0.00	0.00	0.00	0.00	0.00	0.00	0.00	0.00	0.00
0.00	0.00	0.00	0.00	0.00	0.00	0.00	0.00	0.00
0.00	0.00	0.00	0.00	0.00	0.00	0.00	0.00	0.00

0.00	0.00	0.00	0.00	0.00	0.00	0.00	0.00	0.00
0.00	0.00	0.00	0.00	0.00	0.00	0.00	0.00	0.00
0.00	0.00	0.00	0.00	0.00	0.00	0.00	0.00	0.00
0.00	0.00	0.00	0.00	0.00	0.00	0.00	0.00	0.00
0.00	0.00	0.00	0.00	0.00	0.00	0.00	0.00	0.00
0.00	0.00	0.00	0.00	0.00	0.00	0.00	0.00	0.00
0.00	0.00	0.00	0.00	0.00	0.00	0.00	0.00	0.00
0.00	0.00	0.00	0.00	0.00	0.00	0.00	0.00	0.00
0.00	0.00	0.00	0.00	0.00	0.00	0.00	0.00	0.00
0.00	0.00	0.00	0.00	0.00	0.00	0.00	0.00	0.00
0.00	0.00	0.00	0.00	0.00	0.00	0.00	0.00	0.00
0.00	0.00	0.00	0.00	0.00	0.00	0.00	0.00	0.00
0.00	0.00	0.00	0.00	0.00	0.00	0.00	0.00	0.00
0.00	0.00	0.00	0.00	0.00	0.00	0.00	0.00	5.00
0.00	0.00	0.00	0.00	0.00	5.00	0.00	0.00	0.00
0.00	0.00	0.00	0.00	0.00	5.00	0.00	0.00	0.00
0.00	0.00	0.00	0.00	0.00	5.00	0.00	0.00	0.00
0.00	0.00	0.00	0.00	0.00	5.00	0.00	0.00	0.00
0.00	0.00	0.00	0.00	0.00	5.00	0.00	0.00	0.00
3.00	0.00	0.00	0.00	0.00	2.00	0.00	0.00	0.00
5.00	0.00	0.00	0.00	0.00	0.00	0.00	0.00	0.00
0.00	0.00	0.00	0.00	0.00	0.00	0.00	0.00	0.00
0.00	0.00	0.00	0.00	0.00	0.00	0.00	0.00	0.00
0.00	0.00	0.00	0.00	0.00	0.00	0.00	0.00	0.00
0.00	0.00	0.00	0.00	0.00	0.00	0.00	0.00	0.00
0.00	0.00	0.00	0.00	0.00	0.00	0.00	0.00	0.00
0.00	0.00	0.00	0.00	0.00	0.00	0.00	0.00	0.00
0.00	0.00	0.00	0.00	0.00	0.00	0.00	0.00	0.00
0.00	0.00	0.00	0.00	0.00	0.00	0.00	0.00	0.00
0.00	0.00	0.00	0.00	0.00	0.00	0.00	0.00	0.00
0.00	0.00	0.00	0.00	0.00	0.00	0.00	0.00	0.00
0.00	0.00	0.00	0.00	0.00	0.00	0.00	0.00	0.00
0.00	0.00	0.00	0.00	0.00	0.00	0.00	0.00	0.00
0.00	0.00	0.00	5.00	0.00	0.00	0.00	0.00	0.00
0.00	0.00	0.00	5.00	0.00	0.00	0.00	0.00	0.00
0.00	0.00	0.00	5.00	0.00	0.00	0.00	0.00	0.00
0.00	0.00	5.00	0.00	0.00	0.00	0.00	0.00	0.00

0.00	5.00	0.00	0.00	0.00	0.00	0.00	0.00	0.00
0.00	0.00	0.00	0.00	0.00	0.00	0.00	0.00	0.00
0.00	0.00	0.00	0.00	0.00	0.00	0.00	0.00	0.00
0.00	0.00	0.00	0.00	0.00	0.00	0.00	0.00	0.00
0.00	0.00	0.00	0.00	0.00	0.00	0.00	0.00	0.00
0.00	0.00	0.00	0.00	0.00	0.00	0.00	0.00	0.00
0.00	0.00	0.00	0.00	0.00	0.00	0.00	0.00	0.00
0.00	0.00	0.00	0.00	0.00	0.00	0.00	0.00	0.00
0.00	0.00	0.00	0.00	0.00	0.00	0.00	0.00	0.00
0.00	0.00	0.00	0.00	0.00	0.00	0.00	0.00	0.00
0.00	0.00	0.00	0.00	0.00	0.00	0.00	0.00	0.00
0.00	0.00	0.00	0.00	0.00	0.00	0.00	0.00	0.00
0.00	0.00	0.00	0.00	0.00	0.00	0.00	0.00	0.00
0.00	0.00	0.00	0.00	0.00	0.00	0.00	0.00	0.00
0.00	0.00	0.00	0.00	0.00	0.00	0.00	0.00	0.00
0.00	0.00	0.00	0.00	0.00	0.00	0.00	0.00	0.00
0.00	0.00	0.00	0.00	0.00	0.00	0.00	0.00	0.00
0.00	0.00	0.00	0.00	0.00	0.00	0.00	0.00	0.00
0.00	0.00	0.00	0.00	0.00	0.00	0.00	0.00	0.00
0.00	0.00	0.00	0.00	0.00	0.00	0.00	0.00	0.00
0.00	0.00	0.00	0.00	0.00	0.00	0.00	0.00	0.00
0.00	0.00	0.00	0.00	0.00	0.00	0.00	0.00	0.00
0.00	0.00	0.00	0.00	0.00	0.00	0.00	0.00	0.00
0.00	0.00	0.00	0.00	0.00	0.00	0.00	0.00	0.00
0.00	0.00	0.00	0.00	0.00	0.00	0.00	0.00	0.00
0.00	0.00	0.00	0.00	0.00	0.00	0.00	0.00	0.00
0.00	0.00	0.00	0.00	0.00	0.00	0.00	0.00	0.00
0.00	0.00	0.00	0.00	0.00	0.00	0.00	0.00	0.00
0.00	0.00	0.00	0.00	0.00	0.00	0.00	0.00	0.00
0.00	0.00	0.00	0.00	0.00	0.00	0.00	0.00	0.00
0.00	0.00	0.00	0.00	0.00	0.00	0.00	0.00	0.00
0.00	0.00	0.00	0.00	0.00	0.00	0.00	0.00	0.00
0.00	0.00	0.00	0.00	0.00	0.00	0.00	0.00	0.00
0.00	0.00	0.00	0.00	0.00	0.00	0.00	0.00	0.00
0.00	0.00	0.00	0.00	0.00	0.00	0.00	0.00	0.00
0.00	0.00	0.00	0.00	0.00	0.00	0.00	0.00	0.00
0.00	0.00	0.00	0.00	0.00	0.00	0.00	0.00	0.00

0.00	0.00	0.00	0.00	0.00	0.00	0.00	0.00	0.00
0.00	0.00	0.00	0.00	0.00	0.00	0.00	0.00	0.00
0.00	0.00	0.00	0.00	0.00	0.00	0.00	0.00	0.00
0.00	0.00	0.00	0.00	0.00	0.00	0.00	0.00	0.00
0.00	0.00	0.00	0.00	0.00	0.00	0.00	0.00	0.00
0.00	0.00	0.00	0.00	0.00	0.00	0.00	0.00	0.00
0.00	0.00	0.00	0.00	0.00	0.00	0.00	0.00	0.00
0.00	0.00	0.00	0.00	0.00	0.00	0.00	0.00	0.00
0.00	0.00	0.00	0.00	0.00	0.00	0.00	0.00	0.00
0.00	0.00	0.00	0.00	0.00	0.00	0.00	0.00	0.00
0.00	0.00	0.00	0.00	0.00	0.00	0.00	0.00	0.00
0.00	0.00	0.00	0.00	0.00	0.00	0.00	0.00	0.00
0.00	0.00	0.00	0.00	0.00	0.00	0.00	0.00	0.00
0.00	0.00	0.00	0.00	0.00	0.00	0.00	0.00	0.00
0.00	0.00	0.00	0.00	0.00	0.00	0.00	0.00	0.00
0.00	0.00	0.00	0.00	0.00	0.00	0.00	0.00	0.00
0.00	0.00	0.00	0.00	0.00	0.00	0.00	0.00	0.00
0.00	0.00	0.00	0.00	0.00	0.00	0.00	0.00	0.00
0.00	0.00	0.00	0.00	0.00	0.00	0.00	0.00	0.00
0.00	0.00	0.00	0.00	0.00	0.00	0.00	0.00	0.00
0.00	0.00	0.00	0.00	0.00	0.00	0.00	0.00	0.00
0.00	0.00	0.00	0.00	0.00	0.00	0.00	0.00	0.00
0.00	0.00	0.00	0.00	0.00	0.00	0.00	0.00	0.00
0.00	0.00	0.00	0.00	0.00	0.00	0.00	0.00	0.00
0.00	0.00	0.00	0.00	0.00	0.00	0.00	0.00	0.00
0.00	0.00	0.00	0.00	0.00	0.00	0.00	0.00	0.00
0.00	0.00	0.00	0.00	0.00	0.00	0.00	0.00	0.00
0.00	0.00	0.00	0.00	0.00	0.00	0.00	0.00	0.00
0.00	0.00	0.00	0.00	0.00	0.00	0.00	0.00	0.00
0.00	0.00	0.00	0.00	0.00	0.00	0.00	0.00	0.00
0.00	0.00	0.00	0.00	0.00	0.00	0.00	0.00	0.00
0.00	0.00	0.00	0.00	0.00	0.00	0.00	0.00	0.00
0.00	0.00	0.00	0.00	0.00	0.00	0.00	0.00	0.00
0.00	0.00	0.00	0.00	0.00	0.00	0.00	0.00	0.00
0.00	0.00	0.00	0.00	0.00	0.00	0.00	0.00	0.00
0.00	0.00	0.00	0.00	0.00	0.00	0.00	0.00	4.00
0.00	0.00	0.00	0.00	0.00	0.00	0.00	0.00	4.00

0.00	0.00	0.00	0.00	0.00	0.00	0.00	4.00	0.00
0.00	0.00	0.00	0.00	0.00	0.00	4.00	0.00	0.00
0.00	0.00	0.00	0.00	0.00	4.00	0.00	0.00	0.00
0.00	0.00	0.00	0.00	0.00	4.00	0.00	0.00	0.00
0.00	0.00	0.00	0.00	0.00	4.00	0.00	0.00	0.00
4.00	0.00	0.00	0.00	0.00	0.00	0.00	0.00	0.00
0.00	0.00	0.00	0.00	0.00	0.00	0.00	0.00	0.00
0.00	0.00	0.00	0.00	0.00	0.00	0.00	0.00	0.00
0.00	0.00	0.00	0.00	0.00	0.00	0.00	0.00	0.00
0.00	0.00	0.00	0.00	0.00	0.00	0.00	0.00	0.00
0.00	0.00	0.00	0.00	0.00	0.00	0.00	0.00	0.00
0.00	0.00	0.00	0.00	0.00	0.00	0.00	0.00	0.00
0.00	0.00	0.00	0.00	0.00	0.00	0.00	0.00	0.00
0.00	0.00	0.00	0.00	0.00	0.00	0.00	0.00	0.00
0.00	0.00	0.00	0.00	0.00	0.00	0.00	0.00	0.00
0.00	0.00	0.00	0.00	0.00	0.00	0.00	0.00	0.00
0.00	0.00	0.00	0.00	0.00	0.00	0.00	0.00	0.00
0.00	0.00	0.00	0.00	0.00	0.00	0.00	0.00	0.00
0.00	0.00	0.00	0.00	0.00	0.00	0.00	0.00	0.00
0.00	0.00	0.00	0.00	0.00	0.00	0.00	0.00	0.00
0.00	0.00	0.00	0.00	0.00	0.00	0.00	0.00	0.00
0.00	0.00	0.00	0.00	0.00	0.00	0.00	0.00	0.00
0.00	0.00	0.00	0.00	0.00	0.00	0.00	0.00	0.00
0.00	0.00	0.00	0.00	0.00	0.00	0.00	0.00	0.00
0.00	0.00	0.00	0.00	0.00	0.00	0.00	0.00	0.00
0.00	0.00	0.00	0.00	0.00	0.00	0.00	0.00	0.00
0.00	0.00	0.00	0.00	0.00	0.00	0.00	0.00	0.00
0.00	0.00	0.00	0.00	0.00	0.00	0.00	0.00	0.00
0.00	0.00	0.00	0.00	0.00	0.00	0.00	0.00	0.00
0.00	0.00	0.00	0.00	0.00	0.00	0.00	0.00	0.00
0.00	0.00	0.00	0.00	4.00	0.00	0.00	0.00	0.00
0.00	0.00	0.00	0.00	4.00	0.00	0.00	0.00	0.00
0.00	0.00	0.00	0.00	4.00	0.00	0.00	0.00	0.00
0.00	0.00	0.00	0.00	4.00	0.00	0.00	0.00	0.00
0.00	0.00	0.00	4.00	0.00	0.00	0.00	0.00	0.00
0.00	0.00	0.00	4.00	0.00	0.00	0.00	0.00	0.00
0.00	0.00	0.00	4.00	0.00	0.00	0.00	0.00	0.00

0.00	0.00	0.00	4.00	0.00	0.00	0.00	0.00	0.00
0.00	0.00	4.00	0.00	0.00	0.00	0.00	0.00	0.00
0.00	0.00	4.00	0.00	0.00	0.00	0.00	0.00	0.00
0.00	0.00	4.00	0.00	0.00	0.00	0.00	0.00	0.00
0.00	0.00	4.00	0.00	0.00	0.00	0.00	0.00	0.00
0.00	4.00	0.00	0.00	0.00	0.00	0.00	0.00	0.00
0.00	4.00	0.00	0.00	0.00	0.00	0.00	0.00	0.00
0.00	0.00	0.00	0.00	0.00	0.00	0.00	0.00	0.00
0.00	0.00	0.00	0.00	0.00	0.00	0.00	0.00	0.00
0.00	0.00	0.00	0.00	0.00	0.00	0.00	0.00	0.00
0.00	0.00	0.00	0.00	0.00	0.00	0.00	0.00	0.00
0.00	0.00	0.00	0.00	0.00	0.00	0.00	0.00	0.00
0.00	0.00	0.00	0.00	0.00	0.00	0.00	0.00	0.00
0.00	0.00	0.00	0.00	0.00	0.00	0.00	0.00	0.00
0.00	0.00	0.00	0.00	0.00	0.00	0.00	0.00	0.00
0.00	0.00	0.00	0.00	0.00	0.00	0.00	0.00	0.00
0.00	0.00	0.00	0.00	0.00	0.00	0.00	0.00	0.00
0.00	0.00	0.00	0.00	0.00	0.00	0.00	0.00	0.00
0.00	0.00	0.00	0.00	0.00	0.00	0.00	0.00	0.00
0.00	0.00	0.00	0.00	0.00	0.00	0.00	0.00	0.00
0.00	0.00	0.00	0.00	0.00	0.00	0.00	0.00	0.00
0.00	0.00	0.00	0.00	0.00	0.00	0.00	0.00	0.00
0.00	0.00	0.00	0.00	0.00	0.00	0.00	0.00	0.00
0.00	0.00	0.00	0.00	0.00	0.00	0.00	0.00	0.00
0.00	0.00	0.00	0.00	0.00	0.00	0.00	0.00	0.00
0.00	0.00	0.00	0.00	0.00	0.00	0.00	0.00	0.00
0.00	0.00	0.00	0.00	0.00	0.00	0.00	0.00	0.00
0.00	0.00	0.00	0.00	0.00	0.00	0.00	0.00	0.00
0.00	0.00	0.00	0.00	0.00	0.00	0.00	0.00	0.00
0.00	0.00	0.00	0.00	0.00	0.00	0.00	0.00	0.00
0.00	0.00	0.00	0.00	0.00	0.00	0.00	0.00	0.00
0.00	0.00	0.00	0.00	0.00	0.00	0.00	0.00	0.00
0.00	0.00	0.00	0.00	0.00	0.00	0.00	0.00	0.00
0.00	0.00	0.00	0.00	0.00	0.00	0.00	0.00	0.00
0.00	0.00	0.00	0.00	0.00	0.00	0.00	0.00	0.00
0.00	0.00	0.00	0.00	0.00	0.00	0.00	0.00	0.00
0.00	0.00	0.00	0.00	0.00	0.00	0.00	0.00	0.00

0.00	0.00	0.00	0.00	0.00	0.00	0.00	0.00	0.00
0.00	0.00	0.00	0.00	0.00	0.00	0.00	0.00	0.00
0.00	0.00	0.00	0.00	0.00	0.00	0.00	0.00	0.00
0.00	0.00	0.00	0.00	0.00	0.00	0.00	0.00	0.00
0.00	0.00	0.00	0.00	0.00	0.00	0.00	0.00	0.00
0.00	0.00	0.00	0.00	0.00	0.00	0.00	0.00	0.00
0.00	0.00	0.00	0.00	0.00	0.00	0.00	0.00	0.00
0.00	0.00	0.00	0.00	0.00	0.00	0.00	0.00	0.00
0.00	0.00	0.00	0.00	0.00	0.00	0.00	0.00	0.00
0.00	0.00	0.00	0.00	0.00	0.00	0.00	0.00	0.00
0.00	0.00	0.00	0.00	0.00	0.00	0.00	0.00	0.00
0.00	0.00	0.00	0.00	0.00	0.00	0.00	0.00	0.00
0.00	0.00	0.00	0.00	0.00	0.00	0.00	0.00	0.00
0.00	0.00	0.00	0.00	0.00	0.00	0.00	0.00	0.00
0.00	0.00	0.00	0.00	0.00	0.00	0.00	0.00	0.00
0.00	0.00	0.00	0.00	0.00	0.00	0.00	0.00	0.00
0.00	0.00	0.00	0.00	0.00	0.00	0.00	0.00	0.00
0.00	0.00	0.00	0.00	0.00	0.00	0.00	0.00	0.00
0.00	0.00	0.00	0.00	0.00	0.00	0.00	0.00	0.00
0.00	0.00	0.00	0.00	0.00	0.00	0.00	0.00	0.00
0.00	0.00	0.00	0.00	0.00	0.00	0.00	0.00	0.00
0.00	0.00	0.00	0.00	0.00	0.00	0.00	0.00	0.00
0.00	0.00	0.00	0.00	0.00	0.00	0.00	0.00	0.00
0.00	0.00	0.00	0.00	0.00	0.00	0.00	0.00	0.00
0.00	0.00	0.00	0.00	0.00	0.00	0.00	0.00	0.00
0.00	0.00	0.00	0.00	0.00	0.00	0.00	0.00	0.00
0.00	0.00	0.00	0.00	0.00	0.00	0.00	0.00	0.00
0.00	0.00	0.00	0.00	0.00	0.00	0.00	0.00	0.00
0.00	0.00	0.00	0.00	0.00	0.00	0.00	0.00	0.00
0.00	0.00	0.00	0.00	0.00	0.00	0.00	0.00	0.00
0.00	0.00	0.00	0.00	0.00	0.00	0.00	0.00	0.00
0.00	0.00	0.00	0.00	0.00	0.00	0.00	0.00	0.00
0.00	0.00	0.00	0.00	0.00	0.00	0.00	0.00	0.00
0.00	0.00	0.00	0.00	0.00	0.00	0.00	0.00	0.00
0.00	0.00	0.00	0.00	0.00	0.00	0.00	0.00	0.00
0.00	0.00	0.00	0.00	0.00	0.00	0.00	0.00	0.00
0.00	0.00	0.00	0.00	0.00	0.00	0.00	0.00	0.00

0.00	0.00	0.00	0.00	0.00	0.00	0.00	0.00	0.00
0.00	0.00	0.00	0.00	0.00	0.00	0.00	0.00	0.00
0.00	0.00	0.00	0.00	0.00	0.00	0.00	0.00	0.00
0.00	0.00	0.00	0.00	0.00	0.00	0.00	0.00	0.00
0.00	0.00	0.00	0.00	0.00	0.00	0.00	0.00	0.00
0.00	0.00	0.00	0.00	0.00	0.00	0.00	0.00	0.00
0.00	0.00	0.00	0.00	0.00	0.00	0.00	0.00	0.00
0.00	0.00	0.00	0.00	0.00	0.00	0.00	0.00	0.00
0.00	0.00	0.00	0.00	0.00	0.00	0.00	0.00	0.00
0.00	0.00	0.00	0.00	0.00	0.00	0.00	0.00	0.00
0.00	0.00	0.00	0.00	0.00	0.00	0.00	0.00	0.00
0.00	0.00	0.00	0.00	0.00	0.00	0.00	0.00	0.00
0.00	0.00	0.00	0.00	0.00	0.00	0.00	0.00	0.00
0.00	0.00	0.00	0.00	0.00	0.00	0.00	0.00	0.00
0.00	0.00	0.00	0.00	0.00	0.00	0.00	0.00	0.00
0.00	0.00	0.00	0.00	0.00	0.00	0.00	0.00	0.00
0.00	0.00	0.00	0.00	0.00	0.00	0.00	0.00	0.00
0.00	0.00	0.00	0.00	0.00	0.00	0.00	0.00	0.00
0.00	0.00	0.00	0.00	0.00	0.00	0.00	0.00	3.00
0.00	0.00	0.00	0.00	0.00	0.00	0.00	0.00	3.00
0.00	0.00	0.00	0.00	0.00	0.00	0.00	0.00	3.00
0.00	0.00	0.00	0.00	0.00	0.00	0.00	3.00	0.00
0.00	0.00	0.00	0.00	0.00	0.00	0.00	3.00	0.00
0.00	0.00	0.00	0.00	0.00	0.00	0.00	3.00	0.00
0.00	0.00	0.00	0.00	0.00	0.00	3.00	0.00	0.00
0.00	0.00	0.00	0.00	0.00	3.00	0.00	0.00	0.00
0.00	0.00	0.00	0.00	0.00	3.00	0.00	0.00	0.00
0.00	0.00	0.00	0.00	0.00	3.00	0.00	0.00	0.00
0.00	0.00	0.00	0.00	0.00	3.00	0.00	0.00	0.00
0.00	0.00	0.00	0.00	0.00	3.00	0.00	0.00	0.00
3.00	0.00	0.00	0.00	0.00	0.00	0.00	0.00	0.00
3.00	0.00	0.00	0.00	0.00	0.00	0.00	0.00	0.00
3.00	0.00	0.00	0.00	0.00	0.00	0.00	0.00	0.00
3.00	0.00	0.00	0.00	0.00	0.00	0.00	0.00	0.00
3.00	0.00	0.00	0.00	0.00	0.00	0.00	0.00	0.00
0.00	0.00	0.00	0.00	0.00	0.00	0.00	0.00	0.00
0.00	0.00	0.00	0.00	0.00	0.00	0.00	0.00	0.00

0.00	0.00	0.00	0.00	0.00	0.00	0.00	0.00	0.00
0.00	0.00	0.00	0.00	0.00	0.00	0.00	0.00	0.00
0.00	0.00	0.00	0.00	0.00	0.00	0.00	0.00	0.00
0.00	0.00	0.00	0.00	0.00	0.00	0.00	0.00	0.00
0.00	0.00	0.00	0.00	0.00	0.00	0.00	0.00	0.00
0.00	0.00	0.00	0.00	0.00	0.00	0.00	0.00	0.00
0.00	0.00	0.00	0.00	0.00	0.00	0.00	0.00	0.00
0.00	0.00	0.00	0.00	0.00	0.00	0.00	0.00	0.00
0.00	0.00	0.00	0.00	0.00	0.00	0.00	0.00	0.00
0.00	0.00	0.00	0.00	0.00	0.00	0.00	0.00	0.00
0.00	0.00	0.00	0.00	0.00	0.00	0.00	0.00	0.00
0.00	0.00	0.00	0.00	0.00	0.00	0.00	0.00	0.00
0.00	0.00	0.00	0.00	0.00	0.00	0.00	0.00	0.00
0.00	0.00	0.00	0.00	0.00	0.00	0.00	0.00	0.00
0.00	0.00	0.00	0.00	0.00	0.00	0.00	0.00	0.00
0.00	0.00	0.00	0.00	0.00	0.00	0.00	0.00	0.00
0.00	0.00	0.00	0.00	0.00	0.00	0.00	0.00	0.00
0.00	0.00	0.00	0.00	0.00	0.00	0.00	0.00	0.00
0.00	0.00	0.00	0.00	0.00	0.00	0.00	0.00	0.00
0.00	0.00	0.00	0.00	0.00	0.00	0.00	0.00	0.00
0.00	0.00	0.00	0.00	0.00	0.00	0.00	0.00	0.00
0.00	0.00	0.00	0.00	0.00	0.00	0.00	0.00	0.00
0.00	0.00	0.00	0.00	0.00	0.00	0.00	0.00	0.00
0.00	0.00	0.00	0.00	0.00	0.00	0.00	0.00	0.00
0.00	0.00	0.00	0.00	0.00	0.00	0.00	0.00	0.00
0.00	0.00	0.00	0.00	0.00	0.00	0.00	0.00	0.00
0.00	0.00	0.00	0.00	3.00	0.00	0.00	0.00	0.00
0.00	0.00	0.00	0.00	3.00	0.00	0.00	0.00	0.00
0.00	0.00	0.00	3.00	0.00	0.00	0.00	0.00	0.00
0.00	0.00	0.00	3.00	0.00	0.00	0.00	0.00	0.00
0.00	0.00	3.00	0.00	0.00	0.00	0.00	0.00	0.00
0.00	0.00	3.00	0.00	0.00	0.00	0.00	0.00	0.00
0.00	0.00	3.00	0.00	0.00	0.00	0.00	0.00	0.00
0.00	0.00	3.00	0.00	0.00	0.00	0.00	0.00	0.00
0.00	3.00	0.00	0.00	0.00	0.00	0.00	0.00	0.00
0.00	3.00	0.00	0.00	0.00	0.00	0.00	0.00	0.00
0.00	3.00	0.00	0.00	0.00	0.00	0.00	0.00	0.00

0.00	3.00	0.00	0.00	0.00	0.00	0.00	0.00	0.00
0.00	0.00	0.00	0.00	0.00	0.00	0.00	0.00	0.00
0.00	0.00	0.00	0.00	0.00	0.00	0.00	0.00	0.00
0.00	0.00	0.00	0.00	0.00	0.00	0.00	0.00	0.00
0.00	0.00	0.00	0.00	0.00	0.00	0.00	0.00	0.00
0.00	0.00	0.00	0.00	0.00	0.00	0.00	0.00	0.00
0.00	0.00	0.00	0.00	0.00	0.00	0.00	0.00	0.00
0.00	0.00	0.00	0.00	0.00	0.00	0.00	0.00	0.00
0.00	0.00	0.00	0.00	0.00	0.00	0.00	0.00	0.00
0.00	0.00	0.00	0.00	0.00	0.00	0.00	0.00	0.00
0.00	0.00	0.00	0.00	0.00	0.00	0.00	0.00	0.00
0.00	0.00	0.00	0.00	0.00	0.00	0.00	0.00	0.00
0.00	0.00	0.00	0.00	0.00	0.00	0.00	0.00	0.00
0.00	0.00	0.00	0.00	0.00	0.00	0.00	0.00	0.00
0.00	0.00	0.00	0.00	0.00	0.00	0.00	0.00	0.00
0.00	0.00	0.00	0.00	0.00	0.00	0.00	0.00	0.00
0.00	0.00	0.00	0.00	0.00	0.00	0.00	0.00	0.00
0.00	0.00	0.00	0.00	0.00	0.00	0.00	0.00	0.00
0.00	0.00	0.00	0.00	0.00	0.00	0.00	0.00	0.00
0.00	0.00	0.00	0.00	0.00	0.00	0.00	0.00	0.00
0.00	0.00	0.00	0.00	0.00	0.00	0.00	0.00	0.00
0.00	0.00	0.00	0.00	0.00	0.00	0.00	0.00	0.00
0.00	0.00	0.00	0.00	0.00	0.00	0.00	0.00	0.00
0.00	0.00	0.00	0.00	0.00	0.00	0.00	0.00	0.00
0.00	0.00	0.00	0.00	0.00	0.00	0.00	0.00	0.00
0.00	0.00	0.00	0.00	0.00	0.00	0.00	0.00	0.00
0.00	0.00	0.00	0.00	0.00	0.00	0.00	0.00	0.00
0.00	0.00	0.00	0.00	0.00	0.00	0.00	0.00	0.00
0.00	0.00	0.00	0.00	0.00	0.00	0.00	0.00	0.00
0.00	0.00	0.00	0.00	0.00	0.00	0.00	0.00	0.00
0.00	0.00	0.00	0.00	0.00	0.00	0.00	0.00	0.00
0.00	0.00	0.00	0.00	0.00	0.00	0.00	0.00	0.00
0.00	0.00	0.00	0.00	0.00	0.00	0.00	0.00	0.00
0.00	0.00	0.00	0.00	0.00	0.00	0.00	0.00	0.00
0.00	0.00	0.00	0.00	0.00	0.00	0.00	0.00	0.00
0.00	0.00	0.00	0.00	0.00	0.00	0.00	0.00	0.00
0.00	0.00	0.00	0.00	0.00	0.00	0.00	0.00	0.00

0.00	0.00	0.00	0.00	0.00	0.00	0.00	0.00	0.00
0.00	0.00	0.00	0.00	0.00	0.00	0.00	0.00	0.00
0.00	0.00	0.00	0.00	0.00	0.00	0.00	0.00	0.00
0.00	0.00	0.00	0.00	0.00	0.00	0.00	0.00	0.00
0.00	0.00	0.00	0.00	0.00	0.00	0.00	0.00	0.00
0.00	0.00	0.00	0.00	0.00	0.00	0.00	0.00	0.00
0.00	0.00	0.00	0.00	0.00	0.00	0.00	0.00	0.00
0.00	0.00	0.00	0.00	0.00	0.00	0.00	0.00	0.00
0.00	0.00	0.00	0.00	0.00	0.00	0.00	0.00	0.00
0.00	0.00	0.00	0.00	0.00	0.00	0.00	0.00	0.00
0.00	0.00	0.00	0.00	0.00	0.00	0.00	0.00	0.00
0.00	0.00	0.00	0.00	0.00	0.00	0.00	0.00	0.00
0.00	0.00	0.00	0.00	0.00	0.00	0.00	0.00	0.00
0.00	0.00	0.00	0.00	0.00	0.00	0.00	0.00	0.00
0.00	0.00	0.00	0.00	0.00	0.00	0.00	0.00	0.00
0.00	0.00	0.00	0.00	0.00	0.00	0.00	0.00	0.00
0.00	0.00	0.00	0.00	0.00	0.00	0.00	0.00	0.00
0.00	0.00	0.00	0.00	0.00	0.00	0.00	0.00	0.00
0.00	0.00	0.00	0.00	0.00	0.00	0.00	0.00	0.00
0.00	0.00	0.00	0.00	0.00	0.00	0.00	0.00	0.00
0.00	0.00	0.00	0.00	0.00	0.00	0.00	0.00	0.00
0.00	0.00	0.00	0.00	0.00	0.00	0.00	0.00	0.00
0.00	0.00	0.00	0.00	0.00	0.00	0.00	0.00	0.00
0.00	0.00	0.00	0.00	0.00	0.00	0.00	0.00	0.00
0.00	0.00	0.00	0.00	0.00	0.00	0.00	0.00	0.00
0.00	0.00	0.00	0.00	0.00	0.00	0.00	0.00	0.00
0.00	0.00	0.00	0.00	0.00	0.00	0.00	0.00	0.00
0.00	0.00	0.00	0.00	0.00	0.00	0.00	0.00	0.00
0.00	0.00	0.00	0.00	0.00	0.00	0.00	0.00	0.00
0.00	0.00	0.00	0.00	0.00	0.00	0.00	0.00	0.00
0.00	0.00	0.00	0.00	0.00	0.00	0.00	0.00	0.00
0.00	0.00	0.00	0.00	0.00	0.00	0.00	0.00	0.00
0.00	0.00	0.00	0.00	0.00	0.00	0.00	0.00	0.00
0.00	0.00	0.00	0.00	0.00	0.00	0.00	0.00	0.00
0.00	0.00	0.00	0.00	0.00	0.00	0.00	0.00	0.00
0.00	0.00	0.00	0.00	0.00	0.00	0.00	0.00	0.00
0.00	0.00	0.00	0.00	0.00	0.00	0.00	0.00	0.00

0.00	0.00	0.00	0.00	0.00	0.00	0.00	0.00	0.00
0.00	0.00	0.00	0.00	0.00	0.00	0.00	0.00	0.00
0.00	0.00	0.00	0.00	0.00	0.00	0.00	0.00	0.00
0.00	0.00	0.00	0.00	0.00	0.00	0.00	0.00	0.00
0.00	0.00	0.00	0.00	0.00	0.00	0.00	0.00	0.00
0.00	0.00	0.00	0.00	0.00	0.00	0.00	0.00	0.00
0.00	0.00	0.00	0.00	0.00	0.00	0.00	0.00	0.00
0.00	0.00	0.00	0.00	0.00	0.00	0.00	0.00	0.00
0.00	0.00	0.00	0.00	0.00	0.00	0.00	0.00	0.00
0.00	0.00	0.00	0.00	0.00	0.00	0.00	0.00	0.00
0.00	0.00	0.00	0.00	0.00	0.00	0.00	0.00	0.00
0.00	0.00	0.00	0.00	0.00	0.00	0.00	0.00	0.00
0.00	0.00	0.00	0.00	0.00	0.00	0.00	0.00	0.00
0.00	0.00	0.00	0.00	0.00	0.00	0.00	0.00	0.00
0.00	0.00	0.00	0.00	0.00	0.00	0.00	0.00	0.00
0.00	0.00	0.00	0.00	0.00	0.00	0.00	0.00	0.00
0.00	0.00	0.00	0.00	0.00	0.00	0.00	0.00	0.00
0.00	0.00	0.00	0.00	0.00	0.00	0.00	0.00	0.00
0.00	0.00	0.00	0.00	0.00	0.00	0.00	0.00	0.00
0.00	0.00	0.00	0.00	0.00	0.00	0.00	0.00	0.00
0.00	0.00	0.00	0.00	0.00	0.00	0.00	0.00	0.00
0.00	0.00	0.00	0.00	0.00	0.00	0.00	0.00	0.00
0.00	0.00	0.00	0.00	0.00	0.00	0.00	0.00	0.00
0.00	0.00	0.00	0.00	0.00	0.00	0.00	0.00	0.00
0.00	0.00	0.00	0.00	0.00	0.00	0.00	0.00	0.00
0.00	0.00	0.00	0.00	0.00	0.00	0.00	0.00	2.00
0.00	0.00	0.00	0.00	0.00	0.00	0.00	0.00	2.00
0.00	0.00	0.00	0.00	0.00	0.00	0.00	0.00	2.00
0.00	0.00	0.00	0.00	0.00	0.00	0.00	0.00	2.00
0.00	0.00	0.00	0.00	0.00	0.00	0.00	0.00	2.00
0.00	0.00	0.00	0.00	0.00	0.00	0.00	2.00	0.00
0.00	0.00	0.00	0.00	0.00	0.00	2.00	0.00	0.00
0.00	0.00	0.00	0.00	0.00	2.00	0.00	0.00	0.00
0.00	0.00	0.00	0.00	0.00	2.00	0.00	0.00	0.00
0.00	0.00	0.00	0.00	0.00	2.00	0.00	0.00	0.00
0.00	0.00	0.00	0.00	0.00	2.00	0.00	0.00	0.00
0.00	0.00	0.00	0.00	0.00	2.00	0.00	0.00	0.00

0.00	0.00	0.00	0.00	0.00	2.00	0.00	0.00	0.00
2.00	0.00	0.00	0.00	0.00	0.00	0.00	0.00	0.00
2.00	0.00	0.00	0.00	0.00	0.00	0.00	0.00	0.00
2.00	0.00	0.00	0.00	0.00	0.00	0.00	0.00	0.00
2.00	0.00	0.00	0.00	0.00	0.00	0.00	0.00	0.00
2.00	0.00	0.00	0.00	0.00	0.00	0.00	0.00	0.00
0.00	0.00	0.00	0.00	0.00	0.00	0.00	0.00	0.00
0.00	0.00	0.00	0.00	0.00	0.00	0.00	0.00	0.00
0.00	0.00	0.00	0.00	0.00	0.00	0.00	0.00	0.00
0.00	0.00	0.00	0.00	0.00	0.00	0.00	0.00	0.00
0.00	0.00	0.00	0.00	0.00	0.00	0.00	0.00	0.00
0.00	0.00	0.00	0.00	0.00	0.00	0.00	0.00	0.00
0.00	0.00	0.00	0.00	0.00	0.00	0.00	0.00	0.00
0.00	0.00	0.00	0.00	0.00	0.00	0.00	0.00	0.00
0.00	0.00	0.00	0.00	0.00	0.00	0.00	0.00	0.00
0.00	0.00	0.00	0.00	0.00	0.00	0.00	0.00	0.00
0.00	0.00	0.00	0.00	0.00	0.00	0.00	0.00	0.00
0.00	0.00	0.00	0.00	0.00	0.00	0.00	0.00	0.00
0.00	0.00	0.00	0.00	0.00	0.00	0.00	0.00	0.00
0.00	0.00	0.00	0.00	0.00	0.00	0.00	0.00	0.00
0.00	0.00	0.00	0.00	0.00	0.00	0.00	0.00	0.00
0.00	0.00	0.00	0.00	0.00	0.00	0.00	0.00	0.00
0.00	0.00	0.00	0.00	0.00	0.00	0.00	0.00	0.00
0.00	0.00	0.00	0.00	0.00	0.00	0.00	0.00	0.00
0.00	0.00	0.00	0.00	0.00	0.00	0.00	0.00	0.00
0.00	0.00	0.00	0.00	0.00	0.00	0.00	0.00	0.00
0.00	0.00	0.00	0.00	0.00	0.00	0.00	0.00	0.00
0.00	0.00	0.00	0.00	0.00	0.00	0.00	0.00	0.00
0.00	0.00	0.00	0.00	0.00	0.00	0.00	0.00	0.00
0.00	0.00	0.00	0.00	0.00	0.00	0.00	0.00	0.00
0.00	0.00	0.00	0.00	0.00	0.00	0.00	0.00	0.00
0.00	0.00	0.00	0.00	0.00	0.00	0.00	0.00	0.00
0.00	0.00	0.00	0.00	0.00	0.00	0.00	0.00	0.00
0.00	0.00	0.00	0.00	0.00	0.00	0.00	0.00	0.00
0.00	0.00	0.00	0.00	0.00	0.00	0.00	0.00	0.00
0.00	0.00	0.00	0.00	0.00	0.00	0.00	0.00	0.00
0.00	0.00	0.00	0.00	0.00	0.00	0.00	0.00	0.00

0.00	0.00	0.00	0.00	0.00	0.00	0.00	0.00	0.00
0.00	0.00	0.00	0.00	0.00	0.00	0.00	0.00	0.00
0.00	0.00	0.00	0.00	0.00	0.00	0.00	0.00	0.00
0.00	0.00	0.00	0.00	0.00	0.00	0.00	0.00	0.00
0.00	0.00	0.00	0.00	2.00	0.00	0.00	0.00	0.00
0.00	0.00	0.00	0.00	2.00	0.00	0.00	0.00	0.00
0.00	0.00	0.00	0.00	2.00	0.00	0.00	0.00	0.00
0.00	0.00	0.00	0.00	2.00	0.00	0.00	0.00	0.00
0.00	0.00	0.00	2.00	0.00	0.00	0.00	0.00	0.00
0.00	0.00	0.00	2.00	0.00	0.00	0.00	0.00	0.00
0.00	0.00	0.00	2.00	0.00	0.00	0.00	0.00	0.00
0.00	0.00	0.00	2.00	0.00	0.00	0.00	0.00	0.00
0.00	0.00	2.00	0.00	0.00	0.00	0.00	0.00	0.00
0.00	0.00	2.00	0.00	0.00	0.00	0.00	0.00	0.00
0.00	2.00	0.00	0.00	0.00	0.00	0.00	0.00	0.00
0.00	2.00	0.00	0.00	0.00	0.00	0.00	0.00	0.00
0.00	2.00	0.00	0.00	0.00	0.00	0.00	0.00	0.00
0.00	2.00	0.00	0.00	0.00	0.00	0.00	0.00	0.00
0.00	0.00	0.00	0.00	0.00	0.00	0.00	0.00	0.00
0.00	0.00	0.00	0.00	0.00	0.00	0.00	0.00	0.00
0.00	0.00	0.00	0.00	0.00	0.00	0.00	0.00	0.00
0.00	0.00	0.00	0.00	0.00	0.00	0.00	0.00	0.00
0.00	0.00	0.00	0.00	0.00	0.00	0.00	0.00	0.00
0.00	0.00	0.00	0.00	0.00	0.00	0.00	0.00	0.00
0.00	0.00	0.00	0.00	0.00	0.00	0.00	0.00	0.00
0.00	0.00	0.00	0.00	0.00	0.00	0.00	0.00	0.00
0.00	0.00	0.00	0.00	0.00	0.00	0.00	0.00	0.00
0.00	0.00	0.00	0.00	0.00	0.00	0.00	0.00	0.00
0.00	0.00	0.00	0.00	0.00	0.00	0.00	0.00	0.00
0.00	0.00	0.00	0.00	0.00	0.00	0.00	0.00	0.00
0.00	0.00	0.00	0.00	0.00	0.00	0.00	0.00	0.00
0.00	0.00	0.00	0.00	0.00	0.00	0.00	0.00	0.00
0.00	0.00	0.00	0.00	0.00	0.00	0.00	0.00	0.00

D15	D14	D13	C14	C13	D12	D11	D10	C12
11.00	161.00	0.00	60103.00	317.00	383.00	61537.00	62140.00	4214.00
0.00	159.00	53.00	0.00	0.00	26.00	0.00	0.00	1783.00
22894.00	40088.00	23663.00	893.00	1082.00	3042.00	1317.00	1224.00	41185.00
0.00	0.00	0.00	0.00	0.00	0.00	0.00	0.00	544.00
0.00	0.00	0.00	0.00	0.00	0.00	0.00	0.00	0.00
1147.00	0.00	0.00	0.00	0.00	98.00	0.00	0.00	0.00
0.00	0.00	0.00	519.00	32976.00	24952.00	69.00	92.00	0.00
0.00	0.00	0.00	0.00	0.00	0.00	0.00	0.00	0.00
0.00	0.00	0.00	235.00	12.00	41.00	39.00	116.00	0.00
0.00	0.00	0.00	0.00	0.00	0.00	0.00	0.00	0.00
0.00	11.00	0.00	269.00	12601.00	18525.00	0.00	47.00	0.00
0.00	0.00	1135.00	82.00	0.00	0.00	52.00	0.00	771.00
0.00	0.00	0.00	0.00	6.00	16.00	13.00	40.00	0.00
0.00	0.00	0.00	0.00	0.00	0.00	0.00	0.00	0.00
0.00	0.00	0.00	0.00	0.00	0.00	0.00	0.00	0.00
0.00	0.00	0.00	0.00	0.00	0.00	0.00	0.00	0.00
0.00	0.00	0.00	230.00	13165.00	8816.00	0.00	0.00	0.00
0.00	0.00	0.00	0.00	0.00	0.00	0.00	0.00	0.00
10.00	10.00	22.00	45.00	61.00	119.00	51.00	200.00	6.00
0.00	6.00	0.00	54.00	0.00	0.00	0.00	0.00	0.00
0.00	0.00	0.00	0.00	0.00	0.00	0.00	0.00	0.00
0.00	2084.00	0.00	0.00	0.00	0.00	0.00	0.00	0.00
0.00	0.00	0.00	0.00	36.00	0.00	0.00	0.00	11.00
0.00	0.00	0.00	0.00	0.00	0.00	0.00	0.00	0.00
0.00	0.00	0.00	0.00	0.00	0.00	0.00	0.00	0.00
690.00	1577.00	1480.00	46.00	61.00	169.00	0.00	36.00	59.00
0.00	0.00	0.00	0.00	0.00	0.00	0.00	0.00	0.00
0.00	0.00	0.00	0.00	0.00	0.00	0.00	0.00	0.00
0.00	0.00	0.00	0.00	0.00	0.00	0.00	0.00	0.00
0.00	0.00	0.00	457.00	0.00	0.00	26.00	0.00	0.00
1178.00	102.00	975.00	0.00	0.00	0.00	36.00	41.00	0.00
0.00	0.00	0.00	0.00	0.00	0.00	0.00	50.00	0.00
89.00	0.00	0.00	0.00	0.00	0.00	0.00	0.00	0.00
0.00	0.00	0.00	0.00	0.00	0.00	0.00	0.00	0.00
0.00	0.00	0.00	0.00	0.00	0.00	0.00	0.00	0.00

0.00	0.00	0.00	0.00	0.00	0.00	0.00	8.00	0.00
670.00	0.00	1728.00	29.00	22.00	0.00	9.00	24.00	0.00
0.00	1515.00	1075.00	12.00	6.00	39.00	0.00	0.00	1883.00
0.00	0.00	0.00	0.00	0.00	0.00	0.00	0.00	0.00
0.00	0.00	0.00	0.00	0.00	0.00	0.00	0.00	0.00
0.00	0.00	0.00	3.00	0.00	0.00	0.00	0.00	0.00
0.00	0.00	0.00	0.00	0.00	0.00	0.00	0.00	0.00
7.00	0.00	0.00	26.00	22.00	0.00	52.00	24.00	90.00
147.00	1093.00	0.00	0.00	0.00	74.00	0.00	0.00	0.00
0.00	0.00	0.00	0.00	0.00	0.00	0.00	0.00	0.00
6.00	0.00	6562.00	0.00	0.00	0.00	0.00	0.00	0.00
0.00	0.00	0.00	0.00	0.00	0.00	0.00	0.00	0.00
0.00	0.00	0.00	0.00	0.00	0.00	0.00	0.00	0.00
131.00	1279.00	0.00	0.00	0.00	8.00	0.00	25.00	472.00
2504.00	0.00	3808.00	0.00	0.00	0.00	0.00	0.00	0.00
723.00	0.00	1269.00	20.00	9.00	0.00	0.00	5.00	0.00
0.00	0.00	0.00	0.00	0.00	0.00	0.00	0.00	0.00
0.00	0.00	0.00	0.00	0.00	0.00	0.00	0.00	0.00
0.00	0.00	0.00	0.00	0.00	0.00	0.00	0.00	0.00
0.00	0.00	0.00	0.00	0.00	0.00	0.00	0.00	0.00
0.00	0.00	0.00	0.00	7.00	0.00	0.00	0.00	0.00
4804.00	0.00	4.00	0.00	0.00	0.00	0.00	0.00	0.00
0.00	0.00	0.00	0.00	0.00	0.00	0.00	0.00	0.00
0.00	3.00	0.00	0.00	0.00	0.00	0.00	0.00	0.00
394.00	221.00	8.00	0.00	9.00	0.00	4.00	21.00	0.00
0.00	0.00	0.00	0.00	0.00	0.00	0.00	0.00	0.00
0.00	0.00	0.00	3.00	3.00	0.00	0.00	2.00	873.00
0.00	0.00	0.00	0.00	0.00	0.00	0.00	0.00	0.00
0.00	0.00	0.00	0.00	0.00	0.00	0.00	0.00	0.00
0.00	0.00	0.00	0.00	0.00	0.00	0.00	0.00	0.00
0.00	0.00	0.00	21.00	0.00	0.00	24.00	0.00	0.00
3729.00	0.00	2.00	0.00	0.00	0.00	5.00	0.00	3.00
0.00	1260.00	0.00	7.00	0.00	8.00	0.00	0.00	1594.00
0.00	0.00	0.00	0.00	0.00	22.00	0.00	0.00	0.00
0.00	0.00	0.00	0.00	0.00	0.00	0.00	0.00	0.00
0.00	0.00	0.00	4.00	5.00	0.00	0.00	0.00	0.00
0.00	0.00	0.00	0.00	0.00	8.00	0.00	0.00	0.00

0.00	0.00	0.00	0.00	0.00	0.00	0.00	0.00	0.00
0.00	0.00	427.00	0.00	0.00	0.00	0.00	0.00	0.00
0.00	0.00	0.00	0.00	0.00	0.00	0.00	0.00	0.00
0.00	0.00	0.00	0.00	0.00	0.00	0.00	0.00	0.00
10.00	0.00	0.00	9.00	0.00	4.00	3.00	9.00	7.00
0.00	0.00	0.00	0.00	0.00	0.00	4.00	3.00	0.00
0.00	0.00	0.00	0.00	0.00	0.00	0.00	0.00	0.00
0.00	0.00	0.00	0.00	0.00	5.00	11.00	10.00	0.00
25.00	0.00	5.00	0.00	15.00	0.00	3.00	5.00	17.00
0.00	0.00	0.00	0.00	0.00	0.00	0.00	0.00	0.00
0.00	2543.00	0.00	0.00	0.00	0.00	0.00	0.00	0.00
0.00	0.00	0.00	0.00	0.00	0.00	0.00	0.00	0.00
0.00	0.00	80.00	0.00	0.00	0.00	19.00	0.00	0.00
0.00	219.00	644.00	0.00	0.00	0.00	0.00	0.00	7.00
0.00	0.00	0.00	0.00	0.00	0.00	0.00	0.00	0.00
0.00	0.00	2310.00	0.00	0.00	0.00	0.00	0.00	0.00
0.00	0.00	0.00	0.00	0.00	0.00	0.00	0.00	0.00
0.00	0.00	0.00	0.00	0.00	0.00	0.00	0.00	0.00
0.00	0.00	103.00	0.00	0.00	58.00	0.00	0.00	215.00
0.00	0.00	0.00	0.00	0.00	0.00	0.00	0.00	0.00
6.00	0.00	0.00	0.00	11.00	37.00	9.00	319.00	0.00
0.00	16.00	0.00	52.00	76.00	7.00	23.00	33.00	0.00
0.00	0.00	359.00	14.00	19.00	0.00	0.00	14.00	0.00
0.00	0.00	2017.00	0.00	0.00	0.00	0.00	0.00	0.00
557.00	0.00	0.00	0.00	0.00	0.00	0.00	26.00	0.00
0.00	0.00	1390.00	0.00	0.00	0.00	0.00	0.00	0.00
0.00	0.00	0.00	0.00	0.00	0.00	0.00	0.00	0.00
0.00	0.00	0.00	0.00	0.00	0.00	0.00	0.00	0.00
0.00	0.00	0.00	0.00	46.00	55.00	0.00	181.00	0.00
0.00	0.00	0.00	0.00	0.00	0.00	0.00	0.00	0.00
0.00	0.00	0.00	0.00	0.00	0.00	0.00	0.00	0.00
3.00	0.00	325.00	0.00	0.00	0.00	0.00	0.00	0.00
79.00	12.00	589.00	0.00	0.00	0.00	0.00	3.00	0.00
0.00	49.00	0.00	76.00	25.00	44.00	49.00	41.00	0.00
0.00	0.00	0.00	0.00	0.00	0.00	0.00	0.00	0.00
0.00	0.00	0.00	0.00	0.00	6.00	0.00	0.00	0.00
0.00	0.00	0.00	0.00	0.00	0.00	0.00	0.00	0.00

0.00	21.00	0.00	128.00	94.00	0.00	0.00	0.00	0.00
929.00	0.00	2.00	0.00	0.00	0.00	0.00	0.00	398.00
0.00	0.00	0.00	0.00	0.00	0.00	0.00	0.00	0.00
0.00	0.00	0.00	0.00	0.00	0.00	0.00	0.00	0.00
333.00	0.00	0.00	0.00	0.00	0.00	0.00	0.00	505.00
0.00	0.00	0.00	0.00	0.00	0.00	0.00	0.00	0.00
0.00	1468.00	0.00	5.00	6.00	0.00	0.00	0.00	0.00
0.00	0.00	0.00	13.00	0.00	0.00	0.00	0.00	0.00
0.00	0.00	0.00	132.00	115.00	81.00	31.00	0.00	0.00
0.00	0.00	0.00	0.00	0.00	0.00	0.00	0.00	0.00
0.00	0.00	0.00	0.00	0.00	0.00	0.00	0.00	0.00
0.00	0.00	1345.00	0.00	0.00	0.00	0.00	0.00	0.00
0.00	0.00	0.00	0.00	0.00	0.00	0.00	0.00	0.00
0.00	0.00	0.00	32.00	0.00	0.00	0.00	84.00	0.00
0.00	0.00	0.00	0.00	0.00	0.00	0.00	0.00	0.00
0.00	0.00	0.00	0.00	0.00	0.00	0.00	3.00	0.00
0.00	0.00	0.00	0.00	0.00	0.00	0.00	0.00	0.00
0.00	0.00	0.00	0.00	0.00	0.00	0.00	0.00	0.00
0.00	0.00	0.00	0.00	0.00	0.00	0.00	0.00	0.00
0.00	0.00	0.00	0.00	0.00	0.00	0.00	0.00	0.00
0.00	0.00	0.00	0.00	0.00	0.00	0.00	0.00	0.00
0.00	0.00	0.00	0.00	0.00	0.00	0.00	0.00	0.00
0.00	0.00	0.00	0.00	0.00	0.00	0.00	0.00	0.00
0.00	0.00	0.00	0.00	0.00	48.00	5.00	6.00	0.00
0.00	0.00	924.00	0.00	0.00	0.00	0.00	0.00	0.00
1199.00	0.00	0.00	0.00	0.00	0.00	0.00	0.00	0.00
0.00	0.00	0.00	0.00	0.00	0.00	0.00	0.00	0.00
0.00	0.00	0.00	0.00	0.00	0.00	0.00	33.00	0.00
1176.00	0.00	0.00	0.00	0.00	0.00	0.00	0.00	0.00
1171.00	0.00	0.00	0.00	0.00	0.00	0.00	0.00	0.00
0.00	0.00	0.00	0.00	0.00	39.00	24.00	56.00	0.00
1166.00	0.00	0.00	0.00	0.00	0.00	0.00	0.00	0.00
0.00	0.00	0.00	0.00	202.00	0.00	0.00	0.00	0.00
0.00	0.00	0.00	0.00	0.00	0.00	0.00	0.00	0.00
0.00	0.00	10.00	0.00	0.00	0.00	0.00	0.00	0.00
0.00	0.00	0.00	0.00	0.00	0.00	0.00	0.00	0.00
0.00	0.00	0.00	0.00	0.00	0.00	0.00	0.00	0.00

0.00	0.00	0.00	0.00	0.00	0.00	0.00	0.00	0.00
0.00	0.00	0.00	0.00	0.00	101.00	90.00	143.00	0.00
0.00	0.00	0.00	53.00	46.00	34.00	26.00	32.00	0.00
0.00	0.00	0.00	0.00	0.00	13.00	0.00	7.00	0.00
225.00	0.00	20.00	15.00	0.00	17.00	0.00	0.00	651.00
0.00	0.00	0.00	0.00	0.00	2.00	0.00	0.00	0.00
0.00	0.00	0.00	0.00	0.00	0.00	0.00	0.00	0.00
0.00	0.00	0.00	0.00	0.00	0.00	0.00	0.00	0.00
0.00	624.00	304.00	0.00	3.00	0.00	0.00	0.00	0.00
0.00	0.00	0.00	0.00	0.00	0.00	0.00	10.00	0.00
0.00	0.00	919.00	0.00	0.00	0.00	0.00	0.00	0.00
0.00	0.00	865.00	0.00	0.00	0.00	0.00	0.00	0.00
0.00	0.00	0.00	0.00	0.00	0.00	0.00	0.00	0.00
0.00	0.00	0.00	2.00	0.00	0.00	0.00	0.00	0.00
0.00	0.00	0.00	0.00	0.00	0.00	0.00	4.00	0.00
0.00	0.00	0.00	0.00	0.00	0.00	0.00	0.00	0.00
783.00	0.00	0.00	0.00	0.00	0.00	0.00	0.00	0.00
0.00	8.00	0.00	36.00	38.00	56.00	0.00	27.00	0.00
0.00	0.00	0.00	0.00	0.00	0.00	0.00	0.00	0.00
0.00	0.00	0.00	0.00	0.00	0.00	0.00	0.00	0.00
0.00	0.00	0.00	0.00	0.00	0.00	0.00	0.00	0.00
0.00	0.00	0.00	0.00	0.00	0.00	0.00	0.00	0.00
0.00	0.00	0.00	0.00	0.00	0.00	0.00	0.00	0.00
0.00	0.00	0.00	0.00	0.00	0.00	0.00	0.00	0.00
0.00	764.00	0.00	3.00	3.00	0.00	0.00	0.00	0.00
0.00	0.00	0.00	0.00	271.00	0.00	0.00	0.00	0.00
0.00	0.00	0.00	12.00	6.00	29.00	16.00	33.00	0.00
0.00	0.00	0.00	0.00	41.00	0.00	0.00	0.00	0.00
0.00	0.00	0.00	0.00	0.00	0.00	0.00	0.00	0.00
0.00	0.00	0.00	0.00	0.00	0.00	0.00	0.00	0.00
0.00	0.00	742.00	0.00	0.00	0.00	0.00	0.00	0.00
0.00	18.00	0.00	60.00	58.00	23.00	7.00	13.00	0.00
0.00	0.00	0.00	0.00	0.00	0.00	0.00	0.00	0.00
742.00	0.00	0.00	0.00	0.00	0.00	0.00	0.00	0.00
741.00	0.00	0.00	0.00	0.00	0.00	0.00	0.00	0.00
0.00	0.00	0.00	0.00	0.00	0.00	0.00	0.00	0.00
0.00	0.00	0.00	0.00	0.00	0.00	35.00	0.00	62.00

0.00	0.00	0.00	0.00	0.00	0.00	0.00	0.00	380.00
0.00	0.00	0.00	0.00	0.00	0.00	0.00	0.00	0.00
0.00	0.00	303.00	0.00	0.00	0.00	0.00	0.00	0.00
0.00	0.00	0.00	0.00	0.00	0.00	0.00	0.00	0.00
0.00	0.00	0.00	5.00	0.00	0.00	0.00	0.00	0.00
0.00	0.00	418.00	0.00	0.00	0.00	0.00	0.00	0.00
0.00	0.00	0.00	0.00	0.00	0.00	0.00	0.00	0.00
0.00	0.00	0.00	0.00	0.00	0.00	22.00	0.00	0.00
683.00	0.00	0.00	0.00	0.00	0.00	0.00	0.00	0.00
0.00	0.00	0.00	29.00	18.00	29.00	0.00	22.00	0.00
0.00	0.00	0.00	0.00	0.00	0.00	0.00	0.00	0.00
0.00	0.00	0.00	0.00	0.00	0.00	0.00	0.00	0.00
0.00	0.00	0.00	0.00	0.00	37.00	0.00	45.00	0.00
13.00	0.00	0.00	0.00	0.00	0.00	0.00	6.00	0.00
0.00	0.00	0.00	0.00	0.00	0.00	0.00	0.00	0.00
0.00	0.00	0.00	0.00	0.00	0.00	0.00	0.00	0.00
0.00	0.00	0.00	0.00	0.00	0.00	0.00	0.00	0.00
0.00	0.00	0.00	0.00	0.00	8.00	0.00	0.00	0.00
0.00	0.00	0.00	0.00	0.00	0.00	0.00	0.00	0.00
0.00	0.00	0.00	0.00	0.00	0.00	0.00	4.00	0.00
0.00	0.00	0.00	0.00	0.00	0.00	0.00	0.00	0.00
0.00	0.00	0.00	0.00	0.00	0.00	0.00	0.00	0.00
0.00	0.00	0.00	0.00	0.00	0.00	0.00	0.00	0.00
0.00	0.00	0.00	0.00	0.00	0.00	0.00	0.00	0.00
0.00	0.00	0.00	0.00	0.00	0.00	0.00	0.00	0.00
0.00	0.00	0.00	0.00	0.00	0.00	0.00	0.00	0.00
0.00	0.00	0.00	0.00	0.00	0.00	0.00	0.00	0.00
0.00	0.00	0.00	0.00	0.00	0.00	0.00	0.00	0.00
0.00	0.00	0.00	0.00	0.00	0.00	0.00	0.00	0.00
0.00	0.00	567.00	0.00	0.00	0.00	0.00	0.00	0.00
0.00	0.00	0.00	0.00	0.00	0.00	0.00	0.00	0.00
0.00	0.00	0.00	0.00	108.00	0.00	0.00	0.00	0.00
0.00	0.00	0.00	0.00	0.00	0.00	0.00	0.00	0.00
0.00	0.00	0.00	0.00	0.00	19.00	14.00	27.00	0.00
0.00	0.00	233.00	0.00	0.00	0.00	0.00	0.00	0.00
0.00	0.00	0.00	66.00	60.00	0.00	0.00	0.00	0.00
0.00	0.00	0.00	0.00	0.00	0.00	7.00	0.00	0.00

0.00	0.00	0.00	0.00	0.00	0.00	2.00	0.00	0.00
0.00	0.00	0.00	0.00	0.00	0.00	0.00	0.00	0.00
0.00	0.00	0.00	0.00	0.00	0.00	0.00	0.00	0.00
0.00	5.00	0.00	11.00	0.00	8.00	7.00	8.00	0.00
0.00	0.00	0.00	0.00	0.00	0.00	0.00	0.00	0.00
0.00	0.00	0.00	0.00	0.00	0.00	0.00	2.00	0.00
0.00	0.00	0.00	0.00	0.00	0.00	0.00	0.00	0.00
0.00	0.00	0.00	0.00	0.00	0.00	0.00	0.00	0.00
0.00	0.00	0.00	0.00	0.00	0.00	0.00	0.00	0.00
0.00	0.00	502.00	0.00	0.00	0.00	0.00	0.00	0.00
0.00	0.00	0.00	0.00	6.00	14.00	0.00	6.00	0.00
0.00	0.00	0.00	0.00	0.00	0.00	0.00	0.00	496.00
0.00	0.00	0.00	0.00	0.00	0.00	0.00	0.00	0.00
0.00	0.00	0.00	0.00	0.00	0.00	0.00	0.00	0.00
0.00	0.00	68.00	0.00	0.00	0.00	268.00	0.00	0.00
0.00	487.00	0.00	0.00	2.00	0.00	0.00	0.00	0.00
0.00	0.00	0.00	0.00	0.00	0.00	0.00	0.00	0.00
0.00	0.00	0.00	0.00	0.00	3.00	0.00	0.00	0.00
0.00	0.00	0.00	0.00	0.00	0.00	0.00	0.00	0.00
0.00	0.00	0.00	0.00	0.00	0.00	0.00	0.00	0.00
0.00	0.00	0.00	0.00	19.00	0.00	35.00	0.00	0.00
18.00	0.00	0.00	0.00	0.00	0.00	0.00	0.00	36.00
0.00	0.00	0.00	0.00	0.00	0.00	0.00	0.00	0.00
0.00	0.00	0.00	0.00	0.00	0.00	0.00	0.00	0.00
0.00	0.00	0.00	0.00	0.00	0.00	0.00	0.00	0.00
0.00	0.00	0.00	16.00	0.00	0.00	47.00	45.00	0.00
0.00	0.00	0.00	0.00	0.00	0.00	0.00	0.00	0.00
0.00	0.00	0.00	0.00	0.00	0.00	0.00	0.00	0.00
0.00	0.00	0.00	0.00	0.00	0.00	0.00	0.00	0.00
12.00	0.00	23.00	0.00	0.00	0.00	0.00	5.00	49.00
0.00	458.00	0.00	0.00	0.00	0.00	0.00	0.00	0.00
0.00	0.00	0.00	0.00	0.00	0.00	0.00	0.00	0.00
0.00	0.00	0.00	0.00	0.00	0.00	0.00	0.00	0.00
0.00	0.00	0.00	0.00	0.00	0.00	0.00	0.00	459.00
0.00	0.00	0.00	0.00	0.00	0.00	0.00	0.00	0.00
0.00	0.00	0.00	0.00	0.00	0.00	0.00	0.00	0.00
0.00	0.00	0.00	0.00	0.00	0.00	0.00	0.00	0.00

0.00	0.00	0.00	0.00	0.00	0.00	0.00	0.00	0.00
0.00	0.00	0.00	0.00	0.00	0.00	0.00	0.00	0.00
0.00	0.00	440.00	0.00	0.00	0.00	0.00	0.00	0.00
0.00	0.00	0.00	0.00	0.00	0.00	0.00	0.00	0.00
0.00	0.00	0.00	0.00	0.00	0.00	9.00	0.00	0.00
0.00	0.00	0.00	0.00	0.00	0.00	0.00	0.00	0.00
0.00	0.00	0.00	0.00	0.00	0.00	0.00	3.00	0.00
0.00	0.00	0.00	0.00	0.00	0.00	0.00	0.00	0.00
0.00	0.00	0.00	0.00	0.00	0.00	0.00	0.00	0.00
0.00	0.00	0.00	0.00	0.00	0.00	0.00	0.00	0.00
0.00	0.00	0.00	0.00	0.00	0.00	0.00	0.00	0.00
392.00	0.00	0.00	0.00	0.00	0.00	0.00	0.00	0.00
0.00	385.00	0.00	0.00	0.00	0.00	0.00	0.00	0.00
0.00	0.00	0.00	0.00	0.00	0.00	0.00	0.00	0.00
0.00	0.00	0.00	0.00	40.00	8.00	0.00	0.00	0.00
0.00	0.00	0.00	0.00	0.00	0.00	0.00	0.00	0.00
0.00	0.00	0.00	0.00	0.00	0.00	0.00	0.00	0.00
0.00	0.00	0.00	0.00	0.00	0.00	0.00	0.00	0.00
0.00	0.00	0.00	0.00	0.00	0.00	0.00	0.00	0.00
0.00	0.00	0.00	0.00	0.00	0.00	0.00	0.00	0.00
0.00	0.00	0.00	0.00	0.00	0.00	0.00	0.00	0.00
0.00	0.00	0.00	0.00	0.00	0.00	0.00	0.00	0.00
0.00	0.00	0.00	0.00	0.00	0.00	0.00	0.00	0.00
0.00	0.00	0.00	0.00	0.00	0.00	0.00	0.00	0.00
0.00	8.00	0.00	0.00	25.00	0.00	0.00	0.00	0.00
0.00	0.00	0.00	0.00	0.00	0.00	0.00	0.00	0.00
352.00	0.00	0.00	0.00	0.00	0.00	0.00	0.00	0.00
0.00	0.00	0.00	0.00	0.00	0.00	0.00	0.00	0.00
0.00	0.00	0.00	0.00	0.00	0.00	0.00	0.00	0.00
0.00	0.00	0.00	41.00	2.00	31.00	0.00	13.00	0.00
0.00	0.00	0.00	0.00	0.00	0.00	0.00	0.00	108.00
0.00	0.00	0.00	0.00	0.00	0.00	0.00	0.00	0.00
0.00	0.00	0.00	0.00	0.00	0.00	0.00	0.00	0.00
0.00	0.00	0.00	0.00	0.00	0.00	0.00	0.00	0.00
0.00	0.00	0.00	0.00	0.00	0.00	0.00	0.00	0.00
0.00	0.00	0.00	0.00	0.00	0.00	0.00	0.00	0.00
0.00	0.00	0.00	0.00	0.00	0.00	0.00	0.00	0.00

0.00	6.00	0.00	44.00	0.00	0.00	6.00	5.00	0.00
0.00	0.00	0.00	27.00	31.00	0.00	0.00	8.00	0.00
0.00	0.00	0.00	0.00	0.00	0.00	0.00	0.00	0.00
0.00	0.00	173.00	0.00	0.00	8.00	0.00	0.00	0.00
0.00	0.00	0.00	0.00	0.00	0.00	0.00	41.00	0.00
0.00	0.00	0.00	0.00	0.00	0.00	0.00	0.00	0.00
0.00	0.00	0.00	0.00	0.00	65.00	38.00	72.00	0.00
0.00	0.00	0.00	0.00	0.00	0.00	0.00	0.00	0.00
0.00	0.00	0.00	0.00	0.00	0.00	0.00	0.00	0.00
0.00	0.00	0.00	0.00	0.00	0.00	0.00	0.00	0.00
0.00	0.00	0.00	0.00	0.00	0.00	0.00	0.00	0.00
0.00	0.00	0.00	0.00	0.00	0.00	0.00	0.00	0.00
0.00	0.00	0.00	0.00	0.00	0.00	0.00	0.00	0.00
0.00	0.00	0.00	5.00	8.00	0.00	7.00	6.00	0.00
0.00	0.00	0.00	0.00	0.00	0.00	0.00	0.00	0.00
0.00	0.00	0.00	2.00	0.00	0.00	0.00	0.00	0.00
0.00	0.00	0.00	0.00	0.00	0.00	0.00	0.00	0.00
0.00	0.00	0.00	0.00	0.00	0.00	0.00	0.00	0.00
0.00	0.00	0.00	0.00	0.00	0.00	0.00	0.00	0.00
0.00	0.00	0.00	0.00	0.00	0.00	0.00	0.00	0.00
0.00	0.00	0.00	0.00	0.00	0.00	0.00	0.00	32.00
0.00	0.00	0.00	0.00	0.00	0.00	0.00	0.00	0.00
0.00	0.00	0.00	0.00	0.00	0.00	0.00	0.00	0.00
0.00	0.00	0.00	0.00	0.00	0.00	0.00	0.00	0.00
0.00	0.00	0.00	0.00	0.00	0.00	0.00	0.00	0.00
0.00	0.00	0.00	0.00	0.00	0.00	0.00	0.00	0.00
0.00	0.00	0.00	0.00	0.00	0.00	0.00	0.00	0.00
0.00	0.00	0.00	0.00	62.00	27.00	0.00	0.00	0.00
0.00	0.00	0.00	0.00	0.00	0.00	0.00	0.00	5.00
180.00	0.00	0.00	0.00	0.00	0.00	0.00	0.00	7.00
0.00	0.00	0.00	0.00	0.00	0.00	0.00	0.00	0.00
0.00	0.00	0.00	0.00	0.00	0.00	0.00	0.00	0.00
0.00	0.00	0.00	0.00	0.00	0.00	0.00	0.00	0.00
0.00	295.00	0.00	0.00	4.00	0.00	0.00	0.00	0.00
0.00	0.00	0.00	0.00	0.00	0.00	0.00	0.00	0.00
0.00	0.00	0.00	0.00	0.00	0.00	0.00	0.00	0.00
0.00	0.00	0.00	0.00	0.00	0.00	0.00	0.00	0.00

0.00	0.00	0.00	0.00	0.00	0.00	0.00	0.00	0.00
0.00	0.00	0.00	0.00	0.00	0.00	0.00	0.00	0.00
0.00	0.00	0.00	0.00	0.00	0.00	0.00	0.00	0.00
0.00	0.00	0.00	0.00	0.00	0.00	0.00	0.00	0.00
0.00	0.00	0.00	0.00	0.00	0.00	0.00	0.00	0.00
0.00	0.00	0.00	13.00	19.00	16.00	0.00	7.00	0.00
0.00	0.00	0.00	0.00	0.00	0.00	0.00	0.00	0.00
0.00	0.00	0.00	0.00	0.00	0.00	0.00	0.00	0.00
0.00	0.00	0.00	0.00	0.00	0.00	0.00	0.00	0.00
0.00	0.00	0.00	41.00	0.00	34.00	0.00	0.00	0.00
0.00	0.00	0.00	0.00	0.00	2.00	0.00	0.00	0.00
0.00	0.00	265.00	0.00	0.00	0.00	0.00	0.00	0.00
0.00	0.00	0.00	0.00	0.00	0.00	0.00	0.00	0.00
0.00	0.00	0.00	0.00	0.00	0.00	0.00	0.00	0.00
0.00	21.00	0.00	0.00	0.00	0.00	72.00	0.00	0.00
0.00	0.00	0.00	0.00	0.00	0.00	0.00	0.00	0.00
0.00	0.00	0.00	0.00	0.00	0.00	0.00	0.00	0.00
0.00	0.00	0.00	0.00	0.00	0.00	0.00	0.00	0.00
0.00	257.00	0.00	0.00	0.00	0.00	0.00	0.00	0.00
0.00	0.00	0.00	0.00	0.00	0.00	0.00	0.00	0.00
0.00	0.00	0.00	0.00	0.00	0.00	0.00	0.00	0.00
0.00	0.00	0.00	0.00	0.00	0.00	0.00	0.00	0.00
0.00	0.00	0.00	0.00	5.00	0.00	0.00	0.00	0.00
0.00	0.00	0.00	11.00	18.00	0.00	0.00	0.00	0.00
0.00	0.00	0.00	0.00	0.00	0.00	47.00	0.00	0.00
0.00	0.00	0.00	0.00	0.00	0.00	0.00	0.00	0.00
0.00	0.00	0.00	0.00	0.00	0.00	0.00	0.00	0.00
0.00	0.00	0.00	0.00	0.00	0.00	0.00	0.00	0.00
0.00	0.00	0.00	0.00	0.00	0.00	0.00	6.00	0.00
0.00	0.00	0.00	0.00	0.00	0.00	0.00	0.00	0.00
0.00	0.00	0.00	0.00	0.00	0.00	0.00	0.00	0.00
0.00	0.00	0.00	0.00	0.00	0.00	0.00	0.00	0.00
0.00	0.00	0.00	0.00	0.00	0.00	0.00	0.00	0.00
0.00	0.00	240.00	0.00	0.00	0.00	0.00	0.00	0.00
0.00	0.00	0.00	0.00	0.00	0.00	0.00	0.00	0.00
0.00	0.00	0.00	0.00	0.00	0.00	0.00	0.00	0.00
0.00	0.00	0.00	0.00	0.00	0.00	0.00	0.00	0.00

0.00	0.00	0.00	0.00	0.00	0.00	0.00	0.00	0.00
0.00	0.00	0.00	0.00	0.00	0.00	0.00	0.00	0.00
0.00	0.00	0.00	0.00	0.00	0.00	0.00	0.00	0.00
0.00	0.00	0.00	0.00	0.00	0.00	0.00	0.00	0.00
0.00	0.00	0.00	0.00	0.00	0.00	0.00	0.00	0.00
0.00	0.00	229.00	0.00	0.00	0.00	0.00	0.00	0.00
0.00	0.00	0.00	0.00	0.00	0.00	0.00	0.00	0.00
0.00	219.00	0.00	0.00	0.00	0.00	0.00	0.00	0.00
0.00	0.00	0.00	0.00	0.00	0.00	0.00	0.00	0.00
0.00	0.00	0.00	0.00	0.00	0.00	0.00	0.00	0.00
0.00	0.00	0.00	13.00	0.00	0.00	0.00	0.00	0.00
0.00	0.00	0.00	0.00	0.00	0.00	0.00	0.00	0.00
0.00	0.00	0.00	0.00	12.00	0.00	10.00	0.00	0.00
0.00	0.00	0.00	31.00	0.00	0.00	5.00	0.00	0.00
0.00	220.00	0.00	0.00	0.00	0.00	0.00	0.00	0.00
0.00	219.00	0.00	0.00	0.00	0.00	0.00	0.00	0.00
0.00	219.00	0.00	0.00	0.00	0.00	0.00	0.00	0.00
0.00	0.00	0.00	0.00	0.00	0.00	0.00	0.00	0.00
0.00	0.00	0.00	0.00	0.00	0.00	0.00	0.00	0.00
0.00	0.00	0.00	0.00	0.00	0.00	0.00	0.00	0.00
0.00	0.00	0.00	17.00	0.00	40.00	0.00	0.00	0.00
0.00	0.00	0.00	28.00	34.00	0.00	0.00	0.00	0.00
0.00	0.00	0.00	0.00	0.00	0.00	0.00	0.00	0.00
0.00	0.00	0.00	0.00	0.00	0.00	0.00	0.00	0.00
0.00	0.00	0.00	12.00	0.00	0.00	0.00	0.00	0.00
0.00	0.00	0.00	0.00	0.00	0.00	0.00	0.00	0.00
0.00	0.00	0.00	0.00	0.00	0.00	0.00	0.00	0.00
0.00	0.00	0.00	0.00	0.00	11.00	7.00	20.00	0.00
0.00	0.00	0.00	5.00	5.00	6.00	8.00	6.00	0.00
0.00	0.00	0.00	36.00	0.00	0.00	0.00	29.00	0.00
0.00	0.00	0.00	0.00	0.00	0.00	0.00	0.00	0.00
0.00	0.00	0.00	0.00	0.00	0.00	0.00	0.00	0.00
0.00	0.00	0.00	22.00	30.00	0.00	0.00	0.00	0.00
0.00	0.00	0.00	0.00	0.00	0.00	0.00	0.00	0.00
0.00	0.00	0.00	5.00	0.00	0.00	0.00	0.00	0.00
0.00	0.00	0.00	0.00	0.00	0.00	0.00	0.00	0.00
0.00	0.00	0.00	0.00	0.00	0.00	0.00	0.00	0.00

0.00	0.00	0.00	0.00	0.00	0.00	0.00	0.00	0.00
0.00	0.00	186.00	0.00	0.00	0.00	0.00	0.00	0.00
0.00	0.00	0.00	0.00	0.00	0.00	0.00	0.00	0.00
0.00	0.00	0.00	0.00	0.00	0.00	0.00	0.00	0.00
0.00	0.00	0.00	0.00	0.00	19.00	0.00	0.00	0.00
0.00	0.00	0.00	0.00	0.00	0.00	0.00	0.00	13.00
0.00	0.00	0.00	0.00	0.00	0.00	0.00	2.00	0.00
0.00	0.00	0.00	0.00	0.00	0.00	0.00	0.00	0.00
0.00	0.00	0.00	0.00	0.00	0.00	0.00	0.00	138.00
0.00	192.00	0.00	0.00	0.00	0.00	0.00	0.00	0.00
0.00	0.00	0.00	0.00	0.00	0.00	0.00	0.00	0.00
0.00	0.00	0.00	0.00	0.00	0.00	0.00	0.00	0.00
0.00	0.00	0.00	0.00	0.00	0.00	0.00	0.00	0.00
0.00	193.00	0.00	0.00	0.00	0.00	0.00	0.00	0.00
0.00	0.00	0.00	0.00	0.00	0.00	0.00	0.00	0.00
0.00	0.00	0.00	15.00	10.00	0.00	0.00	5.00	0.00
0.00	0.00	0.00	0.00	0.00	0.00	0.00	0.00	0.00
0.00	0.00	0.00	11.00	16.00	0.00	0.00	0.00	0.00
0.00	0.00	0.00	0.00	0.00	0.00	0.00	0.00	0.00
0.00	0.00	0.00	0.00	5.00	11.00	5.00	6.00	0.00
0.00	0.00	0.00	10.00	0.00	13.00	0.00	8.00	0.00
0.00	0.00	0.00	0.00	0.00	0.00	0.00	0.00	0.00
0.00	0.00	0.00	0.00	0.00	0.00	0.00	0.00	0.00
0.00	0.00	0.00	0.00	0.00	0.00	0.00	0.00	0.00
0.00	0.00	0.00	0.00	0.00	0.00	0.00	0.00	0.00
0.00	0.00	0.00	0.00	0.00	0.00	0.00	0.00	0.00
0.00	0.00	0.00	0.00	0.00	0.00	0.00	0.00	0.00
0.00	0.00	0.00	0.00	0.00	0.00	0.00	0.00	0.00
0.00	0.00	0.00	10.00	0.00	0.00	0.00	10.00	0.00
0.00	0.00	0.00	0.00	0.00	0.00	58.00	10.00	0.00
0.00	0.00	0.00	0.00	0.00	0.00	0.00	0.00	0.00
0.00	0.00	0.00	0.00	0.00	0.00	0.00	0.00	0.00
0.00	0.00	0.00	8.00	5.00	24.00	21.00	16.00	0.00
0.00	0.00	0.00	0.00	0.00	0.00	13.00	0.00	0.00
0.00	17.00	0.00	0.00	0.00	0.00	0.00	0.00	0.00
176.00	0.00	0.00	0.00	0.00	0.00	0.00	0.00	0.00
0.00	0.00	0.00	14.00	0.00	0.00	7.00	15.00	0.00

0.00	0.00	0.00	0.00	0.00	0.00	0.00	0.00	0.00
0.00	0.00	175.00	0.00	0.00	0.00	0.00	0.00	0.00
0.00	0.00	0.00	0.00	0.00	0.00	0.00	0.00	0.00
0.00	0.00	0.00	0.00	0.00	0.00	0.00	0.00	0.00
0.00	0.00	0.00	0.00	0.00	0.00	0.00	0.00	0.00
0.00	0.00	0.00	0.00	0.00	0.00	0.00	0.00	0.00
0.00	0.00	0.00	0.00	0.00	0.00	0.00	0.00	0.00
0.00	0.00	0.00	0.00	0.00	0.00	0.00	0.00	0.00
0.00	0.00	0.00	0.00	0.00	0.00	0.00	0.00	0.00
0.00	0.00	0.00	0.00	0.00	0.00	0.00	0.00	0.00
0.00	0.00	44.00	0.00	0.00	0.00	0.00	0.00	0.00
0.00	0.00	0.00	0.00	0.00	0.00	0.00	0.00	0.00
0.00	0.00	0.00	0.00	0.00	0.00	0.00	0.00	0.00
0.00	0.00	0.00	0.00	0.00	0.00	0.00	0.00	0.00
0.00	170.00	0.00	0.00	0.00	0.00	0.00	0.00	0.00
0.00	0.00	0.00	0.00	0.00	0.00	0.00	0.00	0.00
68.00	0.00	0.00	0.00	0.00	0.00	0.00	0.00	0.00
0.00	0.00	0.00	0.00	0.00	0.00	0.00	0.00	0.00
0.00	10.00	0.00	0.00	35.00	0.00	0.00	0.00	0.00
0.00	5.00	0.00	23.00	22.00	0.00	0.00	0.00	0.00
0.00	0.00	0.00	18.00	21.00	0.00	0.00	0.00	0.00
0.00	0.00	0.00	0.00	0.00	0.00	0.00	0.00	0.00
0.00	0.00	0.00	0.00	0.00	0.00	0.00	0.00	0.00
0.00	0.00	0.00	0.00	0.00	0.00	0.00	0.00	0.00
0.00	0.00	0.00	0.00	0.00	0.00	0.00	0.00	0.00
0.00	0.00	0.00	0.00	0.00	0.00	0.00	0.00	0.00
0.00	0.00	162.00	0.00	0.00	0.00	0.00	0.00	0.00
0.00	0.00	0.00	0.00	0.00	0.00	0.00	0.00	0.00
0.00	0.00	0.00	0.00	0.00	0.00	0.00	0.00	0.00
0.00	0.00	0.00	0.00	0.00	0.00	0.00	0.00	0.00
0.00	0.00	0.00	0.00	0.00	0.00	0.00	0.00	0.00
0.00	0.00	0.00	0.00	0.00	0.00	0.00	0.00	10.00
0.00	0.00	0.00	0.00	0.00	0.00	0.00	0.00	0.00
0.00	0.00	0.00	0.00	0.00	0.00	0.00	0.00	0.00
0.00	0.00	0.00	0.00	0.00	0.00	0.00	0.00	0.00
0.00	0.00	0.00	0.00	0.00	0.00	0.00	0.00	0.00
0.00	0.00	0.00	0.00	0.00	0.00	0.00	0.00	0.00

0.00	7.00	0.00	0.00	0.00	0.00	0.00	0.00	0.00
0.00	0.00	0.00	0.00	0.00	0.00	0.00	0.00	0.00
0.00	0.00	0.00	0.00	0.00	0.00	0.00	0.00	0.00
0.00	0.00	0.00	0.00	0.00	0.00	0.00	0.00	0.00
0.00	0.00	0.00	0.00	0.00	0.00	0.00	0.00	0.00
0.00	0.00	0.00	0.00	0.00	0.00	0.00	0.00	0.00
0.00	0.00	0.00	0.00	0.00	0.00	0.00	0.00	0.00
0.00	0.00	0.00	0.00	7.00	11.00	0.00	0.00	0.00
0.00	0.00	0.00	0.00	0.00	0.00	0.00	0.00	0.00
0.00	0.00	0.00	0.00	0.00	0.00	8.00	0.00	0.00
0.00	0.00	0.00	0.00	0.00	0.00	0.00	0.00	0.00
0.00	0.00	0.00	0.00	0.00	0.00	0.00	0.00	0.00
0.00	0.00	0.00	0.00	0.00	0.00	0.00	0.00	0.00
0.00	151.00	0.00	0.00	0.00	0.00	0.00	0.00	0.00
0.00	0.00	0.00	0.00	0.00	0.00	0.00	0.00	0.00
0.00	0.00	0.00	30.00	25.00	0.00	0.00	0.00	0.00
0.00	0.00	0.00	0.00	0.00	0.00	0.00	0.00	0.00
0.00	0.00	0.00	0.00	0.00	0.00	0.00	0.00	0.00
0.00	0.00	0.00	0.00	0.00	18.00	16.00	19.00	0.00
0.00	0.00	0.00	0.00	0.00	0.00	0.00	0.00	0.00
0.00	0.00	0.00	0.00	0.00	0.00	0.00	0.00	0.00
0.00	0.00	0.00	0.00	0.00	0.00	0.00	0.00	0.00
0.00	0.00	0.00	0.00	0.00	0.00	0.00	0.00	0.00
0.00	0.00	0.00	0.00	0.00	15.00	0.00	0.00	0.00
0.00	0.00	0.00	0.00	0.00	0.00	0.00	0.00	0.00
0.00	0.00	0.00	0.00	0.00	0.00	0.00	0.00	0.00
0.00	6.00	0.00	8.00	13.00	0.00	0.00	0.00	0.00
0.00	0.00	0.00	0.00	0.00	0.00	0.00	0.00	0.00
0.00	0.00	142.00	0.00	0.00	0.00	0.00	0.00	0.00
0.00	0.00	0.00	0.00	0.00	0.00	0.00	0.00	0.00
0.00	0.00	0.00	0.00	2.00	0.00	0.00	0.00	0.00
0.00	3.00	0.00	19.00	21.00	0.00	0.00	0.00	0.00
0.00	0.00	0.00	41.00	0.00	15.00	4.00	0.00	0.00
0.00	0.00	0.00	0.00	0.00	0.00	0.00	0.00	0.00
0.00	0.00	0.00	0.00	0.00	0.00	0.00	0.00	0.00
0.00	137.00	0.00	0.00	0.00	0.00	0.00	0.00	0.00
0.00	0.00	0.00	0.00	0.00	0.00	0.00	0.00	0.00

0.00	0.00	0.00	33.00	0.00	8.00	0.00	0.00	0.00
0.00	0.00	0.00	0.00	0.00	0.00	0.00	0.00	0.00
0.00	0.00	0.00	38.00	40.00	0.00	0.00	0.00	0.00
0.00	0.00	0.00	0.00	0.00	0.00	3.00	0.00	0.00
0.00	0.00	0.00	0.00	0.00	0.00	0.00	0.00	0.00
0.00	0.00	0.00	0.00	5.00	5.00	13.00	9.00	0.00
0.00	131.00	0.00	0.00	0.00	0.00	0.00	0.00	0.00
0.00	0.00	0.00	0.00	0.00	0.00	0.00	0.00	0.00
0.00	0.00	0.00	0.00	0.00	0.00	0.00	0.00	0.00
0.00	0.00	0.00	0.00	0.00	0.00	0.00	0.00	0.00
0.00	0.00	0.00	0.00	0.00	0.00	0.00	0.00	0.00
0.00	0.00	0.00	0.00	0.00	2.00	0.00	0.00	0.00
0.00	0.00	3.00	0.00	0.00	0.00	0.00	0.00	0.00
0.00	0.00	0.00	0.00	0.00	0.00	0.00	0.00	0.00
0.00	0.00	0.00	0.00	0.00	0.00	0.00	0.00	0.00
0.00	0.00	0.00	0.00	23.00	0.00	0.00	0.00	0.00
0.00	0.00	0.00	0.00	0.00	0.00	0.00	0.00	0.00
0.00	0.00	0.00	0.00	0.00	0.00	0.00	0.00	0.00
0.00	0.00	0.00	0.00	0.00	0.00	0.00	0.00	0.00
0.00	0.00	0.00	0.00	0.00	0.00	0.00	0.00	0.00
0.00	0.00	0.00	0.00	0.00	0.00	0.00	0.00	0.00
0.00	0.00	122.00	0.00	0.00	0.00	0.00	0.00	0.00
0.00	0.00	0.00	0.00	0.00	0.00	0.00	0.00	0.00
3.00	7.00	0.00	0.00	0.00	0.00	0.00	0.00	0.00
0.00	0.00	0.00	0.00	0.00	0.00	0.00	0.00	0.00
0.00	0.00	0.00	0.00	0.00	0.00	0.00	0.00	0.00
0.00	0.00	0.00	33.00	0.00	0.00	0.00	0.00	0.00
0.00	0.00	0.00	0.00	0.00	0.00	0.00	0.00	0.00
0.00	0.00	0.00	0.00	0.00	0.00	0.00	0.00	0.00
0.00	0.00	0.00	0.00	0.00	0.00	0.00	0.00	0.00
0.00	0.00	0.00	0.00	0.00	0.00	0.00	0.00	0.00
0.00	0.00	0.00	0.00	0.00	0.00	0.00	0.00	0.00
0.00	0.00	0.00	0.00	0.00	0.00	0.00	6.00	0.00
0.00	0.00	0.00	0.00	0.00	0.00	0.00	0.00	0.00
0.00	0.00	0.00	0.00	0.00	0.00	0.00	0.00	0.00
0.00	0.00	0.00	0.00	0.00	0.00	0.00	0.00	0.00
0.00	0.00	0.00	0.00	0.00	0.00	0.00	0.00	0.00

0.00	0.00	0.00	0.00	0.00	0.00	0.00	0.00	0.00
46.00	0.00	0.00	0.00	0.00	0.00	0.00	0.00	68.00
0.00	0.00	0.00	0.00	0.00	0.00	0.00	0.00	0.00
0.00	0.00	0.00	0.00	0.00	0.00	0.00	0.00	0.00
0.00	0.00	0.00	0.00	0.00	0.00	0.00	9.00	0.00
0.00	0.00	0.00	0.00	0.00	0.00	0.00	0.00	0.00
0.00	0.00	0.00	0.00	0.00	0.00	0.00	0.00	0.00
0.00	0.00	0.00	0.00	0.00	0.00	0.00	0.00	0.00
0.00	0.00	0.00	0.00	0.00	0.00	0.00	0.00	0.00
0.00	0.00	0.00	0.00	0.00	0.00	0.00	0.00	0.00
0.00	0.00	0.00	0.00	0.00	0.00	0.00	0.00	0.00
0.00	0.00	0.00	24.00	19.00	2.00	0.00	0.00	0.00
0.00	0.00	0.00	0.00	0.00	0.00	0.00	0.00	0.00
0.00	0.00	0.00	0.00	0.00	0.00	0.00	0.00	0.00
0.00	0.00	0.00	0.00	0.00	0.00	0.00	0.00	0.00
0.00	0.00	0.00	0.00	0.00	0.00	0.00	0.00	0.00
0.00	0.00	0.00	0.00	0.00	0.00	0.00	0.00	0.00
0.00	0.00	0.00	0.00	0.00	0.00	0.00	0.00	0.00
0.00	0.00	0.00	0.00	0.00	0.00	0.00	0.00	0.00
0.00	0.00	0.00	0.00	0.00	0.00	0.00	0.00	0.00
0.00	0.00	0.00	0.00	0.00	0.00	0.00	0.00	0.00
0.00	0.00	0.00	0.00	0.00	0.00	0.00	0.00	0.00
0.00	0.00	0.00	0.00	0.00	0.00	0.00	0.00	0.00
0.00	0.00	0.00	0.00	0.00	0.00	0.00	0.00	0.00
0.00	0.00	0.00	0.00	0.00	0.00	0.00	0.00	0.00
0.00	0.00	0.00	0.00	0.00	0.00	0.00	0.00	0.00
0.00	0.00	0.00	0.00	0.00	0.00	0.00	0.00	0.00
0.00	0.00	0.00	0.00	0.00	0.00	0.00	0.00	0.00
0.00	0.00	0.00	0.00	0.00	0.00	0.00	0.00	0.00
0.00	0.00	0.00	0.00	0.00	0.00	0.00	0.00	0.00
0.00	0.00	0.00	0.00	0.00	0.00	0.00	0.00	0.00
0.00	0.00	0.00	0.00	0.00	0.00	0.00	0.00	0.00
0.00	0.00	0.00	0.00	0.00	0.00	0.00	0.00	0.00
0.00	0.00	0.00	0.00	0.00	0.00	0.00	0.00	0.00
0.00	0.00	0.00	0.00	0.00	0.00	0.00	0.00	0.00
0.00	0.00	0.00	0.00	0.00	0.00	0.00	0.00	0.00
0.00	0.00	0.00	0.00	0.00	0.00	0.00	0.00	0.00

0.00	0.00	0.00	25.00	0.00	0.00	0.00	0.00	0.00
0.00	0.00	0.00	0.00	0.00	0.00	0.00	0.00	0.00
0.00	0.00	0.00	0.00	0.00	0.00	0.00	0.00	0.00
0.00	0.00	101.00	0.00	0.00	0.00	0.00	0.00	0.00
101.00	0.00	0.00	0.00	0.00	0.00	0.00	0.00	0.00
0.00	0.00	0.00	0.00	0.00	0.00	0.00	0.00	0.00
0.00	0.00	0.00	0.00	0.00	0.00	0.00	0.00	0.00
0.00	0.00	0.00	0.00	0.00	0.00	0.00	0.00	0.00
0.00	0.00	0.00	0.00	0.00	0.00	0.00	0.00	0.00
0.00	6.00	0.00	13.00	6.00	0.00	0.00	0.00	0.00
0.00	0.00	0.00	0.00	0.00	0.00	0.00	0.00	0.00
0.00	0.00	99.00	0.00	0.00	0.00	0.00	0.00	0.00
0.00	0.00	0.00	0.00	4.00	8.00	16.00	17.00	0.00
0.00	0.00	0.00	0.00	0.00	0.00	0.00	0.00	0.00
0.00	0.00	0.00	0.00	0.00	0.00	0.00	0.00	0.00
0.00	0.00	0.00	0.00	0.00	0.00	0.00	0.00	0.00
0.00	0.00	0.00	0.00	0.00	0.00	0.00	0.00	0.00
0.00	0.00	0.00	0.00	0.00	0.00	0.00	0.00	0.00
0.00	0.00	0.00	0.00	0.00	0.00	0.00	0.00	0.00
0.00	0.00	0.00	0.00	0.00	0.00	0.00	0.00	0.00
96.00	0.00	0.00	0.00	0.00	0.00	0.00	0.00	0.00
0.00	0.00	0.00	0.00	0.00	0.00	0.00	0.00	0.00
0.00	0.00	0.00	0.00	0.00	5.00	0.00	0.00	0.00
0.00	0.00	0.00	0.00	0.00	0.00	0.00	0.00	0.00
0.00	0.00	0.00	0.00	0.00	0.00	0.00	0.00	0.00
0.00	0.00	0.00	0.00	0.00	0.00	18.00	0.00	0.00
0.00	94.00	0.00	0.00	0.00	0.00	0.00	0.00	0.00
0.00	0.00	0.00	0.00	0.00	0.00	0.00	0.00	0.00
0.00	0.00	0.00	0.00	0.00	0.00	0.00	0.00	0.00
0.00	0.00	0.00	0.00	0.00	0.00	0.00	0.00	0.00
0.00	0.00	0.00	0.00	0.00	0.00	0.00	0.00	0.00
0.00	0.00	0.00	0.00	0.00	0.00	0.00	0.00	0.00
0.00	0.00	0.00	0.00	0.00	0.00	0.00	0.00	0.00
0.00	0.00	0.00	0.00	0.00	0.00	0.00	0.00	0.00
0.00	0.00	0.00	0.00	0.00	0.00	0.00	0.00	0.00
0.00	0.00	0.00	0.00	0.00	0.00	0.00	0.00	0.00
0.00	0.00	0.00	0.00	0.00	0.00	0.00	0.00	0.00

0.00	0.00	0.00	0.00	0.00	0.00	0.00	0.00	0.00
0.00	0.00	0.00	0.00	0.00	0.00	0.00	0.00	0.00
0.00	0.00	0.00	0.00	0.00	0.00	0.00	0.00	0.00
0.00	0.00	0.00	0.00	0.00	0.00	0.00	0.00	0.00
0.00	0.00	0.00	0.00	0.00	7.00	0.00	9.00	0.00
0.00	0.00	0.00	0.00	0.00	0.00	0.00	0.00	0.00
0.00	0.00	0.00	0.00	0.00	31.00	0.00	0.00	0.00
0.00	0.00	0.00	0.00	0.00	0.00	0.00	0.00	0.00
0.00	0.00	0.00	0.00	0.00	0.00	0.00	0.00	0.00
0.00	60.00	0.00	0.00	0.00	0.00	0.00	0.00	0.00
0.00	0.00	0.00	0.00	0.00	0.00	0.00	0.00	0.00
0.00	0.00	0.00	0.00	0.00	0.00	0.00	0.00	0.00
0.00	0.00	0.00	0.00	0.00	0.00	0.00	0.00	0.00
0.00	4.00	0.00	4.00	0.00	0.00	4.00	5.00	0.00
0.00	0.00	0.00	0.00	0.00	0.00	0.00	0.00	0.00
0.00	0.00	0.00	0.00	0.00	0.00	0.00	0.00	0.00
0.00	0.00	0.00	0.00	0.00	0.00	0.00	0.00	0.00
0.00	0.00	0.00	0.00	0.00	0.00	0.00	0.00	0.00
0.00	0.00	0.00	0.00	0.00	0.00	0.00	0.00	0.00
0.00	0.00	0.00	16.00	15.00	0.00	0.00	0.00	0.00
0.00	0.00	0.00	0.00	0.00	0.00	0.00	0.00	0.00
0.00	0.00	0.00	0.00	0.00	0.00	0.00	0.00	0.00
0.00	0.00	0.00	0.00	0.00	0.00	0.00	0.00	0.00
0.00	0.00	0.00	0.00	0.00	0.00	0.00	0.00	0.00
0.00	0.00	0.00	0.00	0.00	0.00	0.00	0.00	0.00
0.00	14.00	0.00	22.00	0.00	0.00	0.00	0.00	0.00
0.00	0.00	0.00	0.00	0.00	0.00	0.00	0.00	0.00
0.00	0.00	0.00	0.00	0.00	0.00	0.00	0.00	0.00
0.00	0.00	0.00	0.00	0.00	0.00	0.00	0.00	0.00
0.00	0.00	0.00	0.00	0.00	0.00	0.00	0.00	0.00
0.00	0.00	0.00	0.00	0.00	0.00	0.00	0.00	0.00
0.00	0.00	0.00	0.00	0.00	0.00	0.00	0.00	0.00
0.00	0.00	0.00	0.00	0.00	0.00	0.00	0.00	0.00
0.00	0.00	0.00	7.00	10.00	0.00	0.00	0.00	0.00
0.00	0.00	0.00	0.00	0.00	0.00	0.00	0.00	0.00
0.00	0.00	0.00	0.00	0.00	0.00	0.00	0.00	0.00
0.00	0.00	0.00	0.00	0.00	0.00	0.00	0.00	0.00

0.00	10.00	0.00	0.00	25.00	14.00	0.00	0.00	0.00
0.00	0.00	0.00	0.00	0.00	0.00	0.00	0.00	0.00
0.00	0.00	0.00	0.00	0.00	0.00	0.00	4.00	0.00
0.00	0.00	0.00	0.00	0.00	0.00	0.00	0.00	0.00
0.00	0.00	0.00	0.00	0.00	0.00	0.00	14.00	0.00
0.00	0.00	0.00	0.00	0.00	0.00	0.00	0.00	0.00
0.00	77.00	0.00	0.00	0.00	0.00	0.00	0.00	0.00
0.00	0.00	0.00	0.00	0.00	0.00	0.00	0.00	0.00
0.00	0.00	0.00	0.00	0.00	0.00	0.00	0.00	0.00
0.00	0.00	0.00	34.00	20.00	0.00	0.00	0.00	0.00
0.00	0.00	0.00	0.00	0.00	0.00	0.00	0.00	0.00
0.00	0.00	0.00	0.00	0.00	0.00	0.00	0.00	0.00
0.00	0.00	0.00	0.00	0.00	0.00	0.00	0.00	0.00
0.00	0.00	0.00	0.00	0.00	0.00	76.00	0.00	0.00
0.00	0.00	0.00	0.00	0.00	0.00	0.00	0.00	0.00
0.00	0.00	0.00	0.00	0.00	0.00	0.00	0.00	0.00
0.00	0.00	0.00	0.00	0.00	0.00	0.00	0.00	0.00
0.00	0.00	0.00	0.00	0.00	0.00	0.00	0.00	0.00
0.00	0.00	0.00	0.00	0.00	0.00	0.00	0.00	0.00
0.00	0.00	0.00	0.00	0.00	0.00	0.00	0.00	0.00
0.00	0.00	0.00	0.00	0.00	0.00	0.00	0.00	0.00
0.00	0.00	0.00	0.00	0.00	0.00	0.00	0.00	0.00
0.00	0.00	0.00	0.00	0.00	0.00	0.00	6.00	0.00
0.00	5.00	0.00	16.00	16.00	0.00	0.00	0.00	0.00
74.00	0.00	0.00	0.00	0.00	0.00	0.00	0.00	0.00
0.00	0.00	0.00	0.00	0.00	0.00	0.00	0.00	0.00
0.00	0.00	0.00	0.00	0.00	0.00	0.00	0.00	0.00
0.00	0.00	0.00	0.00	0.00	0.00	0.00	0.00	0.00
0.00	0.00	0.00	0.00	0.00	0.00	0.00	0.00	0.00
0.00	0.00	0.00	0.00	0.00	0.00	0.00	0.00	0.00
0.00	0.00	0.00	0.00	0.00	0.00	0.00	0.00	0.00
0.00	0.00	0.00	0.00	0.00	0.00	0.00	0.00	0.00
0.00	0.00	0.00	0.00	0.00	15.00	9.00	19.00	0.00
0.00	0.00	0.00	0.00	0.00	0.00	0.00	0.00	0.00
0.00	0.00	0.00	0.00	0.00	15.00	11.00	12.00	0.00
0.00	0.00	0.00	0.00	7.00	0.00	0.00	0.00	0.00
0.00	0.00	0.00	0.00	0.00	41.00	0.00	0.00	0.00

0.00	0.00	0.00	0.00	0.00	0.00	0.00	0.00	0.00
0.00	0.00	0.00	0.00	0.00	0.00	0.00	0.00	0.00
0.00	0.00	0.00	0.00	9.00	0.00	0.00	0.00	0.00
0.00	0.00	0.00	0.00	0.00	0.00	0.00	8.00	0.00
0.00	0.00	0.00	0.00	0.00	0.00	0.00	0.00	0.00
0.00	0.00	0.00	0.00	0.00	0.00	0.00	0.00	0.00
0.00	0.00	0.00	0.00	0.00	0.00	0.00	0.00	0.00
0.00	0.00	0.00	0.00	0.00	0.00	17.00	0.00	0.00
0.00	0.00	0.00	0.00	0.00	0.00	0.00	0.00	0.00
0.00	0.00	0.00	0.00	0.00	0.00	0.00	0.00	0.00
0.00	0.00	0.00	0.00	0.00	0.00	0.00	0.00	0.00
0.00	0.00	0.00	0.00	0.00	0.00	0.00	8.00	0.00
0.00	0.00	0.00	0.00	23.00	0.00	0.00	0.00	0.00
0.00	0.00	0.00	0.00	0.00	0.00	0.00	0.00	0.00
0.00	0.00	0.00	0.00	0.00	0.00	0.00	0.00	0.00
0.00	0.00	0.00	0.00	0.00	0.00	0.00	0.00	0.00
0.00	0.00	0.00	0.00	0.00	0.00	0.00	0.00	0.00
0.00	0.00	0.00	0.00	0.00	0.00	0.00	0.00	0.00
0.00	0.00	0.00	0.00	0.00	0.00	0.00	0.00	0.00
0.00	0.00	0.00	0.00	2.00	3.00	6.00	3.00	0.00
0.00	0.00	0.00	0.00	0.00	0.00	0.00	0.00	0.00
0.00	0.00	0.00	0.00	0.00	0.00	0.00	0.00	0.00
0.00	0.00	0.00	0.00	0.00	0.00	0.00	0.00	0.00
0.00	0.00	0.00	0.00	0.00	0.00	0.00	0.00	0.00
0.00	0.00	0.00	0.00	0.00	0.00	0.00	0.00	0.00
0.00	5.00	0.00	0.00	0.00	0.00	0.00	0.00	0.00
0.00	0.00	0.00	0.00	0.00	14.00	0.00	19.00	0.00
0.00	0.00	0.00	0.00	0.00	0.00	0.00	0.00	0.00
0.00	0.00	0.00	0.00	0.00	0.00	0.00	0.00	0.00
0.00	0.00	0.00	0.00	0.00	0.00	0.00	0.00	0.00
0.00	0.00	0.00	0.00	0.00	0.00	0.00	0.00	0.00
0.00	0.00	0.00	0.00	0.00	0.00	0.00	8.00	0.00
0.00	0.00	0.00	0.00	0.00	0.00	0.00	0.00	0.00
6.00	0.00	8.00	0.00	0.00	0.00	0.00	0.00	0.00
0.00	0.00	0.00	0.00	0.00	0.00	0.00	0.00	0.00
0.00	0.00	0.00	0.00	0.00	0.00	0.00	0.00	0.00
0.00	0.00	0.00	0.00	0.00	0.00	0.00	0.00	0.00

0.00	0.00	0.00	17.00	0.00	0.00	0.00	0.00	0.00
0.00	0.00	0.00	0.00	0.00	0.00	0.00	0.00	0.00
0.00	0.00	0.00	0.00	0.00	0.00	0.00	0.00	0.00
64.00	0.00	0.00	0.00	0.00	0.00	0.00	0.00	0.00
0.00	0.00	0.00	0.00	0.00	0.00	0.00	0.00	0.00
0.00	0.00	0.00	0.00	0.00	0.00	0.00	0.00	0.00
0.00	0.00	0.00	0.00	0.00	0.00	0.00	0.00	0.00
0.00	0.00	0.00	0.00	0.00	23.00	0.00	0.00	0.00
0.00	0.00	0.00	0.00	0.00	0.00	0.00	0.00	0.00
0.00	0.00	0.00	0.00	0.00	15.00	6.00	9.00	0.00
0.00	0.00	0.00	0.00	0.00	0.00	18.00	8.00	0.00
0.00	0.00	0.00	0.00	0.00	0.00	0.00	0.00	0.00
0.00	0.00	0.00	0.00	0.00	15.00	0.00	0.00	0.00
0.00	0.00	0.00	0.00	0.00	0.00	0.00	0.00	0.00
0.00	0.00	0.00	0.00	0.00	0.00	0.00	0.00	0.00
0.00	0.00	0.00	3.00	0.00	9.00	0.00	11.00	0.00
0.00	0.00	0.00	0.00	0.00	0.00	0.00	0.00	0.00
0.00	0.00	0.00	0.00	0.00	0.00	0.00	0.00	0.00
0.00	0.00	0.00	19.00	0.00	0.00	0.00	0.00	0.00
0.00	0.00	0.00	0.00	0.00	30.00	0.00	0.00	0.00
0.00	0.00	0.00	0.00	0.00	0.00	0.00	0.00	0.00
0.00	0.00	0.00	0.00	0.00	0.00	0.00	0.00	0.00
0.00	0.00	0.00	0.00	0.00	0.00	29.00	0.00	0.00
0.00	0.00	0.00	0.00	0.00	0.00	0.00	0.00	0.00
0.00	0.00	0.00	0.00	0.00	0.00	0.00	0.00	0.00
0.00	0.00	0.00	0.00	0.00	0.00	0.00	0.00	0.00
0.00	0.00	0.00	0.00	0.00	0.00	0.00	0.00	0.00
0.00	0.00	0.00	0.00	0.00	0.00	0.00	0.00	0.00
0.00	0.00	0.00	0.00	0.00	0.00	0.00	0.00	0.00
0.00	0.00	0.00	0.00	0.00	0.00	0.00	0.00	0.00
0.00	0.00	0.00	0.00	0.00	0.00	0.00	0.00	0.00
0.00	0.00	0.00	0.00	0.00	0.00	0.00	0.00	0.00
0.00	0.00	0.00	0.00	0.00	0.00	0.00	0.00	0.00
0.00	0.00	0.00	0.00	0.00	0.00	60.00	0.00	0.00
0.00	0.00	0.00	0.00	0.00	0.00	0.00	0.00	0.00
0.00	0.00	0.00	0.00	0.00	0.00	0.00	0.00	0.00
0.00	0.00	0.00	0.00	0.00	0.00	0.00	0.00	0.00

0.00	0.00	0.00	0.00	0.00	0.00	0.00	0.00	0.00
0.00	0.00	0.00	0.00	0.00	0.00	0.00	0.00	0.00
0.00	0.00	0.00	0.00	8.00	0.00	0.00	0.00	0.00
0.00	0.00	0.00	0.00	0.00	0.00	0.00	0.00	0.00
0.00	0.00	0.00	0.00	0.00	0.00	0.00	0.00	0.00
0.00	0.00	0.00	0.00	0.00	0.00	0.00	0.00	0.00
0.00	0.00	0.00	0.00	0.00	0.00	0.00	0.00	0.00
0.00	0.00	0.00	0.00	0.00	0.00	0.00	13.00	0.00
0.00	0.00	0.00	0.00	0.00	0.00	0.00	0.00	0.00
0.00	0.00	0.00	0.00	0.00	0.00	0.00	0.00	0.00
0.00	0.00	0.00	0.00	0.00	0.00	0.00	0.00	0.00
0.00	0.00	0.00	0.00	0.00	0.00	0.00	0.00	0.00
0.00	0.00	0.00	0.00	0.00	0.00	0.00	0.00	0.00
0.00	0.00	0.00	0.00	0.00	0.00	0.00	0.00	0.00
0.00	0.00	0.00	0.00	0.00	0.00	0.00	0.00	0.00
0.00	0.00	0.00	0.00	0.00	31.00	0.00	0.00	0.00
0.00	0.00	0.00	0.00	0.00	0.00	0.00	0.00	0.00
0.00	0.00	0.00	0.00	0.00	0.00	0.00	0.00	0.00
0.00	0.00	0.00	0.00	0.00	57.00	0.00	0.00	0.00
0.00	0.00	0.00	0.00	0.00	0.00	0.00	0.00	0.00
0.00	0.00	0.00	0.00	0.00	0.00	0.00	0.00	0.00
0.00	0.00	0.00	0.00	5.00	0.00	0.00	0.00	0.00
0.00	0.00	0.00	0.00	0.00	0.00	0.00	0.00	0.00
0.00	0.00	0.00	0.00	0.00	0.00	0.00	0.00	0.00
0.00	0.00	0.00	0.00	0.00	0.00	0.00	0.00	0.00
0.00	0.00	0.00	0.00	0.00	0.00	0.00	0.00	0.00
0.00	0.00	0.00	0.00	0.00	0.00	0.00	0.00	0.00
0.00	0.00	0.00	0.00	0.00	0.00	0.00	5.00	0.00
0.00	0.00	0.00	0.00	0.00	0.00	0.00	10.00	0.00
0.00	0.00	0.00	0.00	0.00	0.00	0.00	0.00	0.00
0.00	0.00	0.00	0.00	0.00	0.00	0.00	0.00	0.00
0.00	0.00	56.00	0.00	0.00	0.00	0.00	0.00	0.00
0.00	0.00	0.00	0.00	0.00	0.00	0.00	0.00	0.00
0.00	0.00	0.00	0.00	0.00	0.00	0.00	0.00	0.00
0.00	0.00	0.00	0.00	0.00	0.00	0.00	0.00	0.00
0.00	0.00	0.00	0.00	0.00	0.00	0.00	0.00	0.00
0.00	0.00	0.00	0.00	0.00	0.00	0.00	0.00	0.00

0.00	0.00	0.00	0.00	0.00	0.00	0.00	0.00	0.00
0.00	0.00	0.00	0.00	0.00	0.00	0.00	0.00	0.00
0.00	0.00	0.00	0.00	0.00	0.00	0.00	0.00	0.00
0.00	0.00	0.00	10.00	4.00	0.00	0.00	0.00	0.00
0.00	0.00	0.00	24.00	0.00	0.00	0.00	0.00	0.00
0.00	0.00	0.00	0.00	0.00	0.00	0.00	0.00	0.00
0.00	0.00	0.00	0.00	0.00	0.00	0.00	0.00	0.00
0.00	54.00	0.00	0.00	0.00	0.00	0.00	0.00	0.00
0.00	0.00	0.00	0.00	0.00	0.00	0.00	0.00	0.00
0.00	0.00	0.00	0.00	0.00	0.00	0.00	0.00	0.00
0.00	0.00	0.00	8.00	0.00	0.00	0.00	0.00	0.00
0.00	0.00	0.00	0.00	0.00	0.00	0.00	0.00	0.00
0.00	0.00	0.00	0.00	0.00	0.00	0.00	0.00	0.00
0.00	0.00	0.00	0.00	0.00	0.00	0.00	0.00	0.00
0.00	0.00	0.00	0.00	0.00	0.00	0.00	0.00	0.00
0.00	0.00	0.00	0.00	0.00	0.00	0.00	0.00	0.00
0.00	0.00	0.00	0.00	0.00	0.00	0.00	0.00	0.00
0.00	0.00	0.00	0.00	0.00	0.00	0.00	0.00	0.00
0.00	0.00	0.00	7.00	0.00	0.00	0.00	0.00	0.00
0.00	0.00	0.00	0.00	0.00	0.00	0.00	0.00	0.00
0.00	0.00	0.00	30.00	0.00	0.00	0.00	0.00	0.00
0.00	0.00	0.00	0.00	0.00	0.00	0.00	0.00	0.00
0.00	0.00	0.00	0.00	0.00	0.00	0.00	0.00	0.00
0.00	0.00	0.00	0.00	0.00	9.00	0.00	0.00	0.00
0.00	0.00	0.00	0.00	0.00	0.00	0.00	0.00	0.00
0.00	0.00	0.00	0.00	0.00	0.00	0.00	0.00	0.00
0.00	0.00	0.00	0.00	0.00	0.00	0.00	0.00	0.00
0.00	0.00	0.00	0.00	0.00	0.00	0.00	0.00	0.00
0.00	0.00	0.00	0.00	0.00	0.00	0.00	0.00	0.00
0.00	0.00	50.00	0.00	0.00	0.00	0.00	0.00	0.00
0.00	0.00	0.00	0.00	0.00	0.00	0.00	0.00	0.00
0.00	0.00	0.00	0.00	0.00	0.00	0.00	0.00	0.00
0.00	0.00	0.00	0.00	0.00	0.00	0.00	0.00	0.00
0.00	0.00	0.00	0.00	0.00	0.00	0.00	0.00	0.00
0.00	0.00	0.00	0.00	0.00	0.00	0.00	8.00	0.00
0.00	0.00	0.00	16.00	0.00	0.00	0.00	0.00	0.00
0.00	0.00	0.00	0.00	0.00	0.00	0.00	0.00	0.00

0.00	0.00	0.00	0.00	0.00	0.00	0.00	0.00	0.00
0.00	0.00	0.00	0.00	0.00	0.00	0.00	0.00	0.00
0.00	0.00	0.00	0.00	0.00	0.00	0.00	0.00	0.00
0.00	0.00	0.00	0.00	0.00	0.00	0.00	0.00	0.00
0.00	0.00	0.00	0.00	0.00	0.00	0.00	6.00	0.00
0.00	0.00	0.00	0.00	0.00	0.00	0.00	0.00	0.00
0.00	0.00	0.00	0.00	0.00	0.00	0.00	0.00	0.00
0.00	0.00	0.00	0.00	0.00	0.00	0.00	0.00	0.00
0.00	0.00	0.00	0.00	0.00	0.00	0.00	0.00	0.00
0.00	0.00	0.00	0.00	0.00	0.00	0.00	0.00	0.00
0.00	0.00	0.00	0.00	0.00	0.00	0.00	0.00	0.00
0.00	0.00	0.00	0.00	0.00	0.00	0.00	0.00	0.00
0.00	0.00	0.00	0.00	0.00	0.00	0.00	0.00	0.00
0.00	0.00	0.00	0.00	0.00	0.00	0.00	0.00	0.00
0.00	0.00	0.00	0.00	0.00	0.00	0.00	0.00	0.00
0.00	0.00	0.00	4.00	0.00	3.00	0.00	0.00	0.00
0.00	0.00	0.00	0.00	0.00	0.00	0.00	12.00	0.00
0.00	0.00	0.00	0.00	0.00	0.00	0.00	0.00	0.00
0.00	0.00	0.00	0.00	0.00	0.00	0.00	0.00	0.00
0.00	0.00	0.00	0.00	0.00	0.00	0.00	8.00	0.00
0.00	0.00	0.00	0.00	0.00	0.00	0.00	0.00	0.00
0.00	0.00	0.00	0.00	0.00	0.00	0.00	0.00	0.00
0.00	0.00	0.00	0.00	0.00	0.00	0.00	0.00	0.00
0.00	0.00	0.00	0.00	0.00	0.00	0.00	0.00	0.00
0.00	0.00	0.00	0.00	0.00	0.00	0.00	0.00	0.00
0.00	0.00	0.00	0.00	0.00	0.00	0.00	4.00	0.00
0.00	0.00	0.00	0.00	0.00	0.00	0.00	0.00	0.00
0.00	0.00	0.00	0.00	0.00	0.00	0.00	0.00	0.00
0.00	0.00	0.00	0.00	0.00	0.00	0.00	0.00	0.00
0.00	0.00	0.00	0.00	0.00	0.00	0.00	0.00	0.00
0.00	0.00	0.00	0.00	0.00	0.00	0.00	0.00	0.00
0.00	0.00	9.00	0.00	0.00	0.00	0.00	0.00	3.00
0.00	0.00	0.00	0.00	0.00	0.00	0.00	0.00	0.00
0.00	0.00	0.00	0.00	0.00	0.00	0.00	0.00	3.00
0.00	0.00	0.00	6.00	0.00	0.00	0.00	0.00	0.00
0.00	0.00	0.00	0.00	0.00	0.00	0.00	0.00	0.00
0.00	0.00	0.00	0.00	0.00	0.00	0.00	0.00	0.00

0.00	45.00	0.00	0.00	0.00	0.00	0.00	0.00	0.00
0.00	0.00	0.00	0.00	0.00	0.00	0.00	0.00	0.00
0.00	0.00	0.00	0.00	0.00	0.00	0.00	0.00	0.00
0.00	0.00	0.00	0.00	0.00	0.00	0.00	0.00	0.00
0.00	0.00	0.00	0.00	0.00	0.00	0.00	0.00	0.00
0.00	0.00	0.00	0.00	0.00	0.00	0.00	0.00	0.00
0.00	0.00	0.00	0.00	0.00	0.00	0.00	0.00	0.00
0.00	0.00	0.00	0.00	0.00	13.00	0.00	0.00	0.00
0.00	3.00	0.00	0.00	0.00	0.00	0.00	0.00	0.00
0.00	3.00	0.00	15.00	7.00	0.00	0.00	0.00	0.00
0.00	0.00	0.00	0.00	0.00	0.00	0.00	0.00	0.00
0.00	0.00	44.00	0.00	0.00	0.00	0.00	0.00	0.00
0.00	0.00	0.00	0.00	0.00	0.00	0.00	0.00	0.00
0.00	0.00	0.00	0.00	0.00	0.00	0.00	0.00	0.00
0.00	0.00	0.00	0.00	0.00	0.00	0.00	0.00	0.00
0.00	0.00	0.00	0.00	0.00	0.00	0.00	0.00	0.00
0.00	0.00	0.00	0.00	0.00	0.00	0.00	0.00	0.00
0.00	0.00	0.00	0.00	0.00	0.00	0.00	0.00	0.00
0.00	0.00	0.00	0.00	0.00	0.00	0.00	0.00	0.00
0.00	0.00	0.00	0.00	0.00	0.00	0.00	0.00	0.00
0.00	0.00	0.00	0.00	0.00	0.00	0.00	0.00	0.00
0.00	0.00	0.00	0.00	0.00	0.00	0.00	0.00	0.00
0.00	0.00	0.00	0.00	0.00	0.00	0.00	0.00	0.00
0.00	0.00	0.00	0.00	0.00	0.00	0.00	0.00	0.00
0.00	0.00	0.00	0.00	0.00	0.00	0.00	0.00	0.00
0.00	0.00	0.00	0.00	0.00	0.00	0.00	0.00	0.00
0.00	0.00	0.00	0.00	0.00	0.00	0.00	0.00	0.00
0.00	0.00	0.00	0.00	0.00	0.00	0.00	0.00	0.00
0.00	0.00	0.00	0.00	0.00	0.00	0.00	0.00	0.00
0.00	0.00	0.00	0.00	0.00	0.00	0.00	0.00	0.00
0.00	0.00	0.00	0.00	0.00	0.00	0.00	0.00	0.00
0.00	0.00	0.00	0.00	0.00	0.00	0.00	0.00	0.00
0.00	0.00	0.00	0.00	0.00	0.00	0.00	0.00	0.00
0.00	0.00	0.00	0.00	0.00	0.00	0.00	0.00	0.00
0.00	0.00	0.00	0.00	0.00	0.00	0.00	0.00	0.00
0.00	0.00	0.00	0.00	0.00	0.00	0.00	0.00	0.00
0.00	0.00	0.00	0.00	0.00	0.00	0.00	0.00	0.00

0.00	0.00	0.00	0.00	0.00	0.00	0.00	0.00	0.00
0.00	0.00	0.00	0.00	0.00	0.00	0.00	0.00	0.00
0.00	0.00	0.00	0.00	0.00	0.00	0.00	0.00	0.00
0.00	0.00	0.00	0.00	0.00	0.00	0.00	0.00	0.00
0.00	0.00	0.00	0.00	0.00	0.00	0.00	0.00	0.00
0.00	0.00	0.00	0.00	0.00	0.00	0.00	0.00	0.00
0.00	0.00	0.00	0.00	0.00	0.00	0.00	0.00	0.00
0.00	0.00	0.00	0.00	0.00	0.00	0.00	0.00	0.00
0.00	0.00	0.00	0.00	0.00	0.00	0.00	0.00	0.00
0.00	0.00	0.00	0.00	0.00	0.00	0.00	0.00	0.00
0.00	0.00	0.00	0.00	0.00	0.00	0.00	0.00	0.00
0.00	0.00	0.00	0.00	0.00	0.00	0.00	0.00	0.00
0.00	0.00	0.00	12.00	0.00	0.00	0.00	0.00	0.00
0.00	0.00	0.00	0.00	39.00	0.00	0.00	0.00	0.00
0.00	0.00	0.00	0.00	0.00	0.00	0.00	0.00	0.00
0.00	0.00	0.00	0.00	0.00	0.00	0.00	0.00	0.00
0.00	0.00	0.00	0.00	0.00	0.00	0.00	0.00	0.00
0.00	0.00	0.00	0.00	0.00	0.00	0.00	0.00	0.00
0.00	0.00	0.00	0.00	0.00	0.00	0.00	0.00	0.00
0.00	0.00	0.00	0.00	0.00	0.00	0.00	0.00	0.00
0.00	0.00	0.00	0.00	0.00	0.00	0.00	0.00	0.00
0.00	0.00	0.00	0.00	0.00	0.00	2.00	0.00	0.00
0.00	0.00	0.00	0.00	0.00	0.00	0.00	0.00	0.00
0.00	0.00	0.00	0.00	0.00	0.00	0.00	8.00	0.00
0.00	0.00	0.00	0.00	0.00	0.00	0.00	0.00	0.00
0.00	0.00	0.00	0.00	0.00	0.00	0.00	0.00	0.00
0.00	0.00	0.00	0.00	0.00	0.00	0.00	0.00	0.00
0.00	0.00	0.00	0.00	3.00	0.00	0.00	0.00	0.00
0.00	0.00	0.00	0.00	0.00	0.00	0.00	0.00	0.00
0.00	0.00	0.00	0.00	0.00	0.00	0.00	0.00	0.00
0.00	0.00	0.00	0.00	0.00	0.00	0.00	0.00	0.00
0.00	0.00	0.00	0.00	0.00	0.00	0.00	0.00	0.00
0.00	0.00	0.00	0.00	0.00	0.00	0.00	0.00	0.00
0.00	0.00	0.00	0.00	0.00	0.00	0.00	0.00	0.00
0.00	0.00	0.00	0.00	0.00	0.00	0.00	0.00	0.00
0.00	0.00	0.00	0.00	0.00	0.00	0.00	0.00	0.00
0.00	0.00	0.00	0.00	0.00	0.00	0.00	0.00	0.00

0.00	0.00	0.00	0.00	0.00	0.00	0.00	0.00	0.00
0.00	0.00	0.00	0.00	0.00	0.00	0.00	0.00	0.00
0.00	0.00	0.00	0.00	0.00	10.00	8.00	8.00	0.00
0.00	0.00	0.00	0.00	0.00	0.00	0.00	9.00	0.00
0.00	0.00	0.00	0.00	0.00	0.00	0.00	0.00	0.00
0.00	0.00	0.00	0.00	0.00	0.00	0.00	0.00	0.00
0.00	0.00	0.00	0.00	0.00	0.00	0.00	0.00	0.00
0.00	0.00	0.00	0.00	0.00	0.00	0.00	3.00	0.00
0.00	0.00	0.00	0.00	0.00	0.00	0.00	0.00	0.00
0.00	0.00	0.00	0.00	0.00	4.00	0.00	11.00	0.00
0.00	0.00	0.00	0.00	0.00	0.00	0.00	8.00	0.00
0.00	0.00	0.00	0.00	0.00	0.00	0.00	0.00	0.00
0.00	0.00	0.00	0.00	0.00	0.00	0.00	0.00	0.00
0.00	0.00	0.00	0.00	0.00	0.00	0.00	0.00	0.00
0.00	0.00	0.00	0.00	0.00	0.00	0.00	0.00	0.00
0.00	0.00	0.00	0.00	0.00	36.00	0.00	0.00	0.00
0.00	0.00	0.00	0.00	0.00	0.00	0.00	0.00	0.00
0.00	0.00	0.00	0.00	0.00	0.00	0.00	0.00	0.00
0.00	0.00	0.00	0.00	0.00	0.00	0.00	0.00	0.00
0.00	0.00	0.00	0.00	0.00	0.00	0.00	0.00	0.00
0.00	0.00	0.00	0.00	0.00	0.00	0.00	0.00	0.00
0.00	0.00	0.00	0.00	0.00	0.00	0.00	0.00	0.00
0.00	0.00	0.00	0.00	0.00	0.00	0.00	0.00	0.00
0.00	0.00	0.00	0.00	0.00	0.00	0.00	0.00	0.00
0.00	0.00	0.00	0.00	0.00	0.00	0.00	0.00	0.00
0.00	0.00	0.00	0.00	0.00	0.00	0.00	0.00	0.00
0.00	0.00	0.00	0.00	0.00	0.00	0.00	0.00	0.00
0.00	0.00	0.00	0.00	0.00	0.00	0.00	0.00	0.00
0.00	0.00	0.00	0.00	0.00	0.00	0.00	0.00	0.00
0.00	0.00	0.00	0.00	0.00	0.00	0.00	0.00	0.00
0.00	0.00	0.00	0.00	19.00	0.00	0.00	0.00	0.00
0.00	0.00	0.00	0.00	0.00	0.00	0.00	0.00	0.00
0.00	0.00	0.00	0.00	0.00	0.00	0.00	0.00	0.00
0.00	0.00	0.00	0.00	0.00	0.00	0.00	0.00	0.00
0.00	0.00	0.00	0.00	33.00	0.00	0.00	0.00	0.00
0.00	0.00	0.00	0.00	0.00	0.00	0.00	6.00	0.00
0.00	0.00	0.00	0.00	0.00	0.00	0.00	0.00	0.00

0.00	0.00	0.00	0.00	0.00	0.00	0.00	0.00	0.00
0.00	0.00	0.00	0.00	0.00	0.00	0.00	0.00	0.00
0.00	0.00	0.00	0.00	0.00	0.00	0.00	0.00	0.00
0.00	0.00	0.00	0.00	0.00	0.00	0.00	0.00	0.00
0.00	0.00	0.00	0.00	0.00	0.00	0.00	0.00	0.00
0.00	0.00	0.00	0.00	0.00	0.00	0.00	0.00	0.00
0.00	0.00	0.00	0.00	0.00	0.00	0.00	0.00	0.00
0.00	0.00	0.00	0.00	0.00	0.00	0.00	0.00	0.00
0.00	0.00	0.00	0.00	0.00	0.00	0.00	0.00	0.00
0.00	0.00	0.00	0.00	0.00	0.00	0.00	0.00	0.00
0.00	0.00	0.00	0.00	0.00	0.00	0.00	0.00	0.00
0.00	0.00	0.00	0.00	0.00	0.00	0.00	0.00	0.00
0.00	0.00	0.00	9.00	0.00	0.00	0.00	0.00	0.00
0.00	0.00	0.00	0.00	8.00	0.00	0.00	0.00	0.00
0.00	0.00	0.00	0.00	0.00	0.00	0.00	0.00	0.00
0.00	0.00	0.00	0.00	0.00	32.00	0.00	0.00	0.00
0.00	0.00	32.00	0.00	0.00	0.00	0.00	0.00	0.00
0.00	0.00	0.00	0.00	0.00	0.00	0.00	0.00	0.00
0.00	0.00	0.00	0.00	0.00	0.00	0.00	0.00	0.00
0.00	0.00	0.00	0.00	0.00	0.00	0.00	0.00	0.00
0.00	0.00	0.00	0.00	0.00	0.00	0.00	0.00	0.00
0.00	0.00	0.00	0.00	0.00	0.00	0.00	0.00	0.00
0.00	0.00	0.00	0.00	0.00	0.00	0.00	0.00	0.00
0.00	0.00	0.00	0.00	0.00	0.00	0.00	0.00	0.00
0.00	0.00	0.00	0.00	0.00	0.00	0.00	0.00	0.00
0.00	0.00	0.00	0.00	0.00	0.00	0.00	0.00	0.00
0.00	0.00	0.00	10.00	0.00	0.00	0.00	0.00	0.00
0.00	0.00	0.00	12.00	0.00	0.00	0.00	0.00	0.00
0.00	0.00	0.00	0.00	31.00	0.00	0.00	0.00	0.00
0.00	0.00	0.00	0.00	0.00	0.00	0.00	0.00	0.00
0.00	0.00	0.00	0.00	0.00	0.00	0.00	0.00	0.00
0.00	0.00	0.00	0.00	0.00	31.00	0.00	0.00	0.00
0.00	0.00	0.00	0.00	0.00	0.00	0.00	0.00	0.00
0.00	0.00	0.00	0.00	0.00	0.00	0.00	0.00	0.00
0.00	0.00	0.00	0.00	0.00	0.00	0.00	0.00	0.00
0.00	0.00	0.00	0.00	0.00	0.00	0.00	0.00	0.00
0.00	0.00	0.00	0.00	0.00	0.00	0.00	0.00	0.00

0.00	0.00	0.00	0.00	0.00	0.00	0.00	0.00	0.00
0.00	0.00	0.00	0.00	0.00	0.00	0.00	0.00	0.00
0.00	0.00	0.00	0.00	0.00	0.00	0.00	0.00	0.00
0.00	0.00	0.00	0.00	0.00	0.00	0.00	0.00	0.00
0.00	0.00	0.00	5.00	12.00	0.00	0.00	0.00	0.00
0.00	0.00	0.00	0.00	0.00	0.00	0.00	0.00	0.00
0.00	0.00	0.00	0.00	0.00	0.00	0.00	4.00	0.00
0.00	0.00	0.00	0.00	0.00	0.00	0.00	0.00	0.00
0.00	0.00	0.00	0.00	0.00	0.00	0.00	0.00	0.00
0.00	0.00	0.00	0.00	0.00	0.00	0.00	0.00	0.00
0.00	0.00	0.00	0.00	0.00	0.00	0.00	0.00	0.00
0.00	0.00	0.00	0.00	0.00	0.00	0.00	0.00	0.00
0.00	0.00	0.00	0.00	0.00	0.00	0.00	0.00	0.00
0.00	0.00	0.00	0.00	0.00	0.00	0.00	0.00	0.00
0.00	0.00	0.00	0.00	0.00	0.00	0.00	0.00	0.00
0.00	0.00	0.00	0.00	0.00	0.00	0.00	0.00	0.00
0.00	0.00	0.00	0.00	0.00	0.00	0.00	0.00	0.00
0.00	0.00	0.00	0.00	6.00	0.00	0.00	0.00	0.00
0.00	0.00	0.00	0.00	0.00	0.00	0.00	0.00	0.00
0.00	0.00	0.00	6.00	0.00	0.00	0.00	0.00	0.00
0.00	0.00	0.00	0.00	0.00	0.00	0.00	4.00	0.00
0.00	0.00	0.00	0.00	0.00	0.00	0.00	0.00	0.00
0.00	0.00	0.00	0.00	0.00	0.00	0.00	0.00	0.00
0.00	0.00	0.00	0.00	0.00	0.00	0.00	0.00	0.00
0.00	0.00	0.00	0.00	0.00	0.00	0.00	0.00	0.00
0.00	0.00	0.00	0.00	0.00	0.00	0.00	0.00	0.00
0.00	0.00	0.00	0.00	0.00	0.00	0.00	2.00	0.00
0.00	0.00	0.00	0.00	0.00	0.00	0.00	0.00	0.00
0.00	0.00	0.00	0.00	0.00	0.00	0.00	0.00	0.00
0.00	0.00	0.00	0.00	0.00	6.00	0.00	9.00	0.00
0.00	0.00	0.00	0.00	0.00	0.00	0.00	0.00	0.00
0.00	0.00	0.00	0.00	0.00	0.00	0.00	0.00	0.00
0.00	0.00	0.00	0.00	0.00	0.00	0.00	0.00	0.00
0.00	0.00	0.00	0.00	0.00	0.00	0.00	0.00	0.00
0.00	0.00	0.00	0.00	0.00	0.00	0.00	0.00	0.00
0.00	0.00	0.00	0.00	0.00	0.00	0.00	0.00	0.00
0.00	0.00	0.00	0.00	0.00	0.00	0.00	0.00	0.00

0.00	0.00	0.00	0.00	0.00	0.00	0.00	0.00	0.00
0.00	0.00	0.00	0.00	0.00	0.00	0.00	0.00	0.00
0.00	0.00	0.00	0.00	0.00	0.00	0.00	0.00	0.00
0.00	0.00	0.00	0.00	0.00	0.00	0.00	0.00	0.00
0.00	0.00	0.00	0.00	0.00	0.00	0.00	0.00	0.00
0.00	0.00	0.00	0.00	0.00	0.00	0.00	0.00	0.00
0.00	0.00	0.00	0.00	0.00	0.00	0.00	0.00	0.00
0.00	0.00	0.00	4.00	0.00	0.00	3.00	4.00	0.00
0.00	0.00	0.00	0.00	0.00	10.00	0.00	9.00	0.00
0.00	0.00	0.00	0.00	10.00	0.00	0.00	0.00	0.00
0.00	0.00	0.00	27.00	0.00	0.00	0.00	0.00	0.00
0.00	0.00	0.00	0.00	0.00	0.00	0.00	7.00	0.00
0.00	0.00	0.00	0.00	0.00	0.00	0.00	0.00	0.00
0.00	0.00	0.00	0.00	0.00	0.00	0.00	0.00	0.00
0.00	0.00	0.00	0.00	0.00	0.00	0.00	0.00	0.00
0.00	0.00	0.00	0.00	0.00	0.00	0.00	0.00	0.00
0.00	0.00	0.00	0.00	0.00	0.00	0.00	0.00	0.00
0.00	0.00	0.00	0.00	0.00	0.00	0.00	0.00	0.00
0.00	0.00	0.00	0.00	0.00	0.00	0.00	0.00	0.00
0.00	0.00	0.00	0.00	0.00	0.00	0.00	0.00	0.00
0.00	0.00	0.00	0.00	0.00	0.00	0.00	0.00	0.00
0.00	0.00	0.00	0.00	0.00	0.00	0.00	0.00	0.00
0.00	0.00	0.00	0.00	0.00	0.00	0.00	0.00	0.00
0.00	0.00	0.00	0.00	0.00	0.00	0.00	0.00	0.00
0.00	0.00	0.00	0.00	0.00	0.00	0.00	0.00	0.00
0.00	0.00	0.00	0.00	0.00	0.00	0.00	0.00	0.00
0.00	5.00	0.00	0.00	5.00	0.00	0.00	0.00	0.00
0.00	0.00	0.00	0.00	0.00	0.00	0.00	0.00	0.00
0.00	0.00	0.00	0.00	0.00	0.00	0.00	0.00	0.00
0.00	0.00	0.00	0.00	0.00	0.00	0.00	0.00	0.00
0.00	0.00	0.00	0.00	0.00	5.00	5.00	0.00	0.00
0.00	0.00	0.00	0.00	0.00	0.00	0.00	0.00	0.00
0.00	0.00	0.00	0.00	0.00	0.00	0.00	0.00	0.00
0.00	0.00	0.00	0.00	0.00	0.00	0.00	0.00	0.00
0.00	0.00	0.00	0.00	0.00	0.00	0.00	0.00	0.00
0.00	0.00	0.00	0.00	0.00	0.00	0.00	0.00	0.00
0.00	0.00	0.00	0.00	0.00	0.00	0.00	0.00	0.00

0.00	0.00	0.00	0.00	0.00	0.00	0.00	0.00	0.00
0.00	0.00	0.00	0.00	0.00	0.00	0.00	0.00	0.00
0.00	0.00	0.00	0.00	0.00	0.00	0.00	0.00	0.00
0.00	0.00	0.00	0.00	0.00	0.00	0.00	15.00	0.00
0.00	0.00	0.00	0.00	0.00	6.00	0.00	6.00	0.00
0.00	0.00	0.00	25.00	0.00	0.00	0.00	0.00	0.00
0.00	0.00	0.00	0.00	0.00	0.00	0.00	0.00	0.00
0.00	0.00	0.00	0.00	0.00	0.00	0.00	0.00	0.00
0.00	0.00	0.00	0.00	0.00	0.00	0.00	0.00	0.00
0.00	0.00	0.00	0.00	0.00	0.00	0.00	0.00	0.00
0.00	0.00	0.00	0.00	0.00	0.00	0.00	0.00	0.00
0.00	0.00	0.00	0.00	0.00	0.00	0.00	0.00	0.00
0.00	0.00	0.00	0.00	0.00	0.00	0.00	0.00	0.00
0.00	0.00	0.00	0.00	0.00	0.00	0.00	0.00	0.00
0.00	0.00	0.00	0.00	0.00	0.00	0.00	0.00	0.00
0.00	0.00	0.00	0.00	0.00	0.00	0.00	0.00	0.00
0.00	0.00	0.00	0.00	0.00	0.00	0.00	0.00	0.00
0.00	0.00	0.00	0.00	0.00	0.00	0.00	0.00	0.00
0.00	0.00	0.00	0.00	0.00	0.00	0.00	0.00	0.00
0.00	0.00	0.00	0.00	0.00	0.00	0.00	0.00	0.00
0.00	0.00	0.00	0.00	0.00	0.00	0.00	0.00	0.00
0.00	0.00	0.00	0.00	5.00	0.00	3.00	0.00	0.00
0.00	0.00	0.00	0.00	0.00	0.00	0.00	0.00	0.00
0.00	0.00	0.00	0.00	0.00	0.00	0.00	0.00	0.00
0.00	0.00	0.00	0.00	0.00	0.00	0.00	0.00	0.00
0.00	0.00	0.00	0.00	0.00	6.00	0.00	0.00	0.00
0.00	0.00	0.00	0.00	0.00	0.00	0.00	0.00	0.00
0.00	0.00	0.00	0.00	0.00	0.00	0.00	0.00	0.00
0.00	0.00	0.00	0.00	0.00	0.00	0.00	0.00	0.00
0.00	0.00	0.00	0.00	0.00	0.00	0.00	0.00	0.00
0.00	0.00	0.00	0.00	0.00	0.00	0.00	0.00	0.00
15.00	0.00	0.00	0.00	0.00	0.00	0.00	0.00	0.00
0.00	0.00	0.00	0.00	0.00	0.00	0.00	0.00	0.00
0.00	0.00	0.00	0.00	0.00	0.00	0.00	0.00	0.00
0.00	0.00	0.00	23.00	0.00	0.00	0.00	0.00	0.00
0.00	0.00	0.00	4.00	0.00	0.00	3.00	0.00	0.00
0.00	0.00	0.00	0.00	0.00	0.00	0.00	0.00	0.00

0.00	0.00	0.00	0.00	0.00	0.00	0.00	0.00	0.00
0.00	0.00	0.00	0.00	0.00	0.00	0.00	0.00	0.00
0.00	0.00	0.00	0.00	0.00	0.00	0.00	0.00	0.00
0.00	0.00	0.00	0.00	0.00	0.00	0.00	0.00	0.00
0.00	0.00	0.00	0.00	0.00	0.00	0.00	0.00	0.00
16.00	0.00	7.00	0.00	0.00	0.00	0.00	0.00	0.00
0.00	0.00	0.00	0.00	0.00	0.00	0.00	0.00	0.00
0.00	0.00	0.00	0.00	0.00	3.00	0.00	0.00	0.00
0.00	0.00	0.00	0.00	0.00	0.00	0.00	0.00	0.00
0.00	0.00	0.00	0.00	0.00	0.00	0.00	0.00	0.00
0.00	0.00	0.00	0.00	0.00	0.00	0.00	0.00	0.00
0.00	0.00	0.00	0.00	0.00	0.00	0.00	0.00	0.00
0.00	0.00	0.00	0.00	0.00	4.00	0.00	5.00	0.00
0.00	0.00	0.00	0.00	0.00	0.00	0.00	0.00	0.00
0.00	0.00	0.00	0.00	8.00	0.00	0.00	0.00	0.00
0.00	0.00	0.00	11.00	0.00	0.00	0.00	0.00	0.00
0.00	0.00	0.00	8.00	0.00	0.00	0.00	0.00	0.00
0.00	0.00	0.00	0.00	0.00	0.00	0.00	5.00	0.00
0.00	0.00	0.00	0.00	0.00	6.00	0.00	0.00	0.00
0.00	0.00	0.00	0.00	0.00	0.00	0.00	0.00	0.00
0.00	0.00	0.00	0.00	0.00	0.00	0.00	0.00	0.00
0.00	0.00	0.00	0.00	0.00	0.00	0.00	0.00	0.00
0.00	0.00	0.00	0.00	0.00	9.00	0.00	13.00	0.00
0.00	0.00	0.00	0.00	0.00	10.00	0.00	12.00	0.00
0.00	0.00	0.00	0.00	0.00	0.00	0.00	0.00	0.00
0.00	0.00	0.00	0.00	0.00	0.00	0.00	0.00	0.00
0.00	0.00	0.00	0.00	0.00	0.00	0.00	0.00	0.00
0.00	0.00	0.00	0.00	0.00	0.00	0.00	0.00	0.00
0.00	0.00	0.00	0.00	0.00	0.00	0.00	0.00	0.00
0.00	0.00	0.00	0.00	0.00	0.00	0.00	0.00	0.00
0.00	0.00	0.00	0.00	0.00	0.00	0.00	0.00	0.00
0.00	0.00	0.00	0.00	0.00	0.00	0.00	0.00	0.00
0.00	0.00	0.00	0.00	0.00	0.00	0.00	0.00	0.00
0.00	0.00	0.00	0.00	0.00	0.00	0.00	0.00	6.00
0.00	0.00	0.00	0.00	0.00	0.00	0.00	0.00	0.00
0.00	0.00	0.00	0.00	4.00	0.00	0.00	0.00	0.00
2.00	0.00	11.00	0.00	0.00	0.00	0.00	0.00	8.00

0.00	0.00	0.00	0.00	21.00	0.00	0.00	0.00	0.00
0.00	0.00	0.00	0.00	0.00	0.00	0.00	0.00	0.00
0.00	0.00	0.00	0.00	0.00	0.00	0.00	0.00	0.00
0.00	0.00	0.00	0.00	0.00	4.00	0.00	0.00	0.00
0.00	0.00	0.00	0.00	0.00	0.00	0.00	0.00	0.00
0.00	0.00	0.00	0.00	0.00	0.00	0.00	0.00	0.00
0.00	0.00	0.00	0.00	0.00	0.00	0.00	0.00	0.00
0.00	0.00	0.00	0.00	0.00	0.00	0.00	0.00	0.00
0.00	0.00	0.00	0.00	0.00	0.00	0.00	0.00	0.00
0.00	0.00	0.00	0.00	0.00	0.00	0.00	21.00	0.00
0.00	0.00	0.00	0.00	0.00	0.00	0.00	0.00	0.00
0.00	0.00	0.00	0.00	0.00	0.00	0.00	0.00	0.00
0.00	0.00	0.00	0.00	0.00	0.00	5.00	0.00	0.00
0.00	0.00	0.00	0.00	0.00	0.00	0.00	0.00	0.00
0.00	0.00	0.00	0.00	0.00	0.00	0.00	0.00	0.00
0.00	0.00	0.00	0.00	0.00	0.00	0.00	0.00	0.00
0.00	0.00	0.00	0.00	0.00	0.00	0.00	0.00	0.00
0.00	0.00	0.00	0.00	4.00	0.00	0.00	0.00	0.00
0.00	0.00	0.00	4.00	0.00	3.00	0.00	0.00	0.00
0.00	0.00	0.00	0.00	0.00	0.00	0.00	0.00	0.00
0.00	0.00	0.00	0.00	0.00	0.00	0.00	0.00	0.00
0.00	0.00	0.00	0.00	0.00	0.00	0.00	0.00	0.00
0.00	0.00	0.00	0.00	0.00	0.00	0.00	0.00	0.00
0.00	0.00	0.00	0.00	0.00	0.00	0.00	0.00	0.00
0.00	0.00	0.00	0.00	0.00	0.00	0.00	0.00	0.00
0.00	0.00	0.00	0.00	0.00	0.00	0.00	0.00	0.00
0.00	0.00	0.00	0.00	0.00	0.00	0.00	0.00	0.00
0.00	0.00	0.00	0.00	0.00	0.00	0.00	0.00	0.00
0.00	0.00	0.00	0.00	0.00	0.00	0.00	0.00	0.00
0.00	5.00	0.00	0.00	0.00	0.00	0.00	3.00	0.00
0.00	0.00	0.00	0.00	0.00	9.00	0.00	11.00	0.00
0.00	0.00	0.00	0.00	0.00	0.00	0.00	0.00	0.00
0.00	0.00	0.00	0.00	0.00	0.00	0.00	0.00	0.00
0.00	0.00	0.00	0.00	0.00	0.00	0.00	0.00	0.00
0.00	0.00	0.00	0.00	0.00	0.00	0.00	0.00	0.00
0.00	0.00	0.00	0.00	0.00	0.00	0.00	0.00	0.00
0.00	0.00	0.00	0.00	0.00	0.00	0.00	0.00	0.00

0.00	0.00	0.00	0.00	0.00	0.00	0.00	0.00	0.00
0.00	0.00	0.00	0.00	0.00	0.00	0.00	0.00	0.00
0.00	0.00	0.00	0.00	0.00	0.00	0.00	0.00	0.00
0.00	0.00	0.00	0.00	0.00	0.00	0.00	0.00	0.00
0.00	0.00	0.00	0.00	0.00	0.00	0.00	0.00	0.00
0.00	0.00	0.00	0.00	0.00	0.00	0.00	0.00	0.00
0.00	0.00	0.00	4.00	6.00	0.00	0.00	0.00	0.00
0.00	0.00	0.00	0.00	2.00	0.00	0.00	0.00	0.00
0.00	0.00	0.00	2.00	0.00	0.00	0.00	0.00	0.00
0.00	0.00	0.00	0.00	0.00	0.00	0.00	4.00	0.00
0.00	0.00	0.00	0.00	0.00	0.00	0.00	0.00	0.00
0.00	0.00	0.00	0.00	0.00	0.00	0.00	0.00	0.00
0.00	0.00	0.00	0.00	0.00	0.00	0.00	0.00	0.00
0.00	0.00	0.00	0.00	0.00	0.00	0.00	0.00	0.00
0.00	0.00	0.00	0.00	0.00	0.00	0.00	0.00	0.00
0.00	0.00	0.00	0.00	0.00	0.00	0.00	0.00	0.00
0.00	0.00	0.00	0.00	0.00	5.00	5.00	5.00	0.00
0.00	0.00	0.00	0.00	0.00	19.00	0.00	0.00	0.00
0.00	0.00	0.00	0.00	0.00	0.00	0.00	0.00	0.00
0.00	0.00	0.00	0.00	0.00	0.00	0.00	0.00	0.00
0.00	0.00	0.00	0.00	0.00	0.00	0.00	0.00	0.00
0.00	0.00	0.00	0.00	4.00	0.00	0.00	0.00	0.00
0.00	0.00	0.00	0.00	0.00	0.00	0.00	0.00	0.00
0.00	0.00	0.00	0.00	0.00	0.00	0.00	0.00	0.00
0.00	0.00	0.00	0.00	0.00	0.00	0.00	0.00	0.00
0.00	0.00	0.00	0.00	0.00	0.00	0.00	0.00	0.00
0.00	0.00	0.00	0.00	0.00	0.00	0.00	0.00	0.00
0.00	0.00	0.00	0.00	0.00	0.00	0.00	0.00	0.00
0.00	0.00	0.00	0.00	0.00	0.00	0.00	0.00	0.00
0.00	0.00	0.00	0.00	0.00	0.00	0.00	0.00	0.00
0.00	0.00	0.00	0.00	0.00	0.00	0.00	0.00	0.00
0.00	0.00	0.00	0.00	0.00	0.00	0.00	0.00	0.00
0.00	0.00	0.00	0.00	0.00	0.00	0.00	0.00	0.00
0.00	0.00	0.00	0.00	0.00	0.00	0.00	0.00	0.00
0.00	0.00	0.00	0.00	0.00	0.00	0.00	0.00	0.00
0.00	0.00	0.00	0.00	0.00	0.00	0.00	0.00	0.00
0.00	0.00	0.00	0.00	0.00	0.00	0.00	3.00	0.00

0.00	0.00	0.00	0.00	8.00	0.00	0.00	0.00	0.00
0.00	0.00	0.00	3.00	5.00	0.00	0.00	0.00	0.00
0.00	0.00	0.00	0.00	4.00	0.00	0.00	0.00	0.00
0.00	0.00	0.00	0.00	10.00	0.00	0.00	0.00	0.00
0.00	0.00	0.00	0.00	7.00	0.00	0.00	0.00	0.00
0.00	0.00	0.00	0.00	5.00	0.00	0.00	0.00	0.00
0.00	0.00	0.00	0.00	0.00	0.00	0.00	4.00	0.00
0.00	0.00	0.00	0.00	0.00	0.00	0.00	0.00	0.00
0.00	0.00	0.00	0.00	0.00	0.00	0.00	0.00	0.00
0.00	0.00	0.00	0.00	0.00	0.00	0.00	0.00	0.00
0.00	0.00	0.00	0.00	0.00	0.00	0.00	0.00	0.00
0.00	0.00	0.00	0.00	0.00	0.00	0.00	0.00	0.00
0.00	0.00	0.00	0.00	0.00	0.00	0.00	18.00	0.00
0.00	0.00	0.00	0.00	0.00	0.00	0.00	0.00	0.00
0.00	0.00	0.00	0.00	0.00	0.00	0.00	0.00	0.00
0.00	0.00	0.00	0.00	0.00	0.00	0.00	0.00	0.00
0.00	0.00	0.00	0.00	0.00	0.00	0.00	0.00	0.00
0.00	0.00	0.00	0.00	0.00	0.00	0.00	0.00	0.00
0.00	0.00	0.00	0.00	0.00	0.00	0.00	0.00	0.00
0.00	0.00	0.00	0.00	0.00	0.00	0.00	0.00	0.00
0.00	0.00	0.00	0.00	0.00	0.00	0.00	0.00	0.00
0.00	0.00	0.00	0.00	0.00	0.00	0.00	0.00	0.00
0.00	0.00	0.00	0.00	0.00	0.00	0.00	0.00	0.00
0.00	0.00	0.00	8.00	0.00	0.00	0.00	0.00	0.00
0.00	0.00	0.00	6.00	0.00	0.00	0.00	0.00	0.00
0.00	0.00	0.00	0.00	0.00	0.00	0.00	0.00	0.00
0.00	0.00	0.00	17.00	0.00	0.00	0.00	0.00	0.00
0.00	0.00	0.00	0.00	0.00	0.00	0.00	0.00	0.00
0.00	0.00	0.00	0.00	0.00	0.00	0.00	0.00	0.00
0.00	0.00	0.00	0.00	0.00	0.00	0.00	0.00	0.00
0.00	0.00	0.00	0.00	0.00	0.00	0.00	0.00	0.00
0.00	0.00	0.00	0.00	0.00	0.00	0.00	0.00	0.00
0.00	0.00	0.00	0.00	0.00	0.00	0.00	0.00	0.00
0.00	0.00	0.00	0.00	0.00	0.00	0.00	0.00	0.00
0.00	0.00	0.00	0.00	0.00	0.00	0.00	0.00	0.00
0.00	0.00	0.00	0.00	0.00	0.00	0.00	0.00	0.00
0.00	0.00	0.00	0.00	0.00	0.00	0.00	0.00	0.00

0.00	0.00	0.00	0.00	0.00	0.00	0.00	0.00	0.00
0.00	0.00	0.00	0.00	0.00	0.00	0.00	0.00	0.00
0.00	0.00	0.00	0.00	0.00	0.00	0.00	0.00	0.00
0.00	0.00	0.00	0.00	0.00	0.00	0.00	0.00	0.00
0.00	0.00	0.00	0.00	0.00	0.00	0.00	17.00	0.00
0.00	0.00	0.00	0.00	0.00	0.00	0.00	17.00	0.00
0.00	0.00	0.00	0.00	0.00	0.00	17.00	0.00	0.00
0.00	0.00	0.00	0.00	0.00	17.00	0.00	0.00	0.00
0.00	0.00	17.00	0.00	0.00	0.00	0.00	0.00	0.00
0.00	0.00	0.00	0.00	0.00	0.00	0.00	0.00	0.00
0.00	0.00	0.00	0.00	0.00	0.00	0.00	0.00	0.00
0.00	0.00	0.00	0.00	0.00	0.00	0.00	0.00	0.00
0.00	0.00	0.00	0.00	0.00	0.00	0.00	0.00	0.00
0.00	0.00	0.00	0.00	0.00	0.00	0.00	0.00	0.00
0.00	0.00	0.00	0.00	0.00	0.00	0.00	0.00	0.00
0.00	0.00	0.00	0.00	0.00	0.00	0.00	0.00	0.00
0.00	0.00	0.00	0.00	0.00	0.00	0.00	0.00	0.00
0.00	0.00	0.00	0.00	0.00	0.00	0.00	0.00	0.00
0.00	0.00	0.00	0.00	0.00	0.00	0.00	0.00	0.00
0.00	0.00	0.00	0.00	0.00	0.00	0.00	0.00	0.00
0.00	0.00	0.00	0.00	0.00	0.00	0.00	0.00	0.00
0.00	0.00	0.00	0.00	0.00	0.00	0.00	0.00	0.00
0.00	0.00	0.00	0.00	0.00	0.00	0.00	7.00	0.00
0.00	0.00	0.00	3.00	0.00	0.00	0.00	0.00	0.00
0.00	0.00	0.00	0.00	6.00	0.00	0.00	0.00	0.00
0.00	0.00	0.00	0.00	9.00	0.00	0.00	0.00	0.00
0.00	0.00	0.00	6.00	0.00	0.00	0.00	0.00	0.00
0.00	0.00	0.00	0.00	0.00	0.00	0.00	0.00	0.00
0.00	0.00	0.00	0.00	0.00	0.00	0.00	0.00	0.00
0.00	0.00	0.00	0.00	0.00	0.00	0.00	0.00	0.00
0.00	0.00	0.00	0.00	0.00	0.00	0.00	0.00	0.00
0.00	0.00	0.00	0.00	0.00	0.00	0.00	0.00	0.00
0.00	0.00	0.00	0.00	0.00	0.00	0.00	0.00	0.00
0.00	0.00	0.00	0.00	0.00	0.00	0.00	0.00	0.00
0.00	0.00	0.00	0.00	0.00	0.00	0.00	0.00	0.00
0.00	0.00	0.00	0.00	0.00	0.00	0.00	8.00	0.00
0.00	0.00	0.00	0.00	0.00	0.00	16.00	0.00	0.00

0.00	0.00	0.00	0.00	0.00	16.00	0.00	0.00	0.00
0.00	0.00	0.00	0.00	0.00	16.00	0.00	0.00	0.00
0.00	0.00	0.00	0.00	0.00	0.00	0.00	0.00	0.00
0.00	0.00	0.00	0.00	0.00	0.00	0.00	0.00	0.00
0.00	0.00	0.00	0.00	0.00	0.00	0.00	0.00	0.00
0.00	0.00	0.00	0.00	0.00	0.00	0.00	0.00	0.00
0.00	0.00	0.00	0.00	0.00	0.00	0.00	0.00	0.00
0.00	0.00	0.00	0.00	0.00	0.00	0.00	0.00	0.00
0.00	0.00	0.00	0.00	0.00	0.00	0.00	0.00	0.00
0.00	0.00	0.00	0.00	0.00	0.00	0.00	0.00	0.00
0.00	0.00	0.00	0.00	0.00	10.00	0.00	0.00	0.00
0.00	0.00	0.00	0.00	0.00	0.00	0.00	0.00	0.00
0.00	0.00	0.00	0.00	0.00	0.00	0.00	0.00	0.00
0.00	0.00	0.00	0.00	0.00	0.00	0.00	0.00	0.00
0.00	0.00	0.00	0.00	0.00	0.00	0.00	0.00	0.00
0.00	0.00	0.00	0.00	0.00	0.00	0.00	0.00	0.00
0.00	0.00	0.00	0.00	0.00	0.00	0.00	0.00	0.00
0.00	0.00	0.00	0.00	0.00	0.00	0.00	0.00	0.00
0.00	0.00	0.00	0.00	0.00	0.00	0.00	0.00	0.00
0.00	0.00	0.00	0.00	0.00	0.00	0.00	0.00	0.00
0.00	0.00	0.00	0.00	2.00	0.00	0.00	0.00	0.00
0.00	0.00	0.00	4.00	3.00	0.00	0.00	0.00	0.00
0.00	0.00	0.00	0.00	2.00	0.00	0.00	0.00	0.00
0.00	0.00	0.00	0.00	0.00	0.00	6.00	0.00	0.00
0.00	0.00	0.00	0.00	0.00	0.00	0.00	0.00	0.00
0.00	0.00	0.00	0.00	0.00	0.00	0.00	5.00	0.00
0.00	0.00	0.00	0.00	0.00	0.00	0.00	0.00	0.00
0.00	0.00	0.00	0.00	0.00	0.00	0.00	0.00	0.00
0.00	0.00	0.00	0.00	0.00	0.00	0.00	0.00	0.00
0.00	0.00	0.00	0.00	0.00	0.00	0.00	0.00	0.00
0.00	0.00	0.00	0.00	0.00	0.00	0.00	0.00	0.00
0.00	0.00	0.00	0.00	0.00	0.00	0.00	0.00	0.00
0.00	0.00	0.00	0.00	0.00	0.00	0.00	0.00	0.00
0.00	0.00	0.00	0.00	0.00	0.00	0.00	0.00	0.00
0.00	0.00	0.00	0.00	0.00	0.00	0.00	0.00	0.00
0.00	0.00	0.00	0.00	0.00	0.00	0.00	0.00	0.00
0.00	0.00	0.00	0.00	0.00	0.00	0.00	0.00	0.00

0.00	0.00	0.00	0.00	0.00	0.00	0.00	0.00	0.00
0.00	0.00	0.00	0.00	0.00	0.00	0.00	0.00	0.00
0.00	0.00	0.00	0.00	0.00	0.00	0.00	0.00	0.00
0.00	0.00	0.00	0.00	0.00	0.00	0.00	0.00	0.00
0.00	0.00	0.00	0.00	0.00	0.00	0.00	4.00	0.00
0.00	0.00	0.00	2.00	0.00	0.00	0.00	0.00	0.00
0.00	0.00	0.00	0.00	0.00	0.00	0.00	0.00	0.00
0.00	0.00	0.00	0.00	0.00	0.00	0.00	0.00	0.00
0.00	0.00	0.00	0.00	0.00	0.00	0.00	0.00	0.00
0.00	0.00	0.00	0.00	0.00	0.00	0.00	0.00	0.00
0.00	0.00	0.00	0.00	0.00	0.00	0.00	0.00	0.00
0.00	0.00	0.00	0.00	0.00	0.00	0.00	0.00	0.00
0.00	0.00	0.00	0.00	0.00	0.00	0.00	0.00	0.00
0.00	0.00	0.00	0.00	0.00	0.00	0.00	0.00	0.00
0.00	0.00	0.00	0.00	0.00	7.00	0.00	7.00	0.00
0.00	0.00	0.00	0.00	0.00	0.00	0.00	14.00	0.00
0.00	0.00	0.00	0.00	0.00	0.00	0.00	0.00	0.00
0.00	0.00	0.00	0.00	0.00	0.00	0.00	0.00	0.00
0.00	0.00	0.00	0.00	0.00	0.00	0.00	0.00	0.00
0.00	0.00	0.00	0.00	0.00	0.00	0.00	0.00	0.00
0.00	0.00	0.00	0.00	0.00	0.00	0.00	0.00	0.00
0.00	0.00	0.00	0.00	0.00	0.00	0.00	0.00	0.00
0.00	0.00	0.00	0.00	0.00	0.00	0.00	0.00	0.00
0.00	0.00	0.00	0.00	0.00	0.00	0.00	0.00	0.00
0.00	0.00	0.00	0.00	0.00	0.00	0.00	0.00	0.00
0.00	0.00	0.00	0.00	0.00	0.00	0.00	0.00	0.00
0.00	0.00	0.00	0.00	0.00	0.00	0.00	0.00	0.00
0.00	0.00	0.00	0.00	0.00	0.00	0.00	0.00	0.00
0.00	0.00	0.00	0.00	0.00	0.00	0.00	0.00	0.00
0.00	0.00	0.00	0.00	0.00	0.00	0.00	0.00	0.00
0.00	0.00	0.00	0.00	0.00	0.00	0.00	0.00	0.00
0.00	0.00	0.00	0.00	0.00	0.00	0.00	0.00	0.00
0.00	0.00	0.00	0.00	0.00	0.00	0.00	0.00	0.00
0.00	0.00	0.00	0.00	0.00	0.00	0.00	0.00	0.00
0.00	0.00	0.00	0.00	0.00	0.00	0.00	0.00	0.00
0.00	0.00	0.00	0.00	0.00	0.00	0.00	0.00	0.00
0.00	0.00	0.00	0.00	0.00	0.00	0.00	0.00	0.00

0.00	0.00	0.00	0.00	0.00	0.00	0.00	0.00	0.00
5.00	0.00	4.00	0.00	0.00	0.00	0.00	0.00	4.00
0.00	0.00	0.00	0.00	7.00	0.00	0.00	0.00	0.00
0.00	0.00	0.00	5.00	0.00	0.00	0.00	0.00	0.00
0.00	0.00	0.00	0.00	0.00	0.00	0.00	2.00	0.00
0.00	0.00	0.00	0.00	0.00	0.00	0.00	0.00	0.00
0.00	0.00	0.00	0.00	0.00	0.00	0.00	2.00	0.00
0.00	0.00	0.00	0.00	0.00	0.00	0.00	0.00	0.00
0.00	0.00	0.00	0.00	0.00	0.00	0.00	0.00	0.00
0.00	0.00	0.00	0.00	0.00	0.00	0.00	0.00	0.00
0.00	0.00	0.00	0.00	0.00	0.00	0.00	0.00	0.00
0.00	0.00	0.00	0.00	0.00	0.00	0.00	0.00	0.00
0.00	0.00	0.00	0.00	0.00	0.00	0.00	0.00	0.00
0.00	0.00	0.00	0.00	0.00	0.00	0.00	0.00	0.00
0.00	0.00	0.00	0.00	0.00	0.00	0.00	0.00	0.00
0.00	0.00	0.00	0.00	0.00	0.00	0.00	0.00	0.00
0.00	0.00	0.00	0.00	0.00	0.00	0.00	0.00	0.00
0.00	0.00	0.00	0.00	0.00	0.00	0.00	0.00	0.00
0.00	0.00	0.00	0.00	0.00	0.00	0.00	0.00	0.00
0.00	0.00	0.00	0.00	0.00	0.00	0.00	0.00	0.00
0.00	0.00	0.00	0.00	0.00	0.00	0.00	0.00	0.00
0.00	0.00	0.00	0.00	0.00	0.00	0.00	0.00	0.00
0.00	0.00	0.00	0.00	0.00	0.00	0.00	0.00	0.00
0.00	0.00	0.00	0.00	0.00	0.00	0.00	0.00	0.00
0.00	0.00	0.00	0.00	0.00	0.00	0.00	0.00	0.00
0.00	0.00	0.00	0.00	0.00	0.00	0.00	0.00	0.00
0.00	0.00	0.00	0.00	0.00	0.00	0.00	0.00	0.00
0.00	0.00	0.00	0.00	0.00	0.00	0.00	0.00	0.00
0.00	0.00	0.00	0.00	0.00	0.00	0.00	0.00	0.00
0.00	0.00	0.00	0.00	0.00	0.00	0.00	0.00	0.00
0.00	0.00	0.00	0.00	0.00	0.00	0.00	0.00	0.00
0.00	0.00	0.00	0.00	0.00	0.00	0.00	0.00	0.00
0.00	0.00	0.00	0.00	0.00	0.00	0.00	0.00	0.00
0.00	0.00	0.00	0.00	0.00	0.00	0.00	0.00	0.00
0.00	0.00	0.00	0.00	0.00	0.00	0.00	0.00	0.00
0.00	0.00	0.00	0.00	0.00	0.00	0.00	0.00	0.00
0.00	0.00	0.00	0.00	0.00	0.00	0.00	0.00	0.00

0.00	0.00	0.00	0.00	0.00	0.00	0.00	0.00	0.00
0.00	0.00	0.00	0.00	0.00	0.00	0.00	0.00	0.00
0.00	0.00	0.00	0.00	0.00	0.00	0.00	0.00	0.00
0.00	0.00	0.00	0.00	0.00	0.00	0.00	0.00	0.00
0.00	0.00	0.00	0.00	0.00	0.00	0.00	0.00	0.00
0.00	0.00	0.00	0.00	0.00	0.00	0.00	8.00	0.00
0.00	0.00	0.00	0.00	0.00	0.00	0.00	0.00	0.00
0.00	0.00	0.00	0.00	0.00	0.00	0.00	0.00	0.00
0.00	0.00	0.00	0.00	12.00	0.00	0.00	0.00	0.00
0.00	0.00	0.00	0.00	6.00	0.00	0.00	0.00	0.00
0.00	0.00	0.00	12.00	0.00	0.00	0.00	0.00	0.00
0.00	0.00	0.00	0.00	0.00	0.00	0.00	0.00	0.00
0.00	0.00	0.00	0.00	0.00	0.00	0.00	0.00	0.00
0.00	0.00	0.00	0.00	0.00	0.00	0.00	0.00	0.00
0.00	0.00	0.00	0.00	0.00	0.00	0.00	0.00	0.00
0.00	0.00	0.00	0.00	0.00	0.00	0.00	0.00	0.00
0.00	0.00	0.00	0.00	0.00	0.00	0.00	0.00	0.00
0.00	0.00	0.00	0.00	0.00	0.00	0.00	0.00	0.00
0.00	0.00	0.00	0.00	0.00	0.00	0.00	0.00	0.00
0.00	0.00	0.00	0.00	0.00	0.00	0.00	0.00	0.00
0.00	0.00	0.00	0.00	0.00	5.00	0.00	4.00	0.00
0.00	0.00	0.00	0.00	0.00	0.00	0.00	0.00	0.00
0.00	0.00	0.00	0.00	0.00	0.00	0.00	0.00	0.00
0.00	0.00	0.00	0.00	0.00	0.00	0.00	0.00	0.00
0.00	0.00	0.00	0.00	0.00	0.00	0.00	0.00	0.00
0.00	0.00	0.00	0.00	0.00	0.00	0.00	0.00	0.00
0.00	0.00	0.00	0.00	0.00	0.00	0.00	0.00	0.00
0.00	0.00	0.00	0.00	0.00	0.00	0.00	0.00	0.00
0.00	0.00	0.00	0.00	0.00	0.00	0.00	0.00	0.00
0.00	0.00	0.00	0.00	0.00	0.00	0.00	0.00	0.00
0.00	0.00	0.00	0.00	0.00	0.00	0.00	0.00	0.00
0.00	0.00	0.00	0.00	0.00	0.00	0.00	0.00	0.00
0.00	0.00	0.00	0.00	0.00	0.00	0.00	0.00	0.00
0.00	0.00	0.00	0.00	0.00	0.00	0.00	0.00	0.00
0.00	0.00	0.00	0.00	0.00	0.00	0.00	0.00	0.00
0.00	0.00	0.00	0.00	0.00	0.00	0.00	0.00	0.00
0.00	0.00	0.00	0.00	0.00	0.00	0.00	0.00	0.00

0.00	0.00	0.00	0.00	0.00	0.00	0.00	0.00	0.00
0.00	0.00	0.00	0.00	0.00	0.00	0.00	0.00	0.00
0.00	0.00	0.00	0.00	0.00	0.00	0.00	0.00	0.00
0.00	0.00	0.00	0.00	0.00	0.00	0.00	0.00	0.00
0.00	0.00	7.00	0.00	0.00	0.00	0.00	0.00	4.00
0.00	0.00	0.00	0.00	11.00	0.00	0.00	0.00	0.00
0.00	0.00	0.00	5.00	0.00	0.00	0.00	0.00	0.00
0.00	0.00	0.00	0.00	0.00	0.00	0.00	0.00	0.00
0.00	0.00	0.00	0.00	0.00	0.00	0.00	0.00	0.00
0.00	0.00	0.00	0.00	0.00	0.00	0.00	0.00	0.00
0.00	0.00	0.00	0.00	0.00	0.00	0.00	0.00	0.00
0.00	0.00	0.00	0.00	0.00	0.00	0.00	0.00	0.00
0.00	0.00	0.00	0.00	0.00	0.00	0.00	0.00	0.00
0.00	0.00	0.00	0.00	0.00	0.00	0.00	0.00	0.00
0.00	0.00	0.00	0.00	0.00	0.00	0.00	0.00	0.00
0.00	0.00	0.00	0.00	0.00	0.00	0.00	0.00	0.00
0.00	0.00	0.00	0.00	0.00	0.00	0.00	0.00	0.00
0.00	0.00	0.00	0.00	0.00	0.00	0.00	0.00	0.00
0.00	0.00	0.00	0.00	0.00	0.00	0.00	0.00	0.00
0.00	0.00	0.00	0.00	0.00	0.00	0.00	0.00	0.00
0.00	0.00	0.00	0.00	0.00	3.00	0.00	3.00	0.00
0.00	0.00	0.00	0.00	0.00	0.00	0.00	0.00	0.00
0.00	0.00	0.00	0.00	0.00	0.00	0.00	0.00	0.00
0.00	0.00	0.00	0.00	0.00	0.00	0.00	0.00	0.00
0.00	0.00	0.00	0.00	0.00	0.00	0.00	0.00	0.00
0.00	0.00	0.00	0.00	0.00	0.00	0.00	0.00	0.00
0.00	0.00	0.00	0.00	0.00	0.00	0.00	0.00	0.00
0.00	0.00	0.00	0.00	0.00	0.00	0.00	0.00	0.00
0.00	0.00	0.00	0.00	0.00	0.00	0.00	0.00	0.00
0.00	0.00	0.00	0.00	0.00	0.00	0.00	0.00	0.00
0.00	0.00	0.00	0.00	0.00	0.00	0.00	0.00	0.00
0.00	0.00	0.00	0.00	0.00	0.00	0.00	0.00	0.00
0.00	0.00	0.00	0.00	0.00	0.00	0.00	0.00	0.00
0.00	0.00	0.00	0.00	0.00	0.00	0.00	0.00	0.00
0.00	0.00	0.00	0.00	0.00	0.00	0.00	0.00	0.00
0.00	0.00	0.00	0.00	0.00	0.00	0.00	0.00	0.00
0.00	0.00	0.00	0.00	0.00	0.00	0.00	0.00	0.00

0.00	0.00	0.00	0.00	0.00	0.00	0.00	0.00	0.00
0.00	0.00	0.00	0.00	3.00	0.00	0.00	0.00	0.00
0.00	0.00	0.00	0.00	0.00	0.00	0.00	0.00	0.00
0.00	0.00	0.00	0.00	0.00	0.00	0.00	0.00	0.00
0.00	0.00	0.00	0.00	0.00	0.00	0.00	0.00	0.00
0.00	0.00	0.00	0.00	0.00	0.00	0.00	0.00	0.00
0.00	0.00	0.00	0.00	0.00	0.00	0.00	0.00	0.00
0.00	0.00	0.00	0.00	0.00	0.00	0.00	0.00	0.00
0.00	0.00	0.00	0.00	0.00	0.00	0.00	0.00	0.00
0.00	0.00	0.00	0.00	0.00	0.00	0.00	0.00	0.00
0.00	0.00	0.00	0.00	0.00	0.00	0.00	0.00	0.00
0.00	0.00	0.00	0.00	0.00	0.00	0.00	0.00	0.00
0.00	0.00	0.00	0.00	0.00	0.00	0.00	0.00	0.00
0.00	0.00	0.00	0.00	0.00	0.00	0.00	0.00	0.00
0.00	0.00	0.00	0.00	0.00	0.00	0.00	0.00	0.00
0.00	0.00	0.00	0.00	0.00	0.00	0.00	0.00	0.00
0.00	0.00	0.00	0.00	0.00	0.00	0.00	0.00	0.00
0.00	0.00	0.00	0.00	10.00	0.00	0.00	0.00	0.00
0.00	0.00	0.00	0.00	10.00	0.00	0.00	0.00	0.00
0.00	0.00	0.00	0.00	10.00	0.00	0.00	0.00	0.00
0.00	2.00	0.00	3.00	0.00	0.00	0.00	0.00	0.00
0.00	0.00	0.00	0.00	0.00	0.00	0.00	0.00	0.00
0.00	0.00	0.00	0.00	0.00	0.00	0.00	0.00	0.00
0.00	0.00	0.00	0.00	0.00	0.00	0.00	0.00	0.00
0.00	0.00	0.00	0.00	0.00	0.00	0.00	4.00	0.00
0.00	0.00	0.00	0.00	0.00	0.00	0.00	4.00	0.00
0.00	0.00	0.00	0.00	0.00	0.00	0.00	0.00	0.00
0.00	0.00	0.00	0.00	0.00	0.00	0.00	0.00	0.00
0.00	0.00	0.00	0.00	0.00	0.00	0.00	0.00	0.00
0.00	0.00	0.00	0.00	0.00	0.00	0.00	0.00	0.00
0.00	0.00	0.00	0.00	0.00	0.00	0.00	0.00	0.00
0.00	0.00	0.00	0.00	0.00	0.00	0.00	0.00	0.00
0.00	0.00	0.00	0.00	0.00	0.00	0.00	0.00	0.00
0.00	0.00	0.00	0.00	0.00	0.00	0.00	0.00	0.00
0.00	0.00	0.00	0.00	0.00	0.00	0.00	0.00	0.00
0.00	0.00	0.00	0.00	0.00	0.00	0.00	0.00	0.00
0.00	0.00	0.00	0.00	0.00	0.00	0.00	0.00	0.00

0.00	0.00	0.00	0.00	0.00	0.00	0.00	0.00	0.00
0.00	0.00	0.00	0.00	0.00	0.00	0.00	10.00	0.00
0.00	0.00	0.00	0.00	0.00	5.00	0.00	5.00	0.00
0.00	0.00	0.00	0.00	0.00	10.00	0.00	0.00	0.00
0.00	0.00	0.00	0.00	0.00	0.00	0.00	0.00	0.00
0.00	0.00	0.00	0.00	0.00	0.00	0.00	0.00	0.00
0.00	0.00	0.00	0.00	0.00	0.00	0.00	0.00	0.00
0.00	0.00	0.00	0.00	0.00	0.00	0.00	0.00	0.00
0.00	0.00	0.00	0.00	0.00	0.00	0.00	0.00	0.00
0.00	0.00	0.00	0.00	0.00	0.00	0.00	0.00	0.00
0.00	0.00	0.00	0.00	0.00	0.00	0.00	0.00	0.00
0.00	0.00	0.00	0.00	0.00	0.00	0.00	0.00	0.00
0.00	0.00	0.00	0.00	0.00	0.00	0.00	0.00	0.00
0.00	0.00	0.00	0.00	0.00	0.00	0.00	0.00	0.00
0.00	0.00	0.00	0.00	0.00	0.00	0.00	0.00	0.00
0.00	0.00	0.00	0.00	0.00	0.00	0.00	0.00	0.00
0.00	0.00	0.00	0.00	0.00	0.00	0.00	0.00	0.00
0.00	0.00	0.00	0.00	0.00	0.00	0.00	0.00	0.00
0.00	0.00	0.00	0.00	0.00	0.00	0.00	0.00	0.00
0.00	0.00	0.00	0.00	0.00	0.00	0.00	0.00	0.00
0.00	0.00	0.00	0.00	0.00	0.00	0.00	0.00	0.00
0.00	0.00	0.00	0.00	0.00	0.00	0.00	0.00	0.00
0.00	0.00	0.00	0.00	0.00	0.00	0.00	0.00	0.00
0.00	0.00	0.00	0.00	0.00	0.00	0.00	0.00	0.00
0.00	0.00	0.00	0.00	0.00	0.00	0.00	0.00	0.00
0.00	0.00	0.00	0.00	0.00	0.00	0.00	0.00	0.00
0.00	0.00	0.00	0.00	0.00	0.00	0.00	0.00	0.00
0.00	0.00	0.00	0.00	0.00	0.00	0.00	0.00	0.00
0.00	0.00	0.00	0.00	0.00	0.00	0.00	0.00	0.00
0.00	0.00	5.00	0.00	0.00	0.00	0.00	0.00	0.00
0.00	0.00	0.00	0.00	0.00	0.00	0.00	0.00	0.00
0.00	0.00	0.00	0.00	0.00	0.00	0.00	0.00	0.00
0.00	0.00	0.00	0.00	0.00	0.00	0.00	0.00	0.00
0.00	0.00	0.00	0.00	0.00	0.00	0.00	0.00	0.00
0.00	0.00	0.00	0.00	0.00	0.00	0.00	0.00	0.00
0.00	0.00	0.00	0.00	0.00	0.00	0.00	0.00	0.00
0.00	0.00	0.00	0.00	0.00	0.00	0.00	0.00	0.00

0.00	0.00	0.00	0.00	0.00	0.00	4.00	0.00	0.00
0.00	0.00	0.00	0.00	0.00	0.00	0.00	0.00	9.00
0.00	0.00	0.00	0.00	9.00	0.00	0.00	0.00	0.00
0.00	0.00	0.00	6.00	3.00	0.00	0.00	0.00	0.00
0.00	0.00	0.00	9.00	0.00	0.00	0.00	0.00	0.00
0.00	0.00	0.00	5.00	0.00	0.00	0.00	0.00	0.00
0.00	0.00	0.00	2.00	0.00	0.00	0.00	0.00	0.00
0.00	0.00	0.00	0.00	0.00	0.00	0.00	3.00	0.00
0.00	0.00	0.00	0.00	0.00	0.00	0.00	0.00	0.00
0.00	0.00	0.00	0.00	0.00	0.00	0.00	0.00	0.00
0.00	0.00	0.00	0.00	0.00	0.00	0.00	0.00	0.00
0.00	0.00	0.00	0.00	0.00	0.00	0.00	0.00	0.00
0.00	0.00	0.00	0.00	0.00	0.00	0.00	0.00	0.00
0.00	0.00	0.00	0.00	0.00	0.00	0.00	0.00	0.00
0.00	0.00	0.00	0.00	0.00	0.00	0.00	0.00	0.00
0.00	0.00	0.00	0.00	0.00	0.00	0.00	7.00	0.00
0.00	0.00	0.00	0.00	0.00	0.00	0.00	0.00	0.00
0.00	0.00	0.00	0.00	0.00	0.00	0.00	0.00	0.00
0.00	0.00	0.00	0.00	0.00	0.00	0.00	0.00	0.00
0.00	0.00	0.00	0.00	0.00	0.00	0.00	0.00	0.00
0.00	0.00	0.00	0.00	0.00	0.00	0.00	9.00	0.00
0.00	0.00	0.00	0.00	0.00	0.00	0.00	9.00	0.00
0.00	0.00	0.00	0.00	0.00	0.00	6.00	3.00	0.00
0.00	0.00	0.00	0.00	0.00	0.00	0.00	0.00	0.00
0.00	0.00	0.00	0.00	0.00	0.00	0.00	0.00	0.00
0.00	0.00	0.00	0.00	0.00	0.00	0.00	0.00	0.00
0.00	0.00	0.00	0.00	0.00	0.00	0.00	0.00	0.00
0.00	0.00	0.00	0.00	0.00	0.00	0.00	0.00	0.00
0.00	0.00	0.00	0.00	0.00	0.00	0.00	0.00	0.00
0.00	0.00	0.00	0.00	0.00	0.00	0.00	0.00	0.00
0.00	0.00	0.00	0.00	0.00	0.00	0.00	0.00	0.00
0.00	0.00	0.00	0.00	0.00	0.00	0.00	0.00	0.00
0.00	0.00	0.00	0.00	0.00	0.00	0.00	0.00	0.00
0.00	0.00	0.00	0.00	0.00	0.00	0.00	0.00	0.00
0.00	0.00	0.00	0.00	0.00	0.00	0.00	0.00	0.00
0.00	0.00	0.00	0.00	0.00	0.00	0.00	0.00	0.00
0.00	0.00	0.00	0.00	0.00	0.00	0.00	0.00	0.00

0.00	0.00	0.00	0.00	0.00	0.00	0.00	0.00	0.00
0.00	0.00	0.00	0.00	0.00	0.00	0.00	0.00	0.00
0.00	0.00	0.00	0.00	0.00	0.00	0.00	0.00	0.00
0.00	0.00	0.00	0.00	0.00	0.00	0.00	0.00	0.00
0.00	0.00	0.00	0.00	0.00	0.00	0.00	0.00	0.00
0.00	0.00	0.00	0.00	0.00	0.00	0.00	0.00	0.00
0.00	0.00	0.00	0.00	0.00	0.00	0.00	0.00	0.00
0.00	0.00	0.00	0.00	0.00	0.00	0.00	0.00	0.00
0.00	0.00	0.00	0.00	0.00	0.00	0.00	0.00	0.00
0.00	0.00	0.00	0.00	0.00	0.00	0.00	0.00	0.00
0.00	0.00	0.00	0.00	0.00	0.00	0.00	0.00	0.00
0.00	0.00	0.00	0.00	0.00	0.00	0.00	0.00	0.00
0.00	0.00	0.00	0.00	0.00	0.00	0.00	0.00	0.00
0.00	0.00	0.00	0.00	0.00	0.00	0.00	0.00	0.00
0.00	0.00	0.00	0.00	0.00	0.00	0.00	0.00	0.00
0.00	0.00	0.00	0.00	0.00	0.00	0.00	0.00	0.00
0.00	0.00	0.00	0.00	0.00	0.00	0.00	0.00	0.00
0.00	0.00	0.00	0.00	0.00	0.00	0.00	0.00	0.00
0.00	0.00	0.00	0.00	0.00	0.00	0.00	0.00	0.00
0.00	0.00	0.00	0.00	4.00	0.00	0.00	2.00	0.00
0.00	0.00	0.00	8.00	0.00	0.00	0.00	0.00	0.00
0.00	0.00	0.00	0.00	0.00	0.00	0.00	0.00	0.00
0.00	0.00	0.00	0.00	0.00	0.00	0.00	0.00	0.00
0.00	0.00	0.00	0.00	0.00	0.00	0.00	0.00	0.00
0.00	0.00	0.00	0.00	0.00	0.00	0.00	0.00	0.00
0.00	0.00	0.00	0.00	0.00	0.00	0.00	0.00	0.00
0.00	0.00	0.00	0.00	0.00	0.00	0.00	0.00	0.00
0.00	0.00	0.00	0.00	0.00	0.00	0.00	0.00	0.00
0.00	0.00	0.00	0.00	0.00	0.00	0.00	0.00	0.00
0.00	0.00	0.00	0.00	0.00	0.00	0.00	0.00	0.00
0.00	0.00	0.00	0.00	0.00	0.00	0.00	0.00	0.00
0.00	0.00	0.00	0.00	0.00	0.00	0.00	5.00	0.00
0.00	0.00	0.00	0.00	0.00	0.00	0.00	0.00	0.00
0.00	0.00	0.00	0.00	0.00	0.00	0.00	0.00	0.00
0.00	0.00	0.00	0.00	0.00	0.00	0.00	0.00	0.00
0.00	0.00	0.00	0.00	0.00	0.00	0.00	0.00	0.00
0.00	0.00	0.00	0.00	0.00	0.00	0.00	0.00	0.00

0.00	0.00	0.00	0.00	0.00	0.00	0.00	0.00	0.00
0.00	0.00	0.00	0.00	0.00	0.00	0.00	0.00	0.00
0.00	0.00	0.00	0.00	0.00	0.00	0.00	0.00	0.00
0.00	0.00	0.00	0.00	0.00	0.00	0.00	0.00	0.00
0.00	0.00	0.00	0.00	0.00	0.00	0.00	0.00	0.00
0.00	0.00	0.00	0.00	0.00	0.00	0.00	0.00	0.00
0.00	0.00	0.00	0.00	0.00	0.00	0.00	0.00	0.00
0.00	0.00	0.00	0.00	0.00	0.00	0.00	0.00	0.00
0.00	0.00	0.00	0.00	0.00	0.00	0.00	0.00	0.00
0.00	0.00	0.00	0.00	0.00	0.00	0.00	0.00	0.00
0.00	0.00	0.00	0.00	0.00	0.00	0.00	0.00	0.00
0.00	0.00	0.00	0.00	0.00	0.00	0.00	0.00	0.00
0.00	0.00	0.00	0.00	0.00	0.00	0.00	0.00	0.00
0.00	0.00	0.00	0.00	0.00	0.00	0.00	0.00	0.00
0.00	0.00	0.00	0.00	0.00	0.00	0.00	0.00	0.00
0.00	0.00	0.00	0.00	0.00	0.00	0.00	0.00	0.00
0.00	0.00	0.00	0.00	0.00	0.00	0.00	0.00	0.00
0.00	0.00	0.00	0.00	0.00	0.00	0.00	0.00	0.00
0.00	0.00	0.00	0.00	0.00	0.00	0.00	0.00	0.00
0.00	0.00	0.00	0.00	0.00	0.00	0.00	0.00	0.00
0.00	0.00	0.00	0.00	0.00	0.00	0.00	0.00	0.00
0.00	0.00	0.00	0.00	0.00	0.00	0.00	0.00	0.00
0.00	0.00	0.00	0.00	0.00	0.00	0.00	0.00	0.00
0.00	0.00	0.00	0.00	0.00	0.00	0.00	0.00	0.00
0.00	0.00	0.00	0.00	0.00	0.00	0.00	0.00	0.00
0.00	0.00	0.00	0.00	0.00	0.00	0.00	0.00	0.00
0.00	0.00	0.00	0.00	0.00	0.00	0.00	0.00	0.00
0.00	0.00	0.00	0.00	0.00	0.00	0.00	0.00	0.00
0.00	0.00	0.00	0.00	0.00	0.00	0.00	0.00	0.00
0.00	0.00	0.00	0.00	0.00	0.00	0.00	0.00	0.00
0.00	0.00	0.00	0.00	0.00	0.00	0.00	0.00	0.00
0.00	0.00	0.00	0.00	0.00	0.00	0.00	0.00	0.00
0.00	0.00	0.00	0.00	0.00	0.00	0.00	0.00	0.00
0.00	0.00	0.00	0.00	0.00	0.00	0.00	0.00	0.00
4.00	0.00	0.00	0.00	0.00	0.00	0.00	0.00	3.00
0.00	0.00	0.00	0.00	7.00	0.00	0.00	0.00	0.00
0.00	0.00	0.00	0.00	2.00	0.00	0.00	0.00	0.00

0.00	0.00	0.00	0.00	7.00	0.00	0.00	0.00	0.00
0.00	0.00	0.00	0.00	7.00	0.00	0.00	0.00	0.00
0.00	0.00	0.00	7.00	0.00	0.00	0.00	0.00	0.00
0.00	0.00	0.00	0.00	0.00	0.00	0.00	0.00	0.00
0.00	0.00	0.00	0.00	0.00	0.00	0.00	0.00	0.00
0.00	0.00	0.00	0.00	0.00	0.00	0.00	0.00	0.00
0.00	0.00	0.00	0.00	0.00	0.00	0.00	0.00	0.00
0.00	0.00	0.00	0.00	0.00	0.00	0.00	0.00	0.00
0.00	0.00	0.00	0.00	0.00	0.00	0.00	0.00	0.00
0.00	0.00	0.00	0.00	0.00	0.00	0.00	0.00	0.00
0.00	0.00	0.00	0.00	0.00	0.00	0.00	0.00	0.00
0.00	0.00	0.00	0.00	0.00	0.00	0.00	0.00	0.00
0.00	0.00	0.00	0.00	0.00	0.00	0.00	0.00	0.00
0.00	0.00	0.00	0.00	0.00	0.00	0.00	0.00	0.00
0.00	0.00	0.00	0.00	0.00	0.00	0.00	0.00	0.00
7.00	0.00	0.00	0.00	0.00	0.00	0.00	0.00	0.00
0.00	0.00	0.00	0.00	0.00	0.00	0.00	0.00	0.00
0.00	0.00	0.00	0.00	0.00	0.00	0.00	0.00	0.00
0.00	0.00	0.00	0.00	0.00	0.00	0.00	0.00	0.00
0.00	0.00	0.00	0.00	0.00	0.00	0.00	0.00	0.00
0.00	0.00	0.00	0.00	0.00	0.00	0.00	0.00	0.00
0.00	0.00	0.00	0.00	0.00	0.00	0.00	0.00	0.00
0.00	0.00	0.00	0.00	0.00	0.00	0.00	0.00	0.00
0.00	0.00	0.00	0.00	0.00	0.00	0.00	0.00	0.00
0.00	0.00	0.00	0.00	0.00	0.00	0.00	0.00	0.00
0.00	0.00	0.00	0.00	0.00	0.00	0.00	0.00	0.00
0.00	0.00	0.00	0.00	0.00	0.00	0.00	0.00	0.00
0.00	0.00	0.00	0.00	0.00	0.00	0.00	0.00	0.00
0.00	0.00	0.00	0.00	0.00	0.00	0.00	0.00	0.00
0.00	0.00	0.00	0.00	0.00	0.00	0.00	0.00	0.00
0.00	0.00	0.00	0.00	0.00	0.00	0.00	0.00	0.00
0.00	0.00	0.00	0.00	0.00	0.00	0.00	0.00	0.00
0.00	0.00	0.00	0.00	0.00	0.00	0.00	0.00	0.00
0.00	0.00	0.00	0.00	0.00	0.00	0.00	0.00	0.00
0.00	0.00	0.00	0.00	0.00	0.00	0.00	0.00	0.00
0.00	0.00	0.00	0.00	0.00	0.00	0.00	0.00	0.00
0.00	0.00	0.00	0.00	0.00	0.00	0.00	0.00	0.00

0.00	0.00	0.00	0.00	0.00	0.00	0.00	0.00	0.00
0.00	0.00	0.00	0.00	0.00	0.00	0.00	0.00	0.00
0.00	0.00	0.00	0.00	0.00	0.00	0.00	0.00	0.00
0.00	0.00	0.00	0.00	0.00	0.00	0.00	0.00	0.00
0.00	0.00	0.00	0.00	0.00	0.00	0.00	0.00	0.00
0.00	0.00	0.00	0.00	0.00	0.00	0.00	0.00	0.00
0.00	0.00	0.00	0.00	0.00	0.00	0.00	0.00	0.00
0.00	0.00	0.00	0.00	0.00	0.00	0.00	0.00	0.00
0.00	0.00	0.00	0.00	0.00	0.00	0.00	0.00	0.00
0.00	0.00	0.00	0.00	0.00	0.00	0.00	0.00	0.00
0.00	0.00	0.00	0.00	0.00	0.00	0.00	0.00	0.00
0.00	0.00	0.00	0.00	0.00	0.00	0.00	0.00	0.00
0.00	0.00	0.00	0.00	0.00	0.00	0.00	0.00	0.00
0.00	0.00	0.00	0.00	0.00	0.00	0.00	0.00	0.00
0.00	0.00	0.00	0.00	0.00	0.00	0.00	0.00	0.00
0.00	0.00	0.00	0.00	0.00	0.00	0.00	0.00	0.00
0.00	0.00	0.00	0.00	0.00	0.00	0.00	0.00	0.00
0.00	0.00	0.00	0.00	0.00	0.00	0.00	0.00	0.00
0.00	0.00	0.00	0.00	0.00	0.00	0.00	0.00	0.00
0.00	0.00	0.00	0.00	0.00	0.00	0.00	0.00	0.00
0.00	0.00	0.00	0.00	0.00	0.00	0.00	0.00	0.00
0.00	0.00	0.00	0.00	0.00	0.00	0.00	0.00	0.00
0.00	0.00	0.00	0.00	0.00	0.00	0.00	0.00	0.00
0.00	0.00	0.00	0.00	0.00	0.00	0.00	0.00	0.00
0.00	0.00	0.00	0.00	0.00	0.00	0.00	0.00	0.00
0.00	0.00	0.00	0.00	0.00	0.00	0.00	0.00	0.00
0.00	0.00	0.00	3.00	0.00	0.00	0.00	0.00	0.00
0.00	0.00	0.00	0.00	0.00	0.00	0.00	0.00	6.00
0.00	0.00	0.00	0.00	6.00	0.00	0.00	0.00	0.00
0.00	0.00	0.00	0.00	6.00	0.00	0.00	0.00	0.00
0.00	0.00	0.00	6.00	0.00	0.00	0.00	0.00	0.00
0.00	0.00	0.00	4.00	0.00	0.00	0.00	0.00	0.00
0.00	0.00	0.00	6.00	0.00	0.00	0.00	0.00	0.00
0.00	0.00	0.00	2.00	0.00	0.00	0.00	0.00	0.00
0.00	0.00	0.00	0.00	0.00	0.00	0.00	0.00	0.00
0.00	0.00	0.00	0.00	0.00	0.00	0.00	0.00	0.00
0.00	0.00	0.00	0.00	0.00	0.00	0.00	0.00	0.00

0.00	0.00	0.00	0.00	0.00	0.00	0.00	0.00	0.00
0.00	0.00	0.00	0.00	0.00	0.00	0.00	0.00	0.00
0.00	0.00	0.00	0.00	0.00	0.00	0.00	0.00	0.00
0.00	0.00	0.00	0.00	0.00	0.00	0.00	0.00	0.00
0.00	0.00	0.00	0.00	0.00	0.00	0.00	0.00	0.00
0.00	0.00	0.00	0.00	0.00	0.00	0.00	0.00	0.00
0.00	0.00	0.00	0.00	0.00	0.00	0.00	0.00	0.00
0.00	0.00	0.00	0.00	0.00	0.00	0.00	0.00	0.00
0.00	0.00	0.00	0.00	0.00	0.00	0.00	0.00	0.00
0.00	0.00	0.00	0.00	0.00	2.00	0.00	0.00	0.00
0.00	0.00	0.00	0.00	0.00	0.00	0.00	0.00	0.00
0.00	0.00	0.00	0.00	0.00	0.00	0.00	0.00	0.00
0.00	0.00	0.00	0.00	0.00	0.00	0.00	0.00	0.00
0.00	0.00	0.00	0.00	0.00	0.00	0.00	6.00	0.00
0.00	0.00	0.00	0.00	0.00	0.00	0.00	6.00	0.00
0.00	0.00	0.00	0.00	0.00	6.00	0.00	0.00	0.00
6.00	0.00	0.00	0.00	0.00	0.00	0.00	0.00	0.00
0.00	0.00	0.00	0.00	0.00	0.00	0.00	0.00	0.00
0.00	0.00	0.00	0.00	0.00	0.00	0.00	0.00	0.00
0.00	0.00	0.00	0.00	0.00	0.00	0.00	0.00	0.00
0.00	0.00	0.00	0.00	0.00	0.00	0.00	0.00	0.00
0.00	0.00	0.00	0.00	0.00	0.00	0.00	0.00	0.00
0.00	0.00	0.00	0.00	0.00	0.00	0.00	0.00	0.00
0.00	0.00	0.00	0.00	0.00	0.00	0.00	0.00	0.00
0.00	0.00	0.00	0.00	0.00	0.00	0.00	0.00	0.00
0.00	0.00	0.00	0.00	0.00	0.00	0.00	0.00	0.00
0.00	0.00	0.00	0.00	0.00	0.00	0.00	0.00	0.00
0.00	0.00	0.00	0.00	0.00	0.00	0.00	0.00	0.00
0.00	0.00	0.00	0.00	0.00	0.00	0.00	0.00	0.00
0.00	0.00	0.00	0.00	0.00	0.00	0.00	0.00	0.00
0.00	0.00	0.00	0.00	0.00	0.00	0.00	0.00	0.00
0.00	0.00	0.00	0.00	0.00	0.00	0.00	0.00	0.00
0.00	0.00	0.00	0.00	0.00	0.00	0.00	0.00	0.00
0.00	0.00	0.00	0.00	0.00	0.00	0.00	0.00	0.00
0.00	0.00	0.00	0.00	0.00	0.00	0.00	0.00	0.00
0.00	0.00	0.00	0.00	0.00	0.00	0.00	0.00	0.00
0.00	0.00	0.00	0.00	0.00	0.00	0.00	0.00	0.00

0.00	0.00	0.00	0.00	0.00	0.00	0.00	0.00	0.00
0.00	0.00	0.00	0.00	0.00	0.00	0.00	0.00	0.00
0.00	0.00	0.00	0.00	0.00	0.00	0.00	0.00	0.00
0.00	0.00	0.00	0.00	0.00	0.00	0.00	0.00	0.00
0.00	0.00	0.00	0.00	0.00	0.00	0.00	0.00	0.00
0.00	0.00	0.00	0.00	0.00	0.00	0.00	0.00	0.00
0.00	0.00	0.00	0.00	0.00	0.00	0.00	0.00	0.00
0.00	0.00	0.00	0.00	0.00	0.00	0.00	0.00	0.00
0.00	0.00	0.00	0.00	0.00	0.00	0.00	0.00	0.00
0.00	0.00	0.00	0.00	0.00	0.00	0.00	0.00	0.00
0.00	0.00	0.00	0.00	0.00	0.00	0.00	0.00	0.00
0.00	0.00	0.00	0.00	0.00	0.00	0.00	0.00	0.00
0.00	0.00	0.00	0.00	0.00	0.00	0.00	0.00	0.00
0.00	0.00	0.00	0.00	0.00	0.00	0.00	0.00	0.00
0.00	0.00	0.00	0.00	0.00	0.00	0.00	0.00	0.00
0.00	0.00	0.00	0.00	0.00	0.00	0.00	0.00	0.00
0.00	0.00	0.00	0.00	0.00	0.00	0.00	0.00	0.00
0.00	0.00	0.00	0.00	0.00	0.00	0.00	0.00	0.00
0.00	0.00	0.00	0.00	0.00	0.00	0.00	0.00	0.00
0.00	0.00	0.00	0.00	0.00	0.00	0.00	0.00	0.00
0.00	0.00	0.00	0.00	0.00	0.00	0.00	0.00	0.00
0.00	0.00	0.00	0.00	0.00	0.00	0.00	0.00	0.00
0.00	0.00	0.00	0.00	0.00	0.00	0.00	0.00	0.00
0.00	0.00	0.00	0.00	0.00	0.00	0.00	0.00	0.00
0.00	0.00	0.00	0.00	0.00	0.00	0.00	0.00	0.00
0.00	0.00	0.00	0.00	0.00	0.00	0.00	0.00	0.00
0.00	0.00	0.00	0.00	0.00	0.00	0.00	0.00	0.00
0.00	0.00	0.00	0.00	0.00	0.00	0.00	0.00	0.00
0.00	0.00	0.00	0.00	0.00	0.00	0.00	0.00	0.00
0.00	0.00	0.00	0.00	0.00	0.00	0.00	0.00	0.00
0.00	0.00	0.00	0.00	0.00	0.00	0.00	0.00	0.00
0.00	0.00	0.00	0.00	0.00	0.00	0.00	0.00	0.00
0.00	0.00	0.00	0.00	0.00	0.00	0.00	0.00	0.00
0.00	0.00	0.00	0.00	0.00	0.00	0.00	0.00	0.00
0.00	0.00	0.00	0.00	0.00	0.00	0.00	0.00	0.00
0.00	0.00	0.00	0.00	0.00	0.00	0.00	0.00	0.00
0.00	0.00	0.00	0.00	0.00	0.00	0.00	0.00	0.00

0.00	0.00	0.00	0.00	0.00	0.00	0.00	0.00	0.00
0.00	0.00	0.00	0.00	0.00	0.00	0.00	0.00	0.00
0.00	0.00	0.00	0.00	0.00	0.00	0.00	0.00	0.00
0.00	0.00	0.00	0.00	0.00	0.00	0.00	0.00	5.00
0.00	0.00	0.00	0.00	5.00	0.00	0.00	0.00	0.00
0.00	0.00	0.00	0.00	3.00	0.00	0.00	0.00	0.00
0.00	0.00	0.00	0.00	3.00	0.00	0.00	0.00	0.00
0.00	0.00	0.00	0.00	3.00	0.00	0.00	0.00	0.00
0.00	0.00	0.00	0.00	5.00	0.00	0.00	0.00	0.00
0.00	0.00	0.00	0.00	5.00	0.00	0.00	0.00	0.00
0.00	0.00	0.00	5.00	0.00	0.00	0.00	0.00	0.00
0.00	0.00	0.00	5.00	0.00	0.00	0.00	0.00	0.00
0.00	0.00	0.00	5.00	0.00	0.00	0.00	0.00	0.00
0.00	0.00	0.00	0.00	0.00	0.00	0.00	0.00	0.00
0.00	0.00	0.00	0.00	0.00	0.00	0.00	0.00	0.00
0.00	0.00	0.00	0.00	0.00	0.00	0.00	0.00	0.00
0.00	0.00	0.00	0.00	0.00	0.00	0.00	0.00	0.00
0.00	0.00	0.00	0.00	0.00	0.00	0.00	0.00	0.00
0.00	0.00	0.00	0.00	0.00	0.00	0.00	0.00	0.00
0.00	0.00	0.00	0.00	0.00	0.00	0.00	0.00	0.00
0.00	0.00	0.00	0.00	0.00	0.00	0.00	0.00	0.00
0.00	0.00	0.00	0.00	0.00	0.00	0.00	0.00	0.00
0.00	0.00	0.00	0.00	0.00	0.00	0.00	0.00	0.00
0.00	0.00	0.00	0.00	0.00	0.00	0.00	0.00	0.00
0.00	0.00	0.00	0.00	0.00	0.00	0.00	0.00	0.00
0.00	0.00	0.00	0.00	0.00	0.00	0.00	0.00	0.00
0.00	0.00	0.00	0.00	0.00	0.00	0.00	0.00	0.00
0.00	0.00	0.00	0.00	0.00	0.00	0.00	5.00	0.00
0.00	0.00	0.00	0.00	0.00	0.00	0.00	5.00	0.00
0.00	0.00	0.00	0.00	0.00	0.00	0.00	5.00	0.00
0.00	0.00	0.00	0.00	0.00	0.00	5.00	0.00	0.00
0.00	0.00	0.00	0.00	0.00	5.00	0.00	0.00	0.00
0.00	0.00	5.00	0.00	0.00	0.00	0.00	0.00	0.00
0.00	0.00	0.00	0.00	0.00	0.00	0.00	0.00	0.00
0.00	0.00	0.00	0.00	0.00	0.00	0.00	0.00	0.00
0.00	0.00	0.00	0.00	0.00	0.00	0.00	0.00	0.00
0.00	0.00	0.00	0.00	0.00	0.00	0.00	0.00	0.00

0.00	0.00	0.00	0.00	0.00	0.00	0.00	0.00	0.00
0.00	0.00	0.00	0.00	0.00	0.00	0.00	0.00	0.00
0.00	0.00	0.00	0.00	0.00	0.00	0.00	0.00	0.00
0.00	0.00	0.00	0.00	0.00	0.00	0.00	0.00	0.00
0.00	0.00	0.00	0.00	0.00	0.00	0.00	0.00	0.00
0.00	0.00	0.00	0.00	0.00	0.00	0.00	0.00	0.00
0.00	0.00	0.00	0.00	0.00	0.00	0.00	0.00	0.00
0.00	0.00	0.00	0.00	0.00	0.00	0.00	0.00	0.00
0.00	0.00	0.00	0.00	0.00	0.00	0.00	0.00	0.00
0.00	0.00	0.00	0.00	0.00	0.00	0.00	0.00	0.00
0.00	0.00	0.00	0.00	0.00	0.00	0.00	0.00	0.00
0.00	0.00	0.00	0.00	0.00	0.00	0.00	0.00	0.00
0.00	0.00	0.00	0.00	0.00	0.00	0.00	0.00	0.00
0.00	0.00	0.00	0.00	0.00	0.00	0.00	0.00	0.00
0.00	0.00	0.00	0.00	0.00	0.00	0.00	0.00	0.00
0.00	0.00	0.00	0.00	0.00	0.00	0.00	0.00	0.00
0.00	0.00	0.00	0.00	0.00	0.00	0.00	0.00	0.00
0.00	0.00	0.00	0.00	0.00	0.00	0.00	0.00	0.00
0.00	0.00	0.00	0.00	0.00	0.00	0.00	0.00	0.00
0.00	0.00	0.00	0.00	0.00	0.00	0.00	0.00	0.00
0.00	0.00	0.00	0.00	0.00	0.00	0.00	0.00	0.00
0.00	0.00	0.00	0.00	0.00	0.00	0.00	0.00	0.00
0.00	0.00	0.00	0.00	0.00	0.00	0.00	0.00	0.00
0.00	0.00	0.00	0.00	0.00	0.00	0.00	0.00	0.00
0.00	0.00	0.00	0.00	0.00	0.00	0.00	0.00	0.00
0.00	0.00	0.00	0.00	0.00	0.00	0.00	0.00	0.00
0.00	0.00	0.00	0.00	0.00	0.00	0.00	0.00	0.00
0.00	0.00	0.00	0.00	0.00	0.00	0.00	0.00	0.00
0.00	0.00	0.00	0.00	0.00	0.00	0.00	0.00	0.00
0.00	0.00	0.00	0.00	0.00	0.00	0.00	0.00	0.00
0.00	0.00	0.00	0.00	0.00	0.00	0.00	0.00	0.00
0.00	0.00	0.00	0.00	0.00	0.00	0.00	0.00	0.00
0.00	0.00	0.00	0.00	0.00	0.00	0.00	0.00	0.00
0.00	0.00	0.00	0.00	0.00	0.00	0.00	0.00	0.00
0.00	0.00	0.00	0.00	0.00	0.00	0.00	0.00	0.00
0.00	0.00	0.00	0.00	0.00	0.00	0.00	0.00	0.00
0.00	0.00	0.00	0.00	0.00	0.00	0.00	0.00	0.00

0.00	0.00	0.00	0.00	0.00	0.00	0.00	0.00	0.00
0.00	0.00	0.00	0.00	0.00	0.00	0.00	0.00	0.00
0.00	0.00	0.00	0.00	0.00	0.00	0.00	0.00	0.00
0.00	0.00	0.00	0.00	0.00	0.00	0.00	0.00	0.00
0.00	0.00	0.00	0.00	0.00	0.00	0.00	0.00	0.00
0.00	0.00	0.00	0.00	0.00	0.00	0.00	0.00	0.00
0.00	0.00	0.00	0.00	0.00	0.00	0.00	0.00	0.00
0.00	0.00	0.00	0.00	0.00	0.00	0.00	0.00	0.00
0.00	0.00	0.00	0.00	0.00	0.00	0.00	0.00	0.00
0.00	0.00	0.00	0.00	0.00	0.00	0.00	0.00	0.00
0.00	0.00	0.00	0.00	0.00	0.00	0.00	0.00	0.00
0.00	0.00	0.00	0.00	0.00	0.00	0.00	0.00	0.00
0.00	0.00	0.00	0.00	0.00	0.00	0.00	0.00	0.00
0.00	0.00	0.00	0.00	0.00	0.00	0.00	0.00	0.00
0.00	0.00	0.00	0.00	0.00	0.00	0.00	0.00	0.00
0.00	0.00	0.00	0.00	0.00	0.00	0.00	0.00	0.00
0.00	0.00	0.00	0.00	0.00	0.00	0.00	0.00	0.00
0.00	0.00	0.00	0.00	0.00	0.00	0.00	0.00	0.00
0.00	0.00	0.00	0.00	0.00	0.00	0.00	0.00	0.00
0.00	0.00	0.00	0.00	0.00	0.00	0.00	0.00	0.00
0.00	0.00	0.00	0.00	0.00	0.00	0.00	0.00	0.00
0.00	0.00	0.00	0.00	0.00	0.00	0.00	0.00	0.00
0.00	0.00	0.00	0.00	0.00	0.00	0.00	2.00	0.00
0.00	0.00	0.00	0.00	0.00	0.00	0.00	0.00	4.00
0.00	0.00	0.00	0.00	4.00	0.00	0.00	0.00	0.00
0.00	0.00	0.00	0.00	4.00	0.00	0.00	0.00	0.00
0.00	0.00	0.00	0.00	4.00	0.00	0.00	0.00	0.00
0.00	0.00	0.00	0.00	4.00	0.00	0.00	0.00	0.00
0.00	0.00	0.00	0.00	4.00	0.00	0.00	0.00	0.00
0.00	0.00	0.00	0.00	4.00	0.00	0.00	0.00	0.00
0.00	0.00	0.00	0.00	4.00	0.00	0.00	0.00	0.00
0.00	0.00	0.00	0.00	4.00	0.00	0.00	0.00	0.00
0.00	0.00	0.00	4.00	0.00	0.00	0.00	0.00	0.00
0.00	0.00	0.00	4.00	0.00	0.00	0.00	0.00	0.00
0.00	0.00	0.00	4.00	0.00	0.00	0.00	0.00	0.00
0.00	0.00	0.00	0.00	0.00	0.00	0.00	0.00	0.00
0.00	0.00	0.00	0.00	0.00	0.00	0.00	0.00	0.00

0.00	0.00	0.00	0.00	0.00	0.00	0.00	0.00	0.00
0.00	0.00	0.00	0.00	0.00	0.00	0.00	0.00	0.00
0.00	0.00	0.00	0.00	0.00	0.00	0.00	0.00	0.00
0.00	0.00	0.00	0.00	0.00	0.00	0.00	0.00	0.00
0.00	0.00	0.00	0.00	0.00	0.00	0.00	0.00	0.00
0.00	0.00	0.00	0.00	0.00	0.00	0.00	0.00	0.00
0.00	0.00	0.00	0.00	0.00	0.00	0.00	0.00	0.00
0.00	0.00	0.00	0.00	0.00	0.00	0.00	0.00	0.00
0.00	0.00	0.00	0.00	0.00	0.00	2.00	0.00	0.00
0.00	0.00	0.00	0.00	0.00	0.00	0.00	0.00	0.00
0.00	0.00	0.00	0.00	0.00	0.00	0.00	0.00	0.00
0.00	0.00	0.00	0.00	0.00	0.00	0.00	0.00	0.00
0.00	0.00	0.00	0.00	0.00	0.00	0.00	0.00	0.00
0.00	0.00	0.00	0.00	0.00	0.00	0.00	0.00	0.00
0.00	0.00	0.00	0.00	0.00	0.00	0.00	0.00	0.00
0.00	0.00	0.00	0.00	0.00	0.00	0.00	0.00	0.00
0.00	0.00	0.00	0.00	0.00	0.00	0.00	0.00	0.00
0.00	0.00	0.00	0.00	0.00	0.00	0.00	0.00	0.00
0.00	0.00	0.00	0.00	0.00	0.00	0.00	0.00	0.00
0.00	0.00	0.00	0.00	0.00	0.00	0.00	0.00	0.00
0.00	0.00	0.00	0.00	0.00	0.00	0.00	4.00	0.00
0.00	0.00	0.00	0.00	0.00	0.00	0.00	4.00	0.00
0.00	0.00	0.00	0.00	0.00	0.00	0.00	4.00	0.00
0.00	0.00	0.00	0.00	0.00	0.00	0.00	4.00	0.00
0.00	0.00	0.00	0.00	0.00	0.00	0.00	4.00	0.00
0.00	0.00	0.00	0.00	0.00	0.00	0.00	4.00	0.00
0.00	0.00	0.00	0.00	0.00	0.00	0.00	4.00	0.00
0.00	0.00	0.00	0.00	0.00	0.00	4.00	0.00	0.00
0.00	0.00	0.00	0.00	0.00	0.00	4.00	0.00	0.00
4.00	0.00	0.00	0.00	0.00	0.00	0.00	0.00	0.00
0.00	0.00	0.00	0.00	0.00	0.00	0.00	0.00	0.00
0.00	0.00	0.00	0.00	0.00	0.00	0.00	0.00	0.00
0.00	0.00	0.00	0.00	0.00	0.00	0.00	0.00	0.00
0.00	0.00	0.00	0.00	0.00	0.00	0.00	0.00	0.00
0.00	0.00	0.00	0.00	0.00	0.00	0.00	0.00	0.00
0.00	0.00	0.00	0.00	0.00	0.00	0.00	0.00	0.00
0.00	0.00	0.00	0.00	0.00	0.00	0.00	0.00	0.00

0.00	0.00	0.00	0.00	0.00	0.00	0.00	0.00	0.00
0.00	0.00	0.00	0.00	0.00	0.00	0.00	0.00	0.00
0.00	0.00	0.00	0.00	0.00	0.00	0.00	0.00	0.00
0.00	0.00	0.00	0.00	0.00	0.00	0.00	0.00	0.00
0.00	0.00	0.00	0.00	0.00	0.00	0.00	0.00	0.00
0.00	0.00	0.00	0.00	0.00	0.00	0.00	0.00	0.00
0.00	0.00	0.00	0.00	0.00	0.00	0.00	0.00	0.00
0.00	0.00	0.00	0.00	0.00	0.00	0.00	0.00	0.00
0.00	0.00	0.00	0.00	0.00	0.00	0.00	0.00	0.00
0.00	0.00	0.00	0.00	0.00	0.00	0.00	0.00	0.00
0.00	0.00	0.00	0.00	0.00	0.00	0.00	0.00	0.00
0.00	0.00	0.00	0.00	0.00	0.00	0.00	0.00	0.00
0.00	0.00	0.00	0.00	0.00	0.00	0.00	0.00	0.00
0.00	0.00	0.00	0.00	0.00	0.00	0.00	0.00	0.00
0.00	0.00	0.00	0.00	0.00	0.00	0.00	0.00	0.00
0.00	0.00	0.00	0.00	0.00	0.00	0.00	0.00	0.00
0.00	0.00	0.00	0.00	0.00	0.00	0.00	0.00	0.00
0.00	0.00	0.00	0.00	0.00	0.00	0.00	0.00	0.00
0.00	0.00	0.00	0.00	0.00	0.00	0.00	0.00	0.00
0.00	0.00	0.00	0.00	0.00	0.00	0.00	0.00	0.00
0.00	0.00	0.00	0.00	0.00	0.00	0.00	0.00	0.00
0.00	0.00	0.00	0.00	0.00	0.00	0.00	0.00	0.00
0.00	0.00	0.00	0.00	0.00	0.00	0.00	0.00	0.00
0.00	0.00	0.00	0.00	0.00	0.00	0.00	0.00	0.00
0.00	0.00	0.00	0.00	0.00	0.00	0.00	0.00	0.00
0.00	0.00	0.00	0.00	0.00	0.00	0.00	0.00	0.00
0.00	0.00	0.00	0.00	0.00	0.00	0.00	0.00	0.00
0.00	0.00	0.00	0.00	0.00	0.00	0.00	0.00	0.00
0.00	0.00	0.00	0.00	0.00	0.00	0.00	0.00	0.00
0.00	0.00	0.00	0.00	0.00	0.00	0.00	0.00	0.00
0.00	0.00	0.00	0.00	0.00	0.00	0.00	0.00	0.00
0.00	0.00	0.00	0.00	0.00	0.00	0.00	0.00	0.00
0.00	0.00	0.00	0.00	0.00	0.00	0.00	0.00	0.00
0.00	0.00	0.00	0.00	0.00	0.00	0.00	0.00	0.00
0.00	0.00	0.00	0.00	0.00	0.00	0.00	0.00	0.00
0.00	0.00	0.00	0.00	0.00	0.00	0.00	0.00	0.00
0.00	0.00	0.00	0.00	0.00	0.00	0.00	0.00	0.00

0.00	0.00	0.00	0.00	0.00	0.00	0.00	0.00	0.00
0.00	0.00	0.00	0.00	0.00	0.00	0.00	0.00	0.00
0.00	0.00	0.00	0.00	0.00	0.00	0.00	0.00	0.00
0.00	0.00	0.00	0.00	0.00	0.00	0.00	0.00	0.00
0.00	0.00	0.00	0.00	0.00	0.00	0.00	0.00	0.00
0.00	0.00	0.00	0.00	0.00	0.00	0.00	0.00	0.00
0.00	0.00	0.00	0.00	0.00	0.00	0.00	0.00	0.00
0.00	0.00	0.00	0.00	0.00	0.00	0.00	0.00	0.00
0.00	0.00	0.00	0.00	0.00	0.00	0.00	0.00	0.00
0.00	0.00	0.00	0.00	0.00	0.00	0.00	0.00	0.00
0.00	0.00	0.00	0.00	0.00	0.00	0.00	0.00	0.00
0.00	0.00	0.00	0.00	0.00	0.00	0.00	0.00	0.00
0.00	0.00	0.00	0.00	0.00	0.00	0.00	0.00	0.00
0.00	0.00	0.00	0.00	0.00	0.00	0.00	0.00	0.00
0.00	0.00	0.00	0.00	0.00	0.00	0.00	0.00	0.00
0.00	0.00	0.00	0.00	0.00	0.00	0.00	0.00	0.00
0.00	0.00	0.00	0.00	0.00	0.00	0.00	0.00	0.00
0.00	0.00	0.00	0.00	0.00	0.00	0.00	0.00	0.00
0.00	0.00	0.00	0.00	0.00	0.00	0.00	0.00	0.00
0.00	0.00	0.00	0.00	0.00	0.00	0.00	0.00	0.00
0.00	0.00	0.00	0.00	0.00	0.00	0.00	0.00	0.00
0.00	0.00	0.00	0.00	0.00	0.00	0.00	0.00	0.00
0.00	0.00	0.00	0.00	0.00	0.00	0.00	0.00	0.00
0.00	0.00	0.00	0.00	0.00	0.00	0.00	0.00	0.00
0.00	0.00	0.00	0.00	0.00	0.00	0.00	0.00	0.00
0.00	0.00	0.00	0.00	0.00	0.00	0.00	0.00	0.00
0.00	0.00	0.00	0.00	0.00	0.00	0.00	0.00	0.00
0.00	0.00	0.00	0.00	0.00	0.00	0.00	0.00	0.00
0.00	0.00	0.00	0.00	0.00	0.00	0.00	0.00	0.00
0.00	0.00	0.00	0.00	0.00	0.00	0.00	0.00	0.00
0.00	0.00	0.00	0.00	0.00	0.00	0.00	0.00	0.00
0.00	0.00	0.00	0.00	0.00	0.00	0.00	0.00	0.00
0.00	0.00	0.00	0.00	0.00	0.00	0.00	0.00	0.00
0.00	0.00	0.00	0.00	0.00	0.00	0.00	0.00	0.00
0.00	0.00	0.00	0.00	0.00	0.00	0.00	0.00	0.00
0.00	0.00	0.00	0.00	0.00	0.00	0.00	0.00	0.00
0.00	0.00	0.00	0.00	0.00	0.00	0.00	0.00	0.00

0.00	0.00	0.00	0.00	0.00	0.00	0.00	0.00	0.00
0.00	0.00	0.00	0.00	0.00	0.00	0.00	0.00	0.00
0.00	0.00	0.00	0.00	0.00	0.00	0.00	0.00	0.00
0.00	0.00	0.00	0.00	0.00	0.00	0.00	0.00	0.00
0.00	0.00	0.00	0.00	0.00	0.00	0.00	0.00	0.00
0.00	0.00	0.00	0.00	0.00	0.00	0.00	0.00	0.00
0.00	0.00	0.00	0.00	0.00	0.00	0.00	0.00	0.00
0.00	0.00	0.00	0.00	0.00	0.00	0.00	0.00	0.00
0.00	0.00	0.00	0.00	0.00	0.00	0.00	0.00	0.00
0.00	0.00	0.00	0.00	0.00	0.00	0.00	0.00	0.00
0.00	0.00	0.00	0.00	0.00	0.00	0.00	0.00	0.00
0.00	0.00	0.00	0.00	0.00	0.00	0.00	0.00	0.00
0.00	0.00	0.00	0.00	0.00	0.00	0.00	0.00	0.00
0.00	0.00	0.00	0.00	0.00	0.00	0.00	0.00	0.00
0.00	0.00	0.00	0.00	3.00	0.00	0.00	0.00	0.00
0.00	0.00	0.00	0.00	3.00	0.00	0.00	0.00	0.00
0.00	0.00	0.00	0.00	3.00	0.00	0.00	0.00	0.00
0.00	0.00	0.00	3.00	0.00	0.00	0.00	0.00	0.00
0.00	0.00	0.00	0.00	0.00	0.00	0.00	0.00	0.00
0.00	0.00	0.00	0.00	0.00	0.00	0.00	0.00	0.00
0.00	0.00	0.00	0.00	0.00	0.00	0.00	0.00	0.00
0.00	0.00	0.00	0.00	0.00	0.00	0.00	0.00	0.00
0.00	0.00	0.00	0.00	0.00	0.00	0.00	0.00	0.00
0.00	0.00	0.00	0.00	0.00	0.00	0.00	0.00	0.00
0.00	0.00	0.00	0.00	0.00	0.00	0.00	0.00	0.00
0.00	0.00	0.00	0.00	0.00	0.00	0.00	0.00	0.00
0.00	0.00	0.00	0.00	0.00	0.00	0.00	0.00	0.00
0.00	0.00	0.00	0.00	0.00	0.00	0.00	0.00	0.00
0.00	0.00	0.00	0.00	0.00	0.00	0.00	0.00	0.00
0.00	0.00	0.00	0.00	0.00	0.00	0.00	0.00	0.00
0.00	0.00	0.00	0.00	0.00	0.00	0.00	0.00	0.00
0.00	0.00	0.00	0.00	0.00	0.00	0.00	0.00	0.00
0.00	0.00	0.00	0.00	0.00	0.00	0.00	0.00	0.00
0.00	0.00	0.00	0.00	0.00	0.00	0.00	0.00	0.00
0.00	0.00	0.00	0.00	0.00	0.00	0.00	0.00	0.00
0.00	0.00	0.00	0.00	0.00	0.00	0.00	0.00	0.00
0.00	0.00	0.00	0.00	0.00	0.00	0.00	0.00	0.00

0.00	0.00	0.00	0.00	0.00	0.00	0.00	0.00	0.00
0.00	0.00	0.00	0.00	0.00	0.00	0.00	0.00	0.00
0.00	0.00	0.00	0.00	0.00	0.00	0.00	0.00	0.00
0.00	0.00	0.00	0.00	0.00	0.00	0.00	0.00	0.00
0.00	0.00	0.00	0.00	0.00	0.00	0.00	0.00	0.00
0.00	0.00	0.00	0.00	0.00	0.00	0.00	0.00	0.00
0.00	0.00	0.00	0.00	0.00	0.00	0.00	0.00	0.00
0.00	0.00	0.00	0.00	0.00	0.00	0.00	0.00	0.00
0.00	0.00	0.00	0.00	0.00	0.00	0.00	0.00	0.00
0.00	0.00	0.00	0.00	0.00	0.00	0.00	0.00	0.00
0.00	0.00	0.00	0.00	0.00	0.00	0.00	0.00	0.00
0.00	0.00	0.00	0.00	0.00	0.00	0.00	3.00	0.00
0.00	0.00	0.00	0.00	0.00	0.00	0.00	3.00	0.00
0.00	0.00	0.00	0.00	0.00	0.00	0.00	3.00	0.00
0.00	0.00	0.00	0.00	0.00	0.00	0.00	3.00	0.00
0.00	0.00	0.00	0.00	0.00	0.00	0.00	3.00	0.00
0.00	0.00	0.00	0.00	0.00	0.00	0.00	3.00	0.00
0.00	0.00	0.00	0.00	0.00	0.00	0.00	3.00	0.00
0.00	0.00	0.00	0.00	0.00	0.00	0.00	3.00	0.00
0.00	0.00	0.00	0.00	0.00	0.00	0.00	3.00	0.00
0.00	0.00	0.00	0.00	0.00	0.00	3.00	0.00	0.00
0.00	0.00	0.00	0.00	0.00	3.00	0.00	0.00	0.00
0.00	0.00	0.00	0.00	0.00	3.00	0.00	0.00	0.00
0.00	0.00	3.00	0.00	0.00	0.00	0.00	0.00	0.00
0.00	0.00	3.00	0.00	0.00	0.00	0.00	0.00	0.00
3.00	0.00	0.00	0.00	0.00	0.00	0.00	0.00	0.00
0.00	0.00	0.00	0.00	0.00	0.00	0.00	0.00	0.00
0.00	0.00	0.00	0.00	0.00	0.00	0.00	0.00	0.00
0.00	0.00	0.00	0.00	0.00	0.00	0.00	0.00	0.00
0.00	0.00	0.00	0.00	0.00	0.00	0.00	0.00	0.00
0.00	0.00	0.00	0.00	0.00	0.00	0.00	0.00	0.00
0.00	0.00	0.00	0.00	0.00	0.00	0.00	0.00	0.00
0.00	0.00	0.00	0.00	0.00	0.00	0.00	0.00	0.00
0.00	0.00	0.00	0.00	0.00	0.00	0.00	0.00	0.00
0.00	0.00	0.00	0.00	0.00	0.00	0.00	0.00	0.00
0.00	0.00	0.00	0.00	0.00	0.00	0.00	0.00	0.00
0.00	0.00	0.00	0.00	0.00	0.00	0.00	0.00	0.00

0.00	0.00	0.00	0.00	0.00	0.00	0.00	0.00	0.00
0.00	0.00	0.00	0.00	0.00	0.00	0.00	0.00	0.00
0.00	0.00	0.00	0.00	0.00	0.00	0.00	0.00	0.00
0.00	0.00	0.00	0.00	0.00	0.00	0.00	0.00	0.00
0.00	0.00	0.00	0.00	0.00	0.00	0.00	0.00	0.00
0.00	0.00	0.00	0.00	0.00	0.00	0.00	0.00	0.00
0.00	0.00	0.00	0.00	0.00	0.00	0.00	0.00	0.00
0.00	0.00	0.00	0.00	0.00	0.00	0.00	0.00	0.00
0.00	0.00	0.00	0.00	0.00	0.00	0.00	0.00	0.00
0.00	0.00	0.00	0.00	0.00	0.00	0.00	0.00	0.00
0.00	0.00	0.00	0.00	0.00	0.00	0.00	0.00	0.00
0.00	0.00	0.00	0.00	0.00	0.00	0.00	0.00	0.00
0.00	0.00	0.00	0.00	0.00	0.00	0.00	0.00	0.00
0.00	0.00	0.00	0.00	0.00	0.00	0.00	0.00	0.00
0.00	0.00	0.00	0.00	0.00	0.00	0.00	0.00	0.00
0.00	0.00	0.00	0.00	0.00	0.00	0.00	0.00	0.00
0.00	0.00	0.00	0.00	0.00	0.00	0.00	0.00	0.00
0.00	0.00	0.00	0.00	0.00	0.00	0.00	0.00	0.00
0.00	0.00	0.00	0.00	0.00	0.00	0.00	0.00	0.00
0.00	0.00	0.00	0.00	0.00	0.00	0.00	0.00	0.00
0.00	0.00	0.00	0.00	0.00	0.00	0.00	0.00	0.00
0.00	0.00	0.00	0.00	0.00	0.00	0.00	0.00	0.00
0.00	0.00	0.00	0.00	0.00	0.00	0.00	0.00	0.00
0.00	0.00	0.00	0.00	0.00	0.00	0.00	0.00	0.00
0.00	0.00	0.00	0.00	0.00	0.00	0.00	0.00	0.00
0.00	0.00	0.00	0.00	0.00	0.00	0.00	0.00	0.00
0.00	0.00	0.00	0.00	0.00	0.00	0.00	0.00	0.00
0.00	0.00	0.00	0.00	0.00	0.00	0.00	0.00	0.00
0.00	0.00	0.00	0.00	0.00	0.00	0.00	0.00	0.00
0.00	0.00	0.00	0.00	0.00	0.00	0.00	0.00	0.00
0.00	0.00	0.00	0.00	0.00	0.00	0.00	0.00	0.00
0.00	0.00	0.00	0.00	0.00	0.00	0.00	0.00	0.00
0.00	0.00	0.00	0.00	0.00	0.00	0.00	0.00	0.00
0.00	0.00	0.00	0.00	0.00	0.00	0.00	0.00	0.00
0.00	0.00	0.00	0.00	0.00	0.00	0.00	0.00	0.00
0.00	0.00	0.00	0.00	0.00	0.00	0.00	0.00	0.00
0.00	0.00	0.00	0.00	0.00	0.00	0.00	0.00	0.00

0.00	0.00	0.00	0.00	0.00	0.00	0.00	0.00	0.00
0.00	0.00	0.00	0.00	0.00	0.00	0.00	0.00	0.00
0.00	0.00	0.00	0.00	0.00	0.00	0.00	0.00	0.00
0.00	0.00	0.00	0.00	0.00	0.00	0.00	0.00	0.00
0.00	0.00	0.00	0.00	0.00	0.00	0.00	0.00	0.00
0.00	0.00	0.00	0.00	0.00	0.00	0.00	0.00	0.00
0.00	0.00	0.00	0.00	0.00	0.00	0.00	0.00	0.00
0.00	0.00	0.00	0.00	0.00	0.00	0.00	0.00	0.00
0.00	0.00	0.00	0.00	0.00	0.00	0.00	0.00	0.00
0.00	0.00	0.00	0.00	0.00	0.00	0.00	0.00	0.00
0.00	0.00	0.00	0.00	0.00	0.00	0.00	0.00	0.00
0.00	0.00	0.00	0.00	0.00	0.00	0.00	0.00	0.00
0.00	0.00	0.00	0.00	0.00	0.00	0.00	0.00	0.00
0.00	0.00	0.00	0.00	0.00	0.00	0.00	0.00	0.00
0.00	0.00	0.00	0.00	0.00	0.00	0.00	0.00	0.00
0.00	0.00	0.00	0.00	0.00	0.00	0.00	0.00	0.00
0.00	0.00	0.00	0.00	0.00	0.00	0.00	0.00	0.00
0.00	0.00	0.00	0.00	0.00	0.00	0.00	0.00	0.00
0.00	0.00	0.00	0.00	0.00	0.00	0.00	0.00	0.00
0.00	0.00	0.00	0.00	0.00	0.00	0.00	0.00	0.00
0.00	0.00	0.00	0.00	0.00	0.00	0.00	0.00	0.00
0.00	0.00	0.00	0.00	0.00	0.00	0.00	0.00	0.00
0.00	0.00	0.00	0.00	0.00	0.00	0.00	0.00	0.00
0.00	0.00	0.00	0.00	0.00	0.00	0.00	0.00	0.00
0.00	0.00	0.00	0.00	0.00	0.00	0.00	0.00	0.00
0.00	0.00	0.00	0.00	0.00	0.00	0.00	0.00	0.00
0.00	0.00	0.00	0.00	0.00	0.00	0.00	0.00	0.00
0.00	0.00	0.00	0.00	0.00	0.00	0.00	0.00	0.00
0.00	0.00	0.00	0.00	0.00	0.00	0.00	0.00	0.00
0.00	0.00	0.00	0.00	0.00	0.00	0.00	0.00	0.00
0.00	0.00	0.00	0.00	0.00	0.00	0.00	0.00	0.00
0.00	0.00	0.00	0.00	0.00	0.00	0.00	0.00	0.00
0.00	0.00	0.00	0.00	0.00	0.00	0.00	0.00	0.00
0.00	0.00	0.00	0.00	0.00	0.00	0.00	0.00	0.00
0.00	0.00	0.00	0.00	0.00	0.00	0.00	0.00	0.00
0.00	0.00	0.00	0.00	0.00	0.00	0.00	0.00	0.00
0.00	0.00	0.00	0.00	0.00	0.00	0.00	0.00	0.00

0.00	0.00	0.00	0.00	0.00	0.00	0.00	0.00	0.00
0.00	0.00	0.00	0.00	0.00	0.00	0.00	0.00	0.00
0.00	0.00	0.00	0.00	0.00	0.00	0.00	0.00	0.00
0.00	0.00	0.00	0.00	0.00	0.00	0.00	0.00	0.00
0.00	0.00	0.00	0.00	0.00	0.00	0.00	0.00	0.00
0.00	0.00	0.00	0.00	0.00	0.00	0.00	0.00	0.00
0.00	0.00	0.00	0.00	0.00	0.00	0.00	0.00	0.00
0.00	0.00	0.00	0.00	0.00	0.00	0.00	0.00	0.00
0.00	0.00	0.00	0.00	0.00	0.00	0.00	0.00	0.00
0.00	0.00	0.00	0.00	0.00	0.00	0.00	0.00	0.00
0.00	0.00	0.00	0.00	0.00	0.00	0.00	0.00	0.00
0.00	0.00	0.00	0.00	0.00	0.00	0.00	0.00	0.00
0.00	0.00	0.00	0.00	0.00	0.00	0.00	0.00	0.00
0.00	0.00	0.00	0.00	0.00	0.00	0.00	0.00	0.00
0.00	0.00	0.00	0.00	0.00	0.00	0.00	0.00	0.00
0.00	0.00	0.00	0.00	0.00	0.00	0.00	0.00	0.00
0.00	0.00	0.00	0.00	0.00	0.00	0.00	0.00	0.00
0.00	0.00	0.00	0.00	2.00	0.00	0.00	0.00	0.00
0.00	0.00	0.00	0.00	2.00	0.00	0.00	0.00	0.00
0.00	0.00	0.00	0.00	2.00	0.00	0.00	0.00	0.00
0.00	0.00	0.00	0.00	2.00	0.00	0.00	0.00	0.00
0.00	0.00	0.00	0.00	2.00	0.00	0.00	0.00	0.00
0.00	0.00	0.00	0.00	2.00	0.00	0.00	0.00	0.00
0.00	0.00	0.00	2.00	0.00	0.00	0.00	0.00	0.00
0.00	0.00	0.00	2.00	0.00	0.00	0.00	0.00	0.00
0.00	0.00	0.00	0.00	0.00	0.00	0.00	0.00	0.00
0.00	0.00	0.00	0.00	0.00	0.00	0.00	0.00	0.00
0.00	0.00	0.00	0.00	0.00	0.00	0.00	0.00	0.00
0.00	0.00	0.00	0.00	0.00	0.00	0.00	0.00	0.00
0.00	0.00	0.00	0.00	0.00	0.00	0.00	0.00	0.00
0.00	0.00	0.00	0.00	0.00	0.00	0.00	0.00	0.00
0.00	0.00	0.00	0.00	0.00	0.00	0.00	0.00	0.00
0.00	0.00	0.00	0.00	0.00	0.00	0.00	0.00	0.00
0.00	0.00	0.00	0.00	0.00	0.00	0.00	0.00	0.00
0.00	0.00	0.00	0.00	0.00	0.00	0.00	0.00	0.00
0.00	0.00	0.00	0.00	0.00	0.00	0.00	0.00	0.00
0.00	0.00	0.00	0.00	0.00	0.00	0.00	0.00	0.00

0.00	0.00	0.00	0.00	0.00	0.00	0.00	0.00	0.00
0.00	0.00	0.00	0.00	0.00	0.00	0.00	0.00	0.00
0.00	0.00	0.00	0.00	0.00	0.00	0.00	0.00	0.00
0.00	0.00	0.00	0.00	0.00	0.00	0.00	0.00	0.00
0.00	0.00	0.00	0.00	0.00	0.00	0.00	0.00	0.00
0.00	0.00	0.00	0.00	0.00	0.00	0.00	0.00	0.00
0.00	0.00	0.00	0.00	0.00	0.00	0.00	0.00	0.00
0.00	0.00	0.00	0.00	0.00	0.00	0.00	0.00	0.00
0.00	0.00	0.00	0.00	0.00	0.00	0.00	0.00	0.00
0.00	0.00	0.00	0.00	0.00	0.00	0.00	0.00	0.00
0.00	0.00	0.00	0.00	0.00	0.00	0.00	0.00	0.00
0.00	0.00	0.00	0.00	0.00	0.00	0.00	0.00	0.00
0.00	0.00	0.00	0.00	0.00	0.00	0.00	0.00	0.00
0.00	0.00	0.00	0.00	0.00	0.00	0.00	0.00	0.00
0.00	0.00	0.00	0.00	0.00	0.00	0.00	0.00	0.00
0.00	0.00	0.00	0.00	0.00	0.00	0.00	0.00	0.00
0.00	0.00	0.00	0.00	0.00	0.00	0.00	0.00	0.00
0.00	0.00	0.00	0.00	0.00	0.00	0.00	0.00	0.00
0.00	0.00	0.00	0.00	0.00	0.00	0.00	0.00	0.00
0.00	0.00	0.00	0.00	0.00	0.00	0.00	0.00	0.00
0.00	0.00	0.00	0.00	0.00	0.00	0.00	0.00	0.00
0.00	0.00	0.00	0.00	0.00	0.00	0.00	0.00	0.00
0.00	0.00	0.00	0.00	0.00	0.00	0.00	0.00	0.00
0.00	0.00	0.00	0.00	0.00	0.00	0.00	0.00	0.00
0.00	0.00	0.00	0.00	0.00	0.00	0.00	0.00	0.00
0.00	0.00	0.00	0.00	0.00	0.00	0.00	0.00	0.00
0.00	0.00	0.00	0.00	0.00	0.00	0.00	0.00	0.00
0.00	0.00	0.00	0.00	0.00	0.00	0.00	0.00	0.00
0.00	0.00	0.00	0.00	0.00	0.00	0.00	0.00	0.00
0.00	0.00	0.00	0.00	0.00	0.00	0.00	0.00	0.00
0.00	0.00	0.00	0.00	0.00	0.00	0.00	0.00	0.00
0.00	0.00	0.00	0.00	0.00	0.00	0.00	0.00	0.00
0.00	0.00	0.00	0.00	0.00	0.00	0.00	0.00	0.00
0.00	0.00	0.00	0.00	0.00	0.00	0.00	2.00	0.00
0.00	0.00	0.00	0.00	0.00	0.00	0.00	2.00	0.00
0.00	0.00	0.00	0.00	0.00	0.00	0.00	2.00	0.00
0.00	0.00	0.00	0.00	0.00	2.00	0.00	0.00	0.00

0.00	0.00	2.00	0.00	0.00	0.00	0.00	0.00	0.00
0.00	0.00	2.00	0.00	0.00	0.00	0.00	0.00	0.00
0.00	2.00	0.00	0.00	0.00	0.00	0.00	0.00	0.00
2.00	0.00	0.00	0.00	0.00	0.00	0.00	0.00	0.00
0.00	0.00	0.00	0.00	0.00	0.00	0.00	0.00	0.00
0.00	0.00	0.00	0.00	0.00	0.00	0.00	0.00	0.00
0.00	0.00	0.00	0.00	0.00	0.00	0.00	0.00	0.00
0.00	0.00	0.00	0.00	0.00	0.00	0.00	0.00	0.00
0.00	0.00	0.00	0.00	0.00	0.00	0.00	0.00	0.00
0.00	0.00	0.00	0.00	0.00	0.00	0.00	0.00	0.00
0.00	0.00	0.00	0.00	0.00	0.00	0.00	0.00	0.00
0.00	0.00	0.00	0.00	0.00	0.00	0.00	0.00	0.00
0.00	0.00	0.00	0.00	0.00	0.00	0.00	0.00	0.00
0.00	0.00	0.00	0.00	0.00	0.00	0.00	0.00	0.00
0.00	0.00	0.00	0.00	0.00	0.00	0.00	0.00	0.00
0.00	0.00	0.00	0.00	0.00	0.00	0.00	0.00	0.00
0.00	0.00	0.00	0.00	0.00	0.00	0.00	0.00	0.00
0.00	0.00	0.00	0.00	0.00	0.00	0.00	0.00	0.00
0.00	0.00	0.00	0.00	0.00	0.00	0.00	0.00	0.00
0.00	0.00	0.00	0.00	0.00	0.00	0.00	0.00	0.00
0.00	0.00	0.00	0.00	0.00	0.00	0.00	0.00	0.00
0.00	0.00	0.00	0.00	0.00	0.00	0.00	0.00	0.00
0.00	0.00	0.00	0.00	0.00	0.00	0.00	0.00	0.00
0.00	0.00	0.00	0.00	0.00	0.00	0.00	0.00	0.00
0.00	0.00	0.00	0.00	0.00	0.00	0.00	0.00	0.00
0.00	0.00	0.00	0.00	0.00	0.00	0.00	0.00	0.00
0.00	0.00	0.00	0.00	0.00	0.00	0.00	0.00	0.00
0.00	0.00	0.00	0.00	0.00	0.00	0.00	0.00	0.00
0.00	0.00	0.00	0.00	0.00	0.00	0.00	0.00	0.00
0.00	0.00	0.00	0.00	0.00	0.00	0.00	0.00	0.00
0.00	0.00	0.00	0.00	0.00	0.00	0.00	0.00	0.00
0.00	0.00	0.00	0.00	0.00	0.00	0.00	0.00	0.00
0.00	0.00	0.00	0.00	0.00	0.00	0.00	0.00	0.00

C11	D9	C10	C9	C8	C7	D8	D7	D6
63924.00	5095.00	172.00	94.00	104.00	23147.00	54473.00	58.00	88.00
0.00	291.00	0.00	632.00	1269.00	0.00	0.00	0.00	0.00
1957.00	29967.00	44974.00	45992.00	33806.00	3203.00	2249.00	36275.00	36538.00
0.00	0.00	0.00	0.00	0.00	11.00	13.00	4572.00	4.00
57.00	0.00	0.00	0.00	0.00	0.00	0.00	0.00	0.00
59.00	0.00	0.00	0.00	31.00	0.00	0.00	0.00	0.00
103.00	22.00	91.00	81.00	0.00	86.00	0.00	0.00	0.00
0.00	0.00	0.00	0.00	0.00	0.00	0.00	0.00	0.00
0.00	1032.00	108.00	0.00	17.00	40.00	113.00	4.00	0.00
0.00	0.00	0.00	0.00	0.00	0.00	0.00	0.00	0.00
0.00	12.00	0.00	0.00	46.00	0.00	55.00	0.00	0.00
0.00	39.00	0.00	0.00	35.00	14662.00	0.00	0.00	0.00
0.00	138.00	0.00	0.00	11.00	17.00	60.00	3.00	0.00
5.00	0.00	0.00	0.00	0.00	0.00	0.00	0.00	2.00
0.00	0.00	0.00	0.00	0.00	0.00	0.00	0.00	0.00
0.00	0.00	0.00	0.00	0.00	0.00	0.00	0.00	0.00
35.00	0.00	0.00	0.00	0.00	0.00	0.00	0.00	0.00
0.00	0.00	0.00	0.00	0.00	0.00	0.00	0.00	0.00
83.00	5722.00	38.00	43.00	51.00	29.00	152.00	15.00	7.00
20.00	0.00	2403.00	38.00	0.00	52.00	0.00	0.00	1287.00
0.00	0.00	0.00	0.00	0.00	0.00	0.00	0.00	0.00
0.00	0.00	0.00	0.00	0.00	0.00	31.00	0.00	0.00
0.00	0.00	0.00	12.00	0.00	0.00	0.00	0.00	0.00
0.00	0.00	0.00	0.00	0.00	0.00	0.00	0.00	0.00
0.00	0.00	0.00	0.00	0.00	0.00	0.00	0.00	0.00
87.00	2725.00	602.00	3568.00	487.00	439.00	80.00	0.00	3573.00
0.00	0.00	0.00	0.00	0.00	0.00	0.00	0.00	0.00
0.00	0.00	0.00	0.00	10.00	0.00	13.00	5.00	5.00
0.00	0.00	0.00	0.00	0.00	0.00	0.00	0.00	0.00
0.00	0.00	0.00	0.00	0.00	12794.00	0.00	0.00	29.00
44.00	1183.00	1110.00	1178.00	355.00	135.00	64.00	793.00	2782.00
0.00	0.00	0.00	0.00	0.00	0.00	52.00	0.00	0.00
0.00	0.00	0.00	0.00	0.00	3288.00	0.00	0.00	0.00
0.00	0.00	0.00	0.00	0.00	13.00	0.00	0.00	0.00
0.00	0.00	0.00	0.00	0.00	0.00	0.00	0.00	0.00

0.00	0.00	0.00	0.00	0.00	0.00	5.00	0.00	0.00
0.00	222.00	16.00	14.00	434.00	0.00	0.00	0.00	0.00
7.00	0.00	1005.00	0.00	7.00	0.00	14.00	154.00	416.00
0.00	0.00	0.00	0.00	0.00	0.00	0.00	0.00	0.00
0.00	0.00	0.00	0.00	0.00	0.00	0.00	0.00	0.00
0.00	0.00	0.00	0.00	0.00	0.00	0.00	0.00	0.00
0.00	0.00	0.00	0.00	0.00	0.00	0.00	0.00	0.00
7.00	43.00	465.00	1141.00	253.00	0.00	0.00	993.00	0.00
0.00	0.00	98.00	661.00	747.00	0.00	63.00	88.00	1810.00
0.00	0.00	0.00	0.00	0.00	0.00	0.00	0.00	0.00
0.00	0.00	0.00	0.00	0.00	0.00	0.00	0.00	0.00
4.00	0.00	0.00	0.00	0.00	0.00	0.00	0.00	480.00
0.00	0.00	0.00	0.00	227.00	0.00	0.00	0.00	0.00
23.00	782.00	0.00	79.00	941.00	0.00	43.00	321.00	0.00
0.00	0.00	0.00	0.00	0.00	0.00	0.00	0.00	0.00
0.00	0.00	1049.00	0.00	0.00	77.00	0.00	28.00	0.00
0.00	0.00	0.00	0.00	0.00	0.00	0.00	0.00	0.00
0.00	0.00	0.00	0.00	0.00	0.00	0.00	0.00	0.00
0.00	0.00	0.00	0.00	0.00	0.00	0.00	0.00	0.00
0.00	0.00	0.00	0.00	0.00	0.00	0.00	0.00	0.00
0.00	0.00	0.00	134.00	61.00	0.00	0.00	1294.00	0.00
0.00	0.00	0.00	0.00	0.00	0.00	3.00	0.00	0.00
0.00	0.00	0.00	0.00	0.00	4157.00	0.00	0.00	0.00
14.00	0.00	0.00	0.00	5.00	8.00	28.00	2.00	4538.00
9.00	0.00	414.00	1476.00	0.00	66.00	0.00	152.00	750.00
0.00	0.00	0.00	0.00	0.00	0.00	0.00	419.00	0.00
6.00	592.00	115.00	81.00	46.00	0.00	5.00	2.00	545.00
0.00	0.00	0.00	0.00	0.00	0.00	0.00	0.00	0.00
0.00	0.00	0.00	0.00	0.00	0.00	0.00	0.00	0.00
0.00	0.00	0.00	0.00	0.00	0.00	0.00	0.00	0.00
48.00	0.00	2753.00	0.00	0.00	21.00	0.00	470.00	0.00
0.00	0.00	0.00	0.00	0.00	0.00	0.00	0.00	0.00
12.00	0.00	0.00	0.00	0.00	12.00	12.00	0.00	633.00
0.00	0.00	0.00	0.00	0.00	0.00	0.00	0.00	0.00
0.00	0.00	0.00	0.00	0.00	0.00	0.00	0.00	0.00
0.00	0.00	4.00	0.00	0.00	0.00	5.00	4.00	0.00
0.00	0.00	0.00	0.00	0.00	0.00	0.00	0.00	0.00

0.00	0.00	0.00	0.00	0.00	0.00	0.00	0.00	0.00
3.00	2.00	0.00	0.00	0.00	0.00	0.00	0.00	0.00
0.00	0.00	0.00	0.00	0.00	0.00	0.00	0.00	0.00
0.00	0.00	0.00	0.00	0.00	0.00	0.00	0.00	0.00
35.00	0.00	2133.00	0.00	0.00	0.00	0.00	0.00	8.00
0.00	2909.00	3.00	0.00	0.00	0.00	0.00	0.00	0.00
0.00	0.00	0.00	0.00	0.00	0.00	0.00	0.00	0.00
10.00	2799.00	0.00	5.00	0.00	0.00	0.00	0.00	0.00
3.00	0.00	2.00	2540.00	0.00	0.00	0.00	0.00	0.00
0.00	0.00	0.00	0.00	0.00	0.00	0.00	0.00	0.00
11.00	0.00	0.00	0.00	0.00	0.00	0.00	0.00	5.00
0.00	0.00	0.00	0.00	0.00	0.00	0.00	0.00	0.00
0.00	0.00	410.00	0.00	0.00	0.00	0.00	0.00	0.00
0.00	0.00	0.00	0.00	171.00	0.00	6.00	4.00	0.00
0.00	0.00	0.00	0.00	0.00	0.00	0.00	12.00	0.00
0.00	0.00	0.00	0.00	0.00	0.00	0.00	0.00	0.00
0.00	0.00	0.00	0.00	0.00	0.00	0.00	0.00	0.00
0.00	0.00	0.00	0.00	0.00	0.00	0.00	0.00	0.00
0.00	0.00	0.00	701.00	305.00	0.00	0.00	0.00	0.00
0.00	0.00	0.00	0.00	0.00	2.00	0.00	2197.00	0.00
9.00	0.00	14.00	9.00	21.00	27.00	381.00	13.00	16.00
39.00	13.00	4.00	12.00	6.00	19.00	27.00	0.00	0.00
0.00	0.00	377.00	0.00	0.00	0.00	15.00	0.00	0.00
0.00	0.00	0.00	0.00	0.00	0.00	0.00	0.00	0.00
0.00	0.00	0.00	0.00	0.00	0.00	25.00	0.00	0.00
0.00	0.00	0.00	0.00	0.00	0.00	0.00	588.00	0.00
0.00	0.00	0.00	0.00	0.00	0.00	0.00	0.00	0.00
0.00	0.00	0.00	0.00	0.00	0.00	0.00	1107.00	0.00
0.00	0.00	0.00	16.00	33.00	0.00	197.00	0.00	0.00
0.00	0.00	0.00	0.00	0.00	0.00	0.00	0.00	1734.00
0.00	0.00	0.00	0.00	0.00	0.00	0.00	0.00	0.00
0.00	0.00	0.00	0.00	717.00	4.00	0.00	513.00	0.00
0.00	0.00	0.00	0.00	428.00	5.00	8.00	0.00	378.00
99.00	20.00	0.00	34.00	29.00	0.00	55.00	35.00	40.00
0.00	0.00	0.00	0.00	0.00	0.00	0.00	0.00	0.00
0.00	0.00	0.00	0.00	1711.00	6.00	0.00	0.00	0.00
0.00	0.00	0.00	0.00	0.00	0.00	0.00	0.00	0.00

0.00	0.00	0.00	0.00	0.00	0.00	0.00	0.00	0.00
0.00	0.00	0.00	0.00	0.00	0.00	0.00	0.00	0.00
0.00	0.00	0.00	0.00	0.00	0.00	0.00	6.00	0.00
0.00	0.00	0.00	197.00	1260.00	0.00	12.00	0.00	0.00
0.00	27.00	0.00	0.00	316.00	45.00	0.00	0.00	0.00
10.00	0.00	0.00	0.00	652.00	0.00	0.00	0.00	853.00
7.00	0.00	0.00	0.00	0.00	0.00	0.00	0.00	0.00
0.00	0.00	0.00	0.00	959.00	0.00	0.00	496.00	0.00
8.00	0.00	43.00	36.00	24.00	19.00	0.00	0.00	0.00
0.00	0.00	0.00	0.00	0.00	0.00	0.00	0.00	0.00
0.00	0.00	0.00	0.00	0.00	0.00	0.00	0.00	0.00
0.00	0.00	0.00	0.00	0.00	0.00	0.00	0.00	0.00
9.00	0.00	0.00	0.00	0.00	0.00	3.00	0.00	1421.00
60.00	0.00	24.00	0.00	25.00	84.00	56.00	23.00	0.00
0.00	0.00	0.00	0.00	0.00	0.00	0.00	0.00	0.00
0.00	0.00	0.00	0.00	0.00	0.00	0.00	0.00	0.00
0.00	0.00	0.00	0.00	0.00	0.00	0.00	0.00	0.00
0.00	0.00	0.00	0.00	0.00	0.00	0.00	0.00	0.00
0.00	0.00	0.00	0.00	0.00	0.00	0.00	0.00	0.00
0.00	0.00	0.00	0.00	0.00	3.00	0.00	1219.00	0.00
0.00	0.00	0.00	0.00	0.00	0.00	0.00	0.00	0.00
0.00	0.00	0.00	0.00	0.00	0.00	0.00	0.00	0.00
0.00	0.00	0.00	0.00	0.00	0.00	0.00	0.00	1033.00
21.00	1084.00	0.00	0.00	0.00	0.00	6.00	0.00	0.00
0.00	0.00	0.00	0.00	0.00	0.00	0.00	0.00	0.00
0.00	0.00	0.00	0.00	0.00	0.00	0.00	0.00	0.00
0.00	0.00	0.00	0.00	0.00	0.00	0.00	0.00	0.00
0.00	0.00	388.00	0.00	0.00	0.00	0.00	0.00	0.00
0.00	0.00	0.00	0.00	0.00	0.00	0.00	0.00	0.00
0.00	0.00	0.00	0.00	0.00	0.00	0.00	0.00	0.00
36.00	8.00	13.00	17.00	24.00	40.00	83.00	19.00	17.00
0.00	0.00	0.00	0.00	0.00	0.00	0.00	0.00	0.00
0.00	0.00	0.00	78.00	0.00	0.00	0.00	0.00	0.00
0.00	0.00	0.00	0.00	0.00	0.00	0.00	0.00	0.00
0.00	0.00	0.00	0.00	745.00	4.00	0.00	0.00	0.00
0.00	0.00	0.00	0.00	0.00	5.00	0.00	1065.00	0.00
0.00	0.00	0.00	0.00	0.00	0.00	0.00	1050.00	0.00

0.00	0.00	0.00	0.00	0.00	0.00	0.00	0.00	0.00
108.00	40.00	87.00	82.00	20.00	57.00	63.00	16.00	22.00
27.00	8.00	9.00	8.00	0.00	17.00	40.00	0.00	12.00
0.00	991.00	0.00	0.00	0.00	0.00	0.00	0.00	0.00
0.00	0.00	0.00	0.00	0.00	0.00	0.00	0.00	0.00
0.00	0.00	0.00	0.00	571.00	0.00	8.00	0.00	0.00
0.00	0.00	0.00	0.00	0.00	0.00	0.00	988.00	0.00
0.00	0.00	0.00	0.00	0.00	0.00	0.00	0.00	0.00
0.00	0.00	0.00	0.00	0.00	0.00	0.00	0.00	0.00
0.00	0.00	6.00	828.00	0.00	0.00	0.00	0.00	0.00
0.00	0.00	0.00	0.00	0.00	0.00	0.00	0.00	0.00
0.00	0.00	0.00	0.00	0.00	0.00	0.00	0.00	0.00
0.00	0.00	0.00	0.00	0.00	0.00	0.00	0.00	0.00
0.00	93.00	419.00	0.00	0.00	0.00	0.00	0.00	0.00
0.00	0.00	0.00	0.00	0.00	0.00	3.00	0.00	0.00
0.00	0.00	0.00	0.00	846.00	3.00	0.00	0.00	0.00
0.00	0.00	0.00	0.00	0.00	0.00	0.00	0.00	0.00
0.00	0.00	25.00	0.00	6.00	0.00	0.00	0.00	7.00
0.00	0.00	0.00	0.00	840.00	0.00	0.00	0.00	0.00
0.00	0.00	0.00	0.00	0.00	0.00	0.00	0.00	0.00
0.00	0.00	0.00	0.00	0.00	0.00	0.00	0.00	0.00
0.00	0.00	0.00	0.00	0.00	0.00	0.00	0.00	0.00
0.00	0.00	0.00	0.00	0.00	2.00	0.00	530.00	0.00
0.00	0.00	0.00	0.00	0.00	0.00	0.00	0.00	0.00
0.00	0.00	0.00	0.00	0.00	0.00	0.00	0.00	0.00
0.00	0.00	0.00	0.00	0.00	0.00	0.00	0.00	0.00
21.00	0.00	9.00	8.00	18.00	38.00	55.00	14.00	14.00
0.00	0.00	0.00	5.00	0.00	0.00	0.00	0.00	0.00
0.00	0.00	0.00	0.00	0.00	0.00	0.00	0.00	0.00
0.00	0.00	0.00	0.00	0.00	0.00	0.00	0.00	0.00
0.00	0.00	0.00	0.00	0.00	0.00	0.00	0.00	0.00
17.00	0.00	0.00	0.00	0.00	0.00	9.00	0.00	0.00
0.00	0.00	0.00	0.00	0.00	0.00	0.00	0.00	0.00
0.00	0.00	0.00	0.00	0.00	0.00	0.00	0.00	0.00
0.00	0.00	0.00	0.00	0.00	0.00	0.00	0.00	0.00
0.00	0.00	0.00	0.00	7.00	28.00	49.00	7.00	14.00
0.00	0.00	0.00	0.00	0.00	25.00	0.00	0.00	0.00

0.00	0.00	0.00	0.00	0.00	0.00	0.00	0.00	0.00
0.00	0.00	0.00	0.00	0.00	0.00	0.00	0.00	0.00
0.00	0.00	0.00	0.00	0.00	0.00	0.00	0.00	0.00
0.00	0.00	0.00	0.00	0.00	0.00	0.00	718.00	0.00
0.00	0.00	703.00	0.00	0.00	0.00	0.00	0.00	0.00
0.00	0.00	0.00	0.00	0.00	0.00	0.00	0.00	0.00
0.00	0.00	0.00	0.00	0.00	0.00	0.00	0.00	0.00
19.00	19.00	0.00	29.00	0.00	0.00	21.00	536.00	0.00
0.00	0.00	0.00	0.00	0.00	0.00	0.00	0.00	0.00
15.00	0.00	16.00	18.00	16.00	0.00	0.00	0.00	27.00
0.00	0.00	0.00	0.00	0.00	0.00	0.00	0.00	0.00
0.00	0.00	0.00	661.00	0.00	0.00	0.00	0.00	0.00
0.00	0.00	4.00	0.00	10.00	0.00	39.00	0.00	0.00
0.00	0.00	626.00	0.00	0.00	0.00	0.00	0.00	0.00
0.00	0.00	0.00	0.00	0.00	0.00	0.00	640.00	0.00
0.00	642.00	0.00	0.00	0.00	0.00	0.00	0.00	0.00
0.00	0.00	0.00	0.00	0.00	0.00	0.00	0.00	0.00
0.00	0.00	0.00	0.00	612.00	2.00	0.00	0.00	0.00
0.00	0.00	0.00	0.00	0.00	0.00	0.00	0.00	0.00
0.00	620.00	0.00	0.00	0.00	0.00	0.00	0.00	0.00
0.00	0.00	0.00	614.00	0.00	0.00	0.00	0.00	0.00
0.00	0.00	0.00	0.00	609.00	0.00	0.00	0.00	0.00
0.00	0.00	0.00	0.00	0.00	0.00	0.00	0.00	0.00
0.00	0.00	0.00	0.00	0.00	0.00	0.00	0.00	0.00
0.00	0.00	0.00	0.00	586.00	0.00	0.00	0.00	0.00
0.00	0.00	0.00	0.00	0.00	0.00	0.00	0.00	0.00
0.00	0.00	0.00	0.00	0.00	0.00	0.00	0.00	0.00
0.00	0.00	0.00	0.00	567.00	0.00	5.00	0.00	0.00
0.00	0.00	0.00	0.00	0.00	0.00	0.00	0.00	0.00
0.00	0.00	0.00	0.00	0.00	0.00	0.00	0.00	0.00
5.00	0.00	0.00	0.00	0.00	0.00	6.00	0.00	557.00
0.00	0.00	0.00	45.00	0.00	0.00	0.00	0.00	0.00
0.00	0.00	0.00	0.00	0.00	0.00	0.00	0.00	0.00
23.00	5.00	9.00	17.00	0.00	0.00	0.00	0.00	0.00
0.00	0.00	0.00	0.00	0.00	0.00	0.00	0.00	0.00
0.00	0.00	0.00	0.00	0.00	0.00	0.00	0.00	0.00
6.00	7.00	0.00	0.00	15.00	39.00	49.00	6.00	0.00

0.00	552.00	0.00	0.00	0.00	0.00	0.00	0.00	0.00
0.00	0.00	0.00	0.00	0.00	0.00	0.00	0.00	0.00
0.00	0.00	0.00	0.00	0.00	0.00	0.00	0.00	0.00
9.00	6.00	0.00	2.00	0.00	0.00	4.00	7.00	0.00
0.00	0.00	0.00	0.00	0.00	0.00	0.00	0.00	0.00
0.00	505.00	0.00	0.00	0.00	0.00	0.00	0.00	0.00
0.00	0.00	0.00	0.00	0.00	0.00	0.00	0.00	0.00
0.00	0.00	0.00	0.00	0.00	0.00	0.00	506.00	0.00
0.00	0.00	0.00	0.00	0.00	0.00	7.00	0.00	0.00
0.00	0.00	0.00	0.00	0.00	0.00	0.00	0.00	0.00
0.00	0.00	0.00	0.00	8.00	17.00	23.00	5.00	4.00
0.00	0.00	0.00	0.00	0.00	0.00	0.00	0.00	0.00
0.00	0.00	0.00	0.00	0.00	0.00	0.00	0.00	0.00
0.00	0.00	0.00	0.00	0.00	0.00	0.00	0.00	0.00
0.00	0.00	0.00	0.00	0.00	0.00	0.00	0.00	0.00
3.00	0.00	0.00	0.00	0.00	0.00	0.00	0.00	0.00
0.00	0.00	0.00	0.00	0.00	0.00	0.00	0.00	0.00
0.00	0.00	0.00	0.00	482.00	0.00	0.00	0.00	0.00
0.00	0.00	0.00	0.00	0.00	0.00	0.00	0.00	0.00
0.00	0.00	0.00	0.00	484.00	0.00	0.00	0.00	0.00
44.00	0.00	17.00	0.00	0.00	25.00	0.00	0.00	0.00
0.00	0.00	0.00	0.00	0.00	0.00	0.00	0.00	0.00
0.00	0.00	0.00	0.00	0.00	0.00	0.00	0.00	0.00
0.00	0.00	0.00	0.00	0.00	0.00	0.00	0.00	0.00
0.00	0.00	0.00	0.00	0.00	0.00	0.00	0.00	0.00
0.00	0.00	0.00	11.00	0.00	51.00	43.00	0.00	10.00
0.00	0.00	0.00	0.00	0.00	0.00	0.00	0.00	0.00
0.00	0.00	0.00	0.00	0.00	0.00	0.00	0.00	0.00
0.00	0.00	0.00	0.00	0.00	0.00	0.00	0.00	0.00
0.00	2.00	0.00	0.00	0.00	0.00	0.00	0.00	0.00
0.00	0.00	0.00	0.00	0.00	0.00	0.00	0.00	0.00
0.00	0.00	0.00	0.00	0.00	0.00	0.00	0.00	0.00
0.00	0.00	0.00	0.00	0.00	0.00	0.00	0.00	0.00
0.00	0.00	0.00	0.00	0.00	0.00	0.00	0.00	0.00
0.00	0.00	0.00	0.00	0.00	0.00	0.00	0.00	0.00
0.00	0.00	0.00	0.00	0.00	0.00	0.00	452.00	0.00
0.00	0.00	0.00	0.00	447.00	2.00	0.00	0.00	0.00

0.00	0.00	0.00	0.00	0.00	0.00	0.00	0.00	0.00
0.00	0.00	0.00	0.00	0.00	0.00	0.00	0.00	0.00
0.00	0.00	0.00	0.00	0.00	0.00	0.00	0.00	0.00
0.00	0.00	0.00	0.00	0.00	0.00	0.00	0.00	0.00
0.00	0.00	0.00	0.00	0.00	66.00	88.00	13.00	0.00
0.00	0.00	0.00	0.00	0.00	0.00	0.00	0.00	0.00
0.00	0.00	0.00	0.00	0.00	0.00	0.00	0.00	0.00
0.00	0.00	421.00	0.00	0.00	0.00	0.00	0.00	0.00
0.00	0.00	0.00	0.00	0.00	0.00	0.00	0.00	0.00
0.00	0.00	0.00	0.00	0.00	0.00	0.00	0.00	0.00
0.00	400.00	0.00	0.00	0.00	0.00	0.00	0.00	0.00
0.00	0.00	0.00	0.00	0.00	0.00	0.00	0.00	0.00
0.00	0.00	0.00	0.00	0.00	0.00	0.00	0.00	0.00
0.00	0.00	0.00	0.00	0.00	0.00	0.00	0.00	0.00
0.00	0.00	0.00	0.00	0.00	0.00	0.00	0.00	0.00
0.00	0.00	0.00	0.00	0.00	0.00	0.00	0.00	0.00
0.00	0.00	0.00	0.00	0.00	15.00	17.00	0.00	7.00
0.00	0.00	0.00	0.00	0.00	0.00	0.00	0.00	0.00
0.00	0.00	0.00	0.00	0.00	0.00	0.00	0.00	0.00
0.00	0.00	0.00	0.00	0.00	140.00	0.00	0.00	0.00
0.00	0.00	0.00	0.00	0.00	0.00	0.00	0.00	0.00
0.00	0.00	0.00	0.00	0.00	0.00	0.00	0.00	0.00
0.00	0.00	0.00	0.00	0.00	0.00	0.00	0.00	0.00
0.00	0.00	0.00	0.00	0.00	0.00	0.00	0.00	0.00
21.00	0.00	0.00	0.00	0.00	0.00	0.00	0.00	0.00
0.00	0.00	0.00	0.00	0.00	0.00	0.00	0.00	0.00
0.00	0.00	0.00	0.00	0.00	0.00	0.00	0.00	0.00
0.00	0.00	0.00	0.00	0.00	0.00	0.00	0.00	0.00
0.00	0.00	0.00	0.00	0.00	0.00	0.00	0.00	0.00
0.00	0.00	13.00	0.00	10.00	0.00	9.00	0.00	0.00
0.00	0.00	0.00	0.00	0.00	0.00	0.00	0.00	0.00
0.00	0.00	0.00	0.00	0.00	0.00	0.00	0.00	0.00
0.00	0.00	0.00	0.00	0.00	0.00	0.00	0.00	0.00
0.00	0.00	0.00	0.00	0.00	0.00	0.00	0.00	0.00
0.00	0.00	0.00	0.00	0.00	0.00	0.00	0.00	0.00
9.00	0.00	0.00	0.00	0.00	0.00	0.00	0.00	0.00
0.00	0.00	0.00	0.00	0.00	0.00	0.00	0.00	0.00

0.00	0.00	0.00	0.00	0.00	0.00	0.00	0.00	0.00
0.00	0.00	0.00	0.00	0.00	0.00	0.00	6.00	0.00
0.00	0.00	0.00	0.00	0.00	0.00	0.00	0.00	0.00
0.00	0.00	0.00	0.00	0.00	0.00	0.00	0.00	0.00
0.00	0.00	17.00	0.00	0.00	64.00	0.00	0.00	0.00
0.00	0.00	0.00	0.00	0.00	0.00	0.00	0.00	0.00
78.00	20.00	29.00	25.00	0.00	0.00	0.00	0.00	0.00
0.00	0.00	0.00	0.00	0.00	0.00	0.00	0.00	0.00
0.00	0.00	0.00	0.00	0.00	0.00	0.00	0.00	0.00
0.00	0.00	0.00	0.00	6.00	11.00	9.00	7.00	0.00
0.00	0.00	0.00	9.00	0.00	0.00	22.00	0.00	4.00
0.00	0.00	0.00	0.00	0.00	0.00	0.00	0.00	0.00
0.00	0.00	0.00	0.00	0.00	0.00	0.00	0.00	0.00
7.00	0.00	0.00	2.00	0.00	12.00	14.00	6.00	0.00
0.00	0.00	0.00	0.00	0.00	0.00	0.00	0.00	0.00
0.00	0.00	0.00	0.00	0.00	0.00	0.00	0.00	0.00
0.00	0.00	0.00	0.00	0.00	0.00	0.00	0.00	0.00
0.00	0.00	0.00	0.00	93.00	0.00	0.00	0.00	0.00
0.00	0.00	0.00	0.00	0.00	0.00	0.00	0.00	0.00
0.00	0.00	0.00	0.00	0.00	0.00	0.00	0.00	0.00
0.00	0.00	0.00	0.00	0.00	0.00	0.00	0.00	0.00
0.00	0.00	0.00	0.00	0.00	0.00	0.00	0.00	0.00
0.00	0.00	0.00	0.00	0.00	0.00	0.00	0.00	0.00
0.00	0.00	0.00	0.00	0.00	0.00	0.00	0.00	0.00
0.00	0.00	0.00	0.00	0.00	0.00	0.00	0.00	0.00
0.00	0.00	0.00	0.00	0.00	0.00	0.00	0.00	0.00
0.00	113.00	0.00	0.00	0.00	0.00	0.00	0.00	0.00
0.00	0.00	0.00	0.00	0.00	0.00	0.00	0.00	0.00
0.00	0.00	0.00	0.00	0.00	0.00	0.00	0.00	0.00
0.00	0.00	0.00	0.00	0.00	0.00	0.00	0.00	0.00
0.00	0.00	301.00	0.00	0.00	0.00	0.00	0.00	0.00
0.00	0.00	0.00	0.00	0.00	0.00	0.00	301.00	0.00
0.00	0.00	0.00	0.00	0.00	0.00	0.00	0.00	0.00
0.00	0.00	0.00	0.00	0.00	0.00	0.00	0.00	0.00
0.00	0.00	0.00	0.00	0.00	0.00	0.00	0.00	0.00
0.00	0.00	0.00	0.00	0.00	0.00	0.00	0.00	0.00
0.00	0.00	0.00	0.00	0.00	0.00	0.00	0.00	0.00

0.00	0.00	0.00	0.00	0.00	0.00	0.00	0.00	0.00
0.00	0.00	0.00	0.00	0.00	0.00	0.00	0.00	0.00
0.00	0.00	0.00	0.00	3.00	13.00	20.00	0.00	0.00
0.00	0.00	0.00	0.00	0.00	0.00	0.00	282.00	0.00
0.00	0.00	0.00	0.00	0.00	0.00	0.00	0.00	0.00
11.00	6.00	0.00	5.00	0.00	0.00	12.00	0.00	3.00
0.00	0.00	0.00	0.00	0.00	0.00	0.00	0.00	0.00
0.00	0.00	0.00	0.00	0.00	0.00	0.00	0.00	0.00
0.00	0.00	0.00	0.00	0.00	0.00	0.00	0.00	0.00
0.00	0.00	16.00	0.00	0.00	0.00	0.00	0.00	0.00
0.00	263.00	0.00	0.00	0.00	0.00	0.00	0.00	0.00
0.00	0.00	0.00	0.00	0.00	0.00	0.00	0.00	0.00
0.00	0.00	0.00	0.00	0.00	0.00	0.00	0.00	0.00
0.00	0.00	0.00	0.00	263.00	0.00	0.00	0.00	0.00
0.00	0.00	0.00	19.00	0.00	0.00	16.00	0.00	0.00
0.00	0.00	0.00	0.00	0.00	0.00	0.00	0.00	0.00
0.00	0.00	0.00	0.00	0.00	0.00	0.00	0.00	0.00
0.00	0.00	0.00	0.00	0.00	0.00	0.00	0.00	0.00
0.00	0.00	0.00	0.00	0.00	0.00	0.00	0.00	0.00
0.00	0.00	0.00	0.00	0.00	0.00	0.00	0.00	0.00
0.00	0.00	0.00	0.00	0.00	0.00	23.00	0.00	0.00
0.00	0.00	0.00	0.00	0.00	0.00	0.00	0.00	0.00
0.00	0.00	2.00	0.00	186.00	0.00	0.00	0.00	0.00
0.00	0.00	0.00	0.00	0.00	20.00	26.00	4.00	0.00
0.00	0.00	0.00	0.00	0.00	47.00	0.00	0.00	0.00
0.00	0.00	0.00	0.00	0.00	0.00	0.00	0.00	0.00
0.00	0.00	0.00	0.00	0.00	0.00	0.00	0.00	0.00
0.00	0.00	0.00	0.00	0.00	0.00	0.00	0.00	0.00
0.00	0.00	0.00	0.00	0.00	15.00	24.00	4.00	12.00
0.00	0.00	0.00	0.00	0.00	0.00	0.00	0.00	0.00
0.00	0.00	0.00	0.00	0.00	0.00	0.00	0.00	0.00
0.00	0.00	0.00	0.00	0.00	0.00	0.00	0.00	0.00
0.00	0.00	0.00	0.00	0.00	0.00	0.00	0.00	0.00
0.00	0.00	0.00	0.00	0.00	0.00	0.00	0.00	0.00
0.00	0.00	0.00	0.00	0.00	0.00	0.00	212.00	0.00
0.00	0.00	0.00	0.00	0.00	0.00	0.00	0.00	0.00
0.00	0.00	0.00	0.00	0.00	0.00	0.00	0.00	0.00

0.00	0.00	0.00	0.00	0.00	0.00	0.00	0.00	0.00
0.00	0.00	0.00	0.00	0.00	0.00	0.00	0.00	0.00
0.00	0.00	0.00	0.00	0.00	0.00	0.00	0.00	0.00
0.00	0.00	0.00	0.00	0.00	0.00	0.00	0.00	0.00
0.00	0.00	0.00	0.00	0.00	0.00	0.00	0.00	0.00
0.00	0.00	0.00	0.00	0.00	0.00	0.00	0.00	0.00
0.00	0.00	0.00	0.00	0.00	0.00	0.00	0.00	0.00
4.00	0.00	0.00	0.00	0.00	0.00	0.00	0.00	0.00
0.00	0.00	0.00	0.00	0.00	0.00	0.00	0.00	0.00
0.00	0.00	0.00	0.00	0.00	0.00	0.00	0.00	0.00
0.00	0.00	0.00	0.00	0.00	0.00	0.00	0.00	0.00
0.00	0.00	0.00	0.00	0.00	0.00	0.00	0.00	0.00
0.00	0.00	0.00	0.00	0.00	0.00	0.00	153.00	0.00
5.00	0.00	4.00	5.00	0.00	0.00	8.00	0.00	0.00
0.00	0.00	0.00	0.00	0.00	0.00	0.00	0.00	0.00
0.00	0.00	0.00	0.00	0.00	0.00	0.00	0.00	0.00
0.00	0.00	0.00	0.00	0.00	0.00	0.00	0.00	0.00
0.00	0.00	0.00	0.00	0.00	0.00	0.00	0.00	0.00
0.00	0.00	0.00	0.00	0.00	0.00	0.00	218.00	0.00
0.00	0.00	0.00	0.00	0.00	0.00	0.00	0.00	0.00
0.00	0.00	0.00	0.00	20.00	0.00	0.00	0.00	0.00
0.00	0.00	0.00	0.00	0.00	0.00	0.00	0.00	0.00
0.00	0.00	0.00	215.00	0.00	0.00	0.00	0.00	0.00
0.00	0.00	0.00	0.00	0.00	0.00	0.00	0.00	0.00
0.00	0.00	0.00	0.00	6.00	3.00	13.00	2.00	0.00
0.00	0.00	0.00	0.00	0.00	0.00	0.00	0.00	0.00
0.00	0.00	0.00	0.00	0.00	0.00	0.00	0.00	0.00
12.00	4.00	3.00	0.00	10.00	7.00	21.00	0.00	4.00
6.00	0.00	3.00	0.00	7.00	12.00	20.00	4.00	4.00
0.00	0.00	0.00	0.00	0.00	0.00	27.00	0.00	0.00
0.00	0.00	0.00	0.00	0.00	0.00	0.00	0.00	0.00
0.00	0.00	0.00	0.00	0.00	0.00	0.00	0.00	0.00
0.00	0.00	0.00	0.00	0.00	17.00	0.00	0.00	0.00
0.00	0.00	0.00	0.00	0.00	0.00	0.00	0.00	0.00
0.00	0.00	33.00	0.00	0.00	0.00	0.00	0.00	0.00
0.00	0.00	0.00	0.00	0.00	0.00	0.00	0.00	206.00
0.00	0.00	0.00	0.00	0.00	0.00	0.00	0.00	0.00

0.00	0.00	0.00	0.00	0.00	0.00	0.00	0.00	0.00
0.00	0.00	0.00	0.00	0.00	0.00	0.00	0.00	0.00
0.00	0.00	0.00	0.00	0.00	0.00	0.00	0.00	0.00
0.00	0.00	0.00	0.00	0.00	0.00	0.00	0.00	0.00
0.00	0.00	0.00	0.00	9.00	11.00	7.00	3.00	4.00
0.00	0.00	0.00	0.00	0.00	0.00	0.00	0.00	0.00
0.00	0.00	0.00	0.00	0.00	0.00	0.00	0.00	0.00
0.00	0.00	0.00	0.00	0.00	0.00	0.00	0.00	0.00
0.00	0.00	0.00	0.00	0.00	0.00	0.00	0.00	0.00
2.00	0.00	0.00	0.00	0.00	0.00	0.00	0.00	0.00
0.00	0.00	0.00	0.00	0.00	0.00	0.00	0.00	0.00
0.00	0.00	193.00	0.00	0.00	0.00	0.00	0.00	0.00
0.00	0.00	0.00	0.00	0.00	0.00	0.00	0.00	0.00
0.00	0.00	0.00	0.00	0.00	0.00	0.00	0.00	0.00
0.00	0.00	0.00	0.00	0.00	0.00	0.00	0.00	0.00
0.00	6.00	0.00	0.00	0.00	0.00	0.00	0.00	0.00
0.00	0.00	0.00	0.00	0.00	0.00	0.00	0.00	0.00
0.00	0.00	0.00	0.00	0.00	0.00	0.00	0.00	0.00
0.00	0.00	0.00	0.00	0.00	0.00	0.00	0.00	0.00
0.00	0.00	0.00	0.00	0.00	7.00	5.00	5.00	0.00
3.00	5.00	0.00	4.00	5.00	3.00	0.00	4.00	0.00
0.00	0.00	0.00	0.00	0.00	0.00	0.00	0.00	0.00
0.00	0.00	0.00	0.00	0.00	0.00	0.00	104.00	0.00
0.00	0.00	0.00	0.00	0.00	0.00	0.00	0.00	0.00
0.00	0.00	0.00	0.00	0.00	0.00	0.00	0.00	0.00
0.00	0.00	0.00	0.00	0.00	0.00	0.00	0.00	0.00
43.00	0.00	0.00	0.00	0.00	0.00	0.00	0.00	0.00
0.00	0.00	0.00	0.00	0.00	0.00	0.00	0.00	0.00
10.00	0.00	0.00	3.00	0.00	0.00	11.00	2.00	0.00
0.00	0.00	0.00	0.00	0.00	0.00	56.00	0.00	0.00
5.00	0.00	0.00	0.00	0.00	0.00	0.00	0.00	2.00
0.00	0.00	0.00	0.00	0.00	0.00	0.00	0.00	0.00
26.00	9.00	24.00	21.00	0.00	0.00	0.00	0.00	0.00
0.00	0.00	163.00	0.00	0.00	0.00	0.00	0.00	0.00
54.00	0.00	0.00	0.00	0.00	0.00	0.00	0.00	9.00
0.00	0.00	0.00	0.00	0.00	0.00	0.00	0.00	0.00
0.00	0.00	0.00	0.00	0.00	0.00	0.00	0.00	0.00

0.00	0.00	0.00	0.00	0.00	0.00	0.00	0.00	0.00
0.00	0.00	0.00	0.00	0.00	0.00	0.00	0.00	0.00
0.00	0.00	0.00	0.00	0.00	0.00	0.00	0.00	0.00
0.00	0.00	0.00	0.00	0.00	0.00	0.00	0.00	0.00
0.00	0.00	0.00	0.00	0.00	0.00	0.00	0.00	0.00
0.00	0.00	0.00	0.00	0.00	0.00	0.00	0.00	0.00
0.00	0.00	0.00	0.00	0.00	0.00	0.00	0.00	0.00
0.00	0.00	0.00	0.00	0.00	0.00	0.00	0.00	0.00
0.00	0.00	0.00	0.00	0.00	0.00	0.00	0.00	0.00
0.00	0.00	0.00	0.00	0.00	0.00	0.00	0.00	0.00
0.00	127.00	0.00	0.00	0.00	0.00	0.00	0.00	0.00
0.00	0.00	0.00	0.00	0.00	0.00	0.00	0.00	0.00
0.00	0.00	0.00	0.00	0.00	0.00	0.00	0.00	0.00
0.00	0.00	0.00	0.00	0.00	0.00	0.00	0.00	0.00
0.00	0.00	0.00	0.00	0.00	0.00	0.00	0.00	0.00
0.00	0.00	0.00	0.00	0.00	0.00	0.00	0.00	0.00
0.00	0.00	0.00	0.00	0.00	0.00	0.00	0.00	0.00
0.00	0.00	0.00	0.00	0.00	0.00	0.00	0.00	0.00
0.00	0.00	0.00	0.00	0.00	0.00	0.00	0.00	0.00
0.00	0.00	0.00	0.00	0.00	0.00	0.00	0.00	0.00
0.00	0.00	0.00	0.00	0.00	0.00	0.00	0.00	0.00
0.00	0.00	0.00	0.00	0.00	0.00	0.00	0.00	0.00
0.00	0.00	0.00	0.00	0.00	0.00	0.00	0.00	0.00
0.00	0.00	0.00	0.00	38.00	0.00	0.00	0.00	0.00
0.00	0.00	0.00	0.00	0.00	0.00	0.00	0.00	0.00
0.00	0.00	0.00	0.00	0.00	0.00	0.00	0.00	0.00
0.00	0.00	0.00	0.00	0.00	0.00	0.00	0.00	0.00
0.00	0.00	0.00	0.00	0.00	0.00	0.00	0.00	0.00
0.00	0.00	0.00	0.00	0.00	0.00	0.00	0.00	0.00
0.00	0.00	0.00	0.00	0.00	0.00	0.00	0.00	0.00
0.00	0.00	0.00	0.00	0.00	0.00	0.00	0.00	0.00
0.00	0.00	0.00	0.00	0.00	0.00	0.00	0.00	0.00
0.00	0.00	0.00	0.00	0.00	0.00	0.00	0.00	0.00
0.00	0.00	0.00	0.00	0.00	0.00	0.00	0.00	0.00
0.00	0.00	0.00	0.00	0.00	0.00	0.00	0.00	0.00
0.00	0.00	0.00	0.00	0.00	0.00	0.00	0.00	0.00
0.00	0.00	0.00	0.00	0.00	0.00	0.00	0.00	0.00

15.00	0.00	0.00	0.00	0.00	0.00	17.00	0.00	0.00
0.00	0.00	0.00	0.00	0.00	0.00	0.00	0.00	0.00
0.00	0.00	0.00	0.00	0.00	0.00	0.00	0.00	0.00
0.00	0.00	0.00	0.00	0.00	0.00	0.00	71.00	0.00
0.00	0.00	0.00	0.00	0.00	0.00	0.00	0.00	0.00
0.00	0.00	0.00	0.00	0.00	0.00	0.00	0.00	0.00
0.00	0.00	0.00	0.00	0.00	0.00	0.00	0.00	0.00
0.00	0.00	6.00	0.00	0.00	9.00	0.00	0.00	0.00
0.00	0.00	0.00	0.00	0.00	0.00	0.00	0.00	0.00
0.00	0.00	0.00	0.00	0.00	0.00	0.00	145.00	0.00
0.00	0.00	0.00	0.00	0.00	0.00	0.00	0.00	0.00
0.00	0.00	0.00	0.00	0.00	0.00	0.00	0.00	0.00
0.00	0.00	0.00	0.00	0.00	0.00	0.00	0.00	0.00
0.00	0.00	0.00	0.00	0.00	0.00	0.00	0.00	0.00
0.00	0.00	0.00	0.00	0.00	0.00	0.00	0.00	0.00
0.00	0.00	0.00	0.00	0.00	0.00	0.00	0.00	0.00
0.00	0.00	0.00	0.00	0.00	0.00	0.00	0.00	0.00
0.00	0.00	0.00	0.00	0.00	0.00	0.00	0.00	0.00
31.00	7.00	11.00	14.00	3.00	0.00	0.00	7.00	2.00
0.00	0.00	0.00	0.00	0.00	0.00	0.00	0.00	0.00
0.00	0.00	0.00	0.00	0.00	0.00	0.00	0.00	0.00
0.00	0.00	0.00	0.00	0.00	5.00	0.00	0.00	0.00
0.00	0.00	0.00	0.00	0.00	0.00	0.00	0.00	0.00
0.00	0.00	0.00	4.00	11.00	0.00	0.00	0.00	0.00
0.00	143.00	0.00	0.00	0.00	0.00	0.00	0.00	0.00
0.00	0.00	0.00	0.00	0.00	5.00	0.00	0.00	0.00
0.00	0.00	0.00	0.00	0.00	0.00	0.00	0.00	0.00
0.00	0.00	0.00	0.00	0.00	142.00	0.00	0.00	0.00
0.00	0.00	0.00	0.00	0.00	0.00	0.00	0.00	0.00
0.00	0.00	0.00	0.00	0.00	0.00	0.00	0.00	0.00
0.00	0.00	0.00	0.00	0.00	0.00	0.00	0.00	0.00
0.00	0.00	0.00	0.00	0.00	0.00	0.00	0.00	0.00
0.00	0.00	7.00	0.00	0.00	0.00	6.00	0.00	0.00
0.00	0.00	0.00	0.00	0.00	0.00	0.00	138.00	0.00
2.00	0.00	0.00	0.00	0.00	0.00	0.00	0.00	0.00
0.00	0.00	0.00	0.00	0.00	0.00	0.00	0.00	0.00
0.00	0.00	0.00	0.00	0.00	0.00	0.00	0.00	0.00

0.00	0.00	14.00	0.00	0.00	0.00	0.00	0.00	0.00
0.00	0.00	0.00	0.00	0.00	0.00	0.00	0.00	4.00
0.00	0.00	0.00	0.00	0.00	0.00	0.00	0.00	0.00
0.00	0.00	0.00	0.00	0.00	0.00	0.00	4.00	0.00
0.00	0.00	0.00	0.00	0.00	0.00	0.00	0.00	0.00
3.00	0.00	0.00	0.00	0.00	5.00	5.00	0.00	0.00
0.00	0.00	0.00	0.00	0.00	0.00	0.00	0.00	0.00
0.00	0.00	0.00	0.00	0.00	0.00	0.00	0.00	0.00
0.00	0.00	0.00	0.00	0.00	0.00	0.00	0.00	0.00
0.00	0.00	0.00	0.00	0.00	0.00	0.00	0.00	0.00
0.00	0.00	0.00	0.00	0.00	0.00	0.00	0.00	0.00
0.00	0.00	0.00	0.00	0.00	0.00	0.00	0.00	0.00
0.00	0.00	0.00	0.00	0.00	0.00	0.00	0.00	0.00
0.00	0.00	0.00	0.00	0.00	0.00	0.00	0.00	0.00
0.00	0.00	0.00	0.00	0.00	0.00	0.00	0.00	0.00
0.00	10.00	0.00	0.00	0.00	0.00	0.00	0.00	0.00
0.00	0.00	0.00	0.00	0.00	0.00	0.00	0.00	0.00
0.00	0.00	0.00	0.00	0.00	0.00	0.00	123.00	0.00
0.00	0.00	0.00	0.00	0.00	0.00	0.00	0.00	0.00
0.00	0.00	0.00	0.00	0.00	0.00	0.00	0.00	0.00
0.00	0.00	0.00	0.00	0.00	0.00	0.00	0.00	0.00
0.00	0.00	0.00	0.00	0.00	0.00	0.00	0.00	0.00
0.00	0.00	0.00	0.00	0.00	0.00	0.00	0.00	0.00
35.00	0.00	0.00	0.00	0.00	0.00	0.00	0.00	13.00
0.00	0.00	0.00	0.00	0.00	0.00	0.00	0.00	0.00
0.00	0.00	0.00	0.00	0.00	0.00	0.00	120.00	0.00
0.00	0.00	14.00	0.00	0.00	0.00	0.00	0.00	0.00
0.00	0.00	0.00	0.00	0.00	0.00	0.00	0.00	0.00
0.00	0.00	0.00	0.00	0.00	0.00	0.00	0.00	0.00
0.00	0.00	0.00	4.00	0.00	0.00	0.00	0.00	0.00
27.00	0.00	0.00	0.00	0.00	0.00	0.00	0.00	15.00
0.00	0.00	0.00	0.00	0.00	0.00	0.00	0.00	0.00
15.00	0.00	0.00	0.00	0.00	0.00	6.00	0.00	5.00
0.00	0.00	0.00	0.00	0.00	0.00	0.00	0.00	0.00
0.00	0.00	0.00	0.00	0.00	0.00	0.00	0.00	0.00
0.00	0.00	0.00	0.00	0.00	0.00	0.00	0.00	0.00
0.00	0.00	0.00	0.00	0.00	0.00	0.00	0.00	0.00

0.00	0.00	0.00	0.00	0.00	0.00	0.00	0.00	0.00
0.00	0.00	0.00	0.00	0.00	0.00	0.00	0.00	0.00
0.00	0.00	0.00	0.00	0.00	0.00	0.00	0.00	0.00
0.00	0.00	0.00	0.00	0.00	0.00	0.00	0.00	0.00
5.00	0.00	0.00	0.00	0.00	0.00	0.00	0.00	0.00
0.00	0.00	0.00	0.00	0.00	0.00	0.00	0.00	0.00
0.00	0.00	0.00	0.00	0.00	0.00	0.00	0.00	0.00
0.00	0.00	0.00	0.00	0.00	0.00	0.00	0.00	0.00
0.00	0.00	111.00	0.00	0.00	0.00	0.00	0.00	0.00
0.00	111.00	0.00	0.00	0.00	0.00	0.00	0.00	0.00
0.00	0.00	0.00	0.00	0.00	0.00	0.00	0.00	0.00
0.00	0.00	0.00	0.00	0.00	5.00	12.00	0.00	0.00
0.00	0.00	0.00	0.00	0.00	0.00	0.00	0.00	0.00
0.00	0.00	0.00	0.00	0.00	0.00	0.00	0.00	0.00
0.00	0.00	0.00	0.00	0.00	0.00	0.00	0.00	0.00
0.00	0.00	0.00	0.00	0.00	0.00	0.00	0.00	0.00
0.00	0.00	0.00	0.00	0.00	0.00	0.00	0.00	0.00
0.00	0.00	0.00	0.00	0.00	0.00	0.00	0.00	0.00
0.00	0.00	107.00	0.00	0.00	0.00	0.00	0.00	0.00
0.00	0.00	0.00	0.00	0.00	0.00	0.00	0.00	0.00
0.00	0.00	0.00	0.00	0.00	0.00	0.00	0.00	0.00
0.00	0.00	0.00	0.00	0.00	0.00	0.00	0.00	0.00
0.00	0.00	0.00	0.00	0.00	0.00	0.00	0.00	0.00
0.00	0.00	0.00	0.00	0.00	0.00	0.00	0.00	0.00
0.00	0.00	0.00	0.00	0.00	0.00	0.00	0.00	0.00
0.00	0.00	0.00	0.00	0.00	0.00	0.00	0.00	0.00
0.00	0.00	0.00	0.00	0.00	0.00	0.00	0.00	0.00
0.00	0.00	0.00	0.00	0.00	0.00	0.00	0.00	0.00
0.00	0.00	0.00	0.00	0.00	0.00	0.00	0.00	0.00
0.00	0.00	0.00	0.00	0.00	0.00	0.00	0.00	0.00
0.00	0.00	0.00	0.00	0.00	0.00	0.00	0.00	0.00
0.00	0.00	0.00	0.00	0.00	0.00	0.00	0.00	0.00
0.00	0.00	0.00	0.00	0.00	0.00	0.00	0.00	0.00
0.00	0.00	0.00	0.00	53.00	0.00	0.00	0.00	0.00
0.00	0.00	0.00	0.00	0.00	0.00	0.00	0.00	0.00
0.00	0.00	0.00	0.00	0.00	0.00	0.00	0.00	0.00
0.00	0.00	0.00	0.00	0.00	0.00	0.00	0.00	0.00

0.00	0.00	11.00	0.00	0.00	0.00	0.00	0.00	0.00
0.00	0.00	0.00	0.00	0.00	0.00	0.00	0.00	0.00
0.00	0.00	0.00	0.00	0.00	0.00	0.00	0.00	0.00
0.00	0.00	0.00	0.00	0.00	0.00	0.00	0.00	0.00
0.00	0.00	0.00	0.00	0.00	0.00	0.00	0.00	0.00
0.00	0.00	0.00	0.00	0.00	0.00	0.00	0.00	0.00
0.00	0.00	0.00	0.00	0.00	0.00	0.00	0.00	0.00
0.00	0.00	0.00	0.00	0.00	0.00	0.00	0.00	0.00
0.00	0.00	0.00	0.00	0.00	0.00	0.00	0.00	0.00
20.00	0.00	0.00	0.00	0.00	0.00	0.00	0.00	0.00
0.00	0.00	0.00	0.00	0.00	0.00	0.00	0.00	0.00
0.00	0.00	0.00	0.00	0.00	0.00	0.00	0.00	0.00
17.00	0.00	16.00	14.00	0.00	0.00	0.00	0.00	0.00
0.00	0.00	0.00	0.00	0.00	0.00	0.00	0.00	0.00
0.00	0.00	0.00	0.00	0.00	0.00	0.00	0.00	0.00
0.00	0.00	0.00	0.00	0.00	0.00	0.00	98.00	0.00
0.00	0.00	0.00	0.00	0.00	0.00	0.00	0.00	0.00
0.00	0.00	0.00	0.00	0.00	0.00	0.00	0.00	0.00
0.00	0.00	0.00	0.00	0.00	0.00	0.00	0.00	0.00
0.00	0.00	0.00	0.00	0.00	0.00	0.00	0.00	0.00
0.00	0.00	0.00	0.00	0.00	0.00	0.00	0.00	0.00
0.00	0.00	0.00	0.00	0.00	0.00	0.00	0.00	0.00
7.00	0.00	2.00	0.00	0.00	7.00	14.00	0.00	0.00
0.00	0.00	0.00	0.00	0.00	0.00	0.00	0.00	0.00
0.00	0.00	0.00	0.00	0.00	0.00	0.00	0.00	0.00
0.00	0.00	0.00	0.00	0.00	23.00	0.00	0.00	0.00
0.00	0.00	0.00	0.00	0.00	0.00	0.00	0.00	0.00
0.00	0.00	0.00	0.00	0.00	0.00	0.00	0.00	0.00
0.00	0.00	0.00	0.00	0.00	0.00	0.00	0.00	0.00
0.00	0.00	0.00	0.00	0.00	0.00	0.00	0.00	0.00
0.00	0.00	0.00	0.00	0.00	0.00	0.00	0.00	0.00
0.00	0.00	0.00	0.00	0.00	0.00	0.00	0.00	0.00
0.00	0.00	0.00	0.00	0.00	0.00	0.00	0.00	0.00
0.00	0.00	0.00	0.00	0.00	0.00	0.00	0.00	0.00
0.00	7.00	0.00	0.00	0.00	0.00	0.00	0.00	0.00
0.00	0.00	0.00	0.00	0.00	0.00	0.00	0.00	0.00
0.00	0.00	0.00	0.00	0.00	0.00	4.00	0.00	0.00

0.00	0.00	0.00	0.00	0.00	0.00	0.00	0.00	0.00
0.00	0.00	0.00	0.00	0.00	0.00	0.00	0.00	0.00
0.00	0.00	0.00	0.00	0.00	0.00	0.00	0.00	0.00
0.00	0.00	0.00	0.00	0.00	0.00	0.00	0.00	0.00
0.00	0.00	0.00	0.00	0.00	5.00	0.00	0.00	0.00
0.00	0.00	0.00	0.00	0.00	0.00	0.00	0.00	0.00
0.00	0.00	0.00	0.00	0.00	0.00	0.00	0.00	0.00
0.00	0.00	0.00	0.00	0.00	0.00	0.00	0.00	0.00
0.00	0.00	0.00	0.00	0.00	0.00	0.00	0.00	0.00
0.00	0.00	0.00	0.00	0.00	0.00	0.00	21.00	0.00
0.00	0.00	0.00	0.00	0.00	0.00	0.00	0.00	0.00
0.00	0.00	0.00	0.00	0.00	0.00	0.00	0.00	0.00
0.00	0.00	0.00	0.00	0.00	0.00	0.00	0.00	0.00
4.00	0.00	2.00	0.00	0.00	5.00	3.00	4.00	0.00
0.00	0.00	0.00	0.00	0.00	0.00	0.00	0.00	0.00
0.00	0.00	0.00	0.00	0.00	0.00	0.00	0.00	0.00
0.00	0.00	0.00	0.00	0.00	0.00	0.00	0.00	0.00
0.00	0.00	0.00	0.00	0.00	0.00	0.00	0.00	0.00
0.00	0.00	0.00	0.00	0.00	0.00	0.00	0.00	0.00
3.00	0.00	0.00	0.00	0.00	0.00	0.00	0.00	0.00
0.00	0.00	0.00	0.00	0.00	0.00	0.00	0.00	0.00
0.00	0.00	0.00	0.00	0.00	0.00	0.00	0.00	0.00
0.00	0.00	0.00	0.00	0.00	0.00	0.00	0.00	0.00
0.00	0.00	0.00	0.00	0.00	0.00	0.00	0.00	0.00
18.00	0.00	0.00	0.00	0.00	0.00	0.00	0.00	0.00
0.00	0.00	0.00	0.00	0.00	0.00	0.00	0.00	0.00
0.00	0.00	0.00	0.00	0.00	0.00	0.00	0.00	0.00
0.00	0.00	0.00	0.00	0.00	0.00	0.00	0.00	0.00
0.00	0.00	0.00	0.00	0.00	0.00	0.00	0.00	0.00
0.00	0.00	0.00	0.00	0.00	0.00	0.00	0.00	0.00
0.00	0.00	0.00	0.00	0.00	0.00	0.00	0.00	0.00
0.00	0.00	0.00	0.00	0.00	0.00	0.00	0.00	0.00
0.00	0.00	0.00	0.00	0.00	0.00	0.00	0.00	0.00
0.00	0.00	0.00	0.00	0.00	0.00	0.00	0.00	0.00
0.00	0.00	0.00	0.00	0.00	0.00	0.00	0.00	0.00
0.00	0.00	0.00	0.00	0.00	0.00	0.00	0.00	0.00
0.00	0.00	0.00	0.00	0.00	0.00	0.00	0.00	0.00

15.00	0.00	0.00	0.00	0.00	0.00	0.00	0.00	0.00
0.00	0.00	0.00	0.00	0.00	0.00	0.00	0.00	0.00
0.00	0.00	0.00	22.00	0.00	0.00	0.00	0.00	0.00
0.00	0.00	0.00	0.00	0.00	0.00	0.00	0.00	0.00
0.00	0.00	0.00	0.00	0.00	9.00	13.00	0.00	0.00
0.00	0.00	0.00	0.00	0.00	0.00	0.00	0.00	0.00
0.00	0.00	0.00	0.00	0.00	0.00	0.00	0.00	0.00
0.00	0.00	0.00	0.00	0.00	0.00	0.00	0.00	0.00
0.00	0.00	0.00	0.00	0.00	0.00	23.00	0.00	0.00
0.00	0.00	0.00	0.00	0.00	0.00	0.00	0.00	0.00
0.00	0.00	0.00	0.00	0.00	0.00	0.00	0.00	0.00
0.00	0.00	0.00	0.00	0.00	0.00	0.00	0.00	0.00
0.00	0.00	0.00	0.00	0.00	0.00	0.00	0.00	0.00
0.00	0.00	0.00	0.00	0.00	0.00	0.00	0.00	0.00
0.00	0.00	0.00	0.00	0.00	0.00	0.00	0.00	0.00
0.00	0.00	0.00	0.00	0.00	0.00	0.00	0.00	0.00
0.00	0.00	0.00	0.00	0.00	0.00	5.00	0.00	0.00
0.00	0.00	0.00	0.00	0.00	0.00	0.00	0.00	0.00
0.00	0.00	0.00	0.00	0.00	75.00	0.00	0.00	0.00
0.00	0.00	0.00	0.00	0.00	0.00	0.00	0.00	0.00
0.00	0.00	0.00	0.00	0.00	0.00	0.00	0.00	0.00
0.00	0.00	0.00	0.00	0.00	0.00	0.00	0.00	0.00
4.00	0.00	0.00	0.00	0.00	0.00	0.00	0.00	0.00
0.00	0.00	0.00	0.00	0.00	0.00	0.00	0.00	0.00
0.00	0.00	0.00	0.00	0.00	0.00	0.00	0.00	0.00
0.00	0.00	0.00	0.00	0.00	0.00	0.00	74.00	0.00
0.00	0.00	0.00	0.00	0.00	0.00	0.00	0.00	0.00
0.00	0.00	0.00	0.00	0.00	0.00	0.00	0.00	0.00
0.00	0.00	0.00	0.00	0.00	7.00	13.00	0.00	0.00
0.00	0.00	0.00	0.00	0.00	0.00	0.00	0.00	0.00
0.00	0.00	3.00	0.00	0.00	0.00	0.00	0.00	0.00
0.00	0.00	0.00	0.00	0.00	0.00	0.00	0.00	0.00
20.00	0.00	8.00	0.00	0.00	0.00	0.00	0.00	0.00
0.00	0.00	0.00	0.00	0.00	0.00	0.00	0.00	0.00
7.00	0.00	13.00	12.00	0.00	0.00	0.00	0.00	0.00
0.00	0.00	0.00	0.00	0.00	0.00	0.00	3.00	0.00
0.00	0.00	0.00	0.00	0.00	0.00	0.00	0.00	0.00

0.00	0.00	0.00	0.00	0.00	0.00	0.00	0.00	0.00
0.00	0.00	0.00	0.00	0.00	0.00	0.00	0.00	0.00
0.00	0.00	0.00	0.00	0.00	0.00	0.00	0.00	0.00
0.00	0.00	0.00	0.00	0.00	0.00	14.00	0.00	0.00
0.00	0.00	0.00	0.00	0.00	0.00	0.00	0.00	0.00
0.00	0.00	0.00	0.00	0.00	0.00	0.00	0.00	0.00
0.00	0.00	0.00	0.00	0.00	0.00	0.00	0.00	0.00
0.00	0.00	0.00	0.00	0.00	8.00	0.00	0.00	0.00
0.00	0.00	0.00	0.00	0.00	0.00	0.00	0.00	0.00
0.00	0.00	0.00	0.00	0.00	0.00	0.00	0.00	43.00
0.00	0.00	0.00	0.00	0.00	0.00	0.00	0.00	0.00
0.00	6.00	0.00	0.00	0.00	0.00	0.00	0.00	0.00
0.00	0.00	0.00	0.00	0.00	0.00	0.00	0.00	0.00
0.00	0.00	0.00	0.00	0.00	0.00	0.00	0.00	0.00
0.00	0.00	0.00	0.00	0.00	0.00	0.00	0.00	0.00
0.00	0.00	0.00	0.00	0.00	0.00	0.00	0.00	0.00
0.00	0.00	0.00	0.00	0.00	0.00	0.00	0.00	0.00
0.00	0.00	0.00	0.00	0.00	0.00	0.00	0.00	0.00
0.00	0.00	0.00	0.00	0.00	0.00	0.00	0.00	0.00
9.00	0.00	0.00	0.00	0.00	4.00	0.00	0.00	0.00
0.00	0.00	0.00	0.00	0.00	0.00	0.00	0.00	0.00
0.00	0.00	0.00	0.00	0.00	0.00	0.00	0.00	0.00
0.00	0.00	0.00	0.00	0.00	0.00	0.00	0.00	67.00
0.00	0.00	0.00	0.00	0.00	0.00	0.00	0.00	0.00
0.00	0.00	0.00	0.00	0.00	0.00	0.00	0.00	0.00
0.00	0.00	0.00	0.00	0.00	0.00	6.00	0.00	0.00
8.00	5.00	8.00	10.00	0.00	0.00	0.00	0.00	0.00
0.00	0.00	0.00	0.00	0.00	0.00	0.00	0.00	0.00
0.00	0.00	0.00	0.00	0.00	0.00	0.00	0.00	0.00
0.00	0.00	0.00	0.00	0.00	0.00	0.00	0.00	0.00
0.00	0.00	0.00	0.00	0.00	0.00	0.00	0.00	0.00
0.00	0.00	0.00	0.00	0.00	0.00	5.00	0.00	0.00
0.00	0.00	0.00	0.00	0.00	0.00	0.00	0.00	0.00
0.00	0.00	0.00	0.00	0.00	0.00	0.00	0.00	0.00
0.00	0.00	0.00	0.00	0.00	0.00	0.00	0.00	0.00
0.00	0.00	0.00	0.00	0.00	0.00	0.00	0.00	0.00
0.00	0.00	0.00	0.00	0.00	0.00	0.00	0.00	0.00

0.00	0.00	4.00	0.00	0.00	0.00	0.00	0.00	0.00
0.00	0.00	0.00	0.00	0.00	0.00	0.00	0.00	0.00
0.00	0.00	0.00	0.00	0.00	0.00	0.00	0.00	0.00
0.00	0.00	0.00	0.00	0.00	0.00	0.00	0.00	0.00
0.00	0.00	0.00	0.00	0.00	0.00	0.00	0.00	0.00
0.00	0.00	0.00	0.00	0.00	0.00	0.00	0.00	0.00
0.00	0.00	0.00	0.00	0.00	0.00	0.00	0.00	0.00
0.00	0.00	0.00	0.00	0.00	0.00	0.00	0.00	0.00
0.00	0.00	0.00	0.00	0.00	0.00	0.00	0.00	0.00
21.00	0.00	8.00	4.00	0.00	0.00	0.00	0.00	0.00
0.00	0.00	0.00	0.00	0.00	0.00	0.00	0.00	0.00
0.00	0.00	0.00	0.00	0.00	63.00	0.00	0.00	0.00
0.00	0.00	0.00	0.00	5.00	0.00	0.00	0.00	0.00
0.00	0.00	0.00	0.00	0.00	0.00	0.00	0.00	0.00
0.00	0.00	0.00	0.00	0.00	0.00	0.00	0.00	0.00
0.00	0.00	9.00	0.00	0.00	0.00	0.00	0.00	0.00
0.00	0.00	0.00	0.00	0.00	0.00	0.00	0.00	0.00
0.00	0.00	0.00	0.00	0.00	0.00	0.00	0.00	0.00
0.00	0.00	7.00	0.00	0.00	0.00	0.00	0.00	0.00
0.00	21.00	11.00	0.00	0.00	0.00	0.00	0.00	0.00
0.00	0.00	0.00	0.00	0.00	0.00	0.00	0.00	0.00
0.00	0.00	0.00	0.00	0.00	0.00	0.00	0.00	0.00
0.00	0.00	0.00	0.00	0.00	0.00	0.00	0.00	0.00
0.00	0.00	0.00	0.00	0.00	0.00	0.00	0.00	0.00
0.00	0.00	0.00	0.00	0.00	0.00	0.00	0.00	0.00
0.00	0.00	0.00	0.00	0.00	0.00	0.00	0.00	0.00
0.00	0.00	0.00	0.00	0.00	0.00	0.00	0.00	0.00
0.00	0.00	5.00	0.00	0.00	0.00	10.00	0.00	0.00
0.00	9.00	0.00	0.00	0.00	8.00	7.00	6.00	0.00
0.00	0.00	0.00	0.00	0.00	0.00	0.00	0.00	0.00
0.00	0.00	0.00	0.00	0.00	0.00	0.00	0.00	0.00
0.00	0.00	0.00	0.00	0.00	0.00	0.00	0.00	0.00
0.00	0.00	0.00	0.00	0.00	0.00	0.00	0.00	0.00
0.00	0.00	0.00	0.00	0.00	0.00	0.00	0.00	0.00
0.00	0.00	0.00	0.00	0.00	0.00	0.00	0.00	0.00
0.00	0.00	0.00	0.00	0.00	0.00	0.00	0.00	0.00
0.00	0.00	0.00	0.00	0.00	0.00	0.00	0.00	0.00

0.00	0.00	0.00	0.00	0.00	0.00	0.00	0.00	0.00
0.00	0.00	0.00	0.00	0.00	0.00	0.00	0.00	0.00
12.00	0.00	0.00	0.00	0.00	0.00	0.00	0.00	0.00
0.00	0.00	0.00	0.00	0.00	0.00	0.00	0.00	0.00
0.00	0.00	0.00	0.00	0.00	0.00	0.00	0.00	0.00
0.00	0.00	0.00	0.00	0.00	0.00	0.00	0.00	0.00
0.00	0.00	0.00	0.00	0.00	0.00	0.00	0.00	0.00
0.00	0.00	0.00	0.00	0.00	0.00	14.00	0.00	0.00
0.00	0.00	0.00	0.00	0.00	0.00	0.00	0.00	0.00
0.00	0.00	0.00	0.00	0.00	0.00	0.00	0.00	0.00
0.00	0.00	0.00	0.00	0.00	0.00	0.00	0.00	0.00
0.00	0.00	0.00	0.00	0.00	0.00	0.00	0.00	0.00
0.00	0.00	0.00	0.00	0.00	0.00	0.00	58.00	0.00
0.00	0.00	0.00	0.00	0.00	0.00	0.00	0.00	0.00
0.00	0.00	0.00	0.00	0.00	0.00	0.00	0.00	0.00
26.00	0.00	0.00	0.00	0.00	0.00	0.00	0.00	0.00
0.00	0.00	0.00	0.00	0.00	0.00	0.00	0.00	0.00
0.00	0.00	0.00	0.00	0.00	0.00	0.00	0.00	0.00
0.00	0.00	0.00	0.00	0.00	0.00	0.00	0.00	0.00
0.00	0.00	0.00	0.00	0.00	0.00	0.00	0.00	0.00
0.00	0.00	0.00	0.00	0.00	0.00	0.00	0.00	0.00
0.00	0.00	0.00	0.00	0.00	0.00	0.00	0.00	0.00
0.00	0.00	0.00	0.00	0.00	0.00	0.00	0.00	0.00
0.00	0.00	0.00	0.00	0.00	0.00	0.00	0.00	0.00
0.00	0.00	0.00	0.00	0.00	0.00	0.00	0.00	0.00
0.00	0.00	0.00	0.00	0.00	0.00	0.00	0.00	0.00
0.00	0.00	0.00	0.00	0.00	0.00	0.00	0.00	0.00
0.00	0.00	0.00	0.00	0.00	0.00	4.00	0.00	0.00
0.00	0.00	0.00	0.00	0.00	0.00	0.00	0.00	0.00
0.00	0.00	0.00	0.00	0.00	0.00	0.00	0.00	0.00
0.00	0.00	0.00	0.00	0.00	0.00	0.00	0.00	0.00
0.00	0.00	0.00	0.00	0.00	0.00	0.00	0.00	0.00
0.00	0.00	0.00	0.00	0.00	0.00	0.00	0.00	0.00
0.00	0.00	0.00	0.00	0.00	0.00	0.00	0.00	0.00
0.00	0.00	0.00	0.00	0.00	0.00	0.00	0.00	0.00
0.00	0.00	0.00	0.00	0.00	0.00	0.00	0.00	0.00
0.00	0.00	0.00	0.00	0.00	0.00	0.00	0.00	0.00

0.00	0.00	0.00	0.00	0.00	0.00	0.00	0.00	0.00
0.00	0.00	0.00	0.00	0.00	0.00	0.00	0.00	0.00
0.00	0.00	0.00	0.00	0.00	0.00	0.00	0.00	0.00
0.00	0.00	0.00	0.00	0.00	0.00	0.00	0.00	0.00
0.00	0.00	0.00	0.00	0.00	0.00	0.00	0.00	0.00
0.00	0.00	0.00	0.00	0.00	0.00	0.00	0.00	0.00
0.00	0.00	0.00	0.00	0.00	0.00	0.00	0.00	0.00
0.00	0.00	0.00	0.00	0.00	0.00	0.00	0.00	0.00
0.00	0.00	0.00	0.00	0.00	0.00	0.00	0.00	0.00
0.00	0.00	0.00	0.00	0.00	0.00	0.00	0.00	0.00
0.00	0.00	6.00	0.00	0.00	10.00	0.00	0.00	0.00
0.00	0.00	0.00	0.00	0.00	0.00	0.00	0.00	0.00
0.00	0.00	0.00	0.00	0.00	0.00	0.00	0.00	0.00
0.00	0.00	0.00	0.00	0.00	0.00	0.00	0.00	0.00
0.00	0.00	0.00	0.00	0.00	0.00	0.00	0.00	0.00
0.00	0.00	0.00	0.00	0.00	0.00	8.00	4.00	0.00
0.00	0.00	0.00	0.00	0.00	0.00	52.00	0.00	0.00
0.00	0.00	0.00	0.00	0.00	0.00	0.00	0.00	0.00
0.00	0.00	0.00	0.00	0.00	0.00	0.00	0.00	0.00
0.00	0.00	0.00	0.00	0.00	0.00	0.00	0.00	0.00
0.00	0.00	0.00	0.00	0.00	0.00	0.00	0.00	0.00
0.00	0.00	0.00	0.00	0.00	0.00	0.00	0.00	0.00
0.00	0.00	0.00	0.00	0.00	0.00	51.00	0.00	0.00
0.00	0.00	0.00	0.00	0.00	0.00	6.00	0.00	0.00
0.00	0.00	0.00	0.00	0.00	0.00	0.00	0.00	0.00
0.00	0.00	0.00	0.00	0.00	0.00	0.00	0.00	0.00
0.00	0.00	0.00	0.00	0.00	0.00	0.00	0.00	0.00
0.00	0.00	0.00	0.00	0.00	0.00	0.00	0.00	0.00
0.00	0.00	0.00	0.00	0.00	0.00	0.00	0.00	0.00
0.00	0.00	0.00	0.00	0.00	0.00	0.00	0.00	0.00
0.00	0.00	0.00	0.00	0.00	0.00	0.00	0.00	0.00
0.00	0.00	0.00	0.00	0.00	0.00	0.00	0.00	0.00
0.00	0.00	0.00	0.00	0.00	0.00	0.00	0.00	0.00
0.00	0.00	0.00	0.00	0.00	0.00	0.00	0.00	0.00
6.00	0.00	0.00	0.00	0.00	0.00	0.00	0.00	0.00
0.00	0.00	0.00	0.00	0.00	0.00	0.00	0.00	0.00
0.00	0.00	0.00	0.00	0.00	0.00	0.00	0.00	0.00

0.00	0.00	0.00	0.00	0.00	0.00	0.00	0.00	0.00
0.00	49.00	0.00	0.00	0.00	0.00	0.00	0.00	0.00
0.00	0.00	0.00	0.00	0.00	0.00	0.00	0.00	0.00
0.00	0.00	0.00	0.00	0.00	0.00	0.00	0.00	0.00
0.00	0.00	0.00	0.00	5.00	0.00	0.00	0.00	0.00
0.00	0.00	0.00	0.00	0.00	0.00	0.00	0.00	0.00
0.00	0.00	0.00	0.00	0.00	0.00	0.00	0.00	0.00
0.00	0.00	0.00	0.00	0.00	0.00	0.00	0.00	0.00
0.00	0.00	0.00	0.00	0.00	0.00	0.00	0.00	0.00
0.00	0.00	0.00	0.00	0.00	0.00	0.00	0.00	0.00
0.00	0.00	0.00	0.00	0.00	0.00	0.00	0.00	0.00
0.00	0.00	0.00	0.00	0.00	0.00	0.00	0.00	0.00
0.00	0.00	0.00	0.00	0.00	0.00	0.00	0.00	0.00
0.00	0.00	0.00	0.00	0.00	0.00	0.00	0.00	0.00
0.00	0.00	0.00	0.00	0.00	0.00	9.00	0.00	0.00
0.00	6.00	0.00	3.00	0.00	0.00	0.00	0.00	0.00
0.00	0.00	0.00	0.00	0.00	0.00	0.00	0.00	0.00
0.00	0.00	0.00	0.00	0.00	0.00	0.00	0.00	0.00
0.00	0.00	0.00	0.00	0.00	0.00	0.00	0.00	0.00
0.00	0.00	0.00	0.00	0.00	0.00	0.00	0.00	0.00
0.00	0.00	0.00	0.00	0.00	0.00	0.00	47.00	0.00
0.00	0.00	0.00	0.00	0.00	0.00	0.00	0.00	0.00
0.00	0.00	0.00	0.00	0.00	0.00	0.00	0.00	0.00
0.00	0.00	0.00	0.00	0.00	0.00	0.00	0.00	0.00
0.00	0.00	0.00	0.00	0.00	0.00	0.00	0.00	0.00
4.00	0.00	0.00	3.00	0.00	0.00	0.00	0.00	0.00
0.00	0.00	0.00	0.00	0.00	0.00	0.00	0.00	0.00
0.00	0.00	0.00	0.00	0.00	0.00	0.00	0.00	0.00
0.00	0.00	0.00	0.00	0.00	0.00	0.00	0.00	0.00
0.00	0.00	0.00	0.00	0.00	0.00	0.00	0.00	0.00
0.00	0.00	0.00	0.00	0.00	0.00	0.00	0.00	0.00
0.00	0.00	0.00	0.00	0.00	0.00	0.00	0.00	0.00
0.00	0.00	0.00	0.00	0.00	0.00	0.00	0.00	0.00
0.00	0.00	0.00	0.00	0.00	0.00	0.00	0.00	0.00
0.00	0.00	0.00	0.00	0.00	0.00	0.00	0.00	0.00
0.00	0.00	0.00	0.00	0.00	0.00	0.00	0.00	0.00
0.00	0.00	0.00	0.00	0.00	0.00	0.00	0.00	0.00

0.00	0.00	0.00	0.00	0.00	0.00	0.00	0.00	0.00
0.00	0.00	0.00	0.00	0.00	0.00	0.00	0.00	0.00
26.00	0.00	0.00	0.00	0.00	0.00	0.00	0.00	0.00
0.00	0.00	0.00	0.00	0.00	0.00	0.00	0.00	0.00
0.00	0.00	0.00	0.00	0.00	0.00	0.00	0.00	0.00
0.00	0.00	0.00	0.00	0.00	0.00	0.00	0.00	0.00
0.00	0.00	0.00	0.00	0.00	0.00	0.00	0.00	0.00
0.00	0.00	0.00	0.00	0.00	0.00	0.00	0.00	0.00
6.00	0.00	0.00	0.00	0.00	0.00	2.00	2.00	0.00
2.00	0.00	0.00	0.00	0.00	0.00	0.00	0.00	0.00
0.00	0.00	0.00	0.00	0.00	0.00	0.00	0.00	0.00
0.00	0.00	0.00	0.00	0.00	0.00	0.00	0.00	0.00
0.00	0.00	0.00	0.00	0.00	0.00	0.00	0.00	0.00
0.00	0.00	0.00	0.00	0.00	0.00	0.00	0.00	0.00
0.00	0.00	0.00	0.00	0.00	0.00	0.00	0.00	0.00
0.00	0.00	0.00	0.00	0.00	0.00	0.00	0.00	0.00
0.00	0.00	0.00	0.00	0.00	0.00	0.00	0.00	0.00
0.00	0.00	0.00	0.00	0.00	0.00	0.00	0.00	0.00
0.00	0.00	0.00	0.00	0.00	0.00	0.00	10.00	0.00
0.00	0.00	0.00	0.00	0.00	0.00	0.00	0.00	0.00
0.00	0.00	0.00	0.00	0.00	0.00	0.00	0.00	0.00
0.00	0.00	0.00	0.00	0.00	0.00	0.00	0.00	0.00
0.00	0.00	0.00	0.00	0.00	0.00	0.00	0.00	0.00
0.00	0.00	0.00	0.00	0.00	0.00	0.00	0.00	0.00
0.00	0.00	0.00	0.00	0.00	0.00	0.00	0.00	0.00
0.00	0.00	0.00	0.00	0.00	32.00	0.00	0.00	0.00
0.00	0.00	0.00	0.00	0.00	4.00	0.00	0.00	0.00
0.00	0.00	0.00	0.00	0.00	0.00	0.00	0.00	0.00
0.00	0.00	0.00	0.00	0.00	0.00	0.00	0.00	0.00
0.00	0.00	0.00	0.00	0.00	0.00	0.00	0.00	0.00
0.00	0.00	0.00	0.00	0.00	0.00	0.00	0.00	0.00
0.00	0.00	0.00	0.00	0.00	0.00	0.00	0.00	0.00
0.00	0.00	0.00	0.00	0.00	0.00	0.00	0.00	0.00
0.00	0.00	0.00	0.00	0.00	0.00	0.00	0.00	0.00
0.00	0.00	0.00	0.00	0.00	0.00	0.00	0.00	0.00
0.00	0.00	0.00	0.00	0.00	0.00	0.00	0.00	0.00
0.00	0.00	0.00	0.00	0.00	0.00	0.00	0.00	0.00

0.00	0.00	0.00	0.00	0.00	41.00	0.00	0.00	0.00
0.00	0.00	0.00	0.00	0.00	0.00	0.00	0.00	0.00
0.00	0.00	0.00	0.00	0.00	0.00	8.00	0.00	2.00
0.00	0.00	0.00	0.00	0.00	0.00	0.00	0.00	0.00
0.00	0.00	0.00	0.00	0.00	0.00	0.00	0.00	0.00
0.00	0.00	0.00	0.00	0.00	0.00	0.00	0.00	0.00
0.00	0.00	0.00	0.00	0.00	0.00	0.00	0.00	0.00
0.00	0.00	0.00	0.00	0.00	0.00	0.00	0.00	0.00
0.00	0.00	0.00	0.00	0.00	0.00	0.00	0.00	0.00
0.00	0.00	0.00	0.00	0.00	0.00	0.00	40.00	0.00
0.00	0.00	0.00	0.00	0.00	0.00	0.00	0.00	0.00
0.00	0.00	0.00	0.00	0.00	0.00	0.00	0.00	0.00
0.00	0.00	0.00	0.00	0.00	0.00	0.00	0.00	0.00
0.00	0.00	0.00	0.00	0.00	0.00	0.00	0.00	0.00
0.00	0.00	0.00	0.00	0.00	0.00	0.00	0.00	0.00
0.00	0.00	0.00	0.00	0.00	0.00	0.00	0.00	0.00
0.00	0.00	0.00	0.00	0.00	0.00	0.00	0.00	0.00
0.00	0.00	0.00	0.00	0.00	0.00	0.00	0.00	0.00
0.00	0.00	0.00	0.00	0.00	0.00	0.00	0.00	0.00
0.00	0.00	0.00	0.00	0.00	0.00	0.00	0.00	0.00
0.00	0.00	0.00	0.00	0.00	0.00	0.00	0.00	0.00
7.00	0.00	0.00	0.00	0.00	9.00	0.00	0.00	0.00
0.00	0.00	0.00	0.00	0.00	0.00	0.00	0.00	0.00
7.00	4.00	0.00	0.00	0.00	0.00	0.00	0.00	0.00
0.00	0.00	0.00	0.00	0.00	0.00	0.00	0.00	0.00
0.00	0.00	0.00	0.00	0.00	0.00	0.00	0.00	0.00
0.00	0.00	0.00	0.00	0.00	0.00	0.00	0.00	0.00
0.00	0.00	0.00	0.00	0.00	0.00	0.00	0.00	0.00
0.00	0.00	0.00	0.00	0.00	0.00	0.00	0.00	0.00
24.00	0.00	0.00	0.00	0.00	0.00	0.00	0.00	0.00
0.00	0.00	0.00	0.00	0.00	3.00	3.00	0.00	0.00
0.00	0.00	0.00	0.00	0.00	0.00	0.00	0.00	0.00
0.00	0.00	0.00	0.00	0.00	0.00	0.00	0.00	0.00
0.00	0.00	0.00	0.00	0.00	0.00	0.00	38.00	0.00
0.00	0.00	0.00	0.00	0.00	0.00	0.00	0.00	0.00
0.00	0.00	0.00	0.00	0.00	0.00	0.00	0.00	0.00
0.00	0.00	0.00	0.00	0.00	0.00	0.00	0.00	0.00

0.00	0.00	0.00	0.00	0.00	0.00	0.00	0.00	0.00
0.00	0.00	0.00	0.00	0.00	0.00	0.00	0.00	0.00
0.00	0.00	6.00	3.00	0.00	0.00	0.00	0.00	0.00
0.00	0.00	0.00	0.00	0.00	0.00	11.00	0.00	0.00
0.00	0.00	0.00	0.00	0.00	0.00	0.00	0.00	0.00
0.00	0.00	0.00	0.00	0.00	0.00	0.00	0.00	0.00
0.00	0.00	0.00	0.00	37.00	0.00	0.00	0.00	0.00
0.00	0.00	2.00	0.00	0.00	0.00	0.00	0.00	0.00
0.00	0.00	0.00	0.00	0.00	0.00	0.00	0.00	0.00
0.00	0.00	0.00	0.00	0.00	0.00	0.00	0.00	0.00
0.00	0.00	0.00	0.00	0.00	0.00	8.00	0.00	0.00
0.00	0.00	0.00	0.00	0.00	0.00	0.00	0.00	0.00
0.00	0.00	0.00	0.00	0.00	0.00	0.00	0.00	0.00
0.00	0.00	0.00	0.00	0.00	0.00	0.00	0.00	0.00
0.00	0.00	0.00	0.00	0.00	0.00	0.00	0.00	0.00
0.00	0.00	0.00	0.00	0.00	0.00	0.00	0.00	0.00
0.00	0.00	0.00	0.00	0.00	0.00	0.00	0.00	0.00
0.00	0.00	0.00	0.00	0.00	0.00	0.00	0.00	0.00
0.00	0.00	0.00	0.00	0.00	0.00	0.00	0.00	0.00
0.00	0.00	0.00	0.00	0.00	0.00	0.00	0.00	0.00
0.00	0.00	0.00	0.00	0.00	0.00	0.00	0.00	0.00
0.00	0.00	0.00	0.00	0.00	0.00	0.00	0.00	0.00
0.00	0.00	0.00	0.00	0.00	0.00	0.00	0.00	0.00
0.00	0.00	0.00	0.00	0.00	0.00	0.00	0.00	0.00
0.00	0.00	0.00	0.00	0.00	0.00	0.00	0.00	0.00
0.00	0.00	0.00	0.00	0.00	0.00	0.00	0.00	0.00
0.00	0.00	0.00	0.00	0.00	0.00	0.00	0.00	0.00
0.00	0.00	0.00	0.00	0.00	0.00	0.00	0.00	0.00
0.00	0.00	0.00	0.00	0.00	0.00	0.00	0.00	0.00
0.00	0.00	0.00	0.00	0.00	0.00	0.00	0.00	0.00
0.00	0.00	0.00	0.00	0.00	0.00	0.00	0.00	0.00
0.00	0.00	0.00	0.00	0.00	0.00	0.00	0.00	0.00
0.00	0.00	0.00	0.00	0.00	0.00	0.00	0.00	0.00
4.00	0.00	0.00	0.00	0.00	0.00	0.00	0.00	3.00
0.00	0.00	0.00	0.00	0.00	0.00	0.00	0.00	0.00
0.00	0.00	0.00	0.00	0.00	0.00	6.00	0.00	0.00
0.00	0.00	0.00	0.00	0.00	0.00	0.00	0.00	0.00

0.00	0.00	0.00	0.00	0.00	0.00	0.00	0.00	0.00
0.00	0.00	0.00	0.00	0.00	0.00	0.00	0.00	0.00
0.00	0.00	0.00	0.00	0.00	0.00	0.00	0.00	0.00
0.00	0.00	0.00	0.00	0.00	0.00	0.00	0.00	0.00
0.00	0.00	0.00	0.00	0.00	0.00	0.00	0.00	0.00
0.00	33.00	0.00	0.00	0.00	0.00	0.00	0.00	0.00
0.00	0.00	0.00	0.00	0.00	0.00	0.00	0.00	0.00
0.00	0.00	0.00	0.00	0.00	0.00	0.00	0.00	0.00
0.00	0.00	0.00	0.00	0.00	0.00	0.00	0.00	0.00
0.00	0.00	0.00	0.00	0.00	0.00	0.00	0.00	0.00
0.00	0.00	0.00	0.00	0.00	0.00	0.00	0.00	0.00
0.00	0.00	0.00	0.00	0.00	0.00	0.00	0.00	0.00
6.00	0.00	0.00	0.00	0.00	0.00	0.00	0.00	0.00
0.00	0.00	0.00	4.00	0.00	0.00	0.00	0.00	0.00
0.00	0.00	0.00	0.00	0.00	0.00	0.00	0.00	0.00
0.00	0.00	0.00	0.00	0.00	0.00	0.00	0.00	0.00
0.00	0.00	0.00	0.00	0.00	0.00	0.00	0.00	0.00
0.00	0.00	0.00	0.00	0.00	0.00	32.00	0.00	0.00
0.00	0.00	0.00	0.00	0.00	0.00	0.00	0.00	0.00
0.00	0.00	0.00	0.00	0.00	0.00	0.00	0.00	0.00
0.00	0.00	0.00	0.00	0.00	0.00	0.00	0.00	0.00
0.00	0.00	0.00	0.00	0.00	0.00	0.00	0.00	0.00
0.00	0.00	0.00	0.00	0.00	0.00	0.00	0.00	0.00
0.00	0.00	0.00	0.00	0.00	0.00	0.00	0.00	0.00
0.00	0.00	0.00	0.00	0.00	0.00	0.00	0.00	0.00
0.00	0.00	0.00	0.00	0.00	0.00	0.00	0.00	0.00
0.00	0.00	0.00	0.00	0.00	0.00	0.00	0.00	0.00
0.00	0.00	6.00	0.00	0.00	0.00	0.00	0.00	0.00
0.00	0.00	0.00	0.00	0.00	0.00	0.00	0.00	0.00
0.00	0.00	0.00	0.00	0.00	0.00	0.00	0.00	0.00
0.00	0.00	0.00	0.00	0.00	0.00	0.00	0.00	0.00
0.00	0.00	0.00	0.00	0.00	0.00	0.00	0.00	0.00
0.00	0.00	0.00	0.00	0.00	0.00	0.00	0.00	0.00
0.00	0.00	0.00	0.00	0.00	0.00	0.00	0.00	0.00
0.00	0.00	0.00	0.00	0.00	0.00	0.00	0.00	0.00
0.00	0.00	0.00	0.00	0.00	0.00	0.00	0.00	0.00
0.00	0.00	0.00	0.00	0.00	0.00	0.00	0.00	0.00

0.00	0.00	0.00	0.00	0.00	0.00	0.00	0.00	0.00
0.00	0.00	0.00	0.00	0.00	0.00	0.00	0.00	0.00
30.00	0.00	0.00	0.00	0.00	0.00	0.00	0.00	0.00
8.00	0.00	0.00	0.00	0.00	0.00	0.00	0.00	0.00
0.00	0.00	0.00	0.00	0.00	0.00	0.00	0.00	0.00
0.00	0.00	0.00	0.00	0.00	0.00	8.00	0.00	0.00
0.00	0.00	0.00	0.00	0.00	0.00	0.00	0.00	0.00
0.00	0.00	0.00	0.00	0.00	0.00	0.00	0.00	0.00
0.00	0.00	0.00	0.00	0.00	0.00	0.00	0.00	0.00
0.00	0.00	0.00	0.00	0.00	0.00	0.00	0.00	0.00
0.00	0.00	0.00	0.00	0.00	0.00	0.00	0.00	0.00
0.00	0.00	0.00	0.00	0.00	30.00	0.00	0.00	0.00
0.00	0.00	0.00	0.00	0.00	0.00	0.00	0.00	0.00
0.00	0.00	0.00	0.00	0.00	0.00	0.00	0.00	0.00
0.00	0.00	0.00	0.00	0.00	0.00	0.00	0.00	0.00
0.00	0.00	0.00	0.00	0.00	0.00	0.00	0.00	0.00
0.00	0.00	0.00	0.00	0.00	0.00	0.00	0.00	0.00
0.00	0.00	0.00	0.00	0.00	0.00	0.00	0.00	0.00
0.00	0.00	0.00	0.00	0.00	0.00	0.00	0.00	0.00
2.00	0.00	4.00	0.00	0.00	0.00	0.00	0.00	0.00
3.00	0.00	0.00	0.00	0.00	0.00	0.00	0.00	0.00
0.00	0.00	0.00	0.00	0.00	0.00	6.00	0.00	0.00
0.00	0.00	0.00	0.00	9.00	0.00	0.00	0.00	0.00
0.00	0.00	0.00	0.00	0.00	0.00	0.00	0.00	0.00
0.00	0.00	0.00	0.00	0.00	0.00	0.00	0.00	0.00
0.00	0.00	0.00	0.00	0.00	0.00	0.00	0.00	0.00
6.00	0.00	0.00	6.00	0.00	0.00	0.00	0.00	0.00
0.00	0.00	0.00	0.00	0.00	0.00	0.00	0.00	0.00
0.00	0.00	0.00	0.00	0.00	0.00	0.00	0.00	0.00
9.00	0.00	4.00	0.00	0.00	0.00	0.00	0.00	0.00
0.00	0.00	0.00	0.00	0.00	0.00	0.00	0.00	0.00
0.00	0.00	0.00	0.00	0.00	28.00	0.00	0.00	0.00
0.00	0.00	0.00	0.00	0.00	0.00	0.00	0.00	0.00
0.00	0.00	0.00	0.00	0.00	0.00	0.00	0.00	0.00
0.00	0.00	0.00	0.00	0.00	0.00	0.00	0.00	0.00
0.00	0.00	0.00	0.00	0.00	0.00	0.00	0.00	0.00
0.00	0.00	0.00	0.00	0.00	0.00	0.00	0.00	0.00

0.00	0.00	0.00	0.00	0.00	0.00	0.00	0.00	0.00
0.00	0.00	0.00	0.00	0.00	0.00	0.00	0.00	0.00
0.00	0.00	0.00	0.00	0.00	0.00	0.00	0.00	0.00
0.00	0.00	0.00	0.00	0.00	0.00	0.00	0.00	0.00
0.00	0.00	0.00	0.00	0.00	0.00	0.00	0.00	0.00
0.00	0.00	0.00	0.00	0.00	0.00	0.00	0.00	0.00
0.00	0.00	0.00	0.00	0.00	0.00	0.00	0.00	0.00
0.00	0.00	0.00	0.00	0.00	0.00	0.00	0.00	0.00
8.00	0.00	0.00	0.00	0.00	0.00	0.00	0.00	0.00
0.00	0.00	0.00	0.00	0.00	0.00	0.00	0.00	0.00
0.00	0.00	0.00	0.00	0.00	0.00	0.00	0.00	0.00
0.00	0.00	0.00	0.00	0.00	0.00	0.00	0.00	0.00
0.00	0.00	0.00	0.00	0.00	0.00	0.00	0.00	0.00
0.00	0.00	0.00	0.00	0.00	0.00	8.00	0.00	0.00
0.00	0.00	0.00	0.00	0.00	0.00	0.00	0.00	0.00
0.00	0.00	0.00	0.00	0.00	0.00	0.00	0.00	0.00
0.00	0.00	0.00	0.00	0.00	0.00	0.00	0.00	0.00
0.00	0.00	0.00	0.00	0.00	0.00	0.00	0.00	0.00
0.00	0.00	0.00	0.00	0.00	0.00	10.00	0.00	0.00
0.00	0.00	0.00	0.00	0.00	0.00	0.00	27.00	0.00
0.00	27.00	0.00	0.00	0.00	0.00	0.00	0.00	0.00
0.00	0.00	0.00	0.00	0.00	0.00	0.00	0.00	0.00
0.00	0.00	0.00	0.00	0.00	0.00	0.00	0.00	0.00
0.00	0.00	0.00	0.00	0.00	0.00	0.00	0.00	0.00
0.00	0.00	0.00	0.00	0.00	0.00	0.00	0.00	0.00
0.00	0.00	0.00	0.00	0.00	0.00	0.00	0.00	0.00
0.00	0.00	0.00	0.00	0.00	0.00	0.00	0.00	0.00
0.00	0.00	0.00	0.00	0.00	0.00	0.00	0.00	0.00
0.00	0.00	0.00	0.00	0.00	0.00	0.00	0.00	0.00
0.00	0.00	0.00	0.00	0.00	0.00	0.00	0.00	0.00
0.00	0.00	0.00	0.00	4.00	4.00	0.00	0.00	0.00
0.00	0.00	0.00	0.00	0.00	0.00	0.00	0.00	0.00
0.00	0.00	0.00	0.00	0.00	0.00	0.00	0.00	0.00
0.00	0.00	0.00	0.00	0.00	0.00	0.00	0.00	0.00
0.00	0.00	0.00	0.00	0.00	0.00	0.00	0.00	0.00
0.00	0.00	0.00	0.00	0.00	0.00	0.00	0.00	0.00
0.00	0.00	0.00	0.00	0.00	0.00	0.00	0.00	0.00

0.00	0.00	0.00	0.00	0.00	0.00	0.00	0.00	0.00
0.00	0.00	0.00	0.00	0.00	0.00	0.00	0.00	0.00
0.00	0.00	0.00	0.00	0.00	0.00	0.00	0.00	0.00
0.00	0.00	8.00	0.00	0.00	0.00	0.00	2.00	0.00
6.00	3.00	0.00	4.00	0.00	0.00	0.00	0.00	0.00
0.00	0.00	0.00	0.00	0.00	0.00	0.00	0.00	0.00
0.00	0.00	0.00	0.00	0.00	0.00	0.00	0.00	0.00
0.00	0.00	0.00	0.00	0.00	0.00	0.00	0.00	0.00
0.00	0.00	0.00	0.00	0.00	0.00	0.00	0.00	0.00
0.00	0.00	0.00	0.00	0.00	0.00	0.00	0.00	0.00
0.00	0.00	0.00	0.00	0.00	0.00	0.00	0.00	0.00
0.00	0.00	0.00	0.00	0.00	0.00	0.00	0.00	0.00
0.00	0.00	0.00	0.00	0.00	0.00	0.00	0.00	0.00
0.00	0.00	0.00	0.00	0.00	0.00	0.00	0.00	0.00
0.00	0.00	0.00	0.00	0.00	0.00	0.00	0.00	0.00
0.00	0.00	0.00	0.00	0.00	0.00	0.00	0.00	0.00
0.00	0.00	0.00	0.00	0.00	0.00	0.00	0.00	0.00
0.00	0.00	0.00	0.00	0.00	0.00	0.00	0.00	0.00
0.00	0.00	0.00	0.00	0.00	0.00	0.00	0.00	0.00
0.00	0.00	0.00	0.00	0.00	0.00	0.00	0.00	0.00
0.00	0.00	0.00	0.00	0.00	0.00	0.00	0.00	0.00
3.00	0.00	0.00	0.00	0.00	0.00	0.00	0.00	0.00
0.00	0.00	0.00	0.00	0.00	0.00	0.00	0.00	0.00
0.00	0.00	0.00	0.00	0.00	0.00	0.00	0.00	0.00
0.00	0.00	0.00	3.00	0.00	0.00	0.00	0.00	0.00
0.00	0.00	0.00	0.00	0.00	0.00	0.00	0.00	0.00
0.00	0.00	0.00	24.00	0.00	0.00	0.00	0.00	0.00
0.00	0.00	0.00	0.00	0.00	0.00	0.00	0.00	0.00
0.00	0.00	0.00	0.00	0.00	0.00	0.00	0.00	0.00
0.00	0.00	0.00	0.00	0.00	0.00	0.00	0.00	0.00
0.00	0.00	0.00	0.00	0.00	0.00	0.00	0.00	0.00
0.00	0.00	0.00	0.00	0.00	0.00	0.00	0.00	0.00
0.00	0.00	0.00	0.00	0.00	0.00	0.00	0.00	0.00
0.00	0.00	0.00	0.00	0.00	0.00	0.00	0.00	0.00
0.00	0.00	0.00	0.00	0.00	0.00	0.00	0.00	0.00
0.00	0.00	0.00	0.00	0.00	4.00	0.00	0.00	0.00
0.00	0.00	0.00	0.00	0.00	0.00	0.00	0.00	0.00

0.00	0.00	0.00	0.00	0.00	0.00	0.00	0.00	0.00
0.00	0.00	0.00	0.00	0.00	0.00	0.00	0.00	0.00
0.00	0.00	0.00	0.00	0.00	0.00	0.00	0.00	0.00
0.00	0.00	0.00	0.00	0.00	23.00	0.00	0.00	0.00
0.00	0.00	0.00	0.00	23.00	0.00	0.00	0.00	0.00
0.00	0.00	0.00	0.00	0.00	0.00	0.00	0.00	0.00
0.00	0.00	0.00	0.00	0.00	0.00	0.00	0.00	0.00
0.00	0.00	0.00	0.00	0.00	0.00	0.00	0.00	0.00
0.00	0.00	0.00	0.00	0.00	0.00	0.00	0.00	0.00
0.00	0.00	0.00	0.00	0.00	0.00	0.00	0.00	0.00
0.00	0.00	0.00	0.00	0.00	0.00	0.00	0.00	0.00
0.00	0.00	0.00	0.00	0.00	0.00	0.00	0.00	0.00
5.00	0.00	0.00	0.00	0.00	0.00	0.00	0.00	0.00
5.00	0.00	0.00	0.00	0.00	0.00	4.00	0.00	0.00
0.00	0.00	0.00	0.00	0.00	0.00	0.00	0.00	0.00
0.00	0.00	0.00	0.00	0.00	0.00	0.00	0.00	0.00
0.00	0.00	0.00	0.00	0.00	0.00	0.00	0.00	0.00
0.00	0.00	0.00	0.00	0.00	0.00	4.00	0.00	0.00
0.00	0.00	0.00	0.00	0.00	0.00	5.00	0.00	0.00
0.00	0.00	0.00	0.00	0.00	0.00	0.00	0.00	0.00
0.00	0.00	0.00	0.00	0.00	0.00	0.00	0.00	0.00
0.00	0.00	0.00	0.00	0.00	0.00	0.00	0.00	0.00
0.00	0.00	0.00	0.00	0.00	0.00	0.00	0.00	0.00
0.00	0.00	0.00	0.00	0.00	0.00	0.00	0.00	0.00
0.00	0.00	0.00	0.00	0.00	0.00	0.00	0.00	3.00
0.00	0.00	0.00	0.00	0.00	0.00	22.00	0.00	0.00
0.00	0.00	0.00	0.00	0.00	0.00	0.00	0.00	0.00
0.00	0.00	0.00	0.00	0.00	0.00	0.00	0.00	0.00
0.00	0.00	0.00	0.00	0.00	0.00	0.00	0.00	0.00
0.00	0.00	0.00	0.00	0.00	0.00	0.00	0.00	0.00
0.00	0.00	0.00	0.00	0.00	0.00	0.00	0.00	0.00
0.00	0.00	0.00	0.00	0.00	0.00	0.00	0.00	0.00
0.00	0.00	0.00	0.00	0.00	0.00	0.00	0.00	0.00
0.00	0.00	0.00	0.00	0.00	0.00	0.00	0.00	0.00
0.00	0.00	0.00	0.00	0.00	0.00	0.00	0.00	0.00
0.00	0.00	0.00	0.00	0.00	0.00	0.00	0.00	0.00
0.00	0.00	0.00	0.00	0.00	0.00	0.00	0.00	0.00

0.00	0.00	0.00	0.00	0.00	0.00	0.00	0.00	0.00
0.00	0.00	0.00	0.00	0.00	0.00	0.00	7.00	0.00
0.00	0.00	0.00	0.00	0.00	0.00	0.00	0.00	0.00
0.00	0.00	0.00	0.00	0.00	0.00	0.00	0.00	0.00
0.00	0.00	0.00	0.00	0.00	0.00	0.00	0.00	0.00
0.00	0.00	0.00	0.00	0.00	0.00	0.00	0.00	0.00
0.00	0.00	0.00	0.00	0.00	0.00	0.00	0.00	0.00
0.00	0.00	0.00	0.00	0.00	21.00	0.00	0.00	0.00
0.00	0.00	0.00	0.00	0.00	10.00	0.00	0.00	0.00
0.00	0.00	0.00	0.00	0.00	0.00	0.00	0.00	0.00
0.00	0.00	0.00	0.00	0.00	0.00	21.00	0.00	0.00
0.00	0.00	0.00	0.00	0.00	0.00	0.00	0.00	0.00
7.00	0.00	0.00	6.00	0.00	0.00	0.00	0.00	0.00
0.00	0.00	0.00	0.00	0.00	0.00	0.00	0.00	0.00
0.00	0.00	0.00	0.00	0.00	0.00	0.00	0.00	0.00
0.00	0.00	0.00	0.00	0.00	0.00	0.00	0.00	0.00
0.00	0.00	0.00	0.00	0.00	0.00	0.00	0.00	0.00
0.00	0.00	0.00	0.00	0.00	0.00	0.00	0.00	0.00
0.00	0.00	0.00	0.00	0.00	0.00	0.00	0.00	0.00
0.00	0.00	0.00	0.00	0.00	0.00	0.00	0.00	0.00
0.00	0.00	0.00	0.00	0.00	0.00	0.00	0.00	0.00
0.00	0.00	0.00	0.00	0.00	0.00	0.00	0.00	0.00
0.00	0.00	0.00	0.00	0.00	0.00	0.00	0.00	0.00
0.00	0.00	0.00	0.00	0.00	0.00	0.00	0.00	0.00
0.00	0.00	0.00	0.00	0.00	0.00	0.00	0.00	0.00
0.00	0.00	0.00	0.00	0.00	0.00	0.00	0.00	0.00
0.00	0.00	0.00	0.00	0.00	20.00	0.00	0.00	0.00
0.00	0.00	0.00	20.00	0.00	0.00	0.00	0.00	0.00
0.00	0.00	0.00	0.00	0.00	0.00	0.00	0.00	0.00
0.00	0.00	0.00	0.00	0.00	0.00	6.00	0.00	6.00
0.00	0.00	0.00	0.00	0.00	0.00	0.00	0.00	0.00
0.00	0.00	0.00	0.00	0.00	0.00	0.00	0.00	0.00
0.00	0.00	0.00	0.00	0.00	0.00	0.00	0.00	0.00
0.00	0.00	0.00	0.00	0.00	0.00	0.00	0.00	0.00
0.00	0.00	0.00	0.00	0.00	0.00	0.00	0.00	0.00
0.00	0.00	0.00	0.00	0.00	0.00	0.00	0.00	0.00
0.00	0.00	0.00	0.00	0.00	0.00	0.00	0.00	0.00

0.00	0.00	0.00	0.00	0.00	0.00	0.00	0.00	0.00
0.00	0.00	0.00	0.00	0.00	0.00	0.00	0.00	0.00
0.00	0.00	0.00	0.00	0.00	0.00	0.00	0.00	0.00
0.00	0.00	0.00	0.00	0.00	0.00	0.00	0.00	0.00
0.00	0.00	0.00	0.00	0.00	0.00	0.00	0.00	0.00
0.00	0.00	0.00	0.00	0.00	0.00	0.00	0.00	0.00
0.00	0.00	0.00	0.00	0.00	0.00	0.00	0.00	0.00
0.00	0.00	0.00	0.00	0.00	0.00	0.00	0.00	0.00
0.00	0.00	0.00	0.00	0.00	3.00	0.00	0.00	0.00
0.00	0.00	0.00	0.00	0.00	0.00	0.00	0.00	0.00
0.00	0.00	0.00	0.00	0.00	0.00	0.00	0.00	0.00
0.00	0.00	0.00	0.00	0.00	0.00	0.00	0.00	0.00
0.00	0.00	0.00	0.00	0.00	0.00	13.00	0.00	0.00
0.00	0.00	0.00	0.00	0.00	0.00	0.00	0.00	0.00
0.00	0.00	0.00	0.00	0.00	0.00	0.00	0.00	0.00
0.00	0.00	0.00	0.00	0.00	0.00	0.00	0.00	0.00
0.00	0.00	0.00	4.00	0.00	0.00	0.00	0.00	0.00
0.00	0.00	0.00	0.00	0.00	0.00	0.00	0.00	0.00
0.00	0.00	0.00	0.00	0.00	0.00	19.00	0.00	0.00
0.00	0.00	0.00	0.00	0.00	0.00	0.00	0.00	0.00
0.00	0.00	0.00	0.00	0.00	0.00	0.00	0.00	0.00
0.00	0.00	0.00	0.00	0.00	0.00	0.00	0.00	0.00
0.00	0.00	0.00	0.00	0.00	0.00	0.00	0.00	0.00
0.00	0.00	0.00	0.00	0.00	0.00	0.00	0.00	0.00
0.00	0.00	0.00	0.00	0.00	0.00	0.00	0.00	0.00
0.00	0.00	0.00	0.00	0.00	0.00	0.00	0.00	0.00
0.00	0.00	0.00	0.00	0.00	0.00	0.00	0.00	0.00
0.00	0.00	0.00	0.00	0.00	0.00	0.00	0.00	0.00
0.00	0.00	0.00	0.00	0.00	0.00	0.00	0.00	0.00
0.00	0.00	0.00	0.00	0.00	0.00	0.00	0.00	0.00
0.00	0.00	0.00	0.00	0.00	0.00	0.00	0.00	0.00
0.00	0.00	0.00	0.00	0.00	0.00	0.00	0.00	0.00
0.00	0.00	0.00	0.00	0.00	0.00	0.00	0.00	0.00
0.00	0.00	0.00	0.00	0.00	0.00	0.00	0.00	0.00
0.00	0.00	0.00	0.00	0.00	0.00	0.00	0.00	0.00
0.00	0.00	0.00	0.00	0.00	0.00	0.00	0.00	0.00
6.00	0.00	0.00	0.00	0.00	0.00	0.00	0.00	0.00

0.00	0.00	0.00	0.00	0.00	0.00	0.00	0.00	0.00
0.00	0.00	0.00	0.00	0.00	5.00	0.00	0.00	0.00
0.00	0.00	0.00	6.00	0.00	0.00	0.00	0.00	0.00
0.00	0.00	0.00	0.00	0.00	0.00	0.00	0.00	0.00
0.00	0.00	0.00	0.00	0.00	0.00	0.00	0.00	0.00
0.00	3.00	0.00	0.00	0.00	0.00	0.00	0.00	0.00
0.00	0.00	0.00	0.00	0.00	0.00	0.00	0.00	0.00
0.00	0.00	0.00	0.00	0.00	0.00	0.00	0.00	0.00
0.00	0.00	0.00	0.00	0.00	0.00	0.00	0.00	0.00
0.00	0.00	0.00	0.00	0.00	0.00	0.00	0.00	0.00
0.00	0.00	0.00	0.00	0.00	0.00	0.00	0.00	0.00
0.00	0.00	0.00	0.00	0.00	0.00	0.00	0.00	0.00
0.00	0.00	0.00	0.00	0.00	0.00	0.00	0.00	0.00
0.00	0.00	0.00	0.00	0.00	0.00	0.00	0.00	0.00
0.00	0.00	0.00	0.00	0.00	0.00	0.00	0.00	0.00
0.00	0.00	0.00	0.00	0.00	0.00	0.00	0.00	0.00
0.00	0.00	0.00	0.00	0.00	0.00	0.00	0.00	0.00
0.00	0.00	0.00	0.00	0.00	0.00	0.00	0.00	0.00
0.00	0.00	0.00	0.00	0.00	0.00	0.00	0.00	0.00
0.00	0.00	0.00	0.00	0.00	0.00	0.00	0.00	0.00
0.00	0.00	0.00	0.00	0.00	0.00	0.00	0.00	0.00
0.00	0.00	0.00	0.00	0.00	0.00	0.00	0.00	0.00
0.00	0.00	0.00	0.00	0.00	0.00	0.00	0.00	0.00
0.00	0.00	0.00	0.00	0.00	0.00	0.00	0.00	0.00
0.00	0.00	0.00	0.00	0.00	0.00	0.00	0.00	0.00
17.00	0.00	0.00	0.00	0.00	0.00	0.00	0.00	0.00
0.00	0.00	0.00	0.00	0.00	0.00	0.00	0.00	0.00
0.00	0.00	0.00	0.00	0.00	4.00	0.00	0.00	0.00
0.00	0.00	0.00	0.00	0.00	0.00	0.00	0.00	0.00
0.00	0.00	0.00	0.00	0.00	0.00	0.00	0.00	0.00
0.00	0.00	0.00	0.00	0.00	0.00	0.00	0.00	0.00
0.00	0.00	0.00	0.00	0.00	0.00	6.00	0.00	0.00
0.00	0.00	0.00	0.00	0.00	0.00	0.00	0.00	0.00
0.00	0.00	0.00	0.00	0.00	0.00	0.00	0.00	0.00
0.00	0.00	0.00	0.00	0.00	0.00	0.00	0.00	0.00
0.00	0.00	0.00	0.00	0.00	0.00	0.00	0.00	0.00
0.00	0.00	0.00	0.00	0.00	0.00	0.00	11.00	0.00

0.00	0.00	0.00	0.00	0.00	0.00	0.00	0.00	0.00
0.00	0.00	0.00	0.00	0.00	0.00	0.00	0.00	0.00
0.00	0.00	0.00	0.00	0.00	0.00	0.00	0.00	0.00
0.00	0.00	0.00	0.00	0.00	0.00	0.00	0.00	0.00
0.00	0.00	0.00	0.00	0.00	0.00	0.00	0.00	0.00
0.00	0.00	0.00	0.00	0.00	0.00	0.00	0.00	0.00
0.00	0.00	0.00	0.00	0.00	0.00	0.00	0.00	0.00
0.00	0.00	0.00	0.00	0.00	0.00	0.00	0.00	0.00
0.00	0.00	0.00	0.00	0.00	0.00	0.00	0.00	0.00
0.00	0.00	0.00	0.00	0.00	0.00	0.00	0.00	0.00
0.00	0.00	0.00	0.00	0.00	0.00	0.00	0.00	0.00
0.00	0.00	0.00	0.00	0.00	0.00	0.00	0.00	0.00
0.00	0.00	0.00	0.00	0.00	0.00	0.00	0.00	0.00
0.00	0.00	0.00	0.00	0.00	0.00	0.00	0.00	0.00
0.00	0.00	0.00	0.00	0.00	0.00	0.00	0.00	0.00
0.00	0.00	0.00	0.00	0.00	0.00	0.00	0.00	0.00
0.00	0.00	0.00	0.00	0.00	0.00	0.00	0.00	0.00
0.00	0.00	0.00	0.00	0.00	0.00	0.00	0.00	0.00
0.00	0.00	0.00	0.00	0.00	0.00	0.00	0.00	0.00
0.00	0.00	0.00	0.00	0.00	0.00	0.00	0.00	0.00
0.00	0.00	0.00	0.00	0.00	0.00	0.00	0.00	0.00
0.00	0.00	0.00	0.00	0.00	0.00	0.00	0.00	0.00
6.00	0.00	3.00	0.00	0.00	0.00	0.00	0.00	0.00
6.00	0.00	0.00	0.00	0.00	0.00	0.00	0.00	0.00
0.00	0.00	0.00	0.00	0.00	0.00	0.00	0.00	0.00
0.00	0.00	0.00	0.00	0.00	0.00	0.00	0.00	0.00
0.00	0.00	0.00	0.00	0.00	0.00	0.00	0.00	0.00
0.00	0.00	0.00	0.00	0.00	0.00	0.00	0.00	0.00
0.00	0.00	0.00	0.00	0.00	0.00	0.00	0.00	0.00
0.00	0.00	0.00	0.00	0.00	0.00	0.00	3.00	0.00
0.00	0.00	0.00	0.00	0.00	0.00	0.00	0.00	0.00
0.00	0.00	0.00	0.00	0.00	0.00	0.00	0.00	0.00
0.00	0.00	0.00	0.00	0.00	0.00	0.00	0.00	0.00
0.00	0.00	0.00	0.00	0.00	0.00	0.00	0.00	0.00
0.00	0.00	0.00	0.00	0.00	0.00	0.00	0.00	0.00
0.00	0.00	0.00	0.00	0.00	0.00	8.00	0.00	0.00
0.00	0.00	0.00	0.00	0.00	0.00	0.00	0.00	0.00

0.00	0.00	0.00	0.00	0.00	0.00	0.00	0.00	0.00
0.00	0.00	0.00	0.00	0.00	0.00	0.00	0.00	0.00
0.00	0.00	0.00	0.00	0.00	0.00	0.00	0.00	0.00
0.00	0.00	0.00	0.00	0.00	0.00	16.00	0.00	0.00
0.00	0.00	0.00	0.00	0.00	0.00	0.00	0.00	0.00
0.00	0.00	0.00	0.00	0.00	0.00	0.00	0.00	0.00
0.00	0.00	0.00	0.00	0.00	0.00	0.00	0.00	0.00
0.00	0.00	0.00	0.00	0.00	0.00	0.00	0.00	0.00
0.00	0.00	0.00	0.00	0.00	0.00	0.00	0.00	0.00
0.00	0.00	0.00	0.00	0.00	0.00	0.00	0.00	0.00
0.00	0.00	0.00	0.00	0.00	0.00	0.00	0.00	0.00
0.00	0.00	0.00	0.00	0.00	0.00	0.00	0.00	0.00
0.00	0.00	0.00	0.00	0.00	0.00	4.00	0.00	0.00
0.00	0.00	0.00	0.00	0.00	0.00	0.00	0.00	0.00
0.00	0.00	0.00	0.00	0.00	0.00	0.00	0.00	0.00
0.00	0.00	0.00	0.00	0.00	0.00	0.00	0.00	0.00
0.00	0.00	0.00	0.00	0.00	0.00	0.00	0.00	0.00
0.00	0.00	0.00	0.00	0.00	0.00	0.00	0.00	0.00
0.00	0.00	0.00	0.00	0.00	0.00	0.00	0.00	0.00
0.00	0.00	0.00	0.00	0.00	0.00	0.00	0.00	0.00
0.00	0.00	0.00	0.00	0.00	0.00	0.00	0.00	0.00
0.00	0.00	0.00	0.00	0.00	0.00	0.00	0.00	0.00
0.00	0.00	0.00	0.00	0.00	0.00	0.00	0.00	0.00
0.00	0.00	0.00	0.00	0.00	0.00	0.00	0.00	0.00
0.00	0.00	0.00	0.00	0.00	0.00	0.00	0.00	0.00
0.00	0.00	0.00	0.00	0.00	0.00	0.00	0.00	0.00
0.00	0.00	0.00	2.00	0.00	0.00	0.00	0.00	0.00
0.00	0.00	0.00	0.00	0.00	0.00	0.00	0.00	0.00
0.00	0.00	0.00	0.00	0.00	0.00	0.00	0.00	0.00
0.00	0.00	0.00	0.00	0.00	0.00	9.00	0.00	0.00
0.00	0.00	0.00	0.00	0.00	0.00	0.00	0.00	0.00
0.00	0.00	0.00	0.00	0.00	0.00	0.00	0.00	0.00
0.00	0.00	0.00	0.00	0.00	0.00	0.00	0.00	0.00
0.00	0.00	0.00	0.00	0.00	0.00	0.00	0.00	0.00
0.00	0.00	0.00	0.00	0.00	0.00	0.00	0.00	0.00
0.00	0.00	0.00	0.00	0.00	0.00	0.00	0.00	0.00
0.00	0.00	0.00	0.00	0.00	0.00	0.00	0.00	0.00

0.00	0.00	0.00	0.00	0.00	0.00	0.00	0.00	0.00
0.00	0.00	0.00	0.00	0.00	0.00	0.00	0.00	0.00
0.00	0.00	0.00	0.00	0.00	0.00	0.00	0.00	0.00
0.00	0.00	0.00	0.00	0.00	0.00	0.00	0.00	0.00
0.00	0.00	4.00	0.00	0.00	0.00	0.00	0.00	0.00
0.00	0.00	0.00	0.00	0.00	0.00	0.00	0.00	0.00
0.00	0.00	0.00	0.00	0.00	0.00	0.00	0.00	0.00
0.00	0.00	0.00	0.00	0.00	0.00	9.00	0.00	0.00
0.00	0.00	0.00	0.00	0.00	0.00	0.00	0.00	0.00
0.00	0.00	0.00	0.00	0.00	0.00	0.00	0.00	0.00
0.00	0.00	0.00	0.00	0.00	0.00	0.00	0.00	0.00
0.00	0.00	0.00	0.00	0.00	0.00	0.00	0.00	0.00
0.00	0.00	0.00	0.00	0.00	0.00	0.00	0.00	0.00
0.00	0.00	0.00	0.00	0.00	14.00	0.00	0.00	0.00
0.00	0.00	0.00	0.00	0.00	0.00	0.00	0.00	0.00
0.00	0.00	0.00	0.00	0.00	0.00	0.00	0.00	0.00
0.00	0.00	0.00	0.00	0.00	0.00	0.00	0.00	0.00
0.00	0.00	0.00	0.00	0.00	0.00	0.00	0.00	0.00
0.00	0.00	0.00	0.00	0.00	0.00	0.00	0.00	0.00
0.00	0.00	0.00	0.00	0.00	0.00	0.00	0.00	0.00
0.00	0.00	0.00	0.00	0.00	0.00	0.00	0.00	0.00
0.00	0.00	0.00	0.00	0.00	0.00	0.00	0.00	0.00
0.00	0.00	0.00	0.00	0.00	0.00	0.00	0.00	0.00
0.00	0.00	0.00	0.00	0.00	0.00	0.00	0.00	0.00
0.00	0.00	0.00	0.00	0.00	0.00	0.00	0.00	0.00
0.00	0.00	0.00	0.00	0.00	0.00	0.00	0.00	0.00
0.00	0.00	0.00	0.00	0.00	0.00	0.00	0.00	0.00
0.00	0.00	2.00	0.00	0.00	0.00	0.00	0.00	0.00
0.00	0.00	0.00	0.00	0.00	0.00	0.00	0.00	0.00
0.00	0.00	0.00	0.00	0.00	0.00	0.00	0.00	0.00
0.00	0.00	0.00	0.00	0.00	0.00	0.00	0.00	0.00
0.00	0.00	0.00	0.00	0.00	0.00	0.00	0.00	0.00
0.00	0.00	0.00	0.00	0.00	0.00	0.00	0.00	0.00
0.00	0.00	0.00	0.00	0.00	0.00	0.00	0.00	0.00
0.00	0.00	0.00	0.00	0.00	0.00	0.00	0.00	0.00
0.00	0.00	0.00	0.00	0.00	0.00	0.00	0.00	0.00
0.00	0.00	0.00	0.00	0.00	0.00	0.00	0.00	0.00

13.00	0.00	0.00	0.00	0.00	0.00	0.00	0.00	0.00
0.00	0.00	0.00	0.00	0.00	0.00	0.00	0.00	0.00
0.00	0.00	0.00	0.00	0.00	0.00	0.00	0.00	0.00
0.00	0.00	0.00	0.00	0.00	0.00	0.00	0.00	0.00
0.00	0.00	0.00	0.00	0.00	0.00	0.00	0.00	0.00
0.00	0.00	0.00	0.00	0.00	0.00	0.00	0.00	0.00
0.00	0.00	0.00	0.00	0.00	0.00	0.00	0.00	0.00
0.00	0.00	0.00	0.00	0.00	0.00	0.00	0.00	0.00
0.00	0.00	0.00	0.00	0.00	0.00	0.00	0.00	0.00
0.00	0.00	0.00	0.00	0.00	0.00	0.00	0.00	0.00
0.00	0.00	0.00	0.00	0.00	0.00	0.00	0.00	0.00
0.00	0.00	0.00	0.00	0.00	0.00	0.00	0.00	0.00
0.00	0.00	0.00	0.00	13.00	0.00	0.00	0.00	0.00
0.00	0.00	0.00	0.00	0.00	0.00	0.00	0.00	0.00
0.00	0.00	0.00	0.00	0.00	0.00	0.00	0.00	0.00
0.00	0.00	0.00	0.00	0.00	0.00	0.00	0.00	0.00
0.00	0.00	0.00	0.00	0.00	0.00	0.00	0.00	0.00
0.00	0.00	0.00	0.00	0.00	0.00	0.00	0.00	0.00
0.00	0.00	0.00	0.00	0.00	0.00	0.00	0.00	0.00
0.00	0.00	0.00	0.00	0.00	0.00	0.00	0.00	0.00
0.00	0.00	0.00	0.00	0.00	0.00	0.00	0.00	0.00
0.00	0.00	0.00	0.00	0.00	0.00	0.00	0.00	0.00
0.00	0.00	0.00	0.00	0.00	0.00	0.00	0.00	0.00
0.00	0.00	0.00	0.00	0.00	0.00	0.00	0.00	0.00
0.00	0.00	0.00	0.00	0.00	0.00	0.00	0.00	0.00
0.00	0.00	0.00	0.00	0.00	0.00	0.00	0.00	0.00
0.00	0.00	0.00	0.00	0.00	0.00	0.00	0.00	0.00
0.00	0.00	0.00	0.00	0.00	0.00	0.00	0.00	0.00
0.00	0.00	0.00	0.00	0.00	0.00	0.00	0.00	0.00
0.00	0.00	0.00	0.00	0.00	0.00	0.00	0.00	0.00
0.00	0.00	0.00	0.00	0.00	0.00	0.00	0.00	0.00
0.00	0.00	0.00	0.00	0.00	0.00	0.00	0.00	0.00
0.00	0.00	0.00	0.00	0.00	0.00	0.00	0.00	0.00
0.00	0.00	0.00	0.00	0.00	0.00	0.00	0.00	0.00
0.00	0.00	0.00	0.00	0.00	0.00	0.00	0.00	0.00
0.00	0.00	0.00	0.00	0.00	0.00	0.00	0.00	0.00
0.00	0.00	0.00	0.00	0.00	0.00	0.00	0.00	0.00

0.00	0.00	0.00	0.00	0.00	0.00	0.00	0.00	0.00
0.00	0.00	0.00	0.00	0.00	0.00	0.00	0.00	0.00
0.00	0.00	0.00	0.00	0.00	0.00	3.00	0.00	0.00
0.00	0.00	0.00	0.00	0.00	0.00	0.00	0.00	0.00
0.00	0.00	0.00	0.00	0.00	0.00	0.00	0.00	0.00
0.00	0.00	4.00	0.00	0.00	0.00	0.00	0.00	0.00
12.00	0.00	0.00	0.00	0.00	0.00	0.00	0.00	0.00
4.00	0.00	0.00	0.00	0.00	0.00	0.00	0.00	0.00
0.00	0.00	0.00	0.00	0.00	0.00	0.00	0.00	0.00
0.00	0.00	0.00	0.00	0.00	0.00	0.00	0.00	0.00
0.00	0.00	0.00	0.00	0.00	0.00	0.00	0.00	0.00
0.00	0.00	0.00	0.00	0.00	0.00	0.00	0.00	0.00
0.00	0.00	0.00	0.00	0.00	0.00	0.00	0.00	0.00
0.00	0.00	0.00	0.00	0.00	0.00	0.00	0.00	0.00
0.00	0.00	0.00	0.00	0.00	0.00	0.00	0.00	0.00
0.00	0.00	0.00	0.00	0.00	0.00	0.00	0.00	0.00
0.00	0.00	0.00	0.00	0.00	0.00	0.00	0.00	0.00
0.00	0.00	0.00	0.00	0.00	0.00	0.00	0.00	0.00
0.00	0.00	0.00	0.00	0.00	0.00	0.00	0.00	0.00
0.00	0.00	0.00	0.00	0.00	0.00	0.00	0.00	0.00
0.00	0.00	0.00	3.00	0.00	0.00	0.00	0.00	0.00
0.00	0.00	0.00	0.00	0.00	0.00	0.00	0.00	0.00
0.00	0.00	0.00	0.00	0.00	0.00	0.00	0.00	0.00
0.00	0.00	0.00	0.00	0.00	0.00	0.00	0.00	0.00
0.00	0.00	0.00	0.00	0.00	0.00	0.00	0.00	0.00
0.00	0.00	0.00	0.00	0.00	0.00	0.00	0.00	0.00
0.00	0.00	0.00	0.00	0.00	0.00	0.00	0.00	0.00
0.00	0.00	0.00	0.00	0.00	0.00	0.00	0.00	0.00
0.00	0.00	0.00	0.00	0.00	0.00	0.00	0.00	0.00
0.00	0.00	0.00	0.00	0.00	0.00	0.00	0.00	0.00
0.00	0.00	0.00	0.00	0.00	0.00	0.00	0.00	0.00
0.00	0.00	0.00	0.00	0.00	0.00	0.00	0.00	0.00
0.00	0.00	0.00	0.00	0.00	0.00	0.00	0.00	0.00
0.00	0.00	0.00	0.00	0.00	0.00	0.00	0.00	0.00
0.00	0.00	0.00	0.00	0.00	0.00	0.00	0.00	0.00
0.00	0.00	0.00	0.00	0.00	0.00	0.00	0.00	0.00
0.00	0.00	0.00	0.00	0.00	0.00	0.00	0.00	0.00

0.00	0.00	0.00	0.00	0.00	0.00	0.00	0.00	0.00
0.00	0.00	0.00	0.00	0.00	0.00	0.00	0.00	0.00
0.00	0.00	0.00	0.00	0.00	0.00	0.00	0.00	0.00
11.00	0.00	0.00	0.00	0.00	0.00	0.00	0.00	0.00
0.00	0.00	0.00	0.00	0.00	0.00	0.00	0.00	0.00
0.00	0.00	0.00	0.00	0.00	0.00	0.00	0.00	0.00
0.00	0.00	0.00	0.00	0.00	0.00	0.00	0.00	0.00
0.00	0.00	0.00	0.00	0.00	0.00	0.00	0.00	0.00
0.00	0.00	0.00	0.00	0.00	0.00	0.00	0.00	0.00
0.00	0.00	0.00	0.00	0.00	0.00	0.00	0.00	0.00
0.00	0.00	0.00	0.00	0.00	0.00	0.00	0.00	0.00
0.00	0.00	0.00	0.00	0.00	4.00	0.00	0.00	0.00
0.00	0.00	0.00	0.00	0.00	0.00	0.00	0.00	0.00
0.00	0.00	0.00	0.00	0.00	0.00	0.00	0.00	0.00
0.00	0.00	0.00	0.00	0.00	0.00	0.00	0.00	0.00
0.00	0.00	0.00	0.00	0.00	7.00	0.00	0.00	0.00
0.00	0.00	0.00	0.00	0.00	0.00	0.00	0.00	0.00
0.00	0.00	0.00	0.00	0.00	0.00	0.00	0.00	0.00
0.00	0.00	0.00	0.00	0.00	0.00	0.00	0.00	0.00
0.00	0.00	0.00	0.00	0.00	0.00	0.00	0.00	0.00
0.00	0.00	0.00	0.00	0.00	0.00	0.00	0.00	0.00
0.00	0.00	0.00	0.00	0.00	0.00	0.00	0.00	0.00
0.00	0.00	0.00	0.00	0.00	0.00	0.00	0.00	0.00
0.00	0.00	0.00	0.00	0.00	0.00	0.00	0.00	0.00
0.00	0.00	0.00	0.00	0.00	0.00	0.00	0.00	0.00
0.00	0.00	0.00	0.00	0.00	0.00	0.00	0.00	0.00
0.00	0.00	0.00	0.00	0.00	0.00	0.00	0.00	0.00
0.00	0.00	0.00	0.00	0.00	0.00	0.00	0.00	0.00
0.00	0.00	0.00	0.00	0.00	0.00	0.00	0.00	0.00
0.00	0.00	0.00	0.00	0.00	0.00	0.00	0.00	0.00
0.00	0.00	0.00	0.00	0.00	0.00	0.00	0.00	0.00
0.00	0.00	0.00	0.00	0.00	0.00	0.00	0.00	0.00
0.00	0.00	0.00	0.00	0.00	0.00	0.00	0.00	0.00
0.00	0.00	0.00	0.00	0.00	0.00	0.00	0.00	0.00
0.00	0.00	0.00	0.00	0.00	0.00	0.00	0.00	0.00
0.00	0.00	0.00	0.00	0.00	0.00	0.00	0.00	0.00
0.00	0.00	0.00	0.00	0.00	0.00	0.00	0.00	0.00

0.00	0.00	0.00	0.00	0.00	0.00	0.00	0.00	0.00
0.00	0.00	0.00	2.00	0.00	0.00	0.00	0.00	0.00
0.00	0.00	0.00	0.00	0.00	0.00	0.00	0.00	0.00
0.00	0.00	0.00	0.00	0.00	0.00	0.00	0.00	0.00
0.00	0.00	0.00	0.00	0.00	0.00	0.00	0.00	0.00
0.00	0.00	0.00	0.00	0.00	0.00	0.00	0.00	0.00
0.00	0.00	0.00	0.00	0.00	0.00	0.00	0.00	0.00
0.00	0.00	0.00	0.00	0.00	0.00	0.00	0.00	0.00
0.00	0.00	0.00	0.00	0.00	0.00	0.00	0.00	0.00
0.00	0.00	0.00	0.00	0.00	0.00	0.00	0.00	0.00
0.00	0.00	0.00	0.00	0.00	0.00	0.00	0.00	0.00
0.00	0.00	0.00	0.00	0.00	0.00	0.00	0.00	0.00
0.00	0.00	0.00	0.00	0.00	0.00	0.00	0.00	0.00
0.00	0.00	0.00	0.00	0.00	0.00	0.00	0.00	0.00
0.00	0.00	0.00	0.00	0.00	0.00	0.00	0.00	0.00
0.00	0.00	0.00	0.00	0.00	0.00	0.00	0.00	0.00
10.00	0.00	0.00	0.00	0.00	0.00	0.00	0.00	0.00
0.00	0.00	0.00	0.00	0.00	0.00	0.00	0.00	0.00
0.00	0.00	0.00	0.00	0.00	0.00	0.00	0.00	0.00
0.00	0.00	0.00	0.00	0.00	0.00	0.00	0.00	0.00
0.00	0.00	0.00	0.00	0.00	0.00	0.00	0.00	0.00
0.00	0.00	0.00	0.00	0.00	0.00	0.00	0.00	0.00
0.00	0.00	0.00	0.00	0.00	0.00	0.00	0.00	0.00
0.00	0.00	0.00	0.00	0.00	0.00	0.00	0.00	0.00
0.00	0.00	0.00	0.00	0.00	0.00	0.00	0.00	0.00
0.00	0.00	0.00	0.00	0.00	0.00	0.00	0.00	0.00
0.00	0.00	0.00	0.00	0.00	0.00	0.00	0.00	0.00
0.00	0.00	0.00	0.00	0.00	0.00	0.00	0.00	0.00
0.00	0.00	0.00	0.00	0.00	0.00	0.00	0.00	0.00
0.00	0.00	0.00	0.00	0.00	0.00	0.00	0.00	0.00
0.00	0.00	0.00	0.00	0.00	0.00	0.00	0.00	0.00
0.00	0.00	0.00	0.00	0.00	0.00	0.00	0.00	0.00
0.00	0.00	0.00	0.00	0.00	0.00	0.00	0.00	0.00
0.00	0.00	0.00	0.00	0.00	0.00	0.00	0.00	0.00
0.00	0.00	0.00	0.00	0.00	0.00	0.00	0.00	0.00
0.00	0.00	0.00	0.00	0.00	0.00	0.00	0.00	0.00
0.00	0.00	0.00	0.00	0.00	0.00	0.00	0.00	0.00

0.00	0.00	0.00	0.00	0.00	0.00	0.00	0.00	0.00
0.00	0.00	0.00	0.00	0.00	0.00	0.00	0.00	0.00
0.00	0.00	0.00	0.00	0.00	0.00	0.00	0.00	0.00
0.00	0.00	0.00	0.00	0.00	0.00	0.00	0.00	0.00
0.00	0.00	0.00	0.00	0.00	0.00	0.00	0.00	0.00
0.00	0.00	0.00	0.00	0.00	0.00	0.00	0.00	0.00
0.00	0.00	0.00	0.00	0.00	0.00	0.00	0.00	0.00
0.00	0.00	0.00	0.00	0.00	0.00	10.00	0.00	0.00
0.00	0.00	0.00	0.00	0.00	0.00	0.00	0.00	0.00
0.00	0.00	0.00	0.00	0.00	0.00	0.00	0.00	0.00
0.00	0.00	0.00	0.00	0.00	0.00	0.00	0.00	0.00
0.00	0.00	0.00	0.00	0.00	0.00	0.00	0.00	0.00
0.00	0.00	0.00	0.00	0.00	0.00	0.00	0.00	0.00
0.00	0.00	0.00	0.00	0.00	0.00	0.00	0.00	0.00
0.00	0.00	0.00	0.00	0.00	0.00	0.00	0.00	0.00
0.00	0.00	0.00	0.00	0.00	0.00	0.00	0.00	0.00
0.00	0.00	0.00	0.00	0.00	0.00	0.00	0.00	0.00
0.00	0.00	0.00	0.00	0.00	0.00	0.00	0.00	0.00
0.00	0.00	0.00	0.00	0.00	0.00	0.00	0.00	0.00
0.00	0.00	0.00	0.00	0.00	0.00	0.00	0.00	0.00
0.00	0.00	0.00	0.00	0.00	0.00	0.00	0.00	0.00
0.00	0.00	0.00	0.00	0.00	0.00	0.00	0.00	0.00
0.00	0.00	0.00	0.00	0.00	0.00	0.00	0.00	0.00
0.00	0.00	0.00	0.00	0.00	0.00	0.00	0.00	0.00
0.00	0.00	0.00	0.00	0.00	0.00	0.00	0.00	0.00
0.00	0.00	0.00	0.00	0.00	0.00	0.00	0.00	0.00
0.00	0.00	0.00	0.00	0.00	0.00	0.00	0.00	0.00
0.00	0.00	0.00	0.00	0.00	0.00	0.00	0.00	0.00
0.00	0.00	0.00	0.00	0.00	0.00	0.00	0.00	0.00
0.00	0.00	0.00	0.00	0.00	0.00	0.00	0.00	0.00
0.00	0.00	0.00	0.00	0.00	0.00	0.00	0.00	0.00
0.00	0.00	0.00	0.00	0.00	0.00	0.00	0.00	0.00
0.00	0.00	0.00	0.00	0.00	0.00	0.00	0.00	0.00
0.00	0.00	0.00	0.00	0.00	0.00	0.00	0.00	0.00
0.00	0.00	0.00	0.00	0.00	0.00	0.00	0.00	0.00
5.00	0.00	0.00	0.00	0.00	0.00	0.00	0.00	0.00
9.00	0.00	0.00	0.00	0.00	0.00	0.00	0.00	0.00

2.00	0.00	0.00	0.00	0.00	0.00	0.00	0.00	0.00
0.00	0.00	0.00	0.00	0.00	0.00	0.00	0.00	0.00
0.00	0.00	0.00	0.00	0.00	0.00	0.00	0.00	0.00
0.00	0.00	0.00	0.00	0.00	0.00	0.00	0.00	0.00
0.00	0.00	0.00	0.00	0.00	0.00	0.00	0.00	0.00
0.00	0.00	0.00	0.00	0.00	0.00	0.00	0.00	0.00
0.00	0.00	0.00	0.00	0.00	0.00	0.00	0.00	0.00
0.00	0.00	0.00	0.00	0.00	0.00	0.00	0.00	0.00
0.00	0.00	0.00	0.00	0.00	0.00	0.00	0.00	0.00
0.00	0.00	0.00	0.00	0.00	0.00	0.00	0.00	0.00
0.00	0.00	0.00	0.00	0.00	0.00	0.00	0.00	0.00
0.00	0.00	0.00	0.00	0.00	0.00	0.00	0.00	0.00
0.00	0.00	0.00	0.00	0.00	0.00	0.00	0.00	0.00
0.00	0.00	0.00	0.00	0.00	0.00	0.00	2.00	0.00
0.00	0.00	0.00	0.00	0.00	0.00	0.00	0.00	0.00
0.00	0.00	0.00	0.00	0.00	0.00	0.00	0.00	0.00
0.00	0.00	0.00	0.00	0.00	0.00	0.00	0.00	0.00
0.00	0.00	0.00	0.00	0.00	0.00	0.00	0.00	0.00
0.00	0.00	0.00	0.00	0.00	0.00	0.00	0.00	0.00
0.00	0.00	0.00	0.00	0.00	9.00	0.00	0.00	0.00
0.00	0.00	0.00	0.00	0.00	0.00	0.00	0.00	0.00
0.00	0.00	0.00	0.00	0.00	0.00	0.00	0.00	0.00
0.00	0.00	0.00	0.00	0.00	0.00	0.00	0.00	0.00
0.00	0.00	0.00	0.00	0.00	0.00	0.00	0.00	0.00
0.00	0.00	0.00	0.00	0.00	0.00	0.00	0.00	0.00
0.00	0.00	0.00	0.00	0.00	0.00	0.00	0.00	0.00
0.00	0.00	0.00	0.00	0.00	0.00	0.00	0.00	0.00
0.00	0.00	0.00	0.00	0.00	0.00	0.00	9.00	0.00
0.00	0.00	0.00	0.00	0.00	0.00	9.00	0.00	0.00
0.00	0.00	0.00	0.00	0.00	0.00	0.00	0.00	0.00
0.00	0.00	0.00	0.00	0.00	0.00	0.00	0.00	0.00
0.00	0.00	0.00	0.00	0.00	0.00	0.00	0.00	0.00
0.00	0.00	0.00	0.00	0.00	0.00	0.00	0.00	0.00
0.00	0.00	0.00	0.00	0.00	0.00	0.00	0.00	0.00
0.00	0.00	0.00	0.00	0.00	0.00	0.00	0.00	0.00
0.00	0.00	0.00	0.00	0.00	0.00	0.00	0.00	0.00
0.00	0.00	0.00	0.00	0.00	0.00	0.00	0.00	0.00

0.00	0.00	0.00	0.00	0.00	0.00	0.00	0.00	0.00
0.00	0.00	0.00	0.00	0.00	0.00	0.00	0.00	0.00
0.00	0.00	0.00	0.00	0.00	0.00	0.00	0.00	0.00
0.00	0.00	0.00	0.00	0.00	0.00	0.00	0.00	0.00
0.00	0.00	0.00	0.00	0.00	0.00	0.00	0.00	0.00
0.00	0.00	0.00	0.00	0.00	0.00	0.00	0.00	0.00
0.00	0.00	0.00	0.00	0.00	0.00	0.00	0.00	0.00
0.00	0.00	0.00	0.00	0.00	0.00	0.00	0.00	0.00
0.00	0.00	0.00	0.00	0.00	0.00	0.00	0.00	0.00
0.00	0.00	0.00	0.00	0.00	0.00	0.00	0.00	0.00
0.00	0.00	0.00	0.00	0.00	0.00	0.00	0.00	0.00
0.00	0.00	0.00	0.00	0.00	0.00	0.00	0.00	0.00
0.00	0.00	0.00	0.00	0.00	0.00	0.00	0.00	0.00
0.00	0.00	0.00	0.00	0.00	0.00	0.00	0.00	0.00
0.00	0.00	0.00	0.00	0.00	0.00	0.00	0.00	0.00
0.00	0.00	0.00	0.00	0.00	0.00	0.00	0.00	0.00
8.00	0.00	0.00	0.00	0.00	0.00	0.00	0.00	0.00
8.00	0.00	0.00	0.00	0.00	0.00	0.00	0.00	0.00
8.00	0.00	0.00	0.00	0.00	0.00	0.00	0.00	0.00
0.00	0.00	0.00	0.00	0.00	0.00	0.00	0.00	0.00
0.00	0.00	0.00	0.00	0.00	0.00	0.00	0.00	0.00
0.00	0.00	0.00	0.00	0.00	0.00	0.00	0.00	0.00
0.00	0.00	0.00	0.00	0.00	0.00	0.00	0.00	0.00
0.00	0.00	0.00	0.00	0.00	0.00	0.00	0.00	0.00
0.00	0.00	0.00	0.00	0.00	0.00	0.00	0.00	0.00
0.00	0.00	0.00	0.00	0.00	0.00	0.00	0.00	0.00
0.00	0.00	0.00	0.00	0.00	0.00	0.00	0.00	0.00
0.00	0.00	0.00	0.00	0.00	0.00	0.00	0.00	0.00
0.00	0.00	0.00	0.00	0.00	0.00	0.00	0.00	0.00
0.00	0.00	0.00	0.00	0.00	0.00	0.00	0.00	0.00
0.00	0.00	0.00	0.00	0.00	0.00	0.00	0.00	0.00
0.00	0.00	0.00	0.00	0.00	0.00	0.00	0.00	0.00
0.00	0.00	0.00	0.00	0.00	0.00	0.00	0.00	0.00
0.00	0.00	0.00	0.00	0.00	0.00	0.00	0.00	0.00
0.00	0.00	0.00	0.00	0.00	0.00	0.00	0.00	0.00
0.00	0.00	0.00	0.00	0.00	0.00	0.00	0.00	0.00
0.00	0.00	0.00	0.00	0.00	0.00	0.00	0.00	0.00

0.00	0.00	0.00	0.00	0.00	0.00	0.00	0.00	0.00
0.00	0.00	0.00	0.00	0.00	0.00	0.00	0.00	0.00
0.00	0.00	0.00	0.00	0.00	0.00	0.00	0.00	0.00
0.00	0.00	0.00	0.00	0.00	0.00	0.00	0.00	0.00
0.00	0.00	0.00	0.00	0.00	0.00	0.00	0.00	0.00
0.00	0.00	0.00	0.00	0.00	0.00	0.00	0.00	0.00
0.00	0.00	0.00	0.00	0.00	0.00	0.00	0.00	0.00
0.00	0.00	0.00	0.00	0.00	0.00	0.00	0.00	0.00
0.00	0.00	0.00	0.00	0.00	0.00	0.00	0.00	0.00
0.00	0.00	0.00	0.00	0.00	0.00	0.00	0.00	0.00
0.00	0.00	0.00	0.00	0.00	0.00	0.00	0.00	0.00
0.00	0.00	0.00	0.00	0.00	0.00	0.00	0.00	0.00
0.00	0.00	0.00	0.00	0.00	0.00	0.00	0.00	0.00
0.00	0.00	0.00	0.00	0.00	0.00	0.00	0.00	0.00
0.00	0.00	0.00	0.00	0.00	0.00	0.00	0.00	0.00
0.00	0.00	0.00	0.00	0.00	0.00	0.00	0.00	0.00
0.00	0.00	0.00	0.00	0.00	0.00	0.00	0.00	0.00
0.00	0.00	0.00	0.00	0.00	0.00	0.00	0.00	0.00
0.00	0.00	0.00	0.00	0.00	0.00	0.00	0.00	0.00
0.00	0.00	0.00	0.00	0.00	0.00	0.00	0.00	0.00
0.00	0.00	0.00	0.00	0.00	0.00	0.00	0.00	0.00
0.00	0.00	0.00	0.00	0.00	0.00	0.00	0.00	0.00
0.00	0.00	0.00	0.00	0.00	0.00	0.00	0.00	0.00
0.00	0.00	0.00	0.00	0.00	0.00	0.00	0.00	0.00
0.00	0.00	0.00	0.00	0.00	0.00	0.00	0.00	0.00
0.00	0.00	0.00	0.00	0.00	0.00	0.00	0.00	0.00
0.00	0.00	0.00	0.00	0.00	0.00	0.00	0.00	0.00
0.00	0.00	0.00	0.00	0.00	0.00	0.00	0.00	0.00
0.00	0.00	0.00	0.00	0.00	0.00	0.00	0.00	0.00
0.00	0.00	0.00	0.00	0.00	0.00	0.00	0.00	0.00
0.00	0.00	0.00	0.00	0.00	0.00	0.00	0.00	0.00
0.00	0.00	0.00	0.00	0.00	0.00	0.00	0.00	0.00
0.00	0.00	0.00	0.00	0.00	0.00	0.00	0.00	0.00
0.00	0.00	7.00	0.00	0.00	0.00	0.00	0.00	0.00
0.00	0.00	0.00	0.00	0.00	0.00	0.00	0.00	0.00
0.00	0.00	0.00	0.00	0.00	0.00	0.00	0.00	0.00
0.00	0.00	0.00	0.00	0.00	0.00	0.00	0.00	0.00

0.00	0.00	0.00	0.00	0.00	0.00	0.00	0.00	0.00
0.00	0.00	0.00	0.00	0.00	0.00	0.00	0.00	0.00
0.00	0.00	0.00	0.00	0.00	0.00	0.00	0.00	0.00
0.00	0.00	0.00	0.00	0.00	0.00	0.00	0.00	0.00
0.00	0.00	0.00	0.00	0.00	0.00	0.00	0.00	0.00
0.00	0.00	0.00	0.00	0.00	0.00	0.00	0.00	0.00
0.00	0.00	0.00	0.00	0.00	0.00	0.00	0.00	0.00
0.00	0.00	0.00	0.00	0.00	0.00	0.00	0.00	0.00
0.00	0.00	0.00	0.00	0.00	0.00	0.00	0.00	0.00
0.00	0.00	0.00	0.00	0.00	0.00	0.00	0.00	0.00
0.00	0.00	0.00	0.00	0.00	0.00	0.00	0.00	0.00
0.00	0.00	0.00	0.00	0.00	0.00	0.00	0.00	0.00
0.00	0.00	0.00	0.00	0.00	0.00	0.00	0.00	0.00
0.00	0.00	0.00	0.00	0.00	0.00	0.00	0.00	0.00
0.00	0.00	0.00	0.00	0.00	0.00	0.00	0.00	0.00
0.00	0.00	0.00	0.00	0.00	0.00	0.00	0.00	0.00
0.00	0.00	0.00	0.00	0.00	0.00	0.00	0.00	0.00
0.00	0.00	0.00	0.00	0.00	0.00	0.00	0.00	0.00
0.00	0.00	0.00	0.00	0.00	0.00	0.00	0.00	0.00
0.00	0.00	0.00	0.00	0.00	0.00	0.00	0.00	0.00
0.00	0.00	0.00	0.00	0.00	0.00	0.00	0.00	0.00
0.00	0.00	0.00	0.00	0.00	0.00	0.00	0.00	0.00
0.00	0.00	0.00	0.00	0.00	0.00	0.00	0.00	0.00
0.00	0.00	0.00	0.00	0.00	0.00	0.00	0.00	0.00
0.00	0.00	0.00	0.00	0.00	0.00	0.00	0.00	0.00
0.00	0.00	0.00	0.00	0.00	0.00	0.00	0.00	0.00
0.00	0.00	0.00	0.00	0.00	0.00	0.00	0.00	0.00
0.00	0.00	0.00	0.00	0.00	0.00	0.00	0.00	0.00
0.00	0.00	0.00	0.00	0.00	0.00	0.00	0.00	0.00
0.00	0.00	0.00	0.00	0.00	0.00	0.00	0.00	0.00
0.00	0.00	0.00	0.00	0.00	0.00	0.00	0.00	0.00
0.00	0.00	0.00	0.00	0.00	0.00	0.00	0.00	0.00
0.00	0.00	0.00	0.00	0.00	0.00	0.00	0.00	0.00
0.00	0.00	0.00	0.00	0.00	0.00	0.00	0.00	0.00
0.00	0.00	0.00	0.00	0.00	0.00	0.00	0.00	0.00
0.00	0.00	0.00	0.00	0.00	0.00	0.00	0.00	0.00
0.00	0.00	0.00	0.00	0.00	0.00	0.00	0.00	0.00

0.00	0.00	0.00	0.00	0.00	0.00	0.00	0.00	0.00
0.00	0.00	0.00	0.00	0.00	0.00	0.00	0.00	0.00
0.00	0.00	0.00	0.00	0.00	0.00	0.00	0.00	0.00
0.00	0.00	0.00	0.00	0.00	0.00	0.00	0.00	0.00
0.00	0.00	0.00	0.00	0.00	0.00	0.00	0.00	0.00
0.00	0.00	0.00	0.00	0.00	0.00	0.00	0.00	0.00
0.00	0.00	0.00	0.00	0.00	0.00	0.00	0.00	0.00
0.00	0.00	0.00	0.00	0.00	0.00	0.00	0.00	0.00
0.00	0.00	0.00	0.00	0.00	0.00	0.00	0.00	0.00
0.00	0.00	0.00	0.00	0.00	0.00	0.00	0.00	0.00
0.00	0.00	0.00	0.00	0.00	0.00	0.00	0.00	0.00
0.00	0.00	0.00	0.00	0.00	0.00	0.00	0.00	0.00
0.00	0.00	0.00	0.00	0.00	0.00	0.00	0.00	0.00
0.00	0.00	0.00	0.00	0.00	0.00	0.00	0.00	0.00
0.00	0.00	0.00	0.00	0.00	0.00	0.00	0.00	0.00
0.00	0.00	0.00	0.00	0.00	0.00	0.00	0.00	0.00
0.00	0.00	0.00	0.00	0.00	0.00	0.00	0.00	0.00
0.00	0.00	0.00	0.00	0.00	0.00	0.00	0.00	0.00
0.00	0.00	0.00	0.00	0.00	0.00	0.00	0.00	0.00
0.00	0.00	0.00	0.00	0.00	0.00	0.00	0.00	0.00
0.00	0.00	0.00	0.00	0.00	0.00	0.00	0.00	0.00
0.00	0.00	0.00	0.00	0.00	0.00	0.00	0.00	0.00
0.00	0.00	0.00	0.00	0.00	0.00	0.00	0.00	0.00
0.00	0.00	0.00	0.00	0.00	0.00	0.00	0.00	0.00
0.00	0.00	0.00	0.00	0.00	0.00	0.00	0.00	0.00
0.00	0.00	0.00	0.00	0.00	0.00	0.00	0.00	0.00
0.00	0.00	3.00	0.00	0.00	0.00	0.00	0.00	0.00
0.00	0.00	0.00	0.00	0.00	0.00	0.00	0.00	0.00
0.00	0.00	0.00	0.00	0.00	0.00	0.00	0.00	0.00
0.00	0.00	0.00	0.00	0.00	0.00	0.00	0.00	0.00
0.00	0.00	0.00	0.00	0.00	0.00	0.00	0.00	0.00
0.00	0.00	0.00	0.00	0.00	0.00	0.00	0.00	0.00
0.00	0.00	0.00	0.00	0.00	0.00	0.00	0.00	0.00
0.00	0.00	0.00	0.00	0.00	0.00	0.00	0.00	0.00
0.00	0.00	0.00	0.00	0.00	0.00	0.00	0.00	0.00
0.00	0.00	0.00	0.00	0.00	0.00	0.00	0.00	0.00
0.00	0.00	0.00	0.00	0.00	0.00	0.00	0.00	0.00

0.00	0.00	0.00	0.00	0.00	0.00	0.00	0.00	0.00
0.00	0.00	0.00	0.00	0.00	0.00	0.00	0.00	0.00
0.00	0.00	0.00	0.00	0.00	0.00	0.00	0.00	0.00
0.00	0.00	0.00	0.00	0.00	0.00	0.00	0.00	0.00
0.00	0.00	0.00	0.00	0.00	0.00	0.00	0.00	0.00
0.00	0.00	0.00	0.00	0.00	0.00	2.00	0.00	0.00
0.00	0.00	0.00	0.00	0.00	0.00	0.00	0.00	0.00
0.00	0.00	0.00	0.00	0.00	0.00	0.00	0.00	0.00
0.00	0.00	0.00	0.00	0.00	0.00	0.00	0.00	0.00
0.00	0.00	0.00	0.00	0.00	0.00	0.00	0.00	0.00
0.00	0.00	0.00	6.00	0.00	0.00	0.00	0.00	0.00
0.00	0.00	0.00	0.00	0.00	0.00	0.00	0.00	0.00
0.00	0.00	0.00	0.00	0.00	0.00	0.00	0.00	0.00
0.00	0.00	0.00	0.00	0.00	0.00	0.00	0.00	0.00
0.00	0.00	0.00	0.00	0.00	0.00	0.00	0.00	0.00
0.00	0.00	0.00	0.00	0.00	0.00	0.00	0.00	0.00
0.00	0.00	0.00	0.00	0.00	0.00	0.00	0.00	0.00
0.00	0.00	0.00	0.00	0.00	0.00	0.00	0.00	0.00
0.00	0.00	0.00	0.00	0.00	0.00	0.00	0.00	0.00
0.00	0.00	0.00	0.00	0.00	0.00	0.00	0.00	0.00
0.00	0.00	0.00	0.00	0.00	0.00	0.00	0.00	0.00
0.00	0.00	0.00	0.00	0.00	0.00	0.00	0.00	0.00
0.00	0.00	0.00	0.00	0.00	0.00	0.00	0.00	0.00
0.00	0.00	0.00	0.00	0.00	0.00	0.00	2.00	0.00
0.00	0.00	0.00	0.00	0.00	0.00	3.00	0.00	0.00
0.00	0.00	0.00	0.00	0.00	0.00	0.00	0.00	0.00
0.00	0.00	0.00	0.00	0.00	0.00	0.00	0.00	0.00
0.00	0.00	0.00	0.00	0.00	0.00	0.00	6.00	0.00
0.00	0.00	0.00	0.00	0.00	0.00	6.00	0.00	0.00
0.00	0.00	0.00	0.00	0.00	0.00	6.00	0.00	0.00
0.00	0.00	0.00	0.00	0.00	0.00	0.00	0.00	0.00
0.00	0.00	0.00	0.00	0.00	0.00	0.00	0.00	0.00
0.00	0.00	0.00	0.00	0.00	0.00	0.00	0.00	0.00
0.00	0.00	0.00	0.00	0.00	0.00	0.00	0.00	0.00
0.00	0.00	0.00	0.00	0.00	0.00	0.00	0.00	0.00
0.00	0.00	0.00	0.00	0.00	0.00	0.00	0.00	0.00
0.00	0.00	0.00	0.00	0.00	0.00	0.00	0.00	0.00

0.00	0.00	0.00	0.00	0.00	0.00	0.00	0.00	0.00
0.00	0.00	0.00	0.00	0.00	0.00	0.00	0.00	0.00
0.00	0.00	0.00	0.00	0.00	0.00	0.00	0.00	0.00
0.00	0.00	0.00	0.00	0.00	0.00	0.00	0.00	0.00
0.00	0.00	0.00	0.00	0.00	0.00	0.00	0.00	0.00
0.00	0.00	0.00	0.00	0.00	0.00	0.00	0.00	0.00
0.00	0.00	0.00	0.00	0.00	0.00	0.00	0.00	0.00
0.00	0.00	0.00	0.00	0.00	0.00	0.00	0.00	0.00
0.00	0.00	0.00	0.00	0.00	0.00	0.00	0.00	0.00
0.00	0.00	0.00	0.00	0.00	0.00	0.00	0.00	0.00
0.00	0.00	0.00	0.00	0.00	0.00	0.00	0.00	0.00
0.00	0.00	0.00	0.00	0.00	0.00	0.00	0.00	0.00
0.00	0.00	0.00	0.00	0.00	0.00	0.00	0.00	0.00
0.00	0.00	0.00	0.00	0.00	0.00	0.00	0.00	0.00
0.00	0.00	0.00	0.00	0.00	0.00	3.00	0.00	0.00
0.00	0.00	0.00	0.00	0.00	0.00	0.00	0.00	0.00
0.00	0.00	0.00	0.00	0.00	0.00	0.00	0.00	0.00
0.00	0.00	0.00	0.00	0.00	0.00	0.00	0.00	0.00
0.00	0.00	0.00	0.00	0.00	0.00	0.00	0.00	0.00
0.00	0.00	0.00	0.00	0.00	0.00	0.00	0.00	0.00
0.00	0.00	0.00	0.00	0.00	0.00	0.00	0.00	0.00
0.00	0.00	0.00	0.00	0.00	0.00	0.00	0.00	0.00
0.00	0.00	0.00	0.00	0.00	0.00	0.00	0.00	0.00
0.00	0.00	0.00	0.00	0.00	0.00	0.00	0.00	0.00
0.00	0.00	0.00	0.00	0.00	0.00	0.00	0.00	0.00
0.00	0.00	0.00	0.00	0.00	0.00	0.00	0.00	0.00
0.00	0.00	0.00	0.00	0.00	0.00	0.00	0.00	0.00
0.00	0.00	0.00	0.00	0.00	0.00	0.00	0.00	0.00
0.00	0.00	0.00	0.00	0.00	0.00	0.00	0.00	0.00
0.00	0.00	0.00	0.00	0.00	0.00	0.00	0.00	0.00
0.00	0.00	0.00	0.00	0.00	0.00	0.00	0.00	0.00
0.00	0.00	0.00	0.00	0.00	0.00	0.00	0.00	0.00
0.00	0.00	0.00	0.00	0.00	0.00	0.00	0.00	0.00
0.00	0.00	0.00	0.00	0.00	0.00	0.00	0.00	0.00
0.00	0.00	0.00	0.00	0.00	0.00	0.00	0.00	0.00
0.00	0.00	0.00	0.00	0.00	0.00	0.00	0.00	0.00
0.00	0.00	0.00	0.00	0.00	0.00	0.00	0.00	0.00

0.00	3.00	2.00	0.00	0.00	0.00	0.00	0.00	0.00
5.00	0.00	0.00	0.00	0.00	0.00	0.00	0.00	0.00
2.00	0.00	0.00	0.00	0.00	0.00	0.00	0.00	0.00
0.00	0.00	0.00	0.00	0.00	0.00	0.00	0.00	0.00
0.00	0.00	0.00	0.00	0.00	0.00	0.00	0.00	0.00
0.00	0.00	0.00	0.00	0.00	0.00	0.00	0.00	0.00
0.00	0.00	0.00	0.00	0.00	0.00	0.00	0.00	0.00
0.00	0.00	0.00	0.00	0.00	0.00	0.00	0.00	0.00
0.00	0.00	0.00	0.00	0.00	0.00	0.00	0.00	0.00
0.00	0.00	0.00	0.00	0.00	0.00	0.00	0.00	0.00
0.00	0.00	0.00	0.00	0.00	0.00	0.00	0.00	0.00
0.00	0.00	0.00	0.00	0.00	0.00	0.00	0.00	0.00
0.00	0.00	0.00	0.00	0.00	0.00	0.00	0.00	0.00
0.00	0.00	0.00	0.00	0.00	0.00	0.00	0.00	0.00
0.00	0.00	0.00	0.00	0.00	0.00	0.00	0.00	0.00
0.00	0.00	0.00	0.00	0.00	0.00	0.00	0.00	0.00
0.00	0.00	0.00	0.00	0.00	0.00	0.00	0.00	0.00
0.00	0.00	0.00	0.00	0.00	0.00	0.00	0.00	0.00
0.00	0.00	0.00	0.00	0.00	0.00	0.00	0.00	0.00
0.00	0.00	0.00	0.00	0.00	0.00	0.00	0.00	0.00
0.00	0.00	0.00	0.00	0.00	0.00	0.00	0.00	0.00
0.00	0.00	0.00	0.00	0.00	0.00	0.00	0.00	0.00
0.00	0.00	0.00	0.00	0.00	0.00	0.00	0.00	0.00
0.00	0.00	0.00	0.00	0.00	0.00	0.00	0.00	0.00
0.00	0.00	0.00	0.00	0.00	0.00	0.00	0.00	0.00
0.00	0.00	0.00	0.00	0.00	5.00	0.00	0.00	0.00
0.00	0.00	0.00	5.00	0.00	0.00	0.00	0.00	0.00
0.00	0.00	0.00	0.00	0.00	0.00	0.00	0.00	0.00
0.00	0.00	0.00	0.00	0.00	0.00	0.00	0.00	0.00
0.00	0.00	0.00	0.00	0.00	0.00	0.00	0.00	0.00
0.00	0.00	0.00	0.00	0.00	0.00	0.00	0.00	0.00
0.00	0.00	0.00	0.00	0.00	0.00	0.00	0.00	0.00
0.00	0.00	0.00	0.00	0.00	0.00	0.00	0.00	0.00
0.00	0.00	0.00	0.00	0.00	0.00	0.00	0.00	0.00
0.00	0.00	0.00	0.00	0.00	0.00	0.00	0.00	0.00
0.00	0.00	0.00	0.00	0.00	0.00	0.00	0.00	0.00
0.00	0.00	0.00	0.00	0.00	0.00	0.00	0.00	0.00

0.00	0.00	0.00	0.00	0.00	0.00	0.00	0.00	0.00
0.00	0.00	0.00	0.00	0.00	0.00	0.00	0.00	0.00
0.00	0.00	0.00	0.00	0.00	0.00	0.00	0.00	0.00
0.00	0.00	0.00	0.00	0.00	0.00	0.00	0.00	0.00
0.00	0.00	0.00	0.00	0.00	0.00	0.00	0.00	0.00
0.00	0.00	0.00	0.00	0.00	0.00	0.00	0.00	0.00
0.00	0.00	0.00	0.00	0.00	0.00	0.00	0.00	0.00
0.00	0.00	0.00	0.00	0.00	0.00	0.00	0.00	0.00
0.00	0.00	0.00	0.00	0.00	0.00	5.00	0.00	0.00
0.00	0.00	0.00	0.00	0.00	0.00	5.00	0.00	0.00
0.00	0.00	0.00	0.00	0.00	0.00	5.00	0.00	0.00
0.00	0.00	0.00	0.00	0.00	0.00	0.00	0.00	0.00
0.00	0.00	0.00	0.00	0.00	0.00	0.00	0.00	0.00
0.00	0.00	0.00	0.00	0.00	0.00	0.00	0.00	0.00
0.00	0.00	0.00	0.00	0.00	0.00	0.00	0.00	0.00
0.00	0.00	0.00	0.00	0.00	0.00	0.00	0.00	0.00
0.00	0.00	0.00	0.00	0.00	0.00	0.00	0.00	0.00
0.00	0.00	0.00	0.00	0.00	0.00	0.00	0.00	0.00
0.00	0.00	0.00	0.00	0.00	0.00	0.00	0.00	0.00
0.00	0.00	0.00	0.00	0.00	0.00	0.00	0.00	0.00
0.00	0.00	0.00	0.00	0.00	0.00	0.00	0.00	0.00
0.00	0.00	0.00	0.00	0.00	0.00	0.00	0.00	0.00
0.00	0.00	0.00	0.00	0.00	0.00	0.00	0.00	0.00
0.00	0.00	0.00	0.00	0.00	0.00	0.00	0.00	0.00
0.00	0.00	0.00	0.00	0.00	0.00	0.00	0.00	0.00
0.00	0.00	0.00	0.00	0.00	0.00	0.00	0.00	0.00
0.00	0.00	0.00	0.00	0.00	0.00	0.00	0.00	0.00
0.00	0.00	0.00	0.00	0.00	0.00	0.00	0.00	0.00
0.00	0.00	0.00	0.00	0.00	0.00	0.00	0.00	0.00
0.00	0.00	0.00	0.00	0.00	0.00	0.00	0.00	0.00
0.00	0.00	0.00	0.00	0.00	0.00	0.00	0.00	0.00
0.00	0.00	0.00	0.00	0.00	0.00	0.00	0.00	0.00
0.00	0.00	0.00	0.00	0.00	0.00	0.00	0.00	0.00
0.00	0.00	0.00	0.00	0.00	0.00	0.00	0.00	0.00
0.00	0.00	0.00	0.00	0.00	0.00	0.00	0.00	0.00
0.00	0.00	0.00	0.00	0.00	0.00	0.00	0.00	0.00
0.00	0.00	0.00	0.00	0.00	0.00	0.00	0.00	0.00

0.00	0.00	0.00	0.00	0.00	0.00	0.00	0.00	0.00
0.00	0.00	0.00	0.00	0.00	0.00	0.00	0.00	0.00
0.00	0.00	0.00	0.00	0.00	0.00	0.00	0.00	0.00
0.00	0.00	0.00	0.00	0.00	0.00	0.00	0.00	0.00
0.00	0.00	0.00	0.00	0.00	0.00	0.00	0.00	0.00
0.00	0.00	0.00	0.00	0.00	0.00	0.00	0.00	0.00
0.00	0.00	0.00	0.00	0.00	0.00	0.00	0.00	0.00
0.00	0.00	0.00	0.00	0.00	0.00	0.00	0.00	0.00
0.00	0.00	0.00	0.00	0.00	0.00	0.00	0.00	0.00
0.00	0.00	0.00	0.00	0.00	0.00	0.00	0.00	0.00
0.00	0.00	0.00	0.00	0.00	0.00	0.00	0.00	0.00
0.00	0.00	0.00	0.00	0.00	0.00	0.00	0.00	0.00
0.00	0.00	0.00	0.00	0.00	0.00	0.00	0.00	0.00
0.00	0.00	0.00	0.00	0.00	0.00	0.00	0.00	0.00
0.00	0.00	0.00	0.00	0.00	0.00	0.00	0.00	0.00
0.00	0.00	0.00	0.00	0.00	0.00	0.00	0.00	0.00
0.00	0.00	0.00	0.00	0.00	0.00	0.00	0.00	0.00
0.00	0.00	0.00	0.00	0.00	0.00	0.00	0.00	0.00
0.00	0.00	0.00	0.00	0.00	0.00	0.00	0.00	0.00
0.00	0.00	0.00	0.00	0.00	0.00	0.00	0.00	0.00
0.00	0.00	0.00	0.00	0.00	0.00	0.00	0.00	0.00
4.00	0.00	0.00	0.00	0.00	0.00	0.00	0.00	0.00
2.00	0.00	0.00	0.00	0.00	0.00	0.00	0.00	0.00
0.00	0.00	0.00	0.00	0.00	0.00	0.00	0.00	0.00
0.00	0.00	0.00	0.00	0.00	0.00	0.00	0.00	0.00
0.00	0.00	0.00	0.00	0.00	0.00	0.00	0.00	0.00
0.00	0.00	0.00	0.00	0.00	0.00	0.00	0.00	0.00
0.00	0.00	0.00	0.00	0.00	0.00	0.00	0.00	0.00
0.00	0.00	0.00	0.00	0.00	0.00	0.00	0.00	0.00
0.00	0.00	0.00	0.00	0.00	0.00	0.00	0.00	0.00
0.00	0.00	0.00	0.00	0.00	0.00	0.00	0.00	0.00
0.00	0.00	0.00	0.00	0.00	0.00	0.00	0.00	0.00
0.00	0.00	0.00	0.00	0.00	0.00	0.00	0.00	0.00
0.00	0.00	0.00	0.00	0.00	0.00	0.00	0.00	0.00
0.00	0.00	0.00	0.00	0.00	0.00	0.00	0.00	0.00
0.00	0.00	0.00	0.00	0.00	0.00	0.00	0.00	0.00
0.00	0.00	0.00	0.00	0.00	0.00	0.00	0.00	0.00

0.00	0.00	0.00	0.00	0.00	0.00	0.00	0.00	0.00
0.00	0.00	0.00	0.00	0.00	0.00	0.00	0.00	0.00
0.00	0.00	0.00	0.00	0.00	0.00	0.00	0.00	0.00
0.00	0.00	0.00	0.00	0.00	0.00	0.00	0.00	0.00
0.00	0.00	0.00	0.00	0.00	0.00	0.00	0.00	0.00
0.00	0.00	0.00	0.00	0.00	0.00	0.00	0.00	0.00
0.00	0.00	0.00	0.00	0.00	0.00	0.00	0.00	0.00
0.00	0.00	0.00	0.00	0.00	0.00	0.00	0.00	0.00
0.00	0.00	0.00	0.00	0.00	0.00	0.00	0.00	0.00
0.00	0.00	0.00	0.00	0.00	0.00	0.00	0.00	0.00
0.00	0.00	0.00	0.00	0.00	0.00	0.00	0.00	0.00
0.00	0.00	0.00	0.00	0.00	0.00	0.00	0.00	0.00
0.00	0.00	0.00	0.00	0.00	0.00	0.00	0.00	0.00
0.00	0.00	0.00	0.00	0.00	0.00	0.00	0.00	0.00
0.00	0.00	0.00	0.00	0.00	4.00	0.00	0.00	0.00
0.00	0.00	0.00	0.00	0.00	4.00	0.00	0.00	0.00
0.00	0.00	0.00	0.00	4.00	0.00	0.00	0.00	0.00
0.00	0.00	0.00	4.00	0.00	0.00	0.00	0.00	0.00
0.00	0.00	0.00	0.00	0.00	0.00	0.00	0.00	0.00
0.00	0.00	0.00	0.00	0.00	0.00	0.00	0.00	0.00
0.00	0.00	0.00	0.00	0.00	0.00	0.00	0.00	0.00
0.00	0.00	0.00	0.00	0.00	0.00	0.00	0.00	0.00
0.00	0.00	0.00	0.00	0.00	0.00	0.00	0.00	0.00
0.00	0.00	0.00	0.00	0.00	0.00	0.00	0.00	0.00
0.00	0.00	0.00	0.00	0.00	0.00	0.00	0.00	0.00
0.00	0.00	0.00	0.00	0.00	0.00	0.00	0.00	0.00
0.00	0.00	0.00	0.00	0.00	0.00	0.00	0.00	0.00
0.00	0.00	0.00	0.00	0.00	0.00	0.00	0.00	0.00
0.00	0.00	0.00	0.00	0.00	0.00	0.00	0.00	0.00
0.00	0.00	0.00	0.00	0.00	0.00	0.00	0.00	0.00
0.00	0.00	0.00	0.00	0.00	0.00	0.00	0.00	0.00
0.00	0.00	0.00	0.00	0.00	0.00	0.00	0.00	0.00
0.00	0.00	0.00	0.00	0.00	0.00	0.00	0.00	0.00
0.00	0.00	0.00	0.00	0.00	0.00	0.00	0.00	0.00
0.00	0.00	0.00	0.00	0.00	0.00	0.00	0.00	0.00
0.00	0.00	0.00	0.00	0.00	0.00	0.00	0.00	0.00
0.00	0.00	0.00	0.00	0.00	0.00	0.00	0.00	0.00

0.00	0.00	0.00	0.00	0.00	0.00	0.00	0.00	0.00
0.00	0.00	0.00	0.00	0.00	0.00	0.00	0.00	0.00
0.00	0.00	0.00	0.00	0.00	0.00	0.00	0.00	0.00
0.00	0.00	0.00	0.00	0.00	0.00	0.00	0.00	0.00
0.00	0.00	0.00	0.00	0.00	0.00	0.00	0.00	0.00
0.00	0.00	0.00	0.00	0.00	0.00	0.00	0.00	0.00
0.00	0.00	0.00	0.00	0.00	0.00	0.00	0.00	0.00
0.00	0.00	0.00	0.00	0.00	0.00	0.00	0.00	0.00
0.00	0.00	0.00	0.00	0.00	0.00	0.00	0.00	0.00
0.00	0.00	0.00	0.00	0.00	0.00	0.00	0.00	0.00
0.00	0.00	0.00	0.00	0.00	0.00	0.00	0.00	0.00
0.00	0.00	0.00	0.00	0.00	0.00	0.00	0.00	0.00
0.00	0.00	0.00	0.00	0.00	0.00	0.00	0.00	0.00
0.00	0.00	0.00	0.00	0.00	0.00	0.00	0.00	0.00
0.00	0.00	0.00	0.00	0.00	0.00	0.00	0.00	0.00
0.00	0.00	0.00	0.00	0.00	0.00	0.00	0.00	0.00
0.00	0.00	0.00	0.00	0.00	0.00	0.00	0.00	0.00
0.00	0.00	0.00	0.00	0.00	0.00	0.00	0.00	4.00
0.00	0.00	0.00	0.00	0.00	0.00	4.00	0.00	0.00
0.00	0.00	0.00	0.00	0.00	0.00	4.00	0.00	0.00
0.00	0.00	0.00	0.00	0.00	0.00	4.00	0.00	0.00
0.00	0.00	0.00	0.00	0.00	0.00	4.00	0.00	0.00
0.00	0.00	0.00	0.00	0.00	0.00	0.00	0.00	0.00
0.00	0.00	0.00	0.00	0.00	0.00	0.00	0.00	0.00
0.00	0.00	0.00	0.00	0.00	0.00	0.00	0.00	0.00
0.00	0.00	0.00	0.00	0.00	0.00	0.00	0.00	0.00
0.00	0.00	0.00	0.00	0.00	0.00	0.00	0.00	0.00
0.00	0.00	0.00	0.00	0.00	0.00	0.00	0.00	0.00
0.00	0.00	0.00	0.00	0.00	0.00	0.00	0.00	0.00
0.00	0.00	0.00	0.00	0.00	0.00	0.00	0.00	0.00
0.00	0.00	0.00	0.00	0.00	0.00	0.00	0.00	0.00
0.00	0.00	0.00	0.00	0.00	0.00	0.00	0.00	0.00
0.00	0.00	0.00	0.00	0.00	0.00	0.00	0.00	0.00
0.00	0.00	0.00	0.00	0.00	0.00	0.00	0.00	0.00
0.00	0.00	0.00	0.00	0.00	0.00	0.00	0.00	0.00
0.00	0.00	0.00	0.00	0.00	0.00	0.00	0.00	0.00
0.00	0.00	0.00	0.00	0.00	0.00	0.00	0.00	0.00

0.00	0.00	0.00	0.00	0.00	0.00	0.00	0.00	0.00
0.00	0.00	0.00	0.00	0.00	0.00	0.00	0.00	0.00
0.00	0.00	0.00	0.00	0.00	0.00	0.00	0.00	0.00
0.00	0.00	0.00	0.00	0.00	0.00	0.00	0.00	0.00
0.00	0.00	0.00	0.00	0.00	0.00	0.00	0.00	0.00
0.00	0.00	0.00	0.00	0.00	0.00	0.00	0.00	0.00
0.00	0.00	0.00	0.00	0.00	0.00	0.00	0.00	0.00
0.00	0.00	0.00	0.00	0.00	0.00	0.00	0.00	0.00
0.00	0.00	0.00	0.00	0.00	0.00	0.00	0.00	0.00
0.00	0.00	0.00	0.00	0.00	0.00	0.00	0.00	0.00
0.00	0.00	0.00	0.00	0.00	0.00	0.00	0.00	0.00
0.00	0.00	0.00	0.00	0.00	0.00	0.00	0.00	0.00
0.00	0.00	0.00	0.00	0.00	0.00	0.00	0.00	0.00
0.00	0.00	0.00	0.00	0.00	0.00	0.00	0.00	0.00
0.00	0.00	0.00	0.00	0.00	0.00	0.00	0.00	0.00
0.00	0.00	0.00	0.00	0.00	0.00	0.00	0.00	0.00
0.00	0.00	0.00	0.00	0.00	0.00	0.00	0.00	0.00
0.00	0.00	0.00	0.00	0.00	0.00	0.00	0.00	0.00
0.00	0.00	0.00	0.00	0.00	0.00	0.00	0.00	0.00
0.00	0.00	0.00	0.00	0.00	0.00	0.00	0.00	0.00
0.00	0.00	0.00	0.00	0.00	0.00	0.00	0.00	0.00
0.00	0.00	0.00	0.00	0.00	0.00	0.00	0.00	0.00
0.00	0.00	0.00	0.00	0.00	0.00	0.00	0.00	0.00
0.00	0.00	0.00	0.00	0.00	0.00	0.00	0.00	0.00
0.00	0.00	0.00	0.00	0.00	0.00	0.00	0.00	0.00
0.00	0.00	0.00	0.00	0.00	0.00	0.00	0.00	0.00
0.00	0.00	0.00	0.00	0.00	0.00	0.00	0.00	0.00
0.00	0.00	0.00	0.00	0.00	0.00	0.00	0.00	0.00
0.00	0.00	0.00	0.00	0.00	0.00	0.00	0.00	0.00
0.00	0.00	0.00	0.00	0.00	0.00	0.00	0.00	0.00
0.00	0.00	0.00	0.00	0.00	0.00	0.00	0.00	0.00
0.00	0.00	0.00	0.00	0.00	0.00	0.00	0.00	0.00
0.00	0.00	0.00	0.00	0.00	0.00	0.00	0.00	0.00
0.00	0.00	0.00	0.00	0.00	0.00	0.00	0.00	0.00
0.00	0.00	0.00	0.00	0.00	0.00	0.00	0.00	0.00
0.00	0.00	0.00	0.00	0.00	0.00	0.00	0.00	0.00
0.00	0.00	0.00	0.00	0.00	0.00	0.00	0.00	0.00

0.00	0.00	0.00	0.00	0.00	0.00	0.00	0.00	0.00
0.00	0.00	0.00	0.00	0.00	0.00	0.00	0.00	0.00
0.00	0.00	0.00	0.00	0.00	0.00	0.00	0.00	0.00
0.00	0.00	0.00	0.00	0.00	0.00	0.00	0.00	0.00
0.00	0.00	0.00	0.00	0.00	0.00	0.00	0.00	0.00
0.00	0.00	0.00	0.00	0.00	0.00	0.00	0.00	0.00
0.00	0.00	0.00	0.00	0.00	0.00	0.00	0.00	0.00
0.00	0.00	0.00	0.00	0.00	0.00	0.00	0.00	0.00
0.00	0.00	0.00	0.00	0.00	0.00	0.00	0.00	0.00
0.00	0.00	0.00	0.00	0.00	0.00	0.00	0.00	0.00
0.00	0.00	0.00	0.00	0.00	0.00	0.00	0.00	0.00
3.00	0.00	0.00	0.00	0.00	0.00	0.00	0.00	0.00
3.00	0.00	0.00	0.00	0.00	0.00	0.00	0.00	0.00
3.00	0.00	0.00	0.00	0.00	0.00	0.00	0.00	0.00
0.00	0.00	0.00	0.00	0.00	0.00	0.00	0.00	0.00
0.00	0.00	0.00	0.00	0.00	0.00	0.00	0.00	0.00
0.00	0.00	0.00	0.00	0.00	0.00	0.00	0.00	0.00
0.00	0.00	0.00	0.00	0.00	0.00	0.00	0.00	0.00
0.00	0.00	0.00	0.00	0.00	0.00	0.00	0.00	0.00
0.00	0.00	0.00	0.00	0.00	0.00	0.00	0.00	0.00
0.00	0.00	0.00	0.00	0.00	0.00	0.00	0.00	0.00
0.00	0.00	0.00	0.00	0.00	0.00	0.00	0.00	0.00
0.00	0.00	0.00	0.00	0.00	0.00	0.00	0.00	0.00
0.00	0.00	0.00	0.00	0.00	0.00	0.00	0.00	0.00
0.00	0.00	0.00	0.00	0.00	0.00	0.00	0.00	0.00
0.00	0.00	0.00	0.00	0.00	0.00	0.00	0.00	0.00
0.00	0.00	0.00	0.00	0.00	0.00	0.00	0.00	0.00
0.00	0.00	0.00	0.00	0.00	0.00	0.00	0.00	0.00
0.00	0.00	0.00	0.00	0.00	0.00	0.00	0.00	0.00
0.00	0.00	0.00	0.00	0.00	0.00	0.00	0.00	0.00
0.00	0.00	0.00	0.00	0.00	0.00	0.00	0.00	0.00
0.00	0.00	0.00	0.00	0.00	0.00	0.00	0.00	0.00
0.00	0.00	0.00	0.00	0.00	0.00	0.00	0.00	0.00
0.00	0.00	0.00	0.00	0.00	0.00	0.00	0.00	0.00
0.00	0.00	0.00	0.00	0.00	0.00	0.00	0.00	0.00
0.00	0.00	0.00	0.00	0.00	0.00	0.00	0.00	0.00
0.00	0.00	0.00	0.00	0.00	0.00	0.00	0.00	0.00

0.00	0.00	0.00	0.00	0.00	0.00	0.00	0.00	0.00
0.00	0.00	0.00	0.00	0.00	0.00	0.00	0.00	0.00
0.00	0.00	0.00	0.00	0.00	0.00	0.00	0.00	0.00
0.00	0.00	0.00	0.00	0.00	0.00	0.00	0.00	0.00
0.00	0.00	0.00	0.00	0.00	0.00	0.00	0.00	0.00
0.00	0.00	0.00	0.00	0.00	0.00	0.00	0.00	0.00
0.00	0.00	0.00	0.00	0.00	3.00	0.00	0.00	0.00
0.00	0.00	0.00	0.00	0.00	3.00	0.00	0.00	0.00
0.00	0.00	0.00	0.00	0.00	0.00	0.00	0.00	0.00
0.00	0.00	0.00	0.00	0.00	0.00	0.00	0.00	0.00
0.00	0.00	0.00	0.00	0.00	0.00	0.00	0.00	0.00
0.00	0.00	0.00	0.00	0.00	0.00	0.00	0.00	0.00
0.00	0.00	0.00	0.00	0.00	0.00	0.00	0.00	0.00
0.00	0.00	0.00	0.00	0.00	0.00	0.00	0.00	0.00
0.00	0.00	0.00	0.00	0.00	0.00	0.00	0.00	0.00
0.00	0.00	0.00	0.00	0.00	0.00	0.00	0.00	0.00
0.00	0.00	0.00	0.00	0.00	0.00	0.00	0.00	0.00
0.00	0.00	0.00	0.00	0.00	0.00	0.00	0.00	0.00
0.00	0.00	0.00	0.00	0.00	0.00	0.00	0.00	0.00
0.00	0.00	0.00	0.00	0.00	0.00	0.00	0.00	0.00
0.00	0.00	0.00	0.00	0.00	0.00	0.00	0.00	0.00
0.00	0.00	0.00	0.00	0.00	0.00	0.00	0.00	0.00
0.00	0.00	0.00	0.00	0.00	0.00	0.00	0.00	0.00
0.00	0.00	0.00	0.00	0.00	0.00	0.00	0.00	0.00
0.00	0.00	0.00	0.00	0.00	0.00	0.00	0.00	0.00
0.00	0.00	0.00	0.00	0.00	0.00	0.00	0.00	0.00
0.00	0.00	0.00	0.00	0.00	0.00	0.00	0.00	0.00
0.00	0.00	0.00	0.00	0.00	0.00	0.00	0.00	0.00
0.00	0.00	0.00	0.00	0.00	0.00	0.00	0.00	0.00
0.00	0.00	0.00	0.00	0.00	0.00	0.00	0.00	0.00
0.00	0.00	0.00	0.00	0.00	0.00	0.00	0.00	0.00
0.00	0.00	0.00	0.00	0.00	0.00	0.00	0.00	0.00
0.00	0.00	0.00	0.00	0.00	0.00	0.00	0.00	0.00
0.00	0.00	0.00	0.00	0.00	0.00	0.00	0.00	0.00
0.00	0.00	0.00	0.00	0.00	0.00	0.00	0.00	0.00
0.00	0.00	0.00	0.00	0.00	0.00	0.00	0.00	0.00
0.00	0.00	0.00	0.00	0.00	0.00	0.00	0.00	0.00

0.00	0.00	0.00	0.00	0.00	0.00	0.00	0.00	0.00
0.00	0.00	0.00	0.00	0.00	0.00	0.00	0.00	0.00
0.00	0.00	0.00	0.00	0.00	0.00	0.00	0.00	0.00
0.00	0.00	0.00	0.00	0.00	0.00	0.00	0.00	0.00
0.00	0.00	0.00	0.00	0.00	0.00	0.00	0.00	0.00
0.00	0.00	0.00	0.00	0.00	0.00	0.00	0.00	0.00
0.00	0.00	0.00	0.00	0.00	0.00	0.00	0.00	0.00
0.00	0.00	0.00	0.00	0.00	0.00	0.00	0.00	0.00
0.00	0.00	0.00	0.00	0.00	0.00	0.00	0.00	0.00
0.00	0.00	0.00	0.00	0.00	0.00	0.00	0.00	0.00
0.00	0.00	0.00	0.00	0.00	0.00	0.00	0.00	0.00
0.00	0.00	0.00	0.00	0.00	0.00	0.00	0.00	0.00
0.00	0.00	0.00	0.00	0.00	0.00	3.00	0.00	0.00
0.00	0.00	0.00	0.00	0.00	0.00	3.00	0.00	0.00
0.00	3.00	0.00	0.00	0.00	0.00	0.00	0.00	0.00
0.00	0.00	0.00	0.00	0.00	0.00	0.00	0.00	0.00
0.00	0.00	0.00	0.00	0.00	0.00	0.00	0.00	0.00
0.00	0.00	0.00	0.00	0.00	0.00	0.00	0.00	0.00
0.00	0.00	0.00	0.00	0.00	0.00	0.00	0.00	0.00
0.00	0.00	0.00	0.00	0.00	0.00	0.00	0.00	0.00
0.00	0.00	0.00	0.00	0.00	0.00	0.00	0.00	0.00
0.00	0.00	0.00	0.00	0.00	0.00	0.00	0.00	0.00
0.00	0.00	0.00	0.00	0.00	0.00	0.00	0.00	0.00
0.00	0.00	0.00	0.00	0.00	0.00	0.00	0.00	0.00
0.00	0.00	0.00	0.00	0.00	0.00	0.00	0.00	0.00
0.00	0.00	0.00	0.00	0.00	0.00	0.00	0.00	0.00
0.00	0.00	0.00	0.00	0.00	0.00	0.00	0.00	0.00
0.00	0.00	0.00	0.00	0.00	0.00	0.00	0.00	0.00
0.00	0.00	0.00	0.00	0.00	0.00	0.00	0.00	0.00
0.00	0.00	0.00	0.00	0.00	0.00	0.00	0.00	0.00
0.00	0.00	0.00	0.00	0.00	0.00	0.00	0.00	0.00
0.00	0.00	0.00	0.00	0.00	0.00	0.00	0.00	0.00
0.00	0.00	0.00	0.00	0.00	0.00	0.00	0.00	0.00
0.00	0.00	0.00	0.00	0.00	0.00	0.00	0.00	0.00
0.00	0.00	0.00	0.00	0.00	0.00	0.00	0.00	0.00
0.00	0.00	0.00	0.00	0.00	0.00	0.00	0.00	0.00
0.00	0.00	0.00	0.00	0.00	0.00	0.00	0.00	0.00

0.00	0.00	0.00	0.00	0.00	0.00	0.00	0.00	0.00
0.00	0.00	0.00	0.00	0.00	0.00	0.00	0.00	0.00
0.00	0.00	0.00	0.00	0.00	0.00	0.00	0.00	0.00
0.00	0.00	0.00	0.00	0.00	0.00	0.00	0.00	0.00
0.00	0.00	0.00	0.00	0.00	0.00	0.00	0.00	0.00
0.00	0.00	0.00	0.00	0.00	0.00	0.00	0.00	0.00
0.00	0.00	0.00	0.00	0.00	0.00	0.00	0.00	0.00
0.00	0.00	0.00	0.00	0.00	0.00	0.00	0.00	0.00
0.00	0.00	0.00	0.00	0.00	0.00	0.00	0.00	0.00
0.00	0.00	0.00	0.00	0.00	0.00	0.00	0.00	0.00
0.00	0.00	0.00	0.00	0.00	0.00	0.00	0.00	0.00
0.00	0.00	0.00	0.00	0.00	0.00	0.00	0.00	0.00
0.00	0.00	0.00	0.00	0.00	0.00	0.00	0.00	0.00
0.00	0.00	0.00	0.00	0.00	0.00	0.00	0.00	0.00
0.00	0.00	0.00	0.00	0.00	0.00	0.00	0.00	0.00
0.00	0.00	0.00	0.00	0.00	0.00	0.00	0.00	0.00
0.00	0.00	0.00	0.00	0.00	0.00	0.00	0.00	0.00
0.00	0.00	0.00	0.00	0.00	0.00	0.00	0.00	0.00
0.00	0.00	0.00	0.00	0.00	0.00	0.00	0.00	0.00
0.00	0.00	0.00	0.00	0.00	0.00	0.00	0.00	0.00
0.00	0.00	0.00	0.00	0.00	0.00	0.00	0.00	0.00
0.00	0.00	0.00	0.00	0.00	0.00	0.00	0.00	0.00
0.00	0.00	0.00	0.00	0.00	0.00	0.00	0.00	0.00
0.00	0.00	0.00	0.00	0.00	0.00	0.00	0.00	0.00
0.00	0.00	0.00	0.00	0.00	0.00	0.00	0.00	0.00
0.00	0.00	0.00	0.00	0.00	0.00	0.00	0.00	0.00
0.00	0.00	0.00	0.00	0.00	0.00	0.00	0.00	0.00
0.00	0.00	0.00	0.00	0.00	0.00	0.00	0.00	0.00
0.00	0.00	0.00	0.00	0.00	0.00	0.00	0.00	0.00
0.00	0.00	0.00	0.00	0.00	0.00	0.00	0.00	0.00
0.00	0.00	0.00	0.00	0.00	0.00	0.00	0.00	0.00
0.00	0.00	0.00	0.00	0.00	0.00	0.00	0.00	0.00
0.00	0.00	0.00	0.00	0.00	0.00	0.00	0.00	0.00
0.00	0.00	0.00	0.00	0.00	0.00	0.00	0.00	0.00
0.00	0.00	0.00	0.00	0.00	0.00	0.00	0.00	0.00
0.00	0.00	0.00	0.00	0.00	0.00	0.00	0.00	0.00
0.00	0.00	0.00	0.00	0.00	0.00	0.00	0.00	0.00

0.00	0.00	0.00	0.00	0.00	0.00	0.00	0.00	0.00
0.00	0.00	0.00	0.00	0.00	0.00	0.00	0.00	0.00
0.00	0.00	0.00	0.00	0.00	0.00	0.00	0.00	0.00
0.00	0.00	0.00	0.00	0.00	0.00	0.00	0.00	0.00
0.00	0.00	0.00	0.00	0.00	0.00	0.00	0.00	0.00
0.00	0.00	0.00	0.00	0.00	0.00	0.00	0.00	0.00
0.00	0.00	0.00	0.00	0.00	0.00	0.00	0.00	0.00
0.00	0.00	0.00	0.00	0.00	0.00	0.00	0.00	0.00
0.00	0.00	0.00	0.00	0.00	0.00	0.00	0.00	0.00
0.00	0.00	0.00	0.00	0.00	0.00	0.00	0.00	0.00
0.00	0.00	0.00	0.00	0.00	0.00	0.00	0.00	0.00
0.00	0.00	0.00	0.00	0.00	0.00	0.00	0.00	0.00
0.00	0.00	2.00	0.00	0.00	0.00	0.00	0.00	0.00
0.00	0.00	2.00	0.00	0.00	0.00	0.00	0.00	0.00
2.00	0.00	0.00	0.00	0.00	0.00	0.00	0.00	0.00
2.00	0.00	0.00	0.00	0.00	0.00	0.00	0.00	0.00
2.00	0.00	0.00	0.00	0.00	0.00	0.00	0.00	0.00
0.00	0.00	0.00	0.00	0.00	0.00	0.00	0.00	0.00
0.00	0.00	0.00	0.00	0.00	0.00	0.00	0.00	0.00
0.00	0.00	0.00	0.00	0.00	0.00	0.00	0.00	0.00
0.00	0.00	0.00	0.00	0.00	0.00	0.00	0.00	0.00
0.00	0.00	0.00	0.00	0.00	0.00	0.00	0.00	0.00
0.00	0.00	0.00	0.00	0.00	0.00	0.00	0.00	0.00
0.00	0.00	0.00	0.00	0.00	0.00	0.00	0.00	0.00
0.00	0.00	0.00	0.00	0.00	0.00	0.00	0.00	0.00
0.00	0.00	0.00	0.00	0.00	0.00	0.00	0.00	0.00
0.00	0.00	0.00	0.00	0.00	0.00	0.00	0.00	0.00
0.00	0.00	0.00	0.00	0.00	0.00	0.00	0.00	0.00
0.00	0.00	0.00	0.00	0.00	0.00	0.00	0.00	0.00
0.00	0.00	0.00	0.00	0.00	0.00	0.00	0.00	0.00
0.00	0.00	0.00	0.00	0.00	0.00	0.00	0.00	0.00
0.00	0.00	0.00	0.00	0.00	0.00	0.00	0.00	0.00
0.00	0.00	0.00	0.00	0.00	0.00	0.00	0.00	0.00
0.00	0.00	0.00	0.00	0.00	0.00	0.00	0.00	0.00
0.00	0.00	0.00	0.00	0.00	0.00	0.00	0.00	0.00
0.00	0.00	0.00	0.00	0.00	0.00	0.00	0.00	0.00
0.00	0.00	0.00	0.00	0.00	0.00	0.00	0.00	0.00

0.00	0.00	0.00	0.00	0.00	0.00	0.00	0.00	0.00
0.00	0.00	0.00	0.00	0.00	0.00	0.00	0.00	0.00
0.00	0.00	0.00	0.00	0.00	0.00	0.00	0.00	0.00
0.00	0.00	0.00	0.00	0.00	0.00	0.00	0.00	0.00
0.00	0.00	0.00	0.00	0.00	0.00	0.00	0.00	0.00
0.00	0.00	0.00	0.00	0.00	0.00	0.00	0.00	0.00
0.00	0.00	0.00	0.00	0.00	0.00	0.00	0.00	0.00
0.00	0.00	0.00	0.00	0.00	0.00	0.00	0.00	0.00
0.00	0.00	0.00	0.00	0.00	0.00	0.00	0.00	0.00
0.00	0.00	0.00	0.00	0.00	0.00	0.00	0.00	0.00
0.00	0.00	0.00	0.00	0.00	0.00	0.00	0.00	0.00
0.00	0.00	0.00	0.00	0.00	0.00	0.00	0.00	0.00
0.00	0.00	0.00	0.00	0.00	0.00	0.00	0.00	0.00
0.00	0.00	0.00	0.00	0.00	0.00	0.00	0.00	0.00
0.00	0.00	0.00	0.00	0.00	0.00	0.00	0.00	0.00
0.00	0.00	0.00	0.00	0.00	0.00	0.00	0.00	0.00
0.00	0.00	0.00	0.00	0.00	0.00	0.00	0.00	0.00
0.00	0.00	0.00	0.00	0.00	0.00	0.00	0.00	0.00
0.00	0.00	0.00	0.00	0.00	0.00	0.00	0.00	0.00
0.00	0.00	0.00	0.00	0.00	0.00	0.00	0.00	0.00
0.00	0.00	0.00	0.00	0.00	0.00	0.00	0.00	0.00
0.00	0.00	0.00	0.00	0.00	0.00	0.00	0.00	0.00
0.00	0.00	0.00	0.00	0.00	0.00	0.00	0.00	0.00
0.00	0.00	0.00	0.00	0.00	0.00	0.00	0.00	0.00
0.00	0.00	0.00	0.00	0.00	0.00	0.00	0.00	0.00
0.00	0.00	0.00	0.00	0.00	2.00	0.00	0.00	0.00
0.00	0.00	0.00	0.00	0.00	2.00	0.00	0.00	0.00
0.00	0.00	0.00	0.00	2.00	0.00	0.00	0.00	0.00
0.00	0.00	0.00	2.00	0.00	0.00	0.00	0.00	0.00
0.00	0.00	0.00	0.00	0.00	0.00	0.00	0.00	0.00
0.00	0.00	0.00	0.00	0.00	0.00	0.00	0.00	0.00
0.00	0.00	0.00	0.00	0.00	0.00	0.00	0.00	0.00
0.00	0.00	0.00	0.00	0.00	0.00	0.00	0.00	0.00
0.00	0.00	0.00	0.00	0.00	0.00	0.00	0.00	0.00
0.00	0.00	0.00	0.00	0.00	0.00	0.00	0.00	0.00
0.00	0.00	0.00	0.00	0.00	0.00	0.00	0.00	0.00
0.00	0.00	0.00	0.00	0.00	0.00	0.00	0.00	0.00

0.00	0.00	0.00	0.00	0.00	0.00	0.00	0.00	0.00
0.00	0.00	0.00	0.00	0.00	0.00	0.00	0.00	0.00
0.00	0.00	0.00	0.00	0.00	0.00	0.00	0.00	0.00
0.00	0.00	0.00	0.00	0.00	0.00	0.00	0.00	0.00
0.00	0.00	0.00	0.00	0.00	0.00	0.00	0.00	0.00
0.00	0.00	0.00	0.00	0.00	0.00	0.00	0.00	0.00
0.00	0.00	0.00	0.00	0.00	0.00	0.00	0.00	0.00
0.00	0.00	0.00	0.00	0.00	0.00	0.00	0.00	0.00
0.00	0.00	0.00	0.00	0.00	0.00	0.00	0.00	0.00
0.00	0.00	0.00	0.00	0.00	0.00	0.00	0.00	0.00
0.00	0.00	0.00	0.00	0.00	0.00	0.00	0.00	0.00
0.00	0.00	0.00	0.00	0.00	0.00	0.00	0.00	0.00
0.00	0.00	0.00	0.00	0.00	0.00	0.00	0.00	0.00
0.00	0.00	0.00	0.00	0.00	0.00	0.00	0.00	0.00
0.00	0.00	0.00	0.00	0.00	0.00	0.00	0.00	0.00
0.00	0.00	0.00	0.00	0.00	0.00	0.00	0.00	0.00
0.00	0.00	0.00	0.00	0.00	0.00	0.00	0.00	0.00
0.00	0.00	0.00	0.00	0.00	0.00	0.00	0.00	0.00
0.00	0.00	0.00	0.00	0.00	0.00	0.00	0.00	0.00
0.00	0.00	0.00	0.00	0.00	0.00	0.00	0.00	0.00
0.00	0.00	0.00	0.00	0.00	0.00	0.00	0.00	0.00
0.00	0.00	0.00	0.00	0.00	0.00	0.00	0.00	0.00
0.00	0.00	0.00	0.00	0.00	0.00	0.00	0.00	0.00
0.00	0.00	0.00	0.00	0.00	0.00	0.00	0.00	0.00
0.00	0.00	0.00	0.00	0.00	0.00	0.00	0.00	2.00
0.00	0.00	0.00	0.00	0.00	0.00	0.00	2.00	0.00
0.00	0.00	0.00	0.00	0.00	0.00	2.00	0.00	0.00
0.00	0.00	0.00	0.00	0.00	0.00	2.00	0.00	0.00
0.00	0.00	0.00	0.00	0.00	0.00	2.00	0.00	0.00
0.00	0.00	0.00	0.00	0.00	0.00	2.00	0.00	0.00
0.00	0.00	0.00	0.00	0.00	0.00	2.00	0.00	0.00
0.00	0.00	0.00	0.00	0.00	0.00	2.00	0.00	0.00
0.00	0.00	0.00	0.00	0.00	0.00	2.00	0.00	0.00

D5	D4	D3	C6	C5	C4	C3	D2	C2	
15097.00	0.00	199.00	12457.00	54975.00	805.00	61.00	472.00	22447.00	
0.00	0.00	42.00	0.00	0.00	0.00	0.00	0.00	28.00	
1133.00	33165.00	2979.00	2527.00	1763.00	12553.00	12197.00	34918.00	18491.00	
0.00	0.00	18.00	0.00	0.00	0.00	6.00	0.00	0.00	
0.00	0.00	0.00	0.00	0.00	0.00	0.00	0.00	0.00	
0.00	0.00	0.00	129.00	0.00	335.00	0.00	0.00	0.00	
56.00	0.00	29637.00	93.00	0.00	0.00	0.00	0.00	0.00	
0.00	0.00	0.00	0.00	0.00	0.00	0.00	0.00	0.00	
6029.00	0.00	41.00	16778.00	3017.00	45376.00	3101.00	837.00	73.00	
0.00	0.00	0.00	0.00	0.00	0.00	0.00	0.00	0.00	
0.00	0.00	20217.00	39.00	0.00	0.00	0.00	0.00	0.00	
223.00	0.00	0.00	0.00	63.00	16.00	0.00	0.00	55.00	
8922.00	0.00	16.00	15229.00	25.00	73.00	6.00	25.00	9.00	
39.00	0.00	0.00	12.00	5.00	7.00	0.00	4278.00	0.00	
0.00	0.00	0.00	0.00	0.00	0.00	0.00	0.00	0.00	
5.00	0.00	0.00	0.00	0.00	339.00	0.00	0.00	0.00	
0.00	0.00	10376.00	0.00	0.00	0.00	0.00	0.00	0.00	
0.00	0.00	0.00	0.00	0.00	0.00	0.00	0.00	0.00	
19973.00	10.00	51.00	113.00	40.00	189.00	2187.00	60.00	44.00	
0.00	0.00	0.00	0.00	545.00	673.00	0.00	41.00	244.00	
0.00	0.00	0.00	0.00	0.00	0.00	0.00	0.00	0.00	
0.00	0.00	0.00	0.00	0.00	0.00	0.00	8.00	0.00	
2.00	0.00	28.00	0.00	0.00	0.00	0.00	0.00	0.00	
0.00	0.00	0.00	0.00	0.00	0.00	0.00	0.00	0.00	
0.00	0.00	0.00	0.00	0.00	0.00	0.00	0.00	0.00	
67.00	536.00	86.00	106.00	68.00	654.00	1408.00	99.00	1828.00	
0.00	0.00	0.00	0.00	0.00	0.00	0.00	0.00	0.00	
9.00	0.00	27.00	20.00	0.00	3.00	0.00	0.00	0.00	
0.00	0.00	0.00	0.00	0.00	0.00	0.00	0.00	0.00	
0.00	0.00	14.00	0.00	0.00	0.00	0.00	0.00	0.00	
33.00	1538.00	39.00	151.00	48.00	335.00	367.00	0.00	647.00	
4548.00	0.00	0.00	0.00	0.00	0.00	0.00	0.00	0.00	
0.00	0.00	0.00	0.00	0.00	0.00	0.00	0.00	0.00	
4331.00	0.00	0.00	0.00	0.00	49.00	0.00	0.00	0.00	
0.00	0.00	0.00	0.00	0.00	0.00	0.00	0.00	0.00	

0.00	0.00	0.00	0.00	0.00	7.00	0.00	0.00	0.00
80.00	0.00	95.00	27.00	46.00	62.00	694.00	338.00	3381.00
33.00	96.00	37.00	48.00	0.00	12.00	1590.00	1184.00	0.00
0.00	0.00	0.00	0.00	0.00	0.00	0.00	0.00	0.00
0.00	0.00	0.00	6.00	0.00	6.00	0.00	0.00	0.00
0.00	0.00	0.00	0.00	0.00	0.00	0.00	0.00	0.00
0.00	0.00	0.00	0.00	0.00	0.00	0.00	0.00	0.00
0.00	392.00	52.00	57.00	15.00	366.00	294.00	1674.00	865.00
0.00	0.00	33.00	40.00	0.00	237.00	862.00	50.00	300.00
0.00	0.00	0.00	0.00	0.00	0.00	0.00	0.00	0.00
0.00	0.00	0.00	0.00	0.00	0.00	0.00	0.00	0.00
0.00	0.00	0.00	3.00	24.00	0.00	0.00	895.00	0.00
0.00	0.00	0.00	0.00	0.00	0.00	0.00	15.00	0.00
0.00	0.00	0.00	44.00	18.00	33.00	789.00	201.00	482.00
0.00	9.00	0.00	0.00	0.00	0.00	0.00	0.00	0.00
0.00	0.00	68.00	4.00	0.00	64.00	1502.00	259.00	656.00
0.00	0.00	0.00	0.00	0.00	0.00	0.00	0.00	0.00
0.00	0.00	0.00	0.00	0.00	0.00	0.00	0.00	0.00
0.00	0.00	0.00	0.00	0.00	0.00	0.00	0.00	0.00
0.00	0.00	0.00	0.00	0.00	0.00	0.00	0.00	0.00
0.00	0.00	17.00	8.00	0.00	0.00	1126.00	1626.00	0.00
0.00	0.00	0.00	0.00	0.00	0.00	0.00	0.00	0.00
0.00	0.00	0.00	0.00	0.00	0.00	0.00	0.00	0.00
12.00	0.00	15.00	0.00	0.00	0.00	0.00	29.00	0.00
0.00	0.00	39.00	27.00	71.00	4.00	102.00	10.00	412.00
0.00	0.00	0.00	0.00	0.00	0.00	0.00	0.00	0.00
11.00	0.00	11.00	9.00	0.00	10.00	321.00	723.00	680.00
0.00	0.00	0.00	0.00	0.00	0.00	0.00	0.00	0.00
0.00	0.00	0.00	0.00	0.00	0.00	0.00	0.00	0.00
0.00	0.00	0.00	0.00	0.00	0.00	0.00	0.00	0.00
0.00	0.00	0.00	37.00	0.00	9.00	53.00	0.00	0.00
0.00	0.00	0.00	0.00	0.00	0.00	0.00	0.00	0.00
0.00	0.00	0.00	0.00	0.00	8.00	0.00	12.00	0.00
0.00	0.00	0.00	0.00	0.00	0.00	0.00	0.00	0.00
0.00	0.00	0.00	0.00	0.00	0.00	0.00	0.00	0.00
0.00	0.00	3.00	9.00	5.00	9.00	3085.00	11.00	0.00
0.00	0.00	0.00	2891.00	0.00	0.00	0.00	3.00	0.00

0.00	0.00	0.00	0.00	0.00	0.00	3133.00	20.00	0.00
20.00	0.00	0.00	14.00	3.00	5.00	0.00	0.00	1977.00
0.00	0.00	0.00	0.00	0.00	0.00	0.00	0.00	0.00
0.00	0.00	0.00	0.00	0.00	0.00	2964.00	7.00	5.00
0.00	0.00	0.00	0.00	16.00	198.00	0.00	32.00	0.00
9.00	0.00	0.00	0.00	0.00	0.00	0.00	0.00	0.00
0.00	0.00	0.00	0.00	0.00	0.00	0.00	0.00	0.00
11.00	0.00	0.00	0.00	0.00	0.00	0.00	0.00	7.00
0.00	0.00	6.00	0.00	0.00	0.00	0.00	0.00	0.00
0.00	0.00	0.00	9.00	0.00	6.00	2600.00	0.00	0.00
0.00	0.00	0.00	0.00	0.00	0.00	0.00	9.00	0.00
0.00	0.00	0.00	0.00	0.00	0.00	0.00	0.00	0.00
30.00	368.00	83.00	0.00	0.00	0.00	102.00	0.00	586.00
9.00	0.00	4.00	0.00	13.00	0.00	540.00	596.00	0.00
0.00	0.00	0.00	0.00	7.00	0.00	2364.00	9.00	0.00
0.00	0.00	0.00	52.00	0.00	0.00	0.00	0.00	0.00
0.00	0.00	0.00	0.00	0.00	0.00	0.00	0.00	0.00
0.00	0.00	0.00	0.00	0.00	0.00	0.00	0.00	0.00
0.00	0.00	0.00	0.00	0.00	0.00	189.00	0.00	638.00
4.00	0.00	0.00	0.00	0.00	0.00	0.00	0.00	0.00
50.00	9.00	117.00	16.00	0.00	338.00	0.00	0.00	24.00
31.00	0.00	20.00	11.00	0.00	19.00	11.00	51.00	10.00
76.00	0.00	0.00	0.00	0.00	0.00	0.00	0.00	744.00
0.00	0.00	0.00	0.00	0.00	0.00	0.00	0.00	0.00
0.00	0.00	0.00	0.00	7.00	0.00	859.00	0.00	0.00
0.00	0.00	0.00	0.00	0.00	0.00	0.00	0.00	0.00
0.00	0.00	0.00	1820.00	0.00	0.00	0.00	0.00	0.00
0.00	0.00	0.00	19.00	0.00	0.00	6.00	0.00	106.00
0.00	0.00	81.00	104.00	0.00	203.00	0.00	0.00	0.00
0.00	0.00	0.00	0.00	0.00	0.00	0.00	0.00	0.00
0.00	0.00	0.00	0.00	0.00	0.00	0.00	0.00	0.00
0.00	0.00	7.00	7.00	0.00	0.00	0.00	0.00	0.00
4.00	0.00	3.00	26.00	0.00	0.00	0.00	11.00	0.00
73.00	0.00	51.00	82.00	58.00	46.00	30.00	93.00	24.00
0.00	0.00	0.00	0.00	0.00	0.00	0.00	0.00	0.00
4.00	0.00	0.00	15.00	0.00	0.00	0.00	0.00	0.00
0.00	0.00	0.00	22.00	15.00	31.00	1622.00	0.00	0.00

0.00	0.00	0.00	0.00	0.00	0.00	0.00	0.00	0.00
0.00	0.00	0.00	0.00	0.00	0.00	0.00	0.00	272.00
0.00	0.00	0.00	9.00	7.00	0.00	1588.00	0.00	0.00
0.00	0.00	0.00	0.00	0.00	0.00	0.00	0.00	0.00
22.00	27.00	0.00	0.00	0.00	0.00	0.00	0.00	0.00
15.00	0.00	0.00	0.00	0.00	0.00	0.00	0.00	0.00
0.00	0.00	0.00	0.00	0.00	0.00	0.00	9.00	0.00
0.00	0.00	0.00	0.00	32.00	0.00	0.00	0.00	0.00
0.00	0.00	118.00	84.00	24.00	0.00	0.00	0.00	0.00
0.00	0.00	0.00	0.00	0.00	0.00	0.00	1487.00	0.00
0.00	0.00	0.00	0.00	0.00	0.00	0.00	1466.00	0.00
0.00	0.00	0.00	0.00	0.00	0.00	0.00	0.00	0.00
2.00	0.00	0.00	0.00	0.00	0.00	0.00	0.00	0.00
88.00	0.00	62.00	0.00	0.00	95.00	27.00	75.00	24.00
0.00	0.00	0.00	0.00	0.00	0.00	0.00	0.00	0.00
0.00	0.00	0.00	0.00	0.00	0.00	0.00	0.00	0.00
0.00	0.00	0.00	0.00	0.00	0.00	0.00	1350.00	0.00
0.00	0.00	0.00	0.00	0.00	0.00	0.00	0.00	0.00
0.00	0.00	0.00	0.00	0.00	0.00	0.00	0.00	0.00
4.00	0.00	3.00	0.00	0.00	0.00	2.00	0.00	0.00
0.00	0.00	0.00	0.00	0.00	0.00	0.00	1284.00	0.00
0.00	0.00	0.00	0.00	0.00	0.00	0.00	0.00	0.00
0.00	0.00	0.00	0.00	22.00	0.00	0.00	0.00	0.00
0.00	0.00	0.00	0.00	0.00	0.00	0.00	0.00	0.00
0.00	0.00	0.00	0.00	0.00	0.00	0.00	0.00	0.00
0.00	0.00	0.00	0.00	0.00	0.00	0.00	0.00	0.00
0.00	0.00	0.00	1124.00	0.00	0.00	0.00	0.00	0.00
0.00	708.00	0.00	0.00	0.00	0.00	0.00	0.00	0.00
0.00	0.00	0.00	0.00	0.00	0.00	0.00	0.00	0.00
0.00	0.00	0.00	0.00	0.00	0.00	0.00	0.00	0.00
84.00	0.00	91.00	77.00	36.00	85.00	14.00	68.00	12.00
0.00	0.00	0.00	0.00	0.00	0.00	0.00	0.00	0.00
0.00	0.00	278.00	0.00	0.00	0.00	0.00	0.00	0.00
0.00	0.00	0.00	0.00	0.00	0.00	0.00	0.00	0.00
0.00	38.00	5.00	6.00	0.00	0.00	0.00	0.00	113.00
0.00	0.00	0.00	0.00	0.00	0.00	2.00	0.00	0.00
0.00	0.00	0.00	0.00	0.00	0.00	0.00	0.00	0.00

0.00	0.00	0.00	0.00	0.00	0.00	0.00	1047.00	0.00
82.00	0.00	129.00	0.00	0.00	0.00	0.00	0.00	0.00
27.00	0.00	33.00	21.00	0.00	23.00	3.00	23.00	9.00
0.00	0.00	0.00	0.00	0.00	0.00	0.00	0.00	15.00
4.00	0.00	0.00	0.00	0.00	0.00	9.00	0.00	0.00
0.00	0.00	32.00	0.00	0.00	0.00	0.00	388.00	0.00
3.00	0.00	0.00	0.00	0.00	0.00	0.00	0.00	0.00
0.00	0.00	0.00	0.00	0.00	0.00	0.00	0.00	0.00
0.00	0.00	0.00	0.00	0.00	0.00	0.00	0.00	0.00
4.00	0.00	7.00	0.00	0.00	0.00	0.00	5.00	0.00
0.00	0.00	0.00	0.00	0.00	0.00	0.00	0.00	0.00
0.00	0.00	0.00	0.00	0.00	0.00	0.00	0.00	0.00
0.00	0.00	0.00	0.00	0.00	0.00	0.00	889.00	0.00
0.00	0.00	0.00	0.00	19.00	0.00	0.00	0.00	0.00
0.00	0.00	0.00	0.00	0.00	2.00	0.00	0.00	0.00
5.00	0.00	0.00	4.00	0.00	0.00	0.00	0.00	0.00
9.00	0.00	25.00	0.00	0.00	0.00	0.00	0.00	0.00
34.00	0.00	65.00	67.00	45.00	78.00	0.00	51.00	22.00
0.00	0.00	0.00	0.00	0.00	0.00	0.00	0.00	0.00
0.00	0.00	0.00	0.00	0.00	0.00	0.00	0.00	0.00
0.00	0.00	0.00	653.00	0.00	0.00	0.00	0.00	0.00
0.00	0.00	0.00	0.00	0.00	0.00	0.00	828.00	0.00
0.00	0.00	2.00	0.00	0.00	0.00	0.00	0.00	0.00
0.00	0.00	0.00	0.00	0.00	0.00	0.00	0.00	0.00
0.00	0.00	0.00	0.00	0.00	0.00	0.00	0.00	0.00
0.00	0.00	76.00	0.00	0.00	0.00	0.00	0.00	0.00
54.00	0.00	86.00	71.00	28.00	66.00	8.00	30.00	10.00
0.00	0.00	10.00	0.00	0.00	0.00	293.00	0.00	0.00
0.00	25.00	0.00	0.00	0.00	0.00	0.00	748.00	0.00
0.00	0.00	0.00	0.00	0.00	0.00	0.00	761.00	0.00
0.00	0.00	0.00	0.00	0.00	0.00	0.00	0.00	0.00
10.00	0.00	15.00	20.00	0.00	38.00	4.00	25.00	0.00
0.00	754.00	0.00	0.00	0.00	0.00	0.00	0.00	0.00
0.00	0.00	0.00	0.00	0.00	0.00	0.00	0.00	0.00
0.00	0.00	0.00	0.00	0.00	0.00	0.00	0.00	0.00
26.00	0.00	52.00	84.00	49.00	92.00	16.00	66.00	16.00
22.00	0.00	0.00	0.00	0.00	57.00	0.00	0.00	0.00

0.00	0.00	0.00	0.00	0.00	0.00	0.00	0.00	0.00
0.00	0.00	0.00	0.00	0.00	0.00	0.00	0.00	0.00
0.00	0.00	0.00	0.00	0.00	0.00	0.00	0.00	0.00
0.00	0.00	0.00	0.00	0.00	0.00	0.00	0.00	0.00
0.00	0.00	0.00	0.00	0.00	0.00	0.00	0.00	0.00
30.00	0.00	0.00	0.00	0.00	0.00	69.00	0.00	0.00
0.00	0.00	0.00	0.00	0.00	0.00	0.00	0.00	0.00
0.00	0.00	0.00	0.00	0.00	0.00	11.00	0.00	0.00
0.00	0.00	0.00	0.00	0.00	0.00	0.00	0.00	0.00
0.00	0.00	53.00	78.00	0.00	75.00	14.00	35.00	14.00
0.00	0.00	0.00	0.00	0.00	0.00	0.00	0.00	0.00
0.00	0.00	0.00	0.00	0.00	0.00	0.00	0.00	0.00
0.00	0.00	0.00	58.00	0.00	48.00	0.00	0.00	0.00
0.00	0.00	0.00	0.00	0.00	0.00	0.00	0.00	0.00
0.00	0.00	0.00	0.00	0.00	0.00	0.00	0.00	0.00
0.00	0.00	0.00	0.00	0.00	0.00	0.00	0.00	0.00
0.00	0.00	0.00	0.00	0.00	0.00	0.00	0.00	0.00
0.00	0.00	0.00	7.00	0.00	0.00	0.00	0.00	0.00
0.00	0.00	0.00	0.00	0.00	0.00	0.00	0.00	0.00
0.00	0.00	0.00	0.00	0.00	0.00	0.00	0.00	0.00
0.00	0.00	5.00	0.00	0.00	0.00	0.00	0.00	0.00
0.00	0.00	0.00	0.00	0.00	0.00	0.00	0.00	0.00
0.00	0.00	0.00	0.00	0.00	0.00	0.00	0.00	0.00
0.00	0.00	0.00	0.00	0.00	0.00	0.00	0.00	0.00
0.00	0.00	0.00	0.00	0.00	0.00	0.00	0.00	0.00
0.00	0.00	0.00	0.00	0.00	0.00	0.00	0.00	0.00
0.00	0.00	0.00	0.00	0.00	4.00	571.00	0.00	0.00
0.00	0.00	0.00	10.00	0.00	0.00	0.00	0.00	0.00
0.00	0.00	0.00	0.00	0.00	0.00	0.00	0.00	0.00
0.00	0.00	0.00	0.00	0.00	0.00	0.00	0.00	0.00
0.00	0.00	0.00	0.00	0.00	0.00	0.00	0.00	0.00
0.00	0.00	145.00	0.00	0.00	0.00	0.00	0.00	0.00
0.00	0.00	0.00	0.00	0.00	0.00	0.00	0.00	0.00
0.00	0.00	11.00	0.00	0.00	0.00	0.00	0.00	3.00
0.00	99.00	0.00	0.00	0.00	0.00	0.00	0.00	0.00
0.00	0.00	0.00	0.00	0.00	0.00	0.00	0.00	0.00
42.00	0.00	58.00	52.00	28.00	59.00	7.00	62.00	17.00

7.00	0.00	0.00	0.00	0.00	0.00	0.00	0.00	0.00
0.00	0.00	0.00	0.00	0.00	0.00	0.00	0.00	0.00
0.00	0.00	0.00	0.00	529.00	0.00	0.00	0.00	0.00
21.00	0.00	12.00	10.00	5.00	9.00	0.00	10.00	0.00
0.00	0.00	0.00	0.00	0.00	0.00	519.00	0.00	0.00
0.00	0.00	0.00	0.00	0.00	0.00	0.00	0.00	0.00
0.00	0.00	0.00	0.00	0.00	0.00	0.00	0.00	0.00
0.00	0.00	0.00	0.00	0.00	0.00	0.00	0.00	0.00
0.00	0.00	0.00	5.00	0.00	0.00	0.00	0.00	486.00
0.00	0.00	0.00	0.00	0.00	0.00	0.00	0.00	0.00
14.00	0.00	24.00	23.00	0.00	0.00	0.00	0.00	0.00
0.00	0.00	0.00	0.00	0.00	0.00	0.00	0.00	0.00
0.00	0.00	0.00	0.00	0.00	0.00	494.00	0.00	0.00
0.00	493.00	0.00	0.00	0.00	0.00	0.00	0.00	0.00
0.00	0.00	0.00	0.00	0.00	0.00	0.00	0.00	0.00
0.00	0.00	0.00	0.00	0.00	0.00	0.00	0.00	0.00
0.00	0.00	0.00	0.00	0.00	0.00	0.00	0.00	0.00
0.00	0.00	0.00	0.00	0.00	0.00	0.00	0.00	0.00
0.00	0.00	0.00	0.00	0.00	0.00	0.00	488.00	0.00
0.00	0.00	0.00	0.00	0.00	0.00	0.00	0.00	0.00
0.00	0.00	29.00	0.00	34.00	25.00	0.00	32.00	0.00
0.00	0.00	0.00	0.00	0.00	100.00	0.00	0.00	0.00
0.00	0.00	0.00	0.00	0.00	0.00	479.00	0.00	0.00
0.00	0.00	0.00	0.00	0.00	0.00	0.00	0.00	0.00
0.00	0.00	0.00	2.00	0.00	0.00	0.00	0.00	475.00
0.00	0.00	29.00	0.00	0.00	41.00	0.00	30.00	0.00
0.00	0.00	0.00	0.00	0.00	0.00	0.00	0.00	0.00
0.00	0.00	0.00	0.00	0.00	0.00	0.00	0.00	0.00
0.00	0.00	0.00	0.00	0.00	0.00	0.00	0.00	0.00
7.00	0.00	0.00	0.00	0.00	0.00	0.00	0.00	0.00
0.00	0.00	0.00	0.00	0.00	0.00	0.00	0.00	0.00
0.00	0.00	0.00	0.00	0.00	0.00	0.00	0.00	0.00
0.00	463.00	0.00	0.00	0.00	0.00	0.00	0.00	0.00
0.00	0.00	0.00	0.00	0.00	0.00	0.00	0.00	0.00
0.00	0.00	0.00	0.00	0.00	0.00	0.00	0.00	0.00
0.00	0.00	0.00	0.00	0.00	0.00	0.00	0.00	0.00
0.00	0.00	0.00	2.00	0.00	0.00	0.00	0.00	0.00

0.00	0.00	0.00	0.00	0.00	0.00	0.00	0.00	0.00
0.00	0.00	0.00	0.00	0.00	0.00	0.00	0.00	0.00
0.00	0.00	0.00	0.00	0.00	0.00	0.00	0.00	0.00
0.00	0.00	9.00	0.00	4.00	7.00	0.00	0.00	3.00
87.00	0.00	76.00	0.00	35.00	0.00	0.00	24.00	0.00
0.00	0.00	0.00	0.00	0.00	0.00	0.00	0.00	0.00
0.00	0.00	0.00	0.00	0.00	0.00	385.00	3.00	0.00
0.00	0.00	0.00	0.00	0.00	0.00	0.00	0.00	0.00
0.00	0.00	0.00	0.00	0.00	0.00	0.00	408.00	0.00
0.00	0.00	46.00	0.00	0.00	0.00	0.00	0.00	0.00
0.00	0.00	0.00	0.00	0.00	0.00	0.00	0.00	0.00
0.00	0.00	0.00	0.00	0.00	0.00	0.00	0.00	0.00
0.00	0.00	0.00	0.00	0.00	0.00	0.00	0.00	0.00
0.00	0.00	0.00	0.00	0.00	0.00	0.00	0.00	0.00
0.00	0.00	0.00	0.00	0.00	0.00	0.00	0.00	0.00
0.00	0.00	0.00	0.00	0.00	0.00	0.00	0.00	0.00
15.00	0.00	18.00	34.00	20.00	50.00	7.00	45.00	4.00
0.00	0.00	0.00	256.00	0.00	0.00	0.00	0.00	0.00
0.00	0.00	0.00	0.00	0.00	0.00	0.00	0.00	0.00
0.00	0.00	0.00	0.00	0.00	0.00	0.00	0.00	0.00
0.00	0.00	0.00	0.00	0.00	0.00	0.00	0.00	0.00
0.00	0.00	33.00	0.00	0.00	0.00	0.00	0.00	0.00
0.00	0.00	0.00	0.00	360.00	0.00	0.00	0.00	0.00
0.00	0.00	0.00	0.00	0.00	0.00	0.00	0.00	0.00
0.00	0.00	0.00	39.00	66.00	26.00	0.00	0.00	0.00
0.00	0.00	0.00	0.00	0.00	0.00	0.00	0.00	0.00
0.00	0.00	0.00	0.00	0.00	0.00	0.00	0.00	0.00
0.00	0.00	0.00	0.00	0.00	0.00	0.00	0.00	0.00
0.00	0.00	0.00	0.00	0.00	0.00	0.00	0.00	0.00
0.00	0.00	3.00	35.00	0.00	18.00	0.00	0.00	0.00
0.00	0.00	0.00	0.00	0.00	0.00	0.00	0.00	0.00
0.00	0.00	0.00	0.00	0.00	0.00	0.00	0.00	0.00
0.00	0.00	0.00	0.00	0.00	342.00	0.00	0.00	0.00
0.00	0.00	0.00	0.00	0.00	0.00	0.00	0.00	0.00
0.00	0.00	0.00	0.00	0.00	0.00	341.00	0.00	0.00
0.00	154.00	0.00	0.00	0.00	82.00	0.00	0.00	0.00
0.00	0.00	0.00	0.00	0.00	0.00	0.00	0.00	0.00

0.00	0.00	0.00	0.00	0.00	10.00	0.00	0.00	0.00
13.00	0.00	0.00	15.00	10.00	12.00	0.00	19.00	0.00
0.00	0.00	0.00	331.00	0.00	0.00	0.00	0.00	0.00
70.00	0.00	0.00	0.00	0.00	0.00	0.00	0.00	0.00
0.00	0.00	0.00	0.00	0.00	41.00	0.00	27.00	0.00
0.00	0.00	0.00	0.00	0.00	0.00	0.00	0.00	328.00
0.00	0.00	0.00	0.00	0.00	0.00	0.00	0.00	0.00
0.00	0.00	0.00	0.00	0.00	0.00	0.00	0.00	0.00
0.00	0.00	0.00	0.00	0.00	0.00	0.00	0.00	0.00
9.00	0.00	19.00	26.00	18.00	27.00	3.00	40.00	7.00
0.00	0.00	33.00	52.00	0.00	69.00	0.00	0.00	24.00
0.00	0.00	0.00	0.00	0.00	0.00	0.00	0.00	0.00
0.00	0.00	0.00	309.00	0.00	0.00	0.00	0.00	0.00
12.00	0.00	22.00	23.00	14.00	24.00	0.00	20.00	5.00
0.00	0.00	0.00	0.00	0.00	0.00	314.00	6.00	0.00
0.00	0.00	0.00	0.00	0.00	0.00	0.00	0.00	0.00
0.00	0.00	0.00	0.00	0.00	0.00	0.00	0.00	0.00
0.00	0.00	4.00	55.00	98.00	0.00	0.00	0.00	0.00
0.00	0.00	0.00	0.00	0.00	0.00	317.00	0.00	0.00
0.00	0.00	0.00	0.00	0.00	0.00	0.00	317.00	0.00
0.00	263.00	0.00	0.00	0.00	0.00	0.00	0.00	0.00
0.00	0.00	0.00	0.00	0.00	0.00	0.00	2.00	314.00
0.00	0.00	0.00	0.00	0.00	0.00	0.00	0.00	0.00
0.00	0.00	0.00	0.00	0.00	0.00	0.00	0.00	0.00
0.00	0.00	0.00	0.00	0.00	0.00	0.00	0.00	0.00
0.00	0.00	0.00	0.00	0.00	0.00	312.00	0.00	0.00
0.00	0.00	0.00	0.00	0.00	0.00	199.00	0.00	0.00
0.00	0.00	0.00	38.00	57.00	0.00	0.00	0.00	0.00
0.00	0.00	0.00	0.00	0.00	0.00	0.00	0.00	0.00
0.00	0.00	0.00	0.00	0.00	0.00	0.00	0.00	0.00
0.00	0.00	0.00	0.00	0.00	0.00	0.00	0.00	0.00
0.00	0.00	0.00	0.00	0.00	0.00	0.00	0.00	0.00
0.00	0.00	0.00	0.00	0.00	0.00	0.00	0.00	0.00
0.00	0.00	0.00	0.00	0.00	0.00	0.00	0.00	0.00
0.00	0.00	0.00	0.00	0.00	0.00	0.00	0.00	0.00
0.00	0.00	0.00	0.00	0.00	0.00	0.00	0.00	0.00
0.00	0.00	0.00	0.00	0.00	0.00	0.00	0.00	0.00

0.00	0.00	0.00	0.00	0.00	0.00	0.00	0.00	0.00
0.00	0.00	0.00	0.00	0.00	0.00	0.00	0.00	0.00
12.00	0.00	28.00	28.00	25.00	31.00	4.00	24.00	9.00
0.00	0.00	0.00	0.00	0.00	0.00	0.00	0.00	0.00
0.00	0.00	0.00	0.00	0.00	0.00	0.00	279.00	0.00
11.00	0.00	15.00	21.00	13.00	6.00	0.00	13.00	0.00
0.00	0.00	0.00	0.00	0.00	0.00	0.00	0.00	0.00
0.00	0.00	0.00	0.00	0.00	0.00	0.00	270.00	0.00
0.00	0.00	0.00	124.00	58.00	0.00	0.00	0.00	0.00
0.00	0.00	0.00	47.00	0.00	0.00	0.00	0.00	0.00
0.00	0.00	0.00	0.00	0.00	0.00	0.00	0.00	0.00
0.00	0.00	0.00	0.00	0.00	0.00	0.00	0.00	0.00
0.00	0.00	0.00	256.00	0.00	0.00	0.00	0.00	0.00
0.00	0.00	0.00	0.00	0.00	0.00	0.00	0.00	0.00
0.00	0.00	19.00	0.00	0.00	0.00	0.00	0.00	0.00
0.00	0.00	0.00	0.00	0.00	0.00	0.00	0.00	0.00
0.00	0.00	0.00	0.00	0.00	0.00	0.00	0.00	0.00
0.00	0.00	0.00	0.00	0.00	0.00	0.00	0.00	0.00
0.00	0.00	0.00	0.00	0.00	0.00	0.00	0.00	0.00
0.00	0.00	0.00	0.00	0.00	0.00	0.00	0.00	0.00
0.00	0.00	0.00	0.00	0.00	0.00	0.00	0.00	0.00
0.00	0.00	0.00	0.00	0.00	0.00	0.00	0.00	0.00
0.00	0.00	0.00	5.00	0.00	0.00	2.00	0.00	0.00
20.00	0.00	27.00	0.00	0.00	10.00	3.00	0.00	0.00
0.00	0.00	0.00	0.00	33.00	0.00	0.00	0.00	0.00
0.00	0.00	0.00	0.00	0.00	0.00	0.00	0.00	0.00
0.00	0.00	0.00	0.00	0.00	0.00	0.00	0.00	0.00
0.00	0.00	0.00	0.00	0.00	0.00	0.00	0.00	0.00
18.00	0.00	26.00	43.00	16.00	38.00	0.00	38.00	5.00
0.00	0.00	0.00	0.00	0.00	0.00	0.00	0.00	0.00
0.00	0.00	0.00	0.00	0.00	0.00	0.00	0.00	0.00
0.00	0.00	0.00	0.00	0.00	242.00	0.00	0.00	0.00
0.00	0.00	0.00	0.00	0.00	0.00	0.00	0.00	0.00
0.00	0.00	0.00	0.00	0.00	0.00	0.00	0.00	0.00
0.00	0.00	0.00	0.00	0.00	0.00	0.00	0.00	0.00
0.00	0.00	0.00	0.00	0.00	0.00	0.00	0.00	0.00
0.00	0.00	0.00	0.00	0.00	0.00	237.00	0.00	0.00

0.00	0.00	0.00	0.00	0.00	0.00	0.00	0.00	0.00
0.00	0.00	0.00	0.00	0.00	0.00	0.00	0.00	0.00
0.00	0.00	0.00	0.00	0.00	0.00	0.00	0.00	0.00
0.00	0.00	0.00	0.00	0.00	0.00	0.00	0.00	0.00
0.00	0.00	0.00	0.00	0.00	0.00	0.00	0.00	0.00
0.00	0.00	0.00	0.00	0.00	0.00	0.00	0.00	0.00
0.00	0.00	0.00	29.00	21.00	21.00	0.00	23.00	9.00
0.00	0.00	0.00	0.00	0.00	0.00	0.00	5.00	0.00
0.00	0.00	0.00	0.00	0.00	0.00	0.00	0.00	0.00
0.00	0.00	0.00	82.00	63.00	0.00	0.00	0.00	0.00
0.00	0.00	0.00	0.00	10.00	0.00	0.00	0.00	0.00
0.00	0.00	0.00	0.00	0.00	0.00	0.00	0.00	0.00
0.00	0.00	19.00	0.00	0.00	0.00	0.00	0.00	0.00
0.00	0.00	15.00	0.00	3.00	0.00	0.00	2.00	0.00
0.00	0.00	0.00	0.00	0.00	0.00	0.00	0.00	0.00
0.00	0.00	0.00	0.00	0.00	0.00	0.00	0.00	0.00
0.00	0.00	0.00	0.00	0.00	0.00	0.00	0.00	0.00
0.00	0.00	0.00	0.00	0.00	0.00	0.00	0.00	0.00
0.00	0.00	0.00	0.00	0.00	0.00	0.00	0.00	0.00
0.00	0.00	0.00	0.00	0.00	0.00	0.00	0.00	0.00
0.00	0.00	0.00	56.00	0.00	0.00	0.00	0.00	0.00
0.00	0.00	0.00	0.00	0.00	0.00	0.00	0.00	0.00
0.00	0.00	0.00	0.00	0.00	0.00	0.00	0.00	0.00
0.00	0.00	0.00	0.00	0.00	0.00	0.00	0.00	0.00
4.00	0.00	11.00	17.00	11.00	22.00	7.00	13.00	5.00
0.00	0.00	0.00	0.00	0.00	0.00	0.00	0.00	0.00
0.00	0.00	0.00	0.00	0.00	0.00	0.00	0.00	0.00
9.00	0.00	22.00	14.00	7.00	17.00	6.00	9.00	0.00
14.00	0.00	20.00	13.00	5.00	9.00	0.00	7.00	0.00
0.00	0.00	0.00	0.00	0.00	0.00	0.00	19.00	0.00
0.00	0.00	0.00	0.00	0.00	0.00	0.00	0.00	0.00
0.00	0.00	0.00	0.00	0.00	0.00	0.00	0.00	0.00
17.00	0.00	26.00	0.00	0.00	13.00	0.00	0.00	0.00
0.00	0.00	0.00	0.00	0.00	0.00	0.00	0.00	0.00
0.00	0.00	0.00	0.00	0.00	163.00	0.00	0.00	0.00
0.00	0.00	0.00	0.00	0.00	0.00	0.00	0.00	0.00
0.00	0.00	0.00	0.00	0.00	0.00	0.00	0.00	205.00

0.00	0.00	0.00	0.00	0.00	0.00	0.00	0.00	0.00
0.00	0.00	0.00	0.00	0.00	3.00	0.00	0.00	0.00
0.00	0.00	0.00	0.00	0.00	0.00	0.00	0.00	0.00
0.00	0.00	0.00	0.00	0.00	0.00	0.00	0.00	0.00
11.00	0.00	31.00	22.00	0.00	0.00	0.00	0.00	0.00
0.00	0.00	0.00	0.00	0.00	0.00	0.00	0.00	0.00
0.00	0.00	0.00	36.00	0.00	0.00	0.00	0.00	141.00
0.00	0.00	0.00	0.00	0.00	0.00	0.00	0.00	0.00
0.00	0.00	0.00	0.00	0.00	0.00	0.00	0.00	0.00
0.00	0.00	0.00	0.00	0.00	0.00	0.00	0.00	0.00
0.00	0.00	0.00	0.00	0.00	0.00	0.00	0.00	0.00
0.00	0.00	0.00	0.00	0.00	0.00	0.00	0.00	0.00
0.00	0.00	0.00	0.00	0.00	0.00	0.00	0.00	0.00
0.00	0.00	0.00	0.00	0.00	0.00	0.00	0.00	0.00
0.00	0.00	0.00	0.00	0.00	0.00	0.00	0.00	0.00
12.00	0.00	13.00	15.00	5.00	14.00	0.00	12.00	0.00
0.00	0.00	0.00	0.00	0.00	0.00	0.00	0.00	0.00
0.00	0.00	0.00	0.00	0.00	0.00	0.00	15.00	0.00
0.00	0.00	0.00	0.00	0.00	0.00	0.00	0.00	0.00
0.00	0.00	14.00	23.00	0.00	0.00	7.00	0.00	0.00
24.00	0.00	5.00	15.00	8.00	2.00	0.00	9.00	4.00
0.00	0.00	0.00	0.00	0.00	0.00	0.00	0.00	0.00
0.00	0.00	0.00	0.00	0.00	0.00	77.00	0.00	0.00
0.00	0.00	0.00	0.00	0.00	0.00	0.00	0.00	0.00
0.00	0.00	0.00	0.00	0.00	0.00	0.00	0.00	130.00
0.00	0.00	0.00	0.00	0.00	0.00	0.00	0.00	0.00
0.00	0.00	0.00	0.00	0.00	0.00	136.00	0.00	0.00
0.00	0.00	0.00	0.00	0.00	0.00	0.00	0.00	0.00
0.00	0.00	13.00	0.00	0.00	26.00	5.00	9.00	0.00
0.00	0.00	0.00	0.00	0.00	0.00	0.00	0.00	0.00
0.00	0.00	0.00	0.00	0.00	0.00	0.00	8.00	0.00
0.00	0.00	0.00	15.00	0.00	0.00	0.00	0.00	0.00
0.00	0.00	0.00	0.00	0.00	0.00	0.00	0.00	0.00
0.00	0.00	0.00	0.00	0.00	0.00	0.00	0.00	0.00
0.00	0.00	0.00	0.00	0.00	0.00	0.00	49.00	0.00
0.00	0.00	0.00	0.00	0.00	0.00	0.00	0.00	0.00
0.00	0.00	0.00	0.00	0.00	0.00	0.00	9.00	0.00

0.00	0.00	0.00	0.00	0.00	0.00	0.00	0.00	0.00
0.00	0.00	0.00	0.00	0.00	0.00	0.00	0.00	0.00
0.00	0.00	0.00	0.00	0.00	0.00	0.00	0.00	0.00
0.00	0.00	0.00	171.00	0.00	0.00	0.00	0.00	0.00
0.00	0.00	17.00	0.00	14.00	18.00	0.00	19.00	0.00
0.00	0.00	0.00	0.00	0.00	0.00	0.00	0.00	0.00
0.00	0.00	0.00	0.00	0.00	0.00	0.00	0.00	0.00
0.00	0.00	0.00	0.00	0.00	0.00	0.00	0.00	0.00
0.00	0.00	0.00	0.00	0.00	0.00	0.00	0.00	0.00
0.00	0.00	0.00	0.00	0.00	0.00	0.00	0.00	0.00
0.00	0.00	0.00	0.00	0.00	0.00	0.00	0.00	0.00
0.00	0.00	0.00	0.00	0.00	0.00	0.00	0.00	0.00
0.00	0.00	0.00	0.00	0.00	0.00	0.00	0.00	0.00
0.00	0.00	0.00	0.00	0.00	0.00	0.00	0.00	0.00
0.00	0.00	0.00	0.00	0.00	0.00	0.00	0.00	0.00
0.00	0.00	0.00	0.00	0.00	0.00	0.00	0.00	0.00
0.00	0.00	0.00	6.00	0.00	0.00	0.00	0.00	0.00
0.00	0.00	0.00	0.00	0.00	0.00	0.00	0.00	0.00
0.00	0.00	0.00	0.00	0.00	0.00	0.00	0.00	0.00
0.00	0.00	0.00	0.00	0.00	0.00	0.00	0.00	0.00
0.00	0.00	0.00	0.00	0.00	0.00	0.00	0.00	0.00
0.00	0.00	0.00	0.00	0.00	0.00	0.00	0.00	0.00
0.00	0.00	0.00	0.00	0.00	0.00	0.00	0.00	0.00
0.00	0.00	0.00	0.00	0.00	3.00	0.00	0.00	0.00
0.00	0.00	0.00	0.00	0.00	0.00	0.00	0.00	0.00
0.00	0.00	0.00	0.00	0.00	0.00	0.00	0.00	162.00
0.00	0.00	0.00	0.00	0.00	0.00	0.00	0.00	0.00
0.00	0.00	0.00	0.00	0.00	0.00	0.00	0.00	161.00
0.00	0.00	0.00	0.00	0.00	0.00	0.00	0.00	0.00
0.00	0.00	0.00	0.00	0.00	0.00	0.00	0.00	0.00
0.00	0.00	0.00	0.00	0.00	160.00	0.00	0.00	0.00
0.00	0.00	0.00	0.00	0.00	0.00	0.00	0.00	0.00
0.00	0.00	0.00	0.00	0.00	0.00	0.00	0.00	0.00
0.00	0.00	0.00	0.00	0.00	0.00	0.00	0.00	0.00
0.00	0.00	0.00	0.00	0.00	0.00	0.00	0.00	0.00
0.00	0.00	0.00	0.00	0.00	0.00	0.00	0.00	0.00
0.00	0.00	0.00	0.00	0.00	0.00	0.00	0.00	0.00

0.00	0.00	0.00	0.00	0.00	20.00	0.00	16.00	0.00
0.00	0.00	0.00	0.00	156.00	0.00	0.00	0.00	0.00
0.00	0.00	0.00	0.00	0.00	0.00	0.00	0.00	0.00
0.00	0.00	0.00	0.00	0.00	0.00	0.00	0.00	0.00
0.00	0.00	0.00	0.00	0.00	0.00	0.00	0.00	0.00
0.00	0.00	0.00	0.00	0.00	0.00	0.00	0.00	0.00
0.00	0.00	0.00	0.00	0.00	0.00	0.00	0.00	0.00
20.00	0.00	20.00	12.00	0.00	17.00	0.00	0.00	0.00
0.00	0.00	0.00	0.00	0.00	0.00	0.00	0.00	0.00
0.00	0.00	0.00	0.00	0.00	0.00	0.00	0.00	0.00
0.00	0.00	0.00	0.00	0.00	0.00	0.00	0.00	0.00
0.00	0.00	0.00	0.00	0.00	0.00	0.00	0.00	0.00
0.00	137.00	0.00	0.00	0.00	0.00	0.00	0.00	0.00
0.00	0.00	0.00	0.00	0.00	0.00	0.00	0.00	0.00
0.00	0.00	0.00	0.00	0.00	0.00	0.00	0.00	0.00
0.00	0.00	0.00	0.00	0.00	0.00	0.00	0.00	0.00
0.00	0.00	8.00	0.00	0.00	0.00	0.00	0.00	142.00
0.00	0.00	0.00	0.00	0.00	0.00	0.00	150.00	0.00
10.00	0.00	0.00	0.00	0.00	0.00	0.00	0.00	0.00
0.00	0.00	0.00	0.00	0.00	0.00	0.00	0.00	0.00
0.00	0.00	0.00	11.00	0.00	0.00	0.00	0.00	0.00
0.00	0.00	12.00	17.00	0.00	22.00	0.00	31.00	0.00
0.00	0.00	0.00	0.00	0.00	0.00	0.00	0.00	0.00
0.00	0.00	12.00	28.00	0.00	0.00	0.00	0.00	0.00
3.00	0.00	0.00	0.00	0.00	0.00	0.00	0.00	0.00
0.00	0.00	0.00	0.00	0.00	139.00	0.00	0.00	0.00
0.00	0.00	6.00	0.00	0.00	0.00	0.00	0.00	0.00
0.00	0.00	0.00	0.00	0.00	0.00	0.00	0.00	0.00
0.00	0.00	0.00	0.00	0.00	0.00	0.00	0.00	0.00
0.00	0.00	0.00	0.00	0.00	0.00	0.00	0.00	0.00
0.00	0.00	0.00	0.00	0.00	0.00	0.00	0.00	0.00
0.00	0.00	0.00	0.00	0.00	0.00	0.00	0.00	0.00
7.00	0.00	16.00	0.00	0.00	0.00	0.00	0.00	0.00
0.00	0.00	0.00	0.00	0.00	0.00	0.00	0.00	0.00
0.00	0.00	0.00	0.00	0.00	0.00	0.00	0.00	0.00
0.00	0.00	0.00	0.00	0.00	0.00	0.00	0.00	0.00
0.00	0.00	0.00	0.00	0.00	0.00	0.00	0.00	0.00

0.00	0.00	0.00	20.00	0.00	0.00	0.00	0.00	0.00
15.00	0.00	16.00	18.00	0.00	22.00	0.00	17.00	11.00
0.00	0.00	0.00	0.00	0.00	0.00	0.00	0.00	0.00
0.00	0.00	0.00	17.00	0.00	15.00	0.00	16.00	0.00
0.00	0.00	0.00	0.00	0.00	0.00	0.00	0.00	0.00
0.00	0.00	7.00	4.00	11.00	9.00	0.00	0.00	0.00
0.00	0.00	0.00	0.00	0.00	0.00	0.00	0.00	0.00
0.00	0.00	0.00	0.00	0.00	0.00	0.00	0.00	0.00
0.00	0.00	0.00	0.00	0.00	0.00	0.00	0.00	0.00
0.00	0.00	0.00	0.00	0.00	0.00	0.00	0.00	0.00
0.00	0.00	0.00	0.00	0.00	0.00	0.00	0.00	0.00
0.00	0.00	0.00	0.00	0.00	0.00	0.00	125.00	0.00
46.00	0.00	0.00	0.00	0.00	0.00	0.00	0.00	0.00
0.00	98.00	0.00	0.00	0.00	0.00	0.00	0.00	0.00
0.00	0.00	0.00	0.00	0.00	0.00	0.00	0.00	0.00
12.00	0.00	0.00	0.00	0.00	0.00	0.00	0.00	0.00
0.00	0.00	0.00	0.00	0.00	0.00	0.00	0.00	0.00
0.00	0.00	0.00	0.00	0.00	0.00	0.00	0.00	0.00
0.00	0.00	0.00	0.00	0.00	2.00	0.00	0.00	0.00
0.00	0.00	0.00	0.00	0.00	0.00	0.00	0.00	0.00
0.00	0.00	0.00	119.00	0.00	0.00	0.00	0.00	0.00
0.00	0.00	0.00	0.00	0.00	0.00	0.00	0.00	0.00
0.00	0.00	0.00	0.00	0.00	0.00	0.00	0.00	0.00
0.00	0.00	0.00	0.00	0.00	0.00	0.00	35.00	0.00
0.00	0.00	0.00	0.00	0.00	0.00	0.00	0.00	0.00
0.00	0.00	0.00	0.00	0.00	0.00	0.00	0.00	0.00
0.00	0.00	0.00	0.00	0.00	0.00	0.00	0.00	0.00
0.00	0.00	0.00	0.00	0.00	0.00	0.00	0.00	0.00
0.00	0.00	0.00	0.00	0.00	0.00	0.00	0.00	0.00
0.00	0.00	0.00	0.00	0.00	0.00	0.00	0.00	0.00
0.00	0.00	0.00	0.00	0.00	0.00	0.00	33.00	0.00
0.00	0.00	0.00	0.00	0.00	0.00	0.00	0.00	0.00
0.00	0.00	0.00	0.00	0.00	14.00	4.00	18.00	0.00
0.00	0.00	0.00	0.00	0.00	0.00	0.00	0.00	0.00
0.00	0.00	0.00	0.00	0.00	0.00	0.00	0.00	0.00
0.00	0.00	0.00	0.00	0.00	0.00	0.00	0.00	0.00
0.00	0.00	0.00	0.00	0.00	0.00	0.00	0.00	0.00

0.00	0.00	0.00	0.00	0.00	0.00	0.00	0.00	0.00
0.00	0.00	0.00	0.00	0.00	0.00	0.00	0.00	0.00
0.00	0.00	0.00	0.00	0.00	0.00	0.00	0.00	0.00
0.00	0.00	0.00	0.00	0.00	0.00	0.00	0.00	0.00
0.00	0.00	0.00	0.00	34.00	0.00	0.00	0.00	0.00
0.00	0.00	0.00	0.00	0.00	0.00	0.00	0.00	0.00
0.00	0.00	0.00	0.00	0.00	0.00	0.00	0.00	0.00
0.00	0.00	0.00	0.00	0.00	0.00	0.00	0.00	0.00
0.00	0.00	0.00	0.00	0.00	0.00	0.00	0.00	0.00
0.00	0.00	0.00	0.00	0.00	0.00	0.00	0.00	0.00
0.00	0.00	0.00	0.00	0.00	0.00	0.00	0.00	0.00
0.00	0.00	13.00	0.00	0.00	2.00	3.00	3.00	0.00
0.00	0.00	0.00	0.00	0.00	0.00	0.00	0.00	0.00
0.00	0.00	0.00	0.00	0.00	0.00	0.00	0.00	0.00
0.00	0.00	0.00	108.00	0.00	0.00	0.00	0.00	0.00
0.00	0.00	0.00	0.00	0.00	0.00	0.00	0.00	0.00
0.00	0.00	0.00	0.00	0.00	0.00	0.00	0.00	0.00
0.00	0.00	0.00	0.00	0.00	0.00	0.00	0.00	0.00
0.00	0.00	0.00	0.00	0.00	0.00	0.00	0.00	0.00
0.00	0.00	0.00	0.00	0.00	0.00	0.00	0.00	0.00
0.00	0.00	0.00	0.00	0.00	0.00	0.00	0.00	0.00
0.00	0.00	0.00	0.00	0.00	0.00	0.00	0.00	0.00
0.00	0.00	0.00	0.00	0.00	0.00	0.00	0.00	0.00
0.00	0.00	0.00	0.00	0.00	0.00	0.00	0.00	0.00
0.00	0.00	0.00	0.00	0.00	0.00	0.00	0.00	0.00
0.00	0.00	0.00	0.00	0.00	0.00	0.00	0.00	0.00
0.00	0.00	0.00	0.00	0.00	0.00	0.00	0.00	0.00
0.00	0.00	0.00	0.00	0.00	0.00	0.00	0.00	0.00
0.00	0.00	0.00	0.00	0.00	0.00	0.00	0.00	0.00
0.00	0.00	0.00	0.00	0.00	0.00	0.00	0.00	0.00
0.00	0.00	0.00	0.00	59.00	0.00	0.00	0.00	0.00
0.00	0.00	0.00	0.00	102.00	0.00	0.00	0.00	0.00
0.00	0.00	0.00	0.00	0.00	0.00	0.00	0.00	0.00
0.00	0.00	0.00	0.00	0.00	0.00	0.00	0.00	0.00
0.00	0.00	0.00	0.00	0.00	0.00	0.00	0.00	0.00
0.00	0.00	0.00	0.00	0.00	0.00	0.00	0.00	0.00
0.00	0.00	0.00	0.00	0.00	0.00	0.00	0.00	0.00

0.00	0.00	0.00	0.00	0.00	0.00	0.00	0.00	0.00
0.00	0.00	0.00	0.00	0.00	0.00	0.00	0.00	0.00
0.00	0.00	0.00	101.00	0.00	0.00	0.00	0.00	0.00
0.00	0.00	0.00	0.00	0.00	0.00	0.00	0.00	0.00
0.00	0.00	0.00	0.00	0.00	0.00	0.00	0.00	0.00
0.00	0.00	0.00	0.00	0.00	0.00	0.00	0.00	0.00
0.00	0.00	0.00	0.00	0.00	0.00	0.00	0.00	0.00
0.00	0.00	0.00	0.00	0.00	0.00	0.00	0.00	0.00
0.00	0.00	0.00	0.00	0.00	0.00	0.00	0.00	0.00
0.00	0.00	0.00	0.00	0.00	0.00	0.00	18.00	0.00
0.00	0.00	0.00	0.00	0.00	0.00	0.00	0.00	0.00
0.00	0.00	0.00	0.00	0.00	0.00	0.00	0.00	0.00
0.00	0.00	0.00	0.00	0.00	0.00	0.00	0.00	0.00
0.00	0.00	0.00	0.00	0.00	0.00	0.00	0.00	0.00
0.00	0.00	0.00	0.00	0.00	0.00	0.00	0.00	0.00
0.00	0.00	0.00	0.00	0.00	0.00	0.00	0.00	0.00
0.00	0.00	0.00	0.00	0.00	0.00	0.00	0.00	0.00
0.00	0.00	0.00	0.00	0.00	0.00	0.00	0.00	0.00
0.00	0.00	0.00	0.00	0.00	0.00	0.00	0.00	0.00
0.00	0.00	0.00	0.00	0.00	0.00	0.00	0.00	0.00
0.00	0.00	0.00	0.00	0.00	0.00	0.00	0.00	0.00
0.00	0.00	0.00	0.00	0.00	0.00	0.00	0.00	0.00
10.00	0.00	16.00	13.00	5.00	0.00	0.00	0.00	0.00
0.00	0.00	0.00	0.00	0.00	0.00	0.00	0.00	0.00
0.00	0.00	0.00	0.00	0.00	0.00	0.00	0.00	0.00
0.00	0.00	0.00	0.00	13.00	0.00	0.00	0.00	0.00
0.00	0.00	0.00	0.00	0.00	0.00	0.00	0.00	0.00
0.00	0.00	0.00	0.00	0.00	0.00	0.00	0.00	0.00
0.00	0.00	0.00	0.00	0.00	0.00	0.00	0.00	0.00
0.00	0.00	0.00	0.00	0.00	0.00	0.00	93.00	0.00
0.00	0.00	0.00	0.00	0.00	0.00	0.00	0.00	0.00
0.00	0.00	0.00	0.00	0.00	0.00	0.00	0.00	0.00
0.00	0.00	0.00	0.00	0.00	0.00	0.00	0.00	0.00
0.00	0.00	0.00	0.00	0.00	0.00	0.00	0.00	92.00
0.00	0.00	0.00	0.00	0.00	0.00	0.00	0.00	0.00
0.00	0.00	0.00	0.00	0.00	0.00	0.00	0.00	0.00
0.00	0.00	2.00	4.00	2.00	0.00	0.00	0.00	0.00

0.00	0.00	0.00	65.00	0.00	0.00	0.00	0.00	0.00
0.00	0.00	0.00	0.00	90.00	0.00	0.00	0.00	0.00
0.00	0.00	0.00	0.00	0.00	0.00	0.00	0.00	0.00
0.00	0.00	0.00	0.00	0.00	0.00	0.00	0.00	0.00
0.00	0.00	6.00	18.00	0.00	0.00	0.00	11.00	0.00
0.00	0.00	0.00	0.00	0.00	0.00	0.00	0.00	0.00
0.00	0.00	0.00	0.00	0.00	0.00	0.00	42.00	0.00
0.00	0.00	0.00	0.00	0.00	0.00	0.00	0.00	0.00
0.00	0.00	0.00	0.00	0.00	0.00	0.00	0.00	0.00
0.00	0.00	0.00	4.00	0.00	0.00	0.00	0.00	0.00
0.00	0.00	0.00	0.00	0.00	0.00	0.00	0.00	0.00
0.00	0.00	0.00	0.00	0.00	0.00	0.00	0.00	0.00
0.00	0.00	0.00	0.00	0.00	0.00	0.00	0.00	0.00
0.00	0.00	0.00	0.00	4.00	11.00	0.00	6.00	0.00
0.00	0.00	0.00	0.00	0.00	0.00	0.00	0.00	0.00
0.00	0.00	0.00	0.00	0.00	0.00	0.00	0.00	0.00
0.00	0.00	0.00	0.00	0.00	0.00	0.00	0.00	0.00
10.00	0.00	0.00	54.00	0.00	0.00	0.00	0.00	0.00
0.00	0.00	0.00	0.00	0.00	0.00	0.00	0.00	0.00
0.00	0.00	0.00	0.00	0.00	0.00	0.00	0.00	0.00
0.00	0.00	0.00	0.00	0.00	0.00	0.00	0.00	0.00
0.00	0.00	0.00	0.00	0.00	0.00	0.00	0.00	0.00
0.00	0.00	0.00	0.00	0.00	0.00	0.00	0.00	0.00
0.00	0.00	0.00	0.00	83.00	0.00	0.00	0.00	0.00
0.00	0.00	0.00	0.00	0.00	18.00	0.00	19.00	0.00
0.00	0.00	0.00	0.00	0.00	0.00	0.00	0.00	0.00
0.00	0.00	0.00	0.00	0.00	0.00	0.00	0.00	0.00
0.00	0.00	6.00	8.00	0.00	15.00	0.00	11.00	0.00
0.00	0.00	0.00	0.00	0.00	0.00	0.00	0.00	0.00
0.00	0.00	0.00	81.00	0.00	0.00	0.00	0.00	0.00
0.00	0.00	0.00	0.00	0.00	0.00	0.00	0.00	0.00
0.00	0.00	0.00	0.00	0.00	0.00	0.00	0.00	0.00
0.00	0.00	0.00	0.00	0.00	0.00	0.00	0.00	0.00
0.00	0.00	3.00	0.00	0.00	2.00	0.00	4.00	0.00
0.00	0.00	0.00	0.00	0.00	0.00	0.00	0.00	0.00
0.00	0.00	0.00	0.00	0.00	0.00	0.00	0.00	0.00
0.00	0.00	0.00	0.00	0.00	0.00	0.00	0.00	0.00

0.00	0.00	0.00	0.00	0.00	0.00	0.00	0.00	0.00
0.00	0.00	0.00	0.00	0.00	0.00	0.00	0.00	0.00
0.00	0.00	52.00	0.00	0.00	0.00	0.00	0.00	0.00
0.00	0.00	0.00	0.00	0.00	0.00	0.00	0.00	0.00
0.00	0.00	0.00	0.00	14.00	0.00	0.00	0.00	0.00
0.00	0.00	0.00	50.00	0.00	0.00	0.00	0.00	0.00
0.00	0.00	0.00	0.00	0.00	0.00	0.00	0.00	0.00
0.00	0.00	0.00	0.00	0.00	0.00	0.00	0.00	0.00
0.00	0.00	0.00	18.00	0.00	11.00	0.00	0.00	0.00
0.00	0.00	22.00	0.00	0.00	0.00	0.00	0.00	0.00
0.00	0.00	0.00	0.00	0.00	0.00	0.00	0.00	0.00
0.00	0.00	0.00	0.00	0.00	0.00	0.00	0.00	0.00
0.00	0.00	0.00	0.00	0.00	0.00	0.00	0.00	0.00
0.00	0.00	0.00	0.00	0.00	0.00	0.00	0.00	0.00
0.00	0.00	0.00	0.00	0.00	0.00	0.00	0.00	0.00
0.00	0.00	0.00	0.00	0.00	0.00	0.00	0.00	0.00
4.00	0.00	3.00	8.00	0.00	8.00	0.00	10.00	2.00
0.00	0.00	0.00	0.00	0.00	0.00	0.00	0.00	0.00
0.00	0.00	0.00	0.00	0.00	0.00	0.00	0.00	0.00
0.00	0.00	0.00	0.00	0.00	0.00	0.00	0.00	0.00
0.00	0.00	0.00	0.00	0.00	0.00	0.00	0.00	0.00
0.00	0.00	0.00	0.00	0.00	0.00	0.00	0.00	0.00
0.00	0.00	2.00	0.00	0.00	0.00	0.00	3.00	0.00
0.00	0.00	0.00	0.00	0.00	0.00	0.00	0.00	0.00
0.00	0.00	0.00	0.00	0.00	0.00	0.00	0.00	0.00
0.00	0.00	0.00	0.00	0.00	0.00	0.00	0.00	0.00
0.00	0.00	0.00	0.00	0.00	0.00	0.00	0.00	0.00
0.00	0.00	0.00	0.00	0.00	0.00	0.00	0.00	0.00
11.00	0.00	11.00	9.00	0.00	0.00	0.00	0.00	0.00
0.00	0.00	0.00	0.00	0.00	0.00	0.00	0.00	0.00
0.00	0.00	0.00	0.00	0.00	0.00	3.00	0.00	0.00
0.00	0.00	0.00	0.00	0.00	0.00	0.00	0.00	0.00
0.00	0.00	0.00	0.00	0.00	0.00	0.00	0.00	0.00
0.00	0.00	0.00	0.00	0.00	0.00	0.00	0.00	0.00
0.00	0.00	0.00	0.00	0.00	0.00	0.00	0.00	0.00
23.00	0.00	0.00	0.00	0.00	0.00	0.00	0.00	0.00
0.00	0.00	0.00	29.00	0.00	0.00	0.00	0.00	0.00

0.00	0.00	0.00	0.00	0.00	0.00	0.00	0.00	0.00
0.00	0.00	0.00	0.00	0.00	0.00	0.00	0.00	0.00
0.00	0.00	20.00	0.00	0.00	0.00	0.00	0.00	0.00
0.00	0.00	0.00	0.00	0.00	12.00	0.00	0.00	0.00
0.00	0.00	0.00	11.00	0.00	11.00	0.00	0.00	0.00
0.00	0.00	0.00	0.00	0.00	0.00	0.00	0.00	0.00
0.00	0.00	0.00	0.00	0.00	0.00	0.00	0.00	0.00
0.00	0.00	0.00	0.00	21.00	0.00	0.00	0.00	0.00
0.00	0.00	0.00	0.00	0.00	0.00	0.00	0.00	0.00
0.00	0.00	0.00	0.00	0.00	0.00	0.00	0.00	0.00
0.00	0.00	0.00	0.00	0.00	0.00	0.00	0.00	0.00
11.00	0.00	10.00	3.00	0.00	0.00	0.00	0.00	0.00
0.00	0.00	0.00	0.00	0.00	0.00	0.00	0.00	0.00
0.00	0.00	0.00	0.00	0.00	0.00	0.00	0.00	0.00
0.00	0.00	0.00	0.00	0.00	0.00	0.00	0.00	0.00
0.00	0.00	0.00	0.00	0.00	0.00	0.00	0.00	0.00
0.00	0.00	0.00	0.00	0.00	0.00	0.00	0.00	0.00
0.00	0.00	0.00	0.00	0.00	0.00	0.00	0.00	0.00
0.00	0.00	0.00	0.00	0.00	0.00	0.00	0.00	0.00
0.00	0.00	0.00	3.00	6.00	4.00	0.00	0.00	2.00
0.00	0.00	0.00	0.00	0.00	0.00	0.00	0.00	0.00
0.00	0.00	0.00	0.00	0.00	0.00	0.00	0.00	67.00
0.00	0.00	0.00	0.00	0.00	0.00	0.00	0.00	0.00
0.00	0.00	0.00	0.00	0.00	0.00	0.00	0.00	0.00
0.00	0.00	0.00	0.00	0.00	0.00	0.00	0.00	0.00
0.00	0.00	6.00	0.00	8.00	4.00	0.00	12.00	0.00
0.00	0.00	0.00	0.00	0.00	2.00	0.00	0.00	0.00
0.00	0.00	0.00	64.00	0.00	0.00	0.00	0.00	0.00
0.00	0.00	0.00	0.00	0.00	0.00	0.00	66.00	0.00
0.00	0.00	0.00	0.00	0.00	0.00	0.00	0.00	0.00
0.00	0.00	0.00	34.00	0.00	0.00	0.00	0.00	0.00
0.00	0.00	0.00	0.00	0.00	49.00	0.00	0.00	0.00
0.00	0.00	0.00	65.00	0.00	0.00	0.00	0.00	0.00
0.00	0.00	0.00	0.00	0.00	0.00	0.00	0.00	0.00
0.00	0.00	0.00	0.00	0.00	0.00	0.00	0.00	0.00
0.00	0.00	0.00	0.00	0.00	0.00	0.00	0.00	0.00
0.00	0.00	0.00	0.00	0.00	0.00	0.00	0.00	0.00

0.00	0.00	0.00	0.00	0.00	0.00	0.00	0.00	0.00
0.00	0.00	0.00	0.00	0.00	64.00	0.00	0.00	0.00
0.00	0.00	0.00	61.00	0.00	0.00	0.00	0.00	0.00
0.00	0.00	0.00	0.00	0.00	0.00	0.00	0.00	0.00
0.00	0.00	0.00	0.00	0.00	0.00	0.00	0.00	0.00
0.00	0.00	0.00	0.00	0.00	0.00	0.00	0.00	0.00
0.00	0.00	0.00	0.00	0.00	0.00	0.00	0.00	0.00
0.00	0.00	0.00	0.00	0.00	0.00	0.00	0.00	0.00
0.00	0.00	0.00	0.00	0.00	0.00	0.00	0.00	0.00
0.00	0.00	0.00	0.00	0.00	0.00	0.00	0.00	0.00
0.00	0.00	0.00	0.00	0.00	12.00	0.00	9.00	0.00
0.00	0.00	0.00	0.00	0.00	0.00	0.00	0.00	0.00
0.00	0.00	0.00	12.00	0.00	0.00	0.00	0.00	0.00
0.00	0.00	0.00	0.00	0.00	0.00	0.00	0.00	0.00
0.00	0.00	0.00	0.00	0.00	0.00	0.00	0.00	0.00
0.00	0.00	0.00	0.00	0.00	8.00	0.00	0.00	0.00
0.00	0.00	0.00	0.00	0.00	0.00	0.00	0.00	0.00
0.00	0.00	0.00	0.00	0.00	0.00	0.00	0.00	0.00
0.00	0.00	0.00	0.00	0.00	0.00	0.00	0.00	0.00
0.00	0.00	0.00	0.00	0.00	0.00	0.00	0.00	0.00
0.00	0.00	0.00	62.00	0.00	0.00	0.00	0.00	0.00
0.00	0.00	0.00	0.00	0.00	0.00	0.00	0.00	0.00
0.00	0.00	0.00	0.00	0.00	0.00	0.00	0.00	0.00
0.00	0.00	0.00	0.00	0.00	0.00	0.00	0.00	0.00
0.00	0.00	0.00	57.00	0.00	0.00	0.00	0.00	0.00
0.00	0.00	0.00	0.00	0.00	0.00	0.00	0.00	0.00
0.00	0.00	0.00	0.00	0.00	0.00	0.00	0.00	0.00
0.00	0.00	0.00	0.00	0.00	14.00	0.00	0.00	0.00
0.00	0.00	8.00	0.00	0.00	0.00	0.00	7.00	0.00
0.00	0.00	0.00	0.00	0.00	0.00	0.00	0.00	0.00
0.00	0.00	0.00	0.00	0.00	0.00	0.00	0.00	0.00
0.00	0.00	0.00	60.00	0.00	0.00	0.00	0.00	0.00
0.00	0.00	0.00	60.00	0.00	0.00	0.00	0.00	0.00
0.00	0.00	0.00	0.00	0.00	0.00	0.00	0.00	0.00
0.00	0.00	0.00	0.00	0.00	0.00	0.00	0.00	0.00
0.00	0.00	0.00	0.00	0.00	0.00	0.00	0.00	0.00
0.00	0.00	0.00	0.00	0.00	0.00	0.00	0.00	0.00

0.00	0.00	0.00	0.00	0.00	0.00	0.00	0.00	0.00
0.00	0.00	0.00	0.00	0.00	0.00	0.00	0.00	0.00
0.00	0.00	0.00	0.00	0.00	0.00	5.00	19.00	0.00
0.00	0.00	0.00	0.00	0.00	0.00	0.00	0.00	0.00
0.00	0.00	0.00	0.00	0.00	0.00	0.00	0.00	0.00
0.00	0.00	0.00	0.00	0.00	0.00	0.00	0.00	0.00
0.00	0.00	0.00	0.00	0.00	0.00	0.00	0.00	0.00
0.00	0.00	0.00	0.00	0.00	15.00	0.00	0.00	0.00
0.00	0.00	0.00	0.00	0.00	0.00	0.00	0.00	0.00
0.00	0.00	0.00	0.00	0.00	0.00	0.00	0.00	0.00
0.00	0.00	0.00	58.00	0.00	0.00	0.00	0.00	0.00
0.00	0.00	0.00	58.00	0.00	0.00	0.00	0.00	0.00
0.00	0.00	0.00	0.00	0.00	0.00	0.00	0.00	0.00
0.00	0.00	0.00	0.00	0.00	0.00	0.00	0.00	0.00
0.00	0.00	0.00	0.00	0.00	0.00	0.00	0.00	0.00
0.00	0.00	0.00	0.00	0.00	0.00	0.00	0.00	0.00
0.00	0.00	0.00	0.00	0.00	0.00	0.00	0.00	0.00
0.00	0.00	0.00	57.00	0.00	0.00	0.00	0.00	0.00
0.00	0.00	0.00	0.00	0.00	0.00	0.00	0.00	0.00
0.00	0.00	0.00	0.00	0.00	0.00	0.00	0.00	2.00
0.00	0.00	0.00	0.00	0.00	0.00	0.00	0.00	0.00
0.00	0.00	0.00	0.00	0.00	0.00	0.00	0.00	0.00
0.00	0.00	0.00	0.00	0.00	0.00	0.00	0.00	0.00
0.00	0.00	0.00	0.00	0.00	0.00	0.00	0.00	0.00
0.00	0.00	0.00	0.00	0.00	0.00	0.00	0.00	0.00
0.00	0.00	0.00	0.00	0.00	0.00	0.00	0.00	0.00
3.00	0.00	0.00	9.00	0.00	12.00	0.00	10.00	0.00
0.00	0.00	0.00	0.00	0.00	0.00	0.00	4.00	0.00
0.00	0.00	0.00	0.00	0.00	20.00	0.00	0.00	0.00
0.00	0.00	0.00	0.00	0.00	0.00	0.00	0.00	0.00
0.00	0.00	0.00	0.00	0.00	0.00	0.00	0.00	0.00
0.00	0.00	0.00	0.00	0.00	0.00	0.00	0.00	0.00
0.00	0.00	0.00	0.00	0.00	0.00	0.00	0.00	0.00
0.00	0.00	0.00	0.00	0.00	0.00	0.00	0.00	0.00
0.00	0.00	0.00	0.00	0.00	0.00	0.00	0.00	0.00
0.00	0.00	0.00	0.00	0.00	0.00	0.00	0.00	0.00
0.00	0.00	0.00	0.00	0.00	0.00	0.00	0.00	0.00

0.00	0.00	0.00	0.00	0.00	0.00	0.00	0.00	0.00
0.00	0.00	0.00	0.00	0.00	0.00	0.00	0.00	0.00
5.00	0.00	5.00	7.00	0.00	10.00	0.00	6.00	0.00
0.00	0.00	3.00	0.00	0.00	0.00	0.00	3.00	0.00
0.00	0.00	0.00	0.00	0.00	0.00	0.00	0.00	0.00
0.00	0.00	0.00	0.00	0.00	0.00	0.00	0.00	0.00
0.00	0.00	0.00	0.00	0.00	0.00	0.00	0.00	0.00
0.00	0.00	0.00	0.00	0.00	0.00	0.00	0.00	0.00
0.00	0.00	0.00	0.00	0.00	0.00	0.00	0.00	0.00
0.00	0.00	0.00	0.00	0.00	0.00	0.00	0.00	0.00
0.00	0.00	8.00	0.00	0.00	0.00	0.00	0.00	0.00
0.00	0.00	0.00	0.00	0.00	0.00	0.00	0.00	0.00
0.00	0.00	0.00	0.00	0.00	0.00	0.00	0.00	0.00
0.00	0.00	0.00	0.00	0.00	0.00	0.00	0.00	0.00
3.00	0.00	0.00	0.00	0.00	3.00	0.00	0.00	0.00
0.00	0.00	0.00	0.00	0.00	6.00	0.00	0.00	0.00
0.00	0.00	0.00	0.00	0.00	0.00	0.00	0.00	0.00
0.00	0.00	0.00	0.00	0.00	0.00	0.00	0.00	0.00
0.00	0.00	0.00	0.00	0.00	0.00	0.00	0.00	0.00
0.00	0.00	0.00	0.00	0.00	0.00	0.00	0.00	0.00
0.00	0.00	0.00	0.00	0.00	0.00	0.00	0.00	0.00
0.00	0.00	0.00	51.00	0.00	0.00	0.00	0.00	0.00
0.00	0.00	0.00	0.00	0.00	0.00	0.00	0.00	0.00
0.00	0.00	0.00	0.00	0.00	0.00	0.00	0.00	0.00
0.00	0.00	0.00	0.00	0.00	0.00	0.00	0.00	0.00
0.00	0.00	0.00	0.00	0.00	0.00	0.00	0.00	0.00
0.00	0.00	0.00	0.00	0.00	0.00	0.00	0.00	0.00
0.00	0.00	0.00	0.00	0.00	0.00	0.00	0.00	0.00
0.00	0.00	0.00	50.00	0.00	0.00	0.00	0.00	0.00
0.00	0.00	0.00	0.00	0.00	0.00	0.00	0.00	0.00
0.00	0.00	50.00	0.00	0.00	0.00	0.00	0.00	0.00
0.00	0.00	0.00	0.00	0.00	0.00	0.00	0.00	0.00
0.00	0.00	0.00	0.00	0.00	0.00	0.00	0.00	0.00
0.00	0.00	0.00	0.00	0.00	0.00	0.00	0.00	0.00
0.00	0.00	0.00	0.00	0.00	0.00	0.00	0.00	0.00
0.00	0.00	11.00	0.00	0.00	0.00	0.00	0.00	0.00
0.00	0.00	0.00	0.00	0.00	0.00	0.00	0.00	0.00

0.00	0.00	0.00	0.00	0.00	0.00	0.00	0.00	0.00
0.00	0.00	0.00	0.00	0.00	0.00	0.00	0.00	0.00
0.00	0.00	0.00	0.00	0.00	0.00	0.00	0.00	0.00
0.00	0.00	0.00	7.00	0.00	13.00	0.00	7.00	4.00
0.00	0.00	0.00	6.00	0.00	0.00	0.00	0.00	0.00
0.00	0.00	0.00	0.00	0.00	0.00	0.00	0.00	0.00
0.00	0.00	0.00	0.00	0.00	0.00	0.00	0.00	0.00
0.00	0.00	0.00	32.00	0.00	0.00	0.00	0.00	0.00
0.00	0.00	0.00	0.00	0.00	0.00	0.00	0.00	0.00
0.00	0.00	0.00	48.00	0.00	0.00	0.00	0.00	0.00
0.00	0.00	0.00	0.00	0.00	0.00	0.00	0.00	0.00
0.00	0.00	0.00	0.00	0.00	0.00	0.00	0.00	0.00
0.00	0.00	0.00	0.00	0.00	0.00	0.00	0.00	0.00
0.00	0.00	0.00	0.00	0.00	0.00	0.00	0.00	0.00
0.00	0.00	8.00	0.00	0.00	0.00	0.00	7.00	0.00
8.00	0.00	0.00	5.00	0.00	0.00	0.00	0.00	0.00
0.00	0.00	0.00	0.00	0.00	0.00	0.00	0.00	0.00
0.00	0.00	0.00	0.00	0.00	0.00	0.00	0.00	0.00
0.00	0.00	0.00	0.00	0.00	0.00	0.00	0.00	0.00
0.00	0.00	39.00	0.00	0.00	0.00	0.00	0.00	0.00
0.00	0.00	0.00	0.00	0.00	0.00	0.00	0.00	0.00
0.00	0.00	0.00	0.00	0.00	0.00	0.00	0.00	0.00
0.00	0.00	0.00	0.00	0.00	0.00	0.00	0.00	0.00
0.00	0.00	0.00	0.00	0.00	0.00	0.00	0.00	0.00
0.00	0.00	0.00	0.00	0.00	0.00	0.00	0.00	0.00
0.00	0.00	0.00	3.00	0.00	8.00	0.00	4.00	0.00
0.00	0.00	0.00	0.00	0.00	0.00	0.00	0.00	0.00
0.00	0.00	0.00	0.00	0.00	0.00	0.00	0.00	0.00
0.00	0.00	0.00	0.00	0.00	0.00	0.00	0.00	0.00
0.00	0.00	0.00	46.00	0.00	0.00	0.00	0.00	0.00
0.00	0.00	0.00	0.00	0.00	0.00	0.00	0.00	0.00
0.00	0.00	0.00	0.00	0.00	0.00	0.00	0.00	0.00
0.00	0.00	0.00	0.00	0.00	0.00	0.00	0.00	0.00
0.00	0.00	0.00	0.00	0.00	0.00	0.00	0.00	0.00
0.00	0.00	0.00	0.00	0.00	0.00	0.00	0.00	0.00
0.00	0.00	0.00	0.00	0.00	0.00	0.00	0.00	0.00
0.00	0.00	0.00	0.00	0.00	0.00	0.00	0.00	0.00

0.00	0.00	0.00	0.00	0.00	0.00	0.00	0.00	0.00
0.00	0.00	0.00	0.00	0.00	0.00	0.00	0.00	0.00
14.00	0.00	0.00	0.00	0.00	0.00	0.00	0.00	0.00
0.00	0.00	0.00	0.00	0.00	0.00	0.00	0.00	0.00
0.00	0.00	0.00	0.00	0.00	0.00	0.00	0.00	0.00
0.00	0.00	0.00	0.00	0.00	0.00	0.00	0.00	0.00
0.00	0.00	0.00	0.00	0.00	0.00	0.00	0.00	0.00
0.00	0.00	0.00	0.00	0.00	5.00	0.00	0.00	0.00
0.00	0.00	0.00	0.00	0.00	0.00	0.00	13.00	0.00
0.00	0.00	0.00	0.00	0.00	0.00	0.00	0.00	0.00
0.00	0.00	0.00	44.00	0.00	0.00	0.00	0.00	0.00
0.00	0.00	0.00	0.00	0.00	0.00	0.00	0.00	0.00
0.00	0.00	0.00	0.00	0.00	0.00	0.00	0.00	0.00
0.00	0.00	0.00	0.00	0.00	0.00	0.00	0.00	0.00
0.00	0.00	0.00	0.00	0.00	0.00	0.00	0.00	0.00
0.00	0.00	0.00	0.00	6.00	0.00	0.00	2.00	0.00
0.00	0.00	0.00	0.00	0.00	0.00	0.00	0.00	0.00
9.00	0.00	0.00	18.00	0.00	0.00	0.00	0.00	4.00
0.00	0.00	0.00	0.00	0.00	0.00	7.00	0.00	0.00
0.00	0.00	0.00	43.00	0.00	0.00	0.00	0.00	0.00
0.00	0.00	0.00	0.00	0.00	0.00	0.00	0.00	0.00
0.00	0.00	0.00	0.00	0.00	0.00	0.00	0.00	0.00
0.00	0.00	0.00	0.00	0.00	0.00	0.00	0.00	0.00
0.00	0.00	0.00	0.00	0.00	0.00	0.00	0.00	0.00
0.00	0.00	0.00	0.00	0.00	0.00	0.00	0.00	0.00
0.00	0.00	0.00	0.00	0.00	10.00	0.00	0.00	0.00
0.00	0.00	5.00	4.00	0.00	9.00	0.00	8.00	0.00
0.00	0.00	0.00	0.00	0.00	0.00	0.00	0.00	0.00
0.00	0.00	0.00	0.00	0.00	0.00	0.00	0.00	0.00
0.00	0.00	0.00	0.00	0.00	0.00	0.00	0.00	0.00
0.00	0.00	0.00	0.00	0.00	0.00	0.00	0.00	0.00
0.00	0.00	0.00	0.00	0.00	0.00	0.00	0.00	0.00
0.00	0.00	0.00	0.00	0.00	0.00	0.00	0.00	0.00
0.00	0.00	0.00	0.00	0.00	0.00	0.00	0.00	0.00
0.00	0.00	0.00	0.00	0.00	0.00	0.00	0.00	0.00
0.00	0.00	0.00	0.00	0.00	0.00	0.00	0.00	0.00
0.00	0.00	0.00	0.00	0.00	0.00	0.00	0.00	0.00

0.00	0.00	0.00	0.00	0.00	0.00	0.00	0.00	0.00
0.00	0.00	0.00	0.00	0.00	0.00	0.00	0.00	0.00
5.00	0.00	11.00	0.00	0.00	0.00	0.00	0.00	0.00
0.00	0.00	0.00	0.00	0.00	0.00	0.00	0.00	0.00
0.00	0.00	0.00	0.00	0.00	0.00	0.00	0.00	0.00
0.00	0.00	0.00	0.00	0.00	0.00	0.00	0.00	0.00
0.00	0.00	0.00	0.00	0.00	0.00	0.00	0.00	0.00
0.00	0.00	0.00	40.00	0.00	0.00	0.00	0.00	0.00
0.00	0.00	0.00	0.00	0.00	0.00	0.00	0.00	0.00
0.00	0.00	0.00	0.00	0.00	0.00	0.00	0.00	0.00
0.00	0.00	0.00	0.00	0.00	0.00	0.00	0.00	0.00
0.00	0.00	0.00	0.00	0.00	0.00	0.00	0.00	0.00
0.00	0.00	0.00	0.00	0.00	0.00	0.00	0.00	0.00
0.00	0.00	0.00	0.00	0.00	0.00	0.00	0.00	0.00
0.00	0.00	0.00	9.00	0.00	0.00	0.00	0.00	0.00
0.00	0.00	0.00	0.00	0.00	0.00	0.00	0.00	0.00
0.00	0.00	0.00	39.00	0.00	0.00	0.00	0.00	0.00
0.00	0.00	0.00	39.00	0.00	0.00	0.00	0.00	0.00
0.00	0.00	0.00	39.00	0.00	0.00	0.00	0.00	0.00
0.00	0.00	0.00	39.00	0.00	0.00	0.00	0.00	0.00
0.00	0.00	6.00	0.00	0.00	0.00	0.00	0.00	0.00
0.00	0.00	0.00	0.00	3.00	0.00	0.00	4.00	0.00
0.00	0.00	0.00	0.00	0.00	0.00	0.00	0.00	0.00
0.00	0.00	8.00	0.00	0.00	5.00	0.00	0.00	0.00
0.00	0.00	0.00	0.00	0.00	0.00	0.00	0.00	0.00
0.00	0.00	0.00	0.00	0.00	0.00	0.00	0.00	0.00
0.00	0.00	0.00	0.00	0.00	0.00	0.00	0.00	0.00
0.00	0.00	0.00	0.00	0.00	0.00	0.00	0.00	0.00
0.00	0.00	0.00	0.00	0.00	0.00	0.00	0.00	0.00
0.00	0.00	0.00	0.00	0.00	0.00	0.00	0.00	0.00
0.00	0.00	5.00	4.00	3.00	0.00	0.00	0.00	0.00
0.00	0.00	0.00	0.00	0.00	0.00	0.00	0.00	0.00
0.00	0.00	0.00	3.00	0.00	35.00	0.00	0.00	0.00
0.00	0.00	0.00	0.00	0.00	0.00	0.00	0.00	0.00
0.00	0.00	0.00	0.00	0.00	0.00	0.00	0.00	0.00
0.00	0.00	0.00	0.00	0.00	0.00	0.00	0.00	0.00
0.00	0.00	0.00	0.00	0.00	0.00	0.00	0.00	0.00

0.00	0.00	0.00	0.00	0.00	0.00	0.00	0.00	0.00
0.00	0.00	0.00	0.00	0.00	0.00	0.00	0.00	0.00
0.00	0.00	0.00	0.00	0.00	0.00	0.00	0.00	0.00
0.00	0.00	0.00	0.00	0.00	3.00	0.00	0.00	0.00
0.00	0.00	0.00	0.00	0.00	0.00	0.00	0.00	0.00
0.00	0.00	0.00	37.00	0.00	0.00	0.00	0.00	0.00
0.00	0.00	0.00	0.00	0.00	0.00	0.00	0.00	0.00
2.00	0.00	0.00	2.00	0.00	0.00	0.00	0.00	0.00
0.00	0.00	0.00	0.00	0.00	0.00	0.00	0.00	0.00
0.00	0.00	0.00	0.00	0.00	0.00	0.00	0.00	0.00
0.00	0.00	0.00	0.00	0.00	6.00	0.00	0.00	0.00
0.00	0.00	0.00	36.00	0.00	0.00	0.00	0.00	0.00
0.00	0.00	0.00	36.00	0.00	0.00	0.00	0.00	0.00
0.00	0.00	0.00	36.00	0.00	0.00	0.00	0.00	0.00
0.00	0.00	0.00	36.00	0.00	0.00	0.00	0.00	0.00
0.00	0.00	0.00	0.00	0.00	0.00	0.00	0.00	0.00
0.00	0.00	0.00	0.00	0.00	0.00	0.00	0.00	0.00
0.00	0.00	0.00	0.00	0.00	0.00	0.00	0.00	0.00
0.00	0.00	0.00	0.00	0.00	0.00	0.00	0.00	0.00
0.00	0.00	0.00	0.00	0.00	0.00	0.00	0.00	0.00
0.00	0.00	0.00	6.00	0.00	11.00	0.00	8.00	0.00
0.00	0.00	0.00	0.00	0.00	0.00	0.00	0.00	0.00
0.00	0.00	0.00	35.00	0.00	0.00	0.00	0.00	0.00
0.00	0.00	0.00	0.00	0.00	0.00	0.00	0.00	0.00
20.00	0.00	0.00	0.00	0.00	0.00	0.00	0.00	0.00
0.00	0.00	0.00	0.00	0.00	0.00	0.00	0.00	0.00
0.00	0.00	0.00	0.00	0.00	0.00	0.00	0.00	0.00
0.00	0.00	0.00	0.00	0.00	0.00	0.00	0.00	0.00
0.00	0.00	0.00	4.00	0.00	4.00	0.00	11.00	0.00
0.00	0.00	0.00	0.00	0.00	34.00	0.00	0.00	0.00
0.00	0.00	0.00	0.00	0.00	0.00	0.00	0.00	0.00
0.00	0.00	0.00	0.00	0.00	0.00	0.00	0.00	0.00
0.00	0.00	0.00	0.00	0.00	0.00	0.00	0.00	0.00
0.00	0.00	0.00	3.00	0.00	0.00	0.00	5.00	0.00
0.00	0.00	0.00	0.00	0.00	0.00	0.00	0.00	0.00
0.00	0.00	0.00	0.00	0.00	13.00	0.00	0.00	0.00
0.00	0.00	0.00	0.00	0.00	0.00	0.00	0.00	0.00

0.00	0.00	0.00	0.00	0.00	0.00	0.00	0.00	0.00
0.00	0.00	0.00	0.00	0.00	0.00	0.00	0.00	0.00
0.00	0.00	0.00	0.00	0.00	0.00	0.00	0.00	0.00
0.00	0.00	0.00	0.00	33.00	0.00	0.00	0.00	0.00
0.00	0.00	0.00	0.00	0.00	0.00	0.00	0.00	0.00
0.00	0.00	0.00	0.00	0.00	0.00	0.00	0.00	0.00
0.00	0.00	0.00	0.00	0.00	0.00	0.00	0.00	0.00
0.00	0.00	0.00	0.00	0.00	0.00	0.00	0.00	0.00
0.00	0.00	0.00	0.00	0.00	0.00	0.00	0.00	0.00
0.00	0.00	0.00	0.00	0.00	0.00	0.00	0.00	0.00
0.00	0.00	0.00	0.00	0.00	0.00	0.00	0.00	0.00
0.00	0.00	0.00	0.00	0.00	0.00	0.00	0.00	0.00
0.00	0.00	0.00	0.00	9.00	0.00	0.00	0.00	0.00
0.00	0.00	0.00	0.00	0.00	0.00	0.00	0.00	0.00
0.00	0.00	0.00	0.00	0.00	0.00	0.00	0.00	0.00
0.00	0.00	0.00	0.00	0.00	0.00	0.00	0.00	0.00
0.00	0.00	0.00	0.00	0.00	0.00	0.00	0.00	0.00
0.00	0.00	0.00	0.00	0.00	0.00	0.00	0.00	0.00
0.00	0.00	0.00	0.00	0.00	0.00	0.00	0.00	0.00
0.00	0.00	0.00	0.00	0.00	0.00	0.00	0.00	0.00
0.00	0.00	0.00	0.00	0.00	0.00	0.00	0.00	0.00
0.00	0.00	0.00	0.00	0.00	0.00	0.00	0.00	0.00
0.00	0.00	0.00	0.00	0.00	0.00	0.00	0.00	0.00
0.00	0.00	0.00	0.00	0.00	0.00	0.00	0.00	0.00
0.00	0.00	0.00	0.00	0.00	0.00	0.00	0.00	0.00
0.00	0.00	0.00	0.00	0.00	0.00	0.00	0.00	0.00
0.00	0.00	0.00	0.00	0.00	0.00	0.00	0.00	0.00
0.00	0.00	0.00	0.00	0.00	0.00	0.00	0.00	0.00
0.00	0.00	0.00	0.00	0.00	0.00	0.00	0.00	0.00
0.00	0.00	10.00	0.00	0.00	0.00	0.00	0.00	0.00
0.00	0.00	0.00	0.00	0.00	0.00	0.00	0.00	0.00
0.00	0.00	0.00	0.00	0.00	0.00	0.00	0.00	0.00
0.00	0.00	0.00	0.00	0.00	0.00	0.00	0.00	0.00
0.00	0.00	0.00	0.00	0.00	0.00	0.00	0.00	0.00
0.00	0.00	0.00	0.00	0.00	0.00	0.00	0.00	0.00
0.00	0.00	0.00	0.00	0.00	0.00	0.00	0.00	0.00
0.00	0.00	0.00	0.00	0.00	0.00	0.00	0.00	0.00

0.00	0.00	0.00	0.00	0.00	0.00	0.00	0.00	0.00
0.00	0.00	0.00	0.00	0.00	0.00	0.00	0.00	0.00
0.00	0.00	0.00	0.00	0.00	0.00	0.00	0.00	0.00
0.00	0.00	0.00	0.00	10.00	0.00	0.00	0.00	0.00
0.00	0.00	0.00	0.00	0.00	0.00	0.00	0.00	0.00
0.00	0.00	0.00	0.00	0.00	5.00	0.00	0.00	0.00
0.00	0.00	0.00	0.00	0.00	8.00	0.00	0.00	0.00
0.00	0.00	0.00	0.00	0.00	0.00	0.00	0.00	0.00
0.00	0.00	0.00	0.00	6.00	0.00	0.00	7.00	0.00
0.00	0.00	0.00	30.00	0.00	0.00	0.00	0.00	0.00
0.00	0.00	0.00	30.00	0.00	0.00	0.00	0.00	0.00
0.00	0.00	0.00	0.00	0.00	0.00	0.00	0.00	0.00
0.00	0.00	0.00	0.00	0.00	0.00	0.00	0.00	0.00
0.00	0.00	0.00	0.00	0.00	0.00	0.00	0.00	0.00
0.00	0.00	0.00	0.00	0.00	0.00	0.00	0.00	0.00
0.00	0.00	0.00	0.00	0.00	0.00	0.00	0.00	0.00
0.00	0.00	0.00	0.00	0.00	0.00	0.00	0.00	0.00
0.00	0.00	6.00	0.00	0.00	0.00	0.00	0.00	0.00
0.00	0.00	0.00	0.00	0.00	0.00	0.00	0.00	0.00
4.00	0.00	0.00	0.00	0.00	0.00	0.00	0.00	0.00
0.00	0.00	0.00	0.00	0.00	5.00	0.00	0.00	0.00
0.00	0.00	0.00	9.00	0.00	6.00	0.00	0.00	0.00
0.00	0.00	11.00	0.00	0.00	0.00	0.00	0.00	0.00
0.00	0.00	0.00	0.00	29.00	0.00	0.00	0.00	0.00
0.00	0.00	29.00	0.00	0.00	0.00	0.00	0.00	0.00
0.00	0.00	0.00	0.00	0.00	0.00	0.00	0.00	0.00
0.00	0.00	0.00	0.00	0.00	0.00	0.00	0.00	0.00
0.00	0.00	0.00	0.00	0.00	0.00	0.00	0.00	0.00
0.00	0.00	0.00	0.00	0.00	0.00	0.00	0.00	0.00
0.00	0.00	0.00	0.00	0.00	0.00	0.00	0.00	0.00
0.00	0.00	0.00	0.00	0.00	0.00	0.00	0.00	0.00
0.00	0.00	0.00	0.00	0.00	0.00	0.00	0.00	0.00
0.00	0.00	0.00	0.00	0.00	0.00	0.00	0.00	0.00
0.00	0.00	0.00	0.00	0.00	0.00	0.00	0.00	0.00
0.00	0.00	0.00	0.00	0.00	0.00	0.00	0.00	0.00
0.00	0.00	0.00	0.00	0.00	0.00	0.00	0.00	0.00
0.00	0.00	0.00	0.00	0.00	0.00	0.00	0.00	0.00

0.00	0.00	0.00	0.00	0.00	2.00	0.00	0.00	0.00
0.00	0.00	0.00	0.00	0.00	0.00	0.00	0.00	0.00
0.00	0.00	0.00	0.00	0.00	0.00	0.00	0.00	0.00
0.00	0.00	0.00	0.00	0.00	0.00	0.00	0.00	0.00
0.00	0.00	0.00	0.00	0.00	0.00	0.00	0.00	0.00
0.00	0.00	0.00	0.00	0.00	0.00	0.00	0.00	0.00
0.00	0.00	0.00	0.00	0.00	0.00	0.00	0.00	0.00
3.00	0.00	0.00	0.00	0.00	0.00	0.00	0.00	0.00
0.00	0.00	0.00	0.00	0.00	0.00	0.00	0.00	0.00
0.00	0.00	4.00	0.00	0.00	0.00	0.00	0.00	0.00
0.00	0.00	0.00	0.00	0.00	0.00	0.00	0.00	0.00
0.00	0.00	0.00	0.00	0.00	7.00	0.00	0.00	0.00
0.00	0.00	0.00	0.00	0.00	0.00	0.00	0.00	0.00
0.00	0.00	0.00	0.00	0.00	0.00	0.00	0.00	0.00
0.00	0.00	0.00	0.00	27.00	0.00	0.00	0.00	0.00
0.00	0.00	0.00	27.00	0.00	0.00	0.00	0.00	0.00
0.00	0.00	0.00	27.00	0.00	0.00	0.00	0.00	0.00
0.00	0.00	0.00	0.00	0.00	0.00	0.00	0.00	0.00
0.00	0.00	17.00	0.00	0.00	0.00	0.00	0.00	0.00
0.00	0.00	0.00	0.00	0.00	0.00	0.00	0.00	0.00
0.00	0.00	0.00	0.00	0.00	0.00	0.00	0.00	0.00
0.00	0.00	0.00	0.00	0.00	0.00	0.00	0.00	0.00
0.00	0.00	0.00	0.00	0.00	0.00	0.00	0.00	0.00
0.00	0.00	0.00	0.00	0.00	0.00	0.00	0.00	0.00
0.00	0.00	0.00	0.00	0.00	0.00	0.00	0.00	0.00
0.00	0.00	0.00	0.00	0.00	0.00	0.00	0.00	0.00
0.00	0.00	0.00	0.00	0.00	0.00	0.00	0.00	0.00
0.00	0.00	10.00	6.00	0.00	0.00	0.00	0.00	0.00
0.00	0.00	0.00	0.00	0.00	0.00	0.00	0.00	26.00
0.00	0.00	0.00	26.00	0.00	0.00	0.00	0.00	0.00
0.00	0.00	0.00	0.00	0.00	0.00	0.00	0.00	0.00
0.00	0.00	0.00	0.00	0.00	0.00	0.00	0.00	0.00
0.00	0.00	0.00	0.00	0.00	0.00	0.00	0.00	0.00
0.00	0.00	0.00	0.00	0.00	0.00	0.00	0.00	0.00
0.00	0.00	0.00	0.00	0.00	0.00	0.00	0.00	0.00
0.00	0.00	0.00	0.00	0.00	0.00	0.00	0.00	0.00
0.00	0.00	0.00	0.00	0.00	0.00	0.00	0.00	0.00

0.00	0.00	0.00	0.00	0.00	0.00	0.00	0.00	0.00
0.00	0.00	0.00	0.00	0.00	0.00	0.00	0.00	0.00
0.00	0.00	0.00	0.00	0.00	0.00	0.00	0.00	0.00
0.00	0.00	0.00	0.00	0.00	0.00	0.00	0.00	0.00
0.00	0.00	0.00	0.00	0.00	0.00	0.00	0.00	0.00
0.00	0.00	0.00	0.00	0.00	0.00	0.00	0.00	0.00
0.00	0.00	0.00	0.00	0.00	0.00	0.00	0.00	0.00
0.00	0.00	0.00	0.00	0.00	0.00	0.00	0.00	0.00
0.00	0.00	0.00	0.00	0.00	25.00	0.00	0.00	0.00
0.00	0.00	0.00	25.00	0.00	0.00	0.00	0.00	0.00
0.00	0.00	0.00	25.00	0.00	0.00	0.00	0.00	0.00
0.00	0.00	0.00	0.00	0.00	0.00	0.00	0.00	0.00
0.00	0.00	0.00	0.00	0.00	0.00	0.00	0.00	0.00
0.00	0.00	0.00	0.00	0.00	0.00	0.00	0.00	0.00
0.00	0.00	0.00	0.00	0.00	0.00	0.00	0.00	0.00
0.00	0.00	0.00	0.00	0.00	0.00	0.00	0.00	0.00
0.00	0.00	0.00	0.00	0.00	0.00	0.00	0.00	0.00
0.00	0.00	0.00	0.00	0.00	0.00	0.00	0.00	0.00
0.00	0.00	0.00	0.00	0.00	0.00	0.00	0.00	0.00
0.00	0.00	0.00	0.00	0.00	0.00	0.00	0.00	0.00
0.00	0.00	0.00	0.00	0.00	0.00	0.00	0.00	0.00
0.00	0.00	4.00	0.00	0.00	0.00	0.00	0.00	0.00
0.00	0.00	0.00	0.00	0.00	0.00	0.00	0.00	0.00
0.00	0.00	0.00	0.00	0.00	0.00	0.00	0.00	0.00
0.00	0.00	8.00	0.00	0.00	0.00	0.00	0.00	0.00
0.00	0.00	0.00	6.00	0.00	0.00	0.00	0.00	0.00
0.00	0.00	0.00	0.00	0.00	0.00	0.00	0.00	0.00
0.00	0.00	0.00	0.00	0.00	0.00	0.00	0.00	0.00
0.00	0.00	0.00	0.00	0.00	0.00	0.00	0.00	0.00
0.00	0.00	0.00	0.00	0.00	0.00	0.00	0.00	0.00
0.00	0.00	0.00	0.00	0.00	0.00	0.00	0.00	0.00
0.00	0.00	0.00	0.00	0.00	0.00	0.00	0.00	0.00
0.00	0.00	0.00	0.00	0.00	0.00	0.00	0.00	0.00
0.00	0.00	0.00	10.00	0.00	0.00	0.00	5.00	0.00
0.00	0.00	0.00	0.00	0.00	0.00	0.00	0.00	0.00
0.00	0.00	0.00	0.00	4.00	4.00	0.00	0.00	0.00
5.00	0.00	0.00	0.00	0.00	0.00	0.00	0.00	0.00

0.00	0.00	0.00	0.00	0.00	0.00	0.00	0.00	0.00
12.00	0.00	0.00	0.00	0.00	0.00	0.00	0.00	5.00
0.00	0.00	0.00	23.00	0.00	0.00	0.00	0.00	0.00
0.00	0.00	0.00	0.00	0.00	0.00	0.00	0.00	0.00
0.00	0.00	0.00	0.00	0.00	0.00	0.00	0.00	0.00
0.00	0.00	0.00	0.00	0.00	0.00	0.00	0.00	0.00
0.00	0.00	0.00	0.00	0.00	0.00	0.00	0.00	0.00
0.00	0.00	0.00	0.00	0.00	0.00	0.00	0.00	0.00
0.00	0.00	0.00	0.00	0.00	0.00	0.00	0.00	0.00
0.00	0.00	0.00	0.00	0.00	0.00	0.00	0.00	0.00
0.00	0.00	0.00	0.00	0.00	0.00	0.00	0.00	0.00
0.00	0.00	0.00	0.00	0.00	0.00	0.00	0.00	0.00
5.00	0.00	0.00	0.00	0.00	0.00	0.00	0.00	0.00
0.00	0.00	0.00	0.00	0.00	0.00	0.00	10.00	0.00
0.00	0.00	0.00	0.00	0.00	0.00	0.00	0.00	0.00
0.00	0.00	0.00	0.00	0.00	0.00	0.00	0.00	0.00
0.00	0.00	0.00	0.00	0.00	0.00	0.00	0.00	2.00
0.00	0.00	0.00	0.00	0.00	3.00	0.00	0.00	0.00
0.00	0.00	0.00	0.00	0.00	0.00	0.00	0.00	0.00
0.00	0.00	0.00	0.00	0.00	0.00	0.00	0.00	0.00
0.00	0.00	8.00	0.00	0.00	0.00	0.00	0.00	0.00
0.00	0.00	0.00	0.00	0.00	0.00	0.00	0.00	0.00
0.00	0.00	0.00	0.00	0.00	0.00	0.00	0.00	0.00
0.00	0.00	0.00	0.00	0.00	0.00	0.00	0.00	0.00
0.00	0.00	19.00	0.00	0.00	0.00	0.00	0.00	0.00
0.00	0.00	0.00	0.00	0.00	0.00	0.00	0.00	0.00
0.00	0.00	0.00	0.00	0.00	0.00	0.00	0.00	0.00
0.00	0.00	0.00	0.00	0.00	0.00	0.00	0.00	0.00
0.00	0.00	0.00	0.00	0.00	0.00	0.00	0.00	0.00
0.00	0.00	0.00	0.00	0.00	0.00	0.00	0.00	0.00
0.00	0.00	0.00	0.00	0.00	0.00	0.00	0.00	0.00
0.00	0.00	0.00	0.00	0.00	0.00	0.00	0.00	0.00
0.00	0.00	0.00	0.00	0.00	0.00	0.00	0.00	0.00
0.00	0.00	0.00	0.00	0.00	0.00	0.00	0.00	0.00
0.00	0.00	0.00	0.00	0.00	0.00	0.00	0.00	0.00
8.00	0.00	0.00	0.00	0.00	0.00	0.00	0.00	0.00
0.00	0.00	0.00	0.00	0.00	0.00	0.00	0.00	0.00

0.00	0.00	0.00	0.00	0.00	0.00	0.00	0.00	0.00
0.00	0.00	10.00	0.00	0.00	0.00	0.00	0.00	0.00
0.00	0.00	0.00	0.00	0.00	0.00	0.00	0.00	0.00
0.00	0.00	0.00	0.00	0.00	0.00	0.00	0.00	0.00
0.00	0.00	0.00	0.00	0.00	0.00	0.00	0.00	0.00
0.00	0.00	0.00	0.00	0.00	0.00	0.00	0.00	0.00
0.00	0.00	0.00	21.00	0.00	0.00	0.00	0.00	0.00
0.00	0.00	0.00	0.00	0.00	0.00	0.00	0.00	0.00
11.00	0.00	0.00	0.00	0.00	0.00	0.00	0.00	0.00
0.00	0.00	0.00	0.00	0.00	0.00	0.00	0.00	0.00
0.00	0.00	0.00	0.00	0.00	0.00	0.00	0.00	0.00
0.00	0.00	0.00	0.00	0.00	0.00	0.00	0.00	0.00
0.00	0.00	0.00	0.00	0.00	0.00	0.00	0.00	0.00
0.00	0.00	0.00	0.00	0.00	0.00	0.00	0.00	0.00
0.00	0.00	0.00	0.00	0.00	0.00	0.00	0.00	0.00
0.00	0.00	0.00	0.00	0.00	0.00	0.00	0.00	0.00
0.00	0.00	0.00	0.00	0.00	0.00	0.00	0.00	0.00
0.00	0.00	0.00	0.00	0.00	0.00	0.00	0.00	0.00
0.00	0.00	0.00	0.00	0.00	0.00	0.00	2.00	0.00
0.00	0.00	0.00	0.00	0.00	0.00	0.00	0.00	0.00
0.00	0.00	0.00	0.00	0.00	0.00	0.00	0.00	0.00
8.00	0.00	0.00	0.00	0.00	0.00	0.00	0.00	3.00
0.00	0.00	0.00	0.00	0.00	0.00	0.00	0.00	0.00
0.00	0.00	0.00	20.00	0.00	0.00	0.00	0.00	0.00
0.00	0.00	0.00	20.00	0.00	0.00	0.00	0.00	0.00
0.00	0.00	0.00	20.00	0.00	0.00	0.00	0.00	0.00
0.00	0.00	0.00	0.00	0.00	0.00	0.00	0.00	0.00
0.00	0.00	0.00	0.00	0.00	0.00	0.00	0.00	0.00
0.00	0.00	13.00	0.00	0.00	0.00	0.00	0.00	0.00
0.00	0.00	0.00	0.00	0.00	0.00	0.00	0.00	0.00
0.00	0.00	0.00	0.00	0.00	0.00	0.00	0.00	0.00
0.00	0.00	0.00	0.00	0.00	0.00	0.00	0.00	0.00
0.00	0.00	0.00	0.00	0.00	0.00	0.00	0.00	0.00
0.00	0.00	20.00	0.00	0.00	0.00	0.00	0.00	0.00
0.00	0.00	20.00	0.00	0.00	0.00	0.00	0.00	0.00
0.00	0.00	0.00	0.00	0.00	0.00	0.00	0.00	0.00
0.00	0.00	0.00	0.00	0.00	0.00	0.00	0.00	0.00

0.00	0.00	0.00	0.00	0.00	0.00	0.00	0.00	0.00
0.00	0.00	0.00	0.00	0.00	0.00	0.00	0.00	0.00
0.00	0.00	0.00	0.00	0.00	0.00	0.00	0.00	0.00
0.00	0.00	0.00	0.00	0.00	0.00	0.00	0.00	0.00
0.00	0.00	0.00	0.00	0.00	6.00	0.00	0.00	0.00
0.00	0.00	0.00	0.00	0.00	0.00	0.00	0.00	0.00
0.00	0.00	0.00	0.00	0.00	0.00	0.00	0.00	0.00
0.00	0.00	0.00	0.00	0.00	0.00	0.00	0.00	0.00
7.00	0.00	0.00	0.00	0.00	0.00	0.00	5.00	0.00
0.00	0.00	0.00	0.00	0.00	0.00	0.00	0.00	0.00
7.00	0.00	0.00	0.00	0.00	0.00	0.00	0.00	0.00
0.00	0.00	0.00	0.00	0.00	0.00	0.00	0.00	0.00
0.00	0.00	0.00	0.00	0.00	6.00	0.00	0.00	0.00
0.00	0.00	0.00	0.00	19.00	0.00	0.00	0.00	0.00
0.00	0.00	0.00	19.00	0.00	0.00	0.00	0.00	0.00
0.00	0.00	0.00	19.00	0.00	0.00	0.00	0.00	0.00
0.00	0.00	0.00	0.00	0.00	0.00	0.00	0.00	0.00
0.00	0.00	0.00	0.00	0.00	0.00	0.00	0.00	0.00
0.00	0.00	0.00	0.00	0.00	0.00	0.00	0.00	0.00
0.00	0.00	0.00	0.00	0.00	0.00	0.00	0.00	0.00
0.00	0.00	0.00	0.00	0.00	0.00	0.00	0.00	0.00
0.00	0.00	0.00	0.00	0.00	0.00	0.00	0.00	0.00
0.00	0.00	0.00	0.00	0.00	0.00	0.00	0.00	0.00
0.00	0.00	0.00	0.00	0.00	0.00	0.00	0.00	0.00
0.00	0.00	0.00	0.00	0.00	0.00	0.00	0.00	0.00
0.00	0.00	0.00	0.00	0.00	0.00	0.00	0.00	0.00
0.00	0.00	0.00	0.00	0.00	0.00	0.00	0.00	0.00
0.00	0.00	0.00	0.00	0.00	0.00	0.00	0.00	0.00
0.00	0.00	0.00	0.00	0.00	0.00	0.00	0.00	0.00
0.00	0.00	0.00	0.00	0.00	0.00	0.00	0.00	0.00
0.00	0.00	0.00	0.00	0.00	0.00	0.00	0.00	0.00
0.00	0.00	0.00	0.00	0.00	0.00	0.00	0.00	0.00
0.00	0.00	0.00	0.00	0.00	0.00	0.00	0.00	0.00
0.00	0.00	0.00	0.00	0.00	0.00	0.00	0.00	0.00
0.00	0.00	0.00	0.00	0.00	0.00	0.00	0.00	0.00
0.00	0.00	0.00	0.00	0.00	0.00	0.00	0.00	0.00
0.00	0.00	0.00	0.00	0.00	0.00	0.00	4.00	0.00

0.00	0.00	0.00	0.00	0.00	0.00	0.00	0.00	0.00
0.00	0.00	0.00	0.00	0.00	0.00	0.00	0.00	0.00
0.00	0.00	0.00	0.00	0.00	0.00	0.00	0.00	0.00
0.00	0.00	0.00	0.00	0.00	0.00	0.00	0.00	0.00
0.00	0.00	0.00	0.00	0.00	0.00	0.00	0.00	0.00
0.00	0.00	0.00	0.00	0.00	0.00	0.00	0.00	0.00
0.00	0.00	0.00	0.00	0.00	0.00	0.00	0.00	0.00
0.00	0.00	0.00	0.00	0.00	0.00	0.00	0.00	0.00
0.00	0.00	0.00	0.00	0.00	0.00	0.00	0.00	0.00
0.00	0.00	0.00	0.00	18.00	0.00	0.00	0.00	0.00
0.00	0.00	0.00	18.00	0.00	0.00	0.00	0.00	0.00
0.00	0.00	0.00	18.00	0.00	0.00	0.00	0.00	0.00
0.00	0.00	0.00	0.00	0.00	0.00	0.00	0.00	0.00
0.00	0.00	0.00	0.00	0.00	0.00	0.00	0.00	0.00
0.00	0.00	0.00	0.00	0.00	0.00	0.00	0.00	0.00
0.00	0.00	0.00	0.00	0.00	0.00	0.00	0.00	0.00
0.00	0.00	0.00	0.00	0.00	0.00	0.00	0.00	0.00
0.00	0.00	0.00	0.00	0.00	0.00	0.00	0.00	0.00
0.00	0.00	0.00	0.00	0.00	0.00	0.00	0.00	0.00
6.00	0.00	0.00	0.00	0.00	0.00	0.00	0.00	0.00
0.00	0.00	0.00	0.00	0.00	0.00	0.00	0.00	0.00
0.00	0.00	0.00	0.00	0.00	0.00	0.00	0.00	0.00
0.00	0.00	0.00	0.00	0.00	0.00	0.00	0.00	0.00
0.00	0.00	0.00	0.00	0.00	0.00	0.00	0.00	0.00
0.00	0.00	0.00	0.00	0.00	0.00	0.00	0.00	2.00
0.00	0.00	0.00	0.00	0.00	0.00	0.00	0.00	0.00
0.00	0.00	0.00	0.00	0.00	0.00	0.00	0.00	0.00
0.00	0.00	0.00	0.00	0.00	0.00	0.00	0.00	0.00
0.00	0.00	0.00	0.00	0.00	0.00	0.00	0.00	0.00
0.00	0.00	0.00	0.00	0.00	0.00	0.00	0.00	0.00
0.00	0.00	0.00	0.00	0.00	0.00	0.00	0.00	0.00
0.00	0.00	0.00	6.00	0.00	0.00	0.00	0.00	0.00
0.00	0.00	0.00	0.00	0.00	0.00	0.00	0.00	0.00
0.00	0.00	0.00	0.00	0.00	0.00	0.00	0.00	0.00
0.00	0.00	0.00	0.00	0.00	0.00	0.00	0.00	0.00
0.00	0.00	0.00	0.00	0.00	8.00	0.00	0.00	0.00
0.00	0.00	0.00	0.00	6.00	0.00	0.00	0.00	0.00

0.00	0.00	0.00	17.00	0.00	0.00	0.00	0.00	0.00
0.00	0.00	0.00	17.00	0.00	0.00	0.00	0.00	0.00
0.00	0.00	0.00	17.00	0.00	0.00	0.00	0.00	0.00
0.00	0.00	0.00	0.00	0.00	0.00	0.00	0.00	0.00
0.00	0.00	0.00	0.00	0.00	0.00	0.00	0.00	0.00
0.00	0.00	0.00	0.00	0.00	0.00	0.00	0.00	0.00
0.00	0.00	0.00	0.00	0.00	0.00	0.00	0.00	0.00
0.00	0.00	0.00	0.00	0.00	0.00	0.00	0.00	0.00
0.00	0.00	0.00	0.00	0.00	0.00	0.00	0.00	0.00
10.00	0.00	7.00	0.00	0.00	0.00	0.00	0.00	0.00
0.00	0.00	0.00	0.00	0.00	0.00	0.00	0.00	0.00
0.00	0.00	0.00	0.00	0.00	0.00	0.00	0.00	0.00
0.00	0.00	0.00	0.00	0.00	0.00	0.00	0.00	0.00
0.00	0.00	0.00	0.00	0.00	0.00	0.00	0.00	0.00
0.00	0.00	0.00	0.00	0.00	0.00	0.00	0.00	0.00
0.00	0.00	0.00	0.00	0.00	0.00	0.00	0.00	0.00
0.00	0.00	0.00	0.00	0.00	0.00	0.00	0.00	0.00
0.00	0.00	0.00	0.00	0.00	0.00	0.00	0.00	0.00
0.00	0.00	0.00	0.00	0.00	0.00	0.00	0.00	0.00
0.00	0.00	0.00	0.00	0.00	0.00	0.00	0.00	0.00
0.00	0.00	0.00	0.00	0.00	0.00	0.00	0.00	0.00
0.00	0.00	0.00	0.00	0.00	0.00	0.00	0.00	0.00
0.00	0.00	0.00	0.00	0.00	0.00	0.00	0.00	0.00
0.00	0.00	0.00	0.00	0.00	0.00	0.00	0.00	0.00
0.00	0.00	0.00	0.00	0.00	0.00	0.00	5.00	0.00
0.00	0.00	0.00	0.00	0.00	0.00	0.00	0.00	0.00
0.00	0.00	0.00	6.00	0.00	0.00	0.00	0.00	0.00
0.00	0.00	0.00	0.00	0.00	6.00	0.00	0.00	0.00
0.00	0.00	0.00	0.00	0.00	0.00	0.00	0.00	0.00
0.00	0.00	3.00	0.00	0.00	6.00	0.00	0.00	0.00
0.00	0.00	0.00	0.00	0.00	16.00	0.00	0.00	0.00
0.00	0.00	0.00	14.00	0.00	2.00	0.00	0.00	0.00
0.00	0.00	0.00	16.00	0.00	0.00	0.00	0.00	0.00
0.00	0.00	0.00	16.00	0.00	0.00	0.00	0.00	0.00
0.00	0.00	0.00	16.00	0.00	0.00	0.00	0.00	0.00
0.00	0.00	0.00	0.00	0.00	0.00	0.00	0.00	0.00
0.00	0.00	0.00	0.00	0.00	0.00	0.00	0.00	0.00

0.00	0.00	0.00	0.00	0.00	0.00	0.00	0.00	0.00
0.00	0.00	0.00	0.00	0.00	0.00	0.00	0.00	0.00
0.00	0.00	0.00	0.00	0.00	0.00	0.00	0.00	0.00
0.00	0.00	0.00	0.00	0.00	0.00	0.00	0.00	0.00
0.00	0.00	0.00	0.00	0.00	0.00	0.00	0.00	0.00
0.00	0.00	0.00	0.00	0.00	0.00	0.00	0.00	0.00
0.00	0.00	0.00	0.00	0.00	0.00	0.00	0.00	0.00
0.00	0.00	0.00	0.00	0.00	0.00	0.00	0.00	0.00
0.00	0.00	0.00	0.00	0.00	0.00	0.00	0.00	0.00
0.00	0.00	0.00	0.00	0.00	0.00	0.00	0.00	0.00
0.00	0.00	0.00	0.00	0.00	0.00	0.00	0.00	0.00
0.00	0.00	0.00	0.00	0.00	0.00	0.00	0.00	0.00
0.00	0.00	0.00	0.00	0.00	0.00	0.00	0.00	0.00
0.00	0.00	0.00	0.00	0.00	0.00	0.00	0.00	0.00
0.00	0.00	0.00	0.00	0.00	0.00	0.00	0.00	0.00
0.00	0.00	0.00	0.00	0.00	0.00	0.00	0.00	0.00
0.00	0.00	0.00	0.00	0.00	0.00	0.00	0.00	0.00
0.00	0.00	0.00	0.00	0.00	0.00	0.00	0.00	0.00
0.00	0.00	0.00	0.00	0.00	0.00	0.00	0.00	0.00
0.00	0.00	0.00	0.00	0.00	0.00	0.00	0.00	0.00
0.00	0.00	0.00	0.00	0.00	0.00	0.00	0.00	0.00
0.00	0.00	0.00	0.00	0.00	0.00	0.00	0.00	0.00
0.00	0.00	0.00	0.00	0.00	0.00	0.00	0.00	0.00
0.00	0.00	0.00	0.00	0.00	0.00	0.00	0.00	0.00
0.00	0.00	0.00	0.00	0.00	0.00	0.00	0.00	0.00
0.00	0.00	0.00	0.00	0.00	6.00	0.00	0.00	0.00
4.00	0.00	6.00	0.00	0.00	0.00	0.00	0.00	0.00
0.00	0.00	0.00	0.00	0.00	0.00	0.00	0.00	0.00
0.00	0.00	0.00	0.00	0.00	0.00	0.00	0.00	0.00
0.00	0.00	0.00	0.00	0.00	0.00	0.00	6.00	0.00
0.00	0.00	0.00	0.00	0.00	0.00	0.00	0.00	0.00
0.00	0.00	0.00	0.00	0.00	0.00	0.00	0.00	0.00
0.00	0.00	0.00	0.00	0.00	0.00	0.00	0.00	0.00
0.00	0.00	0.00	0.00	0.00	0.00	0.00	0.00	0.00
0.00	0.00	0.00	0.00	0.00	0.00	0.00	0.00	0.00
0.00	0.00	0.00	0.00	0.00	0.00	0.00	0.00	0.00
0.00	0.00	0.00	0.00	0.00	0.00	0.00	0.00	0.00

0.00	0.00	0.00	0.00	0.00	0.00	0.00	0.00	0.00
0.00	0.00	0.00	0.00	0.00	0.00	0.00	0.00	0.00
0.00	0.00	0.00	0.00	0.00	0.00	0.00	0.00	0.00
0.00	0.00	0.00	0.00	0.00	0.00	0.00	0.00	0.00
0.00	0.00	0.00	0.00	0.00	0.00	0.00	0.00	0.00
0.00	0.00	0.00	0.00	0.00	2.00	0.00	0.00	0.00
0.00	0.00	0.00	0.00	0.00	0.00	0.00	0.00	0.00
0.00	0.00	0.00	0.00	0.00	0.00	0.00	0.00	0.00
0.00	0.00	0.00	0.00	0.00	0.00	14.00	0.00	0.00
0.00	0.00	0.00	0.00	14.00	0.00	0.00	0.00	0.00
0.00	0.00	0.00	2.00	0.00	0.00	0.00	0.00	0.00
0.00	0.00	0.00	7.00	0.00	0.00	0.00	5.00	0.00
8.00	0.00	0.00	6.00	0.00	0.00	0.00	0.00	0.00
0.00	0.00	0.00	0.00	0.00	0.00	0.00	0.00	0.00
0.00	0.00	0.00	0.00	0.00	0.00	0.00	0.00	0.00
0.00	0.00	0.00	0.00	0.00	0.00	0.00	0.00	0.00
0.00	0.00	0.00	0.00	0.00	0.00	0.00	0.00	0.00
0.00	0.00	14.00	0.00	0.00	0.00	0.00	0.00	0.00
0.00	0.00	0.00	0.00	0.00	0.00	0.00	0.00	0.00
0.00	0.00	0.00	0.00	0.00	0.00	0.00	0.00	0.00
0.00	0.00	0.00	0.00	0.00	0.00	0.00	0.00	0.00
0.00	0.00	0.00	0.00	0.00	0.00	0.00	0.00	0.00
0.00	0.00	0.00	0.00	0.00	0.00	0.00	0.00	0.00
0.00	0.00	0.00	0.00	0.00	0.00	0.00	0.00	0.00
0.00	0.00	0.00	0.00	0.00	0.00	0.00	0.00	0.00
0.00	0.00	0.00	0.00	0.00	0.00	0.00	0.00	0.00
0.00	0.00	0.00	0.00	0.00	0.00	0.00	0.00	0.00
0.00	0.00	0.00	0.00	0.00	0.00	0.00	0.00	0.00
0.00	0.00	0.00	0.00	0.00	0.00	0.00	0.00	0.00
0.00	0.00	0.00	0.00	0.00	0.00	0.00	0.00	0.00
0.00	0.00	0.00	0.00	0.00	0.00	0.00	0.00	0.00
0.00	0.00	0.00	0.00	0.00	0.00	0.00	0.00	0.00
0.00	0.00	0.00	0.00	0.00	0.00	0.00	0.00	0.00
0.00	0.00	0.00	0.00	0.00	0.00	0.00	0.00	0.00
0.00	0.00	0.00	0.00	0.00	0.00	0.00	0.00	0.00
0.00	0.00	0.00	0.00	0.00	0.00	0.00	0.00	0.00
0.00	0.00	0.00	0.00	0.00	0.00	4.00	0.00	0.00

0.00	0.00	0.00	0.00	0.00	0.00	0.00	0.00	0.00
0.00	0.00	0.00	0.00	0.00	0.00	0.00	0.00	0.00
0.00	0.00	0.00	0.00	0.00	0.00	0.00	0.00	0.00
0.00	0.00	0.00	0.00	0.00	8.00	0.00	0.00	0.00
0.00	0.00	0.00	0.00	0.00	4.00	0.00	0.00	0.00
0.00	0.00	0.00	0.00	0.00	0.00	0.00	0.00	0.00
0.00	0.00	0.00	0.00	0.00	3.00	0.00	0.00	0.00
0.00	0.00	0.00	0.00	0.00	0.00	0.00	0.00	0.00
0.00	0.00	0.00	0.00	0.00	0.00	0.00	0.00	0.00
0.00	0.00	0.00	0.00	0.00	13.00	0.00	0.00	0.00
0.00	0.00	0.00	13.00	0.00	0.00	0.00	0.00	0.00
0.00	0.00	0.00	13.00	0.00	0.00	0.00	0.00	0.00
0.00	0.00	0.00	0.00	0.00	0.00	0.00	0.00	0.00
0.00	0.00	0.00	0.00	0.00	0.00	0.00	0.00	0.00
0.00	0.00	0.00	0.00	0.00	0.00	0.00	6.00	0.00
0.00	0.00	0.00	0.00	0.00	0.00	0.00	0.00	0.00
0.00	0.00	0.00	0.00	0.00	0.00	0.00	0.00	0.00
0.00	0.00	0.00	0.00	0.00	0.00	0.00	0.00	0.00
0.00	0.00	0.00	0.00	0.00	0.00	0.00	0.00	0.00
0.00	0.00	0.00	0.00	0.00	0.00	0.00	0.00	0.00
0.00	0.00	0.00	0.00	0.00	0.00	0.00	0.00	0.00
0.00	0.00	0.00	0.00	0.00	0.00	0.00	0.00	0.00
0.00	0.00	0.00	0.00	0.00	0.00	0.00	0.00	0.00
0.00	0.00	0.00	0.00	0.00	0.00	0.00	0.00	0.00
0.00	0.00	0.00	0.00	0.00	0.00	0.00	0.00	0.00
0.00	0.00	0.00	0.00	0.00	0.00	0.00	0.00	0.00
0.00	0.00	0.00	0.00	0.00	0.00	0.00	0.00	0.00
0.00	0.00	0.00	0.00	0.00	0.00	0.00	0.00	0.00
0.00	0.00	0.00	0.00	0.00	0.00	0.00	0.00	0.00
0.00	0.00	0.00	0.00	0.00	0.00	0.00	0.00	0.00
0.00	0.00	0.00	0.00	0.00	0.00	0.00	0.00	0.00
0.00	0.00	0.00	0.00	0.00	0.00	0.00	0.00	0.00
0.00	0.00	0.00	0.00	0.00	0.00	0.00	0.00	0.00
0.00	0.00	0.00	0.00	0.00	0.00	0.00	0.00	0.00
0.00	0.00	0.00	0.00	0.00	0.00	0.00	0.00	0.00
0.00	0.00	0.00	0.00	0.00	0.00	0.00	0.00	0.00
0.00	0.00	0.00	0.00	0.00	0.00	0.00	0.00	0.00

0.00	0.00	0.00	0.00	0.00	0.00	0.00	0.00	0.00
0.00	0.00	0.00	0.00	0.00	0.00	0.00	0.00	0.00
0.00	0.00	0.00	0.00	0.00	4.00	0.00	0.00	0.00
0.00	0.00	4.00	0.00	0.00	3.00	0.00	0.00	0.00
0.00	0.00	0.00	0.00	0.00	4.00	0.00	0.00	0.00
0.00	0.00	0.00	0.00	0.00	0.00	0.00	0.00	0.00
0.00	0.00	0.00	0.00	0.00	0.00	0.00	0.00	0.00
0.00	0.00	0.00	0.00	0.00	8.00	0.00	0.00	0.00
0.00	0.00	0.00	0.00	0.00	0.00	0.00	0.00	0.00
0.00	0.00	0.00	0.00	0.00	6.00	0.00	0.00	0.00
0.00	0.00	0.00	0.00	0.00	0.00	0.00	0.00	0.00
0.00	0.00	0.00	0.00	0.00	0.00	0.00	0.00	0.00
0.00	0.00	0.00	0.00	0.00	0.00	0.00	0.00	0.00
0.00	0.00	0.00	0.00	0.00	0.00	0.00	0.00	0.00
0.00	0.00	0.00	0.00	0.00	0.00	0.00	0.00	0.00
0.00	0.00	0.00	0.00	0.00	0.00	0.00	0.00	0.00
3.00	0.00	0.00	0.00	0.00	0.00	0.00	0.00	0.00
0.00	0.00	0.00	0.00	12.00	0.00	0.00	0.00	0.00
0.00	0.00	0.00	0.00	12.00	0.00	0.00	0.00	0.00
0.00	0.00	0.00	12.00	0.00	0.00	0.00	0.00	0.00
0.00	0.00	0.00	0.00	0.00	0.00	0.00	0.00	0.00
0.00	0.00	0.00	0.00	0.00	0.00	0.00	0.00	0.00
0.00	0.00	0.00	0.00	0.00	0.00	0.00	0.00	0.00
0.00	0.00	0.00	0.00	0.00	0.00	0.00	12.00	0.00
0.00	0.00	0.00	0.00	0.00	0.00	0.00	0.00	0.00
0.00	0.00	12.00	0.00	0.00	0.00	0.00	0.00	0.00
0.00	12.00	0.00	0.00	0.00	0.00	0.00	0.00	0.00
12.00	0.00	0.00	0.00	0.00	0.00	0.00	0.00	0.00
0.00	0.00	0.00	0.00	0.00	0.00	0.00	0.00	0.00
0.00	0.00	0.00	0.00	0.00	0.00	0.00	0.00	0.00
0.00	0.00	0.00	0.00	0.00	0.00	0.00	0.00	0.00
0.00	0.00	0.00	0.00	0.00	0.00	0.00	0.00	0.00
0.00	0.00	0.00	0.00	0.00	0.00	0.00	0.00	0.00
0.00	0.00	0.00	0.00	0.00	0.00	0.00	0.00	0.00
0.00	0.00	0.00	0.00	0.00	0.00	0.00	0.00	0.00
0.00	0.00	0.00	0.00	0.00	0.00	0.00	0.00	0.00
0.00	0.00	0.00	0.00	0.00	0.00	0.00	0.00	0.00

0.00	0.00	0.00	0.00	0.00	0.00	0.00	0.00	0.00
0.00	0.00	0.00	0.00	0.00	0.00	0.00	0.00	0.00
0.00	0.00	0.00	0.00	0.00	4.00	0.00	0.00	0.00
0.00	0.00	0.00	0.00	0.00	0.00	0.00	0.00	0.00
0.00	0.00	0.00	0.00	0.00	0.00	0.00	0.00	0.00
0.00	0.00	0.00	0.00	0.00	0.00	0.00	0.00	0.00
0.00	0.00	0.00	0.00	0.00	0.00	0.00	0.00	0.00
0.00	0.00	0.00	0.00	0.00	0.00	0.00	0.00	0.00
0.00	0.00	0.00	0.00	0.00	0.00	0.00	0.00	0.00
0.00	0.00	0.00	0.00	0.00	0.00	0.00	0.00	0.00
0.00	0.00	0.00	0.00	0.00	0.00	0.00	0.00	0.00
0.00	0.00	0.00	0.00	0.00	0.00	0.00	0.00	0.00
0.00	0.00	0.00	0.00	0.00	0.00	0.00	4.00	0.00
0.00	0.00	0.00	0.00	0.00	11.00	0.00	0.00	0.00
0.00	0.00	0.00	0.00	0.00	11.00	0.00	0.00	0.00
0.00	0.00	0.00	0.00	4.00	0.00	0.00	0.00	0.00
0.00	0.00	0.00	11.00	0.00	0.00	0.00	0.00	0.00
0.00	0.00	0.00	11.00	0.00	0.00	0.00	0.00	0.00
0.00	0.00	0.00	11.00	0.00	0.00	0.00	0.00	0.00
0.00	0.00	0.00	11.00	0.00	0.00	0.00	0.00	0.00
0.00	0.00	0.00	0.00	0.00	0.00	0.00	0.00	0.00
0.00	0.00	0.00	0.00	0.00	0.00	0.00	0.00	0.00
0.00	0.00	0.00	0.00	0.00	0.00	0.00	0.00	0.00
0.00	0.00	4.00	0.00	0.00	0.00	0.00	2.00	0.00
0.00	0.00	0.00	0.00	0.00	0.00	0.00	0.00	0.00
0.00	0.00	0.00	0.00	0.00	0.00	0.00	0.00	0.00
0.00	0.00	11.00	0.00	0.00	0.00	0.00	0.00	0.00
0.00	0.00	0.00	0.00	0.00	0.00	0.00	0.00	0.00
0.00	0.00	0.00	0.00	0.00	0.00	0.00	0.00	0.00
0.00	0.00	0.00	0.00	0.00	0.00	0.00	0.00	0.00
0.00	0.00	0.00	0.00	0.00	0.00	0.00	0.00	0.00
0.00	0.00	0.00	0.00	0.00	0.00	0.00	0.00	0.00
0.00	0.00	0.00	0.00	0.00	0.00	0.00	0.00	0.00
0.00	0.00	0.00	0.00	0.00	0.00	0.00	0.00	0.00
0.00	0.00	0.00	0.00	0.00	0.00	0.00	0.00	0.00
0.00	0.00	0.00	0.00	0.00	0.00	0.00	0.00	0.00
0.00	0.00	0.00	0.00	0.00	0.00	0.00	0.00	0.00

0.00	0.00	0.00	0.00	0.00	0.00	0.00	0.00	0.00
0.00	0.00	0.00	0.00	0.00	0.00	0.00	0.00	0.00
0.00	0.00	0.00	0.00	0.00	0.00	0.00	0.00	0.00
0.00	0.00	0.00	0.00	0.00	0.00	0.00	0.00	0.00
0.00	0.00	0.00	0.00	0.00	0.00	0.00	0.00	0.00
0.00	0.00	0.00	0.00	0.00	0.00	0.00	0.00	0.00
0.00	0.00	0.00	0.00	0.00	0.00	0.00	0.00	0.00
0.00	0.00	0.00	0.00	0.00	0.00	0.00	0.00	0.00
0.00	0.00	0.00	0.00	0.00	0.00	0.00	0.00	0.00
0.00	0.00	0.00	0.00	0.00	0.00	0.00	0.00	0.00
0.00	0.00	0.00	0.00	0.00	0.00	0.00	0.00	0.00
0.00	0.00	0.00	0.00	0.00	0.00	0.00	0.00	0.00
0.00	0.00	0.00	0.00	0.00	0.00	0.00	0.00	0.00
0.00	0.00	0.00	0.00	0.00	0.00	0.00	0.00	0.00
0.00	0.00	0.00	0.00	0.00	4.00	0.00	0.00	0.00
0.00	0.00	0.00	0.00	0.00	0.00	0.00	0.00	0.00
0.00	0.00	0.00	0.00	0.00	0.00	0.00	0.00	0.00
0.00	0.00	0.00	0.00	0.00	0.00	0.00	0.00	0.00
0.00	0.00	0.00	0.00	0.00	0.00	0.00	0.00	0.00
0.00	0.00	0.00	0.00	0.00	0.00	0.00	0.00	0.00
0.00	0.00	0.00	0.00	0.00	0.00	0.00	0.00	0.00
0.00	0.00	0.00	0.00	0.00	0.00	0.00	0.00	0.00
0.00	0.00	0.00	0.00	0.00	0.00	0.00	0.00	0.00
0.00	0.00	0.00	0.00	0.00	0.00	0.00	0.00	0.00
0.00	0.00	0.00	0.00	0.00	0.00	0.00	0.00	0.00
0.00	0.00	0.00	0.00	0.00	0.00	0.00	0.00	0.00
0.00	0.00	0.00	0.00	0.00	0.00	0.00	0.00	0.00
0.00	0.00	0.00	0.00	6.00	0.00	0.00	0.00	0.00
0.00	0.00	0.00	0.00	0.00	0.00	0.00	0.00	0.00
0.00	0.00	0.00	0.00	0.00	0.00	0.00	0.00	0.00
0.00	0.00	0.00	0.00	0.00	0.00	0.00	0.00	0.00
0.00	0.00	0.00	0.00	0.00	0.00	0.00	0.00	0.00
0.00	0.00	0.00	0.00	0.00	0.00	0.00	0.00	0.00
0.00	0.00	0.00	0.00	0.00	0.00	0.00	0.00	0.00
0.00	0.00	0.00	0.00	0.00	0.00	0.00	0.00	0.00
0.00	0.00	0.00	0.00	0.00	0.00	0.00	0.00	0.00
0.00	0.00	0.00	0.00	10.00	0.00	0.00	0.00	0.00

0.00	0.00	0.00	10.00	0.00	0.00	0.00	0.00	0.00
0.00	0.00	0.00	0.00	0.00	0.00	0.00	0.00	0.00
0.00	0.00	0.00	0.00	0.00	0.00	0.00	0.00	0.00
0.00	0.00	0.00	0.00	0.00	0.00	0.00	0.00	0.00
0.00	0.00	0.00	0.00	0.00	0.00	0.00	10.00	0.00
0.00	0.00	10.00	0.00	0.00	0.00	0.00	0.00	0.00
10.00	0.00	0.00	0.00	0.00	0.00	0.00	0.00	0.00
0.00	0.00	0.00	0.00	0.00	0.00	0.00	0.00	0.00
0.00	0.00	0.00	0.00	0.00	0.00	0.00	0.00	0.00
0.00	0.00	0.00	0.00	0.00	0.00	0.00	0.00	0.00
0.00	0.00	0.00	0.00	0.00	0.00	0.00	0.00	0.00
0.00	0.00	0.00	0.00	0.00	0.00	0.00	0.00	0.00
0.00	0.00	0.00	0.00	0.00	0.00	0.00	0.00	0.00
0.00	0.00	0.00	0.00	0.00	0.00	0.00	0.00	0.00
0.00	0.00	0.00	0.00	0.00	0.00	0.00	0.00	0.00
0.00	0.00	0.00	0.00	0.00	0.00	0.00	0.00	0.00
0.00	0.00	0.00	0.00	0.00	0.00	0.00	0.00	0.00
0.00	0.00	0.00	0.00	0.00	0.00	0.00	0.00	0.00
0.00	0.00	0.00	0.00	0.00	0.00	0.00	0.00	0.00
0.00	0.00	0.00	0.00	0.00	0.00	0.00	0.00	0.00
0.00	0.00	0.00	0.00	0.00	0.00	0.00	0.00	0.00
0.00	0.00	0.00	0.00	0.00	0.00	0.00	0.00	0.00
0.00	0.00	0.00	0.00	0.00	0.00	0.00	0.00	0.00
0.00	0.00	0.00	0.00	0.00	0.00	0.00	0.00	0.00
0.00	0.00	0.00	0.00	0.00	0.00	0.00	0.00	0.00
0.00	0.00	0.00	0.00	0.00	0.00	0.00	0.00	0.00
0.00	0.00	0.00	0.00	0.00	0.00	0.00	0.00	0.00
0.00	0.00	0.00	0.00	0.00	0.00	0.00	0.00	0.00
0.00	0.00	0.00	0.00	0.00	0.00	0.00	0.00	0.00
0.00	0.00	0.00	0.00	0.00	0.00	0.00	0.00	0.00
0.00	0.00	0.00	0.00	0.00	0.00	0.00	0.00	0.00
0.00	0.00	0.00	0.00	0.00	0.00	0.00	0.00	0.00
0.00	0.00	0.00	0.00	0.00	0.00	0.00	0.00	0.00
0.00	0.00	6.00	0.00	0.00	0.00	0.00	0.00	0.00
0.00	0.00	0.00	0.00	0.00	0.00	0.00	0.00	0.00
2.00	0.00	0.00	0.00	0.00	0.00	0.00	0.00	0.00
0.00	0.00	0.00	0.00	0.00	0.00	0.00	0.00	0.00

0.00	0.00	0.00	3.00	0.00	0.00	0.00	0.00	0.00
0.00	0.00	0.00	0.00	0.00	0.00	0.00	0.00	0.00
0.00	0.00	0.00	0.00	0.00	0.00	0.00	0.00	0.00
0.00	0.00	0.00	0.00	0.00	0.00	0.00	0.00	0.00
0.00	0.00	0.00	0.00	0.00	0.00	0.00	0.00	0.00
0.00	0.00	0.00	0.00	0.00	0.00	0.00	0.00	0.00
0.00	0.00	0.00	0.00	0.00	0.00	0.00	0.00	0.00
0.00	0.00	0.00	0.00	0.00	2.00	0.00	0.00	0.00
0.00	0.00	0.00	0.00	0.00	0.00	0.00	0.00	0.00
0.00	0.00	0.00	0.00	0.00	0.00	0.00	0.00	0.00
0.00	0.00	0.00	0.00	0.00	0.00	0.00	0.00	0.00
0.00	0.00	0.00	0.00	0.00	0.00	0.00	0.00	0.00
0.00	0.00	0.00	0.00	0.00	0.00	0.00	0.00	0.00
3.00	0.00	0.00	0.00	0.00	0.00	0.00	0.00	0.00
0.00	0.00	0.00	0.00	0.00	0.00	0.00	0.00	4.00
0.00	0.00	0.00	0.00	0.00	2.00	0.00	0.00	0.00
0.00	0.00	0.00	0.00	9.00	0.00	0.00	0.00	0.00
0.00	0.00	0.00	9.00	0.00	0.00	0.00	0.00	0.00
0.00	0.00	0.00	9.00	0.00	0.00	0.00	0.00	0.00
0.00	0.00	0.00	0.00	0.00	0.00	0.00	0.00	0.00
0.00	0.00	0.00	0.00	0.00	0.00	0.00	0.00	0.00
0.00	0.00	0.00	0.00	0.00	0.00	0.00	0.00	0.00
0.00	0.00	0.00	0.00	0.00	0.00	0.00	0.00	0.00
0.00	0.00	0.00	0.00	0.00	0.00	0.00	6.00	0.00
0.00	0.00	0.00	0.00	0.00	0.00	0.00	9.00	0.00
0.00	0.00	0.00	0.00	0.00	0.00	0.00	0.00	0.00
9.00	0.00	0.00	0.00	0.00	0.00	0.00	0.00	0.00
0.00	0.00	0.00	0.00	0.00	0.00	0.00	0.00	0.00
0.00	0.00	0.00	0.00	0.00	0.00	0.00	0.00	0.00
0.00	0.00	0.00	0.00	0.00	0.00	0.00	0.00	0.00
0.00	0.00	0.00	0.00	0.00	0.00	0.00	0.00	0.00
0.00	0.00	0.00	0.00	0.00	0.00	0.00	0.00	0.00
0.00	0.00	0.00	0.00	0.00	0.00	0.00	0.00	0.00
0.00	0.00	0.00	0.00	0.00	0.00	0.00	0.00	0.00
0.00	0.00	0.00	0.00	0.00	0.00	0.00	0.00	0.00
0.00	0.00	0.00	0.00	0.00	0.00	0.00	0.00	0.00
0.00	0.00	0.00	0.00	0.00	0.00	0.00	0.00	0.00

0.00	0.00	0.00	0.00	0.00	0.00	0.00	0.00	0.00
0.00	0.00	0.00	0.00	0.00	0.00	0.00	0.00	0.00
0.00	0.00	0.00	0.00	0.00	0.00	0.00	0.00	0.00
0.00	0.00	0.00	0.00	0.00	0.00	0.00	0.00	0.00
0.00	0.00	0.00	0.00	0.00	0.00	0.00	0.00	0.00
0.00	0.00	0.00	0.00	0.00	0.00	0.00	0.00	0.00
0.00	0.00	0.00	0.00	0.00	0.00	0.00	0.00	0.00
0.00	0.00	0.00	0.00	0.00	0.00	0.00	0.00	0.00
0.00	0.00	0.00	0.00	0.00	0.00	0.00	0.00	0.00
0.00	0.00	0.00	0.00	0.00	0.00	0.00	0.00	0.00
0.00	0.00	0.00	0.00	0.00	0.00	0.00	0.00	0.00
0.00	0.00	0.00	0.00	0.00	0.00	0.00	0.00	0.00
0.00	0.00	0.00	0.00	0.00	0.00	0.00	0.00	0.00
0.00	0.00	0.00	0.00	0.00	0.00	0.00	0.00	0.00
0.00	0.00	0.00	0.00	0.00	0.00	0.00	0.00	0.00
0.00	0.00	0.00	0.00	0.00	0.00	0.00	0.00	0.00
0.00	0.00	0.00	0.00	0.00	0.00	0.00	0.00	0.00
0.00	0.00	0.00	0.00	0.00	0.00	0.00	0.00	0.00
0.00	0.00	0.00	0.00	0.00	0.00	0.00	0.00	0.00
0.00	0.00	0.00	0.00	0.00	0.00	0.00	0.00	0.00
0.00	0.00	0.00	0.00	0.00	0.00	0.00	0.00	0.00
0.00	0.00	4.00	0.00	0.00	0.00	0.00	0.00	0.00
0.00	0.00	0.00	0.00	0.00	0.00	0.00	0.00	0.00
0.00	0.00	0.00	0.00	3.00	3.00	0.00	0.00	0.00
0.00	0.00	0.00	0.00	0.00	0.00	0.00	0.00	0.00
3.00	0.00	0.00	0.00	0.00	0.00	0.00	0.00	0.00
0.00	0.00	0.00	0.00	0.00	0.00	0.00	3.00	0.00
0.00	0.00	0.00	0.00	0.00	0.00	0.00	0.00	0.00
3.00	0.00	0.00	0.00	0.00	0.00	0.00	0.00	2.00
0.00	0.00	0.00	0.00	0.00	0.00	0.00	0.00	0.00
0.00	0.00	0.00	0.00	0.00	8.00	0.00	0.00	0.00
0.00	0.00	0.00	0.00	3.00	0.00	0.00	0.00	0.00
0.00	0.00	0.00	8.00	0.00	0.00	0.00	0.00	0.00
0.00	0.00	0.00	0.00	0.00	0.00	0.00	0.00	0.00
0.00	0.00	0.00	0.00	0.00	0.00	0.00	8.00	0.00
0.00	8.00	0.00	0.00	0.00	0.00	0.00	0.00	0.00
8.00	0.00	0.00	0.00	0.00	0.00	0.00	0.00	0.00

0.00	0.00	0.00	0.00	0.00	0.00	0.00	0.00	0.00
0.00	0.00	0.00	0.00	0.00	0.00	0.00	0.00	0.00
0.00	0.00	0.00	0.00	0.00	0.00	0.00	0.00	0.00
0.00	0.00	0.00	0.00	0.00	0.00	0.00	0.00	0.00
0.00	0.00	0.00	0.00	0.00	0.00	0.00	0.00	0.00
0.00	0.00	0.00	0.00	0.00	0.00	0.00	0.00	0.00
0.00	0.00	0.00	0.00	0.00	0.00	0.00	0.00	0.00
0.00	0.00	0.00	0.00	0.00	0.00	0.00	0.00	0.00
0.00	0.00	0.00	0.00	0.00	0.00	0.00	0.00	0.00
0.00	0.00	0.00	0.00	0.00	0.00	0.00	0.00	0.00
0.00	0.00	0.00	0.00	0.00	0.00	0.00	0.00	0.00
0.00	0.00	0.00	0.00	0.00	0.00	0.00	0.00	0.00
0.00	0.00	0.00	0.00	0.00	0.00	0.00	0.00	0.00
0.00	0.00	0.00	0.00	0.00	0.00	0.00	0.00	0.00
0.00	0.00	0.00	0.00	0.00	0.00	0.00	0.00	0.00
0.00	0.00	0.00	0.00	0.00	0.00	0.00	0.00	0.00
0.00	0.00	0.00	0.00	0.00	0.00	0.00	0.00	0.00
0.00	0.00	0.00	0.00	0.00	0.00	0.00	0.00	0.00
0.00	0.00	0.00	0.00	0.00	0.00	0.00	0.00	0.00
0.00	0.00	0.00	0.00	0.00	0.00	0.00	0.00	0.00
0.00	0.00	0.00	0.00	0.00	0.00	0.00	0.00	0.00
0.00	0.00	0.00	0.00	0.00	0.00	0.00	0.00	0.00
0.00	0.00	0.00	0.00	0.00	0.00	0.00	0.00	0.00
0.00	0.00	0.00	0.00	0.00	0.00	0.00	0.00	0.00
0.00	0.00	0.00	0.00	0.00	0.00	0.00	0.00	0.00
0.00	0.00	0.00	0.00	0.00	0.00	0.00	0.00	0.00
0.00	0.00	0.00	0.00	0.00	0.00	0.00	0.00	0.00
0.00	0.00	0.00	0.00	0.00	0.00	0.00	0.00	0.00
0.00	0.00	0.00	0.00	0.00	0.00	0.00	0.00	0.00
0.00	0.00	0.00	0.00	0.00	0.00	0.00	0.00	0.00
0.00	0.00	0.00	0.00	0.00	0.00	0.00	0.00	0.00
0.00	0.00	0.00	0.00	0.00	0.00	0.00	0.00	0.00
0.00	0.00	0.00	0.00	0.00	0.00	0.00	0.00	0.00
0.00	0.00	0.00	0.00	0.00	0.00	0.00	0.00	0.00
0.00	0.00	0.00	0.00	0.00	0.00	0.00	0.00	0.00
0.00	0.00	0.00	0.00	0.00	0.00	0.00	0.00	0.00
0.00	0.00	0.00	0.00	3.00	0.00	0.00	0.00	0.00

0.00	0.00	0.00	0.00	0.00	0.00	0.00	0.00	0.00
0.00	0.00	0.00	0.00	0.00	0.00	0.00	0.00	0.00
0.00	0.00	0.00	0.00	0.00	0.00	0.00	0.00	0.00
0.00	0.00	0.00	4.00	0.00	0.00	0.00	0.00	0.00
0.00	0.00	0.00	0.00	0.00	0.00	0.00	0.00	0.00
0.00	0.00	0.00	0.00	0.00	0.00	0.00	0.00	0.00
0.00	0.00	0.00	0.00	0.00	0.00	0.00	0.00	0.00
0.00	0.00	0.00	0.00	0.00	0.00	0.00	0.00	0.00
0.00	0.00	0.00	0.00	0.00	7.00	0.00	0.00	0.00
0.00	0.00	0.00	0.00	0.00	7.00	0.00	0.00	0.00
0.00	0.00	0.00	7.00	0.00	0.00	0.00	0.00	0.00
0.00	0.00	0.00	7.00	0.00	0.00	0.00	0.00	0.00
0.00	0.00	0.00	7.00	0.00	0.00	0.00	0.00	0.00
0.00	0.00	0.00	7.00	0.00	0.00	0.00	0.00	0.00
0.00	0.00	0.00	0.00	0.00	0.00	0.00	0.00	0.00
0.00	0.00	0.00	0.00	0.00	0.00	0.00	0.00	0.00
0.00	0.00	0.00	0.00	0.00	0.00	0.00	0.00	0.00
0.00	0.00	0.00	0.00	0.00	0.00	0.00	0.00	0.00
0.00	0.00	0.00	0.00	0.00	0.00	0.00	7.00	0.00
0.00	0.00	7.00	0.00	0.00	0.00	0.00	0.00	0.00
0.00	0.00	7.00	0.00	0.00	0.00	0.00	0.00	0.00
7.00	0.00	0.00	0.00	0.00	0.00	0.00	0.00	0.00
0.00	0.00	0.00	0.00	0.00	0.00	0.00	0.00	0.00
0.00	0.00	0.00	0.00	0.00	0.00	0.00	0.00	0.00
0.00	0.00	0.00	0.00	0.00	0.00	0.00	0.00	0.00
0.00	0.00	0.00	0.00	0.00	0.00	0.00	0.00	0.00
0.00	0.00	0.00	0.00	0.00	0.00	0.00	0.00	0.00
0.00	0.00	0.00	0.00	0.00	0.00	0.00	0.00	0.00
0.00	0.00	0.00	0.00	0.00	0.00	0.00	0.00	0.00
0.00	0.00	0.00	0.00	0.00	0.00	0.00	0.00	0.00
0.00	0.00	0.00	0.00	0.00	0.00	0.00	0.00	0.00
0.00	0.00	0.00	0.00	0.00	0.00	0.00	0.00	0.00
0.00	0.00	0.00	0.00	0.00	0.00	0.00	0.00	0.00
0.00	0.00	0.00	0.00	0.00	0.00	0.00	0.00	0.00
0.00	0.00	0.00	0.00	0.00	0.00	0.00	0.00	0.00
0.00	0.00	0.00	0.00	0.00	0.00	0.00	0.00	0.00
0.00	0.00	0.00	0.00	0.00	0.00	0.00	0.00	0.00

0.00	0.00	0.00	0.00	0.00	0.00	0.00	0.00	0.00
0.00	0.00	0.00	0.00	0.00	0.00	0.00	0.00	0.00
0.00	0.00	0.00	0.00	0.00	0.00	0.00	0.00	0.00
0.00	0.00	0.00	0.00	0.00	0.00	0.00	0.00	0.00
0.00	0.00	0.00	0.00	0.00	0.00	0.00	0.00	0.00
0.00	0.00	0.00	0.00	0.00	0.00	0.00	0.00	0.00
0.00	0.00	0.00	0.00	0.00	0.00	0.00	0.00	0.00
0.00	0.00	0.00	0.00	0.00	0.00	0.00	0.00	0.00
0.00	0.00	0.00	0.00	0.00	0.00	0.00	0.00	0.00
0.00	0.00	0.00	0.00	0.00	0.00	0.00	0.00	0.00
0.00	0.00	0.00	0.00	0.00	0.00	0.00	0.00	0.00
0.00	0.00	0.00	0.00	0.00	0.00	0.00	0.00	0.00
0.00	0.00	0.00	0.00	0.00	0.00	0.00	0.00	0.00
0.00	0.00	0.00	0.00	0.00	0.00	0.00	0.00	0.00
0.00	0.00	0.00	0.00	0.00	0.00	0.00	0.00	0.00
0.00	0.00	0.00	0.00	0.00	0.00	0.00	0.00	0.00
0.00	0.00	0.00	0.00	0.00	0.00	0.00	0.00	0.00
0.00	0.00	0.00	0.00	0.00	0.00	0.00	0.00	0.00
0.00	0.00	0.00	0.00	0.00	0.00	0.00	0.00	0.00
0.00	0.00	0.00	0.00	0.00	0.00	0.00	0.00	0.00
0.00	0.00	0.00	0.00	0.00	0.00	0.00	0.00	0.00
0.00	0.00	0.00	0.00	0.00	0.00	0.00	0.00	0.00
0.00	0.00	0.00	0.00	0.00	0.00	0.00	0.00	0.00
0.00	0.00	0.00	0.00	0.00	0.00	0.00	0.00	0.00
0.00	0.00	0.00	0.00	0.00	0.00	0.00	0.00	0.00
0.00	0.00	0.00	0.00	0.00	0.00	0.00	0.00	0.00
0.00	0.00	0.00	0.00	0.00	0.00	0.00	0.00	0.00
0.00	0.00	0.00	0.00	0.00	0.00	0.00	0.00	0.00
0.00	0.00	0.00	0.00	0.00	0.00	0.00	0.00	0.00
0.00	0.00	0.00	0.00	0.00	0.00	0.00	0.00	0.00
0.00	0.00	0.00	0.00	0.00	0.00	0.00	0.00	0.00
0.00	0.00	0.00	0.00	0.00	0.00	0.00	0.00	0.00
0.00	0.00	0.00	0.00	0.00	0.00	0.00	0.00	0.00
0.00	0.00	0.00	0.00	0.00	0.00	0.00	0.00	0.00
0.00	0.00	0.00	0.00	0.00	0.00	0.00	0.00	0.00
0.00	0.00	0.00	0.00	0.00	0.00	0.00	0.00	0.00
0.00	0.00	0.00	0.00	0.00	0.00	0.00	0.00	0.00

0.00	0.00	2.00	0.00	0.00	0.00	0.00	0.00	0.00
0.00	0.00	0.00	0.00	0.00	0.00	0.00	0.00	0.00
2.00	0.00	0.00	0.00	0.00	0.00	0.00	0.00	0.00
0.00	0.00	0.00	0.00	0.00	0.00	0.00	0.00	0.00
0.00	0.00	0.00	0.00	0.00	0.00	6.00	0.00	0.00
0.00	0.00	0.00	0.00	0.00	4.00	0.00	0.00	0.00
0.00	0.00	0.00	0.00	0.00	4.00	0.00	0.00	0.00
0.00	0.00	0.00	0.00	0.00	6.00	0.00	0.00	0.00
0.00	0.00	0.00	6.00	0.00	0.00	0.00	0.00	0.00
0.00	0.00	0.00	4.00	0.00	0.00	0.00	0.00	0.00
0.00	0.00	0.00	0.00	0.00	0.00	0.00	0.00	0.00
0.00	0.00	0.00	0.00	0.00	0.00	0.00	0.00	0.00
0.00	0.00	0.00	0.00	0.00	0.00	0.00	0.00	0.00
0.00	0.00	0.00	0.00	0.00	0.00	0.00	0.00	0.00
0.00	0.00	0.00	0.00	0.00	0.00	0.00	0.00	0.00
0.00	0.00	0.00	0.00	0.00	0.00	0.00	0.00	0.00
0.00	0.00	0.00	0.00	0.00	0.00	0.00	0.00	0.00
0.00	0.00	0.00	0.00	0.00	0.00	0.00	0.00	0.00
0.00	0.00	0.00	0.00	0.00	0.00	0.00	0.00	0.00
0.00	0.00	0.00	0.00	0.00	0.00	0.00	0.00	0.00
0.00	0.00	0.00	0.00	0.00	0.00	0.00	6.00	0.00
0.00	0.00	0.00	0.00	0.00	0.00	0.00	6.00	0.00
0.00	0.00	6.00	0.00	0.00	0.00	0.00	0.00	0.00
0.00	0.00	4.00	0.00	0.00	0.00	0.00	0.00	0.00
0.00	0.00	3.00	0.00	0.00	0.00	0.00	0.00	0.00
0.00	6.00	0.00	0.00	0.00	0.00	0.00	0.00	0.00
6.00	0.00	0.00	0.00	0.00	0.00	0.00	0.00	0.00
0.00	0.00	0.00	0.00	0.00	0.00	0.00	0.00	0.00
0.00	0.00	0.00	0.00	0.00	0.00	0.00	0.00	0.00
0.00	0.00	0.00	0.00	0.00	0.00	0.00	0.00	0.00
0.00	0.00	0.00	0.00	0.00	0.00	0.00	0.00	0.00
0.00	0.00	0.00	0.00	0.00	0.00	0.00	0.00	0.00
0.00	0.00	0.00	0.00	0.00	0.00	0.00	0.00	0.00
0.00	0.00	0.00	0.00	0.00	0.00	0.00	0.00	0.00
0.00	0.00	0.00	0.00	0.00	0.00	0.00	0.00	0.00
0.00	0.00	0.00	0.00	0.00	0.00	0.00	0.00	0.00
0.00	0.00	0.00	0.00	0.00	0.00	0.00	0.00	0.00

0.00	0.00	0.00	0.00	0.00	0.00	0.00	0.00	0.00
0.00	0.00	0.00	0.00	0.00	0.00	0.00	0.00	0.00
0.00	0.00	0.00	0.00	0.00	0.00	0.00	0.00	0.00
0.00	0.00	0.00	0.00	0.00	0.00	0.00	0.00	0.00
0.00	0.00	0.00	0.00	0.00	0.00	0.00	0.00	0.00
0.00	0.00	0.00	0.00	0.00	0.00	0.00	0.00	0.00
0.00	0.00	0.00	0.00	0.00	0.00	0.00	0.00	0.00
0.00	0.00	0.00	0.00	0.00	0.00	0.00	0.00	0.00
0.00	0.00	0.00	0.00	0.00	0.00	0.00	0.00	0.00
0.00	0.00	0.00	0.00	0.00	0.00	0.00	0.00	0.00
0.00	0.00	0.00	0.00	0.00	0.00	0.00	0.00	0.00
0.00	0.00	0.00	0.00	0.00	0.00	0.00	0.00	0.00
0.00	0.00	0.00	0.00	0.00	0.00	0.00	0.00	0.00
0.00	0.00	0.00	0.00	0.00	0.00	0.00	0.00	0.00
0.00	0.00	0.00	0.00	0.00	0.00	0.00	0.00	0.00
0.00	0.00	0.00	0.00	0.00	0.00	0.00	0.00	0.00
0.00	0.00	0.00	0.00	0.00	0.00	0.00	0.00	0.00
0.00	0.00	0.00	0.00	0.00	0.00	0.00	0.00	0.00
0.00	0.00	0.00	0.00	0.00	0.00	0.00	0.00	0.00
0.00	0.00	0.00	0.00	0.00	0.00	0.00	0.00	0.00
0.00	0.00	0.00	0.00	0.00	0.00	0.00	0.00	0.00
0.00	0.00	0.00	0.00	0.00	0.00	0.00	0.00	0.00
0.00	0.00	0.00	0.00	0.00	0.00	0.00	0.00	0.00
0.00	0.00	0.00	0.00	0.00	0.00	0.00	0.00	0.00
0.00	0.00	0.00	0.00	0.00	0.00	0.00	0.00	0.00
0.00	0.00	0.00	0.00	0.00	0.00	0.00	0.00	0.00
0.00	0.00	0.00	0.00	0.00	0.00	0.00	0.00	0.00
0.00	0.00	0.00	0.00	0.00	0.00	0.00	0.00	0.00
0.00	0.00	0.00	0.00	0.00	0.00	0.00	0.00	0.00
0.00	0.00	0.00	0.00	0.00	0.00	0.00	0.00	0.00
0.00	0.00	0.00	0.00	0.00	0.00	0.00	0.00	0.00
0.00	0.00	0.00	0.00	0.00	0.00	0.00	0.00	0.00
0.00	0.00	0.00	0.00	0.00	0.00	0.00	0.00	0.00
0.00	0.00	0.00	0.00	0.00	0.00	0.00	0.00	0.00
0.00	0.00	0.00	0.00	0.00	0.00	0.00	0.00	0.00
0.00	0.00	0.00	0.00	0.00	0.00	0.00	0.00	0.00
0.00	0.00	0.00	0.00	0.00	0.00	0.00	0.00	0.00

0.00	0.00	0.00	0.00	0.00	0.00	0.00	0.00	0.00
0.00	0.00	0.00	0.00	0.00	0.00	0.00	0.00	0.00
0.00	0.00	0.00	0.00	0.00	0.00	0.00	0.00	3.00
0.00	0.00	0.00	0.00	0.00	0.00	0.00	0.00	0.00
0.00	0.00	0.00	0.00	0.00	0.00	0.00	0.00	0.00
0.00	0.00	0.00	0.00	0.00	0.00	0.00	0.00	0.00
0.00	0.00	2.00	0.00	0.00	0.00	0.00	0.00	0.00
0.00	0.00	2.00	0.00	0.00	0.00	0.00	0.00	0.00
0.00	0.00	0.00	0.00	0.00	0.00	0.00	0.00	0.00
0.00	0.00	0.00	0.00	0.00	0.00	0.00	0.00	0.00
0.00	0.00	0.00	0.00	0.00	0.00	0.00	0.00	0.00
0.00	0.00	0.00	0.00	0.00	0.00	0.00	0.00	0.00
0.00	0.00	0.00	0.00	0.00	0.00	0.00	0.00	0.00
0.00	0.00	0.00	0.00	0.00	0.00	0.00	0.00	0.00
0.00	0.00	0.00	0.00	0.00	0.00	0.00	0.00	0.00
0.00	0.00	0.00	0.00	0.00	0.00	0.00	0.00	0.00
0.00	0.00	0.00	0.00	0.00	0.00	0.00	0.00	0.00
0.00	0.00	0.00	0.00	0.00	0.00	0.00	0.00	0.00
0.00	0.00	0.00	0.00	0.00	0.00	0.00	0.00	0.00
0.00	0.00	0.00	0.00	0.00	0.00	0.00	0.00	0.00
0.00	0.00	0.00	0.00	0.00	0.00	0.00	0.00	0.00
0.00	0.00	0.00	0.00	0.00	0.00	5.00	0.00	0.00
0.00	0.00	0.00	0.00	0.00	5.00	0.00	0.00	0.00
0.00	0.00	0.00	0.00	5.00	0.00	0.00	0.00	0.00
0.00	0.00	0.00	5.00	0.00	0.00	0.00	0.00	0.00
0.00	0.00	0.00	0.00	0.00	0.00	0.00	0.00	0.00
0.00	0.00	0.00	0.00	0.00	0.00	0.00	0.00	0.00
0.00	0.00	0.00	0.00	0.00	0.00	0.00	0.00	0.00
0.00	0.00	0.00	0.00	0.00	0.00	0.00	0.00	0.00
0.00	0.00	0.00	0.00	0.00	0.00	0.00	0.00	0.00
0.00	0.00	0.00	0.00	0.00	0.00	0.00	0.00	0.00
0.00	0.00	0.00	0.00	0.00	0.00	0.00	0.00	0.00
0.00	0.00	0.00	0.00	0.00	0.00	0.00	0.00	0.00
0.00	0.00	0.00	0.00	0.00	0.00	0.00	0.00	0.00
0.00	0.00	0.00	0.00	0.00	0.00	0.00	0.00	0.00
0.00	0.00	0.00	0.00	0.00	0.00	0.00	0.00	0.00
0.00	0.00	0.00	0.00	0.00	0.00	0.00	0.00	0.00

0.00	0.00	0.00	0.00	0.00	0.00	0.00	0.00	0.00
0.00	0.00	0.00	0.00	0.00	0.00	0.00	5.00	0.00
0.00	0.00	0.00	0.00	0.00	0.00	0.00	0.00	0.00
0.00	0.00	0.00	0.00	0.00	0.00	0.00	0.00	0.00
0.00	0.00	0.00	0.00	0.00	0.00	0.00	0.00	0.00
0.00	0.00	5.00	0.00	0.00	0.00	0.00	0.00	0.00
5.00	0.00	0.00	0.00	0.00	0.00	0.00	0.00	0.00
5.00	0.00	0.00	0.00	0.00	0.00	0.00	0.00	0.00
0.00	0.00	0.00	0.00	0.00	0.00	0.00	0.00	0.00
0.00	0.00	0.00	0.00	0.00	0.00	0.00	0.00	0.00
0.00	0.00	0.00	0.00	0.00	0.00	0.00	0.00	0.00
0.00	0.00	0.00	0.00	0.00	0.00	0.00	0.00	0.00
0.00	0.00	0.00	0.00	0.00	0.00	0.00	0.00	0.00
0.00	0.00	0.00	0.00	0.00	0.00	0.00	0.00	0.00
0.00	0.00	0.00	0.00	0.00	0.00	0.00	0.00	0.00
0.00	0.00	0.00	0.00	0.00	0.00	0.00	0.00	0.00
0.00	0.00	0.00	0.00	0.00	0.00	0.00	0.00	0.00
0.00	0.00	0.00	0.00	0.00	0.00	0.00	0.00	0.00
0.00	0.00	0.00	0.00	0.00	0.00	0.00	0.00	0.00
0.00	0.00	0.00	0.00	0.00	0.00	0.00	0.00	0.00
0.00	0.00	0.00	0.00	0.00	0.00	0.00	0.00	0.00
0.00	0.00	0.00	0.00	0.00	0.00	0.00	0.00	0.00
0.00	0.00	0.00	0.00	0.00	0.00	0.00	0.00	0.00
0.00	0.00	0.00	0.00	0.00	0.00	0.00	0.00	0.00
0.00	0.00	0.00	0.00	0.00	0.00	0.00	0.00	0.00
0.00	0.00	0.00	0.00	0.00	0.00	0.00	0.00	0.00
0.00	0.00	0.00	0.00	0.00	0.00	0.00	0.00	0.00
0.00	0.00	0.00	0.00	0.00	0.00	0.00	0.00	0.00
0.00	0.00	0.00	0.00	0.00	0.00	0.00	0.00	0.00
0.00	0.00	0.00	0.00	0.00	0.00	0.00	0.00	0.00
0.00	0.00	0.00	0.00	0.00	0.00	0.00	0.00	0.00
0.00	0.00	0.00	0.00	0.00	0.00	0.00	0.00	0.00
0.00	0.00	0.00	0.00	0.00	0.00	0.00	0.00	0.00
0.00	0.00	0.00	0.00	0.00	0.00	0.00	0.00	0.00
0.00	0.00	0.00	0.00	0.00	0.00	0.00	0.00	0.00
0.00	0.00	0.00	0.00	0.00	0.00	0.00	0.00	0.00
0.00	0.00	0.00	0.00	0.00	0.00	0.00	0.00	0.00

0.00	0.00	0.00	0.00	0.00	0.00	0.00	0.00	0.00
0.00	0.00	0.00	0.00	0.00	0.00	0.00	0.00	0.00
0.00	0.00	0.00	0.00	0.00	0.00	0.00	0.00	0.00
0.00	0.00	0.00	0.00	0.00	0.00	0.00	0.00	0.00
0.00	0.00	0.00	0.00	0.00	0.00	0.00	0.00	0.00
0.00	0.00	0.00	0.00	0.00	0.00	0.00	0.00	0.00
0.00	0.00	0.00	0.00	0.00	0.00	0.00	0.00	0.00
0.00	0.00	0.00	0.00	0.00	0.00	0.00	0.00	0.00
0.00	0.00	0.00	0.00	0.00	0.00	0.00	0.00	0.00
0.00	0.00	0.00	0.00	0.00	0.00	0.00	0.00	0.00
0.00	0.00	0.00	0.00	0.00	0.00	0.00	0.00	0.00
0.00	0.00	0.00	0.00	0.00	0.00	0.00	0.00	0.00
0.00	0.00	0.00	0.00	0.00	0.00	0.00	0.00	0.00
0.00	0.00	0.00	0.00	0.00	0.00	0.00	0.00	0.00
0.00	0.00	0.00	0.00	0.00	0.00	0.00	0.00	0.00
0.00	0.00	0.00	0.00	0.00	0.00	0.00	0.00	0.00
0.00	0.00	0.00	0.00	0.00	0.00	0.00	0.00	0.00
0.00	0.00	0.00	0.00	0.00	0.00	0.00	0.00	0.00
0.00	0.00	0.00	0.00	0.00	0.00	0.00	0.00	0.00
0.00	0.00	0.00	0.00	0.00	2.00	0.00	0.00	0.00
0.00	0.00	0.00	0.00	0.00	0.00	0.00	0.00	0.00
0.00	0.00	0.00	0.00	0.00	0.00	0.00	0.00	0.00
0.00	0.00	0.00	0.00	0.00	0.00	0.00	0.00	0.00
0.00	0.00	0.00	0.00	0.00	0.00	0.00	0.00	0.00
0.00	0.00	0.00	0.00	0.00	0.00	0.00	0.00	0.00
0.00	0.00	0.00	0.00	0.00	0.00	0.00	0.00	0.00
0.00	0.00	0.00	0.00	0.00	0.00	0.00	0.00	0.00
0.00	0.00	0.00	0.00	0.00	0.00	0.00	0.00	0.00
0.00	0.00	0.00	0.00	0.00	0.00	0.00	0.00	0.00
0.00	0.00	0.00	0.00	0.00	0.00	0.00	0.00	0.00
0.00	0.00	0.00	0.00	0.00	0.00	0.00	0.00	0.00
0.00	0.00	0.00	0.00	0.00	0.00	0.00	0.00	0.00
0.00	0.00	0.00	0.00	0.00	0.00	0.00	0.00	0.00
0.00	0.00	0.00	0.00	0.00	0.00	0.00	0.00	0.00
0.00	0.00	0.00	0.00	0.00	0.00	0.00	0.00	0.00
0.00	0.00	0.00	0.00	0.00	0.00	0.00	0.00	0.00
0.00	0.00	0.00	0.00	0.00	0.00	0.00	0.00	0.00

0.00	0.00	0.00	0.00	0.00	0.00	0.00	0.00	0.00
0.00	0.00	0.00	0.00	0.00	0.00	0.00	0.00	0.00
0.00	0.00	0.00	0.00	0.00	0.00	0.00	0.00	0.00
0.00	0.00	0.00	0.00	0.00	0.00	0.00	0.00	0.00
0.00	0.00	0.00	0.00	0.00	0.00	0.00	0.00	0.00
0.00	0.00	0.00	0.00	0.00	0.00	0.00	0.00	0.00
0.00	0.00	0.00	0.00	0.00	0.00	0.00	0.00	0.00
0.00	0.00	0.00	0.00	0.00	4.00	0.00	0.00	0.00
0.00	0.00	0.00	0.00	0.00	2.00	0.00	0.00	0.00
0.00	0.00	0.00	0.00	4.00	0.00	0.00	0.00	0.00
0.00	0.00	0.00	4.00	0.00	0.00	0.00	0.00	0.00
0.00	0.00	0.00	4.00	0.00	0.00	0.00	0.00	0.00
0.00	0.00	0.00	4.00	0.00	0.00	0.00	0.00	0.00
0.00	0.00	0.00	4.00	0.00	0.00	0.00	0.00	0.00
0.00	0.00	0.00	0.00	0.00	0.00	0.00	0.00	0.00
0.00	0.00	0.00	0.00	0.00	0.00	0.00	0.00	0.00
0.00	0.00	0.00	0.00	0.00	0.00	0.00	0.00	0.00
0.00	0.00	0.00	0.00	0.00	0.00	0.00	0.00	0.00
0.00	0.00	0.00	0.00	0.00	0.00	0.00	0.00	0.00
0.00	0.00	0.00	0.00	0.00	0.00	0.00	0.00	0.00
0.00	0.00	0.00	0.00	0.00	0.00	0.00	0.00	0.00
0.00	0.00	0.00	0.00	0.00	0.00	0.00	0.00	0.00
0.00	0.00	0.00	0.00	0.00	0.00	0.00	0.00	0.00
0.00	0.00	0.00	0.00	0.00	0.00	0.00	0.00	0.00
0.00	0.00	0.00	0.00	0.00	0.00	0.00	0.00	0.00
0.00	0.00	0.00	0.00	0.00	0.00	0.00	0.00	0.00
0.00	0.00	0.00	0.00	0.00	0.00	0.00	0.00	0.00
0.00	0.00	0.00	0.00	0.00	0.00	0.00	0.00	0.00
0.00	0.00	0.00	0.00	0.00	0.00	0.00	0.00	0.00
0.00	0.00	0.00	0.00	0.00	0.00	0.00	0.00	0.00
0.00	0.00	0.00	0.00	0.00	0.00	0.00	0.00	0.00
0.00	0.00	0.00	0.00	0.00	0.00	0.00	0.00	0.00
0.00	0.00	0.00	0.00	0.00	0.00	0.00	0.00	0.00
0.00	0.00	0.00	0.00	0.00	0.00	0.00	0.00	0.00
0.00	0.00	0.00	0.00	0.00	0.00	0.00	0.00	0.00
0.00	0.00	0.00	0.00	0.00	0.00	0.00	0.00	0.00
0.00	0.00	0.00	0.00	0.00	0.00	0.00	0.00	0.00

0.00	0.00	0.00	0.00	0.00	0.00	0.00	0.00	0.00
0.00	0.00	0.00	0.00	0.00	0.00	0.00	0.00	0.00
0.00	0.00	0.00	0.00	0.00	0.00	0.00	0.00	0.00
0.00	0.00	0.00	0.00	0.00	0.00	0.00	0.00	0.00
0.00	0.00	0.00	0.00	0.00	0.00	0.00	0.00	0.00
0.00	0.00	0.00	0.00	0.00	0.00	0.00	0.00	0.00
0.00	0.00	0.00	0.00	0.00	0.00	0.00	0.00	0.00
0.00	0.00	0.00	0.00	0.00	0.00	0.00	4.00	0.00
0.00	0.00	0.00	0.00	0.00	0.00	0.00	4.00	0.00
0.00	0.00	0.00	0.00	0.00	0.00	0.00	4.00	0.00
0.00	0.00	0.00	0.00	0.00	0.00	0.00	0.00	0.00
0.00	0.00	4.00	0.00	0.00	0.00	0.00	0.00	0.00
0.00	0.00	4.00	0.00	0.00	0.00	0.00	0.00	0.00
0.00	0.00	4.00	0.00	0.00	0.00	0.00	0.00	0.00
4.00	0.00	0.00	0.00	0.00	0.00	0.00	0.00	0.00
4.00	0.00	0.00	0.00	0.00	0.00	0.00	0.00	0.00
4.00	0.00	0.00	0.00	0.00	0.00	0.00	0.00	0.00
0.00	0.00	0.00	0.00	0.00	0.00	0.00	0.00	0.00
0.00	0.00	0.00	0.00	0.00	0.00	0.00	0.00	0.00
0.00	0.00	0.00	0.00	0.00	0.00	0.00	0.00	0.00
0.00	0.00	0.00	0.00	0.00	0.00	0.00	0.00	0.00
0.00	0.00	0.00	0.00	0.00	0.00	0.00	0.00	0.00
0.00	0.00	0.00	0.00	0.00	0.00	0.00	0.00	0.00
0.00	0.00	0.00	0.00	0.00	0.00	0.00	0.00	0.00
0.00	0.00	0.00	0.00	0.00	0.00	0.00	0.00	0.00
0.00	0.00	0.00	0.00	0.00	0.00	0.00	0.00	0.00
0.00	0.00	0.00	0.00	0.00	0.00	0.00	0.00	0.00
0.00	0.00	0.00	0.00	0.00	0.00	0.00	0.00	0.00
0.00	0.00	0.00	0.00	0.00	0.00	0.00	0.00	0.00
0.00	0.00	0.00	0.00	0.00	0.00	0.00	0.00	0.00
0.00	0.00	0.00	0.00	0.00	0.00	0.00	0.00	0.00
0.00	0.00	0.00	0.00	0.00	0.00	0.00	0.00	0.00
0.00	0.00	0.00	0.00	0.00	0.00	0.00	0.00	0.00
0.00	0.00	0.00	0.00	0.00	0.00	0.00	0.00	0.00
0.00	0.00	0.00	0.00	0.00	0.00	0.00	0.00	0.00
0.00	0.00	0.00	0.00	0.00	0.00	0.00	0.00	0.00
0.00	0.00	0.00	0.00	0.00	0.00	0.00	0.00	0.00

0.00	0.00	0.00	0.00	0.00	0.00	0.00	0.00	0.00
0.00	0.00	0.00	0.00	0.00	0.00	0.00	0.00	0.00
0.00	0.00	0.00	0.00	0.00	0.00	0.00	0.00	0.00
0.00	0.00	0.00	0.00	0.00	0.00	0.00	0.00	0.00
0.00	0.00	0.00	0.00	0.00	0.00	0.00	0.00	0.00
0.00	0.00	0.00	0.00	0.00	0.00	0.00	0.00	0.00
0.00	0.00	0.00	0.00	0.00	0.00	0.00	0.00	0.00
0.00	0.00	0.00	0.00	0.00	0.00	0.00	0.00	0.00
0.00	0.00	0.00	0.00	0.00	0.00	0.00	0.00	0.00
0.00	0.00	0.00	0.00	0.00	0.00	0.00	0.00	0.00
0.00	0.00	0.00	0.00	0.00	0.00	0.00	0.00	0.00
0.00	0.00	0.00	0.00	0.00	0.00	0.00	0.00	0.00
0.00	0.00	0.00	0.00	0.00	0.00	0.00	0.00	0.00
0.00	0.00	0.00	0.00	0.00	0.00	0.00	0.00	0.00
0.00	0.00	0.00	0.00	0.00	0.00	0.00	0.00	0.00
0.00	0.00	0.00	0.00	0.00	0.00	0.00	0.00	0.00
0.00	0.00	0.00	0.00	0.00	0.00	0.00	0.00	0.00
0.00	0.00	0.00	0.00	0.00	0.00	0.00	0.00	0.00
0.00	0.00	0.00	0.00	0.00	0.00	0.00	0.00	0.00
0.00	0.00	0.00	0.00	0.00	0.00	0.00	0.00	0.00
0.00	0.00	0.00	0.00	0.00	0.00	0.00	0.00	0.00
0.00	0.00	0.00	0.00	0.00	0.00	0.00	0.00	0.00
0.00	0.00	0.00	0.00	0.00	0.00	0.00	0.00	0.00
0.00	0.00	0.00	0.00	0.00	0.00	0.00	0.00	0.00
0.00	0.00	0.00	0.00	0.00	0.00	0.00	0.00	0.00
0.00	0.00	0.00	0.00	0.00	0.00	0.00	0.00	0.00
0.00	0.00	0.00	0.00	0.00	0.00	0.00	0.00	0.00
0.00	0.00	0.00	0.00	0.00	0.00	0.00	0.00	0.00
0.00	0.00	0.00	0.00	0.00	0.00	0.00	0.00	0.00
0.00	0.00	0.00	0.00	0.00	0.00	0.00	0.00	0.00
0.00	0.00	0.00	0.00	0.00	0.00	0.00	0.00	0.00
0.00	0.00	0.00	0.00	0.00	0.00	0.00	0.00	0.00
0.00	0.00	0.00	0.00	0.00	0.00	0.00	0.00	0.00
0.00	0.00	0.00	0.00	0.00	0.00	0.00	0.00	0.00
0.00	0.00	0.00	0.00	0.00	0.00	0.00	0.00	0.00
0.00	0.00	0.00	0.00	0.00	0.00	0.00	0.00	0.00
0.00	0.00	0.00	0.00	0.00	0.00	0.00	0.00	0.00

0.00	0.00	0.00	0.00	0.00	0.00	0.00	0.00	0.00
0.00	0.00	0.00	0.00	0.00	0.00	0.00	0.00	0.00
0.00	0.00	0.00	0.00	0.00	0.00	0.00	0.00	0.00
0.00	0.00	0.00	0.00	0.00	0.00	0.00	0.00	0.00
0.00	0.00	0.00	0.00	0.00	0.00	0.00	0.00	0.00
0.00	0.00	0.00	0.00	0.00	0.00	0.00	0.00	0.00
0.00	0.00	0.00	0.00	0.00	0.00	0.00	0.00	0.00
0.00	0.00	0.00	0.00	0.00	0.00	0.00	0.00	0.00
0.00	0.00	0.00	0.00	0.00	0.00	0.00	0.00	0.00
0.00	0.00	0.00	0.00	0.00	0.00	0.00	0.00	0.00
0.00	0.00	0.00	0.00	0.00	0.00	0.00	0.00	0.00
0.00	0.00	0.00	0.00	0.00	0.00	0.00	0.00	0.00
0.00	0.00	0.00	0.00	0.00	0.00	0.00	0.00	0.00
0.00	0.00	0.00	0.00	0.00	0.00	0.00	0.00	0.00
0.00	0.00	0.00	0.00	0.00	0.00	0.00	0.00	0.00
0.00	0.00	0.00	0.00	0.00	0.00	0.00	0.00	0.00
0.00	0.00	0.00	0.00	0.00	0.00	0.00	0.00	0.00
0.00	0.00	0.00	0.00	0.00	0.00	0.00	0.00	0.00
0.00	0.00	0.00	0.00	0.00	0.00	0.00	0.00	0.00
0.00	0.00	0.00	0.00	0.00	0.00	0.00	0.00	0.00
0.00	0.00	0.00	0.00	0.00	0.00	0.00	0.00	0.00
0.00	0.00	0.00	0.00	0.00	0.00	0.00	0.00	0.00
0.00	0.00	0.00	0.00	0.00	0.00	0.00	0.00	0.00
0.00	0.00	0.00	0.00	0.00	0.00	0.00	0.00	0.00
0.00	0.00	0.00	0.00	0.00	0.00	0.00	0.00	0.00
0.00	0.00	0.00	0.00	0.00	0.00	0.00	0.00	0.00
0.00	0.00	0.00	0.00	0.00	0.00	0.00	0.00	0.00
0.00	0.00	0.00	0.00	0.00	0.00	0.00	0.00	0.00
0.00	0.00	0.00	0.00	0.00	0.00	0.00	0.00	0.00
0.00	0.00	0.00	0.00	0.00	0.00	0.00	0.00	0.00
0.00	0.00	0.00	0.00	0.00	0.00	0.00	0.00	0.00
0.00	0.00	0.00	0.00	0.00	0.00	0.00	0.00	0.00
0.00	0.00	0.00	0.00	0.00	0.00	0.00	0.00	0.00
0.00	0.00	0.00	0.00	0.00	0.00	0.00	0.00	0.00
0.00	0.00	0.00	0.00	0.00	0.00	0.00	0.00	0.00
0.00	0.00	0.00	0.00	0.00	0.00	0.00	0.00	3.00
0.00	0.00	0.00	0.00	0.00	0.00	0.00	0.00	0.00

0.00	0.00	0.00	0.00	0.00	0.00	3.00	0.00	0.00
0.00	0.00	0.00	0.00	0.00	3.00	0.00	0.00	0.00
0.00	0.00	0.00	0.00	0.00	3.00	0.00	0.00	0.00
0.00	0.00	0.00	0.00	0.00	3.00	0.00	0.00	0.00
0.00	0.00	0.00	0.00	0.00	3.00	0.00	0.00	0.00
0.00	0.00	0.00	3.00	0.00	0.00	0.00	0.00	0.00
0.00	0.00	0.00	0.00	0.00	0.00	0.00	0.00	0.00
0.00	0.00	0.00	0.00	0.00	0.00	0.00	0.00	0.00
0.00	0.00	0.00	0.00	0.00	0.00	0.00	0.00	0.00
0.00	0.00	0.00	0.00	0.00	0.00	0.00	0.00	0.00
0.00	0.00	0.00	0.00	0.00	0.00	0.00	0.00	0.00
0.00	0.00	0.00	0.00	0.00	0.00	0.00	0.00	0.00
0.00	0.00	0.00	0.00	0.00	0.00	0.00	0.00	0.00
0.00	0.00	0.00	0.00	0.00	0.00	0.00	0.00	0.00
0.00	0.00	0.00	0.00	0.00	0.00	0.00	0.00	0.00
0.00	0.00	0.00	0.00	0.00	0.00	0.00	0.00	0.00
0.00	0.00	0.00	0.00	0.00	0.00	0.00	0.00	0.00
0.00	0.00	0.00	0.00	0.00	0.00	0.00	0.00	0.00
0.00	0.00	0.00	0.00	0.00	0.00	0.00	0.00	0.00
0.00	0.00	0.00	0.00	0.00	0.00	0.00	0.00	0.00
0.00	0.00	0.00	0.00	0.00	0.00	0.00	0.00	0.00
0.00	0.00	0.00	0.00	0.00	0.00	0.00	0.00	0.00
0.00	0.00	0.00	0.00	0.00	0.00	0.00	0.00	0.00
0.00	0.00	0.00	0.00	0.00	0.00	0.00	0.00	0.00
0.00	0.00	0.00	0.00	0.00	0.00	0.00	0.00	0.00
0.00	0.00	0.00	0.00	0.00	0.00	0.00	0.00	0.00
0.00	0.00	0.00	0.00	0.00	0.00	0.00	0.00	0.00
0.00	0.00	0.00	0.00	0.00	0.00	0.00	0.00	0.00
0.00	0.00	0.00	0.00	0.00	0.00	0.00	0.00	0.00
0.00	0.00	0.00	0.00	0.00	0.00	0.00	0.00	0.00
0.00	0.00	0.00	0.00	0.00	0.00	0.00	0.00	0.00
0.00	0.00	0.00	0.00	0.00	0.00	0.00	0.00	0.00
0.00	0.00	0.00	0.00	0.00	0.00	0.00	0.00	0.00
0.00	0.00	0.00	0.00	0.00	0.00	0.00	0.00	0.00
0.00	0.00	0.00	0.00	0.00	0.00	0.00	0.00	0.00
0.00	0.00	0.00	0.00	0.00	0.00	0.00	0.00	0.00
0.00	0.00	0.00	0.00	0.00	0.00	0.00	0.00	0.00

0.00	0.00	0.00	0.00	0.00	0.00	0.00	0.00	0.00
0.00	0.00	0.00	0.00	0.00	0.00	0.00	3.00	0.00
0.00	0.00	0.00	0.00	0.00	0.00	0.00	3.00	0.00
0.00	0.00	0.00	0.00	0.00	0.00	0.00	3.00	0.00
0.00	0.00	0.00	0.00	0.00	0.00	0.00	3.00	0.00
0.00	0.00	0.00	0.00	0.00	0.00	0.00	0.00	0.00
0.00	0.00	3.00	0.00	0.00	0.00	0.00	0.00	0.00
0.00	0.00	3.00	0.00	0.00	0.00	0.00	0.00	0.00
0.00	0.00	3.00	0.00	0.00	0.00	0.00	0.00	0.00
0.00	0.00	3.00	0.00	0.00	0.00	0.00	0.00	0.00
3.00	0.00	0.00	0.00	0.00	0.00	0.00	0.00	0.00
3.00	0.00	0.00	0.00	0.00	0.00	0.00	0.00	0.00
0.00	0.00	0.00	0.00	0.00	0.00	0.00	0.00	0.00
0.00	0.00	0.00	0.00	0.00	0.00	0.00	0.00	0.00
0.00	0.00	0.00	0.00	0.00	0.00	0.00	0.00	0.00
0.00	0.00	0.00	0.00	0.00	0.00	0.00	0.00	0.00
0.00	0.00	0.00	0.00	0.00	0.00	0.00	0.00	0.00
0.00	0.00	0.00	0.00	0.00	0.00	0.00	0.00	0.00
0.00	0.00	0.00	0.00	0.00	0.00	0.00	0.00	0.00
0.00	0.00	0.00	0.00	0.00	0.00	0.00	0.00	0.00
0.00	0.00	0.00	0.00	0.00	0.00	0.00	0.00	0.00
0.00	0.00	0.00	0.00	0.00	0.00	0.00	0.00	0.00
0.00	0.00	0.00	0.00	0.00	0.00	0.00	0.00	0.00
0.00	0.00	0.00	0.00	0.00	0.00	0.00	0.00	0.00
0.00	0.00	0.00	0.00	0.00	0.00	0.00	0.00	0.00
0.00	0.00	0.00	0.00	0.00	0.00	0.00	0.00	0.00
0.00	0.00	0.00	0.00	0.00	0.00	0.00	0.00	0.00
0.00	0.00	0.00	0.00	0.00	0.00	0.00	0.00	0.00
0.00	0.00	0.00	0.00	0.00	0.00	0.00	0.00	0.00
0.00	0.00	0.00	0.00	0.00	0.00	0.00	0.00	0.00
0.00	0.00	0.00	0.00	0.00	0.00	0.00	0.00	0.00
0.00	0.00	0.00	0.00	0.00	0.00	0.00	0.00	0.00
0.00	0.00	0.00	0.00	0.00	0.00	0.00	0.00	0.00
0.00	0.00	0.00	0.00	0.00	0.00	0.00	0.00	0.00
0.00	0.00	0.00	0.00	0.00	0.00	0.00	0.00	0.00
0.00	0.00	0.00	0.00	0.00	0.00	0.00	0.00	0.00
0.00	0.00	0.00	0.00	0.00	0.00	0.00	0.00	0.00

0.00	0.00	0.00	0.00	0.00	0.00	0.00	0.00	0.00
0.00	0.00	0.00	0.00	0.00	0.00	0.00	0.00	0.00
0.00	0.00	0.00	0.00	0.00	0.00	0.00	0.00	0.00
0.00	0.00	0.00	0.00	0.00	0.00	0.00	0.00	0.00
0.00	0.00	0.00	0.00	0.00	0.00	0.00	0.00	0.00
0.00	0.00	0.00	0.00	0.00	0.00	0.00	0.00	0.00
0.00	0.00	0.00	0.00	0.00	0.00	0.00	0.00	0.00
0.00	0.00	0.00	0.00	0.00	0.00	0.00	0.00	0.00
0.00	0.00	0.00	0.00	0.00	0.00	0.00	0.00	0.00
0.00	0.00	0.00	0.00	0.00	0.00	0.00	0.00	0.00
0.00	0.00	0.00	0.00	0.00	0.00	0.00	0.00	0.00
0.00	0.00	0.00	0.00	0.00	0.00	0.00	0.00	0.00
0.00	0.00	0.00	0.00	0.00	0.00	0.00	0.00	0.00
0.00	0.00	0.00	0.00	0.00	0.00	0.00	0.00	0.00
0.00	0.00	0.00	0.00	0.00	0.00	0.00	0.00	0.00
0.00	0.00	0.00	0.00	0.00	0.00	0.00	0.00	0.00
0.00	0.00	0.00	0.00	0.00	0.00	0.00	0.00	0.00
0.00	0.00	0.00	0.00	0.00	0.00	0.00	0.00	0.00
0.00	0.00	0.00	0.00	0.00	0.00	0.00	0.00	0.00
0.00	0.00	0.00	0.00	0.00	0.00	0.00	0.00	0.00
0.00	0.00	0.00	0.00	0.00	0.00	0.00	0.00	0.00
0.00	0.00	0.00	0.00	0.00	0.00	0.00	0.00	0.00
0.00	0.00	0.00	0.00	0.00	0.00	0.00	0.00	0.00
0.00	0.00	0.00	0.00	0.00	0.00	0.00	0.00	0.00
0.00	0.00	0.00	0.00	0.00	0.00	0.00	0.00	0.00
0.00	0.00	0.00	0.00	0.00	0.00	0.00	0.00	0.00
0.00	0.00	0.00	0.00	0.00	0.00	0.00	0.00	0.00
0.00	0.00	0.00	0.00	0.00	0.00	0.00	0.00	0.00
0.00	0.00	0.00	0.00	0.00	0.00	0.00	0.00	0.00
0.00	0.00	0.00	0.00	0.00	0.00	0.00	0.00	0.00
0.00	0.00	0.00	0.00	0.00	0.00	0.00	0.00	0.00
0.00	0.00	0.00	0.00	0.00	0.00	0.00	0.00	0.00
0.00	0.00	0.00	0.00	0.00	0.00	0.00	0.00	0.00
0.00	0.00	0.00	0.00	0.00	0.00	0.00	0.00	0.00
0.00	0.00	0.00	0.00	0.00	0.00	0.00	0.00	0.00
0.00	0.00	0.00	0.00	0.00	0.00	0.00	0.00	0.00
0.00	0.00	0.00	0.00	0.00	0.00	0.00	0.00	0.00

0.00	0.00	0.00	0.00	0.00	0.00	0.00	0.00	0.00
0.00	0.00	0.00	0.00	0.00	0.00	0.00	0.00	0.00
0.00	0.00	0.00	0.00	0.00	0.00	0.00	0.00	0.00
0.00	0.00	0.00	0.00	0.00	0.00	0.00	0.00	0.00
0.00	0.00	0.00	0.00	0.00	0.00	0.00	0.00	0.00
0.00	0.00	0.00	0.00	0.00	0.00	0.00	0.00	0.00
0.00	0.00	0.00	0.00	0.00	0.00	0.00	0.00	0.00
0.00	0.00	0.00	0.00	0.00	0.00	0.00	0.00	0.00
0.00	0.00	0.00	0.00	0.00	0.00	0.00	0.00	0.00
0.00	0.00	0.00	0.00	0.00	0.00	0.00	0.00	0.00
0.00	0.00	0.00	0.00	0.00	0.00	0.00	0.00	0.00
0.00	0.00	0.00	0.00	0.00	0.00	0.00	0.00	0.00
0.00	0.00	0.00	0.00	0.00	0.00	0.00	0.00	0.00
0.00	0.00	0.00	0.00	0.00	0.00	0.00	0.00	0.00
0.00	0.00	0.00	0.00	0.00	0.00	0.00	0.00	0.00
0.00	0.00	0.00	0.00	0.00	0.00	0.00	0.00	0.00
0.00	0.00	0.00	0.00	0.00	0.00	0.00	0.00	0.00
0.00	0.00	0.00	0.00	0.00	0.00	0.00	0.00	0.00
0.00	0.00	0.00	0.00	0.00	0.00	0.00	0.00	0.00
0.00	0.00	0.00	0.00	0.00	0.00	0.00	0.00	0.00
0.00	0.00	0.00	0.00	0.00	0.00	0.00	0.00	0.00
0.00	0.00	0.00	0.00	0.00	0.00	0.00	0.00	0.00
0.00	0.00	0.00	0.00	0.00	0.00	0.00	0.00	0.00
0.00	0.00	0.00	0.00	0.00	0.00	0.00	0.00	0.00
0.00	0.00	0.00	0.00	0.00	0.00	0.00	0.00	0.00
0.00	0.00	0.00	0.00	0.00	0.00	0.00	0.00	0.00
0.00	0.00	0.00	0.00	0.00	0.00	0.00	0.00	0.00
0.00	0.00	0.00	0.00	0.00	0.00	0.00	0.00	0.00
0.00	0.00	0.00	0.00	0.00	0.00	0.00	0.00	0.00
0.00	0.00	0.00	0.00	0.00	0.00	0.00	0.00	0.00
0.00	0.00	0.00	0.00	0.00	0.00	0.00	0.00	0.00
0.00	0.00	0.00	0.00	0.00	0.00	0.00	0.00	0.00
0.00	0.00	0.00	0.00	0.00	0.00	0.00	0.00	0.00
0.00	0.00	0.00	0.00	0.00	0.00	0.00	0.00	0.00
0.00	0.00	0.00	0.00	0.00	0.00	0.00	0.00	0.00
0.00	0.00	0.00	0.00	0.00	0.00	0.00	0.00	0.00
0.00	0.00	0.00	0.00	0.00	0.00	0.00	0.00	0.00

0.00	0.00	0.00	0.00	0.00	0.00	0.00	0.00	0.00
0.00	0.00	0.00	0.00	0.00	0.00	0.00	0.00	0.00
0.00	0.00	0.00	0.00	0.00	0.00	0.00	0.00	0.00
0.00	0.00	0.00	0.00	0.00	0.00	0.00	0.00	0.00
0.00	0.00	0.00	0.00	0.00	0.00	0.00	0.00	0.00
0.00	0.00	0.00	0.00	0.00	0.00	0.00	0.00	0.00
0.00	0.00	0.00	0.00	0.00	0.00	0.00	0.00	2.00
0.00	0.00	0.00	0.00	0.00	0.00	0.00	0.00	2.00
0.00	0.00	0.00	0.00	0.00	0.00	0.00	0.00	0.00
0.00	0.00	0.00	0.00	0.00	0.00	2.00	0.00	0.00
0.00	0.00	0.00	0.00	0.00	0.00	2.00	0.00	0.00
0.00	0.00	0.00	0.00	0.00	2.00	0.00	0.00	0.00
0.00	0.00	0.00	0.00	0.00	2.00	0.00	0.00	0.00
0.00	0.00	0.00	0.00	0.00	2.00	0.00	0.00	0.00
0.00	0.00	0.00	0.00	0.00	2.00	0.00	0.00	0.00
0.00	0.00	0.00	0.00	0.00	2.00	0.00	0.00	0.00
0.00	0.00	0.00	0.00	2.00	0.00	0.00	0.00	0.00
0.00	0.00	0.00	0.00	2.00	0.00	0.00	0.00	0.00
0.00	0.00	0.00	2.00	0.00	0.00	0.00	0.00	0.00
0.00	0.00	0.00	2.00	0.00	0.00	0.00	0.00	0.00
0.00	0.00	0.00	2.00	0.00	0.00	0.00	0.00	0.00
0.00	0.00	0.00	2.00	0.00	0.00	0.00	0.00	0.00
0.00	0.00	0.00	2.00	0.00	0.00	0.00	0.00	0.00
0.00	0.00	0.00	2.00	0.00	0.00	0.00	0.00	0.00
0.00	0.00	0.00	2.00	0.00	0.00	0.00	0.00	0.00
0.00	0.00	0.00	0.00	0.00	0.00	0.00	0.00	0.00
0.00	0.00	0.00	0.00	0.00	0.00	0.00	0.00	0.00
0.00	0.00	0.00	0.00	0.00	0.00	0.00	0.00	0.00
0.00	0.00	0.00	0.00	0.00	0.00	0.00	0.00	0.00
0.00	0.00	0.00	0.00	0.00	0.00	0.00	0.00	0.00
0.00	0.00	0.00	0.00	0.00	0.00	0.00	0.00	0.00
0.00	0.00	0.00	0.00	0.00	0.00	0.00	0.00	0.00
0.00	0.00	0.00	0.00	0.00	0.00	0.00	0.00	0.00
0.00	0.00	0.00	0.00	0.00	0.00	0.00	0.00	0.00
0.00	0.00	0.00	0.00	0.00	0.00	0.00	0.00	0.00
0.00	0.00	0.00	0.00	0.00	0.00	0.00	0.00	0.00
0.00	0.00	0.00	0.00	0.00	0.00	0.00	0.00	0.00

0.00	0.00	0.00	0.00	0.00	0.00	0.00	0.00	0.00
0.00	0.00	0.00	0.00	0.00	0.00	0.00	0.00	0.00
0.00	0.00	0.00	0.00	0.00	0.00	0.00	0.00	0.00
0.00	0.00	0.00	0.00	0.00	0.00	0.00	0.00	0.00
0.00	0.00	0.00	0.00	0.00	0.00	0.00	0.00	0.00
0.00	0.00	0.00	0.00	0.00	0.00	0.00	0.00	0.00
0.00	0.00	0.00	0.00	0.00	0.00	0.00	0.00	0.00
0.00	0.00	0.00	0.00	0.00	0.00	0.00	0.00	0.00
0.00	0.00	0.00	0.00	0.00	0.00	0.00	0.00	0.00
0.00	0.00	0.00	0.00	0.00	0.00	0.00	0.00	0.00
0.00	0.00	0.00	0.00	0.00	0.00	0.00	0.00	0.00
0.00	0.00	0.00	0.00	0.00	0.00	0.00	0.00	0.00
0.00	0.00	0.00	0.00	0.00	0.00	0.00	0.00	0.00
0.00	0.00	0.00	0.00	0.00	0.00	0.00	0.00	0.00
0.00	0.00	0.00	0.00	0.00	0.00	0.00	0.00	0.00
0.00	0.00	0.00	0.00	0.00	0.00	0.00	0.00	0.00
0.00	0.00	0.00	0.00	0.00	0.00	0.00	0.00	0.00
0.00	0.00	0.00	0.00	0.00	0.00	0.00	0.00	0.00
0.00	0.00	0.00	0.00	0.00	0.00	0.00	2.00	0.00
0.00	0.00	0.00	0.00	0.00	0.00	0.00	0.00	0.00
0.00	0.00	0.00	0.00	0.00	0.00	0.00	0.00	0.00
0.00	0.00	2.00	0.00	0.00	0.00	0.00	0.00	0.00
0.00	0.00	2.00	0.00	0.00	0.00	0.00	0.00	0.00
2.00	0.00	0.00	0.00	0.00	0.00	0.00	0.00	0.00
0.00	0.00	0.00	0.00	0.00	0.00	0.00	0.00	0.00
0.00	0.00	0.00	0.00	0.00	0.00	0.00	0.00	0.00
0.00	0.00	0.00	0.00	0.00	0.00	0.00	0.00	0.00
0.00	0.00	0.00	0.00	0.00	0.00	0.00	0.00	0.00
0.00	0.00	0.00	0.00	0.00	0.00	0.00	0.00	0.00
0.00	0.00	0.00	0.00	0.00	0.00	0.00	0.00	0.00
0.00	0.00	0.00	0.00	0.00	0.00	0.00	0.00	0.00
0.00	0.00	0.00	0.00	0.00	0.00	0.00	0.00	0.00
0.00	0.00	0.00	0.00	0.00	0.00	0.00	0.00	0.00

C1	D1
414.00	29285.00
0.00	35.00
9307.00	7246.00
0.00	8.00
0.00	0.00
0.00	0.00
44.00	86.00
0.00	0.00
386.00	12885.00
0.00	0.00
30.00	47.00
11219.00	25.00
13.00	6065.00
5.00	4.00
0.00	0.00
0.00	0.00
29.00	39.00
0.00	0.00
77.00	74.00
23653.00	0.00
0.00	0.00
0.00	0.00
0.00	34.00
0.00	0.00
0.00	0.00
314.00	67.00
0.00	0.00
0.00	0.00
0.00	0.00
0.00	0.00
0.00	0.00
0.00	490.00
0.00	0.00
0.00	521.00
0.00	0.00

0.00	0.00
341.00	92.00
292.00	169.00
0.00	0.00
0.00	0.00
4.00	0.00
0.00	0.00
253.00	0.00
258.00	0.00
0.00	0.00
0.00	0.00
0.00	0.00
5876.00	0.00
153.00	0.00
0.00	0.00
14.00	9.00
0.00	0.00
0.00	0.00
0.00	0.00
0.00	0.00
8.00	0.00
0.00	0.00
0.00	0.00
0.00	0.00
61.00	105.00
0.00	0.00
243.00	10.00
0.00	0.00
0.00	0.00
0.00	0.00
316.00	0.00
0.00	0.00
0.00	0.00
0.00	0.00
0.00	0.00
0.00	11.00
0.00	5.00

0.00	0.00
5.00	0.00
0.00	0.00
13.00	0.00
246.00	0.00
0.00	0.00
0.00	0.00
0.00	0.00
0.00	10.00
0.00	0.00
0.00	0.00
0.00	0.00
158.00	164.00
7.00	0.00
0.00	0.00
0.00	0.00
0.00	0.00
0.00	0.00
0.00	54.00
0.00	0.00
0.00	0.00
0.00	25.00
18.00	0.00
0.00	0.00
254.00	0.00
0.00	0.00
0.00	0.00
0.00	0.00
0.00	78.00
0.00	0.00
0.00	0.00
0.00	47.00
0.00	0.00
51.00	61.00
0.00	0.00
0.00	0.00
11.00	21.00

0.00	15.00
0.00	0.00
0.00	0.00
31.00	34.00
0.00	63.00
0.00	0.00
0.00	0.00
0.00	0.00
107.00	121.00
0.00	0.00
0.00	0.00
0.00	0.00
0.00	0.00
0.00	79.00
0.00	0.00
0.00	0.00
0.00	0.00
0.00	0.00
0.00	0.00
0.00	0.00
0.00	0.00
0.00	0.00
194.00	0.00
0.00	0.00
0.00	0.00
0.00	0.00
0.00	0.00
0.00	0.00
0.00	0.00
0.00	0.00
44.00	76.00
0.00	0.00
0.00	252.00
0.00	0.00
0.00	0.00
0.00	0.00
0.00	0.00

0.00	0.00
0.00	0.00
25.00	15.00
0.00	0.00
0.00	0.00
0.00	0.00
0.00	0.00
0.00	0.00
0.00	0.00
0.00	7.00
0.00	0.00
0.00	0.00
0.00	0.00
244.00	0.00
0.00	0.00
0.00	0.00
0.00	0.00
97.00	136.00
0.00	0.00
0.00	0.00
0.00	0.00
0.00	0.00
0.00	0.00
0.00	0.00
0.00	0.00
0.00	0.00
43.00	49.00
0.00	10.00
0.00	0.00
0.00	0.00
0.00	0.00
32.00	31.00
0.00	0.00
0.00	0.00
0.00	0.00
80.00	88.00
246.00	0.00

0.00	0.00
0.00	0.00
0.00	0.00
0.00	0.00
7.00	0.00
0.00	0.00
0.00	0.00
32.00	0.00
0.00	0.00
100.00	112.00
0.00	0.00
0.00	0.00
9.00	3.00
0.00	0.00
0.00	0.00
0.00	0.00
0.00	0.00
0.00	0.00
624.00	0.00
0.00	0.00
0.00	0.00
0.00	0.00
0.00	0.00
0.00	0.00
0.00	0.00
0.00	0.00
0.00	4.00
0.00	0.00
0.00	0.00
0.00	0.00
0.00	0.00
0.00	124.00
0.00	0.00
0.00	0.00
0.00	0.00
0.00	0.00
59.00	48.00

0.00	0.00
0.00	0.00
0.00	0.00
8.00	15.00
0.00	0.00
0.00	0.00
0.00	0.00
0.00	0.00
0.00	0.00
0.00	0.00
0.00	0.00
0.00	0.00
0.00	0.00
0.00	0.00
146.00	2.00
0.00	0.00
0.00	0.00
0.00	0.00
0.00	0.00
0.00	0.00
45.00	40.00
0.00	0.00
0.00	0.00
0.00	0.00
0.00	0.00
29.00	0.00
0.00	0.00
0.00	0.00
474.00	0.00
0.00	0.00
0.00	0.00
0.00	0.00
0.00	0.00
0.00	0.00
0.00	0.00
0.00	0.00
0.00	0.00

0.00	0.00
0.00	0.00
0.00	0.00
0.00	0.00
0.00	0.00
0.00	0.00
0.00	0.00
0.00	0.00
0.00	0.00
0.00	0.00
0.00	0.00
0.00	0.00
0.00	0.00
0.00	0.00
0.00	0.00
0.00	0.00
41.00	52.00
0.00	0.00
307.00	0.00
0.00	0.00
363.00	0.00
0.00	0.00
0.00	0.00
0.00	0.00
0.00	27.00
0.00	0.00
0.00	0.00
0.00	0.00
0.00	0.00
39.00	4.00
0.00	0.00
345.00	0.00
0.00	0.00
285.00	0.00
0.00	0.00
0.00	0.00
0.00	0.00

0.00	0.00
22.00	14.00
0.00	0.00
0.00	0.00
0.00	0.00
0.00	0.00
0.00	0.00
0.00	0.00
0.00	0.00
29.00	34.00
107.00	0.00
325.00	0.00
0.00	0.00
14.00	31.00
0.00	0.00
316.00	0.00
0.00	0.00
0.00	59.00
0.00	0.00
0.00	0.00
0.00	0.00
0.00	0.00
0.00	0.00
0.00	0.00
0.00	0.00
0.00	0.00
0.00	0.00
0.00	0.00
0.00	0.00
0.00	0.00
0.00	0.00
0.00	0.00
0.00	0.00
0.00	0.00
0.00	0.00
0.00	0.00
0.00	0.00

0.00	0.00
0.00	0.00
33.00	49.00
0.00	0.00
0.00	0.00
18.00	0.00
271.00	0.00
0.00	0.00
0.00	28.00
28.00	0.00
0.00	0.00
0.00	0.00
0.00	0.00
0.00	0.00
61.00	0.00
261.00	0.00
0.00	0.00
0.00	0.00
0.00	0.00
0.00	0.00
0.00	0.00
0.00	0.00
0.00	3.00
19.00	0.00
0.00	0.00
0.00	0.00
0.00	0.00
0.00	0.00
0.00	0.00
0.00	0.00
0.00	0.00
0.00	0.00
0.00	0.00
0.00	0.00
0.00	0.00
0.00	0.00
0.00	0.00

0.00	0.00
0.00	0.00
0.00	0.00
0.00	0.00
0.00	231.00
0.00	0.00
55.00	64.00
0.00	0.00
0.00	0.00
0.00	32.00
0.00	0.00
0.00	0.00
15.00	12.00
19.00	19.00
0.00	0.00
0.00	0.00
0.00	0.00
0.00	0.00
0.00	0.00
0.00	0.00
14.00	0.00
0.00	0.00
0.00	0.00
0.00	0.00
15.00	24.00
0.00	0.00
0.00	0.00
15.00	15.00
7.00	13.00
0.00	0.00
0.00	0.00
0.00	0.00
11.00	7.00
0.00	0.00
0.00	0.00
0.00	0.00
0.00	0.00

0.00	0.00
0.00	0.00
0.00	0.00
0.00	0.00
0.00	0.00
0.00	0.00
0.00	0.00
0.00	0.00
0.00	0.00
0.00	0.00
0.00	0.00
0.00	0.00
0.00	193.00
0.00	0.00
0.00	0.00
35.00	28.00
0.00	0.00
14.00	21.00
0.00	185.00
3.00	15.00
6.00	2.00
0.00	0.00
0.00	0.00
0.00	14.00
0.00	50.00
0.00	0.00
0.00	0.00
0.00	0.00
0.00	9.00
0.00	39.00
0.00	0.00
0.00	0.00
15.00	7.00
0.00	0.00
0.00	0.00
0.00	0.00
32.00	0.00

0.00	0.00
0.00	0.00
0.00	0.00
0.00	0.00
0.00	33.00
0.00	0.00
0.00	0.00
0.00	0.00
171.00	0.00
0.00	0.00
0.00	0.00
0.00	0.00
0.00	0.00
0.00	0.00
0.00	0.00
168.00	0.00
0.00	0.00
0.00	0.00
0.00	0.00
0.00	0.00
0.00	0.00
0.00	0.00
0.00	0.00
0.00	75.00
0.00	0.00
0.00	0.00
0.00	0.00
0.00	0.00
0.00	0.00
0.00	0.00
0.00	0.00
0.00	134.00
0.00	0.00
0.00	155.00
0.00	0.00
156.00	0.00
156.00	0.00

0.00	0.00
0.00	0.00
0.00	0.00
0.00	0.00
0.00	0.00
0.00	0.00
0.00	0.00
0.00	0.00
0.00	0.00
0.00	0.00
0.00	0.00
0.00	0.00
0.00	0.00
0.00	0.00
0.00	0.00
0.00	0.00
0.00	0.00
0.00	0.00
0.00	0.00
0.00	0.00
0.00	0.00
17.00	17.00
147.00	0.00
0.00	3.00
0.00	0.00
0.00	0.00
2.00	0.00
0.00	0.00
0.00	0.00
0.00	0.00
0.00	0.00
0.00	0.00
0.00	0.00
0.00	0.00
0.00	0.00
0.00	0.00
0.00	0.00

23.00	0.00
15.00	17.00
0.00	0.00
18.00	31.00
0.00	0.00
0.00	3.00
0.00	0.00
0.00	0.00
0.00	0.00
0.00	0.00
0.00	0.00
0.00	0.00
0.00	0.00
0.00	0.00
0.00	0.00
20.00	0.00
0.00	0.00
0.00	0.00
0.00	2.00
0.00	0.00
0.00	0.00
0.00	0.00
0.00	0.00
0.00	0.00
0.00	0.00
0.00	0.00
42.00	0.00
0.00	0.00
0.00	0.00
106.00	0.00
0.00	0.00
0.00	0.00
0.00	0.00
0.00	0.00
0.00	0.00
0.00	116.00
0.00	0.00

0.00	0.00
0.00	0.00
0.00	0.00
113.00	0.00
0.00	65.00
0.00	0.00
0.00	0.00
0.00	0.00
0.00	0.00
0.00	0.00
0.00	0.00
0.00	5.00
0.00	0.00
109.00	0.00
0.00	0.00
0.00	0.00
0.00	0.00
0.00	0.00
0.00	0.00
0.00	0.00
0.00	0.00
0.00	106.00
0.00	0.00
0.00	0.00
0.00	0.00
0.00	0.00
0.00	0.00
0.00	0.00
0.00	103.00
0.00	0.00
0.00	0.00
0.00	0.00
0.00	102.00
0.00	0.00
0.00	0.00
0.00	0.00
0.00	0.00

32.00	0.00
101.00	0.00
0.00	0.00
0.00	0.00
0.00	0.00
0.00	0.00
0.00	0.00
0.00	0.00
0.00	0.00
0.00	0.00
0.00	0.00
0.00	0.00
0.00	0.00
0.00	0.00
0.00	0.00
0.00	0.00
0.00	0.00
0.00	0.00
0.00	0.00
0.00	0.00
0.00	0.00
0.00	0.00
6.00	10.00
0.00	0.00
0.00	0.00
0.00	0.00
0.00	0.00
0.00	0.00
0.00	0.00
0.00	0.00
0.00	0.00
0.00	0.00
0.00	0.00
0.00	0.00
0.00	0.00
0.00	0.00
0.00	0.00

0.00	0.00
0.00	0.00
0.00	0.00
0.00	0.00
0.00	0.00
0.00	0.00
0.00	16.00
0.00	0.00
0.00	0.00
3.00	0.00
0.00	0.00
0.00	0.00
0.00	0.00
0.00	7.00
0.00	0.00
0.00	0.00
0.00	0.00
0.00	22.00
0.00	0.00
0.00	0.00
0.00	0.00
0.00	0.00
83.00	0.00
0.00	0.00
0.00	0.00
0.00	0.00
0.00	0.00
0.00	10.00
0.00	0.00
0.00	0.00
0.00	0.00
0.00	0.00
0.00	0.00
3.00	10.00
0.00	0.00
0.00	0.00
0.00	0.00

0.00	0.00
0.00	0.00
0.00	0.00
0.00	0.00
0.00	0.00
0.00	0.00
0.00	0.00
0.00	0.00
0.00	13.00
0.00	0.00
0.00	0.00
0.00	0.00
0.00	0.00
0.00	0.00
0.00	0.00
0.00	0.00
16.00	19.00
0.00	0.00
0.00	0.00
0.00	0.00
0.00	0.00
0.00	0.00
2.00	0.00
0.00	0.00
0.00	0.00
0.00	0.00
0.00	0.00
0.00	0.00
5.00	8.00
0.00	0.00
0.00	0.00
0.00	0.00
0.00	0.00
0.00	0.00
0.00	0.00
0.00	37.00
0.00	0.00

0.00	70.00
0.00	0.00
7.00	10.00
0.00	0.00
0.00	0.00
0.00	0.00
0.00	0.00
0.00	0.00
0.00	0.00
0.00	0.00
35.00	33.00
10.00	11.00
0.00	0.00
0.00	0.00
0.00	0.00
0.00	68.00
0.00	0.00
0.00	0.00
0.00	0.00
0.00	4.00
0.00	0.00
0.00	0.00
0.00	0.00
0.00	0.00
0.00	0.00
5.00	0.00
0.00	0.00
0.00	0.00
0.00	0.00
0.00	0.00
0.00	0.00
0.00	0.00
0.00	0.00
0.00	0.00
0.00	0.00
33.00	0.00
0.00	0.00

23.00	0.00
0.00	0.00
0.00	0.00
0.00	0.00
0.00	0.00
0.00	0.00
0.00	0.00
0.00	0.00
0.00	0.00
0.00	0.00
0.00	0.00
0.00	0.00
0.00	0.00
0.00	0.00
0.00	0.00
6.00	7.00
0.00	0.00
0.00	0.00
14.00	0.00
0.00	0.00
0.00	0.00
0.00	0.00
0.00	0.00
0.00	0.00
0.00	0.00
0.00	0.00
0.00	0.00
0.00	0.00
0.00	0.00
0.00	0.00
0.00	0.00
0.00	0.00
0.00	0.00
0.00	0.00
0.00	0.00
0.00	0.00
0.00	0.00

0.00	0.00
0.00	0.00
0.00	0.00
0.00	0.00
0.00	0.00
0.00	0.00
0.00	0.00
0.00	0.00
0.00	0.00
0.00	0.00
0.00	0.00
0.00	0.00
0.00	0.00
0.00	0.00
0.00	0.00
0.00	0.00
0.00	0.00
0.00	0.00
0.00	0.00
0.00	0.00
0.00	0.00
0.00	0.00
0.00	0.00
0.00	0.00
0.00	0.00
0.00	0.00
12.00	10.00
4.00	0.00
0.00	0.00
0.00	0.00
0.00	56.00
0.00	0.00
0.00	0.00
0.00	0.00
0.00	0.00
0.00	0.00
0.00	0.00

0.00	0.00
0.00	0.00
8.00	13.00
0.00	0.00
0.00	0.00
0.00	0.00
0.00	54.00
0.00	0.00
0.00	0.00
0.00	0.00
11.00	10.00
0.00	0.00
0.00	0.00
24.00	0.00
6.00	9.00
0.00	0.00
0.00	0.00
0.00	0.00
0.00	0.00
0.00	0.00
0.00	0.00
0.00	0.00
0.00	0.00
0.00	0.00
0.00	0.00
0.00	0.00
0.00	0.00
0.00	0.00
0.00	0.00
0.00	0.00
0.00	0.00
0.00	0.00
0.00	0.00
0.00	0.00
0.00	35.00
0.00	0.00
0.00	0.00

0.00	0.00
0.00	0.00
0.00	0.00
9.00	8.00
0.00	0.00
0.00	0.00
0.00	0.00
0.00	0.00
0.00	0.00
0.00	0.00
0.00	0.00
0.00	0.00
0.00	0.00
0.00	0.00
10.00	0.00
0.00	0.00
0.00	0.00
0.00	0.00
0.00	47.00
0.00	0.00
0.00	0.00
0.00	0.00
0.00	0.00
0.00	0.00
0.00	0.00
0.00	4.00
0.00	0.00
0.00	0.00
0.00	0.00
0.00	0.00
0.00	0.00
0.00	4.00
0.00	0.00
0.00	0.00
0.00	0.00
0.00	0.00
0.00	0.00

0.00	0.00
0.00	0.00
0.00	0.00
0.00	0.00
0.00	0.00
0.00	0.00
0.00	0.00
0.00	0.00
0.00	0.00
0.00	0.00
0.00	0.00
0.00	0.00
0.00	0.00
0.00	0.00
0.00	0.00
5.00	5.00
0.00	0.00
0.00	0.00
0.00	0.00
0.00	0.00
0.00	0.00
0.00	0.00
0.00	0.00
0.00	0.00
0.00	0.00
0.00	0.00
0.00	12.00
0.00	42.00
0.00	0.00
0.00	0.00
0.00	0.00
0.00	0.00
0.00	0.00
0.00	0.00
0.00	0.00
0.00	0.00
0.00	0.00

0.00	0.00
0.00	0.00
0.00	0.00
0.00	0.00
0.00	0.00
0.00	0.00
0.00	0.00
0.00	0.00
0.00	0.00
0.00	0.00
0.00	0.00
0.00	0.00
16.00	0.00
0.00	0.00
0.00	0.00
0.00	0.00
0.00	0.00
0.00	0.00
0.00	0.00
0.00	0.00
0.00	0.00
0.00	0.00
0.00	0.00
0.00	4.00
0.00	0.00
0.00	0.00
0.00	0.00
0.00	0.00
0.00	0.00
0.00	0.00
0.00	5.00
0.00	0.00
0.00	0.00
0.00	0.00
0.00	0.00
0.00	0.00
0.00	0.00

0.00	0.00
0.00	0.00
0.00	0.00
0.00	0.00
0.00	0.00
0.00	0.00
0.00	0.00
0.00	0.00
0.00	0.00
0.00	0.00
0.00	0.00
0.00	0.00
0.00	0.00
0.00	0.00
0.00	0.00
0.00	0.00
0.00	0.00
0.00	0.00
0.00	0.00
0.00	0.00
4.00	6.00
0.00	0.00
0.00	0.00
0.00	0.00
0.00	0.00
0.00	0.00
0.00	0.00
0.00	0.00
7.00	8.00
0.00	0.00
0.00	0.00
0.00	0.00
0.00	0.00
0.00	5.00
0.00	0.00
0.00	0.00
0.00	0.00

0.00	0.00
0.00	0.00
0.00	0.00
0.00	0.00
0.00	0.00
0.00	0.00
0.00	0.00
0.00	0.00
0.00	0.00
0.00	0.00
0.00	0.00
0.00	0.00
5.00	3.00
0.00	10.00
0.00	0.00
0.00	0.00
0.00	0.00
0.00	0.00
0.00	0.00
0.00	0.00
0.00	0.00
0.00	0.00
0.00	0.00
0.00	0.00
0.00	0.00
0.00	0.00
13.00	0.00
9.00	0.00
0.00	0.00
0.00	10.00
0.00	0.00
0.00	0.00
0.00	0.00
0.00	0.00
0.00	0.00
0.00	0.00
0.00	0.00

0.00	0.00
0.00	0.00
0.00	0.00
0.00	0.00
0.00	0.00
0.00	0.00
0.00	0.00
0.00	0.00
0.00	0.00
0.00	0.00
0.00	0.00
0.00	0.00
0.00	30.00
0.00	0.00
0.00	0.00
0.00	0.00
0.00	0.00
0.00	6.00
0.00	0.00
10.00	0.00
0.00	0.00
0.00	0.00
0.00	0.00
0.00	0.00
0.00	0.00
0.00	0.00
0.00	0.00
0.00	0.00
0.00	0.00
0.00	0.00
0.00	0.00
0.00	0.00
0.00	28.00
0.00	0.00
0.00	0.00
0.00	0.00
0.00	0.00

0.00	0.00
0.00	0.00
0.00	0.00
0.00	0.00
0.00	0.00
0.00	0.00
0.00	0.00
8.00	0.00
0.00	0.00
0.00	7.00
0.00	0.00
0.00	0.00
0.00	0.00
0.00	0.00
0.00	0.00
0.00	0.00
0.00	0.00
0.00	27.00
0.00	0.00
0.00	0.00
0.00	0.00
0.00	0.00
0.00	0.00
0.00	0.00
0.00	0.00
0.00	0.00
0.00	0.00
0.00	0.00
0.00	0.00
0.00	0.00
0.00	0.00
0.00	0.00
0.00	0.00
0.00	0.00
0.00	0.00
0.00	0.00
0.00	0.00

0.00	0.00
0.00	0.00
25.00	0.00
0.00	0.00
0.00	0.00
0.00	0.00
0.00	0.00
0.00	0.00
0.00	0.00
0.00	0.00
0.00	0.00
0.00	0.00
0.00	0.00
0.00	0.00
0.00	0.00
0.00	0.00
0.00	0.00
0.00	0.00
0.00	0.00
0.00	0.00
0.00	0.00
0.00	0.00
0.00	0.00
0.00	0.00
0.00	0.00
0.00	0.00
0.00	0.00
0.00	0.00
0.00	0.00
0.00	0.00
0.00	0.00
0.00	0.00
0.00	0.00
5.00	3.00
0.00	0.00
0.00	0.00
0.00	0.00

0.00	0.00
0.00	0.00
0.00	0.00
0.00	0.00
0.00	0.00
0.00	0.00
0.00	0.00
0.00	0.00
0.00	0.00
0.00	0.00
0.00	0.00
0.00	0.00
0.00	0.00
0.00	0.00
0.00	14.00
0.00	0.00
0.00	12.00
0.00	0.00
0.00	0.00
0.00	0.00
0.00	6.00
0.00	0.00
0.00	0.00
0.00	0.00
0.00	0.00
0.00	0.00
0.00	0.00
0.00	0.00
0.00	0.00
0.00	0.00
0.00	0.00
0.00	0.00
0.00	0.00
0.00	0.00
0.00	0.00
9.00	0.00
0.00	0.00

0.00	0.00
0.00	0.00
0.00	0.00
0.00	4.00
0.00	0.00
0.00	0.00
0.00	0.00
0.00	0.00
0.00	0.00
0.00	0.00
0.00	0.00
0.00	0.00
0.00	0.00
0.00	0.00
0.00	0.00
0.00	0.00
0.00	0.00
0.00	0.00
0.00	0.00
0.00	0.00
0.00	0.00
0.00	0.00
0.00	0.00
0.00	0.00
0.00	0.00
0.00	0.00
0.00	0.00
0.00	0.00
0.00	7.00
0.00	0.00
0.00	0.00
0.00	0.00
0.00	0.00
0.00	0.00
0.00	0.00
0.00	0.00
0.00	0.00

0.00	0.00
0.00	0.00
0.00	0.00
0.00	0.00
5.00	0.00
0.00	0.00
0.00	0.00
0.00	17.00
0.00	0.00
0.00	0.00
0.00	0.00
0.00	0.00
0.00	0.00
0.00	0.00
0.00	0.00
0.00	0.00
0.00	0.00
0.00	0.00
0.00	0.00
0.00	0.00
0.00	0.00
2.00	0.00
4.00	0.00
0.00	0.00
0.00	0.00
0.00	0.00
0.00	0.00
0.00	0.00
0.00	0.00
0.00	0.00
0.00	0.00
0.00	0.00
0.00	0.00
0.00	0.00
0.00	0.00
0.00	0.00
0.00	0.00

0.00	4.00
0.00	0.00
0.00	0.00
0.00	0.00
0.00	0.00
0.00	0.00
0.00	0.00
0.00	0.00
0.00	0.00
0.00	0.00
0.00	0.00
0.00	0.00
0.00	0.00
0.00	0.00
0.00	0.00
0.00	0.00
0.00	0.00
0.00	0.00
0.00	0.00
0.00	0.00
0.00	0.00
0.00	0.00
0.00	0.00
9.00	0.00
3.00	0.00
0.00	0.00
0.00	0.00
0.00	0.00
0.00	0.00
0.00	0.00
0.00	0.00
0.00	0.00
0.00	0.00
0.00	0.00
0.00	0.00
0.00	9.00
0.00	0.00

0.00	0.00
0.00	0.00
0.00	0.00
0.00	17.00
0.00	0.00
0.00	0.00
0.00	0.00
0.00	0.00
0.00	0.00
0.00	0.00
0.00	0.00
0.00	0.00
0.00	0.00
0.00	0.00
0.00	0.00
0.00	0.00
0.00	0.00
0.00	0.00
0.00	0.00
0.00	0.00
0.00	0.00
0.00	0.00
0.00	0.00
0.00	0.00
0.00	5.00
0.00	0.00
0.00	4.00
0.00	0.00
0.00	0.00
0.00	4.00
0.00	0.00
0.00	0.00
0.00	0.00
0.00	0.00
0.00	0.00
0.00	0.00
0.00	0.00

0.00	0.00
0.00	0.00
0.00	0.00
0.00	0.00
0.00	0.00
0.00	0.00
0.00	0.00
0.00	0.00
0.00	0.00
0.00	0.00
0.00	0.00
0.00	0.00
0.00	0.00
0.00	0.00
0.00	0.00
0.00	0.00
0.00	0.00
0.00	0.00
0.00	0.00
0.00	0.00
0.00	0.00
0.00	0.00
0.00	0.00
0.00	0.00
0.00	0.00
0.00	0.00
0.00	0.00
0.00	0.00
0.00	0.00
0.00	0.00
0.00	0.00
0.00	0.00
0.00	0.00
0.00	0.00
0.00	0.00
0.00	0.00
0.00	0.00

0.00	0.00
0.00	0.00
0.00	0.00
0.00	0.00
0.00	0.00
0.00	0.00
0.00	0.00
0.00	0.00
0.00	0.00
0.00	0.00
0.00	0.00
0.00	0.00
0.00	0.00
0.00	0.00
0.00	0.00
0.00	0.00
0.00	0.00
0.00	0.00
0.00	0.00
0.00	0.00
0.00	0.00
0.00	0.00
0.00	0.00
0.00	0.00
0.00	0.00
0.00	0.00
0.00	0.00
0.00	0.00
0.00	0.00
0.00	0.00
0.00	0.00
0.00	0.00
0.00	0.00
0.00	0.00
0.00	0.00
5.00	8.00
9.00	0.00

0.00	0.00
0.00	0.00
0.00	0.00
0.00	0.00
0.00	0.00
0.00	0.00
0.00	0.00
0.00	0.00
0.00	0.00
0.00	0.00
0.00	0.00
0.00	0.00
0.00	0.00
0.00	0.00
0.00	0.00
0.00	0.00
0.00	0.00
0.00	0.00
0.00	0.00
0.00	0.00
0.00	0.00
0.00	0.00
0.00	0.00
0.00	0.00
0.00	0.00
0.00	0.00
0.00	0.00
0.00	0.00
0.00	0.00
0.00	0.00
0.00	0.00
0.00	0.00
0.00	0.00
0.00	0.00
0.00	0.00
0.00	0.00
0.00	0.00

0.00	0.00
12.00	0.00
5.00	0.00
5.00	0.00
2.00	6.00
0.00	0.00
0.00	0.00
0.00	0.00
0.00	0.00
0.00	0.00
0.00	0.00
0.00	0.00
0.00	0.00
0.00	0.00
0.00	0.00
0.00	0.00
0.00	0.00
0.00	0.00
0.00	0.00
0.00	0.00
0.00	0.00
0.00	0.00
0.00	0.00
0.00	0.00
0.00	0.00
0.00	0.00
0.00	0.00
0.00	0.00
0.00	0.00
0.00	0.00
0.00	0.00
0.00	0.00
0.00	0.00
0.00	0.00
0.00	0.00
0.00	0.00
0.00	0.00

0.00	0.00
11.00	0.00
7.00	0.00
0.00	0.00
0.00	0.00
0.00	0.00
0.00	0.00
0.00	0.00
0.00	0.00
0.00	0.00
0.00	0.00
0.00	0.00
0.00	0.00
0.00	0.00
0.00	0.00
0.00	0.00
0.00	0.00
0.00	0.00
0.00	0.00
0.00	0.00
0.00	5.00
0.00	0.00
0.00	0.00
0.00	0.00
0.00	0.00
0.00	0.00
0.00	0.00
0.00	0.00
0.00	0.00
0.00	0.00
0.00	0.00
0.00	0.00
0.00	0.00
0.00	0.00
0.00	0.00
0.00	0.00
0.00	0.00

0.00	0.00
0.00	2.00
0.00	0.00
0.00	0.00
0.00	0.00
0.00	0.00
0.00	0.00
0.00	0.00
0.00	0.00
0.00	0.00
0.00	0.00
0.00	0.00
10.00	0.00
6.00	0.00
4.00	0.00
10.00	0.00
0.00	0.00
0.00	0.00
0.00	0.00
0.00	0.00
0.00	0.00
0.00	0.00
0.00	0.00
0.00	0.00
0.00	0.00
0.00	0.00
0.00	0.00
0.00	0.00
0.00	0.00
0.00	0.00
0.00	0.00
0.00	0.00
0.00	0.00
0.00	0.00
0.00	0.00
0.00	0.00
0.00	0.00

0.00	0.00
0.00	0.00
0.00	0.00
0.00	0.00
0.00	0.00
0.00	0.00
0.00	0.00
0.00	0.00
0.00	0.00
0.00	0.00
0.00	0.00
0.00	0.00
0.00	0.00
0.00	0.00
0.00	0.00
0.00	0.00
0.00	0.00
0.00	0.00
0.00	0.00
0.00	0.00
0.00	0.00
0.00	0.00
0.00	0.00
0.00	0.00
0.00	0.00
0.00	0.00
0.00	0.00
0.00	0.00
0.00	0.00
0.00	0.00
0.00	0.00
0.00	0.00
0.00	0.00
3.00	0.00
9.00	0.00
2.00	0.00
0.00	0.00

0.00	0.00
0.00	0.00
0.00	0.00
0.00	0.00
0.00	0.00
0.00	0.00
0.00	0.00
0.00	0.00
0.00	0.00
0.00	0.00
0.00	0.00
0.00	0.00
0.00	0.00
0.00	0.00
0.00	0.00
0.00	0.00
0.00	0.00
0.00	0.00
0.00	0.00
0.00	0.00
0.00	0.00
0.00	0.00
0.00	0.00
0.00	0.00
0.00	0.00
0.00	0.00
0.00	0.00
0.00	0.00
0.00	0.00
0.00	0.00
0.00	0.00
0.00	0.00
0.00	0.00
0.00	0.00
0.00	0.00
0.00	0.00
0.00	0.00

0.00	0.00
0.00	0.00
0.00	0.00
0.00	0.00
0.00	0.00
0.00	0.00
0.00	0.00
0.00	0.00
0.00	0.00
0.00	0.00
0.00	0.00
0.00	0.00
0.00	0.00
0.00	0.00
0.00	0.00
2.00	0.00
0.00	0.00
0.00	0.00
0.00	0.00
0.00	0.00
0.00	0.00
0.00	0.00
0.00	0.00
0.00	0.00
0.00	0.00
0.00	0.00
0.00	0.00
0.00	0.00
0.00	0.00
0.00	0.00
0.00	0.00
0.00	0.00
0.00	0.00
0.00	0.00
0.00	0.00
0.00	0.00
0.00	0.00

0.00	0.00
0.00	0.00
0.00	0.00
0.00	0.00
0.00	0.00
0.00	0.00
0.00	0.00
0.00	0.00
0.00	0.00
0.00	0.00
0.00	0.00
0.00	0.00
0.00	0.00
0.00	0.00
0.00	0.00
0.00	0.00
0.00	0.00
0.00	0.00
0.00	0.00
0.00	0.00
0.00	0.00
0.00	0.00
0.00	0.00
0.00	0.00
0.00	0.00
0.00	0.00
0.00	0.00
0.00	0.00
0.00	0.00
0.00	0.00
0.00	0.00
0.00	0.00
3.00	0.00
0.00	0.00
0.00	0.00
0.00	0.00
0.00	0.00

0.00	0.00
0.00	0.00
0.00	0.00
0.00	0.00
0.00	0.00
0.00	0.00
0.00	0.00
0.00	0.00
0.00	0.00
0.00	0.00
0.00	0.00
0.00	0.00
0.00	0.00
0.00	0.00
0.00	7.00
0.00	0.00
0.00	0.00
0.00	0.00
0.00	0.00
0.00	0.00
0.00	0.00
0.00	0.00
0.00	0.00
0.00	0.00
0.00	0.00
0.00	0.00
0.00	0.00
0.00	0.00
0.00	0.00
0.00	0.00
0.00	0.00
0.00	0.00
0.00	0.00
0.00	0.00
0.00	0.00
0.00	0.00
0.00	0.00

0.00	0.00
0.00	0.00
0.00	0.00
0.00	0.00
0.00	0.00
0.00	0.00
0.00	0.00
0.00	0.00
0.00	0.00
0.00	0.00
0.00	0.00
0.00	0.00
0.00	0.00
0.00	0.00
0.00	0.00
0.00	0.00
0.00	0.00
0.00	0.00
0.00	0.00
0.00	0.00
0.00	0.00
0.00	0.00
0.00	0.00
0.00	0.00
0.00	0.00
6.00	0.00
0.00	0.00
0.00	0.00
0.00	0.00
0.00	0.00
0.00	0.00
0.00	0.00
0.00	0.00
0.00	0.00
0.00	0.00
0.00	0.00
0.00	0.00

0.00	0.00
0.00	0.00
0.00	0.00
0.00	0.00
0.00	0.00
0.00	0.00
0.00	0.00
0.00	0.00
0.00	0.00
0.00	0.00
0.00	0.00
0.00	6.00
0.00	6.00
0.00	0.00
0.00	0.00
0.00	0.00
0.00	0.00
0.00	0.00
0.00	0.00
0.00	0.00
0.00	0.00
0.00	0.00
0.00	0.00
0.00	0.00
0.00	0.00
0.00	0.00
0.00	0.00
0.00	0.00
0.00	0.00
0.00	0.00
0.00	0.00
0.00	0.00
0.00	0.00
0.00	0.00
0.00	0.00
0.00	0.00
0.00	0.00

0.00	0.00
0.00	0.00
0.00	0.00
0.00	0.00
0.00	0.00
0.00	0.00
0.00	0.00
0.00	0.00
0.00	0.00
0.00	0.00
0.00	0.00
0.00	0.00
0.00	0.00
0.00	0.00
0.00	0.00
0.00	0.00
0.00	0.00
0.00	0.00
0.00	0.00
0.00	0.00
0.00	0.00
0.00	0.00
0.00	0.00
0.00	0.00
0.00	0.00
0.00	0.00
0.00	0.00
0.00	0.00
0.00	0.00
0.00	0.00
0.00	0.00
0.00	0.00
0.00	0.00
5.00	0.00
5.00	0.00
5.00	0.00
5.00	0.00

0.00	0.00
0.00	0.00
0.00	0.00
0.00	0.00
0.00	0.00
0.00	2.00
0.00	0.00
0.00	0.00
0.00	0.00
0.00	0.00
0.00	0.00
0.00	0.00
0.00	0.00
0.00	0.00
0.00	0.00
0.00	0.00
0.00	0.00
0.00	0.00
0.00	0.00
0.00	0.00
0.00	0.00
0.00	0.00
0.00	0.00
0.00	0.00
0.00	0.00
0.00	0.00
0.00	0.00
0.00	0.00
0.00	0.00
0.00	0.00
0.00	0.00
0.00	0.00
0.00	0.00
0.00	0.00
0.00	0.00
0.00	0.00
0.00	0.00

0.00	0.00
0.00	0.00
0.00	0.00
0.00	0.00
0.00	0.00
0.00	0.00
0.00	0.00
0.00	0.00
0.00	0.00
0.00	0.00
0.00	0.00
0.00	0.00
0.00	0.00
0.00	0.00
0.00	0.00
0.00	0.00
0.00	0.00
0.00	0.00
0.00	0.00
0.00	0.00
0.00	0.00
0.00	0.00
0.00	0.00
0.00	0.00
0.00	0.00
0.00	0.00
0.00	0.00
0.00	0.00
0.00	0.00
0.00	0.00
0.00	0.00
0.00	0.00
0.00	0.00
0.00	0.00
0.00	0.00
0.00	0.00
0.00	0.00

0.00	0.00
0.00	0.00
0.00	0.00
0.00	0.00
0.00	0.00
0.00	0.00
0.00	0.00
0.00	0.00
0.00	0.00
0.00	0.00
0.00	0.00
0.00	0.00
0.00	0.00
0.00	0.00
0.00	0.00
0.00	0.00
0.00	0.00
0.00	0.00
0.00	0.00
2.00	0.00
4.00	0.00
0.00	0.00
0.00	0.00
0.00	0.00
0.00	0.00
0.00	0.00
0.00	0.00
0.00	0.00
0.00	0.00
0.00	0.00
0.00	0.00
0.00	0.00
0.00	0.00
0.00	0.00
0.00	0.00
0.00	0.00
0.00	0.00

0.00	0.00
0.00	0.00
0.00	0.00
0.00	0.00
0.00	0.00
0.00	0.00
0.00	0.00
0.00	0.00
0.00	0.00
0.00	0.00
0.00	0.00
0.00	0.00
0.00	0.00
0.00	0.00
0.00	0.00
0.00	0.00
0.00	0.00
0.00	0.00
0.00	4.00
0.00	4.00
0.00	0.00
0.00	0.00
0.00	0.00
0.00	0.00
0.00	0.00
0.00	0.00
0.00	0.00
0.00	0.00
0.00	0.00
0.00	0.00
0.00	0.00
0.00	0.00
0.00	0.00
0.00	0.00
0.00	0.00
0.00	0.00
0.00	0.00

0.00	0.00
0.00	0.00
0.00	0.00
0.00	0.00
0.00	0.00
0.00	0.00
0.00	0.00
0.00	0.00
0.00	0.00
0.00	0.00
0.00	0.00
0.00	0.00
0.00	0.00
0.00	0.00
0.00	0.00
0.00	0.00
0.00	0.00
0.00	0.00
0.00	0.00
0.00	0.00
0.00	0.00
0.00	0.00
0.00	0.00
0.00	0.00
0.00	0.00
0.00	0.00
0.00	0.00
0.00	0.00
0.00	0.00
0.00	0.00
0.00	0.00
0.00	0.00
0.00	0.00
0.00	0.00
0.00	0.00
0.00	0.00
0.00	0.00

0.00	0.00
0.00	0.00
0.00	0.00
0.00	0.00
0.00	0.00
0.00	0.00
0.00	0.00
0.00	0.00
0.00	0.00
0.00	0.00
0.00	0.00
0.00	0.00
0.00	0.00
0.00	0.00
0.00	0.00
0.00	0.00
0.00	0.00
0.00	0.00
0.00	0.00
0.00	0.00
0.00	0.00
0.00	0.00
0.00	0.00
0.00	0.00
0.00	0.00
0.00	0.00
0.00	0.00
0.00	0.00
0.00	0.00
0.00	0.00
0.00	0.00
0.00	0.00
0.00	0.00
0.00	0.00
0.00	0.00
0.00	0.00
0.00	0.00

0.00	0.00
0.00	0.00
0.00	0.00
0.00	0.00
0.00	0.00
0.00	0.00
0.00	0.00
0.00	0.00
0.00	0.00
3.00	0.00
3.00	0.00
0.00	0.00
0.00	0.00
0.00	0.00
0.00	0.00
0.00	0.00
0.00	0.00
0.00	0.00
0.00	0.00
0.00	0.00
0.00	0.00
0.00	0.00
0.00	0.00
0.00	0.00
0.00	0.00
0.00	0.00
0.00	0.00
0.00	0.00
0.00	0.00
0.00	0.00
0.00	0.00
0.00	0.00
0.00	0.00
0.00	0.00
0.00	0.00
0.00	0.00
0.00	0.00

0.00	0.00
0.00	0.00
0.00	0.00
0.00	0.00
0.00	0.00
0.00	0.00
0.00	0.00
0.00	0.00
0.00	3.00
0.00	3.00
0.00	3.00
0.00	0.00
0.00	0.00
0.00	0.00
0.00	0.00
0.00	0.00
0.00	0.00
0.00	0.00
0.00	0.00
0.00	0.00
0.00	0.00
0.00	0.00
0.00	0.00
0.00	0.00
0.00	0.00
0.00	0.00
0.00	0.00
0.00	0.00
0.00	0.00
0.00	0.00
0.00	0.00
0.00	0.00
0.00	0.00
0.00	0.00
0.00	0.00
0.00	0.00
0.00	0.00

0.00	0.00
0.00	0.00
0.00	0.00
0.00	0.00
0.00	0.00
0.00	0.00
0.00	0.00
0.00	0.00
0.00	0.00
0.00	0.00
0.00	0.00
0.00	0.00
0.00	0.00
0.00	0.00
0.00	0.00
0.00	0.00
0.00	0.00
0.00	0.00
0.00	0.00
0.00	0.00
0.00	0.00
0.00	0.00
0.00	0.00
0.00	0.00
0.00	0.00
0.00	0.00
0.00	0.00
0.00	0.00
0.00	0.00
0.00	0.00
0.00	0.00
0.00	0.00
0.00	0.00
0.00	0.00
0.00	0.00
0.00	0.00
0.00	0.00

0.00	0.00
0.00	0.00
0.00	0.00
0.00	0.00
0.00	0.00
0.00	0.00
0.00	0.00
0.00	0.00
0.00	0.00
0.00	0.00
0.00	0.00
0.00	0.00
0.00	0.00
0.00	0.00
0.00	0.00
0.00	0.00
0.00	0.00
0.00	0.00
0.00	0.00
0.00	0.00
0.00	0.00
0.00	0.00
0.00	0.00
0.00	0.00
0.00	0.00
0.00	0.00
0.00	0.00
0.00	0.00
0.00	0.00
0.00	0.00
0.00	0.00
0.00	0.00
0.00	0.00
0.00	0.00
0.00	0.00
0.00	0.00
0.00	0.00

0.00	0.00
0.00	0.00
0.00	0.00
0.00	0.00
0.00	0.00
0.00	0.00
0.00	0.00
2.00	0.00
2.00	0.00
2.00	0.00
2.00	0.00
2.00	0.00
0.00	0.00
0.00	0.00
0.00	0.00
0.00	0.00
0.00	0.00
0.00	0.00
0.00	0.00
0.00	0.00
0.00	0.00
0.00	0.00
0.00	0.00
0.00	0.00
0.00	0.00
0.00	0.00
0.00	0.00
0.00	0.00
0.00	0.00
0.00	0.00
0.00	0.00
0.00	0.00
0.00	0.00
0.00	0.00
0.00	0.00
0.00	0.00
0.00	0.00

0.00	0.00
0.00	0.00
0.00	0.00
0.00	0.00
0.00	0.00
0.00	0.00
0.00	0.00
0.00	0.00
0.00	0.00
0.00	0.00
0.00	0.00
0.00	0.00
0.00	0.00
0.00	0.00
0.00	0.00
0.00	0.00
0.00	0.00
0.00	0.00
0.00	0.00
0.00	0.00
0.00	0.00
0.00	0.00
0.00	0.00
0.00	0.00
0.00	0.00
0.00	0.00
0.00	0.00
0.00	0.00
0.00	0.00
0.00	2.00
0.00	2.00
0.00	2.00
0.00	2.00
0.00	0.00
0.00	0.00
0.00	0.00
0.00	0.00

0.00	0.00
0.00	0.00
0.00	0.00
0.00	0.00
0.00	0.00
0.00	0.00
0.00	0.00
0.00	0.00
0.00	0.00
0.00	0.00
0.00	0.00
0.00	0.00
0.00	0.00
0.00	0.00
0.00	0.00
0.00	0.00
0.00	0.00
0.00	0.00
0.00	0.00
0.00	0.00
0.00	0.00
0.00	0.00
0.00	0.00
0.00	0.00
0.00	0.00
0.00	0.00
0.00	0.00
0.00	0.00
0.00	0.00
0.00	0.00
0.00	0.00
0.00	0.00
0.00	0.00

Taxonomy
d__Bacteria;p__Firmicutes;c__Bacilli;o__Lactobacillales;f__Streptococcaceae;g__Streptococcus;s__Streptococcus_pneumoniae
d__Bacteria;p__Proteobacteria;c__Gammaproteobacteria;o__Pseudomonadales;f__Moraxellaceae;g__Acinetobacter;s__Acinetobacter_
d__Bacteria;p__Proteobacteria;c__Gammaproteobacteria;o__Burkholderiales;f__Burkholderiaceae;g__Ralstonia;s__Ralstonia_uncla
d__Bacteria;p__Proteobacteria;c__Betaproteobacteria;o__Burkholderiales;f__Comamonadaceae;g__Comamonas;s__Comamonas_terrigen
d__Bacteria;p__Proteobacteria;c__Gammaproteobacteria;o__Xanthomonadales;f__Xanthomonadaceae;g__Stenotrophomonas;s__Stenotro
d__Bacteria;p__Firmicutes;c__Bacilli;o__Lactobacillales;f__Enterococcaceae;g__Enterococcus;s__Enterococcus_faecium
d__Bacteria;p__Firmicutes;c__Bacilli;o__Staphylococcales;f__Staphylococcaceae;g__Staphylococcus;s__Staphylococcus_unclassif
d__Bacteria;p__Proteobacteria;c__Gammaproteobacteria;o__Burkholderiales;f__Rhodocyclaceae;g__Dechlorosoma;s__Dechlorosoma_u
d__Bacteria;p__Firmicutes;c__Bacilli;o__Lactobacillales;f__Streptococcaceae;g__Streptococcus;s__Streptococcus_salivarius
d__Bacteria;p__Proteobacteria;c__Alphaproteobacteria;o__Sphingomonadales;f__Sphingomonadaceae;g__Sphingopyxis;s__uncultured
d__Bacteria;p__Firmicutes;c__Bacilli;o__Staphylococcales;f__Staphylococcaceae;g__Staphylococcus;s__Staphylococcus_unclassif
d__Bacteria;p__Firmicutes;c__Bacilli;o__Staphylococcales;f__Staphylococcaceae;g__Staphylococcus;s__Staphylococcus_unclassif
d__Bacteria;p__Actinobacteriota;c__Actinobacteria;o__Micrococcales;f__Micrococcaceae;g__Rothia;s__Rothia_unclassified
d__Bacteria;p__Proteobacteria;c__Gammaproteobacteria;o__Burkholderiales;f__Comamonadaceae;g__Delftia;s__Delftia_unclassifie
d__Bacteria;p__Proteobacteria;c__Alphaproteobacteria;o__Rhizobiales;f__Rhizobiaceae;g__Rhizobium;s__uncultured_Rhizobium_sp
d__Bacteria;p__Proteobacteria;c__Betaproteobacteria;o__Burkholderiales;f__Comamonadaceae;g__Comamonas;s__Comamonas_aquatica
d__Bacteria;p__Firmicutes;c__Bacilli;o__Staphylococcales;f__Staphylococcaceae;g__Staphylococcus;s__Staphylococcus_unclassif
d__Bacteria;p__Proteobacteria;c__Alphaproteobacteria;o__Rhizobiales;f__Beijerinckiaceae;g__Methylobacterium-Methylorubrum;s
d__Bacteria;p__Proteobacteria;c__Gammaproteobacteria;o__Enterobacterales;f__Enterobacteriaceae;g__Escherichia-Shigella;s__E
d__Bacteria;p__Firmicutes;c__Bacilli;o__Lactobacillales;f__Streptococcaceae;g__Streptococcus;s__Streptococcus_thermophilus
d__Bacteria;p__Proteobacteria;c__Gammaproteobacteria;o__Enterobacterales;f__Erwiniaceae;g__Pantoea;s__Pantoea_agglomerans
d__Bacteria;p__Firmicutes;c__Bacilli;o__Mycoplasmatales;f__Mycoplasmataceae;g__Ureaplasma;s__Ureaplasma_unclassified
d__Bacteria;p__Cyanobacteria;c__Cyanobacteriia;o__Chloroplast;f__Chloroplast_unclassified;g__Chloroplast_unclassified;s__Ch
d__Bacteria;p__Firmicutes;c__Clostridia;o__Thermincolales;f__Thermincolaceae;g__Thermincola;s__Thermincola_unclassified
d__Bacteria;p__Firmicutes;c__Bacilli;o__Mycoplasmatales;f__Mycoplasmataceae;g__Ureaplasma;s__Ureaplasma_unclassified
d__Bacteria;p__Proteobacteria;c__Gammaproteobacteria;o__Burkholderiales;f__Oxalobacteraceae;g__Herbaspirillum;s__Herbaspiri
d__Bacteria;p__Proteobacteria;c__Gammaproteobacteria;o__Enterobacterales;f__Enterobacteriaceae;g__Enterobacter;s__Enterobac
d__Bacteria;p__Proteobacteria;c__Gammaproteobacteria;o__Enterobacterales;f__Yersiniaceae;g__Serratia;s__uncultured_Serratia
d__Bacteria;p__Firmicutes;c__Bacilli;o__Lactobacillales;f__Enterococcaceae;g__Enterococcus;s__Enterococcus_faecium
d__Bacteria;p__Firmicutes;c__Bacilli;o__Staphylococcales;f__Staphylococcaceae;g__Staphylococcus;s__Staphylococcus_unclassif
d__Bacteria;p__Proteobacteria;c__Gammaproteobacteria;o__Burkholderiales;f__Oxalobacteraceae;g__Herbaspirillum;s__Herbaspiri
d__Bacteria;p__Firmicutes;c__Bacilli;o__Lactobacillales;f__Streptococcaceae;g__Streptococcus;s__Streptococcus_lactarius
d__Bacteria;p__Firmicutes;c__Bacilli;o__Staphylococcales;f__Staphylococcaceae;g__Staphylococcus;s__Staphylococcus_unclassif
d__Bacteria;p__Firmicutes;c__Bacilli;o__Lactobacillales;f__Streptococcaceae;g__Streptococcus;s__Streptococcus_unclassified
d__Bacteria;p__Firmicutes;c__Clostridia;o__Peptostreptococcales-Tissierellales;f__Peptostreptococcaceae;g__Peptostreptococc

d__Bacteria;p__Firmicutes;c__Bacilli;o__Lactobacillales;f__Enterococcaceae;g__Enterococcus;s__Enterococcus_faecalis
d__Bacteria;p__Proteobacteria;c__Betaproteobacteria;o__Burkholderiales;f__Comamonadaceae;g__Comamonas;s__Comamonas_sp._bk_1
d__Bacteria;p__Proteobacteria;c__Gammaproteobacteria;o__Pseudomonadales;f__Moraxellaceae;g__Acinetobacter;s__Acinetobacter_
d__Bacteria;p__Proteobacteria;c__Gammaproteobacteria;o__Enterobacterales;f__Enterobacteriaceae;g__Enterobacter;s__Enterobac
d__Bacteria;p__Firmicutes;c__Clostridia;o__Peptostreptococcales-Tissierellales;f__Peptostreptococcaceae;g__Clostridioides;s
d__Bacteria;p__Firmicutes;c__Bacilli;o__Lactobacillales;f__Carnobacteriaceae;g__Granulicatella;s__uncultured_Granulicatella
d__Bacteria;p__Proteobacteria;c__Gammaproteobacteria;o__Enterobacterales;f__Erwiniaceae;g__Pantoea;s__Pantoea_septica
d__Bacteria;p__Firmicutes;c__Bacilli;o__Paenibacillales;f__Paenibacillaceae;g__Paenibacillaceae_unclassified;s__Paenibacill
d__Bacteria;p__Proteobacteria;c__Gammaproteobacteria;o__Burkholderiales;f__Oxalobacteraceae;g__Herbaspirillum;s__Herbaspiri
d__Bacteria;p__Firmicutes;c__Bacilli;o__Lactobacillales;f__Streptococcaceae;g__Streptococcus;s__Streptococcus_iniae
d__Bacteria;p__Cyanobacteria;c__Cyanobacteriia;o__Chloroplast;f__Chloroplast_unclassified;g__Chloroplast_unclassified;s__Ch
d__Bacteria;p__Proteobacteria;c__Alphaproteobacteria;o__Caulobacterales;f__Caulobacteraceae;g__Brevundimonas;s__Brevundimon
d__Bacteria;p__Firmicutes;c__Bacilli;o__Staphylococcales;f__Staphylococcaceae;g__Staphylococcus;s__Staphylococcus_unclassif
d__Bacteria;p__Firmicutes;c__Bacilli;o__Bacillales;f__Bacillaceae;g__Aeribacillus;s__Aeribacillus_unclassified
d__Bacteria;p__Firmicutes;c__Bacilli;o__Staphylococcales;f__Staphylococcaceae;g__Staphylococcus;s__Staphylococcus_unclassif
d__Bacteria;p__Proteobacteria;c__Gammaproteobacteria;o__Burkholderiales;f__Hydrogenophilaceae;g__Hydrogenophilus;s__Hydroge
d__Bacteria;p__Firmicutes;c__Clostridia;o__Clostridiales;f__Caloramatoraceae;g__Fonticella;s__Fonticella_unclassified
d__Bacteria;p__Firmicutes;c__Bacilli;o__Staphylococcales;f__Staphylococcaceae;g__Staphylococcus;s__Staphylococcus_unclassif
d__Bacteria;p__Firmicutes;c__Bacilli;o__Erysipelotrichales;f__Erysipelotrichaceae;g__Erysipelothrix;s__Erysipelothrix_uncla
d__Bacteria;p__Proteobacteria;c__Alphaproteobacteria;o__Caulobacterales;f__Caulobacteraceae;g__Brevundimonas;s__Brevundimon
d__Bacteria;p__Proteobacteria;c__Gammaproteobacteria;o__Burkholderiales;f__Burkholderiaceae;g__Burkholderia-Caballeronia-Pa
d__Bacteria;p__Firmicutes;c__Bacilli;o__Lactobacillales;f__Lactobacillaceae;g__Ligilactobacillus;s__Ligilactobacillus_uncla
d__Bacteria;p__Firmicutes;c__Bacilli;o__Staphylococcales;f__Staphylococcaceae;g__Staphylococcus;s__Staphylococcus_unclassif
d__Bacteria;p__Firmicutes;c__Clostridia;o__Peptostreptococcales-Tissierellales;f__Family_XI;g__Anaerococcus;s__Anaerococcus
d__Bacteria;p__Firmicutes;c__Clostridia;o__Thermincolales;f__Thermincolaceae;g__Thermincola;s__Thermincola_unclassified
d__Bacteria;p__Bacteroidota;c__Bacteroidia;o__Bacteroidales;f__Prevotellaceae;g__Prevotella;s__uncultured_Prevotella_sp.
d__Bacteria;p__Proteobacteria;c__Gammaproteobacteria;o__Burkholderiales;f__Comamonadaceae;g__Schlegelella;s__Schlegelella_u
d__Bacteria;p__Bacteroidota;c__Bacteroidia;o__Bacteroidales;f__Prevotellaceae;g__Prevotella;s__uncultured_Prevotella_sp.
d__Bacteria;p__Proteobacteria;c__Gammaproteobacteria;o__Enterobacterales;f__Yersiniaceae;g__Serratia;s__Serratia_marcescens
d__Bacteria;p__Firmicutes;c__Clostridia;o__Clostridiales;f__Caloramatoraceae;g__Fonticella;s__Fonticella_unclassified
d__Bacteria;p__Proteobacteria;c__Gammaproteobacteria;o__Pseudomonadales;f__Moraxellaceae;g__Acinetobacter;s__Acinetobacter_
d__Bacteria;p__Proteobacteria;c__Alphaproteobacteria;o__Rickettsiales;f__Mitochondria;g__Mitochondria_unclassified;s__Mitoc
d__Bacteria;p__Proteobacteria;c__Alphaproteobacteria;o__Rhizobiales;f__Bradyrhizobiaceae;g__Bradyrhizobium;s__Bradyrhizobiu
d__Bacteria;p__Proteobacteria;c__Gammaproteobacteria;o__Enterobacterales;f__Yersiniaceae;g__Serratia;s__Serratia_sp._MG-201
d__Bacteria;p__Firmicutes;c__Bacilli;o__Lactobacillales;f__Streptococcaceae;g__Streptococcus;s__Streptococcus_salivarius
d__Bacteria;p__Bacteroidota;c__Bacteroidia;o__Bacteroidales;f__Bacteroidaceae;g__Bacteroides;s__Bacteroides_uniformis
d__Bacteria;p__Proteobacteria;c__Gammaproteobacteria;o__Burkholderiales;f__Burkholderiaceae;g__Burkholderia-Caballeronia-Pa

d__Bacteria;p__Bacteroidota;c__Bacteroidia;o__Bacteroidales;f__Bacteroidaceae;g__Bacteroides;s__Bacteroides_unclassified
d__Bacteria;p__Proteobacteria;c__Gammaproteobacteria;o__Xanthomonadales;f__Xanthomonadaceae;g__Xanthomonas;s__Xanthomonas_p
d__Bacteria;p__Bacteroidota;c__Bacteroidia;o__Bacteroidales;f__Prevotellaceae;g__Prevotella;s__Prevotella_bivia
d__Bacteria;p__Actinobacteriota;c__Actinobacteria;o__Bifidobacteriales;f__Bifidobacteriaceae;g__Bifidobacterium;s__Bifidoba
d__Bacteria;p__Firmicutes;c__Bacilli;o__Lactobacillales;f__Streptococcaceae;g__Lactococcus;s__Lactococcus_lactis
d__Bacteria;p__Bacteroidota;c__Bacteroidia;o__Bacteroidales;f__Rikenellaceae;g__Rikenellaceae_RC9_gut_group;s__Rikenellacea
d__Bacteria;p__Firmicutes;c__Bacilli;o__Lactobacillales;f__Enterococcaceae;g__Enterococcus;s__Enterococcus_faecalis
d__Bacteria;p__Firmicutes;c__Clostridia;o__Oscillospirales;f__UCG-010;g__UCG-010_unclassified;s__UCG-010_unclassified
d__Bacteria;p__Verrucomicrobiota;c__Verrucomicrobiae;o__Verrucomicrobiales;f__Akkermansiaceae;g__Akkermansia;s__Akkermansia
d__Bacteria;p__Bacteroidota;c__Bacteroidia;o__Bacteroidales;f__Bacteroidaceae;g__Bacteroides;s__Bacteroides_unclassified
d__Bacteria;p__Fusobacteriota;c__Fusobacteriia;o__Fusobacteriales;f__Fusobacteriaceae;g__Fusobacterium;s__Fusobacterium_unc
d__Bacteria;p__Proteobacteria;c__Gammaproteobacteria;o__Pseudomonadales;f__Pseudomonadaceae;g__Pseudomonas;s__Pseudomonas_s
d__Bacteria;p__Firmicutes;c__Bacilli;o__Bacillales;f__Bacillaceae;g__Aeribacillus;s__Aeribacillus_pallidus
d__Bacteria;p__Actinobacteriota;c__Actinobacteria;o__Propionibacteriales;f__Propionibacteriaceae;g__Cutibacterium;s__Cutiba
d__Bacteria;p__Actinobacteriota;c__Actinobacteria;o__Bifidobacteriales;f__Bifidobacteriaceae;g__Bifidobacterium;s__Bifidoba
d__Bacteria;p__Firmicutes;c__Bacilli;o__Lactobacillales;f__Streptococcaceae;g__Streptococcus;s__uncultured_Streptococcus_sp
d__Bacteria;p__Firmicutes;c__Clostridia;o__Clostridiales;f__Gracilibacteraceae;g__Gracilibacteraceae_unclassified;s__Gracil
d__Bacteria;p__Proteobacteria;c__Gammaproteobacteria;o__Burkholderiales;f__Oxalobacteraceae;g__Janthinobacterium;s__Janthin
d__Bacteria;p__Firmicutes;c__Bacilli;o__Paenibacillales;f__Paenibacillaceae;g__Paenibacillaceae_unclassified;s__Paenibacill
d__Bacteria;p__Firmicutes;c__Negativicutes;o__Acidaminococcales;f__Acidaminococcaceae;g__Phascolarctobacterium;s__Phascolar
d__Bacteria;p__Actinobacteriota;c__Actinobacteria;o__Bifidobacteriales;f__Bifidobacteriaceae;g__Bifidobacterium;s__Bifidoba
d__Bacteria;p__Firmicutes;c__Bacilli;o__Lactobacillales;f__Lactobacillaceae;g__Lactobacillus;s__Lactobacillus_crispatus
d__Bacteria;p__Proteobacteria;c__Betaproteobacteria;o__Burkholderiales;f__Comamonadaceae;g__Comamonas;s__Comamonas_sp._774B
d__Bacteria;p__Firmicutes;c__Clostridia;o__Peptostreptococcales-Tissierellales;f__Family_XI;g__Anaerococcus;s__Anaerococcus
d__Bacteria;p__Proteobacteria;c__Gammaproteobacteria;o__Pseudomonadales;f__Moraxellaceae;g__Acinetobacter;s__Acinetobacter_
d__Bacteria;p__Firmicutes;c__Bacilli;o__Staphylococcales;f__Staphylococcaceae;g__Staphylococcus;s__Staphylococcus_unclassif
d__Bacteria;p__Proteobacteria;c__Gammaproteobacteria;o__Burkholderiales;f__Burkholderiaceae;g__Burkholderia-Caballeronia-Pa
d__Bacteria;p__Proteobacteria;c__Gammaproteobacteria;o__Burkholderiales;f__Comamonadaceae;g__Comamonas;s__Comamonas_unclass
d__Bacteria;p__Actinobacteriota;c__Actinobacteria;o__Bifidobacteriales;f__Bifidobacteriaceae;g__Bifidobacterium;s__Bifidoba
d__Bacteria;p__Firmicutes;c__Bacilli;o__Bacillales;f__Bacillaceae;g__Aeribacillus;s__Aeribacillus_pallidus
d__Bacteria;p__Proteobacteria;c__Gammaproteobacteria;o__Burkholderiales;f__Comamonadaceae;g__Acidovorax;s__Acidovorax_uncla
d__Bacteria;p__Actinobacteriota;c__Actinobacteriota;o__Corynebacteriales;f__Corynebacteriaceae;g__Corynebacterium;s__Coryne
d__Bacteria;p__Firmicutes;c__Bacilli;o__Brevibacillales;f__Brevibacillaceae;g__Brevibacillus;s__Brevibacillus_unclassified
d__Bacteria;p__Firmicutes;c__Clostridia;o__Oscillospirales;f__Ruminococcaceae;g__Faecalibacterium;s__Faecalibacterium_uncla
d__Bacteria;p__Proteobacteria;c__Gammaproteobacteria;o__Pseudomonadales;f__Pseudomonadaceae;g__Pseudomonas;s__Pseudomonas_s
d__Bacteria;p__Proteobacteria;c__Alphaproteobacteria;o__Alphaproteobacteria_unclassified;f__Alphaproteobacteria_unclassifie
d__Bacteria;p__Bacteroidota;c__Bacteroidia;o__Bacteroidales;f__Bacteroidaceae;g__Bacteroides;s__Bacteroides_unclassified

d__Bacteria;p__Firmicutes;c__Bacilli;o__Lactobacillales;f__Lactobacillaceae;g__Lactobacillus;s__Lactobacillus_iners
d__Bacteria;p__Proteobacteria;c__Gammaproteobacteria;o__Xanthomonadales;f__Xanthomonadaceae;g__Pseudoxanthomonas;s__Pseudox
d__Bacteria;p__Actinobacteriota;c__Actinobacteria;o__Bifidobacteriales;f__Bifidobacteriaceae;g__Bifidobacterium;s__Bifidoba
d__Bacteria;p__Firmicutes;c__Clostridia;o__Thermincolales;f__Thermincolaceae;g__Thermincola;s__Thermincola_unclassified
d__Bacteria;p__Firmicutes;c__Clostridia;o__Thermincolales;f__Thermincolaceae;g__Thermincola;s__Thermincola_unclassified
d__Bacteria;p__Proteobacteria;c__Alphaproteobacteria;o__Rhizobiales;f__Xanthobacteraceae;g__Bradyrhizobium;s__Bradyrhizobiu
d__Bacteria;p__Actinobacteriota;c__Actinobacteria;o__Corynebacteriales;f__Mycobacteriaceae;g__Mycobacterium;s__Mycobacteriu
d__Bacteria;p__Firmicutes;c__Clostridia;o__Thermincolales;f__Thermincolaceae;g__Thermincola;s__Thermincola_unclassified
d__Bacteria;p__Firmicutes;c__Bacilli;o__Bacillales;f__Bacillaceae;g__Geobacillus;s__Geobacillus_sp._enrichment_culture_clon
d__Bacteria;p__Firmicutes;c__Bacilli;o__Lactobacillales;f__Streptococcaceae;g__Streptococcus;s__uncultured_Streptococcus_sp
d__Bacteria;p__Actinobacteriota;c__Actinobacteriota;o__Micrococcales;f__Microbacteriaceae;g__Leucobacter;s__Leucobacter_chr
d__Bacteria;p__Firmicutes;c__Bacilli;o__Lactobacillales;f__Streptococcaceae;g__Streptococcus;s__Streptococcus_parasanguinis
d__Bacteria;p__Bacteroidota;c__Bacteroidia;o__Bacteroidales;f__Bacteroidaceae;g__Bacteroides;s__Bacteroides_unclassified
d__Bacteria;p__Firmicutes;c__Clostridia;o__Clostridiales;f__Clostridiales_unclassified;g__Clostridiales_unclassified;s__Clo
d__Bacteria;p__Proteobacteria;c__Alphaproteobacteria;o__Rhizobiales;f__Rhizobiaceae;g__Phyllobacterium;s__Phyllobacterium_u
d__Bacteria;p__Proteobacteria;c__Alphaproteobacteria;o__Rhizobiales;f__Beijerinckiaceae;g__Methylobacterium-Methylorubrum;s
d__Bacteria;p__Proteobacteria;c__Gammaproteobacteria;o__Xanthomonadales;f__Xanthomonadaceae;g__Lysobacter;s__Lysobacter_sp.
d__Bacteria;p__Firmicutes;c__Bacilli;o__Lactobacillales;f__Streptococcaceae;g__Streptococcus;s__Streptococcus_unclassified
d__Bacteria;p__Firmicutes;c__Bacilli;o__Lactobacillales;f__Streptococcaceae;g__Streptococcus;s__Streptococcus_salivarius
d__Bacteria;p__Proteobacteria;c__Alphaproteobacteria;o__Rhodobacterales;f__Rhodobacteraceae;g__Rubellimicrobium;s__unculturd
d__Bacteria;p__Proteobacteria;c__Alphaproteobacteria;o__Sphingomonadales;f__Sphingomonadaceae;g__Sphingomonadaceae_unclassi
d__Bacteria;p__Firmicutes;c__Clostridia;o__Clostridiales;f__Clostridiaceae;g__Clostridium_sensu_stricto_1;s__Clostridium_se
d__Bacteria;p__Firmicutes;c__Bacilli;o__Lactobacillales;f__Lactobacillaceae;g__Lactobacillus;s__Lactobacillus_crispatus
d__Bacteria;p__Proteobacteria;c__Gammaproteobacteria;o__Pseudomonadales;f__Pseudomonadaceae;g__Pseudomonas;s__uncultured_Ps
d__Bacteria;p__Firmicutes;c__Bacilli;o__Staphylococcales;f__Staphylococcaceae;g__Staphylococcus;s__Staphylococcus_unclassif
d__Bacteria;p__Actinobacteriota;c__Actinobacteria;o__Actinomycetales;f__Actinomycetaceae;g__Actinomyces;s__Actinomyces_uncl
d__Bacteria;p__Proteobacteria;c__Gammaproteobacteria;o__Burkholderiales;f__Burkholderiaceae;g__Burkholderia-Caballeronia-Pa
d__Bacteria;p__Proteobacteria;c__Gammaproteobacteria;o__Enterobacterales;f__Enterobacteriaceae;g__Klebsiella;s__Klebsiella_
d__Bacteria;p__Proteobacteria;c__Betaproteobacteria;o__Burkholderiales;f__Comamonadaceae;g__Comamonas;s__uncultured_Comamon
d__Bacteria;p__Proteobacteria;c__Gammaproteobacteria;o__Enterobacterales;f__Enterobacteriaceae;g__Enterobacter;s__Enterobac
d__Bacteria;p__Proteobacteria;c__Gammaproteobacteria;o__Xanthomonadales;f__Xanthomonadaceae;g__Stenotrophomonas;s__Stenotro
d__Bacteria;p__Actinobacteriota;c__Actinobacteria;o__Propionibacteriales;f__Nocardioidaceae;g__Nocardioides;s__Nocardioides
d__Bacteria;p__Firmicutes;c__Clostridia;o__Lachnospirales;f__Lachnospiraceae;g__Blautia;s__Blautia_unclassified
d__Bacteria;p__Proteobacteria;c__Gammaproteobacteria;o__Enterobacterales;f__Yersiniaceae;g__Serratia;s__uncultured_Serratia
d__Bacteria;p__Firmicutes;c__Clostridia;o__Peptostreptococcales-Tissierellales;f__Family_XI;g__Finegoldia;s__Finegoldia_unc
d__Bacteria;p__Deinococcota;c__Deinococci;o__Thermales;f__Thermaceae;g__Thermus;s__Thermus_unclassified
d__Bacteria;p__Proteobacteria;c__Alphaproteobacteria;o__Rhizobiales;f__Beijerinckiaceae;g__Methylobacterium-Methylorubrum;s

d__Bacteria;p__Firmicutes;c__Bacilli;o__Lactobacillales;f__Streptococcaceae;g__Streptococcus;s__Streptococcus_anginosus
d__Bacteria;p__Cyanobacteria;c__Cyanobacteriia;o__Synechococcales;f__Cyanobiaceae;g__Synechococcus_CC9902;s__Synechococcus_
d__Bacteria;p__Bacteroidota;c__Bacteroidia;o__Flavobacteriales;f__Flavobacteriaceae;g__Flavobacterium;s__Flavobacterium_unc
d__Bacteria;p__Firmicutes;c__Clostridia;o__Oscillospirales;f__Ruminococcaceae;g__Faecalibacterium;s__Faecalibacterium_uncla
d__Bacteria;p__Firmicutes;c__Bacilli;o__Bacilli_unclassified;f__Bacilli_unclassified;g__Bacilli_unclassified;s__Bacilli_unc
d__Bacteria;p__Actinobacteriota;c__Actinobacteria;o__Micrococcales;f__Brevibacteriaceae;g__Brevibacterium;s__Brevibacterium
d__Bacteria;p__Firmicutes;c__Clostridia;o__Lachnospirales;f__Lachnospiraceae;g__Dorea;s__Dorea_unclassified
d__Bacteria;p__Firmicutes;c__Clostridia;o__Christensenellales;f__Christensenellaceae;g__Christensenellaceae_R-7_group;s__Ch
d__Bacteria;p__Proteobacteria;c__Alphaproteobacteria;o__Sphingomonadales;f__Sphingomonadaceae;g__Sphingomonas;s__uncultured
d__Bacteria;p__Proteobacteria;c__Gammaproteobacteria;o__Enterobacterales;f__Pasteurellaceae;g__Haemophilus;s__Haemophilus_u
d__Bacteria;p__Firmicutes;c__Bacilli;o__Bacillales;f__Bacillaceae;g__Oceanobacillus;s__Oceanobacillus_unclassified
d__Bacteria;p__Actinobacteriota;c__Actinobacteria;o__Micrococcales;f__Micrococcaceae;g__Rothia;s__Rothia_unclassified
d__Bacteria;p__Proteobacteria;c__Gammaproteobacteria;o__Burkholderiales;f__Comamonadaceae;g__Schlegelella;s__Schlegelella_u
d__Bacteria;p__Firmicutes;c__Bacilli;o__Lactobacillales;f__Streptococcaceae;g__Lactococcus;s__Lactococcus_sp._UA-V10144
d__Bacteria;p__Proteobacteria;c__Alphaproteobacteria;o__Rhizobiales;f__Rhizobiaceae;g__Allorhizobium-Neorhizobium-Pararhizo
d__Bacteria;p__Proteobacteria;c__Alphaproteobacteria;o__Rhodobacterales;f__Rhodobacteraceae;g__Amylibacter;s__Amylibacter_u
d__Bacteria;p__Proteobacteria;c__Gammaproteobacteria;o__Enterobacterales;f__Enterobacteriaceae;g__Raoultella;s__Raoultella_
d__Bacteria;p__Firmicutes;c__Bacilli;o__Bacillales;f__Bacillaceae;g__Virgibacillus;s__uncultured_Virgibacillus_sp.
d__Bacteria;p__Firmicutes;c__Bacilli;o__Staphylococcales;f__Staphylococcaceae;g__Staphylococcus;s__Staphylococcus_unclassif
d__Bacteria;p__Proteobacteria;c__Gammaproteobacteria;o__Burkholderiales;f__Comamonadaceae;g__Variovorax;s__Variovorax_uncla
d__Bacteria;p__Proteobacteria;c__Gammaproteobacteria;o__Xanthomonadales;f__Rhodanobacteraceae;g__Rhodanobacter;s__Rhodanoba
d__Bacteria;p__Proteobacteria;c__Alphaproteobacteria;o__Caulobacterales;f__Caulobacteraceae;g__Brevundimonas;s__Brevundimon
d__Bacteria;p__Firmicutes;c__Clostridia;o__Peptostreptococcales-Tissierellales;f__Family_XI;g__Anaerococcus;s__Anaerococcus
d__Bacteria;p__Proteobacteria;c__Gammaproteobacteria;o__Pseudomonadales;f__Pseudomonadaceae;g__Pseudomonas;s__Pseudomonas_l
d__Bacteria;p__Firmicutes;c__Thermoanaerobacteria;o__Caldicellulosiruptorales;f__Caldicellulosiraptoraceae;g__Caldicellulos
d__Bacteria;p__Firmicutes;c__Bacilli;o__Staphylococcales;f__Staphylococcaceae;g__Staphylococcus;s__Staphylococcus_unclassif
d__Bacteria;p__Actinobacteriota;c__Actinobacteriota;o__Actinobacteriota_unclassified;f__Actinobacteriota_unclassified;g__Ac
d__Bacteria;p__Firmicutes;c__Bacilli;o__Lactobacillales;f__Enterococcaceae;g__Enterococcus;s__Enterococcus_gallinarum
d__Bacteria;p__Actinobacteriota;c__Actinobacteria;o__Streptosporangiales;f__Nocardiopsaceae;g__Nocardiopsis;s__Nocardiopsis
d__Bacteria;p__Proteobacteria;c__Gammaproteobacteria;o__Xanthomonadales;f__Xanthomonadaceae;g__Lysobacter;s__Lysobacter_sp.
d__Bacteria;p__Proteobacteria;c__Gammaproteobacteria;o__Burkholderiales;f__Neisseriaceae;g__Neisseria;s__Neisseria_unclassi
d__Bacteria;p__Proteobacteria;c__Gammaproteobacteria;o__Burkholderiales;f__Comamonadaceae;g__Aquabacterium;s__Aquabacterium
d__Bacteria;p__Bacteroidota;c__Bacteroidia;o__Bacteroidales;f__Porphyromonadaceae;g__Porphyromonas;s__Porphyromonas_bennoni
d__Bacteria;p__Verrucomicrobiota;c__Verrucomicrobiae;o__Pedosphaerales;f__Pedosphaeraceae;g__ADurb.Bin063-1;s__ADurb.Bin063
d__Bacteria;p__Myxococcota;c__Polyangia;o__Haliangiales;f__Haliangiaceae;g__Haliangium;s__Haliangium_unclassified
d__unclassified;p__unclassified;c__unclassified;o__unclassified;f__unclassified;g__unclassified;s__unclassified
d__Bacteria;p__Firmicutes;c__Bacilli;o__Paenibacillales;f__Paenibacillaceae;g__Paenibacillaceae_unclassified;s__Paenibacill

d__Bacteria;p__Proteobacteria;c__Gammaproteobacteria;o__Pseudomonadales;f__Moraxellaceae;g__Acinetobacter;s__Acinetobacter_
d__Bacteria;p__Firmicutes;c__Clostridia;o__Clostridiales;f__Caloramatoraceae;g__Fonticella;s__Fonticella_unclassified
d__Bacteria;p__Proteobacteria;c__Gammaproteobacteria;o__Burkholderiales;f__Oxalobacteraceae;g__Janthinobacterium;s__Janthin
d__Bacteria;p__Proteobacteria;c__Gammaproteobacteria;o__Burkholderiales;f__Burkholderiaceae;g__Burkholderia-Caballeronia-Pa
d__Bacteria;p__Proteobacteria;c__Gammaproteobacteria;o__Burkholderiales;f__Alcaligenaceae;g__Alcaligenes;s__Alcaligenes_unc
d__Bacteria;p__Proteobacteria;c__Gammaproteobacteria;o__Pseudomonadales;f__Pseudomonadaceae;g__Pseudomonas;s__uncultured_Ps
d__Bacteria;p__Firmicutes;c__Bacilli;o__Lactobacillales;f__Lactobacillaceae;g__HT002;s__HT002_unclassified
d__Bacteria;p__Firmicutes;c__Bacilli;o__Bacillales;f__Bacillaceae;g__Virgibacillus;s__uncultured_Virgibacillus_sp.
d__Bacteria;p__Bacteroidota;c__Bacteroidia;o__Cytophagales;f__Hymenobacteraceae;g__Hymenobacter;s__Hymenobacter_unclassifie
d__Bacteria;p__Firmicutes;c__Bacilli;o__Thermoactinomycetales;f__Thermoactinomycetaceae;g__Kroppenstedtia;s__Kroppenstedtia
d__Bacteria;p__Firmicutes;c__Bacilli;o__Erysipelotrichales;f__Erysipelotrichaceae;g__Erysipelothrix;s__Erysipelothrix_uncla
d__Bacteria;p__Firmicutes;c__Bacilli;o__Paenibacillales;f__Paenibacillaceae;g__Paenibacillus;s__Paenibacillus_unclassified
d__Bacteria;p__Firmicutes;c__Clostridia;o__Lachnospirales;f__Lachnospiraceae;g__Fusicatenibacter;s__Fusicatenibacter_unclas
d__Bacteria;p__Firmicutes;c__Bacilli;o__Bacillales;f__Bacillaceae;g__Geobacillus;s__Geobacillus_stearothermophilus
d__Bacteria;p__Cyanobacteria;c__Cyanobacteriia;o__Chloroplast;f__Chloroplast_unclassified;g__Chloroplast_unclassified;s__Ch
d__Bacteria;p__Firmicutes;c__Bacilli;o__Lactobacillales;f__Streptococcaceae;g__Streptococcus;s__Streptococcus_thermophilus
d__Bacteria;p__Proteobacteria;c__Gammaproteobacteria;o__Burkholderiales;f__Burkholderiaceae;g__Burkholderia-Caballeronia-Pa
d__Bacteria;p__Proteobacteria;c__Gammaproteobacteria;o__Pseudomonadales;f__Moraxellaceae;g__Alkanindiges;s__Alkanindiges_un
d__Bacteria;p__Firmicutes;c__Bacilli;o__Bacillales;f__Bacillaceae;g__Bacillus;s__uncultured_Bacillus_sp.
d__Bacteria;p__Verrucomicrobiota;c__Verrucomicrobiae;o__Verrucomicrobiales;f__Akkermansiaceae;g__Akkermansia;s__Akkermansia
d__Bacteria;p__Proteobacteria;c__Gammaproteobacteria;o__Pseudomonadales;f__Pseudomonadaceae;g__Pseudomonas;s__Pseudomonas_s
d__Bacteria;p__Proteobacteria;c__Gammaproteobacteria;o__Xanthomonadales;f__Xanthomonadaceae;g__Xanthomonas;s__Xanthomonas_p
d__Bacteria;p__Firmicutes;c__Clostridia;o__Christensenellales;f__Christensenellaceae;g__Christensenellaceae_R-7_group;s__Ch
d__Bacteria;p__Firmicutes;c__Bacilli;o__Bacillales;f__Planococcaceae;g__Lysinibacillus;s__Lysinibacillus_unclassified
d__Bacteria;p__Spirochaetota;c__Spirochaetia;o__Spirochaetales;f__Spirochaetaceae;g__Termite_Treponema_cluster;s__Termite_T
d__Bacteria;p__Proteobacteria;c__Alphaproteobacteria;o__Rhizobiales;f__Beijerinckiaceae;g__Bosea;s__Bosea_unclassified
d__Bacteria;p__Proteobacteria;c__Gammaproteobacteria;o__Gammaproteobacteria_Incertae_Sedis;f__Unknown_Family;g__Acidibacter
d__Bacteria;p__Firmicutes;c__Bacilli;o__Bacillales;f__Bacillaceae;g__Anoxybacillus;s__Anoxybacillus_flavithermus
d__Bacteria;p__Proteobacteria;c__Gammaproteobacteria;o__Enterobacterales;f__Yersiniaceae;g__Serratia;s__Serratia_unclassifi
d__Bacteria;p__Proteobacteria;c__Gammaproteobacteria;o__Pseudomonadales;f__Moraxellaceae;g__Acinetobacter;s__Acinetobacter_
d__Bacteria;p__Actinobacteriota;c__Actinobacteria;o__Corynebacteriales;f__Nocardiaceae;g__Rhodococcus;s__Rhodococcus_unclas
d__Bacteria;p__Firmicutes;c__Clostridia;o__Lachnospirales;f__Lachnospiraceae;g__Blautia;s__Blautia_unclassified
d__Bacteria;p__Proteobacteria;c__Gammaproteobacteria;o__Diplorickettsiales;f__Diplorickettsiaceae;g__Diplorickettsiaceae_un
d__Bacteria;p__Firmicutes;c__Clostridia;o__Oscillospirales;f__Ruminococcaceae;g__Faecalibacterium;s__Faecalibacterium_uncla
d__Bacteria;p__Firmicutes;c__Clostridia;o__Peptostreptococcales-Tissierellales;f__Family_XI;g__Ezakiella;s__Ezakiella_uncla
d__Bacteria;p__Firmicutes;c__Bacilli;o__Lactobacillales;f__Lactobacillaceae;g__Limosilactobacillus;s__Limosilactobacillus_u
d__Bacteria;p__Firmicutes;c__Bacilli;o__Lactobacillales;f__Lactobacillaceae;g__Weissella;s__Weissella_unclassified

d__Bacteria;p__Firmicutes;c__Bacilli;o__Bacillales;f__Bacillaceae;g__Bacillus;s__Bacillus_thermoamylovorans
d__Bacteria;p__Cyanobacteria;c__Cyanobacteriia;o__Chloroplast;f__Chloroplast_unclassified;g__Chloroplast_unclassified;s__Ch
d__Bacteria;p__Proteobacteria;c__Alphaproteobacteria;o__Rhizobiales;f__Rhizobiaceae;g__Aureimonas;s__Aureimonas_unclassifie
d__Bacteria;p__Firmicutes;c__Bacilli;o__Lactobacillales;f__Lactobacillaceae;g__Ligilactobacillus;s__Ligilactobacillus_uncla
d__Bacteria;p__Proteobacteria;c__Betaproteobacteria;o__Burkholderiales;f__Comamonadaceae;g__Comamonas;s__uncultured_Comamon
d__Bacteria;p__Actinobacteriota;c__Actinobacteriota;o__Micrococcales;f__Micrococcaceae;g__Arthrobacter;s__Arthrobacter_agil
d__Bacteria;p__Firmicutes;c__Bacilli;o__Erysipelotrichales;f__Erysipelotrichaceae;g__Erysipelothrix;s__Erysipelothrix_uncla
d__Bacteria;p__Actinobacteriota;c__Actinobacteria;o__Micrococcales;f__Micrococcaceae;g__Micrococcus;s__Micrococcus_unclassi
d__Bacteria;p__Proteobacteria;c__Alphaproteobacteria;o__Sphingomonadales;f__Sphingomonadaceae;g__Sphingomonas;s__Sphingomon
d__Bacteria;p__Firmicutes;c__Bacilli;o__Staphylococcales;f__Staphylococcaceae;g__Staphylococcus;s__Staphylococcus_unclassif
d__Bacteria;p__Firmicutes;c__Clostridia;o__Lachnospirales;f__Lachnospiraceae;g__Agathobacter;s__Agathobacter_unclassified
d__Bacteria;p__Firmicutes;c__Bacilli;o__Brevibacillales;f__Brevibacillaceae;g__Brevibacillus;s__Brevibacillus_unclassified
d__Bacteria;p__Proteobacteria;c__Gammaproteobacteria;o__Pseudomonadales;f__Moraxellaceae;g__Acinetobacter;s__uncultured_Aci
d__Bacteria;p__Proteobacteria;c__Gammaproteobacteria;o__Burkholderiales;f__Comamonadaceae;g__Diaphorobacter;s__Diaphorobact
d__Bacteria;p__Proteobacteria;c__Gammaproteobacteria;o__Pseudomonadales;f__Moraxellaceae;g__Enhydrobacter;s__Enhydrobacter_
d__Bacteria;p__Actinobacteriota;c__Actinobacteria;o__Frankiales;f__Geodermatophilaceae;g__Blastococcus;s__Blastococcus_uncl
d__Bacteria;p__Deinococcota;c__Deinococci;o__Deinococcales;f__Deinococcaceae;g__Deinococcus;s__Deinococcus_unclassified
d__Bacteria;p__Proteobacteria;c__Gammaproteobacteria;o__Pseudomonadales;f__Moraxellaceae;g__Acinetobacter;s__Acinetobacter_
d__Bacteria;p__Firmicutes;c__Bacilli;o__Bacillales;f__Bacillaceae;g__Anoxybacillus;s__Anoxybacillus_gonensis
d__Bacteria;p__Firmicutes;c__Bacilli;o__Staphylococcales;f__Staphylococcaceae;g__Staphylococcus;s__Staphylococcus_unclassif
d__Bacteria;p__Firmicutes;c__Clostridia;o__Oscillospirales;f__Ruminococcaceae;g__Faecalibacterium;s__Faecalibacterium_uncla
d__Bacteria;p__Proteobacteria;c__Gammaproteobacteria;o__Burkholderiales;f__Burkholderiaceae;g__Burkholderia-Caballeronia-Pa
d__Bacteria;p__Proteobacteria;c__Gammaproteobacteria;o__Burkholderiales;f__Burkholderiaceae;g__Burkholderia-Caballeronia-Pa
d__Bacteria;p__Proteobacteria;c__Gammaproteobacteria;o__Enterobacterales;f__Erwiniaceae;g__Pantoea;s__Pantoea_agglomerans
d__Bacteria;p__Firmicutes;c__Bacilli;o__Lactobacillales;f__Carnobacteriaceae;g__Atopostipes;s__uncultured_Atopostipes_sp.
d__Bacteria;p__Firmicutes;c__Clostridia;o__Clostridiales;f__Lachnospiraceae;g__Lachnospiraceae_unclassified;s__Lachnospirac
d__Bacteria;p__Proteobacteria;c__Gammaproteobacteria;o__Pseudomonadales;f__Moraxellaceae;g__Acinetobacter;s__Acinetobacter_
d__Bacteria;p__Firmicutes;c__Bacilli;o__Staphylococcales;f__Staphylococcaceae;g__Staphylococcus;s__Staphylococcus_unclassif
d__Bacteria;p__Firmicutes;c__Bacilli;o__Lactobacillales;f__Streptococcaceae;g__Streptococcus;s__uncultured_Streptococcus_sp
d__Bacteria;p__Firmicutes;c__Bacilli;o__Lactobacillales;f__Lactobacillaceae;g__Lactobacillus;s__Lactobacillus_sp._L-YJ
d__Bacteria;p__Armatimonadota;c__Chthonomonadetes;o__Chthonomonadales;f__Chthonomonadaceae;g__Chthonomonas;s__Chthonomonas_
d__Bacteria;p__Cyanobacteria;c__Cyanobacteriia;o__Chloroplast;f__Chloroplast_unclassified;g__Chloroplast_unclassified;s__Ch
d__Bacteria;p__Proteobacteria;c__Gammaproteobacteria;o__Burkholderiales;f__Burkholderiaceae;g__Ralstonia;s__Ralstonia_uncla
d__Bacteria;p__Firmicutes;c__Clostridia;o__Thermincolales;f__Thermincolaceae;g__Thermincola;s__Thermincola_unclassified
d__Bacteria;p__Proteobacteria;c__Betaproteobacteria;o__Burkholderiales;f__Comamonadaceae;g__Comamonas;s__uncultured_Comamon
d__Bacteria;p__Actinobacteriota;c__Actinobacteria;o__Micrococcales;f__Demequinaceae;g__Demequinaceae_unclassified;s__Demequ
d__Bacteria;p__Actinobacteriota;c__Actinobacteria;o__Kineosporiales;f__Kineosporiaceae;g__Quadrisphaera;s__Quadrisphaera_un

```
d__Bacteria;p__Proteobacteria;c__Alphaproteobacteria;o__Rhizobiales;f__Phyllobacteriaceae;g__Mesorhizobium;s__Mesorhizobium
d__Bacteria;p__Proteobacteria;c__Gammaproteobacteria;o__Pseudomonadales;f__Moraxellaceae;g__Acinetobacter;s__uncultured_Aci
d__Bacteria;p__Actinobacteriota;c__Actinobacteria;o__Pseudonocardiales;f__Pseudonocardiaceae;g__Actinomycetospora;s__Actino
d__Bacteria;p__Firmicutes;c__Clostridia;o__Lachnospirales;f__Lachnospiraceae;g__Roseburia;s__Roseburia_unclassified
d__Bacteria;p__Firmicutes;c__Bacilli;o__Thermoactinomycetales;f__Thermoactinomycetaceae;g__Kroppenstedtia;s__Kroppenstedtia
d__Bacteria;p__Proteobacteria;c__Alphaproteobacteria;o__Rickettsiales;f__Mitochondria;g__Mitochondria_unclassified;s__Mitoc
d__Bacteria;p__Bacteroidota;c__Bacteroidia;o__Bacteroidales;f__Bacteroidaceae;g__Bacteroides;s__Bacteroides_unclassified
d__Bacteria;p__Actinobacteriota;c__Actinobacteria;o__Bifidobacteriales;f__Bifidobacteriaceae;g__Bifidobacterium;s__Bifidoba
d__Bacteria;p__Firmicutes;c__Bacilli;o__Lactobacillales;f__Streptococcaceae;g__Streptococcus;s__Streptococcus_anginosus
d__Bacteria;p__Proteobacteria;c__Alphaproteobacteria;o__Caulobacterales;f__Caulobacteraceae;g__Brevundimonas;s__Brevundimon
d__Bacteria;p__Verrucomicrobiota;c__Verrucomicrobiae;o__Verrucomicrobiales;f__Akkermansiaceae;g__Akkermansia;s__Akkermansia
d__Bacteria;p__Firmicutes;c__Clostridia;o__Peptostreptococcales-Tissierellales;f__Family_XI;g__Anaerococcus;s__Anaerococcus
d__Bacteria;p__Firmicutes;c__Bacilli;o__Bacillales;f__Bacillaceae;g__Geobacillus;s__Geobacillus_unclassified
d__Bacteria;p__Proteobacteria;c__Gammaproteobacteria;o__Enterobacterales;f__Enterobacteriaceae;g__Raoultella;s__Raoultella_
d__Bacteria;p__Myxococcota;c__Polyangia;o__Blfdi19;f__Blfdi19_unclassified;g__Blfdi19_unclassified;s__Blfdi19_unclassified
d__Bacteria;p__Proteobacteria;c__Gammaproteobacteria;o__Xanthomonadales;f__Xanthomonadaceae;g__Stenotrophomonas;s__Stenotro
d__Bacteria;p__Actinobacteriota;c__Actinobacteria;o__Micrococcales;f__Microbacteriaceae;g__Candidatus_Aquiluna;s__Candidatu
d__Bacteria;p__Proteobacteria;c__Alphaproteobacteria;o__Sphingomonadales;f__Sphingomonadaceae;g__Novosphingobium;s__Novosph
d__Bacteria;p__Proteobacteria;c__Gammaproteobacteria;o__Pseudomonadales;f__Moraxellaceae;g__Acinetobacter;s__uncultured_Aci
d__Bacteria;p__Proteobacteria;c__Gammaproteobacteria;o__Pseudomonadales;f__Moraxellaceae;g__Acinetobacter;s__Acinetobacter_
d__Bacteria;p__Bacteroidota;c__Bacteroidia;o__Bacteroidales;f__Muribaculaceae;g__Muribaculaceae_unclassified;s__Muribaculac
d__Bacteria;p__Firmicutes;c__Bacilli;o__Lactobacillales;f__Aerococcaceae;g__Aerococcus;s__Aerococcus_sp._148a
d__Bacteria;p__Bacteroidota;c__Bacteroidia;o__Flavobacteriales;f__Weeksellaceae;g__Chryseobacterium;s__Chryseobacterium_unc
d__Bacteria;p__Proteobacteria;c__Gammaproteobacteria;o__Pseudomonadales;f__Pseudomonadaceae;g__Pseudomonas;s__Pseudomonas_s
d__Bacteria;p__Firmicutes;c__Clostridia;o__Clostridiales;f__Lachnospiraceae;g__Lachnospiraceae_unclassified;s__Lachnospirac
d__Bacteria;p__Proteobacteria;c__Betaproteobacteria;o__Burkholderiales;f__Comamonadaceae;g__Comamonas;s__uncultured_Comamon
d__Bacteria;p__Firmicutes;c__Clostridia;o__Peptostreptococcales-Tissierellales;f__Family_XI;g__Anaerococcus;s__Anaerococcus
d__Bacteria;p__Proteobacteria;c__Alphaproteobacteria;o__Rickettsiales;f__Mitochondria;g__Mitochondria_unclassified;s__Mitoc
d__Bacteria;p__Proteobacteria;c__Gammaproteobacteria;o__Xanthomonadales;f__Xanthomonadaceae;g__Thermomonas;s__Thermomonas_u
d__Bacteria;p__Firmicutes;c__Clostridia;o__Lachnospirales;f__Lachnospiraceae;g__Dorea;s__Dorea_unclassified
d__Bacteria;p__Actinobacteriota;c__Actinobacteria;o__Bifidobacteriales;f__Bifidobacteriaceae;g__Bifidobacterium;s__Bifidoba
d__Bacteria;p__Proteobacteria;c__Gammaproteobacteria;o__Aeromonadales;f__Aeromonadaceae;g__Aeromonas;s__Aeromonas_veronii
d__Bacteria;p__Firmicutes;c__Bacilli;o__Brevibacillales;f__Brevibacillaceae;g__Brevibacillus;s__Brevibacillus_unclassified
d__Bacteria;p__Proteobacteria;c__Gammaproteobacteria;o__Pseudomonadales;f__Moraxellaceae;g__Acinetobacter;s__Acinetobacter_
d__Bacteria;p__Proteobacteria;c__Gammaproteobacteria;o__Burkholderiales;f__Oxalobacteraceae;g__Massilia;s__Massilia_unclass
d__Bacteria;p__Proteobacteria;c__Alphaproteobacteria;o__Sphingomonadales;f__Sphingomonadaceae;g__Sphingomonas;s__Sphingomon
d__Bacteria;p__Firmicutes;c__Clostridia;o__Christensenellales;f__Christensenellaceae;g__Christensenellaceae_unclassified;s_
```

d__Bacteria;p__Myxococcota;c__Polyangia;o__Blfdi19;f__Blfdi19_unclassified;g__Blfdi19_unclassified;s__Blfdi19_unclassified
d__Bacteria;p__Bacteroidota;c__Bacteroidia;o__Sphingobacteriales;f__Sphingobacteriaceae;g__Pedobacter;s__Pedobacter_unclass
d__Bacteria;p__Proteobacteria;c__Gammaproteobacteria;o__Burkholderiales;f__Burkholderiaceae;g__Burkholderia-Caballeronia-Pa
d__Bacteria;p__Proteobacteria;c__Gammaproteobacteria;o__Pseudomonadales;f__Pseudomonadaceae;g__Pseudomonas;s__uncultured_Ps
d__Bacteria;p__Firmicutes;c__Clostridia;o__Oscillospirales;f__Ruminococcaceae;g__Faecalibacterium;s__Faecalibacterium_uncla
d__Bacteria;p__Firmicutes;c__Bacilli;o__Lactobacillales;f__Streptococcaceae;g__Streptococcus;s__Streptococcus_mitis
d__Bacteria;p__Proteobacteria;c__Gammaproteobacteria;o__Pseudomonadales;f__Moraxellaceae;g__Acinetobacter;s__Acinetobacter_
d__Bacteria;p__Actinobacteriota;c__Actinobacteria;o__Corynebacteriales;f__Mycobacteriaceae;g__Mycobacterium;s__Mycobacteriu
d__Bacteria;p__Firmicutes;c__Bacilli;o__Staphylococcales;f__Staphylococcaceae;g__Staphylococcus;s__Staphylococcus_unclassif
d__Bacteria;p__Proteobacteria;c__Alphaproteobacteria;o__Rhodobacterales;f__Rhodobacteraceae;g__Planktomarina;s__Planktomari
d__Bacteria;p__Firmicutes;c__Bacilli;o__Thermoactinomycetales;f__Thermoactinomycetaceae;g__Kroppenstedtia;s__Kroppenstedtia
d__Bacteria;p__Firmicutes;c__Bacilli;o__Lactobacillales;f__Lactobacillaceae;g__Lactobacillus;s__Lactobacillus_helveticus
d__Bacteria;p__Proteobacteria;c__Gammaproteobacteria;o__Xanthomonadales;f__Rhodanobacteraceae;g__Luteibacter;s__Luteibacter
d__Bacteria;p__Firmicutes;c__Clostridia;o__Lachnospirales;f__Lachnospiraceae;g__Ruminococcus]_gnavus_group;s__Ruminococcus]
d__Bacteria;p__Firmicutes;c__Bacilli;o__Brevibacillales;f__Brevibacillaceae;g__Brevibacillus;s__Brevibacillus_unclassified
d__Bacteria;p__Proteobacteria;c__Alphaproteobacteria;o__Rhizobiales;f__Hyphomicrobiaceae;g__Hyphomicrobium;s__Hyphomicrobiu
d__Bacteria;p__Firmicutes;c__Bacilli;o__Staphylococcales;f__Staphylococcaceae;g__Staphylococcus;s__Staphylococcus_unclassif
d__Bacteria;p__Proteobacteria;c__Alphaproteobacteria;o__Rhodobacterales;f__Rhodobacteraceae;g__Paracoccus;s__Paracoccus_sp.
d__Bacteria;p__Cyanobacteria;c__Cyanobacteriia;o__Cyanobacteriales;f__Phormidiaceae;g__Tychonema_CCAP_1459-11B;s__Tychonema
d__Bacteria;p__Proteobacteria;c__Alphaproteobacteria;o__Caulobacterales;f__Caulobacteraceae;g__Brevundimonas;s__Brevundimon
d__Bacteria;p__Firmicutes;c__Bacilli;o__Bacilli_unclassified;f__Bacilli_unclassified;g__Bacilli_unclassified;s__Bacilli_unc
d__Bacteria;p__Proteobacteria;c__Gammaproteobacteria;o__Xanthomonadales;f__Xanthomonadaceae;g__Thermomonas;s__uncultured_Th
d__Bacteria;p__Firmicutes;c__Bacilli;o__Lactobacillales;f__Lactobacillaceae;g__HT002;s__HT002_unclassified
d__Bacteria;p__Firmicutes;c__Bacilli;o__Staphylococcales;f__Staphylococcaceae;g__Staphylococcus;s__Staphylococcus_unclassif
d__Bacteria;p__Proteobacteria;c__Gammaproteobacteria;o__Enterobacterales;f__Erwiniaceae;g__Pantoea;s__Pantoea_septica
d__Bacteria;p__Proteobacteria;c__Alphaproteobacteria;o__Caulobacterales;f__Caulobacteraceae;g__Brevundimonas;s__Brevundimon
d__Bacteria;p__Firmicutes;c__Bacilli;o__Bacillales;f__Bacillaceae;g__Anoxybacillus;s__Anoxybacillus_flavithermus
d__Bacteria;p__Firmicutes;c__Clostridia;o__Lachnospirales;f__Lachnospiraceae;g__Ruminococcus]_torques_group;s__Ruminococcus
d__Bacteria;p__Firmicutes;c__Bacilli;o__Lactobacillales;f__Lactobacillaceae;g__HT002;s__HT002_unclassified
d__Bacteria;p__Desulfobacterota;c__Desulfovibrionia;o__Desulfovibrionales;f__Desulfovibrionaceae;g__Desulfovibrionaceae_unc
d__Bacteria;p__Actinobacteriota;c__Actinobacteria;o__Bifidobacteriales;f__Bifidobacteriaceae;g__Bifidobacterium;s__Bifidoba
d__Bacteria;p__Proteobacteria;c__Alphaproteobacteria;o__Rhodobacterales;f__Rhodobacteraceae;g__Rubellimicrobium;s__Rubellim
d__Bacteria;p__Firmicutes;c__Bacilli;o__Lactobacillales;f__Lactobacillaceae;g__HT002;s__HT002_unclassified
d__Bacteria;p__Actinobacteriota;c__Actinobacteriota;o__Micrococcales;f__Intrasporangiaceae;g__Phycicoccus;s__Phycicoccus_sp
d__Bacteria;p__Proteobacteria;c__Gammaproteobacteria;o__Pseudomonadales;f__Moraxellaceae;g__Acinetobacter;s__uncultured_Aci
d__Bacteria;p__Verrucomicrobiota;c__Omnitrophia;o__Omnitrophales;f__Omnitrophaceae;g__Candidatus_Omnitrophus;s__Candidatus_
d__Bacteria;p__Firmicutes;c__Bacilli;o__Staphylococcales;f__Staphylococcaceae;g__Staphylococcus;s__Staphylococcus_unclassif

d__Bacteria;p__Firmicutes;c__Bacilli;o__Lactobacillales;f__Lactobacillaceae;g__HT002;s__HT002_unclassified
d__Bacteria;p__Proteobacteria;c__Gammaproteobacteria;o__Enterobacterales;f__Enterobacteriaceae;g__Escherichia-Shigella;s__E
d__Bacteria;p__Fusobacteriota;c__Fusobacteriia;o__Fusobacteriales;f__Fusobacteriaceae;g__Fusobacterium;s__Fusobacterium_unc
d__Bacteria;p__Proteobacteria;c__Gammaproteobacteria;o__Burkholderiales;f__Oxalobacteraceae;g__Massilia;s__Massilia_unclass
d__Bacteria;p__Proteobacteria;c__Alphaproteobacteria;o__Rhizobiales;f__Xanthobacteraceae;g__Xanthobacter;s__Xanthobacter_au
d__Bacteria;p__Firmicutes;c__Clostridia;o__Clostridiales;f__Clostridiaceae;g__Butyricicoccus;s__Butyricicoccus_pullicaecoru
d__Bacteria;p__Proteobacteria;c__Gammaproteobacteria;o__Burkholderiales;f__Comamonadaceae;g__Comamonas;s__Comamonas_unclass
d__Bacteria;p__Proteobacteria;c__Alphaproteobacteria;o__Sphingomonadales;f__Sphingomonadaceae;g__Sphingomonas;s__Sphingomon
d__Bacteria;p__Firmicutes;c__Bacilli;o__Lactobacillales;f__Streptococcaceae;g__Streptococcus;s__uncultured_Streptococcus_sp
d__Bacteria;p__Firmicutes;c__Clostridia;o__Lachnospirales;f__Lachnospiraceae;g__Blautia;s__Blautia_unclassified
d__Bacteria;p__Firmicutes;c__Clostridia;o__Peptostreptococcales-Tissierellales;f__Family_XI;g__Peptoniphilus;s__Peptoniphil
d__Bacteria;p__Actinobacteriota;c__Actinobacteria;o__Corynebacteriales;f__Mycobacteriaceae;g__Mycobacterium;s__Mycobacteriu
d__Bacteria;p__Proteobacteria;c__Gammaproteobacteria;o__Xanthomonadales;f__Rhodanobacteraceae;g__Dyella;s__Dyella_ginsengis
d__Bacteria;p__Proteobacteria;c__Betaproteobacteria;o__Burkholderiales;f__Oxalobacteraceae;g__Oxalobacter;s__Oxalobacter_sp
d__Bacteria;p__Firmicutes;c__Clostridia;o__Clostridiales;f__Clostridiaceae;g__Clostridiaceae_unclassified;s__Clostridiaceae
d__Bacteria;p__Actinobacteriota;c__Actinobacteriota;o__Streptomycetales;f__Streptomycetaceae;g__Streptomyces;s__Streptomyce
d__Bacteria;p__Cyanobacteria;c__Cyanobacteriia;o__Chloroplast;f__Chloroplast_unclassified;g__Chloroplast_unclassified;s__Ch
d__Bacteria;p__Proteobacteria;c__Betaproteobacteria;o__Burkholderiales;f__Comamonadaceae;g__Comamonas;s__Comamonas_sp._SS-1
d__Bacteria;p__Proteobacteria;c__Gammaproteobacteria;o__Burkholderiales;f__Burkholderiaceae;g__Ralstonia;s__Ralstonia_uncla
d__Bacteria;p__Proteobacteria;c__Alphaproteobacteria;o__Acetobacterales;f__Acetobacteraceae;g__Craurococcus-Caldovatus;s__C
d__Bacteria;p__Proteobacteria;c__Alphaproteobacteria;o__Sphingomonadales;f__Sphingomonadaceae;g__Sphingobium;s__Sphingobium
d__Bacteria;p__Proteobacteria;c__Gammaproteobacteria;o__Enterobacterales;f__Enterobacteriaceae;g__Raoultella;s__Raoultella_
d__Bacteria;p__Firmicutes;c__Bacilli;o__Lactobacillales;f__Lactobacillaceae;g__Ligilactobacillus;s__Ligilactobacillus_uncla
d__Bacteria;p__Firmicutes;c__Clostridia;o__Oscillospirales;f__Ruminococcaceae;g__Faecalibacterium;s__Faecalibacterium_uncla
d__unclassified;p__unclassified;c__unclassified;o__unclassified;f__unclassified;g__unclassified;s__unclassified
d__Bacteria;p__Bacteroidota;c__Bacteroidia;o__Cytophagales;f__Hymenobacteraceae;g__Hymenobacter;s__Hymenobacter_unclassifie
d__Bacteria;p__Proteobacteria;c__Gammaproteobacteria;o__Pseudomonadales;f__Moraxellaceae;g__Acinetobacter;s__uncultured_Aci
d__Bacteria;p__Proteobacteria;c__Gammaproteobacteria;o__Enterobacterales;f__Enterobacteriaceae;g__Klebsiella;s__uncultured_
d__Bacteria;p__Proteobacteria;c__Gammaproteobacteria;o__Enterobacterales;f__Morganellaceae;g__Proteus;s__Proteus_mirabilis
d__Bacteria;p__Proteobacteria;c__Alphaproteobacteria;o__Rhizobiales;f__Hyphomicrobiaceae;g__Hyphomicrobium;s__uncultured_Hy
d__Bacteria;p__Firmicutes;c__Bacilli;o__Staphylococcales;f__Staphylococcaceae;g__Staphylococcus;s__Staphylococcus_unclassif
d__Bacteria;p__Firmicutes;c__Bacilli;o__Lactobacillales;f__Streptococcaceae;g__Streptococcus;s__Streptococcus_thermophilus
d__Bacteria;p__Firmicutes;c__Bacilli;o__Staphylococcales;f__Staphylococcaceae;g__Staphylococcus;s__Staphylococcus_unclassif
d__Bacteria;p__Firmicutes;c__Clostridia;o__Lachnospirales;f__Lachnospiraceae;g__Catonella;s__Catonella_unclassified
d__Bacteria;p__Proteobacteria;c__Gammaproteobacteria;o__Burkholderiales;f__Oxalobacteraceae;g__Massilia;s__Massilia_unclass
d__Bacteria;p__Proteobacteria;c__Gammaproteobacteria;o__Xanthomonadales;f__Xanthomonadaceae;g__Stenotrophomonas;s__Stenotro
d__Bacteria;p__Proteobacteria;c__Gammaproteobacteria;o__Pseudomonadales;f__Pseudomonadaceae;g__Pseudomonas;s__Pseudomonas_s

d__Bacteria;p__Proteobacteria;c__Proteobacteria_unclassified;o__Proteobacteria_unclassified;f__Proteobacteria_unclassified;
d__Bacteria;p__Proteobacteria;c__Gammaproteobacteria;o__Burkholderiales;f__Oxalobacteraceae;g__Herbaspirillum;s__Herbaspiri
d__Bacteria;p__Proteobacteria;c__Gammaproteobacteria;o__Xanthomonadales;f__Xanthomonadaceae;g__Stenotrophomonas;s__Stenotro
d__Bacteria;p__Bacteroidota;c__Bacteroidia;o__Bacteroidales;f__Prevotellaceae;g__Prevotella_9;s__Prevotella_9_unclassified
d__Bacteria;p__Firmicutes;c__Clostridia;o__Clostridiales;f__Ruminococcaceae;g__Acetivibrio;s__uncultured_Acetivibrio_sp.
d__Bacteria;p__Proteobacteria;c__Gammaproteobacteria;o__Enterobacterales;f__Pasteurellaceae;g__Haemophilus;s__Haemophilus_u
d__Bacteria;p__Firmicutes;c__Bacilli;o__Bacillales;f__Sporolactobacillaceae;g__Scopulibacillus;s__Scopulibacillus_unclassif
d__Bacteria;p__Actinobacteriota;c__Actinobacteria;o__Propionibacteriales;f__Nocardioidaceae;g__Marmoricola;s__Marmoricola_u
d__Bacteria;p__Proteobacteria;c__Betaproteobacteria;o__Burkholderiales;f__Comamonadaceae;g__Comamonas;s__uncultured_Comamon
d__Bacteria;p__Firmicutes;c__Bacilli;o__Lactobacillales;f__Streptococcaceae;g__Streptococcus;s__uncultured_Streptococcus_sp
d__Bacteria;p__Proteobacteria;c__Gammaproteobacteria;o__Pseudomonadales;f__Pseudomonadaceae;g__Pseudomonas;s__Pseudomonas_s
d__Bacteria;p__Cyanobacteria;c__Cyanobacteriia;o__Chloroplast;f__Chloroplast_unclassified;g__Chloroplast_unclassified;s__Ch
d__Bacteria;p__Cyanobacteria;c__Cyanobacteriia;o__Chloroplast;f__Chloroplast_unclassified;g__Chloroplast_unclassified;s__Ch
d__Bacteria;p__Bacteroidota;c__Bacteroidia;o__Bacteroidales;f__Rikenellaceae;g__Alistipes;s__Alistipes_sp._Marseille-P5061
d__Bacteria;p__Chloroflexi;c__Chloroflexia;o__Thermomicrobiales;f__JG30-KF-CM45;g__JG30-KF-CM45_unclassified;s__JG30-KF-CM4
d__Bacteria;p__Chloroflexi;c__Chloroflexia;o__Thermomicrobiales;f__JG30-KF-CM45;g__JG30-KF-CM45_unclassified;s__JG30-KF-CM4
d__Bacteria;p__Firmicutes;c__Bacilli;o__Bacillales;f__Bacillaceae;g__Geobacillus;s__Geobacillus_stearothermophilus
d__Bacteria;p__Proteobacteria;c__Alphaproteobacteria;o__Rhizobiales;f__Hyphomicrobiaceae;g__Hyphomicrobium;s__uncultured_Hy
d__Bacteria;p__Firmicutes;c__Bacilli;o__Brevibacillales;f__Brevibacillaceae;g__Brevibacillus;s__Brevibacillus_unclassified
d__Bacteria;p__Proteobacteria;c__Gammaproteobacteria;o__Enterobacterales;f__Erwiniaceae;g__Pantoea;s__Pantoea_septica
d__Bacteria;p__Firmicutes;c__Clostridia;o__Lachnospirales;f__Lachnospiraceae;g__Blautia;s__Blautia_unclassified
d__Bacteria;p__Actinobacteriota;c__Actinobacteria;o__Bifidobacteriales;f__Bifidobacteriaceae;g__Gardnerella;s__Gardnerella_
d__Bacteria;p__Proteobacteria;c__Alphaproteobacteria;o__Caulobacterales;f__Caulobacteraceae;g__Brevundimonas;s__Brevundimon
d__Bacteria;p__Proteobacteria;c__Gammaproteobacteria;o__Enterobacterales;f__Enterobacteriaceae;g__Raoultella;s__Raoultella_
d__Bacteria;p__Proteobacteria;c__Gammaproteobacteria;o__Burkholderiales;f__Methylophilaceae;g__OM43_clade;s__OM43_clade_unc
d__Bacteria;p__Proteobacteria;c__Alphaproteobacteria;o__Sphingomonadales;f__Sphingomonadaceae;g__Sphingomonas;s__Sphingomon
d__Bacteria;p__Firmicutes;c__Bacilli;o__Staphylococcales;f__Staphylococcaceae;g__Staphylococcus;s__Staphylococcus_unclassif
d__Bacteria;p__Proteobacteria;c__Alphaproteobacteria;o__Rhizobiales;f__Rhizobiaceae;g__Ochrobactrum;s__Ochrobactrum_unclass
d__Bacteria;p__Patescibacteria;c__Parcubacteria;o__Candidatus_Nomurabacteria;f__Candidatus_Nomurabacteria_unclassified;g__C
d__Bacteria;p__Firmicutes;c__Clostridia;o__Oscillospirales;f__Ruminococcaceae;g__Faecalibacterium;s__Faecalibacterium_uncla
d__Bacteria;p__Actinobacteriota;c__Actinobacteria;o__Corynebacteriales;f__Nocardiaceae;g__Rhodococcus;s__Rhodococcus_unclas
d__Bacteria;p__Proteobacteria;c__Alphaproteobacteria;o__Rhizobiales;f__Rhizobiaceae;g__Allorhizobium-Neorhizobium-Pararhizo
d__Bacteria;p__Actinobacteriota;c__Actinobacteria;o__Pseudonocardiales;f__Pseudonocardiaceae;g__Saccharopolyspora;s__Saccha
d__Bacteria;p__Firmicutes;c__Bacilli;o__Staphylococcales;f__Staphylococcaceae;g__Staphylococcus;s__Staphylococcus_unclassif
d__Bacteria;p__Proteobacteria;c__Alphaproteobacteria;o__Caulobacterales;f__Caulobacteraceae;g__Brevundimonas;s__Brevundimon
d__Bacteria;p__Proteobacteria;c__Gammaproteobacteria;o__Burkholderiales;f__Burkholderiaceae;g__Burkholderia-Caballeronia-Pa
d__Bacteria;p__Actinobacteriota;c__Actinobacteria;o__Corynebacteriales;f__Corynebacteriaceae;g__Corynebacterium;s__Coryneba

d__Bacteria;p__Proteobacteria;c__Betaproteobacteria;o__Burkholderiales;f__Comamonadaceae;g__Comamonas;s__Comamonas_sp._KR5.
d__Bacteria;p__Actinobacteriota;c__Actinobacteria;o__Corynebacteriales;f__Corynebacteriaceae;g__Corynebacterium;s__Coryneba
d__Bacteria;p__Proteobacteria;c__Betaproteobacteria;o__Burkholderiales;f__Comamonadaceae;g__Comamonas;s__uncultured_Comamon
d__Bacteria;p__Firmicutes;c__Clostridia;o__Clostridiales;f__Heliobacteriaceae;g__Heliobacterium;s__Heliobacterium_modestica
d__Bacteria;p__Firmicutes;c__Bacilli;o__Erysipelotrichales;f__Erysipelatoclostridiaceae;g__Erysipelotrichaceae_UCG-003;s__E
d__Bacteria;p__Proteobacteria;c__Gammaproteobacteria;o__Pseudomonadales;f__Moraxellaceae;g__Acinetobacter;s__Acinetobacter_
d__Bacteria;p__Proteobacteria;c__Alphaproteobacteria;o__Caulobacterales;f__Caulobacteraceae;g__Caulobacter;s__uncultured_Ca
d__Bacteria;p__Proteobacteria;c__Gammaproteobacteria;o__Pseudomonadales;f__Pseudomonadaceae;g__Pseudomonas;s__uncultured_Ps
d__Bacteria;p__Proteobacteria;c__Gammaproteobacteria;o__Pseudomonadales;f__Moraxellaceae;g__Acinetobacter;s__uncultured_Aci
d__Bacteria;p__Firmicutes;c__Bacilli;o__Paenibacillales;f__Paenibacillaceae;g__Paenibacillus;s__Paenibacillus_unclassified
d__Bacteria;p__Calditrichota;c__Calditrichia;o__Calditrichales;f__Calditrichaceae;g__JdFR-76;s__JdFR-76_unclassified
d__Bacteria;p__Firmicutes;c__Bacilli;o__Bacillales;f__Bacillaceae;g__Bacillus;s__Bacillus_methanolicus
d__Bacteria;p__Firmicutes;c__Clostridia;o__Clostridiales;f__Lachnospiraceae;g__Agathobacter;s__Agathobacter_ruminis
d__Bacteria;p__Firmicutes;c__Clostridia;o__Clostridiales;f__Clostridiaceae;g__Clostridium_sensu_stricto_1;s__Clostridium_se
d__Bacteria;p__Proteobacteria;c__Alphaproteobacteria;o__Sphingomonadales;f__Sphingomonadaceae;g__Sphingomonas;s__Sphingomon
d__Bacteria;p__Firmicutes;c__Bacilli;o__Bacillales;f__Bacillaceae;g__Oceanobacillus;s__Oceanobacillus_unclassified
d__Bacteria;p__Actinobacteriota;c__Actinobacteria;o__Bifidobacteriales;f__Bifidobacteriaceae;g__Gardnerella;s__Gardnerella_
d__Bacteria;p__Proteobacteria;c__Alphaproteobacteria;o__SAR11_clade;f__Clade_I;g__Clade_Ia;s__Clade_Ia_unclassified
d__Bacteria;p__Firmicutes;c__Clostridia;o__Clostridia_UCG-014;f__Clostridia_UCG-014_unclassified;g__Clostridia_UCG-014_uncl
d__Bacteria;p__Firmicutes;c__Clostridia;o__Oscillospirales;f__Ruminococcaceae;g__Faecalibacterium;s__Faecalibacterium_uncla
d__Bacteria;p__Firmicutes;c__Clostridia;o__Oscillospirales;f__Oscillospiraceae;g__Intestinimonas;s__Intestinimonas_unclassi
d__Bacteria;p__Firmicutes;c__Bacilli;o__Bacillales;f__Planococcaceae;g__Lysinibacillus;s__Lysinibacillus_unclassified
d__Bacteria;p__Proteobacteria;c__Gammaproteobacteria;o__Burkholderiales;f__Burkholderiaceae;g__Burkholderia-Caballeronia-Pa
d__Bacteria;p__Proteobacteria;c__Gammaproteobacteria;o__Enterobacterales;f__Enterobacteriaceae;g__Klebsiella;s__Klebsiella_
d__Bacteria;p__Proteobacteria;c__Alphaproteobacteria;o__Sphingomonadales;f__Sphingomonadaceae;g__Sphingomonas;s__Sphingomon
d__Bacteria;p__Firmicutes;c__Bacilli;o__Lactobacillales;f__P5D1-392;g__P5D1-392_unclassified;s__P5D1-392_unclassified
d__Bacteria;p__Firmicutes;c__Bacilli;o__Brevibacillales;f__Brevibacillaceae;g__Brevibacillus;s__Brevibacillus_unclassified
d__Bacteria;p__Proteobacteria;c__Alphaproteobacteria;o__Rhizobiales;f__Rhizobiaceae;g__Rhizobium;s__uncultured_Rhizobium_sp
d__Bacteria;p__Firmicutes;c__Clostridia;o__Lachnospirales;f__Lachnospiraceae;g__Sellimonas;s__Sellimonas_unclassified
d__Bacteria;p__Proteobacteria;c__Gammaproteobacteria;o__Pseudomonadales;f__Moraxellaceae;g__Acinetobacter;s__Acinetobacter_
d__Bacteria;p__Proteobacteria;c__Gammaproteobacteria;o__Pseudomonadales;f__Halomonadaceae;g__Halomonas;s__Halomonas_unclass
d__Bacteria;p__Proteobacteria;c__Gammaproteobacteria;o__Enterobacterales;f__Enterobacteriaceae;g__Enterobacter;s__Enterobac
d__Bacteria;p__Firmicutes;c__Bacilli;o__Bacillales;f__Bacillaceae;g__Bacillus;s__Bacillus_sp._LBM_1024
d__Bacteria;p__Proteobacteria;c__Gammaproteobacteria;o__Enterobacterales;f__Erwiniaceae;g__Pantoea;s__Pantoea_sp._Sc1
d__Bacteria;p__Firmicutes;c__Bacilli;o__Lactobacillales;f__Lactobacillaceae;g__Latilactobacillus;s__Latilactobacillus_uncla
d__Bacteria;p__Proteobacteria;c__Gammaproteobacteria;o__Pseudomonadales;f__Pseudomonadaceae;g__Pseudomonas;s__uncultured_Ps
d__Bacteria;p__Firmicutes;c__Clostridia;o__Oscillospirales;f__Ruminococcaceae;g__Subdoligranulum;s__Subdoligranulum_unclass

d__Bacteria;p__Proteobacteria;c__Betaproteobacteria;o__Burkholderiales;f__Comamonadaceae;g__Comamonas;s__Comamonas_sp._V2M3
d__Bacteria;p__Firmicutes;c__Bacilli;o__Lactobacillales;f__Carnobacteriaceae;g__Atopostipes;s__uncultured_Atopostipes_sp.
d__Bacteria;p__Firmicutes;c__Bacilli;o__Lactobacillales;f__Lactobacillaceae;g__Limosilactobacillus;s__Limosilactobacillus_u
d__Bacteria;p__Proteobacteria;c__Alphaproteobacteria;o__Sphingomonadales;f__Sphingomonadaceae;g__Sphingomonas;s__uncultured
d__Bacteria;p__Firmicutes;c__Clostridia;o__Oscillospirales;f__Ruminococcaceae;g__Faecalibacterium;s__Faecalibacterium_uncla
d__Bacteria;p__Gemmatimonadota;c__AKAU4049;o__AKAU4049_unclassified;f__AKAU4049_unclassified;g__AKAU4049_unclassified;s__AK
d__Bacteria;p__Proteobacteria;c__Gammaproteobacteria;o__Pseudomonadales;f__Moraxellaceae;g__Acinetobacter;s__Acinetobacter_
d__Bacteria;p__Bacteroidota;c__Bacteroidia;o__Bacteroidales;f__Prevotellaceae;g__Alloprevotella;s__Alloprevotella_unclassif
d__Bacteria;p__Firmicutes;c__Bacilli;o__Lactobacillales;f__Streptococcaceae;g__Streptococcus;s__Streptococcus_thermophilus
d__Bacteria;p__Cyanobacteria;c__Cyanobacteriia;o__Chloroplast;f__Chloroplast_unclassified;g__Chloroplast_unclassified;s__Ch
d__Bacteria;p__Firmicutes;c__Clostridia;o__Peptostreptococcales-Tissierellales;f__Family_XI;g__Peptoniphilus;s__Peptoniphil
d__Bacteria;p__Proteobacteria;c__Gammaproteobacteria;o__Enterobacterales;f__Enterobacteriaceae;g__Enterobacter;s__Enterobac
d__Bacteria;p__Firmicutes;c__Clostridia;o__Clostridiales;f__Clostridiaceae;g__Clostridium_sensu_stricto_1;s__Clostridium_se
d__Bacteria;p__Firmicutes;c__Clostridia;o__Clostridiales;f__Clostridiaceae;g__Clostridium_sensu_stricto_13;s__Clostridium_s
d__Bacteria;p__Firmicutes;c__Bacilli;o__Thermicanales;f__Thermicanaceae;g__Thermicanus;s__Thermicanus_unclassified
d__Bacteria;p__Firmicutes;c__Bacilli;o__Lactobacillales;f__Streptococcaceae;g__Streptococcus;s__Streptococcus_thermophilus
d__Bacteria;p__Fusobacteriota;c__Fusobacteriia;o__Fusobacteriales;f__Fusobacteriaceae;g__Fusobacterium;s__Fusobacterium_unc
d__Bacteria;p__Campylobacterota;c__Campylobacteria;o__Campylobacterales;f__Helicobacteraceae;g__Helicobacter;s__Helicobacte
d__Bacteria;p__Proteobacteria;c__Alphaproteobacteria;o__Rhodobacterales;f__Rhodobacteraceae;g__HIMB11;s__HIMB11_unclassifie
d__Bacteria;p__Proteobacteria;c__Gammaproteobacteria;o__Pseudomonadales;f__SAR86_clade;g__SAR86_clade_unclassified;s__SAR86
d__Bacteria;p__Proteobacteria;c__Gammaproteobacteria;o__Pseudomonadales;f__SAR86_clade;g__SAR86_clade_unclassified;s__SAR86
d__Bacteria;p__Actinobacteriota;c__Actinobacteria;o__Micrococcales;f__Microbacteriaceae;g__Agromyces;s__Agromyces_unclassif
d__Bacteria;p__Acidobacteriota;c__Blastocatellia;o__Pyrinomonadales;f__Pyrinomonadaceae;g__RB41;s__RB41_unclassified
d__Bacteria;p__Firmicutes;c__Bacilli;o__Aneurinibacillales;f__Aneurinibacillaceae;g__Aneurinibacillus;s__Aneurinibacillus_u
d__Bacteria;p__Proteobacteria;c__Gammaproteobacteria;o__Burkholderiales;f__Burkholderiaceae;g__Burkholderia-Caballeronia-Pa
d__Bacteria;p__Firmicutes;c__Bacilli;o__Lactobacillales;f__Carnobacteriaceae;g__Atopostipes;s__uncultured_Atopostipes_sp.
d__Bacteria;p__Firmicutes;c__Bacilli;o__Staphylococcales;f__Staphylococcaceae;g__Staphylococcus;s__Staphylococcus_unclassif
d__Bacteria;p__Proteobacteria;c__Gammaproteobacteria;o__Burkholderiales;f__Comamonadaceae;g__Comamonas;s__Comamonas_unclass
d__Bacteria;p__Actinobacteriota;c__Actinobacteria;o__Micrococcales;f__Micrococcaceae;g__Rothia;s__Rothia_unclassified
d__Bacteria;p__Proteobacteria;c__Gammaproteobacteria;o__Burkholderiales;f__Comamonadaceae;g__Paucibacter;s__Paucibacter_unc
d__Bacteria;p__Firmicutes;c__Bacilli;o__Bacilli_unclassified;f__Bacilli_unclassified;g__Bacilli_unclassified;s__Bacilli_unc
d__Bacteria;p__Firmicutes;c__Bacilli;o__Bacillales;f__Bacillaceae;g__Bacillus;s__Bacillus_thermoamylovorans
d__Bacteria;p__Proteobacteria;c__Gammaproteobacteria;o__Xanthomonadales;f__Xanthomonadaceae;g__Stenotrophomonas;s__Stenotro
d__Bacteria;p__Firmicutes;c__Clostridia;o__Clostridiales;f__Clostridiales_Family_XIV._Incertae_Sedis;g__Clostridiales_Famil
d__Bacteria;p__Proteobacteria;c__Alphaproteobacteria;o__Sphingomonadales;f__Sphingomonadaceae;g__Sphingomonas;s__Sphingomon
d__Bacteria;p__Firmicutes;c__Bacilli;o__Brevibacillales;f__Brevibacillaceae;g__Brevibacillus;s__Brevibacillus_unclassified
d__Bacteria;p__Firmicutes;c__Bacilli;o__Lactobacillales;f__Streptococcaceae;g__Streptococcus;s__Streptococcus_thermophilus

d__Bacteria;p__Firmicutes;c__Clostridia;o__Caldicoprobacterales;f__Caldicoprobacteraceae;g__Caldicoprobacter;s__Caldicoprob
d__Bacteria;p__Bacteroidota;c__Bacteroidia;o__Flavobacteriales;f__Weeksellaceae;g__Chryseobacterium;s__Chryseobacterium_unc
d__Bacteria;p__Proteobacteria;c__Betaproteobacteria;o__Burkholderiales;f__Comamonadaceae;g__Comamonas;s__uncultured_Comamon
d__Bacteria;p__Actinobacteriota;c__Actinobacteria;o__Micrococcales;f__Micrococcaceae;g__Micrococcus;s__Micrococcus_unclassi
d__Bacteria;p__Actinobacteriota;c__Thermoleophilia;o__Gaiellales;f__Gaiellales_unclassified;g__Gaiellales_unclassified;s__G
d__Bacteria;p__Proteobacteria;c__Gammaproteobacteria;o__Pseudomonadales;f__Moraxellaceae;g__Acinetobacter;s__Acinetobacter_
d__Bacteria;p__Myxococcota;c__Polyangia;o__Polyangiales;f__BIrii41;g__BIrii41_unclassified;s__BIrii41_unclassified
d__Bacteria;p__Firmicutes;c__Clostridia;o__Oscillospirales;f__Ruminococcaceae;g__Subdoligranulum;s__Subdoligranulum_unclass
d__Bacteria;p__Firmicutes;c__Clostridia;o__Clostridiales;f__Clostridiaceae;g__Clostridium_sensu_stricto_1;s__Clostridium_se
d__Bacteria;p__Proteobacteria;c__Gammaproteobacteria;o__Pseudomonadales;f__Moraxellaceae;g__Acinetobacter;s__Acinetobacter_
d__Bacteria;p__Proteobacteria;c__Betaproteobacteria;o__Burkholderiales;f__Comamonadaceae;g__Comamonas;s__Comamonas_sp._TS24
d__Bacteria;p__Firmicutes;c__Bacilli;o__Bacillales;f__Bacillaceae;g__Geobacillus;s__Geobacillus_sp.
d__Bacteria;p__Firmicutes;c__Clostridia;o__Peptostreptococcales-Tissierellales;f__Family_XI;g__Peptoniphilus;s__Peptoniphil
d__Bacteria;p__Actinobacteriota;c__Actinobacteria;o__Propionibacteriales;f__Nocardioidaceae;g__Marmoricola;s__Marmoricola_u
d__Bacteria;p__Proteobacteria;c__Gammaproteobacteria;o__Xanthomonadales;f__Xanthomonadaceae;g__Stenotrophomonas;s__Stenotro
d__Bacteria;p__Proteobacteria;c__Gammaproteobacteria;o__Pseudomonadales;f__Thioglobaceae;g__SUP05_cluster;s__SUP05_cluster_
d__Bacteria;p__Proteobacteria;c__Betaproteobacteria;o__Burkholderiales;f__Comamonadaceae;g__Acidovorax;s__Acidovorax_sp.
d__Bacteria;p__Bacteroidota;c__Bacteroidia;o__Chitinophagales;f__Chitinophagaceae;g__Sediminibacterium;s__Sediminibacterium
d__Bacteria;p__Actinobacteriota;c__Actinobacteria;o__PeM15;f__PeM15_unclassified;g__PeM15_unclassified;s__PeM15_unclassifie
d__Bacteria;p__Firmicutes;c__Bacilli;o__Lactobacillales;f__Streptococcaceae;g__Streptococcus;s__Streptococcus_parasanguinis
d__Bacteria;p__Proteobacteria;c__Gammaproteobacteria;o__Pseudomonadales;f__Pseudomonadaceae;g__Pseudomonas;s__Pseudomonas_m
d__Bacteria;p__Proteobacteria;c__Gammaproteobacteria;o__Burkholderiales;f__Comamonadaceae;g__Hydrogenophaga;s__Hydrogenopha
d__Bacteria;p__Firmicutes;c__Bacilli;o__Lactobacillales;f__Streptococcaceae;g__Streptococcus;s__Streptococcus_thermophilus
d__Bacteria;p__Firmicutes;c__Clostridia;o__Oscillospirales;f__Ruminococcaceae;g__Faecalibacterium;s__Faecalibacterium_uncla
d__Bacteria;p__Verrucomicrobiota;c__Verrucomicrobiae;o__Verrucomicrobiales;f__Akkermansiaceae;g__Akkermansia;s__Akkermansia
d__Bacteria;p__Actinobacteriota;c__Actinobacteria;o__Streptosporangiales;f__Nocardiopsaceae;g__Nocardiopsis;s__Nocardiopsis
d__Bacteria;p__Bacteroidota;c__Bacteroidia;o__Sphingobacteriales;f__Sphingobacteriaceae;g__Pedobacter;s__Pedobacter_unclass
d__Bacteria;p__Actinobacteriota;c__Actinobacteria;o__Corynebacteriales;f__Dietziaceae;g__Dietzia;s__Dietzia_unclassified
d__Bacteria;p__Actinobacteriota;c__Actinobacteria;o__Micrococcales;f__Dermacoccaceae;g__Dermacoccus;s__Dermacoccus_unclassi
d__Bacteria;p__Firmicutes;c__Clostridia;o__Christensenellales;f__Christensenellaceae;g__Christensenellaceae_R-7_group;s__Ch
d__Bacteria;p__Proteobacteria;c__Gammaproteobacteria;o__Burkholderiales;f__Sutterellaceae;g__Sutterella;s__Sutterella_uncla
d__Bacteria;p__Gemmatimonadota;c__Gemmatimonadetes;o__Gemmatimonadales;f__Gemmatimonadaceae;g__Gemmatimonadaceae_unclassifi
d__Bacteria;p__Proteobacteria;c__Alphaproteobacteria;o__Rhodobacterales;f__Rhodobacteraceae;g__HIMB11;s__HIMB11_unclassifie
d__Bacteria;p__Proteobacteria;c__Alphaproteobacteria;o__Rhizobiales;f__Beijerinckiaceae;g__Methylobacterium-Methylorubrum;s
d__Bacteria;p__Desulfobacterota;c__Desulfovibrionia;o__Desulfovibrionales;f__Desulfovibrionaceae;g__Bilophila;s__Bilophila_
d__Bacteria;p__Firmicutes;c__Bacilli;o__Thermicanales;f__Thermicanaceae;g__Thermicanus;s__Thermicanus_unclassified
d__Bacteria;p__Proteobacteria;c__Gammaproteobacteria;o__Burkholderiales;f__Rhodocyclaceae;g__Dechlorosoma;s__Dechlorosoma_u

d__Bacteria;p__Firmicutes;c__Clostridia;o__Lachnospirales;f__Lachnospiraceae;g__Blautia;s__Blautia_unclassified
d__Bacteria;p__Proteobacteria;c__Gammaproteobacteria;o__Enterobacterales;f__Enterobacteriaceae;g__Salmonella;s__Salmonella_
d__Bacteria;p__Firmicutes;c__Bacilli;o__Lactobacillales;f__Lactobacillaceae;g__Lactobacillus;s__Lactobacillus_sp._L-YJ
d__Bacteria;p__Firmicutes;c__Clostridia;o__Lachnospirales;f__Lachnospiraceae;g__Ruminococcus]_torques_group;s__Ruminococcus
d__Bacteria;p__Firmicutes;c__Bacilli;o__Lactobacillales;f__Streptococcaceae;g__Streptococcus;s__uncultured_Streptococcus_sp
d__Bacteria;p__Firmicutes;c__Clostridia;o__Oscillospirales;f__Eubacterium]_coprostanoligenes_group;g__Eubacterium]_coprosta
d__Bacteria;p__Bacteroidota;c__Bacteroidia;o__Bacteroidales;f__Rikenellaceae;g__Alistipes;s__uncultured_Alistipes_sp.
d__Bacteria;p__Firmicutes;c__Clostridia;o__Clostridiales;f__Clostridiaceae;g__Clostridium_sensu_stricto_1;s__Clostridium_se
d__Bacteria;p__Firmicutes;c__Clostridia;o__Clostridiales;f__Gracilibacteraceae;g__Gracilibacteraceae_unclassified;s__Gracil
d__Bacteria;p__Proteobacteria;c__Gammaproteobacteria;o__Xanthomonadales;f__Xanthomonadaceae;g__Stenotrophomonas;s__Stenotro
d__Bacteria;p__Deinococcota;c__Deinococci;o__Thermales;f__Thermaceae;g__Thermus;s__Thermus_unclassified
d__Bacteria;p__Proteobacteria;c__Alphaproteobacteria;o__Rhizobiales;f__Beijerinckiaceae;g__Bosea;s__Bosea_unclassified
d__Bacteria;p__Proteobacteria;c__Gammaproteobacteria;o__Pseudomonadales;f__Pseudomonadaceae;g__Pseudomonas;s__Pseudomonas_s
d__Bacteria;p__Actinobacteriota;c__Actinobacteria;o__Micrococcales;f__Micrococcaceae;g__Kocuria;s__Kocuria_unclassified
d__Bacteria;p__Bacteroidota;c__Bacteroidia;o__Bacteroidales;f__Prevotellaceae;g__Prevotella;s__uncultured_Prevotella_sp.
d__Bacteria;p__Proteobacteria;c__Gammaproteobacteria;o__Enterobacterales;f__Enterobacteriaceae;g__Enterobacter;s__Enterobac
d__Bacteria;p__Proteobacteria;c__Gammaproteobacteria;o__Burkholderiales;f__Rhodocyclaceae;g__Dechlorosoma;s__Dechlorosoma_u
d__Bacteria;p__Firmicutes;c__Bacilli;o__Staphylococcales;f__Staphylococcaceae;g__Staphylococcus;s__Staphylococcus_unclassif
d__Bacteria;p__Firmicutes;c__Negativicutes;o__Veillonellales-Selenomonadales;f__Veillonellaceae;g__Dialister;s__Dialister_u
d__Bacteria;p__Proteobacteria;c__Gammaproteobacteria;o__Burkholderiales;f__Comamonadaceae;g__Variovorax;s__Variovorax_uncla
d__Bacteria;p__Actinobacteriota;c__Actinobacteria;o__Micrococcales;f__Microbacteriaceae;g__Gryllotalpicola;s__Gryllotalpico
d__Bacteria;p__Proteobacteria;c__Alphaproteobacteria;o__Sphingomonadales;f__Sphingomonadaceae;g__Sphingomonas;s__Sphingomon
d__Bacteria;p__Firmicutes;c__Clostridia;o__Clostridiales;f__Clostridiaceae;g__Clostridium_sensu_stricto_1;s__Clostridium_se
d__Bacteria;p__Proteobacteria;c__Gammaproteobacteria;o__Enterobacterales;f__Vibrionaceae;g__Vibrio;s__Vibrio_unclassified
d__Bacteria;p__Acidobacteriota;c__Acidobacteriae;o__Acidobacteriales;f__Acidobacteriaceae_(Subgroup_1);g__Granulicella;s__G
d__Bacteria;p__Proteobacteria;c__Gammaproteobacteria;o__Burkholderiales;f__Burkholderiaceae;g__Burkholderia-Caballeronia-Pa
d__Bacteria;p__Firmicutes;c__Bacilli;o__Lactobacillales;f__Streptococcaceae;g__Streptococcus;s__Streptococcus_thermophilus
d__Bacteria;p__Cyanobacteria;c__Cyanobacteriia;o__Chloroplast;f__Chloroplast_unclassified;g__Chloroplast_unclassified;s__Ch
d__Bacteria;p__Proteobacteria;c__Gammaproteobacteria;o__Pseudomonadales;f__Moraxellaceae;g__Acinetobacter;s__uncultured_Aci
d__Bacteria;p__Proteobacteria;c__Gammaproteobacteria;o__Burkholderiales;f__Oxalobacteraceae;g__Janthinobacterium;s__Janthin
d__Bacteria;p__Firmicutes;c__Bacilli;o__Lactobacillales;f__Lactobacillaceae;g__Secundilactobacillus;s__Secundilactobacillus
d__Bacteria;p__Proteobacteria;c__Gammaproteobacteria;o__Pseudomonadales;f__Moraxellaceae;g__Acinetobacter;s__uncultured_Aci
d__Bacteria;p__Firmicutes;c__Incertae_Sedis;o__DTU014;f__DTU014_unclassified;g__DTU014_unclassified;s__DTU014_unclassified
d__Bacteria;p__Proteobacteria;c__Gammaproteobacteria;o__Steroidobacterales;f__Steroidobacteraceae;g__Steroidobacteraceae_un
d__Bacteria;p__Cyanobacteria;c__Cyanobacteriia;o__Chloroplast;f__Chloroplast_unclassified;g__Chloroplast_unclassified;s__Ch
d__Bacteria;p__Cyanobacteria;c__Cyanobacteriia;o__Cyanobacteriales;f__Chroococcidiopsaceae;g__Chroococcidiopsaceae_unclassi
d__Bacteria;p__Firmicutes;c__Bacilli;o__Lactobacillales;f__Streptococcaceae;g__Streptococcus;s__Streptococcus_sp.

d__Bacteria;p__Chloroflexi;c__OLB14;o__OLB14_unclassified;f__OLB14_unclassified;g__OLB14_unclassified;s__OLB14_unclassified
d__Bacteria;p__Proteobacteria;c__Gammaproteobacteria;o__Burkholderiales;f__Burkholderiaceae;g__Ralstonia;s__Ralstonia_uncla
d__Bacteria;p__Firmicutes;c__Negativicutes;o__Acidaminococcales;f__Acidaminococcaceae;g__Phascolarctobacterium;s__Phascolar
d__Bacteria;p__Firmicutes;c__Bacilli;o__Lactobacillales;f__Streptococcaceae;g__Streptococcus;s__Streptococcus_thermophilus
d__Bacteria;p__Firmicutes;c__Bacilli;o__Thermoactinomycetales;f__Thermoactinomycetaceae;g__Kroppenstedtia;s__Kroppenstedtia
d__Bacteria;p__Firmicutes;c__Clostridia;o__Clostridiales;f__Clostridiaceae;g__Clostridium_sensu_stricto_1;s__Clostridium_se
d__Bacteria;p__Firmicutes;c__Bacilli;o__Bacillales;f__Bacillaceae;g__Bacillus;s__Bacillus_sp._17843
d__Bacteria;p__Cyanobacteria;c__Cyanobacteriia;o__Chloroplast;f__Chloroplast_unclassified;g__Chloroplast_unclassified;s__Ch
d__Bacteria;p__Proteobacteria;c__Gammaproteobacteria;o__Burkholderiales;f__Alcaligenaceae;g__Parapusillimonas;s__Parapusill
d__Bacteria;p__Bacteroidota;c__Bacteroidia;o__Bacteroidales;f__Rikenellaceae;g__Rikenellaceae_RC9_gut_group;s__Rikenellacea
d__Bacteria;p__Actinobacteriota;c__Actinobacteria;o__Actinomycetales;f__Actinomycetaceae;g__Actinomyces;s__Actinomyces_uncl
d__Bacteria;p__Actinobacteriota;c__Actinobacteria;o__Streptosporangiales;f__Thermomonosporaceae;g__Streptomyces]_thermoauto
d__Bacteria;p__Proteobacteria;c__Gammaproteobacteria;o__Enterobacterales;f__Erwiniaceae;g__Pantoea;s__Pantoea_agglomerans
d__Bacteria;p__Firmicutes;c__Bacilli;o__Lactobacillales;f__Streptococcaceae;g__Streptococcus;s__Streptococcus_thermophilus
d__Bacteria;p__Proteobacteria;c__Gammaproteobacteria;o__Burkholderiales;f__Burkholderiaceae;g__Burkholderia-Caballeronia-Pa
d__Bacteria;p__Proteobacteria;c__Alphaproteobacteria;o__Sphingomonadales;f__Sphingomonadaceae;g__Sphingopyxis;s__Sphingopyx
d__Bacteria;p__Proteobacteria;c__Gammaproteobacteria;o__Burkholderiales;f__Comamonadaceae;g__Variovorax;s__Variovorax_uncla
d__Bacteria;p__Proteobacteria;c__Gammaproteobacteria;o__Pseudomonadales;f__Moraxellaceae;g__Acinetobacter;s__Acinetobacter_
d__Bacteria;p__Proteobacteria;c__Gammaproteobacteria;o__Pseudomonadales;f__Pseudomonadaceae;g__Pseudomonas;s__Pseudomonas_r
d__Bacteria;p__Actinobacteriota;c__Actinobacteria;o__Micrococcales;f__Dermacoccaceae;g__Kytococcus;s__Kytococcus_unclassifi
d__Bacteria;p__Proteobacteria;c__Gammaproteobacteria;o__Xanthomonadales;f__Rhodanobacteraceae;g__Pseudofulvimonas;s__Pseudo
d__Bacteria;p__Proteobacteria;c__Alphaproteobacteria;o__Rhodobacterales;f__Rhodobacteraceae;g__Rhodobacter;s__uncultured_Rh
d__Bacteria;p__Cyanobacteria;c__Cyanobacteriia;o__Chloroplast;f__Chloroplast_unclassified;g__Chloroplast_unclassified;s__Ch
d__Bacteria;p__Proteobacteria;c__Gammaproteobacteria;o__Burkholderiales;f__Rhodocyclaceae;g__Dechlorosoma;s__Dechlorosoma_u
d__Bacteria;p__Proteobacteria;c__Betaproteobacteria;o__Burkholderiales;f__Comamonadaceae;g__Comamonas;s__uncultured_Comamon
d__Bacteria;p__Proteobacteria;c__Gammaproteobacteria;o__Pseudomonadales;f__Pseudomonadaceae;g__Pseudomonas;s__Pseudomonas_p
d__Bacteria;p__Cyanobacteria;c__Cyanobacteriia;o__Chloroplast;f__Chloroplast_unclassified;g__Chloroplast_unclassified;s__Ch
d__Bacteria;p__Firmicutes;c__Bacilli;o__Bacillales;f__Bacillaceae;g__Lysinibacillus;s__Lysinibacillus_sphaericus
d__Bacteria;p__Verrucomicrobiota;c__Verrucomicrobiae;o__Verrucomicrobiales;f__Akkermansiaceae;g__Akkermansia;s__Akkermansia
d__Bacteria;p__Proteobacteria;c__Gammaproteobacteria;o__Burkholderiales;f__Rhodocyclaceae;g__Dechlorosoma;s__Dechlorosoma_u
d__Bacteria;p__Bacteroidota;c__Bacteroidia;o__Bacteroidales;f__Prevotellaceae;g__Prevotella_9;s__Prevotella_9_unclassified
d__Bacteria;p__Fusobacteriota;c__Fusobacteriia;o__Fusobacteriales;f__Fusobacteriaceae;g__Cetobacterium;s__Cetobacterium_unc
d__Bacteria;p__Proteobacteria;c__Alphaproteobacteria;o__Alphaproteobacteria_unclassified;f__Alphaproteobacteria_unclassifie
d__Bacteria;p__Actinobacteriota;c__Actinobacteria;o__Propionibacteriales;f__Propionibacteriaceae;g__Cutibacterium;s__Cutiba
d__Bacteria;p__Proteobacteria;c__Alphaproteobacteria;o__Rhizobiales;f__Beijerinckiaceae;g__Methylobacterium-Methylorubrum;s
d__Bacteria;p__Proteobacteria;c__Gammaproteobacteria;o__Burkholderiales;f__Rhodocyclaceae;g__Dechlorosoma;s__Dechlorosoma_u
d__Bacteria;p__Proteobacteria;c__Gammaproteobacteria;o__Burkholderiales;f__Rhodocyclaceae;g__Dechlorosoma;s__Dechlorosoma_u

d__Bacteria;p__Firmicutes;c__Bacilli;o__Lactobacillales;f__Lactobacillaceae;g__Lactobacillus;s__Lactobacillus_oris
d__Bacteria;p__Firmicutes;c__Bacilli;o__Lactobacillales;f__Streptococcaceae;g__Streptococcus;s__Streptococcus_thermophilus
d__Bacteria;p__Bacteroidota;c__Bacteroidia;o__Chitinophagales;f__Chitinophagaceae;g__Haoranjiania;s__Haoranjiania_unclassif
d__Bacteria;p__Proteobacteria;c__Gammaproteobacteria;o__Pseudomonadales;f__Pseudomonadaceae;g__Pseudomonas;s__Pseudomonas_a
d__Bacteria;p__Firmicutes;c__Bacilli;o__Lactobacillales;f__Lactobacillaceae;g__Ligilactobacillus;s__Ligilactobacillus_uncla
d__Bacteria;p__Proteobacteria;c__Gammaproteobacteria;o__Enterobacterales;f__Erwiniaceae;g__Pantoea;s__Pantoea_agglomerans
d__Bacteria;p__Actinobacteriota;c__Actinobacteria;o__Micrococcales;f__Micrococcaceae;g__Rothia;s__Rothia_unclassified
d__Bacteria;p__Firmicutes;c__Clostridia;o__Clostridiales;f__Lachnospiraceae;g__Lachnospiraceae_unclassified;s__Lachnospirac
d__Bacteria;p__Proteobacteria;c__Alphaproteobacteria;o__Rhizobiales;f__Brucellaceae;g__Ochrobactrum;s__Ochrobactrum_sp._P46
d__Bacteria;p__Firmicutes;c__Bacilli;o__Lactobacillales;f__Streptococcaceae;g__Lactococcus;s__Lactococcus_garvieae
d__Bacteria;p__Proteobacteria;c__Betaproteobacteria;o__Burkholderiales;f__Burkholderiales_unclassified;g__Burkholderiales_u
d__Bacteria;p__Firmicutes;c__Clostridia;o__Peptostreptococcales-Tissierellales;f__Family_XI;g__Fenollaria;s__Fenollaria_unc
d__Bacteria;p__Firmicutes;c__Bacilli;o__Bacillales;f__Bacillaceae;g__Oceanobacillus;s__Oceanobacillus_unclassified
d__Bacteria;p__Cyanobacteria;c__Cyanobacteriia;o__Chloroplast;f__Chloroplast_unclassified;g__Chloroplast_unclassified;s__Ch
d__Bacteria;p__Cyanobacteria;c__Cyanobacteriia;o__Chloroplast;f__Chloroplast_unclassified;g__Chloroplast_unclassified;s__Ch
d__Bacteria;p__Proteobacteria;c__Gammaproteobacteria;o__Burkholderiales;f__Burkholderiaceae;g__Ralstonia;s__Ralstonia_uncla
d__Bacteria;p__Firmicutes;c__Clostridia;o__Clostridiales;f__Clostridiaceae;g__Clostridium_sensu_stricto_8;s__Clostridium_se
d__Bacteria;p__Proteobacteria;c__Gammaproteobacteria;o__Enterobacterales;f__Erwiniaceae;g__Pantoea;s__Pantoea_septica
d__Bacteria;p__Proteobacteria;c__Alphaproteobacteria;o__Rhizobiales;f__Xanthobacteraceae;g__Xanthobacter;s__uncultured_Xant
d__Bacteria;p__Cyanobacteria;c__Cyanobacteriia;o__Chloroplast;f__Chloroplast_unclassified;g__Chloroplast_unclassified;s__Ch
d__Bacteria;p__Proteobacteria;c__Gammaproteobacteria;o__Burkholderiales;f__Burkholderiaceae;g__Ralstonia;s__Ralstonia_uncla
d__Bacteria;p__Proteobacteria;c__Gammaproteobacteria;o__Burkholderiales;f__Oxalobacteraceae;g__Massilia;s__Massilia_unclass
d__Bacteria;p__Proteobacteria;c__Gammaproteobacteria;o__Burkholderiales;f__Burkholderiaceae;g__Pandoraea;s__Pandoraea_uncla
d__Bacteria;p__Proteobacteria;c__Gammaproteobacteria;o__Pseudomonadales;f__Halomonadaceae;g__Halomonas;s__Halomonas_unclass
d__Bacteria;p__Proteobacteria;c__Gammaproteobacteria;o__Burkholderiales;f__Comamonadaceae;g__Delftia;s__Delftia_unclassifie
d__Bacteria;p__Proteobacteria;c__Alphaproteobacteria;o__Sphingomonadales;f__Sphingomonadaceae;g__Sphingomonas;s__Sphingomon
d__Bacteria;p__Proteobacteria;c__Gammaproteobacteria;o__Pseudomonadales;f__Moraxellaceae;g__Acinetobacter;s__Acinetobacter_
d__Bacteria;p__Desulfobacterota;c__Desulfuromonadia;o__Geobacterales;f__Geobacteraceae;g__Geobacter;s__Geobacter_unclassifi
d__Bacteria;p__Actinobacteriota;c__Actinobacteria;o__Micrococcales;f__Micrococcaceae;g__Paenarthrobacter;s__Paenarthrobacte
d__Bacteria;p__Proteobacteria;c__Alphaproteobacteria;o__Caulobacterales;f__Caulobacteraceae;g__Brevundimonas;s__Brevundimon
d__Bacteria;p__Proteobacteria;c__Gammaproteobacteria;o__Burkholderiales;f__Rhodocyclaceae;g__Dechlorosoma;s__Dechlorosoma_u
d__Bacteria;p__Proteobacteria;c__Gammaproteobacteria;o__Burkholderiales;f__Alcaligenaceae;g__Achromobacter;s__Achromobacter
d__Bacteria;p__Proteobacteria;c__Gammaproteobacteria;o__Pseudomonadales;f__Moraxellaceae;g__Acinetobacter;s__Acinetobacter_
d__Bacteria;p__Firmicutes;c__Bacilli;o__Lactobacillales;f__Carnobacteriaceae;g__Atopostipes;s__uncultured_Atopostipes_sp.
d__Bacteria;p__Firmicutes;c__Bacilli;o__Bacillales;f__Bacillaceae;g__Bacillus;s__Bacillus_cereus
d__Bacteria;p__Cyanobacteria;c__Cyanobacteriia;o__Chloroplast;f__Chloroplast_unclassified;g__Chloroplast_unclassified;s__Ch
d__Bacteria;p__Firmicutes;c__Negativicutes;o__Acidaminococcales;f__Acidaminococcaceae;g__Phascolarctobacterium;s__unculture

d__Bacteria;p__Proteobacteria;c__Alphaproteobacteria;o__Acetobacterales;f__Acetobacteraceae;g__Acidisoma;s__Acidisoma_uncla
d__Bacteria;p__Bacteroidota;c__Bacteroidia;o__Flavobacteriales;f__Weeksellaceae;g__Chryseobacterium;s__Chryseobacterium_unc
d__Bacteria;p__Proteobacteria;c__Gammaproteobacteria;o__Burkholderiales;f__Rhodocyclaceae;g__Dechlorosoma;s__Dechlorosoma_u
d__Bacteria;p__Proteobacteria;c__Alphaproteobacteria;o__Sphingomonadales;f__Sphingomonadaceae;g__Novosphingobium;s__Novosph
d__Bacteria;p__Firmicutes;c__Clostridia;o__Clostridiales;f__Ruminococcaceae;g__Ruminococcaceae_unclassified;s__Ruminococcac
d__Bacteria;p__Myxococcota;c__Polyangia;o__Blfdi19;f__Blfdi19_unclassified;g__Blfdi19_unclassified;s__Blfdi19_unclassified
d__Bacteria;p__Firmicutes;c__Bacilli;o__Thermoactinomycetales;f__Thermoactinomycetaceae;g__Kroppenstedtia;s__Kroppenstedtia
d__Bacteria;p__Bacteroidota;c__Bacteroidia;o__Sphingobacteriales;f__Sphingobacteriaceae;g__Mucilaginibacter;s__Mucilaginiba
d__Bacteria;p__Firmicutes;c__Clostridia;o__Peptostreptococcales-Tissierellales;f__Peptostreptococcaceae;g__Peptostreptococc
d__Bacteria;p__Firmicutes;c__Bacilli;o__Bacillales;f__Bacillaceae;g__Calditerricola;s__Calditerricola_sp._D08p5
d__Bacteria;p__Proteobacteria;c__Alphaproteobacteria;o__Rickettsiales;f__Mitochondria;g__Mitochondria_unclassified;s__Mitoc
d__Bacteria;p__Cyanobacteria;c__Cyanobacteriia;o__Chloroplast;f__Chloroplast_unclassified;g__Chloroplast_unclassified;s__Ch
d__Bacteria;p__Proteobacteria;c__Gammaproteobacteria;o__Burkholderiales;f__Rhodocyclaceae;g__Dechlorosoma;s__Dechlorosoma_u
d__Bacteria;p__Firmicutes;c__Bacilli;o__Erysipelotrichales;f__Erysipelatoclostridiaceae;g__Erysipelatoclostridium;s__Erysip
d__Bacteria;p__Bacteroidota;c__Bacteroidia;o__Bacteroidales;f__Tannerellaceae;g__Parabacteroides;s__Parabacteroides_distaso
d__Bacteria;p__Firmicutes;c__Clostridia;o__Clostridiales;f__Lachnospiraceae;g__Lachnospiraceae_unclassified;s__Lachnospirac
d__Bacteria;p__Firmicutes;c__Clostridia;o__Lachnospirales;f__Lachnospiraceae;g__Lachnospiraceae_NK4A136_group;s__Lachnospir
d__Bacteria;p__Firmicutes;c__Bacilli;o__Lactobacillales;f__Streptococcaceae;g__Streptococcus;s__Streptococcus_cristatus
d__Bacteria;p__Proteobacteria;c__Gammaproteobacteria;o__Xanthomonadales;f__Xanthomonadaceae;g__Stenotrophomonas;s__Stenotro
d__Bacteria;p__Actinobacteriota;c__Acidimicrobiia;o__Actinomarinales;f__Actinomarinaceae;g__Candidatus_Actinomarina;s__Cand
d__Bacteria;p__Proteobacteria;c__Alphaproteobacteria;o__Rhizobiales;f__Rhizobiaceae;g__Rhizobium;s__uncultured_Rhizobium_sp
d__Bacteria;p__Firmicutes;c__Bacilli;o__Lactobacillales;f__Streptococcaceae;g__Streptococcus;s__uncultured_Streptococcus_sp
d__Bacteria;p__Firmicutes;c__Bacilli;o__Staphylococcales;f__Staphylococcaceae;g__Staphylococcus;s__Staphylococcus_unclassif
d__Bacteria;p__Bacteroidota;c__Bacteroidia;o__Bacteroidales;f__Rikenellaceae;g__Alistipes;s__Alistipes_ihumii
d__Bacteria;p__Firmicutes;c__Desulfotomaculia;o__Desulfotomaculales;f__Desulfotomaculaceae;g__Desulfotomaculum;s__Desulfoto
d__Bacteria;p__Proteobacteria;c__Gammaproteobacteria;o__Enterobacterales;f__Enterobacteriaceae;g__Escherichia;s__Escherichi
d__Bacteria;p__Firmicutes;c__Bacilli;o__Staphylococcales;f__Staphylococcaceae;g__Staphylococcus;s__Staphylococcus_unclassif
d__Bacteria;p__Bacteroidota;c__Bacteroidia;o__Flavobacteriales;f__Flavobacteriaceae;g__NS3a_marine_group;s__NS3a_marine_gro
d__Bacteria;p__Actinobacteriota;c__Actinobacteria;o__Micrococcales;f__Micrococcaceae;g__Rothia;s__Rothia_unclassified
d__Bacteria;p__Actinobacteriota;c__Thermoleophilia;o__Gaiellales;f__Gaiellales_unclassified;g__Gaiellales_unclassified;s__G
d__Bacteria;p__Bacteroidota;c__Bacteroidia;o__Cytophagales;f__Hymenobacteraceae;g__Hymenobacter;s__Hymenobacter_unclassifie
d__Bacteria;p__Bacteroidota;c__Bacteroidia;o__Cytophagales;f__Microscillaceae;g__Ohtaekwangia;s__Ohtaekwangia_unclassified
d__Bacteria;p__Firmicutes;c__Clostridia;o__Lachnospirales;f__Lachnospiraceae;g__Lachnospiraceae_NK4A136_group;s__Lachnospir
d__Bacteria;p__Proteobacteria;c__Alphaproteobacteria;o__Puniceispirillales;f__SAR116_clade;g__Candidatus_Puniceispirillum;s
d__Bacteria;p__Cyanobacteria;c__Cyanobacteriia;o__Chloroplast;f__Chloroplast_unclassified;g__Chloroplast_unclassified;s__Ch
d__Bacteria;p__Actinobacteriota;c__Actinobacteriota;o__Micrococcales;f__Microbacteriaceae;g__Microbacteriaceae_unclassified
d__Bacteria;p__Proteobacteria;c__Gammaproteobacteria;o__Pseudomonadales;f__Moraxellaceae;g__Acinetobacter;s__Acinetobacter_

d__Bacteria;p__Proteobacteria;c__Gammaproteobacteria;o__Burkholderiales;f__Burkholderiaceae;g__Burkholderia-Caballeronia-Pa
d__Bacteria;p__Verrucomicrobiota;c__Verrucomicrobiae;o__Chthoniobacterales;f__Chthoniobacteraceae;g__LD29;s__LD29_unclassif
d__Bacteria;p__Firmicutes;c__Clostridia;o__Lachnospirales;f__Lachnospiraceae;g__Anaerostipes;s__Anaerostipes_unclassified
d__Bacteria;p__Firmicutes;c__Negativicutes;o__Veillonellales-Selenomonadales;f__Selenomonadaceae;g__Megamonas;s__Megamonas_
d__Bacteria;p__Firmicutes;c__Clostridia;o__Clostridiales;f__Lachnospiraceae;g__Lachnospiraceae_unclassified;s__Lachnospirac
d__Bacteria;p__Actinobacteriota;c__Actinobacteria;o__Corynebacteriales;f__Corynebacteriaceae;g__Lawsonella;s__Lawsonella_un
d__Bacteria;p__Proteobacteria;c__Alphaproteobacteria;o__Caulobacterales;f__Caulobacteraceae;g__Brevundimonas;s__uncultured_
d__Bacteria;p__Firmicutes;c__Bacilli;o__Lactobacillales;f__Enterococcaceae;g__Enterococcus;s__Enterococcus_cecorum
d__Bacteria;p__Firmicutes;c__Bacilli;o__Staphylococcales;f__Staphylococcaceae;g__Staphylococcus;s__Staphylococcus_unclassif
d__Bacteria;p__Firmicutes;c__Bacilli;o__Staphylococcales;f__Staphylococcaceae;g__Staphylococcus;s__Staphylococcus_unclassif
d__Bacteria;p__Proteobacteria;c__Alphaproteobacteria;o__Rhodospirillales;f__Acetobacteraceae;g__Acetobacteraceae_unclassifi
d__Bacteria;p__Cyanobacteria;c__Cyanobacteriia;o__Chloroplast;f__Chloroplast_unclassified;g__Chloroplast_unclassified;s__Ch
d__Bacteria;p__Cyanobacteria;c__Cyanobacteriia;o__Chloroplast;f__Chloroplast_unclassified;g__Chloroplast_unclassified;s__Ch
d__Bacteria;p__Firmicutes;c__Bacilli;o__Lactobacillales;f__Streptococcaceae;g__Streptococcus;s__uncultured_Streptococcus_sp
d__Bacteria;p__Proteobacteria;c__Betaproteobacteria;o__Burkholderiales;f__Comamonadaceae;g__Comamonas;s__Comamonas_terrigen
d__Bacteria;p__Proteobacteria;c__Alphaproteobacteria;o__Sphingomonadales;f__Sphingomonadaceae;g__Sphingopyxis;s__Sphingopyx
d__Bacteria;p__Bacteroidota;c__Bacteroidia;o__Flavobacteriales;f__Flavobacteriaceae;g__NS5_marine_group;s__NS5_marine_group
d__Bacteria;p__Cyanobacteria;c__Cyanobacteriia;o__Chloroplast;f__Chloroplast_unclassified;g__Chloroplast_unclassified;s__Ch
d__Bacteria;p__Proteobacteria;c__Gammaproteobacteria;o__Pseudomonadales;f__Moraxellaceae;g__Acinetobacter;s__Acinetobacter_
d__Bacteria;p__Firmicutes;c__Clostridia;o__Oscillospirales;f__Ruminococcaceae;g__Faecalibacterium;s__Faecalibacterium_uncla
d__Bacteria;p__Firmicutes;c__Bacilli;o__Lactobacillales;f__Streptococcaceae;g__Streptococcus;s__uncultured_Streptococcus_sp
d__Bacteria;p__Proteobacteria;c__Alphaproteobacteria;o__Sphingomonadales;f__Sphingomonadaceae;g__Sphingomonas;s__Sphingomon
d__Bacteria;p__Firmicutes;c__Bacilli;o__Erysipelotrichales;f__Erysipelatoclostridiaceae;g__Erysipelatoclostridium;s__Erysip
d__Bacteria;p__Cyanobacteria;c__Cyanobacteriia;o__Chloroplast;f__Chloroplast_unclassified;g__Chloroplast_unclassified;s__Ch
d__Bacteria;p__Firmicutes;c__Bacilli;o__Bacillales;f__Bacillaceae;g__Aeribacillus;s__Aeribacillus_unclassified
d__Bacteria;p__Proteobacteria;c__Gammaproteobacteria;o__Burkholderiales;f__Oxalobacteraceae;g__Massilia;s__Massilia_unclass
d__Bacteria;p__Proteobacteria;c__Gammaproteobacteria;o__Burkholderiales;f__Burkholderiaceae;g__Burkholderia-Caballeronia-Pa
d__Bacteria;p__Proteobacteria;c__Gammaproteobacteria;o__Enterobacterales;f__Erwiniaceae;g__Pantoea;s__Pantoea_sp.
d__Bacteria;p__Firmicutes;c__Clostridia;o__Lachnospirales;f__Lachnospiraceae;g__Ruminococcus]_torques_group;s__Ruminococcus
d__Bacteria;p__Proteobacteria;c__Gammaproteobacteria;o__Pseudomonadales;f__Moraxellaceae;g__Acinetobacter;s__Acinetobacter_
d__Bacteria;p__Firmicutes;c__Clostridia;o__Lachnospirales;f__Lachnospiraceae;g__Eubacterium]_hallii_group;s__Eubacterium]_h
d__Bacteria;p__Proteobacteria;c__Gammaproteobacteria;o__Xanthomonadales;f__Xanthomonadaceae;g__Stenotrophomonas;s__Stenotro
d__Bacteria;p__Proteobacteria;c__Gammaproteobacteria;o__Burkholderiales;f__Comamonadaceae;g__Variovorax;s__Variovorax_uncla
d__Bacteria;p__Actinobacteriota;c__Actinobacteria;o__Micrococcales;f__Micrococcaceae;g__Rothia;s__Rothia_unclassified
d__Bacteria;p__Cyanobacteria;c__Cyanobacteriia;o__Synechococcales;f__Cyanobiaceae;g__Cyanobium_PCC-6307;s__Cyanobium_PCC-63
d__Bacteria;p__Firmicutes;c__Bacilli;o__Thermoactinomycetales;f__Thermoactinomycetaceae;g__Kroppenstedtia;s__Kroppenstedtia
d__Bacteria;p__Proteobacteria;c__Gammaproteobacteria;o__Enterobacterales;f__Enterobacteriaceae;g__Escherichia-Shigella;s__E

d__Bacteria;p__Proteobacteria;c__Gammaproteobacteria;o__Pseudomonadales;f__Moraxellaceae;g__Acinetobacter;s__Acinetobacter_
d__Bacteria;p__Proteobacteria;c__Betaproteobacteria;o__Burkholderiales;f__Comamonadaceae;g__Acidovorax;s__Acidovorax_wauter
d__Bacteria;p__Firmicutes;c__Erysipelotrichia;o__Erysipelotrichales;f__Erysipelotrichaceae;g__Erysipelatoclostridium;s__Ery
d__Bacteria;p__Cyanobacteria;c__Cyanobacteriia;o__Leptolyngbyales;f__Leptolyngbyaceae;g__Leptolyngbya_Es-Yyy1000;s__Leptoly
d__Bacteria;p__Firmicutes;c__Clostridia;o__Lachnospirales;f__Lachnospiraceae;g__Blautia;s__Blautia_unclassified
d__Bacteria;p__Proteobacteria;c__Gammaproteobacteria;o__Competibacterales;f__Competibacteraceae;g__Candidatus_Contendobacte
d__Bacteria;p__Desulfobacterota;c__Desulfovibrionia;o__Desulfovibrionales;f__Desulfovibrionaceae;g__Desulfovibrio;s__Desulf
d__Bacteria;p__Proteobacteria;c__Alphaproteobacteria;o__Sphingomonadales;f__Sphingomonadaceae;g__Sphingomonas;s__Sphingomon
d__Bacteria;p__Firmicutes;c__Bacilli;o__Staphylococcales;f__Staphylococcaceae;g__Staphylococcus;s__Staphylococcus_unclassif
d__Bacteria;p__Actinobacteriota;c__Actinobacteria;o__Corynebacteriales;f__Corynebacteriaceae;g__Corynebacterium;s__Coryneba
d__Bacteria;p__Proteobacteria;c__Gammaproteobacteria;o__Enterobacterales;f__Morganellaceae;g__Proteus;s__Proteus_mirabilis
d__Bacteria;p__Firmicutes;c__Bacilli;o__Thermoactinomycetales;f__Thermoactinomycetaceae;g__Thermoactinomyces;s__Thermoactin
d__Bacteria;p__Firmicutes;c__Clostridia;o__Lachnospirales;f__Lachnospiraceae;g__Anaerostipes;s__Anaerostipes_unclassified
d__Bacteria;p__Bdellovibrionota;c__Bdellovibrionia;o__Bdellovibrionales;f__Bdellovibrionaceae;g__Bdellovibrio;s__Bdellovibr
d__Bacteria;p__Firmicutes;c__Bacilli;o__Lactobacillales;f__Lactobacillaceae;g__Lactobacillus;s__Lactobacillus_iners
d__Bacteria;p__Proteobacteria;c__Alphaproteobacteria;o__Rhodobacterales;f__Rhodobacteraceae;g__Rubellimicrobium;s__Rubellim
d__Bacteria;p__Firmicutes;c__Bacilli;o__Lactobacillales;f__Streptococcaceae;g__Streptococcus;s__Streptococcus_sp.
d__Bacteria;p__Proteobacteria;c__Gammaproteobacteria;o__Burkholderiales;f__Comamonadaceae;g__Pelomonas;s__Pelomonas_unclass
d__Bacteria;p__Actinobacteriota;c__Actinobacteria;o__Micrococcales;f__Micrococcaceae;g__Kocuria;s__Kocuria_unclassified
d__Bacteria;p__Firmicutes;c__Clostridia;o__Clostridiales;f__Lachnospiraceae;g__Lachnospiraceae_unclassified;s__Lachnospirac
d__Bacteria;p__Bacteroidota;c__Bacteroidia;o__Bacteroidales;f__Bacteroidaceae;g__Bacteroides;s__Bacteroides_sartorii
d__Bacteria;p__Bacteroidota;c__Bacteroidia;o__Bacteroidales;f__Bacteroidaceae;g__Bacteroides;s__Bacteroides_unclassified
d__Bacteria;p__Actinobacteriota;c__Actinobacteriota;o__Micrococcales;f__Cellulomonadaceae;g__Cellulomonas;s__Cellulomonas_s
d__Bacteria;p__Firmicutes;c__Bacilli;o__Staphylococcales;f__Staphylococcaceae;g__Staphylococcus;s__Staphylococcus_unclassif
d__Bacteria;p__Actinobacteriota;c__Actinobacteria;o__Micrococcales;f__Micrococcaceae;g__Rothia;s__Rothia_unclassified
d__Bacteria;p__Firmicutes;c__Clostridia;o__Clostridiales;f__Eubacteriaceae;g__Eubacterium;s__Eubacterium_sp._Marseille-P320
d__Bacteria;p__Proteobacteria;c__Alphaproteobacteria;o__Sphingomonadales;f__Sphingomonadaceae;g__Sphingosinicella;s__Sphing
d__Bacteria;p__Proteobacteria;c__Alphaproteobacteria;o__Rhizobiales;f__Rhizobiaceae;g__Allorhizobium-Neorhizobium-Pararhizo
d__Bacteria;p__Firmicutes;c__Thermincolia;o__Carboxydocellales;f__Carboxydocellaceae;g__Carboxydocella;s__Carboxydocella_un
d__Bacteria;p__Firmicutes;c__Bacilli;o__Bacillales;f__Planococcaceae;g__Lysinibacillus;s__Lysinibacillus_unclassified
d__Bacteria;p__Acidobacteriota;c__Acidobacteriae;o__Acidobacteriales;f__Acidobacteriaceae_(Subgroup_1);g__Edaphobacter;s__E
d__Bacteria;p__Proteobacteria;c__Gammaproteobacteria;o__Burkholderiales;f__Alcaligenaceae;g__Alcaligenes;s__Alcaligenes_unc
d__Bacteria;p__Proteobacteria;c__Gammaproteobacteria;o__Burkholderiales;f__Burkholderiaceae;g__Burkholderia-Caballeronia-Pa
d__Bacteria;p__Actinobacteriota;c__Coriobacteriia;o__Coriobacteriales;f__Atopobiaceae;g__Coriobacteriaceae_UCG-002;s__Corio
d__Bacteria;p__Proteobacteria;c__Gammaproteobacteria;o__Enterobacterales;f__Enterobacteriaceae;g__Raoultella;s__Raoultella_
d__Bacteria;p__Actinobacteriota;c__Actinobacteria;o__Corynebacteriales;f__Corynebacteriaceae;g__Lawsonella;s__Lawsonella_un
d__Bacteria;p__Proteobacteria;c__Gammaproteobacteria;o__Pseudomonadales;f__Pseudomonadaceae;g__Pseudomonas;s__Pseudomonas_l

```
d__Bacteria;p__Firmicutes;c__Clostridia;o__Peptostreptococcales-Tissierellales;f__Anaerovoracaceae;g__Family_XIII_AD3011_gr
d__Bacteria;p__Actinobacteriota;c__Actinobacteria;o__Corynebacteriales;f__Mycobacteriaceae;g__Mycobacterium;s__Mycobacteriu
d__Bacteria;p__Proteobacteria;c__Alphaproteobacteria;o__Acetobacterales;f__Acetobacteraceae;g__Acidisoma;s__Acidisoma_uncla
d__Bacteria;p__Proteobacteria;c__Gammaproteobacteria;o__Xanthomonadales;f__Xanthomonadaceae;g__Pseudoxanthomonas;s__Pseudox
d__Bacteria;p__Proteobacteria;c__Alphaproteobacteria;o__Sphingomonadales;f__Sphingomonadaceae;g__Sphingomonas;s__Sphingomon
d__Bacteria;p__Firmicutes;c__Clostridia;o__Clostridiales;f__Lachnospiraceae;g__Lachnospiraceae_unclassified;s__Lachnospirac
d__Bacteria;p__Proteobacteria;c__Betaproteobacteria;o__Burkholderiales;f__Comamonadaceae;g__Comamonas;s__Comamonas_terrigen
d__Bacteria;p__Proteobacteria;c__Alphaproteobacteria;o__Rhizobiales;f__Rhizobiaceae;g__Allorhizobium-Neorhizobium-Pararhizo
d__Bacteria;p__Actinobacteriota;c__Actinobacteria;o__Micrococcales;f__Micrococcaceae;g__Rothia;s__Rothia_unclassified
d__Bacteria;p__Proteobacteria;c__Alphaproteobacteria;o__Sphingomonadales;f__Sphingomonadaceae;g__Parasphingopyxis;s__Parasp
d__Bacteria;p__Firmicutes;c__Clostridia;o__Oscillospirales;f__Ruminococcaceae;g__Faecalibacterium;s__Faecalibacterium_uncla
d__Bacteria;p__Actinobacteriota;c__Actinobacteria;o__Propionibacteriales;f__Nocardioidaceae;g__Nocardioides;s__Nocardioides
d__Bacteria;p__Firmicutes;c__Clostridia;o__Lachnospirales;f__Lachnospiraceae;g__Blautia;s__Blautia_unclassified
d__Bacteria;p__Firmicutes;c__Bacilli;o__Lactobacillales;f__Enterococcaceae;g__Enterococcus;s__Enterococcus_faecium
d__Bacteria;p__Proteobacteria;c__Alphaproteobacteria;o__Sphingomonadales;f__Sphingomonadaceae;g__Blastomonas;s__Blastomonas
d__Bacteria;p__Firmicutes;c__Bacilli;o__Lactobacillales;f__Lactobacillaceae;g__Weissella;s__Weissella_unclassified
d__Bacteria;p__Proteobacteria;c__Gammaproteobacteria;o__Pseudomonadales;f__Moraxellaceae;g__Acinetobacter;s__uncultured_Aci
d__Bacteria;p__Proteobacteria;c__Betaproteobacteria;o__Burkholderiales;f__Comamonadaceae;g__Comamonas;s__Comamonas_terrigen
d__Bacteria;p__Firmicutes;c__Clostridia;o__Clostridiales;f__Lachnospiraceae;g__Lachnospiraceae_unclassified;s__Lachnospirac
d__Bacteria;p__Firmicutes;c__Clostridia;o__Clostridiales;f__Lachnospiraceae;g__Lachnospiraceae_unclassified;s__Lachnospirac
d__Bacteria;p__Proteobacteria;c__Betaproteobacteria;o__Rhodocyclales;f__Azonexaceae;g__Dechloromonas;s__uncultured_Dechloro
d__Bacteria;p__Bacteroidota;c__Bacteroidia;o__Flavobacteriales;f__Flavobacteriaceae;g__Flavobacterium;s__Flavobacterium_unc
d__Bacteria;p__Proteobacteria;c__Gammaproteobacteria;o__Burkholderiales;f__Neisseriaceae;g__Neisseriaceae_unclassified;s__N
d__Bacteria;p__Cyanobacteria;c__Cyanobacteriia;o__Chloroplast;f__Chloroplast_unclassified;g__Chloroplast_unclassified;s__Ch
d__Bacteria;p__Proteobacteria;c__Alphaproteobacteria;o__Sphingomonadales;f__Sphingomonadaceae;g__Sphingomonas;s__Sphingomon
d__Bacteria;p__Firmicutes;c__Bacilli;o__Bacillales;f__Bacillaceae;g__Bacillus;s__Bacillus_unclassified
d__Bacteria;p__Proteobacteria;c__Gammaproteobacteria;o__Pseudomonadales;f__Moraxellaceae;g__Acinetobacter;s__Acinetobacter_
d__Bacteria;p__Firmicutes;c__Clostridia;o__Clostridiales;f__Ruminococcaceae;g__Ruminococcus;s__uncultured_Ruminococcus_sp.
d__Bacteria;p__Firmicutes;c__Clostridia;o__Oscillospirales;f__Ruminococcaceae;g__Incertae_Sedis;s__Incertae_Sedis_unclassif
d__Bacteria;p__Acidobacteriota;c__Acidobacteriae;o__Acidobacteriales;f__Koribacteraceae;g__Candidatus_Koribacter;s__Candida
d__Bacteria;p__Proteobacteria;c__Gammaproteobacteria;o__Burkholderiales;f__Comamonadaceae;g__Polaromonas;s__Polaromonas_unc
d__Bacteria;p__Proteobacteria;c__Gammaproteobacteria;o__Burkholderiales;f__Rhodocyclaceae;g__Thauera;s__Thauera_unclassifie
d__Bacteria;p__Actinobacteriota;c__Actinobacteria;o__Micrococcales;f__Micrococcaceae;g__Rothia;s__Rothia_unclassified
d__Bacteria;p__Firmicutes;c__Bacilli;o__Lactobacillales;f__Streptococcaceae;g__Streptococcus;s__Streptococcus_sp.
d__Bacteria;p__Proteobacteria;c__Gammaproteobacteria;o__Xanthomonadales;f__Xanthomonadaceae;g__Stenotrophomonas;s__Stenotro
d__Bacteria;p__Firmicutes;c__Bacilli;o__Staphylococcales;f__Staphylococcaceae;g__Staphylococcus;s__Staphylococcus_unclassif
d__Bacteria;p__Actinobacteriota;c__Actinobacteria;o__Bifidobacteriales;f__Bifidobacteriaceae;g__Bifidobacterium;s__Bifidoba
```

d__Bacteria;p__Proteobacteria;c__Gammaproteobacteria;o__Xanthomonadales;f__Xanthomonadaceae;g__Stenotrophomonas;s__Stenotro
d__Bacteria;p__Proteobacteria;c__Gammaproteobacteria;o__Burkholderiales;f__Rhodocyclaceae;g__Dechlorosoma;s__Dechlorosoma_u
d__Bacteria;p__Firmicutes;c__Clostridia;o__Clostridiales;f__Lachnospiraceae;g__Blautia;s__uncultured_Blautia_sp.
d__Bacteria;p__Actinobacteriota;c__Actinobacteria;o__Micrococcales;f__Microbacteriaceae;g__Microbacterium;s__Microbacterium
d__Bacteria;p__Actinobacteriota;c__Actinobacteria;o__Micrococcales;f__Micrococcaceae;g__Rothia;s__Rothia_unclassified
d__Bacteria;p__Firmicutes;c__Bacilli;o__Brevibacillales;f__Brevibacillaceae;g__Brevibacillus;s__Brevibacillus_unclassified
d__Bacteria;p__Firmicutes;c__Clostridia;o__Clostridiales;f__Clostridiaceae;g__Clostridium_sensu_stricto_1;s__Clostridium_se
d__Bacteria;p__Firmicutes;c__Clostridia;o__Clostridia_UCG-014;f__Clostridia_UCG-014_unclassified;g__Clostridia_UCG-014_uncl
d__Bacteria;p__Bacteroidota;c__Bacteroidia;o__Sphingobacteriales;f__Sphingobacteriaceae;g__Solitalea;s__Solitalea_unclassif
d__Bacteria;p__Cyanobacteria;c__Cyanobacteriia;o__Chloroplast;f__Chloroplast_unclassified;g__Chloroplast_unclassified;s__Ch
d__Bacteria;p__Actinobacteriota;c__Actinobacteria;o__Micrococcales;f__Micrococcaceae;g__Rothia;s__Rothia_unclassified
d__Bacteria;p__Proteobacteria;c__Gammaproteobacteria;o__Burkholderiales;f__Burkholderiaceae;g__Burkholderia-Caballeronia-Pa
d__Bacteria;p__Firmicutes;c__Bacilli;o__Brevibacillales;f__Brevibacillaceae;g__Brevibacillus;s__Brevibacillus_unclassified
d__Bacteria;p__Proteobacteria;c__Gammaproteobacteria;o__Burkholderiales;f__Comamonadaceae;g__Delftia;s__Delftia_unclassifie
d__Bacteria;p__Proteobacteria;c__Alphaproteobacteria;o__Caulobacterales;f__Caulobacteraceae;g__Brevundimonas;s__Brevundimon
d__Bacteria;p__Cyanobacteria;c__Cyanobacteriia;o__Synechococcales;f__Cyanobiaceae;g__Synechococcus_CC9902;s__Synechococcus_
d__Bacteria;p__Firmicutes;c__Clostridia;o__Oscillospirales;f__Ruminococcaceae;g__Negativibacillus;s__Negativibacillus_uncla
d__Bacteria;p__Proteobacteria;c__Gammaproteobacteria;o__Xanthomonadales;f__Rhodanobacteraceae;g__Dyella;s__Dyella_sp.
d__Bacteria;p__Firmicutes;c__Bacilli;o__Lactobacillales;f__Lactobacillaceae;g__HT002;s__HT002_unclassified
d__Bacteria;p__Firmicutes;c__Bacilli;o__Bacillales;f__Bacillaceae;g__Geobacillus;s__Geobacillus_unclassified
d__Bacteria;p__Actinobacteriota;c__Actinobacteria;o__Propionibacteriales;f__Nocardioidaceae;g__Nocardioides;s__Nocardioides
d__Bacteria;p__Proteobacteria;c__Alphaproteobacteria;o__Sphingomonadales;f__Sphingomonadaceae;g__Sphingomonas;s__uncultured
d__Bacteria;p__Proteobacteria;c__Alphaproteobacteria;o__Sphingomonadales;f__Sphingomonadaceae;g__Sphingopyxis;s__Sphingopyx
d__Bacteria;p__Proteobacteria;c__Alphaproteobacteria;o__Caulobacterales;f__Caulobacteraceae;g__Brevundimonas;s__Brevundimon
d__Bacteria;p__Proteobacteria;c__Gammaproteobacteria;o__Pseudomonadales;f__Moraxellaceae;g__Acinetobacter;s__Acinetobacter_
d__Bacteria;p__Proteobacteria;c__Gammaproteobacteria;o__Xanthomonadales;f__Xanthomonadaceae;g__Stenotrophomonas;s__Stenotro
d__Bacteria;p__Bacteroidota;c__Bacteroidia;o__Flavobacteriales;f__Flavobacteriaceae;g__Ulvibacter;s__Ulvibacter_unclassifie
d__Bacteria;p__Firmicutes;c__Clostridia;o__Clostridiales;f__Ruminococcaceae;g__Ruminococcaceae_unclassified;s__Ruminococcac
d__Bacteria;p__Firmicutes;c__Firmicutes_unclassified;o__Firmicutes_unclassified;f__Firmicutes_unclassified;g__Firmicutes_un
d__Bacteria;p__Proteobacteria;c__Gammaproteobacteria;o__Pseudomonadales;f__Moraxellaceae;g__Psychrobacter;s__Psychrobacter_
d__Bacteria;p__Proteobacteria;c__Gammaproteobacteria;o__Pseudomonadales;f__Moraxellaceae;g__Acinetobacter;s__uncultured_Aci
d__Bacteria;p__Firmicutes;c__Bacilli;o__Bacillales;f__Bacillaceae;g__Geobacillus;s__Geobacillus_unclassified
d__Bacteria;p__Proteobacteria;c__Gammaproteobacteria;o__Burkholderiales;f__Oxalobacteraceae;g__Janthinobacterium;s__Janthin
d__Bacteria;p__Proteobacteria;c__Alphaproteobacteria;o__Rhizobiales;f__Rhizobiaceae;g__Rhizobium;s__uncultured_Rhizobium_sp
d__Bacteria;p__Bacteroidota;c__Bacteroidia;o__Bacteroidales;f__Bacteroidaceae;g__Bacteroides;s__Bacteroides_sartorii
d__Bacteria;p__Cyanobacteria;c__Cyanobacteriia;o__Chloroplast;f__Chloroplast_unclassified;g__Chloroplast_unclassified;s__Ch
d__Bacteria;p__Firmicutes;c__Bacilli;o__Lactobacillales;f__Streptococcaceae;g__Streptococcus;s__uncultured_Streptococcus_sp

d__Bacteria;p__Actinobacteriota;c__Actinobacteriota;o__Micromonosporales;f__Micromonosporaceae;g__Micromonospora;s__Micromo
d__Bacteria;p__Bacteroidota;c__Bacteroidia;o__Bacteroidales;f__Muribaculaceae;g__Muribaculaceae_unclassified;s__Muribaculac
d__Bacteria;p__Bacteroidota;c__Bacteroidia;o__Chitinophagales;f__Saprospiraceae;g__Rubidimonas;s__Rubidimonas_unclassified
d__Bacteria;p__Bacteroidota;c__Bacteroidia;o__Cytophagales;f__Cyclobacteriaceae;g__Algoriphagus;s__Algoriphagus_unclassifie
d__Bacteria;p__Proteobacteria;c__Gammaproteobacteria;o__Enterobacterales;f__Erwiniaceae;g__Pantoea;s__Pantoea_sp._S3
d__Bacteria;p__Proteobacteria;c__Alphaproteobacteria;o__Alphaproteobacteria_unclassified;f__Alphaproteobacteria_unclassifie
d__Bacteria;p__Proteobacteria;c__Gammaproteobacteria;o__Burkholderiales;f__Burkholderiaceae;g__Ralstonia;s__Ralstonia_uncla
d__Bacteria;p__Deinococcota;c__Deinococci;o__Deinococcales;f__Deinococcaceae;g__Deinococcus;s__Deinococcus_unclassified
d__Bacteria;p__Proteobacteria;c__Alphaproteobacteria;o__Rhizobiales;f__Beijerinckiaceae;g__Methylobacterium-Methylorubrum;s
d__Bacteria;p__Proteobacteria;c__Gammaproteobacteria;o__Xanthomonadales;f__Xanthomonadaceae;g__Stenotrophomonas;s__Stenotro
d__Bacteria;p__Firmicutes;c__Clostridia;o__Clostridiales;f__Ruminococcaceae;g__Ruminococcaceae_unclassified;s__Ruminococcac
d__Bacteria;p__Proteobacteria;c__Gammaproteobacteria;o__Xanthomonadales;f__Xanthomonadaceae;g__Stenotrophomonas;s__Stenotro
d__Bacteria;p__Proteobacteria;c__Gammaproteobacteria;o__Xanthomonadales;f__Xanthomonadaceae;g__Stenotrophomonas;s__Stenotro
d__Bacteria;p__Proteobacteria;c__Gammaproteobacteria;o__Pseudomonadales;f__Pseudomonadaceae;g__Pseudomonas;s__Pseudomonas_p
d__Bacteria;p__Bacteroidota;c__Bacteroidia;o__Flavobacteriales;f__Flavobacteriaceae;g__NS5_marine_group;s__NS5_marine_group
d__Bacteria;p__Firmicutes;c__Thermoanaerobacteria;o__Thermoanaerobacterales;f__Thermoanaerobacteraceae;g__Brockia;s__Brocki
d__Bacteria;p__Proteobacteria;c__Alphaproteobacteria;o__Rhizobiales;f__Xanthobacteraceae;g__Rhodopseudomonas;s__Rhodopseudo
d__Bacteria;p__Proteobacteria;c__Gammaproteobacteria;o__Enterobacterales;f__Enterobacteriaceae;g__Klebsiella;s__Klebsiella_
d__Bacteria;p__Proteobacteria;c__Gammaproteobacteria;o__Pseudomonadales;f__Moraxellaceae;g__Acinetobacter;s__Acinetobacter_
d__Bacteria;p__Campylobacterota;c__Campylobacteria;o__Campylobacterales;f__Helicobacteraceae;g__Helicobacter;s__Helicobacte
d__Bacteria;p__Proteobacteria;c__Gammaproteobacteria;o__Enterobacterales;f__Enterobacteriaceae;g__Enterobacter;s__Enterobac
d__Bacteria;p__Bacteroidota;c__Bacteroidia;o__Chitinophagales;f__Chitinophagaceae;g__Taibaiella;s__Taibaiella_unclassified
d__Bacteria;p__Bdellovibrionota;c__Bdellovibrionia;o__Bacteriovoracales;f__Bacteriovoracaceae;g__Peredibacter;s__Peredibact
d__Bacteria;p__Firmicutes;c__Clostridia;o__Lachnospirales;f__Lachnospiraceae;g__Blautia;s__Blautia_unclassified
d__Bacteria;p__Firmicutes;c__Clostridia;o__Peptococcales;f__Peptococcaceae;g__Peptococcus;s__Peptococcus_unclassified
d__Bacteria;p__Firmicutes;c__Clostridia;o__Thermincolales;f__Thermincolaceae;g__Thermincola;s__Thermincola_unclassified
d__Bacteria;p__Actinobacteriota;c__Actinobacteria;o__Corynebacteriales;f__Corynebacteriaceae;g__Corynebacterium;s__Coryneba
d__Bacteria;p__Firmicutes;c__Clostridia;o__Clostridiales;f__Lachnospiraceae;g__Anaerostipes;s__Anaerostipes_sp.
d__Bacteria;p__Proteobacteria;c__Alphaproteobacteria;o__Sphingomonadales;f__Sphingomonadaceae;g__Novosphingobium;s__Novosph
d__Bacteria;p__Actinobacteriota;c__Actinobacteria;o__Corynebacteriales;f__Corynebacteriaceae;g__Corynebacterium;s__Coryneba
d__Bacteria;p__Proteobacteria;c__Gammaproteobacteria;o__Burkholderiales;f__Oxalobacteraceae;g__Massilia;s__Massilia_unclass
d__Bacteria;p__Proteobacteria;c__Gammaproteobacteria;o__Pseudomonadales;f__Moraxellaceae;g__Acinetobacter;s__Acinetobacter_
d__Bacteria;p__Firmicutes;c__Clostridia;o__Lachnospirales;f__Lachnospiraceae;g__Lachnospiraceae_NK4A136_group;s__Lachnospir
d__Bacteria;p__Proteobacteria;c__Alphaproteobacteria;o__Rhodobacterales;f__Rhodobacteraceae;g__Paracoccus;s__Paracoccus_sp.
d__Bacteria;p__Firmicutes;c__Bacilli;o__Lactobacillales;f__Lactobacillaceae;g__Leuconostoc;s__Leuconostoc_unclassified
d__Bacteria;p__Firmicutes;c__Bacilli;o__Bacillales;f__Bacillaceae;g__Geobacillus;s__Geobacillus_thermodenitrificans
d__Bacteria;p__Cyanobacteria;c__Cyanobacteriia;o__Chloroplast;f__Chloroplast_unclassified;g__Chloroplast_unclassified;s__Ch

```
d__Bacteria;p__Actinobacteriota;c__Actinobacteria;o__Pseudonocardiales;f__Pseudonocardiaceae;g__Lechevalieria;s__Lechevalie
d__Bacteria;p__Proteobacteria;c__Alphaproteobacteria;o__Rhizobiales;f__Beijerinckiaceae;g__Methylobacterium-Methylorubrum;s
d__Bacteria;p__Proteobacteria;c__Gammaproteobacteria;o__Pseudomonadales;f__Moraxellaceae;g__Acinetobacter;s__Acinetobacter_
d__Bacteria;p__Firmicutes;c__Clostridia;o__Peptostreptococcales-Tissierellales;f__Peptostreptococcaceae;g__Intestinibacter;
d__Bacteria;p__Proteobacteria;c__Betaproteobacteria;o__Burkholderiales;f__Comamonadaceae;g__Pelomonas;s__Pelomonas_puraquae
d__Bacteria;p__Cyanobacteria;c__Cyanobacteriia;o__Chloroplast;f__Chloroplast_unclassified;g__Chloroplast_unclassified;s__Ch
d__Bacteria;p__Bacteroidota;c__Bacteroidia;o__Bacteroidales;f__Muribaculaceae;g__Muribaculaceae_unclassified;s__Muribaculac
d__Bacteria;p__Proteobacteria;c__Gammaproteobacteria;o__Burkholderiales;f__Burkholderiaceae;g__Pandoraea;s__Pandoraea_uncla
d__Bacteria;p__Cyanobacteria;c__Cyanobacteriia;o__Chloroplast;f__Chloroplast_unclassified;g__Chloroplast_unclassified;s__Ch
d__Bacteria;p__Actinobacteriota;c__Thermoleophilia;o__Gaiellales;f__Gaiellaceae;g__Gaiella;s__Gaiella_unclassified
d__Bacteria;p__Bacteroidota;c__Bacteroidia;o__Sphingobacteriales;f__Sphingobacteriaceae;g__Pedobacter;s__Pedobacter_unclass
d__Bacteria;p__Firmicutes;c__Bacilli;o__Lactobacillales;f__Streptococcaceae;g__Streptococcus;s__Streptococcus_unclassified
d__Bacteria;p__Proteobacteria;c__Gammaproteobacteria;o__Burkholderiales;f__Rhodocyclaceae;g__Dechlorosoma;s__Dechlorosoma_u
d__Bacteria;p__Proteobacteria;c__Betaproteobacteria;o__Burkholderiales;f__Comamonadaceae;g__Comamonas;s__uncultured_Comamon
d__Bacteria;p__Firmicutes;c__Clostridia;o__Lachnospirales;f__Lachnospiraceae;g__Blautia;s__Blautia_unclassified
d__Bacteria;p__Firmicutes;c__Clostridia;o__Lachnospirales;f__Lachnospiraceae;g__Blautia;s__Blautia_unclassified
d__Bacteria;p__Firmicutes;c__Desulfotomaculia;o__Desulfotomaculales;f__Desulfotomaculaceae;g__Desulfotomaculum;s__Desulfoto
d__Bacteria;p__Planctomycetota;c__Planctomycetes;o__Planctomycetales;f__Rubinisphaeraceae;g__SH-PL14;s__SH-PL14_unclassifie
d__Bacteria;p__Firmicutes;c__Clostridia;o__Peptococcales;f__Peptococcaceae;g__Peptococcus;s__Peptococcus_unclassified
d__Bacteria;p__Proteobacteria;c__Alphaproteobacteria;o__Rhizobiales;f__Bradyrhizobiaceae;g__Bradyrhizobium;s__uncultured_Br
d__Bacteria;p__Proteobacteria;c__Alphaproteobacteria;o__Rhizobiales;f__Beijerinckiaceae;g__Methylobacterium-Methylorubrum;s
d__Bacteria;p__Firmicutes;c__Negativicutes;o__Veillonellales-Selenomonadales;f__Veillonellaceae;g__Megasphaera;s__Megasphae
d__Bacteria;p__Proteobacteria;c__Gammaproteobacteria;o__Xanthomonadales;f__Xanthomonadaceae;g__Stenotrophomonas;s__Stenotro
d__Bacteria;p__Proteobacteria;c__Gammaproteobacteria;o__Burkholderiales;f__Comamonadaceae;g__Delftia;s__Delftia_unclassifie
d__Bacteria;p__Proteobacteria;c__Betaproteobacteria;o__Burkholderiales;f__Comamonadaceae;g__Comamonas;s__Comamonas_sp._SMCC
d__Bacteria;p__Firmicutes;c__Clostridia;o__Clostridiales;f__Lachnospiraceae;g__Lachnospiraceae_unclassified;s__Lachnospirac
d__Bacteria;p__Firmicutes;c__Clostridia;o__Clostridiales;f__Clostridiaceae;g__Clostridium_sensu_stricto_1;s__Clostridium_se
d__Bacteria;p__Proteobacteria;c__Gammaproteobacteria;o__Burkholderiales;f__Burkholderiaceae;g__Burkholderia-Caballeronia-Pa
d__Bacteria;p__Firmicutes;c__Bacilli;o__Lactobacillales;f__Streptococcaceae;g__Streptococcus;s__Streptococcus_unclassified
d__Bacteria;p__Actinobacteriota;c__Actinobacteria;o__Pseudonocardiales;f__Pseudonocardiaceae;g__Pseudonocardia;s__Pseudonoc
d__Bacteria;p__Firmicutes;c__Erysipelotrichia;o__Erysipelotrichales;f__Erysipelotrichales_unclassified;g__Erysipelotrichale
d__Bacteria;p__Actinobacteriota;c__Actinobacteria;o__Bifidobacteriales;f__Bifidobacteriaceae;g__Bifidobacterium;s__Bifidoba
d__Bacteria;p__Proteobacteria;c__Betaproteobacteria;o__Betaproteobacteria_unclassified;f__Betaproteobacteria_unclassified;g
d__Bacteria;p__Proteobacteria;c__Gammaproteobacteria;o__Pseudomonadales;f__Pseudomonadaceae;g__Pseudomonas;s__Pseudomonas_s
d__Bacteria;p__Firmicutes;c__Bacilli;o__Lactobacillales;f__Streptococcaceae;g__Streptococcus;s__Streptococcus_equinus
d__Bacteria;p__Firmicutes;c__Clostridia;o__Lachnospirales;f__Lachnospiraceae;g__Anaerostipes;s__Anaerostipes_unclassified
d__Bacteria;p__Cyanobacteria;c__Cyanobacteriia;o__Chloroplast;f__Chloroplast_unclassified;g__Chloroplast_unclassified;s__Ch
```

d__Bacteria;p__Actinobacteriota;c__Actinobacteriota;o__Micrococcales;f__Intrasporangiaceae;g__Serinicoccus;s__Serinicoccus_
d__Bacteria;p__Proteobacteria;c__Alphaproteobacteria;o__Caulobacterales;f__Caulobacteraceae;g__Brevundimonas;s__Brevundimon
d__Bacteria;p__Deinococcota;c__Deinococci;o__Thermales;f__Thermaceae;g__Meiothermus;s__Meiothermus_unclassified
d__Bacteria;p__Firmicutes;c__Bacilli;o__Mycoplasmatales;f__Mycoplasmataceae;g__Ureaplasma;s__Ureaplasma_unclassified
d__Bacteria;p__Actinobacteriota;c__Actinobacteria;o__Corynebacteriales;f__Corynebacteriaceae;g__Corynebacterium;s__Coryneba
d__Bacteria;p__Proteobacteria;c__Alphaproteobacteria;o__Rhizobiales;f__Xanthobacteraceae;g__Xanthobacter;s__Xanthobacter_sp
d__Bacteria;p__Proteobacteria;c__Alphaproteobacteria;o__Sphingomonadales;f__Sphingomonadaceae;g__Sphingopyxis;s__Sphingopyx
d__Bacteria;p__Firmicutes;c__Clostridia;o__Lachnospirales;f__Lachnospiraceae;g__Dorea;s__Dorea_unclassified
d__Bacteria;p__Firmicutes;c__Desulfotomaculia;o__Desulfotomaculales;f__Desulfurisporaceae;g__Desulfofundulus;s__Desulfofund
d__Bacteria;p__SAR324_clade(Marine_group_B);c__SAR324_clade(Marine_group_B)_unclassified;o__SAR324_clade(Marine_group_B)_un
d__Bacteria;p__Actinobacteriota;c__Actinobacteria;o__Micrococcales;f__Micrococcaceae;g__Rothia;s__Rothia_unclassified
d__Bacteria;p__Proteobacteria;c__Gammaproteobacteria;o__Burkholderiales;f__Burkholderiaceae;g__Ralstonia;s__Ralstonia_uncla
d__Bacteria;p__Proteobacteria;c__Gammaproteobacteria;o__Burkholderiales;f__Alcaligenaceae;g__Achromobacter;s__Achromobacter
d__Bacteria;p__Proteobacteria;c__Alphaproteobacteria;o__Sphingomonadales;f__Sphingomonadaceae;g__Sphingopyxis;s__uncultured
d__Bacteria;p__Actinobacteriota;c__Thermoleophilia;o__Gaiellales;f__Gaiellaceae;g__Gaiella;s__Gaiella_unclassified
d__Bacteria;p__Verrucomicrobiota;c__Verrucomicrobiae;o__Verrucomicrobiales;f__Rubritaleaceae;g__Persicirhabdus;s__Persicirh
d__Bacteria;p__Firmicutes;c__Bacilli;o__Lactobacillales;f__Streptococcaceae;g__Streptococcus;s__uncultured_Streptococcus_sp
d__Bacteria;p__Actinobacteriota;c__Actinobacteriota;o__Micrococcales;f__Microbacteriaceae;g__Yonghaparkia;s__Yonghaparkia_s
d__Bacteria;p__Firmicutes;c__Clostridia;o__Oscillospirales;f__Oscillospiraceae;g__UCG-005;s__UCG-005_unclassified
d__Bacteria;p__Proteobacteria;c__Gammaproteobacteria;o__Xanthomonadales;f__Rhodanobacteraceae;g__Luteibacter;s__Luteibacter
d__Bacteria;p__Bacteroidota;c__Bacteroidia;o__Flavobacteriales;f__Weeksellaceae;g__Chryseobacterium;s__Chryseobacterium_unc
d__Bacteria;p__Bacteroidota;c__Bacteroidia;o__Flavobacteriales;f__Weeksellaceae;g__Chryseobacterium;s__Chryseobacterium_unc
d__Bacteria;p__Proteobacteria;c__Alphaproteobacteria;o__Sphingomonadales;f__Sphingomonadaceae;g__Sphingobium;s__Sphingobium
d__Bacteria;p__Planctomycetota;c__Phycisphaerae;o__Phycisphaerales;f__Phycisphaeraceae;g__SM1A02;s__SM1A02_unclassified
d__Bacteria;p__Cyanobacteria;c__Cyanobacteriia;o__Chloroplast;f__Chloroplast_unclassified;g__Chloroplast_unclassified;s__Ch
d__unclassified;p__unclassified;c__unclassified;o__unclassified;f__unclassified;g__unclassified;s__unclassified
d__Bacteria;p__Proteobacteria;c__Alphaproteobacteria;o__Rhodobacterales;f__Rhodobacteraceae;g__Sulfitobacter;s__Sulfitobact
d__Bacteria;p__Proteobacteria;c__Alphaproteobacteria;o__Sphingomonadales;f__Sphingomonadaceae;g__Sphingomonas;s__Sphingomon
d__Bacteria;p__Bacteroidota;c__Bacteroidia;o__Bacteroidales;f__Bacteroidaceae;g__Bacteroides;s__Bacteroides_acidifaciens
d__Bacteria;p__Cyanobacteria;c__Cyanobacteriia;o__Chloroplast;f__Chloroplast_unclassified;g__Chloroplast_unclassified;s__Ch
d__Bacteria;p__Proteobacteria;c__Gammaproteobacteria;o__Pseudomonadales;f__Moraxellaceae;g__Acinetobacter;s__uncultured_Aci
d__Bacteria;p__Proteobacteria;c__Alphaproteobacteria;o__Sphingomonadales;f__Sphingomonadaceae;g__Sphingopyxis;s__Sphingopyx
d__Bacteria;p__Proteobacteria;c__Gammaproteobacteria;o__Burkholderiales;f__Rhodocyclaceae;g__Dechlorosoma;s__Dechlorosoma_u
d__Bacteria;p__Proteobacteria;c__Gammaproteobacteria;o__Enterobacterales;f__Yersiniaceae;g__Serratia;s__uncultured_Serratia
d__Bacteria;p__Bacteroidota;c__Bacteroidia;o__Cytophagales;f__Hymenobacteraceae;g__Hymenobacter;s__Hymenobacter_unclassifie
d__Bacteria;p__Firmicutes;c__Clostridia;o__Thermincolales;f__Thermincolaceae;g__Thermincola;s__Thermincola_unclassified
d__Bacteria;p__Actinobacteriota;c__Actinobacteria;o__Micrococcales;f__Dermacoccaceae;g__Kytococcus;s__Kytococcus_unclassifi

d__Bacteria;p__Firmicutes;c__Bacilli;o__Erysipelotrichales;f__Erysipelotrichaceae;g__Erysipelothrix;s__Erysipelothrix_uncla
d__Bacteria;p__Firmicutes;c__Negativicutes;o__Veillonellales-Selenomonadales;f__Veillonellaceae;g__Dialister;s__Dialister_u
d__Bacteria;p__Firmicutes;c__Clostridia;o__Monoglobales;f__Monoglobaceae;g__Monoglobus;s__Monoglobus_unclassified
d__Bacteria;p__Proteobacteria;c__Gammaproteobacteria;o__Xanthomonadales;f__Xanthomonadaceae;g__Stenotrophomonas;s__Stenotro
d__Bacteria;p__Proteobacteria;c__Gammaproteobacteria;o__Xanthomonadales;f__Xanthomonadaceae;g__Stenotrophomonas;s__Stenotro
d__Bacteria;p__Proteobacteria;c__Gammaproteobacteria;o__Xanthomonadales;f__Xanthomonadaceae;g__Stenotrophomonas;s__Stenotro
d__Bacteria;p__Firmicutes;c__Firmicutes_unclassified;o__Firmicutes_unclassified;f__Firmicutes_unclassified;g__Firmicutes_un
d__Bacteria;p__Proteobacteria;c__Alphaproteobacteria;o__Rhodobacterales;f__Rhodobacteraceae;g__Pararhodobacter;s__Pararhodo
d__Bacteria;p__Actinobacteriota;c__Actinobacteriota;o__Micrococcales;f__Intrasporangiaceae;g__Janibacter;s__Janibacter_anop
d__Bacteria;p__Proteobacteria;c__Gammaproteobacteria;o__Burkholderiales;f__Oxalobacteraceae;g__Massilia;s__Massilia_unclass
d__Bacteria;p__Proteobacteria;c__Betaproteobacteria;o__Burkholderiales;f__Comamonadaceae;g__Comamonas;s__Comamonas_sp._M2T2
d__Bacteria;p__Cyanobacteria;c__Cyanobacteriia;o__Cyanobacteriales;f__Chroococcidiopsaceae;g__Aliterella;s__Aliterella_uncl
d__Bacteria;p__Firmicutes;c__Bacilli;o__Lactobacillales;f__Lactobacillaceae;g__Limosilactobacillus;s__Limosilactobacillus_u
d__Bacteria;p__Proteobacteria;c__Gammaproteobacteria;o__Enterobacterales;f__Erwiniaceae;g__Pantoea;s__Pantoea_sp.
d__Bacteria;p__Firmicutes;c__Bacilli;o__Lactobacillales;f__Lactobacillaceae;g__Levilactobacillus;s__Levilactobacillus_uncla
d__Bacteria;p__Actinobacteriota;c__Actinobacteria;o__Corynebacteriales;f__Mycobacteriaceae;g__Mycobacterium;s__Mycobacteriu
d__Bacteria;p__Actinobacteriota;c__Actinobacteria;o__Corynebacteriales;f__Mycobacteriaceae;g__Mycobacterium;s__Mycobacteriu
d__Bacteria;p__Proteobacteria;c__Alphaproteobacteria;o__Rhizobiales;f__Bradyrhizobiaceae;g__Nitrobacter;s__Nitrobacter_sp._
d__Bacteria;p__Bacteroidota;c__Bacteroidia;o__Sphingobacteriales;f__env.OPS_17;g__env.OPS_17_unclassified;s__env.OPS_17_unc
d__Bacteria;p__Firmicutes;c__Bacilli;o__Lactobacillales;f__Streptococcaceae;g__Streptococcus;s__Streptococcus_unclassified
d__Bacteria;p__Firmicutes;c__Negativicutes;o__Veillonellales-Selenomonadales;f__Selenomonadaceae;g__Megamonas;s__Megamonas_
d__Bacteria;p__Bacteroidota;c__Bacteroidia;o__Bacteroidales;f__Tannerellaceae;g__Parabacteroides;s__Parabacteroides_merdae
d__Bacteria;p__Bacteroidota;c__Bacteroidia;o__Bacteroidales;f__Bacteroidaceae;g__Bacteroides;s__uncultured_Bacteroides_sp.
d__Bacteria;p__Firmicutes;c__Clostridia;o__Lachnospirales;f__Lachnospiraceae;g__Lachnoclostridium;s__Lachnoclostridium_uncl
d__Bacteria;p__Proteobacteria;c__Gammaproteobacteria;o__Burkholderiales;f__Comamonadaceae;g__Variovorax;s__Variovorax_uncla
d__unclassified;p__unclassified;c__unclassified;o__unclassified;f__unclassified;g__unclassified;s__unclassified
d__Bacteria;p__WPS-2;c__WPS-2_unclassified;o__WPS-2_unclassified;f__WPS-2_unclassified;g__WPS-2_unclassified;s__WPS-2_uncla
d__Bacteria;p__Proteobacteria;c__Alphaproteobacteria;o__Sphingomonadales;f__Sphingomonadaceae;g__Sphingomonas;s__Sphingomon
d__Bacteria;p__Firmicutes;c__Clostridia;o__Peptostreptococcales-Tissierellales;f__Family_XI;g__Finegoldia;s__Finegoldia_unc
d__Bacteria;p__Firmicutes;c__TSAC18;o__TSAC18_unclassified;f__TSAC18_unclassified;g__TSAC18_unclassified;s__TSAC18_unclassi
d__Bacteria;p__Firmicutes;c__Bacilli;o__Erysipelotrichales;f__Erysipelatoclostridiaceae;g__Erysipelatoclostridium;s__Erysip
d__Bacteria;p__Cyanobacteria;c__Cyanobacteriia;o__Chloroplast;f__Chloroplast_unclassified;g__Chloroplast_unclassified;s__Ch
d__Bacteria;p__Firmicutes;c__Bacilli;o__Brevibacillales;f__Brevibacillaceae;g__Brevibacillus;s__Brevibacillus_unclassified
d__Bacteria;p__Proteobacteria;c__Betaproteobacteria;o__Burkholderiales;f__Comamonadaceae;g__Comamonas;s__uncultured_Comamon
d__Bacteria;p__Proteobacteria;c__Alphaproteobacteria;o__Rhizobiales;f__Rhizobiaceae;g__Rhizobium;s__Rhizobium_rosettiforman
d__Bacteria;p__Firmicutes;c__Bacilli;o__Staphylococcales;f__Staphylococcaceae;g__Staphylococcus;s__Staphylococcus_unclassif
d__Bacteria;p__Firmicutes;c__Bacilli;o__Erysipelotrichales;f__Erysipelotrichaceae;g__Erysipelothrix;s__Erysipelothrix_uncla

d__Bacteria;p__Proteobacteria;c__Gammaproteobacteria;o__Burkholderiales;f__Comamonadaceae;g__Delftia;s__Delftia_unclassifie
d__Bacteria;p__Proteobacteria;c__Alphaproteobacteria;o__Paracaedibacterales;f__Paracaedibacteraceae;g__Paracaedibacteraceae
d__Bacteria;p__Firmicutes;c__Clostridia;o__Oscillospirales;f__Oscillospiraceae;g__Flavonifractor;s__Flavonifractor_unclassi
d__Bacteria;p__Proteobacteria;c__Gammaproteobacteria;o__Gammaproteobacteria_Incertae_Sedis;f__Unknown_Family;g__Acidibacter
d__Bacteria;p__Proteobacteria;c__Alphaproteobacteria;o__Alphaproteobacteria_unclassified;f__Alphaproteobacteria_unclassifie
d__Bacteria;p__Actinobacteriota;c__Actinobacteria;o__Micrococcales;f__Micrococcaceae;g__Rothia;s__Rothia_unclassified
d__Bacteria;p__Proteobacteria;c__Alphaproteobacteria;o__Acetobacterales;f__Acetobacteraceae;g__Roseomonas;s__Roseomonas_unc
d__Bacteria;p__Firmicutes;c__Clostridia;o__Peptostreptococcales-Tissierellales;f__Peptostreptococcaceae;g__Romboutsia;s__Ro
d__Bacteria;p__Proteobacteria;c__Alphaproteobacteria;o__Rhizobiales;f__Beijerinckiaceae;g__Methylobacterium-Methylorubrum;s
d__Bacteria;p__Proteobacteria;c__Gammaproteobacteria;o__Burkholderiales;f__Comamonadaceae;g__Acidovorax;s__Acidovorax_uncla
d__Bacteria;p__Firmicutes;c__Firmicutes_unclassified;o__Firmicutes_unclassified;f__Firmicutes_unclassified;g__Firmicutes_un
d__Bacteria;p__Actinobacteriota;c__Actinobacteria;o__Micrococcales;f__Microbacteriaceae;g__Humibacter;s__Humibacter_unclass
d__Bacteria;p__Proteobacteria;c__Alphaproteobacteria;o__Rhizobiales;f__Methylocystaceae;g__Methylocystis;s__uncultured_Meth
d__Bacteria;p__Firmicutes;c__Bacilli;o__Lactobacillales;f__Streptococcaceae;g__Streptococcus;s__Streptococcus_salivarius
d__Bacteria;p__Firmicutes;c__Bacilli;o__Lactobacillales;f__Streptococcaceae;g__Streptococcus;s__Streptococcus_unclassified
d__Bacteria;p__Firmicutes;c__Bacilli;o__Brevibacillales;f__Brevibacillaceae;g__Brevibacillus;s__Brevibacillus_unclassified
d__Bacteria;p__Firmicutes;c__Bacilli;o__Bacillales;f__Bacillaceae;g__Bacillus;s__Bacillus_sp._PSu2_15
d__Bacteria;p__Proteobacteria;c__Gammaproteobacteria;o__Enterobacterales;f__Enterobacteriaceae;g__Klebsiella;s__Klebsiella_
d__Bacteria;p__Actinobacteriota;c__Actinobacteria;o__Micrococcales;f__Micrococcaceae;g__Rothia;s__Rothia_unclassified
d__Bacteria;p__Firmicutes;c__Clostridia;o__Peptostreptococcales-Tissierellales;f__Peptostreptococcaceae;g__Romboutsia;s__Ro
d__Bacteria;p__Proteobacteria;c__Gammaproteobacteria;o__Burkholderiales;f__Burkholderiaceae;g__Burkholderia-Caballeronia-Pa
d__Bacteria;p__Cyanobacteria;c__Cyanobacteriia;o__Chloroplast;f__Chloroplast_unclassified;g__Chloroplast_unclassified;s__Ch
d__Bacteria;p__Proteobacteria;c__Gammaproteobacteria;o__Burkholderiales;f__Burkholderiaceae;g__Burkholderia-Caballeronia-Pa
d__Bacteria;p__Firmicutes;c__Bacilli;o__Lactobacillales;f__Streptococcaceae;g__Streptococcus;s__Streptococcus_mitis
d__Bacteria;p__Proteobacteria;c__Alphaproteobacteria;o__Sphingomonadales;f__Sphingomonadaceae;g__Sphingomonas;s__Sphingomon
d__Bacteria;p__Proteobacteria;c__Gammaproteobacteria;o__Enterobacterales;f__Erwiniaceae;g__Pantoea;s__Pantoea_agglomerans
d__Bacteria;p__Bacteroidota;c__Bacteroidia;o__Bacteroidales;f__Muribaculaceae;g__Muribaculaceae_unclassified;s__Muribaculac
d__Bacteria;p__Firmicutes;c__Clostridia;o__Thermincolales;f__Thermincolaceae;g__Thermincola;s__Thermincola_unclassified
d__Bacteria;p__Bacteroidota;c__Bacteroidia;o__Flavobacteriales;f__Cryomorphaceae;g__Cryomorphaceae_unclassified;s__Cryomorp
d__Bacteria;p__Firmicutes;c__Bacilli;o__Paenibacillales;f__Paenibacillaceae;g__Paenibacillus;s__Paenibacillus_unclassified
d__Bacteria;p__Proteobacteria;c__Gammaproteobacteria;o__Pseudomonadales;f__Pseudomonadaceae;g__Pseudomonas;s__uncultured_Ps
d__Bacteria;p__Firmicutes;c__Bacilli;o__Lactobacillales;f__Streptococcaceae;g__Streptococcus;s__Streptococcus_mitis
d__Bacteria;p__Firmicutes;c__Bacilli;o__Staphylococcales;f__Staphylococcaceae;g__Staphylococcus;s__Staphylococcus_unclassif
d__Bacteria;p__Firmicutes;c__Clostridia;o__Oscillospirales;f__Oscillospiraceae;g__Oscillospiraceae_unclassified;s__Oscillos
d__Bacteria;p__Proteobacteria;c__Gammaproteobacteria;o__Pseudomonadales;f__Moraxellaceae;g__Acinetobacter;s__Acinetobacter_
d__Bacteria;p__Proteobacteria;c__Gammaproteobacteria;o__Burkholderiales;f__Neisseriaceae;g__Neisseria;s__Neisseria_unclassi
d__Bacteria;p__Proteobacteria;c__Alphaproteobacteria;o__Rhizobiales;f__Hyphomicrobiaceae;g__Rhodoplanes;s__uncultured_Rhodo

d__Bacteria;p__Firmicutes;c__Clostridia;o__Clostridia_UCG-014;f__Clostridia_UCG-014_unclassified;g__Clostridia_UCG-014_uncl
d__Bacteria;p__Firmicutes;c__Bacilli;o__Lactobacillales;f__Streptococcaceae;g__Streptococcus;s__uncultured_Streptococcus_sp
d__Bacteria;p__Firmicutes;c__Firmicutes_unclassified;o__Firmicutes_unclassified;f__Firmicutes_unclassified;g__Firmicutes_un
d__Bacteria;p__Proteobacteria;c__Gammaproteobacteria;o__Burkholderiales;f__Comamonadaceae;g__Schlegelella;s__Schlegelella_u
d__Bacteria;p__Proteobacteria;c__Gammaproteobacteria;o__Pseudomonadales;f__Pseudomonadaceae;g__Pseudomonas;s__uncultured_Ps
d__Bacteria;p__Actinobacteriota;c__Actinobacteria;o__Micrococcales;f__Micrococcaceae;g__Kocuria;s__Kocuria_unclassified
d__Bacteria;p__Proteobacteria;c__Alphaproteobacteria;o__Rhizobiales;f__Rhizobiaceae;g__Rhizobium;s__uncultured_Rhizobium_sp
d__Bacteria;p__Actinobacteriota;c__Actinobacteria;o__Frankiales;f__Geodermatophilaceae;g__Modestobacter;s__Modestobacter_un
d__Bacteria;p__Bacteroidota;c__Bacteroidia;o__Flavobacteriales;f__Crocinitomicaceae;g__Fluviicola;s__Fluviicola_unclassifie
d__Bacteria;p__Proteobacteria;c__Gammaproteobacteria;o__Xanthomonadales;f__Xanthomonadaceae;g__Stenotrophomonas;s__Stenotro
d__Bacteria;p__Firmicutes;c__Clostridia;o__Thermincolales;f__Thermincolaceae;g__Thermincola;s__Thermincola_unclassified
d__Bacteria;p__Bacteroidota;c__Bacteroidia;o__Bacteroidales;f__Muribaculaceae;g__Muribaculaceae_unclassified;s__Muribaculac
d__Bacteria;p__Firmicutes;c__Clostridia;o__Oscillospirales;f__Ruminococcaceae;g__Negativibacillus;s__Negativibacillus_uncla
d__Bacteria;p__Firmicutes;c__Clostridia;o__Lachnospirales;f__Lachnospiraceae;g__Blautia;s__Blautia_unclassified
d__Bacteria;p__Cyanobacteria;c__Cyanobacteriia;o__Chloroplast;f__Chloroplast_unclassified;g__Chloroplast_unclassified;s__Ch
d__Bacteria;p__Proteobacteria;c__Alphaproteobacteria;o__Sphingomonadales;f__Sphingomonadaceae;g__Sphingomonas;s__Sphingomon
d__Bacteria;p__Actinobacteriota;c__Actinobacteriota;o__Micrococcales;f__Micrococcaceae;g__Arthrobacter;s__Arthrobacter_wolu
d__unclassified;p__unclassified;c__unclassified;o__unclassified;f__unclassified;g__unclassified;s__unclassified
d__Bacteria;p__Proteobacteria;c__Gammaproteobacteria;o__Enterobacterales;f__Vibrionaceae;g__Vibrio;s__Vibrio_unclassified
d__Bacteria;p__Proteobacteria;c__Gammaproteobacteria;o__Pseudomonadales;f__Moraxellaceae;g__Acinetobacter;s__Acinetobacter_
d__Bacteria;p__Proteobacteria;c__Betaproteobacteria;o__Burkholderiales;f__Comamonadaceae;g__Comamonas;s__uncultured_Comamon
d__Bacteria;p__Proteobacteria;c__Alphaproteobacteria;o__Sphingomonadales;f__Sphingomonadaceae;g__Sphingopyxis;s__Sphingopyx
d__Bacteria;p__Proteobacteria;c__Betaproteobacteria;o__Betaproteobacteria_unclassified;f__Betaproteobacteria_unclassified;g
d__Bacteria;p__Proteobacteria;c__Betaproteobacteria;o__Burkholderiales;f__Comamonadaceae;g__Acidovorax;s__Acidovorax_sp._12
d__Bacteria;p__Proteobacteria;c__Alphaproteobacteria;o__Sphingomonadales;f__Sphingomonadaceae;g__Sphingomonas;s__Sphingomon
d__Bacteria;p__Proteobacteria;c__Gammaproteobacteria;o__Burkholderiales;f__Rhodocyclaceae;g__Dechlorosoma;s__Dechlorosoma_u
d__Bacteria;p__Firmicutes;c__Clostridia;o__Christensenellales;f__Christensenellaceae;g__Christensenellaceae_R-7_group;s__Ch
d__Bacteria;p__Firmicutes;c__Clostridia;o__Clostridiales;f__Clostridiaceae;g__Clostridium;s__Clostridium_sp._Marseille-P382
d__Bacteria;p__Proteobacteria;c__Gammaproteobacteria;o__Enterobacterales;f__Enterobacteriaceae;g__Atlantibacter;s__Atlantib
d__Bacteria;p__Firmicutes;c__Bacilli;o__Lactobacillales;f__Lactobacillaceae;g__Pediococcus;s__Pediococcus_pentosaceus
d__Bacteria;p__Proteobacteria;c__Betaproteobacteria;o__Betaproteobacteria_unclassified;f__Betaproteobacteria_unclassified;g
d__Bacteria;p__Firmicutes;c__Bacilli;o__Paenibacillales;f__Paenibacillaceae;g__Paenibacillus;s__Paenibacillus_unclassified
d__Bacteria;p__Proteobacteria;c__Alphaproteobacteria;o__Sphingomonadales;f__Sphingomonadaceae;g__Sphingopyxis;s__Sphingopyx
d__Bacteria;p__Proteobacteria;c__Alphaproteobacteria;o__Rhizobiales;f__Rhizobiaceae;g__Rhizobium;s__Rhizobium_sp._MR50
d__Bacteria;p__Actinobacteriota;c__Actinobacteria;o__Corynebacteriales;f__Mycobacteriaceae;g__Mycobacterium;s__Mycobacteriu
d__Bacteria;p__Proteobacteria;c__Alphaproteobacteria;o__Rhizobiales;f__Beijerinckiaceae;g__Methylobacterium-Methylorubrum;s
d__Bacteria;p__Patescibacteria;c__Saccharimonadia;o__Saccharimonadales;f__Saccharimonadaceae;g__TM7a;s__TM7a_unclassified

d__Bacteria;p__Proteobacteria;c__Gammaproteobacteria;o__Burkholderiales;f__Rhodocyclaceae;g__Dechlorosoma;s__Dechlorosoma_u
d__Bacteria;p__Proteobacteria;c__Gammaproteobacteria;o__Xanthomonadales;f__Xanthomonadaceae;g__Stenotrophomonas;s__Stenotro
d__Bacteria;p__Proteobacteria;c__Gammaproteobacteria;o__Enterobacterales;f__Pasteurellaceae;g__Haemophilus;s__Haemophilus_u
d__Bacteria;p__Firmicutes;c__Clostridia;o__Clostridiales;f__Lachnospiraceae;g__Lachnospiraceae_unclassified;s__Lachnospirac
d__Bacteria;p__Proteobacteria;c__Betaproteobacteria;o__Burkholderiales;f__Comamonadaceae;g__Comamonas;s__Comamonas_kersters
d__Bacteria;p__Bacteroidota;c__Bacteroidia;o__Bacteroidales;f__Muribaculaceae;g__Muribaculaceae_unclassified;s__Muribaculac
d__Bacteria;p__Actinobacteriota;c__Actinobacteria;o__Actinomycetales;f__Actinomycetaceae;g__Actinomyces;s__Actinomyces_uncl
d__Bacteria;p__Proteobacteria;c__Alphaproteobacteria;o__Rickettsiales;f__Mitochondria;g__Mitochondria_unclassified;s__Mitoc
d__Bacteria;p__Firmicutes;c__Clostridia;o__Oscillospirales;f__Ruminococcaceae;g__Subdoligranulum;s__Subdoligranulum_unclass
d__Bacteria;p__Proteobacteria;c__Alphaproteobacteria;o__Acetobacterales;f__Acetobacteraceae;g__Acidocella;s__Acidocella_unc
d__Bacteria;p__Proteobacteria;c__Gammaproteobacteria;o__Burkholderiales;f__Burkholderiaceae;g__Burkholderia-Caballeronia-Pa
d__Bacteria;p__Proteobacteria;c__Gammaproteobacteria;o__Pseudomonadales;f__Moraxellaceae;g__Acinetobacter;s__Acinetobacter_
d__Bacteria;p__Firmicutes;c__Clostridia;o__Lachnospirales;f__Lachnospiraceae;g__Anaerostipes;s__Anaerostipes_unclassified
d__Bacteria;p__Firmicutes;c__Bacilli;o__Bacillales;f__Bacillaceae;g__Geobacillus;s__Geobacillus_subterraneus
d__Bacteria;p__Proteobacteria;c__Gammaproteobacteria;o__Xanthomonadales;f__Xanthomonadaceae;g__Stenotrophomonas;s__Stenotro
d__Bacteria;p__Deinococcota;c__Deinococci;o__Deinococcales;f__Deinococcaceae;g__Deinococcus;s__Deinococcus_unclassified
d__Bacteria;p__Proteobacteria;c__Gammaproteobacteria;o__Pseudomonadales;f__Moraxellaceae;g__Acinetobacter;s__Acinetobacter_
d__Bacteria;p__Bacteroidota;c__Bacteroidia;o__Bacteroidales;f__Bacteroidaceae;g__Bacteroides;s__Bacteroides_unclassified
d__Bacteria;p__Firmicutes;c__Bacilli;o__Lactobacillales;f__Carnobacteriaceae;g__Granulicatella;s__Granulicatella_elegans
d__Bacteria;p__Firmicutes;c__Clostridia;o__Oscillospirales;f__Ruminococcaceae;g__Fournierella;s__Fournierella_unclassified
d__Bacteria;p__Firmicutes;c__Clostridia;o__Caldicoprobacterales;f__Caldicoprobacteraceae;g__Caldicoprobacter;s__Caldicoprob
d__Bacteria;p__Firmicutes;c__Clostridia;o__Lachnospirales;f__Lachnospiraceae;g__Sellimonas;s__Sellimonas_unclassified
d__Bacteria;p__Firmicutes;c__Clostridia;o__Clostridiales;f__Ruminococcaceae;g__Ruminococcus;s__Ruminococcus_sp._Marseille-P
d__Bacteria;p__Proteobacteria;c__Gammaproteobacteria;o__Enterobacterales;f__Hafniaceae;g__Hafnia-Obesumbacterium;s__Hafnia-
d__Bacteria;p__Proteobacteria;c__Gammaproteobacteria;o__Burkholderiales;f__Rhodocyclaceae;g__Dechlorosoma;s__Dechlorosoma_u
d__Bacteria;p__Bacteroidota;c__Bacteroidia;o__Bacteroidales;f__Bacteroidaceae;g__Bacteroides;s__Bacteroides_unclassified
d__Bacteria;p__Firmicutes;c__Bacilli;o__Lactobacillales;f__Lactobacillaceae;g__Lactiplantibacillus;s__Lactiplantibacillus_u
d__Bacteria;p__Proteobacteria;c__Alphaproteobacteria;o__Sphingomonadales;f__Sphingomonadaceae;g__Sphingopyxis;s__Sphingopyx
d__Bacteria;p__Acidobacteriota;c__Vicinamibacteria;o__Vicinamibacterales;f__Vicinamibacteraceae;g__Vicinamibacteraceae_uncl
d__Bacteria;p__Proteobacteria;c__Alphaproteobacteria;o__Sneathiellales;f__Sneathiellaceae;g__Sneathiella;s__Sneathiella_sp.
d__Bacteria;p__Firmicutes;c__Clostridia;o__Oscillospirales;f__Hungateiclostridiaceae;g__Fastidiosipila;s__Fastidiosipila_un
d__Bacteria;p__Proteobacteria;c__Gammaproteobacteria;o__Pseudomonadales;f__Moraxellaceae;g__Acinetobacter;s__Acinetobacter_
d__Bacteria;p__Bacteroidota;c__Bacteroidia;o__Bacteroidales;f__Bacteroidaceae;g__Bacteroides;s__uncultured_Bacteroides_sp.
d__Bacteria;p__Bacteroidota;c__Bacteroidia;o__Bacteroidales;f__Tannerellaceae;g__Parabacteroides;s__Parabacteroides_distaso
d__Bacteria;p__Proteobacteria;c__Gammaproteobacteria;o__Enterobacterales;f__Enterobacteriaceae;g__Enterobacter;s__Enterobac
d__Bacteria;p__Proteobacteria;c__Gammaproteobacteria;o__Enterobacterales;f__Enterobacteriaceae;g__Enterobacter;s__Enterobac
d__Bacteria;p__Proteobacteria;c__Gammaproteobacteria;o__Pseudomonadales;f__Pseudomonadaceae;g__Pseudomonas;s__uncultured_Ps

d__Bacteria;p__Firmicutes;c__Clostridia;o__Clostridiales;f__Clostridiaceae;g__Clostridium_sensu_stricto_1;s__Clostridium_se
d__Bacteria;p__Firmicutes;c__Bacilli;o__Staphylococcales;f__Staphylococcaceae;g__Staphylococcus;s__Staphylococcus_unclassif
d__Bacteria;p__Actinobacteriota;c__Actinobacteria;o__Corynebacteriales;f__Corynebacteriaceae;g__Corynebacterium;s__Coryneba
d__Bacteria;p__Firmicutes;c__Clostridia;o__Lachnospirales;f__Lachnospiraceae;g__Lachnospiraceae_NK4A136_group;s__Lachnospir
d__Bacteria;p__Proteobacteria;c__Alphaproteobacteria;o__Rhizobiales;f__Rhizobiaceae;g__Rhizobium;s__uncultured_Rhizobium_sp
d__Bacteria;p__Proteobacteria;c__Alphaproteobacteria;o__Rhodobacterales;f__Rhodobacteraceae;g__Rubellimicrobium;s__Rubellim
d__Bacteria;p__Patescibacteria;c__Saccharimonadia;o__Saccharimonadales;f__Saccharimonadales_unclassified;g__Saccharimonadal
d__Bacteria;p__Firmicutes;c__Clostridia;o__Clostridiales;f__Lachnospiraceae;g__Marvinbryantia;s__uncultured_Marvinbryantia_
d__Bacteria;p__Cyanobacteria;c__Cyanobacteriia;o__Chloroplast;f__Chloroplast_unclassified;g__Chloroplast_unclassified;s__Ch
d__Bacteria;p__Bacteroidota;c__Bacteroidia;o__Bacteroidales;f__Bacteroidaceae;g__Bacteroides;s__Bacteroides_unclassified
d__Bacteria;p__Actinobacteriota;c__Actinobacteria;o__Corynebacteriales;f__Dietziaceae;g__Dietzia;s__Dietzia_unclassified
d__Bacteria;p__Firmicutes;c__Clostridia;o__Clostridia_UCG-014;f__Clostridia_UCG-014_unclassified;g__Clostridia_UCG-014_uncl
d__Bacteria;p__Firmicutes;c__Bacilli;o__Lactobacillales;f__Lactobacillaceae;g__Lactobacillus;s__uncultured_Lactobacillus_sp
d__Bacteria;p__Firmicutes;c__Bacilli;o__Paenibacillales;f__Paenibacillaceae;g__Paenibacillaceae_unclassified;s__Paenibacill
d__Bacteria;p__Proteobacteria;c__Alphaproteobacteria;o__Sphingomonadales;f__Sphingomonadaceae;g__Novosphingobium;s__Novosph
d__Bacteria;p__Actinobacteriota;c__Thermoleophilia;o__Solirubrobacterales;f__Solirubrobacteraceae;g__Conexibacter;s__Conexi
d__Bacteria;p__Actinobacteriota;c__Actinobacteria;o__Micrococcales;f__Micrococcaceae;g__Rothia;s__Rothia_unclassified
d__Bacteria;p__Actinobacteriota;c__Actinobacteriota;o__Micrococcales;f__Intrasporangiaceae;g__Janibacter;s__Janibacter_sp._
d__Bacteria;p__Proteobacteria;c__Gammaproteobacteria;o__Enterobacterales;f__Hafniaceae;g__Edwardsiella;s__Edwardsiella_tard
d__Bacteria;p__Proteobacteria;c__Gammaproteobacteria;o__Pseudomonadales;f__Moraxellaceae;g__Acinetobacter;s__Acinetobacter_
d__Bacteria;p__Firmicutes;c__Bacilli;o__Thermicanales;f__Thermicanaceae;g__Hydrogenibacillus;s__Hydrogenibacillus_unclassif
d__Bacteria;p__Firmicutes;c__Bacilli;o__Lactobacillales;f__Streptococcaceae;g__Streptococcus;s__uncultured_Streptococcus_sp
d__Bacteria;p__Proteobacteria;c__Alphaproteobacteria;o__Rhizobiales;f__Hyphomicrobiaceae;g__Devosia;s__uncultured_Devosia_s
d__Bacteria;p__Proteobacteria;c__Gammaproteobacteria;o__Xanthomonadales;f__Xanthomonadaceae;g__Stenotrophomonas;s__Stenotro
d__Bacteria;p__Proteobacteria;c__Alphaproteobacteria;o__Rhizobiales;f__Rhizobiaceae;g__Rhizobium;s__uncultured_Rhizobium_sp
d__Bacteria;p__Proteobacteria;c__Gammaproteobacteria;o__Burkholderiales;f__Comamonadaceae;g__Acidovorax;s__Acidovorax_uncla
d__Bacteria;p__Proteobacteria;c__Gammaproteobacteria;o__Burkholderiales;f__Methylophilaceae;g__OM43_clade;s__OM43_clade_unc
d__Bacteria;p__Firmicutes;c__Clostridia;o__Clostridiales;f__Lachnospiraceae;g__Lachnospiraceae_unclassified;s__Lachnospirac
d__Bacteria;p__Actinobacteriota;c__Actinobacteria;o__Micrococcales;f__Micrococcaceae;g__Kocuria;s__Kocuria_unclassified
d__Bacteria;p__Proteobacteria;c__Gammaproteobacteria;o__Burkholderiales;f__Rhodocyclaceae;g__Zoogloea;s__Zoogloea_unclassif
d__Bacteria;p__Firmicutes;c__Clostridia;o__Oscillospirales;f__Oscillospiraceae;g__Oscillospiraceae_unclassified;s__Oscillos
d__Bacteria;p__Firmicutes;c__Bacilli;o__Lactobacillales;f__Enterococcaceae;g__Enterococcus;s__Enterococcus_faecium
d__Bacteria;p__Proteobacteria;c__Gammaproteobacteria;o__Enterobacterales;f__Enterobacteriaceae;g__Raoultella;s__Raoultella_
d__Bacteria;p__Proteobacteria;c__Alphaproteobacteria;o__Rhodobacterales;f__Rhodobacteraceae;g__Rhodobacter;s__uncultured_Rh
d__Bacteria;p__Firmicutes;c__Clostridia;o__Peptostreptococcales-Tissierellales;f__Peptostreptococcaceae;g__Peptostreptococc
d__Bacteria;p__Proteobacteria;c__Alphaproteobacteria;o__Rhizobiales;f__Rhizobiaceae;g__Rhizobium;s__uncultured_Rhizobium_sp
d__Bacteria;p__Bacteroidota;c__Bacteroidia;o__Bacteroidales;f__Muribaculaceae;g__Muribaculaceae_unclassified;s__Muribaculac

d__Bacteria;p__Proteobacteria;c__Betaproteobacteria;o__Burkholderiales;f__Comamonadaceae;g__Comamonas;s__Comamonas_kersters
d__Bacteria;p__Verrucomicrobiota;c__Verrucomicrobiae;o__Opitutales;f__Opitutaceae;g__IMCC26134;s__IMCC26134_unclassified
d__Bacteria;p__Proteobacteria;c__Alphaproteobacteria;o__Rhizobiales;f__Rhizobiaceae;g__Daeguia;s__Daeguia_unclassified
d__Bacteria;p__Firmicutes;c__Bacilli;o__Lactobacillales;f__Lactobacillaceae;g__Companilactobacillus;s__Companilactobacillus
d__Bacteria;p__Actinobacteriota;c__Coriobacteriia;o__Coriobacteriales;f__Eggerthellaceae;g__Eggerthella;s__Eggerthella_uncl
d__Bacteria;p__Firmicutes;c__Bacilli;o__Lactobacillales;f__Lactobacillaceae;g__Lactobacillus;s__Lactobacillus_helveticus
d__Bacteria;p__Proteobacteria;c__Alphaproteobacteria;o__Sphingomonadales;f__Sphingomonadaceae;g__Novosphingobium;s__Novosph
d__Bacteria;p__Firmicutes;c__Bacilli;o__Lactobacillales;f__Lactobacillaceae;g__Lactobacillus;s__uncultured_Lactobacillus_sp
d__Bacteria;p__Proteobacteria;c__Gammaproteobacteria;o__Pseudomonadales;f__Moraxellaceae;g__Acinetobacter;s__Acinetobacter_
d__Bacteria;p__Proteobacteria;c__Alphaproteobacteria;o__Sphingomonadales;f__Sphingomonadaceae;g__Sphingomonas;s__Sphingomon
d__Bacteria;p__Actinobacteriota;c__Actinobacteria;o__Micrococcales;f__Micrococcaceae;g__Rothia;s__Rothia_unclassified
d__Bacteria;p__Actinobacteriota;c__Actinobacteria;o__Corynebacteriales;f__Nocardiaceae;g__Gordonia;s__Gordonia_unclassified
d__Bacteria;p__Actinobacteriota;c__Actinobacteria;o__Micrococcales;f__Microbacteriaceae;g__Leifsonia;s__Leifsonia_unclassif
d__Bacteria;p__Proteobacteria;c__Betaproteobacteria;o__Burkholderiales;f__Comamonadaceae;g__Comamonas;s__uncultured_Comamon
d__Bacteria;p__Firmicutes;c__Bacilli;o__Lactobacillales;f__Lactobacillaceae;g__Limosilactobacillus;s__Limosilactobacillus_u
d__Bacteria;p__Firmicutes;c__Clostridia;o__Clostridiales;f__Ruminococcaceae;g__Ruminococcus;s__Ruminococcus_sp.
d__Bacteria;p__Proteobacteria;c__Gammaproteobacteria;o__Pseudomonadales;f__Pseudomonadaceae;g__Pseudomonas;s__Pseudomonas_s
d__Bacteria;p__Bacteroidota;c__Bacteroidia;o__Flavobacteriales;f__Flavobacteriaceae;g__Flavobacterium;s__Flavobacterium_unc
d__Bacteria;p__Firmicutes;c__Clostridia;o__Clostridiales;f__Heliobacteriaceae;g__Heliobacterium;s__Heliobacterium_modestica
d__Bacteria;p__Proteobacteria;c__Gammaproteobacteria;o__Enterobacterales;f__Enterobacteriaceae;g__Citrobacter;s__uncultured
d__Bacteria;p__Bacteroidota;c__Bacteroidia;o__Flavobacteriales;f__Weeksellaceae;g__Soonwooa;s__Soonwooa_unclassified
d__Bacteria;p__Firmicutes;c__Clostridia;o__Clostridiales;f__Lachnospiraceae;g__Lachnospiraceae_unclassified;s__Lachnospirac
d__Bacteria;p__Bacteroidota;c__Bacteroidia;o__Cytophagales;f__Spirosomaceae;g__Spirosoma;s__Spirosoma_unclassified
d__Bacteria;p__Firmicutes;c__Thermovenabulia;o__Thermovenabulales;f__Thermovenabulales_unclassified;g__Thermovenabulales_un
d__Bacteria;p__Firmicutes;c__Clostridia;o__Oscillospirales;f__Ruminococcaceae;g__Subdoligranulum;s__Subdoligranulum_unclass
d__Bacteria;p__Firmicutes;c__Clostridia;o__Oscillospirales;f__Eubacterium]_coprostanoligenes_group;g__Eubacterium]_coprosta
d__Bacteria;p__Firmicutes;c__Bacilli;o__Paenibacillales;f__Paenibacillaceae;g__Paenibacillus;s__Paenibacillus_unclassified
d__Bacteria;p__Bacteroidota;c__Bacteroidia;o__Flavobacteriales;f__Flavobacteriaceae;g__Flavobacterium;s__Flavobacterium_unc
d__Bacteria;p__Proteobacteria;c__Gammaproteobacteria;o__Pseudomonadales;f__SAR86_clade;g__SAR86_clade_unclassified;s__SAR86
d__Bacteria;p__Deinococcota;c__Deinococci;o__Deinococcales;f__Deinococcaceae;g__Deinococcus;s__Deinococcus_unclassified
d__Bacteria;p__Firmicutes;c__Bacilli;o__Thermicanales;f__Thermicanaceae;g__Hydrogenibacillus;s__Hydrogenibacillus_unclassif
d__Bacteria;p__Actinobacteriota;c__Actinobacteria;o__Micrococcales;f__Micrococcaceae;g__Rothia;s__Rothia_unclassified
d__Bacteria;p__Firmicutes;c__Bacilli;o__Staphylococcales;f__Staphylococcaceae;g__Staphylococcus;s__Staphylococcus_unclassif
d__Bacteria;p__Bacteroidota;c__Bacteroidia;o__Sphingobacteriales;f__Sphingobacteriaceae;g__Sphingobacterium;s__Sphingobacte
d__Bacteria;p__Proteobacteria;c__Gammaproteobacteria;o__Burkholderiales;f__Burkholderiaceae;g__Burkholderia-Caballeronia-Pa
d__Bacteria;p__Bacteroidota;c__Bacteroidia;o__Bacteroidales;f__Rikenellaceae;g__Alistipes;s__Alistipes_massiliensis
d__Bacteria;p__Firmicutes;c__Bacilli;o__Erysipelotrichales;f__Erysipelatoclostridiaceae;g__Erysipelatoclostridium;s__Erysip

d__Bacteria;p__Proteobacteria;c__Gammaproteobacteria;o__Xanthomonadales;f__Rhodanobacteraceae;g__Rhodanobacter;s__unculture
d__Bacteria;p__Firmicutes;c__Clostridia;o__Oscillospirales;f__Ruminococcaceae;g__Fournierella;s__Fournierella_unclassified
d__Bacteria;p__Chloroflexi;c__KD4-96;o__KD4-96_unclassified;f__KD4-96_unclassified;g__KD4-96_unclassified;s__KD4-96_unclass
d__Bacteria;p__Actinobacteriota;c__Actinobacteria;o__Micrococcales;f__Micrococcaceae;g__Rothia;s__Rothia_unclassified
d__Bacteria;p__Bacteroidota;c__Bacteroidia;o__Chitinophagales;f__Chitinophagaceae;g__Flavisolibacter;s__Flavisolibacter_unc
d__Bacteria;p__Firmicutes;c__Bacilli;o__Erysipelotrichales;f__Erysipelotrichaceae;g__Dubosiella;s__Dubosiella_unclassified
d__Bacteria;p__Proteobacteria;c__Gammaproteobacteria;o__Enterobacterales;f__Erwiniaceae;g__Tatumella;s__Tatumella_terrea
d__Bacteria;p__Bacteroidota;c__Bacteroidia;o__Bacteroidales;f__Bacteroidaceae;g__Bacteroides;s__Bacteroides_uniformis
d__Bacteria;p__Proteobacteria;c__Gammaproteobacteria;o__Aeromonadales;f__Aeromonadaceae;g__Zobellella;s__Zobellella_sp._XT-
d__Bacteria;p__Firmicutes;c__Clostridia;o__Clostridiales;f__Clostridiaceae;g__Clostridium;s__Clostridium_sp._Clone-49
d__Bacteria;p__Firmicutes;c__Clostridia;o__Oscillospirales;f__Ruminococcaceae;g__Ruminococcaceae_unclassified;s__Ruminococc
d__Bacteria;p__Proteobacteria;c__Gammaproteobacteria;o__Xanthomonadales;f__Xanthomonadaceae;g__Stenotrophomonas;s__Stenotro
d__Bacteria;p__Firmicutes;c__Clostridia;o__Oscillospirales;f__Oscillospiraceae;g__UCG-002;s__UCG-002_unclassified
d__Bacteria;p__Firmicutes;c__Clostridia;o__Lachnospirales;f__Lachnospiraceae;g__Shuttleworthia;s__Shuttleworthia_unclassifi
d__Bacteria;p__Firmicutes;c__Clostridia;o__Clostridiales;f__Lachnospiraceae;g__Lachnospiraceae_unclassified;s__Lachnospirac
d__Bacteria;p__Firmicutes;c__Firmicutes_unclassified;o__Firmicutes_unclassified;f__Firmicutes_unclassified;g__Firmicutes_un
d__Bacteria;p__Firmicutes;c__Bacilli;o__Bacillales;f__Bacillaceae;g__Bacillus;s__Bacillus_kokeshiiformis
d__Bacteria;p__Firmicutes;c__Bacilli;o__Erysipelotrichales;f__Erysipelatoclostridiaceae;g__Catenibacterium;s__Catenibacteri
d__Bacteria;p__Firmicutes;c__Clostridia;o__Oscillospirales;f__Ruminococcaceae;g__Subdoligranulum;s__Subdoligranulum_unclass
d__Bacteria;p__Proteobacteria;c__Gammaproteobacteria;o__Gammaproteobacteria_unclassified;f__Gammaproteobacteria_unclassifie
d__Bacteria;p__Proteobacteria;c__Gammaproteobacteria;o__Enterobacterales;f__Aeromonadaceae;g__Zobellella;s__Zobellella_uncl
d__Bacteria;p__Bacteroidota;c__Bacteroidia;o__Bacteroidales;f__Prevotellaceae;g__Prevotella_9;s__Prevotella_9_unclassified
d__Bacteria;p__Actinobacteriota;c__Actinobacteriota;o__Micrococcales;f__Microbacteriaceae;g__Microbacteriaceae_unclassified
d__Bacteria;p__Proteobacteria;c__Gammaproteobacteria;o__Enterobacterales;f__Enterobacteriaceae;g__Pseudocitrobacter;s__Pseu
d__Bacteria;p__Firmicutes;c__Bacilli;o__Bacillales;f__Bacillaceae;g__Bacillus;s__Bacillus_tequilensis
d__Bacteria;p__Proteobacteria;c__Gammaproteobacteria;o__CCD24;f__CCD24_unclassified;g__CCD24_unclassified;s__CCD24_unclassi
d__Bacteria;p__Proteobacteria;c__Alphaproteobacteria;o__Sphingomonadales;f__Sphingomonadaceae;g__Sphingopyxis;s__Sphingopyx
d__Bacteria;p__Firmicutes;c__Clostridia;o__Lachnospirales;f__Lachnospiraceae;g__Lachnospira;s__Lachnospira_unclassified
d__Bacteria;p__Firmicutes;c__Bacilli;o__Lactobacillales;f__Lactobacillaceae;g__HT002;s__HT002_unclassified
d__Bacteria;p__Proteobacteria;c__Gammaproteobacteria;o__Xanthomonadales;f__Xanthomonadaceae;g__Stenotrophomonas;s__Stenotro
d__Bacteria;p__Proteobacteria;c__Gammaproteobacteria;o__Enterobacterales;f__Enterobacteriaceae;g__Raoultella;s__Raoultella_
d__Bacteria;p__Bacteroidota;c__Bacteroidia;o__Bacteroidales;f__Muribaculaceae;g__Muribaculaceae_unclassified;s__Muribaculac
d__Bacteria;p__Bacteroidota;c__Bacteroidia;o__Bacteroidales;f__Muribaculaceae;g__Muribaculaceae_unclassified;s__Muribaculac
d__Bacteria;p__Desulfobacterota;c__Desulfovibrionia;o__Desulfovibrionales;f__Desulfovibrionaceae;g__Desulfovibrio;s__Desulf
d__Bacteria;p__Proteobacteria;c__Gammaproteobacteria;o__Enterobacterales;f__Enterobacteriaceae;g__Klebsiella;s__Klebsiella_
d__Bacteria;p__Firmicutes;c__Bacilli;o__Lactobacillales;f__Lactobacillaceae;g__Pediococcus;s__Pediococcus_acidilactici
d__Bacteria;p__Actinobacteriota;c__Coriobacteriia;o__Coriobacteriales;f__Atopobiaceae;g__Coriobacteriaceae_UCG-002;s__Corio

d__Bacteria;p__Actinobacteriota;c__Actinobacteriota;o__Geodermatophilales;f__Geodermatophilaceae;g__Geodermatophilaceae_unc
d__Bacteria;p__Firmicutes;c__Clostridia;o__Oscillospirales;f__Butyricicoccaceae;g__Butyricicoccus;s__Butyricicoccus_unclass
d__Bacteria;p__Cyanobacteria;c__Cyanobacteriia;o__Chloroplast;f__Chloroplast_unclassified;g__Chloroplast_unclassified;s__Ch
d__Bacteria;p__Firmicutes;c__Clostridia;o__Clostridia_UCG-014;f__Clostridia_UCG-014_unclassified;g__Clostridia_UCG-014_uncl
d__Bacteria;p__Cyanobacteria;c__Cyanobacteriia;o__Chloroplast;f__Chloroplast_unclassified;g__Chloroplast_unclassified;s__Ch
d__Bacteria;p__Cyanobacteria;c__Cyanobacteriia;o__Chloroplast;f__Chloroplast_unclassified;g__Chloroplast_unclassified;s__Ch
d__Bacteria;p__Firmicutes;c__Bacilli;o__Lactobacillales;f__Streptococcaceae;g__Streptococcus;s__uncultured_Streptococcus_sp
d__Bacteria;p__Proteobacteria;c__Gammaproteobacteria;o__Burkholderiales;f__Comamonadaceae;g__Schlegelella;s__Schlegelella_u
d__Bacteria;p__Proteobacteria;c__Gammaproteobacteria;o__Enterobacterales;f__Hafniaceae;g__Edwardsiella;s__Edwardsiella_tard
d__Bacteria;p__Cyanobacteria;c__Cyanobacteriia;o__Synechococcales;f__Cyanobiaceae;g__Synechococcus_CC9902;s__Synechococcus_
d__Bacteria;p__Actinobacteriota;c__Actinobacteria;o__Propionibacteriales;f__Nocardioidaceae;g__Nocardioides;s__Nocardioides
d__Bacteria;p__Actinobacteriota;c__Actinobacteriota;o__Micrococcales;f__Microbacteriaceae;g__Mycetocola;s__Mycetocola_sp.
d__Bacteria;p__Firmicutes;c__Clostridia;o__Clostridiales;f__Lachnospiraceae;g__Lachnospiraceae_unclassified;s__Lachnospirac
d__Bacteria;p__Bacteroidota;c__Bacteroidia;o__Bacteroidales;f__Muribaculaceae;g__Muribaculaceae_unclassified;s__Muribaculac
d__Bacteria;p__Proteobacteria;c__Gammaproteobacteria;o__Burkholderiales;f__Rhodocyclaceae;g__Dechlorosoma;s__Dechlorosoma_u
d__Bacteria;p__Proteobacteria;c__Betaproteobacteria;o__Betaproteobacteria_unclassified;f__Betaproteobacteria_unclassified;g
d__Bacteria;p__Proteobacteria;c__Gammaproteobacteria;o__Burkholderiales;f__Comamonadaceae;g__Variovorax;s__Variovorax_uncla
d__Bacteria;p__Actinobacteriota;c__Actinobacteria;o__Micrococcales;f__Microbacteriaceae;g__Curtobacterium;s__Curtobacterium
d__Bacteria;p__Firmicutes;c__Clostridia;o__Clostridia_UCG-014;f__Clostridia_UCG-014_unclassified;g__Clostridia_UCG-014_uncl
d__Bacteria;p__Bdellovibrionota;c__Oligoflexia;o__Oligoflexales;f__Oligoflexaceae;g__Oligoflexaceae_unclassified;s__Oligofl
d__Bacteria;p__Proteobacteria;c__Gammaproteobacteria;o__Burkholderiales;f__Neisseriaceae;g__Neisseria;s__Neisseria_unclassi
d__Bacteria;p__Actinobacteriota;c__Actinobacteria;o__Corynebacteriales;f__Nocardiaceae;g__Gordonia;s__Gordonia_unclassified
d__Bacteria;p__Proteobacteria;c__Gammaproteobacteria;o__Burkholderiales;f__Rhodocyclaceae;g__Dechloromonas;s__Dechloromonas
d__Bacteria;p__Proteobacteria;c__Betaproteobacteria;o__Burkholderiales;f__Alcaligenaceae;g__Bordetella;s__Bordetella_bronch
d__Bacteria;p__Proteobacteria;c__Alphaproteobacteria;o__Rhizobiales;f__Hyphomicrobiaceae;g__Hyphomicrobium;s__Hyphomicrobiu
d__Bacteria;p__Proteobacteria;c__Gammaproteobacteria;o__Xanthomonadales;f__Rhodanobacteraceae;g__Dyella;s__Dyella_ginsengis
d__Bacteria;p__Firmicutes;c__Bacilli;o__Bacillales;f__Bacillaceae;g__Virgibacillus;s__uncultured_Virgibacillus_sp.
d__Bacteria;p__Proteobacteria;c__Gammaproteobacteria;o__Enterobacterales;f__Erwiniaceae;g__Pantoea;s__Pantoea_sp._Sc1
d__Bacteria;p__Firmicutes;c__Clostridia;o__Lachnospirales;f__Lachnospiraceae;g__Anaerostipes;s__Anaerostipes_unclassified
d__Bacteria;p__Firmicutes;c__Negativicutes;o__Veillonellales-Selenomonadales;f__Selenomonadaceae;g__Megamonas;s__Megamonas_
d__Bacteria;p__Cyanobacteria;c__Cyanobacteria_unclassified;o__Cyanobacteria_unclassified;f__Cyanobacteria_unclassified;g__C
d__Bacteria;p__Proteobacteria;c__Alphaproteobacteria;o__Rickettsiales;f__Rickettsiaceae;g__Rickettsia;s__Rickettsia_endosym
d__Bacteria;p__Firmicutes;c__Clostridia;o__Clostridiales;f__Clostridiales_unclassified;g__Clostridiales_unclassified;s__Clo
d__Bacteria;p__Proteobacteria;c__Gammaproteobacteria;o__Pseudomonadales;f__Moraxellaceae;g__Acinetobacter;s__Acinetobacter_
d__Bacteria;p__Cyanobacteria;c__Cyanobacteriia;o__Synechococcales;f__Cyanobiaceae;g__Synechococcus_CC9902;s__Synechococcus_
d__Bacteria;p__Proteobacteria;c__Betaproteobacteria;o__Burkholderiales;f__Comamonadaceae;g__Acidovorax;s__uncultured_Acidov
d__Bacteria;p__Proteobacteria;c__Gammaproteobacteria;o__Xanthomonadales;f__Xanthomonadaceae;g__Luteimonas;s__Luteimonas_ars

d__Bacteria;p__Bacteroidota;c__Bacteroidia;o__Bacteroidales;f__Muribaculaceae;g__Muribaculaceae_unclassified;s__Muribaculac
d__Bacteria;p__Proteobacteria;c__Betaproteobacteria;o__Burkholderiales;f__Comamonadaceae;g__Comamonas;s__uncultured_Comamon
d__Bacteria;p__Firmicutes;c__Clostridia;o__Oscillospirales;f__Oscillospiraceae;g__Colidextribacter;s__Colidextribacter_uncl
d__Bacteria;p__Firmicutes;c__Clostridia;o__Clostridiales;f__Ruminococcaceae;g__Ruminococcus;s__Ruminococcus_sp.
d__Bacteria;p__Firmicutes;c__Bacilli;o__Lactobacillales;f__Lactobacillaceae;g__Limosilactobacillus;s__Limosilactobacillus_u
d__Bacteria;p__Proteobacteria;c__Gammaproteobacteria;o__Pseudomonadales;f__Moraxellaceae;g__Acinetobacter;s__uncultured_Aci
d__Bacteria;p__Cyanobacteria;c__Cyanobacteriia;o__Chloroplast;f__Chloroplast_unclassified;g__Chloroplast_unclassified;s__Ch
d__Bacteria;p__Proteobacteria;c__Alphaproteobacteria;o__Rhizobiales;f__Beijerinckiaceae;g__Methylobacterium-Methylorubrum;s
d__Bacteria;p__Firmicutes;c__Clostridia;o__Lachnospirales;f__Lachnospiraceae;g__Anaerostipes;s__Anaerostipes_unclassified
d__Bacteria;p__Actinobacteriota;c__Coriobacteriia;o__Coriobacteriales;f__Coriobacteriaceae;g__Collinsella;s__Collinsella_un
d__Bacteria;p__Firmicutes;c__Clostridia;o__Oscillospirales;f__Ruminococcaceae;g__Incertae_Sedis;s__Incertae_Sedis_unclassif
d__Bacteria;p__Actinobacteriota;c__Actinobacteria;o__Bifidobacteriales;f__Bifidobacteriaceae;g__Gardnerella;s__Gardnerella_
d__Bacteria;p__Proteobacteria;c__Gammaproteobacteria;o__Burkholderiales;f__Alcaligenaceae;g__Achromobacter;s__Achromobacter
d__Bacteria;p__Proteobacteria;c__Alphaproteobacteria;o__Sphingomonadales;f__Sphingomonadaceae;g__Sphingomonas;s__Sphingomon
d__Bacteria;p__Chloroflexi;c__Anaerolineae;o__Anaerolineales;f__Anaerolineaceae;g__Anaerolineaceae_unclassified;s__Anaeroli
d__Bacteria;p__Bacteroidota;c__Bacteroidia;o__Flavobacteriales;f__Weeksellaceae;g__Chryseobacterium;s__Chryseobacterium_unc
d__Bacteria;p__Actinobacteriota;c__Actinobacteriota;o__Actinobacteriota_unclassified;f__Actinobacteriota_unclassified;g__Ac
d__Bacteria;p__Bacteroidota;c__Bacteroidia;o__Bacteroidales;f__Dysgonomonadaceae;g__Proteiniphilum;s__Proteiniphilum_unclas
d__Bacteria;p__Bacteroidota;c__Bacteroidia;o__Cytophagales;f__Microscillaceae;g__OLB12;s__OLB12_unclassified
d__Bacteria;p__Proteobacteria;c__Alphaproteobacteria;o__Alphaproteobacteria_unclassified;f__Alphaproteobacteria_unclassifie
d__Bacteria;p__Firmicutes;c__Clostridia;o__Oscillospirales;f__Oscillospiraceae;g__UCG-002;s__UCG-002_unclassified
d__Bacteria;p__Actinobacteriota;c__Coriobacteriia;o__Coriobacteriales;f__Coriobacteriaceae;g__Collinsella;s__Collinsella_un
d__Bacteria;p__Firmicutes;c__Clostridia;o__Lachnospirales;f__Lachnospiraceae;g__Coprococcus;s__Coprococcus_unclassified
d__Bacteria;p__Firmicutes;c__Negativicutes;o__Veillonellales-Selenomonadales;f__Veillonellaceae;g__Megasphaera;s__Megasphae
d__Bacteria;p__Firmicutes;c__Bacilli;o__Staphylococcales;f__Staphylococcaceae;g__Staphylococcus;s__Staphylococcus_unclassif
d__Bacteria;p__Fusobacteriota;c__Fusobacteriia;o__Fusobacteriales;f__Fusobacteriaceae;g__Fusobacterium;s__Fusobacterium_unc
d__Bacteria;p__Firmicutes;c__Clostridia;o__Peptostreptococcales-Tissierellales;f__Family_XI;g__Peptoniphilus;s__Peptoniphil
d__Bacteria;p__Proteobacteria;c__Gammaproteobacteria;o__Enterobacterales;f__Enterobacteriaceae;g__Escherichia-Shigella;s__E
d__Bacteria;p__Firmicutes;c__Negativicutes;o__Veillonellales-Selenomonadales;f__Veillonellaceae;g__Megasphaera;s__Megasphae
d__Bacteria;p__Proteobacteria;c__Gammaproteobacteria;o__Enterobacterales;f__Yersiniaceae;g__Serratia;s__Serratia_unclassifi
d__Bacteria;p__Proteobacteria;c__Alphaproteobacteria;o__Rhizobiales;f__Beijerinckiaceae;g__Methylobacterium-Methylorubrum;s
d__Bacteria;p__Bacteroidota;c__Bacteroidia;o__Bacteroidales;f__Muribaculaceae;g__Muribaculaceae_unclassified;s__Muribaculac
d__Bacteria;p__Firmicutes;c__Clostridia;o__Clostridiales;f__Oscillospiraceae;g__Oscillibacter;s__Oscillibacter_sp.
d__Bacteria;p__Firmicutes;c__Clostridia;o__Thermincolales;f__Thermincolaceae;g__Thermincola;s__Thermincola_unclassified
d__Bacteria;p__Proteobacteria;c__Gammaproteobacteria;o__Enterobacterales;f__Erwiniaceae;g__Pantoea;s__Pantoea_agglomerans
d__Bacteria;p__Firmicutes;c__Bacilli;o__Lactobacillales;f__Enterococcaceae;g__Enterococcus;s__Enterococcus_sp.
d__Bacteria;p__Firmicutes;c__Bacilli;o__Lactobacillales;f__Lactobacillaceae;g__Levilactobacillus;s__Levilactobacillus_uncla

d__Bacteria;p__Proteobacteria;c__Gammaproteobacteria;o__Enterobacterales;f__Aeromonadaceae;g__Aeromonas;s__Aeromonas_unclas
d__Bacteria;p__Proteobacteria;c__Gammaproteobacteria;o__Burkholderiales;f__Comamonadaceae;g__Limnohabitans;s__Limnohabitans
d__Bacteria;p__Actinobacteriota;c__Acidimicrobiia;o__Acidimicrobiales;f__Acidimicrobiaceae;g__Acidimicrobiaceae_unclassifie
d__Bacteria;p__Proteobacteria;c__Gammaproteobacteria;o__Pseudomonadales;f__Pseudomonadaceae;g__Pseudomonas;s__Pseudomonas_s
d__Bacteria;p__Proteobacteria;c__Gammaproteobacteria;o__Pseudomonadales;f__Oleiphilaceae;g__Oleiphilus;s__Oleiphilus_unclas
d__Bacteria;p__Firmicutes;c__Clostridia;o__Lachnospirales;f__Lachnospiraceae;g__CHKCI001;s__CHKCI001_unclassified
d__Bacteria;p__Firmicutes;c__Moorellia;o__Moorellales;f__Moorellaceae;g__Moorella;s__Moorella_unclassified
d__Bacteria;p__Firmicutes;c__Bacilli;o__Lactobacillales;f__Lactobacillaceae;g__Lactobacillus;s__Lactobacillus_sp._L-YJ
d__Bacteria;p__Proteobacteria;c__Gammaproteobacteria;o__Burkholderiales;f__Rhodocyclaceae;g__Thauera;s__Thauera_unclassifie
d__Bacteria;p__Bacteroidota;c__Bacteroidia;o__Flavobacteriales;f__Weeksellaceae;g__Cloacibacterium;s__Cloacibacterium_uncla
d__Bacteria;p__Actinobacteriota;c__Actinobacteria;o__Micrococcales;f__Bogoriellaceae;g__Georgenia;s__Georgenia_unclassified
d__Bacteria;p__Actinobacteriota;c__Actinobacteria;o__Micrococcales;f__Micrococcaceae;g__Rothia;s__Rothia_unclassified
d__Bacteria;p__Firmicutes;c__Clostridia;o__Caldicoprobacterales;f__Caldicoprobacteraceae;g__Caldicoprobacter;s__Caldicoprob
d__Bacteria;p__Firmicutes;c__Clostridia;o__Lachnospirales;f__Lachnospiraceae;g__Lachnospira;s__Lachnospira_unclassified
d__Bacteria;p__Proteobacteria;c__Alphaproteobacteria;o__Azospirillales;f__Azospirillaceae;g__Skermanella;s__Skermanella_unc
d__Bacteria;p__Proteobacteria;c__Gammaproteobacteria;o__Xanthomonadales;f__Xanthomonadaceae;g__Stenotrophomonas;s__Stenotro
d__Bacteria;p__Firmicutes;c__Bacilli;o__Staphylococcales;f__Staphylococcaceae;g__Staphylococcus;s__Staphylococcus_unclassif
d__Bacteria;p__Proteobacteria;c__Betaproteobacteria;o__Betaproteobacteria_unclassified;f__Betaproteobacteria_unclassified;g
d__Bacteria;p__Actinobacteriota;c__Actinobacteria;o__Frankiales;f__Nakamurellaceae;g__Nakamurella;s__Nakamurella_unclassifi
d__Bacteria;p__Firmicutes;c__Bacilli;o__Bacillales;f__Bacillaceae;g__Geobacillus;s__Geobacillus_sp.
d__Bacteria;p__Proteobacteria;c__Gammaproteobacteria;o__Xanthomonadales;f__Xanthomonadaceae;g__Stenotrophomonas;s__Stenotro
d__Bacteria;p__Proteobacteria;c__Gammaproteobacteria;o__Enterobacterales;f__Enterobacteriaceae;g__Enterobacter;s__Enterobac
d__Bacteria;p__Proteobacteria;c__Gammaproteobacteria;o__Enterobacterales;f__Enterobacteriaceae;g__Enterobacter;s__Enterobac
d__Bacteria;p__Firmicutes;c__Clostridia;o__Lachnospirales;f__Lachnospiraceae;g__Ruminococcus]_gauvreauii_group;s__Ruminococ
d__Bacteria;p__Firmicutes;c__Clostridia;o__Clostridia_UCG-014;f__Clostridia_UCG-014_unclassified;g__Clostridia_UCG-014_uncl
d__Bacteria;p__Firmicutes;c__Clostridia;o__Peptostreptococcales-Tissierellales;f__Peptostreptococcaceae;g__Acetoanaerobium;
d__Bacteria;p__Proteobacteria;c__Gammaproteobacteria;o__Pseudomonadales;f__Pseudomonadaceae;g__Pseudomonas;s__uncultured_Ps
d__Bacteria;p__Proteobacteria;c__Alphaproteobacteria;o__Rhizobiales;f__Beijerinckiaceae;g__Methylobacterium-Methylorubrum;s
d__Bacteria;p__Actinobacteriota;c__Actinobacteria;o__Micrococcales;f__Micrococcaceae;g__Micrococcus;s__Micrococcus_unclassi
d__Bacteria;p__Proteobacteria;c__Gammaproteobacteria;o__Xanthomonadales;f__Rhodanobacteraceae;g__Ahniella;s__Ahniella_uncla
d__Bacteria;p__Proteobacteria;c__Betaproteobacteria;o__Burkholderiales;f__Comamonadaceae;g__Curvibacter;s__Curvibacter_font
d__Bacteria;p__Firmicutes;c__Clostridia;o__Clostridiales;f__Clostridiales_unclassified;g__Clostridiales_unclassified;s__Clo
d__Bacteria;p__Firmicutes;c__Desulfotomaculia;o__Desulfotomaculales;f__Desulfotomaculaceae;g__Desulfotomaculum;s__Desulfoto
d__Bacteria;p__Firmicutes;c__Clostridia;o__Clostridiales;f__Ruminococcaceae;g__Ruminococcaceae_unclassified;s__Ruminococcac
d__Bacteria;p__Firmicutes;c__Clostridia;o__Thermincolales;f__Thermincolaceae;g__Thermincola;s__Thermincola_unclassified
d__Bacteria;p__Proteobacteria;c__Gammaproteobacteria;o__Burkholderiales;f__Comamonadaceae;g__RS62_marine_group;s__RS62_mari
d__Bacteria;p__Firmicutes;c__Clostridia;o__Oscillospirales;f__Ruminococcaceae;g__Subdoligranulum;s__Subdoligranulum_unclass

d__Bacteria;p__Proteobacteria;c__Alphaproteobacteria;o__Rhizobiales;f__Kaistiaceae;g__Kaistia;s__Kaistia_unclassified
d__Bacteria;p__Actinobacteriota;c__Actinobacteria;o__Corynebacteriales;f__Mycobacteriaceae;g__Mycobacterium;s__Mycobacteriu
d__Bacteria;p__Proteobacteria;c__Betaproteobacteria;o__Burkholderiales;f__Alcaligenaceae;g__Candidimonas;s__Candidimonas_hu
d__Bacteria;p__Bacteroidota;c__Bacteroidia;o__Bacteroidales;f__Bacteroidaceae;g__Bacteroides;s__Bacteroides_unclassified
d__Bacteria;p__Deinococcota;c__Deinococci;o__Deinococcales;f__Deinococcaceae;g__Deinococcus;s__Deinococcus_unclassified
d__Bacteria;p__Proteobacteria;c__Gammaproteobacteria;o__Enterobacterales;f__Enterobacteriaceae;g__Escherichia-Shigella;s__E
d__Bacteria;p__Proteobacteria;c__Gammaproteobacteria;o__Burkholderiales;f__Oxalobacteraceae;g__Massilia;s__Massilia_unclass
d__Bacteria;p__Firmicutes;c__Bacilli;o__Lactobacillales;f__Streptococcaceae;g__Streptococcus;s__Streptococcus_parasanguinis
d__Bacteria;p__Fusobacteriota;c__Fusobacteriia;o__Fusobacteriales;f__Leptotrichiaceae;g__Leptotrichia;s__Leptotrichia_uncla
d__Bacteria;p__Cyanobacteria;c__Cyanobacteriia;o__Synechococcales;f__Cyanobiaceae;g__Cyanobium_PCC-6307;s__Cyanobium_PCC-63
d__Bacteria;p__Actinobacteriota;c__Actinobacteria;o__Corynebacteriales;f__Corynebacteriaceae;g__Lawsonella;s__Lawsonella_un
d__Bacteria;p__Proteobacteria;c__Gammaproteobacteria;o__Enterobacterales;f__Enterobacteriaceae;g__Enterobacter;s__Enterobac
d__Bacteria;p__Proteobacteria;c__Gammaproteobacteria;o__Xanthomonadales;f__Xanthomonadaceae;g__Stenotrophomonas;s__Stenotro
d__Bacteria;p__Firmicutes;c__Clostridia;o__Lachnospirales;f__Lachnospiraceae;g__A2;s__A2_unclassified
d__Bacteria;p__Proteobacteria;c__Gammaproteobacteria;o__Burkholderiales;f__Rhodocyclaceae;g__Dechlorosoma;s__Dechlorosoma_u
d__Bacteria;p__Firmicutes;c__Bacilli;o__Staphylococcales;f__Staphylococcaceae;g__Staphylococcus;s__Staphylococcus_unclassif
d__Bacteria;p__Firmicutes;c__Bacilli;o__Lactobacillales;f__Streptococcaceae;g__Streptococcus;s__uncultured_Streptococcus_sp
d__Bacteria;p__Firmicutes;c__Bacilli;o__Erysipelotrichales;f__Erysipelatoclostridiaceae;g__Catenibacterium;s__Catenibacteri
d__Bacteria;p__Proteobacteria;c__Gammaproteobacteria;o__Burkholderiales;f__Oxalobacteraceae;g__Herbaspirillum;s__Herbaspiri
d__Bacteria;p__Firmicutes;c__Clostridia;o__Clostridiales;f__Clostridiales_unclassified;g__Clostridiales_unclassified;s__Clo
d__Bacteria;p__Actinobacteriota;c__Actinobacteria;o__Propionibacteriales;f__Nocardioidaceae;g__Nocardioides;s__Nocardioides
d__Bacteria;p__Proteobacteria;c__Gammaproteobacteria;o__Xanthomonadales;f__Xanthomonadaceae;g__Stenotrophomonas;s__Stenotro
d__Bacteria;p__Proteobacteria;c__Alphaproteobacteria;o__Rhodospirillales;f__Terasakiellaceae;g__Terasakiellaceae_unclassifi
d__Bacteria;p__Firmicutes;c__Clostridia;o__Oscillospirales;f__Butyricicoccaceae;g__Butyricicoccus;s__Butyricicoccus_unclass
d__Bacteria;p__Cyanobacteria;c__Cyanobacteriia;o__Chloroplast;f__Chloroplast_unclassified;g__Chloroplast_unclassified;s__Ch
d__Bacteria;p__Proteobacteria;c__Betaproteobacteria;o__Betaproteobacteria_unclassified;f__Betaproteobacteria_unclassified;g
d__Bacteria;p__Cyanobacteria;c__Cyanobacteriia;o__Chloroplast;f__Chloroplast_unclassified;g__Chloroplast_unclassified;s__Ch
d__Bacteria;p__Firmicutes;c__Firmicutes_unclassified;o__Firmicutes_unclassified;f__Firmicutes_unclassified;g__Firmicutes_un
d__Bacteria;p__Proteobacteria;c__Gammaproteobacteria;o__Legionellales;f__Legionellaceae;g__Legionella;s__uncultured_Legione
d__Bacteria;p__Proteobacteria;c__Gammaproteobacteria;o__Burkholderiales;f__Rhodocyclaceae;g__Dechloromonas;s__Dechloromonas
d__Bacteria;p__Actinobacteriota;c__Actinobacteria;o__Bifidobacteriales;f__Bifidobacteriaceae;g__Bifidobacterium;s__Bifidoba
d__Bacteria;p__Firmicutes;c__Clostridia;o__Clostridiales;f__Lachnospiraceae;g__Glucerabacter;s__Glucerabacter_canisensis
d__Bacteria;p__Chloroflexi;c__Anaerolineae;o__Caldilineales;f__Caldilineaceae;g__Caldilineaceae_unclassified;s__Caldilineac
d__Bacteria;p__Bacteroidota;c__Bacteroidia;o__Flavobacteriales;f__Crocinitomicaceae;g__Fluviicola;s__Fluviicola_unclassifie
d__Bacteria;p__Firmicutes;c__Bacilli;o__Lactobacillales;f__Streptococcaceae;g__Streptococcus;s__uncultured_Streptococcus_sp
d__Bacteria;p__Firmicutes;c__Bacilli;o__Bacillales;f__Bacillaceae;g__Bacillus;s__Bacillus_hisashii
d__Bacteria;p__Cyanobacteria;c__Cyanobacteria_unclassified;o__Cyanobacteria_unclassified;f__Cyanobacteria_unclassified;g__C

d__Bacteria;p__Proteobacteria;c__Alphaproteobacteria;o__Acetobacterales;f__Acetobacteraceae;g__Acetobacter;s__Acetobacter_u
d__Bacteria;p__Proteobacteria;c__Gammaproteobacteria;o__Enterobacterales;f__Erwiniaceae;g__Pantoea;s__Pantoea_agglomerans
d__Bacteria;p__Bacteroidota;c__Bacteroidia;o__Sphingobacteriales;f__Sphingobacteriaceae;g__Pedobacter;s__Pedobacter_unclass
d__Bacteria;p__Proteobacteria;c__Gammaproteobacteria;o__Burkholderiales;f__Rhodocyclaceae;g__Thauera;s__Thauera_unclassifie
d__Bacteria;p__Proteobacteria;c__Alphaproteobacteria;o__Rhizobiales;f__Beijerinckiaceae;g__Methylobacterium-Methylorubrum;s
d__Bacteria;p__Cyanobacteria;c__Cyanobacteriia;o__Chloroplast;f__Chloroplast_unclassified;g__Chloroplast_unclassified;s__Ch
d__Bacteria;p__Desulfobacterota;c__Desulfovibrionia;o__Desulfovibrionales;f__Desulfovibrionaceae;g__Desulfovibrio;s__Desulf
d__Bacteria;p__Firmicutes;c__Clostridia;o__Oscillospirales;f__Oscillospiraceae;g__UCG-002;s__UCG-002_unclassified
d__Bacteria;p__Proteobacteria;c__Alphaproteobacteria;o__Sphingomonadales;f__Sphingomonadaceae;g__Sphingopyxis;s__Sphingopyx
d__Bacteria;p__Desulfobacterota;c__Desulfovibrionia;o__Desulfovibrionales;f__Desulfovibrionaceae;g__Desulfovibrionaceae_unc
d__Bacteria;p__Proteobacteria;c__Gammaproteobacteria;o__Burkholderiales;f__Sutterellaceae;g__Parasutterella;s__Parasutterel
d__Bacteria;p__Proteobacteria;c__Gammaproteobacteria;o__Enterobacterales;f__Enterobacteriaceae;g__Klebsiella;s__uncultured_
d__Bacteria;p__Bacteroidota;c__Bacteroidia;o__Bacteroidales;f__Bacteroidaceae;g__Bacteroides;s__Bacteroides_unclassified
d__Bacteria;p__Firmicutes;c__Clostridia;o__Oscillospirales;f__Oscillospiraceae;g__Intestinimonas;s__Intestinimonas_unclassi
d__Bacteria;p__Bacteroidota;c__Bacteroidia;o__Bacteroidales;f__Bacteroidaceae;g__Bacteroides;s__Bacteroides_acidifaciens
d__Bacteria;p__Firmicutes;c__Clostridia;o__Clostridiales;f__Clostridiales_unclassified;g__Clostridiales_unclassified;s__Clo
d__Bacteria;p__Firmicutes;c__Clostridia;o__Oscillospirales;f__Ruminococcaceae;g__Ruminococcaceae_unclassified;s__Ruminococc
d__Bacteria;p__Proteobacteria;c__Alphaproteobacteria;o__Sphingomonadales;f__Sphingomonadaceae;g__Sphingopyxis;s__uncultured
d__Bacteria;p__Proteobacteria;c__Gammaproteobacteria;o__Xanthomonadales;f__Xanthomonadaceae;g__Stenotrophomonas;s__Stenotro
d__Bacteria;p__Proteobacteria;c__Betaproteobacteria;o__Burkholderiales;f__Comamonadaceae;g__Comamonadaceae_unclassified;s__
d__Bacteria;p__Bacteroidota;c__Bacteroidia;o__Cytophagales;f__Spirosomaceae;g__Emticicia;s__Emticicia_unclassified
d__Bacteria;p__Proteobacteria;c__Alphaproteobacteria;o__Alphaproteobacteria_unclassified;f__Alphaproteobacteria_unclassifie
d__Bacteria;p__Proteobacteria;c__Gammaproteobacteria;o__Burkholderiales;f__Nitrosomonadaceae;g__Ellin6067;s__Ellin6067_uncl
d__Bacteria;p__Firmicutes;c__Thermaerobacteria;o__Thermaerobacterales;f__Thermaerobacteraceae;g__Thermaerobacter;s__Thermae
d__Bacteria;p__Bacteroidota;c__Ignavibacteria;o__Ignavibacteriales;f__PHOS-HE36;g__PHOS-HE36_unclassified;s__PHOS-HE36_uncl
d__Bacteria;p__Firmicutes;c__Clostridia;o__Clostridiales;f__Lachnospiraceae;g__Lachnospiraceae_unclassified;s__Lachnospirac
d__Bacteria;p__Proteobacteria;c__Alphaproteobacteria;o__Rhizobiales;f__Stappiaceae;g__Stappiaceae_unclassified;s__Stappiace
d__Bacteria;p__Chloroflexi;c__Chloroflexia;o__Thermomicrobiales;f__JG30-KF-CM45;g__JG30-KF-CM45_unclassified;s__JG30-KF-CM4
d__Bacteria;p__Desulfobacterota;c__Desulfovibrionia;o__Desulfovibrionales;f__Desulfovibrionaceae;g__Bilophila;s__Bilophila_
d__Bacteria;p__Firmicutes;c__Clostridia;o__Clostridiales;f__Lachnospiraceae;g__Lachnospiraceae_unclassified;s__Lachnospirac
d__Bacteria;p__Firmicutes;c__Clostridia;o__Thermincolales;f__Thermincolaceae;g__Thermincola;s__Thermincola_unclassified
d__Bacteria;p__Proteobacteria;c__Alphaproteobacteria;o__Rhizobiales;f__Beijerinckiaceae;g__Methylobacterium-Methylorubrum;s
d__Bacteria;p__Elusimicrobiota;c__Elusimicrobia;o__Lineage_IV;f__Lineage_IV_unclassified;g__Lineage_IV_unclassified;s__Line
d__Bacteria;p__Gemmatimonadota;c__Gemmatimonadetes;o__Gemmatimonadales;f__Gemmatimonadaceae;g__Gemmatimonadaceae_unclassifi
d__Bacteria;p__Proteobacteria;c__Gammaproteobacteria;o__Pseudomonadales;f__Moraxellaceae;g__Acinetobacter;s__Acinetobacter_
d__Bacteria;p__Firmicutes;c__Clostridia;o__Clostridiales;f__Lachnospiraceae;g__Roseburia;s__Roseburia_sp._11SE38
d__Bacteria;p__Actinobacteriota;c__Actinobacteria;o__Micrococcales;f__Micrococcaceae;g__Rothia;s__Rothia_unclassified

d__Bacteria;p__Proteobacteria;c__Alphaproteobacteria;o__Parvibaculales;f__Parvibaculaceae;g__Parvibaculum;s__Parvibaculum_u
d__Bacteria;p__Firmicutes;c__Bacilli;o__Lactobacillales;f__Streptococcaceae;g__Streptococcus;s__Streptococcus_agalactiae
d__Bacteria;p__Firmicutes;c__Clostridia;o__Clostridiales;f__Clostridiales_unclassified;g__Clostridiales_unclassified;s__Clo
d__Bacteria;p__Firmicutes;c__Clostridia;o__Oscillospirales;f__Oscillospiraceae;g__Oscillibacter;s__Oscillibacter_unclassifi
d__Bacteria;p__Firmicutes;c__Bacilli;o__Bacillales;f__Bacillaceae;g__Bacillus;s__Bacillus_sp._BO7
d__Bacteria;p__Firmicutes;c__Clostridia;o__Christensenellales;f__Christensenellaceae;g__Christensenellaceae_R-7_group;s__Ch
d__Bacteria;p__Proteobacteria;c__Gammaproteobacteria;o__Burkholderiales;f__B1-7BS;g__B1-7BS_unclassified;s__B1-7BS_unclassi
d__Bacteria;p__Firmicutes;c__Clostridia;o__Oscillospirales;f__Ruminococcaceae;g__Fournierella;s__Fournierella_unclassified
d__Bacteria;p__Firmicutes;c__Bacilli;o__Brevibacillales;f__Brevibacillaceae;g__Brevibacillus;s__Brevibacillus_unclassified
d__Bacteria;p__Firmicutes;c__Clostridia;o__Lachnospirales;f__Lachnospiraceae;g__Blautia;s__Blautia_unclassified
d__Bacteria;p__Proteobacteria;c__Alphaproteobacteria;o__Rhodobacterales;f__Rhodobacteraceae;g__Rubellimicrobium;s__Rubellim
d__Bacteria;p__Firmicutes;c__Clostridia;o__Clostridiales;f__Lachnospiraceae;g__Lachnospiraceae_unclassified;s__Lachnospirac
d__Bacteria;p__Proteobacteria;c__Gammaproteobacteria;o__Xanthomonadales;f__Rhodanobacteraceae;g__Rhodanobacter;s__Rhodanoba
d__Bacteria;p__Proteobacteria;c__Betaproteobacteria;o__Burkholderiales;f__Comamonadaceae;g__Comamonas;s__uncultured_Comamon
d__Bacteria;p__Synergistota;c__Synergistia;o__Synergistales;f__Synergistaceae;g__Pyramidobacter;s__Pyramidobacter_piscolens
d__Bacteria;p__Proteobacteria;c__Gammaproteobacteria;o__Burkholderiales;f__Burkholderiaceae;g__Burkholderia-Caballeronia-Pa
d__Bacteria;p__Proteobacteria;c__Alphaproteobacteria;o__SAR11_clade;f__Clade_I;g__Clade_Ia;s__Clade_Ia_unclassified
d__Bacteria;p__Firmicutes;c__Bacilli;o__Bacillales;f__Bacillaceae;g__Geobacillus;s__uncultured_Geobacillus_sp.
d__Bacteria;p__Proteobacteria;c__Alphaproteobacteria;o__Rhodospirillales;f__Acetobacteraceae;g__Stella;s__uncultured_Stella
d__Bacteria;p__Proteobacteria;c__Gammaproteobacteria;o__Enterobacterales;f__Yersiniaceae;g__Serratia;s__Serratia_sp.
d__Bacteria;p__Firmicutes;c__Clostridia;o__Christensenellales;f__Christensenellaceae;g__Christensenellaceae_R-7_group;s__Ch
d__Bacteria;p__Proteobacteria;c__Gammaproteobacteria;o__Pseudomonadales;f__Moraxellaceae;g__Acinetobacter;s__uncultured_Aci
d__Bacteria;p__Bacteroidota;c__Bacteroidia;o__Bacteroidales;f__Prevotellaceae;g__Prevotella_9;s__Prevotella_9_unclassified
d__Bacteria;p__Bacteroidota;c__Bacteroidia;o__Bacteroidales;f__Bacteroidaceae;g__Bacteroides;s__uncultured_Bacteroides_sp.
d__Bacteria;p__Firmicutes;c__Negativicutes;o__Veillonellales-Selenomonadales;f__Veillonellaceae;g__Dialister;s__Dialister_u
d__Bacteria;p__Proteobacteria;c__Alphaproteobacteria;o__Rhizobiales;f__Xanthobacteraceae;g__Rhodopseudomonas;s__Rhodopseudo
d__Bacteria;p__Bacteroidota;c__Bacteroidia;o__Bacteroidales;f__Muribaculaceae;g__Muribaculaceae_unclassified;s__Muribaculac
d__Bacteria;p__Bacteroidota;c__Bacteroidia;o__Bacteroidales;f__Bacteroidaceae;g__Bacteroides;s__Bacteroides_fragilis
d__Bacteria;p__Firmicutes;c__Clostridia;o__Thermincolales;f__Thermincolaceae;g__Thermincola;s__Thermincola_unclassified
d__Bacteria;p__Proteobacteria;c__Betaproteobacteria;o__Burkholderiales;f__Comamonadaceae;g__Comamonas;s__uncultured_Comamon
d__Bacteria;p__Proteobacteria;c__Betaproteobacteria;o__Betaproteobacteria_unclassified;f__Betaproteobacteria_unclassified;g
d__Bacteria;p__Proteobacteria;c__Gammaproteobacteria;o__Pseudomonadales;f__Moraxellaceae;g__Acinetobacter;s__uncultured_Aci
d__Bacteria;p__Firmicutes;c__Bacilli;o__Brevibacillales;f__Brevibacillaceae;g__Brevibacillus;s__Brevibacillus_unclassified
d__Bacteria;p__Proteobacteria;c__Gammaproteobacteria;o__Burkholderiales;f__Oxalobacteraceae;g__Massilia;s__Massilia_unclass
d__Bacteria;p__Proteobacteria;c__Gammaproteobacteria;o__Xanthomonadales;f__Xanthomonadaceae;g__Stenotrophomonas;s__Stenotro
d__Bacteria;p__Proteobacteria;c__Alphaproteobacteria;o__SAR11_clade;f__Clade_III;g__Clade_III_unclassified;s__Clade_III_unc
d__Bacteria;p__Proteobacteria;c__Gammaproteobacteria;o__Burkholderiales;f__Alcaligenaceae;g__Achromobacter;s__Achromobacter

d__Bacteria;p__Proteobacteria;c__Gammaproteobacteria;o__Enterobacterales;f__Enterobacteriaceae;g__Citrobacter;s__Citrobacte
d__Bacteria;p__Cyanobacteria;c__Cyanobacteriia;o__Cyanobacteriales;f__Chroococcidiopsaceae;g__Aliterella;s__Aliterella_uncl
d__Bacteria;p__Proteobacteria;c__Gammaproteobacteria;o__Pseudomonadales;f__Halieaceae;g__OM60(NOR5)_clade;s__OM60(NOR5)_cla
d__Bacteria;p__Actinobacteriota;c__Actinobacteria;o__Actinomycetales;f__Actinomycetaceae;g__Actinomyces;s__Actinomyces_uncl
d__Bacteria;p__Firmicutes;c__Clostridia;o__Oscillospirales;f__Ruminococcaceae;g__Incertae_Sedis;s__Incertae_Sedis_unclassif
d__Bacteria;p__Proteobacteria;c__Gammaproteobacteria;o__Burkholderiales;f__SC-I-84;g__SC-I-84_unclassified;s__SC-I-84_uncla
d__Bacteria;p__Chloroflexi;c__Anaerolineae;o__RBG-13-54-9;f__RBG-13-54-9_unclassified;g__RBG-13-54-9_unclassified;s__RBG-13
d__Bacteria;p__Firmicutes;c__Clostridia;o__Clostridiales;f__Lachnospiraceae;g__Blautia;s__uncultured_Blautia_sp.
d__Bacteria;p__Cyanobacteria;c__Cyanobacteriia;o__Chloroplast;f__Chloroplast_unclassified;g__Chloroplast_unclassified;s__Ch
d__Bacteria;p__Actinobacteriota;c__Actinobacteria;o__Bifidobacteriales;f__Bifidobacteriaceae;g__Bifidobacterium;s__Bifidoba
d__Bacteria;p__Actinobacteriota;c__Actinobacteria;o__Micrococcales;f__Micrococcaceae;g__Rothia;s__Rothia_unclassified
d__Bacteria;p__Actinobacteriota;c__Actinobacteria;o__Corynebacteriales;f__Mycobacteriaceae;g__Mycobacterium;s__Mycobacteriu
d__Bacteria;p__Firmicutes;c__Clostridia;o__Oscillospirales;f__Ruminococcaceae;g__Faecalibacterium;s__Faecalibacterium_uncla
d__Bacteria;p__Firmicutes;c__Thermincolia;o__Carboxydocellales;f__Carboxydocellaceae;g__Carboxydocella;s__Carboxydocella_un
d__Bacteria;p__Firmicutes;c__Clostridia;o__Oscillospirales;f__Ruminococcaceae;g__Subdoligranulum;s__Subdoligranulum_unclass
d__Bacteria;p__Firmicutes;c__Clostridia;o__Clostridiales;f__Clostridiaceae;g__Clostridium;s__Clostridium_sp._AT4
d__Bacteria;p__Firmicutes;c__Bacilli;o__Lactobacillales;f__Enterococcaceae;g__Enterococcus;s__Enterococcus_faecium
d__Bacteria;p__Proteobacteria;c__Gammaproteobacteria;o__Burkholderiales;f__Burkholderiaceae;g__Limnobacter;s__Limnobacter_u
d__Bacteria;p__Firmicutes;c__Bacilli;o__Bacillales;f__Bacillaceae;g__Aeribacillus;s__Aeribacillus_unclassified
d__Bacteria;p__Proteobacteria;c__Alphaproteobacteria;o__Rhizobiales;f__Rhizobiaceae;g__Rhizobium;s__Rhizobium_rhizoryzae
d__Bacteria;p__Firmicutes;c__Clostridia;o__Clostridiales;f__Lachnospiraceae;g__Lachnospiraceae_unclassified;s__Lachnospirac
d__Bacteria;p__Firmicutes;c__Bacilli;o__Lactobacillales;f__Enterococcaceae;g__Enterococcus;s__Enterococcus_gallinarum
d__Bacteria;p__Proteobacteria;c__Gammaproteobacteria;o__Enterobacterales;f__Enterobacteriaceae;g__Klebsiella;s__Klebsiella_
d__Bacteria;p__Firmicutes;c__Clostridia;o__Oscillospirales;f__Eubacterium]_coprostanoligenes_group;g__Eubacterium]_coprosta
d__Bacteria;p__Desulfobacterota;c__Desulfovibrionia;o__Desulfovibrionales;f__Desulfovibrionaceae;g__Desulfovibrionaceae_unc
d__Bacteria;p__Proteobacteria;c__Alphaproteobacteria;o__Sphingomonadales;f__Sphingomonadaceae;g__Sphingopyxis;s__Sphingopyx
d__Bacteria;p__Firmicutes;c__Clostridia;o__Thermincolales;f__Thermincolaceae;g__Thermincola;s__Thermincola_unclassified
d__Bacteria;p__Proteobacteria;c__Gammaproteobacteria;o__Enterobacterales;f__Aeromonadaceae;g__Aeromonas;s__Aeromonas_unclas
d__Bacteria;p__Bacteroidota;c__Bacteroidia;o__Bacteroidales;f__Prevotellaceae;g__Prevotella_9;s__Prevotella_9_unclassified
d__Bacteria;p__Firmicutes;c__Bacilli;o__Lactobacillales;f__Enterococcaceae;g__Enterococcus;s__Enterococcus_faecium
d__Bacteria;p__Proteobacteria;c__Alphaproteobacteria;o__Rhodobacterales;f__Rhodobacteraceae;g__Paracoccus;s__Paracoccus_mar
d__Bacteria;p__Firmicutes;c__Bacilli;o__Lactobacillales;f__Streptococcaceae;g__Streptococcus;s__Streptococcus_sp._oral_clon
d__Bacteria;p__Firmicutes;c__Bacilli;o__Lactobacillales;f__Streptococcaceae;g__Streptococcus;s__Streptococcus_sp.
d__Bacteria;p__Firmicutes;c__Clostridia;o__Peptostreptococcales-Tissierellales;f__Peptostreptococcaceae;g__Clostridioides;s
d__Bacteria;p__Firmicutes;c__Clostridia;o__Lachnospirales;f__Lachnospiraceae;g__ASF356;s__ASF356_unclassified
d__Bacteria;p__Firmicutes;c__Negativicutes;o__Veillonellales-Selenomonadales;f__Veillonellaceae;g__Megasphaera;s__Megasphae
d__Bacteria;p__Firmicutes;c__Clostridia;o__Clostridiales;f__Clostridiaceae;g__Clostridium;s__Clostridium_sp._Clone-49

d__Bacteria;p__Bacteroidota;c__Bacteroidia;o__Bacteroidales;f__Prevotellaceae;g__Prevotella_9;s__Prevotella_9_unclassified
d__Bacteria;p__Firmicutes;c__Bacilli;o__Bacillales;f__Bacillaceae;g__Geobacillus;s__Geobacillus_unclassified
d__Bacteria;p__Bacteroidota;c__Bacteroidia;o__Flavobacteriales;f__Cryomorphaceae;g__Owenweeksia;s__Owenweeksia_unclassified
d__Bacteria;p__Cyanobacteria;c__Cyanobacteriia;o__Chloroplast;f__Chloroplast_unclassified;g__Chloroplast_unclassified;s__Ch
d__Bacteria;p__Proteobacteria;c__Alphaproteobacteria;o__Rickettsiales;f__Mitochondria;g__Mitochondria_unclassified;s__Mitoc
d__Bacteria;p__Fusobacteriota;c__Fusobacteriia;o__Fusobacteriales;f__Fusobacteriaceae;g__Fusobacterium;s__Fusobacterium_unc
d__Bacteria;p__Firmicutes;c__Negativicutes;o__Veillonellales-Selenomonadales;f__Selenomonadaceae;g__Megamonas;s__Megamonas_
d__Bacteria;p__Firmicutes;c__Thermovenabulia;o__Thermovenabulales;f__Thermovenabulales_unclassified;g__Thermovenabulales_un
d__Bacteria;p__Bacteroidota;c__Bacteroidia;o__Bacteroidales;f__Rikenellaceae;g__Alistipes;s__Alistipes_sp._Marseille-P5061
d__Bacteria;p__Firmicutes;c__Clostridia;o__Lachnospirales;f__Lachnospiraceae;g__Shuttleworthia;s__Shuttleworthia_unclassifi
d__Bacteria;p__Firmicutes;c__Bacilli;o__Bacillales;f__Bacillaceae;g__Aeribacillus;s__Aeribacillus_pallidus
d__Bacteria;p__Proteobacteria;c__Gammaproteobacteria;o__Burkholderiales;f__Methylophilaceae;g__OM43_clade;s__OM43_clade_unc
d__Bacteria;p__Proteobacteria;c__Alphaproteobacteria;o__Puniceispirillales;f__SAR116_clade;g__SAR116_clade_unclassified;s__
d__Bacteria;p__Proteobacteria;c__Gammaproteobacteria;o__Burkholderiales;f__Comamonadaceae;g__Ideonella;s__Ideonella_unclass
d__Bacteria;p__Firmicutes;c__Clostridia;o__Peptostreptococcales-Tissierellales;f__Peptostreptococcaceae;g__Romboutsia;s__Ro
d__Bacteria;p__Proteobacteria;c__Betaproteobacteria;o__Betaproteobacteria_unclassified;f__Betaproteobacteria_unclassified;g
d__Bacteria;p__Bacteroidota;c__Bacteroidia;o__Bacteroidales;f__Rikenellaceae;g__Alistipes;s__Alistipes_unclassified
d__Bacteria;p__Proteobacteria;c__Gammaproteobacteria;o__Burkholderiales;f__Rhodocyclaceae;g__Dechloromonas;s__Dechloromonas
d__Bacteria;p__Bacteroidota;c__Bacteroidia;o__Flavobacteriales;f__Flavobacteriaceae;g__Flavobacterium;s__Flavobacterium_unc
d__Bacteria;p__Proteobacteria;c__Gammaproteobacteria;o__Steroidobacterales;f__Steroidobacteraceae;g__Steroidobacteraceae_un
d__Bacteria;p__Cyanobacteria;c__Cyanobacteriia;o__Chloroplast;f__Chloroplast_unclassified;g__Chloroplast_unclassified;s__Ch
d__Bacteria;p__Firmicutes;c__Clostridia;o__Lachnospirales;f__Lachnospiraceae;g__Hungatella;s__Hungatella_unclassified
d__Bacteria;p__Firmicutes;c__Negativicutes;o__Veillonellales-Selenomonadales;f__Selenomonadaceae;g__Megamonas;s__Megamonas_
d__Bacteria;p__Firmicutes;c__Firmicutes_unclassified;o__Firmicutes_unclassified;f__Firmicutes_unclassified;g__Firmicutes_un
d__Bacteria;p__Proteobacteria;c__Gammaproteobacteria;o__Cardiobacteriales;f__Wohlfahrtiimonadaceae;g__Wohlfahrtiimonas;s__W
d__Bacteria;p__Bacteroidota;c__Bacteroidia;o__Sphingobacteriales;f__Sphingobacteriaceae;g__Pedobacter;s__Pedobacter_unclass
d__Bacteria;p__Proteobacteria;c__Gammaproteobacteria;o__Enterobacterales;f__Enterobacteriaceae;g__Enterobacter;s__Enterobac
d__Bacteria;p__Proteobacteria;c__Gammaproteobacteria;o__Burkholderiales;f__Hydrogenophilaceae;g__Tepidiphilus;s__Tepidiphil
d__Bacteria;p__Proteobacteria;c__Gammaproteobacteria;o__Acidithiobacillales;f__Acidithiobacillaceae;g__Acidithiobacillus;s_
d__Bacteria;p__Proteobacteria;c__Gammaproteobacteria;o__Burkholderiales;f__Comamonadaceae;g__Hydrogenophaga;s__Hydrogenopha
d__Bacteria;p__Firmicutes;c__Bacilli;o__Erysipelotrichales;f__Erysipelatoclostridiaceae;g__Erysipelotrichaceae_UCG-003;s__E
d__Bacteria;p__Proteobacteria;c__Alphaproteobacteria;o__Sphingomonadales;f__Sphingomonadaceae;g__Sphingopyxis;s__uncultured
d__Bacteria;p__Proteobacteria;c__Alphaproteobacteria;o__Sphingomonadales;f__Sphingomonadaceae;g__Sphingopyxis;s__Sphingopyx
d__Bacteria;p__Firmicutes;c__Negativicutes;o__Veillonellales-Selenomonadales;f__Selenomonadaceae;g__Megamonas;s__Megamonas_
d__Bacteria;p__Bacteroidota;c__Bacteroidia;o__Bacteroidales;f__Muribaculaceae;g__Muribaculaceae_unclassified;s__Muribaculac
d__Bacteria;p__Firmicutes;c__Clostridia;o__Lachnospirales;f__Lachnospiraceae;g__Acetatifactor;s__Acetatifactor_unclassified
d__Bacteria;p__Proteobacteria;c__Betaproteobacteria;o__Burkholderiales;f__Comamonadaceae;g__Comamonas;s__uncultured_Comamon

```
d__Bacteria;p__Bacteroidota;c__Bacteroidota_unclassified;o__Bacteroidota_unclassified;f__Bacteroidota_unclassified;g__Bacte
d__Bacteria;p__Actinobacteriota;c__Actinobacteriota;o__Propionibacteriales;f__Propionibacteriaceae;g__Propionibacterium;s__
d__Bacteria;p__Proteobacteria;c__Gammaproteobacteria;o__Pseudomonadales;f__Pseudohongiellaceae;g__Pseudohongiella;s__Pseudo
d__Bacteria;p__Proteobacteria;c__Gammaproteobacteria;o__Enterobacterales;f__Enterobacteriaceae;g__Cronobacter;s__Cronobacte
d__Bacteria;p__Actinobacteriota;c__Actinobacteria;o__Bifidobacteriales;f__Bifidobacteriaceae;g__Bifidobacterium;s__Bifidoba
d__Bacteria;p__Firmicutes;c__Clostridia;o__Clostridia_UCG-014;f__Clostridia_UCG-014_unclassified;g__Clostridia_UCG-014_uncl
d__Bacteria;p__Bacteroidota;c__Bacteroidia;o__Flavobacteriales;f__Cryomorphaceae;g__Cryomorphaceae_unclassified;s__Cryomorp
d__Bacteria;p__Proteobacteria;c__Alphaproteobacteria;o__Sphingomonadales;f__Sphingomonadaceae;g__Sphingomonas;s__Sphingomon
d__Bacteria;p__Actinobacteriota;c__Acidimicrobiia;o__Actinomarinales;f__Actinomarinaceae;g__Candidatus_Actinomarina;s__Cand
d__Bacteria;p__Firmicutes;c__Clostridia;o__Oscillospirales;f__Butyricicoccaceae;g__Butyricicoccus;s__Butyricicoccus_unclass
d__Bacteria;p__Firmicutes;c__Bacilli;o__Bacillales;f__Bacillaceae;g__Bacillus;s__uncultured_Bacillus_sp.
d__Bacteria;p__Firmicutes;c__Clostridia;o__Oscillospirales;f__Ruminococcaceae;g__Fournierella;s__Fournierella_unclassified
d__Bacteria;p__Firmicutes;c__Clostridia;o__Oscillospirales;f__Eubacterium]_coprostanoligenes_group;g__Eubacterium]_coprosta
d__Bacteria;p__Proteobacteria;c__Gammaproteobacteria;o__Burkholderiales;f__Comamonadaceae;g__Ottowia;s__Ottowia_unclassifie
d__Bacteria;p__Firmicutes;c__Clostridia;o__Oscillospirales;f__Ruminococcaceae;g__Negativibacillus;s__Negativibacillus_uncla
d__unclassified;p__unclassified;c__unclassified;o__unclassified;f__unclassified;g__unclassified;s__unclassified
d__Bacteria;p__Proteobacteria;c__Alphaproteobacteria;o__Rhizobiales;f__Rhizobiaceae;g__Neorhizobium;s__Neorhizobium_alkalis
d__Bacteria;p__Planctomycetota;c__Planctomycetes;o__Planctomycetales;f__Rubinisphaeraceae;g__SH-PL14;s__SH-PL14_unclassifie
d__Bacteria;p__Chloroflexi;c__Anaerolineae;o__1-20;f__1-20_unclassified;g__1-20_unclassified;s__1-20_unclassified
d__Bacteria;p__Verrucomicrobiota;c__Chlamydiae;o__Chlamydiales;f__Parachlamydiaceae;g__Neochlamydia;s__Neochlamydia_unclass
d__Bacteria;p__Firmicutes;c__Clostridia;o__Oscillospirales;f__Ruminococcaceae;g__Incertae_Sedis;s__Incertae_Sedis_unclassif
d__Bacteria;p__Bacteroidota;c__Bacteroidia;o__Bacteroidales;f__Tannerellaceae;g__Parabacteroides;s__Parabacteroides_distaso
d__Bacteria;p__Firmicutes;c__Bacilli;o__Bacillales;f__Bacillaceae;g__Bacillus;s__Bacillus_anthracis
d__Bacteria;p__Bacteroidota;c__Bacteroidia;o__Bacteroidales;f__Barnesiellaceae;g__Barnesiella;s__Barnesiella_unclassified
d__Bacteria;p__Proteobacteria;c__Gammaproteobacteria;o__Pseudomonadales;f__Pseudomonadaceae;g__Pseudomonas;s__Pseudomonas_s
d__Bacteria;p__Firmicutes;c__Clostridia;o__Lachnospirales;f__Lachnospiraceae;g__Blautia;s__Blautia_unclassified
d__Bacteria;p__Proteobacteria;c__Gammaproteobacteria;o__Burkholderiales;f__Burkholderiaceae;g__Ralstonia;s__Ralstonia_uncla
d__Bacteria;p__Actinobacteriota;c__Actinobacteria;o__Corynebacteriales;f__Mycobacteriaceae;g__Mycobacterium;s__Mycobacteriu
d__Bacteria;p__Proteobacteria;c__Alphaproteobacteria;o__Sphingomonadales;f__Sphingomonadaceae;g__Sphingopyxis;s__Sphingopyx
d__Bacteria;p__Proteobacteria;c__Deltaproteobacteria;o__Deltaproteobacteria_unclassified;f__Deltaproteobacteria_unclassifie
d__Bacteria;p__Proteobacteria;c__Alphaproteobacteria;o__Acetobacterales;f__Acetobacteraceae;g__Roseomonas;s__Roseomonas_unc
d__Bacteria;p__Bacteroidota;c__Bacteroidia;o__Flavobacteriales;f__Weeksellaceae;g__Chryseobacterium;s__Chryseobacterium_unc
d__Bacteria;p__Bacteroidota;c__Bacteroidia;o__Bacteroidales;f__Prevotellaceae;g__Prevotella_9;s__Prevotella_9_unclassified
d__Bacteria;p__Firmicutes;c__Negativicutes;o__Acidaminococcales;f__Acidaminococcaceae;g__Phascolarctobacterium;s__Phascolar
d__Bacteria;p__Firmicutes;c__Negativicutes;o__Veillonellales-Selenomonadales;f__Selenomonadaceae;g__Megamonas;s__Megamonas_
d__Bacteria;p__Proteobacteria;c__Gammaproteobacteria;o__Burkholderiales;f__Oxalobacteraceae;g__Massilia;s__Massilia_unclass
d__Bacteria;p__Desulfobacterota;c__Desulfovibrionia;o__Desulfovibrionales;f__Desulfovibrionaceae;g__Desulfovibrionaceae_unc
```

d__Bacteria;p__Proteobacteria;c__Alphaproteobacteria;o__Rhizobiales;f__Bradyrhizobiaceae;g__Bradyrhizobium;s__Bradyrhizobiu
d__Bacteria;p__Firmicutes;c__Clostridia;o__Oscillospirales;f__Eubacterium]_coprostanoligenes_group;g__Eubacterium]_coprosta
d__Bacteria;p__Proteobacteria;c__Gammaproteobacteria;o__Pseudomonadales;f__Moraxellaceae;g__Acinetobacter;s__Acinetobacter_
d__Bacteria;p__Actinobacteriota;c__Actinobacteria;o__Corynebacteriales;f__Corynebacteriaceae;g__Corynebacterium;s__Coryneba
d__Bacteria;p__Firmicutes;c__Clostridia;o__Oscillospirales;f__Ruminococcaceae;g__Anaerotruncus;s__Anaerotruncus_unclassifie
d__Bacteria;p__Bacteroidota;c__Bacteroidia;o__Bacteroidales;f__Prevotellaceae;g__Prevotella_9;s__Prevotella_9_unclassified
d__Bacteria;p__Firmicutes;c__Clostridia;o__Lachnospirales;f__Lachnospiraceae;g__Lachnospiraceae_NK4A136_group;s__Lachnospir
d__Bacteria;p__Firmicutes;c__Clostridia;o__Oscillospirales;f__Oscillospiraceae;g__Oscillibacter;s__Oscillibacter_unclassifi
d__Bacteria;p__Proteobacteria;c__Alphaproteobacteria;o__Sphingomonadales;f__Sphingomonadaceae;g__Sphingopyxis;s__Sphingopyx
d__Bacteria;p__Firmicutes;c__Bacilli;o__Lactobacillales;f__Lactobacillaceae;g__Lactobacillus;s__uncultured_Lactobacillus_sp
d__Bacteria;p__Proteobacteria;c__Alphaproteobacteria;o__Sphingomonadales;f__Sphingomonadaceae;g__Sphingobium;s__Sphingobium
d__Bacteria;p__Proteobacteria;c__Gammaproteobacteria;o__Legionellales;f__Legionellaceae;g__Legionella;s__uncultured_Legione
d__Bacteria;p__Actinobacteriota;c__Actinobacteria;o__Micrococcales;f__Microbacteriaceae;g__Leucobacter;s__Leucobacter_uncla
d__Bacteria;p__Firmicutes;c__Clostridia;o__Oscillospirales;f__Ruminococcaceae;g__Ruminococcus;s__Ruminococcus_unclassified
d__Bacteria;p__Bacteroidota;c__Bacteroidia;o__Flavobacteriales;f__NS9_marine_group;g__NS9_marine_group_unclassified;s__NS9_
d__Bacteria;p__Firmicutes;c__Bacilli;o__Bacillales;f__Bacillaceae;g__Bacillus;s__Bacillus_sp._MT03
d__Bacteria;p__Proteobacteria;c__Betaproteobacteria;o__Burkholderiales;f__Comamonadaceae;g__Acidovorax;s__Acidovorax_sp._N1
d__Bacteria;p__Firmicutes;c__Clostridia;o__Clostridia_UCG-014;f__Clostridia_UCG-014_unclassified;g__Clostridia_UCG-014_uncl
d__Bacteria;p__Firmicutes;c__Bacilli;o__Lactobacillales;f__Lactobacillaceae;g__Ligilactobacillus;s__Ligilactobacillus_uncla
d__Bacteria;p__Proteobacteria;c__Gammaproteobacteria;o__Burkholderiales;f__Methylophilaceae;g__OM43_clade;s__OM43_clade_unc
d__Bacteria;p__Proteobacteria;c__Gammaproteobacteria;o__Pseudomonadales;f__Halieaceae;g__OM60(NOR5)_clade;s__OM60(NOR5)_cla
d__Bacteria;p__Actinobacteriota;c__Actinobacteria;o__Micromonosporales;f__Micromonosporaceae;g__Micromonosporaceae_unclassi
d__Bacteria;p__Proteobacteria;c__Betaproteobacteria;o__Burkholderiales;f__Comamonadaceae;g__Acidovorax;s__Acidovorax_sp.
d__Bacteria;p__Cyanobacteria;c__Cyanobacteriia;o__Chloroplast;f__Chloroplast_unclassified;g__Chloroplast_unclassified;s__Ch
d__Bacteria;p__Proteobacteria;c__Gammaproteobacteria;o__Burkholderiales;f__Burkholderiaceae;g__Polynucleobacter;s__Polynucl
d__Bacteria;p__Firmicutes;c__Bacilli;o__Bacillales;f__Bacillaceae;g__Anoxybacillus;s__Anoxybacillus_sp._XA-1
d__Bacteria;p__Firmicutes;c__Clostridia;o__Clostridiales;f__Ruminococcaceae;g__Ruminococcaceae_unclassified;s__Ruminococcac
d__Bacteria;p__Firmicutes;c__Bacilli;o__Brevibacillales;f__Brevibacillaceae;g__Brevibacillus;s__Brevibacillus_unclassified
d__Bacteria;p__Firmicutes;c__Bacilli;o__Exiguobacterales;f__Exiguobacteraceae;g__Exiguobacterium;s__Exiguobacterium_unclass
d__Bacteria;p__Bacteroidota;c__Bacteroidia;o__Flavobacteriales;f__Flavobacteriaceae;g__NS5_marine_group;s__NS5_marine_group
d__Bacteria;p__Proteobacteria;c__Gammaproteobacteria;o__Pseudomonadales;f__Porticoccaceae;g__SAR92_clade;s__SAR92_clade_unc
d__Bacteria;p__MBNT15;c__MBNT15_unclassified;o__MBNT15_unclassified;f__MBNT15_unclassified;g__MBNT15_unclassified;s__MBNT15
d__Bacteria;p__Proteobacteria;c__Betaproteobacteria;o__Burkholderiales;f__Burkholderiales_unclassified;g__Burkholderiales_u
d__Bacteria;p__Actinobacteriota;c__Actinobacteria;o__Corynebacteriales;f__Corynebacteriaceae;g__Corynebacterium;s__Coryneba
d__Bacteria;p__Proteobacteria;c__Alphaproteobacteria;o__Rickettsiales;f__Mitochondria;g__Mitochondria_unclassified;s__Mitoc
d__Bacteria;p__Firmicutes;c__Clostridia;o__Lachnospirales;f__Lachnospiraceae;g__Sellimonas;s__Sellimonas_unclassified
d__Bacteria;p__Proteobacteria;c__Alphaproteobacteria;o__Sphingomonadales;f__Sphingomonadaceae;g__Novosphingobium;s__Novosph

d__Bacteria;p__Proteobacteria;c__Alphaproteobacteria;o__Sphingomonadales;f__Sphingomonadaceae;g__Sphingomonas;s__uncultured
d__Bacteria;p__Firmicutes;c__Thermovenabulia;o__Thermovenabulales;f__Thermovenabulales_unclassified;g__Thermovenabulales_un
d__Bacteria;p__Actinobacteriota;c__Actinobacteria;o__Propionibacteriales;f__Nocardioidaceae;g__Nocardioides;s__Nocardioides
d__Bacteria;p__Firmicutes;c__Bacilli;o__Lactobacillales;f__Lactobacillaceae;g__Companilactobacillus;s__Companilactobacillus
d__Bacteria;p__Firmicutes;c__Bacilli;o__Lactobacillales;f__Lactobacillaceae;g__Lactobacillus;s__Lactobacillus_manihotivoran
d__Bacteria;p__Actinobacteriota;c__Actinobacteria;o__PeM15;f__PeM15_unclassified;g__PeM15_unclassified;s__PeM15_unclassifie
d__Bacteria;p__Actinobacteriota;c__Actinobacteria;o__Micrococcales;f__Microbacteriaceae;g__Humibacter;s__Humibacter_unclass
d__Bacteria;p__Chloroflexi;c__Chloroflexia;o__Thermomicrobiales;f__JG30-KF-CM45;g__JG30-KF-CM45_unclassified;s__JG30-KF-CM4
d__Bacteria;p__Proteobacteria;c__Gammaproteobacteria;o__Enterobacterales;f__Enterobacteriaceae;g__Enterobacter;s__unculture
d__unclassified;p__unclassified;c__unclassified;o__unclassified;f__unclassified;g__unclassified;s__unclassified
d__Bacteria;p__Bacteroidota;c__Bacteroidia;o__Cytophagales;f__Hymenobacteraceae;g__Hymenobacter;s__Hymenobacter_unclassifie
d__Bacteria;p__Proteobacteria;c__Gammaproteobacteria;o__Burkholderiales;f__Rhodocyclaceae;g__Dechlorosoma;s__Dechlorosoma_u
d__Bacteria;p__Proteobacteria;c__Gammaproteobacteria;o__Pseudomonadales;f__Moraxellaceae;g__Acinetobacter;s__Acinetobacter_
d__Bacteria;p__Proteobacteria;c__Alphaproteobacteria;o__Rhizobiales;f__Rhizobiaceae;g__Rhizobium;s__uncultured_Rhizobium_sp
d__Bacteria;p__Proteobacteria;c__Gammaproteobacteria;o__Enterobacterales;f__Alteromonadaceae;g__Rheinheimera;s__Rheinheimer
d__Bacteria;p__Proteobacteria;c__Betaproteobacteria;o__Burkholderiales;f__Comamonadaceae;g__Comamonas;s__Comamonas_terrigen
d__Bacteria;p__Proteobacteria;c__Gammaproteobacteria;o__Burkholderiales;f__Rhodocyclaceae;g__Dechlorosoma;s__Dechlorosoma_u
d__Bacteria;p__Bacteroidota;c__Bacteroidia;o__Bacteroidales;f__Prevotellaceae;g__Prevotella_9;s__Prevotella_9_unclassified
d__Bacteria;p__Firmicutes;c__Bacilli;o__Lactobacillales;f__Enterococcaceae;g__Enterococcus;s__Enterococcus_faecium
d__Bacteria;p__Firmicutes;c__Negativicutes;o__Veillonellales-Selenomonadales;f__Veillonellaceae;g__Dialister;s__Dialister_u
d__Bacteria;p__Actinobacteriota;c__Actinobacteria;o__Corynebacteriales;f__Dietziaceae;g__Dietzia;s__Dietzia_unclassified
d__Bacteria;p__Firmicutes;c__Negativicutes;o__Veillonellales-Selenomonadales;f__Selenomonadaceae;g__Megamonas;s__Megamonas_
d__Bacteria;p__Firmicutes;c__Bacilli;o__Lactobacillales;f__Streptococcaceae;g__Streptococcus;s__uncultured_Streptococcus_sp
d__Bacteria;p__Proteobacteria;c__Alphaproteobacteria;o__Rhizobiales;f__Beijerinckiaceae;g__Bosea;s__Bosea_unclassified
d__Bacteria;p__Bacteroidota;c__Bacteroidia;o__Cytophagales;f__Hymenobacteraceae;g__Adhaeribacter;s__Adhaeribacter_unclassif
d__Bacteria;p__Actinobacteriota;c__Coriobacteriia;o__Eggerthellales;f__Eggerthellaceae;g__Enterorhabdus;s__Enterorhabdus_sp
d__Bacteria;p__Firmicutes;c__Clostridia;o__Clostridiales;f__Clostridiaceae;g__Clostridium;s__Clostridium_sp._Clone-17
d__Bacteria;p__Firmicutes;c__Firmicutes_unclassified;o__Firmicutes_unclassified;f__Firmicutes_unclassified;g__Firmicutes_un
d__Bacteria;p__Proteobacteria;c__Betaproteobacteria;o__Burkholderiales;f__Comamonadaceae;g__Comamonas;s__Comamonas_terrigen
d__Bacteria;p__Campylobacterota;c__Campylobacteria;o__Campylobacterales;f__Helicobacteraceae;g__Helicobacter;s__Helicobacte
d__Bacteria;p__Firmicutes;c__Negativicutes;o__Veillonellales-Selenomonadales;f__Veillonellaceae;g__Dialister;s__Dialister_u
d__Bacteria;p__Firmicutes;c__Negativicutes;o__Veillonellales-Selenomonadales;f__Veillonellaceae;g__Veillonella;s__Veillonel
d__Bacteria;p__Bacteroidota;c__Bacteroidia;o__Bacteroidales;f__Bacteroidaceae;g__Bacteroides;s__uncultured_Bacteroides_sp.
d__Bacteria;p__Firmicutes;c__Clostridia;o__Clostridia_UCG-014;f__Clostridia_UCG-014_unclassified;g__Clostridia_UCG-014_uncl
d__Bacteria;p__Firmicutes;c__Clostridia;o__Lachnospirales;f__Lachnospiraceae;g__Blautia;s__Blautia_unclassified
d__Bacteria;p__Proteobacteria;c__Gammaproteobacteria;o__Xanthomonadales;f__Xanthomonadaceae;g__Stenotrophomonas;s__Stenotro
d__Bacteria;p__Firmicutes;c__Bacilli;o__Bacillales;f__Bacillaceae;g__Virgibacillus;s__uncultured_Virgibacillus_sp.

d__Bacteria;p__Firmicutes;c__Clostridia;o__Clostridiales;f__Lachnospiraceae;g__Lachnospiraceae_unclassified;s__Lachnospirac
d__Bacteria;p__Firmicutes;c__Bacilli;o__Lactobacillales;f__Lactobacillaceae;g__Ligilactobacillus;s__Ligilactobacillus_uncla
d__Bacteria;p__Bacteroidota;c__Bacteroidia;o__Bacteroidales;f__Rikenellaceae;g__Rikenella;s__Rikenella_unclassified
d__Bacteria;p__Proteobacteria;c__Gammaproteobacteria;o__Ectothiorhodospirales;f__Ectothiorhodospiraceae;g__Ectothiorhodospi
d__Bacteria;p__Proteobacteria;c__Gammaproteobacteria;o__Xanthomonadales;f__Xanthomonadaceae;g__Lysobacter;s__Lysobacter_unc
d__Bacteria;p__Proteobacteria;c__Gammaproteobacteria;o__Enterobacterales;f__Colwelliaceae;g__Colwellia;s__Colwellia_unclass
d__Bacteria;p__Bacteroidota;c__Bacteroidia;o__Bacteroidales;f__Rikenellaceae;g__Alistipes;s__Alistipes_unclassified
d__Bacteria;p__Firmicutes;c__D8A-2;o__D8A-2_unclassified;f__D8A-2_unclassified;g__D8A-2_unclassified;s__D8A-2_unclassified
d__Bacteria;p__Firmicutes;c__Clostridia;o__Oscillospirales;f__Oscillospiraceae;g__UCG-005;s__UCG-005_unclassified
d__Bacteria;p__Proteobacteria;c__Betaproteobacteria;o__Betaproteobacteria_unclassified;f__Betaproteobacteria_unclassified;g
d__Bacteria;p__Proteobacteria;c__Gammaproteobacteria;o__Pseudomonadales;f__Pseudomonadaceae;g__Pseudomonas;s__uncultured_Ps
d__Bacteria;p__Firmicutes;c__Clostridia;o__Clostridiales;f__Clostridiaceae;g__Clostridiaceae_unclassified;s__Clostridiaceae
d__Bacteria;p__Bacteroidota;c__Bacteroidia;o__Sphingobacteriales;f__Sphingobacteriaceae;g__Solitalea;s__Solitalea_unclassif
d__Bacteria;p__Firmicutes;c__Clostridia;o__Clostridiales;f__Lachnospiraceae;g__Roseburia;s__uncultured_Roseburia_sp.
d__Bacteria;p__Firmicutes;c__Clostridia;o__Clostridia_UCG-014;f__Clostridia_UCG-014_unclassified;g__Clostridia_UCG-014_uncl
d__Bacteria;p__Firmicutes;c__Bacilli;o__Bacillales;f__Bacillaceae;g__Caldibacillus;s__Caldibacillus_debilis
d__Bacteria;p__Proteobacteria;c__Alphaproteobacteria;o__Rhizobiales;f__Rhizobiaceae;g__Aureimonas;s__Aureimonas_unclassifie
d__Bacteria;p__Chlamydiae;c__Chlamydiia;o__Chlamydiales;f__Chlamydiales_unclassified;g__Chlamydiales_unclassified;s__Chlamy
d__Bacteria;p__Firmicutes;c__Bacilli;o__Lactobacillales;f__Streptococcaceae;g__Streptococcus;s__uncultured_Streptococcus_sp
d__Bacteria;p__Firmicutes;c__Bacilli;o__Staphylococcales;f__Staphylococcaceae;g__Staphylococcus;s__Staphylococcus_unclassif
d__Bacteria;p__Actinobacteriota;c__Actinobacteria;o__Corynebacteriales;f__Corynebacteriaceae;g__Corynebacterium;s__Coryneba
d__Bacteria;p__Firmicutes;c__Clostridia;o__Oscillospirales;f__Butyricicoccaceae;g__Butyricicoccus;s__Butyricicoccus_unclass
d__Bacteria;p__Firmicutes;c__Bacilli;o__Bacillales;f__Bacillaceae;g__Bacillus;s__Bacillus_sp._MO-02
d__Bacteria;p__Proteobacteria;c__Gammaproteobacteria;o__Burkholderiales;f__Oxalobacteraceae;g__Undibacterium;s__Undibacteri
d__Bacteria;p__Firmicutes;c__Bacilli;o__Thermoactinomycetales;f__Thermoactinomycetaceae;g__Thermoactinomyces;s__Thermoactin
d__Bacteria;p__Proteobacteria;c__Alphaproteobacteria;o__Rhodobacterales;f__Rhodobacteraceae;g__Ascidiaceihabitans;s__Ascidi
d__Bacteria;p__Firmicutes;c__Clostridia;o__Clostridia_UCG-014;f__Clostridia_UCG-014_unclassified;g__Clostridia_UCG-014_uncl
d__Bacteria;p__Firmicutes;c__Bacilli;o__Brevibacillales;f__Brevibacillaceae;g__Brevibacillus;s__Brevibacillus_unclassified
d__Bacteria;p__Proteobacteria;c__Alphaproteobacteria;o__Alphaproteobacteria_unclassified;f__Alphaproteobacteria_unclassifie
d__Bacteria;p__Desulfobacterota;c__Desulfovibrionia;o__Desulfovibrionales;f__Desulfovibrionaceae;g__Desulfovibrionaceae_unc
d__Bacteria;p__Planctomycetota;c__Planctomycetes;o__Planctomycetales;f__Rubinisphaeraceae;g__SH-PL14;s__SH-PL14_unclassifie
d__Bacteria;p__Acidobacteriota;c__Vicinamibacteria;o__Subgroup_17;f__Subgroup_17_unclassified;g__Subgroup_17_unclassified;s
d__Bacteria;p__Gemmatimonadota;c__Gemmatimonadetes;o__Gemmatimonadales;f__Gemmatimonadaceae;g__Gemmatimonadaceae_unclassifi
d__Bacteria;p__Gemmatimonadota;c__Gemmatimonadetes;o__Gemmatimonadales;f__Gemmatimonadaceae;g__Gemmatimonas;s__Gemmatimonas
d__Bacteria;p__Proteobacteria;c__Alphaproteobacteria;o__Rhizobiales;f__Beijerinckiaceae;g__1174-901-12;s__1174-901-12_uncla
d__Bacteria;p__Proteobacteria;c__Gammaproteobacteria;o__Burkholderiales;f__Alcaligenaceae;g__Verticiella;s__Verticiella_unc
d__Bacteria;p__Actinobacteriota;c__Actinobacteria;o__Corynebacteriales;f__Corynebacteriaceae;g__Corynebacterium;s__Coryneba

d__Bacteria;p__Proteobacteria;c__Alphaproteobacteria;o__Sphingomonadales;f__Sphingomonadaceae;g__Sphingomonas;s__Sphingomon
d__Bacteria;p__Proteobacteria;c__Betaproteobacteria;o__Burkholderiales;f__Comamonadaceae;g__Comamonas;s__Comamonas_terrigen
d__Bacteria;p__Proteobacteria;c__Gammaproteobacteria;o__Burkholderiales;f__Sutterellaceae;g__Parasutterella;s__Parasutterel
d__Bacteria;p__Proteobacteria;c__Gammaproteobacteria;o__Burkholderiales;f__Comamonadaceae;g__Tepidimonas;s__Tepidimonas_unc
d__Bacteria;p__Proteobacteria;c__Gammaproteobacteria;o__Pseudomonadales;f__Moraxellaceae;g__Acinetobacter;s__Acinetobacter_
d__Bacteria;p__Firmicutes;c__Clostridia;o__Oscillospirales;f__Oscillospiraceae;g__NK4A214_group;s__NK4A214_group_unclassifi
d__Bacteria;p__Firmicutes;c__Clostridia;o__Clostridiales;f__Clostridiales_unclassified;g__Clostridiales_unclassified;s__Clo
d__Bacteria;p__Actinobacteriota;c__Thermoleophilia;o__Solirubrobacterales;f__Solirubrobacteraceae;g__Patulibacter;s__Patuli
d__Bacteria;p__Firmicutes;c__Clostridia;o__Clostridiales;f__Clostridiales_unclassified;g__Clostridiales_unclassified;s__Clo
d__Bacteria;p__Proteobacteria;c__Alphaproteobacteria;o__Rhizobiales;f__Rhizobiaceae;g__Rhizobium;s__uncultured_Rhizobium_sp
d__Bacteria;p__Bacteroidota;c__Bacteroidia;o__Bacteroidales;f__Muribaculaceae;g__Muribaculaceae_unclassified;s__Muribaculac
d__Bacteria;p__Firmicutes;c__Clostridia;o__Clostridiales;f__Clostridiales_unclassified;g__Clostridiales_unclassified;s__Clo
d__Bacteria;p__Firmicutes;c__Negativicutes;o__Veillonellales-Selenomonadales;f__Veillonellaceae;g__Dialister;s__Dialister_u
d__Bacteria;p__Proteobacteria;c__Alphaproteobacteria;o__Rhizobiales;f__Beijerinckiaceae;g__Methylobacterium-Methylorubrum;s
d__Bacteria;p__Firmicutes;c__Bacilli;o__Lactobacillales;f__Enterococcaceae;g__Enterococcus;s__Enterococcus_sp._T0_6
d__Bacteria;p__Bacteroidota;c__Bacteroidia;o__Flavobacteriales;f__Cryomorphaceae;g__Cryomorphaceae_unclassified;s__Cryomorp
d__Bacteria;p__Firmicutes;c__Thermincolia;o__Carboxydocellales;f__Carboxydocellaceae;g__Carboxydocella;s__Carboxydocella_un
d__Bacteria;p__Actinobacteriota;c__Actinobacteria;o__Pseudonocardiales;f__Pseudonocardiaceae;g__Saccharopolyspora;s__Saccha
d__Bacteria;p__Firmicutes;c__Clostridia;o__Oscillospirales;f__Oscillospiraceae;g__Intestinimonas;s__Intestinimonas_unclassi
d__Bacteria;p__Firmicutes;c__Clostridia;o__Monoglobales;f__Monoglobaceae;g__Monoglobus;s__Monoglobus_unclassified
d__Bacteria;p__Firmicutes;c__Bacilli;o__Alicyclobacillales;f__Alicyclobacillaceae;g__Alicyclobacillus;s__Alicyclobacillus_u
d__Bacteria;p__Firmicutes;c__Firmicutes_unclassified;o__Firmicutes_unclassified;f__Firmicutes_unclassified;g__Firmicutes_un
d__Bacteria;p__Cyanobacteria;c__Cyanobacteriia;o__Chloroplast;f__Chloroplast_unclassified;g__Chloroplast_unclassified;s__Ch
d__Bacteria;p__Firmicutes;c__Clostridia;o__Clostridiales;f__Ruminococcaceae;g__Ruminococcaceae_unclassified;s__Ruminococcac
d__Bacteria;p__Proteobacteria;c__Gammaproteobacteria;o__Pseudomonadales;f__SAR86_clade;g__SAR86_clade_unclassified;s__SAR86
d__Bacteria;p__Proteobacteria;c__Gammaproteobacteria;o__Pseudomonadales;f__OM182_clade;g__OM182_clade_unclassified;s__OM182
d__Bacteria;p__Firmicutes;c__Clostridia;o__Clostridiales;f__Ruminococcaceae;g__Anaerofilum;s__Anaerofilum_sp._Marseille-P33
d__Bacteria;p__Proteobacteria;c__Gammaproteobacteria;o__Burkholderiales;f__Comamonadaceae;g__Acidovorax;s__Acidovorax_uncla
d__Bacteria;p__Firmicutes;c__Clostridia;o__Clostridiales;f__Ruminococcaceae;g__Ruminococcaceae_unclassified;s__Ruminococcac
d__Bacteria;p__Actinobacteriota;c__Actinobacteria;o__Corynebacteriales;f__Corynebacteriaceae;g__Corynebacterium;s__Coryneba
d__Bacteria;p__Firmicutes;c__Clostridia;o__Oscillospirales;f__Butyricicoccaceae;g__Butyricicoccus;s__Butyricicoccus_unclass
d__Bacteria;p__Proteobacteria;c__Gammaproteobacteria;o__Gammaproteobacteria_unclassified;f__Gammaproteobacteria_unclassifie
d__Bacteria;p__Proteobacteria;c__Gammaproteobacteria;o__Xanthomonadales;f__Rhodanobacteraceae;g__Rhodanobacter;s__unculture
d__Bacteria;p__Firmicutes;c__Clostridia;o__Christensenellales;f__Christensenellaceae;g__Christensenellaceae_R-7_group;s__Ch
d__Bacteria;p__Firmicutes;c__Bacilli;o__Lactobacillales;f__Lactobacillaceae;g__Weissella;s__Weissella_unclassified
d__Bacteria;p__Gemmatimonadota;c__Gemmatimonadetes;o__Gemmatimonadales;f__Gemmatimonadaceae;g__Roseisolibacter;s__Roseisoli
d__Bacteria;p__Actinobacteriota;c__Actinobacteriota;o__Corynebacteriales;f__Corynebacteriaceae;g__Corynebacterium;s__uncult

d__Bacteria;p__Firmicutes;c__Clostridia;o__Lachnospirales;f__Lachnospiraceae;g__Eubacterium]_eligens_group;s__Eubacterium]_
d__Bacteria;p__Actinobacteriota;c__Actinobacteria;o__Micrococcales;f__Microbacteriaceae;g__Microbacterium;s__Microbacterium
d__Bacteria;p__Acidobacteriota;c__Acidobacteriota_unclassified;o__Acidobacteriota_unclassified;f__Acidobacteriota_unclassif
d__Bacteria;p__Actinobacteriota;c__Actinobacteria;o__Corynebacteriales;f__Mycobacteriaceae;g__Mycobacterium;s__Mycobacteriu
d__Bacteria;p__Proteobacteria;c__Gammaproteobacteria;o__Pseudomonadales;f__Pseudohongiellaceae;g__Pseudohongiella;s__Pseudo
d__Bacteria;p__Actinobacteriota;c__Actinobacteria;o__Frankiales;f__Geodermatophilaceae;g__Geodermatophilus;s__Geodermatophi
d__Bacteria;p__Proteobacteria;c__Betaproteobacteria;o__Burkholderiales;f__Comamonadaceae;g__Xenophilus;s__Xenophilus_aerola
d__Bacteria;p__Proteobacteria;c__Alphaproteobacteria;o__Sphingomonadales;f__Sphingomonadaceae;g__Sphingopyxis;s__Sphingopyx
d__Bacteria;p__Proteobacteria;c__Betaproteobacteria;o__Burkholderiales;f__Comamonadaceae;g__Comamonas;s__uncultured_Comamon
d__Bacteria;p__Proteobacteria;c__Gammaproteobacteria;o__Pseudomonadales;f__Pseudohongiellaceae;g__Pseudohongiella;s__Pseudo
d__Bacteria;p__Firmicutes;c__Clostridia;o__Clostridiales;f__Clostridiales_unclassified;g__Clostridiales_unclassified;s__Clo
d__Bacteria;p__Firmicutes;c__Clostridia;o__Lachnospirales;f__Lachnospiraceae;g__Lachnospiraceae_NK4A136_group;s__Lachnospir
d__Bacteria;p__Firmicutes;c__Clostridia;o__Oscillospirales;f__Oscillospiraceae;g__Oscillibacter;s__Oscillibacter_unclassifi
d__Bacteria;p__Proteobacteria;c__Alphaproteobacteria;o__Rhizobiales;f__Beijerinckiaceae;g__Methylobacterium-Methylorubrum;s
d__Bacteria;p__Actinobacteriota;c__Actinobacteria;o__Micrococcales;f__Micrococcaceae;g__Rothia;s__Rothia_unclassified
d__Bacteria;p__Proteobacteria;c__Gammaproteobacteria;o__Burkholderiales;f__Sutterellaceae;g__Parasutterella;s__Parasutterel
d__Bacteria;p__Proteobacteria;c__Alphaproteobacteria;o__Rhizobiales;f__Beijerinckiaceae;g__Bosea;s__Bosea_unclassified
d__Bacteria;p__Deinococcota;c__Deinococci;o__Thermales;f__Thermaceae;g__Thermus;s__Thermus_unclassified
d__Bacteria;p__Proteobacteria;c__Gammaproteobacteria;o__Burkholderiales;f__Burkholderiaceae;g__Cupriavidus;s__Cupriavidus_u
d__Bacteria;p__Firmicutes;c__Clostridia;o__Lachnospirales;f__Lachnospiraceae;g__Roseburia;s__Roseburia_unclassified
d__Bacteria;p__Bacteroidota;c__Bacteroidia;o__Bacteroidales;f__Tannerellaceae;g__Parabacteroides;s__Parabacteroides_sp.
d__Bacteria;p__Firmicutes;c__Clostridia;o__Peptococcales;f__Peptococcaceae;g__Peptococcaceae_unclassified;s__Peptococcaceae
d__Bacteria;p__Bacteroidota;c__Bacteroidia;o__Bacteroidales;f__Muribaculaceae;g__Muribaculaceae_unclassified;s__Muribaculac
d__Bacteria;p__Firmicutes;c__Clostridia;o__Oscillospirales;f__Oscillospiraceae;g__Oscillospiraceae_unclassified;s__Oscillos
d__Bacteria;p__Proteobacteria;c__Betaproteobacteria;o__Burkholderiales;f__Comamonadaceae;g__Comamonas;s__Comamonas_sp.
d__Bacteria;p__Actinobacteriota;c__Actinobacteria;o__Micrococcales;f__Microbacteriaceae;g__Microbacterium;s__Microbacterium
d__Bacteria;p__Bacteroidota;c__Bacteroidia;o__Bacteroidales;f__Prevotellaceae;g__Prevotella_9;s__Prevotella_9_unclassified
d__Bacteria;p__Proteobacteria;c__Gammaproteobacteria;o__Pseudomonadales;f__Pseudomonadaceae;g__Pseudomonas;s__Pseudomonas_f
d__Bacteria;p__Proteobacteria;c__Gammaproteobacteria;o__Enterobacterales;f__Aeromonadaceae;g__Aeromonas;s__Aeromonas_unclas
d__Bacteria;p__Proteobacteria;c__Gammaproteobacteria;o__Xanthomonadales;f__Xanthomonadaceae;g__Xanthomonas;s__Xanthomonas_s
d__Bacteria;p__Firmicutes;c__Clostridia;o__Lachnospirales;f__Lachnospiraceae;g__Ruminococcus]_torques_group;s__Ruminococcus
d__Bacteria;p__Proteobacteria;c__Alphaproteobacteria;o__Sphingomonadales;f__Sphingomonadaceae;g__Sphingomonas;s__Sphingomon
d__Bacteria;p__Firmicutes;c__Clostridia;o__Oscillospirales;f__Ruminococcaceae;g__CAG-352;s__CAG-352_unclassified
d__Bacteria;p__Firmicutes;c__Bacilli;o__Lactobacillales;f__Lactobacillaceae;g__Limosilactobacillus;s__Limosilactobacillus_u
d__Bacteria;p__Firmicutes;c__Clostridia;o__Clostridiales;f__Lachnospiraceae;g__Eisenbergiella;s__Eisenbergiella_sp.
d__Bacteria;p__Proteobacteria;c__Gammaproteobacteria;o__Pseudomonadales;f__Pseudomonadaceae;g__Pseudomonas;s__Pseudomonas_n
d__Bacteria;p__Firmicutes;c__Clostridia;o__Oscillospirales;f__Ruminococcaceae;g__Anaerotruncus;s__Anaerotruncus_unclassifie

d__Bacteria;p__Firmicutes;c__Bacilli;o__Bacillales;f__Bacillaceae;g__Geobacillus;s__Geobacillus_unclassified
d__Bacteria;p__Firmicutes;c__Clostridia;o__Oscillospirales;f__Butyricicoccaceae;g__Butyricicoccus;s__Butyricicoccus_unclass
d__Bacteria;p__Actinobacteriota;c__Acidimicrobiia;o__Actinomarinales;f__Actinomarinaceae;g__Candidatus_Actinomarina;s__Cand
d__Bacteria;p__Bacteroidota;c__Bacteroidota_unclassified;o__Bacteroidota_unclassified;f__Bacteroidota_unclassified;g__Bacte
d__Bacteria;p__Proteobacteria;c__Alphaproteobacteria;o__Alphaproteobacteria_unclassified;f__Alphaproteobacteria_unclassifie
d__Bacteria;p__Cyanobacteria;c__Cyanobacteriia;o__Chloroplast;f__Chloroplast_unclassified;g__Chloroplast_unclassified;s__Ch
d__Bacteria;p__Bacteroidota;c__Bacteroidia;o__Flavobacteriales;f__Flavobacteriaceae;g__NS4_marine_group;s__NS4_marine_group
d__Bacteria;p__Proteobacteria;c__Gammaproteobacteria;o__Xanthomonadales;f__Rhodanobacteraceae;g__Dyella;s__Dyella_unclassif
d__Bacteria;p__Firmicutes;c__Symbiobacteriia;o__Symbiobacteriales;f__Symbiobacteraceae;g__Symbiobacterium;s__Symbiobacteriu
d__Bacteria;p__Firmicutes;c__Clostridia;o__Clostridiales;f__Lachnospiraceae;g__Blautia;s__uncultured_Blautia_sp.
d__Bacteria;p__Bacteroidota;c__Bacteroidia;o__Chitinophagales;f__Chitinophagaceae;g__Terrimonas;s__Terrimonas_unclassified
d__Bacteria;p__Myxococcota;c__Polyangia;o__Haliangiales;f__Haliangiaceae;g__Haliangium;s__Haliangium_unclassified
d__Bacteria;p__Firmicutes;c__Bacilli;o__Bacillales;f__Bacillaceae;g__Geobacillus;s__Geobacillus_stearothermophilus
d__Bacteria;p__Proteobacteria;c__Gammaproteobacteria;o__Burkholderiales;f__Comamonadaceae;g__Zhizhongheella;s__Zhizhongheel
d__Bacteria;p__Actinobacteriota;c__Actinobacteria;o__Micrococcales;f__Microbacteriaceae;g__ML602J-51;s__ML602J-51_unclassif
d__Bacteria;p__Verrucomicrobiota;c__Verrucomicrobiae;o__Pedosphaerales;f__Pedosphaeraceae;g__ADurb.Bin063-1;s__ADurb.Bin063
d__Bacteria;p__Methylomirabilota;c__Methylomirabilia;o__Rokubacteriales;f__Rokubacteriales_unclassified;g__Rokubacteriales_
d__Bacteria;p__Firmicutes;c__Bacilli;o__Lactobacillales;f__Enterococcaceae;g__Enterococcus;s__Enterococcus_thailandicus
d__Bacteria;p__Proteobacteria;c__Gammaproteobacteria;o__Xanthomonadales;f__Xanthomonadaceae;g__Lysobacter;s__Lysobacter_sp.
d__Bacteria;p__Firmicutes;c__Bacilli;o__Staphylococcales;f__Staphylococcaceae;g__Staphylococcus;s__Staphylococcus_unclassif
d__Bacteria;p__Firmicutes;c__Clostridia;o__Clostridiales;f__Lachnospiraceae;g__Lachnospiraceae_unclassified;s__Lachnospirac
d__Bacteria;p__Firmicutes;c__Clostridia;o__Lachnospirales;f__Lachnospiraceae;g__Blautia;s__Blautia_unclassified
d__Bacteria;p__Proteobacteria;c__Betaproteobacteria;o__Burkholderiales;f__Comamonadaceae;g__Comamonas;s__Comamonas_sp._JMC-
d__Bacteria;p__Firmicutes;c__Bacilli;o__Lactobacillales;f__Streptococcaceae;g__Streptococcus;s__uncultured_Streptococcus_sp
d__Bacteria;p__Planctomycetota;c__Phycisphaerae;o__Tepidisphaerales;f__Tepidisphaerales_unclassified;g__Tepidisphaerales_un
d__Bacteria;p__Proteobacteria;c__Alphaproteobacteria;o__Rhizobiales;f__Hyphomicrobiaceae;g__Hyphomicrobium;s__uncultured_Hy
d__Bacteria;p__Sumerlaeota;c__Sumerlaeia;o__Sumerlaeales;f__Sumerlaeaceae;g__Sumerlaea;s__Sumerlaea_unclassified
d__Bacteria;p__Proteobacteria;c__Gammaproteobacteria;o__Enterobacterales;f__Morganellaceae;g__Providencia;s__Providencia_sp
d__Bacteria;p__Firmicutes;c__Clostridia;o__Clostridia_UCG-014;f__Clostridia_UCG-014_unclassified;g__Clostridia_UCG-014_uncl
d__Bacteria;p__Firmicutes;c__Clostridia;o__Oscillospirales;f__Ruminococcaceae;g__Faecalibacterium;s__Faecalibacterium_uncla
d__Bacteria;p__Proteobacteria;c__Alphaproteobacteria;o__Rhizobiales;f__Xanthobacteraceae;g__Xanthobacter;s__uncultured_Xant
d__Bacteria;p__Firmicutes;c__Clostridia;o__Thermincolales;f__Thermincolaceae;g__Thermincola;s__Thermincola_unclassified
d__Bacteria;p__Proteobacteria;c__Alphaproteobacteria;o__Sphingomonadales;f__Sphingomonadaceae;g__Sphingopyxis;s__Sphingopyx
d__Bacteria;p__Proteobacteria;c__Gammaproteobacteria;o__Xanthomonadales;f__Xanthomonadaceae;g__Stenotrophomonas;s__Stenotro
d__Bacteria;p__Firmicutes;c__Bacilli;o__Staphylococcales;f__Staphylococcaceae;g__Staphylococcus;s__Staphylococcus_unclassif
d__Bacteria;p__Firmicutes;c__Clostridia;o__Lachnospirales;f__Lachnospiraceae;g__Lachnospiraceae_NK4A136_group;s__Lachnospir
d__Bacteria;p__Firmicutes;c__Bacilli;o__Staphylococcales;f__Staphylococcaceae;g__Staphylococcus;s__Staphylococcus_unclassif

d__Bacteria;p__Firmicutes;c__Clostridia;o__Lachnospirales;f__Lachnospiraceae;g__Roseburia;s__Roseburia_unclassified
d__Bacteria;p__Proteobacteria;c__Alphaproteobacteria;o__Sphingomonadales;f__Sphingomonadaceae;g__Sphingopyxis;s__Sphingopyx
d__Bacteria;p__Firmicutes;c__Clostridia;o__Peptostreptococcales-Tissierellales;f__Family_XI;g__Anaerococcus;s__Anaerococcus
d__Bacteria;p__Proteobacteria;c__Gammaproteobacteria;o__Enterobacterales;f__Alteromonadaceae;g__Rheinheimera;s__Rheinheimer
d__Bacteria;p__Proteobacteria;c__Gammaproteobacteria;o__Burkholderiales;f__Rhodocyclaceae;g__Dechlorosoma;s__Dechlorosoma_u
d__Bacteria;p__Firmicutes;c__Clostridia;o__Thermincolales;f__Thermincolaceae;g__Thermincola;s__Thermincola_unclassified
d__Bacteria;p__Bacteroidota;c__Bacteroidia;o__Bacteroidales;f__Bacteroidaceae;g__Bacteroides;s__Bacteroides_unclassified
d__Bacteria;p__Firmicutes;c__Clostridia;o__Oscillospirales;f__Ruminococcaceae;g__Ruminococcus;s__Ruminococcus_unclassified
d__Bacteria;p__Desulfobacterota;c__Desulfovibrionia;o__Desulfovibrionales;f__Desulfovibrionaceae;g__Bilophila;s__Bilophila_
d__Bacteria;p__Actinobacteriota;c__Actinobacteria;o__Micrococcales;f__Micrococcaceae;g__Rothia;s__Rothia_unclassified
d__Bacteria;p__Firmicutes;c__Clostridia;o__Oscillospirales;f__Ruminococcaceae;g__Subdoligranulum;s__Subdoligranulum_unclass
d__Bacteria;p__Proteobacteria;c__Alphaproteobacteria;o__Azospirillales;f__Azospirillaceae;g__Azospirillum;s__Azospirillum_u
d__Bacteria;p__Proteobacteria;c__Gammaproteobacteria;o__Pseudomonadales;f__Pseudomonadaceae;g__Pseudomonas;s__Pseudomonas_h
d__Bacteria;p__Proteobacteria;c__Gammaproteobacteria;o__Pseudomonadales;f__Pseudomonadaceae;g__Pseudomonas;s__Pseudomonas_s
d__Bacteria;p__Firmicutes;c__Clostridia;o__Thermincolales;f__Thermincolaceae;g__Thermincola;s__Thermincola_unclassified
d__Bacteria;p__Firmicutes;c__Clostridia;o__Oscillospirales;f__Ruminococcaceae;g__Harryflintia;s__Harryflintia_unclassified
d__Bacteria;p__Bacteroidota;c__Bacteroidia;o__Bacteroidales;f__Muribaculaceae;g__Muribaculaceae_unclassified;s__Muribaculac
d__Bacteria;p__Firmicutes;c__Bacilli;o__Erysipelotrichales;f__Erysipelotrichaceae;g__Faecalibaculum;s__Faecalibaculum_uncla
d__Bacteria;p__Firmicutes;c__Negativicutes;o__Veillonellales-Selenomonadales;f__Selenomonadaceae;g__Megamonas;s__Megamonas_
d__Bacteria;p__Bacteroidota;c__Bacteroidia;o__Bacteroidales;f__Rikenellaceae;g__Rikenellaceae_RC9_gut_group;s__Rikenellacea
d__Bacteria;p__Firmicutes;c__Clostridia;o__Oscillospirales;f__Oscillospiraceae;g__Colidextribacter;s__Colidextribacter_uncl
d__Bacteria;p__Armatimonadota;c__Fimbriimonadia;o__Fimbriimonadales;f__Fimbriimonadaceae;g__Fimbriimonadaceae_unclassified;
d__Bacteria;p__Proteobacteria;c__Alphaproteobacteria;o__Rhizobiales;f__Hyphomicrobiaceae;g__Hyphomicrobium;s__uncultured_Hy
d__Bacteria;p__Desulfobacterota;c__Desulfovibrionia;o__Desulfovibrionales;f__Desulfovibrionaceae;g__Desulfovibrionaceae_unc
d__Bacteria;p__Proteobacteria;c__Alphaproteobacteria;o__Rhizobiales;f__Beijerinckiaceae;g__Bosea;s__Bosea_unclassified
d__Bacteria;p__Proteobacteria;c__Gammaproteobacteria;o__Burkholderiales;f__Comamonadaceae;g__RS62_marine_group;s__RS62_mari
d__Bacteria;p__Firmicutes;c__Clostridia;o__Oscillospirales;f__Eubacterium]_coprostanoligenes_group;g__Eubacterium]_coprosta
d__Bacteria;p__Proteobacteria;c__Alphaproteobacteria;o__Rhodobacterales;f__Rhodobacteraceae;g__Rubellimicrobium;s__Rubellim
d__Bacteria;p__Verrucomicrobiota;c__Verrucomicrobiae;o__Chthoniobacterales;f__Chthoniobacteraceae;g__Chthoniobacter;s__Chth
d__Bacteria;p__Actinobacteriota;c__Actinobacteria;o__Micrococcales;f__Brevibacteriaceae;g__Brevibacterium;s__Brevibacterium
d__Bacteria;p__Proteobacteria;c__Alphaproteobacteria;o__Holosporales;f__Holosporaceae;g__Holosporaceae_unclassified;s__Holo
d__Bacteria;p__Cyanobacteria;c__Cyanobacteriia;o__Chloroplast;f__Chloroplast_unclassified;g__Chloroplast_unclassified;s__Ch
d__Bacteria;p__Firmicutes;c__Clostridia;o__Oscillospirales;f__Eubacterium]_coprostanoligenes_group;g__Eubacterium]_coprosta
d__Bacteria;p__Firmicutes;c__Clostridia;o__Clostridia_UCG-014;f__Clostridia_UCG-014_unclassified;g__Clostridia_UCG-014_uncl
d__Bacteria;p__Firmicutes;c__Bacilli;o__Bacillales;f__Bacillaceae;g__Geobacillus;s__uncultured_Geobacillus_sp.
d__Bacteria;p__Proteobacteria;c__Gammaproteobacteria;o__Burkholderiales;f__Oxalobacteraceae;g__Massilia;s__Massilia_unclass
d__Bacteria;p__Bacteroidota;c__Bacteroidia;o__Flavobacteriales;f__Flavobacteriaceae;g__Formosa;s__Formosa_unclassified

d__Bacteria;p__Firmicutes;c__Clostridia;o__Oscillospirales;f__Oscillospiraceae;g__Intestinimonas;s__Intestinimonas_unclassi
d__Bacteria;p__Firmicutes;c__Clostridia;o__Clostridiales;f__Lachnospiraceae;g__Blautia;s__uncultured_Blautia_sp.
d__Bacteria;p__Firmicutes;c__Bacilli;o__Erysipelotrichales;f__Erysipelotrichaceae;g__Erysipelotrichaceae_unclassified;s__Er
d__Bacteria;p__Firmicutes;c__Clostridia;o__Clostridia_UCG-014;f__Clostridia_UCG-014_unclassified;g__Clostridia_UCG-014_uncl
d__Bacteria;p__Deinococcota;c__Deinococci;o__Deinococcales;f__Deinococcaceae;g__Deinococcus;s__Deinococcus_unclassified
d__Bacteria;p__Proteobacteria;c__Alphaproteobacteria;o__Acetobacterales;f__Acetobacteraceae;g__Craurococcus-Caldovatus;s__C
d__Bacteria;p__Firmicutes;c__Thermoanaerobacteria;o__Thermoanaerobacterales;f__Thermoanaerobacteraceae;g__Thermanaeromonas;
d__Bacteria;p__Firmicutes;c__Clostridia;o__Oscillospirales;f__Ruminococcaceae;g__Incertae_Sedis;s__Incertae_Sedis_unclassif
d__Bacteria;p__Proteobacteria;c__Gammaproteobacteria;o__Legionellales;f__Legionellaceae;g__Legionella;s__uncultured_Legione
d__Bacteria;p__Nitrospirota;c__Nitrospiria;o__Nitrospirales;f__Nitrospiraceae;g__Nitrospira;s__Nitrospira_unclassified
d__Bacteria;p__Cyanobacteria;c__Cyanobacteriia;o__Chloroplast;f__Chloroplast_unclassified;g__Chloroplast_unclassified;s__Ch
d__Bacteria;p__Proteobacteria;c__Gammaproteobacteria;o__Pseudomonadales;f__Pseudohongiellaceae;g__Pseudohongiella;s__Pseudo
d__Bacteria;p__Firmicutes;c__Bacilli;o__Bacillales;f__Bacillaceae;g__Bacillaceae_unclassified;s__Bacillaceae_unclassified
d__Bacteria;p__Proteobacteria;c__Alphaproteobacteria;o__Rhizobiales;f__Devosiaceae;g__Devosiaceae_unclassified;s__Devosiace
d__unclassified;p__unclassified;c__unclassified;o__unclassified;f__unclassified;g__unclassified;s__unclassified
d__Bacteria;p__Firmicutes;c__Clostridia;o__Lachnospirales;f__Lachnospiraceae;g__Blautia;s__Blautia_unclassified
d__Bacteria;p__Proteobacteria;c__Gammaproteobacteria;o__Enterobacterales;f__Enterobacteriaceae;g__Citrobacter;s__Citrobacte
d__Bacteria;p__Proteobacteria;c__Alphaproteobacteria;o__Alphaproteobacteria_unclassified;f__Alphaproteobacteria_unclassifie
d__Bacteria;p__Proteobacteria;c__Alphaproteobacteria;o__Puniceispirillales;f__SAR116_clade;g__Candidatus_Puniceispirillum;s
d__Bacteria;p__Nitrospirota;c__Nitrospiria;o__Nitrospirales;f__Nitrospiraceae;g__Nitrospira;s__Nitrospira_unclassified
d__Bacteria;p__Acidobacteriota;c__Acidobacteriae;o__Acidobacteriales;f__Acidobacteriales_unclassified;g__Acidobacteriales_u
d__Bacteria;p__Firmicutes;c__Clostridia;o__Clostridiales;f__Lachnospiraceae;g__Lachnospiraceae_unclassified;s__Lachnospirac
d__Bacteria;p__Firmicutes;c__Clostridia;o__Clostridia_UCG-014;f__Clostridia_UCG-014_unclassified;g__Clostridia_UCG-014_uncl
d__Bacteria;p__Actinobacteriota;c__Actinobacteria;o__Micrococcales;f__Micrococcaceae;g__Kocuria;s__Kocuria_unclassified
d__Bacteria;p__Fusobacteriota;c__Fusobacteriia;o__Fusobacteriotales;f__Fusobacteriotaceae;g__Fusobacteriotaceae_unclassifie
d__Bacteria;p__Proteobacteria;c__Gammaproteobacteria;o__Pseudomonadales;f__Moraxellaceae;g__Acinetobacter;s__Acinetobacter_
d__Bacteria;p__Firmicutes;c__Clostridia;o__Clostridiales;f__Ruminococcaceae;g__Ruminococcus;s__uncultured_Ruminococcus_sp.
d__Bacteria;p__Firmicutes;c__Desulfotomaculia;o__Desulfotomaculales;f__Desulfotomaculaceae;g__Desulfotomaculum;s__Desulfoto
d__Bacteria;p__Actinobacteriota;c__Actinobacteria;o__Corynebacteriales;f__Mycobacteriaceae;g__Mycobacterium;s__Mycobacteriu
d__Bacteria;p__Firmicutes;c__Clostridia;o__Caldicoprobacterales;f__Caldicoprobacteraceae;g__Caldicoprobacter;s__Caldicoprob
d__Bacteria;p__Bacteroidota;c__Bacteroidia;o__Bacteroidales;f__Bacteroidaceae;g__Bacteroides;s__Bacteroides_finegoldii
d__Bacteria;p__Proteobacteria;c__Gammaproteobacteria;o__Burkholderiales;f__Rhodocyclaceae;g__Dechlorosoma;s__Dechlorosoma_u
d__Bacteria;p__Firmicutes;c__Bacilli;o__Bacillales;f__Bacillaceae;g__Bacillus;s__Bacillus_halodurans
d__Bacteria;p__Proteobacteria;c__Gammaproteobacteria;o__Enterobacterales;f__Enterobacteriaceae;g__Escherichia-Shigella;s__E
d__Bacteria;p__Acidobacteriota;c__Acidobacteriae;o__Bryobacterales;f__Bryobacteraceae;g__Bryobacter;s__Bryobacter_unclassif
d__unclassified;p__unclassified;c__unclassified;o__unclassified;f__unclassified;g__unclassified;s__unclassified
d__Bacteria;p__Proteobacteria;c__Gammaproteobacteria;o__Burkholderiales;f__Comamonadaceae;g__Hydrogenophaga;s__Hydrogenopha

d__Bacteria;p__Proteobacteria;c__Alphaproteobacteria;o__Rhodobacterales;f__Rhodobacteraceae;g__Paracoccus;s__Paracoccus_sp.
d__Bacteria;p__Proteobacteria;c__Alphaproteobacteria;o__Rhizobiales;f__Rhizobiaceae;g__Shinella;s__Shinella_kummerowiae
d__Bacteria;p__Bacteroidota;c__Bacteroidia;o__Bacteroidales;f__Muribaculaceae;g__Muribaculaceae_unclassified;s__Muribaculac
d__Bacteria;p__Bacteroidota;c__Bacteroidia;o__Bacteroidales;f__Prevotellaceae;g__Prevotellaceae_Ga6A1_group;s__Prevotellace
d__Bacteria;p__Firmicutes;c__Clostridia;o__Peptostreptococcales-Tissierellales;f__Family_XI;g__Anaerococcus;s__Anaerococcus
d__Bacteria;p__Proteobacteria;c__Alphaproteobacteria;o__Rhizobiales;f__Rhizobiales_Incertae_Sedis;g__Phreatobacter;s__Phrea
d__Bacteria;p__Proteobacteria;c__Gammaproteobacteria;o__Pseudomonadales;f__Moraxellaceae;g__Acinetobacter;s__Acinetobacter_
d__Bacteria;p__Firmicutes;c__Clostridia;o__Oscillospirales;f__Oscillospiraceae;g__UCG-002;s__UCG-002_unclassified
d__Bacteria;p__Proteobacteria;c__Alphaproteobacteria;o__Rhizobiales;f__Rhizobiaceae;g__Rhizobium;s__uncultured_Rhizobium_sp
d__Bacteria;p__Firmicutes;c__Negativicutes;o__Veillonellales-Selenomonadales;f__Veillonellaceae;g__Veillonella;s__Veillonel
d__Bacteria;p__Firmicutes;c__Clostridia;o__Lachnospirales;f__Lachnospiraceae;g__Eubacterium]_ruminantium_group;s__Eubacteri
d__Bacteria;p__Actinobacteriota;c__Actinobacteria;o__Micrococcales;f__Micrococcaceae;g__Rothia;s__Rothia_unclassified
d__Bacteria;p__Firmicutes;c__Clostridia;o__Clostridiales;f__Ruminococcaceae;g__Pseudoflavonifractor;s__Pseudoflavonifractor
d__Bacteria;p__Proteobacteria;c__Gammaproteobacteria;o__Pseudomonadales;f__Moraxellaceae;g__Acinetobacter;s__Acinetobacter_
d__Bacteria;p__Firmicutes;c__Bacilli;o__Bacillales;f__Paenibacillaceae;g__Paenibacillus;s__Paenibacillus_sp._DXL2
d__Bacteria;p__Firmicutes;c__Clostridia;o__Oscillospirales;f__Ruminococcaceae;g__Subdoligranulum;s__Subdoligranulum_unclass
d__Bacteria;p__Proteobacteria;c__Gammaproteobacteria;o__Pseudomonadales;f__Saccharospirillaceae;g__Oceanobacter;s__Oceanoba
d__Bacteria;p__Proteobacteria;c__Gammaproteobacteria;o__Xanthomonadales;f__Xanthomonadaceae;g__Stenotrophomonas;s__Stenotro
d__Bacteria;p__Proteobacteria;c__Alphaproteobacteria;o__Sphingomonadales;f__Sphingomonadaceae;g__Altererythrobacter;s__Alte
d__Bacteria;p__Actinobacteriota;c__Actinobacteria;o__Corynebacteriales;f__Corynebacteriaceae;g__Corynebacterium;s__Coryneba
d__Bacteria;p__Deinococcota;c__Deinococci;o__Deinococcales;f__Deinococcaceae;g__Deinococcus;s__Deinococcus_unclassified
d__Bacteria;p__Firmicutes;c__Clostridia;o__Clostridiales;f__Lachnospiraceae;g__Roseburia;s__Roseburia_sp._499
d__Bacteria;p__Firmicutes;c__Clostridia;o__Clostridiales;f__Clostridiaceae;g__Clostridium;s__uncultured_Clostridium_sp.
d__Bacteria;p__Firmicutes;c__Clostridia;o__Clostridiales;f__Lachnospiraceae;g__Robinsoniella;s__Robinsoniella_sp.
d__Bacteria;p__Bacteroidota;c__Bacteroidia;o__Bacteroidales;f__Marinifilaceae;g__Odoribacter;s__Odoribacter_unclassified
d__Bacteria;p__Proteobacteria;c__Alphaproteobacteria;o__Rhizobiales;f__Rhizobiaceae;g__Aminobacter;s__Aminobacter_unclassif
d__Bacteria;p__Firmicutes;c__Negativicutes;o__Acidaminococcales;f__Acidaminococcaceae;g__Phascolarctobacterium;s__Phascolar
d__Bacteria;p__Bacteroidota;c__Bacteroidia;o__Flavobacteriales;f__Weeksellaceae;g__Chryseobacterium;s__Chryseobacterium_unc
d__Bacteria;p__Proteobacteria;c__Alphaproteobacteria;o__Sphingomonadales;f__Sphingomonadaceae;g__Sphingomonas;s__uncultured
d__Bacteria;p__Proteobacteria;c__Gammaproteobacteria;o__Burkholderiales;f__Oxalobacteraceae;g__Massilia;s__Massilia_unclass
d__Bacteria;p__Firmicutes;c__Clostridia;o__Lachnospirales;f__Lachnospiraceae;g__Blautia;s__Blautia_unclassified
d__Bacteria;p__Firmicutes;c__Negativicutes;o__Veillonellales-Selenomonadales;f__Veillonellaceae;g__Megasphaera;s__Megasphae
d__Bacteria;p__Firmicutes;c__Negativicutes;o__Veillonellales-Selenomonadales;f__Selenomonadaceae;g__Megamonas;s__Megamonas_
d__Bacteria;p__Firmicutes;c__Clostridia;o__Oscillospirales;f__Oscillospiraceae;g__UCG-005;s__UCG-005_unclassified
d__Bacteria;p__Proteobacteria;c__Gammaproteobacteria;o__Burkholderiales;f__Methylophilaceae;g__OM43_clade;s__OM43_clade_unc
d__Bacteria;p__Firmicutes;c__Bacilli;o__Lactobacillales;f__Streptococcaceae;g__Streptococcus;s__Streptococcus_constellatus
d__Bacteria;p__Firmicutes;c__Firmicutes_unclassified;o__Firmicutes_unclassified;f__Firmicutes_unclassified;g__Firmicutes_un

```
d__Bacteria;p__Proteobacteria;c__Alphaproteobacteria;o__Parvibaculales;f__PS1_clade;g__PS1_clade_unclassified;s__PS1_clade_
d__Bacteria;p__Proteobacteria;c__Gammaproteobacteria;o__Burkholderiales;f__Sutterellaceae;g__Sutterella;s__Sutterella_uncla
d__Bacteria;p__Firmicutes;c__Clostridia;o__Oscillospirales;f__Butyricicoccaceae;g__Butyricicoccus;s__Butyricicoccus_unclass
d__Bacteria;p__Cyanobacteria;c__Cyanobacteriia;o__Cyanobacteriales;f__Chroococcidiopsaceae;g__Aliterella;s__Aliterella_uncl
d__Bacteria;p__Firmicutes;c__Clostridia;o__Clostridiales;f__Caloramatoraceae;g__Caloramator;s__Caloramator_unclassified
d__Bacteria;p__Firmicutes;c__Bacilli;o__Erysipelotrichales;f__Erysipelotrichaceae;g__ZOR0006;s__ZOR0006_unclassified
d__Bacteria;p__Bacteroidota;c__Bacteroidia;o__Bacteroidales;f__Bacteroidaceae;g__Bacteroides;s__uncultured_Bacteroides_sp.
d__Bacteria;p__Proteobacteria;c__Gammaproteobacteria;o__Burkholderiales;f__Sutterellaceae;g__Sutterella;s__Sutterella_uncla
d__Bacteria;p__Firmicutes;c__Bacilli;o__Bacillales;f__Sporolactobacillaceae;g__Scopulibacillus;s__Scopulibacillus_unclassif
d__Bacteria;p__Firmicutes;c__Clostridia;o__Clostridiales;f__Lachnospiraceae;g__Lachnospiraceae_unclassified;s__Lachnospirac
d__Bacteria;p__Proteobacteria;c__Gammaproteobacteria;o__UBA10353_marine_group;f__UBA10353_marine_group_unclassified;g__UBA1
d__Bacteria;p__Proteobacteria;c__Gammaproteobacteria;o__Pseudomonadales;f__Oleiphilaceae;g__Oleiphilus;s__Oleiphilus_unclas
d__Bacteria;p__Proteobacteria;c__Gammaproteobacteria;o__Pseudomonadales;f__Pseudomonadaceae;g__Pseudomonas;s__Pseudomonas_p
d__Bacteria;p__Bacteroidota;c__Bacteroidia;o__Flavobacteriales;f__Flavobacteriaceae;g__NS4_marine_group;s__NS4_marine_group
d__Bacteria;p__Acidobacteriota;c__Vicinamibacteria;o__Subgroup_17;f__Subgroup_17_unclassified;g__Subgroup_17_unclassified;s
d__Bacteria;p__Proteobacteria;c__Alphaproteobacteria;o__Rhodobacterales;f__Rhodobacteraceae;g__Rhodobacteraceae_unclassifie
d__Bacteria;p__Proteobacteria;c__Deltaproteobacteria;o__Desulfuromonadales;f__Geobacteraceae;g__Geobacter;s__uncultured_Geo
d__Bacteria;p__Proteobacteria;c__Alphaproteobacteria;o__Sphingomonadales;f__Sphingomonadaceae;g__Sphingorhabdus;s__Sphingor
d__Bacteria;p__Proteobacteria;c__Gammaproteobacteria;o__Burkholderiales;f__Rhodocyclaceae;g__Denitratisoma;s__Denitratisoma
d__Bacteria;p__Firmicutes;c__Clostridia;o__Oscillospirales;f__Oscillospiraceae;g__Flavonifractor;s__Flavonifractor_unclassi
d__Bacteria;p__Firmicutes;c__Clostridia;o__Lachnospirales;f__Lachnospiraceae;g__Eubacterium]_hallii_group;s__Eubacterium]_h
d__Bacteria;p__Firmicutes;c__Clostridia;o__Oscillospirales;f__Ruminococcaceae;g__Subdoligranulum;s__Subdoligranulum_unclass
d__Bacteria;p__Cyanobacteria;c__Cyanobacteriia;o__Cyanobacteriales;f__Chroococcidiopsaceae;g__Aliterella;s__Aliterella_uncl
d__Bacteria;p__Bacteroidota;c__Bacteroidia;o__Bacteroidales;f__Bacteroidales_unclassified;g__Bacteroidales_unclassified;s__
d__Bacteria;p__Proteobacteria;c__Gammaproteobacteria;o__Burkholderiales;f__Rhodocyclaceae;g__Thauera;s__Thauera_unclassifie
d__Bacteria;p__Proteobacteria;c__Gammaproteobacteria;o__Pseudomonadales;f__Pseudomonadaceae;g__Pseudomonas;s__Pseudomonas_g
d__Bacteria;p__Firmicutes;c__Clostridia;o__Lachnospirales;f__Lachnospiraceae;g__Roseburia;s__Roseburia_unclassified
d__Bacteria;p__Bacteroidota;c__Bacteroidia;o__Bacteroidales;f__Tannerellaceae;g__Parabacteroides;s__Parabacteroides_distaso
d__Bacteria;p__Firmicutes;c__Clostridia;o__Clostridia_unclassified;f__Clostridia_unclassified;g__Clostridia_unclassified;s_
d__Bacteria;p__Firmicutes;c__Clostridia;o__Oscillospirales;f__Oscillospiraceae;g__UCG-002;s__UCG-002_unclassified
d__Bacteria;p__Proteobacteria;c__Alphaproteobacteria;o__Caulobacterales;f__Caulobacteraceae;g__Brevundimonas;s__Brevundimon
d__Bacteria;p__Bacteroidota;c__Bacteroidia;o__Chitinophagales;f__Saprospiraceae;g__Saprospiraceae_unclassified;s__Saprospir
d__Bacteria;p__Proteobacteria;c__Gammaproteobacteria;o__Pseudomonadales;f__Moraxellaceae;g__Psychrobacter;s__Psychrobacter_
d__Bacteria;p__Proteobacteria;c__Gammaproteobacteria;o__Burkholderiales;f__Sutterellaceae;g__Sutterella;s__Sutterella_uncla
d__Bacteria;p__Proteobacteria;c__Alphaproteobacteria;o__Acetobacterales;f__Acetobacteraceae;g__Komagataeibacter;s__Komagata
d__Bacteria;p__Proteobacteria;c__Alphaproteobacteria;o__Sphingomonadales;f__Sphingomonadaceae;g__Sphingomonas;s__Sphingomon
d__Bacteria;p__Acidobacteriota;c__Acidobacteriae;o__Acidobacteriales;f__Acidobacteriales_unclassified;g__Acidobacteriales_u
```

d__Bacteria;p__Proteobacteria;c__Betaproteobacteria;o__Betaproteobacteria_unclassified;f__Betaproteobacteria_unclassified;g
d__Bacteria;p__Proteobacteria;c__Gammaproteobacteria;o__Pseudomonadales;f__Porticoccaceae;g__SAR92_clade;s__SAR92_clade_unc
d__Bacteria;p__Proteobacteria;c__Alphaproteobacteria;o__Rhizobiales;f__Devosiaceae;g__Devosia;s__Devosia_unclassified
d__Bacteria;p__Patescibacteria;c__Parcubacteria;o__Candidatus_Nomurabacteria;f__Candidatus_Nomurabacteria_unclassified;g__C
d__Bacteria;p__Proteobacteria;c__Gammaproteobacteria;o__Aeromonadales;f__Aeromonadaceae;g__Aeromonas;s__Aeromonas_caviae
d__Bacteria;p__Myxococcota;c__Myxococcia;o__Myxococcales;f__Anaeromyxobacteraceae;g__Anaeromyxobacter;s__Anaeromyxobacter_u
d__Bacteria;p__Proteobacteria;c__Gammaproteobacteria;o__Pseudomonadales;f__Halieaceae;g__OM60(NOR5)_clade;s__OM60(NOR5)_cla
d__Bacteria;p__Actinobacteriota;c__Actinobacteria;o__Micrococcales;f__Microbacteriaceae;g__Humibacter;s__Humibacter_unclass
d__Bacteria;p__Bacteroidota;c__Bacteroidota_unclassified;o__Bacteroidota_unclassified;f__Bacteroidota_unclassified;g__Bacte
d__Bacteria;p__Actinobacteriota;c__Actinobacteriota;o__Actinobacteriota_unclassified;f__Actinobacteriota_unclassified;g__Ac
d__Bacteria;p__Firmicutes;c__Clostridia;o__Oscillospirales;f__Ruminococcaceae;g__Faecalibacterium;s__Faecalibacterium_uncla
d__Bacteria;p__Firmicutes;c__Clostridia;o__Lachnospirales;f__Lachnospiraceae;g__Lachnospiraceae_NK4A136_group;s__Lachnospir
d__Bacteria;p__Firmicutes;c__Bacilli;o__Bacillales;f__Bacillaceae;g__Bacillus;s__uncultured_Bacillus_sp.
d__Bacteria;p__Proteobacteria;c__Betaproteobacteria;o__Burkholderiales;f__Comamonadaceae;g__Comamonas;s__Comamonas_sp.
d__Bacteria;p__Firmicutes;c__Clostridia;o__Clostridiales;f__Peptococcaceae;g__Peptococcaceae_unclassified;s__Peptococcaceae
d__Bacteria;p__Proteobacteria;c__Gammaproteobacteria;o__Burkholderiales;f__Neisseriaceae;g__Neisseria;s__Neisseria_unclassi
d__Bacteria;p__Firmicutes;c__Bacilli;o__Lactobacillales;f__Streptococcaceae;g__Streptococcus;s__Streptococcus_iniae
d__Bacteria;p__Proteobacteria;c__Alphaproteobacteria;o__Rhizobiales;f__Beijerinckiaceae;g__Bosea;s__Bosea_unclassified
d__Bacteria;p__Firmicutes;c__Bacilli;o__Bacillales;f__Bacillaceae;g__Geobacillus;s__Geobacillus_unclassified
d__Bacteria;p__Firmicutes;c__Clostridia;o__Lachnospirales;f__Lachnospiraceae;g__Lachnospiraceae_UCG-010;s__Lachnospiraceae_
d__Bacteria;p__Firmicutes;c__Firmicutes_unclassified;o__Firmicutes_unclassified;f__Firmicutes_unclassified;g__Firmicutes_un
d__Bacteria;p__Proteobacteria;c__Gammaproteobacteria;o__Xanthomonadales;f__Xanthomonadaceae;g__Stenotrophomonas;s__Uncultur
d__Bacteria;p__Proteobacteria;c__Alphaproteobacteria;o__Sphingomonadales;f__Sphingomonadaceae;g__Sphingopyxis;s__Sphingopyx
d__Bacteria;p__Actinobacteriota;c__Actinobacteria;o__Micromonosporales;f__Micromonosporaceae;g__Micromonosporaceae_unclassi
d__Bacteria;p__Firmicutes;c__Clostridia;o__Clostridiales;f__Lachnospiraceae;g__Anaerotignum;s__Anaerotignum_sp.
d__Bacteria;p__Firmicutes;c__Negativicutes;o__Veillonellales-Selenomonadales;f__Veillonellaceae;g__Dialister;s__Dialister_u
d__Bacteria;p__Proteobacteria;c__Gammaproteobacteria;o__Burkholderiales;f__Oxalobacteraceae;g__Herbaspirillum;s__Herbaspiri
d__Bacteria;p__Proteobacteria;c__Gammaproteobacteria;o__Pseudomonadales;f__Moraxellaceae;g__Acinetobacter;s__Acinetobacter_
d__Bacteria;p__Proteobacteria;c__Alphaproteobacteria;o__Sphingomonadales;f__Sphingomonadaceae;g__Sphingopyxis;s__Sphingopyx
d__Bacteria;p__Firmicutes;c__Clostridia;o__Oscillospirales;f__Eubacterium]_coprostanoligenes_group;g__Eubacterium]_coprosta
d__Bacteria;p__Firmicutes;c__Bacilli;o__Lactobacillales;f__Lactobacillaceae;g__Ligilactobacillus;s__Ligilactobacillus_uncla
d__Bacteria;p__Proteobacteria;c__Betaproteobacteria;o__Burkholderiales;f__Comamonadaceae;g__Comamonas;s__uncultured_Comamon
d__Bacteria;p__Desulfobacterota;c__Desulfuromonadia;o__Bradymonadales;f__Bradymonadales_unclassified;g__Bradymonadales_uncl
d__Bacteria;p__Actinobacteriota;c__Actinobacteria;o__Micrococcales;f__Cellulomonadaceae;g__Cellulomonas;s__Cellulomonas_unc
d__Bacteria;p__Proteobacteria;c__Betaproteobacteria;o__Rhodocyclales;f__Azonexaceae;g__Dechloromonas;s__Dechloromonas_agita
d__Bacteria;p__Firmicutes;c__Clostridia;o__Clostridiales;f__Ruminococcaceae;g__Ruminococcaceae_unclassified;s__Ruminococcac
d__Bacteria;p__Bdellovibrionota;c__Bdellovibrionia;o__Bdellovibrionales;f__Bdellovibrionaceae;g__Bdellovibrio;s__Bdellovibr

d__Bacteria;p__Firmicutes;c__Bacilli;o__Acholeplasmatales;f__Acholeplasmataceae;g__Acholeplasma;s__Acholeplasma_unclassifie
d__Bacteria;p__Firmicutes;c__Bacilli;o__Lactobacillales;f__Streptococcaceae;g__Streptococcus;s__Streptococcus_mitis
d__Bacteria;p__Chloroflexi;c__Anaerolineae;o__Anaerolineales;f__Anaerolineaceae;g__Anaerolineaceae_unclassified;s__Anaeroli
d__Bacteria;p__Firmicutes;c__Clostridia;o__Thermincolales;f__Thermincolaceae;g__Thermincola;s__Thermincola_unclassified
d__Bacteria;p__Firmicutes;c__Clostridia;o__Oscillospirales;f__Oscillospiraceae;g__Oscillospiraceae_unclassified;s__Oscillos
d__Bacteria;p__Bacteroidota;c__Bacteroidia;o__Bacteroidales;f__Marinifilaceae;g__Odoribacter;s__Odoribacter_unclassified
d__Bacteria;p__Firmicutes;c__Clostridia;o__Lachnospirales;f__Lachnospiraceae;g__GCA-900066575;s__GCA-900066575_unclassified
d__Bacteria;p__Firmicutes;c__Clostridia;o__Peptostreptococcales-Tissierellales;f__Anaerovoracaceae;g__Eubacterium]_brachy_g
d__Bacteria;p__Bacteroidota;c__Bacteroidia;o__Bacteroidales;f__Muribaculaceae;g__Muribaculaceae_unclassified;s__Muribaculac
d__Bacteria;p__Firmicutes;c__Clostridia;o__Clostridiales;f__Lachnospiraceae;g__Lachnospiraceae_unclassified;s__Lachnospirac
d__Bacteria;p__Bacteroidota;c__Bacteroidia;o__Bacteroidales;f__Bacteroidaceae;g__Bacteroides;s__Bacteroides_massiliensis
d__Bacteria;p__Bacteroidota;c__Bacteroidia;o__Bacteroidales;f__Tannerellaceae;g__Parabacteroides;s__Parabacteroides_sp.
d__Bacteria;p__Proteobacteria;c__Betaproteobacteria;o__Burkholderiales;f__Comamonadaceae;g__Acidovorax;s__uncultured_Acidov
d__Bacteria;p__Proteobacteria;c__Gammaproteobacteria;o__Burkholderiales;f__Rhodocyclaceae;g__Dechlorosoma;s__Dechlorosoma_u
d__Bacteria;p__Desulfobacterota;c__Desulfovibrionia;o__Desulfovibrionales;f__Desulfovibrionaceae;g__Desulfovibrionaceae_unc
d__Bacteria;p__Proteobacteria;c__Alphaproteobacteria;o__Sphingomonadales;f__Sphingomonadaceae;g__Sphingopyxis;s__Sphingopyx
d__Bacteria;p__Proteobacteria;c__Betaproteobacteria;o__Burkholderiales;f__Comamonadaceae;g__Comamonas;s__uncultured_Comamon
d__Bacteria;p__Bacteroidota;c__Bacteroidia;o__Bacteroidales;f__Prevotellaceae;g__Alloprevotella;s__Alloprevotella_unclassif
d__Bacteria;p__Firmicutes;c__Clostridia;o__Oscillospirales;f__Ruminococcaceae;g__Ruminococcus;s__Ruminococcus_unclassified
d__Bacteria;p__Cyanobacteria;c__Cyanobacteriia;o__Chloroplast;f__Chloroplast_unclassified;g__Chloroplast_unclassified;s__Ch
d__Bacteria;p__Proteobacteria;c__Alphaproteobacteria;o__Rhodobacterales;f__Rhodobacteraceae;g__Yoonia-Loktanella;s__Yoonia-
d__Bacteria;p__Firmicutes;c__Clostridia;o__Peptostreptococcales-Tissierellales;f__Peptostreptococcaceae;g__Peptostreptococc
d__Bacteria;p__Firmicutes;c__Clostridia;o__Oscillospirales;f__Oscillospiraceae;g__Oscillospiraceae_unclassified;s__Oscillos
d__Bacteria;p__Firmicutes;c__Bacilli;o__Erysipelotrichales;f__Erysipelotrichaceae;g__Erysipelotrichaceae_unclassified;s__Er
d__Bacteria;p__Firmicutes;c__Negativicutes;o__Veillonellales-Selenomonadales;f__Veillonellaceae;g__Megasphaera;s__Megasphae
d__Bacteria;p__Bacteroidota;c__Bacteroidia;o__Flavobacteriales;f__Flavobacteriaceae;g__NS5_marine_group;s__NS5_marine_group
d__Bacteria;p__Nitrospirota;c__Nitrospiria;o__Nitrospirales;f__Nitrospiraceae;g__Nitrospira;s__Nitrospira_unclassified
d__Bacteria;p__Firmicutes;c__Bacilli;o__Bacillales;f__Sporolactobacillaceae;g__Sporolactobacillaceae_unclassified;s__Sporol
d__Bacteria;p__Proteobacteria;c__Proteobacteria_unclassified;o__Proteobacteria_unclassified;f__Proteobacteria_unclassified;
d__Bacteria;p__Proteobacteria;c__Alphaproteobacteria;o__SAR11_clade;f__Clade_II;g__Clade_II_unclassified;s__Clade_II_unclas
d__Bacteria;p__Firmicutes;c__Clostridia;o__Clostridiales;f__Lachnospiraceae;g__Lachnospiraceae_unclassified;s__Lachnospirac
d__Bacteria;p__Firmicutes;c__Clostridia;o__Clostridiales;f__Lachnospiraceae;g__Blautia;s__uncultured_Blautia_sp.
d__Bacteria;p__Proteobacteria;c__Gammaproteobacteria;o__Pseudomonadales;f__Moraxellaceae;g__Perlucidibaca;s__Perlucidibaca_
d__Bacteria;p__Marinimicrobia_(SAR406_clade);c__Marinimicrobia_(SAR406_clade)_unclassified;o__Marinimicrobia_(SAR406_clade)
d__Bacteria;p__Proteobacteria;c__Gammaproteobacteria;o__Xanthomonadales;f__Xanthomonadaceae;g__Stenotrophomonas;s__uncultur
d__Bacteria;p__Actinobacteriota;c__Actinobacteriota;o__Actinobacteriota_unclassified;f__Actinobacteriota_unclassified;g__Ac
d__Bacteria;p__Proteobacteria;c__Gammaproteobacteria;o__Pseudomonadales;f__Halieaceae;g__Luminiphilus;s__Luminiphilus_uncla

d__Bacteria;p__Proteobacteria;c__Alphaproteobacteria;o__Rhizobiales;f__Rhizobiaceae;g__Aliihoeflea;s__Aliihoeflea_unclassif
d__Bacteria;p__Actinobacteriota;c__Acidimicrobiia;o__Actinomarinales;f__Actinomarinaceae;g__Candidatus_Actinomarina;s__Cand
d__Bacteria;p__Proteobacteria;c__Gammaproteobacteria;o__Pseudomonadales;f__Halieaceae;g__Halioglobus;s__Halioglobus_unclass
d__Bacteria;p__Actinobacteriota;c__Actinobacteriota;o__Actinobacteriota_unclassified;f__Actinobacteriota_unclassified;g__Ac
d__Bacteria;p__Proteobacteria;c__Gammaproteobacteria;o__Burkholderiales;f__Nitrosomonadaceae;g__MND1;s__MND1_unclassified
d__Bacteria;p__Proteobacteria;c__Alphaproteobacteria;o__Rhodobacterales;f__Rhodobacteraceae;g__Rhodobacter;s__uncultured_Rh
d__Bacteria;p__Proteobacteria;c__Alphaproteobacteria;o__Rhodobacterales;f__Rhodobacteraceae;g__Paracoccus;s__Paracoccus_aer
d__Bacteria;p__Firmicutes;c__Clostridia;o__Oscillospirales;f__Eubacterium]_coprostanoligenes_group;g__Eubacterium]_coprosta
d__Bacteria;p__Firmicutes;c__Clostridia;o__Clostridiales;f__Lachnospiraceae;g__Lachnospiraceae_unclassified;s__Lachnospirac
d__Bacteria;p__Firmicutes;c__Clostridia;o__Oscillospirales;f__Oscillospiraceae;g__Intestinimonas;s__Intestinimonas_unclassi
d__Bacteria;p__Nitrospirota;c__Nitrospiria;o__Nitrospirales;f__Nitrospiraceae;g__Nitrospira;s__Nitrospira_unclassified
d__Bacteria;p__Proteobacteria;c__Alphaproteobacteria;o__Micropepsales;f__Micropepsaceae;g__Micropepsaceae_unclassified;s__M
d__Bacteria;p__Actinobacteriota;c__Actinobacteriota;o__Actinobacteriota_unclassified;f__Actinobacteriota_unclassified;g__Ac
d__Bacteria;p__Proteobacteria;c__Gammaproteobacteria;o__Burkholderiales;f__Sutterellaceae;g__Sutterella;s__Sutterella_uncla
d__Bacteria;p__Bacteroidota;c__Bacteroidia;o__Bacteroidales;f__Prevotellaceae;g__Prevotella;s__uncultured_Prevotella_sp.
d__Bacteria;p__Proteobacteria;c__Alphaproteobacteria;o__Sphingomonadales;f__Sphingomonadaceae;g__Sphingomonas;s__Sphingomon
d__Bacteria;p__Firmicutes;c__Clostridia;o__Oscillospirales;f__Ruminococcaceae;g__Incertae_Sedis;s__Incertae_Sedis_unclassif
d__Bacteria;p__Firmicutes;c__Bacilli;o__Paenibacillales;f__Paenibacillaceae;g__Paenibacillus;s__Paenibacillus_unclassified
d__Bacteria;p__WPS-2;c__WPS-2_unclassified;o__WPS-2_unclassified;f__WPS-2_unclassified;g__WPS-2_unclassified;s__WPS-2_uncla
d__Bacteria;p__Proteobacteria;c__Alphaproteobacteria;o__Rhodobacterales;f__Rhodobacteraceae;g__Sulfitobacter;s__Sulfitobact
d__Bacteria;p__Proteobacteria;c__Gammaproteobacteria;o__Pseudomonadales;f__Spongiibacteraceae;g__Litorivivens;s__Litorivive
d__Bacteria;p__Planctomycetota;c__Planctomycetes;o__Planctomycetales;f__Gimesiaceae;g__Gimesia;s__Gimesia_unclassified
d__Bacteria;p__Cyanobacteria;c__Cyanobacteriia;o__Chloroplast;f__Chloroplast_unclassified;g__Chloroplast_unclassified;s__Ch
d__Bacteria;p__Firmicutes;c__Clostridia;o__Oscillospirales;f__Eubacterium]_coprostanoligenes_group;g__Eubacterium]_coprosta
d__Bacteria;p__Firmicutes;c__Clostridia;o__Clostridia_UCG-014;f__Clostridia_UCG-014_unclassified;g__Clostridia_UCG-014_uncl
d__Bacteria;p__Firmicutes;c__Clostridia;o__Lachnospirales;f__Lachnospiraceae;g__Robinsoniella;s__Robinsoniella_unclassified
d__Bacteria;p__Firmicutes;c__Clostridia;o__Clostridiales;f__Ruminococcaceae;g__Ruminococcaceae_unclassified;s__Ruminococcac
d__Bacteria;p__Firmicutes;c__Negativicutes;o__Veillonellales-Selenomonadales;f__Veillonellaceae;g__Veillonella;s__Veillonel
d__Bacteria;p__Firmicutes;c__Clostridia;o__Clostridiales;f__Lachnospiraceae;g__Lachnospiraceae_unclassified;s__Lachnospirac
d__Bacteria;p__Firmicutes;c__Bacilli;o__Lactobacillales;f__Streptococcaceae;g__Streptococcus;s__Streptococcus_danieliae
d__Bacteria;p__Proteobacteria;c__Gammaproteobacteria;o__Oceanospirillales;f__Oceanospirillales_unclassified;g__Oceanospiril
d__Bacteria;p__Actinobacteriota;c__Actinobacteria;o__Bifidobacteriales;f__Bifidobacteriaceae;g__Bifidobacterium;s__Bifidoba
d__Bacteria;p__Proteobacteria;c__Gammaproteobacteria;o__Pseudomonadales;f__Porticoccaceae;g__SAR92_clade;s__SAR92_clade_unc
d__Bacteria;p__Firmicutes;c__Bacilli;o__Bacillales;f__Bacillaceae;g__Bacillus;s__Bacillus_acidicola
d__Bacteria;p__Bacteroidota;c__Bacteroidia;o__Bacteroidales;f__Bacteroidaceae;g__Bacteroides;s__Bacteroides_thetaiotaomicro
d__Bacteria;p__Firmicutes;c__Clostridia;o__Oscillospirales;f__Ruminococcaceae;g__Incertae_Sedis;s__Incertae_Sedis_unclassif
d__Bacteria;p__Firmicutes;c__Bacilli;o__Bacillales;f__Bacillaceae;g__Bacillus;s__Bacillus_paralicheniformis

d__Bacteria;p__Firmicutes;c__Clostridia;o__Clostridiales;f__Lachnospiraceae;g__Lachnospiraceae_unclassified;s__Lachnospirac
d__Bacteria;p__Verrucomicrobiota;c__Verrucomicrobiae;o__Verrucomicrobiales;f__Akkermansiaceae;g__Akkermansia;s__Akkermansia
d__Bacteria;p__Bacteroidota;c__Ignavibacteria;o__Ignavibacteriales;f__PHOS-HE36;g__PHOS-HE36_unclassified;s__PHOS-HE36_uncl
d__Bacteria;p__Proteobacteria;c__Alphaproteobacteria;o__Rhodospirillales;f__AEGEAN-169_marine_group;g__AEGEAN-169_marine_gr
d__Bacteria;p__Proteobacteria;c__Alphaproteobacteria;o__Sphingomonadales;f__Sphingomonadaceae;g__Sphingopyxis;s__uncultured
d__Bacteria;p__Acidobacteriota;c__Acidobacteriota_unclassified;o__Acidobacteriota_unclassified;f__Acidobacteriota_unclassif
d__Bacteria;p__Bacteroidota;c__Bacteroidia;o__Bacteroidales;f__Rikenellaceae;g__Alistipes;s__Alistipes_massiliensis
d__Bacteria;p__Proteobacteria;c__Gammaproteobacteria;o__Enterobacterales;f__Alteromonadaceae;g__Glaciecola;s__Glaciecola_un
d__Bacteria;p__Firmicutes;c__Clostridia;o__Oscillospirales;f__Eubacterium]_coprostanoligenes_group;g__Eubacterium]_coprosta
d__Bacteria;p__Actinobacteriota;c__Actinobacteriota;o__Actinobacteriota_unclassified;f__Actinobacteriota_unclassified;g__Ac
d__Bacteria;p__Myxococcota;c__Polyangia;o__Haliangiales;f__Haliangiaceae;g__Haliangium;s__Haliangium_unclassified
d__Bacteria;p__Firmicutes;c__Bacilli;o__Bacillales;f__Bacillaceae;g__Bacillus;s__uncultured_Bacillus_sp.
d__Bacteria;p__Firmicutes;c__Clostridia;o__Eubacteriales;f__Anaerofustaceae;g__Anaerofustis;s__Anaerofustis_unclassified
d__Bacteria;p__Firmicutes;c__Clostridia;o__Clostridiales;f__Ruminococcaceae;g__Ruminococcaceae_unclassified;s__Ruminococcac
d__Bacteria;p__Proteobacteria;c__Alphaproteobacteria;o__Rhizobiales;f__Beijerinckiaceae;g__Methylobacterium-Methylorubrum;s
d__Bacteria;p__Proteobacteria;c__Betaproteobacteria;o__Burkholderiales;f__Alcaligenaceae;g__Alcaligenaceae_unclassified;s__
d__Bacteria;p__Firmicutes;c__Clostridia;o__Clostridiales;f__Eubacteriaceae;g__Intestinibacillus;s__Intestinibacillus_sp._Ma
d__Bacteria;p__Actinobacteriota;c__Actinobacteriota;o__Actinobacteriota_unclassified;f__Actinobacteriota_unclassified;g__Ac
d__Bacteria;p__Actinobacteriota;c__Actinobacteriota;o__Propionibacteriales;f__Propionibacteriaceae;g__Propionibacteriaceae_
d__Bacteria;p__Deinococcota;c__Deinococci;o__Deinococcales;f__Trueperaceae;g__Truepera;s__Truepera_unclassified
d__Bacteria;p__Firmicutes;c__Clostridia;o__Clostridiales;f__Ruminococcaceae;g__Ruminococcaceae_unclassified;s__Ruminococcac
d__Bacteria;p__Actinobacteriota;c__Actinobacteria;o__Micrococcales;f__Micrococcaceae;g__Micrococcus;s__Micrococcus_unclassi
d__Bacteria;p__Proteobacteria;c__Gammaproteobacteria;o__Pseudomonadales;f__Moraxellaceae;g__Acinetobacter;s__Acinetobacter_
d__Bacteria;p__Proteobacteria;c__Betaproteobacteria;o__Betaproteobacteria_unclassified;f__Betaproteobacteria_unclassified;g
d__Bacteria;p__Actinobacteriota;c__Actinobacteria;o__Corynebacteriales;f__Corynebacteriaceae;g__Corynebacterium;s__Coryneba
d__Bacteria;p__Actinobacteriota;c__Actinobacteria;o__Micrococcales;f__Micrococcaceae;g__Rothia;s__Rothia_unclassified
d__Bacteria;p__Proteobacteria;c__Betaproteobacteria;o__Burkholderiales;f__Burkholderiales_unclassified;g__Burkholderiales_u
d__Bacteria;p__Bacteroidota;c__Bacteroidia;o__Flavobacteriales;f__Flavobacteriaceae;g__Flavobacterium;s__Flavobacterium_unc
d__Bacteria;p__Firmicutes;c__Bacilli;o__Lactobacillales;f__Streptococcaceae;g__Streptococcus;s__uncultured_Streptococcus_sp
d__Bacteria;p__Proteobacteria;c__Gammaproteobacteria;o__Burkholderiales;f__Burkholderiaceae;g__Lautropia;s__Lautropia_uncla
d__Bacteria;p__Actinobacteriota;c__Actinobacteria;o__Frankiales;f__Acidothermaceae;g__Acidothermus;s__Acidothermus_unclassi
d__Bacteria;p__Fusobacteriota;c__Fusobacteriia;o__Fusobacteriales;f__Leptotrichiaceae;g__Leptotrichia;s__Leptotrichia_uncla
d__Bacteria;p__Nitrospirota;c__Leptospirillia;o__Leptospirillales;f__Leptospirillaceae;g__Leptospirillum;s__Leptospirillum_
d__Bacteria;p__Firmicutes;c__Firmicutes_unclassified;o__Firmicutes_unclassified;f__Firmicutes_unclassified;g__Firmicutes_un
d__Bacteria;p__Bacteroidota;c__Bacteroidia;o__Bacteroidales;f__Prevotellaceae;g__Prevotellaceae_UCG-003;s__Prevotellaceae_U
d__Bacteria;p__Bacteroidota;c__Sphingobacteriia;o__Sphingobacteriales;f__Sphingobacteriales_unclassified;g__Sphingobacteria
d__Bacteria;p__Bacteroidota;c__Bacteroidia;o__Bacteroidales;f__Prevotellaceae;g__Alloprevotella;s__Alloprevotella_unclassif

d__Bacteria;p__Actinobacteriota;c__Coriobacteriia;o__Coriobacteriales;f__Eggerthellaceae;g__Eggerthella;s__Eggerthella_uncl
d__Bacteria;p__Firmicutes;c__Clostridia;o__Oscillospirales;f__Oscillospiraceae;g__UCG-005;s__UCG-005_unclassified
d__Bacteria;p__Actinobacteriota;c__Actinobacteria;o__Micrococcales;f__Microbacteriaceae;g__Microbacterium;s__Microbacterium
d__Bacteria;p__Firmicutes;c__Clostridia;o__Lachnospirales;f__Lachnospiraceae;g__Eubacterium]_eligens_group;s__Eubacterium]_
d__Bacteria;p__Proteobacteria;c__Alphaproteobacteria;o__Rhizobiales;f__Bradyrhizobiaceae;g__Bradyrhizobium;s__uncultured_Br
d__Bacteria;p__Bacteroidota;c__Bacteroidia;o__Bacteroidales;f__Bacteroidaceae;g__Bacteroides;s__uncultured_Bacteroides_sp.
d__Bacteria;p__Firmicutes;c__Clostridia;o__Clostridia_unclassified;f__Clostridia_unclassified;g__Clostridia_unclassified;s_
d__Bacteria;p__Firmicutes;c__Bacilli;o__Staphylococcales;f__Staphylococcaceae;g__Staphylococcus;s__Staphylococcus_unclassif
d__Bacteria;p__Firmicutes;c__Bacilli;o__Lactobacillales;f__Streptococcaceae;g__Streptococcus;s__Streptococcus_unclassified
d__Bacteria;p__Campylobacterota;c__Campylobacteria;o__Campylobacterales;f__Helicobacteraceae;g__Helicobacter;s__Helicobacte
d__Bacteria;p__Proteobacteria;c__Gammaproteobacteria;o__Burkholderiales;f__Comamonadaceae;g__Rhodoferax;s__Rhodoferax_uncla
d__Bacteria;p__Bacteroidota;c__Bacteroidia;o__Bacteroidales;f__Rikenellaceae;g__Alistipes;s__Alistipes_unclassified
d__Bacteria;p__Firmicutes;c__Bacilli;o__Brevibacillales;f__Brevibacillaceae;g__Brevibacillus;s__Brevibacillus_unclassified
d__Bacteria;p__Proteobacteria;c__Alphaproteobacteria;o__Caulobacterales;f__Caulobacteraceae;g__Brevundimonas;s__Brevundimon
d__Bacteria;p__Desulfobacterota;c__Desulfovibrionia;o__Desulfovibrionales;f__Desulfovibrionaceae;g__Desulfovibrionaceae_unc
d__Bacteria;p__Proteobacteria;c__Gammaproteobacteria;o__Xanthomonadales;f__Xanthomonadaceae;g__Stenotrophomonas;s__Stenotro
d__Bacteria;p__Firmicutes;c__Clostridia;o__Oscillospirales;f__Ruminococcaceae;g__Ruminococcus;s__Ruminococcus_unclassified
d__Bacteria;p__Proteobacteria;c__Alphaproteobacteria;o__Rhizobiales;f__Xanthobacteraceae;g__Bradyrhizobium;s__Bradyrhizobiu
d__Bacteria;p__Firmicutes;c__Clostridia;o__Lachnospirales;f__Lachnospiraceae;g__Eubacterium]_ventriosum_group;s__Eubacteriu
d__Bacteria;p__Firmicutes;c__Clostridia;o__Thermincolales;f__Thermincolaceae;g__Thermincola;s__Thermincola_unclassified
d__Bacteria;p__Bacteroidota;c__Bacteroidia;o__Bacteroidales;f__Muribaculaceae;g__Muribaculaceae_unclassified;s__Muribaculac
d__Bacteria;p__Proteobacteria;c__Gammaproteobacteria;o__Burkholderiales;f__Methylophilaceae;g__Methylophilus;s__Methylophil
d__Bacteria;p__Firmicutes;c__Clostridia;o__Clostridiales;f__Caloramatoraceae;g__Fonticella;s__Fonticella_unclassified
d__Bacteria;p__Bacteroidota;c__Bacteroidia;o__Bacteroidales;f__Prevotellaceae;g__Prevotella;s__Prevotella_unclassified
d__Bacteria;p__Desulfobacterota;c__Desulfuromonadia;o__Bradymonadales;f__Bradymonadales_unclassified;g__Bradymonadales_uncl
d__Bacteria;p__Firmicutes;c__Clostridia;o__Oscillospirales;f__Butyricicoccaceae;g__UCG-009;s__UCG-009_unclassified
d__Bacteria;p__Firmicutes;c__Clostridia;o__Clostridia_vadinBB60_group;f__Clostridia_vadinBB60_group_unclassified;g__Clostri
d__Bacteria;p__Firmicutes;c__Clostridia;o__Clostridia_UCG-014;f__Clostridia_UCG-014_unclassified;g__Clostridia_UCG-014_uncl
d__Bacteria;p__Bacteroidota;c__Bacteroidia;o__Bacteroidales;f__Muribaculaceae;g__Muribaculaceae_unclassified;s__Muribaculac
d__Bacteria;p__Bacteroidota;c__Bacteroidia;o__Sphingobacteriales;f__NS11-12_marine_group;g__NS11-12_marine_group_unclassifi
d__Bacteria;p__Firmicutes;c__Clostridia;o__Oscillospirales;f__Butyricicoccaceae;g__Butyricicoccaceae_unclassified;s__Butyri
d__Bacteria;p__Proteobacteria;c__Betaproteobacteria;o__Burkholderiales;f__Comamonadaceae;g__Delftia;s__uncultured_Delftia_s
d__Bacteria;p__Firmicutes;c__Clostridia;o__Oscillospirales;f__Ruminococcaceae;g__Anaerotruncus;s__Anaerotruncus_unclassifie
d__Bacteria;p__Firmicutes;c__Clostridia;o__Clostridia_vadinBB60_group;f__Clostridia_vadinBB60_group_unclassified;g__Clostri
d__Bacteria;p__Bacteroidota;c__Bacteroidia;o__Bacteroidales;f__Tannerellaceae;g__Parabacteroides;s__Parabacteroides_sp.
d__Bacteria;p__Bacteroidota;c__Bacteroidia;o__Bacteroidales;f__Muribaculaceae;g__Muribaculaceae_unclassified;s__Muribaculac
d__Bacteria;p__Proteobacteria;c__Gammaproteobacteria;o__Enterobacterales;f__Aeromonadaceae;g__Aeromonas;s__Aeromonas_unclas

d__Bacteria;p__Actinobacteriota;c__Actinobacteria;o__Micrococcales;f__Brevibacteriaceae;g__Brevibacterium;s__Brevibacterium
d__Bacteria;p__Proteobacteria;c__Gammaproteobacteria;o__Pseudomonadales;f__Moraxellaceae;g__Cavicella;s__Cavicella_unclassi
d__Bacteria;p__Proteobacteria;c__Gammaproteobacteria;o__Burkholderiales;f__Alcaligenaceae;g__Achromobacter;s__Achromobacter
d__Bacteria;p__Firmicutes;c__Clostridia;o__Oscillospirales;f__Eubacterium]_coprostanoligenes_group;g__Eubacterium]_coprosta
d__Bacteria;p__Proteobacteria;c__Gammaproteobacteria;o__Burkholderiales;f__Comamonadaceae;g__Acidovorax;s__Acidovorax_uncla
d__Bacteria;p__Proteobacteria;c__Gammaproteobacteria;o__Pseudomonadales;f__Moraxellaceae;g__Acinetobacter;s__uncultured_Aci
d__Bacteria;p__Bacteroidota;c__Bacteroidia;o__Bacteroidales;f__Bacteroidaceae;g__Bacteroides;s__Bacteroides_unclassified
d__Bacteria;p__Firmicutes;c__Clostridia;o__Clostridia_vadinBB60_group;f__Clostridia_vadinBB60_group_unclassified;g__Clostri
d__Bacteria;p__Proteobacteria;c__Gammaproteobacteria;o__Enterobacterales;f__Pasteurellaceae;g__Aggregatibacter;s__Aggregati
d__Bacteria;p__Proteobacteria;c__Gammaproteobacteria;o__Enterobacterales;f__Alteromonadaceae;g__Glaciecola;s__Glaciecola_un
d__Bacteria;p__Bacteroidota;c__Bacteroidia;o__Bacteroidales;f__Rikenellaceae;g__Alistipes;s__Alistipes_inops
d__Bacteria;p__Gemmatimonadota;c__Gemmatimonadetes;o__Gemmatimonadales;f__Gemmatimonadaceae;g__Gemmatimonadaceae_unclassifi
d__Bacteria;p__Cyanobacteria;c__Cyanobacteriia;o__Chloroplast;f__Chloroplast_unclassified;g__Chloroplast_unclassified;s__Ch
d__Bacteria;p__Proteobacteria;c__Gammaproteobacteria;o__Enterobacterales;f__Hafniaceae;g__Edwardsiella;s__Edwardsiella_tard
d__Bacteria;p__Gemmatimonadota;c__Gemmatimonadota;o__Gemmatimonadales;f__Gemmatimonadaceae;g__Gemmatimonas;s__Gemmatimonas_
d__Bacteria;p__Proteobacteria;c__Alphaproteobacteria;o__Rhodospirillales;f__AEGEAN-169_marine_group;g__AEGEAN-169_marine_gr
d__Bacteria;p__Proteobacteria;c__Gammaproteobacteria;o__PLTA13;f__PLTA13_unclassified;g__PLTA13_unclassified;s__PLTA13_uncl
d__Bacteria;p__Firmicutes;c__Negativicutes;o__Veillonellales-Selenomonadales;f__Veillonellaceae;g__Dialister;s__Dialister_u
d__Bacteria;p__Cyanobacteria;c__Cyanobacteriia;o__Chloroplast;f__Chloroplast_unclassified;g__Chloroplast_unclassified;s__Ch
d__Bacteria;p__Firmicutes;c__Bacilli;o__Bacillales;f__Bacillaceae;g__Caldibacillus;s__Caldibacillus_debilis
d__Bacteria;p__Cyanobacteria;c__Cyanobacteriia;o__Chloroplast;f__Chloroplast_unclassified;g__Chloroplast_unclassified;s__Ch
d__Bacteria;p__Proteobacteria;c__Gammaproteobacteria;o__Xanthomonadales;f__Xanthomonadaceae;g__Stenotrophomonas;s__Stenotro
d__Bacteria;p__Firmicutes;c__Firmicutes_unclassified;o__Firmicutes_unclassified;f__Firmicutes_unclassified;g__Firmicutes_un
d__Bacteria;p__Actinobacteriota;c__Actinobacteria;o__Pseudonocardiales;f__Pseudonocardiaceae;g__Saccharopolyspora;s__Saccha
d__Bacteria;p__Firmicutes;c__Clostridia;o__Clostridia_UCG-014;f__Clostridia_UCG-014_unclassified;g__Clostridia_UCG-014_uncl
d__Bacteria;p__Planctomycetota;c__Planctomycetes;o__Planctomycetales;f__Schlesneriaceae;g__Planctopirus;s__Planctopirus_unc
d__Bacteria;p__Bacteroidota;c__Bacteroidia;o__Bacteroidales;f__Muribaculaceae;g__Muribaculaceae_unclassified;s__Muribaculac
d__Bacteria;p__Proteobacteria;c__Gammaproteobacteria;o__Enterobacterales;f__Pasteurellaceae;g__Aggregatibacter;s__Aggregati
d__Bacteria;p__Actinobacteriota;c__Actinobacteria;o__Propionibacteriales;f__Nocardioidaceae;g__Nocardioides;s__Nocardioides
d__Bacteria;p__Firmicutes;c__Clostridia;o__Clostridiales;f__Lachnospiraceae;g__Murimonas;s__Murimonas_intestini
d__Bacteria;p__Proteobacteria;c__Gammaproteobacteria;o__Gammaproteobacteria_unclassified;f__Gammaproteobacteria_unclassifie
d__Bacteria;p__Proteobacteria;c__Alphaproteobacteria;o__Caulobacterales;f__Caulobacteraceae;g__Brevundimonas;s__Brevundimon
d__unclassified;p__unclassified;c__unclassified;o__unclassified;f__unclassified;g__unclassified;s__unclassified
d__Bacteria;p__Proteobacteria;c__Gammaproteobacteria;o__Burkholderiales;f__Methylophilaceae;g__OM43_clade;s__OM43_clade_unc
d__Bacteria;p__Actinobacteriota;c__Actinobacteria;o__Frankiales;f__Sporichthyaceae;g__hgcI_clade;s__hgcI_clade_unclassified
d__Bacteria;p__Proteobacteria;c__Alphaproteobacteria;o__Alphaproteobacteria_unclassified;f__Alphaproteobacteria_unclassifie
d__Bacteria;p__Firmicutes;c__Clostridia;o__Lachnospirales;f__Lachnospiraceae;g__GCA-900066575;s__GCA-900066575_unclassified

d__Bacteria;p__Proteobacteria;c__Gammaproteobacteria;o__Pseudomonadales;f__Moraxellaceae;g__Acinetobacter;s__Acinetobacter_
d__Bacteria;p__Firmicutes;c__Clostridia;o__Clostridiales;f__Ruminococcaceae;g__Ruminococcaceae_unclassified;s__Ruminococcac
d__Bacteria;p__Cyanobacteria;c__Cyanobacteriia;o__Chloroplast;f__Chloroplast_unclassified;g__Chloroplast_unclassified;s__Ch
d__Bacteria;p__Bacteroidota;c__Bacteroidia;o__Bacteroidales;f__Marinifilaceae;g__Butyricimonas;s__Butyricimonas_unclassifie
d__Bacteria;p__Firmicutes;c__Clostridia;o__Clostridiales;f__Lachnospiraceae;g__Lachnospiraceae_unclassified;s__Lachnospirac
d__Bacteria;p__Bacteroidota;c__Bacteroidia;o__Bacteroidales;f__Rikenellaceae;g__Alistipes;s__Alistipes_sp._enrichment_cultu
d__Bacteria;p__Proteobacteria;c__Gammaproteobacteria;o__PLTA13;f__PLTA13_unclassified;g__PLTA13_unclassified;s__PLTA13_uncl
d__Bacteria;p__Proteobacteria;c__Alphaproteobacteria;o__Rhizobiales;f__Rhizobiaceae;g__Allorhizobium-Neorhizobium-Pararhizo
d__Bacteria;p__Proteobacteria;c__Gammaproteobacteria;o__Pseudomonadales;f__Porticoccaceae;g__SAR92_clade;s__SAR92_clade_unc
d__Bacteria;p__Firmicutes;c__Clostridia;o__Clostridiales;f__Lachnospiraceae;g__Lachnospiraceae_unclassified;s__Lachnospirac
d__Bacteria;p__Proteobacteria;c__Gammaproteobacteria;o__Burkholderiales;f__Oxalobacteraceae;g__Massilia;s__Massilia_unclass
d__Bacteria;p__Proteobacteria;c__Gammaproteobacteria;o__Legionellales;f__Legionellaceae;g__Legionella;s__uncultured_Legione
d__Bacteria;p__Desulfobacterota;c__Desulfobacterota_unclassified;o__Desulfobacterota_unclassified;f__Desulfobacterota_uncla
d__Bacteria;p__Proteobacteria;c__Gammaproteobacteria;o__Legionellales;f__Legionellaceae;g__Legionellaceae_unclassified;s__L
d__Bacteria;p__Firmicutes;c__Bacilli;o__Lactobacillales;f__Lactobacillaceae;g__HT002;s__HT002_unclassified
d__Bacteria;p__Firmicutes;c__Bacilli;o__Staphylococcales;f__Gemellaceae;g__Gemella;s__Gemella_unclassified
d__Bacteria;p__Firmicutes;c__Clostridia;o__Oscillospirales;f__Oscillospiraceae;g__NK4A214_group;s__NK4A214_group_unclassifi
d__Bacteria;p__Proteobacteria;c__Betaproteobacteria;o__Betaproteobacteria_unclassified;f__Betaproteobacteria_unclassified;g
d__Bacteria;p__Firmicutes;c__Bacilli;o__Lactobacillales;f__Lactobacillaceae;g__Ligilactobacillus;s__Ligilactobacillus_uncla
d__Bacteria;p__Firmicutes;c__Clostridia;o__Lachnospirales;f__Lachnospiraceae;g__Eubacterium]_fissicatena_group;s__Eubacteri
d__Bacteria;p__Firmicutes;c__Clostridia;o__Oscillospirales;f__Oscillospiraceae;g__Intestinimonas;s__Intestinimonas_unclassi
d__Bacteria;p__Bacteroidota;c__Bacteroidia;o__Sphingobacteriales;f__Sphingobacteriaceae;g__Sphingobacterium;s__Sphingobacte
d__Bacteria;p__Planctomycetota;c__Planctomycetes;o__Planctomycetales;f__Gimesiaceae;g__Gimesiaceae_unclassified;s__Gimesiac
d__Bacteria;p__Bacteroidota;c__Bacteroidia;o__Cytophagales;f__Spirosomaceae;g__Spirosoma;s__Spirosoma_unclassified
d__Bacteria;p__Firmicutes;c__Clostridia;o__Clostridiales;f__Clostridiales_unclassified;g__Clostridiales_unclassified;s__Clo
d__Bacteria;p__Acidobacteriota;c__Vicinamibacteria;o__Vicinamibacterales;f__Vicinamibacteraceae;g__Vicinamibacteraceae_uncl
d__Bacteria;p__Firmicutes;c__Firmicutes_unclassified;o__Firmicutes_unclassified;f__Firmicutes_unclassified;g__Firmicutes_un
d__Bacteria;p__Firmicutes;c__Bacilli;o__Bacillales;f__Bacillaceae;g__Bacillus;s__Bacillus_licheniformis
d__Bacteria;p__Actinobacteriota;c__Actinobacteria;o__Corynebacteriales;f__Corynebacteriaceae;g__Lawsonella;s__Lawsonella_un
d__Bacteria;p__Firmicutes;c__Clostridia;o__Lachnospirales;f__Lachnospiraceae;g__Lachnospiraceae_UCG-004;s__Lachnospiraceae_
d__Bacteria;p__Actinobacteriota;c__Actinobacteria;o__Corynebacteriales;f__Corynebacteriaceae;g__Corynebacterium;s__Coryneba
d__Bacteria;p__Actinobacteriota;c__Coriobacteriia;o__Coriobacteriales;f__Atopobiaceae;g__Atopobium;s__Atopobium_unclassifie
d__Bacteria;p__Acidobacteriota;c__Acidobacteriae;o__Acidobacteriales;f__Acidobacteriaceae_(Subgroup_1);g__Edaphobacter;s__E
d__Bacteria;p__Actinobacteriota;c__Actinobacteria;o__Micrococcales;f__Microbacteriaceae;g__Microbacterium;s__Microbacterium
d__Bacteria;p__Nitrospirota;c__4-29-1;o__4-29-1_unclassified;f__4-29-1_unclassified;g__4-29-1_unclassified;s__4-29-1_unclas
d__Bacteria;p__Firmicutes;c__Clostridia;o__Monoglobales;f__Monoglobaceae;g__Monoglobus;s__Monoglobus_unclassified
d__Bacteria;p__Proteobacteria;c__Gammaproteobacteria;o__Burkholderiales;f__Rhodocyclaceae;g__Dechloromonas;s__Dechloromonas

d__Bacteria;p__Firmicutes;c__Clostridia;o__Clostridiales;f__Lachnospiraceae;g__Pseudobutyrivibrio;s__uncultured_Pseudobutyr
d__Bacteria;p__Firmicutes;c__Bacilli;o__Erysipelotrichales;f__Erysipelatoclostridiaceae;g__Erysipelatoclostridium;s__Erysip
d__Bacteria;p__Firmicutes;c__Clostridia;o__Clostridia_UCG-014;f__Clostridia_UCG-014_unclassified;g__Clostridia_UCG-014_uncl
d__Bacteria;p__Firmicutes;c__Bacilli;o__Lactobacillales;f__Enterococcaceae;g__Enterococcus;s__Enterococcus_sp.
d__Bacteria;p__Proteobacteria;c__Alphaproteobacteria;o__Rhodobacterales;f__Rhodobacteraceae;g__Yoonia-Loktanella;s__Yoonia-
d__Bacteria;p__Firmicutes;c__Bacilli;o__Erysipelotrichales;f__Erysipelotrichaceae;g__Turicibacter;s__Turicibacter_unclassif
d__Bacteria;p__Firmicutes;c__Clostridia;o__Oscillospirales;f__Ruminococcaceae;g__Incertae_Sedis;s__Incertae_Sedis_unclassif
d__Bacteria;p__Myxococcota;c__Polyangia;o__Haliangiales;f__Haliangiaceae;g__Haliangium;s__Haliangium_unclassified
d__Bacteria;p__Cyanobacteria;c__Cyanobacteriia;o__Chloroplast;f__Chloroplast_unclassified;g__Chloroplast_unclassified;s__Ch
d__Bacteria;p__Proteobacteria;c__Deltaproteobacteria;o__Desulfuromonadales;f__Geobacteraceae;g__Geobacter;s__uncultured_Geo
d__Bacteria;p__Actinobacteriota;c__Actinobacteria;o__Corynebacteriales;f__Mycobacteriaceae;g__Mycobacterium;s__Mycobacteriu
d__Bacteria;p__Firmicutes;c__Clostridia;o__Clostridiales;f__Ruminococcaceae;g__Ruminococcus;s__Ruminococcus_sp._ID1
d__Bacteria;p__Actinobacteriota;c__Coriobacteriia;o__Coriobacteriales;f__Coriobacteriaceae;g__Collinsella;s__Collinsella_un
d__Bacteria;p__Actinobacteriota;c__Actinobacteria;o__Micrococcales;f__Microbacteriaceae;g__Aurantimicrobium;s__Aurantimicro
d__Bacteria;p__Actinobacteriota;c__Actinobacteria;o__Pseudonocardiales;f__Pseudonocardiaceae;g__Saccharopolyspora;s__Saccha
d__Bacteria;p__Firmicutes;c__Bacilli;o__Erysipelotrichales;f__Erysipelotrichaceae;g__Erysipelothrix;s__Erysipelothrix_uncla
d__Bacteria;p__Firmicutes;c__Clostridia;o__Lachnospirales;f__Lachnospiraceae;g__Lachnospiraceae_NK4A136_group;s__Lachnospir
d__Bacteria;p__Bacteroidota;c__Bacteroidia;o__Bacteroidales;f__Muribaculaceae;g__Muribaculaceae_unclassified;s__Muribaculac
d__Bacteria;p__Firmicutes;c__Bacilli;o__Staphylococcales;f__Gemellaceae;g__Gemella;s__Gemella_unclassified
d__Bacteria;p__Bacteroidota;c__Bacteroidia;o__Sphingobacteriales;f__Sphingobacteriaceae;g__Pedobacter;s__Pedobacter_unclass
d__Bacteria;p__Bacteroidota;c__Bacteroidia;o__Bacteroidales;f__Prevotellaceae;g__Prevotellaceae_NK3B31_group;s__Prevotellac
d__Bacteria;p__Bacteroidota;c__Bacteroidia;o__Bacteroidales;f__Muribaculaceae;g__Muribaculaceae_unclassified;s__Muribaculac
d__Bacteria;p__Firmicutes;c__Clostridia;o__Oscillospirales;f__Ruminococcaceae;g__Ruminococcus;s__Ruminococcus_unclassified
d__Bacteria;p__Proteobacteria;c__Alphaproteobacteria;o__Rhizobiales;f__Rhizobiaceae;g__Phyllobacterium;s__Phyllobacterium_u
d__Bacteria;p__Proteobacteria;c__Alphaproteobacteria;o__Rhizobiales;f__Beijerinckiaceae;g__Methylobacterium-Methylorubrum;s
d__Bacteria;p__Verrucomicrobiota;c__Verrucomicrobiae;o__Verrucomicrobiales;f__Akkermansiaceae;g__Akkermansia;s__Akkermansia
d__Bacteria;p__Firmicutes;c__Clostridia;o__Lachnospirales;f__Lachnospiraceae;g__Ruminococcus]_gauvreauii_group;s__Ruminococ
d__Bacteria;p__Proteobacteria;c__Betaproteobacteria;o__Burkholderiales;f__Comamonadaceae;g__Acidovorax;s__uncultured_Acidov
d__Bacteria;p__Firmicutes;c__Clostridia;o__Clostridiales;f__Lachnospiraceae;g__Kineothrix;s__Kineothrix_sp.
d__Bacteria;p__Bacteroidota;c__Bacteroidia;o__Bacteroidales;f__Muribaculaceae;g__Muribaculaceae_unclassified;s__Muribaculac
d__Bacteria;p__Firmicutes;c__Bacilli;o__Bacillales;f__Marinococcaceae;g__Geomicrobium;s__Geomicrobium_unclassified
d__Bacteria;p__Firmicutes;c__Negativicutes;o__Veillonellales-Selenomonadales;f__Selenomonadaceae;g__Mitsuokella;s__Mitsuoke
d__unclassified;p__unclassified;c__unclassified;o__unclassified;f__unclassified;g__unclassified;s__unclassified
d__Bacteria;p__Firmicutes;c__Clostridia;o__Oscillospirales;f__Oscillospiraceae;g__Oscillibacter;s__Oscillibacter_unclassifi
d__Bacteria;p__Firmicutes;c__Bacilli;o__Staphylococcales;f__Staphylococcaceae;g__Staphylococcus;s__Staphylococcus_unclassif
d__Bacteria;p__Firmicutes;c__Bacilli;o__Staphylococcales;f__Staphylococcaceae;g__Staphylococcus;s__Staphylococcus_unclassif
d__Bacteria;p__Firmicutes;c__Bacilli;o__Staphylococcales;f__Staphylococcaceae;g__Staphylococcus;s__Staphylococcus_unclassif

d__Bacteria;p__Firmicutes;c__Clostridia;o__Peptococcales;f__Peptococcaceae;g__Peptococcus;s__Peptococcus_unclassified
d__Bacteria;p__Firmicutes;c__Clostridia;o__Lachnospirales;f__Lachnospiraceae;g__Lachnospira;s__Lachnospira_unclassified
d__Bacteria;p__Actinobacteriota;c__Actinobacteria;o__Bifidobacteriales;f__Bifidobacteriaceae;g__Bifidobacterium;s__Bifidoba
d__Bacteria;p__Firmicutes;c__Firmicutes_unclassified;o__Firmicutes_unclassified;f__Firmicutes_unclassified;g__Firmicutes_un
d__Bacteria;p__Proteobacteria;c__Alphaproteobacteria;o__Caulobacterales;f__Hyphomonadaceae;g__SWB02;s__SWB02_unclassified
d__Bacteria;p__Bacteroidota;c__Bacteroidia;o__Bacteroidales;f__Bacteroidaceae;g__Bacteroides;s__uncultured_Bacteroides_sp.
d__Bacteria;p__Firmicutes;c__Firmicutes_unclassified;o__Firmicutes_unclassified;f__Firmicutes_unclassified;g__Firmicutes_un
d__Bacteria;p__Patescibacteria;c__Saccharimonadia;o__Saccharimonadales;f__Saccharimonadaceae;g__Candidatus_Saccharimonas;s_
d__Bacteria;p__Firmicutes;c__Clostridia;o__Lachnospirales;f__Lachnospiraceae;g__Eubacterium]_xylanophilum_group;s__Eubacter
d__Bacteria;p__Firmicutes;c__Bacilli;o__Erysipelotrichales;f__Erysipelotrichaceae;g__Holdemanella;s__Holdemanella_unclassif
d__Bacteria;p__Actinobacteriota;c__Actinobacteria;o__Actinomycetales;f__Actinomycetaceae;g__Actinomyces;s__Actinomyces_uncl
d__Bacteria;p__Firmicutes;c__Clostridia;o__Lachnospirales;f__Lachnospiraceae;g__Tuzzerella;s__Tuzzerella_unclassified
d__Bacteria;p__Firmicutes;c__Clostridia;o__Clostridiales;f__Lachnospiraceae;g__Tyzzerella;s__Tyzzerella_sp.
d__Bacteria;p__Bacteroidota;c__Bacteroidia;o__Bacteroidales;f__Muribaculaceae;g__Muribaculaceae_unclassified;s__Muribaculac
d__Bacteria;p__Firmicutes;c__Clostridia;o__Lachnospirales;f__Lachnospiraceae;g__Lachnospiraceae_NK4A136_group;s__Lachnospir
d__Bacteria;p__Patescibacteria;c__Gracilibacteria;o__JGI_0000069-P22;f__JGI_0000069-P22_unclassified;g__JGI_0000069-P22_unc
d__Bacteria;p__Proteobacteria;c__Gammaproteobacteria;o__Xanthomonadales;f__Xanthomonadaceae;g__Stenotrophomonas;s__Stenotro
d__Bacteria;p__Firmicutes;c__Clostridia;o__Clostridiales;f__Lachnospiraceae;g__Lachnospiraceae_unclassified;s__Lachnospirac
d__Bacteria;p__Actinobacteriota;c__Actinobacteria;o__Corynebacteriales;f__Corynebacteriaceae;g__Corynebacterium;s__Coryneba
d__Bacteria;p__Firmicutes;c__Clostridia;o__Oscillospirales;f__Ruminococcaceae;g__Ruminococcus;s__Ruminococcus_unclassified
d__Bacteria;p__Firmicutes;c__Bacilli;o__Lactobacillales;f__Lactobacillaceae;g__Lactobacillus;s__Lactobacillus_crispatus
d__Bacteria;p__Cyanobacteria;c__Cyanobacteriia;o__Chloroplast;f__Chloroplast_unclassified;g__Chloroplast_unclassified;s__Ch
d__Bacteria;p__Proteobacteria;c__Alphaproteobacteria;o__Micavibrionales;f__Micavibrionales_unclassified;g__Micavibrionales_
d__Bacteria;p__Firmicutes;c__Clostridia;o__Clostridia_UCG-014;f__Clostridia_UCG-014_unclassified;g__Clostridia_UCG-014_uncl
d__Bacteria;p__Bacteroidota;c__Bacteroidia;o__Bacteroidales;f__Muribaculaceae;g__Muribaculum;s__Muribaculum_sp.
d__Bacteria;p__Proteobacteria;c__Betaproteobacteria;o__Burkholderiales;f__Comamonadaceae;g__Comamonadaceae_unclassified;s__
d__Bacteria;p__Actinobacteriota;c__Actinobacteriota;o__Micrococcales;f__Microbacteriaceae;g__Agreia;s__Agreia_pratensis
d__Bacteria;p__Firmicutes;c__Clostridia;o__Clostridiales;f__Lachnospiraceae;g__Roseburia;s__Roseburia_sp.
d__Bacteria;p__Firmicutes;c__Clostridia;o__Clostridiales;f__Lachnospiraceae;g__Lachnospiraceae_unclassified;s__Lachnospirac
d__Bacteria;p__Firmicutes;c__Clostridia;o__Lachnospirales;f__Lachnospiraceae;g__Lachnospiraceae_UCG-003;s__Lachnospiraceae_
d__Bacteria;p__Proteobacteria;c__Betaproteobacteria;o__Burkholderiales;f__Comamonadaceae;g__Comamonas;s__Comamonas_jiangdue
d__Bacteria;p__Firmicutes;c__Clostridia;o__Clostridiales;f__Caloramatoraceae;g__Fonticella;s__Fonticella_unclassified
d__Bacteria;p__Proteobacteria;c__Gammaproteobacteria;o__Burkholderiales;f__Rhodocyclaceae;g__Dechlorosoma;s__Dechlorosoma_u
d__Bacteria;p__Proteobacteria;c__Gammaproteobacteria;o__Xanthomonadales;f__Xanthomonadaceae;g__Stenotrophomonas;s__Stenotro
d__Bacteria;p__Firmicutes;c__Clostridia;o__Clostridiales;f__Clostridiaceae;g__Clostridium;s__Clostridium_sp._Clone-17
d__Bacteria;p__Firmicutes;c__Clostridia;o__Oscillospirales;f__Ruminococcaceae;g__UBA1819;s__UBA1819_unclassified
d__Bacteria;p__Proteobacteria;c__Gammaproteobacteria;o__Enterobacterales;f__Aeromonadaceae;g__Aeromonadaceae_unclassified;s

d__Bacteria;p__Firmicutes;c__Clostridia;o__Clostridiales;f__Ruminococcaceae;g__Acetivibrio;s__uncultured_Acetivibrio_sp.
d__Bacteria;p__Firmicutes;c__Firmicutes_unclassified;o__Firmicutes_unclassified;f__Firmicutes_unclassified;g__Firmicutes_un
d__Bacteria;p__Firmicutes;c__Clostridia;o__Clostridia_UCG-014;f__Clostridia_UCG-014_unclassified;g__Clostridia_UCG-014_uncl
d__Bacteria;p__Actinobacteriota;c__Coriobacteriia;o__Coriobacteriales;f__Eggerthellaceae;g__Senegalimassilia;s__Senegalimas
d__Bacteria;p__Proteobacteria;c__Alphaproteobacteria;o__Dongiales;f__Dongiaceae;g__Dongia;s__Dongia_unclassified
d__Bacteria;p__Firmicutes;c__Clostridia;o__Oscillospirales;f__Eubacterium]_coprostanoligenes_group;g__Eubacterium]_coprosta
d__Bacteria;p__Proteobacteria;c__Gammaproteobacteria;o__Xanthomonadales;f__Xanthomonadaceae;g__Stenotrophomonas;s__Stenotro
d__Bacteria;p__Firmicutes;c__Bacilli;o__Erysipelotrichales;f__Erysipelotrichaceae;g__Holdemanella;s__Holdemanella_unclassif
d__Bacteria;p__Nitrospirota;c__4-29-1;o__4-29-1_unclassified;f__4-29-1_unclassified;g__4-29-1_unclassified;s__4-29-1_unclas
d__Bacteria;p__Bacteroidota;c__Bacteroidia;o__Flavobacteriales;f__Cryomorphaceae;g__Vicingus;s__Vicingus_unclassified
d__Bacteria;p__Firmicutes;c__Bacilli;o__Lactobacillales;f__Lactobacillaceae;g__Leuconostoc;s__Leuconostoc_unclassified
d__Bacteria;p__Proteobacteria;c__Alphaproteobacteria;o__Rhodobacterales;f__Rhodobacteraceae;g__Amylibacter;s__Amylibacter_u
d__Bacteria;p__Verrucomicrobiota;c__Verrucomicrobiae;o__Pedosphaerales;f__Pedosphaeraceae;g__SCGC_AAA164-E04;s__SCGC_AAA164
d__Bacteria;p__Cyanobacteria;c__Cyanobacteriia;o__Chloroplast;f__Chloroplast_unclassified;g__Chloroplast_unclassified;s__Ch
d__Bacteria;p__Bacteroidota;c__Bacteroidota_unclassified;o__Bacteroidota_unclassified;f__Bacteroidota_unclassified;g__Bacte
d__Bacteria;p__Firmicutes;c__Thermacetogenia;o__Thermacetogeniales;f__Thermacetogeniaceae;g__Syntrophaceticus;s__Syntrophac
d__Bacteria;p__Firmicutes;c__Clostridia;o__Clostridiales;f__Ruminococcaceae;g__Ruminococcaceae_unclassified;s__Ruminococcac
d__Bacteria;p__Nitrospirota;c__Thermodesulfovibrionia;o__Thermodesulfovibrionia_unclassified;f__Thermodesulfovibrionia_uncl
d__Bacteria;p__Actinobacteriota;c__Thermoleophilia;o__Gaiellales;f__Gaiellales_unclassified;g__Gaiellales_unclassified;s__G
d__Bacteria;p__Actinobacteriota;c__Coriobacteriia;o__Coriobacteriales;f__Eggerthellaceae;g__DNF00809;s__DNF00809_unclassifi
d__Bacteria;p__Proteobacteria;c__Alphaproteobacteria;o__Puniceispirillales;f__Puniceispirillales_unclassified;g__Puniceispi
d__Bacteria;p__Bacteroidota;c__Flavobacteriia;o__Flavobacteriales;f__Flavobacteriaceae;g__Siansivirga;s__Siansivirga_jejuen
d__Bacteria;p__Proteobacteria;c__Gammaproteobacteria;o__Pseudomonadales;f__SAR86_clade;g__SAR86_clade_unclassified;s__SAR86
d__Bacteria;p__Firmicutes;c__Bacilli;o__Lactobacillales;f__Listeriaceae;g__Brochothrix;s__Brochothrix_unclassified
d__Bacteria;p__Firmicutes;c__Bacilli;o__Bacillales;f__Bacillaceae;g__Bacillus;s__Bacillus_farraginis
d__Bacteria;p__Proteobacteria;c__Alphaproteobacteria;o__Acetobacterales;f__Acetobacteraceae;g__Gluconacetobacter;s__Glucona
d__Bacteria;p__Proteobacteria;c__Alphaproteobacteria;o__Sphingomonadales;f__Sphingomonadaceae;g__Novosphingobium;s__Novosph
d__Bacteria;p__Actinobacteriota;c__Actinobacteria;o__Pseudonocardiales;f__Pseudonocardiaceae;g__Pseudonocardia;s__Pseudonoc
d__Bacteria;p__Firmicutes;c__Clostridia;o__Clostridia_UCG-014;f__Clostridia_UCG-014_unclassified;g__Clostridia_UCG-014_uncl
d__Bacteria;p__Proteobacteria;c__Alphaproteobacteria;o__Puniceispirillales;f__SAR116_clade;g__SAR116_clade_unclassified;s__
d__Bacteria;p__Firmicutes;c__Clostridia;o__Oscillospirales;f__Oscillospiraceae;g__Oscillibacter;s__Oscillibacter_unclassifi
d__Bacteria;p__Proteobacteria;c__Alphaproteobacteria;o__Rhodospirillales;f__AEGEAN-169_marine_group;g__AEGEAN-169_marine_gr
d__Bacteria;p__Planctomycetota;c__Planctomycetes;o__Gemmatales;f__Gemmataceae;g__Gemmataceae_unclassified;s__Gemmataceae_un
d__Bacteria;p__Actinobacteriota;c__Acidimicrobiia;o__IMCC26256;f__IMCC26256_unclassified;g__IMCC26256_unclassified;s__IMCC2
d__Bacteria;p__Firmicutes;c__Clostridia;o__Christensenellales;f__Christensenellaceae;g__Christensenellaceae_R-7_group;s__Ch
d__Bacteria;p__Bacteroidota;c__Bacteroidia;o__Bacteroidales;f__Rikenellaceae;g__Rikenella;s__Rikenella_unclassified
d__Bacteria;p__Bacteroidota;c__Bacteroidia;o__Flavobacteriales;f__Flavobacteriaceae;g__NS4_marine_group;s__NS4_marine_group

d__Bacteria;p__Proteobacteria;c__Gammaproteobacteria;o__Burkholderiales;f__Comamonadaceae;g__Pseudacidovorax;s__Pseudacidov
d__Bacteria;p__Nitrospirota;c__Nitrospiria;o__Nitrospirales;f__Nitrospiraceae;g__Nitrospira;s__Nitrospira_unclassified
d__Bacteria;p__Sva0485;c__Sva0485_unclassified;o__Sva0485_unclassified;f__Sva0485_unclassified;g__Sva0485_unclassified;s__S
d__Bacteria;p__Actinobacteriota;c__Actinobacteria;o__PeM15;f__PeM15_unclassified;g__PeM15_unclassified;s__PeM15_unclassifie
d__Bacteria;p__Firmicutes;c__Thermoanaerobacteria;o__Thermoanaerobacterales;f__Thermoanaerobacteraceae;g__Thermanaeromonas;
d__Bacteria;p__Proteobacteria;c__Betaproteobacteria;o__Betaproteobacteria_unclassified;f__Betaproteobacteria_unclassified;g
d__Bacteria;p__Bacteroidota;c__Bacteroidia;o__Bacteroidales;f__Prevotellaceae;g__Prevotella;s__Prevotella_unclassified
d__Bacteria;p__Proteobacteria;c__Gammaproteobacteria;o__Enterobacterales;f__Shewanellaceae;g__Shewanella;s__Shewanella_uncl
d__Bacteria;p__Firmicutes;c__Clostridia;o__Clostridia_UCG-014;f__Clostridia_UCG-014_unclassified;g__Clostridia_UCG-014_uncl
d__Bacteria;p__Firmicutes;c__Clostridia;o__Christensenellales;f__Christensenellaceae;g__Christensenellaceae_R-7_group;s__Ch
d__Bacteria;p__Firmicutes;c__Clostridia;o__Oscillospirales;f__Oscillospiraceae;g__UCG-005;s__UCG-005_unclassified
d__Bacteria;p__Proteobacteria;c__Gammaproteobacteria;o__Tenderiales;f__Tenderiaceae;g__Candidatus_Tenderia;s__Candidatus_Te
d__Bacteria;p__Firmicutes;c__Clostridia;o__Peptococcales;f__Peptococcaceae;g__Peptococcaceae_unclassified;s__Peptococcaceae
d__Bacteria;p__Proteobacteria;c__Gammaproteobacteria;o__Pseudomonadales;f__Porticoccaceae;g__SAR92_clade;s__SAR92_clade_unc
d__Bacteria;p__Firmicutes;c__Clostridia;o__Oscillospirales;f__Oscillospiraceae;g__Flavonifractor;s__Flavonifractor_unclassi
d__Bacteria;p__Proteobacteria;c__Alphaproteobacteria;o__Rhodobacterales;f__Rhodobacteraceae;g__Sulfitobacter;s__Sulfitobact
d__Bacteria;p__Firmicutes;c__Clostridia;o__Lachnospirales;f__Lachnospiraceae;g__Lachnospiraceae_UCG-010;s__Lachnospiraceae_
d__Bacteria;p__Firmicutes;c__Clostridia;o__Thermincolales;f__Thermincolaceae;g__Thermincola;s__Thermincola_unclassified
d__Bacteria;p__Synergistota;c__Synergistia;o__Synergistales;f__Synergistaceae;g__Cloacibacillus;s__Cloacibacillus_unclassif
d__Bacteria;p__Acidobacteriota;c__Acidobacteriae;o__Subgroup_2;f__Subgroup_2_unclassified;g__Subgroup_2_unclassified;s__Sub
d__Bacteria;p__Actinobacteriota;c__Actinobacteria;o__Propionibacteriales;f__Propionibacteriaceae;g__Brooklawnia;s__Brooklaw
d__Bacteria;p__Firmicutes;c__Clostridia;o__Peptostreptococcales-Tissierellales;f__Family_XI;g__Anaerosalibacter;s__Anaerosa
d__Bacteria;p__Firmicutes;c__Clostridia;o__Oscillospirales;f__Ruminococcaceae;g__Ruminococcus;s__Ruminococcus_unclassified
d__Bacteria;p__Proteobacteria;c__Alphaproteobacteria;o__Rhizobiales;f__Beijerinckiaceae;g__Methylobacterium-Methylorubrum;s
d__Bacteria;p__Bacteroidota;c__Bacteroidia;o__Flavobacteriales;f__Flavobacteriaceae;g__Ulvibacter;s__Ulvibacter_unclassifie
d__unclassified;p__unclassified;c__unclassified;o__unclassified;f__unclassified;g__unclassified;s__unclassified
d__Bacteria;p__Proteobacteria;c__Alphaproteobacteria;o__Sphingomonadales;f__Sphingomonadaceae;g__Sphingobium;s__Sphingobium
d__Bacteria;p__Desulfobacterota;c__Syntrophia;o__Syntrophales;f__Syntrophaceae;g__Syntrophus;s__Syntrophus_unclassified
d__Bacteria;p__Actinobacteriota;c__Acidimicrobiia;o__Microtrichales;f__Microtrichaceae;g__Sva0996_marine_group;s__Sva0996_m
d__Bacteria;p__Actinobacteriota;c__RBG-16-55-12;o__RBG-16-55-12_unclassified;f__RBG-16-55-12_unclassified;g__RBG-16-55-12_u
d__Bacteria;p__MBNT15;c__MBNT15_unclassified;o__MBNT15_unclassified;f__MBNT15_unclassified;g__MBNT15_unclassified;s__MBNT15
d__Bacteria;p__Proteobacteria;c__Alphaproteobacteria;o__Rhizobiales;f__Rhizobiales_Incertae_Sedis;g__Rhizobiales_Incertae_S
d__Bacteria;p__Proteobacteria;c__Alphaproteobacteria;o__Rhodobacterales;f__Rhodobacteraceae;g__Yoonia-Loktanella;s__Yoonia-
d__Bacteria;p__Proteobacteria;c__Gammaproteobacteria;o__Pseudomonadales;f__Marinomonadaceae;g__Marinomonas;s__Marinomonas_u
d__Bacteria;p__Firmicutes;c__Clostridia;o__Lachnospirales;f__Lachnospiraceae;g__Tuzzerella;s__Tuzzerella_unclassified
d__Bacteria;p__Proteobacteria;c__Alphaproteobacteria;o__Rhizobiales;f__Rhizobiales_Incertae_Sedis;g__Bauldia;s__Bauldia_unc
d__Bacteria;p__Firmicutes;c__Clostridia;o__Clostridia_UCG-014;f__Clostridia_UCG-014_unclassified;g__Clostridia_UCG-014_uncl

d__Bacteria;p__Bacteroidota;c__Bacteroidia;o__Bacteroidales;f__Marinifilaceae;g__Odoribacter;s__Odoribacter_unclassified
d__Bacteria;p__Firmicutes;c__Clostridia;o__Oscillospirales;f__Ruminococcaceae;g__Anaerotruncus;s__Anaerotruncus_unclassifie
d__Bacteria;p__SAR324_clade(Marine_group_B);c__SAR324_clade(Marine_group_B)_unclassified;o__SAR324_clade(Marine_group_B)_un
d__Bacteria;p__Firmicutes;c__Clostridia;o__Clostridiales;f__Clostridiales_unclassified;g__Clostridiales_unclassified;s__Clo
d__unclassified;p__unclassified;c__unclassified;o__unclassified;f__unclassified;g__unclassified;s__unclassified
d__unclassified;p__unclassified;c__unclassified;o__unclassified;f__unclassified;g__unclassified;s__unclassified
d__Bacteria;p__Proteobacteria;c__Gammaproteobacteria;o__Burkholderiales;f__Comamonadaceae;g__Limnohabitans;s__Limnohabitans
d__Bacteria;p__Proteobacteria;c__Alphaproteobacteria;o__Puniceispirillales;f__SAR116_clade;g__SAR116_clade_unclassified;s__
d__Bacteria;p__Actinobacteriota;c__Actinobacteria;o__Streptosporangiales;f__Nocardiopsaceae;g__Nocardiopsis;s__Nocardiopsis
d__Bacteria;p__Proteobacteria;c__Gammaproteobacteria;o__Burkholderiales;f__Nitrosomonadaceae;g__Ellin6067;s__Ellin6067_uncl
d__Bacteria;p__Proteobacteria;c__Alphaproteobacteria;o__Caulobacterales;f__Hyphomonadaceae;g__Hyphomonadaceae_unclassified;
d__Bacteria;p__Proteobacteria;c__Gammaproteobacteria;o__Burkholderiales;f__Nitrosomonadaceae;g__IS-44;s__IS-44_unclassified
d__Bacteria;p__Verrucomicrobiota;c__Verrucomicrobiae;o__S-BQ2-57_soil_group;f__S-BQ2-57_soil_group_unclassified;g__S-BQ2-57
d__Bacteria;p__Chloroflexi;c__KD4-96;o__KD4-96_unclassified;f__KD4-96_unclassified;g__KD4-96_unclassified;s__KD4-96_unclass
d__Bacteria;p__Actinobacteriota;c__Acidimicrobiia;o__Actinomarinales;f__Actinomarinaceae;g__Candidatus_Actinomarina;s__Cand
d__Bacteria;p__MBNT15;c__MBNT15_unclassified;o__MBNT15_unclassified;f__MBNT15_unclassified;g__MBNT15_unclassified;s__MBNT15
d__Bacteria;p__Firmicutes;c__Clostridia;o__Oscillospirales;f__Oscillospiraceae;g__Intestinimonas;s__Intestinimonas_unclassi
d__Bacteria;p__Firmicutes;c__Clostridia;o__Clostridiales;f__Ruminococcaceae;g__Gorbachella;s__Gorbachella_massiliensis
d__Bacteria;p__Firmicutes;c__Clostridia;o__Oscillospirales;f__Eubacterium]_coprostanoligenes_group;g__Eubacterium]_coprosta
d__Bacteria;p__Fusobacteriota;c__Fusobacteriia;o__Fusobacteriales;f__Leptotrichiaceae;g__Sneathia;s__Sneathia_unclassified
d__Bacteria;p__Acidobacteriota;c__Acidobacteriia;o__Acidobacteriotales;f__Acidobacteriotales_unclassified;g__Acidobacteriot
d__Bacteria;p__Bacteroidota;c__Bacteroidia;o__Bacteroidales;f__Tannerellaceae;g__Parabacteroides;s__Parabacteroides_sp._J15
d__Bacteria;p__Bacteroidota;c__Bacteroidia;o__Bacteroidales;f__Bacteroidaceae;g__Bacteroides;s__Bacteroides_unclassified
d__Bacteria;p__Proteobacteria;c__Deltaproteobacteria;o__Desulfuromonadales;f__Geobacteraceae;g__Geobacteraceae_unclassified
d__Bacteria;p__Acidobacteriota;c__Acidobacteriae;o__Subgroup_2;f__Subgroup_2_unclassified;g__Subgroup_2_unclassified;s__Sub
d__Bacteria;p__Firmicutes;c__Clostridia;o__Clostridia_UCG-014;f__Clostridia_UCG-014_unclassified;g__Clostridia_UCG-014_uncl
d__Bacteria;p__Cyanobacteria;c__Vampirivibrionia;o__Gastranaerophilales;f__Gastranaerophilales_unclassified;g__Gastranaerop
d__Bacteria;p__Bacteroidota;c__Bacteroidia;o__Chitinophagales;f__Chitinophagaceae;g__Edaphobaculum;s__Edaphobaculum_unclass
d__Bacteria;p__Gemmatimonadota;c__Gemmatimonadetes;o__Gemmatimonadales;f__Gemmatimonadaceae;g__Gemmatimonadaceae_unclassifi
d__Bacteria;p__Cyanobacteria;c__Cyanobacteriia;o__Chloroplast;f__Chloroplast_unclassified;g__Chloroplast_unclassified;s__Ch
d__Bacteria;p__Bacteroidota;c__Bacteroidia;o__Bacteroidales;f__Tannerellaceae;g__Parabacteroides;s__Parabacteroides_distaso
d__Bacteria;p__Gemmatimonadota;c__Gemmatimonadetes;o__Gemmatimonadales;f__Gemmatimonadaceae;g__Gemmatimonadaceae_unclassifi
d__Bacteria;p__Firmicutes;c__Clostridia;o__Clostridiales;f__Lachnospiraceae;g__Lachnospiraceae_unclassified;s__Lachnospirac

lwoffii
ssifiec
a
phomonas_maltophilia

iec
nclassifiec

_Sphingopyxis_sp.
iec
iec

c
.

iec
__Methylobacterium-Methylorubrum_unclassifiec
scherichia-Shigella_unclassifiec

loroplast_unclassifiec

llum_unclassifiec
ter_sp._B5
_sp.

iec
llum_unclassifiec

iec

us;s__Peptostreptococcus_unclassifiec

8
sp.
ter_sp.
__Clostridioides_unclassified
_sp.

aceae_unclassified
llum_unclassified

loroplast_unclassified
as_sp._NA61
ied

ied
nophilus_unclassified

ied
ssified
as_sp._mixed_culture_JK-Clone-05
raburkholderia;s__Burkholderia-Caballeronia-Paraburkholderia_unclassified
ssified
ied
_unclassified

nclassified

sp._H1
hondria_unclassified
m_elkanii
1-85-GX

raburkholderia;s__Burkholderia-Caballeronia-Paraburkholderia_unclassified

haseoli

cterium_unclassifiec

e_RC9_gut_group_unclassifiec

_unclassifiec

lassifiec
p._QD03

cterium_unclassifiec
cterium_unclassifiec
.
ibacteraceae_unclassifiec
obacterium_unclassifiec
aceae_unclassifiec
ctobacterium_succinatutens
cterium_unclassifiec

2_12ER2A
_unclassifiec
sp._1B3
iec
raburkholderia;s__Burkholderia-Caballeronia-Paraburkholderia_unclassifiec
ifiec
cterium_unclassifiec

ssifiec
bacterium_tuberculostearicum

ssifiec
p._RZ1
d;g__Alphaproteobacteria_unclassified;s__Alphaproteobacteria_unclassifiec

anthomonas_unclassified
cterium_unclassified

m_unclassified
m_unclassified

e_RA-555
.
omiireducens

stridiales_unclassified
nclassified
__Methylobacterium-Methylorubrum_unclassified
_1PNM-17

ed_Rubellimicrobium_sp.
fied;s__Sphingomonadaceae_unclassified
nsu_stricto_1_unclassified

eudomonas_sp.
ied
assified
raburkholderia;s__Burkholderia-Caballeronia-Paraburkholderia_unclassified
aerogenes
as_sp.
ter_sp._huazhou07
phomonas_maltophilia
_unclassified

_sp.
lassified

__Methylobacterium-Methylorubrum_unclassified

CC9902_unclassified
lassified
ssified
lassified
_unclassified

ristensenellaceae_R-7_group_unclassified
_Sphingomonas_sp.
nclassified

nclassified

bium-Rhizobium;s__Allorhizobium-Neorhizobium-Pararhizobium-Rhizobium_unclassified
nclassified
terrigena

ied
ssified
cter_sp._A2-59
as_staleyi
_unclassified
urida
iruptor;s__Caldicellulosiruptor_unclassified
ied
tinobacteriota_unclassified;s__Actinobacteriota_unclassified

_unclassified
_1PNM-17
fied
_unclassified
s
-1_unclassified

aceae_unclassified

sp.

obacterium_unclassified
raburkholderia;s__Burkholderia-Caballeronia-Paraburkholderia_unclassified
lassified
eudomonas_sp.

d
_unclassified
ssified

sified

loroplast_unclassified

raburkholderia;s__Burkholderia-Caballeronia-Paraburkholderia_unclassified
classified

_unclassified
p._AY-2011-RS11
haseoli
ristensenellaceae_R-7_group_unclassified

reponema_cluster_unclassified

;s__Acidibacter_unclassified

ed
sp._DSP11
sified

classified;s__Diplorickettsiaceae_unclassified
ssified
ssified
nclassified

loroplast_unclassifiec
c
ssifiec
as_sp.
is
ssifiec
fiec
as_sp._NR13
iec

netobacter_sp.
er_unclassifiec
unclassifiec
assifiec

oryzae

iec
ssifiec
raburkholderia;s__Burkholderia-Caballeronia-Paraburkholderia_unclassifiec
raburkholderia;s__Burkholderia-Caballeronia-Paraburkholderia_unclassifiec

eae_unclassifiec
sp.
iec
.

unclassifiec
loroplast_unclassifiec
ssifiec

as_sp.
inaceae_unclassifiec
classifiec

_sp._UASWS1009
netobacter_sp.
mycetospora_unclassified

_unclassified
hondria_unclassified

cterium_unclassified

as_intermedia
_unclassified
_unclassified

ornithinolytica

phomonas_sp.
s_Aquiluna_unclassified
ingobium_sp._15R35
netobacter_sp.
sp._R088
eae_unclassified

lassified
p.
eae_unclassified
as_sp.
_unclassified
hondria_unclassified
nclassified

cterium_unclassified

calcoaceticus
ified
as_melonis
_Christensenellaceae_unclassified

ifiec
raburkholderia;s__Burkholderia-Caballeronia-Paraburkholderia_unclassifiec
eudomonas_sp.
ssifiec

baylyi
m_unclassifiec
iec
na_unclassifiec
_unclassifiec

_unclassifiec
_gnavus_group_unclassifiec

m_sp._URHE0077
iec

_CCAP_1459-11B_unclassifiec
as_sp._NA61
lassifiec
ermomonas_sp.

iec

as_staleyi

]_torques_group_unclassifiec

lassified;s__Desulfovibrionaceae_unclassifiec
cterium_unclassifiec
icrobium_roseum

._LZB055
netobacter_sp.
Omnitrophus_unclassifiec
iec

scherichia-Shigella_unclassified
lassified
ified
totrophicus
m
ified
as_sp._M_Sw_oHS_10/11_6_2(2)
.

us_unclassified
m_unclassified
oli
._W1.09-142
_unclassified
s_sp._34zhy
loroplast_unclassified

ssified
raurococcus-Caldovatus_unclassified
_sp._C22(2011)
ornithinolytica
ssified
ssified

d
netobacter_sp.
Klebsiella_sp.

phomicrobium_sp.
ied

ied

ified
phomonas_maltophilia
p.

g__Proteobacteria_unclassified;s__Proteobacteria_unclassified
llum_unclassified
phomonas_sp._D2

nclassified
ied
nclassified
as_sp.
.
p._TS1138
loroplast_unclassified
loroplast_unclassified

5_unclassified
5_unclassified

phomicrobium_sp.

unclassified
as_sp._NA61
unclassified
lassified
as_sp._MDB2-16
ied
ified
andidatus_Nomurabacteria_unclassified;s__Candidatus_Nomurabacteria_unclassified
ssified
sified
bium-Rhizobium;s__Allorhizobium-Neorhizobium-Pararhizobium-Rhizobium_unclassified
ropolyspora_unclassified
ied
as_sp._mixed_culture_JK-Clone-05
raburkholderia;s__Burkholderia-Caballeronia-Paraburkholderia_unclassified
cterium_unclassified

5
cterium_unclassified
as_sp.
ldum
rysipelotrichaceae_UCG-003_unclassified
sp._MG-2011-22-CW
ulobacter_sp.
eudomonas_sp.
netobacter_sp.

nsu_stricto_1_unclassified
as_rubra

unclassified

assified;s__Clostridia_UCG-014_unclassified
ssified
fied

raburkholderia;s__Burkholderia-Caballeronia-Paraburkholderia_unclassified
pneumoniae
as_panni

.

sp._4_JDE-2009
ified
ter_cancerogenus

ssified
eudomonas_sp.
ified

nclassifiec
_Sphingomonas_sp.
ssifiec
AU4049_unclassifiec
sp.
iec

loroplast_unclassifiec
us_unclassifiec
ter_sp._NJ-1
nsu_stricto_1_unclassifiec
ensu_stricto_13_unclassifiec

lassifiec
r_unclassifiec
c
_clade_unclassifiec
_clade_unclassifiec
iec

nclassifiec
raburkholderia;s__Burkholderia-Caballeronia-Paraburkholderia_unclassifiec

iec
ifiec

lassifiec
lassifiec

phomonas_sp._1-2004
y_XIV._Incertae_Sedis_unclassified;s__Clostridiales_Family_XIV._Incertae_Sedis_unclassifiec
as_sp._M72C20

acter_unclassified
lassified
as_sp.
fied
aiellales_unclassified
sp._XH7

ified
nsu_stricto_1_unclassified
sp._AKB-2008-HE55

us_unclassified
nclassified
phomonas_maltophilia
unclassified

_unclassified
d

onteilii
ga_unclassified

ssified
_unclassified
_unclassified
ified

fied
ristensenellaceae_R-7_group_unclassified
ssified
ed;s__Gemmatimonadaceae_unclassified
d
__Methylobacterium-Methylorubrum_unclassified
unclassified

nclassified

enterica

]_torques_group_unclassified
.
noligenes_group_unclassified;s__Eubacterium]_coprostanoligenes_group_unclassified

nsu_stricto_1_unclassified
ibacteraceae_unclassified
phomonas_unclassified

p._SG4502

ter_cloacae
nclassified
ied
nclassified
ssified
la_unclassified
as_sp.
nsu_stricto_1_unclassified

ranulicella_unclassified
raburkholderia;s__Burkholderia-Caballeronia-Paraburkholderia_unclassified

loroplast_unclassified
netobacter_sp.
obacterium_unclassified
_unclassified
netobacter_sp.

classified;s__Steroidobacteraceae_unclassified
loroplast_unclassified
fied;s__Chroococcidiopsaceae_unclassified

ssifiec
ctobacterium_unclassifiec

_unclassifiec
nsu_stricto_1_unclassifiec

loroplast_unclassifiec
imonas_unclassifiec
e_RC9_gut_group_unclassifiec
assifiec
trophicus;s__Streptomyces]_thermoautotrophicus_unclassifiec

raburkholderia;s__Burkholderia-Caballeronia-Paraburkholderia_unclassifiec
is_sp._25alexandra2
ssifiec
sp._1116(2011)
esinovorans
ec
fulvimonas_unclassifiec
odobacter_sp.
loroplast_unclassifiec
nclassifiec
as_sp.
lecoglossicida
loroplast_unclassifiec

_unclassifiec
nclassifiec

lassifiec
d;g__Alphaproteobacteria_unclassified;s__Alphaproteobacteria_unclassifiec
cterium_unclassifiec
__Methylobacterium-Methylorubrum_unclassifiec
nclassifiec
nclassifiec

iec
eruginosa
ssifiec

eae_unclassifiec

nclassified;s__Burkholderiales_unclassifiec
lassifiec

loroplast_unclassifiec
loroplast_unclassifiec
ssifiec
nsu_stricto_8_unclassifiec

hobacter_sp.
loroplast_unclassifiec
ssifiec
ifiec
ssifiec
ifiec
c
as_sp._KIN163
guillouiae
ec
r_unclassifiec
as_unclassifiec
nclassifiec
_unclassifiec
sp.

loroplast_unclassifiec
d_Phascolarctobacterium_sp.

ssifiec
lassifiec
nclassifiec
ingobium_unclassifiec
eae_unclassifiec

_unclassifiec
cter_unclassifiec
us;s__Peptostreptococcus_unclassifiec

hondria_unclassifiec
loroplast_unclassifiec
nclassifiec
elatoclostridium_unclassifiec
nis
eae_unclassifiec
aceae_NK4A136_group_unclassifiec

phomonas_sp.
idatus_Actinomarina_unclassifiec
.
.
iec

maculum_unclassifiec
a_sp._CPD32
iec
up_unclassifiec

aiellales_unclassifiec
c

aceae_NK4A136_group_unclassifiec
__Candidatus_Puniceispirillum_unclassifiec
loroplast_unclassifiec
;s__Microbacteriaceae_unclassifiec
sp._AH3

raburkholderia;s__Burkholderia-Caballeronia-Paraburkholderia_unclassified
ied

unclassified
eae_unclassified
classified
Brevundimonas_sp.

ied
ied
ed;s__Acetobacteraceae_unclassified
loroplast_unclassified
loroplast_unclassified
.
a
is_italica
_unclassified
loroplast_unclassified
johnsonii
ssified
.
as_sp._LBS8
elatoclostridium_unclassified
loroplast_unclassified

ified
raburkholderia;s__Burkholderia-Caballeronia-Paraburkholderia_unclassified

]_torques_group_unclassified
guillouiae
allii_group_unclassified
phomonas_maltophilia
ssified

07_unclassified
_unclassified
scherichia-Shigella_unclassified

sp._FR-W3CA
sii
sipelatoclostridium_sp._SNUG30386
ngbya_Es-Yyy1000_unclassified

r;s__Candidatus_Contendobacter_unclassified
ovibrio_unclassified
as_turrisvirgatae
ied
cterium_unclassified

omyces_unclassified

io_unclassified

icrobium_unclassified

ified

eae_unclassified

p._LZB056
ied

2
osinicella_unclassified
bium-Rhizobium;s__Allorhizobium-Neorhizobium-Pararhizobium-Rhizobium_unclassified
classified

daphobacter_unclassified
lassified
raburkholderia;s__Burkholderia-Caballeronia-Paraburkholderia_unclassified
bacteriaceae_UCG-002_unclassified
unclassified
classified
urida

oup;s__Family_XIII_AD3011_group_unclassifiec
m_unclassifiec
ssifiec
anthomonas_sp.
as_melonis
eae_unclassifiec
a
bium-Rhizobium;s__Allorhizobium-Neorhizobium-Pararhizobium-Rhizobium_unclassifiec

hingopyxis_unclassifiec
ssifiec
_unclassifiec

_sp._AKB-2008-RN9

netobacter_sp.
a
eae_unclassifiec
eae_unclassifiec
monas_sp.
lassifiec
eisseriaceae_unclassifiec
loroplast_unclassifiec
as_sp._JB13

sp._AH3

iec
tus_Koribacter_unclassifiec
lassifiec
c

phomonas_maltophilia
iec
cterium_unclassifiec

phomonas_maltophilia
nclassified

_unclassified

nsu_stricto_1_unclassified
assified;s__Clostridia_UCG-014_unclassified
ied
loroplast_unclassified

raburkholderia;s__Burkholderia-Caballeronia-Paraburkholderia_unclassified

d
as_sp._NA61
CC9902_unclassified
ssified

_unclassified
_Sphingomonas_sp.
is_italica
as_sp.
sp.
phomonas_maltophilia
d
eae_unclassified
classified;s__Firmicutes_unclassified
sp._NJ-76
netobacter_sp.

obacterium_unclassified
.

loroplast_unclassified
.

nospora_chersina
eae_unclassified

c

d;g__Alphaproteobacteria_unclassified;s__Alphaproteobacteria_unclassified
ssified

__Methylobacterium-Methylorubrum_unclassified
phomonas_sp._SAP52_1
eae_unclassified
phomonas_maltophilia
phomonas_sp._GR_2-01
oae
_unclassified
a_unclassified
monas_unclassified
unclassified
sp.
r_unclassified
ter_sp._Ma6

er_unclassified

cterium_unclassified

ingobium_unclassified
cterium_unclassified
ified
lwoffii
aceae_NK4A136_group_unclassified
_DMF

loroplast_unclassified

ria_unclassified
__Methylobacterium-Methylorubrum_unclassified
sp._SG-3
s__Intestinibacter_unclassified

loroplast_unclassified
eae_unclassified
ssified
loroplast_unclassified

ified

nclassified
as_sp.

maculum_unclassified
d

adyrhizobium_sp.
__Methylobacterium-Methylorubrum_unclassified
ra_unclassified
phomonas_sp._I_51-G7P1A3B1
d
_B0670
eae_unclassified
nsu_stricto_1_unclassified
raburkholderia;s__Burkholderia-Caballeronia-Paraburkholderia_unclassified

ardia_unclassified
s_unclassified;s__Erysipelotrichales_unclassified
cterium_unclassified
__Betaproteobacteria_unclassified;s__Betaproteobacteria_unclassified
p._HUBA6

loroplast_unclassified

sp._BSJ2
as_sp._BACT-25

cterium_unclassified
._IMCC1720
is_italica

ulus_unclassified
classified;f__SAR324_clade(Marine_group_B)_unclassified;g__SAR324_clade(Marine_group_B)_unclassified;s__SAR324_clade(Marine

ssified
_unclassified
_Sphingopyxis_sp.

abdus_unclassified
.
p.

_sp._NW-2013-Rh13
lassified
lassified
_yanoikuyae

loroplast_unclassified

er_unclassified
as_sp.

loroplast_unclassified
netobacter_sp.
is_sp._25alexandra2
nclassified
_sp.
d

ed

ssifiec
nclassifiec

phomonas_maltophilia
phomonas_sp._S1
phomonas_maltophilia
classified;s__Firmicutes_unclassifiec
bacter_sp.
helis
ifiec
B14
assifiec
nclassifiec

ssifiec
m_unclassifiec
m_unclassifiec
NRB5220
lassifiec

unclassifiec

assifiec
ssifiec

ssifiec
as_unclassifiec
lassifiec
fiec
elatoclostridium_unclassifiec
loroplast_unclassifiec

as_sp.
s
iec
ssifiec

c
_unclassified;s__Paracaedibacteraceae_unclassified
fied
;s__Acidibacter_unclassified
d;g__Alphaproteobacteria_unclassified;s__Alphaproteobacteria_unclassified

lassified
mboutsia_unclassified
__Methylobacterium-Methylorubrum_unclassified
ssified
classified;s__Firmicutes_unclassified
ified
ylocystis_sp.

pneumoniae

mboutsia_unclassified
raburkholderia;s__Burkholderia-Caballeronia-Paraburkholderia_unclassified
loroplast_unclassified
raburkholderia;s__Burkholderia-Caballeronia-Paraburkholderia_unclassified

as_melonis

eae_unclassified

haceae_unclassified

eudomonas_sp.

ied
piraceae_unclassified
sp.
fied
planes_sp.

assified;s__Clostridia_UCG-014_unclassified
.
classified;s__Firmicutes_unclassified
nclassified
eudomonas_sp.

.
classified
d
phomonas_sp.

eae_unclassified
ssified

loroplast_unclassified
as_koreensis
wensis

sp._CBMAI_1180
as_sp.
is_unclassified
__Betaproteobacteria_unclassified;s__Betaproteobacteria_unclassified
322-1
as_panni
nclassified
ristensenellaceae_R-7_group_unclassified
0
acter_hermannii

__Betaproteobacteria_unclassified;s__Betaproteobacteria_unclassified

is_sp._SQ89

m_unclassified
__Methylobacterium-Methylorubrum_unclassified

nclassified
phomonas_maltophilia
nclassified
eae_unclassified
ii
eae_unclassified
assified
hondria_unclassified
ified
lassified
raburkholderia;s__Burkholderia-Caballeronia-Paraburkholderia_unclassified
sp._Cai-20

phomonas_sp._7-3

sp.

acter_unclassified

3120
Obesumbacterium_unclassified
nclassified

nclassified
is_witflariensis
assified;s__Vicinamibacteraceae_unclassified
_JNU-L078
classified
radioresistens

nis
ter_cloacae
ter_sp.
eudomonas_sp.

nsu_stricto_1_unclassified
ied
cterium_unclassified
aceae_NK4A136_group_unclassified
.
icrobium_unclassified
es_unclassified;s__Saccharimonadales_unclassified
sp.
loroplast_unclassified

assified;s__Clostridia_UCG-014_unclassified
.
aceae_unclassified
ingobium_unclassified
bacter_unclassified

enrichment_culture_clone_NCP4-3
a
sp.
ied
.
p.
phomonas_sp._68B
.
ssified
lassified
eae_unclassified

ied
piraceae_unclassified

ornithinolytica
odobacter_sp.
us;s__Peptostreptococcus_unclassified
.
eae_unclassified

ii

_unclassifiec
assifiec

ingobium_stygium
.
sp.
as_zeae

iec
as_sp.
nclassifiec

p._QD03
lassifiec
ldum
_Citrobacter_sp.

eae_unclassifiec

classified;s__Thermovenabulales_unclassifiec
ifiec
noligenes_group_unclassified;s__Eubacterium]_coprostanoligenes_group_unclassifiec

lassifiec
_clade_unclassifiec

iec

iec
rium_unclassifiec
raburkholderia;s__Burkholderia-Caballeronia-Paraburkholderia_unclassifiec

elatoclostridium_unclassifiec

d_Rhodanobacter_sp.

ifiec

lassifiec

41

aceae_unclassifiec
phomonas_maltophilia

ec
eae_unclassifiec
classified;s__Firmicutes_unclassifiec

um_unclassifiec
ifiec
d;g__Gammaproteobacteria_unclassified;s__Gammaproteobacteria_unclassifiec
assifiec

;s__Microbacteriaceae_unclassifiec
docitrobacter_unclassifiec

fiec
is_witflariensis

phomonas_maltophilia
sp.
eae_unclassifiec
eae_unclassifiec
ovibrio_unclassifiec
pneumoniae

bacteriaceae_UCG-002_unclassifiec

lassified;s__Geodermatophilaceae_unclassified
ified
loroplast_unclassified
assified;s__Clostridia_UCG-014_unclassified
loroplast_unclassified
loroplast_unclassified
.
nclassified
a
CC9902_unclassified
_unclassified

eae_unclassified
eae_unclassified
nclassified
__Betaproteobacteria_unclassified;s__Betaproteobacteria_unclassified
ssified
_unclassified
assified;s__Clostridia_UCG-014_unclassified
exaceae_unclassified
fied

_unclassified
ialis
m_facile
oli

unclassified
yanobacteria_unclassified;s__Cyanobacteria_unclassified
biont_of_Noctuid_moth_species
stridiales_unclassified
junii
CC9902_unclassified
orax_sp.
enica

eae_unclassifiec
as_sp.
assifiec

nclassifiec
netobacter_sp.
loroplast_unclassifiec
__Methylobacterium-Methylorubrum_unclassifiec

classifiec
iec
unclassifiec
_unclassifiec
as_unclassifiec
neaceae_unclassifiec
lassifiec
tinobacteriota_unclassified;s__Actinobacteriota_unclassifiec
sifiec

d;g__Alphaproteobacteria_unclassified;s__Alphaproteobacteria_unclassifiec

classifiec

ra_unclassifiec
iec
lassifiec
us_unclassifiec
scherichia-Shigella_unclassifiec
ra_unclassifiec
ec
__Methylobacterium-Methylorubrum_unclassifiec
eae_unclassifiec

ssifiec

sifiec
_unclassifiec
d;s__Acidimicrobiaceae_unclassifiec
p._MG-2011-7-BJ
sifiec

c
ssifiec

acter_unclassifiec

lassifiec
phomonas_sp._D2
iec
__Betaproteobacteria_unclassified;s__Betaproteobacteria_unclassifiec
ec

phomonas_maltophilia
ter_sp._NJ-64
ter_asburiae
cus]_gauvreauii_group_unclassifiec
assified;s__Clostridia_UCG-014_unclassifiec
s__Acetoanaerobium_unclassifiec
eudomonas_sp.
__Methylobacterium-Methylorubrum_unclassifiec
fiec
ssifiec
anus
stridiales_unclassifiec
maculum_unclassifiec
eae_unclassifiec

ne_group_unclassifiec
ifiec

m_unclassified
mi

schericha-Shigella_unclassified
ified

ssified
07_unclassified
classified
ter_sp.
phomonas_sp._RKHZ-44

nclassified
ied
.
um_unclassified
llum_unclassified
stridiales_unclassified
_unclassified
phomonas_unclassified
ed;s__Terasakiellaceae_unclassified
ified
loroplast_unclassified
__Betaproteobacteria_unclassified;s__Betaproteobacteria_unclassified
loroplast_unclassified
classified;s__Firmicutes_unclassified
lla_sp.
_unclassified
cterium_unclassified

eae_unclassified
d
.

yanobacteria_unclassified;s__Cyanobacteria_unclassified

nclassified

ified
d
__Methylobacterium-Methylorubrum_unclassified
loroplast_unclassified
ovibrio_unclassified

is_witflariensis
lassified;s__Desulfovibrionaceae_unclassified
la_unclassified
Klebsiella_sp.

fied

stridiales_unclassified
aceae_unclassified
_Sphingopyxis_sp.
phomonas_maltophilia
Comamonadaceae_unclassified

d;g__Alphaproteobacteria_unclassified;s__Alphaproteobacteria_unclassified
assified
robacter_unclassified
assified
eae_unclassified
ae_unclassified
5_unclassified
unclassified
eae_unclassified

__Methylobacterium-Methylorubrum_unclassified
age_IV_unclassified
ed;s__Gemmatimonadaceae_unclassified
guillouiae

nclassifiec

stridiales_unclassifiec
ec

ristensenellaceae_R-7_group_unclassifiec
fiec

icrobium_sp.
eae_unclassifiec
cter_terrae
as_sp.

raburkholderia;s__Burkholderia-Caballeronia-Paraburkholderia_unclassifiec

_sp.

ristensenellaceae_R-7_group_unclassifiec
netobacter_sp.

nclassifiec
monas_unclassifiec
eae_unclassifiec

as_sp.
__Betaproteobacteria_unclassified;s__Betaproteobacteria_unclassifiec
netobacter_sp.

ifiec
phomonas_maltophilia
lassifiec
_unclassifiec

r_werkmanii
assifiec
de_unclassifiec
assifiec
iec
ssifiec
-54-9_unclassifiec

loroplast_unclassifiec
cterium_unclassifiec

m_unclassifiec
ssifiec
classifiec
ifiec

nclassifiec

eae_unclassifiec

pneumoniae
noligenes_group_unclassified;s__Eubacterium]_coprostanoligenes_group_unclassifiec
lassified;s__Desulfovibrionaceae_unclassifiec
is_witflariensis

sifiec

cusii
e_ASCC01

__Clostridioides_unclassifiec

ra_unclassifiec

loroplast_unclassifiec
hondria_unclassifiec
lassifiec
unclassifiec
classified;s__Thermovenabulales_unclassifiec

ec

lassifiec
SAR116_clade_unclassifiec
ifiec
mboutsia_unclassifiec
__Betaproteobacteria_unclassified;s__Betaproteobacteria_unclassifiec

_unclassifiec
lassifiec
classified;s__Steroidobacteraceae_unclassifiec
loroplast_unclassifiec

unclassifiec
classified;s__Firmicutes_unclassifiec
ohlfahrtiimonas_unclassifiec
ifiec
ter_sp.
us_unclassifiec
_Acidithiobacillus_unclassifiec
ga_unclassifiec
rysipelotrichaceae_UCG-003_unclassifiec
_Sphingopyxis_sp.
is_sp._SQ8[illegible]
unclassifiec
eae_unclassifiec

as_sp.

roidota_unclassified;s__Bacteroidota_unclassified
Propionibacterium_westphaliense
hongiella_unclassified
r_dublinensis
cterium_unclassified
assified;s__Clostridia_UCG-014_unclassified
haceae_unclassified
as_sp.
idatus_Actinomarina_unclassified
ified

noligenes_group_unclassified;s__Eubacterium]_coprostanoligenes_group_unclassified
d
ssified

oli
d

ified
ied
nis

tutzeri

ssified
m_unclassified
is_sp._25alexandra2
d;g__Deltaproteobacteria_unclassified;s__Deltaproteobacteria_unclassified
lassified
lassified

ctobacterium_succinatutens
unclassified
ified
lassified;s__Desulfovibrionaceae_unclassified

m_sp._enrichment_culture_clone_MWE_N32
noligenes_group_unclassified;s__Eubacterium]_coprostanoligenes_group_unclassifiec
bereziniae
cterium_unclassifiec
c

aceae_NK4A136_group_unclassifiec
ec
is_unclassifiec
.
_yanoikuyae
lla_sp.
ssifiec

marine_group_unclassifiec

3
assified;s__Clostridia_UCG-014_unclassifiec
ssifiec
lassifiec
de_unclassifiec
fied;s__Micromonosporaceae_unclassifiec

loroplast_unclassifiec
eobacter_unclassifiec

eae_unclassifiec

ifiec
_unclassifiec
lassifiec
_unclassifiec
nclassified;s__Burkholderiales_unclassifiec
cterium_unclassifiec
hondria_unclassifiec

ingobium_endophyticum

_Sphingomonas_sp.
classified;s__Thermovenabulales_unclassified
_unclassified
_unclassified
s
d
ified
5_unclassified
d_Enterobacter_sp.

d
nclassified
unclassified
.
a_unclassified
a
nclassified

nclassified

unclassified
.

ied
.

classified;s__Firmicutes_unclassified
a
r_unclassified
nclassified
la_unclassified

assified;s__Clostridia_UCG-014_unclassified

phomonas_acidaminiphila

eae_unclassified
ssified

raceae_unclassified;s__Ectothiorhodospiraceae_unclassified
lassified
ified

__Betaproteobacteria_unclassified;s__Betaproteobacteria_unclassified
eudomonas_sp.
_unclassified
ied

assified;s__Clostridia_UCG-014_unclassified

d
diales_unclassified
.
ied
cterium_unclassified
ified

um_unclassified
omyces_unclassified
aceihabitans_unclassified
assified;s__Clostridia_UCG-014_unclassified

d;g__Alphaproteobacteria_unclassified;s__Alphaproteobacteria_unclassified
lassified;s__Desulfovibrionaceae_unclassified
d
__Subgroup_17_unclassified
ed;s__Gemmatimonadaceae_unclassified
_unclassified
ssified
lassified
cterium_unclassified

as_rhizophila
a
la_unclassified
lassified
sp._10_SBR2
ed
stridiales_unclassified
bacter_unclassified
stridiales_unclassified
.
eae_unclassified
stridiales_unclassified
nclassified
__Methylobacterium-Methylorubrum_unclassified

haceae_unclassified
classified
ropolyspora_unclassified
fied

nclassified
classified;s__Firmicutes_unclassified
loroplast_unclassified
eae_unclassified
_clade_unclassified
_clade_unclassified
74
ssified
eae_unclassified
cterium_unclassified
ified
d;g__Gammaproteobacteria_unclassified;s__Gammaproteobacteria_unclassified
d_Rhodanobacter_sp.
ristensenellaceae_R-7_group_unclassified

bacter_unclassified
ured_Corynebacterium_sp.

eligens_group_unclassified
_unclassified
ied;g__Acidobacteriota_unclassified;s__Acidobacteriota_unclassified
m_unclassified
hongiella_unclassified
lus_unclassified
tus
is_unclassified
as_sp.
hongiella_unclassified
stridiales_unclassified
aceae_NK4A136_group_unclassified
ed
__Methylobacterium-Methylorubrum_unclassified

la_unclassified

nclassified

_unclassified
eae_unclassified
piraceae_unclassified

_unclassified

luorescens
sified
p._Air81
]_torques_group_unclassified
as_panni

nclassified

itroreducens
d

ifiec
idatus_Actinomarina_unclassifiec
roidota_unclassified;s__Bacteroidota_unclassifiec
d;g__Alphaproteobacteria_unclassified;s__Alphaproteobacteria_unclassifiec
loroplast_unclassifiec
_unclassifiec
iec
m_unclassifiec

la_unclassifiec
iec
-1_unclassifiec
unclassified;s__Rokubacteriales_unclassifiec

_1PNM-17
iec
eae_unclassifiec

UBL_19
.
classified;s__Tepidisphaerales_unclassifiec
phomicrobium_sp.

._AAI-8
assified;s__Clostridia_UCG-014_unclassifiec
ssifiec
hobacter_sp.

is_witflariensis
phomonas_maltophilia
iec
aceae_NK4A136_group_unclassifiec
iec

is_witflariensis
_unclassified
a_unclassified
nclassified

unclassified

ified
nclassified
elmanticensis
p._RKEM_1577

eae_unclassified
ssified
unclassified
e_RC9_gut_group_unclassified
assified
s__Fimbriimonadaceae_unclassified
phomicrobium_sp.
lassified;s__Desulfovibrionaceae_unclassified

ne_group_unclassified
noligenes_group_unclassified;s__Eubacterium]_coprostanoligenes_group_unclassified
icrobium_unclassified
oniobacter_unclassified
_unclassified
sporaceae_unclassified
loroplast_unclassified
noligenes_group_unclassified;s__Eubacterium]_coprostanoligenes_group_unclassified
assified;s__Clostridia_UCG-014_unclassified

ified

fiec

ysipelotrichaceae_unclassifiec
assified;s__Clostridia_UCG-014_unclassifiec

raurococcus-Caldovatus_unclassifiec
s__Thermanaeromonas_unclassifiec
iec
lla_sp.

loroplast_unclassifiec
hongiella_unclassifiec

ae_unclassifiec

r_freundii
d;g__Alphaproteobacteria_unclassified;s__Alphaproteobacteria_unclassifiec
__Candidatus_Puniceispirillum_unclassifiec

nclassified;s__Acidobacteriales_unclassifiec
eae_unclassifiec
assified;s__Clostridia_UCG-014_unclassifiec

d;s__Fusobacteriotaceae_unclassifiec
radioresistens

maculum_unclassifiec
m_unclassifiec
acter_unclassifiec

nclassifiec

scherichia-Shigella_unclassifiec
iec

ga_unclassifiec

_G200V

eae_unclassified
ae_Ga6A1_group_unclassified
_unclassified
tobacter_unclassified
septicus

.
la_unclassified
um]_ruminantium_group_unclassified

_sp.
sp._CJ-S-R2A8

ified
cter_unclassified
phomonas_sp._SAP52_1
rerythrobacter_unclassified
cterium_unclassified

ied
ctobacterium_unclassified
lassified
_Sphingomonas_sp.
ified

ra_unclassified
unclassified

lassified

classified;s__Firmicutes_unclassified

unclassified
ssified
ified
assified

ssified
ied
eae_unclassified
0353_marine_group_unclassified;s__UBA10353_marine_group_unclassified
sified
sychrotolerans
_unclassified
__Subgroup_17_unclassified
d;s__Rhodobacteraceae_unclassified
bacter_sp.
habdus_unclassified
_unclassified
fied
allii_group_unclassified
ified
assified
Bacteroidales_unclassified
d
ingeri

nis
_Clostridia_unclassified

as_sp._0312MAR21U9
aceae_unclassified
sp._X_159
ssified
eibacter_unclassified
as_sp._L7-453-1
nclassified;s__Acidobacteriales_unclassified

__Betaproteobacteria_unclassified;s__Betaproteobacteria_unclassifiec
lassifiec

andidatus_Nomurabacteria_unclassified;s__Candidatus_Nomurabacteria_unclassifiec

nclassifiec
de_unclassifiec
ifiec
roidota_unclassified;s__Bacteroidota_unclassifiec
tinobacteriota_unclassified;s__Actinobacteriota_unclassifiec
ssifiec
aceae_NK4A136_group_unclassifiec

_unclassifiec
fiec

UCG-010_unclassifiec
classified;s__Firmicutes_unclassifiec
ed_Stenotrophomonas_sp.
is_italica
fied;s__Micromonosporaceae_unclassifiec

nclassifiec
llum_unclassifiec
sp._CBMAI_118C
is_alaskensis
noligenes_group_unclassified;s__Eubacterium]_coprostanoligenes_group_unclassifiec
ssifiec
as_sp.
assified;s__Bradymonadales_unclassifiec
lassifiec
ta
eae_unclassifiec
io_unclassifiec

c

neaceae_unclassified

piraceae_unclassified

roup;s__Eubacterium]_brachy_group_unclassified
eae_unclassified
eae_unclassified

orax_sp.
nclassified
lassified;s__Desulfovibrionaceae_unclassified
is_alaskensis
as_sp.
ied

loroplast_unclassified
Loktanella_unclassified
us;s__Peptostreptococcus_unclassified
piraceae_unclassified
ysipelotrichaceae_unclassified
ra_unclassified
_unclassified

actobacillaceae_unclassified
g__Proteobacteria_unclassified;s__Proteobacteria_unclassified
sified
eae_unclassified

piscinae
_unclassified;f__Marinimicrobia_(SAR406_clade)_unclassified;g__Marinimicrobia_(SAR406_clade)_unclassified;s__Marinimicrobia
ed_Stenotrophomonas_sp.
tinobacteriota_unclassified;s__Actinobacteriota_unclassified
ssified

iec
idatus_Actinomarina_unclassifiec
ifiec
tinobacteriota_unclassified;s__Actinobacteriota_unclassifiec

odobacter_sp.
ius
noligenes_group_unclassified;s__Eubacterium]_coprostanoligenes_group_unclassifiec
eae_unclassifiec
fiec

icropepsaceae_unclassifiec
tinobacteriota_unclassified;s__Actinobacteriota_unclassifiec
ssifiec

as_anadarae
iec

ssifiec
er_unclassifiec
ns_unclassifiec

loroplast_unclassifiec
noligenes_group_unclassified;s__Eubacterium]_coprostanoligenes_group_unclassifiec
assified;s__Clostridia_UCG-014_unclassifiec

eae_unclassifiec
la_unclassifiec
eae_unclassifiec

lales_unclassified;s__Oceanospirillales_unclassifiec
cterium_unclassifiec
lassifiec

r
iec

eae_unclassified
_unclassified
assified
oup_unclassified;s__AEGEAN-169_marine_group_unclassified
_Sphingopyxis_sp.
ied;g__Acidobacteriota_unclassified;s__Acidobacteriota_unclassified

classified
noligenes_group_unclassified;s__Eubacterium]_coprostanoligenes_group_unclassified
tinobacteriota_unclassified;s__Actinobacteriota_unclassified

eae_unclassified
__Methylobacterium-Methylorubrum_unclassified
Alcaligenaceae_unclassified
rseille-P6563
tinobacteriota_unclassified;s__Actinobacteriota_unclassified
unclassified;s__Propionibacteriaceae_unclassified

eae_unclassified
fied
sp._1132(2011)
__Betaproteobacteria_unclassified;s__Betaproteobacteria_unclassified
cterium_unclassified

nclassified;s__Burkholderiales_unclassified
lassified
.
ssified
fied
ssified
unclassified
classified;s__Firmicutes_unclassified
CG-003_unclassified
les_unclassified;s__Sphingobacteriales_unclassified
ied

assifiec

_unclassifiec
eligens_group_unclassifiec
adyrhizobium_sp.

_Clostridia_unclassifiec
iec

r_unclassifiec
ssifiec

as_vesicularis
lassified;s__Desulfovibrionaceae_unclassifiec
phomonas_maltophilia

m_unclassifiec
m]_ventriosum_group_unclassifiec

eae_unclassifiec
us_unclassifiec

assified;s__Bradymonadales_unclassifiec

dia_vadinBB60_group_unclassified;s__Clostridia_vadinBB60_group_unclassifiec
assified;s__Clostridia_UCG-014_unclassifiec
eae_unclassifiec
ed;s__NS11-12_marine_group_unclassifiec
cicoccaceae_unclassifiec
p.
c
dia_vadinBB60_group_unclassified;s__Clostridia_vadinBB60_group_unclassifiec

eae_unclassifiec
sifiec

_unclassified
fied
_unclassified
noligenes_group_unclassified;s__Eubacterium]_coprostanoligenes_group_unclassified
ssified
netobacter_sp.

dia_vadinBB60_group_unclassified;s__Clostridia_vadinBB60_group_unclassified
bacter_unclassified
classified

ed;s__Gemmatimonadaceae_unclassified
loroplast_unclassified
a
sp._enrichment_culture_clone_AOCRB-EC-6
oup_unclassified;s__AEGEAN-169_marine_group_unclassified
assified
nclassified
loroplast_unclassified

loroplast_unclassified
phomonas_sp.
classified;s__Firmicutes_unclassified
ropolyspora_unclassified
assified;s__Clostridia_UCG-014_unclassified
lassified
eae_unclassified
bacter_unclassified
_unclassified

d;g__Gammaproteobacteria_unclassified;s__Gammaproteobacteria_unclassified
as_sp._NA61

lassified

d;g__Alphaproteobacteria_unclassified;s__Alphaproteobacteria_unclassified

johnsonii
eae_unclassifiec
loroplast_unclassifiec
c
eae_unclassifiec
re_clone
assifiec
bium-Rhizobium;s__Allorhizobium-Neorhizobium-Pararhizobium-Rhizobium_unclassifiec
lassifiec
eae_unclassifiec
ifiec
lla_sp.
ssified;g__Desulfobacterota_unclassified;s__Desulfobacterota_unclassifiec
egionellaceae_unclassifiec

ec
__Betaproteobacteria_unclassified;s__Betaproteobacteria_unclassifiec
ssifiec
um]_fissicatena_group_unclassifiec
fiec
rium_unclassifiec
eae_unclassifiec

stridiales_unclassifiec
assified;s__Vicinamibacteraceae_unclassifiec
classified;s__Firmicutes_unclassifiec

classifiec
UCG-004_unclassifiec
cterium_unclassifiec
c
daphobacter_unclassifiec
_unclassifiec
sifiec

_unclassifiec

ivibrio_sp.
elatoclostridium_unclassifiec
assified;s__Clostridia_UCG-014_unclassifiec

Loktanella_unclassifiec
iec
iec

loroplast_unclassifiec
bacter_sp.
m_unclassifiec

classifiec
bium_unclassifiec
ropolyspora_unclassifiec
ssifiec
aceae_NK4A136_group_unclassifiec
eae_unclassifiec

ifiec
eae_NK3B31_group_unclassifiec
eae_unclassifiec

nclassifiec
__Methylobacterium-Methylorubrum_unclassifiec
_unclassifiec
cus]_gauvreauii_group_unclassifiec
orax_sp.

eae_unclassifiec

lla_unclassifiec

ec
iec
iec
iec

cterium_unclassified
classified;s__Firmicutes_unclassified

classified;s__Firmicutes_unclassified
_Candidatus_Saccharimonas_unclassified
ium]_xylanophilum_group_unclassified
ied
assified

eae_unclassified
aceae_NK4A136_group_unclassified
lassified;s__JGI_0000069-P22_unclassified
phomonas_sp.
eae_unclassified
cterium_unclassified

loroplast_unclassified
unclassified;s__Micavibrionales_unclassified
assified;s__Clostridia_UCG-014_unclassified

Comamonadaceae_unclassified

eae_unclassified
UCG-003_unclassified
nsis

nclassified
phomonas_chelatiphaga

__Aeromonadaceae_unclassified

classified;s__Firmicutes_unclassifiec
assified;s__Clostridia_UCG-014_unclassifiec
silia_unclassifiec

noligenes_group_unclassified;s__Eubacterium]_coprostanoligenes_group_unclassifiec
phomonas_unclassifiec
iec
sifiec

nclassifiec
-E04_unclassifiec
loroplast_unclassifiec
roidota_unclassified;s__Bacteroidota_unclassifiec
eticus_unclassifiec
eae_unclassifiec
assified;g__Thermodesulfovibrionia_unclassified;s__Thermodesulfovibrionia_unclassifiec
aiellales_unclassifiec
ec
rillales_unclassified;s__Puniceispirillales_unclassifiec
sis
_clade_unclassifiec

cetobacter_unclassifiec
ingobium_unclassifiec
ardia_unclassifiec
assified;s__Clostridia_UCG-014_unclassifiec
SAR116_clade_unclassifiec
ec
oup_unclassified;s__AEGEAN-169_marine_group_unclassifiec
classifiec
6256_unclassifiec
ristensenellaceae_R-7_group_unclassifiec

_unclassifiec

orax_unclassifiec

va0485_unclassifiec
c
s__Thermanaeromonas_unclassifiec
__Betaproteobacteria_unclassified;s__Betaproteobacteria_unclassifiec

assifiec
assified;s__Clostridia_UCG-014_unclassifiec
ristensenellaceae_R-7_group_unclassifiec

nderia_unclassifiec
_unclassifiec
lassifiec
fiec
er_unclassifiec
UCG-010_unclassifiec

iec
group_2_unclassifiec
nia_unclassifiec
libacter_unclassifiec

__Methylobacterium-Methylorubrum_unclassifiec
c

_sp.

arine_group_unclassifiec
nclassified;s__RBG-16-55-12_unclassifiec
_unclassifiec
edis_unclassified;s__Rhizobiales_Incertae_Sedis_unclassifiec
Loktanella_unclassifiec
nclassifiec

lassifiec
assified;s__Clostridia_UCG-014_unclassifiec

c
classified;f__SAR324_clade(Marine_group_B)_unclassified;g__SAR324_clade(Marine_group_B)_unclassified;s__SAR324_clade(Marine
stridiales_unclassified

_unclassified
SAR116_clade_unclassified
_unclassified
assified
s__Hyphomonadaceae_unclassified

_soil_group_unclassified;s__S-BQ2-57_soil_group_unclassified
ified
idatus_Actinomarina_unclassified
_unclassified
fied

noligenes_group_unclassified;s__Eubacterium]_coprostanoligenes_group_unclassified

ales_unclassified;s__Acidobacteriotales_unclassified
02

;s__Geobacteraceae_unclassified
group_2_unclassified
assified;s__Clostridia_UCG-014_unclassified
hilales_unclassified;s__Gastranaerophilales_unclassified
ified
ed;s__Gemmatimonadaceae_unclassified
loroplast_unclassified
nis
ed;s__Gemmatimonadaceae_unclassified
eae_unclassified

e_group_B)_unclassified

a_(SAR406_clade)_unclassified

e_group_B)_unclassified
