# Supplementary material for: Bacterial Colonization in the Airways and Intestines of Twin and Singleton Preterm Neonates: A Single-Center Study
Source: Can J Infect Dis Med Microbiol. 2023 Aug 1;2023:2973605. doi: 10.1155/2023/2973605 (PMC10409585; doi:10.1155/2023/2973605)
Supplement: Supplementary Materials — Supplementary Material 1: sequence information identified in singleton and twin preterm neonates. Supplementary Material 2: abundance of bacterial taxa and corresponding 16SrRNA gene sequences in singleton and twin preterm neonates. Supplementary Material 3: class-, order-, family-, and species-level differences were determined using the Kruskal–Wallis test. Supplementary Material 4: three levels of annotation in the KEGG database on metabolic pathways. [file 2973605.f1.zip › Supplementary Material 4 KEGG_level1.pdf]

Sample gene sequences were annotated using the KEGG database on metabolic pathways based on their metabolic function prediction using PICRUSt2.

KEGGLevel1	description	A1	A10
Genetic Information Processing	Genetic Information Processing	33936384.75	24727898.53
Unclassified	Unclassified	29152591.17	22824867.12
Cellular Processes	Cellular Processes	7992618.08	6967753.85
Organismal Systems	Organismal Systems	1580022.68	1315423.83
Environmental Information Processing	Environmental Information Processing	26930613.05	19546303.31
Human Diseases	Human Diseases	2822849.71	2243678
Metabolism	Metabolism	85411583.05	64989596.14

A11	A12	A13	A14	A15	A16	A17	A18
25133060.22	23850292.27	28252823.64	17253773.49	16639555.44	19235445.16	30303085.41	18874169.04
15223611.51	21849359.91	26055497.67	15581367.02	14524341.79	16013047.17	27939304.88	17467533.56
1646013.14	6951222.10	7830102.24	4810913.92	3772614.51	3989543.25	8210143.91	5084889.07
532759.85	1269191.62	1481093.58	890845.68	780407.07	830220.09	1583403.29	992739.67
22868246.48	19374347.82	22095493.86	13629485.34	13030969.38	15561940.20	23177863.24	14429314.82
1213336.89	2188794.95	2624943.30	1594315.94	1354956.36	1483764.47	2716034.35	1704875.10
51691794.67	63146357.85	73942557.19	44640836.89	41582315.58	47032970.18	78979495.82	48897901.69

A19	A2	A20	A3	A4	A5	A6	A7
19029715.73	16855246.96	15289300.12	39309375.92	17126598.94	29407818.24	37340765.21	38722411.21
17644499.07	15840847.78	12297225.89	36294698.94	17066491.47	18618572.72	28855141.19	35796650.41
5024297.79	4427325.56	2541293.51	10613722.31	4621808.38	2209911.41	6641043.89	10718769.02
1002555.36	747795.08	528271.92	2047053.77	809718.71	789505.33	1402501.33	2047507.05
14464904.15	18909600.00	11038231.63	30433469.75	14319067.96	18727139.98	31262072.97	30576665.06
1684363.47	1133464.20	1147338.62	3495800.01	1493204.50	1614693.74	2673101.12	3473406.24
49498394.92	42667567.96	37430635.57	102905155.01	44370340.46	63545638.12	87694096.30	102331618.93

A8	A9	B1	B10	B11	B12	B13	B14
39713111.86	30079185.96	36021670.37	22401422.90	25114908.92	29151016.41	19383705.39	22935467.22
36676638.24	27758654.48	21524215.01	20701159.93	23334677.94	27048936.96	17912801.87	21228252.97
10854839.64	8773885.55	2253722.99	6221246.37	6467743.86	7595794.90	5315962.94	6319787.03
2107668.01	1602755.62	762779.40	1174332.27	1328606.86	1523192.87	1018247.45	1206037.51
30846240.68	24636209.61	32625133.20	17426415.20	18751988.16	22092372.38	15060927.63	17917335.24
3537316.08	2870597.34	1705558.66	2078976.86	2168631.50	2575658.03	1718596.09	2077714.78
104354529.75	80112717.75	73615257.90	58301590.39	65216579.12	75908188.63	50448543.50	60001285.60

B15	B16	B17	B18	B19	B2	B20	B3
14157125.61	17872119.66	6308887.71	17776787.53	17194384.15	34525988.54	17994976.86	40682489.53
9396033.81	16524853.65	5480819.83	16456601.54	16005968.97	31080678.34	16619115.78	37435748.84
1408678.90	4906201.37	1524088.24	4813691.42	4682522.74	10550443.26	5012397.34	11393984.81
465244.60	939418.42	263982.72	930535.66	902503.85	1814766.54	948673.40	2150708.18
8343459.17	13829659.88	5479621.37	13727761.65	13425033.23	29510748.36	14095311.87	31932974.18
725711.25	1611560.81	514283.85	1590912.66	1569544.23	3111831.07	1663526.57	3696612.60
28260059.48	46630886.78	16296746.62	46291244.16	44725819.57	91353988.96	46733184.43	106774185.92

B4	B5	B6	B7	B8	B9	C1	C10
38199559.50	15178833.30	38497138.60	39445043.52	40724981.08	11606053.75	18408062.23	17113368.68
35378999.85	14096553.10	35588634.72	36754138.72	37567820.30	9356882.67	12359909.41	15133270.54
10115173.55	4177346.01	10308227.61	10503213.99	11053901.15	2230854.97	2191261.51	5307974.18
1997098.95	791917.07	2023224.24	2064811.36	2127580.12	465783.61	438732.74	846276.24
29419553.25	11832704.41	29354651.92	30159973.85	31026611.61	9383178.21	14346535.77	16900187.54
3363217.63	1353626.83	3441339	3577320.17	3725554.21	857002.80	1342166.23	1928430.99
100476798.97	39032277.41	100854418.11	102233667.36	105922356.64	27838539.09	41905547.87	47268306.32

C11	C12	C13	C14	C15	C16	C17	C18
9784486.10	14239801.73	27677435.58	9728466.44	9767823.54	8911981.37	8867403.05	27008875.09
5605041.95	13028524.58	18034155.86	5471332.36	5453932.61	4937661.23	4995035.53	23548872.04
712797.09	4765096.47	1264359.44	594855.75	615859.40	568493.60	597780.61	5479513.20
228978.64	776527.58	347469.17	213167.76	221808.78	198985.21	206722.40	1219763.83
7990647.39	14383744.46	18201207.68	7673623.10	7824750.59	7209698.97	7168746.92	23774244.63
439770.30	1663961.35	1509505.19	420363.78	414108.26	381191.88	390141.79	2118862.88
19398480.91	41246444.33	62394405.99	19185106.90	19056549.97	17357162.05	17401766.17	71571575.31

C19	C2	C20	C3	C4	C5	C6	C7
13324089.90	15189667.80	10463814.95	17481183.26	9694939.10	9442003.20	17409076.61	13817679.79
7112772.11	12595224.30	5758467.70	15049932.06	7101320.74	5480470.40	13023074.96	9225228.05
909637.20	4654417.67	838088.75	4157554.93	1940580.18	754639.93	3241673.77	1036643.65
372438.67	672790.06	331092.21	874087.17	323826.90	227239.80	845141.92	266518.82
8920815.88	14930612.56	7349687.20	15378405.80	8642919.30	7716852.36	16489935.64	10773147.87
579690.84	1438793.53	498159.75	1424591.57	803545.26	450381.36	1145301.61	996712.51
25509337.80	39890255.84	20569390.51	46884750.55	23668187.72	19193185.21	46423837.76	31865710.58

C8	C9	D1	D10	D11	D12	D13	D14
14075959.81	16032775.17	11466178.73	9666512.28	9087168.98	25113795.84	18761607.90	15768593.44
12472105.34	14852339.35	7066790.00	5514718.49	5083279.57	16613251.80	15376798.40	14224340.81
4689891.39	5889596.67	1421975.39	665151.79	582955.58	1359751.06	4603681.45	5126821.93
727938.48	824291.03	379535.04	224587.28	204782.13	339179.65	873489.21	867438.08
14771997.01	17868006.88	8753946.40	7814550.80	7293694.97	16870227.66	17095582.71	16271545.51
1605730.67	1935558.80	659460.28	416827.80	391414.48	1417374.56	1775500.24	1727750.36
40463472.64	46501910.25	24312296.91	19044118.26	17712377.26	57105436.75	50093834.53	47213869.84

D15	D16	D17	D18	D19	D2	D20	D3
14801264.38	19077321.95	13516581.02	9973353.54	9429343.85	20162106.13	9541776.11	29165598.96
12185313.10	12087821.49	9348068.40	5538705.71	5147491.29	17118234.34	5584043.02	19223435.66
3892070.07	979463.94	688409.86	606428.37	536461.45	6373781.41	804577.93	1583178.57
673513.24	282051.04	204400	219075.83	206250.21	1080856.24	236076.34	399055.66
13898163.40	13133215.02	10311591.70	7953997.05	7536453.47	19616649.21	7780883.50	19482782.73
1406506.84	1007778.77	1182084.20	425998.47	385162.30	2188358.30	463610.89	1637993.22
38694211.64	41943864.35	32373950.13	19485441.07	18143970.88	56259789.33	19332421.71	66244311.31

D4	D5	D6	D7	D8	D9
9515225.00	24233113.99	17929306.62	18751146.62	8913790.08	18259797.72
8874277.65	23600284.84	15860095	16526110.17	5190103.53	17118759.72
3630231.06	4124198.91	5866374.55	6268668.60	729547.79	5369721.92
515659.57	745138.68	907402.07	989077.01	220982.89	812619.33
10767533.31	23625273.03	19696462.83	19840323.87	7284402.26	18897044.58
1247017.74	1575011.19	1983177.47	2079095.80	416670.90	1762495.60
28011339.52	55175820.54	51212933.08	52916516.77	17967094.16	48639554.62
