# Supplementary material for: Bacterial Colonization in the Airways and Intestines of Twin and Singleton Preterm Neonates: A Single-Center Study
Source: Can J Infect Dis Med Microbiol. 2023 Aug 1;2023:2973605. doi: 10.1155/2023/2973605 (PMC10409585; doi:10.1155/2023/2973605)
Supplement: Supplementary Materials — Supplementary Material 1: sequence information identified in singleton and twin preterm neonates. Supplementary Material 2: abundance of bacterial taxa and corresponding 16SrRNA gene sequences in singleton and twin preterm neonates. Supplementary Material 3: class-, order-, family-, and species-level differences were determined using the Kruskal–Wallis test. Supplementary Material 4: three levels of annotation in the KEGG database on metabolic pathways. [file 2973605.f1.zip › Supplementary Material 4 KEGG_level2.pdf]

Sample gene sequences were annotated using the KEGG database on metabolic pathways based on their metabolic function prediction using PICRUSt2.

KEGGLevel2	description	A1
Poorly Characterized	Poorly Characterized	11012812.82
Replication and Repair	Replication and Repair	14889870.48
Glycan Biosynthesis and Metabolism	Glycan Biosynthesis and Metabolism	3507318.28
Digestive System	Digestive System	79018.26
Nervous System	Nervous System	85528
Metabolism	Metabolism	3951396.28
Cardiovascular Diseases	Cardiovascular Diseases	78330
Metabolism of Other Amino Acids	Metabolism of Other Amino Acids	3557521.44
Cell Growth and Death	Cell Growth and Death	1115302.76
Environmental Adaptation	Environmental Adaptation	321151.36
Carbohydrate Metabolism	Carbohydrate Metabolism	16097980.18
Immune System Diseases	Immune System Diseases	114115.24
Folding, Sorting and Degradation	Folding, Sorting and Degradation	5032069.88
Circulatory System	Circulatory System	92341.50
Lipid Metabolism	Lipid Metabolism	6833540.65
Infectious Diseases	Infectious Diseases	1092862.97
Metabolism of Cofactors and Vitamins	Metabolism of Cofactors and Vitamins	7079792.93
Nucleotide Metabolism	Nucleotide Metabolism	6464959.08
Amino Acid Metabolism	Amino Acid Metabolism	17737688.56
Translation	Translation	9305689.06
Energy Metabolism	Energy Metabolism	10223582.61
Sensory System	Sensory System	0
Transport and Catabolism	Transport and Catabolism	606179.88
Signaling Molecules and Interaction	Signaling Molecules and Interaction	334636.92
Enzyme Families	Enzyme Families	3999026.81
Excretory System	Excretory System	88077.77
Xenobiotics Biodegradation and Metabolism	Xenobiotics Biodegradation and Metabolism	5723608.20
Cell Motility	Cell Motility	6271135.44
Endocrine System	Endocrine System	804409.99
Membrane Transport	Membrane Transport	21503455.27
Genetic Information Processing	Genetic Information Processing	5330113.72
Biosynthesis of Other Secondary Metabolites	Biosynthesis of Other Secondary Metabolites	1119350.92
Transcription	Transcription	4708755.33

Cancers	Cancers	425276.62
Signal Transduction	Signal Transduction	5092520.86
Metabolism of Terpenoids and Polyketides	Metabolism of Terpenoids and Polyketides	3067213.39
Cellular Processes and Signaling	Cellular Processes and Signaling	8858268.35
Neurodegenerative Diseases	Neurodegenerative Diseases	997167.11
Metabolic Diseases	Metabolic Diseases	115097.77
Immune System	Immune System	109495.80

A10	A11	A12	A13	A14	A15	A16	A17
8486813.77	6360419.63	8126190.39	9677337.77	5784477.08	5463807.41	6113958.67	10401555.83
10783405.26	11310671.06	10415429.78	12327980.97	7550188	7290340.07	8459592.47	13218378.78
2682831.16	1982035.70	2575102.52	3083786.08	1860856.04	1756080.98	1932105.47	3280737.84
61145.82	54373.06	59203.95	68414.81	42568.72	40728.37	45869.35	73008.62
64888.36	54876.96	61297.09	75847.46	45653.87	43766.76	47672.93	82884.87
3047897.01	2298892.81	2980975.37	3486121.11	2121225.85	1937287.69	2168794.88	3692672.83
66967.08	7981	66975.66	82934.68	50022.66	35744.34	36629.66	80006.48
2760619.71	1928552.63	2711789.18	3114663.80	1879691.72	1712780.89	1923107.31	3324675.18
855983.10	620546.69	843309.35	1000593	603111.39	540426.74	600757.16	1033064.61
260198.86	172211.34	251549.88	291710.77	175599.07	160677.90	179030.73	313228.21
11173718.59	14922660.60	10949616.70	12759925.46	7774431.61	7726448.68	9369440.39	13597691.57
78123.55	103688.46	75900.64	87404.38	53190.71	55566.17	65863.37	95707.58
3869428.59	2782512.17	3719816.12	4415706.30	2683181.27	2482062.08	2765354.43	4740230.32
80955.54	8786	85489.49	89460.01	57447.49	38907.01	41860.99	92720.68
5365416.07	3463396.21	5228611.47	6051277.93	3617962.55	3295352.19	3673675.48	6488072.06
802644.93	712580.05	773928.05	941719.65	574204.29	525778.61	600767.20	989637.11
5578008.15	3316234.40	5371057.43	6392025.00	3866575.02	3515262.27	3842445.21	6814208.42
4644460.92	5044789.74	4485627.95	5317914.91	3251009.31	3184749.59	3693895.78	5682525.27
13882820.06	8585125.06	13521842.75	15741964.20	9497790.19	8571633.14	9493436.00	16816375.43
6682019.77	7186433.37	6418176.24	7651301.90	4680253.03	4579814.46	5310792.58	8214166.14
7911093.13	5760108.38	7670251.80	9045952.81	5456978.57	5024596.40	5624612.12	9604530.21
0	8	0	0	0	0	9	0
507696.03	201065.17	485128.76	571558.37	338836.82	300671.73	319645.75	611513.32
239347.94	262690.65	242849.37	263487.83	165852.51	153775.91	181561.31	287432.28
3028636.83	2439937.78	2942312.91	3445182.11	2109297.98	1925588.97	2186574.63	3689497.71
76860.61	9031.56	74266.68	86047.11	52532.91	43655.32	44143.76	90680.13
4749400.72	1791964.49	4563356.94	5341187.47	3130076.08	2832894.46	3000207.76	5768343.03
5604074.72	824401.28	5622783.99	6257950.87	3868965.71	2931516.04	3069140.34	6565565.98
671178.15	221485.47	644961.91	756204.35	451856.16	391693.86	412539.79	808009.48
15149792.39	20419380.13	15057443.23	17143671.82	10591816.16	10423376.87	12668799.36	17940493.64
4001057.33	3415520.30	3822930.05	4593657.78	2756170.22	2655150.33	2976039.68	4926404.58
873691.83	622404.37	844935.29	993634.36	588375.52	548103.66	612222.37	1077606.90
3393044.91	3853443.62	3296870.13	3857834.47	2340151.19	2287338.83	2699705.68	4130310.17

372953.48	44744.15	361800.68	443554.16	264826	209792.10	213160.16	448121.85
4157162.98	2186175.70	4074055.22	4688334.21	2871816.67	2453816.60	2711579.53	4949937.32
2338898.97	1834585.31	2281852.91	2655043.06	1607792.30	1488824.35	1681247.66	2835232.20
7289099.01	3148778.77	6919264.10	8298381.01	4919493.87	4468096.36	4754253.94	8918671.64
844369.80	240476.04	832410.62	978552.93	593836.61	472294.49	501453.02	1006657.24
78619.16	103867.19	77779.30	90777.50	58235.67	55780.65	65891.06	95904.09
100196.49	11987.46	92422.62	113409.07	65187.46	60977.85	59093.54	122871.30

A18	A19	A2	A20	A3	A4	A5	A6
6487325.21	6569577.44	5962578.58	4781602.22	13525352.81	6111737.53	7568937.46	11198237.96
8222193.48	8288423.91	7183207.72	6753768.32	17148167.72	7394878.62	13323664.04	16539005.28
2065883.84	2081395.43	2374122.10	1406826.99	4266905.68	2434244.20	2058699.44	3552061.82
45692.53	46280.11	30359.84	35988.38	96099.21	42563.10	69085.42	84997.75
47446.53	49448.96	48955.36	36997.39	103962.61	50079.50	113303.84	92858.86
2290123.65	2295212.96	2414749.96	1562927.96	4803096.25	2545373.40	2383410.48	4041984.01
50608.34	48456.56	14402.32	22316.34	102384.34	38156.50	9811.02	62045.02
2048866.23	2080134.21	1771882.38	1504863.33	4365581.02	1815022.25	2335668.27	3531669.42
638303.63	644354.83	399553.64	440143.05	1357418.13	520799.67	726833.72	1124536.15
195407.22	196270.90	224678.06	118764.81	401968.40	169009.90	159321.34	324535.21
8308716.52	8398278.56	9014613.38	7443720.12	17681204.48	8058675.01	14194771.40	19263812.42
58928.89	60681.98	52126.24	42023.42	126663.28	50843.70	71936.94	132900.17
2968891.89	2993583.35	2483912.46	2246247.71	6148250.86	2627656.43	4079410.80	5033648.13
57060.51	55171.87	16857.48	25277.50	121717.02	39663.50	12002.02	69750.02
4003587.50	4065601.37	3023317	2824894.56	8479451.85	3427505.24	4158466.65	6655237.08
617719.86	610533.92	597610.58	522832.05	1260082.71	603937.67	860487.23	1155152.03
4249394.10	4297623.26	3583404.44	3393039.59	8866112.66	3797373.87	6308183.98	6776456.82
3529024.72	3570600.52	3222054.98	2999250.58	7390037.38	3236318.59	5937392.13	7274991.36
10420865.16	10556765.38	8519453.70	7890236.92	21962173.13	8943534.74	13089631.13	16971623.89
5130826.41	5180055.52	4235970.26	4260071.78	10648668.50	4478743.28	8504614.91	10378799.52
5986165.50	6041551.29	4599700.32	4550249.30	12521267.28	5402721.24	7702489.50	10302729.32
0	0	0	0	0	0	0	0
381175.72	387040.29	214396.74	220923.33	791708.85	311857.59	303716.20	536769.38
168350.42	170526.70	136368.44	133784.21	369199.78	142954.67	241965.47	369784.18
2279200.38	2284099.25	2273842.50	1699269.23	4750414.06	2149305.50	2951295.57	4123944.38
58563.72	59578.24	51151.68	26919.04	120746.81	50039.24	55986.03	67380.46
3601069.49	3681249.52	2223667.94	1996464.08	7545042.09	3062307.03	1959921.16	4984368.06
4065409.72	3992902.67	3813375.18	1880227.13	8464595.33	3789151.12	1179361.49	4979738.36
510661.66	516213.62	302811.52	248773.06	1043802.20	392068.33	363696.51	675101.34
11159294.82	11210276.03	15941915.62	8920235.86	23652874.55	11237273.35	15890641.03	26073720.61
3087914.18	3109994.15	2569731.76	2168581.09	6362710.29	3010182.52	3441666.80	5590820.48
645100.96	657488.21	717324.72	462789.42	1370403.21	590269.93	709101.74	1125994.20
2552257.26	2567652.95	2952156.52	2029212.31	5364288.84	2625320.61	3500128.49	5389312.28

285695.47	281337.31	165821.96	158690.22	577734.95	234611.91	175212.26	339218.16
3101669.58	3084101.42	2831315.94	1984211.56	6411395.42	2938839.94	2594533.48	4818568.18
1760027.29	1783607.92	1344184.50	1259031.45	3706562.17	1453062.86	2140017.15	3131207.53
5602170.52	5669714.52	4893787.48	3784114.62	11603539.59	5399198.02	5224557.98	8024098.74
633898.37	624809.15	235110.08	344912.28	1304168.25	498987.32	343751.67	846162.50
58024.17	58544.55	68393.02	56564.31	124766.48	66667.40	153494.62	137623.24
77907.50	79591.66	72981.14	35551.74	158757.52	66295.14	16110.17	87877.69

A7	A8	A9	B1	B10	B11	B12	B13
13342449.58	13681574.23	10301957.04	9006883.83	7679798.27	8712050.02	10076371.77	6662517.91
16898287.87	17307615.92	13137678.92	16234244.24	9761471.87	10925782.12	12688239.83	8451746.78
4208744.95	4310333.05	3255339.05	2807637.92	2470662.29	2740746.78	3190691.27	2115158.42
96400.25	95956.36	74353.92	77856.41	53861.68	60471.28	70513.56	47823.59
103001.42	105033.08	83964.91	82433.61	57957.29	63878.06	78530.61	49007.26
4766210.50	4855242.75	3817792.16	3266561.07	2757466.59	3002488.96	3517049.20	2367667.66
102855.34	103514.68	93875.48	9802.32	64532.34	59521	75138.53	49092.32
4372138.46	4445032.12	3407449.42	2734826.88	2443611.29	2745699.52	3181414.94	2133924.24
1367538.71	1370488.58	1104349.40	884724.51	775751.13	828152.88	1004753.37	649183.24
401662.03	413729.34	315151.84	243073.41	233802.67	258201.20	298445.37	201686.37
17600084.50	17912116.92	13969735.65	21327484.89	10029341.37	11022344.12	12911925.69	8622748.04
126014.52	127223.44	94026.35	148351.89	68198.25	81246.14	92277.86	61641.01
6062542.42	6217142.13	4682838.52	3979450.24	3513025.83	3957873.84	4577668.26	3038227.70
125742.51	125891.51	103911.48	11397.99	70692.50	71272.50	81088.52	61738.99
8457155	8641961.74	6595599.98	4889115.39	4763669.62	5387357.26	6207225.06	4156103.12
1235676.45	1272829.30	1012503.52	1007556.40	753725.40	793207.90	936188.15	622207.28
8769823.89	8937239.42	6840701.98	4750895.85	5055646.83	5643828.30	6612543.68	4346753.89
7300647.79	7447306.10	5685242.92	7247013.06	4189334.46	4688041.28	5489090.34	3618059.98
21887237.76	22303253.22	17093472.41	12213589.12	12394443.90	13936051.22	16177833.39	10778449.95
10466663.35	10748035.46	8106631.54	10318993.90	6065508.25	6834956.84	7948204.07	5239956.50
12434712.33	12646651.36	9772163.47	8195716.54	7133517.04	7908310.72	9272331.46	6138652.46
0	0	0	0	0	0	0	0
792729.59	813864.17	618937.04	284368.05	451869.36	514669.12	587164.61	391886.76
375610.78	382919.57	297458.40	377858.35	204876.26	225537.40	255874.63	182847.36
4697023.46	4846209.52	3713579.58	3482178.74	2747520.60	3023763.86	3492284.04	2356371.55
122221.79	124246.97	92886.09	14847.22	67389.76	79549.28	90114.23	60009.42
7534782.53	7695055.27	5812389.98	2467369.87	4205131.37	4901407.02	5630184.87	3698223.48
8558500.72	8670486.89	7050599.11	1084630.43	4993625.88	5124921.86	6003876.92	4274892.94
1043480.73	1083342.15	815073.95	318405.97	600232.12	691644.12	783473.65	519049.98
23789097.62	23924772.50	19197991.66	29177378.39	13477540.30	14503831.30	17180624.07	11663217.09
6268601.97	6434027.40	4878561.16	4855107.15	3649207.38	4109128.92	4772197.25	3138442.27
1366686.89	1410314.01	1081483.27	882556.45	784396.63	871994.18	1014772.11	671119.53
5294917.57	5440318.35	4152036.98	5488981.99	3061416.95	3396296.12	3936904.25	2653774.41

575987.56	583041.53	487842.15	58291.22	348603.52	355711.56	432852.83	281536.13
6411956.66	6538548.61	5140759.55	3069896.46	3743998.64	4022619.46	4655873.68	3214863.18
3702581.37	3759057.02	2885560.04	2616873.19	2084314.99	2347034.86	2727891.78	1812978.84
11419388.36	11705793.86	8760344.12	4395662.96	6614687.69	7511010.04	8683318.74	5744174.03
1309258.62	1325563.96	1082405.91	329188.23	772395.06	803752.62	948906.76	643823.77
123613.75	125143.17	99943.93	152368.60	71522.29	75192.28	90293.90	60295.58
154998.32	159468.60	117413.43	14764.79	90396.25	103590.42	121026.93	78931.84

B14	B15	B16	B17	B18	B19	B2	B20
7895260.83	3359041.72	6147646.98	1966458.32	6121611.53	5943871.67	11516153.28	6161190.07
10002559.03	6423367.73	7797842.29	2730960.11	7755325.49	7491592.73	15154041.84	7848230.93
2508523.87	1557124.06	1946479.76	581652.16	1944049.42	1900842.61	3695732.06	1977735.58
56266.51	50081	43972.17	11672.78	43757.56	42769.78	90725.50	44071.27
59791.28	51152.80	45842.97	18426.48	46062.74	45421.85	85425.51	45446.06
2818836.08	1482362.38	2183640.29	814954.50	2163757.09	2112094.41	4364999.63	2214722.85
61962.34	2533	47705.66	10992	46576.34	46853.68	91474.34	50782.02
2542687.40	1066086.35	1974059.92	637892.65	1953956.31	1877377.15	3939199.04	1965789.21
790500.19	361904.19	612342.13	190647.57	607676.07	590724.76	1204067.14	613943.53
238278.69	106249.92	185226.16	63796.56	183655.73	180449.83	364477.72	188249.31
10297022.34	5804739.16	7966005.57	3404331.36	7905923.35	7649929.55	16120834.66	8003130.54
72582.45	52321.60	56731.33	20879.48	56634.99	53101.45	113809.78	55150.04
3593029.65	1799692.29	2802406.81	894408.77	2787325.73	2698842.83	5334453.83	2823518.52
73261.51	2979	56973.99	11900	54906.51	51794.01	136220	58385.52
4944669.46	1773358.41	3845147.05	1182124.43	3805728.33	3652823.75	7529098.75	3826270.40
748465.66	395282.86	579193.06	239410.20	573627.56	569698.78	1084052.59	599911.09
5169114.94	2646468.82	4021636.77	1363584.18	4001646.65	3886295.67	7673756.84	4044719.31
4297067.72	3066286.62	3346139.21	1236894.84	3333086.90	3228934.81	6523526.44	3363969.10
12802958.18	5153339.87	9953449.47	3396541.35	9875458.74	9500721.61	19577152.35	9956978.47
6199357.42	4166533.43	4839700.84	1731978.05	4817752.75	4661082.48	9237685.86	4869605.08
7304984.07	3407813.55	5687347.77	1920220.83	5646233.25	5468240.50	11132236.42	5715890.55
0	0	0	0	0	0	0	0
467367.61	164862.57	363835.83	97419.13	360560.17	350480.54	686211.24	365226.80
216672.31	176966.45	167996.52	85410.91	164379.55	156520.56	372734.14	167057.04
2794119.64	1478038.07	2168605.95	815347.41	2145793.31	2089382.65	4221638.69	2197119.75
70272.56	9092.66	55011	14897.54	54763.49	52257.02	108616.29	54888.48
4380038.69	906333.07	3420947.14	945036.69	3397672.09	3268056.83	6423898.94	3382768.56
5061919.23	881912.14	3930023.41	1236021.54	3845455.18	3741317.44	8660164.88	4033227.01
614959.95	180107.22	479096.47	121168.88	473994.09	457356.34	898992.95	484021.31
13890593.10	7099305.13	10701497.42	4496313.23	10641986.27	10407536.39	23086784.52	10900996.30
3719517.95	2085861.03	2898053.03	967243.15	2888270.21	2804721.11	5444284.61	2926590.81
802535.83	451021.62	620068.40	257631.34	614280.01	602471.19	1187300.73	621565.75
3140521.12	1767532.16	2432169.72	951540.78	2416383.56	2342866.11	4799807.01	2453622.33

344218.74	72903.49	267261.81	69500.59	264029.70	263879.31	505005.57	278621.16
3810069.83	1067187.59	2960165.94	897897.23	2921395.83	2860976.28	6051229.70	3027258.53
2157563.46	949449.88	1680999.77	555489.38	1667415.80	1600743.25	3329614.04	1677247.21
6794638.11	2468768.68	5295513.35	1732163.86	5282962.71	5145281.78	9755240.82	5316612.05
778095.86	124808.53	604831.45	150655.99	594480.83	582216.12	1199764.41	622498.03
72389.73	77861.77	55837.50	22845.59	55563.24	53794.89	117724.38	56564.23
93207.01	65582	73295.66	22120.48	73395.54	72455.02	130308.57	73611.45

B3	B4	B5	B6	B7	B8	B9	C1
13926417.64	13196670.50	5243120.95	13264009.54	13666850.08	13983100	3608835.14	4800855.82
17760431.07	16649484.47	6617396.42	16776585.05	17184277.33	17751462.78	5141197.01	8266761.79
4408563.95	4156900.67	1679328.29	4178883.80	4358132.88	4443073.08	1123155.99	1944793.96
98641.04	92948.56	38660.83	91892.32	95715.72	95997.36	24809.06	42502.34
104673.90	103197.79	39224.66	99576.29	101834.22	105487.28	28076.81	49217.74
5001059.15	4664736.98	1851258.42	4679944.58	4824568.50	4971169.16	1275751.91	1684434.92
111703.02	97568.49	37492	102442.32	106258	114778.82	20184.98	17003.07
4549868.24	4260195.76	1634291.80	4277415.22	4280453.28	4465058.04	1129323.72	1516491.43
1411628.25	1327253.54	494076.12	1331406.45	1328660.83	1404623.62	352482.97	505004.16
421853.57	387620.38	161749.47	391610.55	406022.38	418502.72	101902.70	83974.66
18355197.81	17224456.44	6663863.07	17214321.32	17416191.50	18188035.67	5725589.03	9144465.47
129688.29	125031.17	46528.33	124311	123987.76	126475.33	41162	74421.70
6364187.48	5989932.25	2376604.52	6046908.72	6202151.49	6376005.38	1615693.41	2345262.98
136743.03	113758	46982	118634.99	116508.50	127476.98	23043.98	16716.24
8845136.71	8288315.80	3184230.24	8338776.44	8385571.71	8709248.19	2158185	2784733.71
1317736.34	1210337.21	502868.70	1235276.43	1300759.74	1351039.05	355213.51	794240.94
9138834.02	8665088.81	3392158.71	8698655.51	8888940.07	9157472.99	2229831.37	3262205.80
7618608.28	7202779.98	2822205.81	7215546.39	7361432.58	7604873.98	2259472.04	4027914.39
22850864.81	21449874.15	8294777.40	21556886.97	21755505.87	22567500.20	5553685.02	8850421.66
10984739.14	10368167.69	4096571.66	10449382.41	10697816.76	11032842.18	3212410.87	5403109.39
12987357.41	12222500.58	4741677.75	12280511.91	12454839.63	12895049.63	3296526.75	4251719.61
0	0	0	0	0	0	0	0
831604.06	771066.02	303858.81	781156.83	805335.90	821566.05	180641.32	206928.39
394699.64	352473.54	143279.88	354279.98	351841.26	377877.99	108162.31	298047.83
4966143.05	4588197.30	1859867.38	4644913.15	4769659.38	4975562.50	1293154.54	2205504.02
125861.31	119740.29	44731.99	120570.40	120104.69	122068.76	24086.75	16036.84
7775627.33	7451159.53	2844034.16	7471135.68	7539914.98	7693617.83	1707996.75	2023125.87
9150752.50	8016853.99	3379411.08	8195664.33	8369217.26	8827711.48	1697730.68	1479328.96
1100909.88	1025472.22	395901.46	1046667.82	1059183.62	1095111.72	231893.14	214012.23
24742195.34	22918806.77	9138036.33	22752624.72	23330832.84	23969628.60	7733018.39	11997494.02
6575229.16	6216309.72	2447206.97	6271578.22	6440387.25	6608506.77	1758512.03	2537965.89
1419474.90	1335564.39	534329.06	1331197.85	1365000.01	1428057.96	357491.71	616715.59
5573131.84	5191975.09	2088260.70	5224262.42	5360797.94	5564670.74	1636752.46	2392928.07

611184.12	558677.17	220008.49	572063.39	598672.86	618926.88	115605.37	104811.42
6796079.20	6148272.94	2551388.20	6247747.22	6477299.75	6679105.02	1541997.51	2050993.92
3858509.41	3631765.56	1381513.74	3646173.87	3658025.47	3794806.57	1004127.17	1277456.36
11933042.89	11301282.65	4554966.76	11373102.38	11822332.89	12005044.37	2713783.59	3336652.78
1397122.50	1250836.98	499559.82	1287801.55	1326545.35	1386323.43	284374.70	254630.15
129178.33	120766.61	47169.49	119444.31	121096.46	128010.70	40462.24	97058.95
162025.45	154361.71	64666.66	154271.87	165442.23	162935.30	31971.17	16272.69

C10	C11	C12	C13	C14	C15	C16	C17	C18
5600273.26	2275330.21	4835422.51	7558776.35	2237628.38	2218899.98	2020696.18	2033279.31	8401204.64
7571471.84	4503104.06	6310726.88	12437126.23	4477211.72	4503238.53	4113072.69	4086930.51	11676217.03
1883118.57	884609.98	1526016.34	1959655.76	860087.16	879696.28	798558.97	795733.01	3147704.48
35498.06	23759.07	24949.76	61017.66	23266.46	23601.66	21775.38	21446.67	94221.41
38758.20	26122.15	40399.35	64555.07	26262.36	26842.18	23614.23	24364.67	143991.01
2526929.52	803132.67	2203728.20	2221567.35	766367.94	779700.02	700662.01	716394.93	3711528.24
70384.66	3643.32	65167	2509.18	2252.43	2420	2543	2861.50	24917.15
1839803.32	733106.85	1634829.49	2362553.78	722812.58	718375.82	654944.32	656000.86	2812370.69
626235.10	258087.15	589436.11	630174.58	250920.61	253602.89	233616	232565.56	769207.63
135496.22	53837.52	114556.36	127794.83	51627.94	51968.48	47729.34	47690.86	216645.76
9207082.69	4459998.91	7817726.80	14949943.99	4448277.35	4409499.38	4019033.38	4014381.89	13591496.67
62165.76	25348.40	43406.76	62865.82	24996.85	25307.41	22988.93	23095.11	102606.14
2586788.35	1068734.95	2195017.79	3720675.26	1068908.20	1055400.18	957282.73	967212.45	4348410.02
58994	3184.50	48604	2498.37	2227.54	2197.36	2332	2517.78	31291.24
3850920.98	1298174.13	3517233.89	4132882.49	1272474.60	1253513.47	1148438.84	1154867.30	4479733.71
711536.41	260976.19	598402.92	922949.16	256907.67	253431.12	232084.53	233979.66	990366.29
3646380.12	1521597.57	3159974.96	5988421.96	1533442.17	1495812.77	1359467.63	1371053.66	6963794.26
3282700.71	2267758.71	2696989.79	5690561.40	2252525.43	2276436.72	2084925.12	2058456.30	5463351.69
9738434.94	3709146.25	8480227.66	12926217.69	3690990.74	3641825.32	3312297.14	3319939.30	13070861.50
4238159.62	3057630.38	3434011.11	7904686.56	3048361.33	3076432.64	2812949.46	2779745.91	7266774.80
5568861.20	1878764.30	4883410.57	7581496.07	1873695.34	1828364.84	1650400.32	1675762.84	11675519.36
0	0	0	0	0	0	0	0	50
286164.11	105395.54	260418.52	195054.50	100223.39	103708.22	93609.12	94236.72	361409.58
183081.43	226964.45	165940.73	190719.80	216701.02	228222.42	212211.29	205498.50	222127.11
2237690.27	1109541.19	1906713.99	2754307.09	1083210.29	1096910.65	1003110.57	1000383.54	3858463.00
48610.19	2931.91	47335.93	2457.48	2168.36	2388.79	1808.77	3085.44	97323.86
3858449.34	730490.76	3750472.74	1476550.14	651344.62	664441.91	612352.46	634123.09	3312396.68
4395574.97	349314.40	3915241.84	439130.36	243711.75	258548.29	241268.48	270978.33	4348895.99
480827.17	72456.81	454526.66	84774.01	64118.67	68494.26	58583.59	65842.76	533102.64
13701742.13	6830756.30	11605647.03	15607291.27	6580574.02	6715333.27	6182871.97	6141929.98	19810614.48
2580329.91	1266118.41	2162649.20	3129045.28	1234112.01	1260173.27	1139067.74	1140322.60	4678594.22
631988.24	242721.08	532441.79	697316.23	237294.09	241208.12	214887.24	216310.61	959837.53
2716948.87	1155016.71	2300045.95	3614947.53	1133985.19	1132752.19	1028676.49	1033514.18	3717473.24

340971.64	19886.18	313004.02	133189.32	16438.48	14253.91	13217.37	16371.82	294112.30
3015363.98	932926.64	2612156.70	2403196.61	876348.06	881194.90	814615.71	821318.44	3741503.04
1522875.94	562571.18	1340406.31	1874499.39	558952.53	550464.69	498746.06	504753.77	2236045.74
4425737.85	1260460.66	3826724.67	5124766.88	1233224.03	1195159.34	1077235.30	1105038.69	6757544.94
617494.62	78631.94	540091.88	262361.55	70081.53	67836.45	64215.24	66980.26	516667.91
125877.90	51284.27	103888.77	125630.16	49686.82	50859.37	46142.81	46853.44	190193.09
48092.40	46686.68	46155.52	4371.75	43496.43	46316.05	43141.90	41774.22	103137.91

C19	C2	C20	C3	C4	C5	C6	C7	C8
2931826.98	4644262.67	2321042.75	5488044.22	2706835.10	2216465.97	4794653.69	3688438.23	4562254.93
6107963.05	6799261.79	4819535.47	7857722.21	4334388.61	4355159.92	7887907.32	6237511.70	6222119.97
1196603.21	1615045.40	853490.17	2366522.66	1071239.54	885005.74	1602570.45	1102194.09	1466181.57
32039.37	37238.14	25196.23	57421.65	21156.23	23423.86	41324.43	30715.89	25575.02
54172.12	40327.29	46900.88	80595.39	26107.39	27221.63	92626.41	33622.86	49952.26
1038001.91	2024549.78	831839.72	2418719.91	1132080.53	780828.10	2308210.91	1169414.70	2088702.30
3338	46097.68	4810.64	34328.34	21787.82	4870.18	21961.15	6400.18	60004.68
895042.05	1598697.38	723632.73	1847391.16	889283.17	722661.73	1824851.15	1176436.27	1594990.03
342799.07	515553.93	278432.72	544126.38	306000.05	262857.43	482286.97	342538.13	551837.18
67961.51	133478.55	52738.22	139171.81	55618.94	51807.73	109277.42	70598.41	119498.71
5798787.27	8059086.50	4555467.38	9716324.11	5020003.20	4375303.43	10089756.69	7387685.31	7927099.60
40937.56	51216.72	32004.35	57944.19	39582.65	25383.81	48967.98	34589.46	41981.76
1620124.22	2158868.38	1309408.27	2621156.64	1303902.13	1049040.90	2400503.56	1755040.65	2148061.47
3620	54577.01	4093.98	38828	19835.49	5004.51	30455.99	5897.51	50205.01
1554517.34	3139945.74	1268156.11	3362744.14	1682163.94	1277218.26	3372867.52	2227322.96	3261706.38
317742.18	537463.75	255404.56	602505	361093.02	254546.10	476872.21	653279.98	582210.65
2113109.59	3190669.46	1840818.88	3913456.50	1795263	1501972.73	3632024.85	2811740.78	3193287.83
2920109.12	3011371.57	2264851.94	3513868.32	2023337.95	2193655.45	3468527.59	2984837.50	2736244.91
4985005.20	8164899.26	4089109.81	9446576.58	4948130.25	3730704.26	9728261.66	6494734.78	8500281.51
4114041.76	3962343.66	3182765.48	4482577.04	2661430.23	2932798.96	4672209.51	4030581.05	3450404.23
2629697.23	4551022.88	2176700.98	5525201.63	2531085.69	1884816.45	4968750.53	3514502.25	4792907.43
0	0	0	0	417	0	11	15	0
136405.87	253500.11	118504.83	383060.81	124503.38	105237.54	346069.52	142529.60	252275.54
193432.19	223401.98	154816.88	246561.82	113622.70	207651.42	256380.31	280992.23	158482.12
1394415.94	1966060.01	1127581.42	2166552.82	1231924.04	1071495.78	2016694.85	1526652.38	1851188.57
21668.75	32601.56	22856.72	49526.29	14661.91	3094.51	57665.44	4972.94	37273.29
824039.44	2768129.98	733148.83	2749262.99	1407620.38	725882.94	3557770.31	1232183.15	3297874.43
430432.26	3885363.63	441151.20	3230367.74	1510076.75	386544.96	2413317.28	551575.92	3885778.67
176100.52	319659.05	158034.64	440671.23	170022.42	72627.77	472120.75	99586	401975.33
7756591.95	12048920.31	6311569.68	12614647.78	7191732.93	6613029.10	14010492.54	9044103.47	12136888.12
1538480.48	2213515.99	1247996.54	2663786.94	1337534.77	1227400.35	2257091.67	1795311.85	2147851.68
349818.64	557825.23	254112.81	807922.86	349364.36	259657.94	619434.80	400066.50	572344.64
1481960.87	2269193.97	1152105.73	2519727.37	1395218.13	1105003.42	2448456.22	1794546.39	2255374.14

37857.14	226730.30	43294.57	213537.98	105728.97	27189.60	144587.24	72961.78	294535.13
970791.74	2658290.27	883300.64	2517196.20	1337563.67	896171.84	2223062.79	1448052.17	2476626.77
848192.77	1267502.43	682319.45	1468926.78	718772.20	564810.50	1542327.36	1007354.61	1269365.74
1604462.74	3712895.86	1357588.69	4479380.99	1924870.34	1255775.98	3663118.69	2572063.27	3673296.43
102815.03	489673.28	98172.31	403862.57	214873.44	89503.69	338134.22	161672.64	524556.30
77000.93	87611.80	64473.32	112413.49	60479.36	48887.98	114778.81	67808.47	102442.15
16876.40	54908.46	21271.54	67872.80	16007.52	44059.79	41660.48	21110.21	43458.86

C9	D1	D10	D11	D12	D13	D14	D15
5427248.04	2761798.11	2228422.87	2073576.37	6923801.36	5772277.23	5294505.40	4468438.79
7054867.24	5226948.02	4449703.34	4190430.97	11278650.30	8290395.98	7022616.09	6615974.93
1867954.72	999830.53	870304.23	814499.66	1802705.96	1916524.16	1762447.99	1559359.01
29097.50	25916.45	23616.10	22417.79	55510.08	47557.55	26477.37	28012.46
38113.22	45122.62	26788.56	23769.36	58295.87	69767.60	55637.38	40391.82
2574242.03	1051920.17	789317.77	716983.01	2082279.51	2409770.25	2450542.82	2033388.43
73818.34	12592.16	2522	2499.66	5280.34	47079.36	65159.02	45240.64
1787449.29	876211.35	717989.15	667946.25	2166324.68	1946113.52	1834347.46	1538628.39
614398.75	328362.63	251130.18	237214.88	582040.62	618418.23	629441.60	487930.64
135268.15	66151.65	52488.40	48817.36	118421.22	151128.05	123837.19	120583.99
9142728.21	5289938.47	4392746.42	4081608.70	13612933.35	9754024.26	9289672.57	8115179.52
58209.13	32675.91	24783.12	23118.81	57751.45	68922.72	53752.21	55274.79
2463898.75	1454299.44	1052008.38	983934.07	3386188.88	2832728.20	2373274.77	2160027.68
64176	10384	2301.20	2687.49	4433.51	50428.02	49997	41437.98
3851161.39	1630086.50	1255620.95	1174469.09	3814088.67	3745102.29	4000467.51	3036552.47
690122.22	310483.66	253110.54	237270.84	847679.43	725113.65	619257.99	567880.88
3643941.05	2077818.83	1503776.53	1397511.84	5441257.48	4231301.06	3652339.34	2931915.37
2979718.57	2461555.05	2241410.76	2116957.10	5148002.94	3747584.55	3049981.53	2937552.25
9507518.49	4880694.14	3650118.04	3384893.09	11823450.79	9980296.70	9556165.53	7643588.38
3778387.57	3374518.86	3025059.63	2862701.58	7128456.47	4995564.39	3821086.97	3769729.91
5481992.69	2650936.09	1851153.74	1704152.11	6934403.27	6639261.78	5485131.74	4485599.27
0	0	0	0	0	0	0	0
273957.55	138814.35	104577.40	96402.28	183566.36	293968	284642.11	226080.07
164570.18	171606.60	222361.94	214200.04	175356.14	246796.21	174453.44	180333.98
2240229.25	1293974.14	1091325.02	1022666.38	2529119.08	2452139.06	2120335.14	1885411.94
48228.67	20669.98	2668.21	1764.69	4050.71	54544.16	52026.37	36248.94
3896318.51	1056940.55	676127.62	618680.94	1473226.19	3346207.37	4201212	2755409.59
5001240.37	954798.41	309444.21	249338.42	594144.08	3691295.22	4212738.22	3178059.36
459261.44	179854.57	71368.45	61241.45	92991.88	443981.97	511255.30	363527.74
14473709.70	7428787.76	6696011.25	6244275.19	14426626.81	14019187.07	13253594.96	11407667.56
2383376.27	1452816.93	1258125.93	1169998.24	2873652.03	2793345.46	2345094.37	2230191.01
644557.58	320191.97	243689.94	218356.16	643866.13	682434.39	676208.28	533855.60
2735621.61	1410412.41	1139740.93	1050102.36	3320500.19	2642919.33	2551615.61	2255531.86

353192	76850.85	15938.37	14250.14	132243.91	280382.70	317359.21	229970.47
3229727	1153552.04	896177.61	835219.74	2268244.71	2829599.43	2843497.11	2310161.86
1458340.50	774119.29	549855.86	510635.94	1716058.21	1652845.39	1585560.75	1271159.85
4467473.01	1800254.79	1238851.92	1122721.95	4733518.90	4401405.46	4134198.22	3453294.87
635880.13	157188.74	70073.44	67529.08	258222.04	532152.80	550499.02	415060.20
124336.98	69668.96	50400.33	46745.95	116197.39	121849.01	121722.91	93079.86
50146.05	31435.77	45356.36	44083.99	5476.38	56081.86	48207.47	43310.31

D16	D17	D18	D19	D2	D20	D3	D4	D5
5040732.09	3675181.14	2272281	2113118.75	6380348.40	2249228.04	8007886.95	3235862.37	7970505.02
8619818.40	6068228.22	4599262.57	4356217.63	8954840.63	4385594.07	13098640.54	4213027.97	10394961.26
1426386.29	1005226.59	887364.65	841116.23	2142684.31	863355.35	2087391.04	1081574.19	3168638.47
42863.51	30285.41	24083.02	23080.21	43776	21907.29	64178.16	17385.67	63920.82
45800.43	32064.41	26402.83	25008.57	66249.76	25966.85	68944.10	17918	104646.39
1532866.67	1049117.34	779464.87	723795.37	2759278.90	819349.42	2398059.91	1602451.94	4264477.22
2928	1119.68	2432.27	1576.14	82392.16	5628.14	6372.68	50202.32	2958.68
1589760.05	1157814.66	734766.51	682273.12	2265608.86	730218.38	2506999.44	1096317.02	2159573.60
448648.72	309723.49	258980.62	243308.41	775820.74	257029.92	679008.34	381781.11	476308.58
91304.55	63049.93	52818.76	49779.51	178078.89	53012.71	138434.09	80310.99	172203.78
9968769.28	7652939.81	4522320.29	4217206.34	11074856.04	4420570.31	15772229.70	5471881.85	13205733.94
45053.76	30988.98	25726.40	24120.65	64018.55	25775.80	66547.05	34850.83	68650.77
2469227.57	1749369.35	1081620.97	1006078.09	3038125.93	1067048.56	3932179.43	1479656.49	3304818.55
3144	1153.01	2286.25	1467.48	79550.51	4555.48	5387.51	41592.99	3269.01
2782973.32	2267049.53	1287393.60	1189297.03	4601929.26	1314546.60	4416288.45	2350153.56	3389832.77
611787.25	880448.05	261527.04	241832.64	782000.98	261153.26	977367.70	440736.92	946167.97
3885609.07	2938134.60	1533165.69	1419744.34	4362139.78	1497644.62	6326961.96	2146493.92	4692249.84
4023857.76	2918559.79	2321122.44	2211370.84	3905327.14	2191431.75	5983678.11	1765226.39	4855106.97
8575050.83	6708043.46	3722236.96	3452121.30	11893564.26	3716345.30	13736516.31	5708483.59	9772352.58
5556904.07	3928710.51	3142482.52	2992262.66	5049446.91	2938603.36	8291604.33	2187099.95	6208967.69
4903841.64	3573238.68	1875560.77	1717534.89	6619455.93	1905149.31	8056894.90	3309511.21	6165410.36
0	6	0	0	2244	0	0	75	0
149079.85	143506.77	103287.56	97856.45	396182.28	105423.43	214499.35	168105.13	266910.08
194943.70	355946.29	231452.40	226904.83	263623.44	210523.51	203252.33	105167.32	220578.09
1948861.90	1492136.49	1113926.38	1054812.05	2662572.28	1085419.68	2934613.67	1351329	3041437.95
2830.82	1496.34	2030.13	1500.85	63907.30	4343.99	4577.21	29735.66	60191.64
1108522.42	1170442.63	684188.75	613941.02	4060207.37	799243.73	1681982.28	2479160.65	2370610.40
381735.37	235179.60	244160.19	195296.59	5201778.39	442124.58	689670.88	3080344.82	3380980.25
75763.01	73434.72	64865.83	59075.28	594418.37	83795.52	110601.77	298463.60	287217.79
11252295.22	8549079.41	6825230.33	6473034.99	15921044.81	6648720.67	16668233.55	8700383.23	19710487.08
2214875.74	1832446.95	1267858.13	1201063.49	2986115.55	1238014.59	3332363.94	1438231.40	4708457.51
476948.50	419557.03	240303.35	224507.04	817037.19	243327.61	749718.08	372505.59	747637.19
2431371.91	1770272.94	1149987.48	1074785.47	3119692.66	1150530.12	3843174.66	1635440.59	4324366.49

81500.77	64734.90	14785.20	9529.23	380036.67	30515.61	153740.86	233984.28	134215.07
1685976.10	1406566	897314.32	836513.65	3431980.96	921639.32	2611296.85	1961982.76	3694207.86
1253283.29	1070806.86	563091.68	520046.68	1854406.91	565169.07	1991037.37	878702.55	1607236.47
3299346.99	2791322.97	1219101.71	1109513.68	4992491.49	1277450.97	5485124.86	2597731.94	6656845.09
177249.53	142884.09	70172.85	59733.01	751134.65	88471.46	299578.26	407419.07	243998.70
89259.46	61908.50	51354.71	48370.63	128775.29	52066.62	134386.67	79824.32	179020
20344.72	2910.18	46589.01	46338.31	52631.41	42494.50	6932.82	30177.66	53689.25

D6	D7	D8	D9
5837235.59	6017283.28	2086672.94	5999616.32
7945811.61	8338683.18	4098161.43	7941101.82
2037273.90	2044536.24	814918.64	2252015.80
34132.87	46029.09	21443	38476.36
54216.76	66178.32	24854.65	58488.36
2732641.90	2783734.80	755014.55	2996555.83
73560.38	74190.02	4436.18	50216.14
2014367.70	2133086.86	677967.95	1866174.36
702688.77	725187.07	239109.88	562687.67
152611.11	178494.37	49422.95	148011.63
10333621.48	10175815.88	4094274	10222307.83
68224.19	60751.59	23219.96	58541.57
2657572.13	2840794.15	988640.86	2688488.67
62239.57	71785.01	3920.01	44749.91
4101158.40	4224357.65	1207892.86	3594731.20
727102.68	751861.49	239192.99	748131.17
3947769.64	4301370.88	1419210.53	3889142.56
3459317.31	3670134.71	2050879.81	3521066.51
10248915.52	11127657.48	3466220.48	9640911.25
4422054.60	4629268.22	2761900.58	4476849.44
5931972.06	6415805.62	1767412.09	5710344.40
0	0	0	0
286674.04	322567.37	100180.66	270721.48
201307.55	226640.54	200170.61	182379.70
2400383.13	2477575.17	1014498.56	2414825.49
52978.94	58979.59	3470.48	50776.62
4391925.79	3921280.87	697569.36	3347425.93
4877011.74	5220914.16	390257.25	4536312.77
493721.66	505681.01	76775.73	410467.27
16235723.53	16267317.44	6223270.68	15474603.88
2681304.15	2827337.57	1164744.09	3051830.80
716365.02	722675.80	233527.17	708208.96
2903868.28	2942401.07	1065087.21	3153357.79

363550.61	377275.19	23706.71	282266.08
3259431.75	3346365.89	860960.97	3240061
1629863.13	1702219.61	522722.71	1472400.33
4608913.36	4897754.52	1183671.95	5070756.77
623007.50	685890.60	78586.07	489447.24
127732.11	129126.91	47528.99	133893.40
57501.16	61929.62	41096.07	61649.18
