# Supplementary material for: Bacterial Colonization in the Airways and Intestines of Twin and Singleton Preterm Neonates: A Single-Center Study
Source: Can J Infect Dis Med Microbiol. 2023 Aug 1;2023:2973605. doi: 10.1155/2023/2973605 (PMC10409585; doi:10.1155/2023/2973605)
Supplement: Supplementary Materials — Supplementary Material 1: sequence information identified in singleton and twin preterm neonates. Supplementary Material 2: abundance of bacterial taxa and corresponding 16SrRNA gene sequences in singleton and twin preterm neonates. Supplementary Material 3: class-, order-, family-, and species-level differences were determined using the Kruskal–Wallis test. Supplementary Material 4: three levels of annotation in the KEGG database on metabolic pathways. [file 2973605.f1.zip › Supplementary Material 4 KEGG_level3.pdf]

Sample gene sequences were annotated using the KEGG database on metabolic pathways based on their metabolic function prediction using PICRUSt2.

KEGGLevel3
Vasopressin-regulated water reabsorption
Transcription machinery
DNA repair and recombination proteins
Pantothenate and CoA biosynthesis
Primary immunodeficiency
NOD-like receptor signaling pathway
Drug metabolism - other enzymes
Phototransduction
Transporters
Biosynthesis of ansamycins
Biosynthesis of unsaturated fatty acids
Citrate cycle (TCA cycle)
Influenza A
Xylene degradation
Systemic lupus erythematosus
Prostate cancer
Starch and sucrose metabolism
Glycosyltransferases
Drug metabolism - cytochrome P450
Bacterial invasion of epithelial cells
Sulfur metabolism
Olfactory transduction
One carbon pool by folate
Other ion-coupled transporters
Bisphenol degradation
Glycosphingolipid biosynthesis - lacto and neolacto series
Glycerolipid metabolism
Tropane, piperidine and pyridine alkaloid biosynthesis
Lipid metabolism
Histidine metabolism
Benzoate degradation
G protein-coupled receptors
Renin-angiotensin system

Tryptophan metabolism
Gastric acid secretion
Secondary bile acid biosynthesis
D-Arginine and D-ornithine metabolism
Nitrotoluene degradation
Other glycan degradation
Steroid hormone biosynthesis
Peptidases
Transcription factors
Salivary secretion
Electron transfer carriers
Stilbenoid, diarylheptanoid and gingerol biosynthesis
Viral myocarditis
mRNA surveillance pathway
Other transporters
Biotin metabolism
Prenyltransferases
D-Glutamine and D-glutamate metabolism
Leishmaniasis
Proteasome
Melanogenesis
Photosynthesis - antenna proteins
Toluene degradation
Chaperones and folding catalysts
Protein processing in endoplasmic reticulum
Valine, leucine and isoleucine biosynthesis
Phenylpropanoid biosynthesis
Folate biosynthesis
Glycosphingolipid biosynthesis - ganglio series
Cell motility and secretion
Pathways in cancer
Transcription related proteins
Phosphatidylinositol signaling system
p53 signaling pathway

GnRH signaling pathway
C5-Branched dibasic acid metabolism
Lysine biosynthesis
Pertussis
Butirosin and neomycin biosynthesis
Type II diabetes mellitus
Ascorbate and aldarate metabolism
Apoptosis
Oocyte meiosis
Neuroactive ligand-receptor interaction
Butanoate metabolism
Styrene degradation
Alzheimer's disease
Carbon fixation in photosynthetic organisms
Vibrio cholerae pathogenic cycle
Glyoxylate and dicarboxylate metabolism
Secretion system
Insulin signaling pathway
Methane metabolism
Aminobenzoate degradation
Retinol metabolism
Zeatin biosynthesis
Pores ion channels
Isoflavonoid biosynthesis
Fc gamma R-mediated phagocytosis
ABC transporters
Fluorobenzoate degradation
RIG-I-like receptor signaling pathway
Renal cell carcinoma
Lysine degradation
Germination
Ribosome
Pancreatic secretion
Amino acid metabolism

Protein export
Hypertrophic cardiomyopathy (HCM)
DNA replication proteins
Betalain biosynthesis
Notch signaling pathway
Photosynthesis proteins
Aldosterone-regulated sodium reabsorption
Primary bile acid biosynthesis
Colorectal cancer
Cellular antigens
Bile secretion
Protein digestion and absorption
Chloroalkane and chloroalkene degradation
Protein folding and associated processing
Energy metabolism
Oxidative phosphorylation
African trypanosomiasis
Amino sugar and nucleotide sugar metabolism
Nitrogen metabolism
Restriction enzyme
Limonene and pinene degradation
General function prediction only
Purine metabolism
Amoebiasis
Bladder cancer
Biosynthesis of type II polyketide products
Staphylococcus aureus infection
Pentose and glucuronate interconversions
Spliceosome
Ethylbenzene degradation
Ubiquinone and other terpenoid-quinone biosynthesis
Basal transcription factors
Endocytosis
Sphingolipid metabolism

Glutathione metabolism
Hematopoietic cell lineage
Chagas disease (American trypanosomiasis)
Cytochrome P450
Linoleic acid metabolism
Base excision repair
Homologous recombination
Plant-pathogen interaction
Glycine, serine and threonine metabolism
Fatty acid biosynthesis
Inositol phosphate metabolism
Chromosome
Various types of N-glycan biosynthesis
Lipoic acid metabolism
N-Glycan biosynthesis
Amino acid related enzymes
Phosphonate and phosphinate metabolism
Nicotinate and nicotinamide metabolism
Pyruvate metabolism
Tuberculosis
Non-homologous end-joining
Peroxisome
Pyrimidine metabolism
Flagellar assembly
Glycosphingolipid biosynthesis - globo series
Metabolism of xenobiotics by cytochrome P450
Huntington's disease
Ion channels
Glycerophospholipid metabolism
Lipopolysaccharide biosynthesis proteins
PPAR signaling pathway
Sesquiterpenoid biosynthesis
Tyrosine metabolism
Glutamatergic synapse

Vascular smooth muscle contraction
alpha-Linolenic acid metabolism
Penicillin and cephalosporin biosynthesis
Mineral absorption
Chlorocyclohexane and chlorobenzene degradation
Sulfur relay system
Polyketide sugar unit biosynthesis
beta-Alanine metabolism
Cysteine and methionine metabolism
Ubiquitin system
Pentose phosphate pathway
Thiamine metabolism
Riboflavin metabolism
Bacterial toxins
Proximal tubule bicarbonate reclamation
Atrazine degradation
Biosynthesis of vancomycin group antibiotics
Prion diseases
Arginine and proline metabolism
Cardiac muscle contraction
Signal transduction mechanisms
Caprolactam degradation
Alanine, aspartate and glutamate metabolism
Phenylalanine, tyrosine and tryptophan biosynthesis
Glycosaminoglycan degradation
Caffeine metabolism
Others
Two-component system
Parkinson's disease
Taurine and hypotaurine metabolism
Ribosome biogenesis in eukaryotes
Carotenoid biosynthesis
Ribosome Biogenesis
Nucleotide metabolism

Aminoacyl-tRNA biosynthesis
RNA polymerase
Biosynthesis of siderophore group nonribosomal peptides
RNA transport
Toxoplasmosis
Ether lipid metabolism
Epithelial cell signaling in Helicobacter pylori infection
DNA replication
Cytoskeleton proteins
Lipid biosynthesis proteins
Biosynthesis of type II polyketide backbone
Membrane and intracellular structural molecules
Carbohydrate digestion and absorption
Meiosis - yeast
Cyanoamino acid metabolism
Chronic myeloid leukemia
Glioma
beta-Lactam resistance
Synthesis and degradation of ketone bodies
Adipocytokine signaling pathway
Neurotrophin signaling pathway
Novobiocin biosynthesis
Wnt signaling pathway
Biosynthesis and biodegradation of secondary metabolites
Selenocompound metabolism
Cell division
Isoquinoline alkaloid biosynthesis
Progesterone-mediated oocyte maturation
Arachidonic acid metabolism
Bacterial chemotaxis
Nucleotide excision repair
Geraniol degradation
Lipopolysaccharide biosynthesis
Mismatch repair

Sporulation
Flavonoid biosynthesis
Small cell lung cancer
Endocrine and other factor-regulated calcium reabsorption
Translation proteins
Flavone and flavonol biosynthesis
Peptidoglycan biosynthesis
Inorganic ion transport and metabolism
RNA degradation
Galactose metabolism
Indole alkaloid biosynthesis
Amyotrophic lateral sclerosis (ALS)
Glycan biosynthesis and metabolism
Cell cycle - Caulobacter
Antigen processing and presentation
Carbon fixation pathways in prokaryotes
Protein kinases
Metabolism of cofactors and vitamins
Function unknown
Dioxin degradation
D-Alanine metabolism
Translation factors
Steroid biosynthesis
Type I diabetes mellitus
Polycyclic aromatic hydrocarbon degradation
Bacterial motility proteins
Vibrio cholerae infection
Fatty acid metabolism
Terpenoid backbone biosynthesis
Long-term potentiation
Fatty acid elongation in mitochondria
Porphyrin and chlorophyll metabolism
Phototransduction - fly
Cholinergic synapse

Vitamin B6 metabolism
Photosynthesis
Fructose and mannose metabolism
Shigellosis
Streptomycin biosynthesis
Glycolysis / Gluconeogenesis
Replication, recombination and repair proteins
Phenylalanine metabolism
Bacterial secretion system
Carbohydrate metabolism
Phosphotransferase system (PTS)
Lysosome
VEGF signaling pathway
Valine, leucine and isoleucine degradation
Tetracycline biosynthesis
Naphthalene degradation
Biosynthesis of 12-, 14- and 16-membered macrolides
ErbB signaling pathway
Propanoate metabolism
Calcium signaling pathway
MAPK signaling pathway - yeast

description	A1	A10	A11
Vasopressin-regulated water reabsorption	0	0	0
Transcription machinery	1362324.66	1054213.06	786408.33
DNA repair and recombination proteins	5013687.86	3625689.87	3835134.36
Pantothenate and CoA biosynthesis	962729.61	718600.21	606141.06
Primary immunodeficiency	114033.24	78037.28	103653.46
NOD-like receptor signaling pathway	54224.65	49342.61	5941.23
Drug metabolism - other enzymes	316430.13	212517.69	309013.30
Phototransduction	0	0	0
Transporters	9333113.97	6079954.70	11373606.80
Biosynthesis of ansamycins	76222.08	55767.98	54112.25
Biosynthesis of unsaturated fatty acids	483450.77	403545.36	143407.30
Citrate cycle (TCA cycle)	1448594.27	1152211.16	610211.04
Influenza A	68027	58369.08	7018
Xylene degradation	52792.47	33930.61	50928.23
Systemic lupus erythematosus	82	86.27	35
Prostate cancer	54215.65	49339.94	5941.23
Starch and sucrose metabolism	696453.45	342288.09	1269528.16
Glycosyltransferases	447760.01	315536.28	322178.60
Drug metabolism - cytochrome P450	543858.92	468529.22	102834.61
Bacterial invasion of epithelial cells	5035.64	15	185
Sulfur metabolism	689047.80	527553.53	440454.35
Olfactory transduction	0	0	4
One carbon pool by folate	821533.08	614262.40	499035.22
Other ion-coupled transporters	2816847.28	2262411.58	1175464.28
Bisphenol degradation	155700.04	135935.29	16489.83
Glycosphingolipid biosynthesis - lacto and neolacto series	0	0	0
Glycerolipid metabolism	593975.07	414228.83	521408.86
Tropane, piperidine and pyridine alkaloid biosynthesis	236941.86	207472.44	24434.91
Lipid metabolism	253051.11	204029.45	165893.44
Histidine metabolism	882112.67	731399.78	180526.82
Benzoate degradation	1127401.04	938489.10	300254.98
G protein-coupled receptors	0	0	0
Renin-angiotensin system	34041.50	29626	3716

Tryptophan metabolism	928556.68	783704.60	230011.23
Gastric acid secretion	0	0	4
Secondary bile acid biosynthesis	25767.17	5329	95132
D-Arginine and D-ornithine metabolism	8385.06	7350.27	988.50
Nitrotoluene degradation	84311.24	78565.69	9484.56
Other glycan degradation	44746	22604.96	97221.96
Steroid hormone biosynthesis	27067.65	19754.67	2010.33
Peptidases	2988124.51	2220431.36	1964650.84
Transcription factors	3064351.69	2146842.74	2807595.37
Salivary secretion	0	0	0
Electron transfer carriers	2587.61	1439.35	448.79
Stilbenoid, diarylheptanoid and gingerol biosynthesis	1451	1556.81	189
Viral myocarditis	68027	58369.08	7018
mRNA surveillance pathway	0	0	0
Other transporters	552265.19	442375.82	241737.88
Biotin metabolism	294920.40	244227.23	76575.38
Prenyltransferases	399608.47	279029	364117.38
D-Glutamine and D-glutamate metabolism	284145.80	214591.71	167147.75
Leishmaniasis	10	0	0
Proteasome	55756.65	49498.94	5945.23
Melanogenesis	99.50	24	18.50
Photosynthesis - antenna proteins	0	0	0
Toluene degradation	375956.92	317937.86	85378.90
Chaperones and folding catalysts	2322445.69	1828637.85	1164818.98
Protein processing in endoplasmic reticulum	82585.09	65004.94	55029.73
Valine, leucine and isoleucine biosynthesis	1184426.65	898104.06	626449.02
Phenylpropanoid biosynthesis	112285.62	78346.61	103343.46
Folate biosynthesis	750696.17	620598.86	169396.68
Glycosphingolipid biosynthesis - ganglio series	20271.50	8449.67	48234.73
Cell motility and secretion	692883.83	584572.33	212492.90
Pathways in cancer	176356.11	154517.23	18593.46
Transcription related proteins	6202.67	3610.01	510.19
Phosphatidylinositol signaling system	208702.49	163167.37	114017.92
p53 signaling pathway	68027	58369.08	7018

GnRH signaling pathway	10	0	4
C5-Branched dibasic acid metabolism	455429.42	357818.92	183992.49
Lysine biosynthesis	1004323.77	726108.04	748404.41
Pertussis	135797	122432.18	15695.83
Butirosin and neomycin biosynthesis	44141.34	31548.61	50775.23
Type II diabetes mellitus	46669.21	30459.55	50649.46
Ascorbate and aldarate metabolism	281704.61	195891.36	304086.65
Apoptosis	109082.25	95913.02	11380.33
Oocyte meiosis	0	0	4
Neuroactive ligand-receptor interaction	0	0	0
Butanoate metabolism	1647818.75	1326984.56	626552.19
Styrene degradation	177845.92	153689.50	64967.56
Alzheimer's disease	266378.80	210799.90	119369.06
Carbon fixation in photosynthetic organisms	803153.04	579170.86	636502.09
Vibrio cholerae pathogenic cycle	201736.88	154733.96	114102.38
Glyoxylate and dicarboxylate metabolism	1494879.07	1266345.39	428087.96
Secretion system	4250410.53	3491117.61	1412456.27
Insulin signaling pathway	119572.69	85490.22	104935.69
Methane metabolism	1587303.76	1187776.27	1225688.59
Aminobenzoate degradation	509515.35	421662.10	189802.01
Retinol metabolism	134702	104848.90	60322.46
Zeatin biosynthesis	67425.26	47907.61	53038.23
Pores ion channels	1196978.11	1012219.15	264905.57
Isoflavonoid biosynthesis	1722	1961	237
Fc gamma R-mediated phagocytosis	10	0	0
ABC transporters	5298480.90	4004222.74	3946492.67
Fluorobenzoate degradation	216199.80	188388.75	23664.23
RIG-I-like receptor signaling pathway	1001.50	1483.94	105
Renal cell carcinoma	54103.46	46808.21	5630.23
Lysine degradation	684996.96	580677.83	113510.04
Germination	5272.52	7718	782.50
Ribosome	3685604.40	2601931.27	3007649.55
Pancreatic secretion	0	0	0
Amino acid metabolism	167060.77	126531.71	109412.84

Protein export	1056921.65	775394.42	707901.64
Hypertrophic cardiomyopathy (HCM)	10303	8598	963
DNA replication proteins	1778399.77	1260569.25	1427441.74
Betalain biosynthesis	109.50	80.27	45.50
Notch signaling pathway	0	0	4
Photosynthesis proteins	599410.03	438328.71	430501.07
Aldosterone-regulated sodium reabsorption	0	0	0
Primary bile acid biosynthesis	32958.67	10816.27	95689
Colorectal cancer	68027	58369.08	7018
Cellular antigens	125415.76	114624.69	12395.96
Bile secretion	2.50	8.27	0
Protein digestion and absorption	7185.50	7485	874
Chloroalkane and chloroalkene degradation	383898.27	304829.02	178146.69
Protein folding and associated processing	1714437.53	1366273.52	731582.46
Energy metabolism	1483108.38	1158225.68	847249.54
Oxidative phosphorylation	2519304.79	2023795.73	1185790.03
African trypanosomiasis	40712.90	34523.94	4239.73
Amino sugar and nucleotide sugar metabolism	1593624.89	878096.79	2517385.23
Nitrogen metabolism	1463542.62	1145027.78	657080.85
Restriction enzyme	312685.52	236670.03	171809.50
Limonene and pinene degradation	285977.08	246091.92	28297.69
General function prediction only	7304557.89	5533195.18	4632688.25
Purine metabolism	3784886.66	2766013.24	2787657.95
Amoebiasis	21076.39	20448.27	2033
Bladder cancer	4537.40	5549.94	535.23
Biosynthesis of type II polyketide products	69	0	12
Staphylococcus aureus infection	89501.15	5764.35	284294
Pentose and glucuronate interconversions	467234.45	276195.54	693107.15
Spliceosome	0	0	7
Ethylbenzene degradation	168456.75	142932.03	18139.19
Ubiquinone and other terpenoid-quinone biosynthesis	543777.66	400556.03	427830.24
Basal transcription factors	156.50	128	14
Endocytosis	10	0	0
Sphingolipid metabolism	38434.52	14486.22	96052.06

Glutathione metabolism	1108355.14	908820.79	437268.10
Hematopoietic cell lineage	44	30	0
Chagas disease (American trypanosomiasis)	27290.90	22099.94	2431.23
Cytochrome P450	44	30	0
Linoleic acid metabolism	119355.14	105954.21	13514.23
Base excision repair	757991.33	541733.06	630902.99
Homologous recombination	1475582.93	1069503.70	1074866.31
Plant-pathogen interaction	321151.36	260198.86	172211.34
Glycine, serine and threonine metabolism	1522842.25	1190450.79	755445.92
Fatty acid biosynthesis	991332.95	739320.06	700999.47
Inositol phosphate metabolism	218570.79	168931.14	114388.73
Chromosome	2994188.63	2269118.89	1880082.70
Various types of N-glycan biosynthesis	0	0	0
Lipoic acid metabolism	131605.87	94346.22	105931.19
N-Glycan biosynthesis	6482.14	6001.94	879
Amino acid related enzymes	2398526.54	1789870.32	1539711.87
Phosphonate and phosphinate metabolism	125705.18	90827.43	105407.02
Nicotinate and nicotinamide metabolism	801012.22	632806.67	407688.64
Pyruvate metabolism	2158367.72	1596343.26	1510478.21
Tuberculosis	299247.70	218254.58	215602.42
Non-homologous end-joining	26120	22695.54	2642.50
Peroxisome	572865.48	485526.75	151216.94
Pyrimidine metabolism	2680072.42	1878447.68	2257131.79
Flagellar assembly	1199864.31	1080933.43	118235.28
Glycosphingolipid biosynthesis - globo series	42842.92	10989.67	142996.73
Metabolism of xenobiotics by cytochrome P450	537020.70	462904.22	102301.61
Huntington's disease	319165.25	269849.84	78899.29
Ion channels	26506.44	12323.35	48704.96
Glycerophospholipid metabolism	987129.75	757698.57	609071.29
Lipopolysaccharide biosynthesis proteins	934371.70	792367.91	192774.77
PPAR signaling pathway	448262.42	377854.11	92050.59
Sesquiterpenoid biosynthesis	0	0	0
Tyrosine metabolism	736087.12	625090.03	169787.17
Glutamatergic synapse	85528	64888.36	54864.96

Vascular smooth muscle contraction	0	0	0
alpha-Linolenic acid metabolism	111364.81	95471.01	11346.69
Penicillin and cephalosporin biosynthesis	51891.90	45245.01	5392.23
Mineral absorption	69534.19	53365.54	53362.50
Chlorocyclohexane and chlorobenzene degradation	153279.77	126401.56	62255.23
Sulfur relay system	607751.77	479921.04	247882.41
Polyketide sugar unit biosynthesis	171355.75	111176.76	201331.12
beta-Alanine metabolism	600186.17	515637.56	59371.07
Cysteine and methionine metabolism	1487352.68	1092209.27	1074932.44
Ubiquitin system	34333.32	27590.94	3728.73
Pentose phosphate pathway	1030835.81	678873.08	1213880.75
Thiamine metabolism	566486.94	429669.78	334864.99
Riboflavin metabolism	400092.65	316108.80	227157.50
Bacterial toxins	182714.72	112399.90	201589.73
Proximal tubule bicarbonate reclamation	88077.77	76860.61	9031.56
Atrazine degradation	107660.17	95164.75	11213.23
Biosynthesis of vancomycin group antibiotics	44768.20	28453.94	50445.23
Prion diseases	58024.62	51770.94	6110.73
Arginine and proline metabolism	1803630.05	1387809.39	1108627.83
Cardiac muscle contraction	92341.50	80955.54	8786
Signal transduction mechanisms	842229.15	647453.67	502349.99
Caprolactam degradation	201011.64	172739.19	19659.02
Alanine, aspartate and glutamate metabolism	1489717.49	1118615.97	937686.10
Phenylalanine, tyrosine and tryptophan biosynthesis	1330483.22	1043512.38	550902.08
Glycosaminoglycan degradation	20408.40	8454.67	48263.73
Caffeine metabolism	899	1460.27	109
Others	1347648.28	977447.12	967494.64
Two-component system	4770440.43	3922271.79	1922004.82
Parkinson's disease	160368.50	139324.62	15820
Taurine and hypotaurine metabolism	245865.75	177471.85	162487.15
Ribosome biogenesis in eukaryotes	121562.91	95179.28	58848.36
Carotenoid biosynthesis	50552.35	43043.16	4252
Ribosome Biogenesis	2776632.22	2035261.17	1994893.88
Nucleotide metabolism	114230.52	76634.70	103131.21

Aminoacyl-tRNA biosynthesis	1770968.69	1266795.77	1381067.44
RNA polymerase	281922.48	191861.11	259418.92
Biosynthesis of siderophore group nonribosomal peptides	57262.04	41667.02	52108.88
RNA transport	103383.58	69233.68	103133.96
Toxoplasmosis	68027	58369.08	7018
Ether lipid metabolism	13168.17	11111	1282
Epithelial cell signaling in Helicobacter pylori infection	126236.41	99394.55	59094.46
DNA replication	1043887.53	738567.25	844376.90
Cytoskeleton proteins	459004.09	366814.55	186739.75
Lipid biosynthesis proteins	1515121.16	1203000.80	707687.31
Biosynthesis of type II polyketide backbone	10	0	0
Membrane and intracellular structural molecules	1739109.89	1489408.80	368465.95
Carbohydrate digestion and absorption	2296.07	287.01	132.56
Meiosis - yeast	33497.50	31401.35	3316
Cyanoamino acid metabolism	352061.38	260728.61	265383.44
Chronic myeloid leukemia	0	0	4
Glioma	0	0	4
beta-Lactam resistance	37751.25	29528.34	3772.23
Synthesis and degradation of ketone bodies	471838.48	393720.33	138680.66
Adipocytokine signaling pathway	148208.23	128843.88	14819.46
Neurotrophin signaling pathway	0	0	4
Novobiocin biosynthesis	209794.38	172404.10	67696.25
Wnt signaling pathway	0	0	8
Biosynthesis and biodegradation of secondary metabolites	170770.71	135863.65	62595.44
Selenocompound metabolism	632464.64	454209.65	525290.18
Cell division	205597.51	170785.49	67192.42
Isoquinoline alkaloid biosynthesis	88933.81	78819.15	9509.46
Progesterone-mediated oocyte maturation	54215.65	49339.94	5941.23
Arachidonic acid metabolism	161271.97	134615.57	62541.19
Bacterial chemotaxis	1031930.17	945812.51	104546.63
Nucleotide excision repair	588732.37	406039.29	567958.84
Geraniol degradation	672654.85	587065.59	69742.15
Lipopolysaccharide biosynthesis	693356.23	612743.71	75566.66
Mismatch repair	1211280.06	849488.41	1047264.72

Sporulation	62047.65	51386.94	52908.50
Flavonoid biosynthesis	22951.50	20509.27	2684.50
Small cell lung cancer	68037	58369.08	7018
Endocrine and other factor-regulated calcium reabsorption	0	0	0
Translation proteins	1673611.09	1261870.17	1049507.49
Flavone and flavonol biosynthesis	110.90	5	17
Peptidoglycan biosynthesis	1297079.38	905682.35	1053919.52
Inorganic ion transport and metabolism	742449.61	619327.88	262029.99
RNA degradation	872275.71	643380.46	597205.45
Galactose metabolism	470238.20	212570.89	971154.65
Indole alkaloid biosynthesis	0	56.27	31
Amyotrophic lateral sclerosis (ALS)	193229.94	172624.50	20276.96
Glycan biosynthesis and metabolism	163008.72	144612.23	17057.01
Cell cycle - Caulobacter	904696.01	670299.65	598828.36
Antigen processing and presentation	54215.65	49339.94	5941.23
Carbon fixation pathways in prokaryotes	2015902.16	1621322.10	759173.06
Protein kinases	1010858.30	808175.47	475286.94
Metabolism of cofactors and vitamins	347852.28	283791.14	127694.46
Function unknown	3708254.93	2953618.59	1727731.38
Dioxin degradation	80683.06	46938.49	99599.96
D-Alanine metabolism	200352.32	130981.84	205209.42
Translation factors	847537.26	613618.60	640840.18
Steroid biosynthesis	1297.50	1604.27	175.50
Type I diabetes mellitus	68428.56	48159.61	53217.73
Polycyclic aromatic hydrocarbon degradation	19919	19292.50	1968.16
Bacterial motility proteins	3580336.87	3210514.23	414879.62
Vibrio cholerae infection	9888	8240	818
Fatty acid metabolism	1260006.87	1054759.90	264398.32
Terpenoid backbone biosynthesis	973717.67	707639.28	745075.96
Long-term potentiation	0	0	4
Fatty acid elongation in mitochondria	0	0	0
Porphyrin and chlorophyll metabolism	1290023.27	1113049.51	176753.64
Phototransduction - fly	0	0	4
Cholinergic synapse	0	0	4

Vitamin B6 metabolism	382213.06	288933.54	224537.40
Photosynthesis	545918.41	388118.15	424918.34
Fructose and mannose metabolism	893754.40	380243.33	1984881.58
Shigellosis	276	0	48
Streptomycin biosynthesis	310366.86	204698.68	354167.60
Glycolysis / Gluconeogenesis	1674780.08	1088619.10	1877611.70
Replication, recombination and repair proteins	1446650.01	1032186	1213393.22
Phenylalanine metabolism	652750.60	555452.44	158437.56
Bacterial secretion system	1766276.73	1438264.14	598245.90
Carbohydrate metabolism	81192.41	41208.93	147081.67
Phosphotransferase system (PTS)	855173.14	136233.20	3088578.49
Lysosome	33304.40	22169.28	49848.23
VEGF signaling pathway	10	0	0
Valine, leucine and isoleucine degradation	1631881.88	1359815.16	390692.57
Tetracycline biosynthesis	267590.64	191056.71	212052.42
Naphthalene degradation	511667.01	428553.15	145863.19
Biosynthesis of 12-, 14- and 16-membered macrolides	0	0	0
ErbB signaling pathway	0	0	4
Propanoate metabolism	1565694.27	1252305.98	617314.11
Calcium signaling pathway	0	0	8
MAPK signaling pathway - yeast	113367.94	71723.82	150128.96

A12	A13	A14	A15	A16	A17	A18	A19	A2
0	0	0	0	0	0	0	0	0
1021888.83	1197132.38	721326.62	663988.63	745050.13	1281616.39	791644.65	796923.54	609024.90
3515784.72	4129910.58	2542543.73	2447932.58	2848244.98	4431985.48	2755761.29	2781986.53	2447593.32
693358.78	824962.50	503659.12	470652.66	535138.41	875239.94	545254.09	553404.17	485029.52
75860.64	87188.38	53128.71	55530.17	65824.37	95498.58	58853.89	60625.80	52116.24
45606.25	55708.56	32232.67	29923.82	29017.02	60460.41	38547.75	39151.74	27910.32
203807.98	245213.82	148954.45	158083.96	187803.66	264053.30	159357.88	162762.30	200684.32
0	0	0	0	3	0	0	0	0
6127124.02	6902570.68	4335454.93	4514048.31	5768054.13	7127328.57	4420866.22	4433968.67	7337656.72
53485.82	68148.53	40435.71	40999.70	44252.37	70548.91	42889.71	43462.56	80133.68
379502.25	460133.97	265294.19	242388.16	254300.06	498922.08	308609.07	316833.05	245189.98
1109805.41	1312759.44	795218.94	708056.29	776124.55	1401984.37	875142.84	884207.53	600482.66
58450.66	73311.68	44343.66	31623.84	32286.66	68762.48	44586.34	42304.56	12383.32
35730.42	36943.37	23911.11	21360.08	26514.44	42114.24	21850.83	22075.82	24497.36
40	216	62	36	39	209	75	56.18	10
45606.25	55532.39	32060.67	29358.15	28989.02	60456.74	38547.75	39148.07	27905.32
333947.38	407199.84	251360.21	328368.61	460988.26	453714.05	243830.75	250213.39	616363.64
302689.90	369092.11	226011.01	219613.43	247534.66	393964.49	237984.19	241999.30	424295.44
445728.16	524573.81	304711.37	271520.62	280409.98	572185.38	355860.52	366296.66	226549.28
105.12	196.19	1227.11	836.79	126.85	416	55	200	16831.30
507815.56	602799.02	356811.79	343309.83	391375.06	641750.88	397919.27	407541.43	424337.58
0	0	0	0	3	0	0	0	0
594486.46	702660.49	430887.98	399310.62	448653.79	755084.09	468372.64	472248.39	360633.94
2113360.21	2580488.30	1520078.06	1458815.54	1573554.75	2797176.82	1773679.76	1814994.73	1558001.52
129180.04	151011.01	89049.56	78416.89	79196.91	166959.36	105270.23	107525.51	31843.86
0	0	0	0	0	0	0	0	0
404453.71	472575.39	285063.35	285876.57	345943.25	505440.89	311508.44	314490.87	313507.32
200892.74	233126.83	138260.29	118847.04	119704.49	251676.99	155665.26	157804.56	130800.60
188874.72	227364.86	132270.05	133027.45	149692.19	249980.36	152026.91	157745.32	279528.68
712220.80	823454.15	492401.57	414139.67	441993.38	886042.03	547072.66	553226.46	435602.80
907639.81	1056303.23	616099.20	550100.10	579222.44	1135697.36	715618.61	728017.08	335321.38
0	13.50	0	0	0	0	0	0	0
26640	33390	18466.50	17572	17692	38093.67	23607	24546	7201.50

783133.13	870769.89	522740.51	432059.69	466496.04	925783.58	568199.62	576769.04	360971.30
0	0	0	0	3	0	0	0	0
4532	5733	3131.17	12189.93	23777.50	7567.87	4236	4383.50	1272.22
7240.31	9752.67	5661.67	4316.43	5110	8849.53	5724.89	5402.18	1480.66
72611.75	91345.09	51007.60	44602.62	44490.91	93966.07	59197.31	58303.38	66617.28
20829.29	28926.14	16243.30	26178.90	33319.46	34101.30	16294.66	17017.39	38857.96
24083	22563.17	15342.33	8214.17	8979.92	23483.53	12158.83	11840.92	4798.36
2168602.06	2523658.96	1545774.57	1426972.25	1635543.64	2713261.41	1658920.47	1666791	1694750.78
2090398.05	2440285.18	1482875.54	1486310.60	1790187.72	2612176.61	1613718.16	1622255.53	2231176.70
0	0	0	0	3	0	0	0	0
1481.62	3215.37	1710.37	4330.31	1525.05	3050.53	869.02	857.95	50947.76
1162.20	2020	1852	1164	1017	3194.60	776	1334.54	453
58450.66	73311.68	44343.66	31620.34	32286.66	68762.48	44586.34	42304.56	12383.32
0	0	0	0	0	0	0	0	0
425947.89	501698.61	298589.40	268326.16	292107.51	541901.89	333345.59	337185.35	302363.56
235228.72	278189.14	166020.81	144952.98	151596.04	298836.62	186601.78	187805.45	173375.10
271234.53	323516.75	197901.22	195539.92	233360.22	341523.03	211226.67	213057.16	148659.80
207965.19	243686.02	148087.56	139498.24	155602.65	259135.68	162615.81	165872.81	134395.38
0	0	7	0	0	134	0	0	0
45895.25	55887.39	35668.67	29387.65	29063.02	61640.74	38562.75	39268.07	27998.32
31.83	61.50	12.83	50.67	11.33	51.33	19.17	64.17	7.16
0	24	492	0	0	0	0	108	0
306407.14	359000.55	215074.29	186488.69	194602.48	385866.63	242575.58	246410.28	190926.56
1759302.82	2074589.27	1254056.24	1154828.91	1265079.86	2226416.94	1404411.37	1413573.08	1137870.30
61663.89	73510.90	42903.50	40801.82	46695.85	81129.54	49332.56	49260.24	30960.66
875033.64	1024282.98	633660.49	557981.96	631474.53	1088599.94	673512.74	681174.66	574918.68
80089.26	89660.63	51949.79	49038.99	61758.12	99587.08	54966.06	56133.88	51830.78
592311.75	708564.77	431151.03	371365.58	387707.99	760804.05	474604.75	480881.38	394125.34
8376.25	10333.05	5766.84	9709.15	14550.27	10984.14	5890.83	6038.64	18713.74
556843.39	658168.38	390459.03	346815.75	367848.32	708514.19	451030.85	453753.87	299177.66
149127.30	182121.46	108828.50	87523.14	88890.28	186670.23	118942.15	117696.80	67854.92
4662.92	4938.73	3208.06	2798.32	2015.91	4884.59	2014.83	2030.25	51298.72
155338.40	187015.95	110625.65	104425.96	115699.76	199189.08	124676.28	126221.05	104239.98
58450.66	73311.68	44343.66	31620.34	32286.66	68762.48	44586.34	42304.56	12383.32

0	0	7	0	3	134	0	0	0
351990.33	409273.30	254689.68	214206.59	237743.12	430600.81	266919.44	269065.23	282579.98
700932.70	831651	505728.69	486439.33	563888.01	888817.85	550872.78	555864.56	469273.88
109823.18	138654.12	77652.75	77051.52	74112.92	153739.65	96867.66	100463.35	195851.56
29276.06	34383.86	19390.44	21596.75	27729.27	38945.68	21650.64	22518.49	23306.74
31466.24	35323.80	23140.73	21930.90	27285.79	36025.98	21156.53	21200.56	40248.98
208062.48	214102.54	133013.26	131085.13	166046.43	220278.33	132118.49	133484.13	243381.34
93247.02	114695.85	67869.15	54552.35	54543.99	113754.24	73595.51	72621.75	21224.86
0	0	0	0	3	0	0	0	0
0	0	0	3.50	0	0	0	0	0
1305855.12	1498154.83	907575.84	782095.31	853706.10	1587029.86	985107.03	994756.40	720918.46
145777.07	172536.55	97668.95	92619.61	98599.68	182482.74	113694.08	118643.94	35010.94
210139.92	246794.05	152505.49	124215.25	138355	251710.65	158838.30	155137.18	63064.30
560181.23	673021.94	405048.37	403000.82	463162.57	712100.71	438794.15	444748.07	503934.34
148248.55	183041.21	113274.61	96496.49	109047.94	193077.96	121971.85	116099.37	133240.12
1258488.27	1422512.35	856177.06	722274.09	772217.51	1487753.53	930207.94	938645.26	635857.80
3342011.27	3948176.71	2362651.12	2071447.83	2211980.12	4258505.52	2654665.44	2666881.65	2358323.42
81456.84	98104.27	59167.88	60261.65	70240.73	104267.84	66277.11	66961.53	86550.54
1140302.65	1355528.40	802369.08	796240.61	924128.22	1435955.60	890598.01	910269.16	710026.86
417617.81	464793.49	281275	243531.80	265862.11	502272.95	307540.36	313529.16	161747.82
99557.82	118557.92	70848.95	66150.56	72192.79	132233.65	82178.78	83775.46	58326.04
46118.06	54950.86	33783.94	33492.75	38499.27	58976.35	36708.64	37097.49	27981.04
961104.74	1160235.26	679241.01	611196.72	624985.81	1243978.15	783924.10	798042.80	689019.68
2126	1690	1189	677	997	2231.34	1133	1056	244
0	0	7	0	0	134	0	0	0
4081828.12	4497691.39	2810960.64	2575013.99	3030373.37	4595140.32	2890443.89	2901710.65	4761568.36
170597.06	214357.08	118144.48	115825.84	114225.58	235035.26	151384.19	157721.55	79806.08
1199.12	2168.12	880.12	1695.88	1085.50	1686.15	812	1267.85	17162.50
45070.39	53277.39	32417.17	26544.65	27614.60	57317.01	35808.06	36244.17	27566.28
582374.20	650668.51	391895.54	316848.49	335662.72	688748.89	425422.05	429463.32	299703.88
4263.66	7123.50	2435	2052	1802	8356	3270.49	3040.50	1208
2502260.32	2982309.64	1829644.50	1809454.03	2116720.48	3203211.69	1994365.94	2016936.70	1547983.68
0	0	0	3.50	0	0	0	0	0
126070.84	143309.40	88155.23	82651.05	92348.03	153318.25	93684.66	93320.51	179186.38

746133.71	887137.53	544541.72	513120.98	589164.20	952556.49	592191.79	598629.10	481982.02
8525	9623	5679	4124	4343	11244	6022	6152	2019
1220418.87	1449404.19	891380.92	869447.60	1014316.16	1548056.50	961866.02	966813.14	861605.96
31.83	131	19.83	50.67	17.33	247.33	19.17	64.35	7.16
0	0	0	0	0	0	0	0	0
421893.04	504644.44	307677.52	297875.82	340272.43	536765.35	333017.01	340433	254641.90
0	0	0	0	0	0	0	0	0
11176	11747.50	7067.17	14182.43	26234.50	14090.87	7429	7542.68	2513.22
58450.66	73311.68	44343.66	31620.34	32286.66	68762.48	44586.34	42304.56	12383.32
114926.30	122537.49	73131.25	59491.47	62168.77	134168.71	79940.95	82022.02	74996.16
0	7.50	0	0	0	49	0	0.18	12
6800.50	7861.50	4429	4560.50	4222.75	9773.50	5391	5500.75	1443.72
292778.58	343893.65	201927.16	187644.68	207254.70	372356.14	231466.59	235517.65	170777.46
1319251.07	1569399.24	947695.60	839781.30	910506.47	1668056.58	1048345.61	1049440	764274.36
1123397.51	1333846.49	803242.66	739265.16	818295.29	1400598.02	881298.26	878857.31	696347.78
1958395.44	2295547.13	1375766.93	1240796.58	1367341.46	2454870.43	1532048.07	1545801.53	1081765.44
32186.08	39806.29	23156.50	20410.53	20260.60	44107.24	26415.17	27712.49	25328.62
847298.28	1032145.49	638009.71	762784.32	1033695.19	1105264.59	664967.77	665999.81	807271.78
1127213.50	1326111.12	823035.54	696418.76	768175.53	1381735.35	871942.92	855698.51	556365.40
218087.74	272593.24	158359.59	163153.73	175751.14	298311.27	193402.37	194450.58	73792.36
244493.67	274298.87	163933.67	134511.21	139220.64	294739.90	181182.30	184622.85	123688.14
5306222.60	6299654.40	3768344.13	3609433.32	4090553.17	6771903.62	4220230.40	4276712.44	3590719.38
2667329.60	3164981.96	1925067.91	1874938.05	2154644.39	3382156.81	2100714.91	2131746.31	1916346.96
21045.51	20859.67	13052.67	9866.33	11416	22415.27	13628.89	13917.18	4123.86
5095.42	5999.56	2825.34	3125.48	3089.94	6018.91	3224.83	3639.15	17728.80
16.12	27.88	2.12	39	10	7	11	50	0
4889.53	7534.12	7878.02	35289.25	70977.83	14372.01	4476.95	4698.52	1716.16
282290.03	314954.49	189613.16	216932.95	294573.43	341592.51	193769.64	195131.44	497940.74
0	0	0	0	0	0	0	0	0
130423.22	166508.46	92406.78	88014.44	86203.91	179793.88	114856.61	118150.82	68523.18
379054.62	465096.02	280436.40	271609.72	318996.86	496648.87	312230.33	311651.94	291331.98
29.20	361	7	56.50	19	276	16	75	23
0	0	7	0	0	134	0	0	0
15875.15	20159.61	12301.79	18533.09	27699.11	23657.75	8531.55	9196.34	20725.30

878360.35	1023064.26	607262.88	546195.59	591178.80	1097223.08	680853.64	694062.93	576220.48
11	0	7	0	2	134	0	24	3
22590.25	25626.79	16042.17	11072.86	11248.27	28424.24	14830	15480.32	22160.96
11	0	7	0	2	164	0	24	3
94226.94	119556.44	66241.26	65241.94	64875.45	132907.61	86563.89	88970.25	25218.84
529423.28	617789.59	382234.83	366514.37	435695.22	661138.75	406027.44	410272.16	375356.78
1024958.65	1227444.41	752572.77	729927.67	836738.27	1320680.28	825179.30	832808.27	685687.50
251549.88	291710.77	175599.07	160677.90	179030.73	313228.21	195407.22	196270.90	224678.06
1153644.94	1356522.66	816911.10	749840.68	829947.77	1447610.64	896795.30	912415.87	760822.72
720224.99	835695.91	504923.85	480340.73	556894.65	893955.76	549977.15	558277.12	437207.14
167576.09	194630.40	117605.78	103713.49	115083.34	206716.54	122745.48	124072.99	288867.64
2170914.34	2588764.82	1556747.59	1480655.18	1670575.81	2778003.41	1742319.94	1758067.67	1561157.68
0	0	0	0	0	0	6	0	6
90762.24	107799.97	66128.67	64075.87	76599.66	115549.24	72034.61	72918.53	72121.14
5103.61	7370.67	4870	4010.17	3471.50	9087.17	4593.64	4755.85	1321.52
1715243.99	2052240.15	1238796.80	1184826.14	1336715.33	2199975.81	1370741.38	1387439.78	1237193.08
87648.39	102158.54	61737.75	61908.78	74253.49	110261.32	68921.64	70707.40	54314.82
597724.22	722148.92	422804.53	411141.05	447075.23	778837.80	488036.54	499396.02	449377.56
1561392.14	1809710.36	1100607.73	1035598.42	1209879.46	1927005.57	1196078.55	1208230.75	1053016.82
211075.58	257127.01	157533.13	144654.63	166852.99	267780.81	167534.15	166005.42	116129.88
23070.26	26023.34	14917.66	10675.34	11531.66	29741.32	15629.84	15477.70	4832.32
464990.89	545765.10	324820.86	281841.24	297016.23	582103.34	365371.89	370311.38	192789.20
1818298.35	2152932.95	1325941.40	1309811.54	1539251.39	2300368.46	1428309.81	1438854.21	1305708.02
1120813.86	1190928.13	753052.32	528989.29	560966.26	1242987.30	750611.04	729461.75	841536.72
10954.48	13839.43	7156.96	20282.75	35852.27	14966.84	7413.72	7433.64	19354.62
439909.22	517822.64	301145.54	268770.45	277753.65	564179.78	352695.46	362638.49	225035.12
264657.20	309832.06	187879.09	152178.46	161127.04	322470.47	203076.10	201117.66	76067.28
12189.51	15136.75	11435.82	13160.74	17686.22	15927.34	8002.89	8621.04	19700.14
730880.83	858599.72	514709.06	490144.63	552914.16	918014.35	568112.60	578255.01	525977.02
755719.11	907434.73	539828.18	478144.47	486656.41	959173.38	618498.64	620980.39	782847.14
363438.86	425112.85	251462.52	216178.96	224975	455171.75	286782.20	291378.95	136969.28
0	0	0	0	3	0	0	0	0
596624.78	703446.75	411931.49	373833.14	386456.42	761933.91	480968.96	492200.97	346473.88
61297.09	75847.46	45653.87	43766.76	47666.93	82884.87	47446.53	49448.96	48955.36

0	0	0	0	3	0	0	0	0
92771.84	107429	64165.35	52635.11	53802.23	115412.43	72694.61	72537	71750.72
41620.16	50758.65	28615.98	26038.66	25987.10	56340.37	33601.17	34949.65	27052.02
51930.47	59193	36903.50	34430.17	41101.66	61989.10	40136.81	40622.20	12015.46
120095.57	142028.65	80135.16	76320.97	82081.59	154035.73	94946.68	98286.58	79747.40
460942.51	554825.51	335907.48	301314.69	328029.35	591489.73	371414.71	373348.71	380075.24
115196.87	124715.97	83516.45	76079.49	99128.99	133496.82	78221.44	76404.37	123658.32
516337.73	577466.07	348612.08	282222.59	289189.42	611945.73	380829.83	383006.04	284482.20
1059841.69	1240138.99	760428.78	722751.15	834130.20	1328809.38	822847.62	830345.59	869914.30
24856.15	34046.22	20985.34	17630.48	18554.02	36327.25	22728	23358.57	23746.32
657483.40	780479.81	469574.61	515509.51	643223.75	833744.22	503665.73	515204.34	877361.26
417068.34	493098.21	302076.55	284128.68	317634.55	523187.14	326490.64	330721.31	318536.52
297615.93	359040.61	212699.42	206065.72	227226.16	389588.16	242507.28	248068.59	224076.62
115733.56	125800.09	81285.44	81120.20	101706.32	137336.23	80406.58	79883.64	41672.14
74266.68	86047.11	52532.91	43655.32	44143.76	90680.13	58563.72	59578.24	51151.68
93113.31	107148.49	64641.91	53505.15	54736.26	113413.45	72788.18	72697.87	37933.94
29942.11	32394.19	21949.22	19400.09	25007.75	34104.77	19980.09	19566.41	39630.20
49006.73	57955.39	34044.55	29187.98	29628.37	63075.25	39570.72	40241.15	11807.80
1347022.30	1570736.45	932413.50	888501.88	1003101.50	1684210.29	1036398.08	1055451.75	989048.92
85489.49	89460.01	57447.49	38907.01	41857.99	92720.68	57060.51	55171.87	16857.48
629453.31	737477.57	454499.65	402648.69	460691.93	783653.53	487576.53	482604.26	461390.36
172502.82	196410.76	115649.49	94363.18	95699.63	206023.54	126182.46	129155.78	90492.18
1075477.95	1284661.73	769409.29	735885.09	825335.69	1372476.46	850786.33	862780.88	713828.16
1005527.55	1194394.03	734475.65	648234.22	703489.25	1278201.79	797035.70	805183.21	676209.64
8381.45	10467.05	5767.84	10475.65	14648.77	12188.14	5907.83	6058.64	18754.82
1086	1991	619	1136	983	1349.34	754	1237.18	438
954324.58	1125613.14	690568.85	649018.05	750956.28	1199900.26	737927.71	742678.33	679199.40
3847081.96	4418817.96	2710275.82	2295623.45	2525276.13	4665742.65	2922670.58	2904688.05	2694629.38
143940.15	162771.69	101791.15	70527.35	74144.65	161483.16	101646.85	97476.43	29240.80
179467.58	200170.74	125070.57	111674.38	130784.58	214883.67	129918.29	130237.32	141213.52
91219.44	109261.85	67586.04	59929.67	66014.32	117131.92	74408.81	73941.35	71648.10
45708	51288.67	29213.35	22601.65	22625	50657.65	26943	29758.44	10792
1947397.21	2327494.17	1416342.20	1374042.92	1575727.08	2502459.14	1571193.01	1584007.81	1408879.66
83060.42	85558.36	61094.48	50951.86	63158.82	87225.11	54317.37	50981.42	116446.54

1220780.56	1452730.24	890756.93	868397.51	1008316.42	1553469.25	967508.39	978171.51	748072.82
184554.05	220055.91	135942.03	136983.10	164448.83	236241.17	146878.45	148398.88	111931.92
39726.79	45013.30	33098.80	29057.60	33849.63	50439.12	29743.04	30693.41	92772.96
64210.74	78529.93	46282.79	51993	62590.61	86569.88	54480.53	55131.62	66454.54
58450.66	73311.68	44343.66	31620.34	32286.66	68762.48	44586.34	42304.56	12383.32
10739.83	12500.34	7036.17	5185.50	5533.33	14574.82	7462	7742.67	2350
96875.93	113708.08	69125.85	63016.61	68141.20	119050.64	77269.51	76035.15	55708.48
718130.53	848993.37	526884.93	505095.26	594518.43	904835.06	561386.93	562560.70	454673
345547.17	428624.73	250455.48	230602.94	245305.11	458909.86	286093.30	288749.16	203804.54
1176087.13	1357764.79	820685.59	727365.90	803278.38	1450935.58	893408.20	904103.56	658535.06
0	0	7	0	0	134	0	0	0
1424420.21	1692796.27	1004702.31	878438.84	901144	1819488.55	1142940.39	1152714.89	1007885.52
472.98	1352.81	1236.22	1734.20	538.94	1197.02	164.72	156.98	16888.66
31123	36647.50	22005	17446.50	17318	36942.90	21137	22623.90	7230
261095.99	291607.13	175569.07	164197.56	194472.97	312966.75	184090.08	189171.84	159795.80
0	0	0	0	0	0	0	0	0
0	0	0	0	3	0	0	0	0
26429.13	33608.31	19222.50	18394.47	19380.77	39126.70	23476	24430.81	23949.86
392268.78	435035.59	259541.58	219722.67	237821.11	462856.19	287619.73	292355.26	123267.12
127788.13	144003.34	90678.76	68272.43	70628.71	149834.15	95428.43	94114.90	44177.72
0	0	0	0	3	0	0	0	0
167260.39	196374.67	118398.88	102916.37	110227.16	209273.74	129992.84	130670.71	122606.84
0	0	0	0	0	0	0	0	0
141646.53	153188.64	96426.14	76126.69	82989.83	157860.26	95256.53	95069.49	147530.80
449533.63	514113.53	315039.71	302812.14	360634.12	545779.69	334229.47	338512.08	355263
169274.92	191711.63	117025.56	99839.74	107077.76	202397.47	128776.28	128688.35	88713.22
73325.16	90961.73	50056.28	48194.69	46252.36	98139.86	59861.96	62080.50	68930.94
45606.25	55532.39	32060.67	29358.15	28989.02	60456.74	38547.75	39148.07	27905.32
130109.70	148618.97	86954.81	79540.93	86467.87	161481.35	97953.12	101157.36	114061.56
965564.55	1061582.24	664425.08	479619.86	501658.23	1089018.89	671413.52	652743.94	812631.62
393772.10	463594.09	286097.91	286524.27	346175.99	498194.08	305422.32	308393.64	227587.52
564429.08	654972.89	384781.85	333042.67	337911.48	706972.68	452871.67	459880.16	202837.06
587976.59	698600.27	416007.74	360876.24	359041.43	735798.54	480054.49	480464.50	489971.28
818957.03	976056.58	596807.66	593567.80	701795.95	1045743.90	648600.40	652044.10	564713.64

34519.76	57335.77	25499.21	24098.86	28368	68002.73	28355.64	26875.85	11782.24
17125.03	24002.57	12299.33	13996.38	13342.33	26497.10	17248.17	18657.85	5392.66
58450.66	73311.68	44350.66	31620.34	32286.66	68896.48	44586.34	42304.56	12383.32
0	0	0	0	0	0	0	0	0
1218699.07	1437499.80	873577.73	823705.09	929068.58	1539216.74	966293.40	972636.91	826838.40
0.50	8	0	421.50	96.25	64.50	6	4.75	40.22
875071.84	1037722.63	639204.17	626790.22	737030.70	1110473.84	689239.84	696647.08	579999.58
598594.39	708130.35	425254.27	371533.75	395148.81	742151.78	468401.87	470955.97	423297.96
621021.79	735709.48	449118.32	424977.55	488768.13	790669.63	490250.71	496145.58	401279.60
208099.69	256389.75	157778.13	231549.13	335289.36	282973.69	157953.22	158900.11	335912.36
0	69.50	0	0	9	32	0	0.18	0
164666.62	201199.74	117616.33	96185.45	98197.96	207917.71	130766.40	130836.73	54929.90
142294.87	162379	98952.25	80617.07	81783.84	171455.32	109147.41	108465.89	148887.72
660488.67	775937.97	468893.58	436807.55	496605.51	813604.99	498984.78	506804.62	358715.46
45606.25	55532.39	32060.67	29358.15	28989.02	60456.74	38547.75	39148.07	27905.32
1579886.90	1842114.21	1110529.49	979602.31	1060367.69	1962950.38	1225187.95	1236176.99	843052.36
773699.85	921523.15	563516.41	498616.72	551028.99	976072.30	620279.91	617284.25	579088.72
280238.96	322990.11	197470.39	167680.81	183863.30	341699.92	211434.57	211794.63	231083.52
2819967.79	3377683.37	2016132.95	1854374.09	2023405.50	3629652.21	2267094.81	2292865	2371859.20
49838.57	54418.68	34417.17	33134.37	44055.88	55749.06	30688.47	29996.82	43711.52
124140.01	152644.84	92650.43	99955.18	121881.28	163629.73	101682.58	103161.61	64716.52
592307.97	700976.07	429640.57	415997.33	481423.67	751324.26	468869.73	471866.53	392931.46
1217.83	2218	732.83	1276.67	1004.33	1873.43	807.17	1518.35	493.16
46313.06	55453.70	35094.94	33849.75	38605.27	59878.11	36867.64	37343.99	28144.04
20831.15	21179.68	13696.81	9809.69	10294.99	24333.39	12265.51	12820.63	3873.80
3190858.41	3576815.77	2201032.83	1692303.95	1761210.74	3774649.93	2357291.86	2321947.82	1955402.30
10127	8431.67	6558	3662	3999	8566.33	5454	5113	1753
1040461.49	1180946.53	704772.05	592513.76	624149.63	1262897.55	786516.14	796397.43	476449.98
687938.38	807219.33	489318.75	473698.11	552866.23	859655.57	533986.14	541154.43	382559.80
0	0	0	0	3	0	0	0	0
0	0	0	0	0	0	0	0	0
1101697.81	1278023.80	781560.88	631980.74	646200.62	1330821.06	826612.32	827582.55	537542.78
0	0	0	0	3	0	0	0	0
0	0	0	0	0	0	0	0	0

272190.74	333882.65	198300.68	193828.09	213423.11	357377.80	224470.34	229169.47	218927.90
374563.48	446162.55	275247.85	267351.67	309789.16	478401.51	296658.12	300774.60	225576.44
363912.23	451913.43	284210.07	430913.71	644067.64	486289.57	292660.04	294571.33	527068.18
60	111.14	9.16	177.42	10.28	28	44	200	0
204510.83	234847.61	146502.20	145631.14	184721.19	250900.27	145950.69	146544.76	262271.90
1050035.90	1244866.20	766349.59	806260.91	1015541.46	1340013.73	816563.48	830024.56	922641.50
966429.59	1188316.20	698655.93	737491.35	845763.34	1286151.48	802623.59	814058.83	678231.04
548441.09	621268.13	369209.33	314252.08	331250.52	657738.05	407621.95	413105.15	275687.80
1366440.72	1625569.01	960612.61	859803.18	916449.98	1778128.47	1090587.57	1105498.41	896905.56
36866.60	52781.68	27719.11	46170.09	58641.54	60419.25	30264.61	33677.64	111836.02
140039.10	169664.03	122136.86	403063.56	741941.76	181390.76	102731.70	102216.65	587461.56
20137.87	25793.27	14008.96	18830.49	22629.52	29275.98	15803.83	16728.91	21607.54
0	0	7	0	0	134	0	0	0
1366323.99	1517728.78	917787.45	746039.62	803494.64	1607426.81	992589.99	1001344.14	509804.66
183563.48	218482.32	129850.22	130362.16	154513.08	233976.40	146263.59	147860.64	111471.50
408767.26	475698.46	280160.05	258781.32	271198.96	517824.77	332829.94	340699.20	150568.46
0	13.50	0	0	0	0	0	0	0
0	0	0	0	0	0	0	0	0
1243379.95	1410833.23	852647.84	737100.22	811260.79	1492730.20	926986.12	935771.29	604949.22
0	0	0	0	3	0	0	0	0
71634.86	82500.30	50908.20	53767.19	70600.64	84871.59	54322.72	53192.32	32446.58

A20	A3	A4	A5	A6	A7	A8	A9	B1
0	0	0	0	0	0	0	0	0
553309.59	1654878	671947.39	892981.89	1384325.40	1638581.19	1685867.34	1290126.20	1118368.75
2259460.70	5773522.21	2484960.66	4557422.08	5574170.45	5704317.45	5821320.33	4406708.17	5514091.73
488838.63	1143939.07	495695.86	996756.32	995823.41	1130981.72	1151504.90	886586.45	872153.80
41991.42	126476.28	50812.70	71921.94	132714.17	125916.52	127114.44	93283.35	148334.89
17517.37	77975.76	32691.40	7963.02	43271.74	75885.66	78582.75	57331.84	7212.98
163215.46	341166.90	165184.36	347301.28	388867.52	332951.18	342783.32	260782.94	444125.22
0	0	0	0	0	0	0	0	0
3908998.84	9471354.87	4679595.82	7703903.63	12656651.41	9592447.11	9503176.69	7855466.89	16298404.69
34891.16	90399.92	42029.94	69202.44	84001.77	88279.54	89224.70	73067.55	78064.41
169911.36	648358.92	254292.90	180269.42	419516.83	636546.02	659391.59	483519.75	196351.89
769940.02	1828543.79	762380.54	1499244.06	1337902.88	1809799.19	1846342.43	1405563.08	873766.07
19806.34	88382.84	33569.50	8724.02	54484.02	89080.34	88718.68	83765.48	8677.32
10150.73	52859.04	40475.20	6947.73	63717.45	51881.27	53610.77	42159.50	72521.60
32	187	31	15	186	98	109	743	17
17423.37	77932.09	32688.07	7685.35	43234.08	75871.66	78582.75	57326.51	7164.31
263804.54	542452.24	346017.70	634677.72	1162436.92	534004.64	558685.94	446171.23	1822106.98
200661.42	502281.44	397774.56	366824.32	487435.13	497193.58	505237.04	397284.22	463436.61
178243.60	751390.05	278762.63	133977.77	441288.80	747683.30	766115.39	559397.70	135350.48
1426.02	547.10	10173.60	3247.87	524.35	305.80	441.49	264.75	321.56
330875.79	843155.92	359832.46	540074.95	722016.57	841424.56	850322.92	649446.74	623742.14
0	0	0	0	0	0	0	0	0
361886.94	978772.33	403222.35	712031.38	841495.22	964984.46	990109.84	748382.76	719148.51
1645134.67	3676411.73	1747269.37	2913319.59	2646016.13	3596311.52	3663640.41	2666661.53	1661159.46
48220.67	217109.94	77987.40	43498.27	116898.99	215092.88	221651.32	161182.98	21317.68
0	0	0	0	0	0	0	0	0
353160.38	659331.12	285251.49	655609.74	696674.75	652129.16	664504.20	511922.16	741226.38
101710.68	326605.83	145004.02	130549.44	179218.77	326417.05	333507	254355.26	32248.03
99638.93	320969.10	143780.96	128374.33	279787.15	316278.57	328041.73	247273.94	232566.54
488040.82	1153920.93	430099.87	774274.43	697801.15	1147973.05	1178900.01	893780.99	248885.76
375826.06	1484560.60	556393.92	267413.62	941494.45	1487631.31	1522839.41	1152781.27	406371.29
0	0	0	0	0	0	0	0	0
10625	48437.51	17087	4502	25727	46937.67	50258.16	33074	4129

393755.74	1224660.82	440690.36	498150.61	767670.47	1244426.24	1256408.95	978897.65	316580.02
0	0	0	0	0	0	0	0	0
16911.68	9072.68	3454.50	38095.79	71337.47	7736.27	8816.96	5741.50	135036.09
17726.68	10947	4582.83	38441.34	7124.75	11247	11238.61	12035.33	1079.99
27396.08	114813.48	101810.25	12182.10	68661.20	114997.52	122229.39	103666.41	11548.53
7650.07	37183.66	28685.75	27304.04	89651.91	34860.84	37952.15	26728.10	140635.32
5125.50	32533.21	12865.87	3033.08	17460.29	32898.82	32146.34	26958.50	3032.13
1279625.66	3499293.54	1560086.62	2283950.48	3151381.83	3458468.11	3567658.48	2724329.17	2807834.65
1333742.51	3402956.96	1828943.17	2301148.50	3668860.52	3355520.49	3445962.70	2627659.59	3997247.58
0	0	0	0	0	0	0	0	0
680.07	3927.25	50975.20	1386.40	2694.08	2570.48	2832.48	3542.61	1805.57
513	2693	823	22064	1395.90	3049	2327.60	2856.50	1780
19806.34	88379.34	33569.50	8724.02	54484.02	89080.34	88718.68	83765.48	8677.32
0	0	0	0	0	0	0	0	0
179879.05	706286.80	323217.73	166506.65	525151.50	691969.77	717180.31	532003.63	336074.82
117089.61	387182.39	168188.77	141083.90	248128.31	381097.66	391844	296545.23	105935.40
171778.46	443114.18	181232.20	364102.02	477534.39	439763.81	445855.34	348989.22	523220.97
119525.35	342875.92	138620.43	234425.30	288125.67	341805.48	347162.58	261744.11	241409.81
9	10	0	0	14	0	0	36	0
17498.37	78103.09	32783.07	73448.35	43557.08	76043.67	78842.75	57798.51	12668.31
23.83	79.84	52	15.67	62.67	80.17	117.50	68.33	2
0	0	0	0	0	0	0	48	0
155287.68	507062.65	246560.83	249108.76	311656.12	504071.95	513413.02	384057.83	121003.17
863690.20	2890855.74	1241345.30	1228288.97	2269231.71	2860031.15	2932473.84	2202616.98	1649637.95
22175.71	98750.83	40201.84	10114.85	90468.38	96509.76	102728.92	78542.83	76657.97
591149.37	1421966.29	586412.67	1222595.12	1147011.42	1410619.54	1440047.73	1109842.28	906716.15
24324.56	124065.80	49581.68	12106.96	132573.15	124705.38	131808.84	97834.77	145579.66
425924.55	987385.30	419504.57	882275.53	616597.89	972758.73	998686.18	749342.46	249651.10
2505.70	14170.40	4925.66	1284.24	40138.51	13286.12	14428.04	9919.35	68635.36
218770.48	919911.98	382130.84	150876.19	609266.16	909046.89	941144.65	695767.81	288186.80
68547.42	240960.69	96761.27	82858.15	140143.58	239140.30	242721.07	198180.33	24464.62
1523.44	7316.29	26869.51	2282.21	4224.46	6679.20	6821.54	5570.71	940.73
88186.85	258604.02	110152.51	125282.06	210833.80	255553.11	262044.12	200320.62	160841.80
19806.34	88379.34	33569.50	8724.02	54484.02	89080.34	88718.68	83765.48	8677.32

9	10	0	0	14	0	0	36	0
241768.86	564817.63	241651.34	509178.21	412959.34	561496.52	570144.85	447931.72	268444.57
434039	1148049.06	471601.02	812954.73	1114999.31	1135473.63	1165688.26	893359.02	1072207.87
44083.58	199806.76	123371.94	19470.81	110941.79	193331.86	203273.46	138466.01	18368.47
24852.38	46556.92	21357.24	42028.51	60624.72	45498.89	50742.43	36707.84	71826.50
24709.92	47452.82	25445.77	64313.58	60731.23	47923.85	47447.57	40927.93	73890.97
107422.20	300860.75	161056.53	145858.38	359112.73	314059.96	308258.24	257665.63	432123.10
33134.50	148886.20	53912.23	14505.95	86452.19	149506.54	149177.11	126062.14	13958.15
0	0	0	0	0	0	0	0	0
0	3.50	0	0	0	0	0	0	0
689093.38	2084823.45	852703.42	1067415.72	1486428.84	2096465.12	2126841.80	1654355.28	889697.02
52806.03	240277.50	86323.05	23390.64	163603.48	245124.22	246212.48	195761.63	88446.47
101159.23	325736.68	124500.77	150898.27	253522.31	326155.58	329931.73	273825.96	169154.73
365003.69	926044.98	444767.32	700799.25	908835.38	915850.34	930954.03	729181.29	911421.88
68816.54	236175.48	129869.91	84008.21	207373.64	225939	241858.62	193418.88	160034.58
574224.74	1974432.78	854422.10	612158.61	1290612.39	2004752.48	2004236.74	1594960.83	591487.13
1633647.76	5467873.47	2435750.80	2113265.80	3765031.26	5380430.54	5623589.92	4243725.62	1969526.92
30362.44	134057.69	61851.24	36221.32	138937.36	133805.08	136199.28	103832.20	149704.79
665999.32	1880167.06	844855.04	1068044.13	1788267.24	1877174.20	1895909.50	1474150.61	1742044.02
171703.67	658978.44	243997.15	187129.83	459879.46	668209.53	674475.16	526987.02	265731.14
53763.10	170238.30	67995.17	79117.15	128056.25	166832.99	171389.97	123654.68	84615.01
31733.05	76492.76	31090.93	67131.30	75623.37	75170.33	77130.26	58298.34	76442.97
417553.38	1626284.98	698729.52	378577.48	993892.11	1611273.57	1644905.87	1222167.15	356917.07
532	2072	738	319	1447	2375	3070	2334	196
9	10	0	0	14	0	0	36	0
2437418.44	6215876.03	2854766.01	4305319.55	5865548.95	6368666.78	6225087.24	5181529.81	5652336.30
69705.70	309303.41	141653.95	29795.77	163640.78	304109.98	313634.54	221258.13	27465.65
593	2821.67	911.67	461.80	1343.87	3231	2263.10	2683.08	383.50
31308.71	74639.26	30503.70	66448.78	42411.48	74188.30	75419.64	57052.34	8622.99
314302.80	912242.60	335826.49	432859.22	530711.48	924480.54	935296.45	728285.81	154475.44
1163.50	5829.50	1854	626.40	3264.90	4234.70	5974.55	9039	750.05
1764601.63	4156718.55	1701899.07	3715163.77	4191260.97	4084183.49	4190174.54	3161055.99	4328397.44
0	3.50	0	0	0	0	0	0	0
58529.82	198959.59	180493.37	81556.70	178261.63	199525.78	200412.17	158365.11	155996.37

538424.02	1235842.80	511434.18	1122611.85	1113729.78	1217535.99	1247616.25	941615.31	1019993.47
2510	14005	4587	1087	7561	13775	14796	10110	1125
842706.36	2005256.14	889941.25	1718646.54	2013225.21	1972698.35	2024377.40	1545958.82	2051488.15
35.83	89.84	52	15.67	76.67	80.17	117.50	152.83	2
0	0	0	0	0	0	0	0	0
286179.90	703173.83	283506.71	579801.66	653168.44	696326.30	706435.05	540974.06	619318.45
0	0	0	0	0	0	0	0	0
18264.68	17224.35	5623.50	38906.79	75866.97	16641.27	18525.96	14304.50	135778.09
19806.34	88379.34	33569.50	8724.02	54484.02	89080.34	88718.68	83765.48	8677.32
51391.91	175360.98	60711.47	53882.28	96500.20	180643.72	184214.98	138028.60	15922.45
0	30.17	3	0	43	63	0	0	20
2380	10743.50	4074.90	1084.92	6519.10	10862.70	12329	8553	917.16
168181.68	483524.44	183410.83	223706.29	366724.73	479408.06	491271.53	376964.06	248219.77
630919.75	2158787.41	954157.86	887771.01	1601604.97	2137585.28	2185597.16	1685060.95	1034913.40
502854.04	1809604.49	903248.09	661427.10	1507789.38	1796098.70	1838247.24	1461061.02	1200325.41
1037256.68	3194078.09	1288399.67	1540583.89	2425423.99	3177801.29	3234491.30	2470856.99	1670806.81
11904.36	57479.76	24558.37	27153.44	30428.41	56084.30	58840.46	41122.51	6694
696557.42	1396680.40	686005.02	1588281.12	2450658.35	1354731.17	1416807.10	1099565.03	3607150.31
663447.81	1785305.66	861450.31	1182303.42	1384125.12	1761782.40	1803575.98	1442556.37	941432.17
134818.60	380311.49	164890.12	155408.46	310982.63	369821.77	383993.03	276964.55	239916.57
128662.81	389303.31	140789.25	173261.77	211132.71	393261.05	398659.24	307278.75	37073.93
3226546.30	8810689.02	3869191.35	5179181.49	7625185.45	8699185.02	8920720.05	6721246.45	6564583.86
1744280.81	4407894.25	1941829.86	3383119.58	4178405.54	4363088.80	4438803.01	3386447.17	4001818.28
21359.68	30399.83	9211.50	40212.55	16583.14	31871	30923.87	24884.83	2625.09
1789.04	7434.23	7519.87	771.94	4446.98	8626.62	8880.71	7716.01	684.66
17	44.50	35	12.88	41.12	27	55	69.75	0
154508.85	12414.59	4089.05	373421.12	205850.70	9007.57	9355.44	6507.31	405600.57
180434.83	435793.80	249846.22	316842.86	698563.20	438131.19	448736.15	358217.68	986064.67
0	0	0	0	0	0	0	0	0
54367.47	235255.26	97534.59	27353.91	127114.17	228174.15	237940.08	172261.78	21271.14
261602.49	632323.89	313046.67	514195.98	620958.47	619465.98	642605.21	490098.35	609295.17
35	124.17	42	12	118.90	158.67	95.60	742.50	194
9	10	0	0	14	0	0	36	0
4104.20	23873.67	21932.05	47356.84	84041.52	23731.67	25305.98	23574.26	140691.45

400783.59	1438960.95	581801.70	475514.12	1024137.24	1439786.60	1469082.22	1107100.57	610700.95
9	18	4	0	14	10	40	36	4
6301.53	37032.91	16629.37	24818.77	19811.74	36653.46	38151.80	28283.18	4817
9	18	4	0	14	10	40	36	4
39757.87	170611.77	61111.36	16930.23	92532.48	165291.68	174970.64	120373.26	15387.79
349520.25	857842	358539.19	758850.57	872012.25	851733.72	866679.64	667979.83	909733.09
690981.86	1712718.12	747468.65	1419588.51	1611970.52	1679606.61	1721471.87	1292929.12	1547677.64
118764.81	401968.40	169009.90	159321.34	324535.21	401662.03	413729.34	315151.84	243073.41
786696.06	1891749.29	792649.60	1451546.87	1468850.98	1884428.82	1911337.50	1468187.41	1080836.97
445652.26	1169394.15	457650.91	733584.78	1078013.05	1162385.18	1182634.44	907635.36	994078.51
88707.85	267745.10	104885.74	128995.50	219139.12	267811.40	269037.63	218082.83	162285.65
1284911.72	3602723.60	1533742.67	2118637.03	3125265.25	3544340.48	3643605.98	2747150.52	2671482.75
0	0	0	0	0	0	0	207	0
78667.49	150876.26	66371.94	172169.15	148188.39	149112.95	151956.64	114592.70	152024.01
2079.50	9300.34	3617.86	22963.88	5627.47	8873.27	9738.59	7069.33	2653.08
1062994.80	2850682	1218792.10	1928063.36	2510531.62	2806164.55	2882367.95	2180816.57	2200437.19
61202.28	144370.75	59256.42	109897.37	146238.08	144580.63	147252.92	110681.69	150278.59
342927.22	1010296.59	438848.78	531056.62	788834.44	995833.37	1026591.42	760467.42	576763.57
1087541.35	2527745.37	1088685.04	1949881.48	2324215.30	2518757.27	2553826.34	1974424.80	2149623.33
120627.37	342110.02	142101.22	212441.97	328807.51	336870.23	345205.81	277833.75	307794.16
6701	34969.18	11640	3074.68	20031.88	34139.34	37662.64	29478.82	3028.16
213706.96	756172.22	293770.07	299949.59	483953.03	758887.27	777139.53	593101.28	213924.20
1254969.77	2982143.13	1294488.73	2554272.55	3096585.82	2937558.99	3008503.09	2298795.75	3245194.78
333501.99	1601495.63	808023.48	162392.31	923949.70	1639006.83	1658985.63	1386123.34	152988.75
3232.70	17452.84	11063.14	2067.95	108840.20	16447.68	18683.08	13858.10	203727.61
176781.10	741989.55	276395.63	133273.43	436504.46	737628.13	756430.22	551101.04	134679.15
106851.93	418101.92	161551.94	101493.74	270150.84	419093.93	426029.44	339399.06	107961.58
19017.74	20400.78	21749.17	83859.39	45232.78	19509.98	20535.98	16333.35	73297.23
457430.17	1200885.48	609157.93	808540.92	1026536.48	1191562.76	1217546.35	932085.04	868327.88
312505.08	1257197.55	787908.35	206587.14	774312.27	1240427.50	1272803.92	950232.59	255577.15
146278.17	591443.42	209992.73	206457.17	357550.14	591309.24	615020.58	459330.16	131221.73
0	0	0	0	0	0	0	0	0
268773.17	997612.40	422084.48	284650.21	615193.63	995518.07	1013365.22	753724.35	231844.12
36997.39	103962.61	50079.50	113303.84	92858.86	103001.42	105033.08	83964.91	82433.61

0	0	0	0	0	0	0	0	0
33367.11	150366.24	66218.95	16236.15	82925.83	148203.54	153867.54	114487.87	14039.82
15449.03	71226.87	29896.29	7280.07	39208.59	69772.70	75582.90	52402.25	6790.34
33412.68	84279.42	28045.43	67454.41	77567.54	84686.44	83013.15	64904.16	76286.48
44384.22	201290.75	101669.99	20766.08	140978.48	202137.97	207019.45	159949.14	85201.16
347366.86	769768.81	355003.88	674066.72	559892.83	754689.32	771157.72	586345.09	354997.64
35090.73	165746.73	65553.56	83491.42	230967.61	169865.32	171405.90	142801.98	291470.84
247970.38	808398.84	333538.64	288419.59	447063.22	815842.60	825985.94	643425.03	77481.14
631149.69	1725822.40	730385.77	1165057.54	1621803.11	1708351.06	1745013.61	1331209.44	1539820.60
39809.54	47005.92	22005.87	121285.15	26034.28	44208.80	45868.23	33754.18	7847.76
502031.39	1084384.48	504009.70	1045946.80	1395290.21	1074791.68	1089246.07	851801.60	1739135.74
341827.93	688909.97	291156.96	724133.06	581134.38	681743.25	687622.12	527260.04	483548.57
171311.16	505694.47	224528.32	309582.35	406080.39	499288.73	510808.84	378826.66	323819.07
63374.56	173434.52	60494.03	104223.80	228051.20	175457.08	178168.61	143096.45	288638.67
26919.04	120746.81	50039.24	55986.03	67380.46	122221.79	124246.97	92886.09	14847.22
35518.06	147390.91	50621.35	19529.36	82138.10	149637.13	148676.38	119500.16	14079.94
8908.03	43429.26	19266.41	26470.55	59220.73	44049.23	44261.43	36901.67	73544.57
17831.37	81821.58	33477.87	7831.22	44926.01	81094.89	83242.60	60902.68	7327.23
765764.19	2197489.36	966061.74	1117174.90	1866897.09	2193330.72	2226617.82	1712620.62	1564996.34
25277.50	121717.02	39663.50	12002.02	69750.02	125742.51	125891.51	103911.48	11397.99
370337.50	1005707.58	494741.31	630569.40	873279.59	991886.62	1021869.90	801482.50	714919.18
57368.60	274911.30	104177.78	26204.69	150373.71	279549.06	280743.98	221186.83	24828.64
628499.86	1784607.24	757886.02	1086564.96	1556537.81	1759573.03	1802209.76	1374300.15	1339212.19
649460.11	1658740.55	677788.32	1318019.98	1203491.29	1638027.92	1672472.44	1271509.75	801569.64
2514.70	14302.28	9906.36	1372.27	40313.01	13358.72	14561.44	10356.35	68722.75
452	2575	797	168	1221.50	3011	2212	2385	109
605134.85	1562977.01	764062.38	1159305.65	1458787.32	1541801.27	1574323.38	1221988.85	1386322.68
1871427.80	6042400.81	2785238.09	2457454.26	4448212.66	6045423.64	6163937.10	4848722.66	2696775.04
45083.84	210096.36	73233	20726.04	124238.04	214822.85	214610.19	187676.96	20075.31
119748.06	281385.31	120369.61	222976.88	253913.20	280794.74	285021.66	222165.95	232630.42
48684.95	149383.72	62916.10	96558.55	117623.02	146544.83	151760.06	115020.28	85262.27
15943.72	69730.34	20075	13430.52	37012.02	76844	70254.17	63829.83	5561.98
1211288.19	3241128.14	1404552.23	2223997.19	3020544.34	3183657.52	3273614.07	2453918.84	2853073.13
69492.59	116791.05	107180.36	169520.75	138857.61	116386.54	115952.56	96983.33	151277.64

802034.53	2018340.38	822504.23	1652490.80	1981453.89	1987966.45	2034990.39	1549603.53	1987748.59
142125.21	306329.71	124388.05	305986.10	336007.46	300657.22	308392.71	233508.69	373171.66
28295.39	66978.10	53398.75	152857.69	69186.16	64622.99	66880.74	46951.93	81716.40
55100.26	110536.87	53596.14	86432.84	127867.23	106981.68	112506.93	81525.10	145479.67
19806.34	88379.34	33569.50	8724.02	54484.02	89080.34	88718.68	83765.48	8677.32
3921.02	17214.51	5672.83	3009.68	9932.01	16365.83	18561.18	15312.99	1648.99
51677.77	153896.75	73410.71	56763.78	118852.18	153066.55	154866.46	122981.01	82723
510407.33	1171143.21	514418.35	1083878.97	1186325.86	1153098.50	1179899.46	908246.27	1216860.51
174340.39	586528.06	237219.89	258199.10	431809.08	573041.75	593388.49	454735.98	264672.08
587912.72	1889240.47	744416.38	901056.91	1444971.93	1887442.90	1926475.97	1486209.87	1004712.37
9	10	0	0	14	0	0	36	0
578054.57	2362325.09	1094070.07	473363.65	1447910.44	2337490.39	2399263.67	1794217.91	495629.79
195.70	1042.62	10439.77	546.09	868.11	788.11	614.21	896.76	632.77
9936	49948	16092	26324	27725	52816	48456	44745.50	5918
129101.10	408383.86	161047.68	196486.06	389209.58	413505.90	421307.74	327709.29	377669.39
0	0	0	0	0	0	0	0	0
0	0	0	0	0	0	0	0	0
42909.92	49432.53	22309.80	83905.81	26250.80	46490.06	49865.40	32645.26	4671.12
161586.39	616401.86	202937.56	136039.77	399613.95	628627.76	633642.52	486090.33	190375.38
44051.25	191841.65	70397.29	108815	109576.09	195476.91	203163.88	161406.75	26184.14
0	0	0	0	0	0	0	0	0
104188.02	269813.04	112218.57	182937.82	182168.47	268811.24	274944.41	214520.86	96663.85
0	0	0	0	0	0	0	0	0
43349.57	213858.24	128917.14	23127.71	151960.13	214656.56	215808.06	175970.15	88361.95
272546.35	717747.20	321974.42	481115.48	724991.09	718273.34	725276.17	562920.75	750331.90
73267.49	267643.24	109753.98	86057.11	180165.42	268869.84	271305.07	208438.54	94015.44
27514.40	125946.74	58020.35	12317.51	68348.22	125433.56	129056.81	97686.59	11444.41
17423.37	77932.09	32688.07	7685.35	43234.08	75871.66	78582.75	57326.51	7164.31
75618.63	212258.08	80000.14	96144.47	147214.59	214368.48	217287.95	162587.85	86072.91
301914.17	1403148.67	599275.07	144779.99	816794.31	1440793.92	1429804.03	1227466.56	134548.86
243935.90	641551.78	257228.55	545980.35	721216.14	634103.99	650449.47	499519.54	818712.52
207700.51	931528.25	338740.69	94468.42	503123.25	933208.38	951133.75	701082.63	84899.70
218193.03	967584.59	589297.27	98418.01	543988.43	963353.92	978545.12	736316.56	91999.33
565143.20	1348441.48	596939.30	1117585.31	1414787.72	1324249.43	1362149.13	1039707.83	1501169.69

41359.16	54097.94	17209.25	127763.78	64715.56	34108.40	55237.70	64212.33	79125.88
8076.83	35103.52	12617	3288.67	18038.50	33969.84	34698.69	23506.33	2933
19815.34	88389.34	33569.50	8724.02	54498.02	89080.34	88718.68	83801.48	8677.32
0	0	0	0	0	0	0	0	0
694305.78	1999249.76	864067.80	1167782.25	1736103.76	1973572.23	2024300.11	1530013.65	1496565.38
5	114.20	4953.70	42.82	110.10	48.60	77.80	230	52.96
657484.79	1447432.58	601065.25	1331877.59	1461754.89	1420943.32	1458383.67	1103367.45	1512250.71
257914.75	975113.50	479246.75	295511.33	677742.85	971626.18	982439.25	762811.11	367078.90
417283.01	1027923.67	424882.29	849594.91	930734.07	1013523.73	1038454.42	782165.62	857647.14
315556.70	346498.52	271430.34	774310.45	856531.48	330281.85	346339.48	272273.33	1396534.32
3	0	0	0	0	0	0	48.50	0
73985.91	268411.71	106223.74	62802.40	153325.30	268091.37	271750	220601.25	24669.38
49111.77	225484.42	113041.28	22637.92	127138.16	224843.59	229031.08	175276.77	21413.37
377266.21	1070204.59	417225.94	677279.75	955874.94	1076135.83	1084136.79	849776.28	856171.04
17423.37	77932.09	32688.07	7685.35	43234.08	75871.66	78582.75	57326.51	7164.31
946907.41	2568146.33	1068435.89	1554496.48	1811128.02	2552937.40	2598716.83	1989893.36	1074411.95
419634.57	1251102.52	589214.88	667345.09	972548.55	1238545.35	1278511.04	989214.41	674340.09
185482.41	446894.19	206350.63	310472.53	318531.16	446038.77	448282.22	353665.74	180375.27
1555055.92	4714663.79	2242546.18	2389755.97	3573052.51	4643264.56	4760854.18	3580710.59	2442299.97
29719.77	69580.62	51499.73	46522.12	107494.15	70756.80	72876.55	67017.26	142147.35
136259.54	212511.19	93830.52	288392.13	250866.59	206302.17	212704.28	159666.70	293244.69
378362.22	972560.84	433275.51	729971.76	940050.07	957329.38	984989.47	745507.80	919032.80
531.83	2864.52	937	212.67	1490.17	3408.17	2678.50	2940.33	181
31854.39	77313.66	41221.63	89181.04	76892.01	75689.90	77695.60	59016	78477.63
5895.49	28317.73	8353.68	24880.03	16021.83	31480.16	29419.51	30962.81	4228.99
1070470.58	4873422.97	2144632.68	613990.09	2807185.27	4905658.22	4988308.74	3982273.23	532420.74
2436.67	13295.33	3243	1452.67	6833.67	14278	12243.67	10986.33	1223.33
451638.76	1659820.82	615981.87	483439.41	1007108.76	1669815.49	1705605.62	1301856.41	362174.71
439186.55	1124369.46	437007.83	840681.39	1081932.65	1117241.98	1137342.66	872676.78	1066830.06
0	0	0	0	0	0	0	0	0
0	0	0	0	0	0	0	0	0
586391.79	1744383.94	701664.27	983773.32	1016050.80	1749531.25	1746247.32	1415580.98	255831.76
0	0	0	0	0	0	0	0	0
0	0	0	0	0	0	0	0	0

162808.68	466110.15	207150.21	262009.22	385108.87	458192.80	467872.98	349364.25	318109.88
254578.70	621195.41	251473.84	536385.72	609764.56	611415.84	626245.75	475056.05	612539.12
346814.69	618943.57	379069.68	901938.72	1667428.42	596161.38	617041.75	479783.95	2843261.93
68	152	140	48	162.86	108	230.86	188	0
112222.77	314107.92	131901.28	212077.46	415311.81	317024.40	322302.63	263817.28	508259.58
871712.20	1735847.07	780668.51	1960326.52	2180735.47	1713327.39	1749741.96	1343177.38	2696704.63
597145.88	1651639.27	893775.25	936525.66	1682270.50	1621488.44	1669526.87	1251069.13	1722663.73
230868.21	865984.26	375379.38	219230.80	542617.72	877380.18	888684.19	698427.92	217372.91
703265.81	2261046.52	977409.80	1029903.39	1561222.92	2224211.48	2349016.64	1739052.38	841672.72
59201.62	72964.23	104721.17	118885	136505.63	70035.77	68933	57089.42	210029.18
236905.01	236723.66	289750.92	738248.66	2225266.07	223341.71	223902.01	178216.96	4415437.76
7207.37	35526.63	18087.52	3766.61	52802.35	33842.32	36724.64	25799.76	70443.85
9	10	0	0	14	0	0	36	0
644743.10	2128645.93	737876.92	778488.40	1327506.81	2161490.41	2184843.33	1698510.45	538633.92
156815.04	305415.36	123843.30	254906.75	301417.75	300247.74	306853.83	233575.61	298047.36
171692.01	685259.52	249494.71	136939.48	437310.18	684257.93	693712.77	505411.49	198832.50
0	0	0	0	0	0	0	0	0
0	0	0	0	0	0	0	0	0
708109.95	1971635.53	775843.13	1059715.25	1421797.77	1985513.26	2006830.44	1565761.28	869099.44
0	0	0	0	0	0	0	0	0
24587.91	110380.59	43449.34	11797.16	159507.72	110979.91	112567.39	91680.27	212279.62

B10	B11	B12	B13	B14	B15	B16	B17	B18
0	0	0	0	0	0	0	0	0
948153.79	1048842.04	1220518.65	817871.89	968625.05	543342.04	754266.26	262989.06	746550.03
3263960.27	3668043.32	4252245.32	2843630.56	3362174.47	2093322.85	2622436.82	904516.39	2607845.01
652282.89	724181.66	852851.40	557901.38	665780	332180.08	516722.02	183682.30	514936.37
67879.25	81205.14	92196.86	61581.01	72523.45	52321.60	56661.33	20869.48	56575.99
44651.16	51300.78	59225.40	39149.92	45931.36	34742.50	36184.33	10920.74	36153.96
192263.67	214329.76	253701.84	165395.12	196518.86	243724.14	152218.51	71219.64	152111.17
0	0	0	0	0	0	0	0	0
5371442.74	5642794.74	6822871.55	4645368.91	5549295.93	3538662.26	4261352.43	2080181.12	4247649.27
53399.57	54947.64	69608.89	43125.61	52724.21	65979.63	40123.17	24637.29	40692.37
366926.93	425375.76	487940.46	316659.26	378432.35	84957.18	294211.66	72975.18	293198.92
1040610.78	1169700.64	1354294.77	900695.14	1067270.30	466195.92	830992.34	270613.64	825403.76
56747.34	50641	65847.03	42182.32	53592.34	2167	41268.66	9813	40342.34
28414.34	29808.70	32522.85	25733.33	30457.64	33580.97	23430.50	12898.57	22377.86
319	41	81	60	59	0	70	10	59
44651.16	51296.64	59225.40	39149.92	45931.36	26370.83	36184.33	10919.41	36153.96
319967.03	327494.68	399335.18	256744.34	317281.56	489022.98	236741.79	196736.24	237364.07
293947.92	312132.12	377368.32	243955.13	294967.76	246708.78	225541.52	80582.22	226739.31
414805.32	491983.70	562058.44	367555.07	435959.78	38005.29	340804.16	76820.99	338838.71
159.79	109	85.38	89	209.72	9339.34	15.34	2928.66	133.62
471679.14	534570.16	629555.65	406661.30	487920.35	165246.29	379142.65	129723.77	377979.44
0	0	0	0	0	0	0	0	0
556160.02	624865.32	724136.70	482603.04	571102.81	342313.35	444957.81	148491.89	442358.13
2049404.54	2413415.64	2781367.79	1805552.29	2127915.62	869909.15	1665009.92	597030.81	1674651.57
118476.86	144986.92	163843.81	107610.41	125337.46	18397.83	98805.16	21081.71	98149.19
0	0	0	0	0	0	0	0	0
370581.74	413673.24	482265.11	322170.38	382047.04	218607.10	296675.50	126461.26	294669.26
183996.85	209871.84	241299.90	162578.44	191542.30	98216.49	148925.98	58241.97	147780.64
184870.92	208841.34	241409.68	158390.25	189264.70	93114.19	146650.98	45524.11	146550.04
650253.99	735798.30	846320.59	568771.37	672412.64	161052.47	523334.97	186134.65	517808.56
830439.07	973832.80	1109801.44	730849.01	863235.99	108615.65	674145.10	180095.69	668468
0	0	0	0	0	0	0	0	0
26964	34431	37880	23841.50	28023	976	22084	4574	22007.50

677043.64	760354.50	873935.93	599166.82	714117.13	100673.83	555289.46	151069.75	545665.62
0	0	0	0	0	0	0	0	0
5105	5949	6171.10	4470	5304	2059.47	4206.67	3496.87	4226.50
7767.27	6362.14	9136.64	4914.33	6518	2016.47	4906.67	2948.87	4872
72892.72	74590.50	91300.78	57897.85	69294.48	25322.66	53445.97	37772.24	52723.07
21544.01	23077.28	27189	17963.93	22268.29	83086.01	16468.67	7933.01	16730.47
15376.33	15488	17762.83	15090.42	18235.16	3997.83	13843.17	2676.17	12820.67
2005355.74	2211619.32	2557397.29	1727659.35	2050038.06	1249775.35	1589762.60	646091.33	1571704.26
1939316.42	2151778.24	2488324.80	1685868.59	1994127.13	1096579.10	1539159.79	635782.78	1531739.61
0	0	0	0	0	0	0	0	0
2343.97	1005.14	3086.71	1472.68	2062.95	44662.83	669.34	3495.61	1262.58
1147.60	1070.28	2460.20	527	1248	15	900	276	997
56747.34	50641	65847.03	42182.32	53592.34	2167	41268.66	9813	40342.34
0	0	0	0	0	0	0	0	0
402190.54	451257.62	516573.10	350249.63	416046.08	166158.96	322442.14	109368.20	319418.05
220913.96	248266.20	287983.48	190961.10	226510.69	85960.47	176706.51	60285.73	174747.26
254140.23	277084.84	329508.97	214830.35	257821.30	173631.61	200351.34	72780.51	199010.11
190367.70	219176.20	254913.49	167009.35	197869.91	107846.57	154460.33	51875.75	153808.55
0	0	5	0	0	0	0	0	0
44777.16	51510.14	59279.90	39149.92	46006.36	26706.83	36184.33	10953.41	36153.96
47.83	59.50	36	30.50	52.67	1	15.33	11	34.17
0	0	0	0	0	0	0	0	0
286526.95	328770.92	375270.69	250976.57	296688.11	77127.11	230917.85	54099.07	229670.17
1655917.14	1869646.20	2148708.76	1440111.57	1697216.02	831762.36	1325618.14	387726.98	1318204.03
59973.10	64715.14	74507.61	49882.41	58959.28	27076.83	45902.16	13951.99	45212.07
808929.07	898175.22	1046192.95	695753.96	830597.92	245106.63	643683.19	211768.61	638021.80
68522.44	77853.42	86362.65	60215.42	72450.47	11112.46	55297.33	13063.98	53481.75
562458.74	637712.96	736677.83	486287.12	576130.86	265401.15	449025	149032.33	446467.97
7948.99	8504.50	9672.89	6979.34	8580.77	22443.83	6290.17	1747.37	6211.36
531586.71	607551.56	688732.58	461536.51	542629.35	115485.58	425199.98	103435.98	421531.44
143707.09	150179.28	180304.03	118039.24	143049.48	33697.33	111452.49	29464.11	110266.50
3963.57	2871.50	4301.68	3096.68	4221.66	15675.16	2480.17	5222.98	2601.41
148879.37	166178.06	194286.13	127142.26	151708.05	63621.94	118098.34	38780.54	117631.60
56747.34	50641	65847.03	42182.32	53592.34	2167	41268.66	9813	40342.34

12	0	5	0	0	0	0	0	0
322853.24	351805.34	415124.68	275664.02	330272.53	77997.10	255136.03	78898.92	252408.54
656935.33	732422.14	855955.66	564011.74	669917.76	391581.14	520817.29	226430.16	517904.30
115905.53	136790	153671.79	100374.37	118755.10	56471.13	91002.84	19196.47	92708.20
28452.76	30552.42	34072.20	23230.17	28076.06	29108.97	21425	9976.69	21001.13
27563.69	26126.64	33044.02	22613.25	27752.67	35574.80	21056.50	10129.52	20868.61
164257.76	169721.20	201706.34	146530.52	176477.89	116352.74	133858.51	61958.86	131451.03
89450.93	90647.50	111572.56	72063.99	88627.34	25122.83	68986.49	15595.17	68276.05
0	0	0	0	0	0	0	0	0
0	0	0	0	0	0	0	0	0
1174171.28	1311551.04	1515933.91	1025196.11	1218902.53	335461.14	946298.27	321993.58	936030.93
134417.02	154225.48	182520.87	115269.15	139427.21	23154.46	108762.64	27484.62	108337.23
194406.12	198507.28	236373.06	160642.07	193970.95	54265.27	150672.98	43544.07	147801.23
528593.66	580119.46	691932.31	449124.17	538002.77	456517.49	416627.17	171039.01	415686.35
150903.24	152939.42	177430.15	121102.18	143386.33	67740.92	111159.98	37098.50	108705.71
1110446.33	1216368.20	1426165.87	966332.73	1155148.59	271487.63	899242.57	255175.15	887198.75
3183322.76	3566177.72	4053490.65	2732334.59	3231178.25	1085802.81	2509408.94	716739.93	2482564.12
77818.58	87771.64	102478.43	66661.09	78645.06	42485.76	61346.50	17996.06	61478.35
1055418.88	1178933.14	1404895.97	911341.02	1089750.64	473151.16	848810.60	333765.52	846028.63
362812.37	412396.96	472807.41	324310.06	382999.22	74794.90	299357.48	83877.42	294480.25
95079.79	113319.06	127446.34	84490.58	98591.92	16579.14	77213.01	21183.06	77286.72
43513.26	48890.14	57103.70	37500.67	44413.06	43902.97	34682	12680.07	34515.23
919497.55	1060575.12	1229265.56	796342.72	947629.65	299967.84	739386.66	185416.22	738033.52
1503	1645	1615	1195	1325	58	1020	234	825.67
12	0	5	0	0	0	0	0	0
3477065.71	3668534.48	4450487.03	3053303.90	3637429.29	1549728.24	2799648.36	1043502.56	2785801.47
170658.66	214148	243591.68	150155.31	177197.30	12039.83	139598.81	29892.37	140591.55
1081.93	993	2566.13	632	1335.29	4468.67	927	278.33	1087.62
42308.59	48241.64	55226.60	36707	43525.78	5159.50	33999.50	8731.70	33770.20
505397.92	568724.10	654369.79	445340.16	531171.84	71259.41	412461.31	117323.43	405185.65
5998.70	3274.90	6418.90	3376	3335.75	132	2479.50	505.50	2514
2358811.26	2660395.34	3100584.55	2037642.83	2412766.19	1775425.48	1883887.19	690331.76	1875200.01
0	0	0	0	0	0	0	0	0
113006.27	122482.04	143788.04	98575.39	116721.99	81253.58	89693.98	58196.78	89136.98

703386.39	791700.80	919498.17	606531.71	719054.84	402665.10	560437.18	196285.58	557440.39
7785	8880	9291.50	6910	8370	366	6437	1179	6234
1146935.36	1273219.28	1482908.82	986658.93	1169580.23	924396.45	910556.85	331034.71	905328.89
211.83	81.50	41	30.50	52.67	1	15.33	11	34.17
0	0	0	0	0	0	0	0	0
395571.51	442945.26	525949.23	338786.19	405866.09	266092.92	316102.83	108955.60	315237.04
0	0	0	0	0	0	0	0	0
9645	10727	11241.60	8308	10034	2307.47	7706.67	4173.87	7327.50
56747.34	50641	65847.03	42182.32	53592.34	2167	41268.66	9813	40342.34
96953.38	109726.06	122448.23	87071.84	103566.51	43312.33	80257.33	20761.07	78752.49
0	0	0	0	0	0	0	0	0
6987	7889.50	8115	5828.75	6738.50	22026	5253	1022	5160.20
271262.90	311349.26	362197.76	235715.74	279964.17	59691.92	218459.82	69949.64	216896.83
1247262.52	1380468.68	1605779.38	1068067.94	1268721.27	438477.85	988660.96	285007.97	980460.20
1056949.43	1143538.92	1347661.44	894525.25	1065713	619240.51	827334.89	298241.67	819804.37
1824393.25	2033380.92	2359617.37	1578161.40	1869879.30	644184.92	1459042.95	411461.02	1448090.16
31410.75	38336.14	44145.56	27154.17	32858.07	4383.83	25422.83	5318.37	25307.53
820804.10	878457.32	1038314.03	678554.16	814392.94	839185.31	626699.32	366022.35	622866.17
1052472.35	1112892.50	1305502.77	889189.72	1057361.43	492136.40	820186.77	279868.28	808641.05
218105.02	260924.50	297088.57	189692.99	220610.43	167973.23	174468.84	56535.55	174761.90
212629.80	244889.34	282049.55	188828.58	225017.47	25694.84	174997.49	49550.38	172470.56
4990055.99	5669632.66	6553549.94	4335656.52	5134886.89	2382071.54	4001927.20	1328854.72	3982436.52
2490384.34	2796090.88	3280127.88	2151771.13	2560460.61	1673772.07	1993572.36	712180.31	1987457.77
16339.37	17789.64	20105.24	15222.83	17879	707.67	14041.67	4325.33	13695.67
4442	4712	6397.74	3275.33	4527.44	3341.83	3088.17	759.37	3154.36
142.50	12	13.38	14	33	0	0	11	22.62
5785.22	6621.50	6674.19	4874.18	5948.03	75422.46	4451.09	68373.24	4503.65
246814.44	260793.70	306582.96	210706.40	255089.94	185080.99	191480.54	96840.69	189451.19
0	0	0	0	0	0	0	0	0
131984.75	160410.64	184695.69	113863.67	135153.37	21500.17	105808.51	30386.97	106176.96
377929.62	415107.54	479426.29	315285.93	375641.02	162081.90	290078.37	83136.80	289061.32
135.10	21.64	22.20	23	55	2	8	13	33
12	0	5	0	0	0	0	0	0
12344.99	11484.92	16510.54	9183.34	12701.10	40459.80	8926.34	3869.90	8681.05

808140.50	921242.26	1062856.16	704184.85	840839.29	226474.11	654168.49	166025.43	648285.68
0	0	5	0	9	0	0	2	0
19972.92	21613.50	24815.06	17110.17	21557.40	4138.83	16197.50	3104.37	15751.86
0	0	5	0	9	0	0	2	0
96081.83	121422.64	136648.97	85050.75	98824.03	14155.83	78199.50	16960.91	78763.34
484824.47	537297.38	627890.47	420155.94	499948.53	289185.12	388926.99	133388.98	385995.49
971751.14	1099731.16	1277663.62	843417.67	996043.19	682012.68	778279.50	276632.20	775700.82
233802.67	258201.20	298445.37	201686.37	238278.69	106249.92	185226.16	63796.56	183655.73
1065322.05	1197029.10	1404778.44	924037.25	1100199.69	496865.22	853975.81	286350.04	851915.45
661044.79	734356.56	852494.80	573449.54	682623.90	343000.52	530806.49	175144.80	526636.91
154196.18	160786.06	192839.44	129988.77	156959.30	72412.89	119635.68	38783.35	119386.95
2062881.78	2323098.06	2687946.24	1782900.88	2108963.81	1055559.73	1644434.31	540022.79	1637701.74
0	0	0	0	0	0	0	0	0
85567.28	96979.42	111616.23	74058.93	87801.07	33666.64	68266.84	30946.76	68018.25
6518.50	6704.50	7429.34	4944	5898.25	12454.67	4458.50	967.37	4537.60
1626333.79	1832189.22	2137298.13	1399595.74	1660011.62	980072.87	1293030.64	476562.53	1287247.72
80936.31	93902.92	107553.77	71121.76	84162.23	20608.26	65284.33	23545.64	65327.59
575632.96	663310.24	768553.55	495365.46	588095.47	335967.12	458014.83	143372.26	458760.91
1422080.42	1587318.14	1844169.90	1238338.32	1469687.26	604313.03	1144425	409862.87	1134769.14
204047.36	214112.06	255105.69	167847.57	202278.04	109911.54	157232.16	50935.58	155625.47
20985.80	21937.42	24803.94	17459.66	20980.84	884	15855.66	2998	15159.34
431593.87	491905.62	561361.67	374357	445908.34	122092.40	347604.33	90380.56	344373.19
1698950.12	1891950.40	2208962.46	1466288.85	1736607.11	1392514.55	1352566.85	524714.53	1345629.13
948015.90	930860.98	1096213.92	812316.26	963977.78	171191.88	741744.72	264634.64	718183.23
10232.49	10621.28	11955.54	8626.34	10303.77	31466.63	7538.17	3443.45	7415.25
410347.82	487529.06	555564.27	363949.24	431060.11	37789.29	337126.83	75756.91	335236.87
245946.22	261613.92	304806.01	207649.90	249464.18	46551.30	194037.99	48719.48	191014.70
11073.18	11655.14	14167.29	9066.59	11656.63	29635.14	8405.67	3961.60	8360.56
678123.63	761358.22	887436.72	587390.90	698802.51	398307.11	543333	198589.69	539869.56
736280.89	820530.62	949730.99	629176.73	743719.09	306534.32	577615.21	137385.18	577618.13
336078.69	392890.06	443278.20	291992.73	346797.80	73021.30	270052.48	67048.84	266906.53
0	0	0	0	0	0	0	0	0
558554.96	657394.82	750325.14	495275.01	580942.50	140614.74	453656.49	141459.92	454192.74
57957.29	63878.06	78530.61	49007.26	59791.28	51152.80	45842.97	18426.48	46062.74

0	0	0	0	0	0	0	0	0
85837.58	98039.14	109780.23	75607.42	88851.16	12607.16	69146.84	15959.44	68076.08
40291.09	48779	54531.47	34768.50	41680.98	10621.17	32251.83	6697.03	31688.58
46049.93	52427.28	61476.04	41572	48796.75	5848.67	38598.50	10428.37	38174.61
112354.10	131564.14	151225.29	97147.31	116404.58	12421.30	90698.49	21286.24	90042.21
439517.19	492535.04	575789.91	376986.22	447380.88	195488.02	347874.66	123444.94	346538.64
99524.52	97149.28	114598.78	83493.51	99581.28	74687.52	76739.13	32841.60	74813.67
451118.91	504563.54	579625.77	398142.17	472334.39	155800.49	366914.96	108096.67	361476.85
980989.79	1094882.66	1271629.72	854206.99	1010195.09	591404.73	784351.48	312885.65	780081.64
26919.69	31139.14	37194.13	21766.42	26548.61	4132.50	20626.17	6174.70	20750.03
614676.50	669923.80	800085.88	523350.21	628437.58	536232.23	484492.69	220392.79	485028.36
384997.71	432216.32	509958.22	335345.56	397916.42	294813.72	309700.33	124616.68	308916.67
286370.59	329659.84	381417.85	248845.36	294911.48	149022.12	229770.34	71483.44	229693.43
96849.70	104156.20	119259.11	86708.93	101449.17	104018.98	79333.52	60688.24	77266.50
67389.76	79549.28	90114.23	60009.42	70272.56	9092.66	55011	14897.54	54763.49
84504.99	93679	110237.84	73248.66	86651.12	3115.84	67877.50	19329.36	67459.61
25656.42	24768.14	29709.37	21450.92	25934.19	20294.63	19866.99	8401.03	19332.97
46382.93	53710.64	60672.10	40835	48315.15	4821.83	37817.17	7958.37	37580.86
1235912.41	1389643.40	1623105.52	1075872.48	1279704.37	591562.76	992303.53	370217.94	987987.31
70692.50	71272.50	81088.52	61738.99	73261.51	2979	56973.99	11900	54906.51
590481.85	634076.66	732439.54	503063.67	596081.49	258928.48	462683.78	172104.28	456777.19
150579.34	169193.28	199036.59	131561.32	159588.63	15294.32	123208.32	26229.28	121470.28
1013532.19	1137807.04	1331192.56	871400.45	1037326.60	652569	806765.95	296296.18	803015.35
945404.40	1061129.22	1235325.38	816721.48	968382.08	432974.58	754363.33	255284.12	750394.80
7974.59	8514.64	9690.59	6999.34	8596.77	35880.83	6290.17	1747.37	6234.36
967	973	2410	527	1196	15	900	270	991.67
883923.82	981075.42	1143939.94	762454.62	908186.98	486323.82	705467.94	284257.14	699310.03
3530258.11	3787906.62	4380593.33	3032400.25	3592724.11	980891.52	2791016.60	842698	2753508.13
127439.84	121913.50	146935.55	103921.31	126853.85	5146	98242.65	21713	95248.85
156051.13	171774.34	198744.78	137187.67	162607.28	87123.24	126292.17	42077.24	123948.64
87512.15	97720.14	112089.03	75120.09	88198.04	37768.80	69074.16	23322.64	68453.56
35449.67	35063	49405.18	27992.33	38468.50	1683.35	29118.35	13765.65	28599.01
1850795.99	2094296.92	2422085.19	1603634.51	1893713.49	1100568.85	1478966.17	498837.17	1474033.52
67560.98	64255.56	74950.74	60329.78	69826.74	88176.23	53428.85	29521.91	51979.11

1147406.51	1285610.48	1505293.59	988444.76	1172096.04	800247.54	914702.49	338438.10	910039.38
173811.64	195654.20	228038.60	150010.93	177713.94	127609.02	138735.67	52755.94	138060.92
37657.97	41688.50	45381.46	34574.44	40890.95	49246.45	30632.02	7974.55	31075.08
63440.35	74895.78	84437.02	54841.18	64298.66	56596.94	50228.83	24491.18	50098.78
56747.34	50641	65847.03	42182.32	53592.34	2167	41268.66	9813	40342.34
10322.34	10549	11358.84	8546.66	10036.33	454.67	7724.67	2967.33	7339.67
89586.01	97368.64	115436.36	77071.84	90761.55	62526.14	71065.33	27260.68	70664.17
670411.78	738258.82	861401.65	576903.05	683950.61	491553.31	532932.51	197124.45	528827.58
340358.26	380041.90	448274.55	285839.49	342053.43	144533.10	266658.64	86802.12	266019.37
1072017.41	1192882.88	1379326.79	928107.59	1106975.33	418801.36	859605.79	245916.22	849698.94
0	0	5	0	0	0	0	0	0
1355634.14	1530674.22	1755971.64	1177145.53	1393761.96	471801.73	1085873.17	265329.01	1080285.53
824.75	154.50	922.52	422.84	731.26	22206.33	120.67	222.41	422.75
26496	26253.50	36592.17	21684.50	28491	1228	21852	4964	21851
225362.72	248871.90	290024.56	196967.69	237417.38	113264.86	182768.97	64981.06	179694.72
0	0	0	0	0	0	0	0	0
0	0	0	0	0	0	0	0	0
26927.99	34128.12	37986.93	23858.51	28094.31	8893.85	21993.51	18552.97	21937.74
337100.67	388516.90	441130.86	302732.81	358942.72	33138.74	280308.13	79994.32	275586.67
114659.86	125195.28	140570.62	97374.24	115510.06	37252.33	89413.83	20619.57	87413.58
0	0	0	0	0	0	0	0	0
155571	171914.06	200338.27	133927.61	158631.34	79610.63	123249.48	56979.67	122318.43
0	0	0	0	0	0	0	0	0
119164.63	124282.70	146164.24	104325.76	125522.17	46975.98	95724.17	22369.46	93373.08
402987.78	442942.52	518335.15	351428.07	418091.27	232319.33	323940.99	134988.51	321195.40
152091.67	168755.14	194393.73	133685.26	157460.44	52408.79	122936.66	37677.35	121582.08
71208.07	82305.78	97918.43	60724.68	72676.19	58302.66	56338.64	25208.90	56544.56
44651.16	51296.64	59225.40	39149.92	45931.36	26370.83	36184.33	10919.41	36153.96
116956.57	134076.06	153770.73	103295.17	123885.28	46292.97	96242.50	28253.51	95422.70
839561.07	810134.48	981019.42	705983.35	843851.27	127839.57	653251.39	210254.37	637560.84
365824.12	405834.96	472899.12	315430.02	373907.04	283594.22	291338.15	111138.10	289006.21
516664.05	616340.20	693990.06	463825.55	543821.10	34883.49	427430.12	96321.51	424711.37
566183.10	631503.50	727820.28	487026.93	573471.87	219509.26	447599.01	97102.94	447687.69
773897.15	858361.72	1000480.65	665190.07	787010.31	602859.37	613081.50	234104.49	609760.41

44689.20	28451.64	56521.44	29107.78	29988.40	24202.28	21211.23	121397.54	21506.68
18954.36	24958.64	29508.70	16303	19592.96	568	15552.33	3438	15987.67
56747.34	50641	65852.03	42182.32	53592.34	2167	41268.66	9813	40342.34
0	0	0	0	0	0	0	0	0
1142015.94	1283875.64	1487406.91	989899.92	1168718.76	647317.57	911508.83	301672.43	906090.42
22	4.50	14.50	0.75	14.50	6171	0	0	7
820031.80	919158.34	1069834.32	709486.68	840717.30	599039.73	654678.34	250743.25	650875.25
560768.82	611972.40	718547.75	482641.96	577726.42	165111.04	447620.97	136403.36	445400.07
582535.16	656627.38	762689.78	503799.45	597863.66	311860.65	465764.17	155871.17	463026.61
199900.68	208029.54	251506.41	163625.70	197235.55	382901.32	149994.83	148092.72	150657.74
164	22	0	0	0	0	0	0	0
158219.95	168007.28	200120.04	130775.49	159491.73	14024.13	124060.66	28721.07	122835.19
131574.97	142516.14	163405.70	114640.77	134952.62	40922.81	104409.67	25432.83	103598.64
603056.86	660610.88	790741.61	513252.43	619789.51	333386.36	480234.98	160275.40	477206.68
44651.16	51296.64	59225.40	39149.92	45931.36	26370.83	36184.33	10919.41	36153.96
1452849.50	1630658.02	1892751.53	1262107.97	1496041.01	649083.28	1166682.47	384618.40	1155051.90
742164.86	812144.54	934881.75	628712.20	744072.58	228262.72	578843.35	169254.08	574089.05
254649.32	274488.98	323189.54	220243.44	262873.75	79358.38	203446.32	74853.26	201355.08
2689742.28	3042417.36	3522821.83	2326861.39	2760373.94	976970.18	2145719.78	637603.60	2139175.01
40832.43	38238.06	46598.65	32653.33	40880.66	43553.94	30613.17	17036.10	29212
120878.97	136863.70	160224.62	102968.35	122847.65	120633.02	95323.01	43353.48	95346.88
557541.99	622038.18	723714.69	480273.13	568285	395925.82	442842	156557.20	439927.50
1203.83	1056.50	2512	585.50	1387.67	16	929.33	311	1089.17
43958.60	49065.64	57249.88	37682.33	44637.06	42286.97	34781	12716.07	34694.63
15685.15	14934.64	19857.09	13358.50	16510.67	592.66	12389.99	2729.34	12244.19
2865690.65	3003884.50	3478369.03	2470753.84	2912036.75	438347.59	2268368.66	674330.41	2223691.74
5997.67	6198	6959.67	6940.33	7504	307	6067	1199	5763
925000.98	1062401.44	1210869.48	815455.38	967586.88	154195.20	753280.79	204373.96	742321.39
631806.76	711321.02	830476.69	547855.20	651969.41	373278.17	508715.32	182650.27	504487.07
0	0	0	0	0	0	0	0	0
0	0	4	0	0	0	0	0	0
994104.68	1055063.68	1277284.31	848267.66	1015754.47	481596.27	790498.87	286821.59	780112.09
0	0	0	0	0	0	0	0	0
0	0	0	0	0	0	0	0	0

264148.59	303146.06	355191.48	227341.77	270878.73	146886.86	210682.84	60531.34	211287.53
352538.75	394811.26	462126.63	303280.69	360162.48	261401.09	280752.33	100789.23	279518.68
355930.02	386908.96	463860.43	297712.47	356806.35	469980.62	273703.91	280246.34	275433.23
122.86	48	60	56	143.74	0	0	44	84
186456.64	187834.62	226212.86	153232.95	185955.05	148327.39	142198.97	64681.13	140684
981890.13	1093880.32	1277894.14	845706.43	1007242.59	629632.79	780516.99	380528.61	778085.72
944366.92	1080086.98	1251565.61	811233.70	963112.78	681124.88	749607.88	276595.92	752165.53
487871.12	544712.74	628534.01	426124.06	506964.91	150392.57	393416.13	111674.11	388466.32
1313134.39	1495159.10	1689361.31	1122461.62	1336740.92	518947.28	1032709.98	281807.80	1022852.46
39259.66	41909.48	58594.98	30633.44	39907.18	82289.22	28809.84	18765.64	30840.51
132574.70	131165.26	164413.53	109748.07	135948.71	406164.54	98377.71	374081.82	103118.95
20263.49	22763.50	25797.94	17529.76	21459.27	42770.17	16231.50	7038.57	16186.98
0	0	5	0	0	0	0	0	0
1181963.24	1325788.76	1518869.57	1042172.44	1241014.03	147209.92	965999.89	253084.26	947571.48
173730.24	194880.76	226040.75	149487.68	176888.99	86167.22	138343.84	53875.52	137685.74
375872.91	455435.20	513351.88	340973.83	396709.33	57610.79	313278.33	87090.53	313185.94
0	0	0	0	0	0	0	0	0
0	0	0	0	0	0	0	0	0
1100742.48	1229605.18	1424111.75	963602.72	1145817.43	328482.47	892787.10	278185.25	880387.77
0	0	0	0	0	0	0	0	0
64861.16	68534.78	80989.22	55320.67	65637.67	22674.13	51051	16418.69	50256.10

B19	B2	B20	B3	B4	B5	B6	B7	B8
0	0	0	0	0	0	0	0	0
721680.58	1479574.87	758930.10	1728062.32	1602472.38	639481.56	1621084.61	1647589.61	1720910.12
2511161.14	5160830.28	2631105.82	5978945.47	5608603.56	2217858.56	5641950.84	5751991.92	5944607.17
501065.45	995294.39	520161.74	1176476.06	1117011.82	434686.93	1118456.54	1147856.58	1179195.01
53052.45	113732.78	55127.04	129619.79	124778.84	46314.33	124212	123472.76	126270.33
35527.76	64614.25	36458.02	80141.06	75368.87	32199.33	76061	81788.08	80567.83
148716.79	291334.21	152390.49	344571.15	332101.37	132449.13	327429.02	337303.03	351505.98
0	0	0	0	0	0	0	0	0
4172567.10	9770573.88	4366611.25	9902966.33	9176574.29	3628529.37	8997284.29	9235146.83	9457782.95
40884.82	76072.10	41052.30	90852.04	90098.58	34190.81	86812.51	92201.67	93888.70
284591.73	502498.92	290844.27	664182.10	637779.95	249555.97	641141.24	662025.30	675988.49
796186.15	1591664.81	833605.02	1893797.21	1784491.12	696623.58	1797710.43	1826407.87	1886491.70
41208.68	80926.34	44803.02	96427.02	84623.66	32028	88740.32	92710	99160.32
19980.37	57465.36	22444.19	57143.75	50167.38	19342.50	50245.49	46211.74	55334.84
49	77	23	68.50	252.33	214	99	515	205
35527.76	64594.65	36434.02	80141.06	75359.87	32199.33	76061	81788.08	80562.83
240971.16	470940.77	241680.42	551199.55	526097.35	210150.36	510754.04	541827.03	583717.98
225355.47	431260.41	230277.94	512703.08	492235.51	195563.11	484215.62	507505.46	519804.99
326297.70	604097.56	332964.09	768674.25	740473.77	287448.15	742802.55	749917.47	767817.39
69	384.70	24.53	137.90	565.19	527.34	164.87	398.91	225.36
367601.65	721548.78	376118.45	861093.69	830593.57	312290.61	825977.63	833607.71	856600.67
0	0	0	0	0	0	0	0	0
425011.17	856813.04	446784.43	1016465.79	951765.74	376062.78	961204.52	978299.48	1017457.82
1627151.24	2961797.48	1659469.50	3706163.44	3611171.70	1431027.42	3601833.84	3760327.73	3742190.05
93648.40	181259.72	97228.22	221941.03	215462.90	82751.83	216539.53	217842.84	219599.70
0	4	0	0	0	0	0	0	0
283222.31	597787.64	298079.28	682007.62	641862.70	248096.47	643379.76	648386.30	674608.60
142646.12	290496.48	148737.38	340089.47	319056.02	126784.31	319452.50	326190.13	333987.73
148166.49	256225.79	146450.26	323228.09	309794.82	137074.64	304924.82	335838.06	330380.85
495560.19	1019970.96	522606.10	1205476.96	1123432.23	437534.30	1135481.01	1136783.50	1189670.80
637800.49	1290217.86	669843.27	1545813.61	1473154.27	547754.14	1487078.19	1482819.81	1518877.61
0	0	0	0	0	0	0	0	0
21331	30182.17	21294	48347	48169.16	19202	48914	50266.34	51516.67

517467.16	1158635.77	548856.71	1296543.23	1194931.20	447648.49	1203338.30	1176756.73	1240558.41
0	0	0	0	0	0	0	0	0
4312	8027.20	4222.83	8842.60	7606.47	4596.67	7807.80	10896.34	10158.94
5631.67	7790.67	5774	10858	10416.83	4560.67	10458.33	12255.50	12769.54
55145.53	95162.10	56783.48	120924.64	111790.86	46019.97	113255.75	120015.56	123064.80
17464.41	31982.11	16428.11	37395.44	34827.71	16786.31	33145.76	41383.32	42872.16
10040.99	41717.07	12679.17	36666.50	32407.87	8515.83	31883.50	24032.17	32697.76
1519491.85	3120519.45	1599193.27	3655255.59	3378821.74	1353346.14	3415007.21	3467206.57	3655767.61
1487974.89	3052851.63	1555171.50	3529595.18	3291437.23	1331822.15	3302987.81	3407244.41	3526780.92
0	0	0	0	0	0	0	0	0
1871.37	2799.11	1147.29	2400.10	4325.76	1301.32	1601.65	3323.13	3270.63
1287	1008	640	1659	3837	268	2164	1736	1622
41208.68	80926.34	44803.02	96427.02	84623.66	32028	88740.32	92710	99160.32
0	0	0	0	0	0	0	0	0
309851.95	598422.69	321833.19	733397.20	679824.35	280855.78	686679.64	717129.39	739988.84
169334.89	332329.41	177493.26	401819.85	378728.06	148236.15	383022.82	388888.04	403189.32
193110.56	393462	201941.43	458164.75	433115.22	164830.99	434487.15	437371.44	458771.26
146712.63	303028.31	152939.53	353536.10	337769.72	125425.65	338414.07	335498.63	347661.64
0	0	0	3	115	0	0	0	0
35608.76	64851.65	36631.02	80211.06	76136.87	32229.33	76177	81821.08	82375.83
12	80.33	18.17	66.51	98.67	121	57	187.50	33.66
0	24	0	312	0	0	0	0	0
221012.28	437946.08	230487.50	525588.58	497537.18	193893.14	500116.34	509802.45	520681.89
1277127.46	2541890.89	1338482.27	3008201.53	2811406.70	1131735.01	2845300.74	2937691.94	2998292.91
44849.17	86712.45	47743.20	104598.05	95098.38	40964.16	97403.49	101676.28	107303.36
616808.74	1257222.18	646883.74	1480543.60	1383109.81	536720.43	1393132.98	1417176.74	1471571.09
50889.15	110403.21	53803.36	131409.96	119661.55	46048.32	121876.89	117045.52	134308.86
433217.59	827629.81	450402.03	1015823.19	962828.56	382718.29	970596.82	995372.50	1023134.12
5926.02	12399.48	6058.85	15018.64	13362.60	5500.83	13327.37	13715.84	15748.10
411796.10	770605.29	428918.60	956370.24	892706.19	371127.63	908810.64	945607.09	966154.36
109372.80	209265.16	115240.56	253712.90	233107.96	92639.83	238464.02	249905.26	256519.18
2528.07	6148.17	2407	7034.92	7526.39	1969.16	6201.11	6343.09	7565.93
115543.67	216465.49	118998.61	266711.57	251485.47	101240.66	252864.45	262738.08	268466.54
41208.68	80926.34	44803.02	96427.02	84623.66	32028	88740.32	92710	99160.32

0	0	27	3	115	0	0	0	0
245876.77	507452.80	257826.08	587373.55	550580.39	211661.30	552805.78	560801.17	585176.79
501851.40	1011583.03	525117.35	1191512.51	1117641.77	438602.95	1126538.95	1150249.90	1193401.51
93021.39	139651.92	91291.09	199091.71	194901.55	86800.13	192649.81	213972.86	208792.74
21799.76	37016.19	22019.35	48977.59	42729.73	21309.33	43197	47829.14	52250.50
20420.79	50420.65	21574.54	50021.57	45428.02	17566.16	44159.65	43914.48	48141.96
125222.37	343314.97	134038.05	324970.27	291569.41	107679.80	285318.70	272886.04	293023.15
67918.47	131910.64	71667.03	158038.01	144577.36	55096.83	148370.82	153833.81	158332.87
0	0	0	0	0	0	0	0	0
0	0	0	0	0	0	0	0	0
896177.88	1919808	945537.94	2191192.27	2036624.24	774994.91	2051378.35	2047162.93	2134112.48
107341.04	202646.58	107288.78	244481.25	238800.95	90844.66	235114.93	244101.43	240036.08
144461.44	307403.28	156405.14	349781.58	311991.89	123873.33	322037.55	329255.06	348149.10
404607.32	808874.62	418066.48	950610.90	908813.96	346579.75	904224.64	924192.45	954453.13
110235.29	199189.13	119446.75	251111.37	223063.55	102125.98	234460.27	255597.79	269063.42
855175.22	1898529.34	902921.16	2081396.92	1923717.80	732252.28	1929128.36	1932324.95	1996946.70
2419788.85	4622284.22	2538813.35	5713544.27	5273976.41	2195613.83	5386113.75	5604298.78	5785572.19
60496.81	118894.14	62748.72	137891.52	132301.91	52403.48	131999.42	138189.10	138488.67
827226.89	1667827.85	846335.21	1923433.39	1846913.23	706416.21	1828534.40	1855683.70	1900737.49
281730.09	622238.37	294942.33	688028.38	643130.26	250799.82	643871.20	644777.06	662383.84
73617.39	138339.82	76452.20	173073.32	168023.62	67096	168826.73	173977.80	175884.58
33295.16	66742.59	34845.85	78866.66	74494.70	29197.33	75031	76404.88	79202.04
717981.97	1316341.18	735453.27	1660429.89	1603148.52	623716.92	1608798.41	1651560.07	1679411.15
1136	2154	1208	2313	1899	1304	1997	1768	2693
0	0	27	3	115	0	0	0	0
2715412.90	6597081.54	2852947.92	6531102.04	6047022.92	2333936.13	5917034.25	5959040.22	6081506.07
135612.05	216790.85	135723.52	307269.69	312842.02	116555.83	312127.26	319532.72	314590.77
1399.50	1080.67	692.41	1737.33	3396.97	268	2139.87	1866.07	1776.64
32636.36	63744.17	34003.52	77141.82	73009.43	28412.50	73662.70	75407.18	76796.03
383297.57	854487.50	409061.99	966267.02	893391.07	327770	902490.86	872879.44	929433.37
2211	4601.23	2409	5673.95	4653.85	2626.48	5719.10	3006	6873.50
1809094.78	3609203.71	1890762.19	4281234.11	4050529.48	1585891.66	4077619.98	4150478.63	4299187.59
0	0	0	0	0	0	0	0	0
86778.26	195405.12	91642.29	208523.01	194537.02	75344.31	191915.87	195515.30	201497.82

538065.95	1070384.01	562856.47	1275148.46	1203649.08	472177.12	1212982.63	1235014.66	1278894.56
5645	10548	5979	15276	12944.83	5464	13702	13548	15618.50
875088.20	1776191.93	919465.45	2079130	1944862.83	770427.37	1962806.17	2010182.29	2084848.37
12	80.33	18.17	69.51	295.50	121	57	187.50	33.66
0	0	0	0	0	0	0	0	0
305540.11	604121.37	315045.66	719250.89	690038.71	261485.80	688080.03	693803.52	719792.78
0	0	0	0	0	0	0	0	0
7051	16413.20	7540.83	18133.60	15431.97	7036.67	16092.80	17484.84	19704.94
41208.68	80926.34	44803.02	96427.02	84623.66	32028	88740.32	92710	99160.32
74993.75	169643.65	78832.09	188906.50	168144.56	67334.33	169581.27	166940.72	177493.52
0	65	34	4	0	0	2	40	63.50
5400.80	7906.54	5367	11364.80	9780.70	6027	10027	12270.80	12881.60
209352	409498.67	217677.33	496289.71	475318.14	183425.51	477135.65	483901.92	495977.01
953771.50	1879217.90	1001683.05	2248796.67	2103646.83	833700.57	2131621.50	2186918.52	2255455.11
804474.77	1625482.70	846753.71	1890533.51	1764085.52	697315.50	1780365.78	1839292.73	1891006.33
1399124.24	2837405.34	1463455.10	3323375.03	3110260.76	1231414.23	3134025.63	3190135.06	3292206.58
24568.69	36871.40	24181.31	56757.32	57632.53	21207.83	57170.37	56577.25	60444.83
614744.11	1203164.21	640539.73	1434104.60	1352879.69	541845.89	1357593	1402367.06	1492344.09
786621.78	1658598.87	848281.07	1895218.72	1720355.01	686029.76	1759599.24	1803310.48	1887849.86
168802.82	301371.52	176985.17	388329.48	376894.11	146276.18	386016.59	396887.46	397132.45
163202.59	352185.64	172180.82	406993.61	385066.03	140083.17	385476.36	374168.98	393819.90
3858743.39	7561271.31	4005741.71	9079809.75	8598192.58	3398227.82	8643433.66	8872319.89	9090799.89
1927610.73	3863986.90	1998760.96	4532479.85	4307025.75	1677013.46	4304289.13	4388475	4520495.17
13159.67	35884.37	13951	33037.50	28614.11	11847.67	28105.33	28058	28203.87
3925.03	5548.91	3337.02	7331.30	7837.59	2700.83	6395.03	6152.34	6728.20
8.50	50.50	0	3	64.62	96	23.62	6	2.50
4941.67	12530.30	4729.89	9346.71	8210.48	7212.11	8120.81	10725.98	10867.78
183439.80	423611.04	192580.93	457788.42	424410.35	163630.72	416386.27	416314.03	451497.73
0	0	0	0	0	0	0	0	0
102819.08	161154.98	103707.38	233982.68	236499.01	87903.50	237763.03	244316	244633.12
287333.17	517530.69	298946.26	654299.37	615391.32	253539.63	623033.66	660073.97	674826.92
16	146	156.50	28	234	96	42	550	189
0	0	27	3	115	0	0	0	0
9084.78	24652.96	8767.09	22706.06	24976.60	6898.64	21675.49	22178.05	27193.90

628703.53	1231237.95	649250.57	1491880.66	1406836.36	546924.12	1414342.96	1430238.01	1476204.09
0	19	0	3	121	0	10	0	28
14515.69	27772.90	14807.14	38911.31	35416.36	12818.83	35632.37	33295.41	40122
0	19	0	3	121	0	10	0	28
77152.26	117189.32	77732.61	169882.98	170205.53	68435.83	172180.63	181941.71	177260.11
372822.72	780952.10	390447.99	891327.95	831919.33	327689.97	833896.78	848366.95	881261.85
749297.57	1481736.97	782515.73	1761788.63	1668328.28	660613.41	1678700	1722358.69	1771316.11
180449.83	364477.72	188249.31	421853.57	387620.38	161749.47	391610.55	406022.38	418502.72
824375.25	1666426.20	856378.06	1952469.86	1851228.05	717252.09	1846042.38	1877438.34	1932793.87
507749.07	1051731.26	528991.12	1216860.89	1135034.91	447087.13	1137929.01	1158760.61	1198198.64
115713.91	246294.49	120312.72	277256.53	260806.90	104398.98	256692.62	260678.92	276173.28
1587270.33	3103218.42	1657659.97	3731178.74	3501551.32	1404250.45	3538712.05	3654029.21	3743987.23
0	21	0	0	0	0	0	3	0
65834.41	130534.29	68559.04	155277.12	147537.89	57750.83	147928.77	150788.10	155549.21
4634.40	6828.15	4638.50	9781.15	8613.22	4965.83	8564.50	11066.40	10835.47
1249813.52	2434754.75	1299494.93	2931465.52	2786188.22	1095669.51	2800440.04	2865447	2957454.99
63986.07	126009.24	65655.44	147211.55	141434.05	57193.32	140297.75	145994.60	147093.08
448135.80	814773.62	458698.24	1030817.70	994133.79	392532.44	995081.49	1031169.60	1050320.05
1090225.18	2311292.18	1144691.29	2628223.20	2462170.13	952090.27	2470718.43	2486982.74	2579258.36
153600.83	298635.48	162333.69	358774.98	330380.74	132636.48	336745.39	351647.11	365824.76
14759.34	27327.50	15745.68	37535.52	32539.16	15206	33492.66	34623	39952.32
333846.52	659480.33	349351.95	795625.22	737673.40	287914.66	748677.24	768183.06	782634.15
1301324.08	2659539.54	1365208.14	3086128.43	2895754.23	1145192.35	2911257.26	2972957.58	3084378.81
694643.21	1771530.01	762757.52	1767570.69	1498788.36	635853.95	1535611.60	1527139.88	1675685.30
7589.92	18285.67	7945.09	18092.50	16341.19	7321.82	16101.39	18024.04	20872.90
323129.03	597222.56	330139.25	759565.24	730421.27	285160.15	733735.22	743730.97	758813.53
186435.90	380523.61	198477.32	447092.22	401338.92	161856.33	412773.88	425017.84	442468.64
8830.38	15861.11	8316.53	19381.65	19533.48	7927.33	17930.49	19836.72	21875.37
524857.20	1048023.78	542369.26	1237239.84	1170162.15	459667.95	1170614.91	1198074.96	1229512
569134.38	1063188.37	590880.72	1298692.44	1228568.51	504366.38	1238839.21	1306386.62	1315898.13
255404.34	497819.62	270093.75	622890.36	583517.94	219356.49	596149.05	594885.61	619023.71
0	0	0	0	0	0	0	0	0
437150.13	848627.93	450220.64	1023218.02	987074.92	386823.45	984893.15	1011073.94	1014657.80
45421.85	85425.51	45446.06	104673.90	103197.79	39224.66	99576.29	101834.22	105487.28

0	0	0	0	0	0	0	0	0
65048.41	132310.99	69632.55	159250.25	146388.37	57233.16	150045.77	151682.70	157295.43
31707.86	47797.10	31606.57	72228.19	70143.41	28434.16	70227.08	71424.78	75441.26
36661.40	82146.72	38272.84	86838.33	81955.01	32021.17	81613.83	82213.54	82092.03
86752.05	164447.90	88525.13	206641.34	200331.84	74162.50	199457.51	201340.23	203718.42
336105.40	655080.77	351145.66	789914.16	754513.96	290991.94	759025.39	773629.56	794398.31
73444.31	188698.78	80585.74	182413.42	156503.34	66812.14	157777.09	163318.36	174368.14
341213.86	767995.32	367259.46	859311.35	790677.34	295123.31	800848.44	786553.39	826049.38
753247.40	1558790.35	789320.96	1794813.82	1676150.99	665624.36	1684353.14	1720776.61	1780487.34
20561.69	28376.94	20533.18	45147.81	47487.14	16793.50	47125.37	48306.18	49728
474290.35	969916.20	484936.44	1106189.34	1058362.44	413170.22	1039380.48	1071928.59	1104137.88
296782.23	616557.51	309232.25	706808.97	673697.91	255727.28	672630.33	676902.78	698899.50
223907.62	416506.11	228498.52	514655.39	495302.96	198206.97	494793.94	514122.92	518546.43
72696.43	187229.38	79908.42	186411.49	164795.50	68018.22	166768.22	165063.82	178509.10
52257.02	108616.29	54888.48	125861.31	119740.29	44731.99	120570.40	120104.69	122068.76
65874.70	139043.52	69390.60	154365.50	145458.84	56183.84	146269.60	148462.68	149217.52
18611.76	49101.28	20628.36	47886.61	41083.61	16409.66	41456.25	41990.14	45738.05
36417.36	68289.07	37639.35	85155.90	79619.14	32096.83	80826.25	84114.68	84975.87
954840.29	1957669.97	991725.13	2273645.07	2149792.59	837168.90	2143471.40	2180794.66	2240219.29
51794.01	136220	58385.52	136743.03	113758	46982	118634.99	116508.50	127476.98
446790.18	920120.85	474630.78	1059892.96	962435.16	401643.42	981475.94	1025051.94	1061624.50
115129.42	247927.06	120532.72	288504.48	273278.53	95097.32	272376.25	261676.62	278569.86
776365.84	1533668.84	808000.47	1838586.46	1747343.59	674793.57	1753075.19	1784346.41	1847020.31
725406.09	1448291.10	759579.12	1717613.35	1615550.86	635649.24	1627288.12	1671735.22	1715537.93
5934.02	12893.88	6112.85	15092.24	13500.03	5515.49	13377.17	13954.84	15930.60
1252	938	570	1602	3106	268	2119	1728	1565
675386.36	1374913.21	706495.26	1614544.03	1520503.42	589923.93	1527917.76	1557429.76	1616710.38
2695998.39	5725666.86	2855299.34	6411627.89	5789979.30	2407162.88	5886128.44	6103008.22	6294875.45
93010.69	217146.34	103188.54	233174.05	198381.66	79010	207377.31	209223.50	226640.30
116326.77	271161.59	125619.64	295827.67	273411.17	102056.16	275366.30	266409.22	286287.79
66993.32	129644.19	71101.64	155899.88	144948.79	59704.16	148078.95	155014.26	158321.23
27415.67	64382.17	26079.32	70396	71854.83	17136.35	66918.33	54632	65424.05
1429917.83	2788526.12	1491325.68	3346308.24	3151643.50	1263878.66	3181483.70	3287674.60	3364432.24
49828.87	146663.87	57022.63	128684.70	107317.65	46171.48	108616.04	110005.69	119537.48

879370.88	1765358.40	918170.98	2079955.86	1968289.22	768249.07	1978986.25	2012845.23	2084959.83
133194.64	267234.51	139364.23	315446.34	297831.48	116860.99	300148	305413.92	316790.70
30577.87	60747.72	30694.30	70705.38	62697.86	29238.65	60526.97	69487.59	68774.46
48904.95	89093.72	50641.15	112840.20	108208.06	43146.83	109724.04	114892.72	114981.27
41208.68	80926.34	44803.02	96427.02	84623.66	32028	88740.32	92710	99160.32
7007.34	13417.01	7498.51	17574.67	16059.14	8076.67	16150.99	17682	19366
68674.52	148187.04	73874.40	162973.50	149573.19	58457.33	152646.24	155070.29	158541.37
510744.70	1055469.18	539114.97	1218623.07	1132953.57	450410.77	1144008.36	1168747.91	1216842.91
260047	467807.10	269753.67	602755.34	574972.80	225674.30	581452.82	602651.14	619513.02
817763.65	1703960.92	860015.91	1983970.82	1843328.69	711445.09	1855613.35	1869445.62	1946701.54
0	0	0	3	115	0	0	0	0
1053632.16	1999446.33	1091613.66	2451080.28	2294604.04	939728.66	2318905.24	2422921.36	2460694.77
707.58	607.24	397.43	433.91	1212.85	612.66	249.49	1191.38	960.23
21942	47190	20964.50	50131	51080.66	16649	46713	46072.50	46341
172847.81	384919.31	180000.35	428583.87	395714.56	150849.64	394277.04	390247.91	415897.15
0	0	0	0	0	0	0	0	0
0	0	0	0	0	0	0	0	0
21518.19	31270.91	21444.38	48030.71	48474.85	19728.18	48593.35	50832.78	51757.35
258541.86	586777.03	275059.68	654934.88	603403.42	222177.17	610436.72	589548.12	620643.62
84584.43	187422.04	93405.65	211570.43	185909.67	72619.16	193487.35	193866.99	205486.18
0	0	0	0	0	0	0	0	0
118845.30	242285.22	124896.25	282136.66	261740.50	105023.48	263741.28	272255.55	280292.39
0	0	0	0	0	0	0	0	0
89714.07	215338.42	95946.28	226810.89	206549.03	78844.81	205215.27	203253.24	219956.77
308612.24	675131.18	323221.92	748020.77	697094.54	270336.61	696104.52	698468.10	732503.02
116298.64	255911.99	124084.20	283393.64	259683.59	101909.32	263566.15	264634.45	274006.56
55248.02	96064.14	55184.63	126707.72	126542.59	46917.99	124408.07	126241.23	129188.31
35527.76	64594.65	36434.02	80141.06	75359.87	32199.33	76061	81788.08	80562.83
91865.88	186470.52	94136.02	221345.95	206253.92	79409.50	206170.17	208128.81	212691.03
626979.24	1550648.86	678343.86	1533419.74	1319550.64	556383.30	1342031.74	1370177.66	1448923.11
279476.76	578098.10	292884.15	665616.68	622052.02	247797.14	625324.09	639659	665561.70
403503.17	811324.52	423880.97	972850.03	920349.43	350032.49	932727.88	938460.69	948103.69
437807.24	852862.48	459266.60	1008336.86	944457.13	387744.26	955970.21	1008546.85	1005460.48
590971.97	1190217.36	619291.17	1396285.01	1306674.40	523142.75	1317694.10	1354318.36	1403085.12

20525	52273.16	22878.49	54532	42226.58	22198.90	46958.80	40980	66807.23
15837	18583.50	14726.46	32488.01	36631.76	12829	35517	36985.41	34739.47
41208.68	80926.34	44803.02	96430.02	84738.66	32028	88740.32	92710	99160.32
0	0	0	0	0	0	0	0	0
876130.30	1763044.60	919192.83	2075592.12	1947315.69	770217.37	1966715.15	2006944.30	2071741.45
8	156.40	13.50	37.60	115.22	14.66	24.80	211	117.90
626996.75	1266006.51	656126.92	1493451.60	1404994.77	551564.26	1415342.57	1437546.51	1495650.25
437172.17	872921.51	454174.07	1019709.19	946502.91	378830.91	948752.97	987791.73	1004022.78
446524.40	887157.12	466126.72	1060966.41	1001640.12	391713.46	1008894.10	1024011.79	1065012.41
150164.53	309941.64	155206.16	345196.10	329906.83	135882.13	321822.83	344084.92	367656.87
0	0	0	0	81.83	0	0	0	0
121890.73	226402.11	126787.68	281918.75	259505.37	102723.33	264786.56	278934.27	284089.52
100988.27	212640.38	106977.43	239787.94	216615.42	90903.64	219551.03	229580.20	234297.95
459655.61	944040.16	476508.98	1107032.22	1046971.86	390302.29	1047582.31	1036044.52	1100789.43
35527.76	64594.65	36434.02	80141.06	75359.87	32199.33	76061	81788.08	80562.83
1108060.76	2293930.96	1166912.44	2673437.71	2509721.56	961667.42	2530819.01	2535908.37	2640290.87
569890.80	1101100.24	597926.48	1310884.46	1209254.56	506521.24	1229895.94	1302452.81	1319766.89
195325.72	421648.48	205761.55	468242.21	432872.44	170029.30	433746.83	440269.36	456398.46
2085128.28	3954881.97	2155448.36	4846607.89	4598477.92	1844893.13	4620575.88	4794530.19	4892300.11
27748.73	75903.44	31322.61	77645.51	67725.55	21249.50	68506.98	62027.55	74162.55
93342.57	171925.47	96068.30	214638.27	206841.19	81822.32	207305.81	214787.92	220592.35
426800.72	855859.72	447603.44	1008500.85	944548.64	375701.28	953489.49	976911.32	1010960.02
1272	1385.33	706.17	1673.01	3669	581	2315	1952.50	1880.66
33374.10	67303.73	34989.69	79156.76	75338.59	29603.33	75284.66	77181.98	79868.74
12422.32	34986.34	12527.65	29799.88	28899.34	10460.66	26057.66	28925.52	25712.46
2159647.99	4870178.91	2322371.96	5247006.73	4623542.19	1961499.53	4736568.17	4869248.58	5083590.05
4494.67	22896.67	5662.67	14737	12346.33	4795	12024.33	9995	10622
703263.27	1496735.60	747995.10	1749864.94	1633693.78	605416.49	1655339.30	1623351.68	1705346.53
483924.90	1003913.28	507439.06	1165859.32	1099546.50	416741.31	1105674.39	1104446.40	1152848.36
0	0	0	0	0	0	0	0	0
0	0	0	0	51.33	0	0	0	0
755529.33	1651391.34	798599.01	1820780.36	1700826.43	647219.94	1704368.58	1696880.33	1779241.95
0	0	0	0	0	0	0	0	0
0	0	0	0	0	0	0	0	0

206526.62	376056.81	210892.33	472536.90	459840.71	178381.47	458711.31	474607.97	481228.08
269457.75	539904.63	281676.14	640625.08	605803.78	235793.97	609251.33	618198.34	643118.25
271311.12	546423.60	279748.15	622610.20	605614.93	235410.57	598783.01	625143.54	646028.55
0	196	2.58	0	270.86	384	76	1.14	10.28
140284.79	309047.25	146697.70	331725.48	301249.43	125278.63	297822.88	310564.97	330060.53
754627.93	1535302.23	780685.15	1780238.10	1690164.83	664621.58	1682753.85	1718641.94	1784212.47
731738.63	1364405.31	755442.38	1692836.68	1618039.60	635341.38	1623999.89	1682369.11	1706970.57
372916.80	804512.79	393020.93	912613.83	845662.15	325641.11	849250.36	853121.59	883587.34
996430.17	1822234.47	1038513.93	2362828	2191715.73	894506.21	2239253.67	2308913.58	2405666.22
33181.39	46778.77	28553.82	63344.06	75348.76	25353.12	64715.16	74308.93	71024.38
103337.37	274610.41	104109.85	231754.70	229517.42	85450.79	212938.76	223433.43	239101.17
16634.02	26730.91	15847.85	35975.84	33277.62	15944.15	32479.59	37152.84	38931.90
0	0	0	3	115	0	0	0	0
891621.23	2022510.98	956712.34	2266095.56	2078376.70	767879	2107091.09	2036925.79	2171106.15
132763.94	262933.46	137919.06	313515.59	296775.84	116744.84	299262.32	305537.32	313865.42
297544.76	594555.78	308850.02	704396.66	677766.05	262532.01	681253.52	693209.38	688920.46
0	0	0	0	0	0	0	0	0
0	0	0	0	0	0	0	0	0
835803.07	1843178.38	888821.30	2073661.55	1927060.03	719450.48	1943095.17	1908639.77	2007257.64
0	0	0	19.50	0	0	0	0	0
49434.22	109097.35	52960.58	117717.24	106693.17	42984.66	108754.33	111553.45	115763.03

B9	C1	C10	C11	C12	C13	C14	C15	C16
0	0	0	4.60	0	0	0	2.40	0
439784.86	574567.04	696583.74	271356.63	610102.98	823639.60	268912.68	267183.32	241891.11
1731401.10	2764662.66	2493690.07	1476671.23	2068975.46	4143090.61	1469573.64	1478004.33	1347948.94
308392.38	616510.28	566400.93	277906	483018.47	984390.42	279033.66	276107.61	251912.36
41142	73982.70	60445.76	25192.40	43343.26	62804.82	24946.85	25224.41	22869.93
15734.25	8269.30	23917.10	23318.09	23048.76	2174.54	21774.80	23195.07	21583.41
115882.87	275342.76	195034.90	144670.30	153010.13	370942.28	144046.60	145111.89	133146.39
0	0	0	0	0	0	0	0	0
3612346.88	6173092.83	6226743.70	3747960.63	5185120.58	7654175.64	3622802.54	3720745.44	3425570.52
26225.18	46927.89	42391.51	68394.95	31111.60	64542.83	65866.82	68825.54	64330.92
149466.87	126736.78	267543.77	61292.88	249235.72	133673.75	56386.47	56813.38	51901.63
435178.50	648179.20	754873.46	141272.78	652472.48	1519613.31	158457.96	130705.89	115939.63
17727.98	16744.32	70339.66	3484.82	64769	2388.18	2127.18	2319	2470
17462.41	50395.27	77635.56	26093.78	70957.17	3647.84	23250.23	25531.52	23520.74
20	439	1720	156	63.50	61	50	83	119
15729.42	7783.55	23852.97	1998.42	21644.09	1935.87	1580.47	1646.74	1137.08
300616.82	791542.54	447717.60	320059.36	312441.20	635489.64	313364.92	326471.14	290967.79
148315.30	505642.90	314564.82	151419.35	239446.39	375715.32	148754.66	150165.57	136720.97
156500.75	143784.28	272513.02	37372.26	258698.87	69727.62	30991.91	31669.42	29797.48
13.16	60423.08	5060.20	325	536.50	247.23	407.80	558.53	86
227010.73	276277.84	323098.93	130963.21	276923	610723.71	132794.37	127164.65	116308.75
0	0	0	0	0	0	0	0	0
269005.18	432935.15	382977.62	244694.69	319212.20	621563.95	242556.36	245303.89	223853.87
899459.53	1284128.50	1206268.02	422868.47	1023450.97	3329980.91	457243.05	403625.86	362073.56
43511.31	22361.03	72287.35	4364.26	97828.52	4028.75	3620.83	3865.29	2804.83
0	0	0	0	0	0	0	0	0
205875.36	360052.36	278470.32	195714.75	227597.66	851352.18	202849.96	194310.45	179001.65
64265.13	89789.71	154321.99	31786.94	140502.02	126363.06	30161.19	30019.04	26741.05
90732.90	152511.32	243296.57	80595.52	212261.11	131284.30	73850.77	75678.63	70991.42
238617.33	533133.16	538081.32	39106.12	444765.98	976692.12	53957.74	33031.96	25101.45
329835.44	335870.30	917788.08	115221.56	851350.04	212473.57	96072.03	97057.82	92076.33
0	0	0	0	0	0	0	7.50	0
9756	1508.08	3832	302.50	4239.92	166	165.58	125	89

265280.32	264948.59	511629.25	56364.77	494483.06	557904.61	58485.42	46543.04	42167.87
0	0	0	0	0	4	0	0	0
15565.16	8111.82	4240.30	903.60	2505.92	59671.60	2204.26	959.20	392.75
4896.49	12162.48	20515.28	1302.72	18132	59811.11	2291.60	815.32	861.69
23273.23	21886.97	73267.40	6756.63	106304.10	6684.20	4739.31	5598.50	3563.09
18088.52	8468.74	13242.96	109120.55	8419.91	5222.24	103098.62	112249.64	103508.23
5372.33	1666.84	5862.87	653.92	10474.42	551.17	540.83	580.91	239.33
975338.78	1677413.77	1620160.06	812134.66	1392188.98	2188166.55	800827.08	807409.46	734655.73
1095388.77	1618090.31	1886734.45	766024.71	1582714.76	2484941.05	747267.15	746965.16	677726.53
0	0	0	0	0	4	0	0	0
493.52	5063.88	4134.52	44657.71	8652.84	2045.43	41639.78	45383.05	41606.79
499	1003.72	2512.67	559.52	13334	678	683.98	657.56	343.98
17727.98	16744.32	70339.66	3484.82	64769	2388.18	2127.18	2319	2470
0	0	0	0	0	2	0	0	0
173332.53	214544.15	321011.29	45926.88	277137.22	138400.72	40288.59	40780.08	34609.05
85249.01	104450.06	131573.30	31395.31	114471.62	126836.90	30293.74	29449.63	26142.53
144939.51	174276.01	181329.66	78957.45	146926.97	309875.67	80815.38	79059.74	70690.22
92116	136427.38	125310.34	73877.33	106778.45	186762.96	73247.61	74196.31	67612.50
0	0	0	0	0	0	0	0	0
15745.92	8616.90	26115.97	2308.27	21835.09	2609.87	1973.12	3187.04	1319.73
4.33	138.50	0	3.50	4.50	7	0	3	11
0	274	854.50	1450	204	912	372	586.50	48
108583.79	97783.75	272793.48	17104.95	245901.95	189836.92	15766.19	13252.69	10471.30
735184.90	929998.28	1210965.17	448064.16	1031509.87	963822.30	431075.13	440115.91	399532.25
25325.40	21345.86	72391.06	5118.19	68311.09	4367.16	3847.39	3977.13	3167.68
358169.72	807858.70	672449.16	391686.08	556685.33	1167471.29	389015.88	389877.50	357607.67
36859.51	11106.53	27400.07	3495.76	24700.92	4311.72	2979.12	4621.62	2095.51
222400.85	436193.54	405131.66	200054.89	350278.68	853839.77	205062.16	197563.29	181264.43
8159.26	2273.92	2540.29	572.51	487.69	869.50	592.83	736.16	487.33
206598.44	142180.08	269944.84	82316.58	235482.08	74087.07	74526.92	78540.48	72311.56
48249.65	40330.05	119578.66	7427.13	109902.51	64868.10	6711.87	5439.71	4869.02
1112.28	7770.85	4296	747	2707.25	724.80	625.25	1090.48	365
67795.51	102431.20	89896.46	49927.52	78330.94	124668.71	49017.96	49667.89	45259.45
17727.98	16744.32	70339.66	3484.82	64769	2391.18	2127.18	2319	2470

0	4	0	0	6	42	0	0	0
133006.31	278170.21	267197.37	125841.39	222628.14	489659.31	126939.27	125251.52	113900.99
349035.55	655334.95	575070.14	321772.48	478482.97	752818.69	317091.22	322105.33	293655.86
40063.88	23253.79	78607.81	6720.50	60275.26	4394.60	3563.94	5392.28	3144
16911.91	40485.69	26815.30	24332.34	19849.51	61727.72	23901.75	24166.88	22209.85
17105.83	47446.27	61558.67	25464.26	49202.84	62738.77	24724.57	25166.08	23053.13
107886.80	108478.38	269106.80	61136.79	240445.85	191187.03	56365.34	56526.55	51936
29906.97	20328.60	78128.73	4144.57	69768.92	2775.18	2424.51	2619.85	2802
0	0	0	0	0	0	0	0	0
0	0	0	0	0	0	0	0	0
488259.16	734372.97	1148551.29	267021.10	1043930.73	1003605.37	258281.42	250832.66	229590.27
55889.37	64059.96	150840.69	8926	143837.76	6313.36	5712.34	5908.31	5702.65
80680.53	85386.62	168151.07	31346.24	145480.18	126301.24	30514.28	29435.53	27343.13
275771.39	402278.56	408445.57	248320.92	345335.27	685337.74	246789.82	248906.50	226410.07
64791.85	67673.16	112003.52	29256.06	91956.93	65543.48	27063.83	27770.84	24748.57
432264.52	445307.04	949078.43	107185.82	859948.36	640837.55	100114.83	93373.69	81237.94
1256625.99	1687292.59	2575342.68	878263.44	2236143.11	1842525.91	817690.48	841019.04	772717.98
43761.43	68808.35	61220.57	25939.37	47969.02	4328.63	23559.65	25592.65	22888.71
552733.81	711266.42	819705.56	323329.57	739846.16	1068712.73	320133.73	319326.59	286546.82
152807.32	201176.83	375029.25	88145.42	387069.47	134862.41	78072.59	80401.38	74702.31
42245.50	90497.75	80888.41	26280.15	73664.60	62766.07	25048.92	25304.42	23334.37
23327.25	41675.93	31561.23	23916.59	25863.76	61742.05	23950.25	24215.97	22108.02
350047.70	317970.15	492713.72	139869.82	412873.55	263606.52	129505.76	133863.74	121014.29
450	452	0	8	398	18	0	0	0
0	4	0	0	6	42	0	0	0
1803667.80	2844475.77	3688820.92	1422932.48	3191234.56	4492708.43	1388184.85	1382036.28	1277868.56
62853.21	41217.57	171787.18	8984.40	157216.44	5702.08	4762.94	5315.34	5142.99
507.50	215.84	322.33	21363.17	1456.67	213.34	20136.16	21469.24	20415.41
14792.25	15802.18	25386.03	1943.89	23489.42	60544.05	3004.22	1473.97	1261.94
189231.41	173066.77	362408.80	67348.16	353137.72	491652.05	70363.53	60916.98	56278.21
1007	29937.74	30751.98	3643.90	34085	2827.78	3259.72	1912.13	2421.51
1287322.87	2237339.02	1698498.95	1250968.22	1379754.28	3507550.10	1255648.85	1261829.88	1152348.60
0	0	0	0	0	4	0	0	0
55504.90	66627.62	70460.97	7885.55	57820.68	66459.33	7800.31	7830.04	3966.98

350856.68	673468.92	542869.54	298263.56	450443.22	1101885.06	303201.52	296939.75	271693.94
2457	258.75	45	158.50	398	121	125.25	101	73
619587.41	1089719.47	927020.87	628997.17	752197.25	1667422.92	626715.64	632102.89	578528.71
4.33	171.17	7	32.17	4.50	107	85.33	52.66	35.33
0	0	0	0	0	0	0	0	0
204185.09	341627.60	296693.75	200268.44	242488.50	559032.45	196677.40	198639.43	179209.27
0	0	0	0	0	4	0	0	0
16891.16	8250.49	4312.30	1047.77	4138.92	59818.60	2350.59	1101.86	503.08
17727.98	16744.32	70339.66	3484.82	64769	2388.18	2127.18	2319	2470
32937	24368.43	49734.35	4262.04	50776.84	62480.33	4605.05	3467.78	2609.04
0	94.75	0	11.67	36	41	11	15	7
2099	580.17	821.80	197.92	457	348.50	277.08	610.16	155.33
120142.34	190054.76	228188.70	58663.94	225031.46	249700.96	58499.13	56497.83	50921.63
520706.91	622215	856266.96	230664.27	739399.24	640689.68	219447.45	222700.91	199290.19
476281.16	470938.13	971961.34	217334.21	865408.46	471305.35	200479.85	207138.83	183431.01
797764.83	796162.99	1472517.78	274558.59	1321786.83	1444969.24	272809.46	255616.63	230324.67
11225.59	2872.65	5115.80	508	5901.92	503.10	412.32	860.43	208
659260.36	1124162.67	691020.76	771558.89	536911.09	1603112.80	758984.61	777960.35	714300.54
446956.43	582025.93	899633.27	212298.95	786735.69	1114092.97	211592.91	201956.49	180888
105723.63	179153.75	164545.34	95859.40	125655.38	186954.08	94106.96	97384.20	88536.49
75247.06	73059.77	146337.13	31993.80	163784.28	185615.08	32672.03	29260.76	26705.72
2430520.38	3425118.28	3629424.92	1620138.21	3113630.48	5378356.00	1604073.93	1587877.42	1447946.16
1307955	2212695.10	1950869.65	1218630.39	1610161.82	3217222.70	1210110.29	1219832.79	1115659.43
5707.99	12648.80	19918.28	1459.31	19514.50	59929.28	2395.58	917.41	973.27
1378.09	7407	31474.66	1547.10	28430	1064.94	887.56	1055.49	1009.33
0	11.67	0	2.67	0	61	55.33	34.66	26.33
48806.36	345747.73	46563.89	110200.91	29606.85	533756.48	117249.55	109709.01	103722.87
183595.70	203878.79	277866.29	89434	234033.70	380701.40	90386.73	88068.43	77146.13
0	0	0	0	0	0	0	0	0
47607.43	41880.46	83803.96	26451.77	78453.11	3256.38	23092.75	24509.94	22948.54
190739.67	181457.11	365891.40	24833.16	301026.14	490201.89	27946.30	18304.23	13997.73
1.50	1018.37	498	149.67	1589	176	176.33	137.26	76.33
0	4	0	0	6	42	0	0	0
17806.58	8046.27	10358.98	45167.65	16642.80	5385.56	43624.83	48375.82	42416.85

337433.72	330519.73	535300.32	163683.90	481596.49	382515.47	154107.02	156738.43	144004.40
0	0	0	7	0	6	5	5	6
6379.26	1858.07	1266.80	514.50	2458	496.10	426.24	882.93	244
0	0	0	12	0	6	32	5	6
35331.59	13307.17	37579.35	2218.24	50838.19	2154.38	1950.40	2025.15	1419.19
266330.92	411703.56	388130.61	223007.99	342221.09	678121.15	222335.74	220902.98	202771.16
506886.69	852563.02	685634.58	482528.49	547332.45	1295390.16	482072.74	486377.52	443513.92
101902.70	83974.66	135496.22	53837.52	114556.36	127794.83	51627.94	51968.48	47729.34
493941.60	833126.25	794678.61	382070.30	687573.19	1532978.67	388542.13	379349.72	345774.83
339903.99	536414.73	548449.59	297355.40	476742.52	804003.94	292803.73	290711.88	270588.16
83387.94	141858.45	163733.47	54389.28	144225.19	129274.71	52864.16	52946.28	48986.23
1004690.39	1430281.24	1580274.85	717472.10	1338757.66	2056862.79	707284.62	708433.25	646723.34
0	0	0	2	6	26	39	17	10
43682.43	69907.53	58240.75	26117.67	50307.77	180848.93	28025.60	25380.57	23038.04
1780.50	2026.31	4145.01	21956.16	6062.17	1091.78	20811.58	22081	20837.77
800796.21	1356184.36	1224012.67	649922.89	1014088.50	1803901.68	646022.05	650311.27	591111.09
45176.07	76140.86	75949.45	26669.73	59230.85	122028.98	26407.82	25375.77	23259.13
256334.89	332574.31	418239.71	159682.52	351199.68	501717.05	157667.14	158844.30	141944.38
719669.49	1181414.95	1199070.82	494813.60	1015765.89	2203264.97	504945.50	482390.76	443370.26
100945.31	180337.96	230513.92	78964.18	198778.28	190062.19	75964.79	77274.67	70733.33
6262.32	11537.44	34750	2826.53	48955	1693.34	1661.41	1597.23	1585.82
168356.06	203064.12	281876.54	61676.81	256963.47	192839.63	58968.40	58874.83	51963.38
951517.04	1815219.29	1331831.06	1049128.32	1086827.97	2473338.70	1042415.14	1056603.93	969265.69
305172.11	277497.05	856193.74	56486.68	764025	38640.11	34942.90	37893.74	36811.63
22641.75	5543.25	6506.06	1684.52	1541.03	2946.75	2052.70	2838.49	1197.94
155014.09	140490.06	256772.91	36583.04	241779.87	69238.84	30530.57	31196.14	29280.37
90694.46	89872.36	189203.60	32339.19	165452.85	66821.19	28729.43	28858.30	27321.57
11847.18	37183.39	15845.42	65446.98	12367.43	60958.39	63167.38	66728.14	61889.43
328729.30	476659.92	521027	233105.89	444873.95	804116.16	232065.62	229063.57	208992.49
270899.92	363406.10	549834.93	34900.27	452088.01	146270.43	28100.99	29029.94	20441.40
124821.14	92101.88	267276.69	37219.98	261518.62	72283.05	33467.33	34440.71	30116.42
0	134.17	0	2.67	0	48	55.33	33.66	23.33
215600.99	308824.04	582765.83	98717.02	528482.66	202727.27	87306.67	89359.35	82207.97
28076.81	49217.74	38758.20	26122.15	40399.35	64555.07	26262.36	26842.18	23614.23

0	0	0	0	0	0	0	0	0
29684.44	21852.66	51420.73	3219.37	42071.84	2348.20	2230	2274.63	1876.88
13961.08	31583.80	27384.39	44439.67	21866.26	1374.41	41292.42	44112.71	41643.32
22524.65	15454.09	25787.78	1756.15	22475.92	60080.28	2433	928.97	1012.61
47527.88	44623.80	160192.92	9438.24	153276.76	6254.89	5239.96	6055.36	5561.65
186232.49	264062.50	303951.52	109653.33	264042.31	729730.02	116938.20	106171.07	96626.75
64731.95	133543.30	130919.99	9801.88	91196.51	9181.23	8064.25	9278.73	5952.32
156507.04	143767.39	355130.11	45737.02	341432.87	315667.58	46056.60	40334.56	36091.12
498890.63	866248.47	747229.11	443729.07	636024.76	1061331.07	436547.80	443914.52	405089.63
9534.09	10727.83	7480.90	1125.01	7840.42	118978.43	3809.96	1274.81	517.91
405696.19	584799.07	601567.79	394903.61	488766.20	1115760.78	391858.28	394558.84	360081.37
192918.46	270726.87	255596.23	256730.77	228029.60	790775.70	258775.85	257719.16	238347.27
125799.18	118972.07	181061.68	35387.94	162104.96	248070.75	36263.18	32510.39	28644.38
63378.13	236496.01	117501.66	157255.43	102796.46	67281.08	148928.59	158019	147712.82
24086.75	16036.84	48610.19	2927.31	47335.93	2449.48	2168.36	2386.39	1808.77
29988.73	35964.03	66872.68	4086.67	61818.17	2687.14	2508.75	2292.61	2620.32
16421.91	33198.49	29803.22	2526.25	21430.17	2469.94	2101.84	2797.50	1541.58
16308.26	7750.64	25106.78	1655.89	20876.92	1027.48	991.39	1012.23	829.44
599855.14	819069.52	949930.35	311130.85	841575.44	1191180.50	309410.52	303083.81	272799.12
23043.98	16716.24	58994	3184.50	48604	2498.37	2227.54	2197.36	2332
275209.10	387607.59	420901.55	161510.32	329899.07	620351.31	162437.83	159823.60	143346.83
52806.90	38437.66	149803.30	9060.30	157238.18	5935.12	5335.01	5580.44	5167.18
486088.67	730278.11	683543.28	314754.89	569096.84	1003481.99	316075.38	315589.21	282610.39
415383.07	844004.32	730489.61	479212.17	605607.89	1172223.35	471600.78	479179.70	440231.74
8159.26	3172.09	2630.77	868.58	1019.37	1190.60	804.16	1166.08	705.66
496	134.17	0	33.67	1594	41	43.33	48.16	30.33
448810.14	693682.47	674431.34	394678.72	570014.08	1108637.17	387184.69	390866.42	358039.10
1432538.82	1909760.18	2875715.63	858404.50	2494509.17	2275877.41	805049.20	807773.69	747147.07
40771.96	33543.56	129333.66	6669.32	113373	4899.55	4367.72	4527.36	4802
78580.25	151585.99	152478.30	53846.69	129815.44	245926.71	54912.21	52849.77	47740.19
38458.27	48844.10	56899.83	25296.18	45460.18	63027.32	25035.05	25735.72	22922.68
12431.31	35098.46	21243.67	1934.01	19877.67	2132.67	1853.14	1779.72	1202.32
946771.20	1508038.22	1181632.44	864125.98	945569.61	2042361.84	855010.79	865446.46	793421.10
41337.22	87118.93	55302.81	69803.76	44195.85	182768.22	69615.91	71349.14	64521.59

612614.66	1056734.69	848622.60	602006.25	701416.92	1486511.95	599168.60	606954.55	555016.51
101577.33	199252.35	133132.68	117485.70	105639.21	306190.88	117629.03	118466.45	108982.52
20721.39	12320.53	9220.35	2699.44	1399.17	60697.36	2924.26	2606.06	836.93
38709.50	77496.03	42556.32	46851.83	31303.51	122773.03	47110.23	46924.74	43255.38
17727.98	16744.32	70339.66	3484.82	64769	2388.18	2127.18	2319	2470
3012.65	16855.59	49760.66	2763.60	43703.50	1865.51	1472.83	1640.38	1612.99
39392.82	61015.73	56422.54	25394.09	45541.68	62784.67	24821.34	25057.02	22840.49
364625.04	649985.77	537209.83	383675.59	432270.81	1044773.24	383078.81	385359.56	353752.20
151802.59	170976.80	317060.50	67334.89	280352.96	199148.19	64326.84	65095.92	56865.44
472137.80	635701.09	931896.71	299476.88	848998.98	763750.69	288279.53	288337.02	264584.07
0	652.50	0	0	0	0	0	0	0
505805.39	505299.42	1011457.27	235218.20	885022.53	285205.57	207288.55	218079.03	199973.55
185.41	26373.33	8888.48	21793.33	1980.84	535.88	20545.38	22047.53	20600.44
9443.50	1991.92	4366.67	806.67	17761	515	583.08	620.66	437.33
115584.75	173796.72	165265.73	78697.81	152354.86	191340.04	76565.25	78248.92	70153.47
0	0	0	0	0	0	0	0	0
0	0	0	0	0	0	0	0	0
9845.73	56378.65	9641.73	22304.80	7026.59	119257.94	23919.66	22370.13	20896.33
137924.73	128845.57	291488.78	38606.44	280412.86	130665.55	35099.74	32890.80	31370.83
37820.82	43667.87	124644.94	6993.04	119144.51	6011.46	5345.64	6686.16	4341.38
0	0	0	0	0	0	0	0	0
61452.15	108161.15	126141.13	30602.11	104511.19	185341.52	31676.36	30065.34	26351.14
0	0	0	0	0	0	0	0	0
46023.95	65298.25	121178.01	29572.79	104094.51	6829.30	25677.78	28340.69	25354.43
221477.23	330763.49	312240.55	174343.99	267626.74	496550.71	172826.21	174624.98	158124.60
61118.02	37152.13	94519.43	6344.77	83000.93	64713.83	5992.08	5038.08	4016.80
24910.66	16382.09	50939.20	3773.97	46294.51	3242.20	2672.36	3341.22	2324.21
15729.42	7783.55	23852.97	1998.42	21644.09	1935.87	1580.47	1646.74	1137.08
49005.74	70623.93	93406.22	27601.89	91614.43	122623.34	27254.12	25694.43	24033.71
275730.54	248245.98	746055.10	71573.11	651229.34	37731.10	52269.61	54632.35	53304.63
210918.15	319119.58	301091.60	174449.26	263281.83	438120.02	172865.48	176108.98	159134.11
185970.19	110599.04	347331.12	19161.40	345521.46	13505.68	12647.44	12060.60	10986.98
196511.07	118979.06	401221.12	24470.59	351450	20325.17	17427.26	19729.52	14667.99
430494.99	737189.05	623669.43	413475.70	516735.33	1111652	411623.64	414351.79	379114.49

19941.19	197242.88	143623.09	46651.32	147358.34	144970.97	48336.04	42962.54	38674.32
7355.33	1617.43	3769.80	214.50	4390.67	315.50	189.43	196.60	108
17727.98	16744.32	70339.66	3484.82	64769	2388.18	2127.18	2319	2470
0	0	0	0	0	4	0	0	0
550614.98	791881.96	812087.15	425414.49	673105.88	1061540.63	417684.29	423377.28	385573.21
0	186.55	38	103.90	13	119.60	94.50	207.41	52
448600.41	935281.59	588432.61	539615.45	465495.77	1405997.97	538405.36	541682.88	499981.68
220771.17	215526.26	430412.14	71452.69	389762.14	198576.77	62705.71	65150.75	57187.04
292813.93	437042.69	423014.19	204202.43	351035.79	799282.42	208062.88	203734.47	184424.47
237854.15	426767.09	255672.34	518746.18	204123.41	978613.85	511511.75	528196.98	486883.28
0	32.67	7	28.67	0	100	85.33	49.66	24.33
55919.49	38076.97	105699.51	6621.30	94908.93	63312.09	5478.71	4003.03	3919.10
44723.57	27420.12	89373.07	6127.85	77365.51	4592.81	4016.46	4811.10	3504.50
295404.52	465939.32	473400.04	249651.09	437137.19	624493.22	245785.84	248043.38	227906.67
15729.42	7783.55	23852.97	1998.42	21644.09	1935.87	1580.47	1646.74	1137.08
604096.47	809626.84	1077380.87	290300.57	950577.04	1600050.16	298963.31	279396.29	252600.58
317815.76	528090.25	617530.21	297394.53	514525.01	566134.54	282351.21	289496.19	268448.84
106748.50	159265.54	269411.15	38609.16	241868.60	367168.60	40784.34	34337.19	31003.11
1178314.76	1375737.54	1970848.34	655192.00	1721792.03	2180420.35	633554.45	631022.56	572750.02
28907.67	84281.96	82525.23	48098.38	86461.34	63305.39	45408.28	47739.33	44227.17
77552.17	161327.39	97613.24	114947.66	77861.79	361950.22	116398.26	115191.76	107097.22
288534.37	474657.33	409949.48	268381.92	330506.61	682460.32	266387.81	269541.29	245985.19
503.33	974.51	78.34	314.01	1741.84	544	427.49	357.98	140.99
23356.41	49612.68	64319.23	25820.01	54685.93	62891.39	24962.25	25693.29	23089.68
5342.97	10965.03	42186.67	3095.86	59675.84	2386.64	2075.72	2199.39	1947.30
965025.44	782609.13	2476265.63	153919.72	2219634.54	163610.96	92172.40	100926.28	94286.78
2431.33	4889.33	15384.33	664	14295	427.67	347.92	370	444
350973.97	370633.98	755025.06	88731.84	725640.34	390317.86	82890.20	78339.01	69343.24
340964.60	437577.99	455665.74	227820.17	387835.36	801389.56	230423.29	226610.09	206489.67
0	0	0	0	0	0	0	0	0
0	0	0	0	0	40	44	37	21
366807.04	377167.03	619845.24	140183.86	576360.28	879180.27	145893.84	130834.89	117651.48
0	0	0	0	0	0	0	0	0
0	0	0	0	0	0	0	0	0

126256.78	230814.10	180533.19	98330.61	150300.96	248230.26	96875.42	98490.39	89336.79
188008	332179.43	270530.97	197274.05	219514.08	497665.07	193562.34	196771.76	178064.16
435953.18	617174.49	335199.30	356132.63	270365.89	866139.38	353860.20	358172.82	327061.49
0	32	0	0	0	28	0	0	0
120480.88	259230.26	203009.96	81005.06	147956.62	194318.56	79509.33	81299.13	72031.86
632948.39	1213320.84	883134.51	521153.28	702132.21	2017457.29	531827.89	519886.76	473408.23
507372.60	778318.29	673003.33	398599.66	567655.92	935541.59	387511.77	400164.81	358898.52
184613.52	212006.44	517404.64	33390.66	472934.45	201049.72	25574.27	24275.15	20718.40
518854.77	703255.85	986254.05	285204.51	851365.36	881457.71	270441.09	272495.19	245294.54
38571.20	120198.58	101645.39	73358.70	84824.93	186116.77	71694.12	74803.57	66254.20
541522.95	589376.98	224580.78	496395.24	141783.42	736423.58	481455.06	499037.32	461420.37
12285.26	3860.27	4287.57	43718.73	3449.05	2172.87	41254.99	44833.39	41645.74
0	0	0	0	0	0	0	0	0
458180.86	446337.98	848742.17	119940.79	797288.87	810804.68	120997.35	104287.78	96942.91
93146.82	178380.61	127072.32	95359.90	105459.36	363238.32	97523.17	93901.66	87851.72
154059.04	182549.39	209126.06	77373	214563.56	69565.75	71619.48	74658.71	68750.19
0	0	0	0	0	0	0	0	0
0	0	0	0	0	0	0	0	0
470011.52	645038.78	963292.46	236350.20	889536.46	1175226.60	238514.49	224156.71	204223.23
0	69.50	62.50	80	4.50	19	18	7.50	0
41663.18	38733.04	49689.39	24514.62	39312.09	2631.49	22262.90	23745.82	22209.19

C17	C18	C19	C2	C20	C3	C4	C5	C6
0	0	3.40	0	0	0	3.60	0	0
243265.40	1067070.46	376626.81	620797.13	297574.82	693835.56	344626.21	268092.97	594437.61
1342849.42	3950597.97	2069193.11	2262980.19	1644410.27	2590580.62	1437909.91	1427818.93	2692819.22
251685.83	870838.53	405208.12	474085.50	326413.62	545717.74	328488.08	269503.43	617398.40
23013.11	101727.64	40776.56	48611.72	31736.35	56169.19	38522.15	25267.81	48650.48
20939.11	51576.54	8529.99	27443.73	10670.79	38524.84	7851.47	21217.27	15472.55
131003.84	274773.07	180129.32	174271.67	137886	226971.80	136331.58	143232.43	193714.82
0	0	0	0	0	0	139	0	0
3380762.23	9259192.80	4144201.35	5710908.20	3371030.05	5997737.48	3610759.39	3628847.08	7135749.11
61885.73	76170.50	46917.62	60337.37	45021.80	58694.13	29538.78	64403.74	89498.47
53395.32	354167.16	72428.48	201913.76	62309.89	213635.19	98746.99	62423.91	182011.84
125241.92	1002020.84	330128.09	605113.48	312647.22	750156.70	356401.26	160219.27	781316.09
2777.50	23985.90	3175.67	43484.68	4691.31	33988.34	21500.82	4666.18	21365.15
23281.95	99138.27	25197.53	59442.17	18362.31	62883.43	34389.98	23417.98	73069.83
82	878.50	161	2605	268	1775	1060.50	116	317.50
1541.44	50942.10	2269.99	19852.07	1834.46	28537.38	7554.39	2883.52	11280.39
290296.65	920361.51	481777.08	443288.86	341656.86	672787.66	401385.25	328468.46	589174.13
136409.10	523863.37	247971.07	269308.38	178656.98	308149.20	249681.93	159623.17	348702.02
31307.05	297925.65	37829.89	201410.02	41406.34	190714.85	89503.27	41055.05	195036.35
568.74	27266.78	7227.71	708.86	7799.80	16114.40	20424.06	2606.80	7352.10
116514.31	537444.67	162443.96	289301.76	128241.80	352289.31	154770.48	129831.76	266932.31
0	25	0	0	0	0	139	0	5.50
222051.02	651517.33	340351.65	361026.80	269224.20	443824.40	228248.65	239423.38	436226.92
374891.75	2244138.66	591706.93	976699.71	519504.66	1327562.15	566749.93	408183.92	1258810.87
4394.64	41239.09	14559.48	48009.70	15080.27	57005.97	19470.21	5188.38	60490.87
0	0	0	0	0	0	0	0	0
175771.50	475742.65	195037.45	271594.06	154002.08	291323.40	168486.41	188060.72	310565.27
27715.29	161926.71	29787.28	119245.80	33007.81	139149.62	59065.69	34533.60	108017.21
71008.17	271252.43	83718.20	179982.13	66692.11	169715.89	108949.51	74903.62	165858.95
31215.72	606030.21	117683.85	379407.54	112037.33	502696.46	280592.59	70127.77	394474.97
96921.15	492669.18	107110.26	598650.22	97850.10	556062.41	293777.01	111586.20	704458
0	0	10	0	0	0	6	0	0
159	1676.67	176.33	2641	211.33	3337	316.33	211	780

46486.05	438990.63	91459.44	420179.14	77398.99	371642.67	189756.08	66494.32	487645.88
0	25	2	0	0	0	139	0	13.50
733	3280.32	1477.33	345.40	396.25	22591.85	2212.27	491.15	7777.75
992.06	8337.17	929.50	11820.22	1502.39	10655.18	7035.56	1694.34	12959.86
5450.56	124769.55	6518.78	43998.25	6670.45	48681.95	24814.88	8094.22	93047.83
99315.39	42890.49	88121.06	65300.50	57615.16	250353.76	11274.56	95836.80	60092.27
451.30	13366.32	1135.82	6099	555.65	25401.30	1680.83	417.75	25784.17
733356	2830211.86	1089551.98	1429919.65	844874.36	1646431.35	920465.32	795942.14	1512427.90
683196.37	2399808.50	954607.68	1517341.73	739062.70	1689570.63	956102.32	725593.82	1693119.08
0	0	2	0	0	0	139	0	8
40110.98	116906.90	35093.32	18911.76	19337.32	26140.27	4830.46	39018.64	12706.70
863	1568.33	9876.16	4727	10692.86	2608.19	2188.38	405	19711.53
2777.50	23985.90	3175.67	43484.68	4691.31	33988.34	21500.82	4666.18	21365.15
0	0	0	0	0	0	0	0	0
38397.21	486936.72	57664.13	228206.84	58428.93	276253.97	145402.32	52293.75	209411.24
26986.05	254693.95	40068.37	109008.66	32530	156756.30	51448.36	30137.95	82205.94
71902.07	410929.75	133953.72	154105.18	112977.04	198284.78	91446.13	79611.41	207934.04
67421.45	255685.82	109505.25	111514.24	88527.37	143007.54	70808.26	71533.66	145041.07
0	0	0	0	0	0	0	0	0
3001.44	51571.10	31171.50	20674.07	31619.45	48016.38	10620.54	3016.02	58910.89
44	571	36.50	141	7	2023	141.50	0	120.50
252	344688	366	0	108	4121	232	0	84
14626.08	316978.17	54887.55	184709.06	58869.52	210984.05	86853.42	19770.72	234294.02
403174.21	1867505.06	616829.06	999644.91	484968.93	1198210.48	567118.97	438665.40	968137.79
4031	99962.25	5317.48	46511.10	5116.42	69449.58	23260.19	6041.60	44130.17
354192.24	1057829.48	544966.92	587907.83	441154.17	638307.02	420171.02	375909.28	830330.65
3244.94	98491.05	5977.66	43984.49	3746.73	98997.03	14888.84	6616.49	39366.13
182322.36	667109.62	288246.94	357933.34	255885.19	412862.98	217756.67	195352.72	419664.87
437	4158.20	11823.94	3647	1151.38	64148.90	1815.08	1046.75	3613.71
72321.33	407416.47	77026.63	242515.21	65028.11	275229.95	104859.25	80860.76	206710.35
6141.44	126669.92	16790.16	84727.14	18416.95	82289.65	37406.53	10234.04	60116.61
739.42	43916.74	943.75	3973.33	394.14	15883.43	2248.50	1148.50	1341.56
44457.58	196828.64	57620.36	80055.83	43020.34	103297.59	49438.10	50547.25	76374.03
2779.50	23985.90	3175.67	43484.68	4691.31	33988.34	21500.82	4666.18	21365.15

0	25	4	0	2	0	139	538	37.50
114070.51	356486.23	188925.72	218254.40	157042.18	252482.61	146168.87	120681.91	322109.73
291168.25	771571.40	411529.78	490405.94	329759.03	581285.05	341692.08	314804.33	537841.28
5681.94	183656.99	6877.41	63167.41	6303.96	127261.19	30695.48	6924.56	54790.93
21454.17	52712.65	23339.98	30044.78	15463.49	45111.70	20632.45	24522.94	23417.04
23098.17	101494.42	33706.78	45141.01	27304.34	51346.08	30122.88	24369.70	46763.61
53222.84	292807.01	30664.94	212945.55	37767.40	221488.36	96487.91	61784.44	231089.26
3062	30242.32	3979.67	55359.48	5516.51	50652.77	24256.80	5140.28	28834.18
0	25	0	0	0	0	139	0	5.50
0	0	0	0	0	0	0	0	0
235762.71	1130870.69	391890.47	852873.20	333159.12	844837.43	492001.40	270310.12	1025762.20
6825.36	103708.55	19259.83	105789.51	15388.78	94154.45	57209.92	10018.76	164019.18
28119.09	123696.21	49267.38	132147.69	44647.47	116272.34	63824.92	33281.80	105801.27
224658.44	882367.32	298575.46	379561.45	244745.56	479722.44	224243.38	247768.17	486803.25
25651.59	198977.05	37031.30	85743.07	30617.79	98081.13	44275.48	27082.76	86884.18
89033.78	1094833.75	140955.58	734564.06	124582.21	822140.67	354505.60	127705.95	702209.75
781064.65	3206014.66	1019532.91	2028319.54	809635.39	2192782.70	1103126.98	816925.80	1839459.06
23165.61	143347.78	48309.10	51658.21	31510.84	70778.72	39488.04	27063.18	62933.14
289365.80	1369980.02	414736.14	675250.08	333371.49	859964.14	406664.83	321014.99	839353.84
76842.15	287890.15	105154	297903.28	86233.01	250040.31	148325.53	80726.53	373377.39
23459.03	80191.21	28077.31	64636.10	28662.44	68623.75	35362.82	26403.85	92941.54
21834.17	55133.57	32673.64	29997.85	25453.66	41526.74	20423.87	23049.74	36482.21
122732.73	789418.29	165187.55	415062.11	129266.77	561062.61	195178.48	150631.48	416869.61
6	249.50	14	296	0	2	69	34	58
0	0	4	0	2	0	0	538	32
1272749.22	5543343.69	1647189.72	3253850.32	1405700.89	3239718.05	1797775.66	1444530.55	3776998.47
6846.88	108419.24	7185.92	95710.36	11662.71	103754.32	49881.94	8060.86	128273.55
19291.67	619.27	6072.42	7612.66	8764.29	810.58	601.66	19421	14823.54
1822.50	51717.75	11344.50	21390.39	11891.18	19763.93	8347.32	2684.34	27465.57
58174.94	264036.22	62994.12	297811.12	65627	281031	120411.58	71377.06	326403.35
1827.46	23599.05	1754.80	45674.52	2744.91	28204.68	17067.44	3051.32	6100.76
1139457.30	2918503.10	1726092.91	1582570.92	1343057.79	1851219.97	1089697.54	1204885.88	1934393.87
0	0	2	0	0	0	0	0	8
6708.02	263007.25	32380.12	70876	20585.75	113704.65	38559.76	10610.05	83406.82

270722.98	868214.02	462085.68	471087.94	362687.89	538511.55	334004.90	293202.32	551719.56
84	931.25	162.33	2613	119.33	340	287	204	596
571661.74	1469961.25	809278.39	837940.90	634956.05	1002373.10	555443.01	604997.05	942260
63	874	73.50	148	31	2029	42.33	28	290
0	24.17	0	0	0	0	4	0	5.50
178500.36	1965682.42	266019.96	261728.47	207767.57	334939.15	178027.02	188926.53	308628.01
0	0	2	0	0	0	0	0	8
840	4133.32	1763.83	975.40	617.25	22908.85	2742.10	568.15	30916.75
2777.50	23985.90	3175.67	43484.68	4691.31	33988.34	21500.82	4666.18	21365.15
3169.56	72027.99	8878.83	58700.31	5257.21	72316.85	19950.42	7597.08	44626.18
13	493.91	78.66	16	92.66	16	194	67	6705
215.50	2215.42	870	3194.80	185	24084.20	1233.58	925.85	1189
51964.88	206578.53	61411.87	177548.34	54840.49	193273.32	96564.40	61689.32	304084.66
205631.58	1375083.84	360094.14	671563.46	302755.39	788526.55	367171.67	236760.90	766167.66
193214.59	1201839.37	298579.89	705168.79	256138.88	909909.80	378575.55	214985.71	907445.26
241334.94	2234594.56	430586.22	1156898.49	382398.75	1298213.59	563390.45	299213.31	1048671.37
846.44	11827.41	10049.75	4216	10235.04	12639.43	1671.33	1533	17240.21
700118.25	1493926.87	914628.67	727674.66	680461.55	1061976.12	539218.02	722290.09	998508.40
190734.13	1377865.10	353104.90	681805.97	307922.35	852336.82	370045.25	211364.09	815746.72
86465.96	202258.58	61312.49	128917.69	58316.32	185744.83	76307.42	89775.26	51734.73
27827.15	102315.49	33034.45	117212.27	30868.29	119583.48	48090.82	32836.77	179539.38
1450904.37	5669256.07	2031817.21	3041547.41	1598688.26	3631868.59	1870051.04	1575396.37	3199201.85
1105545.28	3191076.54	1597955.41	1748900.40	1247327.68	2044714.17	1137791.83	1184106.03	2002882.66
1017.06	10867.92	1316.50	19211.22	1664.84	12709.36	7345.98	1176.09	3753.49
1311.44	16761.56	1101.15	13791.34	1769.36	14970.34	9276.09	2055.34	2983.37
59	541	54.75	0	68	1688	8.33	0	63
99398.97	16205.17	106091.71	49182.17	72729.60	10385.12	69419.85	101588.31	54262.19
80021.45	441000.74	98451.52	276987.23	80699.63	391382.76	134847.53	97011.05	356717
0	0	0	0	0	0	0	0	2
22761.97	56296.76	17666.49	52120.60	14135.20	62473.62	23008.24	23878.15	80391.25
20638.22	681587.37	90272.59	229303.14	97083.87	309642.95	124812.77	30374.67	269419.73
129	925.44	170.17	1793	179.53	2907.43	1315.23	151	7106.87
0	0	4	0	2	0	0	538	32
42534.94	45138.45	58459.53	32614.64	40444.15	158364.93	9648.76	40046.87	78739.97

144923.64	812458.11	187168.06	449686.09	161555.15	465160.35	217439.64	164488.75	421390.65
2	0	0	0	0	0	0	0	52
800.44	11307.24	10155.58	6799	10243.37	9653	1860.50	1720	17676.21
2	5	2.50	3	0	0	64	0	69
2027.56	16891.46	3071	19921.36	2415.55	33492.11	7953.24	2804.54	29462.29
202831.29	525589.18	326134.93	359739.43	267145.63	371685.49	221300.61	219154.45	444002.64
439496.04	1100511.82	665476.66	633090.50	521715.19	783525.36	420570.93	464851.26	778857.45
47690.86	216645.76	67961.51	133478.55	52738.22	139171.81	55618.94	51807.73	109277.42
343668.95	1131197.33	501832.21	702781.72	413663.99	836533.27	438967.36	381609.97	886234.73
265360.66	745781.51	333890.29	502877.25	254653.27	469371.08	296542.04	292138.17	468987.49
48259.26	227071.95	62048.19	150812.64	49458.08	158736.29	80689.78	58097.97	206385.92
645472.12	2338556.67	929479.54	1338624.58	727593.33	1577446.11	804170.84	691266.42	1434472.86
8	69	0	0	0	3	2	0	0
23533.61	172481.64	43284.31	54288.10	37104.67	73264.88	29823.01	25672.68	72361.19
20120.23	6355.76	16233.90	15506.28	19295.60	12275.43	2563.68	20246.22	24550.49
588073.84	1898776.27	879681.51	1047111.55	695745.68	1285636.92	698415.21	637646.80	1205539.13
23785.31	83930.42	48180.65	59229.42	32718.41	92739.60	44356.28	27787.30	95188.45
143348.46	729130.80	216657.40	332816.09	169583.50	460184.60	197827.87	159248.17	351104.45
442515.27	1750769.76	649145.64	1021501.27	512613.72	1109571.52	624702.77	487524.76	1171937.83
71170.37	389077.19	107185.51	163038.60	88820.94	171435.90	111410.24	77597.10	160794.04
1874	15155	2208.64	25119.34	3865.76	15254.29	13974.89	5219.84	34432.76
54598.58	343565.49	110906.16	223368.29	99449.54	275960.60	120015.79	65866.44	321151.79
952911.02	2272275.15	1322153.71	1262471.17	1017524.26	1469154.15	885546.12	1009549.42	1465644.93
40151.90	785132.65	56483.36	794723.13	64234.13	631325.38	295503.93	65889.63	443780.49
1786.86	18652.14	24677.78	6358.62	2522.75	102165.59	5808.17	2063.12	9116.03
30637.49	292443.15	37339.38	191866.13	40433.56	185155.75	85086.44	39874.71	193234.19
27803.95	195289.86	39588.02	153791.11	35712.98	124260.30	68977.11	35529.24	104563.04
60023.75	54168.43	66845.72	28195.60	52552.99	28448.42	15440.05	57843.02	71001.15
209649.47	752420.10	299174.97	450891.85	243549.40	488379.56	255270.84	224945.64	489097.09
28104.90	864096.51	114293.44	365079.27	64907.27	541149.15	232987.03	55695.02	273576.32
33745.41	245425.63	79840.09	167273.90	76709	217557.32	81432.83	33806.63	256904.66
15	0	0	0	49	6	8.33	0	7
85613.67	462190.23	112421.57	400441.43	108543.42	407430.62	210232.73	102165.96	467728.94
24364.67	143916.01	54172.12	40327.29	46900.88	80595.39	25829.39	27221.63	92609.91

0	0	0	0	0	0	139	0	0
2298.94	47529.82	3295.92	40792.61	3492.55	38910.69	16006.46	3224.93	21412.91
39766.14	20281.66	37528.57	30465.59	24269.59	18634.53	17342.58	39186.07	30202.65
1506.56	72651.13	10743.57	22588.29	11540.97	18680.79	8288.09	1353.14	24749.79
7235.88	109500.82	8610.83	104260.69	12174.04	98194.76	51615.44	10258.36	166427.06
98735.76	659513.76	170102.33	251562.96	144681.05	285986.53	138028.46	105629.54	282857.03
8662.38	201338.39	95454.68	99553.02	56772.30	175290.53	85125.88	20062.35	129130.16
38837.17	391426.13	40640.35	271266.22	45663.39	306428.29	118103.52	47719.57	315376.27
400749.22	1217716.02	574385.46	707180.78	457410.08	824364.59	452858.55	430689.01	775568.47
1580.94	42745.76	19781.09	3563.33	20506.52	24220.71	2973.62	644.75	34223.04
356416.62	1068524.70	456998.49	584669.67	359732.17	677816.40	335840.11	384648.49	778949.49
233450.93	517324.42	237688.88	286783.73	216952.04	323777.48	140754.99	236155.12	323923.48
31930.29	298821.09	80855.21	156257.87	76499.26	192111.64	71255.91	37756.96	178253.78
142305.19	95930.69	117697.64	136506.07	97006.68	145796.55	78226.23	142211.32	140752.98
3085.44	97323.86	21661.35	32601.56	22856.72	49526.29	14658.31	3094.51	57649.44
2769.60	90674.66	3674.57	51082.66	5084.78	42526.66	20665.30	5044.02	54323.78
2307.94	53969.39	26638.64	23023.67	16601.55	36205.98	21298.16	5360.67	35137.14
1119	48585.65	1389.31	22612.89	1612.01	18200.86	7625.04	1873.14	9855.85
274839.19	1696680.91	386291.97	803279.47	308223.06	1047314.92	462456.68	321581.03	976363.05
2517.78	31291.24	3620	54577.01	4093.98	38828	19696.49	5004.51	30455.99
145128.52	738130.93	238694.07	385509.12	184168.12	434245.42	213215.33	153710.01	425959.16
6831.80	85687.93	7949.64	93999.80	10601.48	88451.87	45074.75	11845.20	145504.48
284126.14	1326464.28	477040.73	601794.55	365308.90	878081.53	388271.96	317860.23	758062.39
434560.12	1050318.83	601913.62	660814.60	509448.08	733407.32	431730.70	462562.54	830692.48
640.80	13400.60	12390.12	8980	1278.58	85509.53	2470.11	1323.55	4599.01
44	212	38	3	87	3	159.33	0	196
356451.08	1194004.63	507297.68	631174.67	405018.91	720079.34	374952.13	380294.48	829855.94
755170.05	3485071.51	890112.30	2538716.45	824198.26	2375743.60	1263169.74	821649.88	2118298.34
5295.28	55341.64	6795.67	98077.69	8785.29	72832.34	41291.31	9719.69	51917.14
48281	249521.63	77734.84	122215.33	61446.21	152640.43	78592.91	52276.72	162499.75
23390.61	104332.65	43757.23	50446.12	36804.70	60524.64	27913.69	24111.61	62420.75
1225	156657.71	2292.48	19157.32	2385.65	19974.29	8115.30	5665.67	19422.62
782940.10	1922680.76	1120251.88	1135192.87	856699.52	1214281.80	736203.27	823813.25	1242182.13
63817.95	177390.50	80159.71	71078.34	59346.54	81316.84	42541.55	63351.96	87268.19

548509.05	1415617.44	824900.53	780091.45	641566.97	872668.36	529682.15	578453.18	957377.65
106923.41	249668.84	150556.21	129262.11	115288.68	133413.75	93174.37	111165.63	153790.66
3331.20	97867.54	48292	9596.70	50003.55	36872.43	6673.35	2299.07	82373.42
42102.78	165334.73	44688.62	39913.49	29625.55	46713.78	32684.81	43449.66	40726.97
2777.50	23987.40	3175.67	43484.68	4691.31	33988.34	21500.82	4666.18	21365.15
2184	18490	2085.08	28717.34	3383.65	26747.34	15647.09	2621.01	62228.08
22492.11	84326.91	24667.70	47664.39	16771.27	60872.12	26731.13	24351.45	29597.23
348915.40	784907.59	496580.53	498702.01	394257.17	559346.53	325325.70	369042.28	565387.87
60003.54	355682.80	112253.12	212945.37	90946.70	279371.18	124829.81	67203.01	255966.65
266786.67	1151895.72	393574.75	749620.14	328226.94	773237.87	409175.03	296513.79	758026.53
0	0	0	0	0	0	0	0	0
205234.06	1115804.60	246827.22	803451.33	208030.93	926400.19	379362.59	237089.36	625883
19711.61	18835.95	20341.14	11439.05	13377.60	14640.66	11162.56	21077.87	8651.14
959.50	33742.42	10554.33	14474	10887.99	8185.33	2615.83	1439	29819.33
70627.39	328397.49	100365.93	178381.88	78713.96	238069.20	103545.75	80328.70	250313.92
0	24.17	0	0	0	0	4	0	5.50
0	25	0	0	0	0	139	0	5.50
20190.47	13994.75	20720.05	10789.91	13708.73	8092.97	11754.47	20201.50	17321.99
32357.55	149319.59	43864.32	220356.53	34126.57	167531.77	99820.40	41405.83	187821.06
7187.30	91114.46	45464.51	78092.87	47760.01	118437.81	40950.33	8125.44	140064.56
0	25	0	0	0	0	139	0	5.50
27259.61	128387.02	52681.34	100809.06	45837.17	131523.64	62155.97	35248.71	116259.74
0	49.17	0	0	0	0	4	0	11
25935.01	183996.43	28042.06	86672.45	21971.18	111153.96	52228.04	29271.16	95627.46
157116.55	513423.63	216599.89	298214.82	165002.09	348052.33	178196.17	170844.37	307482.87
5295.38	112951.93	16474.64	78301.32	16346.33	98442.84	30130.76	8597.77	58414.83
2996.80	55016.42	4450.72	36557.58	3986.68	63076.82	16943.90	7038.08	43841.24
1541.44	50942.10	2269.99	19852.07	1834.46	28537.38	7554.39	2883.52	11280.39
24008.17	90581.92	18869.31	83286.07	19715.23	72237.85	39312.54	27246.37	61100.25
56444.67	719166.70	63333.39	765215.40	69822.82	560905.62	269795.65	85824.68	535172.24
159503.27	465960.67	271720.55	276918.30	215652.24	323029.45	178348.37	171807.81	372696.77
14581.54	188233.37	18068.42	229204.64	22068.68	224266.49	96835.83	20368.08	191327.37
20000.92	617975.93	27908.01	287230.26	25681.62	391976.42	121847.45	34831.46	174870.88
374327.23	1024976.88	537890.70	566146.54	409939.83	634481.26	377344.35	401001.88	622977.75

36536.35	181573.48	63653.52	181978.92	54536.36	126406.47	99270.05	44205.61	85554.15
183.50	30559.34	382	64	210.20	4845.43	668.73	187	7431.20
2777.50	23985.90	3175.67	43484.68	4691.31	33988.34	21500.82	4666.18	21365.15
0	0	2	0	0	0	0	0	8
383376.55	1360150.41	558539.99	714179.17	430313.77	794358.34	431924.97	411201.37	716228.08
121.30	8507.25	132	802	23	2727	251.05	69.80	422.10
488909.81	1056242.48	653183.89	593635.09	502380.83	610791.68	442789.53	514339.65	703449.72
62562.92	540667.91	110379.93	336585.02	100196.25	399432.44	168803.73	78133.36	356698.02
185966.12	758898.07	314837.08	365824.07	259828.01	456761.41	227895.45	201841.27	460525.08
472035.99	594468.86	486238.37	365653.62	332779.88	535205.74	199345.84	470567.05	398473.88
19	318	32	7	24	6	39.83	28	106
4642.94	93754.55	5774.65	83043.90	7414.56	72296.73	33155.06	9099.82	65996.92
4668.22	169690.72	6475.41	74948.75	6173.22	82298.53	29650.57	6899.48	46279.12
225764.56	681211.99	325089.40	402235.77	257336.91	451299.94	257487.60	251611.97	402262.81
1541.44	50942.10	2269.99	19852.07	1834.46	28537.38	7554.39	2883.52	11280.39
257384.50	1393328.52	440012.54	862863.08	366369.90	1033975.96	463727.82	299788.91	904896.52
267025.54	1028246.14	304861.46	536137.36	282707.06	520121.47	311394.72	275553.64	504197.95
33400.85	328666.13	65504.37	199363.80	54963.45	190650.57	102887.05	39463.67	153139.40
582374.94	2731948.57	900009.77	1602715.26	722354.49	1856175.63	836784.06	641069.60	1595451.84
43292.26	110730.17	45669.28	66749.38	32395.43	68925.70	46584.14	44098.40	96253.05
104016.29	169190.29	113917.58	96369.16	88503.76	90638.24	71205.08	105988.32	114598.31
243346.07	740306.12	354350.59	374128.81	275010.95	437168.49	245248.77	258085.38	435108.14
330	61482.17	516.84	45	438	5061	911.99	65.50	20374
23755.27	88698.67	43294.15	42470.79	37168.98	61067.41	30356.48	24518.28	68015.20
2812.42	20859.62	12607.13	40253.34	14527.33	26694.76	16255.62	4046.84	93743.20
114378.22	2488913.84	198362.39	2112479.73	216147.55	1758765.56	819947.36	167627.64	1178397.90
621	6716.33	659.67	10763.67	835.33	8648.67	4257.33	633.67	1759.33
76115.22	549322.20	125775.42	529895.33	119793.68	553549.35	257996.95	94243.93	638555.07
206104.29	673032.06	320365.23	400623.79	258071.58	449578.98	230980.57	219083.15	466087.06
0	25	0	0	0	0	139	0	5.50
23	191	97	0	36	0	10	0	7
122733.22	1759888.02	233565.35	614036.25	229774.68	716197.55	253659.82	153359.21	625819.80
0	25	0	0	0	0	139	0	5.50
0	25	0	0	0	0	0	0	5.50

88914.64	280210.28	108833.46	150493.88	101105.41	210492.23	115824.05	98584.59	162704.75
177018.36	1569568.75	263852.05	243613.58	205775.56	309639.22	169984.46	186908.69	297634.51
325687.77	783763.34	476757.05	332902.79	368882.98	477607.01	284860.53	337638.85	551790.26
176	2164	128	0	0	6728	0	0	32
72383.39	386738.85	164785.38	179881.02	103024.55	291115.93	143161.81	91558.75	212793.97
473264.55	1436706.33	775008.03	795906.17	614540.25	950575.84	572404.33	511643.18	1168342.54
359544.34	1383014.45	422584.16	601786.60	349382.54	779308.07	393830.59	380607.51	585028.62
25924.14	501431.83	57257.73	373849.79	56756.53	401533.75	193936.76	42177.38	547548.48
254894.60	1296747.82	424958.74	779089.89	341443.52	938502.52	447550.39	277608.44	863174.98
65755.79	235850.98	70850.42	98380.59	47784.06	139856.05	69787.99	68954.78	75920.79
452459.28	505315.51	520709.23	276752.36	383759.83	245907.03	232520.51	445117.23	395110.92
39638.14	17844.09	25495.71	30131.82	19053.29	107100.21	4487.59	38833.10	24885.73
0	0	0	0	0	0	0	0	0
101146.83	647627.86	165546.29	691933.80	148033.55	657311.46	318636.95	135698.58	703827.86
85018.30	219856.97	90447.14	124690.62	61978.35	106954.95	80226.85	92069.85	105325.49
68317.13	192114.12	71277.69	180354.80	59547.03	182313.01	82208.31	73996.81	204026.80
0	0	0	0	0	0	0	0	0
0	25	0	0	0	0	0	0	5.50
208434.32	997884.09	315169.43	735938.90	249444.13	789559	401144	236711.84	806990.21
0	63	10	11	0	322.50	186.50	22.50	44.50
21690.81	59441.55	23049.08	39506.99	16082.04	37832.51	24761.33	23952.21	28323.92

C7	C8	C9	D1	D10	D11	D12	D13	D14
0	0	0	0	2.40	0	0	0	0
417505.04	605372.14	714742.17	369821.42	267769.41	246975.90	754332.26	756416.66	698611.64
2076040.04	2049528.86	2317724.81	1750608.72	1461722.25	1372625.83	3757049.54	2788565.18	2316874.28
461620.74	490470.41	552251.81	357847.94	274425.75	256629.10	892945.21	590765.68	539597.93
34006.46	41134.76	52774.13	32541.91	24671.12	23056.81	57564.45	66162.72	51996.21
10534.77	21090.18	24712.15	15611.93	22650.79	22022.85	2677.44	27513.93	22352.90
188857.96	164207.20	180827.83	153025.05	142885.96	134551.01	335142.89	209380.04	191316.61
5	0	0	0	0	0	0	0	0
4592964.88	5615264.48	6695082.45	3867329.00	3691031.09	3446633.36	7052219.80	6669826.03	6117043.74
50534.11	38081.87	56783.24	51205.24	67454.94	64558.94	58831.07	52799.13	47933.21
85136.63	237282.74	283887.78	89164.63	58165.46	53926.80	129693.85	257659.16	279089.10
578272.07	654369.26	748217.01	338302.96	137731.96	122148.70	1377162.42	772022.48	738337.18
6261.18	59355.68	73545.34	12244.16	2402	2414.66	5112.34	46346.36	64447.02
22167.71	63266.09	72693.15	27353.05	25205.21	23336.59	6626.15	63302.68	81495.89
583	847	5435	134	112	62	187	2760	1756
2819.10	21055.51	24656.82	5819.01	1934.46	1465.19	2549.77	27513.93	22082.90
374349.59	374071.37	421138.12	376724.21	325680.68	294630.11	581545.13	531198.07	454426.50
216705.19	251869.64	311108.13	190230.08	148194.91	138643.76	344486.17	326984.74	310210.13
68158.39	232257.48	263728.67	63290.48	33867.62	31181.69	73931.87	250133.94	270169.42
14975	1466.14	741	5554.66	729.15	203.26	513.90	5348.64	947.80
253504.08	270304.13	312184.44	158080.70	128727.38	119929.85	554394.93	349650.74	301790.82
5	0	0	0	0	0	0	0	0
313153.08	327512	356194.99	288581.23	242126.81	228166.87	562634.88	455436.75	364578.77
1430433.56	986563.70	1102428.30	597722.40	419935.58	378093.36	3001201.07	1383961.24	1096494.36
7266.11	74365.13	59570.32	19577.35	3776.83	2374.66	5783.21	60419.82	104775.15
0	0	0	0	0	0	0	0	0
376746.07	232931.59	275069.21	189480.96	192771.53	181970.65	763980.36	345978.43	291750.72
70985.44	142694.68	155196.39	49915.22	31295.46	27868	118743.47	136168.21	161535.65
88394.80	192472.77	269980.86	91374.92	75884.65	71929.93	126957.65	216135.63	227911.32
371088.32	454318.78	518080.74	165091.52	40260.67	28910.19	886268.51	546677.49	501287.28
211622.71	724135.85	936279.57	183421.72	101077.13	92970.21	227765.21	694692.78	938315.71
0	0	0	0	4	0	0	0	0
359.50	2376.17	930	629	232	149.67	305	2428	4318

245391.70	478843.96	523084.46	125721.95	50064.97	44511.22	515983.22	483443.10	568049.78
5	0	0	0	0	0	2	0	0
20742.42	1458.67	312.15	1072.55	1276.90	428.30	53110.36	4362.57	2213
22274.02	17389.61	23495.25	3957.02	1063.46	868.77	53989.17	15482.46	18020.41
8066.69	84562.28	82750.87	18683.44	5723.46	3642.93	8587.01	69621.49	130321.40
40377.87	8804.69	25273.16	66800.50	109117.08	104294.50	5002.74	16872.43	9128.22
806.17	2553.67	1248.67	854.41	795.20	340	696.10	9048.67	16388.40
1167750.37	1382760.52	1587042.79	973550.87	802367.43	747937.49	2000245.20	1807277.62	1560531.53
1223550.96	1541427.49	1903089.01	919491.81	755763.76	692388.55	2290881.15	1718889.65	1729675.40
5	0	0	0	0	0	2	0	0
16902.18	10960.29	4421.81	25179.03	44654.26	42285.60	2918.69	22150.30	15539.24
1365.52	7113.60	5748.33	6585.98	397.20	126.50	472.67	8342.80	21993.73
6261.18	59355.68	73545.34	12244.16	2402	2414.66	5112.34	46346.36	64447.02
0	0	0	0	0	0	0	0	109.50
90119.46	257346.08	335922.56	93067.13	43937.38	36735.06	136492.59	313574.15	304163.60
91016.13	109756.99	129165.11	47943.57	30794.88	28120.09	117526.66	148624.99	136160.83
150980.48	152554.73	169172.95	117507.67	78191.54	72222.99	279893.34	241357.96	174596.36
97220.34	98921.17	113847.44	91291.26	73271.33	68693.48	169608.14	154250.95	123357.30
2	0	0	0	0	0	0	0	0
3907.45	27527.01	24861.82	24572.66	3734.96	1636.19	3048.77	36699.43	35845.90
10	61	134	0	3	0.83	8	2	0
484.50	170	1033	1538	1995.50	1122	1683.50	78776	60
88571.64	222521.42	288184.48	72527.69	14996.92	11099.18	179671.36	243627.30	274982.91
567475.93	973987.70	1162501.38	577661.92	438503.22	410758.33	896676.56	1237205.68	1062019
7080.35	65757.79	74089.16	15824.62	4405.73	3353.36	6702.80	65460.59	74289.16
562296.44	578332.39	630663.82	470313.98	386775.33	364942.49	1059494.29	713010.94	620549.15
4521.31	29694.28	32346.35	10909.40	5609.90	2177.20	5470.48	40570.23	35869.70
378170.64	337645.67	389495.52	266599.12	197299.27	187295.89	770765.04	448771.12	387120.55
498.78	1078.67	6215.69	2297.20	607.25	406	418.73	4746.37	1399.66
72046.56	217752.99	286979.94	89926.04	78576.61	74324.08	76237.36	267736.21	244157.39
32026.74	104078.97	124036.66	29492.68	6170.77	5415.24	62390.45	107541.83	111436.89
8141.11	2685.68	5824	1133.34	1348.73	447.99	1243.01	8039.82	5681.33
80359.78	78587.42	83152.05	52538.84	49430.37	46384.21	113760.56	114413.84	85265.56
6261.18	59355.68	73545.34	12244.16	2402	2414.66	5112.34	46346.36	64447.02

8	0	0	106.67	0	0	0	0	0
227486.06	226383.10	249487.41	168271.45	124951.25	116447.98	443606.69	260723.26	250214.56
432017.48	480101.78	544435.14	373544.19	318901.23	299007.22	686401.43	586192.82	541502.15
10626.03	62291.39	101312.99	12554.63	7219.93	3652.27	9209.76	62223.19	71504.99
31239.42	25079.35	29463.82	21020.56	24059.41	22406.36	56000.67	34827.89	30391.65
33931.03	47443.96	64200.49	31472.42	24900.89	23136.63	57915.98	63515.61	58241.29
118835.28	237355.14	306542.40	72567.83	58110.79	53028.90	183280.98	236274.78	276879.98
7665.93	65180.62	83962.67	13144.55	2863.06	2935.07	5772.54	55961.83	70080.75
5	0	0	0	0	0	0	0	0
0	0	0	0	0	0	0	0	0
507126.73	971081.96	1124690.72	419924.03	254192.96	234348.21	936665.96	1006471.48	1176425.06
15942.58	141925.81	162463.74	26860.39	6394.58	5005.01	12132.57	127281.73	170262.22
64274.45	143746.32	171215.49	57608.82	29277.14	28341.36	118300.33	144899.88	149215.80
338648	363441.57	409492.99	280264.51	246891.91	229691.46	620933.90	509628.58	408880.08
38122.88	85287.99	110290.62	41129.05	28359.89	25623.53	62538.10	110544.46	98007.95
310914.63	837383.11	997692.63	230347.71	101642.70	84065.02	609269.03	848802.46	952385.91
1080825.39	2071542.74	2544950.08	1036716.33	851416.99	797767.89	1746902.28	2221962.70	2297215.14
14250.36	48645.43	62924.37	35538.85	25912.95	23606.42	6129.96	77100.49	56744.45
541066.16	746360.56	800490.38	414439.88	319336.52	292742.33	975295.38	968329.16	860011.38
118265.13	341185.20	384845.30	114005.62	81145.47	75461.82	136908.43	326241.58	442834.64
51106.70	69425.08	77235	31851.08	25348.23	23584.27	58791.42	84976.05	93915.52
31267.33	25913.35	27905.15	26697.39	23759.84	22459.82	55573.74	37794.05	28907.48
181698.55	393049.88	472950.36	170326.17	135802.49	125500.92	252789.83	490324.42	434607.04
0	5	57	118	15	0	31	2	505
3	0	0	106.67	0	0	0	0	0
2398154.89	3484496.32	4114296.55	1727308.10	1390000.52	1279062.71	4140282.09	3926879.65	3750921.17
17919.37	132501.72	187562.32	24208.98	6275.62	4912.91	12801.21	130861.72	174139.98
7753.34	1027.67	777.08	9898.16	20771.11	20595.95	248.17	1054	2303.67
22944.46	23667.78	25834.50	11429.51	1834.31	1535.39	54728.34	33681.54	24906.97
224713.03	327387.09	367368.05	103310.58	62868.74	58985.42	450726.27	321517.54	403176.24
2269.06	35536.42	59532.55	15710.19	3760.18	3540.84	5897	21935.60	40469.06
1731885.13	1401594.75	1503227.65	1407204.28	1239821.59	1171369.19	3155492.59	2032140.01	1558405.05
0	0	0	0	0	0	2	0	0
36002.87	62451.45	78262	25822.49	9960.40	5179.35	63478.27	88608.33	80124.70

498149.32	453299.19	497215.61	382578.42	293605.14	277731.55	993994.93	616006.55	498723.73
139	649	273	348	120	85	168	733	712
841754.30	740249.10	844675.04	689651.46	622439.03	588487.18	1506909.81	1020345.42	826596.99
28.67	719	134	51.33	3	4.83	25	707	497
0	0	0	0	0	0	0	0	0
262066.56	238450.44	270481.37	231370.18	200963.20	186828.46	507249.32	651501.06	268143.98
0	0	0	0	0	0	2	0	0
21014.09	2118.67	1187.15	1294.88	1384.90	509.30	53275.36	5636.57	6801
6261.18	59355.68	73545.34	12244.16	2402	2414.66	5112.34	46346.36	64447.02
26656.67	52802.76	57427.50	10737.19	3874.96	2843.16	57590.48	59765.63	57128.84
28	297.50	0	124	0	0	26	0	2.33
155.42	1023.50	290	555.83	659	139.50	276.86	1491.84	2312
143247.49	217889.15	227364.51	77843.29	56976.25	51109.51	230368.09	223326.67	259119.75
417382.73	735793.03	822483.07	360082.55	226393.93	205032.34	601458.12	919934.02	810689.45
273976.41	800965.81	986050.12	343921.06	210639.62	189648.34	458334.65	869093.55	963456.71
648968.07	1279585.71	1494331.58	516565.12	264459.58	241074.04	1339264.27	1533562.75	1438749.27
715.61	5578.84	2239	7047.84	1106.93	277.33	678.67	5988.50	6520.40
896569.91	580172.13	658129.89	723051.90	764172.81	722783.83	1446238.72	911231.17	691885.52
509258.33	750684.44	914197.92	368836.93	208464.74	187122.07	1026582.72	912364.49	895544.20
120017.84	124982.45	123624.25	79462.90	96095.40	91139.30	170677.34	163013.47	120964.28
93281.07	138121.72	142721.78	44488.05	29765.73	27566.41	170438.63	142482.47	199745.26
2591400.98	2951095.64	3455747.04	1896256.71	1587971.55	1482920.76	4906776.85	3832779.86	3454174.87
1667924.83	1636028.37	1787130.58	1363004.80	1204058.12	1133720.22	2916086.36	2190851.79	1823016.10
22234.51	17455.21	23272.50	4222.93	1196.12	947.27	54154.10	16935.26	19056.81
2642.12	27021.51	31573.34	5621.33	1194.83	1005	2350.67	18952.68	30038.41
15.67	346.50	134	12.33	4	0	8	440	0
399539.69	33205.20	17188.32	74241.85	106308.85	105131.30	473908.90	116865.23	15500.69
200292.44	271756.41	327054.98	120794.74	90184.78	78010.40	350213.81	296396.59	325599.68
0	0	0	0	0	0	0	0	0
37882.13	64280.83	75090.06	23080.25	24827.04	23733.52	6780.29	66161.28	89007.61
196483.95	290668	373199.01	113193.08	22769.70	16908.95	450271.86	372134.17	326168.12
396.67	2998.10	3314	495.33	158.10	121	285	2169.80	3250.74
3	0	0	106.67	0	0	0	0	0
17739.16	10617.02	18660.95	42904.63	47826.14	42774.43	5169.30	19320.30	21023.22

226856.92	454395.94	515334.67	204086.64	157931.51	148031.51	361326.34	548887.07	501086.50
0	285.50	0	0	0	0	1	0	1468
625.11	4782.17	1239	7109.84	1089.43	291.83	684.67	4331.50	5089.40
142	0	4	0	5	5	5	265	1468
3828.30	36232.96	22355.32	7482.95	2148.16	1240.46	2852.37	28879.14	47344.41
326213.45	335414.76	376992.04	278624.86	218363.15	206157.49	615168.94	429649.32	393226.44
649200.67	549836.88	588976.92	546236.38	479376.98	452362.50	1168202.13	782587.02	617006.39
70598.41	119498.71	135268.15	66151.65	52488.40	48817.36	118421.22	151128.05	123837.19
705552.01	738018.10	756461.57	475061.65	378363.56	351743.94	1387616.41	850894.35	764537.11
412314.13	471052.49	552501.12	311140.82	287553.44	275559.31	733877.19	562246.99	532238.32
102957.96	157361.84	182996.45	68845	53167.58	49584.71	121987.31	178472.36	170085.30
1027800.73	1298235.35	1528274.57	857673.62	704438.43	663145.88	1879998.05	1591428	1434192.31
5	0	0	5	0	0	2	0	82
81726.74	51788.74	57310.84	38249.34	25928.10	24230.14	162953.75	87542.87	54861.43
8994.45	7375.34	6884.83	18356.36	21440.23	20922.60	1024.60	6503.67	12991.34
949728.60	1016260.24	1148103.58	777266.19	644218.54	603584.25	1639571.41	1317605.78	1131341.57
48291.88	60478.61	77780.45	36276.62	25783.44	24140.91	111653.13	69881.23	60902.88
252928.25	355254.86	398952.41	205798.22	159669.33	146485.36	459540.43	452732.31	396747.22
1007200.27	1008893.67	1166850.44	612410.21	481898.47	452885.75	1997887.77	1275793.88	1163290.19
105036.75	193217.98	219363.30	106118.19	76484.50	72318.09	176930.74	235126.86	211424.55
4703.02	38111.14	46328	7914.98	2084.09	1558.16	3910.34	29468.08	52982.06
126253.21	247560.53	260693.63	115031.36	61035.59	54628.66	181883.64	285398.69	280816.70
1316912.67	1100216.54	1192587.99	1098550.25	1037352.64	983236.88	2231916.58	1556732.76	1226965.43
85581.31	784567.40	983022.50	174283.47	48760.01	35124.23	75582.59	683204.21	831138.89
1297.02	5497.34	11442.23	7092.57	3173.35	1058.64	2607.02	9281.71	6442.40
66992.61	216927.14	248112	60680.37	33334.77	30634.42	72705.87	239508.60	251140.08
52971.81	158679.27	192560.49	52982.81	29366.21	28464.74	67216.17	168614.61	166082.10
44360.22	8321.79	4288.49	48693.07	64611.82	62357.68	54155.42	29072.59	13807.84
416722.81	431224.13	527550.15	282289.52	228212.25	213506.83	733162.64	551195.10	502764.06
113804.39	414041.87	553054.49	113188.55	32958.74	23304.23	151151.97	478247.17	506670.79
62399.61	224093.53	248748.32	90016.72	35616.26	31103.63	73861.05	236309.44	293123.31
15.67	0	0	12.33	0	0	4	0	0
178098.75	486653.55	599184.45	157859.52	91849.63	83661.31	204355.51	490928.38	582244.53
33612.86	49952.26	38113.22	45122.62	26788.56	23769.36	58295.87	69767.60	55637.38

5	0	0	0	0	0	0	0	0
13074.76	36908.30	52216.83	8344.35	2909.02	2492.29	4350.02	45448.97	51847.77
17907.35	18525.85	22782.33	27896.50	43058.56	42123.95	2474.38	20307.14	19471.15
22582.68	22186.61	26425	11438.34	1531.68	1431.56	54490.34	38425.79	21344.91
24964.78	133599.71	182475.33	25097.13	6715.17	5051.07	13094.20	128320.79	175550.33
283583.66	261488.75	299846.87	164158.69	107725.77	100998.37	657930.58	371459.60	301982.66
15060.71	103069.66	141720.28	59564.48	10544.43	5900.59	13335.92	140453.09	129397.82
148192.86	314590.16	359218.57	87119.03	42792.04	37222.37	293785.06	329699.47	401161.36
573848.53	621371.73	692458.44	514072.41	441501.26	413046.54	967589.21	802366.94	689829.55
34289.95	9594.71	5232.25	14616.23	1695.88	1012.12	105597.10	19829.59	11365.87
558611.02	533923.58	639493.61	413240.67	390712.66	362739.43	1011439.53	710130.03	609842.37
338387.75	226367.13	246177.57	240027.75	254515.97	242291.73	708648.96	324545.03	252328.64
116460.08	168578.32	193514.82	83732.41	34490.93	30279.91	227249.66	204681.32	189390.90
209975.34	97357.57	102854.19	112176.34	153871.16	148999.20	63610.24	157957.99	103516.76
4972.94	37273.29	48228.67	20669.98	2665.81	1764.69	4046.71	54544.16	52026.37
29922.35	71946.37	75933.66	11282.60	2986.70	2321.96	5530.02	80980.99	71720.67
3834.67	24087.84	31838.32	16477.43	3170.37	1499.48	3355.07	34670.90	33435.31
2303.46	17724.28	25652.40	4170.54	1458.80	971.80	2103.84	26180.14	23062.47
638784.28	902704.59	977877.03	418789.24	307714.37	277622.89	1094474.80	1086734.18	959758.86
5892.51	50205.01	64176	10384	2301.20	2687.49	4433.51	50428.02	49997
300135.91	331959.44	412949.89	217053.91	161311.13	147058.96	565861.85	477554.67	386434.29
15275.80	130231.66	151221.01	24493.04	6748.10	5592.07	12321.03	111031.52	180040.63
509274.73	598266.90	655425.67	418949.72	315605.03	288047.91	912545.82	812581.62	673625.02
619170.38	589260.20	668720.80	544151.18	474117.02	450020.16	1063002.72	784379.91	651899.67
754.35	2687.27	9702.77	2951.37	1093.05	602.40	692.52	8408.85	2392.80
31.67	285.50	0	51.33	13.25	0	11	1002	3603
558102.10	551921.63	645783.34	440984.79	390067.25	364480.66	1012991.15	750013.40	645997.71
1355769.11	2363286.33	3104496.30	1078792.73	823160.52	766108.44	2150023.17	2671627.44	2718242.90
12181.69	109572.69	137721.34	22726.16	4703.20	5102.15	9560.85	96774.38	114444.02
118787.21	133339.28	144624.80	71182.57	52623.52	48526.36	223551.97	166525.82	146586.27
33804.70	44448.86	51308.15	36383.37	25479.35	23515.94	57448.65	67664.22	50046.88
39652.85	20909.02	25684.67	5415.99	1826.76	1472.15	2586.67	66639.61	29768.67
1080162.25	930176.84	1041547.85	918853.67	852504.84	809768.12	1846292.47	1354540.90	1017683.42
90014.46	40025.31	53910.73	61525.70	70228.79	65051.94	164521.22	80583.96	47208.83

774442.28	714021.54	769277.53	676820.94	595945.93	563660.59	1342198.75	995112.89	792919.39
153093.72	105576.41	114476.43	120603.85	116049.66	110616.91	275001.78	165443.22	120077.83
21566.60	7067.51	3203.17	33701.86	4020.50	1482.79	55439.69	20622.31	17489.23
59672.24	26717.20	33800.96	36982.39	45898.82	44337.18	109628.95	65329.17	30658.02
6261.18	59355.68	73545.34	12244.16	2402	2414.66	5112.34	46346.36	64447.02
12024.59	38857.18	56457.34	8164.48	1841.40	1622.33	3999.34	35760.34	48415.35
47787.37	48140.70	51232.14	25483.02	25345.74	23548.64	57821.91	64022.95	48860.69
521854.40	435746.69	481173.74	418438.15	378991.16	359026.42	942550.05	595344.24	490558.85
108603.26	284680.95	313339.82	125430.36	67076.80	60079.03	189067.32	274335.34	303426.50
439657.33	801679.37	942672.46	406650.27	288473.83	270468.65	713660.16	904342.43	966035.21
0	0	0	0	0	0	0	0	0
253291.05	812266.33	1045225.96	308212.02	223800.77	208661.05	297278	836866.88	912345.21
7939.79	2067.41	2382.50	13798.28	21425.42	20846.73	710.88	7639.92	2818.13
1728.17	18017	5344.33	7038.83	660.90	320.50	734.17	18384	24465.33
104620.30	170518.28	177577.85	93307.80	78346.70	71333.53	176987.41	206386.23	184986.15
0	0	0	0	0	0	0	0	0
5	0	0	0	0	0	0	0	0
71005.61	5155.76	1614.13	15299.39	22062.78	21296.41	105889.21	19719.60	2736.07
83407.83	240568.34	287864.68	64594.10	33827.20	32673.51	127463.91	226998.97	299645.64
19739.43	105743.69	121867.93	47744.32	7669.78	4915.71	10138.10	100628.11	134986.64
5	0	0	0	0	0	0	0	0
81918.51	109911.90	127047.04	56456.18	30969.61	27227.92	168941.76	120584.12	121785.44
0	0	0	0	0	0	0	0	0
27680.55	105576.20	126785.21	35739.92	28920.93	25781.74	12075.62	102033.06	117632.91
248084.90	271546.51	291344.05	193687.74	173633.66	160740.51	451634.91	340547.84	314809.07
29644.71	79355.81	95516.22	25430.49	6146.11	4567.21	60835.15	88180.57	85904.18
5572.69	50849.86	55180.40	10961.13	4093.29	2565.47	5277.21	42249.19	58429.54
2819.10	21055.51	24656.82	5819.01	1934.46	1465.19	2549.77	27513.93	22082.90
64662.89	81798.62	93364	29875.20	25690.30	24731.97	112632.73	90551.57	93780.66
98002.13	700927.14	941830.72	161657.42	62579.49	51835.23	68982.44	674108.41	751662.16
229610.45	263942.93	297554.43	225088.21	173983.35	161393.83	399099.97	348194.10	308078.84
47433.86	273052.98	320831.52	54883.74	14527.79	12821.31	25575.98	267238.25	359859.07
37814.63	314238.51	427417.36	66834.53	21786.02	17109.25	33046.43	350729.88	391665.99
560334.64	511054.26	573167.69	452711.64	408304.90	385673.68	1005761.47	704814.62	583099.93

75605.96	172939.09	197959.15	126577.43	52072.36	42663.47	137791.74	118092.13	197380.25
191.50	4200.27	798	694.17	123.70	48.50	205.50	9012.80	8129.40
6263.18	59355.68	73545.34	12244.16	2402	2414.66	5112.34	46346.36	64447.02
0	0	0	0	0	0	2	0	0
557767.75	649991.11	769809.77	489537.70	420152.92	394924.04	969023.79	888667.81	729765.76
35.40	275	67.40	154.70	236.20	56.40	176.36	666	946.74
681942.41	460588.24	516856.06	532074.37	531933.60	508158.28	1264273.78	714749.34	521464.66
119916.27	375566.40	453586.27	131049.98	68855.05	59291.40	196215.62	381029.29	416703.60
360553.99	356406.32	400151.66	274886.90	202337.68	188444.15	722238.14	486066.76	389048.45
547526.79	228812.15	258644.63	381157.36	516689.23	490819.69	877243.37	366088.73	257778.59
23.67	658	0	51.33	0	4	17	440	497
29941.23	94833.74	108730.41	19700.41	5268.09	4649.03	61040.85	95683.79	97694.63
9249.53	73493.14	99389.17	15879.74	5778.46	3808.57	8253.14	86650.33	75252.04
326877.85	409283.88	451546.41	295935.09	245204.22	231544.65	570421.57	497726.04	470448.50
2819.10	21055.51	24656.82	5819.01	1934.46	1465.19	2549.77	27513.93	22082.90
714188.88	926666.42	1036199.14	455143.29	282762.34	261259.17	1457793.77	1087657.58	1065876.83
358760.01	468428.05	653182.46	320423.27	288952.59	274723.89	528868.88	644596.44	558335.61
145904.14	229755	269371.24	79497.84	36044.69	32219.05	336530.50	247182.64	258082.24
1097037.25	1611159.29	1971501	865541.40	640451.32	590655.61	2017024.51	1939497.37	1840330.53
50550.44	66873.67	76051.82	41895.50	46676.77	44337.05	59724.85	66623	89766.96
162107.84	73810.47	84226.21	95302.67	112543.49	108388.81	323788.55	114452.45	83437.52
350614.45	333445.04	379225.43	298274.21	265409.10	250050.56	617395.06	480777.20	371264.71
432.51	1322	2215.34	459.31	289.10	134.83	335.50	15848.34	6410
33877.44	54998.19	60136.49	38196.54	25499.44	23609.32	58281.41	58333.40	63481.62
6536.04	42246.02	56164.33	13091.64	2132.95	1408.99	4119.85	44844.68	70011.67
259389.22	2115603.18	2763047.33	493427.16	131027.91	102299.93	260511.73	2059647.26	2326510.67
1092.67	11829.67	15616.67	2533.33	466	448	1014	11034.34	13450.67
259013.27	635100.63	733598.24	186313.44	82451.12	72083.43	375821.48	641824.74	834714.65
388938.19	382970.60	422028.32	283208.28	224032.85	210756.54	724503.48	505130.87	450868.29
5	0	0	0	0	0	0	0	0
0	0	0	0	5	6	8	0	6
401378.89	619281.79	714101.97	292069.35	138297.60	121389.17	803434.49	881057.27	747327.95
5	0	0	0	0	0	0	0	0
0	0	0	0	0	0	0	0	0

129307.83	146538.84	156342	111925.74	98109.96	92130.36	226495.12	180033.50	164141.48
246317.67	217244.16	243581.87	224697.48	197552.57	184382.73	451205.48	547791.42	246075.18
458776.16	290242.48	290231.91	378101.76	351294.96	330333.07	778398.16	442721.80	346155.59
0	244	536	0	0	0	0	0	0
115239.74	177176.59	214122.39	120026.75	81752.58	72450.62	180130.42	247835.41	209817.21
945321.80	736083.08	827518.17	644458.53	515523.16	480158.47	1818475.14	1028058.82	853887.56
544835.63	577881.26	604837.01	413533.25	400247.30	369731.96	859094.38	684070.92	610365.97
115917.58	473195.82	569219.86	106062.73	28556.32	20295.66	202965.81	427695.98	544945.32
458073.84	833784.27	978955.63	409275.17	277908.94	255345.89	825108.62	887264.11	905433.96
87256.63	88559.14	101507.53	66240.90	75680.63	67606.04	171292.70	99088.77	102503.94
514084.47	131800.31	140424.99	388159.16	485653.71	465465.34	662114.02	313254.58	182980.95
16273.39	4715.01	13263.92	23676.32	43541.81	41773.62	1682.72	8569.31	3825.41
2	0	0	0	0	0	0	0	0
368852.95	755566.38	856434.88	230499.28	109321.37	100513.89	752455.38	756267.67	923419.30
164773.40	103189.96	116317.10	80944.50	92557.11	89894.92	326512.62	143216.75	113560.05
109973.22	172951.70	184999.54	76522.96	74381.07	69956.34	69232.08	209846.76	236240.37
0	0	0	0	0	0	0	0	0
0	0	0	0	0	0	0	0	0
553444.60	819210.32	944039.84	341740.11	226792.43	209624.43	1079519.33	889638.35	1022478.58
43.50	13	61.50	129	115.25	69.50	109	2	4
11877.78	34740.02	42017.15	22091.47	23471.47	22657.59	4351.98	43556.15	39984.65

D15	D16	D17	D18	D19	D2	D20	D3	D4
0	0	0	4.60	5.40	3.80	0	0	0
597161.72	559233.02	404953	271767.43	253745.63	889722.85	274597.26	883062.39	416433.78
2205431.82	2862996.33	2035216.29	1507987.27	1427640.46	2973056.74	1440617.57	4362087.20	1381560.88
443762.20	649934.91	465801.09	282670	264533.31	655287.19	271458.53	1036833.77	324010.79
53404.29	44927.76	30955.98	25635.40	24051.65	63888.55	25716.80	66410.05	32776.83
18602.99	10189.96	1542.84	23324.86	23219.78	24794.13	21210.06	3403.95	14981.83
177899.70	260561.59	182815.53	148371.96	141521.39	227490.08	139964.20	389703.50	109243.51
0	0	2	0	0	748	0	0	25
5398745.69	5673611.72	4256421.45	3772582.76	3602976.76	7074947.90	3616566.77	8154842.52	3956822.01
41452.62	61997.76	31751.75	69672.55	68511.85	48558.93	62269.78	69062.71	27945.50
192793.51	96652.65	64905.95	57177.97	52484.56	319902.08	62789.16	150757.78	167000.80
619453.08	894165.26	665135.91	143752.42	116080.73	931997.08	156957.61	1593523.74	452614.26
42855.64	2817.33	1043.68	2311.60	1502.81	76415.16	5473.81	6068.18	50125.32
54193.63	12329.96	17818.92	25327.52	24156.70	71238.68	27531.91	7466.05	52310.32
1870.50	126	33	91	69	130	59	137	2074
18598.32	1694.63	1085.17	1372.53	1215.28	24605.70	2492.05	3334.62	14981.83
489754.23	472498.77	361772.90	323980.08	309817.68	541497.53	314817.81	678045.93	231866.65
272085.64	267497.43	216430.75	152356.72	143434.67	354008.10	149798.01	396673.16	177163.64
188015.81	53401.75	64639.12	32657.20	28499.75	305933.48	42531.66	83850.09	167232.63
2556.18	538.26	30853.64	79.86	186.20	7220.40	583.80	347.40	249
266798.21	385857.41	252042.41	131764.54	121203.34	401604.37	127295.69	641954.12	176812.97
0	0	2	0	0	748	0	0	25
327978.32	438060.73	290323.94	249433.03	237269.86	487067.89	235285.57	655830.63	207932.49
1043629.82	1993698.39	1792764.84	432834.46	377888.97	1300258.53	428128.97	3465761.18	640593.25
46353.94	3532.93	1869.51	3045.34	2124.58	79354.44	7015.96	7532.86	39169.66
0	0	11	0	0	0	0	0	0
269092.42	544323.26	414785.57	203681.68	189263.26	342375.67	187896.72	886397.57	150521.16
111887.71	81659.44	77094.77	29786.65	26772.45	179829.55	33424.57	137446.98	97081.31
173823.30	103145.80	64107.09	77609.95	73351.47	236527.37	80590.06	145893.83	168079.63
366865.45	553216.38	460168.52	38872.81	21358.59	673858.74	57094.19	1030160.42	315351.95
600942.97	165248.34	180865.15	102221.20	88412.42	914490.80	134478.51	255459.71	608022.52
0	0	0	0	0	0	0	0	0
4108	131.67	89	160.67	119.33	7369	292	422	179

385193.49	332085.16	271417.41	52782.97	38319.91	674977.16	69110.33	595169.41	333265.93
0	0	2	0	6	748	0	0	25
8181.34	33172	29264.67	1463.98	536.70	2402.50	2240.17	61199.80	126.67
12627.80	33450.70	28922.84	1524.90	522.89	19872.48	1795.89	62284.74	14180.33
55873.25	4669.68	2708.18	4681.67	3105.61	85883.67	9611.91	11269.21	51231.67
32088.45	44339.97	6329.35	111488.95	111734.90	40275.09	98968.65	6180.03	7533
8222.66	818.52	586	606.83	377.15	7246.30	1370.82	721.17	291
1419935.70	1522649.43	1169396.09	820176.67	773613.43	1989473.09	803063.03	2325609.41	961679.58
1536387.51	1656978.54	1215140.56	756495.92	704833.60	2066931.30	763566.49	2640393.46	1152662.81
0	0	2	0	6	748	0	0	25
9689.78	17527.71	1696.84	44276.60	44442.77	16875.35	39310.31	2367.97	1941
3487.80	374.34	207	416.67	154.66	2872.73	445.16	860.62	2642
42855.64	2817.33	1043.68	2311.60	1502.81	76415.16	5473.81	6068.18	50125.32
0	0	0	0	0	0	0	0	0
250892.85	92757.80	83914.16	39419.41	34226.52	336494.10	53186.67	158545.93	205177.31
101224.14	81257.31	123616.44	29527.64	26767.63	151515.90	31920.49	137294.35	80646.82
175671.43	201984.01	167124.75	80986.43	74231.31	229522.79	80141.59	325701.38	97567.66
106408.68	132264.45	92071.90	75381.19	71632.06	155262.06	71827.04	197008.44	66892.33
0	0	0	0	0	0	0	0	0
28097.32	2216.63	1261.67	1509.53	1439.78	30715.70	3162.55	4255.97	15504.83
2.50	52.50	13	3.50	21.50	2374	1.50	67	25
112	348	120	252	132	60	204	1982	0
198374.61	112226.04	93047.72	14128.18	8248.25	286733.58	26170.67	205883.49	182555.79
984455.01	707716.66	508382.71	444414.62	419246.41	1413443.47	450290.30	1043371.89	712043.90
58592.93	4344.40	2655.68	3756.23	2915.29	80244.76	8121.76	7940.89	51179.32
508857.19	795362.36	539594.69	398357.64	377128.86	803869.20	373878.07	1230917.48	367471.60
36670.78	3285.46	2505.17	2736.93	2179.21	63184.95	5360.07	7227.14	16966.33
309261.60	547949.81	400403.22	204823.02	190299.43	493113.79	193993.22	894713.34	231672.65
5544.31	604.38	1498.50	654.59	649.75	14086.34	806.55	874.42	1961
215343.42	74404.40	38316.68	79640.39	75332.03	315322.55	81168.53	88012.51	164345.64
83365.59	38847.11	31620.69	5813.40	3717.56	134114.79	11113.30	72571.96	80508.15
7443.33	423.98	15828.17	310.69	237.75	2485.67	875.15	938.13	3158
78206.69	87894.54	91854.33	50181.68	47620.65	111289.54	47854.25	132432.97	46617.33
42855.64	2817.33	1043.68	2315.60	1502.81	76415.16	5473.81	6068.18	50125.32

0	0	2	1	0	751	0	0	25
199065.70	318100.67	238306.31	128098.66	119486.46	302350.15	121951.03	515218.79	148051.80
476089.80	539229.76	430095.50	325267.22	310089.08	674355.06	316127.02	800683.27	323132.78
65611.92	5145.72	3531.51	3302.76	2724.44	90199.02	7182.34	8231.26	57874.99
30271.79	43126.60	30554.91	24688.55	23566.47	43394.82	23204.68	65183.69	16026.83
47228.58	44500.70	30784.25	25585.61	24125.87	62103.56	25606.28	67118.05	40888.66
210871.37	128740.92	124409.57	57376.10	51753.68	314173.32	64741.60	209543.58	197541.30
48566.44	3427.83	1405.68	2711.43	1852.01	82590.03	5760.65	6829.28	54868.32
0	0	2	0	0	748	0	0	25
0	0	0	0	0	0	0	0	0
847179.97	658358.72	490126.29	258439.49	232065.81	1317472.33	286843.57	1081560.59	727850.68
103336.71	8181.44	3149.59	6405.92	4091.95	158697.31	13661.60	12839.22	103144.31
119573.55	82647.93	61469.27	30561.66	26887.87	200473.72	34099.92	137577.03	109286.81
359055.96	473961.61	321080.48	253039.97	239384.27	497322.87	241056.47	724174.95	236776.15
86843.39	47128.85	32261.59	27671.46	25511.12	116155.79	30016.37	72681.19	68165.32
680060.69	396445.50	299070.78	93903.04	74818.32	1120357.08	129768.21	704796.10	625640.87
1861287.56	1355425.58	907285.59	849243.81	803007.75	3091057.44	867729.46	2012751.77	1600628.69
53176.27	12230.66	2684.31	24847.88	24429.29	78261.77	25002.74	7758.48	33234.33
692865.94	711901.69	554389.91	323334.81	300114.26	985982.78	326361.73	1137422.28	483154.83
276942.37	109682.40	97172.95	82605.43	75747.78	384240.79	91968.92	154749.90	250453.44
67336.56	45172.48	61317.59	26079.65	24052.42	84595.32	28122.44	67623.34	46285.99
29361.13	43466.93	30471.41	24683.30	23563.77	37735.13	23020.35	64677.69	17041
366107.76	200921.41	163645.93	134293.74	125827.95	578302.09	138362.20	291893.03	277817.29
961	6	11	0	0	42	35	33	24
0	0	0	1	0	3	0	0	0
3036769.08	3029890.81	2417116.18	1417568.54	1317758.51	4177865.19	1414773.95	4787188.52	2447668.29
99638.92	7189.95	2652.86	5881.05	3889.45	148626.78	12433.07	12127.37	115044.64
6109	8460.13	282.17	21883.62	21903.25	3025.58	18792.39	178.25	214
21911.63	34335.15	29491.84	2129.27	999.47	33093.93	3147.44	63169.16	15401
275363.45	298435	254471.59	66494.84	55447.01	473915.57	72389.20	520722.62	235777.45
8237.10	1746.99	1264.50	1456.04	1401.26	43036.30	2935.46	7366.85	15617.50
1519915.91	2425187.66	1721907.41	1291616.01	1226305.29	2044489.09	1204876.76	3669922.42	877847.33
0	0	0	0	6	0	0	0	0
71675.76	38715.92	48308.58	5294.18	3383.03	93238.52	10940.97	71483.89	40333

459534.75	724408.37	509178.89	307006.94	286885.50	645244.58	291264.54	1153813.11	294306.14
2385	110.67	76	120.67	73.33	5977	154.33	304.50	77
805805.45	1164546.31	820523.62	645307.52	613889.30	1087246.21	606978.21	1750927.91	508322.97
1171.50	104.50	31	49.50	41.50	1818.50	15.50	126.17	8
0	0	0	0	0	0	0	0	0
245655.65	383994.27	242482.95	202066.40	191115.87	361048.47	189433.18	591521.40	150288.80
0	0	0	0	6	0	0	0	0
10541.34	33487.50	29432.67	1769.48	689.20	7025	2659.67	61323.97	228.67
42855.64	2817.33	1043.68	2311.60	1502.81	76415.16	5473.81	6068.18	50125.32
54617.85	36397.55	30875.01	3724.44	2044.05	97442.66	5423.49	66688.02	37830.66
120	117.34	40	98.67	57.66	295.67	181.66	68	0
3077	296	601	252.50	240.30	7178.50	344.50	563.17	1696
182344.01	161550.64	167873.36	59317.98	52475.51	273601.13	64788.66	267126.27	135743.31
704326.30	444306.10	453052.04	223332.41	205269.92	975327.43	238765.89	699144.17	506737.43
731463.25	344290.73	188662.75	203935.92	187570.72	1051373.81	232317.35	530966.52	629860.91
1161028.75	905123.50	646866.17	265251.20	231096.72	1774288.18	305043.25	1555503.17	920051.52
6134.33	491.72	282.50	332.19	236.08	9356.23	868.05	831.75	801
764021.79	1181849.11	870171.98	792486.08	762610.78	870522.88	727761.12	1676844.49	353361.31
678752.96	696035.53	544716.75	207186.71	184087.22	997814.39	225493.07	1187579.85	561192.57
121609.16	140704.39	123757.25	97901.18	94766.69	146200.23	93261.66	198960.50	83560.50
114170.54	114947.21	105509.87	31496.43	26701.47	185986.67	33445.24	196959.31	89730.65
2908282.83	3588574.07	2590534.11	1630492.90	1521554.61	4181278.86	1593341.31	5680673.61	2069860.73
1727146.74	2248829.05	1639849.75	1243483.35	1180622.56	2290302.83	1184472.47	3387052.50	1062074.08
15178.10	33488.05	29226.84	1589.58	572.89	25935.83	1951.39	62345.14	14868.33
20383.65	989.22	447.84	846.80	591.30	34643.93	2815.20	2528.76	22817.66
0	46	18.50	0	20	1826	107	67.67	0
72204.73	338036.85	626918.53	119136.76	111121.55	31926.48	102086.90	545239.59	11377
283539.75	242464.29	232712.21	87701.29	78799.71	374510.82	98603.28	404971	188219.14
0	0	0	0	0	0	0	0	0
64008.95	12240.76	48083.19	25181.47	24055.68	75075.62	25603.78	6760.07	45572.99
280973.55	278585.40	238337.98	20617.60	9975.82	385489.34	35591.18	518489.19	224612.28
1449.80	233	79.50	101	111.50	6523.63	387.50	399.27	778
0	0	0	1	0	3	0	0	0
31512.95	20171.05	4395.34	46214.59	46169.40	24571.79	43959.72	7162.39	3779

430111.94	275013.83	203054.45	159024	147664.11	658471.73	166040.15	416387.11	323894.28
0	0	0	7	0	203	0	16	0
6620.33	518.39	320	380.86	245.41	14138.80	836.71	1029.75	691
0	4.50	11	9.50	1.50	9	1.50	0	0
22672.79	1858.31	1177.17	1707.42	1301.95	42587.28	3842.74	4064.91	13542
358083.32	460948.11	316835.88	227371.93	213947.28	484777.28	218073.93	713154.40	225540.31
609103.40	903998.26	637350.21	496244.75	472378.69	803124.89	464802.23	1359060.50	347466.49
120583.99	91304.55	63049.93	52818.76	49779.51	178078.89	53012.71	138434.09	80310.99
658749.49	993010.15	747990.03	389687.44	361680.66	1016075.51	374233.02	1611735.41	451351.45
438949.21	557341.48	393142.22	299657.36	283948.73	653423.05	285034.77	850840.58	323485.10
143140.58	93124.22	124348.35	53837.11	50082.70	218141.79	56441.94	141778.86	109469.98
1307625.21	1414028.52	982097	724440.90	681160.90	1830713.74	709158.93	2181550.70	918660.21
0	3	0	4	0	3	0	13	0
53943.73	109760.16	103548.09	27000.99	24117.89	74544.41	25533.84	187949.60	33335
8830.67	9136.89	752.17	22335.27	22293.19	11924.13	19569.90	1566.55	3579.50
1022732.76	1248350.28	962800.01	660154.50	624478.20	1427141.86	638087.69	1906826.14	673277.77
54508.93	77906.63	44471.65	26684.27	24445.35	89471.43	26395.57	128260.93	45083.99
344672.59	338091.68	245820.83	159483.40	148741.06	480330.98	161273.64	535159.48	242098.97
978368.48	1406959.56	1090754.04	504317.95	462544.30	1412314.80	492543.14	2312969.24	704291.06
168064.67	135928.70	93355.49	78875.09	73749.13	248797.26	81281.64	205654.91	144273.31
27976.72	1847.66	812.84	1774.21	1124.46	42920.99	4017.66	4388.58	27807.66
215587.44	131180.56	140735.93	58574.51	52945.70	371881.56	66101.36	212018.97	164267.13
1210405.51	1775028.71	1278710.04	1077639.09	1030748.28	1615024.31	1006959.28	2596625.61	703152.31
621034.16	43524.19	20413.47	33441.95	24279.80	1084602.89	73180.20	87256.95	606730.03
19739.63	1797.15	2656	1729.62	1574.21	19432.77	3547.87	3959.55	2471
179609.15	52927.37	64429.95	32209.37	28227.02	288825.72	41242.43	82704.87	155921.30
126087.51	49971.93	47908.44	29544.23	26566.40	228488.11	33737.02	77811	123261.97
20444.44	59126.06	30257.58	67767.55	66475.46	23667.24	59982.26	62386.23	1712.67
455597.78	533647.64	455483.02	235538.79	219187.30	610712.75	231562.90	847513.71	308916.96
410445.11	95242.53	120717.56	24798.57	16640.60	546277.03	49311.96	172571.06	335804.94
191904.79	55030.59	50925.55	33606.54	29733.80	325423.26	44073.84	86580.91	166257.96
741	0	0	0	0	754	4	37.67	25
415383.99	152023.99	176216.16	91316.89	81835.37	594738.73	112385.42	234350.64	378051.91
40391.82	45800.43	32060.41	26402.83	25008.57	64753.76	25966.85	68944.10	17868

0	0	2	0	0	748	0	0	25
41854.78	2609.76	16634.51	1927.55	1384.36	59148.54	4192.60	4852.75	31796.66
22418.80	18057.79	1038.91	44716.27	44514.83	33430.39	39687.09	2398.60	13607.33
16623.30	33772.55	29042.34	1610.35	698.79	29192.86	2042.56	62879.49	15013
108036.94	7971.55	18481.53	6859.21	4341.28	160626.80	13982.72	13227.34	116432.64
249760.33	443696.38	309122.68	112716.93	100266.19	348951.68	109311.83	762365.66	171482.49
106642.87	9333.73	5556.45	7323.48	5766.39	151958.13	15097.09	16239.32	81742.82
272095.88	192038.99	153852.74	41846.65	33651.47	434143.50	53470.80	341103.04	232162.13
624729.79	755716.67	551556.04	448037.64	427985.12	919340.43	426794.72	1129379.36	401679.79
9550.77	65934.63	42406.67	2124.43	600.65	12120.05	1434.22	122047.80	1384
564174.21	767826.55	547134.25	403535.30	380623.28	707031.36	386887.37	1174476.91	363865.47
213912.19	534085.39	308934.59	265393.74	253347.62	319502.91	239245.63	823787.82	137945.85
150131.84	150370.77	136306.11	33750.05	28618.70	217773.06	37891.54	263579.59	113185.48
105271.69	99420.09	294813.70	159960.41	158385.32	142513.54	145117.76	74178.08	65623.99
36248.94	2830.82	1496.34	2025.53	1483.45	63903.50	4343.99	4577.21	29735.66
59048.49	3560.98	47121.52	2764.44	1762.24	62443.84	5383.97	6084.67	45621.99
29724.45	2385.22	1506.07	1936.97	1524.04	36347.15	4070.94	4308.42	18130.83
15881.79	1109.28	533.17	820.63	602.03	30559.98	1857.10	2216.14	14762.33
786778.57	777004.70	721561.95	307305.98	281436.61	1208612.96	317368.09	1270985.33	564099.25
41437.98	3144	1151.01	2286.25	1467.48	78802.51	4555.48	5387.51	41567.99
367487.92	405466.62	319509.24	162939.73	149673.16	491900.65	164002.88	658278.04	240058.65
98705.40	7181.34	2964.86	5792.70	3586.29	169949.79	13986.04	12480.84	102460.64
603366.52	675570.47	524878.34	318158.48	297907.85	851681.53	316525.05	1065437.19	371604.63
516224.49	830149.22	587569.72	486361	466040.53	859245.32	453952.92	1237885.60	389834.44
7096.43	743.38	2037.50	841.31	894.45	15133.27	998.55	1219.09	2048
0	9	17	29	3	971	68	37.67	25
624365.61	763325.23	544649.03	398195.28	376806.32	783779.73	381275.34	1168883.84	380154.65
2198470.39	1587353.29	1313137.26	823379.65	765521.95	3267484.73	850397.34	2473756.78	1888188.10
84293.62	6000.33	2203.69	4629.85	2979.29	155287.67	10041.29	11510.69	91701.31
129365.31	157190.66	135754.41	54855.29	49638.79	172946.54	54884.19	258019.77	90533.32
49631.62	44824.31	30888.08	25560.40	24199.95	66747.10	25217.90	67043.95	30615.83
24294.69	2084.66	77286.52	1647.91	945.15	45481	2213.65	3592.73	14292.33
1044789.03	1462531.71	1033618.59	882837.06	843990.61	1416332.09	823796.68	2147242.83	588447.64
73055.21	127939.28	90235.66	71767.41	68728.45	71461	66261.13	189133.68	32348.17

750008.35	1056694.98	745853.76	618778	589989.70	1000350.53	580944.24	1561774.51	447705.16
120532.83	214927.35	150099.88	121623.13	116094.74	156514.88	111978.87	319319.54	65566
15445.15	33675.09	30033.67	1462.18	554.79	25836.97	2404.19	62644.83	2391.85
38194.77	85705.24	60293.15	48631.10	46419.10	44341.26	44120.42	127628.03	18407.83
42855.64	2817.33	1043.68	2311.60	1502.81	76415.16	5473.81	6068.18	50125.32
30426.32	2426.32	16230.68	1927.52	1292.89	45656.71	4471.49	3804.02	38053.99
49430.29	44081.72	61389.92	25164.28	24092.87	61575.85	24179.11	67619.68	30977
470138.12	726564.53	512975.84	394771.48	375328.62	633662.94	368757.50	1094604.63	289655.98
239676.48	135932.90	99335.20	65136.76	59092.42	353939.54	76596.40	221716.61	203303.80
699569.95	536904.40	404724.55	292892.29	272785.90	1115628.05	301442.47	827603.93	583974.22
0	0	0	0	0	0	0	0	0
718430.38	247847.71	175129.50	216614.44	203374.46	1126091.73	239350.82	340825.04	646804.19
8192.16	8677.62	598.07	22121.50	22065.46	5612.97	19338.57	667.50	626.67
14029	882.84	365.50	705.17	345.66	12522.17	907.16	1303.67	2307
158400.59	137020.73	94719.14	79132.33	74030.16	238086.60	77759.90	205072.71	103331.99
0	0	0	0	0	0	0	0	0
0	0	2	0	0	748	0	0	25
6307.70	74172.45	103710.42	23972.25	22447.10	9383.97	20114.75	122366.08	435
204361.41	88444.79	78960.46	35133.54	29684.55	353128.42	45297.04	146113.54	190129.12
95737.86	6622.96	18635.69	4873.71	3556.08	155633.64	11933.39	12438.76	83760.48
0	0	2	0	0	748	0	0	25
91946.73	113859.45	90404.83	30402.27	26889.95	154077.93	32346.34	196797.28	74019.65
0	0	0	0	0	0	0	0	0
90441.80	14926.13	19236.26	26535.79	25380.55	122466.51	30628.92	13110.22	82583.48
281191.19	340467.59	243218.72	176169.48	166511.46	387018.46	170737.65	525039	173283.32
70292.93	38007.79	31520.35	4924.60	3189.53	114798.04	8602.14	71350.89	59945.49
38072.47	3359.10	2010.34	2632.34	1997.29	63709.62	5046.26	6114.73	31687.66
18598.32	1694.63	1085.17	1372.53	1215.28	24605.70	2492.05	3334.62	14981.83
69673.65	78273.62	74898.26	27276.85	24532.01	123515.38	27420.83	129566.63	59579.82
575821.35	51742.20	34635.29	54649.10	45813.99	892554.04	85163.31	80495.20	551772.55
286065.77	310441.44	215794.55	177745.97	168591.66	374231.32	173486.82	464467.45	173430.49
225836.22	15648.86	37052.56	11725.31	8009.53	390995.48	27535.34	28256.24	212113.28
296003.77	21827.38	12042.56	16449.97	11455.13	428807.07	33165.74	37254.82	259660.94
545745.12	774447.24	546621.99	423618.54	402156.26	725106.52	399701.22	1168399.17	340582.98

59705.62	89772.77	85050.48	39117.26	37740.68	176653.93	43586.99	176665.19	63067.35
2327.80	277.50	146	235	197.50	3709.13	336.17	308.60	281
42855.64	2817.33	1043.68	2311.60	1502.81	76415.16	5473.81	6068.18	50125.32
0	0	0	0	6	0	0	0	0
687649.16	750820.88	521753.01	429523.75	407312.42	939053.76	412617.77	1126275.92	450586.64
10	56	82	27.90	31.40	674.80	131	87.40	87
507525	985194.18	642751.20	556705.65	532439.33	712737.51	507188.12	1467079.36	291352.17
343477.29	137195.40	98510.45	63585.04	56416.35	492758.22	78816	224058.23	282364.27
370036.57	520910.50	376361.05	210092.29	194724.27	507405.69	203463.36	838384.11	233755.81
334928.87	737809.52	513314.93	536435.16	521173.99	352831.89	478823	1016800.33	134343.32
1169	43	16	41	17	192.50	11	59.17	8
69223.73	37520.06	30769.52	4616.48	2697.42	136325.17	8736.13	70463.40	68406.65
71094.58	4848.39	2724.18	3716.02	2688.78	102787.13	7723.05	9066.70	60521.99
382479.56	441520.72	306906.63	253248.42	239607.93	603545.38	244888.30	664807.21	274455.47
18598.32	1694.63	1085.17	1372.53	1215.28	24605.70	2492.05	3334.62	14981.83
851712.44	996833.14	783110.73	292725.35	260182.97	1270761.57	303124.84	1690131.30	646224.90
465476.24	426207.97	322729.40	293740.21	281197.12	673090.19	282355.15	609004.26	389649.42
202035.44	217088.06	163201.53	37174.09	29473.75	289531.85	44793.41	387492.19	174454.13
1560155.96	1452158.02	1084647.03	641788.10	591564.14	2199069.54	655886.73	2327213.34	1166001.64
59556.14	53687.45	47068.66	48483.66	46302.88	92942.44	47440.33	68293.74	53474.82
93918.07	244406.47	161748.81	120148.40	114176.83	110336.06	107307.19	373823.70	46955.33
367190.23	481960.17	336149.52	275059.95	261358.01	477186.84	259647.36	717992.59	224076.16
3487.66	605.50	296	419.66	297.50	5804	527.50	388.01	187
45851.28	44758.76	31124.25	25769.10	24244.76	66671.73	26460.34	67268.62	38935.66
29747.81	3016.26	1137.34	2525.66	1423.70	41379.60	4353.51	4405.04	34416.66
1741527.37	150536.08	80795.64	90932.38	66110.38	2870681.92	207184.67	300202.12	1718538.44
9525.66	610.33	192.67	371	307.33	17361	1219.33	1182.67	11209.33
548873.70	252165.52	282083.46	79929.09	65309.57	888801.74	109725	433972.19	478541.39
398468.77	530966.27	407518.33	233416.28	217215.98	555652.47	224979.38	841659.34	252768.81
0	0	2	0	0	748	0	0	25
741	71	49	69	52	0	113	5.50	0
490801.36	537835.79	441934.94	135681.39	117615.43	807298.46	142685.14	942801.66	411518.94
0	0	2	0	0	748	0	0	25
0	0	0	0	0	0	0	0	0

147917.29	174504.64	121789.78	98705.18	94405.17	205620.53	94643.40	262899.19	93248.66
229617.36	349786.49	228429.28	199939.79	190218.24	330573.30	187137.08	526625.83	135009.47
415508.12	613581.05	474271.87	366383.45	349613.82	411947.18	340829.11	903466.38	157688.50
0	184	28	0	80	6504	0	68	0
187152.52	138557.87	111727.68	80569.02	75694.68	259745.30	83102.02	210670.95	119606.48
851385.11	1315308	1032870.92	537669.41	500068.55	1059726.07	513428.48	2109716.44	476191.29
616514.32	668188.85	569740.01	398855.53	379894.51	828016.25	386847.16	992133.40	369103.32
353581.10	126937.08	99809.56	25385.20	16008.53	592816.45	49934	230853.05	359878.42
731609.42	598233.86	435411.09	276474.14	255090.43	1315167.38	291116.30	951601.29	615640.07
88082.22	129018.67	76308.73	73170.80	69994.50	103145.19	70466.15	196940.86	59201.49
379255.81	595133.25	532845.10	509361.08	494201.54	262006.90	458534.19	761849.45	79624.17
10492.63	17899.29	2770.84	44712.05	44910.75	24297.72	39322.07	2480.38	3838
0	0	0	0	0	0	0	0	0
649672.29	497959.61	379913.94	114054.35	92404.98	1122935.74	138465.58	871410.39	543706.22
109350.98	236747.55	176976.98	98740.84	93002.40	143752.19	89880.52	377830.06	64952.82
172780.79	69361.99	126542.69	75728.79	71968.54	232672.82	77093.88	80018.04	111107.81
0	0	0	0	0	0	0	0	0
0	0	0	0	0	0	0	0	0
733727.57	741537.14	588539.50	234404.75	207666.53	1139981.76	250173.04	1248517.32	600886.22
2.50	10	10.50	0	0	773.50	0	110.50	25
33482.28	10718.27	1563.91	23752.99	23371.05	52433.19	23387.73	4996.60	27152.33

D5	D6	D7	D8	D9
0	0	1.20	0	0
682789.74	763676.66	811194.09	254202.32	744908.32
3606440.18	2615394.33	2790397.56	1346671.19	2635829.72
721985.16	614604.07	629468.74	256212.24	585945.64
68548.77	61869.19	59464.59	23066.96	54629.12
26804.79	28724.56	30300.06	20496.66	30565.45
327857.61	217538.64	220408.66	130932.47	234680.13
0	0	0	0	0
9055733.27	7767434.03	7399566.97	3403141.66	7185232.05
75643.59	72639.19	56467.02	60624.84	66774.05
178657.24	294015.70	283808.92	59142.11	239935.95
829550.65	777034.59	855642.73	141199.71	770297.22
2722.68	73032.38	74155.02	4220.18	50201.14
115730	81740.60	71520.91	24272.68	86884.20
102	6355	1287	153	3912.45
21770.46	28690.90	29857.73	2330.99	28853.12
1003883.85	560556.51	452669.23	298033.66	616369.30
644537.93	360192.60	339521.16	137968.24	396747.95
130830.58	280027.39	277202.02	36736.60	211952.41
62541.26	2623	9472.60	572.40	18253.80
400793.33	328639.48	369596.36	119977.68	325565.51
0	0	0	0	0
535099.57	408013.09	442509.32	223079.26	414962.67
2397236.97	1191989.02	1363130.96	387110.60	1446808.24
32339.36	85313.66	83465.79	5430.94	48487.25
0	0	0	0	0
399496.15	313836.18	337033.10	175722.49	313216.08
163980.61	157187.94	172915.59	31700.53	161657.46
179964.42	274853.28	255821.52	72675.24	236331.39
260236.37	518884.20	593531.30	49298.30	484229.39
318917.39	995649.79	884368	112062.63	732780.65
0	0	0	7	0
272	944	716.33	318	326

271970.51	577547.37	605339.36	58652.58	431597.04
0	0	0	0	0
614.13	3019.38	1693.23	1190.85	1155.40
996.47	21868.34	21965.57	1549.02	15333.45
143829.28	107135.39	70939.51	7529.69	94612.04
122854.18	31687.59	16497.91	97095.14	61309.49
20495.67	9398.03	9298.83	906.27	6979.50
2210338.61	1754569.22	1842303.97	747203.86	1758850.12
3451776.14	2002414.84	1985556.01	705526.93	2276049.64
0	0	0	0	0
224086.46	6670.79	14391.45	39049.71	67125.44
9304.82	18906.93	3185.73	916.10	4103.35
2722.68	73032.38	74155.02	4220.18	50201.14
0	0	0	0	0
360220.05	366321.51	338391.62	46796.77	350514.81
190564.13	136283.38	153926.54	30363.97	157979.13
225083.15	192035.46	212841.44	73599.22	183973.81
182813.66	139795.74	147965.93	67397.92	134748.40
0	0	0	0	0
48861.81	31910.41	35529.73	4546.99	31933.62
0	0	2310	1	180.67
806	187	9652	664.50	340
248977.19	292485.34	279828.69	20824.44	264407.01
1454787.09	1210528.31	1312334.41	414437.89	1251381.14
24025.68	76467.30	80649.88	5928.32	70473.80
826492.40	665661.58	731202.16	357115.62	665147.21
43387.30	47855.31	45546.69	6616.68	47448.90
546956.62	401969.91	444370.33	183745.88	401649.91
7198.89	8801.49	3783.83	706.67	11794.69
275230.37	309271.45	295874.72	76798.80	283903.56
55221.33	132032.62	135816.48	8758.21	102608.83
100403.05	8584.20	2730.49	1066.22	33739.47
121088.73	89430.10	102876.38	45847.90	93683.96
2722.68	73032.38	74155.02	4220.18	50201.14

0	0	0	6	0
344525.33	247922.48	286518.06	116855.64	259363.07
599567.65	576306.79	621082.66	294627.94	574512.39
344068.55	114078.29	94451.12	8040.88	175972.32
35442.29	34596.34	29125.63	21711.37	36635.39
65103.26	63951.92	64378.94	23518.58	62556.34
363807.15	311081.50	309040.52	57796.23	320273.08
3335.98	90570.14	84886.91	4805.95	61953.93
0	0	0	0	0
0	0	0	0	0
1015785.16	1193163.91	1219961.13	253150.69	1041202.02
32942.80	188884.30	158366.93	10229.18	121242.52
79913.44	172110.59	186414.75	30612.64	136558.25
603136.11	485998.06	478692.49	228390.90	482668.49
168254.49	99553.37	105034.59	27324.42	126517.12
925226.60	1074952.52	1120535.03	114597.55	992523.16
3590238.96	2524764.47	2856377.77	805346.43	2729306.28
93335.99	67675.06	62790.85	23984.23	71572.51
1003797.19	973785.96	981044.19	301916.12	902639.24
236278.26	448451.23	394810.31	81707.73	321334.11
61792.11	93810.25	72405.19	24680.92	68551.45
44116.62	34960.50	35111.96	21705.37	36019.39
658811.67	510726.93	538969.64	129939.81	534965.67
23	4	3	28	7
0	0	0	6	0
4252437.61	4683612.62	4761426.50	1319649.27	4046984.52
46496.87	195677.03	152034.32	9751.23	139897.80
5111	85.70	1771.83	18252.42	2230.61
30728.19	30309.34	31803.73	2205.04	23554.57
234996.24	392161.02	427289.43	65518.29	321625.80
3615.40	33539.08	37885.10	3447.41	30883.24
2396262.91	1803067.77	1868514.34	1133105.18	1779357.77
0	0	0	0	0
402305.69	83606.84	90684.50	9906.02	162199.22

702926.72	555222.20	586791.82	271954.58	547025.03
236	528	35	216	15
1335970.85	966032.85	995163.41	569371.23	994642.65
46.67	853	2317	34	186.62
0	0	0	2	0
378705.49	272165.83	376140.25	180634.38	301014.36
0	0	0	0	0
700.80	5589.58	2234.23	1340.85	1173.35
2722.68	73032.38	74155.02	4220.18	50201.14
26587.27	72777.27	74567.26	5420.18	57019.92
26	18.33	7	53	4.45
271.17	3371	1514	842	6064.50
124137.09	272631.01	257263.21	56858.49	192910.15
1057591.16	930495.81	950738.55	221228.54	871389.75
1310052.47	1066309.69	1042244.07	209636.53	1098520.12
1146024.12	1570618.77	1658637.87	275070.86	1401326.91
29585.22	3042	4230.16	1391.27	7106.62
1576140.50	822894.06	735805.39	688599.37	922198.66
1238658.37	942172.62	1022952.52	206179.20	964897.63
159971.90	146828.16	149451.72	87692.90	162564.80
87616.18	174498.55	172601.78	30593.79	117648.97
4941064.48	3807632.75	3939766.32	1477900.37	3852512.39
2835512.77	2055171.52	2176264.39	1106465.57	2070530.21
1334.94	23160.12	24096.97	1610.58	16783.59
21049.73	26452.99	31487.21	1970.11	26847.28
5.67	0	2236	28	3
69547.11	29682.84	22256.59	92969.82	23395.82
530741.15	397075.90	340385.81	87092.31	402547.43
0	0	0	0	0
72916.04	80138.37	68285.07	23427.10	71048.71
443060.85	352711.28	351929.58	28993.86	368606.28
274.47	799.97	4480.40	265.60	582.50
0	0	0	6	0
103388.99	25741.02	21312.65	43367.94	41946.56

522801.76	546238.79	596176.84	152169.07	495279.75
3	0	0	10	0
29642.22	3151	3583.83	1455.27	6628.45
11	557	0	12	0
1921.94	37583.97	38403.78	2805.50	17817.78
529697.65	422221.36	444908.22	202129.58	398013.27
1053735.83	698765.34	756188.42	437736.79	729872.21
172203.78	152611.11	178494.37	49422.95	148011.63
997354.75	824423.89	966312.03	354087.53	839116.34
503221.13	555999.90	596770.88	267091.36	514261.30
105704.58	189007.01	214800.72	51927.92	148829.99
1900385.48	1617613.66	1705534.29	655998.96	1634125.69
0	0	0	12	1.50
95001.54	55716.18	65532.22	24131.94	66994.65
14652	14080.88	8314.53	19320.17	10731.28
1605001.48	1258996.52	1352537.36	597012.74	1291043.36
73568.60	79153.71	87557.75	24185.51	65576.76
499787.99	439457.73	440181.14	151570.08	438244.85
1336031.58	1254066.72	1329989.31	451753.10	1193124.26
158534.26	229097.73	240047.92	73226.14	197446.78
2298.42	52889.28	29718.94	3266.14	30558.24
229016.29	273333.36	309546.48	61447.20	241378.16
2019594.20	1404145.79	1493870.32	944414.24	1450536.30
809662.49	955133.33	1066191.15	65760.63	947353.90
34714.53	15548.08	10169.93	3442.88	25392.33
130234.41	264693.68	263126.68	35769.43	201732.74
91306.40	188754.10	205226.98	31009.09	153227.60
90737.13	14179.63	13129.68	56933.05	20550.02
804882.57	563746.66	581147.27	212936.53	579749.36
888907.56	548614.61	570052.26	43460.41	646346.71
109913.12	269820.32	279950.85	39136.87	205086.47
5.67	0	0	24	1.50
267028.99	626170.68	581939.31	96822.66	489954.89
104646.39	54216.76	66178.32	24854.65	58488.36

0	0	0	0	0
62879.03	51140.48	49470.22	3819.46	54025.80
45959.27	20727.20	21936.86	38240.48	26475.40
10595.80	23077.01	41355.10	1785.48	17701.21
46556.04	208637.42	159208.51	10521.23	143423.82
495239.11	328355.22	341627.32	101616.47	335743
106500.37	145644.48	136377.04	13437.53	133536.93
335405.43	395900.21	424775.84	47563.77	351263.53
983940.04	743068.72	858868.28	406413.21	768091.93
38792.99	4938.63	10487.96	1743.93	11442.59
917350.47	766185.06	682845.45	358969.13	736687.44
425356.33	310739.85	327965.64	229755.13	297045.60
269491.73	213421.86	218015.82	35323.15	204236.58
103253.69	114350.65	138943.60	137810.38	104809.76
60191.64	52978.94	58978.39	3470.48	50776.62
3843.51	109164.21	81901.83	4432.68	55952.97
38974.06	33466.87	33620.60	4040.19	37114.86
21179.26	23056.67	26272.63	1682.55	22254.42
1104574.64	1083825	1213634.56	295469.67	1053948.31
3269.01	62239.57	71785.01	3920.01	44749.91
697185.68	458404.78	470974.76	151793.89	483161.04
66664.42	161072.87	158054.91	9938.44	119743.03
970754.47	755099.98	811516.09	295463.51	781592.24
897411.18	688943.26	794839.66	435345.78	723800.73
27395.76	16274.09	5036.43	1170.67	19082.37
20.67	1257	138	70	543.95
1315762.02	727527.15	780209.10	357659.45	799078.92
3537733.02	3126659.90	3191099.28	793579.87	3108139.13
6017.69	135285.95	145947.03	8189.19	94951.05
196226.67	172381.21	174097.92	49750.44	160888.86
74443.85	54408.69	58837.29	23875.81	54806.06
2049.50	26992.44	40665.67	2508.30	15449.69
1920410.26	1170951.17	1264137.36	775343.64	1270144.74
337142.41	61067.95	71577.64	62622.85	130581.94

1129268.58	910171.20	952261.96	545653.51	866294.79
189526.14	136976.81	141170.57	105092.36	131817.33
265734.63	15880.26	11414.18	3250.63	70186.86
89072.23	40275.30	35624.22	40524.67	52363.87
2722.68	73032.38	74155.02	4220.18	50201.14
1686.35	51102.86	52310.55	2886.01	37899.50
76672.89	64461.91	70227.33	23384.18	65413.39
728748.47	556916.97	591157.68	346329.71	549047.61
225608.61	325501.77	336952.40	67234.07	299529.19
783708.76	987374.34	1024790.01	280293.86	873667.76
0	0	0	0	0
1262740.30	1020850.82	1060137.49	221661.10	1078340.09
53027.85	7666.53	3152.99	18762.52	14706.20
9728.17	24646.50	15617.33	1129	4909.25
190812.07	234800.66	220740.71	74241.57	196612.07
0	0	0	2	0
0	0	0	0	0
33888.58	1944.56	8281.69	19296.14	8777.73
72038.23	300836.13	317297.23	37176.82	216317.50
61926.22	126591.38	130055.25	10998.64	104448.50
0	0	0	0	0
120358.69	130277.50	145345.12	31121.70	129365.53
0	0	0	2	0
259312.51	129253.82	143269.98	28518.60	158571.92
495930.56	329152.38	362977.30	160467.74	345318.99
94521.66	100240.40	114851.69	8036.13	97260.30
63210.69	65172.51	59116.09	5247.99	63027.50
21770.46	28690.90	29857.73	2330.99	28853.12
54301.26	105159.27	104799.66	25042.67	79002.59
473192.31	953907.20	934860.33	77032.66	812022.99
366121.55	347185.19	346366	162352.17	309091.98
116984.50	328180.64	358029.18	20911.41	251283.34
520137.19	412092.24	459736.93	30883.80	452369.47
871562.83	668792.63	679248.66	374305.66	659920.45

57002.76	158870.02	188573.83	46958.19	222986.13
196.80	3103.80	5458.73	131.10	476.12
2722.68	73032.38	74155.02	4220.18	50201.14
0	0	0	0	0
1130997.18	827483.83	884460.65	388362.80	866695.89
19986.90	7	20.20	186.40	5730
908240.43	629982.32	631423.26	480858.66	628240.01
626193.77	452028.56	474573.26	72079.54	474808.25
540185.15	450150.06	473373.03	188412.68	440489.49
982419.41	346742.35	267661.04	460982.21	458591.67
43.67	853	7	27	5.95
45581.91	103800.19	122029.21	7092.60	82455.92
165068.02	98359	100626.25	7447.67	117120.06
460521.75	514439.75	550527.81	228954.75	445623.35
21770.46	28690.90	29857.73	2330.99	28853.12
1036969.45	1110439.01	1190289.92	277061.32	1054179.69
831088.34	645256.91	635271.20	267282.70	655975.37
270217.02	256572.64	287189.08	38050.90	253155.01
3029440.54	2029602.84	2077516.96	608772.57	2147103.93
149028.37	91766.38	75885.91	43049.23	95993.04
161018.38	95076.66	96829	100642.91	101152.55
599509.86	443180.47	449893.05	243397.77	453882.21
160.01	4412.60	9539	271	1007.46
113916.74	63780.19	64747.97	24010.41	71337.06
11356.04	81821.91	48327.99	3813.18	41034.44
1872516.84	2642469.44	2882910.28	180229.89	2477406.69
541.67	12187.66	21206.34	777.67	10211
401680.51	792202.30	794448.09	93848.14	616575.31
504238.86	489827.09	517284.67	208629.88	446876.32
0	0	0	0	0
0	0	0	51	0
644216.50	754636.74	967859.97	140648.56	689225.51
0	0	0	0	0
0	0	0	0	0

258937.31	166405.30	187206.39	90705.54	195700.29
356520.30	247965.33	328800.02	177517.13	277712.57
1086131.47	417795.05	353594.73	314157.09	516452.92
0	0	8944	0	0
211787.23	233618.93	229278.47	78199.68	223768.06
1374261.03	955748.50	946361.06	474446.48	962470.03
1918956.53	691736.77	753916.11	365722.44	987158.14
401717.23	613631.57	574825.40	40060.74	515770.99
1458229.96	1023266.17	1093216.70	267986.30	1077499.33
365190.35	111266.91	98152.71	69168.48	171280
1353847.28	236646.24	156729.50	427147.02	435581.70
37893.79	13340.68	13020.89	38727.46	29343.32
0	0	0	0	0
351306.63	924194.94	994739.88	120331.91	700480.63
140283.67	115737.65	125570.07	83369.55	113531.61
131675.14	229096.57	216281.62	70281.99	169308.91
0	0	0	0	0
0	0	0	0	0
814175.01	1019395.32	1060005.67	224712.91	881377.58
63	14.50	276	49	29.50
35323.11	43327.25	52114.23	21480.20	38208.41
