# Supplementary material for: Bacterial Colonization in the Airways and Intestines of Twin and Singleton Preterm Neonates: A Single-Center Study
Source: Can J Infect Dis Med Microbiol. 2023 Aug 1;2023:2973605. doi: 10.1155/2023/2973605 (PMC10409585; doi:10.1155/2023/2973605)
Supplement: Supplementary Materials — Supplementary Material 1: sequence information identified in singleton and twin preterm neonates. Supplementary Material 2: abundance of bacterial taxa and corresponding 16SrRNA gene sequences in singleton and twin preterm neonates. Supplementary Material 3: class-, order-, family-, and species-level differences were determined using the Kruskal–Wallis test. Supplementary Material 4: three levels of annotation in the KEGG database on metabolic pathways. [file 2973605.f1.zip › Supplementary Material 3 Class, Order, Family and Species levels.pdf]

We created stacked bar plots of groups A – D for abundance at the phylum, order, family, genus, and species levels. We constructed heat maps displaying the top 30 compositions for relative abundance.

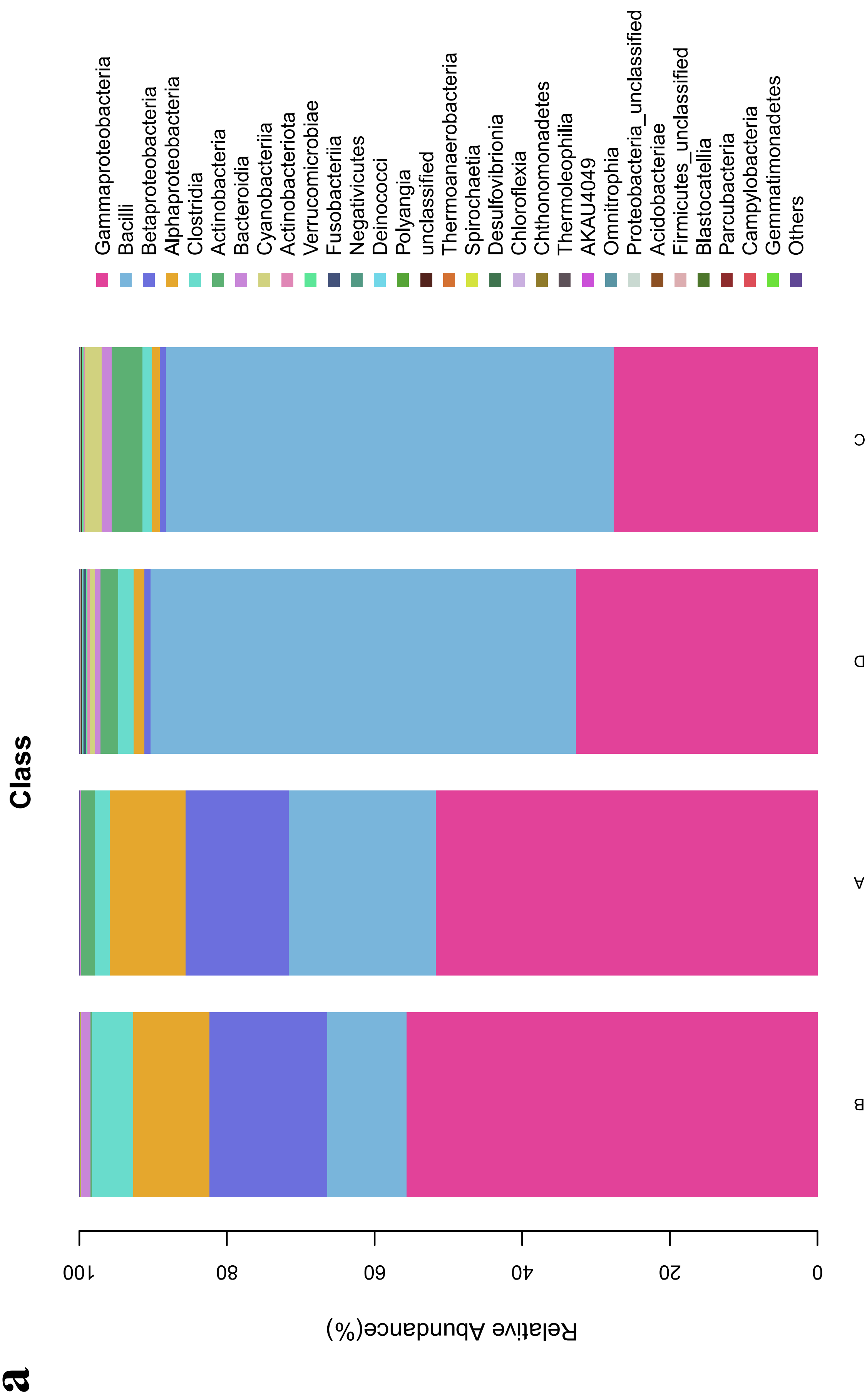

b

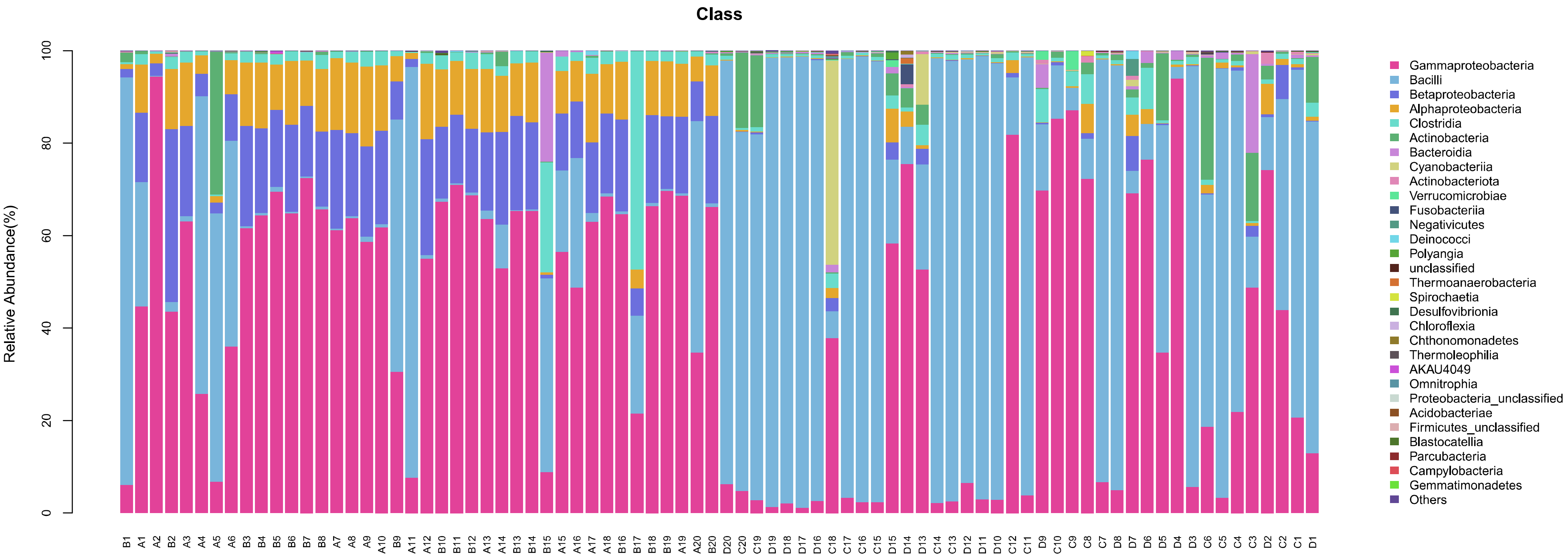

Class
Relative Abundance(%)
0
20
40
60
80
100
B1 A1 A2 B2 A3 A4 A5 A6 B3 B4 B5 B6 B7 B8 A7 A8 A9 A10 B9 A11 A12 B10 B11 B12 A13 A14 B13 B14 B15 A15 A16 A17 A18 B16 B17 B18 B19 A19 A20 B20 D20 C20 C19 D19 D18 D17 D16 C18 C17 C16 C15 D15 D14 D13 C14 C13 D12 D11 D10 C12 C11 D9 C10 C9 C8 C7 D8 D7 D6 D5 D4 D3 C6 C5 C4 C3 D2 C2 C1 D1
Gammaproteobacteria
Bacilli
Betaproteobacteria
Alphaproteobacteria
Clostridia
Actinobacteria
Bacteroidia
Cyanobacteriia
Actinobacteriota
Verrucomicrobiae
Fusobacteriia
Negativicutes
Deinococci
Polyangia
unclassified
Thermoanaerobacteria
Spirochaetia
Desulfovibrionia
Chloroflexia
Chthonomonadetes
Thermoleophilia
AKAU4049
Omnitrophia
Proteobacteria_unclassified
Acidobacteriae
Firmicutes_unclassified
Blastocatellia
Parcubacteria
Campylobacteria
Gemmatimonadetes
Others

c

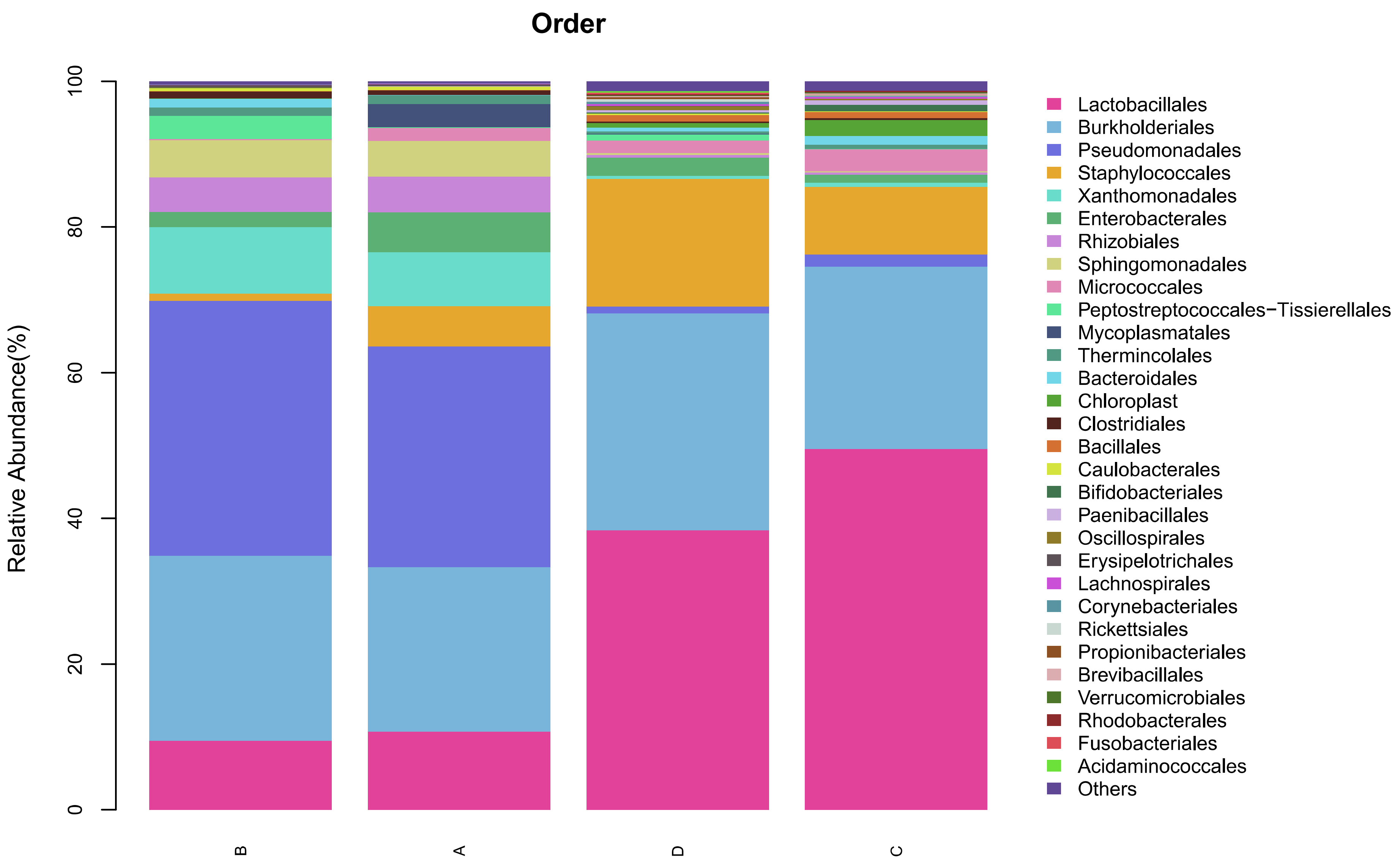
Order
Relative Abundance(%)
0
20
40
60
80
100
B
A
D
C
Lactobacillales
Burkholderiales
Pseudomonadales
Staphylococcales
Xanthomonadales
Enterobacterales
Rhizobiales
Sphingomonadales
Micrococcales
Peptostreptococcales−Tissierellales
Mycoplasmatales
Thermincolales
Bacteroidales
Chloroplast
Clostridiales
Bacillales
Caulobacterales
Bifidobacteriales
Paenibacillales
Oscillospirales
Erysipelotrichales
Lachnospirales
Corynebacteriales
Rickettsiales
Propionibacteriales
Brevibacillales
Verrucomicrobiales
Rhodobacterales
Fusobacteriales
Acidaminococcales
Others

d

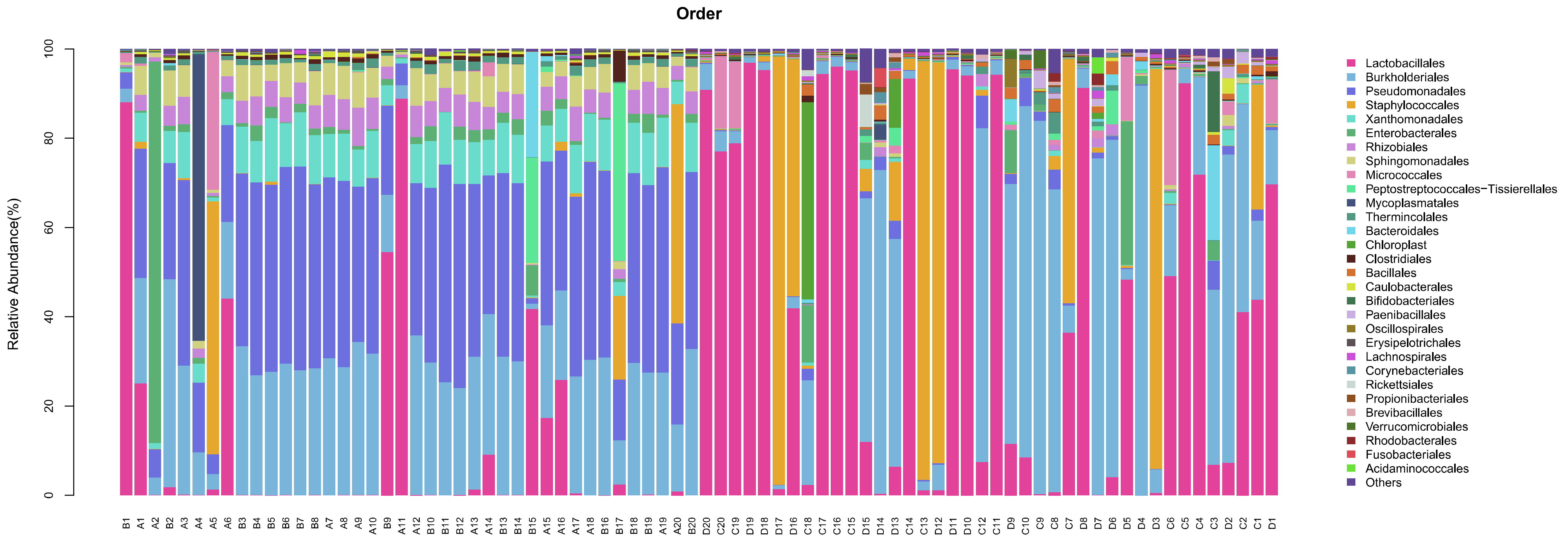
Order
Relative Abundance(%)
0
20
40
60
80
100
B1 A1 A2 B2 A3 A4 A5 A6 B3 B4 B5 B6 B7 B8 A7 A8 A9 A10 B9 A11 A12 B10 B11 B12 A13 A14 B13 B14 B15 A15 A16 A17 A18 B16 B17 B18 B19 A19 A20 B20 D20 C20 C19 D19 D18 D17 D16 C18 C17 C16 C15 D15 D14 D13 C14 C13 D12 D11 D10 C12 C11 D9 C10 C9 C8 C7 D8 D7 D6 D5 D4 D3 C6 C5 C4 C3 D2 C2 C1 D1
Lactobacillales
Burkholderiales
Pseudomonadales
Staphylococcales
Xanthomonadales
Enterobacterales
Rhizobiales
Sphingomonadales
Micrococcales
Peptostreptococcales−Tissierellales
Mycoplasmatales
Thermincolales
Bacteroidales
Chloroplast
Clostridiales
Bacillales
Caulobacterales
Bifidobacteriales
Paenibacillales
Oscillospirales
Erysipelotrichales
Lachnospirales
Corynebacteriales
Rickettsiales
Propionibacteriales
Brevibacillales
Verrucomicrobiales
Rhodobacterales
Fusobacteriales
Acidaminococcales
Others

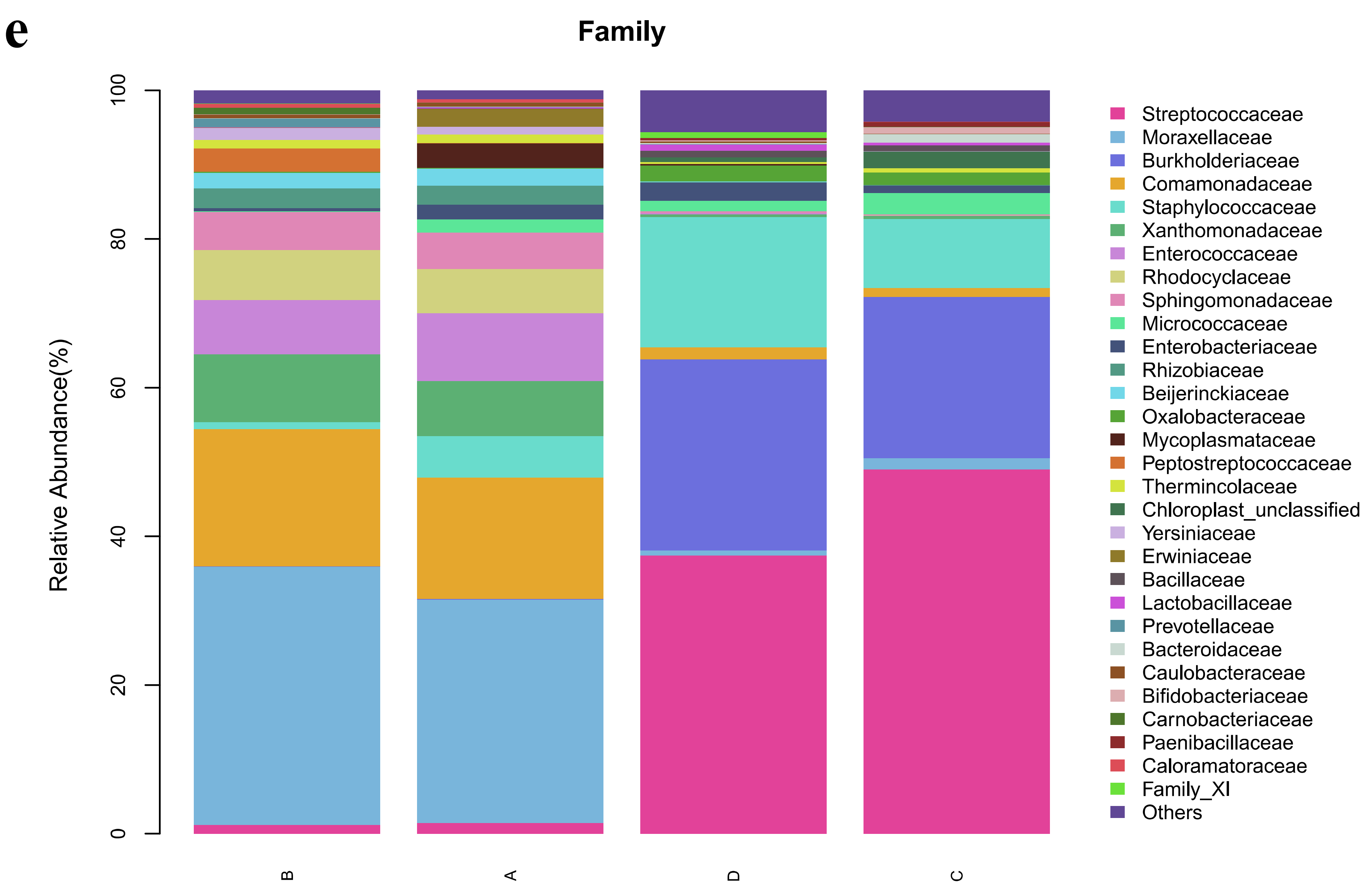
e
Family
Relative Abundance(%)
0
20
40
60
80
100
B
A
D
C
Streptococcaceae
Moraxellaceae
Burkholderiaceae
Comamonadaceae
Staphylococcaceae
Xanthomonadaceae
Enterococcaceae
Rhodocyclaceae
Sphingomonadaceae
Micrococcaceae
Enterobacteriaceae
Rhizobiaceae
Beijerinckiaceae
Oxalobacteraceae
Mycoplasmataceae
Peptostreptococcaceae
Thermincolaceae
Chloroplast_unclassified
Yersiniaceae
Erwiniaceae
Bacillaceae
Lactobacillaceae
Prevotellaceae
Bacteroidaceae
Caulobacteraceae
Bifidobacteriaceae
Carnobacteriaceae
Paenibacillaceae
Caloramatoraceae
Family_XI
Others

f

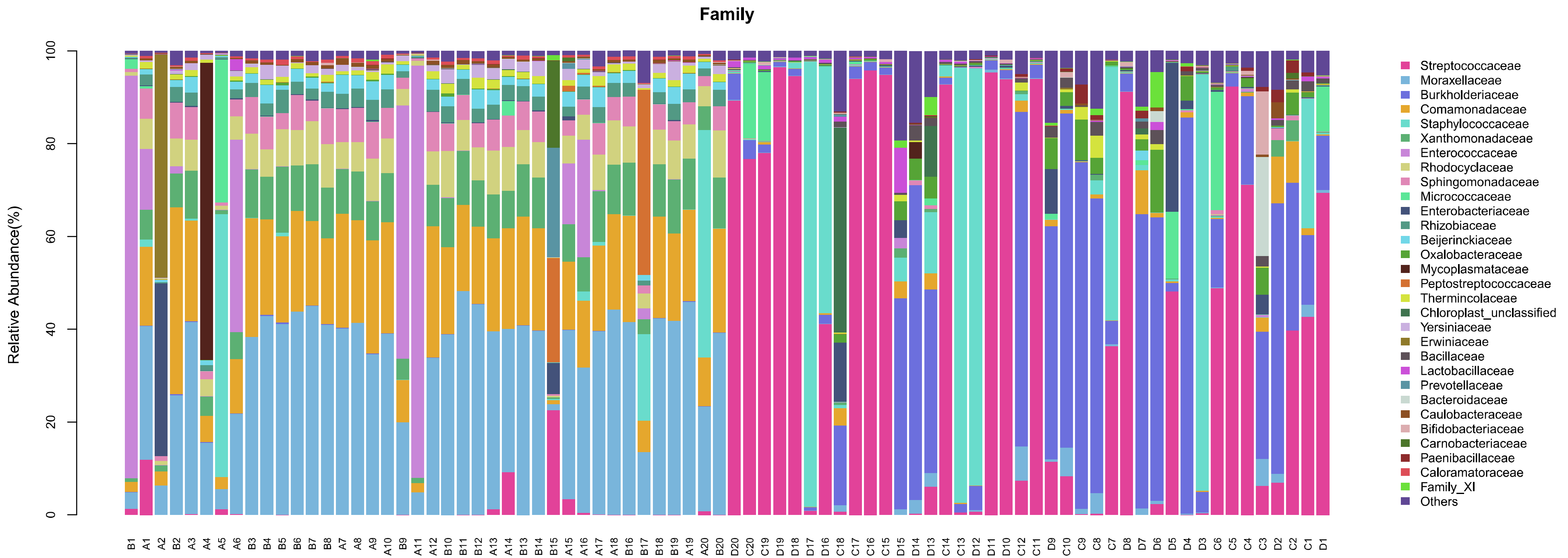
Family
Relative Abundance(%)
0
20
40
60
80
100
B1 A1 A2 B2 A3 A4 A5 A6 B3 B4 B5 B6 B7 B8 A7 A8 A9 A10 B9 A11 A12 B10 B11 B12 A13 A14 B13 B14 B15 A15 A16 A17 A18 B16 B17 B18 B19 A19 A20 B20 D20 C20 C19 D19 D18 D17 D16 C18 C17 C16 C15 D15 D14 D13 C14 C13 D12 D11 D10 C12 C11 D9 C10 C9 C8 C7 D8 D7 D6 D5 D4 D3 C6 C5 C4 C3 D2 C2 C1 D1
Streptococcaceae
Moraxellaceae
Burkholderiaceae
Comamonadaceae
Staphylococcaceae
Xanthomonadaceae
Enterococcaceae
Rhodocyclaceae
Sphingomonadaceae
Micrococcaceae
Enterobacteriaceae
Rhizobiaceae
Beijerinckiaceae
Oxalobacteraceae
Mycoplasmataceae
Peptostreptococcaceae
Thermincolaceae
Chloroplast_unclassified
Yersiniaceae
Erwiniaceae
Bacillaceae
Lactobacillaceae
Prevotellaceae
Bacteroidaceae
Caulobacteraceae
Bifidobacteriaceae
Carnobacteriaceae
Paenibacillaceae
Caloramatoraceae
Family_XI
Others

g

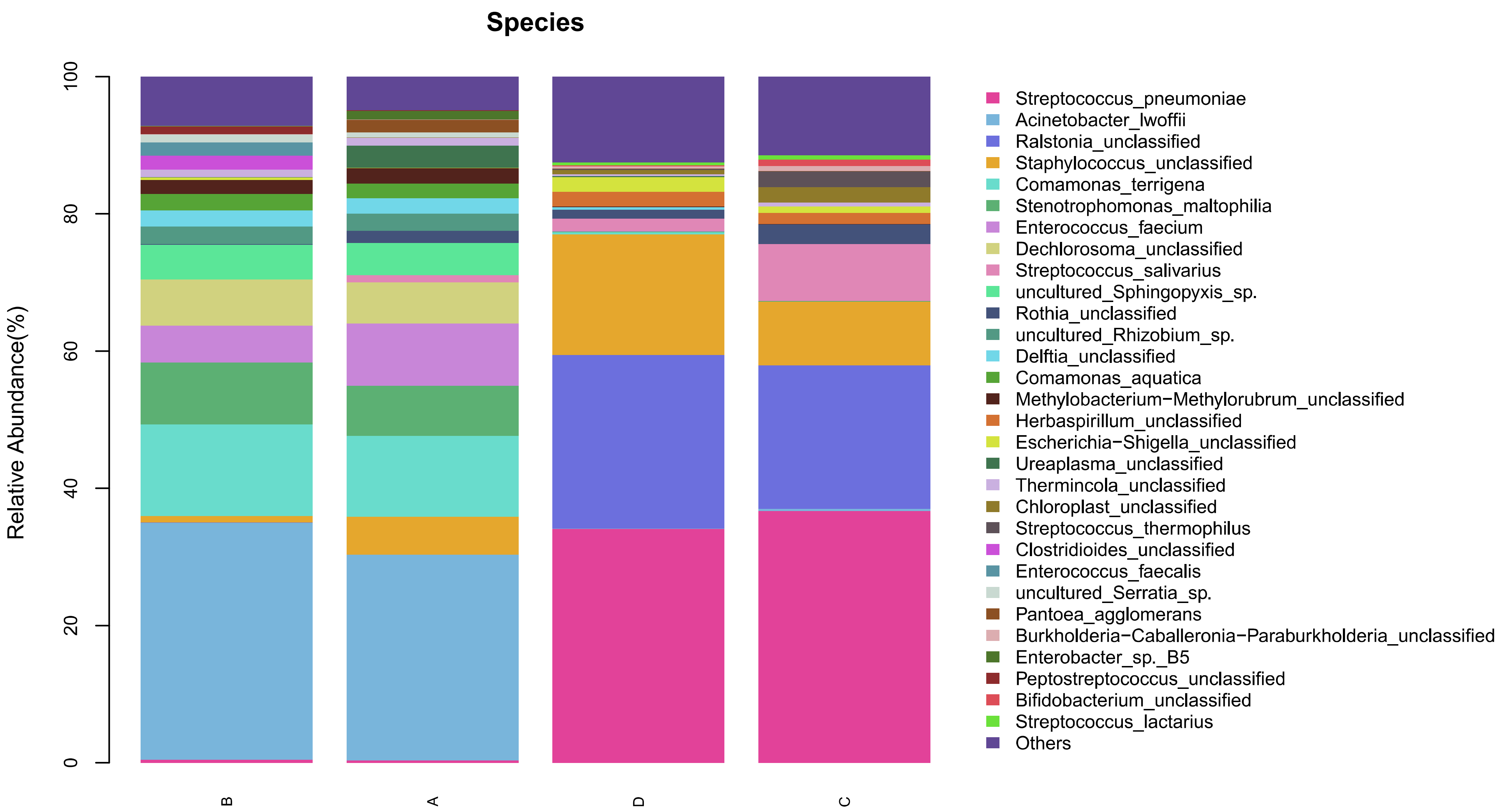
Species
Relative Abundance(%)
0
20
40
60
80
100
B
A
D
C
Streptococcus_pneumoniae
Acinetobacter_lwoffii
Ralstonia_unclassified
Staphylococcus_unclassified
Comamonas_terrigena
Stenotrophomonas_maltophilia
Enterococcus_faecium
Dechlorosoma_unclassified
Streptococcus_salivarius
uncultured_Sphingopyxis_sp.
Rothia_unclassified
uncultured_Rhizobium_sp.
Delftia_unclassified
Comamonas_aquatica
Methylobacterium−Methylorubrum_unclassified
Herbaspirillum_unclassified
Escherichia−Shigella_unclassified
Ureaplasma_unclassified
Thermincola_unclassified
Chloroplast_unclassified
Streptococcus_thermophilus
Clostridioides_unclassified
Enterococcus_faecalis
uncultured_Serratia_sp.
Pantoea_agglomerans
Burkholderia−Caballeronia−Paraburkholderia_unclassified
Enterobacter_sp._B5
Peptostreptococcus_unclassified
Bifidobacterium_unclassified
Streptococcus_lactarius
Others

h

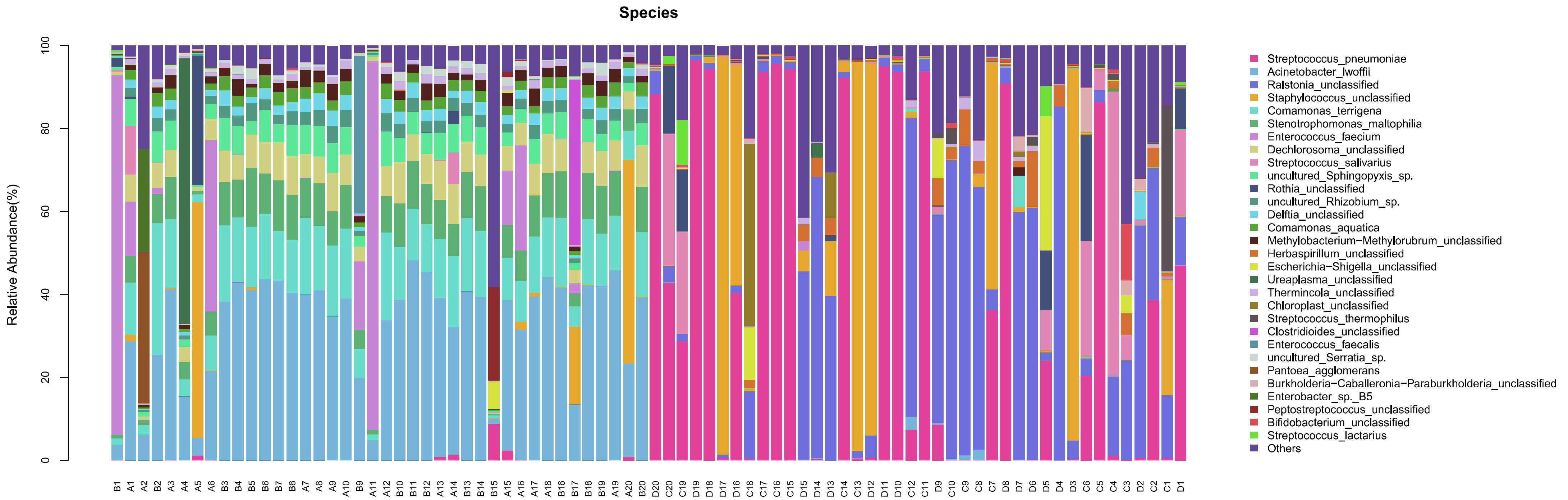
Species
Relative Abundance(%)
0
20
40
60
80
100
B1 A1 A2 B2 A3 A4 A5 A6 B3 B4 B5 B6 B7 B8 A7 A8 A9 A10 B9 A11 A12 B10 B11 B12 A13 A14 B13 B14 B15 A15 A16 A17 A18 B16 B17 B18 B19 A19 A20 B20 D20 C20 C19 D19 D18 D17 D16 C18 C17 C16 C15 D15 D14 D13 C14 C13 D12 D11 D10 C12 C11 D9 C10 C9 C8 C7 D8 D7 D6 D5 D4 D3 C6 C5 C4 C3 D2 C2 C1 D1
Streptococcus_pneumoniae
Acinetobacter_lwoffii
Ralstonia_unclassified
Staphylococcus_unclassified
Comamonas_terrigena
Stenotrophomonas_maltophilia
Enterococcus_faecium
Dechlorosoma_unclassified
Streptococcus_salivarius
uncultured_Sphingopyxis_sp.
Rothia_unclassified
uncultured_Rhizobium_sp.
Delftia_unclassified
Comamonas_aquatica
Methylobacterium−Methylorubrum_unclassified
Herbaspirillum_unclassified
Escherichia−Shigella_unclassified
Ureaplasma_unclassified
Thermincola_unclassified
Chloroplast_unclassified
Streptococcus_thermophilus
Clostridioides_unclassified
Enterococcus_faecalis
uncultured_Serratia_sp.
Pantoea_agglomerans
Burkholderia−Caballeronia−Paraburkholderia_unclassified
Enterobacter_sp._B5
Peptostreptococcus_unclassified
Bifidobacterium_unclassified
Streptococcus_lactarius
Others

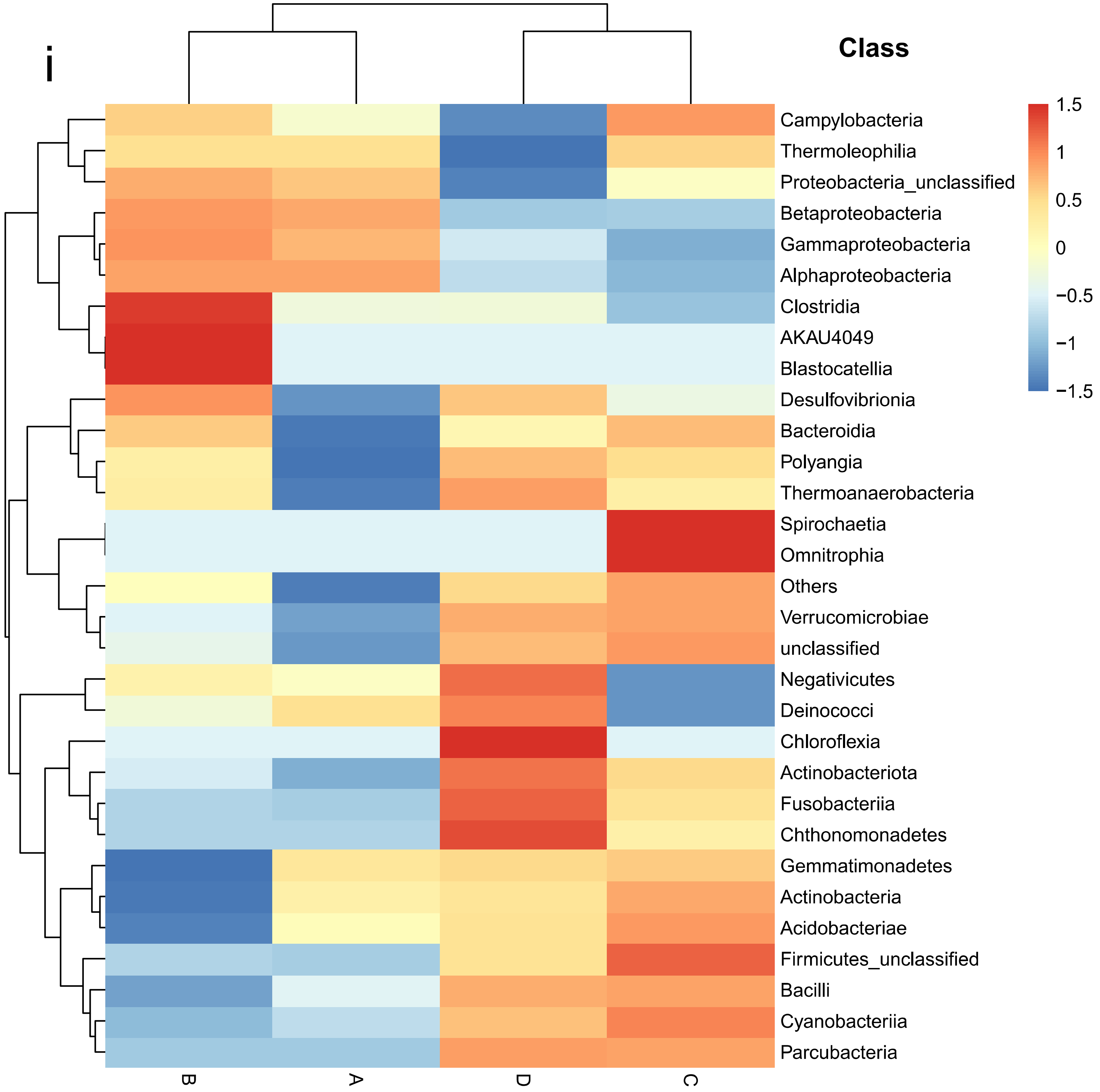

i
Class
Campylobacteria
Thermoleophilia
Proteobacteria_unclassified
Betaproteobacteria
Gammaproteobacteria
Alphaproteobacteria
Clostridia
AKAU4049
Blastocatellia
Desulfovibrionia
Bacteroidia
Polyangia
Thermoanaerobacteria
Spirochaetia
Omnitrophia
Others
Verrucomicrobiae
unclassified
Negativicutes
Deinococci
Chloroflexia
Actinobacteriota
Fusobacteriia
Chthonomonadetes
Gemmatimonadetes
Actinobacteria
Acidobacteriae
Firmicutes_unclassified
Bacilli
Cyanobacteriia
Parcubacteria
1.5
1
0.5
0
−0.5
−1
−1.5
B
A
D
C

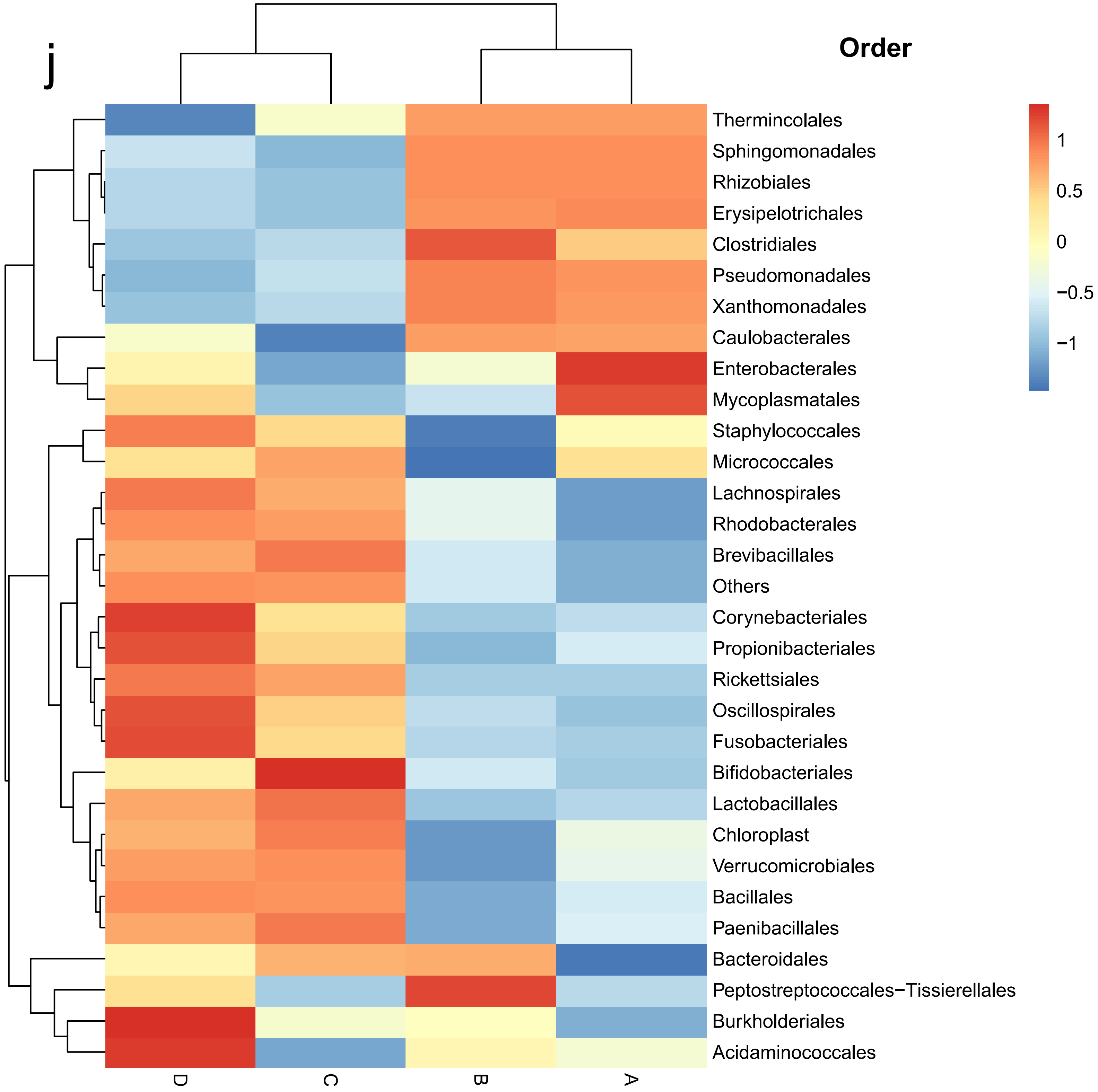
j
Order
Thermincolales
Sphingomonadales
Rhizobiales
Erysipelotrichales
Clostridiales
Pseudomonadales
Xanthomonadales
Caulobacterales
Enterobacterales
Mycoplasmatales
Staphylococcales
Micrococcales
Lachnospirales
Rhodobacterales
Brevibacillales
Others
Corynebacteriales
Propionibacteriales
Rickettsiales
Oscillospirales
Fusobacteriales
Bifidobacteriales
Lactobacillales
Chloroplast
Verrucomicrobiales
Bacillales
Paenibacillales
Bacteroidales
Peptostreptococcales−Tissierellales
Burkholderiales
Acidaminococcales
1
0.5
0
−0.5
−1
D
C
B
A

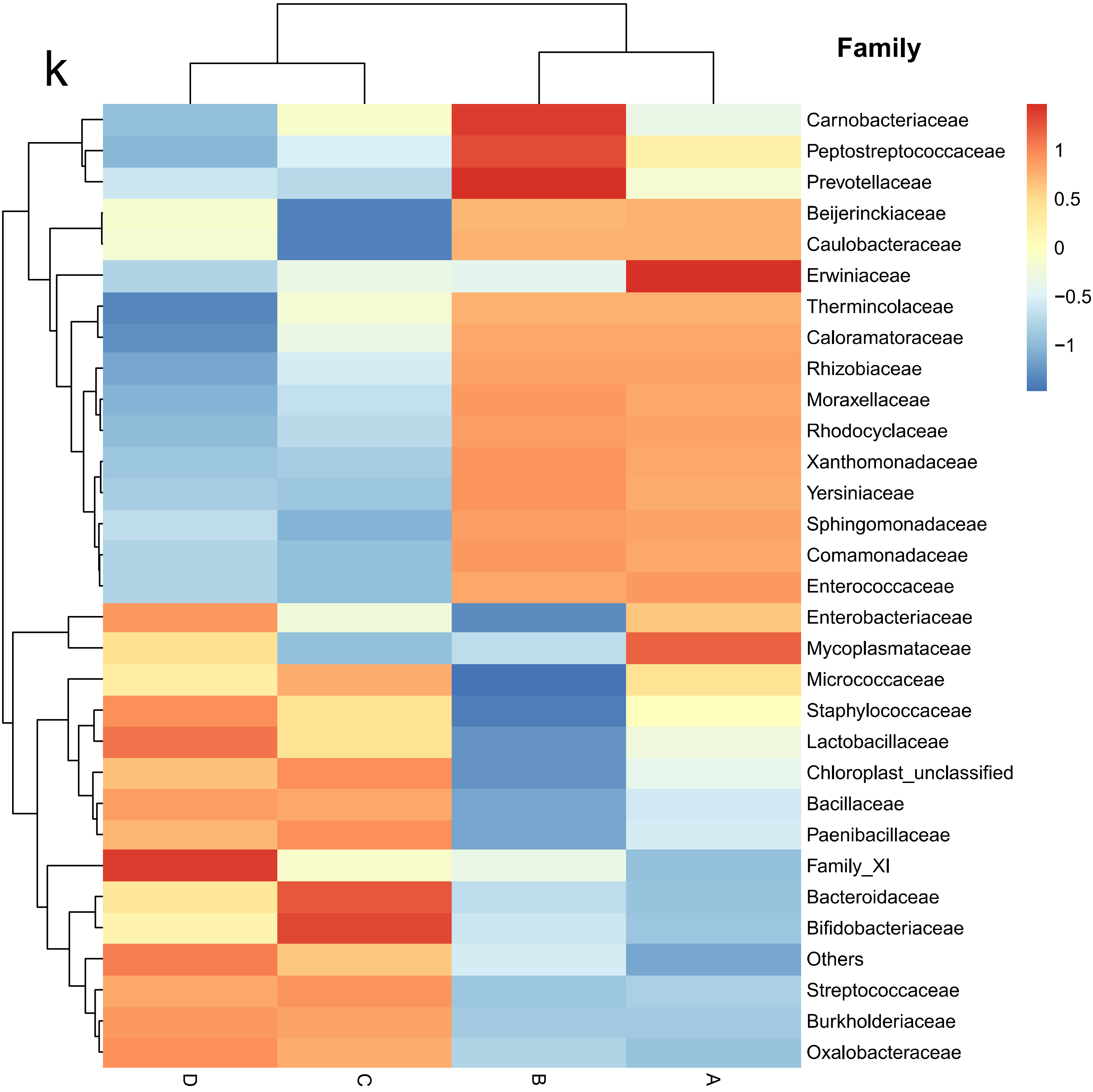
k
Family
Carnobacteriaceae
Peptostreptococcaceae
Prevotellaceae
Beijerinckiaceae
Caulobacteraceae
Erwiniaceae
Thermincolaceae
Caloramatoraceae
Rhizobiaceae
Moraxellaceae
Rhodocyclaceae
Xanthomonadaceae
Yersiniaceae
Sphingomonadaceae
Comamonadaceae
Enterococcaceae
Enterobacteriaceae
Mycoplasmataceae
Micrococcaceae
Staphylococcaceae
Lactobacillaceae
Chloroplast_unclassified
Bacillaceae
Paenibacillaceae
Family_XI
Bacteroidaceae
Bifidobacteriaceae
Others
Streptococcaceae
Burkholderiaceae
Oxalobacteraceae
1
0.5
0
−0.5
−1
D
C
B
A

I

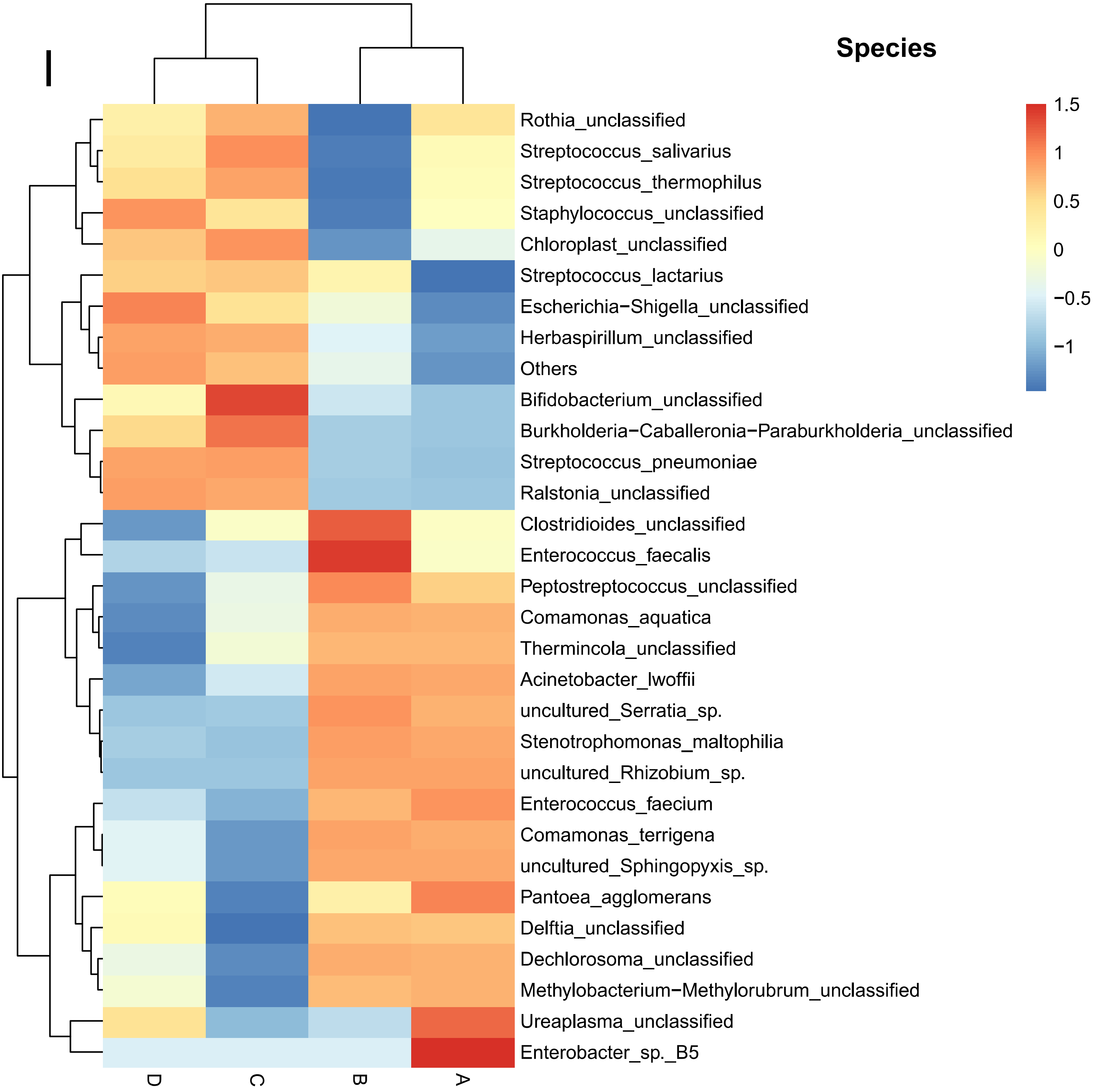
Species
1.5
1
0.5
0
−0.5
−1
Rothia_unclassified
Streptococcus_salivarius
Streptococcus_thermophilus
Staphylococcus_unclassified
Chloroplast_unclassified
Streptococcus_lactarius
Escherichia−Shigella_unclassified
Herbaspirillum_unclassified
Others
Bifidobacterium_unclassified
Burkholderia−Caballeronia−Paraburkholderia_unclassified
Streptococcus_pneumoniae
Ralstonia_unclassified
Clostridioides_unclassified
Enterococcus_faecalis
Peptostreptococcus_unclassified
Comamonas_aquatica
Thermincola_unclassified
Acinetobacter_lwoffii
uncultured_Serratia_sp.
Stenotrophomonas_maltophilia
uncultured_Rhizobium_sp.
Enterococcus_faecium
Comamonas_terrigena
uncultured_Sphingopyxis_sp.
Pantoea_agglomerans
Delftia_unclassified
Dechlorosoma_unclassified
Methylobacterium−Methylorubrum_unclassified
Ureaplasma_unclassified
Enterobacter_sp._B5
D
C
B
A
